# Molecular evolutionary analysis of human primary microcephaly genes

**DOI:** 10.1186/s12862-021-01801-0

**Published:** 2021-05-03

**Authors:** Nashaiman Pervaiz, Hongen Kang, Yiming Bao, Amir Ali Abbasi

**Affiliations:** 1 National Center for Bioinformatics, Program of Comparative and Evolutionary Genomics, Faculty of Biological Sciences, Quaid-i-Azam University, Islamabad, 45320 Pakistan; 2 China National Center for Bioinformation and National Genomics Data Center, Beijing Institute of Genomics, Chinese Academy of Sciences, Beijing, 100101 China

**Keywords:** Molecular evolution, Brain, Primary microcephaly, MCPH, Primates, Positive selection

## Abstract

**Background:**

There has been a rapid increase in the brain size relative to body size during mammalian evolutionary history. In particular, the enlarged and globular brain is the most distinctive anatomical feature of modern humans that set us apart from other extinct and extant primate species. Genetic basis of large brain size in modern humans has largely remained enigmatic. Genes associated with the pathological reduction of brain size (primary microcephaly-MCPH) have the characteristics and functions to be considered ideal candidates to unravel the genetic basis of evolutionary enlargement of human brain size. For instance, the brain size of microcephaly patients is similar to the brain size of *Pan troglodyte* and the very early hominids like the *Sahelanthropus tchadensis* and *Australopithecus afarensis*.

**Results:**

The present study investigates the molecular evolutionary history of subset of autosomal recessive primary microcephaly (MCPH) genes; CEP135, ZNF335, PHC1, SASS6, CDK6, MFSD2A, CIT, and KIF14 across 48 mammalian species. Codon based substitutions site analysis indicated that ZNF335, SASS6, CIT, and KIF14 have experienced positive selection in eutherian evolutionary history. Estimation of divergent selection pressure revealed that almost all of the MCPH genes analyzed in the present study have maintained their functions throughout the history of placental mammals. Contrary to our expectations, human-specific adoptive evolution was not detected for any of the MCPH genes analyzed in the present study.

**Conclusion:**

Based on these data it can be inferred that protein-coding sequence of MCPH genes might not be the sole determinant of increase in relative brain size during primate evolutionary history.

**Supplementary Information:**

The online version contains supplementary material available at 10.1186/s12862-021-01801-0.

## Background

The enlarged and globular brain distinguish modern humans not only from extant non-human primates but also from their extinct Homo relatives [1]. The modern human brain is approximately three-fold larger in size than that of our closest extant relative, the chimpanzee, and extinct early hominids. From developmental perspective, the larger brain size in humans is attributed to human-specific fast and prolonged neonatal and postnatal brain growth and patterning [2–4]. The evolutionary expansion of human brain size is heterogeneous across brain regions. For instance, the most notable expansion occurred in the neocortex that has been directly related to the emergence of higher cognitive capabilities, such as language, intelligence, and social learning [5]. Most of the expansion in brain size occurred in the last 2–3 million years of human evolution [6, 7]. The genetic basis of divergence between the highly cognitive human brain and supposedly lesser cognitive non-human primate brain has largely remained enigmatic. It has been speculated that complexity of modern human brain arose through changes in protein-coding genes and non-coding regulatory elements [8]. In particular, in relation to the evolutionary expansion of cerebral cortex, genes associated with primary microcephaly (MCPH) have been the focus of immense attention in the past couple of decades [9]. Primary microcephaly is an autosomal congenital disorder, which is characterized by significant reduction in brain mass, particularly the cerebral cortex with no other neuroanatomical abnormalities [10]. The brain size of microcephaly patients is similar to that of very primitive extinct hominids *Sahelanthropus tchadensis* (brain size; 370 cm^3^) and *Australopithecus afarensis* (brain size; 450 cm^3^). Therefore, the small brain size in microcephaly patients is considered as an example of evolutionary retrogression [7, 11]. Furthermore, microcephaly patients have mild to severe cognitive impairment [10]. MCPH is a heterogeneous disorder and associated with mutations in at least sixteen gene loci (MCPH1-16) [12]. The underlying MCPH genes are named as *MCPH1, WDR62, CDK5RAP2, CASC5, ASPM, CENPJ, STIL, CEP135, CEP152, ZNF335, PHC1, CDK6, SASS6, MFSD2A, CIT, and KIF14* [12–27]. Almost all MCPH-associated genes express predominantly in the fetal brain and regulate neurogenesis and brain size through participation in several important cellular mechanisms including DNA replication, repair, cytokinesis, intracellular transport and autophagy [27]. Therefore, investigation of MCPH genes can reveal molecular mechanisms that are critical in the regulation of brain size and complexity [28, 29]. Indeed, many previously reported molecular evolutionary investigations have implicated MCPH genes (like *ASPM* and *CDK5RAP2*) in the expansion and reduction of brain size during primate history [30, 31].

In this study, the roles of MCPH genes in the evolutionary enlargement of human brain size was explored through molecular evolutionary analysis of eight newly identified microcephaly genes; *CEP135, ZNF335, PHC1, CDK6, SASS6, MFSD2A, CIT,* and *KIF14*. Sequence data from 48 eutherian species was employed to investigate the signatures of episodic positive selection and diversifying selection during mammalian evolutionary history. Results obtained in the present study provide a broader perspective on the evolutionary link between primary microcephaly genes and human brain size.

## Results

### Molecular evolutionary analysis of MCPH genes

In order to investigate the genetic basis of the evolutionary expansion of human brain, eight newly identified *MCPH* genes (*CEP135, ZNF335, PHC1, CDK6, SASS6, MFSD2A, CIT*, and *KIF14*) were considered as candidates for evolutionary analysis. We assembled the coding sequence for each of this selected subset of MCPH genes from a broad range of eutherian genomes which include 21 primatomorphans, 9 glirans, 6 carnivores, 2 perissodactylans, 5 cetartiodactylans, 3 chiropterans, and one animal each from Eulipotyphla and Proboscidea (Additional file 1: Figure S1). These 48 eutherian genomes provide enough genomic coverage and evolutionary breadth to perform a thorough molecular evolutionary genetic analysis. For each of these selected subsets of MCPH genes, orthologous sequence information was classified into three data sets. i.e., primates (20 genomes), nonprimate placental mammals (28 genomes), and placental mammals (48 genomes). Each of these selected subsets of datasets is subjected to maximum likelihood based codon substitution models.

### Signature of pervasive positive selection in MCPH genes

In order to examine whether the pervasive positive selection has operated on selected subset of MCPH genes (*CEP135, ZNF335, PHC1, CDK6, SASS6, MFSD2A, CIT*, and *KIF14*), three pairs of site models (M1 & M2, M7 & M8, and M8a & M8) based on codon substitutions were used. These codon substitutions site models assume that selective pressure ω vary among the amino acid sites but not across the lineage [32]. The signature of positive selection is considered optimal only if two out of the three null models (M1, M7, and M8a) are rejected in the favor of more complex alternative models (M2 and M8). The results of codon substitutions site pair models (M1/M2, M7/M8, and M8a/M8) revealed the consistent signature of pervasive positive selection only for *KIF1*4 gene in primates (Table 1 and Additional file 2: Table S1). Furthermore, significant signatures of positive selection were also detected in non-primate placental mammals and placental mammals for three (*ZNF335, SASS6,* and *CIT*) and two MCPH genes (*ZNF335*, and *KIF14*) respectively (Table 1 and Additional file 2: Table S1). Positively selected codon sites were identified by using the Bayes Empirical Bayes (BEB) method implemented in M8 codon substitution site model [33] (Table 2).

**Table 1:** Signature of pervasive positive selection in primary microcephaly genes

Genes	Data sets	No. of sequences	(M2-M1)			(M8-M7)			(M8-M8a)		
			LRT	P value	q Value	LRT	P value	q Value	LRT	P value	q Value
ZNF335	Nonprimate mammals	27	0	1	1	24.13	0.000005	0.000013	9.34	0.002	0.008
	Mammals	45	0	1	1	27.41	0.000001	0.000005	18.62	0.00002	0.0002
SASS6	Nonprimate mammals	25	0	1	1	21.58	0.00002	0.00004	7.196	0.007	0.02
CIT	Nonprimate mammals	22	0	1	1	38.84	< 0.0000001	< 0.0000001	18.21	0.00002	0.0002
	Mammals	37	0.000002	1	1	24.4	0.000005	0.00001	0.002	0.96	0.80
KIF14	Primates	17	13.46	0.002	0.00	20.76	0.00003	0.0006	11.63	0.0006	0.004
	Mammals	29	0	1	1	26.67	0.0000001	0.000005	10.36	0.001	0.005

Positive selection is inferred if two out of three site model comparisons significantly reject null hypothesis using the cutoff of q value 0.05. Null models: M1, M7, and M8a, Alternative models: M2 and M8, LRT: likelihood ratio test. False discovery rate q value corrections over p values were calculated using q value package in R

**Table 2:** Positive selected sites detected by using M8 codon substitution site model

Gene	Data sets	No. of codon under positive selection	Sites under positive selection (BEB > 80%)
ZNF335	Non-primate mammals	7	225A^α^, 365P, 414G, 761S, 830P, 983V^α^, 1133S
	Mammals	7	93H, 225A^α^, 738A, 761S, 830P, 938V^β^, 1133S
SASS6	Non-primate mammals	7	99A, 241I, 494 T, 524S, 550 T, 581I^α^, 585C
CIT	Non-primate mammals	8	11P, 12L^α^, 17A, 150R, 279T^β^, 334F, 1479 T, 1723I
KIF14	Primates	12	107L, 203P, 204S, 342 V, 875H^β^, 1413F, 1474S, 1499R^α^, 1532Q, 1602 K, 1605H, 1612G
	Mammals	10	29A, 107L, 166I, 203P, 204S, 232 T, 1303I, 1525H, 1608S^α^, 1632S

Positively selected sites are identifies using Bayes Empirical Bayes (BEB) analysis implemented in M8 codon substitution site model with cutoff of posterior probability > 0.80%. Sites with P value <  = 0.05 are labeled as α and with P value <  = 0.01 are labeled as β

### Signatures of episodic positive selection

We next examined the imprints of transient or episodic positive selection in different lineages from ancestral primate branch to human terminal branch by employing branch site model (Additional file 2: Table S2). Branch site model allows the ratio of nonsynonymous (dN) to synonymous substitution (dS) rates ω to vary not only across the branches in the phylogeny but also across sites [34]. The significance of the transient imprint of adaptive evolution was determined by likelihood ratio tests (LRTs) of null model (similar to branch-site model except ω2 is restricted to one for the predefined lineage of interest) against branch-site model through log likelihood score for each model. False positive results obtained by branch-site model were eliminated by estimating the false discovery rate q value. Branch site calculations revealed no significant patterns of episodic positive selection in any lineage from primate ancestor to human terminal branch for all MCPH genes analyzed except for KIF14 in homininae ancestral branch (Additional file 2: Table S2).

### Divergent selection of MCPH genes across mammals

Functional divergence among orthologous proteins during evolution may not necessarily be reflected as a signature of positive selection at the sequence level. Instead, sequence evolutionary rate variations among different clades of phylogeny can also be taken as a metric of adaptive functional diversity. For each of the eight MCPH coding regions, we estimated the divergent selective pressure between different partitions of mammalian phylogeny (primate vs. nonprimate, simians vs. nonsimians, catarrhini vs. noncatarrhini, hominidae vs. nonhominidae and hominini vs. nonhominini placental mammals) by employing clade model c (CmC) (Table 3). Similar to site and branch-site analysis, false positive data in CmC analysis was eradicated by q value [35]. The CmC analysis revealed that selective pressure on different parts of phylogeny is not significantly divergent for all analyzed microcephaly loci except for *SASS6* in the comparison of simians and nonsimians placental mammals (Table 3). In this particular case of *SASS6*, approximately 34% of sites evolved under divergent selective pressure between simians (ωs = 0.499) and nonsimians placental mammals (ωns = 0.214) (Table 3).

**Table 3:** Divergent selection constraint parameters estimation and likelihood scores for eight MCPH genes on different partition of mammalian phylogeny

Gene	Model	Parameter estimation (ω)	lnL	P value	q value
CEP135	CmC-Primate	p_0_ = 0.560, ω_0_ = 0.0231, p_1_ = 0.142,ω_1_ = 1.0, p_2_ = 0.298, ω_np_ = 0.251, ω_p_ = 0.315	− 26,080.0893	0.057	0.34
	CmC-Simians	p_0_ = 0.296, ω_0_ = 0.27, p_1_ = 0.141, ω_1_ = 1.0, p_2_ = 0.56, ω_ns_ = 0.023, ω_s_ = 0.021	− 26,081.8471	0.75	0.66
	CmC-catarrhini	p_0_ = 0.293, ω_0_ = 0.271, p_1_ = 0.141, ω_1_ = 1.0, p_2_ = 0.57, ω_nc_ = 0.023, ω_c_ = 0.0397	− 26,081.3823	0.31	0.58
	CmC-greatapes	p_0_ = 0.295, ω_0_ = 0.269, p_1_ = 0.141, ω_1_ = 1.0, p_2_ = 0.564, ω_ng_ = 0.0235, ω_g_ = 0.0184	− 26,081.8801	0.85	0.66
	CmC-hominini	p_0_ = 0.56, ω_0_ = 0.023 p_1_ = 0.141, ω_1_ = 1.0, p_2_ = 0.296, ω_nh_ = 0.27, ω_h_ = 0.59	− 26,081.1383	0.22	0.57
	M2a_rel	p_0_ = 0.295, ω_0_ = 0.269, p_1_ = 0.0141, ω_1_ = 1.0, p_2_ = 0.564, ω_2_ = 0.0235	− 26,081.8982	NA	NA
ZNF335	CmC-Primate	p_0_ = 0.75, ω_0_ = 0.015, p_1_ = 0.029, ω_1_ = 1.0, p_2_ = 0.221, ω_np_ = 0.255, ω_p_ = 0.285	− 32,294.0420	0.30	0.58
	CmC-simians	p_0_ = 0.751, ω_0_ = 0.0155, p_1_ = 0.029, ω_1_ = 1.0, p_2_ = 0.22, ω_ns_ = 0.256, ω_s_ = 0.312	− 32,293.5981	0.16	0.57
	CmC-catarrhini	p_0_ = 0.751, ω_0_ = 0.0154, p_1_ = 0.029, ω_1_ = 1.0, p_2_ = 0.22, ω_nc_ = 0.266, ω_c_ = 0.27	− 32,293.5586	0.15	0.57
	CmC-greatapes	p_0_ = 0.75, ω_0_ = 0.0154, p_1_ = 0.029, ω_1_ = 1.0, p_2_ = 0.22, ω_ng_ = 0.259, ω_g_ = 0.297	− 32,294.4826	0.66	0.66
	CmC-hominini	p_0_ = 0.75, ω_0_ = 0.0154, p_1_ = 0.029, ω_1_ = 1.0, p_2_ = 0.22, ω_nh_ = 0.26, ω_h_ = 0.25	− 32,294.5720	0.92	0.69
	M2a_rel	p_0_ = 0.75, ω_0_ = 0.015, p_1_ = 0.029, ω_1_ = 1.0, p_2_ = 0.221, ω_2_ = 0.26	− 32,294.5769	NA	NA
PHC1	CmC-Primate	p_0_ = 0.784, ω_0_ = 0.020, p_1_ = 0.021, ω_1_ = 1.0, p_2_ = 0.195, ω_np_ = 0.339, ω_p_ = 0.395	− 20,737.4013	0.29	0.58
	CmC-simians	p_0_ = 0.194, ω_0_ = 0.348, p_1_ = 0.021, ω_1_ = 1.0, p_2_ = 0.78, ω_ns_ = 0.020, ω_s_ = 0.018	− 20,737.9248	0.82	0.66
	CmC-catarrhini	p_0_ = 0.78, ω_0_ = 0.020, p_1_ = 0.021, ω_1_ = 1.0, p_2_ = 0.19, ω_nc_ = 0.347, ω_c_ = 0.364	− 20,737.9367	0.86	0.66
	CmC-greatapes	p_0_ = 0.78, ω_0_ = 0.020, p_1_ = 0.021, ω_1_ = 1.0, p_2_ = 0.19, ω_ng_ = 0.349, ω_g_ = 0.179	− 20,737.3879	0.29	0.58
	CmC-hominini	p_0_ = 0.78, ω_0_ = 0.020, p_1_ = 0.021, ω_1_ = 1.0, p_2_ = 0.19, ω_nh_ = 0.35, ω_h_ = 0.13	− 20,737.5787	0.38	0.65
	M2a_rel	p_0_ = 0.784, ω_0_ = 0.020, p_1_ = 0.021, ω_1_ = 1.0, p_2_ = 0.194, ω_2_ = 0.348	− 20,737.9512	NA	NA
SASS6	CmC-Primate	p_0_ = 0.612, ω_0_ = 0.017, p_1_ = 0.077, ω_1_ = 1.0, p_2_ = 0.310, ω_np_ = 0.253, ω_p_ = 0.270	− 14,706.9216	0.67	0.66
	CmC-simians	p_0_ = 0.58, ω_0_ = 0.013, p_1_ = 0.082, ω_1_ = 1.0, p_2_ = 0.34, ω_ns_ = 0.214, ω_s_ = 0.499	− 14,697.4440	**0.00001**	**0.0003**
	CmC-catarrhini	p_0_ = 0.31, ω_0_ = 0.26, p_1_ = 0.077, ω_1_ = 1.0, p_2_ = 0.62, ω_nc_ = 0.017, ω_c_ = 0.024	− 14,706.9516	0.72	0.66
	CmC-greatapes	p_0_ = 0.61, ω_0_ = 0.17, p_1_ = 0.078, ω_1_ = 1.0, p_2_ = 0.31, ω_ng_ = 0.25, ω_g_ = 0.47	− 14,706.3050	0.23	0.57
	CmC-hominini	p_0_ = 0.62, ω_0_ = 0.017, p_1_ = 0.077, ω_1_ = 1.0, p_2_ = 0.31, ω_nh_ = 0.26, ω_h_ = 1.082	− 14,705.0071	**0.045**	0.33
	M2a_rel	p_0_ = 0.614, ω_0_ = 0.017, p_1_ = 0.077, ω_1_ = 1.0, p_2_ = 0.308, ω_2_ = 0.259	− 14,707.0141	NA	NA
CDK6	CmC-Primate	p_0_ = 0.895, ω_0_ = 0.011, p_1_ = 0.012, ω_1_ = 1.0, p_2_ = 0.0926, ω_np_ = 0.298, ω_p_ = 0.257	− 5156.14749	0.76	0.66
	CmC-Simians	p_0_ = 0.897, ω_0_ = 0.012, p_1_ = 0.014, ω_1_ = 1.0, p_2_ = 0.088, ω_ns_ = 0.271, ω_s_ = 0.428	− 5155.82423	0.39	0.65
	CmC-catarrhini	p_0_ = 0.902, ω_0_ = 0.012, p_1_ = 0.011, ω_1_ = 1.0, p_2_ = 0.087, ω_nc_ = 0.32, ω_c_ = 0.16	− 5156.01860	0.55	0.66
	CmC-greatapes	p_0_ = 0.089, ω_0_ = 0.298, p_1_ = 0.012, ω_1_ = 1.0, p_2_ = 0.899, ω_ng_ = 0.012, ω_g_ = 0.00	− 5156.08170	0.63	0.66
	CmC-hominini	p_0_ = 0.089, ω_0_ = 0.296, p_1_ = 0.012, ω_1_ = 1.0, p_2_ = 0.898, ω_nh_ = 0.012, ω_h_ = 0.00	− 5156.12730	0.71	0.66
	M2a_rel	p_0_ = 0.897, ω_0_ = 0.011, p_1_ = 0.012, ω_1_ = 1.0, p_2_ = 0.0906, ω_2_ = 0.294	− 5156.19494	NA	NA
MFSD2A	CmC-Primate	p_0_ = 0.731, ω_0_ = 0.0152, p_1_ = 0.062, ω_1_ = 1.0, p_2_ = 0.207, ω_np_ = 0.257, ω_p_ = 0.279	− 11,299.7769	0.66	0.66
	CmC-simians	p_0_ = 0.719, ω_0_ = 0.014, p_1_ = 0.066, ω_1_ = 1.0, p_2_ = 0.21, ω_ns_ = 0.23, ω_s_ = 0.38	− 11,297.2803	**0.02**	0.30
	CmC-catarrhini	p_0_ = 0.725, ω_0_ = 0.015, p_1_ = 0.065, ω_1_ = 1.0, p_2_ = 0.21, ω_nc_ = 0.25, ω_c_ = 0.33	− 11,299.5559	0.43	0.66
	CmC-greatapes	p_0_ = 0.73, ω_0_ = 0.015, p_1_ = 0.062, ω_1_ = 1.0, p_2_ = 0.21, ω_ng_ = 0.262, ω_g_ = 0.289	− 11,299.8560	0.85	0.66
	CmC-hominini	p_0_ = 0.73, ω_0_ = 0.015, p_1_ = 0.061, ω_1_ = 1.0, p_2_ = 0.21, ω_nh_ = 0.266, ω_h_ = 0.196	− 11,299.8222	0.75	0.66
	M2a_rel	p_0_ = 0.732, ω_0_ = 0.0154, p_1_ = 0.061, ω_1_ = 1.0, p_2_ = 0.206, ω_2_ = 0.265	-11,299.8738	NA	NA
CIT	CmC-Primate	p_0_ = 0.89, ω_0_ = 0.0058, p_1_ = 0.014, ω_1_ = 1.0, p_2_ = 0.095, ω_np_ = 0.24, ω_p_ = 0.19	− 35,246.2438	0.18	0.57
	CmC-simians	p_0_ = 0.095, ω_0_ = 0.226, p_1_ = 0.015, ω_1_ = 1.0, p_2_ = 0.89, ω_ns_ = 0.0059, ω_s_ = 0.0043	− 35,246.9740	0.57	0.66
	CmC-catarrhini	p_0_ = 0.094, ω_0_ = 0.227, p_1_ = 0.015, ω_1_ = 1.0, p_2_ = 0.89, ω_nc_ = 0.0057, ω_c_ = 0.0088	− 35,246.8939	0.49	0.66
	CmC-greatapes	p_0_ = 0.89, ω_0_ = 0.0058, p_1_ = 0.015, ω_1_ = 1.0, p_2_ = 0.094, ω_ng_ = 0.2279, ω_g_ = 0.2074	− 35,247.1167	0.86	0.66
	CmC-hominini	p_0_ = 0.89, ω_0_ = 0.0057, p_1_ = 0.015, ω_1_ = 1.0, p_2_ = 0.095, ω_nh_ = 0.224, ω_h_ = 0.379	− 35,246.9556	0.55	0.66
	M2a_rel	p_0_ = 0.89, ω_0_ = 0.0058, p_1_ = 0.015, ω_1_ = 1.0, p_2_ = 0.095, ω_2_ = 0.227	− 35,247.1321	NA	NA
KIF14	CmC-Primate	p_0_ = 0.43, ω_0_ = 0.043, p_1_ = 0.15, ω_1_ = 1.0, p_2_ = 0.42, ω_np_ = 0.36, ω_p_ = 0.39	− 31,056.0434	0.58	0.66
	CmC-simians	p_0_ = 0.43, ω_0_ = 0.043, p_1_ = 0.14, ω_1_ = 1.0, p_2_ = 0.42, ω_ns_ = 0.36, ω_s_ = 0.42	− 31,055.4805	0.23	0.57
	CmC-catarrhini	p_0_ = 0.43, ω_0_ = 0.042, p_1_ = 0.15, ω_1_ = 1.0, p_2_ = 0.42, ω_nc_ = 0.36, ω_c_ = 0.41	− 31,055.9687	0.50	0.66
	CmC-greatapes	p_0_ = 0.42, ω_0_ = 0.37, p_1_ = 0.15, ω_1_ = 1.0, p_2_ = 0.43, ω_ng_ = 0.042, ω_g_ = 0.13	− 31,054.6979	0.083	0.42
	CmC-hominini	p_0_ = 0.42, ω_0_ = 0.37, p_1_ = 0.15, ω_1_ = 1.0, p_2_ = 0.43, ω_nh_ = 0.042, ω_h_ = 0.25	− 31,053.9072	**0.03**	0.30
	M2a_rel	p_0_ = 0.42, ω_0_ = 0.37, p_1_ = 0.15, ω_1_ = 1.0, p_2_ = 0.43, ω_2_ = 0.043	− 31,056.1959	NA	NA

*lnL* log likelihood score, *ω* ratio of non-synonymous (dN) to synonymous (dS) substitutions rate, *p* proportion of sites, *NA* not applicable, *np* non-primate eutherian, *p* primate, *ns* non-simians eutherian, *s* simians, *nc* non-catarrhini eutherian, *c* catarrhini, *ng* non-greatapes eutherian, *g* greatapes, *nh* non-hominini eutherian, *h* hominini. Significant p and q values are highlighted in bold

## Discussion

Compared to other primates, including great apes, humans have very large brains. Weighing approximately 1,400 g, our brains are roughly three times larger than those of other great apes such as chimpanzees (395 g) and gorillas (490 g) [36]. In particular, during 4–5 million years of human evolution, an enormous increase in brain size has occurred, from a brain mass of 450 g found in Australopithecines to about 1400 g in modern Homo sapiens [37]. Similar to overall increase in brain size/mass, the neocortex of brain (which is involved in higher-order brain functions such as cognition, reasoning and language) has significantly enlarged in the hominin lineage after the divergence of closely related chimpanzee lineage aproximately ~ 6–7 million years ago [7]. This expansion in neocortex size in the hominin lineage might have occurred prior to the split of anatomically modern humans from archaic hominins (Neanderthals and Denisovans) approximately 550,000–750,000 years ago, as both modern humans and Neanderthals exhibit comparable overall brain size [38, 39]. However, cranial lobe size (that demarcate different regions of neocortex) does differ between anatomically modern humans and Neanderthals, most prominently parieto-temporal lobe of the neocortex has increased and orbitofrontal cortex is wider in modern humans as compared to Neanderthals [38, 40]. This indicates that certain neocortical regions have evolved after the split of Neanderthals and anatomically modern humans. The size of the neocortex is predominantly determined by the magnitude of neurogenesis and cytokinesis during fetal development. During neurogenesis, cortical neurons originate from a progenitor cell in the ventricular zone of the developing brain [41]. The progenitor cells undergo successive cycles of proliferative division before entering to neurogenic division and formation of the subventricular zone [42–45]. Massive expansion in the neocortex size during human evolution has been attributed to extraordinary expansion in neuron number through increased rate of neural progenitor cell division [5]. This expansion can be explained in the context of the “*radial unit hypothesis*” of cortical development [46]. This hypothesis proposes a general mechanism for a rapid increase in neocortical surface area during evolution through prolonged proliferative symmetric division period. This could yield an increased number of radial columnar units that ultimately generate neurons and consequently expand the neocortical surface area [46]. An alternate hypothesis, “*intermediate progenitor model”* suggests that during mammalian evolution the expansion in neocortical surface area and folding have occurred due to the escalation in basal progenitor pool size (BP originates from apical radial glia; the main neural progenitor cells in the ventricular zone) and their subsequent expansion in the subventricular zone [47]. Recently, another hypothesis linked the evolutionary expansion of neocortex to increased proliferative capacity of basal radial glia (bRG) [48]. Though the timing of brain development is conserved across mammals, however species specific differences in the duration of cortical neurogenesis (6 days in mice, 60 days in macaque, and 100 days in humans) might have contributed to the differences in neocortex size and complexity during primate history [5, 49]. Species-specific difference in BP pool size abundance and temporal aspects of neocorticogenesis might have some lineage specific genetic underpinnings [48].

The majority of MCPH gene products are known to play an important role in the regulation of duration and mode of cell division and hence identified as prime suspect in evolutionary expansion of mammalian/primate cerebral cortex [43, 50, 51]. Initial evaluation of MCPH genes has revealed that *MCPH1, CDK5RAP2, ASPM,* and *CENPJ* evolved adaptively in the human lineage [9, 52, 53]. Further investigations have extended the signatures of positive selection in these MCPH loci (*MCPH1, CDK5RAP2, ASPM*, and *CENPJ*) from human to all anthropoid primates [31]. Recently, the inclusion of the non-primate eutherian species in evolutionary genetic studies of MCPH genes revealed the signatures of pervasive positive selection on *ASPM, CDK5RAP2, MCPH1, CENPJ, CEP152* and *WDR62* throughout the eutherians history [54].

The present study investigates the molecular evolutionary history of eight newly identified MCPH genes by employing sequence data from 48 eutherian genomes and rigorous maximum likelihood based codon substitution models. The codon substitutions site analysis indicated that positive selection occurred during different stages of eutherian evolution in four MCPH genes, *ZNF335, SASS6, CIT,* and *KIF14* (Table 1 and Additional file 2: Table S1). Furthermore, the codon based site models revealed an inconsistent signature of pervasive positive selection in primates for all of the microcephaly genes analyzed in the present study except *KIF14* (Table 1 and Additional file 2: Table S1). Positive selection often acts for a brief period of evolutionary time or transiently on protein-coding intervals [34]. During the course of primate history brain enlargement seems to have happened episodically, therefore, transient or episodic positive selection could be of particular relevance to genes involved in brain size expansion [7]. Intriguingly, for the majority of MCPH genes analyzed in the present study, the branch-site model was unable to identify any signatures of episodic positive selection from primate ancestor to human terminal branch (Additional file 2: Table S2). These data obtained through codon substitutions site and branch-site models were corroborated further by employing clade model C (CmC) and conclusively showed that majority of the MCPH genes have maintained their conserved functions throughout the history of placental mammals (Table 3).

Multiple sequence alignments of MCPH proteins analyzed in the present study have shown human specific substitutions for ZNF335, KIF14, PHC1, MFSD2A and CIT (Additional file 2: Table S3). Majority of these human specific substitutions appear to have fixed prior to the divergence of fully modern humans from archaic humans (Neandertals and Denisovans) some 450,000 years ago (Additional file 2: Table S3). Regardless the fact that human-specific adoptive evolution has not been detected in any of the MCPH genes analyzed here, we speculate that human specific replacements in subset of MCPH proteins could potentially be important in modifying their functions during the course of hominin evolution, The evolutionary relevance of these hominin-specific amino acid substitutions in evolution of brain size and complexity needs to be validated through further studies.

## Conclusion

The present study demonstrates that the evolutionary enlargement of human brain cannot be attributed solely to the protein-coding sequences of MCPH genes [55]. Instead, complex conditional effects of human specific coding and non-coding regulatory changes in MCPH and other brain related loci might have been instrumental in the evolution of human brain size during the Pliocene–Pleistocene era.

## Methods

### Sequence acquisition and alignment

Full-length coding and amino acid sequences of eight MCPH genes (*CEP135, ZNF335, PHC1, CDK6, SASS6, MFSD2A, CIT, and KIF14*) for 48 eutherian species were retrieved from Ensemble and NCBI (National Center for Biotechnology Information) by using bidirectional BLAST hit strategy [56–58]. These sequence data include 20 primates and 28 nonprimate eutherian species (Additional file 1: Figure S1 and Additional file 3: Data S1).

The complete genomic sequences of archaic humans, the Neandertals and Denisovans were downloaded from Max Planck Institute for Evolutionary Anthropology website (http://www.eva.mpg.de/) in binary SAM (BAM) file format with 50 × and 30 × sequence coverage, respectively [59, 60]. MCPH gene sequences were retrived by calculating the consensus sequences of respective chromosomes from BAM (binary alignment Map) files of archaic genomes and was compared with human MCPH genes by using UGENE software [61].

The orthologous coding sequences were aligned through PRANK with default parameters for the empirical codon model by using phylogenetic information as a guide [62]. PRANK concedes insertion and deletion as a distinct evolutionary event and introduces indels instead of aligning too divergent sequences and therefore reduces the number of false positive for evolutionary analysis [63, 64].

### Analysis of substitution parameters and sites under positive selection

At the protein level, the ratio of nonsynonymous (dN) to synonymous (dS) substitution rates ω measures the selective pressure [32, 65]. The value of ω delineates the strength and direction of natural selection operating on protein-coding sequence; ω = 1, ω < 1, and ω > 1, indicate neutral evolution, negative selection and positive selection respectively. For each of eight MCPH genes within every three datasets (primates, nonprimate eutherians, all eutherians), selective pressure ω was estimated using five different codon substitution site models (M1, M2, M7, M8 and M8a) implemented in CodeML program from PAML4.7 software [32, 66, 67]. In this study, a well-accepted phylogeny of eutherians was used for each gene [68]. In order to estimate the sites under positive selection, we compared the LRT of three pairs of site models. The first pair compare the null model M1 (nearly neutral model that assume the existence of two classes of sites with ω = 1 and ω < 1) and alternative model M2 (positive selection model that assume an additional third class of site with ω > 1) [33, 67]. The other two pairs are null model M7 (beta) and alternative model M8 (beta, and ω_2_ > 1), and the last pair comparison between null model M8a (beta and ω_2_ = 1) and alternative model M8 [65, 69]. For this study, positive selection is inferred, if two out of three site pair models significantly reject the null model in the favor of alternative model. In addition, positively selected sites were identified by using Bayes empirical Bayes method implemented in M8 codon substitution site model [33].

### Analysis of episodic positive selection

To evaluate whether or not the signature of positive selection was restricted to specific lineage, we used branch site approach implemented in CodeML [34]. This model allows for ω variation not only among prespecified branches but also among sites. The branch site model allows that phylogeny can be divided into prespecified foreground branch (ω_2_ >  = 1, proportion of sites may be under positive selection) and background branch (where proportion of sites experienced either purifying selection or neutral evolution 0 < ω_2_ <  = 1). The inference of positive selection was conducted by calculating LRT between this branch site model and null model (it is the same as branch site model but with ω_2_ = 1 for foreground branch) [34]. We used eutherian phylogenetic tree as an input for the detection of episodic selection at different evolutionary time point from primate ancestral branch to human terminal branch.

### Analysis of divergent selection constraint

To determine the pattern of divergence in selective constraint across the phylogeny, we undertook the clade model C (CmC) approach implemented in CodeML [70]. Clade model C assumes that proportions of sites have evolved under divergent selective pressure but not necessarily under positive selection in two or more partition of phylogeny defined as priory. The LRT was conducted by comparing the CmC model with null model M2a-rel [71]. Both alternative CmC and null M2a-rel models have possessed three classes of sites with ω = 1, ω < 1. The third class of site in M2a-rel has single ω ratio (ω_2_ > 0) that is shared between all clades of phylogeny while CmC third class of site has ω ratios equal to the partitions of the phylogeny and varies among the partition of phylogeny [70, 71].

### Statistical analysis

P values were calculated in Chi-square program of PAMLX 1.2 package [72]. The P values of the multiple testing hypothesis were corrected for false discovery rate by using the q value package in R3.5.0 [35, 73]. The q values were calculated for each analysis, ranked in ascending order by applying the bootstrap method for π_0_ estimation and specified fdr.level = 0.05 in q value package in R3.5.0 [74].

## Supplementary Information

**Additional file 1**: Supplemental Figures.

**Additional file 2**: Supplemental Tables.

**Additional file 3.** Data S1.

## Data Availability

The datasets analyzed during the current study are available in the Ensembl database (http://www.ensembl.org), NCBI database (https://www.ncbi.nlm.nih.gov/) and Additional file 3: Data S1.

## Abbreviations

MCPH: Autosomal recessive primary microcephaly
BEB: Bayes Empirical Bayes
dN: Nonsynonymous substitutions rate
dS: Synonymous substitutions rate
LRTs: Likelihood ratio tests
BP: Basal progenitor
bRG: Basal radial glia
CmC: Clade model C
NCBI: National Center for Biotechnology Information
BAM: Binary alignment map
