# Supplementary material for: Molecular evolutionary analysis of human primary microcephaly genes
Source: BMC Ecol Evol. 2021 May 3;21:76. doi: 10.1186/s12862-021-01801-0 (PMC8091745; doi:10.1186/s12862-021-01801-0)
Supplement: Supplementary file 1 — Additional file 1: Supplemental Figures. [file 12862_2021_1801_MOESM1_ESM.pdf]

Additional Information for: Molecular evolutionary analysis of human primary microcephaly genes

Nashaiman Pervaiz[1], Hongen Kang[2], Yiming Bao[2*] & Amir Ali Abbasi[1*]

[1] National Center for Bioinformatics, Program of Comparative and Evolutionary Genomics, Faculty of Biological Sciences, Quaid-i-Azam University, Islamabad 45320, Pakistan.

[2] National Genomics Data Center & CAS Key Laboratory of Genome Sciences and Information, Beijing Institute of Genomics, Chinese Academy of Sciences, Beijing 100101, China

*Corresponding authors:

Amir Ali Abbasi: **abbasiam@qau.edu.pk**

Tel Office: +92-51-90644302

Yiming Bao: **baoym@big.ac.cn**

Tel Office: +86-10-84097858

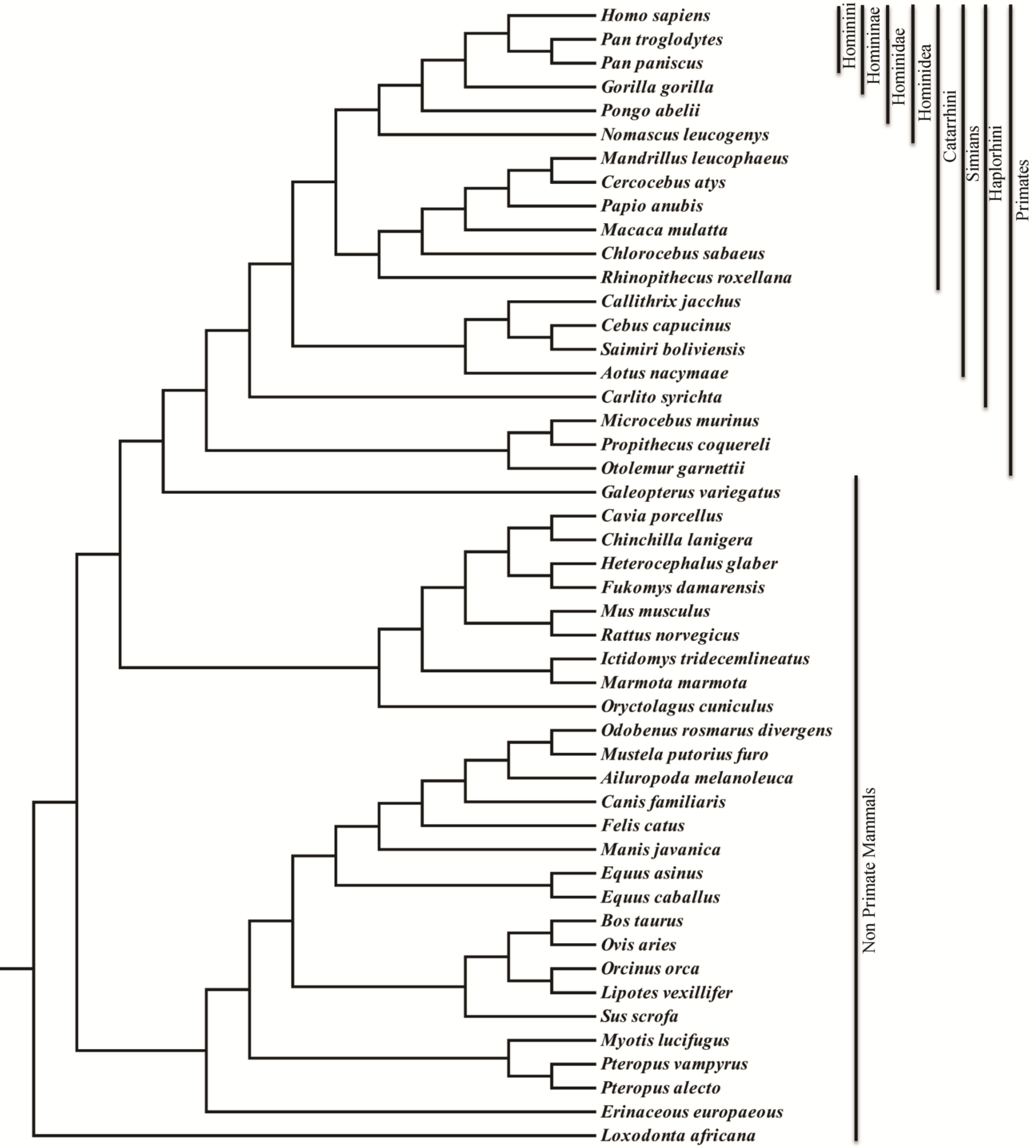

Homo sapiens
Pan troglodytes
Pan paniscus
Gorilla gorilla
Pongo abelii
Nomascus leucogenys
Mandrillus leucophaeus
Cercocebus atys
Papio anubis
Macaca mulatta
Chlorocebus sabaeus
Rhinopithecus roxellana
Callithrix jacchus
Cebus capucinus
Saimiri boliviensis
Aotus nacymaae
Carlito syrichta
Microcebus murinus
Propithecus coquereli
Otolemur garnettii
Galeopterus variegatus
Cavia porcellus
Chinchilla lanigera
Heterocephalus glaber
Fukomys damarensis
Mus musculus
Rattus norvegicus
Ictidomys tridecemlineatus
Marmota marmota
Oryctolagus cuniculus
Odobenus rosmarus divergens
Mustela putorius furo
Ailuropoda melanoleuca
Canis familiaris
Felis catus
Manis javanica
Equus asinus
Equus caballus
Bos taurus
Ovis aries
Orcinus orca
Lipotes vexillifer
Sus scrofa
Myotis lucifugus
Pteropus vampyrus
Pteropus alecto
Erinaceous europaeous
Loxodonta africana
Hominini
Homininae
Hominidae
Hominidea
Catarrhini
Simians
Haplorhini
Primates
Non Primate Mammals

Figure S1. Phylogenetic tree of placental mammals. The tree shows forty eight placental mammals genomes from primates to afrotherian that are used in this study.
