# Supplementary material for: Molecular evolutionary analysis of human primary microcephaly genes
Source: BMC Ecol Evol. 2021 May 3;21:76. doi: 10.1186/s12862-021-01801-0 (PMC8091745; doi:10.1186/s12862-021-01801-0)
Supplement: Supplementary file 2 — Additional file 2: Supplemental Tables. [file 12862_2021_1801_MOESM2_ESM.pdf]

Additional Information for: Molecular evolutionary analysis of human primary microcephaly genes

Nashaiman Pervaiz[1], Hongen Kang[2], Yiming Bao[2]* & Amir Ali Abbasi[1]*

[1] National Center for Bioinformatics, Program of Comparative and Evolutionary Genomics, Faculty of Biological Sciences, Quaid-i-Azam University, Islamabad 45320, Pakistan.

[2] National Genomics Data Center & CAS Key Laboratory of Genome Sciences and Information, Beijing Institute of Genomics, Chinese Academy of Sciences, Beijing 100101, China

*Corresponding authors:

Amir Ali Abbasi:**abbasiam@qau.edu.pk**

Tel Office: +92-51-90644302

YimingBao:**baoym@big.ac.cn**

Tel Office:+86-10-84097858

Supplementary Tables

Table S1: Likelihood ratio test statistics for three pairs of codon substitution site models comparison for eight MCPH genes.

Genes	Data sets	No. of Sequences	(M2-M1)			(M8-M7)			(M8-M8a)		
			LRT	P value	q value	LRT	P value	q value	LRT	P value	q value
**CEP135**	Primates	18	1.0864	0.58	1	8.08	0.02	0.03	2.16	0.14	0.20
	Nonprimate mammals	24	0	1	1	25.06	0.000003	0.00001	1.68	0.20	0.24
	Mammals	42	0	1	1	40.329	<0.0000001	<0.0000001	2.38	0.12	0.19
**ZNF335**	Primates	18	0	1	1	11.019	0.004	0.007	0.816	0.37	0.38
**PHC1**	Primates	20	0	1	1	0.00002	1.0	0.83	0.043	0.83	0.75
	Nonprimate mammals	26	0.00002	1	1	1.98	0.37	0.35	4.74	0.03	0.07
	Mammals	46	0	1	1	5.11	0.08	0.08	4.87	0.03	0.07
**CDK6**	Primates	17	0	1	1	0.0002	0.99	0.83	0.359	0.55	0.38
	Nonprimate mammals	26	0	1	1	6.425	0.04	0.04	0.811	0.37	0.38
	Mammals	43	0	1	1	8.098	0.02	0.03	2.02	0.16	0.21
**SAAS6**	Primates	19	3.215	0.2	1	9.46	0.009	0.01	3.027	0.08	0.13
	Mammals	44	0	1	1	25.10	0.000003	0.00001	3.19	0.07	0.13
**MFSD2A**	Primates	18	0	1	1	0.99	0.61	0.55	0.023	0.88	0.77
	Nonprimate mammals	25	0	1	1	6.91	0.03	0.04	1.85	0.17	0.21
	Mammals	43	0.00013	0.99	1	16.9	0.0002	0.0004	4.29	0.04	0.08
**CIT**	Primates	15	0.715	0.69	1	10.66	0.005	0.008	0.76	0.38	0.38
	Mammals	37	0.000002	1	1	24.4	0.000005	0.00001	0.002	0.96	0.80
**KIF14**	Nonprimate mammals	12	0	1	1	9.19	0.01	0.02	4.68	0.03	0.07

Positive selection is inferred if two out of three site model comparisons significantly reject null hypothesis using the cutoff of q value 0.05. Null models: M1, M7, and M8a, Alternative models: M2 and M8, LRT: likelihood ratio test. False discovery rate q value corrections over p values were calculated using q value package in R.

Table S2: Estimation of positive selection by using branch site model (CodeML) at various evolutionary timepoints ranging from primate ancestor to human terminal branch for eight MCPH genes

Gene	Branch	ω_2	LRT	P value	q value	Positive selected sites
**CEP135**	Human	1.0	0.0003	0.98	0.98	NS
	Hominini	1.0	0.0	1.0	0.98	NS
	Homininae	1.0	0.0001	0.99	0.98	NS
	Hominidae	1.0	0.0	1.0	0.98	NS
	Hominidea	1.0	0.00006	0.99	0.98	NS
	Catarrhini	1.0	0.0	1.0	0.98	NS
	Simians	1.0	0.0	1.0	0.98	NS
	Haplorhini	1.0	0.0	1.0	0.98	NS
	Primates	2.40	0.0488	0.83	0.98	NS
**ZNF335**	Human	1.0	0.0	1.0	0.98	NS
	Hominini	3.95	0.0305	0.86	0.98	NS
	Homininae	1.0	0.0	1.0	0.98	NS
	Hominidae	1.0	0.0	1.0	0.98	NS
	Hominidea	1.0	0.0	1.0	0.98	NS
	Catarrhini	1.0	0.0	1.0	0.98	NS
	Simians	1.0	0.0	1.0	0.98	NS
	Haplorhini	1.0	0.0	1.0	0.98	NS
	Primates	1.0	0.0	1.0	0.98	NS
**PHC1**	Human	1.0	0.0	1.0	0.98	NS
	Hominini	1.0	0.0	1.0	0.98	NS
	Homininae	1.0	0.0	1.0	0.98	NS
	Hominidae	1.0	0.0	1.0	0.98	NS
	Hominidea	1.0	0.0	1.0	0.98	NS
	Catarrhini	1.0	0.008	0.93	0.98	NS
	Simians	1.0	0.29	0.59	0.98	NS
	Haplorhini	14.93	0.468	0.49	0.98	NS
	Primates	1.0	0.00006	0.99	0.98	NS
**CDK6**	Human	1.0	0.00006	0.99	0.98	NS
	Hominini	1.0	0.0	1.0	0.98	NS
	Hominidae	2.94	0.00001	1.0	0.98	NS
	Hominidea	1.0	0.0	1.0	0.98	NS
	Catarrhini	1.0	0.0	1.0	0.98	NS
	Simians	1.0	0.0	1.0	0.98	NS
	Primates	1.0	0.0	1.0	0.98	NS
**SASS6**	Human	1.0	0.0	1.0	0.98	NS
	Hominini	4.64	0.165	0.68	0.98	NS
	Hominidae	1.0	0.092	0.76	0.98	NS
	Hominidea	1.0	0.0	1.0	0.98	NS
	Catarrhini	1.0	0.069	0.79	0.98	NS
	Simians	1.0	0.216	0.64	0.98	NS
	Haplorhini	1.0	0.00008	0.99	0.98	NS
	Primates	1.0	0.0	1.0	0.98	NS
**MFSD2A**	Human	1.0	0.0	1.0	0.98	NS
	Hominini	3.195	0.00003	1.0	0.98	NS
	Homininae	1.0	0.065	0.79	0.98	NS
	Hominidae	1.0	0.053	0.82	0.98	NS
	Catarrhini	11.65	0.266	0.61	0.98	NS
	Simians	22.54	2.129	0.14	0.98	NS
	Primates	1.0	0.00002	1.0	0.98	NS

**CIT**	Human	1.0	0.0	1.0	0.98	NS
	Hominini	1.0	0.0003	0.98	0.98	NS
	Homininae	1.0	0.0	1.0	0.98	NS
	Hominidae	1.0	0.0	1.0	0.98	NS
	Catarrhini	1.0	0.0	1.0	0.98	NS
	Simians	999	8.301	0.004	0.11	1897A**
	Haplorhini	1.06	0.0056	0.94	0.98	NS
	Primates	1.0	0.0005	0.98	0.98	NS
**KIF14**	Human	1.0	0.0	1.0	0.98	NS
	Hominini	1.0	0.0	1.0	0.98	NS
	Homininae	123.22	11.071	0.0009	0.04	619M*
	Hominidae	1.0	0.0	1.0	0.98	NS
	Hominidea	1.0	0.0	1.0	0.98	NS
	Catarrhini	3.34	0.427	0.51	0.98	NS
	Simians	1.0	0.0	1.0	0.98	NS
	Haplorhini	1.0	0.0	1.0	0.98	NS
	Primates	1.0	0.068	0.79	0.98	NS

ω2: ratio of nonsynonymous to synonymous substitutions rate for selected branch, LRT: likelihood ratio test, Positively selected sites were detected using a Bayes Empirical Bayes (BEB) method with posterior probability >= 0.95. Sites inferred under selection at the 5% cutoff are labeled with * and at 1% cutoff are labeled with **, NS: No positive selected site found.

Table S3: Chimpanzee, hominin and human specific amino acids replacements in MCPH genes since the divergence from hominini ancestor

**Gene**	Residue number	Hominini ancestor	Chimpanzee	Denisovans	Neandertals	Human
**CEP135**	581	I	V	I	I	I
	691	R	K	R	R	R
	844	A	S	A	A	A
	936	I	L	I	I	I
**ZNF335**	83	G	G	S	S	S
	294	T	T	S	S	S
	359	R	R	P	R	R
	384	P	P	R	P	P
	403	M	L	M	M	M
	770	P	P	S	S	S
	856	A	V	A	A	A
	1317	E	E	D	D	D
**PHC1**	103	I	M	I	I	I
	518	T	T	A	A	A
**SASS6**	443	V	A	V	V	V
**MFSD2A**	276	A	A	S	S	S
	290	S	R	S	S	S
	415	Q	L	Q	Q	Q
**CIT**	13	D	D	E	D	D
	78	R	R	W	W	R
	229	I	I	V	V	V
	331	T	S	T	T	T
	332	S	G	S	S	S
	338	I	V	I	I	I
**KIF14**	73	K	K	R	R	R
	204	S	N	S	S	S
	208	E	Q	E	E	E
	289	P	P	T	T	T
	321	F	L	F	F	F
	330	A	A	T	A	A
	339	E	Q	E	E	E
	395	M	M	T	T	T
	605	A	A	S	A	A
	637	I	V	I	I	I
	733	N	N	S	S	S
	1081	M	M	V	V	V
	1165	V	V	A	A	A
	1315	E	E	E	G	E
	1361	S	S	L	L	L
	1363	I	T	I	I	I
	1391	L	Q	L	L	L
	1403	N	N	N	H	N
	1408	S	G	S	S	S
	1543	S	N	S	S	S
	1624	R	R	R	H	R
