# Supplementary material for: Molecular evolutionary analysis of human primary microcephaly genes
Source: BMC Ecol Evol. 2021 May 3;21:76. doi: 10.1186/s12862-021-01801-0 (PMC8091745; doi:10.1186/s12862-021-01801-0)
Supplement: Supplementary file 3 — Additional file 3. Data S1. [file 12862_2021_1801_MOESM3_ESM.pdf]

Supplementary Information for: Molecular evolutionary analysis of human primary microcephaly genes

Nashaiman Pervaiz[1], Hongen Kang[2], Yiming Bao[2]* & Amir Ali Abbasi[1]*

[1] National Center for Bioinformatics, Program of Comparative and Evolutionary Genomics, Faculty of Biological Sciences, Quaid-i-Azam University, Islamabad 45320, Pakistan.

[2] National Genomics Data Center & CAS Key Laboratory of Genome Sciences and Information, Beijing Institute of Genomics, Chinese Academy of Sciences, Beijing 100101, China

*Corresponding authors:

Amir Ali Abbasi: **abbasiam@qau.edu.pk**

Tel Office: +92-51-90644302

Yiming Bao: **baoym@big.ac.cn**

Tel Office: +86-10-84097858

Data

CEP135

```
>Homo sapiens ENST00000257287.4
ATGACTACAGCTGTAGAGAGAAAGTATATTAATATTAGGAAAAGGCTGGATCAGCTGGGA
TACCGCCAGACTCTGACAGTGGAGTGTTTACCTTTGGTAGAAAAACTTTTCAGCGACTTA
GTTCATACAACTGAGAGCCTTCGGCAATCAAAATTATCTGCTGTGAAAGCTGAAAAAGAA
AGTGCCAATTTTGATTTTGTTTTGGAACCCTATAAACTTGAAAATGCAAGATTGAGTAGA
GAAAATAATGAATTATACCTAGAGTTAATGAAACTGAGAGAACATTCAGACCAACACGTT
AAAGAGTTGAAAACTTCATTGAAGAAATGTGCACGTGAAACAGCTGATCTGAAATTTCTG
AATAACCAATATGCTCATAAACTCAAACTGTTGGAGAAAGAGAGCAAAGCTAAGAATGAA
AGAATTCAACAACTTCAAGAAAAGAATTTGCATGCTGTAGTACAAACTCCAGGTGGCAAG
AAAAGAAGTATTGCTTTCAGGCGCCAGCGTATGCAAATTGATGAACCGGTTCCTCCCTCT
GAAGTCAGTTCATATCCAGTTCCTCAACCAGATGACCCTTACATTGCAGACCTCCTTCAA
GTGGCTGATAACAGGATTCAAGAACTTCAACAGGAAGTCCACCAGCTACAAGAAAAGTTA
GCAATGATGGAAAGTGGGGTGAGAGACTATAGCAAGCAGATTGAGCTAAGAGAACGAGAG
ATAGAACGACTGTCAGTTGCTTTGGATGGTGGTCGGTCCCCTGATGTCCTTTCTCTGGAG
TCTAGAAATAAAACCAATGAAAAGCTTATTGCTCATTTAAATATTCAGGTTGACTTTCTT
CAGCAAGCTAATAAAGACCTGGAGAAGCGTATACGAGAGCTTATGGAAACCAAGGAAACA
GTGACATCTGAAGTCGTTAATTTAAGTAACAAAAATGAAAAACTCTGCCAAGAATTAACT
GAAATAGATCAGTTAGCACAGCAGTTGGAAAGACATAAAGAAGAAGTGCTTGAGACTGCT
GATAAAGAGCTTGGGGAAGCAAAGAAAGAGATTAAAAGAAAGCTCTCTGAAATGCAGGAT
CTTGAAGAAACAATGGCAAAACTTCAGCTGGAATTGAACTTATGCCAGAAAGAAAAGGAG
AGACTGAGTGATGAACTCCTTGTAAAATCAGACCTAGAAACTGTTGTTCATCAGCTTGAA
CAAGAAAAGCAAAGACTTAGCAAAAAAGTTGAAAGTTTTGCAGTTACAGAACGACAACTT
ACTCTGGAGGTTGAGAGGATGAGACTAGAACATGGAATAAAACGTCGAGACAGGTCACCT
TCTCGTTTAGATACATTTCTGAAAGGTATAGAAGAAGAACGAGATTATTATAAGAAAGAG
CTAGAGAGACTCCAACATATAATACAGCGAAGATCTTGCTCTACAAGTTATAGCGCACGT
GAAAAAAGTTCAATATTTAGAACACCAGAAAAGGGTGATTACAATTCAGAAATTCATCAG
ATCACAAGAGAAAGAGATGAACTTCAGCGTATGCTAGAAAGATTTGAAAAATATATGGAA
GATATACAGTCCAATGTTAAATTATTGACAGCAGAAAGAGATAAACTAAGTGTCTTATAT
AATGAAGCTCAGGAAGAATTATCTGCCCTAAGAAAGGAATCCACCCAAACCACAGCACCC
CATAATATTGTTAGTCTTATGGAAAAGGAAAAAGAACTTGCGTTATCTGACTTAAGAAGA
ATTATGGCAGAAAAGGAAGCTTTAAGAGAAAAATTAGAGCATATTGAAGAAGTGAGTCTT
TTTGGAAAATCAGAATTAGAGAAAACTATTGAACATTTGACATGTGTTAATCATCAGCTT
GAAAGCGAAAAATATGAATTAAAGTCTAAAGTGTTAATAATGAAAGAAACAATAGAGTCG
TTAGAGAACAAATTAAAAGTCCAAGCTCAAAAATTTAGCCATGTGGCTGGTGACTCATCT
CATCAGAAAACAGAGGTGAACTCACTTAGGATAGTGAATGAGCAGCTACAGCGGTCAGTT
GATGACTATCAGCACCGACTTTCCATAAAAAGAGGTGAACTTGAATCAGCCCAAGCACAA
ATTAAAATACTGGAGGAAAAGATAGATGAACTAAACCTTAAGATGACTTCACAGGATGAG
GAGGCTCATGTAATGAAAAAGACCATTGGTGTTATTGATAAAGAAAAAGACTTTCTCCAG
GAGACTGTAGATGAGAAGACAGAAAAGATTGCAAATTTGCAAGAAAACCTAGCTAATAAA
GAAAAAGCTGTTGCTCAAATGAAGATAATGATCTCAGAGTGTGAATCATCTGTGAACCAG
CTGAAAGAAACATTGGTTAATCGAGATCGTGAGATAAACAGCCTCCGGCGCCAGCTTGAT
GCAGCTCACAAAGAACTCGATGAAGTAGGAAGATCTAGAGAAATCGCTTTTAAGGAAAAC
AGAAGACTGCAAGATGACCTGGCTACAATGGCAAGAGAAAATCAAGAAATCTCATTGGAA
```

TTGGAAGCAGCAGTGCAAGAAAAAGAAGAAATGAAGAGCAGAGTTCATAAATACATAACA
GAGGTGTCACGATGGGAGAGCTTAATGGCTGCCAAGGAAAAAGAAAATCAAGATTTGTTA
GATAGATTTCAGATGCTTCATAACCGTGCTGAAGACTGGGAGGTCAAAGCCCATCAAGCT
GAGGGAGAAAGCAGCTCAGTTCGACTGGAACTTCTTTCTATTGACACTGAGAGGAGACAT
CTTCGAGAAAGAGTGGAGCTATTAGAAAAAGAAATTCAAGAGCACATAAATGCCCATCAT
GCTTATGAATCTCAGATCTCATCAATGGCAAAAGCCATGTCTCGATTAGAAGAAGAGCTG
AGACATCAAGAAGATGAGAAAGCAACAGTATTAAATGACTTGTCATCTCTTAGAGAACTT
TGCATTAAACTTGATTCAGGCAAAGATATTATGACCCAGCAATTGAATTCGAAAAACCTT
GAGTTTGAGAGGGTTGTGGTGGAATTAGAAAATGTAAAGTCAGAGTCAGACCTACTGAAA
AAACAACTTTCAAATGAGAGACATACAGTTAAAAACCTCGAATCATTGTTGGCTACAAAC
AGAGATAAAGAATTTCATTCTCACTTAACCTCCCACGAGAAGGATACAGAAATCCAGCTA
CTTAAGGAGAAGTTAACCCTTTCTGAAAGCAAATTAACTAGTCAAAGCCGGGAAAACACC
ATGCTTCGAGCTAAAGTGGCACAGTTACAAACAGATTATGATGCTCTGAAAAGGCAGATC
TCAACTGAAAGATACGAACGAGAACGAGCAATCCAAGAGATGCGTCGACATGGTCTTGCT
ACACCACCCCTTAGTTCCACTCTGAGGTCTCCTTCACATTCTCCTGAACATAGAAATGTG
>marmoset ENSCJAT00000035495.2
ATGACTACAGCTGTAGAAAGAAAGTATATTAATATTAGAAAAAGACTGGATCAGCTGGGA
TACCGCCAGACTCTGACAGTGGAGTGTTTACCTTTGGTAGAAAAACTTTTCAGTGACTTA
GTTCATACAACAGAGAGCCTTCGGCAATCAAAATTATCTGCTGTGAAAGCTGAAAAAGAA
AGTGCCAATTTTGATTTTGTTTTGGAACCCTATAAACTTGAAAATGCAAGATTGAGTAGA
GAAAATAATGAATTGTACCTAGAGTTAATGAAACTGAGAGAACATTCAGACCAACGCATT
AAAGAGTTGAAAACTTCATTGAAGAAGTGTGCACGTGAAACATCTGATCTGAAATTTCTA
AATAACCAATATGTTCATAAACTCAAACTATTGGAGAAAGAGAGCAAAGCTAAGAATGAA
AGAATTCAACAACTTCAAGAAAAGAATTTGCATGCTGTAGTACAAACTCCAGGTGGCAAA
AAAAGAAGTATTGCTTTCAGGCGCCAGCGTATGCAAATTGATGAACCGGTTCCTCCCTCT
GAAGTCAGTTCATATCCAGTTCCGCAGCCAGATGACCCTTACATTGCAGACCTCCTACAA
GTGGCTGATAACAGGATTCAAGAACTTCAACAGGAAGTGCACCAGCTACAAGAAAAGTTA
GCAGTGATGGAAAGTGGGGTGAGAGATTATAGCAAGCAGATTGAGCTACGAGAACAAGAG
ATAGAACGATTGTCAGTTGCTATGGATGGTGGTCGTTCTCCTGATGTCCTCTCTCTGGAG
TCTAGAAATAAGACCAATGAAAAGCTTATTGCTCAGTTAAATATTCAGGTTGACTTTCTT
CAGCAAGCTAATAAAGACCTGGAGAAGCATATACAGGAGCTCACGGAAACTAAGGAAACA
GTGACATCTGAAGTTGTTAATTTAAGTAACAAAAATGAAAAACTCTGCCAAGAATTAACT
GAAATAGATCAGTTAGCACAGCAGTTGGAAAGACATAAAGAAGAAGTGCTTGAGACTGCT
GATAAAGAGCTTGGGGAAGCAAAGAAAGAGATTAAAAGAAAGCTCTCTGAAATGCGGGAT
CTTGAAGAAACAATGGCAAAACTTCAACTGGAATTGAACTTATGCCATAAAGAAAAGGAG
AGACTAAGTGATGAACTCCTTGTAAAATCAGATCTAGAAACTGTTGTTCATCAGCTTGAA
CAAGAAAAGCAAAGACTTAGCAAAAAAGTCGAAAATTTTGCAGTTACAGAAAGAGAACTT
ACTTTGGAAGTTGAGAGGATGAGACTAGAACATGGAATAAAACGTCGAGACAGGTCACCT
TCTCGTTTAGATACATTTCTGAAAGGTATAGAAGAAGAACGAGATTATTATAAGAAAGAG
CTAGAGAGACTCCAACATATAATACAGCGAAGATCTTGCTCTGCAAATTATAGCGCACGT
GAAAAAAGTTCAATATTTAGAACATCAGAAAAGGGTGATTACAATTCAGAAATTCATCAG
ATCACAAGAGAAAGAGATGAACTTCAACATATGCTAGAAAGATTTGAAAAATATATGGAG
GATATACAGTCCAATATTAAATTATTGACAGCAGAAAGAGATAAACTAAGTGTCTTATAT
AATGAAGCTCAGGAAGAATTATCTGCCCTAAGACAGGAATCCACCCAAACTACAACACCC
CATAATATTGTTAGTCTTATGGAAAAGGAAAAAGAACTTGCATTATCTGACTTAAGAAGA
ATTATGACAGAAAAGGAAGCTTTAAAAGAAAAATTAGAGCATATCGAGGAAATGGATCTT
TATGGAAAATCAGAATTAGAGAAAACTATTGAACATTTGACATGTGTTAATCATCAGCTT

GAAAGCGAAAAATATGAATTAAAGTCTAAAGTGTTAATAATGAAAGAAACAATAGAGTCA
TTAGAGAACAAATTAAAAGTCCAAGCTCAAAAATTTAGCCATGTGGCTGGTGACTCATCT
CATCAGAAAACGGAGGTGAACTCACTTAGGATGGTGAATGAGCAGCTACAGCAGTCACTT
GATGACTGTCAGCACCGACTTTCCATAAAAAGGAGTGAACTTGACTCAGCCCAAGCACAA
ATTAAAATACTGGAGGAAAAAATAGATGAACTAAACCTTAAGATGACTTCACAGGATGAG
GAGGCTCATGTAATGAAAAAGACCATTGGTGTTATTGATAAAGAAAAAGACTTTCTCCAG
GATACTGTAGATGAGAAGACAGAAGAGATCGCAAATTTGCAAGAAAACCTAGCTAATAAA
GAAAAAGCTATTGCTCATATGAAGATAATGGTCTCAGAGTGTGAATCATCTATGAACCAG
CTAAAGGAAACACTGACTAATCGAGACCGTGAGATAAACAGTCTCCGGCGCCAGCTTGAT
GCAGCTCACAAAGAACTCGATGAAGTAGGAAGATCTAGAGAAATCGCTTTTAAGGAAAAC
AGAAGATTGCAAGATGATCTGGTTACAATGGCAAGAGAAAACCAAGAAATCTCACTGGAA
TTGGAAGCAGCAGTGCAAGAAAAAGAAGAAATGAAGAGCAGAGTTCATAAATACATAACA
GAGGTGTCACGATGGGAGAGCTTAATGGCTGCTAAGGAAAAAGAAAATCAAGATTTGTTA
GACAGATTTCAGATGGTTCATAACCGTGCTGAAGACTGGGAGGTCAAAGCCCATCAAGCT
GAGGGAGAAAGCAGCTCAGTTCGGCTGGAACTTCTTTCTATTGACACTGAGAGGAGACAT
CTTCGAGAAAGAGTGGAGCTGTTAGAGAAAGAAATTCAAGAGCACATAAATGCCCATCAT
GCTTATGAATCTCAGATCTCGTCAATGGCAAAAGCCATGTCTCGATTAGAAGAAGAGCTG
AGACGTCAAGAAGAGGAGAAAGTGACAGTATTAAATGACTTGTCATCTCTTAGAGAACTT
TGCATTAAACTTGATTCAGGCAAAGATATTATGACCCAGCAACTGAATTCCAAAAACCTT
GAGTTTGAGAGGGTTATGGTAGAATTAGAAAATGTAAAGTCAGAGTCAGACCTATTGAAA
AAACAACTGTCAAATGAGAGACATACAGTTAAAAACCTTGAATCATTGTTGGCTACAAAC
AGAGATAAAGAATTTCATTCTCACTTAACCTCCCATGAGAAGGATACAGAAATCCAGCTA
CTTAAGGAGAAATTAACCCTTTCTGAAAGCAAATTAACTAGTCAAAGCCGGGAAAATACC
ATGCTTCGGGCTAAAGTGGCACAGTTACAAACAGATTATGATGCTCAGAAAAGACAGATC
TCAACTGAGAGATACGAACGAGAACGAGCAATCCAAGAGATGCGTCGACATGGTCTTCCT
ACACCACCCCTTAGTTCTACTCTGAGGTCTCCTTCACATTCTCCTGAACGTATCAATGTG

>gorilla ENSGGOT00000003706.2
ATGACTACAGCTGTAGAAAGAAAGTATATTAATATTAGGAAAAGACTGGATCAGCTGGGA
TACCGCCAGACTCTGACAGTGGAGTGTTTACCTTTGGTAGAAAAACTTTTCAGCGACTTA
GTTCATACAACTGAGAGCCTTCGGCAATCAAAATTATCTGCTGTGAAAGCTGAAAAAGAA
AGTGCCAATTTTGATTTTGTTTTGGAACCCTATAAACTTGAAAATGCAAGATTGAGTAGA
GAAAATAATGAATTATACCTAGAGTTAATGAAACTGAGAGAACATTCAGACCAACATGTT
AAAGAGTTGAAAACTTCATTGAAGAAATGTGCACGTGAAACAGCTGATCTGAAATTTCTG
AATAACCAATATGCTCATAAACTCAAACTGTTGGAGAAAGAGAGCAAAGCTAAGAATGAA
AGAATTCAACAACTTCAAGAAAAGAATTTGCATGCTGTAGTACAAACTCCAGGTGGCAAG
AAAAGAAGTATTGCTTTCAGGCGCCAGCGTATGCAAATTGATGAACCGGTTCCTCCCTCT
GAAGTCAGTTCATATCCAGTTCCTCAACCAGATGACCCTTACATTGCAGACCTCCTTCAA
GTGGCTGATAACAGGATTCAAGAACTTCAACAGGAAGTCCACCAGCTACAAGAAAAGTTA
GCAATGATGGAAAGTGGGGTGAGAGACTATAGCAAGCAGATTGAGCTAAGAGAACGAGAG
ATAGAACGACTGTCAGTTGCTTTGGATGGTGGTCGGTCCCCTGATGTCCTTTCTCTGGAG
TCTAGAAATAAAACCAATGAAAAGCTTATTGCTCATTTAAATATTCAGGTTGACTTTCTT
CAGCAAGCTAATAAAGACCTGGAGAAGCGTATACAAGAGCTTATGGAAACCAAGGAAACA
GTGACATCTGAAGTCGTTAATTTAAGTAACAAAAATGAAAAACTCTGCCAAGAATTAACT
GAAATAGATCAGTTAGCACAGCAGTTGGAAAGACATAAAGAAGAAGTGCTTGAGACTGCT
GATAAAGAGCTTGGGGAAGCAAAGAAAGAGATTAAAAGAAAGCTCTCTGAAATGCGGGAT
CTTGAAGAAACAATGGCAAAACTTCAGCTGGAATTGAACTTATGCCAGAAAGAAAAGGAG

AGACTGAGTGATGAACTCCTTGTAAAATCAGACCTAGAAACTGTTGTTCATCAGCTTGAA
CAAGAAAAGCAAAGACTTAGCAAAAAAGTTGAAAGTTTTGCAGTTACAGAACGACAACTT
ACTCTGGAGGTTGAGAGGATGAGACTAGAACATGGAATAAAACGTCGAGACAGGTCACCT
TCTCGTTTAGATACATTTCTGAAAGGTATAGAAGAAGAACGAGATTATTATAAGAAAGAG
CTAGAGAGACTCCAACATATAATACAGCGAAGATCTTGCTCTACAAGTTATAGCGCACGT
GAAAAAAGTTCAATATTTAGAACACCAGAAAAGGGTGATTACAATTCAGAAATTCATCAG
ATCACAAGAGAAAGAGATGAACTTCAGCGTATGCTAGAAAGATTTGAAAAATATATGGAA
GATATACAGTCCAATGTTAAATTATTGACAGCAGAAAGAGATAAACTAAGTGTCTTATAT
AATGAAGCTCAGGAAGAATTATCTGCCCTAAGAAAGGAATCCACCCAAACCACAGCACCC
CATAATATTGTTAGTCTTATGGAAAAGGAAAAAGAACTTGCGTTATCTGACTTAAGAAGA
ATTATGGCAGAAAAGGAAGCTTTAAGAGAAAAATTAGAGCATATTGAAGAAGTGAGTCTT
TTTGGAAAATCAGAATTAGAGAAAACTATTGAACATTTGACATGTGTTAATCATCAGCTT
GAAAGCGAAAAATATGAATTAAAGTCTAAAGTGTTAATAATGAAAGAAACAATAGAGTCG
TTAGAGAACAAATTAAAAGTCCAAGCTCAAAAATTTAGCCATGTGGCTGGTGACTCATCT
CATCAGAAAACAGAGGTGAACTCACTTAGGATAGTAAATGAGCAGCTACAGCGGTCAATT
GATGACCATCAGCACCGACTTTCCATAAAAAGAGGTGAACTTGAATCAGCCCAAGCACAA
ATTAAAATACTGGAGGAAAAGATAGATGAACTAAACCTTAAGATGACTTCACAGGATGAG
GAGGCTCATGTAATGAAAAAGACCATTGGTGTTATTGATAAAGAAAAAGACTTTCTCCAG
GAGACTGTAGATGAGAAGACAGAAAAGATTGCAAATTTGCAAGAAAACCTAGCTAATAAA
GAAAAAGCTGTTGCTCAAATGAAGATAATGATCTCAGAGTGTGAATCATCTGTGAACCAG
CTGAAAGAAACATTGGTTAATCAAGATCGTGAGATAAACAGCCTCCGGCGCCAGCTTGAT
GCAGCTCACAAAGAACTCGATGAAGTAGGAAGATCTAGAGAAATCGCTTTTAAGGAAAAC
AGAAGACTGCAAGATGACCTGGCTACAATGGCAAGAGAAAACCAAGAAATCTCATTGGAA
TTGGAAGCAGCAGTGCAAGAAAAAGAAGAAATGAAGAGCAGAGTTCATAAATACATAACA
GAGGTGTCACGATGGGAGAGCTTAATGGCTGCCAAGGAAAAAGAAAATCAAGATTTGTTA
GACAGATTTCAGATGCTTCATAACCGTGCTGAAGACTGGGAGGTCAAAGCCCATCAAGCT
GAGGGAGAAAGCAGCTCAGTTCGACTGGAACTTCTTTCTATTGACACTGAGAGGAGACAT
CTTCGAGAAAGAGTGGAGCTATTAGAAAAAGAAATTCAAGAGCACATAAATGCCCATCAT
GCTTATGAATCTCAGATCTCATCAATGGCAAAAGCCATGTCTCGATTAGAAGAAGAGCTG
AGACATCAAGAAGATGAGAAAGCAACAATATTAAATGACTTGTCATCTCTTAGAGAACTT
TGCATTAAACTTGATTCAGGCAAAGATATTATGACCCAGCAATTGAATTCGAAAAACCTT
GAGTTTGAGAGGGTTGTGGTGGAATTAGAAAATGTAAAGTCAGAGTCAGACCTACTGAAA
AAACAACTGTCAAATGAGAGGCATACAGTTAAAAACCTCGAATCATTGTTGGCTACAAAC
AGAGATAAAGAATTTCATTCTCACTTAACCTCCCATGAGAAGGATACAGAAATCCAGCTA
CTTAAGGAGAAGTTAACCCTTTCTGAAAGCAAATTAACTAGTCAAAGCCGGGAAAACACC
ATGCTTCGAGCTAAAGTGGCACAGTTACAAACAGATTATGATGCTCTGAAAAGGCAGATC
TCAACTGAAAGATACGAACGAGAACGAGCAATCCAAGAGATGCGTCGACATGGTCTTGCT
ACACCACCCCTTAGTTCCACTCTGAGGTCTCCTTCACATTCTCCTGAACATATAAATGTG

>mouse lemur ENSMICT00000050751.1
ATGACTACAGCTGTAGAGAGAAAGTATGTTCATATTAGAAAAAGATTGGATCAGCTGGGA
TATCGCCAGACTCTGACAGTGGAATGTTTACCTTTAGTAGAAAAACTTTTCAGTGACCTA
GTTCATACAACAGAAAGCCTTCGGCAGTCAAAATTATCTGCTGTAAAAGCAGAAAAAGAA
AGTGCCAATTTTGATTTTGTTTTGGAACCCTATAAAGTTGAAAATGGAAGATTGAGTAGG
GAAAATAATGACTTATACCTGGAGTTAATGAAACTGAGAGAACTCTCAGACCAACACATT
AAAGAGTTGAAAACTACAGTGAAGAAGTGTGCACGTGAAACTGCTGATCTAAAATTTCTA
AATAACCAATATGTTCATAAACTCAAACTTTTGGAGAAAGAAAGCAAAGCTAAGAATGAA

AAAATTCAACAACTTCAAGAAAAGAATTTGCAAGCTGTAGTACAAACTCCAGGTGGCAAG
AAAAGAAATATTGCTTTTAGGCGCCAGCGTATGCAAATTGATGAGCCAGTCCCTCCCTCT
GAAGTAAGCTCCTATCCAGTTCCACAGCCAGATGACCCTTACATTGCCGACCTCCTGCAA
GTGGCTGATAACAGGATTCAAGAACTTCAACAGGAAGTCCACCAGTTACAAGAGAAGTTA
GCAGTGATGGAAAGTGGAGTGAGAGATTATAGCAAGCAGATTGAGCTAAGAGAACGAGAG
ATAGAACGACTGTCCGTTGCTTTGGATGGTGGTCGCTCCCCTGATGTCCTCTCTCTGGAG
TCCAGAAATAAAGCCAATGAAAAGCTTATTGCTCATTTAAATATTCAGGTTGACTTTCTT
CAGCAAGCTAATAAAGACCTGGAGAAGAATATACAAGAGCTTATGGAAACCAAGGAAACG
GTGACATCTGAAGTTGTTAATTTAAGTGAAAGAAATGAAAAACTCTGCCAAGAATTAACT
GAAATAGACCATTTAGCACAACAGTTGGAAAGACATAAGGAAGAAGTCCTTGAGACTGCT
GATAAAGAACTTGAGGAAGCAAAGAAAGAGATTAAAAGAAAGCTCTCTGAAATGCGAGAT
CTGGAAGAAACAGTGGCGAAGCTCCAACTGGAATTAGAGTTATGCAATAAAGAAAAGGAT
AAGCTCAATGATGAACTCCATATAAAATCAGACCTGGAAGCTGTTGTTCATCAGCTTGAA
CAAGAAAAGCACAGACTTAGTAAAAAAGTGGAAAGTTTTACAGTTACAGAAAGAGAACTA
ACTTTGGAAGTTGAGAGGATGAGACTAGAACATGGAATAAAACGTCGAGACAAGTCACCT
TCTCGTTTAGATACATTTCTGAAAGGTATAGAAGAAGAACGAGATTACTATAAGAAAGAG
CTAGAAAAAGTCCAACATATAATACACCGACGATCTTGCCCTTTAAGTTATAGTGTGCGT
GAAAAAAGTTCAATATATAGAACACCAGAAAAGGGTGATTACAATTCAGACATTCATCTG
ATCACAAGAGAAAGAGATGAACTTCAGCGTATGGTAGAAAGATTTGAAAAATATATGGAG
GATATACAGTCCAATGTTAAATTACTGACATCAGAAAGAGATAAACTAAGTGTCTTATAT
AATGAAGCTAAGGAAGAATTATCAGCCCTAAGACAGGAATCCAGCCGAAGTACAGCCTCC
CATAATATTGTTAGTCTTATGGAAAAGGAAAAAGAACTTGCATTGTCTGACTTAAGAAGA
ATTATGGCAGAAAAGGAAGACTTAAGGGAAAAGTTAAAAAATATCCAGGAAATGGGTCTT
CATGGAAAGTCAGAATTAGAGAAAACTATTGAACATTTGAAATATGTTAATCATCAGCTT
GAAAGCGAAAAATATGAATTAAAGGCTAAAATGTTATTAATGAAAGAAACAATAGAGTCA
TTAGAGAACAAATCAAAACTCCATGCTCAAAAACTTAGCCATGTAATTTGTGGTGACTCA
TCTCATCAGAAGGCAGAGGTGAACTCACTTAGGATGGTAAATGAGCAGCTACAGCGGTCG
CTTGATGACTATCAGCATCGACTTTCCCTAAAAAGAGGTGAACTTGAATCAGCCCAAGCA
CAAATTAAAACACTGGAGGAAAAAATAGATAAACTAAACCTTAAGATGACTTCACAGGAT
GAGGAGGCTCATGTAATGAAAAAGACAATAGGTGTTATTGATAAAGAAAAAGACTTTCTT
CAGGACACTGTAGATGAGAAGACAGAAAAGATCGCAAATTTGCAAGAAAACATAGTTACT
AAAGAAAAAACTATTGCTCAGATGACGCTAACAGTCTCAGAGTGTGAATTATCTATGAAC
CAGCTAAAGGAAACATTGACTAATCGAGAACGTGAGATAGGCAGCCTCCGGCGCCAGCTT
GATTCAGCTCACAAAGAACTTGATGAAGTAGGAAGATCGAGAGAAATATCTTTTAAGGAA
AACAGAAGATTACAAGATGATCTGGCTACAATGGCAAGAGAAAACCAGGAAATCTCATTG
GAATTGGAAGCAGCAGTGCAAGAAAAAGAAGATATGAAGAGTAGAGTTCATAATTACATA
ACTGAGGTGTCACGATGGGAGAGCTTAATGGCTGCTAAGGAAAAAGAAAATCAAGATTTG
TTGGATAGATTTCAGATGCTCCATCACCGGGCTGAAGACTGGGAGGTGAAAGCCCAGCAG
GCTGAGGGAGAAAGCAGCTCTGTTCGGCTGGAACTTCTTTCTATCGACACAGAGAGGAGA
CACCTGCGAGAAAGAGTGGAGTTATTAGAAAAAGAAATTCAAGAGCACATAAATGCCCAT
CATGCTTATGAATCTCAGATCTCATCAATGGCAAAAGCCATGTCTAGATTAGAAGAAGAG
CTGAGACATCTAGAAGATGAGAAAGCATCAGTTTTGAATGATCTGTCATCTCTTAGAGAA
CTCTGCATTAAGCTTGATTCAGGCAAAGATATTATGACCCAACAATTGAATTCCAAACAC
CTTGAGTTTGAGAGGGTTGCAGTGGAATTAGAAAATGTAAAATCGGAATCAGACCTGTTA
AAAAAACAACTGGCAAATGAGAGACATACAGTTAAAAACCTCGAATCATTGTTGGCTACA
AATAGAGACAAAGAATTTCATTGTCATTTAACCTCCCATGAGAAGGATACAGAAATTCAG
CTACTTAAAGAGAAGTTAAGCCTTTCAGAAAGCAAACTAACTACTCAAAGCCGGGAAAAT

ACCATGCTTCGAGCTAAAGTGGTACAGTTACAAGCAGATTTTGATGCTTTGAAACGACAG
ATTTCAGCTGAAAGATATGAACGGGAACGAGCAATCCAAGAGATGCGCCGCCAGGGTCTT
CCCACGCCGCCCTTTAGTTCCACTCTGAGGTCTCCTTCACCTTCTCCGGATCATCTAAAT
GCG
>gibbon ENSNLET00000011086.1
ATGACTACAGCTGTAGAAAGAAAGTATATTAATATTAGGAAAAGACTGGATCAGCTGGGA
TACCGCCAGACTCTGACAGTGGAGTGTTTACCTTTGGTAGAAAAACTTTTCAGCGACTTA
GTTCATACAACTGAGAGCCTTCGGCTATCAAAATTATCTGCTGTGAAAGCTGAAAAAGAA
AGTGCCAATTTTGATTTTATTTTGGAACCCTATAAACTTGAAAATGCAAGATTGAGTAGA
GAAAATAATGAATTATACCTAGAGTTAATGAAACTGAGAGAACATTCAGACCAACACGTT
AAAGAGTTGAAAACTTCATTGAAGAAATGTGCACGTGAAACAGCTGATCTGAAATTTCTA
AATAACCAATATGTTCATAAACTCAAACTGTTGGAGAAAGAGAGCAAAGCTAAAAATGAA
AGAATTCAACAACTTCAAGAAAAGAATTTGCATGCTATAGTACAAACTCCAGGTGGCAAG
AAAAGAAGTATTGCTTTCAGGCGCCAGCGTATGCAAATTGATGAACCGGTTCCTCCCTCT
GAAGTCAGTTCATATCCAGTTCCTCAACCAGATGACCCTTACATTGCAGACCTCCTACAA
GTGGCTGATAACAGGATTCAAGAACTTCAACAGGAAGTCCACCAGCTACAAGAAAAGTTA
GCAATGATGGAAAGTGGGGTGAGAGACTATAGCAAGCAGATTGAGCTAAGAGAACGAGAG
ATAGAACGACTGTCAGTTGCTTTGGATGGTGGTCGGTCCCCTGATGTCCTCTCTCTGGAG
TCTAGAAACAAAACCAATGAAAAGCTTATTGCTCATTTAAATATTCAGGTTGACTTTCTT
CAGCAAGCTAATAAAGACCTGGAGAAGCGTATACGAGAGCTTATGGAAACCAAGGAAACA
GTGACATCTGAAGTCGTTAATTTAAGTAACAAAAATGAAAAACTCTGCCAAGAATTAACT
GAAATAGATCAGTTAGCACAGCAGTTGGAAAGACGTAAAGAAGAAGTGCTTGAGACTGCT
GATAAAGAGCTTGGGGAAGCAAAGAAAGAGATTAAAAGAAAGCTCTCTGAAATGCGGGAT
CTTGAAGAAACAATGGCAAAACTTCAACTGGAATTGAACTTATGCCAGAAAGAAAAGGAG
AGACTGAGTGATGAACTCCTTGTAAAATCAGACCTAGAAACTGTTGTTCATCAGCTTGAA
CAAGAAAAGCAAAGACTTAGCAAAAAAGTTGAAAGTTTTGCAGTTACAGAACGACAACTT
ACTCTGGAAGTTGAGAGGATGAGACTAGAACATGGAATAAAACGTCGAGACAGGTCACCT
TCTCGTTTAGATACGTTTCTGAAAGGTATAGAAGAAGAACGAGATTATTATAAGAAAGAG
CTAGAGAGACTCCAACATATAATACAGCGAAGATCTTGCTCTACAAGTTATAGCACACGT
GAAAAAAGTTCAACATTTAGAACATCAGAAAAGGGTGATTACAATTCAGAAATTCATCAG
ATCACAAGAGAAAGAGATGAACTTCAGCGTATGCTAGAAAGATTTGAAAAATATATGGAG
GATATACAGTCCAATGTTAAATTATTGACAGCAGAAAGAGATAAACTAAGTGTCTTATAT
AATGAAGCTCAGGAAGAATTATCTGCCCTAAGAAAGGAATCCACCCAAACCACAGCACCC
CATAATATTGTTAGTCTTATGGAAAAGGAAAAAGAACTTGCGTTATCTGACTTAAGAAGA
ATTATGGCAGAAAAGGAAGCTTTAAGAGAAAAATTAGAGCATATTGAAGAAATGAATCTT
TTTGGAAAATCAGAATTAGAGAAAACTATTGAACATTTGACATGTGTTAATCATCAGCTT
GAAAGCGAAAAATATGAATTAAAGTCTAAAGTGTTAATAATGAAAGAAACAATAGAGTCG
TTAGAGAACAAATTAAAAGTCCAAGCTCAAAAATTTAGCCATGTGGCTGGTGACTCATCT
CATCAGAAAACAGAGGTGAACTCACTTAGGATAGTAAATGAGCAGCTACAGCGGTCAGTT
GATGACTGTCAGCACCGACTTTCCATAAAAAGAGGTGAACTTGAATCAGCCCAAGCACAA
ATTAAAATACTGGAGGAAAAGATAGATGAACTAAACCTTAAGATGACTTCACAGGATGAG
GAGGCTCATGTAATGAAAAAGACCATTGGTGTTATTGATAAAGAAAAAGACCTTCTCCAG
GAGACTGTAGATGAGAAGACAGAAGAGATTGCAAATTTGCAAGAAAACCTAGCTAATAAA
GAAAAAGCTGTTGCTCAAATGAAGATAATGATCTCAGAGTGTGAATCGTCTGTGAACCAG
CTGAAAGAAACATTGGTTAATCGAGATCGTGAGATAAACAGCCTCCGGCGCCAGCTTGAT
GCAGCTCACAAGGAACTCGATGAAGCAGGAAGATCTAGAGAAATCGCTTTTAAGGAAAAC
AGAAGATTGCAAGATGACCTGGCTACAATGGCAAGAGAAAACCAAGAAATCTCATTGGAA

TTGGAAGCAGCAGTGCAAGAAAAAGAAGAAATGAAGAGCAGAGTTCATAAATACATAACA
GAGGTGTCACGATGGGAGAGCTTAATGGCTGCCAAGGAAAAAGAAAATCAAGATTTGTTA
GATAGATTTCAGATGCTTCATAACCGTGCTGAAGACTGGGAGGTCAAAGCCCATCAAGCT
GAGGGAGAAAGCAGCTCAGTTCGACTGGAACTTCTTTCCATTGACACTGAGAGGAGACGT
CTTCGAGAAAGAGTGGAGCTATTAGAAAAAGAAATTCAAGAGCACATAAATGCCCATCAT
GCTTACGAATCGCAGATCTCATCAATGGCAAAAGCCATGTCTCGATTAGAAGAAGAGCTG
AGACATCAAGAAGATGAGAAAGCAACAGTATTAAATGACTTGTCATCTCTTAAAGAACTT
TGCATTAAACTTGATTCAGGCAAAGATGTTATGACCCAGCAATTGAATTCGAAAAACCTT
GAGTTTGAGAGGGTTGTGGTGGAACTAGAAAATGTAAAGTCAGAGTCAGACCTACTGAAA
AAACAACTGTCAAATGAGAGACATACAGTTAAAAACCTTGAATCATTGTTGGCTACAAAC
AGAGATAAAGAATTTCATTCTCACTTAACCTCCCGTGAGAAGGATACAGAAATCCAGCTA
CTTAAGGAGAAGTTAACCCTTTCCGAAAGCAAATTAACTAGTCAAAGCCGGGAAAACACC
ATGCTTCGAGCCAAAGTGGCGCAGGTACAAACAGATTATGATGCTCTGAAAAGGCAGATC
TCAACTGAAAGATACGAACGAGAACGAGCAATCCAAGAGATGCGTCGACATGGTCTTGCT
ACACCACCCCTTAGTTCCACTCTGAGGTCTCCTTCACATTCTCCTGAAAATATAAATGTG

>bushbabby ENSOGAT00000014653.2
ATGACTACAGCTATAGAAAGAAAGTATGCTCATATTAGAAAAAGATTGGATCAGCTGGGT
TACCGCCACACTCTGACAGTGGAGAGTTTACCGTTAGTAGAAAAACTTTTCAGTGACCTA
GTTCATACAACAGAAAGCCTTCGGCAGTCAAAATTATCTACTGTGAAAGCTGAAAAAGAC
AGTGCCAATTTTGATTTTGTTTTGGAACCCTATAAACTTGAAAATACAAGGTTGAGTAAG
GAAAATAATGAATTATACCTGGAGTTAATGAAACTGAGAGAACACTCCGACCAACACATT
AAAGAGTTGAAAACTACACTGAAGAAGAATACACGTGAAACATCTGATCTCAAATTTCTA
AATACCCAGTATGTTCATAAACTCAAACTTTTGGAGAAAGAGAGCAAAGCTAAGAATGAA
AAAATTCAACAACTTCAAGAAAAGAATTTACAAGCTGTAGTCCAAACTCCTGGTGGCAAG
AAAAGAAGCATTGCTTTTAGGCGGCAGCGTATGCAAATTGATGAACCAGTTCCTCCCTCT
GAAGTCAGTTCCTATCCAGTTCCCCAGCCAGAAGACCCTTACATTGCAGATCTCCTGCAA
GTGGCTGATAACAGGATTCAAGAACTTCAACAGGAAGTCCACCAGTTACAAGAAAAGTTA
GCAGTGATGGAAAATGGAGTGATAGATTATAGCAAGCAGATTGAGCTAAGAGAACGAGAG
ATAGAACGACTGTCAGTTGCTTTGGATGGTGGTCGCTCCCCTGATGTCCTGTCTCTGGAG
TCTAGAAATAAAGCCAATGAAAAGCTTATTGCTCATTTAAATATTCAGGTTGACTTTCTT
CAGCAAGCTAATAAGGACCTGGAGAAGCGTATGCAAGAGCTTATGGAAAGCAAGGAAACA
GTTACATCTGAAGTTGTTAATTTAAGTAACAAAAATGAAAAACTGTGCCAAGAATTAACC
GAAATAGACCAGCTAGCACAGCAGTTACAAAGACATAAAGAAGAAGTGCTCCTGACCGCT
GACAAGGAGCTGGGGGAAGCAAAGAAAGAGATTAAAAGAAAATTCTTTGAAATGCGGGAT
CTTGAAGAAACAGTTGCCAAACTTGAACTGGAATTAAACTTATGCAATAAAGAAAAGGAG
AGACTGAGTGAGGAACTCGTTGTAAAATCAGACCTGGAAACTGTTGTTTATCAGCTTGAA
CAAGAAAAGCAAAGACTTAGCAAAAAAATGGAAAATTTTGCAGTTACAGAAAAGGAACTA
ACTTTGGAAGTTGAAAGGATGAGACTAGAACACGGAATAAAACGTCGAGACCGGTCACCT
TCTCGTTTAGATACATTTCTGAAAGGTATAGAAGAAGAACGAGATTATTATAAGAAAGAG
CTAGAAAGACTCCAGCATGCAATACAGCGACGATCTTGCTCTATACATTATAATACACGT
GAAAAAAGTTCAAGTAAAACCCCAGAAAAGGGTGATTACCATGCAGAGATTCATGTGATC
ACAAGAGAAAGAGATGAACTTCAGTGTATGCTAGAAAAATTTGAAAAATACATGGAGGAT
ATACAGTCCAACGTTAAATTATTGACGGCAGAAAGAGATAATCTAAGCGTCTTATATAAT
GAAGCTCAGAAAGAATTGTCAGCCCTAAGACAGGAATCTACCCTAACTCCAACTTCCCAT
AATATTGTTAGTCTTATGGAAAAGGAAAAAGAACTTGCATTATCTGATTTAAGAAGAATT
ATGGCAGAAAAGGAATCTTTAAGAGACAAGTTACATAGTGTCCAGGAAATGAGTCTTCAT

GGAAAGTCAGAATTAGAAAAAACTATTGAACATTTGACATGTGTTAATCATCAGCTTGAA
AGTGAAAGATGTGAATTAAAGTCTAAAATGTTAATGATGAAAGAAACAATAGACACTCTA
GAGAACAAATCAAAACTTCAAGCTAAAAGACTTAGCTTTGCAACGGGTGACTCATCTCAT
CACAAAACAGAGATGAACTCACTTAGGATAGTAAATGAGCAGCTACAGCGGTCACTTGAT
GACTGTCAACACCGACTTTCCATAAAAAGAGAGGAACTTGAATCAGCCCACGCACAAATT
AAAATACTGGAACAAAATCTGGATAAACTAAACCTTAAGATGACTTCACAGGATGAGGAG
GCTCATGTAATGAAAAAGACCATTGGTGTTATTGATAAAGAAAAAGACTTTCTTCAGGAG
ACTGTAGATGAGAAGACAGAAAAGATTGCAAATTTACAGGAAACCCTAGCTACTAAAGAA
AAAGCTATTGCTCAGATGACGGTAACAGTCTCAGAGTGTGAATTAAGTATAAACCAACTC
AAGGAAACAGTGAGTAATCGAGAACGTGAGATAAACAGCCTCCTGCGCCAGCTGGATACT
GCTCGCAAGGAACTTGATGAAGTAGGAAAAGCGAGAGAAATACTTTTTAAAGAAAACAGA
AGATTACAAGATGATTTGGCTACAATGTCGAGAGAGAACCAGGAAATCTCATTGGAGTTG
GAAACAGCAGTACAAGAAAAAGAAGATATGAAGAATAGAGTTCATAGTTACATAACTGAG
GTGTCACGATGGGAAAGCTTAATGGCCACCAAGGAAAAAGAAAATCAAGATTTGTTAGAC
AGATTTCAGATGCTTCATCACCGTGCAGAAGACTGGGAGGTGAAAGCCCATCAGGCTGAG
GGAGAAAGCAGCTCGGTCCGACTGGAGCTGCTTTCTATTGACACAGAGAGGAGACACCTT
CGAGAAAGAGTGGAGCTATTAGAAAAAGAAATTCAAGAGCACATAAATGCCCATCATGCT
TATGAATCTCAGATCTCATCAATGGCAAAAGCCATTTCTAGATTAGAAGAAGAGCTGAGA
TGTCAAGAAGAAGAGAAAGCAGTAGTTTTAAATGATGTGTCTTCTCTCAGAGAACTTTGC
ATTAAGCTTGATTCAGGCAAAGATATTATGACCCAACAATTGAACACCAAAAACCTTGAG
CTTGAGAGGGCTGTAGTGGAATTAGAAAATGTAAAATCAGAAACAGACCTGTTGAAAAAG
CAATTGTCAAATGAGAGACAAACTGTTAAAAACCTTGAATCATTGTTGGCTACAAATAGA
GATAAAGAATTTCATTCTCATTTAACTTCCCATGAGAAGGATACAGAAATTCAGCTACTT
AAAGAGAAGTTAACCCTTTCAGAAAGCAAATCAACTAGTCAAACTCGGGAAAATGCCATG
CTCCGAGCTAAAGTGGTACAGTTAGAGACGGATTATGATACTCTGAAAAGACAGATTTCA
AATGAAAGATACGAACGAGAGCGAGCAGTCCAAGAGATGCGTCGACATGGTCTTTCTACA
TCACCCTTTAGTTCTACTCTGAGGTCTCCTGAATGTTCTCCTGAACATTTAAATATG
>chimpanzee ENSPTRT00000029959.4
ATGACTACAGCTGTAGAAAGAAAGTATATTAATATTAGGAAAAGACTGGATCAGCTGGGA
TACCGCCAGACTCTGACAGTGGAGTGTTTACCTTTGGTAGAAAAACTTTTCAGCGACTTA
GTTCATACAACTGAGAGCCTTCGGCAATCAAAATTATCTGCTGTGAAAGCTGAAAAAGAA
AGTGCCAATTTTGATTTTGTTTTGGAACCCTATAAACTTGAAAATGCAAGATTGAGTAGA
GAAAATAATGAATTATACCTAGAGTTAATGAAACTGAGAGAACATTCAGACCAACATGTT
AAAGAGTTGAAAACTTCATTGAAGAAATGTGCACGTGAAACAGCTGATCTGAAATTTCTG
AATAACCAATATGCTCATAAACTCAAACTGTTGGAGAAAGAGAGCAAAGCTAAGAATGAA
AGAATTCAACAACTTCAAGAAAAGAATTTGCATGCTGTAGTACAAACTCCAGGTGGCAAG
AAAAGAAGTATTGCTTTCAGGCGCCAGCGTATGCAAATTGATGAACCGGTTCCTCCCTCT
GAAGTCAGTTCATATCCAGTTCCTCAACCAGATGACCCTTACATTGCAGACCTCCTTCAA
GTGGCTGATAACAGGATTCAAGAACTTCAACAGGAAGTCCACCAGCTACAAGAAAAGTTA
GCAATGATGGAAAGTGGGGTGAGAGACTATAGCAAGCAGATTGAGCTAAGAGAACGAGAG
ATAGAACGACTGTCAGTTGCTTTGGATGGTGGTCGGTCCCCTGATGTCCTTTCTCTGGAG
TCTAGAAATAAAACCAATGAAAAGCTTATTGCTCATTTAAATATTCAGGTTGACTTTCTT
CAGCAAGCTAATAAAGACCTGGAGAAGCGTATACGAGAGCTTATGGAAACCAAGGAAACA
GTGACATCTGAAGTCGTTAATTTAAGTAACAAAAATGAAAAACTCTGCCAAGAATTAACT
GAAATAGATCAGTTAGCACAGCAGTTGGAAAGACATAAAGAAGAAGTGCTTGAGACTGCT
GATAAAGAGCTTGGGGAAGCAAAGAAAGAGATTAAAAGAAAGCTCTCTGAAATGCGGGAT
CTTGAAGAAACAATGGCAAAACTTCAGCTGGAATTGAACTTATGCCAGAAAGAAAAGGAG

AGACTGAGTGATGAACTCCTTGTAAAATCAGACCTAGAAACTGTTGTTCATCAGCTTGAA
CAAGAAAAGCAAAGACTTAGCAAAAAAGTTGAAAGTTTTGCAGTTACAGAACGACAACTT
ACTCTGGAGGTTGAGAGGATGAGACTAGAACATGGAATAAAACGTCGAGACAGGTCACCT
TCTCGTTTAGATACATTTCTGAAAGGTATAGAAGAAGAACGAGATTATTATAAGAAAGAG
CTAGAGAGACTCCAACATATAATACAGCGAAGATCTTGCTCTACAAGTTATAGCGCACGT
GAAAAAAGTTCAATATTTAGAACACCAGAAAAGGGTGATTACAATTCAGAAATTCATCAG
ATCACAAGAGAAAGAGATGAACTTCAGCGTATGCTAGAAAGATTTGAAAAATATATGGAA
GATATACAGTCCAATGTTAAATTATTGACAGCAGAAAGAGATAAACTAAGTGTCTTATAT
AATGAAGCTCAGGAAGAATTATCTGCCCTAAGAAAAGAATCCACCCAAACCACAGCACCC
CATAATATTGTTAGTCTTATGGAAAAGGAAAAAGAACTTGCGTTATCTGACTTAAGAAGA
GTTATGGCAGAAAAGGAAGCTTTAAGAGAAAAATTAGAGCATATTGAAGAAGTGAGTCTT
TTTGGAAAATCAGAATTAGAGAAAACTATTGAACATTTGACATGTGTTAATCATCAGCTT
GAAAGCGAAAAATATGAATTAAAGTCTAAAGTGTTAATAATGAAAGAAACAATAGAGTCG
TTAGAGAACAAATTAAAAGTCCAAGCTCAAAAATTTAGCCATGTGGCTGGTGACTCATCT
CATCAGAAAACAGAGGTGAACTCACTTAGGATAGTAAATGAGCAGCTACAGCGGTCAGTT
GATGACTATCAGCACCGACTTTCCATAAAAAAAGGTGAACTTGAATCAGCCCAAGCACAA
ATTAAAATACTGGAGGAAAAGATAGATGAACTAAACCTTAAGATGACTTCACAGGATGAG
GAGGCTCATGTAATGAAAAAGACCATTGGTGTTATTGATAAAGAAAAAGACTTTCTCCAG
GAGACTGTAGATGAGAAGACAGAAAAGATTGCAAATTTGCAAGAAAACCTAGCTAATAAA
GAAAAAGCTGTTGCTCAAATGAAGCTAATGATCTCAGAGTGTGAATCATCTGTGAACCAG
CTGAAAGAAACATTGGTTAATCGAGATCGTGAGATAAACAGCCTCCGGCGCCAGCTTGAT
GCAGCTCACAAAGAACTCGATGAAGTAGGAAGATCTAGAGAAATCGCTTTTAAGGAAAAC
AGAAGACTGCAAGATGACCTGGCTACAATGGCAAGAGAAAATCAAGAAATCTCATTGGAA
TTGGAAGCATCAGTGCAAGAAAAAGAAGAAATGAAGAGCAGAGTTCATAAATACATAACA
GAGGTGTCACGATGGGAGAGCTTAATGGCTGCCAAGGAAAAAGAAAATCAAGATTTGTTA
GATAGATTTCAGATGCTTCATAACCGTGCTGAAGACTGGGAGGTCAAAGCCCATCAAGCT
GAGGGAGAAAGCAGCTCAGTTCGACTGGAACTTCTTTCTATTGACACTGAGAGGAGACAT
CTTCGAGAAAGAGTGGAGCTATTAGAAAAAGAAATTCAAGAGCACTTAAATGCCCATCAT
GCTTATGAATCTCAGATCTCATCAATGGCAAAAGCCATGTCTCGATTAGAAGAAGAGCTG
AGACATCAAGAAGATGAGAAAGCAACAGTATTAAATGACTTGTCATCTCTTAGAGAACTT
TGCATTAAACTTGATTCAGGCAAAGATATTATGACCCAGCAATTGAATTCGAAAAACCTT
GAGTTTGAGAGGGTTGTGGTGGAATTAGAAAATGTAAAGTCAGAGTCAGACCTACTGAAA
AAACAACTGTCAAATGAGAGACATACAGTTAAAAACCTCGAATCATTGTTGGCTACAAAC
AGAGATAAAGAATTTCATTCTCACTTAACCTCCCACGAGAAGGATACAGAAATCCAGCTA
CTTAAGGAGAAGTTAACCCTTTCTGAAAGCAAATTAACTAGTCAAAGCCGGGAAAACACC
ATGCTTCGAGCTAAAGTGGCACAGTTACAAACAGATTATGATGCTCTGAAAAGGCAGATC
TCAACTGAAAGATACGAACGAGAACGAGCAATCCAAGAGATGCGTCGACATGGTCTTGCT
ACACCACCCCTTAGTTCCACTCTGAGGTCTCCTTCACATTCTCCTGAACATAGAAATGTG

>bonobo XM_003806480.2
ATGACTACAGCTGTAGAAAGAAAGTATATTAATATTAGGAAAAGACTGGATCAGCTGG
GATACCGCCAGACTCTGACAGTGGAGTGTTTACCTTTGGTAGAAAAACTTTTCAGCGACTTAGTTCATAC
AACTGAGAGCCTTCGGCAATCAAAATTATCTGCTGTGAAAGCTGAAAAAGAAAGTGCCAATTTTGATTTT
GTTTTGGAACCCTATAAACTTGAAAATGCAAGATTGAGTAGAGAAAATAATGAATTATACCTAGAGTTAA
TGAAACTGAGAGAACATTCAGACCAACATGTTAAAGAGTTGAAAACTTCATTGAAGAAATGTGCACGTGA
AACAGCTGATCTGAAATTTCTGAATAACCAATATGCTCATAAACTCAAACTGTTGGAGAAAGAGAGCAAA
GCTAAGAATGAAAGAATTCAACAACTTCAAGAAAAGAATTTGCATGCTGTAGTACAAACTCCAGGTGGCA

AGAAAAGAAGTATTGCTTTCAGGCGCCAGCGTATGCAAATTGATGAACCGGTTCCTCCCTCTGAAGTCAG
TTCATATCCAGTTCCTCAACCAGATGACCCTTACATTGCAGACCTCCTTCAAGTGGCTGATAACAGGATT
CAAGAACTTCAACAGGAAGTCCACCAGCTACAAGAAAAGTTAGCAATGATGGAAAGTGGGGTGAGAGACT
ATAGCAAGCAGATTGAGCTAAGAGAACGAGAGATAGAACGACTGTCAGTTGCTTTGGATGGTGGTCGGTC
CCCTGATGTCCTTTCTCTGGAGTCTAGAAATAAAACCAATGAAAAGCTTATTGCTCATTTAAATATTCAG
GTTGACTTTCTTCAGCAAGCTAATAAAGACCTGGAGAAGCGTATACGAGAGCTTATGGAAACCAAGGAAA
CAGTGACATCTGAAGTCGTTAATTTAAGTAACAAAAATGAAAAACTCTGCCAAGAATTAACTGAAATAGA
TCAGTTAGCACAGCAGTTGGAAAGACATAAAGAAGAAGTGCTTGAGACTGCTGATAAAGAGCTTGGGGAA
GCAAAGAAAGAGATTAAAAGAAAGCTCTCTGAAATGCGGGATCTTGAAGAAACAATGGCAAAACTTCAGC
TGGAATTGAACTTATGCCAGAAAGAAAAGGAGAGACTGAGTGATGAACTCCTTGTAAAATCAGACCTAGA
AACTGTTGTTCATCAGCTTGAACAAGAAAAGCAAAGACTTAGCAAAAAAGTTGAAAGTTTTGCAGTTACA
GAACGACAACTTACTCTGGAGGTTGAGAGGATGAGACTAGAACATGGAATAAAACGTCGAGACAGGTCAC
CTTCTCGTTTAGATACATTTCTGAAAGGTATAGAAGAAGAACGAGATTATTATAAGAAAGAGCTAGAGAG
ACTCCAACATATAATACAGCGAAGATCTTGCTCTACAAGTTATAGCGCACGTGAAAAAAGTTCAATATTT
AGAACACCAGAAAAGGGTGATTACAATTCAGAAATTCATCAGATCACAAGAGAAAGAGATGAACTTCAGC
GTATGCTAGAAAGATTTGAAAAATATATGGAAGATATACAGTCCAATGTTAAATTATTGACAGCAGAAAG
AGATAAACTAAGTGTCTTATATAATGAAGCTCAGGAAGAATTATCTGCCCTAAGAAAGGAATCCACCCAA
ACCACAGCACCCCATAATATTGTTAGTCTTATGGAAAAGGAAAAAGAACTTGCGTTGTCTGACTTAAGAA
GAGTTATGGCAGAAAAGGAAGCTTTAAGAGAAAAATTAGAGCATATTGAAGAAGTGAGTCTTTTTGGAAA
ATCAGAATTAGAGAAAACTACTGAACATTTGACATGTGTTAATCATCAGCTTGAAAGCGAAAAATATGAA
TTAAAGTCTAAAGTGTTAATAATGAAAGAAACAATAGAGTCGTTAGAGAACAAATTAAAAGTCCAAGCTC
AAAAATTTAGCCATGTGGCTGGTGACTCATCTCATCAGAAAACAGAGGTGAACTCACTTAGGATAGTAAA
TGAGCAGCTACAGCGGTCAGTTGATGACTATCAGCACCGACTTTCCATAAAAAGAGGTGAACTTGAATCA
GCCCAAGCACAAATTAAAATACTGGAGGAAAAGATAGATGAACTAAACCTTAAGATGACTTCACAGGATG
AGGAGGCTCATGTAATGAAAAAGACCATTGGTGTTATTGATAAAGAAAAAGACTTTCTCCAGGAGACTGT
AGATGAGAAGACAGAAAATATTGCAAATTTGCAAGAAAACCTAGCTAATAAAGAAAAAGCTGTTGCTCAA
ATGAAGCTAATGATCTCAGAGTGTGAATCATCTGTGAACCAGCTGAAAGAAACACTGGTTAATCGAGATC
ATGAGATAAACAGCCTCCGGCGCCAGCTTGATGCAGCTCACAAAGAACTCGATGAAGTAGGAAGATCTAG
AGAAATCGCTTTTAAGGAAAACAGAAGACTGCAAGATGACCTGGCTACAATGGCAAGAGAAAATCAAGAA
ATCTCATTGGAATTGGAAGCATCAGTGCAAGAAAAAGAAGAAATGAAGAGCAGAGTTCATAAATACATAA
CAGAGGTGTCACGATGGGAGAGCTTAATGGCTGCCAAGGAAAAAGAAAATCAAGATTTGTTAGATAGATT
TCAGATGCTTCATAACCGTGCTGAAGACTGGGAGGTCAAAGCCCATCAAGCTGAGGGAGAAAGCAGCTCA
GTTCGACTGGAACTTCTTTCTATTGACACTGAGAGGAGACATCTTCGAGAAAGAGTGGAGCTATTAGAAA
AAGAAATTCAAGAGCACATAAATGCCCATCATGCTTATGAATCTCAGATCTCATCAATGGCAAAAGCCAT
GTCTCGATTAGAAGAAGAGCTGAGACATCAAGAAGATGAGAAAGCAACAGTATTAAATGACTTGTCATCT
CTTAGAGAACTTTGCATTAAACTTGATTCAGGCAAAGATATTATGACCCAGCAATTGAATTCGAAAAACC
TTGAGTTTGAGAGGGTTGTGGTGGAATTAGAAAATGTAAAGTCAGAGTCAGACCTACTGAAAAAACAACT
GTCAAATGAGAGACATACAGTTAAAAACCTCGAATCATTGTTGGCTACAAACAGAGATAAAGAATTTCAT
TCTCACTTAACCTCCCACGAGAAGGATACAGAAATCCAGCTACTTAAGGAGAAGTTAACCCTTTCTGAAA
GCAAATTAACTAGTCAAAGCCGGGAAAACACCATGCTTCGAGCTAAAGTGGCACAGTTACAAACAGATTA
TGATGCTCTGAAAAGGCAGATCTCAACTGAAAGATACGAACGAGAACGAGCAATCCAAGAGATGCGTCGA
CATGGTCTTGCTACACCACCCCTTAGTTCCACTCTGAGGTCTCCTTCACATTCTCCTGAACATAGAAATG
TG
>orangutan ENSPPYT00000017154.2
ATGACTACAGCTGTAGAAAGAAAGTATGTTAATATTAGGAAAAGACTGGATCAGCTGGGA
TACCGCCAGACTCTGACAGTGGAGTGTTTACCTTTGGTAGAAAAACTTTTCAGCGATTTA
GTTCATACAACTGAGAGCCTTCGGCAATCAAAATTATCTGCTGTGAAAGCTGAAAAAGAA

AGTGCCAATTTTGATTTTGTTTTGGAACCCTATAAACTTGAAAATGCAAGATTGAGTAGA
GAAAATAATGAATTATACCTAGAGTTAATGAAACTGAGAGAACATTCAGACCAACACGTT
AAAGAGTTGAAAACTTCATTGAAGAAGTGTGCACGTGAAACAGCTGATCTGAAATTTCTA
AATAACCAATATGTTCATAAACTCAAACTGTTGGAGAAAGAGAGCAAAGCTAAGAATGAA
AGAATTCAACAACTTCAGGAAAAGAATTTGCATGCTGTAGTACAGACTCCAGGTGGCAAG
AAAAGAAGTATTGCTTTCAGGCGCCAGCGTATGCAAATTGATGAACCGGTTCCTCCCTCT
GAAGTCAGTTCATATCCAGTTCCTCAACCAGATGACCCTTACATTGCAGACCTCCTACAA
GTGGCTGATAACAGGATTCAAGAACTTCAACAGGAAGTCCACCAGCTACAAGAAAAGTTA
GCAATGATGGAAAGTGGGGTGAGAGACTATAGCAAGCAGATTGAGCTAAGAGAACGAGAG
ATAGAACGACTGTCAGTTGCTTTGGATGGTGGTCGGTCCCCTGATGTCCTTTCTCTGGAG
TCTAGAAATAAAACCAATGAAAAGCTTATTGCTCATTTGAATATTCAGGTTGACTTTCTT
CAGCAAGCTAATAAAGACCTGGAGAAGCGTATACGAGAGCTTATGGAAACCAAGGAAACA
GTGACATCTGAAGTTGTTAATTTAAGTAACAAAAATGAAAAACTCTGCCAAGAATTAACT
GAAATAGATCAGTTAGCACAGCAGTTGGAAAGACATAAAGAAGAAGTGCTTGAGACTGCT
GATAAAGAGCTTGGGGAAGCAAAGAAAGAGATTAAAAGAAAGCTGTCTGAAATGCGGGAT
CTTGAAGAAACAATGGCAAAACTTCAACTGGAATTGAACTTATGCCAGAAAGAAAAGGAG
AGACTGAGTGATGAACTCCTTGTAAAATCAGACCTAGAAACTGTTGTTCATCAGCTTGAA
CAAGAAAAGCAAAGACTTAGCAAAAAAGTTGAAAGTTTTGCAGTTACAGAACGACAACTT
ACTCTGGAAGTTGAGAGGATGAGACTAGAACATGGAATAAAACGTCGAGACAAGTCACCT
TCTCGTTTAGATACATTTCTGAAAGGTATAGAAGAAGAACGAGATTATTATAAGAAAGAG
CTAGAGAGACTCCAACATATAATACAGCGAAGATCTTGCTCTACAAGTTATAGTGCACGT
GAAAAAAGTTCAATATTTAGAACACCAGAAAAGGGTGATTACAATTCAGAAATTCATCAG
ATCACAAGAGAAAGAGATGAACTTCAGCGTATGCTAGAAAGATTTGAAAAATATATGGAG
GATATACAGTCCAATGTTAAATTATTGACAGCAGAAAGAGATAAACTAAGTGTCTTATAT
AATGAAGCTCAGGAAGAATTATCTGCCCTAAGAAAGGAATCCACCCAAACCACAGCACCC
CATAATATTGTTAGTCTTATGGAAAAGGAAAAAGAACTTGCGTTATCTGACTTAAGAAGA
ATTATGGCAGAAAAGGAAGCTTTAAGAGAAAAAATTAGAGCATATTGAAGAAATGAGTCTT
TTTGGAAAATCAGAATTAGAGAAAACTATTGAACATTTGACATGTGTTAATCATCAGCTT
GAAAGCGAAAAATATGAATTAAAGTCTAAAGTGTTAATAATGAAAGAAACAATAGAGTCA
TTAGAGAACAAATTAAAAGTCCAAGCTCAAAAATTTAGCCATGTGGCTGGTGACTCATCT
CATCAGAAAACAGAGGTGAACTCACTTAGGATAGTAAATGAGCAGCTACAGCGGTCAGTT
GATGACTATCAGCACCGACTTTCCATAAAAAGAGGTGAACTTGAATCAGCCCAAACACAA
ATTAAAATACTGGAGGAAAAGATAGATGAACTAAACCTTAAGATGACTTCACAGGATGAG
GAGGCTCATGTAATGAAAAAGACTATTGGTGTTATTGATAAAGAAAAAGACTTTCTCCAG
GAGACTGTAGATGAGAAGACAGAAAAGATTGCAAATTTGCAAGAAAACCTAGCAAATAAA
GAAAAAGCTGTTGCTCAAATGAAGCTAATGATCTCAGAGTGTGAATCATCTGTGAACCAG
CTGAAAGAAACATTGGTTAATCGAGATCGTGAGATAAACAGCCTCCGGCGCCAGCTTGAT
GCAGCTCACAAAGAACTCGATGAAGTAGGAAGATCTAGAGAAATCGCTTTTAAGGAAAAC
AGAAGACTGCAAGATGACCTGGCTACAATGGCAAGAGAAAACCAAGAAATCTCATTGGAA
TTGGAAGCAGCAGTGCAAGAAAAAGAGGAAATGAAGAGCAGAGTTCATAAATACATAACA
GAGGTGTCACGATGGGAGAGCTTAATGGCTGCCAAGGAAAAAGAAAATCAAGATTTGTTA
GATAGATTTCAGATGCTTCATAACCGTGCTGAAGACTGGGAGGTCAAAGCCCATCAAGCT
GAGGGAGAAAGCAGCTCAGTTCGACTGGAACTTCTTTCTATTGACACTGAGAGGAGACAT
CTTCGAGAAAGAGTGGAGCTATTAGAAAAAGAAATTCAAGAGCACATAAATGCCCATCAT
GCTTATGAATCTCAGATCTCATCAATGGCAAAAGCCATGTCTCGATTAGAAGAAGAGCTG
AGACATCAAGAAGATGAGAAAGCAACAGTATTAAATGACTTGTCATCTCTTAGAGAACTT
TGCATTAAACTTGATTCAGGCAAAGATATCATGACCCAGCAATTGAATTCGAAAAACCTT

GAGTTTGAGAGGGTTGTGGTAGAATTAGAAAATGTAAAGTCAGAGTCAGACCTACTGAAA
AAACAACTGTCAAATGAGAGACATACAGTTAAAAACCTCGAATCATTGTTGGCTACAAAC
AGAGATAAAGAATTTCATTCTCACTTAACCTCCCATGAGAAGGATACAGAAATCCAGCTA
CTTAAGGAGAAGTTAACCCTTTCCGAAAGCAAATTAACTAGTCAAAGCCGGGAAAACACC
ATGCTTCGAGCTAAAGTGGCACAGTTACAAACAGATTATGATGCTCTGAAAAGGCAGATC
TCAACTGAAAGATACGAACGAGAACGAGCAATCCAAGAGATGCGTCGACATGGTCTTCCT
ACACCACCCCTTAGTTCCACTCTGAGGTCTCCTTCACATTCTCCTGAACATATAAATGTA

>Macaca fascicularis XM_005555245.2
ATGACTACAGCTGTAGAAAGAAAGTATATTAATATTAGGAAAA
GACTGGATCAGCTGGGATACCGCCAGACTCTGACAGTGGAGTGTTTACCTTTGGTAGAAAAACTTTTCAG
CGACTTAGTTCATACAACAGAGAGCCTTCGGCAATCAAAATTATCTACTGTGAAAGCTGAAAAAGAAAGT
GCCAATTTTGATTTTGTTTTGGAACCCTATAAACTTGAAAATGCAAGATTGAGTAGAGAAAATAATGAAT
TATACCTAGAGTTAATGAAACTGAGAGAACACTCGGACCAACACGTTAAAGAGTTGAAAACTTCATTGAA
GAAGTGTGCACGTGAAACAGATGATCTGAAATTTCTAAATAACCAATATGTTCATAAACTCAAACTGTTG
GAGAAAGAGAGCAAAGCTAAGAATGAAAGAATTCAACAACTTCAAGAAAAGAATTTGCATGCTGTAGTAC
AAACTCCAGGTGGCAAGAAAAGAAGTATTGCTTTCAGGCGCCAGCGTATGCAAATTGATGAACCGGTTCC
TCCCTCTGAAGTCAGTTCATATCCGGTTCCTCAACCAGATGACCCTTACATTGCAGACCTCCTACAAGTG
GCTGATAACAGGATTCAAGAACTTCAACAGGAAGTCCACCAGCTACAAGAAAAGTTAGCAGTGATGGAAA
GTGGGGTGAGCGATTATAGCAAGCAGATTGAGCTAAGAGAACGAGAGATAGAACGACTGTCAGTTGCTTT
GGATGGTGGTCGGTCCCCTGATGTCCTCTCTCTGGAGTCTAGAAATAAAACCAATGAAAAGCTTATTGCT
CATTTAAATATTCAGGTTGACTTTCTTCAGCAAGCTAATAAAGACCTGGAGAAGCGTATACGAGAGCTTA
TGGAAACCAAGGAAACAGTGACATCTGAAGTTGTTAATTTAAGTAACAAAAATGAAAAACTCTGCCAAGA
ATTAACTGAAATAGATCAGTTAGCACAGCAGTTGGAAAGACATAAAGAAGAAGTGCTTGAGACTGCTGAT
AAAGAACTTGGGGAAGCAAAGAAAGAGATTAAAAGAAAGCTGTCTGAAATGCAGGATCTTGAAGAAACAA
TGGCAAAACTTCAACTGGAATTGAACTTATGCCATAAAGAAAAGGAGAGACTGAGTGATGAACTCCTTGT
AAAATCAGACCTTGAAACTGTTGTTCATCAGCTTGAACAAGAAAAGCAAAGACTTAGCAAAAAAGTTGAA
AGTTTTGTAGTTACAGAAAGAGAACTTACTCTGGAAGTTGAGAGGATGAGACTAGAACATGGAATAAAAC
GTCGAGACAGGTCACCTTCTCGTTTAGATACATTTCTGAAAGGTATAGAAGAAGAACGAGATTATTATAA
GAAAGAGCTAGAGAGACTCCAACATATAATACAGCGAAGATCTTGCTCTACAAATTATGGTGCACGTGAA
AAAAATTCAATATTTAGGACACCAGAAAAGGGTGATTACAATTCAGAAATTCATCAGATCACAAGAGAAA
GAGATGAACTTCAGCGTATGCTGGAAAGATTTGAAAAATATATGGAGGATATACAGTCAAATGTTAAATT
ACTGACAGCAGAAAGAGATAAACTAAGTGTCTTATATAATGAAGCTCAAGAAGAATTATCTGCCCTAAGA
AAGGAATCCACCCAAACCACAGCACCCCATAATATTGTTAGTCTTATGGAAAAGGAAAAAGAACTTGCGT
TATCTGACTTAAGAAGAATTATGGCAGAAAAGGAAGCTTTAAGAGAAAAATTAGAGCATATTGAAGAAAT
GAGTCTTTTTGGAAAATCAGAATTAGAGAAAACTATTGAACATTTGACATGTGTTAATCATCAGCTTGAA
AGCGAAAAATATGAATTAAAGTCTAAAGTGTTAATAATGAAAGAAACAATAGAGTCGTTAGAGAACAAAT
TAAAAGTCCAAGCTCAAAAATTTAGCCATGTGGCTGGTGACTCATCTCATCAGAAAACAGAGGTGAACTC
ACTTAGGATAGTAATTGAGCAGCTACAGCGGTCAGTTGATGACTATCAGCACCGACTTTCCATTAAAAGA
GGTGAACTTGAATCAGCCCAAGCACAAATTAAAATACTGGAGGAAAAAATAGATGAATTAAACCTTAAGA
TGACTTCACAAGATGAGGAGGCTCATGTAATGAAAAAGACCATTGGTGTTATTGATAAAGAAAAAGACTT
TCTCCAGGAGACTGTAGATGAGAAGACAGAAAAGATTGCAGATTTGCAAGAAAACCTAGCTAATAAAGAG
AAAACTGTTGCTCAAATGAAGCTAATGATCTCAGAGTGTGAATCAGCTGTGAACCAGCTAAAAGAAACAT
TAGTTAATCGAGATCGTGAGATAAACAGCCTCCGGCGCCAGCTTGATGCAGCTCACAAAGAACTCGATGA
AGTAGGAAGATCTAGAGAAATTGCTTTTAAGGAAAACAGAAGATTGCAAGATGACCTGGCTACAATAGCA
AGAGAAAACCAAGAAATCTCATTGGAATTGGAAGCAGCAGTGCAAGAAAAAGAAGAAATGAAGAGCAGAG
TTCATAAATACATAACAGAGGTGTCACGATGGGAGAGCTTAATGGCTGCCAAGGAAAAAGAAAATCAAGA

TTTGTTAGATAGATTTCAGATGCTTCATAACCGTGCTGAAGACTGGGAGGTCAAAGCCCATCAAGCGGAG
GGAGAAAGCAGCTCAGTTCGACTGGAACTTCTTTCTATTGACACTGAGAGGAGACATCTTCGAGAAAGAG
TGGAGCTATTAGAAAAAGAAATTCAAGAGCACATAAATGCCCATCATGCTTATGAATCTCAGATCTCATC
AATGGCAAAAGCCATGTCTCGATTAGAAGAAGAGCTGAGACATCAAGAAGATGAGAAAGCAACAGTATTA
AATGACTTATCATCTCTTAGAGAACTTTGCATTAAACTTGATTCAGGCAAAGATATTATGACGCAGCAAC
TGAATTCCAAAAACCTTGAGTTTGAGAGGGTTGTAGTGGAATTAGAAAATGTAAAGTCAGAGTCAGACCT
ATTGAAAAAACAACTGTCAAATGAGAGACATACAGTTAAAAACCTCGAATCATTGTTGGCTACAAACAGA
GATAAAGAATTTCATTCTCACTTAACCTCCCATGAGAAGGATACAGAAATCCAGCTACTTAAGGAGAAGT
TAACCCTTTCTGAAAGCAAATTAACTAGTCAAAGCCGGGAAAACACCATGCTTCGAGCTAAAGTGGCACA
GTTACAAACAGATTATGATGCCCTGAAAAGGCAGATCTCAACTGAAAGATATGAACGAGAACGAGCAATC
CAAGAGATGCGTCGACATGGTCTTCCTACACCACCCCTTAGTTCCACTCTGAGGTCTCCTTCACATTCTC
CTGAACATATAAATGCG
>Mandrillus leucophaeus XM_011992507.1
ATGACTACAGCTGTAGAAAGA
AAGTATATTAATATTAGGAAAAGACTGGATCAGCTGGGATACCGCCAGACTCTGACAGTGGAGTGTTTAC
CTTTGGTAGAAAAACTTTTCAGCGACTTAGTTCATACAACAGAGAGCCTTCGGCAATCAAAATTATCTGC
TGTGAAAGCTGAAAAAGAAAGTGCCAATTTTGATTTTGTTTTGGAACCCTATAAACTTGAAAATGCAAGA
TTGAGTAGAGAAAATAATGAATTATACCTAGAGTTAATGAAACTGAGAGAACATTCGGACCAACACGTTA
AAGAGTTGAAAACTTCATTGAAGAAGTGTGCACGTGAAACAGATGATCTGAAATTTCTAAATAACCAATA
TGTTCATAAACTCAAACTGTTGGAGAAAGAGAGCAAAGCTAAGAATGAAAGAATTCAACAACTTCAAGAA
AAGAATTTGCATGCTGTAGTACAAACTCCAGGTGGCAAGAAAAGAAGTATTGCTTTCAGGCGCCAGCGTA
TGCAAATTGATGAACCGGTTCCTCCCTCTGAAGTCAGTTCATATCCGGTTCCTCAACCAGATGACCCTTA
CATTGCAGACCTCCTACAAGTGGCTGATAACAGGATTCAAGAACTTCAACAGGAAGTCCACCAGCTACAA
GAAAAGTTAGCAGTGATGGAAAGTGGGGTGAGCGATTATAGCAAGCAGATTGAGCTAAGAGAACGAGAGA
TAGAACGACTGTCAGTTGCTTTGGATGGTGGTAGGTCCCCTGATGTCCTCTCTCTGGAGTCTAGAAATAA
AACCAATGAAAAGCTTATTGCTCATTTAAATATTCAGGTTGACTTTCTTCAGCAAGCTAATAAAGACCTG
GAGAAGCGTATACGAGAGCTTATGGAAACCAAGGAAACGGTGACATCTGAGGTTGTTAATTTAAGTAACA
AAAATGAAAAACTCTGCCAAGAATTAACTGAAATAGATCAGTTAGCACAGCGGTTGGAAAGACATAAAGA
AGAAGTGCTTGAGACTGCTGATAAAGAACTTGGGGAAGCAAAGAAAGAGATTAAAAGAAAGCTGTCTGAA
ATGCAGGATCTTGAAGAAACAATGGCAAAACTTCAACTGGAATTGAACTTATGCCATAAAGAAAAGGAGA
GACTGAGTGATGAACTCCTTGTAAAATCAGACCTTGAAACTGTTGTTCATCAGCTTGAACAAGAAAAGCA
AAGACTTAGCAAAAAAGTTGAAAGTTTTGTAGTTACAGAAAGAGAACTTACTCTGGAAGTTGAGAGGATG
AGACTAGAACATGGAATAAAACGTCGAGACAGGTCACCTTCTCGTTTAGATACATTTCTGAAAGGTATAG
AAGAAGAACGAGATTATTATAAGAAAGAGCTAGAGAGACTCCAACATATAATACAGCGAAGATCTTGCTC
TACAAATTATGGTGCACGTGAAAAAAATTCAATATTTAGAACACCAGAAAAGGGTGATTACAATTCAGAA
ATTCATCAGATCACAAGAGAAAGAGATGAACTTCAGCGTATGCTGGAAAGATTTGAAAAATATATGGAGG
ATATACAGTCAAATGTTAAATTACTGACAGCAGAAAGAGATAAACTAAGTGTCTTATATAATGAAGCTCA
AGAAGAATTATCTGCCCTAAGAAAGGAATCCACCCAAACCACAGCACCCCATAATATTGTTAGTCTTATG
GAAAAGGAAAAAGAACTTGCGTTATCTGACTTAAGAAGAATTATGGCAGAAAAGGAAGCTTTAAGAGAAA
AATTAGAGCATATTGAAGAAATGAGTCTTTTTGGAAAATCAGAATTAGAGAAAACTATTGAACATTTGAC
ATGTGTTAATCATCAGCTTGAAAGCGAAAAATATGAATTAAAGTCTAAAGTGTTAATAATGAAAGAAACA
ATAGAGTCGTTAGAGAACAAGTTAAAAGTCCAAGCTCAAAAATTTAGCCATGTGGCTGGTGACTCATCTC
ATCAGAAAACAGAGGTGAACTCACTTAGGATAGTAATTGAGCAGCTACAGCGGTCAGTTGATGACTATCA
GCACCGACTTTCCATTAAAAGAGGTGAACTTGAATCAGCCCAAGCACAAATTAAAATACTGGAGGAAAAA
ATAGATGAATTAAACCTTAAGATGACTTCACAGGATGAGGAGGCTCATGTAATGAAAAAGACCATTGGTG
TTATTGATAAAGAAAAAGACTTTCTCCAGGAGACTGTAGATGAGAAGACAGAAAAGATTGCAAATTTGCA
AGAAAACCTAGCTAATAAAGAGAAAACTGTTGCTCAAATGAAGCTAATGATCTCAGAGTGTGAATCAGCT

GTGAACCAGCTAAAAGAAACATTAGTTAATCGAGATCGTGAGATAAACAGCCTCCGGCGCCAGCTTGATG
CAGCTCACAAAGAACTCGATGAAGTAGGAAGATCTAGAGAAATTGCTTTTAAGGAAAACAGAAGATTGCA
AGATGACCTGGCTACAATAGCAAGAGAAAACCAAGAAATCTCATTGGAATTGGAAGCAGCAGTGCAAGAA
AAAGAAGAAATGAAGAGCAGAGTTCATAAATACATAACAGAGGTGTCACGATGGGAGAGCTTAATGGCTG
CCAAGGAAAAAGAAAATCAAGATTTGTTAGATAGATTTCAGATGCTTCATAACCGTGCTGAAGACTGGGA
GGTCAAAGCCCATCAAGCGGAGGGAGAAAGCAGCTCAGTTCGACTGGAACTTCTTTCTATTGACACTGAG
AGGAGACATCTTCGAGAAAGAGTGGAGCTATTAGAAAAAGAAATTCAAGAGCACATAAATGCCCATCATG
CTTATGAATCTCAGATCTCATCCATGGCAAAAGCCATGTCTCGATTAGAAGAAGAGCTGAGACATCAAGA
AGATGAGAAAGCAACAGTATTAAATGACTTGTCATCTCTTAGAGAACTTTGCATTAAACTTGATTCAGGC
AAAGATATTATGACCCAGCAACTGAATTCCAAAAGCCTTGAGTTTGAGAGGGTTGTAGTGGAATTAGAAA
ATGTAAAGTCAGAGTCAGACCTATTGAAAAAACAACTGTCAAATGAGAGACATACAGTTAAAAACCTCGA
ATCATTGCTGGCTACAAACAGAGATAAAGAATTTCATTCTCACTTAACCTCCCATGAGAAGGATACAGAA
ATCCAGCTACTTAAGGAGAAGTTAACTCTTTCTGAAAGCAAATTAACTAGTCAAAGCCGGGAAAACACCA
TGCTTCGAGCTAAAGTGGCACAGTTACAAACAGATTATGATGCCCTGAAAAGGCAGATCTCAACTGAAAG
ATATGAACGAGAACGAGCAATCCAAGAGATGCGTCGACATGGTCTTCCTACACCACCCCTTAGTTCCACT
CTGAGGTCTCCTTCACATTCTCCTGAACATATAAATGCG
>Chlorocebus sabaeus XM_007998650.1
ATGACTACAGCTGTAGAA
AGAAAGTATATTAATATTAGGAAAAGACTGGATCAGCTGGGATACCGCCAGACTCTGACAGTGGAGTGTT
TACCTTTGGTAGAAAAACTTTTCAGCGACTTAGTTCATACAACAGAGAGCCTTCGGCGATCAAAATTATC
TACTGTGAAAGCTGAAAAAGAAAGTGCCAATTTTGATTTTGTTTTGGAACCCTATAAACTTGAAAATGCA
AGATTGAGTAGAGAAAATAATGAATTATACCTAGAGTTAATGAAACTGAGAGAACATTCGGACCAACACG
TTAAAGAGTTGAAAACTTCATTGAAGAAGTGTGCACGTGAAACAGATGATCTGAAATTTCTAAATAACCA
ATATGTTCATAAACTCAAACTGTTGGAGAAAGAGAGCAAAGCTAAGAATGAAAGAATTCAACAACTTCAA
GAAAAGAATTTGCATGCTGTAGTACAAACTCCAGGTGGCAAGAAAAGAAGTATTGCTTTCAGGAGCCAGC
GTATGCAAATTGATGAACCGGTTCCTCCCTCTGAAGTCAGTTCATATCCGGTTCCTCAACCAGATGACCC
TTACATTGCAGACCTCCTACAAGTGGCTGATAACAGGATTCAAGAACTTCAACAGGAAGTCCACCAGCTA
CAAGAAAAGTTAGCAGTGATGGAAAGTGGGGTGAGCGATTATAGCAAGCAGATTGAGCTAAGAGAACGAG
AGATAGAACGACTGTCAGTTGCTTTGGATGGTGGTCGGTCCCCTGATGTCCTCTCTCTGGAGTCTAGAAA
TAAAACCAATGAAAAGCTTATTGCTCATTTAAATATTCAGGTTGACTTTCTTCAGCAAGCTAATAAAGAC
CTGGAGAAGCGTATACGAGAGCTTATGGAAACCAAGGAAACGGTGACATCTGAAGTTGTTAATTTAAGTA
ACAAAAATGAAAAACTCTGCCAAGAATTAACTGAAATAGATCAGTTAGCACAGCAGTTGGAAAGACATAA
AGAAGAAGTGCTTGAGACTGCTGATAAAGAACTTGGGGAAGCAAAGAAAGAGATTAAAAGAAAGCTGTCT
GAAATGCAGGATCTTGAAGAAACAATGGCAAAACTTCAACTGGAATTGAACTTATGCCATAAAGAAAAGG
AGAGACTGAGTGATGAACTCCTTGTAAAATCAGACCTTGAAACTGTTGTTCATCAGCTTGAACAAGAAAA
GCAAAGACTTAGCAAAAAAGTTGAAAGTTTTGTAGTTACAGAAAGAGAACTTACTCTGGAAGTTGAGAGG
ATGAGACTAGAACATGGAATAAAACGTCGAGACAGGTCACCTTCTCGTTTAGATACATTTCTGAAAGGTA
TAGAAGAAGAACGAGATTATTATAAGAAAGAGCTAGAGAGACTCCAACATATAATACAGCGAAGATCTTG
CTCTACAAATTATGGCGCACGTGAAAAAAATTCAGTATTTAGAACACCAGAAAAGGGTGATTACAATTCA
GAAATTCATCAGATCACAAGAGAAAGAGATGAACTTCAGCGTATGCTGGAAAGATTTGAAAAATATATGG
AGGATATACAGTCAAATGTTAAATTACTGACAGCAGAAAGAGATAAACTAAGTGTCTTATATAATGAAGC
TCAAGAGGAATTATCTGCCCTAAGAAAGGAATCCACCCAAACCACAGCACCCCATAATATTGTTAGTCTT
ATGGAAAAGGAAAAAGAACTTGCTTTATCTGACTTAAGAAGAATTATGGCGGAAAAGGAAGCTTTAAGAG
AAAAATTAGAGCATATTGAAGAAATGAGTCTTTTTGGAAAATCAGAATTAGAGAAAACTATTGAACAATT
GACATGTGTTAATCATCAGCTTGAAAGCGAAAAATATGAATTAAAGTCTAAAGTGTTAATAATGAAAGAA
ACAATAGAGTCGTTAGAGAACAAATTAAAAGTCCAAGCTCAAAAATTTAGCCATGTGGCTGGTGACTCAT
CTCATCAGAAAACAGAGGTGAACTCACTTAGGATAGTAATTGAGCAGCTACAGCGGTCAGTTGATGACTA

TCAGCACCGACTTTCCATTAAAAGAGGTGAACTTGAATCAGCCCAAGCACAAATTAAAATACTGGAGGAA
AAAATAGATGAATTAAACCTTAAGATGACTTCACAGGATGAGGAGGCTCATGTAATGAAAAAGACCATTG
GTGTTATTGATAAAGAAAAAGACTTTCTCCAGGAGACTGTAGATGAGAAGACAGAAAAGATTGCAAATTT
GCAAGAAAACCTAGCTAATAAAGAGAAAACTGTTGCTCAAATGAAGCTAATGATCTCAGAGTGTGAATCA
GCTGTGAACCAGCTAAAAGAAACATTAGTTAATCGAGATCGTGAGATAAACAGCCTCCGGCGCCAGCTTG
ATGCAGCTCACAAAGAACTCGATGAAGTAGGAAGATCTAGAGAAATTGCTTTTAAGGAAAACAGAAGATT
GCAAGATGACCTGGCTACAATAGCAAGAGAAAACCAAGAAATCTCATTGGAATTGGAAGCAGCAGTGCAA
GAAAAAGAAGAAATGAAGAGCAGAGTTCATAAATACATAACAGAGGTGTCACGATGGGAGAGCTTAATGG
CTGCCAAGGAAAAAGAAAATCAAGATTTGTTAGATAGATTTCAGATGCTTCATAACCGTGCTGAAGACTG
GGAGGTCAAAGCCCATCAAGCGGAGGGAGAAAGCAGCTCAGTTCGACTGGAACTTCTTTCTATTGACACT
GAGAGGAGACATCTTCGAGAAAGAGTGGAGCTATTAGAAAAAGAAATTCAAGAGCACATAAATGCCCATC
ATGCTTATGAATCTCAGATCTCATCAATGGCAAAAGCCATGTCTCGATTAGAAGAAGAGCTGAGACATCA
AGAAGATGAGAAAGCAACAGTATTAAATGACTTGTCATCTCTTAGAGAACTTTGCATTAAACTTGATTCA
GGCAAAGATATTATGACCCAGCAACTGAATTCCAAAAACCTTGAGTTTGAGAGGGTTGTAGTGGAATTAG
AAAATGTAAAGTCAGAGTCAGACCTACTGAAAAAACAACTGTCAAATGAGAGACATACAGTTAAAAACCT
CGAATCATTGTTGGCTACAAACAGAGATAAAGAATTTCATTCTCACTTAACCTCCCATGAGAAGGATACA
GAAATCCAGCTACTTAAGGAGAAGTTAACCCTTTCTGAAAGCAAATTAACTAGTCAAAGCCGGGAAAACA
CCATGCTTCGAGCTAAAGTGGCACAGTTACAAACAGATTATGATGCCCTGAAAAGGCAGATCTCAACTGA
AAGATATGAACGAGAACGAGCAATCCAAGAGATGCGTCGACATGGTCTTCCTACACCACCCCTTAGTTCT
ACTCTGAGGTCTCCTTCACATTCTCCTGAACATATACATACG
>Cercocebus atys XM_012073496.1
ATGACTACAGCTGTAGAAAGAAAGTATATTA
ATATTAGGAAAAGACTGGATCAGCTGGGATACCGCCAGACTCTGACAGTGGAGTGTTTACCTTTGGTAGA
AAAACTTTTCAGCGACTTAGTTCATACAACAGAGAGCCTTCGGCAATCAAAATTATCTACTGTGAAAGCT
GAAAAAGAAAGTGCCAATTTTGATTTTGTTTTGGAACCCTATAAACTTGAAAATGCAAGATTGAGTAGAG
AAAATAATGAATTATACCTAGAGTTAATGAAACTGAGAGAACATTCGGACCAACACGTTAAAGAGTTGAA
AACTTCATTGAAGAAGTGTACACGTGAAACAGATGATCTGAAATTTCTAAATAACCAATATGTTCATAAA
CTCAAACTGTTGGAGAAAGAGAGCAAAGCTAAGAATGAAAAAATTCAACAACTTCAAGAAAAGAATTTGC
ATGCTGTAGTACAAACTCCAGGTGGCAAGAAAAGAAGTATTGCTTTCAGGCGCCAGCGTATGCAAATTGA
TGAACCGGTTCCTCCCTCTGAAGTCAGTTCATATCCGGTTCCTCAACCAGATGACCCTTACATTGCAGAC
CTCCTACAAGTGGCTGATAACAGGATTCAAGAACTTCAACAGGAAGTCCACCAGCTACAAGAAAAGTTAG
CAGTGATGGAAAGTGGGGTGAGCGATTATAGCAAGCAGATTGAGCTAAGAGAACGAGAGATAGAACGACT
GTCAGTTGCTTTGGATGGTGGTAGGTCCCCTGATGTCCTCTCTCTGGAGTCTAGAAATAAAACCAATGAA
AAACTTATTGCTCATTTAAATATTCAGGTTGACTTTCTTCAGCAAGCTAATAAAGACCTGGAGAAGCGTA
TACGAGAGCTTATGGAAACCAAGGAAACGGTGACATCTGAAGTTGTTAATTTAAGTAACAAAAATGAAAA
ACTCTGCCAAGAATTAACTGAAATAGATCAGTTAGCACAGCAGTTGGAAAGACATAAAGAAGAAGTGCTT
GAGACTGCTGATAAAGAACTTGGGGAAGCAAAGAAAGAGATTAAAAGAAAGCTGTCTGAAATGCAGGATC
TTGAAGAAACAATGGCAAAACTTCAACTGGAATTGAACTTATGCCATAAAGAAAAGGAGAGACTGAGTGA
TGAACTCCTTGTAAAATCAGACCTTGAAACTGTTGTTCATCAGCTTGAACAAGAAAAGCAAAGACTTAGC
AAAAAAGTTGAAAGTTTTGTAGTTACAGAAAGAGAACTTATTCTGGAAGTTGAGAGGATGAGACTAGAAC
ATGGAATAAAACGTCGAGACAGGTCACCTTCTCGTTTAGATACATTTCTGAAAGGTATAGAAGAAGAACG
AGATTATTATAAGAAAGAGCTAGAGAGACTCCAACATATAATACAGCGAAGATCATGCTCTACAAATTAT
GGTGCACGTGAAAAAAATTCAATATTTAGAACACCAGAAAAGGGTGATTACAACTCAGAAATTCATCAGA
TCACAAGAGAAAGAGATGAACTTCAGCGTATGCTGGAAAGATTTGAAAAATATATGGAGGATATACAGTC
AAATGTTAAATTACTGACAGCAGAAAGAGATAAACTAAGTGTCTTATATAATGAAGCTCAGGAAGAATTA
TCTGCCCTAAGAAAGGAATCCACCCAAACCACAGCACCCCATAATATTGTTAGTCTTATGGAAAAGGAAA
AAGAACTTGCGTTATCTGACTTAAGAAGAATTATGGCAGAAAAGGAAGCTTTAAGAGAAAAGTTAGAGCA

TATTGAAGAAATGAGTCTTTTTGGAAAATCAGAATTAGAGAAAACTATTGAACATTTGACATGTGTTAAT
CATCAGCTTGAAAGCGAAAAATATGAATTAAAGTCTAAAGTGTTAATAATGAAAGAAACAATAGAGTCGT
TAGAGAACAAATTAAAAGTCCAAGCTCAAAAATTTAGCCATGTGGCTGGTGACTCATCTCATCAGAAAAC
AGAGGTGAACTCACTTAGGATAGTAATTGAGCAGCTACAGCGGTCAGTTGATGACTATCAGCACCGACTT
TCCATTAAAAGAGGTGAACTTGAATCAGCCCAAGCACAAATTAAAATACTGGAGGAAAAAATAGATGAAT
TAAACCTTAAGATGACTTCACAGGATGAGGAGGCTCATGTAATGAAAAAGACCATTGGTGTTATTGATAA
AGAAAAAGACTTTCTCCAGGAGACTGTAGATGAGAAGACAGAAAAGATTGCAAATTTGCAAGAAAACCTA
GCTAATAAAGAGAAAACTGTTGCTCAAATGAAGCTAATGATCTCAGAGTGTGAATCAGCTGTGAACCAGC
TAAAAGAAACATTAGTTAATCGAGATCGTGAGATAAACAGCCTCCGGCGCCAGCTTGATGCAGCTCACAA
AGAACTCGATGAAGTAGGAAGATCTAGAGAAATTGCTTTTAAGGAAAACAGAAGATTGCAAGATGATCTG
GCTACAATAGCAAGAGAAAACCAAGAAATCTCATTGGAATTGGAAGCAGCAGTGCAAGAAAAAGAAGAAA
TGAAGAGCAGAGTTCATAAATACATAACAGAGGTGTCACGATGGGAGAGCTTAATGGCTGCCAAGGAAAA
AGAAAATCAAGATTTGTTAGATAGATTTCAGATGCTTCATAACCGTGCTGAAGACTGGGAGGTCAAAGCC
CATCAAGCGGAGGGAGAAAGCAGCTCAGTTCGACTGGAACTTCTTTCTATTGACACTGAGAGGAGACATC
TTCGAGAAAGAGTGGAGCTATTAGAAAAAGAAATTCAAGAGCACATAAATGCCCATCATGCTTATGAATC
TCAGATCTCATCAATGGCAAAAGCCATGTCTCGATTAGAAGAAGAGCTGAGACATCAAGAAGATGAGAAA
GCAACAGTATTAAATGACTTGTCATCTCTTAGAGAACTTTGCATTAAACTTGATTCAGGCAAAGATATTA
TGACCCAGCAACTGAATTCCAAAAACCTTGAGTTTGAGAGGGTTGTAGTGGAATTAGAAAATGTAAAGTC
AGAGTCAGACCTATTGAAAAAACAACTGTCAAATGAGAGACATACAGTTAAAAACCTCGAATCATTGTTG
GCTAAAAACAGAGATAAAGAATTTCATTCTCACTTAACCTCCCATGAGAAGGATACAGAAATCCAGCTAC
TTAAGGAGAAGTTAACCCTTTCTGAAAGCAAATTAACTAGTCAAAGCCGGGAAAACACCATGCTTCGAGC
TAAAGTGGCACAGTTACAAACAGATTATGATGCCCTGAAAAGGCAGGTCTCAACTGAAAGATATGAACGA
GAACGAGCAATCCAAGAGATGCGTCGACATGGTCTTCCTACACCACCCCTTAGTTCCACTCTGAGGTCTC
CTTCACATTCTCCTGAACATATAAATGCG
>Cebus capucinus imitator XM_017540218.1
ATGACTACAGCTGTAGAAAGAAAGTATATTAATATTAGAAAAAGACTGGATCA
GCTGGGATACCGCCAGACTCTGACAGTGGAGTGTTTACCTTTGGTAGAAAAACTTTTCAGTGACTTAGTT
CATACAACAGAGAGCCTTCGGCAATCAAAATTATCTGCTGTGAAAGCTGAAAAAGACAGTGCCAATTTTG
ATTTTGTTTTGGAACCCTATAAACTTGAAAATGCAAGATTGAGTAGAGAAAATAATGAATTGTACCTAGA
GTTAATGAAACTGAGAGAACATTCAGACCAACACATGAAAGAGTTGAAAACTTCATTGAAGAAGTGTGCA
CGTGAAACATCTGATCTGAAATTTCTGAATAACCAGTATGTTCATAAACTCAAACTATTGGAGAAAGAGA
GCAAAGCTAAGAATGAAAGAATTCAACAACTTCAAGAAAAGAATTTGCATGCTGTAGTACAAACTCCAGG
TGGCAAAAAAAGAAGTATTGCTTTCAGGCGCCAGCGTATGCAAATTGATGAACCGGTTCCTCCCTCTGAA
GTCAGTTCATATCCAGTTCCGCAACCAGATGACCCTTACATTGCAGACCTCCTGCAAGTGGCTGATAACA
GGATTCAAGAACTTCAACAGGAAGTGCACCAGCTACAGGAAAAGTTAGCAGTGATGGAAAGTGGGGTGAG
AGATTATAGCAAGCAGATTGAGCTAAGAGAACGAGAGATAGAACGATTGTCAGTTGCTTTGGATGGTGGT
CGCTCTCCTGATGTCCTCTCTCTGGAGTCTAGAAATAAGACCAATGAAAAGCTTATTGCTCAGTTAAATA
TTCAGGTTGACTTTCTTCAGCAAGCTAATAAAGACCTGGAGAAGCATATACAAGAGCTCATGGAAACCAA
GGAAACAGTGACATCTGAAGTTGTTAATTTAAGTAACAAAAATGAAAAACTCTGCCAAGAATTAACTGAA
ATAGATCAGTTAGCACAGCAGTTGGAAAGACATAAAGAAGAAGTGCTTGAGACTGCTGATAAAGAGCTTG
GGGAAGCAAAGAAAGAGATTAAAAGAAAGCTCTCTGAAATGCGGGATCTTGAAGAAACAATGGCAAAACT
TCAACTGGAATTGAACTTATGCCATAAAGAAAAGGAGAGACTGAGTGATGAACTCCTTGTAAAATCAGAT
CTAGAAACTGTTGTTCATCAGCTTGAACAAGAAAAGCAAAGACTTAGCAAAAAAGTTGAAAATTTTGCAG
TTACAGAAAAAGAACTTACTTTGGAAGTTGAAAGGATGAGACTAGAACATGGAATAAAACGTCGAGACAG
GTCACCTTCTCGTTTAGATACATTTCTGAAAGGTATAGAAGAAGAACGAGATTATTATAAGAAAGAGCTA
GAGAGACTCCAACATATAATACAGCGAAGATCTTGCTCTGCAAATTATAGTGCACGTGAAAAAAGTTCAA
TATTTAGAACACCAGAAAAGGGTGATTACAATTCAGAAATTCATCAGATCACAAGAGAAAGAGATGAACT

TCAACATATGCTAGAAAGATTTGAAAAATATATGGAGGATATACAGTCCAATGTTAAATTATTGACAGCA
GAAAGAGATAAACTAAGTGTCTTATATAATGAAGCTCAGGAAGAATTATCCGCCCTAAGACAGGAATCCA
CCCAAACCACAACACCCCATAATATTGTTAGTCTTATGGAAAAGGAAAAAGAGCTTGCATTATCTGACTT
AAGAAGAATTATGACAGAAAAGGAAGCTTTAAAAGAAAAATTAGAGCGTATCGAGGAAATGGGTCTTTAT
GGAAAATCGGAATTAGAGAAAACTATTGAACATTTGACATGTGTTAATCATCAGCTTGAAAGCGAAAAAT
ATGAATTAAAGTCTAAAGTATTAATAATGAAAGAAACAATAGAGTCGTTAGAGAACAAATTAAAAGTCCA
AGCTCAAAAGTTGAGCCATGTGGCTGGTGACTCATCTCATCAGAAAACAGAGGTGAACTCACTTAGGATG
GTGAATGAGCAGCTACAGCGGTCACTTGATGACTGTCAGCACCGACTTTCCATAAAAAGGAGTGAACTTG
ACTCAGCCCAAGCACAAATTAAAATACTGGAGGAAAAAATAGATGAACTAAACCTTAAGATGACTTCACA
GGATGAGGAGGCTCATGTAATGAAAAAGACCATTGGTGTTATTGATAAAGAAAAAGACTTTCTCCAGGAT
ACTGTAGATGAGAAGACAGAAAAGATCGCAAATTTGCAAGAAAACCTAGCTAATAAAGAAAAAGCTGTTG
CTCATATGAAGATAATGGTCTCAGAGTGTGAATCATCTATGAACCAGCTAAAGGAAACATTGACTAATCG
AGATCGTGAGATAAACAGTCTCCGGCGCCAGCTTGATGCAGCTCACAAAGAACTTGATGAAGTAGGAAGA
TCTAGAGAAATTGCTTTTAAGGAAAACAGAAGATTGCAAGATGATCTGGTTACAATGGCAAGAGAAAACC
AAGAAATCTCACTGGAATTGGAAGCAGCAGTGCAAGAAAAAGAAGAAATGAAGAGCAGAGTTCATAAATA
CATAACAGAGGTGTCACGCTGGGAGAGCTTAATGGCTGCTAAGGAAAAAGAAAATCAAGATTTGTTAGAC
AGATTTCAGATGGTTCATAACCGTGCTGAAGACTGGGAGGTCAAAGCCCATCAAGCTGAGGGAGAAAGCA
GCTCAGTTCGACTGGAACTTCTTTCTATTGACACTGAGAGGAGACATCTTCGAGAAAGAGTGGAGCTGTT
AGAAAAAGAAATTCAAGAGCACATAAATGCCCATCATGCTTATGAATCTCAGATCTCATCAATGGCAAAA
GCCATGTCTCGATTAGAAGAAGAGCTGAGACGTCAAGAAGAGGAGAAAGTGACAGTATTAAATGACTTGT
CATCTCTTAGAGAACTTTGCATTAAACTTGATTCAGGCAAAGATATTATGACCCAGCAACTGAACTCCAA
AAACCTTGAGTTTGAGAGGGTCATGGTGGAATTAGAAAATGTAAAGTCAGAGTCAGACGTATTGAAAAAA
CAACTGTCAAATGAGAGACATACAGTTAAAAACCTTGAATCATTGTTGGCTACAAATAGAGATAAAGAAT
TTCATTCTCACTTAACCTCCCATGAGAAGGATACAGAAATCCAGCTACTTAAGGAGAAATTAACCCTTTC
TGAAAGCAAATTAACTAGTCAAAGCCGGGAAAATACCATGCTTCGAGCTAAAGTGGCACAGTTACAAACA
GATTATGATGCTCAGAAAAGACAGATCTCAACTGAGAGATATGAACGAGAACGAGCAATACAAGAGATGC
GTCGACATGGTCTTCCTACACCACCCCTTAGTTCTACTCTGAGGTCTCCTTCACATTCTCCTGAACATAT
CAGTGTG
>Saimiri boliviensis boliviensis XM_010344136.1
ATGACTACAGCTGTAGAAAGAAAGTATGTTAATATTAGAAAAAGACTGGATCAGCTGGGATACCGCCAGA
CTCTGACAGTGGAGTGTTTACCTTTGGTAGAAAAACTTTTCAGTGACTTAGTTCATACAACAGAGAGCCT
TCGGCAATCAAAATTATCTGCTGTGAAAGCTGAAAAAGAAAGTGCCAATTTTGATTTTGTTTTGGAACCC
TATAAACTTGAAAATGCAAGATTGAGTAGAGAAAATAATGAATTGTACCTAGAGTTAATGAAACTGAGAG
AACACTCAGACCAACACATTAAAGAATTGAAAACTTCATTGAAGAAGTGTGCACGTGAAACATCTGATCT
GAAATTTCTAAATAACCAGTATGTTCATAAACTCAAACTATTGGAGAAAGAGAGCAAAGCTAAGAATGAA
AGAATTCAACAACTTCAAGAAAAGAATTTGCATGCTGTAGTACAAACTCCAGGTGGCAAAAAAAGAAGTA
TTGCTTTCAGGCGCCAGCGTATGCAAATTGATGAACCAGTTCCTCCCTCTGAAGTCAGTTCGTATCCAGT
TCCGCAACCAGATGACCCTTACATTGCAGACCTCCTGCAAGTGGCTGATAACAGGATTCAAGAACTTCAA
CAGGAAGTGCAACAGCTACAAGAAAAGTTAGCGGTGATGGAAAGTGGGGTGAGAGATTATAGCAAGCAGA
TCGAGCTAAGAGAGCGAGAGATAGAACGATTGTCAGTTGCCTTGGATGGTGGTCGCTCTCCTGATGTCCT
CTCTCTGGAGTCTAGAAATAAGACCAATGAAAAGCTTATTGCTCAGTTAAATATTCAGGTTGACTTTCTT
CAGCAAGCTAATAAAGACCTGGAGAAGCATATACAAGAGCTCATGGAAACCAAGGAAACAGTGACATCTG
AAGTTGTTAATTTAAGTAACAAAAATGAAAAGCTCTGCCAAGAATTAACTGAAATAGATCAGTTAGCGCA
GCAGTTGGAAAGACATAAAGAAGAAGTGCTTGAGACTGCTGATAAAGAGCTTGGGGAAGCAAAGAAAGAG
ATTAAAAGAAAGCTCTCTGAAATGCGGGATCTTGAAGAAACAATGGCAAAACTTCAACTGGAATTGAACT
TATGCCATAAAGAAAAGGAGAGACTGAGTGATGAACTCCTTGTAAAATCAGATCTAGAAACTGTTGTTCA
TCAGCTTGAACAAGAAAAGCAAAGACTTAGCAAAAAAGTTGAAAATTTTGCAGTTACAGAAAGAGAACTT

ACTTTGGAAGTTGAGAGGATGAGACTAGAACATGGAATAAAACGTCGAGACAGGTCACCTTCTCGTTTAG
ATACATTTCTGAAAGGTATAGAAGAAGAACGAGATTATTATAAGAAAGAGCTAGAGAGACTTCAACATAT
AATACAGCGAAGATCTTGCTCTGCAAATTATAACGCACGTGAAAAAAGTTCAATATTTAGAACACCAGAA
AAGGGTGATTACAATTCAGAAATTCATCAGATCACAAGAGAAAGAGATGAACTTCAACGTATGCTAGAAA
GATTTGAAAAATATATGGAGGATATACAATCCAATGTTAAATTACTGACGGCAGAAAGAGATAAACTAAG
TGTCTTATATAATGAAGCTCAGGAAGAATTATCCGCCCTAAGACAGGAATCCACCCAAACCACAACACCC
CATAATATTGTTAGTCTTATGGAAAAGGAAAAAGAACTTGCGTTATCTGACTTAAGAAGAATTATGACAG
AAAAGGAAGCTTTAAAAGAAAAATTAGAGCATATTGAGGAAATGGGTCTTTATGGAAAATCAGAATTAGA
GAAAACTATTGAACATTTGACATGTGTTAATCATCAGCTTGAAAGTGAAAAATACGAATTAAAGTCTAAA
GTGTTAATAATGAAAGAAACAATAGAGTCGTTAGAGAACAAATTAAAAGTCCAAGCTCAAAAATTTAGCC
ATGTGGCTGGTGACTCATCTCATCAGAAAACGGAGGTGAACTCACTTAGGATGGTGAATGAGCAGCTACA
ACGGTCGCTTGATGACTGTCAGCACCGACTTTCCATAAAAAGGAGTGAACTTGACTCAGCCCAAGCACAA
ATTAAAATACTGGAGGAAAAAATAGATGAACTAAACCTTAAGATGACTTCACAGGATGAGGAGGCTCATG
TAATGAAAAAGACGATTGGTGTTATTGATAAAGAAAAAGACTTTCTCCAGGATACTGTAGATGAGAAGAC
AGAAAAGATCGCAAATTTGCAAGAAAACCTAGCTAATAAAGAAAAAGCTGTTGCTCATATGAAGATAATG
GTCTCAGAGTGTGAATCATCTATGAACCAGCTAAAGGAAACATTGACTAATCGAGATCGTGAGATAAACA
GTCTCCGGCGCCAGCTTGATGCATCTCACAAAGAACTTGATGAAGTAGGAAGATCTAGAGAAATCGCTTT
TAAGGAAAACAGAAGATTGCAGGATGATCTGGTTACAATGGCAAGAGAAAACCAAGAAATCTCACTGGAA
TTGGAAGCAGCAGTGCAAGAAAAAGAAGAAATGAAGAGCAGAGTTCATAAATACATAACAGAGGTGTCAC
GATGGGAGAGCTTAATGGCTGCTAAGGAAAAAGAAAATCAAGATTTGTTAGACAGATTTCAGATGGTTCA
TAACCGTGCTGAAGACTGGGAGGTCAAAGCCCATCAAGCTGAGGGAGAAAGCAGCTCAGTTCGACTGGAA
CTTCTTTCTATTGACACTGAGAGGAGACATCTTCGAGAAAGAGTGGAGCTGTTAGAAAAAGAAATTCAAG
AGCACATAAATGCCCATCATGCTTATGAATCTCAGATCTCATCAATGGCAAAAGCCATGTCTCGATTAGA
AGAAGAGCTGAGACGTCAAGAAGAGGAGAAAGTGACAGTATTAAATGACTTGTCATCTCTTAGAGAACTT
TGCATTAAACTTGATTCAGGCAAAGATATTATGACCCAGCAACTGAATTCCAAAAACCTTGAGTTTGAGA
GGGTTATGGTGGAATTAGAAAATGTAAAGTCAGAGTCAGACCTGTTGAAAAAACAACTGTCAAATGAGAG
ACATACAGTTAAAAATCTTGAATCATTGTTGGCTACAAACAGAGATAAAGAATTTCATTCTCACTTAACC
TCCCATGAGAAGGATACAGAAATCCAGCTACTTAAAGAGAAATTAACCCTTTCTGAAAGCAAATTGACTA
GTCAAAGCCGGGAAAACACCATGCTTCGAGCTAAAGTGGCACAGTTACAAACAGATTATGATGCTCAGAA
AAGACAGATCTCAACTGAGAGATACGAACGAGAACGAGCAATCCAAGAGATGCGTCGACATGGTCTTCCT
ACACCACCCCTTAGTTCTACTCTGAGGTCTCCTTCACATTCTCCTGAACATATCAATGTG
>Carlito syrichta XM_008060323.1
ATGACTACAGCTGTAGAAAGAAAGTATCTTAATATTAGAAAAAGATTGGATCAACTGGGATACCGCCAGA
CTCTGACAGTAGAATGTTTACCTTTGGTAGAAAAACTTTTCAGTGACCTGGTTCACACAACAGAAAGCCT
TCGACAATCAAAATTATCTACTGTGAAAGCTGAAAAAGAAAGTGCCAATTTTGATTTTGTTTTGGAACCC
TATAAACTTGAAAATGCAAGATTGACTAAGGAAAATAATGAATTGTACCTAGAGTTAATGAAACTGAGAG
AACACTCAGATCAACACATTAAAGAGTTGAAAACTACATTGAAGAAGTGTGCTCATGAAACAACTGATCT
GAAATTTCTAAATAATCAGTATGTTCATAAACTCAAACTTTTGGAGAAAGAGAGCAAAGCTAAAAATGAA
AAAATTCAACAGCTTCAAGAAAAGAATTTGCATGCTGTAGTACAGACTCCAGGTGGCAAGAAAAGAAGCA
TTGCTTTCCGGCGTCAGCGTATGCAAATTGATGAACCAGTCCCTCCCTCTGAAGTTAGCTCCTATCCAGT
CCCACAGCCTGAGGACCCTTACATTGCAGACCTCCTGCAGGTGGCTGACAACAGGATTCAAGAGCTTCAA
CAGGAAGTCCACCATCTACAAGAAAAGTTAGCAGCCATGGAAAGTGGAGTGAGAGATTATAGCAGGCAGA
TTGAGCTAAGAGAACGAGAGATAGAACGACTGTCGGTTGCTTTGGATGGTGGTCGCGCCCCTGATGTCAT
CTCTCTGGAGTCCAGGAATAAAAACAATGAAAAGCTCATCGCTCAGTTAAATATTCAGGTTGACTTTCTT
CAGCAAGCTAATAAAGACCTAGAGAAGCATATACAAGAGCTTATGGAAACCAAGGAAACAGTGACATCCG
AAGTTGTTAATTTAAGTAACAAAAATGAAAAACTCTGCCAAGAATTAACGGAAATAGACCAGTTAGCACA
GCAGTTGGAAAAACATAAGGAAGAAGTGCTTGAGACTGCTGATAAGGAGCTTGGGGAAGCAAAGAAAGAG

ATTAAACAAAAGCTGTCTGAAATGCGGAATCTTGAAGAAACAATGGCAAAACTTCAGCTGGAATTAAACT
TATGCCATAAAGAAAAGGAGAGACTGAGTGATGAGCTCCTTATAAAATCTGACCTGGAAACTGTTGTTCA
TCAGCTTGAACAAGAAAAGCAAAGACTTAGCAAAAAAATGGAAAGTTTTACAGTTACAGAGAGAGAACTT
ACTTTGGAAGTTGAGAGGATGAGACTGGAACATGGAATAAAACGACGAGACAGATCGCCTTCTCGTTTGG
ATGCATTTCTAAAAGGCATTGAAGAAGAACGAGATTATTATAAGAAAGAACTAGAAAGACTCCAGCATAT
AATACAGCGAAGATCTTGCTCTACAAGTAATAGTGCACGTGAAAAGAGTTCAACATTTAGAACACAAGAT
AAGGGTGATTACAATTCAGCTGTTCATCAGATTACAAGAGAAAGAGATGAACTTCAGCGTATGCTAGAAA
GATTTGAAAAATATATGGAGGATATACAGTCCAATGTTAAATTATTGACAGCAGAAAGAGATAAACTAAG
TGTGTTGTATAATGAAGCTCAGGAAGAATTATCTGCCCTAAAACAGGAATCTGCCCAAACTGCAGCCTCC
CATAATATTGTTAGTATTATGGAAAAGGAAAAAGAACTTGCGTTATCTGATTTAAGAAGAATTATGGCAG
AAAAGGAAGCTTTAAGAGAAAAGTTACAAAATATCCAGGAAATGAATCTTCTTGGAAAATCAGAATTAGA
AAAAACAATTGAACATTTGACACGTGTTAATCATCAGCTTGAAAGCGAAAAATATGAATTAAAGTCTAAA
ATGTTAATAATGAAAGAAACAATAGAGTCATTAGAGAACAATTCAAAACTCCAAGCTCAAAAACTTAGCC
ATGTGGCTGGTGACTCATCTTATCAGAAAACAGAGATGAACTCACTTAGGGTGGTAAATGAGCAGCTGCA
GCGGTCACTGGATGATTATCAGCACCGACTTTACATAAAAAGAGATGAACTTGAATCAGCTCATGCACAA
ATTAAAATACTAGAGGAAAAAATAGATAAAATAAACCTTAAGATGACTTCACAGGATGAGGAGGCAAATG
TAATGAAAAAGACCATTGGTGTTATTGATAAAGAAAAAGACTTTCTTCAGGAGACGGTAGATGAGAAGAC
AGAAAAGATTGCAAACTTGCAAGAAAACATAGCTAACAAAGAAAAAGCTATTGCTCAGATGAAGATAACA
TTCTCAGAGTGTGAATCATCTCTAAACCAGTTGAAGGAAACCTTGACTAATCGGGACCGGGAGATAAGCA
GCCTCCGGCGCCAGCTTGATGCAGTTCACAAAGAGCTTGAAGAGGTAGGAAGATCTAGAGAAATCTCTCT
TAAGGAGAACAGAAGATTACAAGATGATCTGGCTACAATGGCAAGAGAAAACCAGGAAATCTCGTTGGAA
CTGGACTCAGCAGTGCAAGAAAAAGAAGAAATGAAGAGTAGAGTTCATAATTACATAACTGAGGTGTCAC
GATGGGAGAGCTTAATGGCCACGAAGGAAAAAGAAAATCAGGATTTGTTAGATAGATTTCAGATGCTTCA
CCACCGTGCTGAAGACTGGGAGGTCAAAGCCCATCAAGCTGAGGGGGAAAGCAGCTCAGTGCGGCTGGAG
CTTCTGTCTATTGACACCGAGAGGAGACACCTTCGAGAAAGGGTGGAGCTATTGGAAAAGGAAATTCAAG
AGCATATAAATGCACATCATGCTTACGAATCTCAGATCTCATCAATGGCAAAAGCCATGTCTAGATTAGA
AGAAGAGCTGAGACGTCAAGAAGATGACAAAGCTGCAGTGTTAAATGATTTGTCATCTCTGAGAGAACTG
TGCATTAAGCTTGATTCAGGCAAAGATATTATGACCCAGCAGTTAAAGTCCAAAAACCTTGAGTTTGAGA
GGGTTGTGGTAGAATTAGAAAACGTAAAATCAGAATCAGACCTGTTAAAAAAACAACTGTCAAATGAGAA
ACATACGGTTAAAAGCCTCGAGTCATTGCTGGCTACAAATAGAGATAAAGAGTTTCATTCTCATTTAACT
TCCCACGAGAAGGATACAGAAATTCAGCTACTTAAGGAGAAGCTAACCCTTTCAGAAAGCAAACTAACTA
GTCAAAGCCGGGAAAACACCATGCTTCGAGCTAAAGTGGGACAGTTACAGACAGATTATGATGCTCTGAA
GAGGCAGATTTCAACAGAAAGATATGAACGGGAACGAGCAATCCAAGAGATGCGTCGCCATGGTCTTCCT
ACGCCACCCCTGAGTTCTACTCTGAGATCGCCTTCAAATTCTCCTGAACATATAAATATA
>Rhinopithecus roxellana XM_010355235.1
ATGACTACAGCTGTAGAAAGAAAGTATATTAATATTAGGAAAAGACTGGATCAGCTGGGATACCGCCAGA
CTCTGACAGTAGAGTGTTTACCTTTGGTAGAAAAACTTTTCAGCGACTTAGTTCATACAACAGAGAGCCT
TCGGCAATCAAAATTATCTGCTGTGAAAGCTGAAAAAGAAAGTGCCAATTTTGATTTTGTTTTGGAACCC
TATAAACTTGAAAATGCAAGATTGAGTAGAGAAAATAATGAATTATACCTAGAGTTAATGAAACTGAGAG
AACATTCGGACCAACACGTTAAAGAGTTGAAAACTTCATTGAAGAAGTGTGCACGTGAAACAGATGATCT
GAAATTTCTAAATAACCAATATGTTCATAAACTCAAACTGTTGGAGAAAGAGAGCAAAGCTAAGAATGAA
AGAATTCAACAACTTCAAGAAAAGAATTTGCATGCTGTAGTACAAACTCCAGGTGGCAAGAAAAGAAGTA
TTGCTTTCAGGCGCCAGCGTATGCAAATTGATGAACCGGTTCCTCCCTCTGAAGTCAGTTCATATCCGGT
TCCTCAACCAGATGACCCTTACATTGCAGACCTCCTACAAGTGGCTGATAACAGGATTCAAGAACTTCAA
CAGGAAGTCCGCCAGCTACAAGAAAAGTTAGCAGTGATGGAAAGTGGGGTGAGCGATTATAGCAAGCAGA
TCGAGCTAAGAGAACGAGAGATAGAACGACTGTCAGTTGCTTTGGATGGTGGTCGGTCTCCTGATGTCCT
CTCTCTGGAGTCTAGAAATAAAACCAATGAAAAGCTTATTGCTCATTTAAATATTCAGGTTGACTTTCTT

CAGCAAGCTAATAAAGACCTGGAGAAGCGTATACGAGAGCTTATGGAAACCAAGGAAACGGTGACATCTG
AAGTTGTTAATTTAAGTAACAAAAATGAAAAACTCTGCCAAGAATTAACTGAAATAGATCAGTTAGCACA
GCAATTGGAAAGACATAAAGAAGAAGTGCTTGAGACTGCTGATAAAGAACTTGGGGAAGCAAAGAAAGAG
ATTAAAAGAAAGCTGTCTGAAATGCGGGATCTTGAAGAAACAATGGCAAAACTTCAACTGGAATTGAACT
TATGCCATAAAGAAAAGGAGAGACTGAGTGATGAACTCCTTGTAAAATCAGACCTTGAAACTGTTGTTCA
TCAGCTTGAAGAAGAAAAGCAAAGACTTAGCAAAAAAGTTGAAAGTTTTGTAGTTACAGAAAGAGAACTT
ACTCTGGAAGTTGAGAGGATGAGACTAGAACATGGAATAAAACGTCGAGACAGGTCACCTTCTCGTTTAG
ATACATTTCTGAAAGGTATAGAAGAAGAACGAGATTATTATAAGAAAGAGCTAGAGAGACTCCAACATAT
TATACAGCGAAGATCTTGCTCTACAAATTATGGCGCACGTGAAAAAAATTCAATATTTAGAACACCAGAA
AAGGGTGATTACAATTCAGAAATTCATCAGATCACAAGAGAAAGAGATGAACTTCAGCGTATGCTGGAAA
GATTTGAAAAATATATGGAGGATATACAGTCAAATGTTAAATTACTGACAGCAGAAAGAGATAAACTAAG
TGTCTTATATAAAGAATCTCAAGAAGAATTATCTGCCCTAAGAAAGGAATCCACCCAAACCACAGCACCC
CATAATATTGTTAGTCTTATGGAAAAGGAAAAAGAACTTGCGTTATCTGACTTAAGAAGAATTATGGCAG
AAAAGGAAGCTTTAAGAGAAAAATTAGAGCATATTGAAGAAATGAGTCTTTTTGGAAAATCAGAATTAGA
GAAAACTATTGAACATTTGACATGTGTTAATCATCAGCTTGAAAGCGAAAAATATGAATTAAAGTCTAAA
GTATTAATAATGAAAGAAACAATAGAGTCGTTAGAGAACAAATTAAAAGTCCAAGCTCAAAAATTTAGCC
ATGTGGCTGGTGACTCATCTCATCAGAAAACAGAGGTGAACTCACTTAGGATAGTAAATGAGCAGCTACA
GCGGTCAGTTGATGACTATCAGCACCGACTTTCCATTAAAAGAGGTGAACTTGAATCAGCCCAAGCACAA
ATTAAAATACTGGAGGAAAAAATAGATGAATTAAACCTTAAGATGACTTCACAGGATGAGGAGGCTCATG
TAATGAAAAAGACCATTGGTGTTATTGATAAAGAAAAAGACTTTCTCCAGGAGACTGTAGATGAGAAGAC
AGAAAAGATTGCAAATTTGCAAGAAAACCTAGCTAATAAAGAGAAAACTGTTGCTCAAATGAAGCTAATG
GTCTCAGAGTGTGAATCAGCTGTGAACCAGCTAAAAGAAACATTAGTTAATCGAGATCGTGAGATAAACA
GCCTCCGGCGCCAGCTTGATGCAGCTCACAAAGAACTCGATGAAGTAGGAAGATCTAGAGAAATTGCTTT
TAAGGAAAACAGAAGATTGCAAGATGACCTGGCTACGATGGCAAGAGAAAACCAAGAAATCTCATTGGAA
TTGGAAGCAGCAGTGCAAGAAAAAGAAGAAATGAAGAGCAGAGTTCATAAATACATAACAGAGGTGTCAC
GATGGGAGAGCTTAATGGCTGCCAAGGAAAAAGAAAATCAAGATTTGTTAGATAGATTTCAGATGCTTCA
TAACCGTGCTGAAGACTGGGAGGTCAAAGCCCATCAAGCGGAGGGAGAAAGCAGCTCAGTTCGACTGGAA
CTTCTTTCTATTGACACTGAGAGGAGACATCTTCGAGAAAGAGTGGAGCTATTAGAAAAAGAAATTCAAG
AGCACATAAATGCCCATCATGCTTATGAATCTCAGATCTCATCAATGGCAAAAGCCATGTCTCGATTAGA
AGAAGAGCTGAGACATCAAGAAGATGAGAAAGCAACAGTATTAAATGACTTGTCATCTCTTAGAGAACTT
TGCATTAAACTTGATTCAGGCAAAGATATTATGACCCAGCAATTGAATTCCAAAAACCTTGAGTTTGAGA
GGGTTGTAGTGGAATTAGAAAATGTAAAGTCAGAGTCAGACCTATTGAAAAAACAACTGTCAAATGAGAG
ACATACAGTTAAAAACCTTGAATCATTGTTGGCTACAAACAGAGATAAAGAATTTCATTCTCACTTAACC
TCCCATGAAAAGGATACAGAAATCCAGCTACTTAAGGAGAAGTTAACCCTTTCTGAAAGCAAATTAACTA
GTCAAAGCCGGGAAAACACCATGCTTCGAGCTAAAGTGGCACAGTTACAAACAGATTATGATGCCCTGAA
AAGGCAGATCTCAACTGAAAGATATGAACGAGAACGAGCAATCCAAGAGATGCGTCGACATGGTCTTCCT
ACACCACCCCTTAGTTCCACTCTGAGGTCTCCTTCACATTCTCCTGAACATATAAATGCG
>Aotus nancymaae XM_012441173.1
ATGACTACAGCTGTAGAAAGAAAGTATATTAATATTAGAAAAAGACTGGATCA
GCTGGGATACCACCAGACTCTGACAGTGGAGTGTTTACCTTTGGTAGAAAAACTTTTCAGTGACTTAGTT
CATACAACAGAGAGCCTTCGGCAATCAAAATTATCTGCTGTGAAAGCTGAAAAAGAAAGTGCCAATTTTG
ATTTTGTTTTGGAACCCTATAAACTTGAAAATGCAAGATTGAGTAGAGAAAATAATGAATTGTACCTAGA
GTTAATGAAACTGAGAGAACACTCAGACCAACACATTAAAGAGTTGAAAACTTCATTGAAGAAGTGTGCA
CGTGAAACATCTGATCTGAAATTTCTAAATAACCAATATGTTCATAAACTCAAACTATTGGAGAAAGAGA
GCAAAGCTAAGAATGAAAGAATTCAACAACTTCAAGAAAAGAATTTGCATGCTGTAGTACAAACTCCAGG
TGGCAAAAAAAAGAAGTATTGCTTTCAGGCGCCAGCGTATGCAAATTGATGAACCGGTTCCTCCCTCTGAA
GTCAGTTCATATCCAGTTCCGCAACCAGATGACCCTTACATTGCAGACCTCCTGCAAGTGGCTGATAACA

GGATTCAAGAACTTCAGCAGGAAGTGCACCAGCTACAAGAAAAGTTAGCAGTGATGGAAAGTGGGGTGAG
AGATTATAGCAAGCAGATTGAGCTAAGAGAACGAGAGATAGAACGATTGTCAGTTGCTTTGGATGGTGGT
CGCTCTCCTGATGTCCTCTCTCTGGAGTCTAGAAATAAGACCAATGAAAAGCTTATTGCTCAGTTAAATA
TTCAGGTTGACTTTCTTCAACAAGCTAATAAAGACCTGGAGAAGCATATACAAGAGCTCATGGAAACCAA
GGAAACAGTGACATCTGAAGTTGTTAATTTAAGTAACAAAAATGAAAAACTCTGCCAAGAATTAACTGAA
ATAGATCAGTTAGCGCAGCAGTTGGAAAGACATAAAGAAGAAGTGCTTGAGACTGCTGATAAAGAGCTTG
GGGAAGCAAAGAAAGAGATTAAAAGAAAGCTCTCTGAAATGCGGGATCTTGAAGAAACAATGGCAAAACT
TCAACTGGAATTGAACTTATGCCATAAAGAAAAGGAGAGACTGAGTGATGAACTCCTTGTAAAATCAGAT
CTAGAAACTGTTGTTCATCAGCTTGAACAAGAAAAGCAAAGACTTAGCAAAAAAGTCGAAAATTTTGCAG
TTACAGAAAGAGAACTTACTTTGGAAGTTGAGAGGATGAGACTAGAACATGGAATAAAACGTCGAGACAG
GTCACCTTCTCGTTTAGATACATTTCTGAAAGGTATAGAAGAAGAACGAGATTATTATAAGAAAGAGCTA
GAGAGACTCCAACATATAATACAGCGAAGATCTTGCTCTGCAAATTATAGTGCACGTGAAAAAAGTTCAA
TATTTAGAACACCAGAAAAGGGTGATTACAATTCAGAAATTCATCAGATCACAAGAGAAAGAGATGAACT
TCAACATATGCTAGAAAGATTTGAAAAATATATGGAGGATATACAGTGCAATGTTAAATTATTGACAGCA
GAAAGAGATAAACTAAGTGTCTTATATAATGAAGCTCAGGAAGAATTATCCACCCTAAGACAGGAATCCA
CCCAAACCACAACATCCCATAATATTGTTAGTCTTCTGGAAAAGGAAAAAGAACTTGCATTATCTGACTT
AAGAAGAATTATGACAGAAAAGGAAGCTTTAAAAGAAAAATTAGAGCATATTGAGGAAATGGATCTTTAT
GGAAAATCAGAATTAGAGAAAACTATTGAACATTTGACATGTGTTAATCATCAGCTTGAAAGCGAAAAAT
ATGAATTAAAATCTAAAGTGTTAATAATGAAAGAAACAATAGAGTCGTTAGAGAACAAATTAAAAGTCCA
AGCTCAAAAATTTAGCCATGTGGCTGGTGACTCATCTCATCAGAAAACGGAGGTGAACTCACTTAGGATG
GTGAATGAGCAGCTACAACGGTCACTTGATGACTGTCAGCACCGACTTTCCATAAAAAGGAGTGAACTTG
ACTCAACCCAAGCACAAATTAAAATACTGGAGGAAAAAATAGATGAACTAAACCTTAAGATGACTTCACA
GGATGAGGAGGCTCATGTAATGAAAAAGACCATTGGTATTATTGATAAAGAAAAAGACTTTCTCCAGGAT
ACTGTAGATGAGAAGACAGAAAAGATCGCAAATTTGCAAGAAAACCTAGCTAATAAAGAAAAAGCTGTTG
CTCATATGAAGATAATGGTCTCAGAGTGTGAATCATCTATGAACCAGCTAAAGGAAACATTGACTAATCG
AGATCGTGAGATAAACAGTCTCCGGCGCCAGCTTGATGCAGCCCACAAAGAACTCGATGAAGTAGGAAGA
TCTAGAGAAATCGCTTTTAAGGAAAACAGAAGATTGCAAGATGATCTGGTTACAATGGCAAGAGAAAACC
AAGAAATCTCACTGGAATTGGAAGCAGCAGTGCAAGAAAAAGAAGAAATGAAGAGCAGAGTTCATAAATA
CATAACAGAGGTGTCACGATGGGAGAGCTTAATGGCTGCTAAGGAAAAAGAAAATCAAGATTTGTTAGAC
AGATTTCAGATGGTTCATAACCTTGCTGAAGACTGGGAAGTCAAAGCCCATGAAGCTGAGGGAGAAAGCA
GCTCAGTTCGACTGGAACTTCTTTCTATTGACACTGAGAGGAGACATCTTCGAGAAAGAGTGGAACTGTT
AGAAAAAGAAATTCAAGAGCACATAAATGCCCATCATGCTTATGAATCTCAGATCTCATCAATGGCAAAA
GCCATGTCTCGATTAGAACAAGAGCTGAGACGTCAAGAAGAGGAAAAAGTGACAGTATTAAATGACTTGT
CATCTCTTAGAGAACTTTGCATTAAACTTGATTCAGGCAAAGATGTTATGACCCAGCAACTGAATTCCAA
AAACCTTGAGTTTGAAAGGGTTATGGTGGAATTGGAAAATGTAAAGTCAGCATCAGACCTGTTGAAAAAA
CAACTGTCAAATGAGAGACATACAGTTAAAAACCTTGAATCATTGTTGGCTACAAACAGAGATAAAGAAT
TTCATTCTCACTTAACCTCCCATGAGAAGGATACAGAAATCCAGCTACTTAAGGAGAAATTAACCCTTTC
TGAAAGCAAATTAACTAGTCAAAGCCGGGAAAACACCATGCTTCGAGCTAAAGTGGCACAGTTACAAACA
GATTATGATGCTCAGAAAAGACAGATCTCAACTGAGAGATATGAACGAGAACGAGCAATCCAAGAGATGC
GTCGACATGGTCTTCCTACACCACCCCTTAGTTCTACTCTGAGGTCTCCTTCACATTCTCCTGAACATAT
CAATGTG
>Cavia porcellus ENSCPOT00000003255.2
ATGACGACAGCTGCAGAGAGAAAGTATATTAATATTAGGAAAAGATTGGATCAGCTGGGG
TACCGTCAGACTCTGACAATCGAGTGTTTACCTTTGGTAGAAAAGCTGTTCAGTGACTTG
GTTCATACAACAGAAAGCCTTCGACAAGCAAAATTATCTTCTGTGAAAGCTGAAAAGGAA
AGTGCCAATTTTGATTTTATTTTGGAACCCTATAAACTTGAAAATGCAAGATTGAATAAA
GAAAATAATGAGTTATATCTGGCATTAATGAAACTGAGAGAGTGCTCAGAGCAAAATATT

AAAGAGTTGAAAACTACATTGAAGAAGTGTGCACGTGAAACAGCAGATCTGAAATTTCTA
AATAACCAATACGTTCACAAACTCAAACTGTTGGAGAAAGAGAGCAGAGCTAAAAATGAG
AAAATTCAACAACTTCAAGAAAAGAATTTGCGTGCTGTCGTACAAACTCCAGGTGGAAAG
AAAAGAAGTATTGCCTTCAGACGCCAGCGGATGCAAATCGATGAGCCAGTCCCTCCCTCT
GAAGTCAGTTCTTATCCAGTTCCACAGCCAGAAGACCCTTACATTGCGGACCTCCTGCAA
GTGGCTGATAACAGGATCCAAGAACTTCAGCAGGAAGTCCATGAGCTGCAAGAAAAGTTA
GCAGTGATGGAAAGTGGTGTGAGAGATTATAGCAAGCAGATCGAGCTACGAGAACGAGAG
ATAGAGCGACTGTCAGTTGCTTTGGATGGCAGTCGCTCTCCAGATATCTTGTCTCTGGAG
ACTAAAAATAAAACCAATGAAAAGGTGATTGCTCATTTAAATGTACAGGTTGACTTCCTT
CAGCAAGCTAATAAAGACCTGGAGAAGCGTATTCAAGAGCTTATGGAAACCAAGGAAACA
GTGGCAACTGAAGTAGTTAATTTAAGTAACAAAAATGAAAAACTCTGCCAGGAATTAACT
GAAATAGACCAGTTAGCACAGCAGCTAGAAAGACATAAAGAAGAAGTGCTTGAGACCGCT
GATAAAGAACTCGGGGAAGCAAAGAAAGAGATTAAAAGAAACCTCTGTGAAATGCAAAAT
CTTGAAGAAACCATGGCAAAACTTCAACTGGAATTAGACTTATGCCAAAAAGAAAAAGAA
AGACTGAGTGATGAACTCCTTCTAAAATCAGATCTGGAAACTGTTGTTCATCAGCTTGAA
CAAGAAAAGCAAAGACTTACCAAAAAAATTGAAAGTTTTGCATTAACAGAAAGAGAACTA
ACTTTGGAAGTTGAGAGGATGAGACTAGAACATGGAATAAAACGTCGAGACAAGTTACCT
TCTCGTTTAGATACATTTCTCAAAGGTATAGAAGAAGAACGAGATTATTATAAGAAAGAG
CTAGAAAGACTCCAACATATCATACAGAAAAGATCTTGCTCTATAAATTACTGTGCACGT
GAAAAAAATTCAGTATTTAAAACACCAGAAAAGGGCGATTACAATGCAGAAATTCATCTG
GTCACAAGAGAACGAGATGAACTCCAGTGTATGCTAGAAAGATTTGAAAAATATATGGAA
GATATACAGTGCAATGTTAAATTATTGACAGCAGAAAGAGATAAACTAAGTGTCTTATAT
AATGAAGCTCAGGAAGAATTATGTGCACTAAGACAGGAATCAACCAACACCACTGTCCCT
AGTAGCCTTGTTAATATTATGGAAAAGGAAAAGGAACTTGCTGTATCTGATTTAAGAAGA
ATGATGGCAGAAAAAGAAGATTTAAGAGAAAAGTTAAAAAATATCGAGGAAATGAGTGCT
TTTGGCAAAGCAGAATTAGAGAAAACTATTGAACATTTGACATGTGTTAATCATCAGCTG
GAAAGTGAAAAATACGAATTACAATCTAAAGTGATAATAATGAAAGAAACAGTAGAGTCA
CTAGAGAACAAATCCAAACTCCAAGCTCACAAGCTTAGCCATCTCATGGCTGGTGACTCA
TCCTATCACAAAACAGAGTTGAACTCTCTTAGGATGGTAAATGAGCAGCTACAGCAGTCA
CTTGATGACTATCAGCACCGACTGTCCATAAAAAGAGGTGAACTTCAATCTGCCCATGAA
CAAATTAAAATACTGGAAGAAAAAATAGATACGATAAACCTTAAGATGACTTCACAGGAT
GAAGAGGCTCATATAATGAAAAAGACTATTGGTGTTATTGATAAAGAGAAAGATTTCCTC
CAGGAAACTGTGGATGATAAGACAGAAAAGATTGCAAACTTAGAAGAAGTCCTCACTAGT
AAAGAAAAAGCTGTTGCTCAGATGAAGACAACTATCTCAGAGTATGAATTATCTCTGACC
CGATTAAAGGAAACATTGGGTAATCGAGACCGGGAAATCAGCAGCCTCCGGCAACAGCTT
GATGCGGCTCTCAAAGAACTGAATGAAGTAGGGAGGACTAGAGAAATGTCTTTTAAGGAA
AACAGAAGATTACAAGATGATCTGGCTACAGTGGCGAGAGAAAATCAGGAAATCTCATTG
GAATTGGAGGCAGCAGTGCAAGAAAAAGAAGAAATGAAGAGTAGAGTTCATAATTACATA
ACTGAGGTGTCCCGATGGGAGAGCTTAATGGCTGCTAAGGAGCAAGAAAATCAAGATTTG
TTAGATAGATTTCAGATGCTTCACAACTGTGTCGAAGACTGGGAGATCAAGGCTCATCAA
GCTGAAGGAGAAAGCAGCTCAGTTCGACTAGAACTTCTTTCTATTGACACAGAGAGAAGA
CACCTTCGAGAAAGAGTGGACCTATTAGAAAAAGAAATTCAAGAGCACATAAATGCGCAT
CATGCTTATGAATCTCAGATCTCATCAATGGCAAAAGGCATGGCTAGATTAGAAGAAGAG
CTGAGACATCAAGAAGATGAGAAAGCAACAGTGTTAAGTGATTTGTCTTCTCTTAGAGAA
CTTTGCATTAAGCTCGATTCAGGCAAAGATGTTATGACCCAACAATTGAATGCTAAAAGT
CTTGAGTTGGAGAGGGCACTAGCAGAATTAGAAAATGTAAAATCAGAATCAGAACTATTA
AGAAAACAGCTGTCAAATGAGAGATACACGATTAAAAACCTCGAATCATTGTTGGCTACA

AACAGAGATAAAGAATTTCATTCTCATTTATCCTCCCATGAAAAGGATTCAGAAATTCAG
CTACTTAGGGAGAAGTTAACCCTTTCAGAAAACAAATTAACTAGTCAAAGCCAGGAAAAC
ACCATGCTTCGAACTAAAGTGGGACAGTTACACACAGATTATGATTCTCTAAAAAAGCAG
ATTACAACAGAAAGATATGAACGAGAACGAGCAATCCAAGAGATGCGTCGACATGGTCTT
CCTATGCCACATCTTAGTTCTACCCTGAAGTCTCCTTCACATTCTCCAGAACGAAAGGGT
TTG
>Ictidomys tridecemlineatus XM_005320022.2
ATGACTACAACTGCAGAAAGAAAGTATATTAATA
TTAGAAAAAGATTGGATCAGTTGGGATACCGCCAGACTCTGACATTGGAGTGTTTACCATTGGTGGAAAA
ACTTTTCAGTGACTTAGTTCATACAACAGAAAGCCTTCGACAATCAAAATTATCTACTGTGAAAGCTGAA
AAGGAAAGTGCCAATTTTGATTTTGTATTGGAACCCTATAAACTTGAAAATGCAAGATTGAGTAGGGAAA
ATAATGAATTATACCTGGAATTAATGAAACAGAGAGAGTACTCAGACCAACACATTAAAGAATTGAAAAC
TACTTTGAAAAAGTGTGCACGTGAAACCGCTGATCTGAAATTTCTAAATAACCAATATGTTCATAAACTC
AGACTTTTGGAGAAAGAAAGCAAAGCTAAGAATGAAAAAATTCAGCAACTTCAAGAAAAGAATTTGCATG
CTGTAGTACAAACTCCAGGTGGCAAGAAAAGAAGCATTGCTTTCAGGCGCCAGCGTATGCAAATAGATGA
ACCAGTTCCTCCTTCTGAAGTTAGTGGTTATCCAGTTCCACAGCCAGATGACCCTTACATTGCAGACCTC
CTGGAAGTGGCTGATAATAGGATTCAAGAGCTTCAGCAGGAAGTCCACCATCTACAAGAAAAGTTAGCAA
TGATGGAAAATGGCATAAAAGATTACAGCAAGCAGATTGAGCTAAGAGAACGAGAAATAGAAAGACTGTC
AGGTACTTTGGATGGTGGTCGCTCCCCTGATGTCCTATCTCTGGAGAATAGAAATAAAACCAATGAAAAG
CTTATTGCTCATTTAAATATTCAGGTTGACTTTCTTCAGCAAGCTAATAAAGACCTGGAGAAGCATATTC
AAGAGCTTATGAAAACCAAGGAAACAGTGACGACTGAAGTTGTTAATTTAAGTAACAAAAATGAAAAACT
CTGCCAAGAATTAACTGAAATAGACCAGTTAGCACAGCAGTTGGAAAGACATAAAGAAGAAGTACTTGAG
ACTGCTGATAAAGAACTTGGGGAAGCAAAGAAAGAGATTAAAAGAAACCTCTCTGAAATGCGGAATCTTG
AGGAAACAATGGCAAAACTTCAACTGGAATTAAGTTTATGCCATAAAGAAAAGGAGAGACTAAATGATGA
ACTCCTTCTGAAATCAGACCTAGAAACTGTTGTACATCAGCTTGAACAAGAAAAGCAAAGACTTAGCAAA
AAAATGGAAGGTTTTGCAGCTATAGAAAAAGAACTTACTTTGGAAGTTGAAAGGATGAGGCTAGAACATG
GAATAAAACGTCGAGACAGGTCACCTTCTCGTTTAGATACATTTCTGAAAGGTATAGAAGAAGAACGAGA
TTATTATAAGAAAGAACTAGAAAGACTCCAACATATAATACAACGAAGATCTTGCTCTACAAATCATTGC
GCACGTGAAAAACATTCAGTATTTAAAACACTAGAAAAGGGTGATTATAATTCAGATATTCATCTGATTG
CAAGAGAAAGAGATGAACTTCAGCGTATGCTAGAAAGATTTGAAAAATATATGGAAGATATACAGTCCAA
TGTTAAATTATTGACAGCAGAAAGAGATAAACTAAGTGTCTTATATAATAAAGCTCAGGAAGAATTATCT
GCACTAAAACAGGAATCCACTCAAAGCACAGGGCCAAATAATCTCCTTAGTCTTATGGAAAAGGAAAAAG
AACTTGCATTATCTGACTTAAGAAGAATTATGGAAGAAAAGGAAGCTTTAAAAGAAAAATTAAAAAATAT
CCAGGAAATGAATGTTTTTGGCAAAACAGAATTAGAGAAAACTATTGAACATTTGACATGTGTTAATCAT
CAGCTTGAAAGTGAAAAATATGAATTACAATCGAAAGTGTTCCTAATGAAAGAAAAAATAGAGTCATTAG
AGAACAAATCAAAATTTCAAGCTCAACAAATAAGCCAAATTGGCCATGTGCCTGGTGACTCATCTCATCA
GAAAACAGAGATGAATTCCCTTAGGATAGTAAATGACCAGCTACAGCGGTCACTTGATGAGTATCAGCGC
CGACTTTCCTTAAAAAGAGATGAACTTGAATCGGCCCAAGAACAAATTAAAATACTGGAGGGAAAAATAA
ATGAACTAAACCTTAAGATGACTTCACAGAATGAGGCAGCTCATGTAATGAAAAAGACCATTGGTGTTAT
TGATAAAGAAAAAGACTTTCTTCAGGAGACTGTAGACGAGAAGACAGAAAAGCTTGCAGACTTGCAAGAA
AACCTAGAAAATAAAGAGAAAGCTATTGCTCAGATGAAGAAAACTATCTCAGAGTATGAATTATCCAAGA
ACCAGCTAAAGGAAGCATTGACTAATCGAGAAAGGGAGATAAGCAGCCTTCGACGCCAGGTTGATGCATG
TCACAAAGAACTTGATGAAGTGGGAAGAGCTAGAGAAATAGCTTTTAAGGAAAACAGACGATTACAAGAT
GATCTGAGTACAATGGCAAGAGAAAACCAAGCAATCTCATTGGAATTGGAAGCAGCAATGCAAGAAAAGG
AAGAAATGAAGAGTAGAGTGCATAATTATATAACTGAGGTGTCACGATGTGAGAGTTTAATGGCTGCTAA
GGAACAAGAAAATCAAGATTTGTTAGATAGATTTCAGATGCTTCATAGCCGTGCTGAAGACTGGGAAGTC
AAAGCCCATCAAGCAGAAGGAGAAAGCAGCTCAGTTCGACTGGAACTTCTCTCTATTGATACAGAAAGGA

GACACCTTCGAGAAAGAGTCGAGCTACTAGAAAAAGAAATTCAGGAGCACGTAAATGCACATCATGCTTA
TGAATCTCAAATCTCATCAATGGCAAAAGCCATGTCTAGGTTAGAAGACGAGCTGAGACATCAAGAAAAT
GAGAAAGCAGCAGTATTAAGTGACTTGTCTTCTCTTAGGGAACTTTGCATTAAACTTGATTCAGGCAAAG
ATGTTATGACCCAACAATTGAATTCCAAAAGCCTTGACTTGGAGAGGGCTGTGGTAGAATTAGAAAATAT
GAAGTCAGAATCAGAGCTGTTAAAAAAACAACTGTCAAGTGAGAGACACACGATTAAAAACCTTGAATCA
TTGTTGGCTACAAATAGAGATAAAGAATTTCATACTCATTTAACCTCTCACGAGAAGGATACAGAAATTC
AGTTACTTAAGGAGAAGTTAACCCTTTCAGAAAGCAAAGTAACTAGTCAAAGCCGGGAAAATGCCATGCT
CCGATCTAAAGTGACACAATTACAAACAGATTATGATACTATGAAAAGGCAGATTTCAACGGAAAGATAT
GAACGAGAACGAGCAGTCCAAGAGATGCGTCGACATGGTCTTCCTACACCACCCCTTAGTTCTACTCTGA
AGTCTCCTTTACAGTCTCCTGAACATATAAGTGTA
>mouse ENSMUST00000121979.7
ATGACTACAGCTGCAGAGAGAAAGTATATTAACATTCGGAAGAGGTTAGACCAGCTGGGC
TACCGCCAGACCCTGTCAGTGGACAGTCTGCCTTTGGTAGAAAAACTTTTCAGTGACCTC
GTTCATACGACAGAAAGCCTGCGGCAGTGCAGGTTGTCCTCGGGAAAGGCGGAAAAGGAA
AGTGCCAATCTCGATTTTGTTTTGGAACCTTATAAACTTGAAAATACAAGACTGAATAAG
GAGAATAATGAATTGTATCTGGAGTTAATGAAGCTCAGAGAATGCTCAGACAAGCACATT
AAAGACTTGAAGACGACACTGAAGAAATGTTCCCGTGAGACAGCTGATCTGAAGTTTCTG
AACAACCAGTATGTTCACAAGGTCAAAGTCCTGGAGAAAGAGAGCAAAGCCAAGGATGAA
AAGATCCAGCAGCTTCAAGAGAAGAACCTGCGTGCTGTGGTGCAGACCCCTGGTGGCAGG
AAAAGAAACATTGCATTTAGGCGCCAGCGGATGCAAATCGATGAACCAGCCCCACCCTCA
GAAGTCAGCGCATACCCGGTTCCTCAACCAGAAGACCCATACATTGCGGACCTCCTGCAA
GTGGCTGACAACAGGATTCAGGAGCTGCAGGAGGAGGTCCAGCAGCTGCAGGAGAAGCTA
GCACAGATGGAGAAAGGAGTGCTGGACTACAGCAAGCAGATTGAACTGAGAGAACGAGAA
ATACAGCGACTGTCACTTGCGTTGGATGGCGGTTGTTCCCCTGATGTCCTGTCTCTGGAG
ACCAGAAATAAAACCAATGAGAAACTCATTGCTCACTTAAATGTCCAGGTTGACTTCCTC
CAGCAAGCTAATAAAGAGCTGGAGAAGCATATTCAAGAGCTCATGGAAACCAAGGAAACA
GTGACAACTGAAGTTGTGAACCTCAGCAACAGAAACGAGAAGCTCTGCCAGGAGCTAACC
GAGATCGACCAGTTAGCCCAGCGGCTGGAAAGGCACAAGGAGCAAGTGCTGGAGACAGCA
GACAAGGAGCTCGGGGAGGCAAAGAAAGAGATTAAACGAAACCTCTGTGAAATGCGGAAC
CTTGAGGAAAAAATGTCAAAACTGCAGTGGGAATTGGACTTAAGCCATAAGGAGAAGGAG
AGACTGAACAGTGAGCTGCTTTTAAAGTCAGACCTGGAGACTGTTGTTCATCAGCTCGAA
CAAGAAAAGCAAAGACTTAGCAAAAAGCTGCAGAGTTTTGCAGTCACAGAAAGAGAACTG
ACTCTGGAGGTTGAGAGGATGAGGCTAGAACATGGGATAAAGCGTCGGGACAAGTCACCC
TCTCGTCTGGACACATTTCTGAAAGGCATAGAGGAAGAGCGCGATTATTACAAGAAAGAG
CTGGAGAAACTGCAGCATCTAATCCAGCGGAGATCTTGTGCGATTAATTACTCCGCTCGG
GAAAAACCTCCTGTAGTCAAATGCTCAGAAAAGGGTGACTGCAGCACGGACGTTCACCTG
ATCACAAGAGAAAGGGATGAACTTCAGCGCATGCTGGAGAGATTCGAGAAATACATGGAG
GACATACAGTCCAACGTGAAGCTGCTGACGGCGGAAAGGGACAAGCTGAATGTCCTGTAT
AAGGAAGCAAAGGAAGAGTTGTCTACACTAAGGAAGGAGTCCACTAATTCGACATCCCCC
AACCATCTTGTTAGTTGTGTAGAAAAGGAGAAAGAACGCGCGTTATCTGAGTTGAGAAGA
ATTACAGCAGAAAAGGAAGCTCTGAGAGAAAAGTTGAAAAACATCCAAGAAAGGAATGCT
GTTGGAAAATCAGACTTAGAGAAAACTATTGAACATTTGACATATATTAATCACCAGCTT
GAAAATGAAAAATATGAATTGCAATCTAAAATGTTAATGATGAAAGAAACAGTGGAGTCA
TTAGAGAACAAATCAAAACTCCAAGCTCAAAAGCTTAGCCATGTGACTGGTGACTCATCT
CATCAGAAAACAGAGATGACCTCCCTTAGGATCGTCAGTGAGCAGCTACAGCGCTCACTT
GATGACTGTCAGCACCGACTTTCCATAAAAAGAGGTGAACTTGAATCAGCCCAAGAGCAA
ATTAAAATGCTGGAGCAAAAACTAGAGAATCTCAGCCACAGGATGACGGTGCAGAGCGAA

GAGACTCACGCCATGAAGAAGACCATTGGAGTGATGGACAAAGAAAAGGACTTTCTCCAG
GAGACTGTGGATGAGAAGACAGAAAAGATCGCCAACCTGCAGGAAAGCCTCCTTAGTAAA
GAAAAAGTCATTGCCCAGTTGAAGGTCACAGTTGCAGAGTATGAAACATCACTTAACCAG
CTACAAGAAACTTTGACTACTCGAGACCGAGAGATAAACAGCCTCCGGCGCCAGCTTGAT
GCATCTCACAAGGAGCTTGATGATGTTGGGAAATCTAGAGAGATCTCTTTTAAGGAAAAT
AGAAGGTTACAAGATGACCTGGCCACAATGGCGAGAGAAAATCAGGAGATCTCACTGGAG
CTGGAAGCAGCAGTGCAAGAAAAAGAAGAGATGAAGAGCAGAGTCCATAAGTACATCACT
GAGGTGTCGCGGTGGGAGAGCCTGATGGCCGCCAAGGAAAAAGAAAACAAAGACTTGCTG
GATAGATTCCAGATGCTCCACAGCCGTGCTGAGGACTGGGAGGTCAAGGCTCAGCAAGCG
GAAGGGGAGAACAGCTCAGTCCGCCTGGAGCTGCTGTCTATCGACACAGAGAGAAGGCAT
CTGCGGGAACGAGTGGACCTCCTGGAGAAGGAGATCCAGGAGCACATCAATGCACATCAC
GCATACGAGTCTCAGATCTCGTCAATGGCTAAGGCCATGTCTCAGTTAGAAGAGGAGCTG
CGACGTCATGAGAGCGAGAAAGCCACCATGTTAGGTGACGTGTCGTCTCTCCGAGAACTG
TGCATCAAGCTCGACTCAGGCAAAGACGTCATGACCCAGCAGCTGAATTCCAAAAGCCTA
GAGCTGGAGCGGGCGGTAGCAGAATTAGAGAATGTAAAATCAGAATCAGAACTATTAAAA
AAACAGCTGACGAATGAGAGACAGACAATTAAAAACCTTGAGTCGTTGTTAGCTACAAAC
AGAGATAAAGAGTTCCAGTCCCATTTAACCTCCCACGAGAAGGACACAGAAATCCAGCTG
CTGAAGGAGAAGCTAAATCTCTCAGAAAGCAAACTGACTACCCAAAGCCGAGAAACCTCC
ATGCTTCGAACTAAAGTGACACAGTTGCAGACAGATTATGATAATCTGAAGAGGCAGATG
TCAAACGAGAAGTACGAACGAGAGCGAGCAATCCAAGAGATGCGCCGACTTGGTCTTCCT
ACATCACCTCTGAGCTCTACTCTGAAATCTCCTGTACAGACTCCTGATCATATAAATGCA

>rabbit ENSOCUT00000017920.3
ATGACCACAGCAGCAGAGAGGAAGTACATCAACATTAGGAAGAGATTGGATCAGTTGGGC
TACCGCCAGACTCTGACGGTAGAATGTTTACCCTTGGTGGAAAAGCTTTTCAGTGACTTA
GTTCATACAACAGAAAGTCTTCGGCAATCAAAATTATCTACTGTGAAAGCTGAAAAAGAA
AGTGCCAATTTTGATTTTGTTTTGGAACCTTATAAACTAGAAAATGCAAGATTGAGTAGG
GAAAATAATGAATTATACCTAGAGTTAATGAAACTAAGAGAACACTCAGATCAACACATA
AAAGACCTGAAAACTGCTTTGAAGAAATGTGCACGTGAAACAGCTGACCTGAAATTTCTA
AATAACCAGTATGTTCATAAACTCAGGCTTTTGGAGAAAGAGGGCAAAGCTAAAAATGAA
AAAATCCAACAACTTCAAGAAAAGAATTTGCATGCTGTTGTACAAACTCCAGGGGGCAAG
AAGAGAAGTATTGCTTTCAGGCGGCAGCGAATGCAGATTGACGAGCCAGTCCCTCCATCT
GAAGTCAGTTCATACCCAGTTCCTCAGCCGGAGGACCCTTACATTGCAGACCTCCTGCAG
GTGGCTGATAACAGGATTCAAGAACTTCAACAGGAAGTCCAGCAGTTACAAGAAAAGTTA
GCAGTGATGGAGAGTGGAGTGAGAGACTATAGCAAGCAGATTGAGCTAAGAGAACGAGAG
ATAGAACGATTGTCAATGGCTTTGGACGGGGGTCGTTCCCCTGATGTCCTGACGCTGGAG
AACAGAAATAAAACCAATGAAAAGCTCATCGCTCATTTAAATATCCAGGTTGACTTTCTT
CAGCAAGCTAATAAGGACCTGGAGAAGCACATCCGAGAGCTTATGGAAACCAAGGAAACA
GTGACAACTGAGGTTGTTAACTTAAGTAACAAGAATGAGAAACTCTGCCGAGAGCTAACT
GAAATAGACCAGTTAGCACAGCAGTTGGAGCGACATAAAGAAGAAGTGCTTGAGACCGCT
GATAAAGAACTCGGCGAAGCAAAGCAAGAGATTCAAAGAAATCTCTCTGAAATGCGGACT
CTTGAAGAAACAATGGCAAAACTTCAACTGGAATTAAACTTATGCCATAAAGAAAAGGAG
AGACTGAGTGATGAACTGCTTCTTAAGTCAGACCTGGAAACTGTCATCCACCAACTTGAA
CAAGAGAAACAGAGACTCAGCAAAAAAGTTGAGAGTTTTGCAGTTACAGAAAGAGAACTT
ACTTTGGAAGTTGAGAGAATGAGGCTAGAACATGGAATAAAACGCAGAGACAGATCACCT
TCTCGTTTAGATACATTTCTCAAAGGTATAGAAGAAGAACGAGATTATTATAAGAAAGAA
CTAGAAAGACTCCAGCATTTAATACAGCGAAGATCTTGTTCTACAAATTACTGTGCTCGT

GAAAAAATCCCAATATTTAAAACACAAGAAAAAGGTGATTACAATTCAGAAATTCATCTG
GTCACAAGAGAAAGAGATGAACTTCAGCGTATGCTGGAAAGATTTGAAAAATATATGGAG
GATATACAGTCCAATGTTAAATTATTGACGGCGGAAAGAGATAAACTCAGTGTCTTATAT
AATGAAGCTCAGGAAGAATTGTCTGCGCTTAGACGAGACTCCACCCAAACCACAGTCTCC
AACAATATTGTTAGTCTAATGGAAAAGGAAAAAGAACTTGCGTTATCTGACCTAAGAAGA
GTTATGGCAGAAAAGGAGACTTTGAGAGAAAAATTAAATAATATCCAGGAAATGAATCTT
TTTGGAAAATCAGAATTAGAGAAAACTATTGATCATTTGACACGTGTTAATCATCAGCTT
GAAGATGAAAAATATGAATTAAAATCTGAAGTGTTACTAATGAAAGAAAAAATAGACTCA
TTAGAGAACAAATCAAAACTCCAAGCCCAAAAGCTTTGCCATGTGGCCGGTGACTCATCT
CATCAGAAGTCAGAGATGAACTCACTTAGGATAGTAAATGAGCAGCTAGAGCGGTCACTT
GATGACTATCAGCACCGACTCTCCGTGAAAAGAGGTGAACTTGATTCAGCCCAAGCACAA
ATTAAAATACTGGAGGAGCAAATAGATAAATTAAACCTTAAGATGACTTCACAGGATGAG
GAGGTCCATGTAATGAAAAAGACCATTGGTGTCATTGATAAAGAAAAGGACTTTCTTCAG
GAGACTGTAGACGAGAAGACAGAAAAGATTGCAAACTTGCAAGAAAACCTCGCTAGTAAA
GAAAAAACTATTGCTCAGATGAAGATAACTGTCTCAGAGTTTGATAAATCTATGAACCAG
CTAAAGGAAACCCTGAGTAATCGAGACCGGGAGGTGAGCAGCCTCCGGCGCCAGCTGGAT
GCGGCTCACAAGGAACTCGATGAAGTTGGAAGATCTAGAGACATGGCTTTTAAGGAGAAC
AGAAGATTACAAGATGATCTGGCTACAATGGCAAGAGAAAACCAGGAAATCTCACTGGAA
TTAGAAGCATCAGTACAAGAAAAAGAAGAAATGAAAAATCGAGTTCATAGTTATATAACT
GAAGTATCACGATGGGAGACCTTAATGGCTTCTAAGGAAAAGGAAAATCAAGATTTGTTA
GACAAATTCCAGATGCTTCACAACCGTGCTGAAGACTGGGAAGTCAAAGCCCACCAGGCT
GAGGGGGTGAGCAGTTCAGTCCGGCTGGAGCTTCTTTCTATTGACACAGAGAGGAGACAC
CTTCGAGAAAGAGTGGAACTTCTAGAAAAAGAAATCCAGGAGCACATAAATGCACATCAT
GCATATGAATCCCAAATCTCATCCATGGCAAAGGCCATGTCTAGATTAGAAGAAGAACTG
AGACTTCGGGAAAACGAGAAAGCAGCAGCGTTGAGTGATTTGTCGTCTCTGAGAGAGCTT
TGCATTAAGTTAGATTCAGGCAAAGATGTTACGACCCAACAATTGAATTCCAAAAACCTT
GAGTTTGAGAGGGCTGTGGCAGAATTAGAAAATGTAAAATCAGAGTCTGAGATGTTAAAA
AAACAGCTGTTAAATGAAAGACACACAATTAAAAACCTTGAAGCATTGTTGGCTTCGAAC
AGAGATAAGGAATTTCACTCTCATTTAACCTGTCACGAGAAGGATACCGAGATTCAGCTT
CTCAAAGAGAAGTTGACCCTTTCGGAGAGCAAATTAACTAGTCAAAGCCGAGAGAACACC
ATGCTTCGAGCTAAGGTGGCAGAATTGCAGACAGATCACGACGCTCTGAAGAGGCAGATT
GCAGCGGAAAAATACGAACGAGAACGAGCGATCCAAGAGATGCGTCGACACGGGCTTCCT
ACTCCACCCCTTAGTTCTACTCTGAGGTCTCCTTCACCGACTCCTGAACAGATCAGTGTA

>rat ENSRNOT00000032156.5
ATGACTACAGCTGCAGAGAGAAAGTATATTAACATTAGGAAAAGGTTAGACCAGCTGGGC
TACCGCCAGACCCTGTCAGTGGACAGTCTGCCTTTGGTAGAAAAACTTTTCAGTGATTTG
GTTCACACAACAGAAAGCCTGCGGCAATGCAGGCTGTCCTCGGTAAAAGCAGAAAAGGAC
AGTGCCAATTTCGATTTTGTTCTGGAACCTTATAAACTTGAAAACACAAGACTGAATAAG
GAGAATAATGAATTGTATCTGGAGTTAATGAAGCTGAGAGAATACTCAGACAAGCACATT
AAAGACTTGAAAATCACACTGAAGAAATCCTCACGAGAGACAGCTGATCTGAAGTTTCTG
AATAACCAGTATGTCCACAAGGTCAGACTCCTGGAGAAAGAGAGCAAAGCTAAGGACGAA
AAAATCCAGCAGCTTCAAGAAAAAAATCTGCGTGCGGTAGTACAGACTCCTGGGGGCAGG
AAAAGAAACATTGCGTTTAGGCGCCAGCGGATGCAAATCGATGAGCCAGCCCCACCCTCG
GAAGTCAGCTCCTACCCAGTTCCTCAACCAGAAGACCCGTACATCGCGGACCTCCTGCAA
GTGGCTGACAATAGGATTCAGGAGCTGCAGGAGGAAGTGCACCAGCTGCAAGAGAAACTA
GCGCAGATGGAGAAAGGAGTGCAGGACTACAGCAGGCAGATCGAACTAAGAGAACGAGAA

ATAGAGCGACTGTCCGTTGCTGTGGATGGCGGCCGTTCCCCTGACATCCTGTCTCTGGAG
ACTAGAAATAAAACCAACGAGAAGCTTATTGCTCAGTTAAATATCCAGGTTGACTTCCTT
CAACAAGCTAATAAGGAACTGGAGAGGCGTATCCAAGAGCTCATGGAGACCAAGGCGACA
GTGACAACTGAGGTTGTGAACCTAAGCAACAGAAACGAAAAGCTCTGCCAGGAACTAACA
GAAATCGACCAGATGGCCCAGCAGCTGGAAAGGCACAAGGAACAAGTGCTGGAGACGGCA
GACAGGGAGCTCGGGGAGGCAAAGAAAGAGATTAAAAGAAACCTCTCTGAAATGCGGAAC
CTTGAGGAAAAAAATGTCAAAACTGCAATGGGAATTAGATGTAAGCAATAAGGAGAAGGAG
AGACTGAATGGTGAACTACTTTTAAAGTCAGACCTGGAGACGGTTGTTCATCAGCTTGAA
CAAGAAAAGCAAAGACTTAACAAAAAACTGCAGAGTTTTGCAGTCACAGAAAGAGAACTG
ACTCTGGAGGTTGAGAGGATGAGGCTAGAACACGGGATAAAACGTCGAGACAAGTCGCCC
TCCCGTCTAGACACATTTCTGAAAGGCATAGAGGACGAGCGCGATTATTACAAGAAAGAG
CTGGAGAAACTGCAGCATCTCATCCAGCGAAGATCATGCTCTGTTATTTACTGTGCTCGG
GAGAAACCTCCTATAATCAAATGCTCAGAAAAGGGTGACTGCAATTCCGACATTCATCTG
ATCACAAGAGAAAGGGATGAGCTTCAGCGCATGCTAGAAAGATTTGAGAAATACATGGAG
GATATACAGTCCAACGTGAAACTGCTGACGGCAGAAAGGGACAGACTAAGTGTCCTGTAT
AAGGAAGCAAAGGAAGAGTTATCTGCACTAAGACAGGAGTCCACTGGTTCACTGGCCCCC
AACAATCTTGTAAGTTGTATAGAAAAGGAGAAAGAACGCGCATTATCTGACTTAAGAAGA
ATTACAGCAGAAAAGGAAGCTCTGAGAGAAAAGTTGAAAAATATCCAAGAACTGAATGTT
GTTGGAAAATCAGAATTAGAGAAGACTATTGAACATTTGACGTATATTAATCACCAGCTT
GAAAATGAAAAATATGAATTACAATCTAAAATATTAATAATGAAAGAAACAATAGAATCA
TTAGAGAGCAAATCAAAACTCCAAGCTCAAAAACTTAGCCATGTGACTGGTGACTCATCT
CATCAGAAAACAGAGATGAACTCGCTCAGGATAGTGAGTGAGCAGCTACAGCGGTCACTT
GATGACTGTCAGCACCGACTTTCCATAAAAAGAGGTGAACTCGAATCAGCCCAAGAACAA
ATTAAAGCGCTGGAGCAGAAACTAGAGTCTCTGAGCCACAGGATGACAATGCAAAGCGAA
GAAACTCATGCGATGAAGAAGACCATTGGCGTGATGGACAAAGAAAAGGACTTTCTCCAG
GAGACTGTGGATGAGAAGACGGAAAAGATCGCCAGCCTGCAAGACAGCCTCATTAGTAAA
GAAAAAGCTATTACCCAGTTGAAGGTCACAGTCTCGGAGTGTGAATCGTCACTGAACCAA
CTACAAGAAACTTTGACTAATCGAGACCGAGAGATAAACAGCCTCCGTCGCCAGCTTGAT
GCAACTCACAAGGAACTTGACGATGTTGGGAAATCTAGAGAGATCTCTTATAAGGAAAAT
AGAAGGTTACAAGATGACCTGGCCACAATGGCGAGAGAAAACCAGGAGATCTCACTGGAA
CTGGAAGCAGCAGTGCAAGAGAAAGAAGAGATGAAGAGCAGGGTTCACAAGTATATCACT
GAGGTGTCACGATGGGAGAGCCTAATGGCCGCGAAGGAAAAAGAAAACAAAGACTTGCTA
GATAGATTCCAGATGCTTCACAACCGTGCTGAGGACTGGGAGATCAAAGCTCAGCAAGCG
GAGGGGGAGAACAGCTCAGTCCGCCTAGAGCTGCTGTCTATTGATACAGAGAGAAGGCAC
CTGCGAGAGCGGGTGGAGCTCCTGGAGAAGGAGATCCAGGAGCACATAAATGCACATCAC
GCTTATGAGTCTCAGATCTCATCAATGGCTAAAGCCATGTCTCAGTTAGAAGAAGAGCTT
CGACGACATGAGAGTGAGAAAGCCACCGTGTTAGGTGATGTGTCCTCTCTCCGAGAACTC
TGCATTAAGCTCGACTCAGGCAAAGACATTATGACCCACCAGCTGAATTCCAAAGGCCTT
GAGCTGGAGCGGGCAGTAGCAGAGTTGGAGAATGTAAAATCAGAATCGGAGCTGTTAAAA
AAGCAGCTGATGACTGAGAGGCAGACAATTAACAGCCTTGAGTCGTTGTTAGCTACAAAC
AGAGATAAAGAATTCCAGTCCCACCTAACCTCCCACGAGAAGGACACAGAAATCCAGCTG
CTGAAGGAGAAGTTAAACCTCTCAGAAAGCAAACTAACTACCCAAAGCCGAGAAACCTCC
ATGCTTCGAACTAAAGTGACACAGTTGCAGACAGATTATGATAATCTGAAAAGGCTGATG
TCAAATGAGAAGTACGAACGAGAGCGAGCAATCCAAGAGCTGCGCCGACTCGGTCTTCCC
ACGTCGCCTCTGAGCTCCACTCTGAGATCGCCCATGCAGTCTCCTGAGCATATAAATGCA

>Chinchilla lanigera XM_013518557.1

ATGACGACAGCTGCAGAGAGGAAGTATACTAATAT
TAGGAAGAGATTGGATCAGCTGGGGTACCGGCAGACTCTGACAGTCGAGTGTTTACCTTTGGTAGAAAAG
CTCTTCAGTGACTTGGTTCATACAACAGAAAACCTTCGGCAATCAAAATTATCTTCTGTGAAAGCTGAAA
AGGAAAGTGCCAATTTTGATTTTGCTTTGGAACCCTATAAATTTGAAAATGCGAGATTAAGTAAAGAAAA
TAATGAATTATACCTGGAGTTAATGAAACTGAGAGAGTGTTCAGAGCAGAATATTAAAGAGTTGAAAACT
ACATTGAAGAAGTGTGCACGTGAAACAGCAGATCTGAAATTTCTAAATAACCAGTATGTTCACAAACTCA
AACTTTTGGAGAAAGAGAGCAAAGCTAAAAATGAAAAAATTCAGCAACTTCAAGAAAAGAATTTGCATGC
TGTGGTACAAACTCCAGGTGGCAAGAAAAGGAGCATTGCATTCAGACGCCAGCGAATGCAGATTGACGAG
CCGGTTCCTCCCTCTGAAGTCAGCTCTTACCCAGTTCCGCAGCCCGAGGACCCTTACATTGCAGACCTCC
TGCAAGTGGCCGATAACAGGATCCAAGAACTTCAGCAGGAAGTCCACCAGCTACAAGAAAAGTTAACAGT
GATGGAAAGCGGGGTGAGAGATTACAGCAAGCAGATTGAGCTGCGAGAGCGAGAGATCGAGCGACTGTCA
GTTGCTTTGGATGGTGGTCGCTCCCCAGATGTCTTGTCTCTGGAGACTAAAAATAAAACCAACGAAAAGC
TGATTGCTCACTTAAATGTGCAGGTTGACTTTCTTCAGCAAGCTAATAAAGACCTGGAGAAGCACATTCA
AGAGCTTATGGAAACCAAGGAGACAGTGGCAACTGAAGTGGTGAATTTAAGTAACAAAAACGAAAAGCTC
TGCCAAGAACTAACTGAAATAGACCACTTAGCGCAGCAACTGGAACGACATAAAGAAGAAGTGCTTCAGA
CGGCTGATAAAGAACTTGGGGAAGCAAAGAAAGAGATTAAAAGAAACCTCTGTGAAATGCGGAATCTTGA
AGAAACAATGGCAAAACTTCAACTGGAATTAGACTTATGCCATAAAGAAAAAGAGAGACTGAGTGATGAA
CTCCTTCTAAAATCAGACCTGGAAACTGTTGTTCATCAGCTTGAACAAGAAAAGCAAAGACTTACCAAAA
AAATTGAAAGTTTTGCAGTGACAGAGAGAGAACTTACTTTGGAAGTTGAGAGGATGAGGCTAGAACATGG
AATAAAACGTCGAGACAAGTTACCTTCTCGTTTAGATACATTTCTCAAAGGTATAGAAGAAGAACGAGAT
TATTATAAGAAAGAGCTGGAGAGACTCCAACATATCATACAGAAAAGGTCTTACTCTGTAAATTACTGTG
CACGTGAAAAAAATTCAGTATTTAAAACACCAGAAAAGGGGGATTACAATTCAGAAATTCATCTGGTCAC
AAGAGAAAGAGATGAACTTCAGCGTATGCTAGAAAGATTTGAGAAGTACATGGAAGATATACAGTGCAAT
GTTAAATTATTGACAGCAGAAAGAGATAAACTAAGTGTCTTATATAATGAAGCTCAGGAAGAATTATGTG
CGCTACGACAGGCACCCGCGCACCCCACCGTCCCCAGTAGTCTGGTTAGCCTTATGGAAAAGGAGAAGGA
GCTTGCCTTGTCGGACTTAAGAAGAGTTATGACAGAAAAGGAAGATTTAAGAGAAAAGTTAAAAAATATC
GAGGAAATGAGTGCTTTTAGCAAATCAGAATTAGAGAAAACTATTGAACATTTGACATGTGTTACTCATC
AGCTTGAAAGTGAAAAATACGAATTACAATCTAAAGTGTTAATAATGAAAGAAACGATAGAGTCACTAGA
GAACAAATCCAAACTCCAAGCTCAAAAGCTTAGCCATGTGGCTGGTGACTCATCCCATCAGAAAACAGAG
GTGAACTCCCTTAGGATCGTAAATGAGCAGCTACAGCGGTCACTTGAGGACCATCAGCACCAACTGTCCC
TAAAAAGAAGTGAACTTGAATCTGCCCAGGAACAAATTAAAATGCTGGAGAAAAAAATAGATGAGCTAAA
CGTTAAGATGACCTCACAGAGTGAAGAGGCTCATGTGATGAAGAAGACTATTGGTGTTATTGACAAGGAG
AAAGACTTCCTGCAGGAGACAGTGGATGATAAGACAGAAAAGATCGCAAACTTGCAGGAGGTCCTGGCTG
CTAAAGAAAAAGCTGTTGCTCAGATGAAGATAACTATCTCGGAGTATGAATTATCTCTCAACCAACTAAA
GGAAACACTGAGTAATCGAGACCGGGAAATCAGCAGCCTCCGGCGTCAGCTTGATGGAACTCTCAAAGAA
CTTGATGAAGTAGGAAAGTCTAGGGAAATATCTTTCAAGGAAAACAGAAGATTACAAGATGATCTGGCTA
CAGTGGCAAGAGAAAATCAGGAAATCTCATTGGAATTGGAGGCTGCAGTGCACGAAAAGGAAGAAATGAA
GAGTAGAGTTCATAATTACATAACTGAAGTTTCCCGATGGGAGAGCTTAATGGCTGCTAAGGAGCAAGAA
AATCAAGATTTGTTAGATAGATTTCAGATGCTTCATAACTGCGCTGAAGACTGGGAGATCAAAGCTCATC
AAGCTGAAGGAGAAAGCAGCTCAGTTAGACTGGAACTTCTGTCTATTGACACAGAGAGAAGACACCTTCG
AGAAAGAGTGGACCTATTAGAAAAAGAAATTCAGGAGCACATAAATGCACACCATGCTTATGAATCTCAG
ATCTCATCAATGGCAAAAGGCATGGCTAGATTAGAAGAAGAGCTGAGACGTCAAGAAGATGAGAAAGTGA
CAGTGTTAAATGATATGTCTTCTCTTAGAGAACTCTGCATTAAGCTTGATTCAGGCAAAGATGTTATGAC
CCAACAATTGAATTCTAAAACTCTTGAGTTGGAGAGGGCTCTGGTAGAACTAGAAAATGTAAAATCAGAA
TCAGAACTATTAAAAAAACAACTGTCAAGTGAGAAATACACGATTAAAAACCTTGAATCATTGTTGGCTA
CAAATAGAGATAAAGAATTTCATTCTCATTTAACATCCCACGAGAAGGATACAGAAATCCAGCTACTTAA
GGACAAGTTAACCCTATCAGAAAGCAAACTAACTAGTCAAAGCAGGGAAAACACCATACTTCGAACTAAA

GTGGGACAGTTACAAACAGATTATGATACTCTAAAAAGGCAAATTTCAACAGAGAGATATGAACGAGAAC
GAGCAATCCAAGAGATGCGTCGGCATGGTCTTCCTACGTCACCCCTTAGCTCTACTCTGAAGTCTCCTTC
GCATTCTCCAGAACGTAAAGAA
>Fukomys damarensis XM_010640282.1
ATGACAACAGCTGCAGAGAGGAAGTATATTAATATTAGAAAAAGATTGGATCAGCTGGGATACCGCCAGA
CCCTGACAGTCGAGTGTTTACCTTTGGTAGAAAAGCTGTTCAGTGACTTGGTTCATACAACAGAAAGCCT
TCGCCAGTCAAAGTTATCTTCTGTGAAAGCTGAAAAGGAAAGTGCCAATTTTGATTTTGTTTTGGAGCCC
TATAAACTTGAAAATGCAAGACTGAGTAAAGAAAATAATGAATTATACCTGGAGTTAATGAAACTGAGAG
AATGTTCAGAACAAAATATTAAAGAGTTGAAAACTACATTGAAGAAATGTACACGTGAAACAGCAGATCT
GAAGTTTCTAAATAACCAATATATTCACAAACTCAAACTTTTGGAGAAGGAGAGTAAAGCTAAAAATGAA
AAAATTCAACAACTTCAAGAAAAGAATTTGCATGCTGTAGTACAAACTCCAGGTGGCAAGAAAAGAAGCA
TTGCATTCAGACGCCAGCGTATGCAAATTGACGAGCCAGCTCCTCCCTCTGAAGTCAGTTCCTATCCAGT
TCCACAGCCAGATGACCCTTACATCGCAGACCTCCTGCAAGTGGCTGATAACAGGATCCAAGAACTTCAG
CAGGAAGTCCACCAGCTACAAGAAAAGTTAGCAGTGATGGAAAATGGTGTGAGAGATTACAGCCAGCAGA
TTGAGCTACGAGAACGAGAGATAGAACGACTGTCAGTTGCTTTGGATGGTGGGCGCCCGCCGGATGTCCT
GTCTCTGGAGACTAGAAATAAAACCAATGAAAAGCTGATTGCTCATTTAAATGTGCAGGTTGACTTTCTT
CAGCAAGCTAATAAAGACCTGGAGAAGCACATTCAAGAGCTTATGGAAACCAAGGAAACGGTGGCAACTG
AAGTCGTTAATTTAAGTAACAAAAATGAAAAACTCTGCCAAGAATTAACTGAAATAGACCACTTAGCACA
GCAACTAGAAAGGCATAAAGAAGAAGTGCTTGAGACTGCTGATAAAGAACTTGGAGAAGCAAAGAAAGAG
ATCAAAAGAAACCTCTGTGAAATGCGGAATCTTGAAGAAACAATGACAAAACTTCAACTGGAATTAGACT
TATGCCATAAAGAAAAGGAGAGACTGAGCGATGAACTTCTTCTAAAATCAGACCTGGAAACTGTTGTTCA
TCAGCTTGAACAAGAAAAGCAAAGACTTAGCAAAAAAATTGAAAGTTTTGCAGTTACAGAAAGAGAACTT
ACTTTGGAAGTTGAGAGGATGAGGCTAGAACATGGAATAAAACGTCGAGACAAGTTACCTTCTCGTTTAG
ATACATTTCTCAAAGGTATAGAAGAAGAACGAGATTACTATAAGAAAGAGCTAGAAAAGCTCCAACATAT
AATACAGAGAAGATCTTGCTCTATAAATTACTGTGCACATGAAAGAAATCAAGTATTTAAAACATCGGAA
AAGGGTGATTACAACTCAGAAATTCATCTGGTCACAAGAGAAAGAGATGAACTTCAGCATATGCTAGAAA
GATTTGAAAAATATATGGAAGATATACAGTGCAATGTTAAATTATTGACAGCAGAAAGAGATAAACTAAG
TGTCTTATATAATGAAGCTCAGGAAGAATTATGTGCACTAAGACAGGAGTCAACCCACACCACTGTCCCC
AGTAGTCTTGTTAATCTTATGGAAAAGGAAAAGGAACTTGCCTTATCTGACTTAAGAAGAGTTATGGCAG
AAAAGGAAGATTTAAGGGAAAAGTTAAAAAATATCCAGGAAATGAGTGCTTTGGGCCAATCAGAATTAGA
GAAAACTATTGAACATTTGACATGTGTTAATCATCAGCTTGAAAGTGAAAAATACGAATTACAATCTAAA
GTGTTAATAATGAAAGAAACAATAGAGTCACTAGAGAACAAATCAAAACTCCAAGCTCAAAAGCTAAGCC
ATGTGGCTGGTGACTCATCCCATCAAAAAACAGAGATGAACTCCCTTAGGGTAGTAAATGAGCAGCTACA
GCGATCTCTCGATGATTACCAGCATCGACTGTCCATAAAAAGAGGTGAACTTGAATCTGCCCAGGAACAA
ATTAAAATACTGGAGGAAAAAATAGATGAGCTAAACCTTAAGATGACTTCACAGAATGAAGAGGCTCATG
CAATGAAAAAGACTATTGGTGTTATTGATAAAGAGAAAGACTTCCTCCAGGAGACTGTGGATGAGAAGAC
AGAAAAGATTGCAAACTTGCAAGAAATCCTAGCTAGTAAAGAAAAAGCTATTGCGCAGATGAAGATAACT
GTCTCAGAGTATGAATCATCTATGACGCAACTAAAGGAAACACTTAGTAATCGAGACCGGGAGATAAGCA
GCCTCCGGCGTCAGCTTGATGCAACTCTCAAAGAGCTTGATGAAGTAGGAAAGTCTAGAGAAATATCTTT
CAAGGAAAATAGAAGATTACAAGATGATCTGGCTACAATGGCAAGAGAAAATCAGGAAATCTCATTGGAA
TTGGAGGCAGCAGTCCAAGAAAAAGAAGAAATGAAAAGTAGAGTTCATAATTACATAACTGAGGTGTCCC
GATGGGAGAGCTTAATGGCTGCTAAGGAGCAAGAAAATCAAGATTTGTTAGACAGATTCCAGATGCTTCA
TAACTGTGCTGAAGACTGGGAGATCAAAGCCCATCAAGCTGAAGGAGAAAGCAGCTCAGTCCGACTGGAA
CTTCTTTCTATTGACACAGAGAGAAGACACCTTCGAGAAAGAGTGGATCTACTAGAAAAAGAAATTCAGG
AGCACATAAATGCACATCATGCTTATGAATCTCAGATCTCATCAATGGCAAAAGCTATGGCTAGTTTAGA
AGAAGAGCTGAGACATCAAGAAGAGGAGAAAGCAACAGTATTAAATGATTTGTCTTCTCTTAGAGAACTT
TGCATTAAGTTTGATTCACGCAAAGATGTTATGACCCAACAGTTGAATTCTAAAAACCTTGAGTTGGAGA

GGGCACTTGTAGAATTAGAAAATGTAAAGTCAGAATCAGAACTATTAAAAAAACAACTGTCAAATGAGAG
ATACACGATTAAAAACCTTGAATCACTGTTGGCTACAAATAGAGATAAAGAATTTCATTCTCATTTAAAC
TCCCATGAGAAGGATACAGAAATTCAGCTACTTAAGGAGAAGTTAACCCTTTCAGAAAGCAAATTAACTA
GTCAAAGCAGGGAAAACACCATGCTTCGAGCTAAAGTGGGACAGTTACAAACAGATTATGATTCTCTAAA
AAGGCAAATTTCAACGGAGAGATATGAACGAGAACGAGCGATCCAAGAGATGCGTCGACATGGTCTTCCT
ACATCTCCCCTTAGTTCTACACTGAAGTCTCCTCCACATTCTCCAGAACATAAACAA
>Marmota marmota marmota XM_015482127.1
ATGACTACAACTGCAGAAAGAAAGTATAT
TAATATTAGAAAAAGATTGGATCAGTTGGGATACCGCCAGACTCTGACATTGGAGTGTTTACCATTGGTG
GAAAAACTTTTCAGTGACTTAGTTCATACAACAGAAAGCCTTCGACAATCAAAATTATCTACTGTGAAAG
CTGAAAAGGAAAGTGCCAATTTTGATTTTGTATTGGAACCCTATAAACTTGAAAATGCAAGATTGAGTAG
GGAAAATAATGAATTATACCTGGAATTAATGAAACAGAGAGAGTACTCAGACCAACACATTAAAGAGTTG
AAAACTACTTTGAAAAAGTGTGCACGTGAAACCGCTGATCTGAAATTTCTAAATAACCAATATGTTCATA
AACTCAGACTTTTGGAGAAAGAAAGCAAAGCTAAGAATGAAAAAATTCAGCAACTTCAAGAAAAGAATTT
GCATGCTGTAGTACAAACTCCGGGTGGCAAGAAAAGAAGCATTGCTTTCAGGCGCCAGCGTATGCAAATA
GATGAACCAGTTCCTCCTTCTGAAGTTAGTGGTTATCCAGTTCCACAGCCAGATGACCCTTACATTGCAG
ACCTCCTGGAAGTGGCTGATAATAGGATTCAAGAGCTTCAGCAGGAAGTCCACCATCTACAAGAAAAGTT
AGCAATGATGGAAAATGGCATAAAAGATTACAGCAAGCAGATTGAGCTAAGAGAACGAGAAATAGAAAGA
CTGTCAGGTACTTTGGATGGTGGTCGCTCCCCTGATGTCCTGTCTCTGGAGAATAGAAATAAAACCAATG
AAAAGCTTATTGCTCATTTAAATATTCAGGTTGACTTTCTTCAGCAAGCTAATAAAGACCTGGAGAAGCA
TATTCAAGAGCTTATGAAAACCAAGGAAACAGTGACGACTGAAGTTGTTAATTTAAGTAACAAAAATGAA
AAACTCTGCCAAGAATTAACTGAAATAGACCAGTTAGCACAGCAGTTGGAAAGACATAAAGAAGAAGTAC
TTGAGACTGCTGATAAAGAACTTGGGGAAGCAAAGAAAGAGATTAAAAGAAACCTCTCTGAAATGCGGAA
TCTTGAGGAAACAATGGCAAAACTTCAACTGGAATTAAGTTTATGCCATAAAGAAAAGGAGAGACTAAAT
GATGAACTTCTTCTGAAATCAGACCTAGAAACTGTTGTACATCAGCTTGAACAAGAAAAGCAAAGACTTA
GCAAAAAAATGGAAGGTTTTGCAGCTATAGAAAAAGAACTTACTTTGGAAGTTGAAAGGATGAGGCTAGA
ACATGGAATAAAACGTCGAGACAGGTCACCTTCTCGTTTAGATACATTTCTGAAAGGTATAGAAGAAGAA
CGAGATTATTATAAGAAAGAACTAGAAAGACTCCAACATATAATACAACGAAGATCTTGCTCTACAAATC
ATTGTGCACGTGAAAAACATTCAATATTTAAAACACTAGAAAAGGGTGATTATAATTCAGATATTCATCT
GATTGCAAGAGAAAGAGATGAACTTCAGCGTATGCTAGAAAGATTTGAAAAATATATGGAAGATATACAG
TCCAATGTTAAATTATTGACAGCAGAAAGAGATAAACTAAGTGTCTTATATAATAAAGCTCAAGAAGAAT
TATCTGCACTAAAACAGGAATCCACTCAAAGCACAGGGCCAAATAATCTCCTTAGTCTTATGGAAAAGGA
AAAAGAACTTGCATTATCTGACTTAAGAAGAATTATGGAAGAAAAGGAAGCTTTAAAAGAAAAATTAAAA
AATATCCAGGACATGAATGTTTTTGGCAAAACAGAATTAGAGAAAACTATTGAACATTTGACATGTGTTA
ATCATCAGCTTGAAAGTGAAAAATGTGAATTACAATCGAAAGTGTTCCTAATGAAAGAAAAAATAGAGTC
ATTAGAGAACAAATCAAAGTTTCAAGCTCAACAAATAAGCCAAATTGGCCATGTGCCTGGTGACTCATCT
CATCAGAAAACAGAGATGAATTCCCTTAGGATAGTAAATGACCAGCTACAGCGGTCACTTGATGAGTATC
AGCGCCGACTTTCCTTAAAAAGAGGTGAACTTGAATCAGCCCAAGAACAAATTAAAATACTGGAGGGAAA
AATAAATGAACTAAACCTTAAGATGACTTCACAGAATGAGGCAGCTCATGTAATGAAAAAGACCATTGGT
GTTATTGATAAAGAAAAAGACTTTCTTCAGGAGACTGTAGACGAGAAGACAGAAAAGCTTGCAGACTTGC
AAGAAAACCTAGAAAATAAAGAGAAAGCTATTGCTCAGATGAAGAAAACTATCTCAGAGTATGAATTATC
CAAGAACCAGCTAAAGGAAGCATTGACTAATCGAGAAAGGGAGATAAGCAGCCTTCGACGCCAGGTTGAT
GCATGTCACAAAGAACTTGACGAAGTGGGAAGAGCTAGAGAAATAGCTTTTAAGGAAAACAGACGATTGC
AAGATGATCTGAGTACAATGGCAAGAGAAAACCAAGCAATCTCATTGGAATTGGAAGCAGCAATGCAAGA
AAAGGAAGAAATGAAGAGTAGAGTGCATAATTATATAACTGAGGTGTCACGATGTGAGAGTTTAATGGCT
GCTAAGGAACAAGAAAATCAAGATTTGTTAGATAGATTTCAGATGCTTCATAGCCGTGCTGAAGACTGGG
AAGTCAAAGCCCATCAAGCAGAAGGAGAAAGCAGCTCAGTTCGACTAGAACTTCTCTCTATTGATACAGA

AAGGAGACACCTTCGAGAAAGAGTCGAGCTACTAGAAAAAGAAATTCAGGAGCACGTAAATGCACATCAT
GCTTATGAATCTCAAATCTCATCAATGGCAAAAGCCATGTCTAGATTAGAAGACGAGCTGAGACATCAAG
AAAATGAGAAAGCAGCAGTATTAAGTGACTTGTCTTCTCTTAGGGAACTTTGCATTAAACTTGATTCAGG
CAAAGATGTTATGACCCAACAGTTGAATTCCAAAAGCCTTGACTTGGAGAGGGCTGTGGTAGAATTAGAA
AATATGAAGTCAGAATCAGAGCTGTTAAAAAAACAACTGTCAAGTGAGAGACACACGATTAAAAACCTTG
AATCATTGTTGGCTACAAATAGAGATAAAGAATTTCATACTCGTTTAACCTCCCACGAGAAGGATACAGA
AATTCAGCTACTTAAGGAGAAGTTAACCCTTTCAGAAAGCAAAGTAACTAGTCAAAGCCGGGAAAATGCC
ATGCTCCGATCTAAAGTGACACAATTACAAACAGATTATGATACTATGAAAAGGCAGATTTCAACTGAAA
GATATGAACGAGAACGAGCAGTCCAAGAGATGCGTCGACATGGTCTTCCTACACCACCCCTTAGTTCTAC
TCTGAAGTCTCCTTTACAGTCTCCTGAACATATAAGTGTA
>Odobenus rosmarus divergens XM_012568011.1
ATGACTGCAGCTGCAGAAAGAAAGTACATTAATATTAGAAAAAGGTTGGATCAGTTGGGATACCG
CCAGACTCTGACAGTGGAGTGTTTACCATTGGTAGAAAAACTTTTCAGTGACCTAGTTCATACAACAGAA
AGTCTTCGGAAATCAAAATTATCTACTGTGAAAGCTGAAAAAGAAAGTGCCAATTTTGATTTTGTTTTGG
AACCTTATAAACTTGAAAATGCAAGATTGATCAAGGAAAATAATGAATTATACCTGGAATTAATGAAACT
GAGAGAACAATCAGGTCAACACATTAAAGAGTTGAAAACTACGTTGAAGAAGTGTGCACGTGAAACAACT
GATCTGAAATTTCTAAATAACCAATATGTTCATAAACTCAAACTTTTGGAGAAAGAGAGCAAAGCTAAGA
ATGAAAGAATTCAACAACTTCAAGAAAAGAATTTGCATGCTGTAGTACAAACTCCAGGTGGCAAAAAAAG
AAGTATTGCTTTCAGGCGCCAGCGTATGCAGATTGATGAACCAGTCCCTCCCTCCGAAGTCAGTTCATAT
CCAGTTCCGCAGCCTGATGACCCTTACATTGCAGACCTCCTGCAGGTGGCTGATAACAGAATTCAAGAAC
TGCAACTGGAAGTCCACCAGTTACAAGAAAAGTTAGCAATGATGGAAAGTGGACTGAGAGATTATACGGA
GCAGATTGAGCTAAGAGAACGAGAGATAGAACGACTATCAGTTGCATTGGACAGTGGCCATTCCCCTGAT
ATTCTCTCTCTGGAGACTAGAAATAAAACCAATGAAAAGCTTATTGCTCATTTAAATATTCAGGTTGACT
TTCTTCAGCGAGCTAATAAAGACCTGGAGAAGCGTATACAGGAGCTTATGGAAACCAAGGAAACAGTGAC
TACTGAAGTTGTTAATCTAAGTAACAAAAATGAAAAACTCTGCCAAGAATTAACTGAAATAGATCAGTTA
GCACAGCAGTTAGAAAGACATAAAGAAGAAGTGCTTGAGACTGCCGATAAAGAACTTGAGGAAGCAAAGA
AAGAGATTAAAAGAAAGCTTTCTGAAATGCGGGATCTTGAAGAAACAATGGCAAAACTTCAGCTGGAATT
AAACTTATGCCATAAAGAAAAGGAGAGACTGAGTGATGAACTACTTATAAAATCAGACCTGGAAACTGTT
GTTCATCAGCTTGAACAAGAAAAGCAAAGACTTAACAAAAAAATGGAAAGTTTTGCAGTTACAGAAAGAG
AACTTACTCTGGAAGTTGAAAGGATGAGGCTAGAACATGGAATAAAACGTAGAGACAAGTCACCCTCTCG
TTTAGATACATTTCTGAAAGGTATAGAAGAAGAACGAGATTTTTATAAGAAAGAACTAGAAAGACTCCAA
CATCTAATACAGCGAAGATCTTGCTCTACAAATTATTCTACACGTGAAAAAATTCCAATATTTAAAACGC
TAGAAAAGGGTGATTACAACTCAGAAATTCATCTGATCACAAGAGAAAGAGATGAACTTCAGCGTATGCT
GGAAAGATTTGAAAAACATATGGAGGACATACAGTCCAATGTTAAATTATTGACAGCAGAAAGAGATAAA
CTAAGTGTCTTATATAATGAAGCTCAGGAACAGTTATGTGCACTAAGACAAGAGTCTACCAAAACCACAG
TCTCCCATAATATTGTGAGTCTTATGGAAAAAGAAAAAGAACTTGCATTATCTGACTTAAGAAGGATTAT
GTCAGAAAAAGAAGCTTTAAAAGAAAAGTTAAAAAATCTCCAGGAAATGAGTATTTTTGAAAAATCAGAA
TTAGAGAAAACTATTGAACATTTGACATGTATTAACCATCAGCTTGAAGGTGAAAAATGTGATTTAAAGT
CTAAAGTGTTAATAATGAAAGAAACAGTAGAGTCATTAGAGAACAAAGCAAAACTCCAAGCTCAAAAACT
TAGCCATGTGGCTGGTGACTCATCTCATCGGAAGACAGAGATGAACTCGCTTAGGATAGTAAATGAGCAG
CTACAGAGGTCACTTGATGATTGTCAGCACCGGTTGTCGAAGAAAAGAGGTGAACTTGAATCAGCCCAAG
CCCAAATTAACATACTGGAGGAAAAAATAGGTAAACTACACCTTCAGATGACTTCGCAGAATGAAGAGGC
TCATGTAATGAAAAAGACCATTGGTGTTATTGACAAAGAAAAAGACTTTCTTCAGGAGACTGTAGATGAG
AAGACAGAAAAGATTGCAAACTTGCAAGAAAACCTGGCTAATAAAGAAAAAGCTATTGCTCAGATGAAGA
AGACAGTCTCAGAGTATGAATCTTCTATGAACCAGCTAAAGGAAACGTTGATTAATCGGGACCGAGAAAT
AAGCAGCCTCCGGCGCCAGCTTGATGCAGCTCACAAAGAACTTGATGAGGTAGGAAAATCTAAAGAAATG
TCTTTTAAGGAAAACAGAAGATTACAAGATGATCTGGCTACAATGGCAAGAGAAAACCAGGAAATCTCAC

TGGAATTGGAAGCAGCAGTGCAAGAAAAAGAAGAAATGAAGAGTAGAGTTCATAATTACATAACTGAGGT
GTCACGATGGGAGAGCTTAATGGCTGCTAAGGAAAAAGAAAATCAAGATTTGTTAGATAGATTTCAGATG
CTTCATAACCGTGCTGAAGACTGGGAAGTCAAAGCCCATCAAGCTGAGGGAGAAAGCAGCTCAGTTCGAC
TGGAACTTCTTTCTATTGACACAGAGAGGAGACACCTTCGAGAAAGGGTGGAGCTATTAGAAAAAGAAAT
TCAGGAGCACATAAATGCACATCATGCTTATGAATCTCAGATCTCATCAATGGCAAAAGCCATGTCTAGA
TTAGAAGAAGAGCTGAGACATCAAGAAGATGCAAAAGCGGTGGTGTTGAATGATCTGTCGTCTCTTAGAG
AACTTTGCATTAAGCTTGATTCAGGCAAAGATATTATGACCCAACAACTGAATTCCAAAAGCCTGGAGTT
AGAGAGGGTCATGGCGGAATTAGAAAATACAAAATCAGAAGCAGAACTGTTAAAAAAACAACTGTCAAGC
GAGAGACATACAATTAAAAACCTTGAATCATTGTTGGCTACAAATAGAGATAAGGAATTTCATTCTCATT
TAACATCCCACGAGAAGGATACAGAAATTCAGCTACTTAAAGAGAAGTTAACCCTTTCAGAAAGCAAATT
AACTAGTCAAGGCCGGGAAAACACCATGCTTCGGGCTAAAGTGACACAATTACAAACCGATCACGATGCT
CTGAAAAGGCAGATTTCAACTGAAAGATATGAACGAGAACGAGCAATCCAAGAGATGCGTCGCCATGGTC
TTCCCACACCACCCCTTTGTTCTACTCTGAGGTCTCCTTTACATTCTCCTGAACATATAAACTGT
>Equus caballus XM_014738797.1
ATGGCTACAGCTGTAGAAAGAAAGTATATG
AATATCAGAAGAAGATTGGATCAGCTGGGGTACCGCCAGACTCTGACGGTGGATTGTTTACCTTTGGTAG
AAAAACTTTTCAGTGACCTCGTTCATACCACAGAAAGTCTTCGGAAATCAAAATTATCTGCTCTGAAAGC
TGAAAAAGAAAGTGCCAATTTTGATTTTATTTTGGAACCTTATAAACTTGAAAATGCAAGATTGATCAGG
GAAAATAATGAATTATACCTGGAATTAATGAAACAGAGAGAACAATCAGACCAACACATTAAAGAGTTGA
AAACTACATTGAAGAAGTGTGCACGTGAAACAGCTGATCTCAAATTTCTAAATAACCAGTATGTTCATAA
AGTCAAACTTTTGGAGAAAGAGAGCAAAGCTAAAAATGAAAAAATTCAACAACTCCAAGAAAAGAATTTG
CATGCTGTAGTGCAAACTCCAGGTGGCAAGAAAAGGAGTATTGCTTTCAGGCGCCAGCGTATGCAAATTG
ACGAACCAGTCCCTCCCTCTGAAGTCAGTTCATATCCAGTGCCTCAGCCAGAAGACCCTTACATTGCAGA
CCTCCTGCAGGTGGCTGATAACAGAATTCAAGAACTTCAACAGGAAGTCCACCAGTTACAAGAAAAATTA
GCAATGATGGAAAGTGGACTGAGAGATTATAACAAGCAGATTGAGCTAAGAGAGCGAGAGATAGAACGAC
TGTCGGTTGCATTGGATGGTGGTCGCTCCCCTGACATCCTCTCTCTGGAGACTAGAAATAAAACCAATGA
AAAGCTTATTGCTCATTTAAATATTCAGGTTGACTTTCTTCAGCAAGCTAATAAAGACCTGGAGAAGCAT
ATACAAGAGCTTATGGAAACCAAGGAAACAGTAACAACTGAAGTTGTTAATTTAAGTAACAAAAATGAAA
AACTCTGCCAAGAATTAACTGAAATAGACCAGTTAGCACAGCAGTTGGAAAGACATAAAGAAGAAGTACT
TGAGACTGCTGATAAAGAACTTGAGGAAGCAAAGAAAGAGATTAAAAGAAAACTCTCTGAAATGCGGAAT
CTTGAAGAAACAATGGCAAAACTTCAGCTGGAATTAAACTTATGCCATAAAGAAAAGGAGAGACTGAATG
ATGAACTCCTTATAAAATCAGACCTGGAAACTGTTGTTCATCAGCTTGAACAAGAAAAGCAACGACTTTC
CAAAAAAATTGAAAGTTTTGCAGTTACAGAAAGAGAACTTACTTTGGAAGTTGAGAGAATGAGACTAGAA
CATGGAATAAAACGTCGAGACAAGTCACCTTCTCGTTTAGATACTTTCCTGAAAGGTATAGAAGAAGAAC
GAGATTTTTATAAGAAAGAGCTAGAAAGACTCCAGCGTATAATACAGCGAAGATCTTGCTCTACAAATTA
TTCTGCACGTGAAAAAATTCCAGGATTTAAAACGCTAGAAAAGGGTGATTACAATTCAGAAATTCATGTG
ATCACAAGAGAAAGAGATGAACTTCAACGTATGCTGGAAAGATTTGAAAAATATATGGAGGATATACAAT
CCAATGTTAAATTATTGACAGCAGAAAGAGATAAACTAAGTGTCTTATATAAAGAGGCTCAGGAAGAATT
ATCTGCTCTAAGACAAAAATCTACCCAAACCACAGTCCCCCATAATATTGTTAGTCTCATGGAAAAGGAA
AAAGAACTTGCATTTTCTGACTTAAGAAGGATTACGGCAGAAAAGGAAGCTTTAAAAGAAAAGTTAAAAA
ATCTCCAGGAAATGAATCTTTTTGGAGCATCAGAATTAGAGAAAACTATTCAACATTTGACATGTGTTAA
TCACCAGCTTGAAAGTGAAAAATGTGAATTAAAGTCTAAAATGTTAATAATGAAAGAAACAATAGAATCA
TTAGAGAACAATGCACTACTCCAAGCTCAAAAACTTAGCCATGTGGCTGGTGACTCATCTCATCAGAAAA
CAGAGATGAACTCACTTAGGATAGTAAATGAGCAGCTACAGCGGTCGGTTGATGAGTATCAGCACCGACT
TTCCATAAAAAGAGGTGAACTTGAATCAGCCGAAACACAAATTAAAATTCTGGAGGAAAAAATAGGTAAA
CTACACCTTAAGATGACTTCACAGGATGAAGAGGCTCATGTAATGAAAAAGACCATTGGAGTCATTGATC
AGGAAAAAGATTTTCTTCAGGAGACTGTAGATGAGAAGACAGAAAAGATTGCAAACTTGCAAGAAAACCT

AGCTAATAAAGAAAAAGCTATTGCTCAGATGAAGATAACAGTCTCAGAGTATGAATCATCTATGAACCAG
CTAAAGGAAACATTGATGAATCGGGACCGTGAGATCAGCAGCCTCCGGCGCCAGCTTGATGCAGCTCACA
AAGAACTTGATGAAGTAGGAAGATGTAAAGAAGTGTCCTTTAAGGAAAACAGAAGATTGCAAGATGATCT
GGCTACAATGGCAAGAGAAAACCAGGAAATCTCATTGGAATTAGAAGCAGCAGTGCAAGAAAAAGAAGAA
ATGAAGAGTAGAGTTCATAATTACATAACTGAGGTGTCACGATGGGAGAGCTTAATGGCTGCCAAGGAAA
AAGAAAATCAAGATTTGTTAGATAGATTTCAGATGCTTCATAACCGTGCTGAAGACTGGGAAGTTAAAGC
CCATCAAGCTGAGGGAGAAAGCAGCTCGGTTCGACTGGAACTCCTTTCTATTGACACGGAGAGGAGACAC
CTTCGAGAAAGAGTGGATCTATTGGAAAAAGAAATTCAGGAGCACATAAATGCACATCATGCCTATGAAT
CTCAGATCTCATCAATGGCAAAAGTGGTATCTAGATTAGAAGAAGAGCTGAGACATCAAGGAGAGGAGAA
AGCAGCAGTATTAAATGATATGTCATCTCTTAGAGAACTTTGCATAAAGCTTGATTCTGGGAAAGATATT
ATGACCCAGCAATTGAATTCCAAAAACCTTGAGTTTGAGAGGGTCGTGGTGGAATTAGAAAATGTAAAAT
CAGAATCAGACCTGTTAAGAAAACAATTGTCAAGTGAGAGACATACAATTAAAAACCTTGAATCATTGTT
GGCTACAAATAGAGATAAAGAATTTCATTCTCATTTAACCTCCCATGAGAAGGATACAGAAATTCAGCTA
CTAAAAGAGAAGTTAAGCCTTTCGGAAAGCAAATTAAATAGTCAAAGCCGGGAAAACACCATGCTTCGGG
CTAAAGTGGCACAGTTACAAACAGATCATGATGCTCTGAAAAGGCAGATTTCAGCTGAAAGATATGAACG
AGAACGAGCAATCCAAGAGATGCGTCGCCATGGTCTTCCCACACCACCCCTCAGTTCTACTCTGAGGTCT
CCTTTACATTCTCCTGAAGACGTGAAC
>Equus asinus XM_014835800.1
ATGGCTACAGCTGTAGAAA
GAAAGTATATGAATATCAGAAGAAGATTGGATCAGCTGGGGTACCGCCAGACTCTGACGGTGGATTGTTT
ACCTTTGGTAGAAAAACTTTTCAGTGACCTCGTTCATACCACAGAAAGTCTTCGGAAATCAAAATTATCT
GCTCTGAAAGCTGAAAAAGAAAGTGCCAATTTTGATTTTATTTTGGAACCTTATAAAATTGAAAATGCAA
GATTGATCAGGGAAAATAATGAATTATACCTGGAATTAATGAAACAGAGAGAACAATCAGACCAACACAT
TAAAGAGTTGAAAACTACATTGAAGAAGTGTGCACGTGAAACAGCTGATCTCAAATTTCTAAATAACCAG
TATGTTCATAAAGTCAAACTTTTGGAGAAAGAGAGCAAAGCTAAAAATGAAAAAATTCAACAACTCCAAG
AAAAGAATTTGCATGCTGTAGTGCAAACTCCAGGTGGCAAGAAAAGGAGTATTGCTTTCAGGCGCCAGCG
TATGCAAATTGACGAACCAGTCCCTCCCTCTGAAGTCAGTTCATATCCAGTGCCTCAGCCAGAAGACCCT
TACATTGCAGACCTCCTGCAGGTGGCTGATAACAGAATTCAAGAACTTCAACAGGAAGTCCACCAGTTAC
AAGAAAAATTAGCAATGATGGAAAGTGGACTGAGAGATTATAACAAGCAGATTGAGCTAAGAGAACGAGA
GATAGAACGACTGTCGGTCGCATTGGATGGTGGTCGTTCCCCTGACATCCTCTCTCTGGAGACTAGAAAT
AAAACCAATGAAAAGCTTATTGCTCATTTAAATATTCAGGTTGACTTTCTTCAGCAAGCTAATAAAGACC
TGGAGAAGCATATACAAGAGCTTATGGAAACCAAGGAAACAGTGACAACTGAAGTTGTTAATTTAAGTAA
CAAAAATGAGAAACTCTGCCAAGAATTAACTGAAATAGACCAGTTAGCACAGCAGTTGGAAAGACATAAA
GAAGAAGTACTTGAGACTGCTGATAAAGAACTTGAGGAAGCAAAGAAAGAGATTAAAAGAAAACTCTCTG
AAATGCGGAATCTTGAAGAAACAATGGCAAAACTTCAGCTGGAATTAAACTTATGCCATAAAGAAAAGGA
GAGACTGAATGATGAACTCCTTATAAAATCAGACCTGGAAACTGTTGTTCATCAGCTTGAACAAGAAAAG
CAACGACTTTCCAAAAAAATTGAAAGTTTTGCAGTTACAGAAAGAGAACTTACTTTGGAAGTTGAGAGAA
TGAGACTAGAACATGGAATAAAACGTCGAGACAAGTCACCTTCTCGTTTAGATACATTCCTGAAAGGTAT
AGAAGAAGAACGAGATTTTTATAAGAAAGAGCTAGAAAGACTCCAGCGTATAATACAGCGAAGATCTTGC
TCTACAAATTATTCTGCACGTGAAAAAATTCCAGGATTTAAAACGCTAGAAAAGGGTGATTACAATTCAG
AAATTCATGTGATCACAAGAGAAAGAGATGAACTTCAGCGTATGCTGGAAAGATTTGAAAAATATATGGA
GGATATACAATCCAATGTTAAATTATTGACAGCAGAAAGAGATAAACTAAGTGTCTTATATAAAGAGGCT
CAGGAAGAATTATCTGCTCTAAGACAAAAATCTACCCAAACCACAGTCCCCCATAATATTGTTAGTCTCA
TGGAAAAGGAAAAAGAACTTGCATTATCTGACTTAAGAAGGATTACGGCAGAAAAGGAAGCTTTAAAAGA
AAAGTTAAAAAATCTCCAGGAAATGAATCTTTTTGGAGCATCAGAATTAGAGAAAACTATTCAACATTTG
ACATGTGTTAATCACCAGCTTGAAAGTGAAAAATGTGAATTAAAGTCTAAAATGTTAATAATGAAAGAAA
CAATAGAATCATTAGAGAACAATGCACTACTCCAAGCTCAAAAACTTAGCCATGTGGCTGGTGACTCATC

TCATCAGAAAACAGAGATGAACTCACTTAGGATAGTAAATGAGCAGCTACAGCGGTCAGTTGATGAGTAT
CAGCACCGACTTTCCATAAAAAGAGGTGAACTTGAATCAGCCGAAACACAAATTAAAATTCTGGAGGAAA
AAATAGGTAAACTACACCTTAAGATGACTTCACAGGATGAAGAGGCTCATGTAATGAAAAAGACCATTGG
AGTCATTGATCAGGAAAAAGATTTTCTTCAGGAGACTGTAGATGAGAAGACAGAAAAGATTGCAAACTTG
CAAGAAAACCTAGCTAATAAAGAAAAAGCTATTGCTCAGATGAAGATAACAGTCTCAGAGTATGAATCAT
CTATGAACCAGCTAAAGGAAACATTGATGAATCGGGACCGTGAGATCAGCAGCCTCCGGCGCCAGCTTGA
TGCAGCTCACAAAGAACTTGATGAAGTAGGAAGATGTAAAGAAGTGTCCTTTAAGGAAAACAGAAGATTA
CAAGATGATCTGGCTACAATGGCAAGAGAAAACCAGGAAATCTCATTGGAATTAGAAGCAGCAGTGCAAG
AAAAAGAAGAAATGAAGAGTAGAGTTCATAATTACATAACTGAGGTGTCACGATGGGAGAGCTTAATGGC
TGCCAAGGAAAAAGAAAATCAAGATTTGTTAGATAGATTTCAGATGCTTCATAACCGTGCTGAAGACTGG
GAAGTTAAAGCCCATCAAGCTGAGGGAGAAAGCAGCTCAGTTCGACTGGAACTCCTTTCTATTGACACGG
AGAGGAGACACCTTCGAGAAAGAGTGGATCTATTGGAAAAAGAAATTCAGGAGCACATAAATGCACATCA
TGCCTATGAATCTCAGATCTCATCAATGGCAAAAGTGGTATCTAGATTAGAAGAAGAGCTGAGACATCAA
GGAGAGGAGAAAGCAGCAGTATTAAATGATATGTCATCTCTTAGAGAACTTTGCATAAAGCTTGATTCTG
GGAAAGATATTATGACCCAGCAATTGAATTCCAAAAACCTTGAGTTTGAGAGGGTCGTGGTGGAATTAGA
AAATGTAAAATCAGAATCAGACCTGTTAAGAAAACAATTGTCAAGTGAGAGACATACAATTAAAAACCTT
GAATCATTGTTGGCTACAAATAGAGATAAAGAATTTCATTCTCATTTAACCTCCCATGAGAAGGATACAG
AAATTCAGCTACTAAAAGAGAAGTTAAGCCTTTCGGAAAGCAAATTAAATAGTCAAAGTCGGGAAAACAC
CATGCTTCGGGCTAAAGTGGCACAGTTACAAACAGATCATGATGCTCTGAAAAGGCAGATTTCAGCTGAA
AGATATGAACGAGAACGAGCAATCCAAGAGATGCGTCGCCATGGTCTTCCCACACCACCCCTCAGTTCTA
CTCTGAGGTCTCCTTTACATTCTCCTGAAGACGTGAAC
>Lipotes vexillifer XM_007450260.1
ATGACTACAGCTGCAGAAAGAAAATATATTAATATTAGGAAAA
GATTGGATCAGTTGGGATACCGCCAGACTCTGACAGTGGAGTGTTTACCTTTGGTAGAAAAACTTTTCAG
TGATCTAGTTCATACAACAGAAAGTCTTCGGAAATCAAAATTATCTGCTGTGAAAGCTGAAAAAGAAAGT
GCCAATTTTGATTTTGTATTGGAACCTTATAAACTTGAAAATGCAAGATTGAGTAAGGAAAATAATGAAT
TATACCTGGAATTAATGAAACTGAGAGAACAATCAGGCCAACACATTAAAGAGTTGAAAACCACATTGAA
AAAGTGTGCACGTGAAACAGCTGATCTGAAATTTCTAAATAATCAATATGTTCATAAACTCAAGCTTTTG
GAGAAAGAGAGTAAAGCTAAGAATGAAAAAATTCAACAACTTCAAGAAAAGAATTTGCAAGCTATAGTAC
AAACTCCAGGTGGCAAGAAAAGAAGTATTGCTTTCAGGCGCCAGCGTATGCAAATTGATGAACCAGTCCC
TCCCTCTGAAGTCAGTTCTTATCCAGTTCCTCAGCCAGATGACCCTTACATTGCAGACCTCCTGCAAGTG
GCTGATAACAGAATTCAAGAACTTCAGCAAGAAGTCTACCAGTTACAAGAAAAGTTAGCAGTGATGGAAA
GTGGACTGAGAGATTATAACAAGCAGATTGAACTAAGAGAACGAGAGATAGAACGACTGTCGGTTGCATT
GGATGGGGGTCGCTCCCCTGATATCCTTTCTCTGGAGACTAAAAATAAAGTCAACGAAAAGCTTATTGCT
CATTTAAATATTCAGGTTGACTTTCTTCAGCAAGCTAATAAAGACCTGGAAAAGCATATACAAGAGCTTA
TGGAAACCAAGGAAACAGTAACTACTGAAGTTGTTAATTTAAGTAACAAAAATGAAAAACTCTGCCAGGA
ATTAACTGAAATAGACCAGTTAGCACAGCAGTTGGAAAGACATAAAGAAGAAGTGCTCGAGACTGCCGAC
AAAGAACTTGAGGAAGCAAAGAAAGAGATTAAAAAAAAGCTGTCTGAAATGCAGAATCTTGAAGAAACAA
TGGGAAAACTTCAACTGGAATTAAACTTATGTCATAAAGAAAAGGAGAGGCTGAGTGATGAACTCCTTAT
AAAATCAGATCTGGAAACTGTTGTTCATCAGCTTGAACAAGAAAAGCAAAGACTTAACAAAAAAATTGAA
AGTTTCGCAGTTACAGAAAGAGAACTTACTTTGGAAGTTGAGAGGATGAGGCTAGAACATGGAATAAAAC
GACGAGACAAGTCACCTTCTCGTTTAGATACGTTTCTGAAAGGTATAGAAGATGAACGAGATTTTTATAA
GAAAGAGCTAGAAAGACTCCAGCATTTAATACAGCGAAGATCTTGCTCTCGAAGTCATTCTACATGTGAA
AAAATTCCAGTATTTAAGACACTAGAAAAGGGTGATTACAACTCAGAAATTCATCTGATCACAAGAGAAA
GAGATGAACTTCAGCATATGCTAGAAAGATTTGAAAAACATATGGAGGATATACAGTCCAATGTTAAATT
ATTGACAGCAGAAAGAGATAAACTAAGTGTCTTATATAATGAAGCTCAGGAAGAATTATCTGCACTCAGA
CAAGAATCTACCCAAACCACAGTCTCCCATAATACTGTTAGTCTTATGGAAAAGGAAAAAGAACTTGCAT

TATCTGACTTAAGAAGGATTATGGCAGAAAAAGAAGCTTTAAAAGAAAAGTTAAAACATCTCCATGAAAT
GAGTATTTTGGGAAAATCAGAATTAGAGAAAACTGTTGAACATTTGACATGTGTTAATCACCAGCTTGAA
AACGAAAAAATGTGAATTAAAGTCTAAAATGTTAAAAATGAAAGAAACAATAGAGTCTTTAGAGAAAAAAG
TAGAACTCCAAGCTCAAAAACTTAGCCATGTGGCTGGTGACTCATCTCATCAGAAAACAGAGATGAACTC
ACTTAGGCTAGTAAATGAGCAGCTACAGCGGTCACTTGAGGACCATCAGCACCAACTTTCCATGAAAAGA
AGTGAACTTGAATCAGCACAAGCACAAGTTAAAATACTGGAGGAAAAAATAGATAAACTACACTTCAAGA
TGACTTCACAGAATGAAGAGGCTCATGTAATGAAAAAGACCATTGGTGTTATTGATAAAGAAAAAGACAT
TCTTCAGGAGACTGTAGATGAGAAGACAGAAAAGATTGCAAACTTGCATGAAAACCTAGCTAATAAAGAA
AAAGCTATTTCTCAGATGAAGATAACAGTCTCAGAGTATGAATCTTCTATGAACCACCTAAAGGAAACAT
TGATTAATCGGGACCGTGAGATAAGCAGCCTCCGGCGCCAGCTTGATGCAGCTCACAAGGAACTTGATGA
AGTAGGAAGATCTAAAGAAATGTCTTTTAAGGAAAACAGGAGATTACAGGATGATCTGGCTACAGTGGCA
AGAGAAAACCAGCAAATTTCATTGGAATTAGAAGCAGCAGTGCAAGAAAAAGAAGAAATGAAGAGTAGAG
TTCATAATTACATAACTGAAGTGTCACGATGGGAGAGCTTAATGGCTGCTAAGGAAAGAGAAAATCAAGA
TTTGTTAGATAGATTTCAGATGCTTCATGACCGTGCTGAAGACTGGGAGGTCAAAGCCCATCAAGCTGAG
GGAGAAAGCAGCTCAGTTCGACTGGAACTTCTTTCTATTGACACCGAGAGGAGACACCTTCGAGAAAGAG
TGGAGCTACTAGAAAAAGAAATTCAGGAGCACATAAATGCACATCATGCTTATGAATCTCAGATCTCATC
CATGGCAAAAGCCATGTCTAGATTAGAAGAAGAGCTGAGACACCAAGAAGATGAGAAAGCAGCAGTATTA
AATGATGTGTCGTCTCTTAGAGACCTTTGCATTAAGCTTGATTCAGGCAAAGATATTATGACCCAGCAAT
TGAATTCCAAAAACCTTGAATTTGAGAGGGTTGCAGTGGAATTAGAAAATGTAAAATCAGAATCAGAGCT
GTTAAAAAAACAACTGTCAAGTGAGAGACATACGATTAAAAACCTTGAATCATTGTTGGCTACAAATAGA
GATAAGGAATTTCATTCCCATTTAACCTCCCGCGAGAAGGATACAGAAATTCAGCTACTTAAAGAGAAGT
TGACCCTTTCAGAAAGCAAATTAAATAGTCAAAGCCGGGAAAACACCATGCTTCGGGCTAAAATGGCACA
ATTACAAACAGATCTTGATGTTCTGAAAAGGCAGATTTCAACTGAAAGATATGAACGAGAACGAGCAATC
CAAGAGATGCGTCGGCACGGTCTTCGCACACCACCCCTTAGTTCTACTCTGAAGTCTCCTTTACATTCTC
CTGAACATATAAAC
>Orcinus orca XM_004268257.1
ATGACTACAGCTGCAGAAAGAAAATA
TATTAATATTAGGAAAAGATTGGATCAGTTGGGATACCGCCAGACTCTGACAGTGGAGTGTTTACCTTTG
GTAGAAAAACTTTTCAGTGATCTAGTTCATACAACAGAAAGTCTTCGGAAATCAAAATTATCTGCTGTGA
AAGCTGAAAAAGAAAGTGCCAATTTTGATTTTGTATTGGAACCTTATAAACTTGAAAATGCAAGATTGAG
TAAGGAAAATAATGAATTATACCTGGAATTAATGAAACTGAGAGAACAATCAGGCCAACACATTAAAGAG
TTGAAAACCACATTGAAAAAGTGTGCACGTGAAACAGCTGATCTGAAATTTCTAAATAATCAATATGTTC
ATAAGCTCAAACTTTTGGAGAAAGAGAGTAAAGCTAAGAATGAAAAAATTCAACAACTTCAAGAAAAGAA
TTTGCAAGCTATAGTACAAACTCCAGGTGGCAAGAAAAGAAGTATTGCTTTCAGGCGCCAGCGTATGCAA
ATTGATGAACCAGTCCCTCCCTCTGAAGTCAGTTCTTATCCAGTTCCTCAGCCAGATGACCCTTACATTG
CAGACCTCCTGCAAGTGGCTGATAACAGAATTCAAGAACTTCAGCAAGAAGTCTACCAGTTACAAGAAAA
GTTAGCAGTGATGGAAAGTGGACTGAGAGATTATAACAAGCAGATTGAACTAAGAGAACGAGAGATAGAA
CGACTGTCGGTTGCATTGGATGGGGGTCGCTCCCCTGATATCCTTTCTCTGGAGACTAAAAATAAAGCCA
ACGAAAAGCTTATTGCTCATTTAAATATTCAGGTTGACTTTCTTCAGCAAGCTAATAAAGACCTGGAAAA
GCATATACAAGAGCTTATGGAAACCAAGGAAACAGTAACTACTGAAGTTGTTAATTTAAGTAATAAAAAT
GAAAAACTCTGCCAGGAATTAACTGAAATAGACCAGTTAGCACAGCAGTTGGAAAGACATAAAGAAGAAG
TGCTCGAGACTGCCGACAAAGAACTTGAGGAAGCAAAGAAAGAGATTAAAAAAAAGCTATCTGAAATGCA
GAATCTTGAAGAAACAATGGGAAAACTTCAACTGGAATTAAACTTATGTCATAAAGAAAAGGAGAGGCTG
AGTGATGAACTCCTTATAAAATCAGACCTGGAAACTGTTGTTCATCAGCTTGAACAAGAAAAGCAAAGAC
TTAACAAAAAAATTGAAAGTTTCGCAGTTACAGAAAGAGAACTTACTTTGGAAGTTGAGAGGATGAGGCT
AGAACATGGAATAAAACGACGAGACAAGTCACCTTCTCGTTTAGATACGTTTCTGAAAGGTATAGAAGAT
GAACGAGATTTTTATAAGAAAGAGCTAGAAAGACTCCAGCATTTAATACAGCGAAGATCTTGCTCTCGAA

GTCATTCTACATGTGAAAAAAATTCCAGTAATTAAGACACTAGAAAAGGGTGATTACAACTCAGAAATTCA
TGTGATCACAAGAGAAAGAGATGAACTTCAGCATATGCTAGAGAGATTTGAAAAACATATGGAGGATATA
CAGTCCAATGTTAAATTATTGACAGCAGAAAGAGATAAACTAAGTGTCTTATATAATGAAGCTCAGGAAG
AATTATCTGCACTCAGACAAGAATCTGCCCAAACCACAGTCTCCCATAATACTGTTAGTCTTATGGAAAA
GGAAAAAGAACTTGCATTATCTGACTTAAGAAGGATTATGGCAGAAAGAGAAGCTTTAAAAGAAAAGTTA
AAAAATCTCCATGAAATGAGTATTTTGGGAAAATCCGAATTAGAGAAAACTGTTGAACATTTGACATGTG
TTAATCACCAGCTTGAAAACGAAAAATGTGAATTAAAGTCTAAAATGTTAAAAATGAAAGAAACAATAGA
GTCTTTAGAGAAAAAAGTAAAACTCCAAGCTCAAAAACTTAGCCATGTGGCTGGTGACTCATCTCATCAG
AAAACAGAGATGAACTCACTTAGGCTAGTAAATGAGCAGCTACAGCGGTCACTTGAGGACCATCAGCACC
AACTTTCCATGAAAAGAAGTGAACTTGAATCAGCACAAGCACAAGTTAAAATACTGGAGGAAAAAATAGA
TAAACTACACTTCAAGATGACTTCACAGAATGAAGAGGCTCATGTAATGAAAAAGACCATTGGTGTTATT
GATAAAGAAAAAGACATTCTTCAGGAGACTGTAGATGAGAAGACAGAAAAGATTGCAAACTTGCATGAAA
ACCTAGCTAATAAAGAAAAAGCTATTACTCAGATGAAGATAACAGTCTCAGAGTATGAATCTTCTATGAA
CCACCTAAAGGAAACATTGATTAATCGGGACCGTGAGATAAGCAGCCTCCGGCGCCAGCTTGATGCAGCT
CACAAGGAACTTGATGAAGTAGGAAGATCTAAAGAAATGTCTTTTAAGGAAAACAGAAGATTACAGGATG
ATCTGGCTACAGTGGCAAGAGAAAACCAGCAAATTTCATTGGAATTAGAAGCAGCAGTGCAAGAAAAAGA
AGAAATGAAGAGTAGAGTTCATAATTACATAACTGAAGTGTCACGATGGGAGAGCTTAATGGCTTCTAAG
GAAAGAGAAAATCAAGATTTGTTAGATAGATTTCAGATGCTTCATGACCGTGCTGAAGACTGGGAGGTCA
AAGCCCATCAAGCTGAGGGAGAAAGCAGCTCAGTTCGACTGGAACTTCTTTCTATTGACACCGAGAGGAG
ACACCTTCGAGAAAGAGTGGAGCTACTAGAAAAAGAAATTCAGGAGCACATAAATGCACATCATGCTTAT
GAATCTCAGATCTCATCCATGGCAAAAGCCATGTCTAGATTAGAAGAAGAGCTGAGACACCAAGAAGATG
AGAAAGCAGCAGTATTAAATGATGTGTCATCTCTTAGAGACCTTTGCATTAAGCTTGATTCAGGCAAAGA
TATTATGACCCAGCAATTGAATTCCAAAAACCTTGAATTTGAGAGGGTTGCAGTGGAATTAGAAAATGTA
AAATCAGAATCAGAGCTGTTAAAAAAACAACTGTCAAGTGAGAGACATACGATTAAAAACCTTGAATCAT
TATTGGCTACAAATAGAGATAAAGAATTTCATTCCCATTTAACCTCCCACGAGAAGGATACAGAAATTCA
GCTACTTAAAGAGAAGTTAACCCTTTCAGAAAGCAAATTAAATAGTCAAAGCCGGGAAAACACCATGCTT
CGGGCTAAAATGGCACAATTACAAACAGATCTTGATGTTCTGAAAAGGCAGATTTCAACTGAAAGATATG
AACGAGAACGAGCAATCCAAGAGATGCGTCGGCACGGTCTTCGCACACCACCCCTTAGTTCTACTCTGAA
GTCTCCTTTACATTCTCCTGAACATACAAAC
>Erinaceus europaeus XM_007523852.2
ATGACTACAGCTGCAGAAAGAAAATATATTAATATTAGAAAAAGATTGGATCAGTTGGGATACCGACAGC
CTCTGTCAGTGGAATGTTTGCCTTTGGTAGAAAAACTTTTCAGTGACCTAGTTCATACAACAGAAAGTCT
TCGGAAATCAAAATTAACTGCTGTGAAAGCCGAAAAAGAAAGTGCTAATTTTGATTTTGTTTTGGAGCCT
TACAAACTTGAAAATGCAAAATTGAGCAAGGAAAACAATGAATTATACCTGGAATTAATGAAACTAAGAG
AACATTCAGATCAACACATTAAAGAGTTAAAAACTACTTTGAAGAAATGTGCACGGGAAACATCTGATCT
GAAATTTTTAAATAACCAGTATGTTCATAAACTCAAACTTCTAGAGAAAGAGAGCAAAGCTAAGAATGAA
AAAATTCAACAACTTCAAGAAAAGAATTTGCACGCTGTGGTGTTAACTCCAGGTGGAAAGAAAAGAAGTA
TCGCTTTCAGGCGCCAGTGTATGCACATAGATGAGCCAGTCCCTCCCTCTGAAGTCAGTTCATACCCAGT
TCCTCAGCCAGATGACCCTTACATTGCGGACCTCCTTCAAGTGGCTGACAATAGAATTCAAGAACTTCAA
GAAGAAGTCTACCACTTACAAGAAAAGTTATCAATGATGGAGAATGGACTGAGGAATTACAACATGCAGA
TTGAGCTCAGAGAACGTGAGATTGAACGTTTGTCACTTGCATTGGATAGTGGGCGCTCTCCTGATATCCT
TTCTCTGGAGACTAGAAATAAAAACAATGAAAAGCTTATTGCTCATTTGAATATTCAGGTTGACTTTCTT
CAACAAGCTAATAAAGGTCTGGAGAAGCATATACAAGAGCTTATGGAAACCAAGCAAACAGTGACTTCTG
AAGTTGTTAATTTAAGTAACAAAAATGAAAAACTCTGCCAAGAATTAACTGAAATAGACCAGTTAGCACA
GCAGTTGGAAAGACATAAAGAAGAAGTACTTGAAACTGCTGATAAAGAACTTGAGGAAGCAAAGAAAGAG
ATTAAAAGGAAGCTCTCTGAAATGCGGGACCTTGAAGAAACAATGGCAAAACTTCAACTGGAATTAAGCC
TGTGTCATAAAGAAAAGGAGAGACTGAATGATGAACTTCTTATAAAATCAGACCTAGAAACTGTTGTTCA

TCAGCTTGAACAAGAAAAGCAAAGACTTACCAGAAAATTGGAAGGTTTTGCTACTACAGAAAGAAAACTT
ACTTTGGAAGTTGAGAGGATGAGGGTAGAGCATGGAATAAAGCGTCGAGATAAGTCGCCTTCTCGTTTAG
ATACATTTCTGAAAGGCATTGAAGAAGAACGAGACTATTATAAGAAAGAGCTTGAAAGACTTCAACATAT
AATACATCGAAGATCTTGCTCTACAGCATACTCTTTACGTGATAAAAATTCATCATACAAAACACCAGAA
AAGGATGATTACAATTCAGAAATTCATATGATTGCAAGAGAAAGAGATGAACTTCAACATATGCTAGAAA
GATTTGAAAAACATATGGAAGACATACAGTCCAATGTTAAATTATTGACAGCAGAAAGAGATAAACTAAA
TGTCTTATATAATGAAGCTCAAGAAGAATTAACTGCACTAAGAAAAGAAGCCACTCAAACTGTAGCCTCC
CATAATATTGTTAGTCTTATGGAAAAGGAAAAAGAACTTGCATTGTCTGACTTAAGAAGGATTATGGCAG
AAAAGGAAGCCTTAAAAGAAAAGTTGAAAAGTCTCCAGGAAATGAATCATTTTGGAAAATCAGAGTTAGA
GAAAGTGATAGAGCATTTGACGTGTGTTAATCATCAGCTTGAAGAGGAAAAACGTGAATTAGTGTCTAAG
ATGTTAGTAATGAAAGAAACAATAGAGTCATTAGAAAGTAGAATGAAAATTCAAGCTCACAAACTTAGCC
ATGCAGCCGGTGACACATCTCATCAGAAAACAGAGTTGAACTCACTTAGGATAGTAAATGAGCAGCTACA
GCAGTCACTTGATGATTACCAACACCGACTTTCCATAAAAAGGAATGAGGTTGAAATAGCCCAAGCACAA
ATTAAAACACTGGAGGATAAAATAGATCAGCTGCACCTTAAGATGACCACACAGAATGAGGAGGCCCATG
TAATGAAAAAGACCATTGGTGTTATTGATAAAGAAAAAGACTATCTTCAGGAGACTGTAGATGAAAAGAC
AGAAAAGATCGCAAACTTGCAAGAAAGCCTAGTTGGCAAAGAAAAAGCTATTACTCAAATGAAGGCAGCA
ATCTCAGAGTATGAATCTTCTGTGAACCAACTAAAAGAAACACTGACTAATCGGGACTGTGAGATAAACA
GTCTTCGGCGCCAGCTTGATGCAGCTCACAAAGAACTTGATGAAGTAGGAAGAACTAAAGAACTGTCTTT
TAAGGAAAACAGAAGATTACAAGATGATCTGGCTACGATGGCAAGAGAAAACCAAGAAATCTCATTGGAA
TTGGAAGTAGCAGTGCAAGAAAAGGAAGAAATGAAGAGTAGAGTTCATAATTACATAACTGAGGTGTCAC
GATGGGAGAGCTTGATGGCTACTAAGGAAAAAGAAAATAAAGATTTATTAGATAGATTCCAGATGCTTCA
TAGCCGTGCCGAAGACTGGGAGGTCAAAGCTCACCAAGCTGAGGGACAGAGCAGCTCAGTTCGACTGGAA
CTTCTTTCTGTTGACACAGAGAGGAGACACCTTCGAGAAAGAGTGGAACTATTAGAAAAGGAAATTCAGG
AGCACATAAATGCACATCATGCTTATGAATCTCAGATCTCATCAATGGCAAAAGTCGTGTCTAGGTTAGA
AGAAGAACTGAGGCAGCAAGAAGATGACAAAGCATCAGTGTTAAGTGATCTGACATCTCTTAGAGAACTT
TGCATTAAGCTTGATTCAGGCAAAGATATTATGACCCAACAACTAAATTCCAAAAGCCTTGAGTTGGAAA
GGGTGATGGTGGAATTAGAAAATGTAAAAACAGAATCAGACTTGTTAAAAAAACAACTTTCAAGTGAGAG
ACATACAATCAAAAACCTTGAAACATTGTTGGCTACAAACAGAGATAAAGAATTTCAGTCTCATTTATCC
TCCCACGAGAAGGATACAGAAATTCAGCTACTTAAAGAGAAGTTAAACCTTTCCGAAAGCAAATTAAATA
GTCAAGGCCGGGAAAACACCATGCTTCGGACTAAAGTGGCACAACTACAAACAGATCATGATACTCTGAA
AAGGCAGATTTCAACTGAGAGATTTGAACGAGAACGAGCAATCCAAGAGATGCGTCGACATGGTCTTTCT
ACACAACCCCTTAGTTCTACTTTGAGGTCACCTTTACAGTCTCCTGAACATGTGAATTTA
>cat ENSFCAT00000014193.3
ATGACTACAGCTGCAGAAAGAAAGTATATTAATATTAGAAAAAGATTGGATCAGTTGGGA
TACCGCCAGACTCTGACAGTGGAGTGTTTACCTTTGGTAGAAAAACTTTTCAGTGACCTA
GTTCATACAACAGAAAGTCTTCGGAAATCAAAATTATCTGCTGTGAAAGCTGAAAAAGAA
AGTGCCAATTTTGATTTTGTTTTGGAACCTTATAAACTTGAAAATGCAAGATTGATAAAG
GAAAATAATGAATTATACCTGGAATTAATGAAACTGAGAGAACAATCAAGCCAACATATT
AAAGAGTTGAAAACTACATTGAAGAAGTGTGCACGTGAAACAGCTGATCTGAAGTTTCTA
AATAACCAATATGTTCATAAACTCAAACTTTTAGAAAAAGAGAGTAAAGCTAAGAATGAA
AGAATTCAACAACTTCAAGAAAAGAATTTGCATGCTGTAGTACAAACTCCAGGTGGCAAG
AAAAGAAGTATTGCTTTCAGGCGCCAGCGTATGCAGATAGACGAACCAGTCCCTCCCTCC
GAAGTCAGTTCATATCCAGTTCCTCAGCCAGATGACCCTTACATTGCAGACCTCCTGCAA
GTGGCTGATAACAGAATTCAAGAACTTCAAGAGGAAGTCCACCAGTTACAAGAAAAGTTA
GCAATGATGGAAAGTGAACTGCGAGATTATAACCAGCAGATTGAGCTAAGAGAACGAGAG
ATAGAACGACTATCAGTTGCATTGGACAGTGGCCGTTCCCCTGATGTCCTCTCTCTGGAG
ACTAGAAACAAAACCAATGAAAAGCTTATTGCTCATTTAAATGTTCAGGTTGACTTTCTT

CAGCAAGCTAATAAAGACCTGGAGAAGCATATACAAGAGCTTATGGAAACCAAGGAAACC
GTAACTACTGAAGTTGTTAATTTAAGTAACAAAAATGAAAAACTCTGCCAAGAATTAACC
GAAATAGACCAGTTAGCACAGCAGTTGGAAAGACATAAAGAAGAAGTGCTTGAGACTGCT
GATAAAGAACTTGGGGAAGCAAAGAAAGAGATTAAAAGAAAGCTTTCTGAAATGCGGGAT
CTTGAAGAAACAATGGCAAAACTTCAACTGGAATTAAACTTATGCCATAAAGAAAAGGAG
AGACTGAGTGATGAACTCCTTATAAAATCAGACCTGGAAACTGTTGTTCATCAGCTTGAA
CAAGAAAAGCAAAGACTTAACAAAAAAATGGAAAGTTTTGCAGTTACAGAAAGAGAACTT
ACTCTGGAAGTTGAAAGGATGAGGCTAGAACACGGAATAAAACGTCGAGACAAGTCACCC
TCTCGTTTAGATACGTTTTTGAAAGGTATAGAAGAAGAACGAGATTTTTATAAGAAAGAG
CTGGAAAGACTCCAACATATAATACAGCGAAGATCTTGTTCTACAAATCATTCTACACGT
GAAAAAATTCCAATATTTAAAACACTAGAAAAGGGTGATTACAACTCAGAAGTTCATCTG
ATCACAAGAGAAAGAGATGAACTTCAGCGTATGCTGGAAAGATTTGAAAGACATATGGAG
GATATACAGTCCAATGTTAAATTGTTGACAGCAGAAAGAGATAAACTAAGTGTCTTATAT
AATGAAGCTCAGGAACAGTTACGTGCCCTAAGACAAGAATCCACCCAAACCACAGCCTCC
CATAATATTATTAGTCTTATGGAAAAAGAAAAAGAACTTGCATTATCTGACTTAAGAAGG
ATTATGGCAGAAAAAGAAGCTTTAAAAGAAAAGTTAAAAAATCTCCAGGAAATGAGTATT
TTTGAAAAATCAAAATTAGAGAAAACTATCGAACATTTGACACGTGTTAATCATCAGCAT
GAAGATGAAAAGTGTGAATTAAAGTCTAAAATGTTAATAATGAAAGAAACAGTAGAGTCA
TTAGAGAACAAAGCAAAACTCCAAGCTCAAAAACTTAGCCATGTGGCTGGTGACTCATCT
CATCACAAAACAGAGATGAACTCACTTAGGTTAGTAAATGAGCAGCTACAGCAGTCACTT
GATGATTGTCAGCATCGGCTTTCCAAAAAAAGAGGTGAACTTGAATCAGCTCAATCACAA
ATTAACTTACTGGAGGAAAAAATAGGTAAACTACACCTTCAGATGACTTCACAGAGTGAA
GAGGCTCATGTAATGAAAAAGACCATTGGTGCTATTGATAAAGAAAAAGACTTTCTTCAG
GAGACTGTAGATGAGAAGACCGAAAAGATTGCAAACTTGCAAGAAAACCTGGCTAATAAA
GGAAAAGCTATTGCTCAGATGAAGATAACAGTCTCAGAGTATGACTCTTCTATGAACCAG
CTAAAGGAAACGTTGACTAGTCGGGACCGAGAAATAAGCAGCCTCCGGCGCCAGCTTGAT
ACAGCTCACAAAGAACTTGATGAAGTAGGAAGATCTAAAGAAGTGTCTTTTAAGGAAAAC
AGAAGATTACAAGATGATTTGGCTACAATGGCAAGAGAAAACCAGGAAATCTCATTGGAG
TTGGAAGCAGCAGTGCAAGAAAAAGAAGAAATGAAGAGTAGAGTTCATAATTACATAACT
GAGGTGTCACGATGGGAGAGCTTAATGGCTGCCAAGGAAAAAGAAAATCAAGATTTGTTA
GATAGATTTCAGATGCTTCATAACCGTGCTGAAGACTGGGAGGTCAAAGCCCATCAAGCT
GAGGGAGAAAGCAGCTCAGTTCGACTGGAACTTCTTTCTATTGACACAGAGAGGAGACAC
CTTCGAGAAAGGGTAGAGCTACTAGAAAAAGAAATTCAGGAGCACATAAATGCACATCAT
GCTTATGAGTCTCAGATCTCATCAATGGCAAAAGCCATGTCTAGATTAGAAGAAGAGCTG
AGACACCAAGAAGATGCAAAAGCAGTAGCGTTGAATGATTTGTCATCTCTTAGAGAACTT
TGCATTAAGCTGGATTCAGGCAAAGATCTTATGACCCAACAACTGAATTCCAAAAACCTG
GAGTTTGAGAGGGTTGTGGTGGAATTAGAAAATGTAAAATCAGAAGCAGACCTGTTCAAA
AAACAACTGGCAAGTGAGAGACATACAATTAAAAGCCTTGAATCATTGTTGGCTACAAAT
AGAGATAAAGAATTTCATTCGCATTTAACCTCCCACGAGAAGGATACAGAAATTCAGCTA
CTTAAAGAGAAGTTAACCCTTTCAGAAAGCAAATTAACTAGTCTAGGCCGGGAAAACACC
ATGCTTCGGGCTAAAGTGACACAGTTACAAACCGATCATGATTCTATGAAAAGGCAGATT
TCAACTGAAAGATATGAACGAGAAAGAGCAATCCAAGAGATGCGTCGACATGGTCTTCCC
ACACCACCCCTTAGTTCAACTCTGAGGTCTCCTTTACATTCTCCTGAACATATAAGCTGT

>elephant XM_003415888.2
ATGACTACTGCTGCAGAAAGAAAGTATG
TTAATATTAGGAAAAGACTGGATCAATTGGGATACCGCCAGACTCTGACAGTAGAGTGCTTACCTTTGGT

AGAAAAACTTTTCAGTGACCTAGTTCATACAACAGAGAGCCTTCGGAAATCAAAATTATCTGCTGTGAAA
GCTGAAAAAGAAAGTGCCAATTTTGATTTTGTTTTGGAACCCTATAAAGTTGAAAATGCAAGATTGTGCA
GGGAAAATAATGAATTATACCTGGAATTAATGAAACTAAGAGAACAATCAGGCCAAAAAACTAAAGAGTT
GAAAGCTGCACTGAAGAAGTGTACAAGTGAAACAGGTGACCTGAAATTTCTAAATAACCAGTACGTTCAT
AAGCTCAAACTTTTGGAAAAAGAGAGCAAAGCTAAGGATGAAAAAATACAGCTACTTCAAGAAAAGAATT
TGCAGGCAGTAGTACAAACTCCAGGTGGCAAGAAAAGAAGTATTGCTTTCAGGCGCCAGCGTATGCAAAT
TGACGAACCAGTTCCTCCTTCTGAAGTCACTTCATATCCAGTTCCTCAGCCAGATGACCCTTATATTGCA
GACCTCCTGCAAGTGGCTGATAACAGGATTCAGGAACTTCAACAGGAAGTCTGCCAGTTACAAGAAAAGT
TAGCAGCAATGGAAAATGGAGTGAGAGATTATAACAAGCAGATTGAGCTAAGAGAACAAGAGATAGAACG
GCTGTCAATGGCATTGGATGGTGGTCGCTGCCCTGATATTCTCACTCTGGAGAGTAGAAATAAAACCAAC
GAGAAGCTTATTGCTCATTTAAATGTTCAGGTTGACTTTCTTCAGCAAGCTAATAAAGACCTGGAGAAGC
ATATACAAGAGCTTATGGAAACCAAGGAAACAGTCACTACTGAAGTTGTTCATTTAAGTAACAAAAATGA
AAAACTCTGCCAGGAATTAACGGAAATAGACCAGTTAGCACAGCAGTTGGAAAGACATAAAGAACAAGTG
CTTGAGACTGCCGATAAAGAAATTGGAGAAGCAAAGAAAGAGATTAAAAGAAAACTCTGTGAAATGCAAG
ATCTTGAAGAAACAATATCAAAACTTCAACTGGAATTAGACTTATGCCATAAAGAAAAGGAGAGACTGAG
TGAAGAATTGCTTATAAAATCAGATCTTGAAACTGTTGTTTGTCAACTTGAACAAGAAAAGCAAAGACTT
TCCAAAAAAGTTGAAACTTTTGCAGATAAAGAAAGAGAACTTACTTTGGAAGTTGAGAGGATGAGGCTAG
AACATGGAATAAAACGCCGAGACAGGTCACCTTCTCGTTTAGATACATTTCTGAAAGGCATAGAAGAAGA
ACGAGATTATTATAAGAAAGAGCTAGAAAGACTCCAGCATATGATACAGCGAAGATCTTGCTCTACAAAG
TATTTTACGCGTGAAAAAGTTCACATTTTAAAAACACCAGAGAAGGGTGATTACAACTCAGAAATTCGTC
TCGTCACAAGAGAAAGAGATGAACTTCATTGTATGCTAGAAAGATTTGAAAAACATATGGAGGAAATACA
GGCCAATGTTAAATTATTGACAGCAGAAAGAGATAAACTAAGTGTCTTATATAATCAAGCTCAGGAAGAA
TTATCTGCTCTAAGACAGGAATCCACCCAGACCACAGTCTCCCCAAATATTATCAGTCTTATGGAAGAGG
AAAAAGAATGTGCATTATCTGATTTAAGAAGAAGTATGGTAGAAAAGGAAGCTTTAAGAGAAAAGTTAAT
TAACCTCCAGGAGAAGAGTCTTTTGGGAAAATCAGAATTAGAACAAACTATTGAACATTTGACATGTGTT
AATCATCAGCTTGAAAATGAAAAATGTGAATTAAAGTCAAAAGTGTTAATAATGAACGAAACAATAGAAT
CATTAGAGAACAAAGTAAAACTCCAAGCTCAGAAACTTAGCCATGTTGCTGGTGACTCATCTCATCAGAA
AACAGAGATGAACTCACTTAGGCTAGTAAATGAGCAGCTACAGCGGTCACTTGACGATTATCAGCACCGA
CTTACCGTAAAAAGAGGTGAACTTGAGTCAGCCCAGTCGCAAATTAAAATACTGGAAGAACAGATAGATA
AACTAAACCTTAAGGTGACTTCACAGGATGAGGAGACTAATGTAATGAAAAAGACTATTGGTGTTATTGA
TAAAGAAAAAGACTTTCTTCAGGAGACTGTAGATGAGAAGACAGAAAAGATTGCAAACTTAGAAGAAAGC
TTAGCTAATAAAGAAAAAACTATTGCTCATATGAAGATAACAATCTCAGAGTATGATTCATCTTTGAATC
ATCTAAAGGATACATTGACTAACCGAGACCGTGAGGTAAGCAGCCTCCGGCGCCAGCTTGACGCAGCTCA
CAAAGAACTTGATGAAGTAGGACGATCTAGAGAAATATCTTTGAAGGAAAATAGAAGATTACAAGATGAT
CTGACTACAATGGCAAGAGAAAATCAGGAAATCTCAATGGAATTGGAAGCAGCAGTGCAAGAAAAAGAGG
AAATGAAGAATAGAGTTCATAATTATATAACTGAGGTGTCACGATGGGAGAGCTTAATGGCTACTAAGGA
AAAAGAAAATCAAGATTTGTTAGATAGATTTCAGATGCTTCATAACCGTGCCGAAGACTGGGAGGTTAAA
GCCCATCAAGCTGAGGGAGAAAGCAGCTCAGTTCGACTCGAACTACTTTCTATTGACACTGAGAGGAGAC
ACCTTCGAGAAAGAGTGGACCTATTAGAAAAAGAAATTCAGGAGCACATAAATGCGCATCACGCTTATGA
ATCTCAGATCTCATCGATGGCAAAAGCCATGTCTCGATTAGAAGAAGAGCTTAGACATCAGGAAGAAGAG
AAGGCAGCAGTGTTAAATGATTTATCGTCTCTTAGAGAACTTTGCATTAAGCTTGATTCAGGCAAGGATA
TTATGACTCAACAACTGAATTCCAAAAACCTTGAGTTTGAGAGGGTTGTGGTGGAATTAGAAAATGTAAA
ATCAGAAGCAGACCTGTTTAAAAAACAACTAACAAGTGAGAGACATACAATTAAAAACCTTGAATCATTG
TTGGCTACAAATAGAGATAAAGAATTTCATTCTCATCTATCATCCCATGAGAAGGATACAGAAATTCAGT
TACTTAAAGAGAAGTTAACCCTTTCAGAAAGCAAATTAACTTGCCAAAGCCGGGAAAACACCATGCTTCG
AACTAAAGTGGCGCAATTACAAATAGATTATGATGCTCTGAAAAGACAAATTTCAACTGAAAGATATGAA
CGAGAACGAGCAATCCAAGAGATGCGTCGACATGGCCTTCCTACACCGCCCCTTAGTTCTACCCTGAGGT

CTCCTTCAAATTCTCCTGAACTTAGAAACTTA
>ferret ENSMPUT00000005090.1
ATGACTACAGCTGCAGAAAGAAAGTACATTAATATTAGAAAAAGATTGGATCAGCTGGGA
TACCGCCAGACTCTGACAGTGGAGTGTTTACCGTTGGTAGAAAAACTTTTCAGTGACCTA
GTTCATACAACAGAAAGTCTTCGGAAATCAAAATTATCTACTGTGAAAGCTGAAAAAGAA
AGTGCCAATTTTGATTTTGTTTTGGAACCATATAAACTTGAAAATGCAAGATTGATCAAG
GAGAATAATGAATTATACCTGGAATTAATGAAACTGAGAGAACAATCAGGCCAACACATT
AAAGAGTTGAAAGCTACATTGAAGAAGTGTGCACGTGAAACAGCTGATCTGAAATTTCTA
AATAACCAATATGTTCATAAACTCAAACTTTTGGAGAAAGAGAGCAAAGCTAAGAATGAA
CGAATTCAGCAACTTCAAGAAAAGAATTTGCACGCTGTGGTACAAACTCCAGGTGGCAAG
AAAAGAAATATTGCTTTCAGGCGCCAACGTATGCAGATTGACGAACCAGTCCCTCCCTCC
GAAGTCCGTTCATATCCAGTTCCGCAGCCCGATGACCCTTACATTGCAGACCTCCTACAG
GTGGCTGATAACAGAATTCAAGAACTTCAACTGGAAGTCCACCAGTTACAAGAAAAGTTA
GCAATGATGGAAAGTGGACTGAGAGATTATACCGAGCAGATTGAACTAAGAGAACGAGAG
ATAGAACGACTATCAGTTGCATTGGACAGTGGCCGCTCCCCTGATATTCTCTTTCTGGAG
TCTAGAAATAAGACCAATGAAAAGCTTATTGCTCATTTAAATATTCAGGTTGACTTTCTT
CAGCGAGCTAATAAAGACCTGGAGAAGCATATACAGGAGCTTATGGAAACCAAGGAAACA
GTGACTACTGAAGTTGTTAATTTAAGTAACAAAAATGAAAAACTCTGCCAGGAATTAACT
GAAATAGACCAGTTAGCACAGCAGTTAGAAAGACATAAAGAAGAAGTGCTTGAGACTGCT
GATAAAGAACTTGAAGAAGCAAAGAAAGAGATTAAAAGAAAGCTTTCTGAAATGCGGGAT
CTTGAAGAAACAATGGCAAAACTTCAGCTGGAATTAAATTTATGCCATAAAGAAAAGGAG
AGACTGAGTGATGAACTACTTATAAAATCAGATCTGGAAACTGTTGTTCATCAGCTTGAA
CAAGAAAAGCAAAGACTTAACAAAAAAATGGAAAGTTTTGCGGTTACAGAAAGAGAACTT
ACTCTGGAACTTGAAAGGATGAGGCTAGAACATGGAATAAAACGTCGAGACAAGTCACCC
TCTCGTTTAGATACATTTCTGAAAGGTATAGAAGAAGAACGAGACTATTATAAGAAAGAG
CTAGAAAAACTCCAACATCTAATACAGCGAAGATCTTGCTCTATAAATTACTCTACACGG
GAAAAAATTCCAATATTTAAAACACTAGAAAAGGGTGATTACAACTCAGAAATTCATCTG
ATCACAAGAGAAAGAGATGAACTGCAGCGTATGCTGGAAAGATTTGAAAAACATATGGAG
GATATACAGTCCAATGTTAAATTATTGACAGCAGAAAGAGATAAACTAAGTGTCTTATAT
AATGAAGCTCAGGAACAGTTACATGCACTAAGACAAGAGTCTACCCAAACCACAATCTCC
CATAATATCGTAAGTCTTATGGAAAAAGAAAAAGAAGTTGCGTTATCTGACTTAAGAAGG
ATTATGTCAGAAAAAGAAGCTTTAAAAGAAAAGTTAAAAAATCTCCAGGAAATGAGTATT
TTTGAAAAATCAAAATTAGAGAAAACTATTGAACATTTGACATGTATTAACCATCAGCTT
GAAGATGAAAAACATGATTTAAAGTCTGAAGTGTTAATAATGAAAGAAACAGTAGAGTCA
TTAGAGAACAAAGCAAAACTCCAAGCTCAAAAACTTAGCCATGTGGCTGGTGATTCATCC
CATCGGAAGACAGAGATGAACTCGCTTAGGATGGTAAATGAGCAGCTACAGCGGTCACTT
GATGATTGTCAGCATCGGCTCTCCAAGAAAAGAGGTGAACTTGAATCAGCCCAATCACAA
ATTAACATACTGGAGGAAAAAATAGGTAAACTACACCTTCAGATGACTTCGCAGAATGAA
GAGGCTCATGTAATGAAAAAGACCATTGGTGTTATTGACAAAGAAAAAGACTTTCTTCAG
GAGACTGTAGATGAGAAGACAGAAAAGATTGCAAACTTGCAAGAAAACTTGGTTAATAAA
GAAAAAGCTATTGCTCAGATGAAGAAGACAATCTCAGAGTATGAATCTTCTATGAACCAG
CTAAAGGAAACGTTGACTAATCGGGACCGAGAAATAAGCAGCCTCCGGCGCCAGCTTGAT
GCAGCTCACAAAGAACTCGATGAGGTCGGAAAATCTAAAGAAATATCTTTTAAGGAAAAC
AGAAGATTACAAGATGATCTGACTACAATGGCAAGAGAAAACCAGGAAATCTCACTGGAA
TTGGAAGCAGCAGTGCAAGAAAAAGAAGAAATGAAGAGTAGAGTTCATAATTACATAACT
GAAGTGTCACGATGGGAGAGCTTAATGGCTGCTAAGGAAAAAGAAAATCAAGATTTGTTA
GATAGATTCCAGATGCTTCATAACCGAGCTGAAGACTGGGAGGTCAAAGCCCATCAAGCT

GAGGGAGAAAGCAGCTCAGTTCGACTGGAACTTCTTTCCATTGACACAGAGAGGAGACAC
CTTCGAGAAAGGGTGGAGCTATTAGAAAAAGAAATTCAGGAGCACATAAATGCACACCAT
GCTTATGAATCTCAGATCTCATCAATGGCCAAAGCCATGTCCAGATTAGAAGAAGAGCTG
AGACATCAGGAAGATGCAAAAGCAGCAGTGTTGAATGATTTGTCGTCTCTCAGAGAACTT
TGCATTAAGCTTGATTCAGGCAAAGACATTATGACCCAACAACTGAATTCCAAAAGCCTG
GAGTTAGAGAGGGTTTCGGCGGAATTAGAAAATGTGAAATCAGAAGCAGAACTATTAAAA
AAACAACTGTCAAGTGAGAGACATACAATTAAAAACCTTGAAACATTGTTGGCTACAAAT
AGAGATAAGGAATTTCATTCTCATTTAACATCCCACGAGAAGGATACAGAAATTCAGCTG
CTTAAAGAGAAGTTAACCCTTTCAGAAAGCAAATTAACTAGTCAAGGCCGGGAAAACACC
ATGCTTCGGGCTAAAGTGACACAATTACAAACTGATCACGATGCTCTGAAAAGGCAGATT
TCAACTGAAAGATATGAACGAGAACGCGCAATCCAGGAGATGCGTCGCCATGGTCTTCCC
ACGCCACCCCTTTGTTCTACTTTAAAGTCTCCTTTACATTCTCCTGAACATATAAACTGT

>pteropus alecto XM_006922515.2
ATGACTACAGCTGCAGAAAGAAAATATATTAATATTAGAAAAAG
ATTGGATCAGTTGGGATACCGCCAGACTTTGACAGTGGAATGTTTACCTTTGGTAGAAAAACTTTTCAGT
GACCTAGTTCATACAACAGAAAGTCTTCGGAAATCAAAATTATCTTCTGTGAAAGCTGAAAAAGAAAGTG
CCAATTTCGATTTTGTTTTGGAACCTTATAAACTTGAAAATGCAAGATTGAGCAGGGAAAATAATGAATT
ATACCTGGAATTAATGAAACTGAGAGAACAATCAGCCCAACACATTAAAGAGTTGAAAACTACATTGAAG
AAGTGCGCATGTGAAACAGCTGATCTGAAATTTTTGAATAACCAATATGTTCATAAACTCAAACTTTTGG
AGAAAGAAAGCAAAGCTAAGAATGAACGAATTCAACAACTTCAGGAAAAGAATTTGCATGCTGTCGTACA
AACTCCAGGTGGCAAGAAAAGAAGTATTGCTTTTAGGCGCCAGCGTATGCAAATTGACGAACCAGCCCCT
CCCTCCGAAGTCAGTTCCTATCCAGTTCCTCAGCCAGATGACCCTTACATTGCAGACCTCCTACAAGTGG
CTGATAACAGAATTCAAGAACTTCAACAGGAAGTCCACCAGTTACAAGAAAAATTATCAGTGATGGAAAG
TGGACTGAGAGATTATAACAAGCAGATTGAACTAAGAGAACGAGAAATAGAACGACTGTCCATCGCATTG
GATGGTGGCCGCTCCTCTGATATCCTCTCTCTGGAGACTAAAAATAAAACCAACGAAAAGCTTATCGCTC
ATTTAAATATTCAGGTTGACTTTCTTCAGCAAGCTAATAAAGACCTGGAGAAGCATATACAAGAGCTTAT
GGAAACCAAGGATACAGTGACTACTGAAGTTGTTAACTTAAGTAACAAAAATGAAAAACTCTGCCAAGAA
TTAAGTGAAATAGACCAGTTAGCACAGCAGTTGGAAAGACATAAAGAAGAAGTGCTTGAGACTGCTGATA
AAGAACTTGGGGAAGCAAAGAAAGAGATTAAAAGAAATCTCTCTGAAATGCGGAATCTTGAAGAAACAAT
GGCAAAACTTCAACTGGAATTAAACTTATGCCATAAAGAAAAAGAGAGACTAAATGATGAACTCCTTATA
AAATCAGACTTGGAAACTGTCGTTCATCAGCTTGAACAAGAAAAGCAAAGACTTGCCAAAAAACTGGAAA
GTTTTGCAGTTACAGAAAGAGAACTTACTTTGGAAGTTGAGAGGATGAGGCTAGAACATGGAATAAAACG
TCGAGACAAGTCACCTTCTCGTTTAGATACATTTCTGAAAGGTATAGAAGAAGAACGAGATTTTTATAAG
AAAGAGCTAGAAAGACTTCAACATATAATACAGCGAAGATCTTGCTCTACAAATTATTCTGCACGTGAAA
AAATTCCAATATTTAAAACACTAGAAAAGGGTGATTACAATTCAGAAATTCATCTGATCACAAGAGAAAG
AGATGAACTTCAGCATATGCTGGAAAAGTTTGAAAAATATATGGAGGATATACAGTCCAATGTTAAATTA
TTGACAGCAGAAAGAGATAAACTAAGTGTCTTATATAATGAAGCTCTGGTAGAATTATCTTCACTAAGAC
AAGAATCCACCCAAAGCACAGTCTCCCACAATATTGTTAGTCTTATGGAAAAAGAAAAAGAACTTGTATT
ATCTGACTTAAGAAGGATTATGGCAGAAAAGGAAGCTTTAAAAGACAAGTTAAAAAATCTCCAGGAAATG
AGTCTTCTTGGAAAATCGGAATTAGAGAAAACTATTGAACATTTGACATGTATTAATCACCAGCTTGAAG
ACGAAAACTGTGAATTAAAGTCTAAAATTTTAATAATGAAAGAAGCAATAGATTCACTAGAGAGCAAAGC
AAAACTTCAAGCCCATAAACTTAACCATGTGGCTGGTGACTCCTCTCTTCAGAAAACAGAGATGAATTCA
CTTAGGATAGTAAATGAGCAGCTACAGCGGTCACTCGATGATTATCAGCACCGACTTTCCTTAAAAAGAG
GTGAACTTGAATCAGCCCAAGCACAAATTAAAATACTAGAGGAAAAAATAAGTAAAATACACCTTAAGAT
GACTTCACAAGATGAAGAGGCTCATGTAATGAAAAAGACCATTGGTGTTATTGATAAAGAAAAAGACTTT
CTTCAGGAGACTGTAGATGAGAAGACAGAACAGATTGCAAACTTACAAGAAAACCTAGCTAATAAAGAAA

ATGTTATTGCTCAGATGAAGATAACAGTCTCAGAGTATGAATCTTCTCTGAACCAGCTAAAAGAAACTCT
AACTAGTCGGGACCGTGAGATAAGCAGCCTCCGGCGCCAGCTTGATGCAGCTCACAAAGAACTCGATGAA
GTAGGAAAATCTAAAGAAATATCTTTTAAGGAAAATAGAAGATTACAAGATGACCTGGCTACAGTGGCAA
GAGAAAACCAGGAAATCTCATTGGAATTGGAAGCAGCAGTGCAAGAAAAAGAAGAAATGAAGAGTAGGGT
TCATAATTACATAACTGAGGTGTCACGATGGGAGAGCTTAATGGCTGCTAAGGAAAAAGAAAATCAAGAT
TTGTTAGATAGATTTCAGATGCTTCATGACCGTGCTGAAGACTGGGAGGTCAAAGCCCATCAAGCTGAGG
GAGAAAGCAGCTCAGTTCGCCTTGAACTTCTTTCTATTGACACAGAGAGGAGACACCTTCGTGAAAGAGT
GGAGCTGTTAGAAAAAGAAATTCAGGAGCACATAAATGCACACCATGCATATGAATCTCAGATCTCCTCA
ATGGCAAAAGCTGTATCTAGATTAGAAGAAGAGCTGAGACATCAAGAAGATGAAAAAGCAGCAGTATTCA
ATGATTTGTCATCTCTTAGAGAACTTTGTATTAAGCTTGATTCAGGCAAAGATATTTTGACCCAACAGTT
GAATTCCAAAAGCCTTGAATTTGAGAGGGTAGTGGTGGAATTAGAAAATATAAAATCAGAATCAGAGCTA
TTAAAAAAACAACTGTCGAGTGAGAGACTTACAATTAAAAACCTTGAATCATTGTTGGCTACAAATAGAG
ATAAAGAATTTCATTCTCATTTAACCTCCCACGAGAAAGATACAGAAATTCAGCTACTTAAAGAGAAGTT
AACCCTTTCAGAAAGCAAATTAACTAGTCAAAGCCGAGAAAACACCATGCTTCGGGCTAAAGTGGCACAA
TTACAAACAGATCATGATGCTCTAAAAAGGCAGATTTCAACTGAAAGATATGAACGAGAACGAGCAGTCC
AAGAGATGCGTCGACATGGTCTTCCCACACCACCGCTTAGTTCTACTCTAAGGTCTCCTTCACATTCTCC
TGAACATATAAACATA
>pteropus vampyrus XM_011362619.1
ATGACT
ACAGCTGCAGAAAGAAAATATATTAATATTAGAAAAAGATTGGATCAGTTGGGATACCGCCAGACTTTGA
CAGTGGAATGTTTACCTTTGGTAGAAAAACTTTTCAGTGACCTAGTTCATACAACAGAAAGTCTTCGGAA
ATCAAAATTATCTTCTGTGAAAGCTGAAAAAGAAAGTGCCAATTTTGATTTTGTTTTGGAACCTTATAAA
CTTGAAAATGCAAGATTGAGCAGGGAAAATAATGAATTATACCTGGAATTAATGAAACTGAGAGAACAAT
CAGCCCAACACATTAAAGAGTTGAAAACTACATTGAAGAAGTGCGCGTGTGAAACAGCTGATCTGAAATT
TTTGAATAACCAATATGTTCATAAACTCAAACTTTTGGAGAAAGAAAGCAAAGCTAAGAATGAACGAATT
CAACAACTTCAGGAAAAGAATTTGCATGCTGTTGTACAAACTCCAGGTGGCAAGAAAAGAAGTATTGCTT
TTAGGCGCCAGCGTATGCAAATTGACGAACCAGCCCCTCCCTCCGAAGTCAGTTGCTATCCAGTTCCTCA
GCCAGATGACCCTTACATTGCAGACCTCCTACAAGTGGCTGATAACAGAATTCAAGAACTTCAACAGGAA
GTCCACCAGTTACAAGAAAAATTATCAGTGATGGAAAGTGGACTGAGAGATTATAACAAGCAGATTGAAC
TAAGAGAACGAGAAATAGAACGACTTTCCATCGCATTGGATGGTGGCCGCTCCTCTGATATCCTCTCTCT
GGAGACTAAAAATAAAACCAATGAAAAGCTTATCGCTCATTTAAATATTCAGGTTGACTTTCTTCAGCAA
GCTAATAAAGACCTGGAGAAGCATATACAAGAGCTTATGGAAACCAAGGATACAGTGACTACTGAAGTTG
TTAACTTAAGTAACAAAAATGAAAAACTCTGCCAAGAATTAACTGAAATAGACCAGTTAGCACAGCAGTT
GGAAAGACATAAAGAAGAAGTGCTTGAGACTGCTGATAAAGAACTTGGGGAAGCAAAGAAAGAGATTAAA
AGAAATCTCTCTGAAATGCGGAATCTTGAAGAAACAATGGCAAAACTTCAACTGGAATTAAACTTATGCC
ATAAAGAAAAAGAGAGACTAAATGATGAACTCCTTATAAAATCAGACTTGGAAACTGTCGTTCATCAGCT
TGAACAAGAAAAGCAAAGACTTGCCAAAAAACTGGAAAGTTTTGCAGTTACAGAAAGAGAACTTACTTTG
GAAGTTGAGAGGATGAGGCTAGAACATGGAATAAAACGTCGAGACAAGTCACCTTCTCGTTTAGATACAT
TTCTGAAAGGTATAGAAGAAGAACGAGATTTTTATAAGAAAGAGCTAGAAAGACTTCAACATATAATACA
GCGAAGATCTTGCTCTACAAATTATTCTGCACGTGAAAAAATTCCAATATTTAAAACACTAGAAAAGGGT
GATTACAATTCAGAAATTCATCTGATCACAAGAGAAAGAGATGAACTTCAGCATATGCTGGAAAAGTTTG
AAAAATATATGGAGGATATACAGTCCAATGTTAAATTATTGACAGCAGAAAGAGATAAACTAAGTGTCTT
ATATAATGAAGCTCTGGTAGAATTATCTTCACTAAGACAAGAATCCACCCAAAGCACAGTCTCCCATAAT
ATTGTTAGTCTTATGGAAAAAGAAAAGGAACTTGTATTATCTGACTTAAGAAGGATTATGGCAGAAAAGG
AAGCTTTAAAAGACAAGTTAAAAAATCTCCAGGAAATGAGTCTTCTTGGAAAATCGGAATTAGAGAAAAC
TATTGAACATTTGACATGTATTAATCACCAGCTTGAAGACGAAAACTGTGAATTAAAGTCTAAAATTTTA
ATAATGAAAGAAGCAATAGATTCACTAGAGAGCAAAGCAAAACTTCAAGCCCATAAACTTAACCATGTGG

CTGGTGACTCCTCTCTTCAGAAAACAGAGATGAATTCACTTAGGATAGTAAATGAGCAGCTACAGCGGTC
ACTCGATGATTATCAGCACCGACTTTGCTTAAAAAGAGGTGAACTTGAATCAGCCCAAGCACAAATTAAA
ATACTAGAGGAAAAAATAAGTAAAATACACCTTAAGATGACTTCACAAGATGAAGAGGCTCATGTAATGA
AAAAGACCATTGGTGTTATTGATAAAGAAAAAGACTTTCTTCAGGAGACTGTAGATGAGAAGACAGAACA
GATTGCAAACTTACAAGAAAACCTAGCTAATAAAGAAAACGTTATTGCTCAGATGAAGATAACAGTCTCA
GAGTATGAATCTTCTCTGAACCAGCTAAAAGAAACTCTAACTAGTCGGGACCGTGAGATAAGCAGCCTCC
GGCGCCAGCTTGATGCAGCTCACAAAGAACTTGATGAAGTAGGAAAATCTAAAGAAATATCTTTTAAGGA
AAATAGAAGATTACAAGATGACCTGGCTACAGTGGCAAGAGAAAACCAGGAAATCTCATTGGAATTGGAA
GCAGCAGTGCAAGAAAAAGAAGAAATGAAGAGTAGGGTTCATAATTACATAACTGAGGTGTCACGATGGG
AGAGCTTAATGGCTGCTAAGGAAAAAGAAAATCAAGATTTGTTAGATAGATTTCAGATGCTTCATGACCG
TGCTGAAGACTGGGAGGTCAAAGCCCATCAAGCTGAGGGAGAAAGCAGCTCAGTTCGCCTTGAACTTCTT
TCTATTGACACAGAGAGGAGACACCTTCGTGAAAGAGTGGAGCTGTTAGAAAAAGAAATTCAGGAGCACA
TAAATGCACACCATGCATATGAATCTCAGATCTCCTCAATGGCAAAAGCTGTATCTAGATTAGAAGAAGA
GCTGAGACATCAAGAAGATGAAAAAGCAGCAGTATTCAATGATTTGTCATCTCTTAGAGAACTTTGTATT
AAGCTTGATTCAGGCAAAGATATTTTGACCCAACAGTTGAATTCCAAAAACCTTGAATTTGAGAGGGTAG
TGGTGGAATTAGAAAATATAAAATCAGAATCAGAGCTATTAAAAAAACAACTGTCGAGTGAGAGACTTAC
AATTAAAAACCTTGAATCATTGTTGGCTACAAATAGAGATAAAGAATTTCATTCTCATTTAACCTCTCAC
GAGAAAGATACAGAAATTCAGCTACTTAAAGAGAAGTTAACCCTTTCAGAAAGCAAACTAACTAGTCAAA
GCCGAGAAAACACCATGCTTCGGGCTAAAGTGGCACAATTACAAACAGATCATGATGCTCTGAAAAGGCA
GATTTCAACTGAAAGATATGAACGAGAACGAGCAATCCAAGAGATGCGTCGACATGGTCTTCCCACACCG
CCGCTTAGTTCTACTCTAAGGTCTCCTTCACATTCTCCTGAACATATAAACATA
>myotis lucifugus ENSMLUT00000015042.2
ATGACTACAGCTGCAGAAAGAAAGTATATTAATATTAGAAAAAGATTGGATCAGTTGGGA
TACCGCCAGACTCTGACAGTGGAGTGTTTACCCTTGGTAGAAAAAATTTTCAGTGACCTG
GTTCATACAACAGAAAGTCTTAGGAAATCAAAATTATCTGCTGTGAAAGCTGAAAAAGAA
AGTGCCAATTTTGATTTTGCTTTGGAACCTTACAAACTTGAAAATGCAAAATTGAGCAGG
GAAAATAATGAATTATACCTGGAATTAATGAAACTGAGAGAACAATCGGCCCAACACATT
AAAGAGTTGAAAACTACATTGAAGGAAAGTACACGTGAAACAGCTGATCTGAAATTTCTA
AATAACCAGTATGTTCATAAACTCAAACTTTTGGAGAAAGAGAGCAAAGCTAAGAATGAA
AAAATTCAACAACTTCAAGAAAAGAATTTGCATGCTGTTGTACAGACTCCAGGTGGCAAG
AAAAGAAGTATTGCTTTTAGACGCCAGCGAATGCAAATTGACCAACCAGTCCCTCCCTCT
GAAGTCAGTTCCTATCCAGTTCCTCAGCCAGATGACCCTTACATTGCAGACCTTCTACAA
GTGGCTGATAACAGAATTCAAGAACTTCAACAGGAAGTCCATCAGTTACAAGAAAAATTA
GCAATGATGGAAAGTGGACTGAGAGATTACAGCAAGCAGATTGAATTAAGAGAAAGAGAG
ATAGAACGACTGTCCAATGCATTGGACGGTGGCTGCTCCTCTGATATCCTCTCTCTGGAG
ACTAAAAATAAAACCAACGAAAAGATTATTGCTCATTTAAATATTCAGGTTGACTTTCTT
CAGCAAGCTAATAAAGACCTGGAGAAGCATATACAAGACCTTATGGAAACCAAGGAAACA
GTGACTACTGAAGTTGTTAATTTAAGTAACAAAAATGAAAAACTTTGCCAAGAATTAACT
GAAATAGACCAGTTAGCACAGCAGTTGGAAAGACATAAAGAAGAAGTGCTTGAGACTGCA
GATAAAGAACTTGGGGAAGCAAAGAAAGAGATTAAAAGAAATATTTCTGAAATGCGGAAT
CTTGAAGAAACAATGGCAAAACTTCAACTGGAATTAAACTTATGCCATAAAGAAAAAGAG
AGACTGAGTGATGAGCTTCTTATAAGATCAGACTTGGAAACTGTTGTTCATCAGCTTGAA
CAAGAAAAGCAAAGGCTTAGCAAAAAAATTGAAAGTTTTGGAGATACAGAAAGAGAACTT
ACTTTGGAAGTTGAGAGGATGAGGCTAGAGCATGGAATAAAACGTCGAGACAAGTCACCT
TCTCGTTTAGATACATTTCTGAAAGGTATAGAAGAAGAACGAGATTTTTATAAGAAAGAG
CTAGAAAGACTCCAGCATATAATACAGAGAAGATCTTGCTCTATAAATTATTCTGCACAT
GAAAAAGCTTCAACATTTAAAATGCTAGAAAAGGGTGATTACAATTCTGAAATTCATCTG

ATCACAAGAGAAAGAGATGAGCTTCAACATATGCTGGAAAGATTTGAGAAACATATGGAG
GATATACAGGCCAATGTTAAATTATTGACAGCAGAAAGAGATAAACTAAGTGTTTTATAT
CATGAAGCTCAGGAAGAATTATCTTCACTAAGACAAGAATCCACCCAAAGCACAGTCTCC
CATAATGTTGTTAGTCTGATGGAAAAGGAAAAAGAAGTTGCAATATCTGATTTAAGAAGG
GTTATAGCAGAAAAGGAAGCTTTAAAAGACAAGTTAAAAAGTTTCCAGGAAATGAGTCTT
TTTGGAAAATCAGAATTAGAGAAAACTATTGAACATTTAACATATGTTAATCACCAGCTT
GAAAATGAAAACTGTGAATTAAAGTCTAAAATGTTAATAATGAAAGAATCAATGGAATCA
TTAGAGAACAAAGCAAAATTCCAAGCTCAAAAACTTAGCCATGTGGCTGGTGACTCATCT
CTTCATAAAACAGAGATGAACTCACTTAGGATAGTAAATGAGCAGCTACAGCGGTCACTT
GATGATTATCAGCACCGACTTTCCATCCAAAGAGGTGAACTTGAATCAGCCGAAGCACAA
ATTAAAATGCTGGAGGAAAATATAGGTAAACTACACCTTAAGATGACTTCACAGGATCAA
GAGGCTCATGTCATGAAAAAGACCATTGGTGTTATTGATAAAGAAAAAGATTTCCTTCAG
GAGACTGTAGATGAGAAGACAGAACAGATTGCAAACTTGGAAGAAAACCTTGCTAATAAA
GAAAAAGCTATTGCTCAGATGAAGATAACGTTCTCAGAGTATGAATCTTCTCTGAACCAA
CTAAAGGAAACTCTAACTAATCGGGACCGTGAGATAAACAGCCTCCTGCGCCAGCTTGAT
GCAACTAACAAAGAACTCGATGATGTAGGAAGATCTAAAGAAATATCTTGTAAGGAAAAT
AGAAGATTACAAGATGATCTGGCTACAATGGCAAGAGAAAACCAAGAAATCTCACTGGAA
TTGGAAGCAGCGGTGCAAGAAAAAGAAGAAATGAAGAGTAGAGTTCATAATTACATAACG
GAGGTGTCACGATGGGAGAGCTTAATGGCTGCTAAGGAAAAAGAAAATCAAGATTTGTTA
GATAGATTTCAAATGCTTCATAACCGTGCTGAAGACTGGGAAGTCAAAGCCCACCAAGCT
GAGGGAAAAAGCAGCTCAGTTCGACTGGAACTTCTTTCTATTGACACAGAGAGGAGGCAC
CTTCGTGAAAGAGTGGAGCTATTAGAAAAAGAAATTCAGGAGCACATAAATGCACATCAT
GCCTATGAATCTCAGATCTCATCAATGACAAAAGCCATATCTAGATTAGAAGAAGAGCTG
AGACATCAAGAAGATGAGAAAGCAACAGTATTCAACGATTTGTCATCTCTTAGAGAACTC
TGTATTAAGCTTGACTCAGGCAAAGATATTATGACCCAACAATTGAATGCTAAAAACCTT
GAATATGAGAGGGTTGTGATGGAATTAGAAAATGTAAAATCAGAATCAGACCTGTTAAAA
ACACAACTGTCAAGTGAGAGACATACGATTAAAAATCTTGAATCATTGTTGGCTACGAAT
AGAGATAAAGAATTCCATTCTCATTTAACTTCCCAAGAGAAGGAAACAGAAATTCAGTTA
CTTAAAGAGAAGTTAACTCTTTCAGAAAGCAAATTAACTAGCCAAAGCCGGGAGAACACC
ATGCTTCGGGCTAAAGTGGCACAATTACAAACAGATCATGATGCCATGAAAAGGCAGATA
TCAACTGAAAGATATGAACGAGAACGAGCAATCCAGGAGATGCGTCGGCATGGTCTTCCC
ACACCACCCCTTAGTTCCACTCTGAAATCTCCTTTACATTCTCCTGAACACATCAAC
>panda ENSAMET00000009161.1
ATGACTACAGCTGCAGAAAGAAAGTACATTAATATTAGAAAAAGATTGGATCAGTTGGGA
TACCGCCAGACTCTGACAGTGGACTGTTTACCGTTGGTAGAAAAACTTTTCAGTGACCTA
GTTCATACAACAGAAAGTCTTCGGAAATCAAAATTATCTACTGTGAAAGCTGAAAAAGAA
AGTGCCAATTTTGATTTTGTTTTGGAACCTTATAAACTTGAAAATGCAAGATTGATCAAG
GAAAATAATGAATTATACCTGGAATTAATGAAACTGAGAGAGCAATCAGGCCAACACATT
AAAGAGTTGAAAACTACGTTGAAGAAGTGTGCACGTGAAACAGCTGATCTGAAATTTCTA
AATAACCAATATGTTCATAAACTCAAACTTCTGGAGAAAGAGAGCAAAGCTAAGAATGAA
AGAATTCAACAACTTCAAGAAAAGAATTTGCATGCTGTAGTACAAACTCCAGGTGGCAAG
AAAAGAAGTATTGCTTTCAGGCGCCAGCGTATGCAGATTGATGAACCAGTCCCTCCCTCT
GAAGTCAGTTCATATCCGGTTCCGCAGCCAGATGACCCTTACATTGCAGATCTCCTGCAG
GTGGCTGATAACAGAATTCAAGAACTTCAACTGGAAGTCCACCAGTTACAAGAAAAGTTA
GCAATGATGGAAAGTGGACTGAGAGATTATACTGAGCAGATTGAGCTAAGAGAACGAGAG
ATAGAACGACTATCAGTTGCATTGGACAGTGGCCGCTCCCCTGATATTCTCTCTCTGGAG
ACTAGAAATAAAACCAATGAAAAGCTTATTGCTCATTTAAATATTCAGGTTGACTTTCTT

CAGCGAGCTAATAAAGACCTGGAGAAGCATATACAGGAGCTTATGGAAACCAAGGAAACA
GTGACTACTGAAGTTGTTAATTTAAGTAACAAAAATGAAAAACTCTGCCAAGAATTAACT
GAAATAGACCAGTTAGCACAGCAGTTAGAAAGACATAAAGAAGAAGTGCTTGAGACTGCC
GATAAAGAGCTTGAGGAAGCAAAGAAAGAGATTAAAAGAAAGCTTTCTGAAATGCGGGAT
CTTGAAGAAACAATGGCAAAACTTCAGCTGGAATTAAATTTATGTCATAAAGAAAAGGAG
AGACTAAGTGATGAACTACTTATAAAATCAGACCTGGAAACTGTTGTTCATCAGCTTGAA
CAAGAAAAGCAAAGACTTAACAAAAAAATGGAAAGCTTTGCAGTTACAGAAAGAGAACTT
ACTCTGGAAGTTGAAAGGATGAGGCTAGAACATGGAATAAAACGTCGAGACAAGTCACCC
TCTCGTTTAGATACATTTCTCAAAGGTATAGAAGAAGAACGAGATTTTTATAAGAAAGAG
CTAGAAAGACTCCAACATATAATACAGCGAAGATCTTGCTCTACAAATTATTCTACACGT
GAAAAAATTCCAATATTTAAAACACTAGAAAAGGGCGATTACAACTCAGAAATTCATCTG
ATCACAAGAGAAAGAGATGAACTTCAGCGTATGCTGGAAAGATTTGAAAAACATATGGAG
GATATACAGTCCAATGTTAAATTATTGACAGCAGAAAGAGATAAACTAAGTGTCTTATAT
AATGAAGCTCAGGAACAGTTATGCGCACTAAGAAAGGAGTCCACCCAAAACGCAGTCTCC
CATAATATTGTTAGTCTTATGGAAAAAGAAAAAGAACTTGCATTATCTGACTTAAGAAGG
ATTATGTCAGAAAAAGAAGCTTTAAAGGAAAAGTTAAAAAATCTCCAGGAAGTGAATATT
TTTGAAAAATCAAAATTAGAGAAAACTATTGAACATTTGACATGTATTAACCATCAGCTT
GAAGATGAAAAATGTGATTTGAAGTCTAAAGTGTTAATAATGAAAGAAACAGTAGAGTCA
TTAGAGAACAAAGCAAAACTCCAAGCTCAAAAACTTAACCATGTGGCTGGTGACTCATCT
CATCGGAAGACAGAGATGAACTCACTTAGGATAGTAAATGAGCAGCTACAGAGGTCACTT
GATGATTGTCAGCACCGGCTCTCCATGAAAAGAGGTGAACTTGAATCAGCCCAAGCACAA
ATTAACATACTGGAGGAAAAAATAGGTAAACTACACCTTCAGATGACTTCGCAGAATGAA
GAGGCTCATCTAATGAAAAAGACCATTGGTGTTATTGACAAAGAAAAAGATTTTCTTCAG
GAGACTGTAGATGAGAAGACAGAAAAGATTGCAAACCTGCAAGAAAACCTGGTTAATAAA
GAAAAAGCTATTGCTCAGATGAAGAAGACCATCTCAGAGTATGAATCTTCTATGAACCAG
CTAAAGGAAACGTTGACTAATCGGGACCGAGAAATAAGCAGCCTCCGGCGCCAGCTCGAT
GCAACTCACAAAGAACTTGATGAGGTAGGAAAATCTAAAGAAGTGTCTTTTAAAGAAAAC
AGAAGATTACAAGATGATCTGGCTACAATGGCAAGAGAAAACCAGGAAATCTCACTGGAA
TTGGAAGCAGCCGTGCAAGAAAAAGAAGAAATGAAGAGTAGAGTTCATAATTACATAACT
GAGGTGTCACGATGGGAGAGCTTAATGGCTGCGAAGGAAAAAGAAAATCAAGATTTGTTA
GATAGATTTCAGATGCTTCATAACCGTGCTGAAGACTGGGAGGTCAAAGCCCATCAAGCC
GAGGGAGAAAGCAGCTCAGTTCGACTGGAACTTCTTTCTATTGACACAGAGAGGAGACAC
CTCCGAGAAAGGGTGGAGCTATTAGAAAAAGAAATTCAGGAGCACATAAATGCACATCAC
GCTTATGAATCTCAGATCTCATCCATGGCAAAAGCCATGTCTAGATTGGAAGAAGAGCTG
AGACATCAAGAAGACGCAAAAGCGGCAGTGTTGAATGATCTGTCATCTCTTCGAGAGCTT
TGCATTAAGCTTGATTCAGGCAAAGATATTATGACCCAACAACTGAATTCGAAAAACCTG
GAGTTAGAGAGGGTTGTGGCGGAATTAGAAAATGTAAAATCAGAAGCAGAGCTGTTAAAA
AAACAACTGTCAAGTGAGAGACATACAATTAAAAACCTTGAATCATTGTTGGCTACAAAT
AGAGATAAGGAATTTCATTCTCATTTAACATCCCACGAGAAGGATACAGAAATTCAGCTA
CTTAAAGAGAAGTTAACCCTTTCAGAAAGCAAATTAACTAGTCAAGGCCGGGAAAATACC
ATGCTTCGGGCTAAAGTGACACAATTACAAACCGATCACGATGCTCTGAAAAGGCAGATT
TCAGCTGAAAGATACGAACGAGAACGAGCAATCCAAGAGATGCGTCGCCATGGTCTTCCC
ACACCACCCCTTTGTTCTACTCTGAGGTCTCCTTTACATTCTCCTGAACATATAAACCGT

>cow ENSBTAT00000012468.5
ATGACTACAGCTGCAGAAAGAAAGTATCTTAATATTAGGAAAAGATTGGATCAGTTGGGA
TACCGCCAGACTCTGACAGTGGACTGTATACCTTTGGTAGAAAAACTTTTCAGTGATCTA

GTTCATACAACAGAAAGTCTTCGGAAATCAAAATTATCTGCTGTGAAAGCTGAAAAAGAG
AGTGCCAATTTTGATTTTGTATTGGAACCTTATAAACTTGAAAATGCAAGATTGAGTAAG
GAAAATAATGAATTATACCTGGAATTAATGAAACTGAGAGAACAATCAGGTCAACATATT
AAAGAGTTGAAAACCACATTGAAAAAGTGTGCACGTGAAACAGCTGATCTGAAATTTCTA
AATAATCAATATGTTCATAAACTCAAACTTATGGAGAAAGAAAGCAAAGCTAAGAATGAG
AAAATTCAACAGCTTCAAGAAAAGAATTTGCAAGCTGTAGTTCAAACTCCAGGTGGCAAG
AAAAGAAATATTGCTTTTAGGCGCCAGCGCATGCAGATTGATGAACCAGTCCCTCCCTCT
GAAATCAGTTCATATCCGGTTCCTCAGCCAGATGACCCTTACATCGCAGACCTCCTGCAC
GTGGCTGATAACAGAATTCAAGAACTTCAACAAGAAGTCTGCCAGTTACAAGAGAAGTTA
GCAATGATGGAAAGTGAACTGAGAGATTATAACAAGCAGATTGAACTTAGAGAACGAGAG
ATAGAACGACTGTCGATTGCATTGGATGGGGGTCGCTCCTCTGATATCCTTTCTCTGGAG
ACTAGAAATAAAGCCAACGAAAAGCTTATTGCTCATTTAAATATTCAGGTTGACTTTCTT
CAGCAAGCTAATAAAGATCTGGAAAAGCATATACAAGAGCTTATGGAAACCAAGGAAACA
GTAACATCTGAAGTTGTTAATTTAAGTAACAAAAATGAAAAACTCTGCCAGGAATTAACT
GAAATAGACCAGTTAGCACAGCAGTTGGAAAGACACAAAGAAGAAGTGCTCAAGACTGCT
GACAAAGAACTTGAGGAAGCAAAGAAAGAGATTAAAAAAAAGCTTTCTGAAATGCGGAAT
CTTGAAGAAACAATAGGAAAACTTCAACTGGAATTAAGCTTATGTCATAAAGAAAAGGAG
AGGCTGAGTGATGAACTCCTTATAAAATCAGACCTGGAAACTGTTGTTCATCAGCTTGAA
CAAGAAAAGCAAAGACTTAACAAAAAAATTGAAAGTTTTGCAGTTACAGAAAGAGAACTT
ACTGTGGAAATTGAGAGGATGAGACTAGAACATGGAATAAAACGACGAGACAAGTCACCT
TCTCGTTTAGATACATTTCTGAAAGGTATAGAAGATGAACGAGATTTTTATAAGAAAGAA
CTAGAAAAACTCCAACATATAATACAGCGAAGATCTTGCTCTAGAAGTCATTCTACATGT
GAAAAAACGCCAGTATTTAAAACACTAGAAAAGGGTGATTACGACTCGGATATCCATCTC
ATCACAAGAGAAAGAGACGAACTTCAGCGTATGCTAGAAAGATTTGAAAAGCACATGGTG
GATATACAGTCCAATGTTAAATTATTGACAGCAGAAAGAGATAAACTAAGTGTCTTATAT
AATGAAGCTCAGGAAGAATTATCTGCACTAAAACAAGACTCTACCCAAACTACAGTTTCA
CATAATACTATTAGTCTTATAGAAAAGGAAAAAGAACTTGCATTATCTGACTTAAGAAGG
ATTATGGCAGAAAAGGAAGCTTTAAAAGACAAGTTAAAACATCTCCAGGAAATGAGTGTT
TTTGGAAAATCAGAATTAGAGAAAACTATTGAGCATTTGACATGTGTTAATCACCAGCTT
GAAAACGAAAAATGTGAATTAAAGTCTAAAATATTTATAATGAAAGAAACAATGGAGTCT
TTAGAGAAAAAAGCAAAATTCCAAGCTCAAAAACTTAGCCATGTGGCTGGTGACTCATCT
CATCAGAAAACAGAGATGAACTCCCTTAGGCTAGTAAATGAGCAACTACAGCGGTCACTT
GAGGATTATCAGCACCGACTTAACATGAAAAGAAGTGAACTTGAATCAGCCCAAGCACAA
GTTAAAATACTGGAGGAAAAAATAGGTAAACTACACCTCAGGATGACTTCACAGAATGAA
GAGGCTCATGTAATGAAAAAGACCATTGGTGTTATTGATAAAGAAAAAGACATCCTTCAG
GAGACTGTGGATGAGAAGACAGAAAAGATTGCAAACTTGCATGAAAACCTAGCTAGTAAA
GAAAAAACTATTACTCAGATGAAGATAACAGTCTCAGAGTATGAATCTTCTCTGAACCAC
CTAAAGGAAACATTGATTAATCGGGACCGTGAGATAAGCAGCCTCCGGCGCCAGCTTGAT
GCGGCTCACAAAGAACTTGATGAAGTAGGAAGATCTAAAGAAATGTCTTTTAAGGAAAAC
AGAAGATTACAGGATGATCTGGCTACAATGGCAAGAGAAAACCAGCAAATTTCATTGGAA
TTAGAAGCAGCAGTGCAAGAAAAAGAAGAAATGAAGAGTAGAGTTCATAATTACATAACT
GAAGTGTCACGATGGGAGAGCTTAATGGCTGCTAAGGAAAAAGAAAATCAAGATTTGTTA
GATAGATTTCAGATGCTTCATAACCGCGCTGAAGACTGGGAAGTCAAAGCCCATCAAGCT
GAGGGAGAAAGCAGCTCAGTTCGACTGGAACTTCTTTCTATAGACACAGAGAGGAGACAC
CTTCGAGAGAGAGTGGAGCTGCTAGAAAAAGAAATTCAGGAGCACATGAATGCACATCAT
GCTTATGAATCTCAGATCTCATCAATGGCAAAAGCTATCTCTAGATTAGAAGAAGAGCTG
AGACACCAAGAAGATGAGAAAGCAGCAGTGTTAAATGATTTATCATCTCTTAGAGAACTT

TGCATTAAGCTTGATTCAGGCAAAGATATTATGACCCAACAATTGAATTCCAAAAATCTT
GAATTTGAGAGGGTTACAATGGAATTAGAAAATATAAAATCAGAGTCAGAGCTGTTAAAA
AAACAACTGTTGAGTGAGAGACATACAATTAAAAACCTTGAATCATTGTTGGCTACAAAT
AGAGATAAAGAATTTCATTCCCACTTAACTTCCCATGAGAAGGATACAGAAATTCAGATA
CTTAAAGAGAAGTTGACCCTTTCAGAAAGCAAACTAAATAGTCAAAGCCGGGAAAACACC
ATGCTCCGGGCTAAAATGGCACAATTACAAACAGATCTTGATGTTCTGAAAAGGCAGATT
TCAACTGAAAGATATGAACGAGAACGAGCAATTCAAGAGATGCGTCGGCATGGTCTTCGC
ACGCCACCCCTTAGTTCTACTATGAGGTCTCCTTTACATTCTCCTGAACATATAAAC
>dog XM_539276.4
AT
GACTACAGCTGCAGAAAGAAAGTATATTAATATTAGAAAAAGATTGGATCAGTTGGGATACCGCCAGACT
CTGACAGTGGAGTGTTTACCATTGGTAGAAAAACTTTTCAGTGACCTAGTTCATACAACAGAAAGTCTTC
GGAAATCAAAATTATCTACTGTGAAAGCTGAAAAAGAAAGTGCCAATTTTGATTTTGTTTTGGAACCTTA
TAAACTTGAAAATGCAAGATTGATCAAGGAAAATAATGAATTATACCTGGAGTTAATGAAACTGAGAGAA
CAGTCAAGCCAACACATTAAAGAGTTGAAAACTACGGTGAAGAAGTGTGCACGTGAAACAGCTGATCTGA
AATTTCTAAATAACCAATATGTTCATAAACTCAAACTTTTGGAGAAAGAGAGCAAAGCTAAGAATGAAAG
AATTCAACAACTTCAAGAAAAGAATTTGCATGCTGTAGTACAAACTCCGGGTGGCAAGAAAAGAAGTATT
GCTTTCAGGCGCCAGCGTATGCAGATTGATGAACCAGTCCCTCCCTCTGAAGTCAGTTCATATCCAGTTC
CTCAGCCAGATGACCCTTACATTGCAGACCTCCTGCAGNTGGCTGATAACAGAATTCAAGAACTTCAACA
GGAAGTCCACCAGTTACAAGAAAAGTTAACAATGATGGAAAGTGGACTGAGGGATTATAACGAGCAGATT
GAGCTAAGAGAACGAGAGATAGAACGACTATCAGTTGCATTGGACAGGGGCCGCTCCCCTGATATTCTCT
CTTTGGAGACTAGAAATAAAAGCAATGAGAAGCTTATTGCTCACTTAAATATTCAGGTTGACTTTCTTCA
GCGAGCTAATAAAGACCTAGAGAAGCATATACAAGAGCTTATGGAAACCAAGGAAACAGTGACTACTGAA
GTTGTTAATTTAAGTAACAAAAATGAAAAACTCTGCCAAGAATTAACTGAAATAGACCAGTTAGCACAGC
AGTTAGAAAGACATAAAGAAGAAGTGCTTGAGACTGCCGATAAAGAACTTGGGGAAGCAAAGAAAGAGAT
TAAAAGAAAGCTTTCTGAAATGCGGGATCTTGAAGAAACAATGGCAAAACTTCAACTGGAATTAAACTTA
TGCCATAAAGAAAAGGAGAGACTGAGTGATGAACTACTTATAAAATCAGACCTGGAAACTGTCGTTCATC
AGCTTGAACAAGAAAAGCAAAGACTTAACAAAAAAATGGAAAGTTTTGCAGTTACAGAAAGAGAACTTAC
TCTGGAAGTTGAAAGGATGAGGCTAGAACATGGAATAAAACGTCGAGACAAGTCACCCTCTCGTTTAGAT
ACATTTCTGAAAGGTATAGAGGAAGAACGAGATTTTTACAAGAAAGAGCTAGAAAGACTCCAACATATAA
TACAGCGAAGATCTTGCTCTACAAATTATTCTACACGTGAAAAAATTCCAATATTTAAAACACTAGAAAA
GGGTGATTATAACTCAGAAATTCATCTGATTACAAGAGAAAGAGATGAACTTCAGCGCATGCTGGAAAGA
TTTGAAAAACATATGGAAGATATACAGTCCAATGTTAAGCTATTGACAGCAGAAAGAGATAAACTAAGTG
TCTTATATAATGAAGCTCAGGAACAGTTACATGCACTAAGACAAGAGTCCGCCCAAACCACAGTCTCCCA
TAATATCGTTAGTCTTATGGAAAAGGAAAAAGAACTTGCATTATCTGACCTAAGAAGGATTATGTCAGAA
AAAGAAGCTTTAAAAGAAAAGTTAAAGAGTCTCCAGGAAATGAGTATTTTTGAAAAATCAAAATTAGAGA
AAACTATTGAACATTTGACATGTGTTAATCATCAGCTTGAAGATGAAAGATGTGATTTAAAATCTAAAAT
GTTAATAATGAAAGAAACAGTAGAGTCATTAGAGAACAAAGCAAAACTCCAAGCTCAAAAACTTAGCCAT
GTGGCTGGTGACTCATCTCATCAGAAGACAGAGATGAACTCTCTTAGGCTAGTAAATGAGCAGCTACAGC
GGTCACTTGATGATTGTCAGCACCGGCTCTCCAAGAAAAGAGATGAACTTGAATCAGCCCAAGCACAAAT
AAGCATACTGCAGGAAAAAATAGATAAACTATACCTTCAGATGACTTCGCAGAGTGAAGAGGCTCATGTA
ATGAAAAAGACCATTGATGTTATTGATAAAGAGAAAGACTTTCTTCAGGAGACAGTAGATGAGAAGACAG
AAAAGATTGCAAACTTGCAAGAAAACCTGGTTAATAAAGAAAAAGCTATTGCTCAGATGAAGAAGACAGT
CTCAGAATGTGAATCTTCTATGAACCAGATAAAGGAAACGTTGACTAATCGGGAGCGAGAAATAAGCAGC
CTCCGGCGCCAGCTTGATGCAGCTCACAAAGAACTTGATGAAGTAGGAAAATCTAAAGAAGTGTCTTTTA
AGGAAAACAGAAGATTACAAGAAGATCTGAATACAATGGCAAGAGAAAACCAGGAAATCTCACTGGAATT
GGAAGCAGCAGTTCAAGAAAAAGAAGAAATGAAGAGTAGAGTTCATAATTACATAACTGAAGTGTCACGA

TGGGAGAGCTTAATGGCTGCTAAGGAAAAAGAAAATCAAGATTTGTTAGATAGATTTCAGATGCTTCATA
ACCGTGCTGAAGACTGGGAGGTCAAAGCCCATCAAGCTGAGGGAGAAAGTAGCTCGGTTCGACTGGAACT
TCTTTCAATTGATACTGAGAGGAGACATCTTCGAGAAAGGGTGGAGCTACTAGAAAAAGAAATTCAGGAG
CACATAAATGCACATCATGCTTATGAATCTCAGATTTCATCAATGGCAAAAGCCATGTCTAGATTAGAAG
AAGAGCTGAGGCATCAAGAAGATGCAAAAGCAGCAGTGTTGAATGATTTGTCGTCTCTTAGAGAACTTTG
CATCAAGCTCGACTCAGGCAAAGATATTATGACCCAGCAACTGAATTCCAAAAACCTGGAGTTTGAGAGG
GTTATGGCGGAATTAGAAAATGTAAAATCAGAAGCAGAACTGTTGAAAAAACAACTGTCAAGTGAGAGAC
TTACAATTAAAAACCTTGAATCATTGTTGGCTACAAATAGAGATAAAGAATTTCATTCTCATTTGACTTC
CCACGAGAAGGATACAGAAATTCAGCTACTTAAAGAGAAGTTAACCCTTTCAGAAAGCAAATTAACTAGT
CAAGGCCGGGAAAACACCATGCTTCGAGCTAAAGTGGCACAATTACAAACTGATCATGATGTTCTGAAAA
GGCAGATTTCAACTGAAAGATACGAACGAGAACGAGCAATCCAAGAGATGCGTCGACACGGTCTTCCCAC
ACCACCCCTTAGCTCTACTCTGAGGTCTCCTTTACATTCTCCTGAACATATAAGCTGT
>sheep ENSOART00000003266.1
ATGACTACAGCTGCAGAAAGAAAGTATCTTAATATTAGGAAAAGATTGGATCAGTTGGGA
TACCGCCAGACTCTGACAGTGGACTGTATACCTTTGGTAGAAAAACTTTTCAGTGATCTA
GTTCATACAACAGAAAGTCTTCGGAAATCAAAATTATCTGCTGTGAAAGCGGAAAAAGAG
AGTGCCAATTTTGATTTTGTATTGGAACCTTATAAACTTGAAAATGCAAGGTTGAATAAG
GAAAATAATGAATTATACCTGGAATTAATGAAACTGAGAGAACAATCAGGTCAACATATT
AAAGAGTTGAAAACCACATTGAAAAAGTGTGCACGTGAAACAGCTGATCTGAAATTTCTA
AATAATCAATATGTTCATAAACTCAAACTTATGGAGAAAGAAAGCAAAGCTAAGAATGAG
AAAATTCAGCAGCTTCAAGAAAAGAATTTGCAAGCTGTAGTTCAAACTCCAGGTGGCAAG
AAAAGAAATATTGCTTTCAGGCGCCAGCGGATGCAGATTGATGAACCAGTCCCTCCCTCT
GAAATCAGTTCATATCCGGTTCCTCAGCCAGATGACCCTTACATTGCAGACCTCCTGCAC
GTGGCTGATAACAGAATTCAAGAACTTCAACAAGAAGTCTGCCAGTTACAAGAGAAGTTA
GCAATGATGGAAAGTGAACTGAGAGATTATAACAAGCAGATTGAGCTTAGAGAGCGAGAG
ATAGAACGGCTGTCGACCGCACTGGATGGGGGCCGCTCCTCTGACATCCTTTCTCTGGAG
ACTAGAAATAAAGCCAACGAAAAGCTTATTGCTCATTTAAATATTCAGGTTGACTTTCTT
CAGCAAGCTAATAAAGACCTGGAAAAGCATATACAAGAGCTTATGGAAACCAAGGAAACA
GTAACATCTGAAGTTGTTAATTTAAGTAACAAAAATGAAAAACTCTGCCAGGAATTAACT
GAAATAGACCAGTTAGCGCAGCAGTTGGAAAGACATAAAGAAGAAGTGCTCAAGACTGCT
GACAAAGAACTTGAGGAAGCAAAGAAAGAGATTAAAAAAAAACTTTCTGAAATGCGGAAT
CTTGAAGAAACGATGGGAAAACTTCAGCTGGAATTAAGCTTATGTCATAAAGAAAAGGAG
AGACTGAGTGATGAACTCCTTATAAAATCAGACCTGGAAACTGTTGTTCATCAGCTTGAA
CAGGAAAAGCAAAGACTTAACAAAAAAATTGAAAGTTTTGCAGTTACAGAAAGAGAACTC
ACTGTGGAAGTTGAGAGGATGAGACTAGAACACGGAATAAAACGACGAGACAAGTCACCT
TCTCGTTTAGATACATTTCTGAAAGGTATAGAAGATGAACGAGATTTTTATAAGAAAGAA
CTAGAAAAACTCCAACATATAATACAGCGAAGATCTTGCTCTAGAAGTCATTCTACATGT
GAAAAAATGCCAGTATTTAAAACACTAGAAAAGGGTGATTACGACTCGGATATCGATCTC
ATCACAAGAGAAAGAGATGAACTTCAGCGTATGCTAGAAAGATTTGAAAAGCACATGGTG
GATATACAGTCCAATGTTAAATTATTGACAGCAGAAAGAGATAAACTAAGTGTCTTATAT
AATGAAGCTCAGGAAGAATTATCTGCACTAAGACAAGACTCTACCCAAACTACAGTCTCA
CATAATACTGTTAGTCTTATAGAAAAGGAAAAAGAACTTGCATTATCTGACTTAAGAAGG
ATTATGGCAGAAAAGGAAGCTTTAAAAGACAAGTTAAAACATCTCCAGGAAATGAGTATT
TTTGGAAAATCAGAATTAGAGAAAACTATTGAGCATTTGACATGTGTTAATCACCAGCTT
GAAAACGAAAAATGTGAATTAAAGTCTAAAATATTTATAATGAAAGAAACAATGGAGTCT
TTAGAGAAAAAAGCAAAATTCCAAGCTCAAAAACTTAGCCATGTGGCTGGTGACTCATCT
CATCAGAAAACAGAGATGAACTCTCTTAGGCTACTAAATGAGCAACTACAGCAGTCAGTT

GAGGATTATCAGCACCGACTTAACATGAAAAGAAGTGAACTTGAATCAGCCCAAGCACAA
GTTAAAATACTGGAGGAAAAAATAGGTAAACTACACCTCAGGATGACTTCACAGAATGAA
GAGGCTCATGTAATGAAGAAGACCATTGGTGTTATTGATAAAGAAAAAGACATCCTTCAG
GAGACTGTGGATGAGAAGACAGAAAAGATTGCAAACTTACATGAAAACCTAGCTAGTAAA
GAAAAAACTATTACGCAGATGAAGATAACGGTCTCAGAGTATGAATCCTCTCTGAACCAC
CTAAAGGAAACATTGATTAATCGGGACCATGAGATAAGCAGCCTCCGGCGCCAGCTTGAT
GCGGCTCACAAAGAACTTGATGAAGTAGGAAGATCTAAAGAAATGTCTTTTAAGGAAAAC
AGAAGATTACAGGATGATCTGGCTACAATGGCAAGAGAAAACCAGCAAATTTCATTGGAA
TTAGAAGCAGCAGTGCAAGAAAAAGAAGAAATGAAGAGTAGAGTTCATAATTATATAACT
GAAGTGTCGCGATGGGAGAGCTTAATGGCTGCTAAGGAAAAAGAAAATCAAGATTTGTTA
GATAGATTTCAGATGCTTCATAACCGTGCTGAAGACTGGGAAGTCAAAGCTCATCAAGCC
GAGGGAGAAAGCAGCTCAGTTCGACTGGAACTTCTTTCTATAGACACAGAGAGGAGACAC
CTTCGAGAGAGAGTGGAGCTGCTAGAAAAAGAAATTCAGGAGCACATGAATGCACATCAT
GCTTACGAATCTCAGATCTCATCAATGGCAAAAGCTATTTCTAGATTAGAAGAAGAGCTG
AGACACCAAGAAGATGAGAAAGCAGCAGTGCTAAATGATTTGTCATCTCTTAGGGAACTT
TGCATTAAGCTTGATTCAGGCAAAGATATTATGACCCAACAGTTGAATTCCAAAAATCTT
GAATTTGAGAGGGTTACAATGGAATTAGAAAATATAAAATCAGAGTCAGAGCTGTTAAAA
AAACAACTGTTGAGTGAGAGACATACAATTAAAAACCTTGAATCATTGTTGGCTACAAAT
AGAGATAAAGAATTTCATTCCCATTTAACTTCCCATGAGAAGGATACAGAAATTCAGCTA
CTTAAAGAGAAGTTGACCCTTTCAGAAAGCAAACTAAATAGTCAAAGCCGGGAAAACACC
ATGCTCCGGGCCAAAATGGCACAATTACAAACAGATCTTGATGTTCTGAAAAGGCAGATT
TCAACTGAAAGATATGAACGAGAACGAGCAATTCAAGAGATGCGTCGGCATGGTCTTCGC
ACGCCACCCCTTAGTTCTACTATGAGGTCTCCTTTACATTCTCCTGAACATATAAAC

ZNF335

>Human ENST00000322927.2
ATGGAGGAGAACGAGGTGGAGAGCAGCAGCGACGCGGCCCCTGGGCCTGGCCGGCCCGAG
GAGCCCTCTGAGAGCGGCCTGGGTGTGGGCACCTCAGAAGCCGTGTCCGCCGACAGCAGC
GACGCCGCGGCCGCCCCGGGGCAGGCAGAGGCCGATGACTCTGGCGTGGGGCAAAGCTCG
GACCGCGGCAGCCGTTCTCAGGAGGAGGTATCTGAGAGCAGCTCGAGCGCAGACCCCCTG
CCTAATAGCTACCTCCCTGATTCATCGTCTGTGTCTCATGGGCCAGTGGCAGGGGTGACA
GGCGGTCCCCCAGCACTTGTGCACTCTAGTGCACTCCCAGACCCCAACATGCTGGTGTCC
GACTGCACAGCTTCCTCCTCGGACCTGGGCTCGGCCATCGACAAGATCATCGAGTCCACC
ATCGGGCCCGACCTCATCCAGAACTGCATCACTGTGACCAGTGCTGAGGATGGCGGGGCC
GAGACCACACGGTACCTGATCCTACAGGGCCCAGATGATGGAGCCCCCATGACATCACCA
ATGTCCAGTTCCACCTTGGCCCACAGCCTAGCAGCCATTGAGGCCCTGGCAGATGGCCCC
ACATCCACATCCACATGCCTGGAGGCACAGGGTGGCCCAGCTCCCCGGTGCAGCTGCCC
CCAGCCTCCGGTGCCGAAGAGCCGGACCTGCAGAGCCTGGAGGCCATGATGGAGGTGGTG
GTGGTGCAGCAGTTCAAATGCAAGATGTGCCAGTACCGGAGCAGCACCAAGGCCACACTG
CTGCGCCACATGCGGGAACGCCACTTCCGTCCAGTAGCAGCAGCAGCTGGT
AAAAAAGGACGTCTACGGAAGTGGAGCACCTCCACCAAGAGCCAAGAGGAAGAGGGACCA
GAGGAGGAGGACGATGATGACATTGTAGACGCTGGAGCCATTGATGACCTGGAGGAGGAT
AGCGACTATAATCCAGCTGAGGATGAGCCCCGAGGCCGGCAGCTTCGGCTCCAGCGCCCC
ACCCCCAGTACCCCAAGGCCCCGAAGGAGACCTGGCCGGCCCCGGAAGCTGCCCCGCCTG
GAGATCTCAGACGATGGTGTGGAAGGAGAGCCTCTAGTGAGTTCCCAGAGTGGA
CAGAGCCCTCCAGAGCCACAGGATCCCGAGGCTCCCAGCTCCTCAGGCCCAGGACACCTG
GTGGCCATGGGCAAGGTGAGCAGGACCCCTGTGGAAGCTGGTGTGAGCCAGTCAGATGCA

GAGAACGCAGCCCCCTCCTGCCCGGATGAGCATGACACTCTGCCCCGGCGCCGAGGTCGA
CCTTCCAGGCGCTTCCTAGGCAAGAAATACCGCAAGTACTATTACAAGTCGCCCAAACCA
CTTTTGAGGCCCTTCCTGTGCCGCATCTGTGGTTCTCGCTTTCTGTCCCACGAGGACCTG
CGCTTCCACGTCAACTCCCATGAGGCTGGCGATCCCCAGCTCTTCAAGTGCCTGCAGTGC
AGCTATCGTTCCCGCCGCTGGTCCTCGCTCAAGGAGCACATGTTCAACCACGTGGGCAGC
AAGCCCTACAAGTGTGACGAGTGCAGCTACACCAGTGTCTACCGGAAGGACGTCATTCGG
CACGCCGCTGTGCACAGCCGGGACCGGAAGAAGAGGCCAGATCCGACTCCAAAGCTGAGC
TCTTTCCCCTGCCCTGTGTGTGGCCGTGTGTACCCCATGCAGAAAAGACTCACGCAGCAC
ATGAAGACGCACAGCACTGAGAAGCCCCACATGTGTGACAAGTGTGGAAAGTCCTTTAAG
AAGCGCTACACCTTCAAAATGCACCTGCTCACGCACATCCAGGCTGTTGCCAACCGCAGG
TTCAAGTGTGAGTTCTGTGAGTTTGTTTGTGAAGACAAGAAGGCACTGCTGAACCACCAG
TTGTCCCACGTCAGTGACAAGCCCTTCAAATGCAGCTTTTGTCCCTACCGCACCTTCCGA
GAGGACTTCTTGCTGTCCCATGTGGCTGTCAAGCACACAGGGGCCAAGCCCTTCGCCTGT
GAGTACTGCCACTTCAGCACACGGCACAAGAAGAACCTGCGCCTGCACGTACGGTGCCGA
CACGCAAGCAGCTTCGAGGAATGGGGGAGGCGCCACCCTGAGGAGCCCCCCTCCCGCCGT
CGCCCCTTCTTCTCTCTGCAGCAGATTGAGGAGCTGAAGCAGCAGCACAGTGCGGCCCCT
GGACCACCTCCCAGTTCCCCAGGACCTGAGATACCCCCAGAGGCGACAACTTTCCAG
TCATCTGAGGCTCCCTCATTGCTCTGTTCTGACACCCTGGGCGGCGCCACCATCATCTAC
CAGCAAGGAGCTGAGGAGTCGACAGCGATGGCCACGCAGACAGCCTTGGATCTTCTGCTG
AACATGAGTGCTCAGCGGGAACTGGGGGGCACAGCCCTGCAGGTGGCTGTGGTGAAGTCG
GAAGATGTGGAAGCAGGGTTAGCATCCCCTGGTGGGCAGCCCTCCCCTGAAGGTGCCACT
CCACAGGTGGTCACCCTCCACGTGGCAGAGCCAGGGGGCGGTGCAGCAGCCGAGAGCCAG
CTAGGCCCTCCTGACCTACCGCAGATCACCCTGGCACCTGGTCCATTTGGTGGGACTGGC
TACAGTGTCATCACAGCACCTATGGAGGAGGGAACATCAGCTCCTGGCACACCTTAC
AGCGAGGAGCCCGCAGGAGAGGCAGCCCAGGCTGTGGTTGTGAGTGACACCCTAAAAGAA
GCTGGCACCCACTACATCATGGCTACTGATGGTACCCAGTTGCACCACATTGAGCTCACC
GCAGATGGCTCCATCTCCTTCCCACCAGATGCTCTGGCCTCTGGTGCCAAATGGCCC
CTGCTGCAGTGTGGGGGACTGCCCAGAGACGGCCCTGAGCCCCCATCTCCAGCCAAGACC
CACTGCGTAGGGGACTCCCAGAGCTCTGCCTCCTCACCTCCTGCAACCAGCAAAGCCCTG
GGCCTGGCAGTGCCCCCGTCACCGCCATCTGCAGCCACTGCTGCATCAAAGAAGTTTTCC
TGCAAGATCTGTGCCGAGGCCTTCCCTGGCCGAGCTGAGATGGAGAGTCACAAGCGGGCC
CACGCTGGGCCTGGTGCCTTCAAGTGCCCCGACTGCCCCTTCAGTGCCCGCCAGTGGCCC
GAGGTCCGGGCGCACATGGCACAGCACTCAAGCCTACGGCCCCACCAGTGTAGCCAGTGC
AGCTTTGCCTCCAAGAACAAGAAGGACCTGCGTCGGCACATGCTGACTCACACAAAGGAG
AAGCCTTTTGCATGCCACCTCTGCGGGCAGCGTTTCAACCGTAACGGGCACCTCAAGTTC
CACATCCAGCGGCTGCACAGTCCTGATGGGAGGAAGTCAGGAACCCCTACAGCCCGGGCC
CCTACCCAGACCCCAACCCAGACCATCATCCTGAACAGTGATGACGAAACACTGGCCACC
CTGCACACTGCACTCCAGTCCAGTCACGGGGTCCTGGGCCCAGAGCGGCTACAGCAGGCA
CTGAGCCAGGAACACATCATCGTTGCCCAGGAACAGACAGTGACCAATCAGGAGGAAGCC
GCCTACATCCAAGAGATCACCGCAGATGGCCAGACCGTACAGCACCTGGTGACCTCC
GACAACCAGGTGCAGTATATCATCTCCCAGGATGGTGTCCAGCACCTGCTCCCCCAGGAA
TATGTTGTGGTCCCTGAAGGCCATCACATCCAGGTACAGGAGGGCCAGATCACACACATC
CAGTATGAACAAGGAGCCCCGTTCCTTCAGGAGTCCCAGATCCAGTATGTGCCTGTGTCC
CCAGGCCAGCAGCTTGTCACACAGGCTCAACTTGAGGCTGCAGCACACTCAGCTGTCACA
GCAGTGGCTGATGCTGCCATGGCCCAAGCCCAGGGCCTGTTTGGTACAGACGAGACAGTG
CCCGAACACATTCAACAGCTGCAGCACCAGGGCATCGAGTACGACGTCATCACCCTGGCC
GAC

>Chimpanzee ENSPTRT00000025274.4
ATGGAGGAGAACGAGGTGG
AGAGCAGCAGCGACGCGGCCCCTGGGCCTGGCCGGCCCGAGGAGCCCTCTGAGAGCGGCCTGGGTGTGGG
CACCTCAGAAGCCGTGTCCGCCGACAGCAGCGACGCCGCGGCCGCCCCGGGGCAGGCAGAGGCCGATGAC
TCTGGCGTGGGGCAAAGCTCGGACCGCGGCAGCCGCTCTCAGGAGGAGGTATCTGAGAGCAGCTCGAGCG
CAGACCCCCTGCCTAATGGCTACCTCCCTGATTCATCGTCTGTGTCTCATGGGCCAGTGGCAGGGGTGAC
AGGCGGTCCCCCAGCACTTGTGCACTCTAGTGCACTCCCAGACCCCAACATGCTGGTGTCCGACTGCACA
GCTTCCTCCTCGGACCTGGGCTCGGCCATCGACAAGATCATCGAGTCCACCATCGGGCCCGACCTCATCC
AGAACTGCATCACTGTGACCAGTGCTGAGGATGGCGGGGCCGAGACCACACGGTACCTGATCCTGCAGGG
CCCAGATGATGGAGCCCCCATGACATCACCAATGTCCAGTTCCACCTTGGCCCACAGCCTAGCAGCCATT
GAGGCCCTGGCAGATGGCCCCACATCCACATCCACATGCCTGGAGGCACAGGGTGGGCCCAGCTCCCCGG
TGCAGCTGCCCCCAGCCTCCGGTGCCGAAGAACCGGACCTGCAGAGCCTGGAGGCCATGATGGAGGTGGT
GGTGGTGCAGCAGTTCAAATGCAAGATGTGCCAGTACCGGAGCAGCACCAAGGCCACACTGCTGCGCCAC
ATGCGGGAGCGCCACTTCCGTCCGGTAGCAGCAGCAGCTGGTAAAAAAGGACGTCTACGGA
AGTGGAGCACCTCCACCAAGACCCAAGAGGAAGAGGGACCAGAGGAGGAGGACGATGATGACATTGTAGA
CGCTGGAGCCATCGATGACCTGGAGGAGGATAGCGACTATAATCCAGCTGAGGATGAGCCCCGAGGCCGG
CAGCTTCGGCTCCAGCGCCCCACCCCCAGTACCCCAAGGCCCCGAAGGAGACCTGGCCGGCCCCGGAAGC
TGCCCCGCCTGGAGATATCAGACGATGGTGTGGAAGGAGAGCCTCTAGTGAGTTCCCAGAGTGG
ACAGAGCCCTCCAGAGCCACAGGATCCCGAGGCTCCCAGCTCCTCAGGCCCAGGACACCTGGTGGCCTTG
GGCAAGGTGAGCAGGACCCCTGTGGAAGCTGGTGTGAGCCAGTCAGATGCAGAGAACGCAGCCCCCTCCT
GCCCGGATGAGCACGACACTCTGCCCCGGCGCCGAGGTCGACCTTCCAGGCGCTTCCTAGGCAAGAAATA
CCGCAAGTACTATTACAAGTCGCCCAAACCACTTTTGAGGCCCTTCCTGTGCCGCATCTGTGGTTCTCGC
TTTCTGTCCCACGAGGACCTGCGCTTCCACGTCAACTCCCATGAGGCTGGCGATCCCCAGCTCTTCAAGT
GCCTGCAGTGCAGCTATCGTTCCCGCCGCTGGTCCTCGCTCAAGGAGCACATGTTCAACCACGTGGGCAG
CAAGCCCTACAAGTGTGACGAGTGCAGCTACACCAGTGTCTACCGGAAGGACGTCATTCGGCACGCCGCT
GTGCACAGCCGGGACCGGAAGAAGAGGCCAGATCCGACTCCAAAGCTGAGCTCTTTCCCCTGCCCTGTGT
GTGGCCGTGTGTACCCCATGCAGAAAAGACTCACGCAGCACATGAAGACGCACAGCACTGAGAAGCCCCA
CATGTGTGACAAGTGTGGAAAGTCCTTCAAGAAGCGCTACACCTTCAAAATGCACCTGCTCACGCACATC
CAGGCTGTTGCCAACCGCAGGTTCAAGTGTGAGTTCTGTGAGTTTGTTTGTGAAGACAAGAAGGCACTGC
TGAACCACCAGTTGTCCCACGTCAGTGACAAGCCCTTCAAATGCAGCTTTTGTCCCTACCGCACCTTCCG
AGAGGACTTCTTGCTGTCCCATGTGGCTGTCAAGCACACAGGGGCCAAGCCCTTCGCCTGTGAGTACTGC
CACTTCAGCACACGGCACAAGAAGAACCTGCGCCTGCACGTACGGTGCCGACACGCAAGCAGCTTCGAGG
AATGGGGGAGGCGCCACCCTGAGGAGCCCCCCTCCCGCCGTCGCCCCTTCTTCTCTCTGCAGCAGATTGA
GGAGCTGAAGCAGCAGCACAGTGCGGCCCCTGGACCACCTCCCAGTTCCCCAGGACCTGAGATACCC
CCAGAGGCGACAACTTTCCAGTCATCTGAGGCTCCCTCACTGCTCTGTCCTGACACCCTGGGTGGTGCCA
CCATCATCTACCAGCAAGGAGCTGAGGAGTCGACAGCGATGGCCACGCAGACAGCCTTGGATCTTCTGCT
GAACATGAGTGCCCAGCGGGAACTGGGGGGCACAGCCCTGCAGGTGGCCGTGGTGAAGTCGGAAGATGTG
GAAGCAGGGTTAGCATCCCCTGGTGGGCAGCCCTCCCCTGAAGGTGCCACTCCACAGGTGGTCACCCTCC
ACGTGGCAGAGCCAGGGGGCGGTGCAGTAGCCGAGAGCCAGCTAGGCCCTCCTGACCTACCGCAGATCAC
CCTGGCACCTGGTCCATTTGGTGGGACTGGCTACAGTGTCATCACAGCACCTATGGAGGAGGGGACA
TCAGCTCCTGGCACACCTTACAGCGAGGAGCCCGCAGGAGAGGCAGCCCAGGCTGTGGTTGTGAGTGACA
CCCTAAAAGAAGCTGGCACCCACTACATCATGGCTACTGATGGTACCCAGTTGCACCACATTGAGCTCAC
CGCAGATGGCTCCATCTCCTTCCCACCAGATGCTCTGGCCTCTGGTGCCAAATGGCCCCTGCTGCAG
TGTGGGGGGCTGCCCAGAGACGGCCCTGAGCCCCCATCTCCAGCCAAGACCCACTGCATAGGGGACTCCC
AGAGCTCTGCCTCCTCACCTCCTGCAACCAGCAAAGCCCTGGGCCTGGCAGTGCCCCCGTCACCACCATC
TGCAGCCACTGCTGCATCAAAGAAGTTTTCCTGCAAGATCTGTGCCGAGGCCTTCCCTGGCCGAGCTGAG
ATGGAGAGTCACAAGCGGGCCCACGCTGGGCCTGGTGCCTTCAAGTGCCCCGACTGCCCCTTCAGTGCCC

GCCAGTGGCCCGAGGTCCGGGCACACATGGCACAGCACTCAAGCCTACGGCCCCACCAGTGTAGCCAGTG
CAGCTTTGCCTCCAAGAACAAGAAAGACCTGCGTCGGCACATGCTGACTCACACAAAGGAGAAGCCTTTT
GCATGCCACCTCTGCGGGCAGCGTTTCAACCGTAACGGGCACCTCAAGTTCCACATCCAGCGGCTGCACA
GTCCTGATGGGAGGAAGTCAGGAACCCCTACAGCCCGGGCCCCTACCCAGACCCCAACCCAGACCATCAT
CCTGAACAGTGATGACGAAACACTGGCCACCCTGCACACTGCACTCCAGTCCAGTCACGGGGTCCTGGGC
CCAGAGCGGCTACAGCAGGCACTGAGCCAGGAACACATCATCGTTGCCCAGGAACAGACAGTGACCAATC
AGGAGGAAGCCGCCTACATCCAAGAGATCACCGCAGATGGCCAGACCGTACAGCACCTGGTGACCTC
CGACAACCAGGTGCAGTATATCATCTCCCAGGATGGCGTCCAGCACCTGCTCCCCCAGGAATATGTTGTG
GTCCCTGAAGGCCATCACATCCAGGTACAGGAGGGCCAGATCACACACATCCAGTATGAACAAGGAGCCC
CGTTCCTTCAGGAGTCCCAGATCCAGTATGTGCCTGTGTCCCCAGGCCAGCAGCTTGTCACACAGGCTCA
ACTTGAGGCTGCAGCACACTCAGCTGTCACAGCAGTGGCTGATGCTGCCATGGCCCAAGCCCAGGGCCTG
TTTGGCACAGAGGAGACAGTGCCCGAACACATTCAACAGCTGCAGCACCAGGGCATCGAGTACGACGTCA
TCACCCTGGCCGAC
>Bonobo   ENSPPAT00000062136.1
ATGGAGGAGAACGAGGTGGAGAGCAG
CAGCGACGCGGCCCCTGGGCCTGGCCGGCCCGAGGAGCCCTCTGAGAGCGGCCTGGGTGTGGGCACCTCA
GAAGCCGTGTCCGCCGACAGCAGCGACGCCGCGGCCGCCCCGGGGCAGGCAGAGGCCGATGACTCTGGCG
TGGGGCAAAGCTCGGACCGCGGCAGCCGCTCTCAGGAGGAGGTATCTGAGAGCAGCTCGAGCGCAGACCC
CCTGCCTAATGGCTACCTCCCTGATTCATCATCTGTGTCTCATGGGCCAGTGGCAGGGGTGACAGGCGGT
CCCCCAGCACTTGTGCACTCTACTGCACTCCCAGACCCCAACATGCTGGTGTCCGACTGCACAGCTTCCT
CCTCGGACCTGGGCTCGGCCATCGACAAGATCATCGAGTCCACCATCGGGCCCGACCTCATCCAGAACTG
CATCACTGTGACCAGTGCTGAGGATGGCGGGGCCGAGACCACACGGTACCTGATCCTGCAGGGCCCAGAT
GATGGAGCCCCCATGACATCACCAATGTCCAGTTCCACCTTGGCCCACAGCCTAGCAGCCATTGAGGCCC
TGGCAGATGGCCCCACATCCACATCCACATGCCTGGAGGCACAGGGTGGGCCCAGCTCCCCGGTGCAGCT
GCCCCCAGCCTCCGGTGCCGAAGAACCGGACCTGCAGAGCCTGGAGGCCATGATGGAGGTGGTGGTGGTG
CAGCAGTTCAAATGCAAGATGTGCCAGTACCGGAGCAGCACCAAGGCCACACTGCTGCGCCACATGCGGG
AGCGCCACTTCCGTCCGGTAGCAGCAGCAGCTGGTAAAAAAGGACGTCTACGGAAGTGGAG
CACCTCCACCAAGACCCAAGAGGAAGAGGGACCAGAGGAGGAGGACGATGATGACATTGTAGACGCTGGA
GCCATTGATGACCTGGAGGAGGATAGCGACTATAATCCAGCTGAGGATGAGCCCCGAGGCCGGCAGCTTC
GGCTCCAGCGCCCCACCCCCAGTACCCCAAGGCCCCGAAGGAGACCTGGCCGGCCCCGGAAGCTGCCCCG
CCTGGAGATCTCAGACGATGGTGTGGAAGGAGAGCCTCTAGTGAGTTCCCAGAGTGGACAGAGC
CCTCCAGAGCCACAGGATCCCGAGGCTCCCAGCTCCTCAGGCCCAGGACACCTGGTGGCCATGGGCAAGG
TGAGCAGGACCCCTGTGGAAGCTGGTGTGAGCCAGTCAGATGCAGAGAACGCAGCCCCCTCCTGCCCGGA
TGAGCACGACACTCTGCCCCGGCGCCGAGGTCGACCTTCCAGGCGCTTCCTAGGCAAGAAATACCGCAAG
TACTATTACAAGTCGCCCAAACCACTTTTGAGGCCCTTCCTGTGCCGCATCTGTGGTTCTCGCTTTCTGT
CCCACGAGGACCTGCGCTTCCACGTCAACTCCCATGAGGCTGGCGATCCCCAGCTCTTCAAGTGCCTGCA
GTGCAGCTATCGTTCCCGCCGCTGGTCCTCGCTCAAGGAGCACATGTTCAACCACGTGGGCAGCAAGCCC
TACAAGTGTGACGAGTGCAGCTACACCAGTGTCTACCGGAAGGACGTCATTCGGCACGCCGCTGTGCACA
GCCGGGACCGGAAGAAGAGGCCAGATCCGACTCCAAAGCTGAGCTCTTTCCCCTGCCCTGTGTGTGGCCG
TGTGTACCCCATGCAGAAAAGACTCACGCAGCACATGAAGACGCACAGCACTGAGAAGCCCCACATGTGT
GACAAGTGTGGAAAGTCCTTCAAGAAGCGCTACACCTTCAAAATGCACCTGCTCACGCACATCCAGGCTG
TTGCCAACCGCAGGTTCAAGTGTGAGTTCTGTGAGTTTGTTTGTGAAGACAAGAAGGCACTGCTGAACCA
CCAGTTGTCCCACGTCAGTGACAAGCCCTTCAAATGCAGCTTTTGTCCCTACCGCACCTTCCGAGAGGAC
TTCTTGCTGTCCCATGTGGCTGTCAAGCACACAGGGGCCAAGCCCTTCGCCTGTGAGTACTGCCACTTCA
GCACACGGCACAAGAAGAACCTGCGCCTGCACGTACGGTGCCGACACGCAAGTAGCTTCGAGGAATGGGG
GAGGCGCCACCCTGAGGAGCCCCCCTCCCGCCGTCGCCCCTTCTTCTCTCTGCAGCAGATTGAGGAGCTG
AAGCAGCAGCACAGTGCGGCCCCTGGACCACCTCCCAGTTCCCCAGGACCTGAGATACCTCCAGAGG

CGACAACTTTCCAGTCATCTGAGGCTCCCTCACTGCTCTGTCCTGACACCCTGGGCGGTGCCACCATCAT
CTACCAGCAAGGAGCTGAGGAGTCGACAGCGATGGCCACGCAGACAGCCTTGGATCTTCTGCTGAACATG
AGTGCTCAGCGGGAACTGGGGGGCACAGCCCTGCAGGTGGCCGTGGTGAAGTCAGAAGATGTGGAAGCAG
GGTTAGCATCCCCTGGTGGGCAGCCCTCCCCTGAAGGTGCCACTCCACAGGTGGTCACCCTCCACGTGGC
AGAGCCAGGGGGCGGTGCAGTAGCCGAGAGCCAGCTAGGCCCTCCTGACCTACCGCAGATCACCCTGGCA
CCTGGTCCATTTGGTGGGACTGGCTACAGTGTCATCACAGCACCTATGGAGGAGGGGACATCAGCTC
CTGGCACACCTTACAGCGAGGAGCCCGCAGGAGAGGCAGCCCAGGCTGTGGTTGTGAGTGACACCCTAAA
AGAAGCTGGCACCCACTACATCATGGCTACTGATGGTACCCAGTTGCACCACATTGAGCTCACCGCAGAT
GGCTCCATCTCCTTCCCACCAGATGCTCTGGCCTCTGGTGCCAAATGGCCCCTGCTGCAGTGTGGGG
GGCTGCCCAGAGACGGCCCTGAGCCCCCATCTCCAGCCAAGACCCACTGCACAGGGGACTCCCAGAGCTC
TGCCTCCTCACCTCCTGCAACCAGCAAAGCCCTGGGCCTGGCAGTGCCCCCGTCACCACCATCTGGAGCC
ACTGCTGCATCAAAGAAGTTTTCCTGCAAGATCTGTGCCGAGGCCTTCCCTGGCCGAGCTGAGATGGAGA
GTCACAAGCGGGCCCACGCTGGGCCTGGTGCCTTCAAGTGCCCCGACTGCCCCTTCAGTGCCCGCCAGTG
GCCCGAGGTCCGGGCGCACATGGCACAGCACTCAAGCCTACGGCCCCACCAGTGTAGCCAGTGCAGCTTT
GCCTCCAAGAACAAGAAGGACCTGCGTCGGCACATGCTGACTCACACAAAGGAGAAGCCTTTTGCATGCC
ACCTCTGCGGGCAGCGTTTCAACCGTAACGGGCACCTCAAGTTCCACATCCAGCGGCTGCACAGTCCTGA
TGGGAGGAAGTCAGGAACCCCTACAGCCCGGGCTCCTACCCAGACCCCAACCCAGACCATCATCCTGAAC
AGTGATGACGAAACACTGGCCACCCTGCACACTGCACTCCAGTCCAGTCACGGGGTCCTGGGCCCAGAGC
GGCTACAGCAGGCACTGAGCCAGGAACACATCATCGTTGCCCAGGAACAGACAGTGACCAATCAGGAGGA
AGCCGCCTACATCCAAGAGATCACCGCAGATGGCCAGACCGTACAGCACCTGGTGACCTCCGACAAC
CAGGTGCAGTATATCATCTCCCAGGATGGTGTCCAGCACCTGCTCCCCCAGGAATATGTTGTGGTCCCTG
AAGGCCATCACATCCAGGTACAGGAGGGCCAGATCACACACATCCAGTATGAACAAGGAGCCCCGTTCCT
TCAGGAGTCCCAGATCCAGTATGTGCCTGTGTCCCCAGGCCAGCAGCTTGTCACACAGGCTCAACTTGAG
GCTGCAGCACACTCAGCTGTCACAGCAGTGGCTGATGCTGCCATGGCCCAAGCCCAGGGCCTGTTTGGCA
CAGAGGAGACAGTGCCCGAACACATTCAACAACTGCAGCACCAGGGCATCGAGTACGACGTCATCACCCT
GGCCGAC
>gorilla XM_004062289.2
ATGGAGGAGAACGAGGT
GGAGAGCAGCAGCGACGCGGCCCCTGGGCCTGGCCGGCCCGAGGAGCCCTCTGAGAGCGGCCTGGGTGTG
GGCACCTCAGAAGCCGTGTCCGCCGACAGCAGCGACGCCGCGGCCGCCCCGGGGCAGGCAGAGGCCGATG
ACTCTGGCGTGGGGCAAAGCTCGGACCGCGGCAGCCGCTCTCAGGAGGAGGTATCTGAGAGCAGCTCGAG
CGCAGACCCCCGGCCTAATGGCTACCTCCCTGATTCATCGTCTGTGTCTCATGGGCCAGTGGCAGAGGTG
ACAGGCGGTCCCCCAGCACTTGTGCACTCTAGTGCACTCCCAGACCCCAACATGCTGGTGTCCGACTGCA
CAGCTTCCTCCTCGGACCTGGGCTCGGCCATCGACAAGATCATCGAGTCCACCATCGGGCCCGACCTCAT
CCAGAACTGCATCACTGTGACCAGTGCTGAGGATGGCGGGGCCGAGACCACACGGTACCTGATCCTGCAG
GGCCCAGATGATGGAGCCCCCATGACATCACCAATGTCCAGTTCCACCTTGGCCCACAGCCTAGCAGCCA
TTGAGGCCCTGGCAGATGGCCCCACATCCACATCCACATGCCTGGAGGCACAGGGTGGGCCCAGCTCCCC
GGTGCAGCTGCCCCCAGCCTCCGGTGCCGAAGAACCGGACCTGCAGAGCCTGGAGGCCATGATGGAGGTG
GTGGTGGTGCAGCAGTTCAAATGCAAGATGTGCCAGTACCGGAGCAGCACCAAGGCCACGCTGCTGCGCC
ACATGCGGGAGCGCCACTTCCGTCCAGTAGCAGCAGCAGCTGGTAAAAAAGGACGTCTACG
GAAGTGGAGCACCTCCACCAAGACCCAAGAGGAAGAGGGACCAGAGGAGGAGGACGATGATGACATTGTA
GACGCTGGAGCCATTGATGACCTGGAGGAGGATAGCGACTATAATCCAGCTGAGGATGAGCCCCGAGGCC
GGCAGCTTCGGCTCCAGCGCCCCACCCCCAGTACCCCAAGGCCCCGAAGGAGACCTGGCCGGCCCCGGAA
GCTGCCCCGCCTGGAGATCTCAGACGATGGTGTGGAAGGAGAGCCTCTAGTGAGTTCCCAGAGT
GGACAGAGCCCTCCAGAGCCACAGGATCCCGAGGCTCCCAGCTCCTCAGGCCCAGGACACCTGGTGGCCA
TGGGCAAGGTGAGCAGGACCCCTGTGGAAGCTGGTGTGAGCCAGTCAGATGCAGAGAACGCAGCCCCCTC
CTGCCCGGATGAGCATGACACTCTGCCCCGGCGCCGAGGTCGACCTTCCAGGCGCTTCCTAGGCAAGAAA

TACCGCAAGTACTATTACAAGTCTCCCAAACCGCTTTTGAGGCCCTTCCTGTGCCGCATCTGTGGTTCTC
GCTTTCTGTCCCACGAGGACCTGCGCTTCCACGTCAACTCCCATGAGGCTGGCGATCCCCAGCTCTTCAA
GTGCCTGCAGTGCAGCTATCGTTCCCGCCGCTGGTCCTCGCTCAAGGAGCACATGTTCAACCACGTGGGC
AGCAAGCCCTACAAGTGTGACGAGTGCAGCTACACCAGTGTCTACCGGAAGGACGTCATTCGGCACGCGG
CTGTGCACAGCCGGGACCGGAAGAAGAGGCCAGATCCGACTCCAAAGCTGAGCTCTTTCCCCTGCCCTGT
GTGTGGCCGTGTGTACCCCATGCAGAAAAGACTCACGCAGCACATGAAGACGCACAGCACTGAGAAGCCC
CACATGTGTGACAAGTGTGGAAAGTCCTTTAAGAAGCGCTACACCTTCAAAATGCACCTGCTCACGCACA
TCCAGGCTGTTGCCAACCGCAGGTTCAAGTGTGAGTTCTGTGAGTTTGTTTGTGAAGACAAGAAGGCACT
GCTGAACCACCAGTTGTCCCACGTCAGTGACAAGCCCTTCAAATGCAGCTTTTGTCCCTACCGCACCTTC
CGAGAGGACTTCTTGCTGTCCCATGTGGCTGTCAAGCACACAGGGGCCAAGCCCTTCGCCTGTGAGTACT
GCCACTTCAGCACACGGCACAAGAAGAACCTGCGCCTGCACGTACGGTGCCGACACGCAAGCAGCTTCGA
GGAATGGGGGAGGCGCCACCCTGAGGAGCCCCCCTCCCGCCGTCGCCCCTTCTTCTCTCTGCAGCAGATT
GAGGAGCTGAAGCAGCAGCACAGTGCAGCCCCTGGACCACCTCCCAGTTCCCCAGGACCTGAGATAC
CCCCAGAGGCGACAACTTTCCAGTCATCTGAGGCTCCCTCACTGCTCTGTCCTGACACCCTGGGCGGCGC
CACCATCATCTACCAGCAAGGAGCTGAGGAGTCGACAGCGATGGCCACGCAGACAGCCTTGGATCTTCTG
CTGAACATGAGTGCTCAGCGGGAACTGGGGGGCACAGCCCTGCAGGTGGCCGTGGTGAAGTCGGAAGATG
TGGAAGCAGGGTTAGCATCCCCTGGTGGGCAGCCCTCCCCTGAAGGTGCCACTCCACAGGTGGTCACCCT
CCACGTGGCAGAGCCAGGGGGTGGTGCAGCAGCCGAGAGCCAGCTAGGCCCTCCTGACCTACCGCAGATC
ACCCTGGCACCTGGTCCATTTGGTGGGACTGGCTACAGTGTCATCACAGCACCTATGGAGGAGGGGA
CATCAGCTCCTGGCACACCTTACAGCGAGGAGCCCCCAGGAGAGGCAGCCCAGGCTGTGGTTGTGAGTGA
CACCCTAAAAGAAGCTGGCACCCACTACATCATGGCTACTGATGGTACCCAGTTGCACCACATTGAGCTC
ACCGCAGATGGCTCCATCTCCTTCCCACCAGATGCTCTGGCCTCTGGTGCCAAATGGCCCCTGCTGC
AGTGTGGGGGGCTGCCCAGAGACGGCCCTGAGCCCCCATCTCCAGCCAAGACCCACTGCGTAGGGGACTC
CCAGAGCTCTGCCTCCTCACCTCCTGCAACCAGCAAAGCCCTGGGCCTGGCAGTGCCCCCGTCACCGCCA
TCTGCAGTCACTGCTGCATCAAAGAAGTTTTCCTGCAAGATCTGTGCCGAGGCCTTCCCTGGCCGAGCTG
AGATGGAGAGTCACAAGCGGGCCCACGCTGGGCCTGGTGCCTTCAAGTGCCCCGACTGCCCCTTCAGTGC
CCGCCAGTGGCCCGAGGTCCGGGCGCACATGGCACAGCACTCAAGCCTACGGCCCCACCAGTGTAGCCAG
TGCAGCTTTGCCTCCAAGAACAAGAAGGACCTGCGTCGGCACATGCTGACTCACACAAAGGAGAAGCCTT
TTGCATGCCACCTCTGCGGGCAGCGTTTCAACCGTAACGGGCACCTCAAGTTCCACATCCAGCGGCTGCA
CAGTCCTGATGGGAGGAAGTCAGGAACCCCTACAGCCCGGGCCCCTACCCAGACCCCAACCCAGACCATC
ATCCTGAACAGTGATGACGAAACACTGGCCACCCTGCACACTGCACTCCAATCCAGTCACGGGGTCCTGG
GCCCAGAGCGGCTACAGCAGGCACTGGGCCAGGAACACATCATCGTTGCCCAGGAACAGACAGTGACCAA
TCAGGAGGAAGCCGCCTACATCCAAGAGATCACCGCAGATGGCCAGACCGTACAGCACCTGGTGACC
TCCGACAACCAGGTGCAGTATATCATCTCCCAGGATGGTGTCCAGCACCTGCTCCCCCAGGAATATGTTG
TGGTTCCTGAAGGCCATCACATCCAGGTACAGGAGGGCCAGATCACACACATCCAGTATGAACAAGGAGC
CCCGTTCCTTCAGGAGTCCCAGATCCAGTATGTGCCTGTGTCCCCAGGCCAGCAGCTTGTCACACAGGCT
CAACTTGAGGCTGCAGCACACTCAGCTGTCACAGCAGTGGCTGATGCTGCCATGGCCCAAGCCCAGGGCC
TGTTTGGCACAGAGGAGACAGTGCCTGAACACATTCAACAGCTGCAGCACCAGGGCATCGAGTACGACGT
CATCACCCTGGCCGAC
>MARMOSET ENSCJAT00000033494
ATGGAGGAGAACGAGGTGGAGAGCAGCAGCGATGCGGCCCCTGGGCCTGGACGGCCCGAG
GAGCCCTCTGAGAGCGGCCTGGGTGTGGGCACCTCGGAAGCCGTGTCCGCCGACAGCAGC
GACGCCGCGGCTGCCCCGGGCCAGGCAGAGGCCGACGACTCTCGCGTGGGGCAAAGCTCG
GACAGTGGCAGCCGCTCTCAGGAGGAGGTGTCCGAGAGCAGCTCAAGCGCAGAACCCCTG
CCTAATGGCTACCTCCCTGATTCATCGTCTGTGTCCCGTGGGCCAGTGGCAGGGGTGACA
GGCGGTCCCCCAGCACTTGTGCACTCCAGTGTACTCCCAGACCCCAACATGCTGGTGTCT
GACTGTACAGCTTCCTCCTCGGACCTGGGCTCAGCCATCGACAAGATCATCGAATCCACC

ATTGGGCCCGACCTCATGCAGAGCTGCATTACTGTGACCAGTGCCGAGGATGGCGGGGCC
GAGACCACGAGGTACCTGATCCTGCAGGGCCCAGATGATGGAGCCCCCATGACATCACCA
ATGTCCAGTTCTACCTTGGCCCATAGCCTGGCAGCCATTGAGGCCCTGGCCGATGGCCCC
ACATCCACATCCACATGCCTGGAGCCACAGGGTGGTCCCAGCTCCCCG
GTGCAGCTGCTCCCAGCCTCTGGTGCTGAAGAACCAGACCTGCAGAGCCTGGAGGCCATG
ATGGAGGTGGTGGTGGTGCAGCAGTTCAAGTGCAAGATGTGCCAGTACCGGAGCAGCACC
AAGGCCACACTGCTGCGCCACATGCGGGAGCGCCACTTCCGTCCAGTAGCA
GCAGGAGTTGGTAAAAAAGGACGTCTACGGAAGTGGAGCACCTCCACCAAGACCCAAGAG
GAAGAGGGACCAGAGGAGGAGGACGATGATGACATTGTAGATGCAGGAGCCATTGATGAC
CTGGAGGAGGATAGCGACTATAATCCAGCTGAGGATGAACCCCGAGGCCGGCAGCTTCGG
CTCCAGCACCCCACCCCCAGTACCCCAAGGCCCCGAAGGAGACCTGGCCGGCCCCGGAAG
CTGCCCTGCCTAGAGATCTCAGACGATGGTGTGGAAGGAGAGCCTCTAGTGAGT
TCCCAGAGTGGCCAGAGCCCTCCAGAGCCACAGGATCCCGAGGCTCCCAGCTCCTCAGGC
CCGGGACACCTGGTGGCCATAGGCAAGGCAAGCAGGACCCCTGTGGAAGCGGGTGTGAGC
CAGTCAGATGCAGAGAATGCAGCCCCCTCCTGCCCAGATGAGCATGACACTCCGCCCCGG
CGCCGAGGTCGACCTTCCAGGCGCTTCCTAGGCAAGAAATACCGCAAGTACTATTACAAG
TCACCCAAACCACTTTTGAGGCCCTTCCTTTGCCGCATCTGTGGCTCCCGCTTTCTGTCC
CATGAGGACCTGCGCTTCCATGTCAATTCCCACGAGGCCGGTGATCCCCAGCTCTTCAAG
TGCCTGCAGTGCAGCTATCGCTCCCGTCGCTGGTCCTCTCTCAAGGAGCACATGTTCAAC
CATGTCGGCAGCAAGCCCTACAAGTGTGACGAGTGCAGCTATACCAGTGTCTACCGGAAG
GACGTCATCAGGCACGCAGCTGTGCACAGCCGGGACCGGAAGAAGAGACCAGATCCGACT
CCAAAGCTGAGTTCTTTTCCCTGCCCTGTGTGTGGCCGTGTGTACCCCATGCAGAAAAGA
CTCACGCAGCACATGAAGACACACAGCACTGAGAAGCCCCACATGTGTGACAAGTGTGGA
AAGTCCTTTAAGAAGCGCTACACCTTCAAAATGCACCTGCTTACGCACATCCAGGCTGTC
GCCAACCGCAGGTTCAAGTGTGAGTTCTGCGAGTTCGTTTGTGAAGATAAGAAGGCACTG
CTGAACCACCAGCTGTCCCACGTCAGTGACAAGCCCTTCAAATGCAGCTTTTGTCCCTAC
CGCACCTTCCGAGAAGACTTCCTGCTGTCCCATGTGGCTGTCAAGCATACAGGGGCCAAG
CCCTTCGCCTGTGAGTACTGCCACTTCAGCACACGGCACAAGAAGAATCTCCGCCTGCAC
GTACGGTGTCGACACGCAAGCAGCTTTGAGGAATGGGGGCGGCGCCACCCTGAGGAGCCC
CCTTCCCGCCGTCGCCCCTTCTTCTCCCTGCAGCAGATTGAGGAGCTGAAGCAGCAGCAC
AGTGCGGCCCCCGAACCACCTCCCAGCTCCCCAGGACCTGAGATACCCCCAGAGGCGACA
CCTTTCCAGTCATCTGAGGCTCCCTCACTGCTCTGTCCTGACACCCTGGGTGGCGCCACC
ATCATCTACCAGCAAGGAGCCGAGGAGTCGACCGCGATGGCCACGCAGACAGCCTTGGAT
CTTCTGCTGAACATGAGTGCTCAGCGGGAACTGGGGGGCACAGCCCTGCAGGTGGCCGTG
GTGAAGTCGGAGGACATGGAAGCAGGGTTACCATCCCCTGGTGGGCAGCCCTCCCCTGAA
GGTACCACTCCACAGGTGGTCACCCTCCATGTGGCAGAGCCGGGGGGTGATACAGCAGCC
GAGAGCCAGCTAGGCCCTCCTGACCTACCACAGATCACCTTGGCATCTGGTCCATTTAGT
GGGACTGGCTACAGCGTTATCACAGCACCAATGGAGGAGGGGACGTCAGCTCCTGGC
ACACCTTACAGCGAGGAGCCCCCAGGGGAGGCAGCCCAGGCTGTGGTTGTGACCGACCCC
CTAAAAGAAGCTGGCACCCACTACATCATGGCTGCTGATGGTACACAGTTGCACCACATC
GAGCTCTCCGCAGATGGCTCCATCTCCTTCCCACCAGATGCCCTGACCTCTGGTGCC
AAATGGCCCCTACTGCAGTGTGGGGGGCTGCCCAGAGATGGCCCTGAGCCCCCATCTCCA
GCCAAGACCCACCGGATAGGGGACCCCCAGAGCTCTGTCTCCCCACCTCCTGCAGCCAGC
AAAGCCCTGGGCCCAGCAGTGCCCCCCTCACCACCATCCACAGGCACTGCAGCATCAAAG
AAGTTTTCCTGCAAGATCTGTGCTGAGGCCTTCCCTGGCAGAGCTGAGATGGAGAGTCAC
AAGCGGGCCCACGCTGGGCCTGGTGCCTTCAAGTGCCCCGACTGCCCCTTCAGTGCCCGC
CAGTGGCCTGAGGTCCGGGCACACATGGCGCAGCACTCAAGCCTGCGGCCCCACCAGTGT

AGCCAATGCAGCTTTGCCTCCAAGAACAAGAAGGACCTGCGGCGGCACATGCTGACTCAC
ACCAAGGAGAAGCCTTTTGCGTGCCACCTCTGCGGGCAGCGTTTCAACCGTAATGGGCAC
CTCAAGTTCCACATCCAGCGACTGCACAGTCCTGAGGGGAGGAAGTCAGGAACTCCTACA
GCCCGGGCCCCTACCCAGACCCCAACCCAGACCATCATTCTCAACAGTGATGATGAAACA
CTGGCCACCCTGCACACTGCACTCCAGTCCAGTCACGGGGTCCTGGGCCCAGAGCGGCTG
CAGCAGGCACTGGGCCAGGAACACATCATTGTCGCCCAGGAACAGACAGTGACCAATCAG
GAGGAAGCCACCTACATCCAAGAGATCACCGCAGATGGCCAGACTGTACAGCACCTG
GTAACCTCCGACAACCAGGTACAATACATCATCTCCCAGGATGGTGTCCAGCACCTGCTC
CCCCAGGAATATGTTGTGGTCCCCGAGGGCCATCACATCCAGGTACAGGAGGGCCAGATC
ACACACATCCAGTATGAACAAGGAGCCCCGTTCCTTCAGGAGTCCCAGATCCAGTATGTG
CCTGTGTCCCCAGGCCAGCAGCTAGTCACACAGGCTCAACTTGAGGCTGCAGCACACTCA
GCTGTCACAGCAGTGGCTGATGCTGCCATGGCTCAAGCCCAGGGCCTGTTTGGCACAGAG
GAGGCAGTGCCTGAACACATTCAGCAGCTGCAGCACCAGGGCATCGAATACGACGTCATC
ACCCTGGCCGAC
>OLIVE BABOON ENSPANT00000007256
ATGGAGGAGAACGAGGTGGAGAGCAGCAGCGACGCGGCCCCTGGGCCTGGCCGGCCCGAG
GAGCCCTCTGAGAGCGGCCTGGATGTGGGCACCTCGGAAGCCGTGTCGGCCGACAGCAGC
GACGCCGCGGCCGCCCCGGGGCAGGCAGAGGCCGATGACTCTGGCGTGGGGCAAAGCTCG
GACCGCGGCAGCCGCTCTCAGGAGGAGGTATCCGAGAGCAGCTCGAGCGCAGACCCCCTG
CCTAATGGCTACCTCCCTGATTCATCATCTGTGTCCCATGGGCCAGTGGCAGGGGTGACA
GGCGGTCCCCCAGCACTTGTGCACTCTAGTGCACTCCCAGACCCCAACATGCTGGTATCC
GACTGCACAGCTTCCTCCTCGGACCTGGGCTCAGCCATCGACAAGATCATCGAGTCCACC
ATCGGGCCCGACCTCATCCAGAGCTGCATCACTGTGACCAGTGCCGAGGATGGCGGGGCC
GAGACCACGAGGTACCTGATCCTGCAGGGCCCAGATGATGGAGCCCCCATGACATCACCA
ATGTCCAGTTCCACCTTGGCCCACAGCCTGGCAGCCATTGAGGCCCTGGCAGATGGCCCC
ACATCCACATCCACATGCCTGGAGCCACAGGGTGGGCCCAGCTCCCCG
GTGCAGCTGCCTCCAGCCTCTGGTGCTGAAGAACCAGACCTGCAGAGCCTGGAGGCCATG
ATGGAGGTGGTGGTGGTGCAGCAGTTCAAGTGCAAGATGTGCCAGTACCGGAGCAGCACC
AAGGCCACACTGCTGCGCCACATGCGGGAGCGCCACTTCCGTCCAGTAGCA
GCAGCATCTGGTAAAAAAGGACGTCTACGGAAGTGGAGCACCTCCACCAAGACCCAAGAG
GAAGAGGGACCGGAGGAGGAGGACGATGACGACATTGTAGACGCTGGAGCCATTGATGAC
CTGGAGGAGGATAGCGACTATAATCCAGCTGAGGATGAGCCCCGAGGCCGGCAGCTTCGG
CTCCAGCGCCCCACCCCCAGTACCCCAAGGCCCCGAAGGAGACCTGGCCGGCCCCGGAAG
CTGCCCCGCCTGGAGATCTCAGACGATGGTGTGGAAGGAGAGCCTCTAGTGAGT
TCCCAGAGTGGACAGAGCCCTCCAGAGCCACAGGATCCCGAGGCTCCCAGCTCCTCAGGC
CCAGGACACCTGGTGGCCATGGGCAAGGCAAGCAGGACCCCTGTGGAAGCTGGTGTGAGC
CAGTCAGATGCAGAGAACGCAGCTCCCTCCTGCCCGGATGAGCATGACACTCCACCCCGG
CGCCGAGGTCGACCTTCCAGGCGCTTCCTAGGCAAGAAATACCGCAAGTACTATTACAAG
TCGCCCAAACCGCTTTTGAGGCCCTTCCTGTGCCGCATCTGTGGTTCTCGCTTTCTGTCC
CACGAGGACCTGCGCTTCCACGTCAACTCCCATGAGGCTGGCGATCCCCAGCTCTTCAAG
TGCCTGCAGTGCAGCTATCGTTCCCGCCGCTGGTCCTCTCTCAAGGAGCACATGTTCAAC
CACGTGGGCAGCAAGCCCTACAAGTGTGATGAGTGCAGCTACACCAGTGTCTACCGGAAG
GACGTCATTCGGCACGCCGCTGTGCACAGCCGAGACCGGAAGAAGAGGCCAGATCCGACT
CCAAAGCTGAGCTCTTTCCCCTGCCCTGTGTGTGGCCGTGTGTACCCCATGCAGAAAAGA
CTCACGCAGCACATGAAGACGCACAGCACTGAGAAGCCCCATATGTGTGACAAGTGTGGA
AAGTCCTTTAAGAAGCGCTACACCTTCAAAATGCACCTGCTCACACACATCCAGGCTGTT
GCCAACCGCAGGTTCAAGTGTGAGTTCTGTGAGTTCCTTTGTGAAGACAAGAAGGCACTG

CTGAACCACCAGTTGTCCCACGTCAGTGACAAGCCCTTCAAATGCAGCTTTTGTCCCTAC
CGCACCTTCCGAGAGGACTTCCTGCTGTCCCATGTGGCTGTTAAGCACACAGGGGCCAAG
CCCTTCGCCTGTGAGTACTGCCACTTCAGCACACGGCACAAGAAGAACCTACGCCTGCAC
GTACGGTGCCGACATGCAAGCAGCTTCGAGGAATGGGGGAGGCGCCACCCTGAGGAGCCC
CCCTCCCGCCGTCGCCCCTTCTTCTCTCTGCAGCAGATTGAGGAGCTGAAGCAACAGCAC
AGTGTGGCCCCTGGACCACCTCCCAGCTCCCCAGGACCTGAGATACCCCCAGAGGCG
ACACCTTTCCAGTCATCTGAGGCTCCCTCACTGCTCTGTCCTGACACCCTGGGCGGCGCC
ACCATCATCTACCAGCAAGGAGCCGAGGAGTCGACAGCGATGGCCACGCAGACAGCCTTG
GATCTTCTGCTGAACATGAGTGCCCAGCGGGAACTGGGGGGCACAGCCCTGCAGGTGGCC
GTGGTGAAGTCGGAGGACGTGGAAGCAGGGTTAGCATCCCCTGGTGGGCAGCCCTCCCCT
GAAGGTGCCACTCCACAGGTGGTCACCCTCCATGTGGCAGAGCCGGGGGGCGGTGCGGCA
GCCGAGAGCCAGCTAGGCCCTCCTGACCTACCACAGATCACCCTGGCACCTGGTCCATTT
GGTGGGACTGGCTACAGTGTCATCACGGCACCCATGGAGGAGGGGACATCAGCTCCTGGC
ACACCTTACAGCGAGGAACCCTCAGGAGAGGCAGCCCAGACTGTGGTTGTGAGTGACACC
CTAAAAGAAGCTGGCACCCACTACATCATGGCTACTGATGGTACCCAGTTGCACCACATT
GAGCTCACCGCAGATGGCTCCATCTCCTTCCCACCAGATGCTCTGGCCTCTGGTGCC
AAATGGCCCCTGCTGCAGTGTGGGGGGCTGCCTAGAGATGGCCCTGAGCCTCCATCTCCA
GCCAACACCCACCGTGTAGGGGACCCCCCGAGCTCTGCCTCCCCACCTCCTGCAACCAGC
AAAGCCCTGGGCCTGGCAGTGCCCCCCTCACCGCCGTGTGCAGCCACTGCAGCATCAAAG
AAGTTTTCCTGCAAGATCTGTGCCGAGGCCTTCCCTGGCCGAGCTGAGATGGAGAGTCAC
AAGCGGGCCCATGCTGGGCCTGGTGCCTTCAAGTGCCCTGACTGCCCCTTCAGTGCCCGC
CAGTGGCCTGAGGTCCGGGCGCACATGGCGCAGCACTCAAGCCTGCGGCCCCACCAGTGT
AGCCAGTGCAGTTTCGCCTCCAAGAATAAGAAGGACCTGCGGCGGCACATGCTGACTCAC
ACCAAGGAGAAGCCTTTTGCATGCCACCTCTGCGGGCAGCGTTTCAACCGTAACGGGCAC
CTCAAGTTCCATATCCAGCGGCTGCACAGTCCTGATGGGAGGAAGTCAGGAACCCCTACA
GCCCGGGCTGCTACCCAGACCCCAACCCAGACCATCATCCTGAACAGTGATGACGAAACA
CTGGCCACCCTGCACACTGCACTCCAGTCCAGTCACGGGGTCCTGGGCCCAGAGCGGCTA
CAGCAGGCACTGGGCCAGGAACATATCATTGTTGCCCAGGAGCAGACAGTGACTAATCAG
GAGGAAGCCACCTACATCCAAGAGATCACCGCAGATGGCCAGACCGTACAGCACCTG
GTGACCTCCGACAACCAGGTGCAGTATATCATCTCCCAGGATGGTGTCCAGCACCTGCTC
CCCCAGGAATATGTTGTGGTCCCCGAGGGCCATCACATCCAGGTACAGGAGGGCCAGATC
ACACACATCCAGTATGAACAAGGAGCCCCGTTCCTTCAGGAGTCCCAGATCCAGTATGTG
CCTGTGTCCCCAGGCCAGCAGCTTGTCACACAGGCTCAACTTGAGGCTGCAGCACACTCG
GCTGTAACAGCAGTGGCTGATGCTGCCATGGCCCAAGCCCAAGGCCTGTTTGGCACAGAG
GAGGCAGTGCCTGAACACATTCAACAGCTGCAGCACCAGGGCATCGAGTACGACGTCATC
ACCCTGGCCGAC
>ORANGUTAN ENSPPYT00000012868
ATGGAGGAGAACGAAGTGGAGAGCAGCAGCGACGCGGCTCCTGGGCCTGGCCGGCCCGAG
GAGCCCTCTGAGAGCGGCCTGGGTGTGGGCACCTCAGAAGCCGTGTCCGCCGACAGCAGC
GACGCCGCGGCCGCCCCGGGGCGGGCAGAGGCCGATGACTCTGGCGTGGGGCAAAGCTCG
GACCGCGGCAGCCGCTCTCAGGAGGAGGTATCCGAGAGCAGCTCGAGCGCAGACCCCCTG
CCTAATGGCTACCTCCCTGATTCATCGTCTGTGTCCCATGGGCCAGTGGCAGGGGTGACA
GGTGGTCCCCCAGCACTTGTGCACTCTAGTGCACTCCCAGACCCCAACATGCTGGTGTCC
GACTGCACAGCTTCCTCCTCGGACCTGGGCTCGGCCATCGACAAGATCATCGAGTCCACC
ATCGGGCCCGACCTCATCCAGAGCTGCATCACTGTGACCAGTGCCGAGGATGGCGGGGCC
GAGACCACACGGTACCTGATCCTGCAGGGCCCAGATGATGGAGCCCCGATGACATCACCA
ATGTCCAGTTCCACCTTGGCCCACAGCCTAGCAGCCATTGAGGCCCTGGCAGATGGCCCC

ACATCCACATCCACATGCCTGGAGCCACAGGGTCGGCCCAGCTCC
CCAGTGCAGCTGCCCCCAGCCTCTGGTGCCGAAGAACCAGACCTGCAGAGCCTGGAGGCC
ATGATGGAGGTGGTGGTGGTGCAGCAGTTCAAGTGCAAGATGTGCCAGTACCGGAGCAGC
ACCAAGGCCACACTGCTGCGCCACATGCGGGAGCGCCACTTCCGTCCAGTAGCAGCAGCA
GCTGGTAAAAAAGGACGTCTACGGAAGTGGAGCACCTCCACCAAGACCCAA
GAGGAAGAAGGACAAGAGGAGGAGGACGATGACGACATTGTAGACGCTGGAGCCATTGAT
GACCTGGAGGAGGATAGTGACTATAATCCAGCTGAGGATGAGCCCCGAGGCCGGCAGCTT
CGGCTCCAGCGCCCCACCCCCAGTACCCCAAGGCCCCGAAGGAGACCTGGCCGGCCCCGG
AAACTGCCCCGCCTGGAGATCTCAGACGATGGTGTGGAAGGAGAGCCTCTAGTG
AGTTCCCAGAGTGGACAGAGCCCTCCAGAGCCACAGGATCCCGAGGCTCCCAGCTCCTCA
GGCCCAGGACACCTGGTGGCCATGGGCAAGGTGAGCAGGACGCCTGTGGAAGCTGGTGTG
AGCCAGTCAGATGCAGAGAACGCAGCCCCCTCCTGCCTGGATGAGCATGACACTCCGCCC
CGGCGCCGAGGTCGACCTTCCAGGCGCTTCCTAGGCAAGAAATACCGCAAGTACTATTAC
AAGTCGCCCAAACCGCTTTTGAGGCCCTTCCTGTGCCGCATCTGTGGTTCTCGCTTTCTG
TCCCACGAGGACCTGCGCTTCCACGTCAACTCCCATGAGGCTGGCGATCCCCAGCTCTTC
AAGTGCCTGCAGTGCAGCTATCGTTCCCGCCGCTGGTCCTCGCTCAAGGAGCACATGTTC
AACCACGTGGGCAGCAAACCCTACAAGTGTGACGAGTGCAGCTACACCAGTGTCTACCGG
AAGGACGTCATTCGGCATGCCGCTGTGCACAGCCGGGACCGGAAGAAGAGGCCAGATCCG
ACTCCAAAGCTGAGCTCTTTCCCCTGCCCTGTGTGTGGCCGTGTGTACCCCATGCAGAAA
AGACTCACGCAGCACATGAAGACGCACAGCACTGAGAAGCCCCACATGTGTGACAAGTGT
GGAAAGTCCTTTAAGAAGCGCTACACCTTCAAAATGCACCTGCTCACGCACATCCAGGCT
GTTGCCAACCGCAGGTTCAAGTGTGAGTTCTGTGAGTTCGTTTGTGAAGACAAGAAGGCA
CTGCTGAACCACCAGTTGTCCCACGTCAGTGACAAGCCCTTCAAATGCAGCTTTTGTCCC
TATCGCACCTTCCGAGAGGACTTCCTGCTGTCCCATGTGGCTGTCAAGCATACAGGGGCC
AAGCCCTTCGCCTGTGAGTACTGCCACTTCAGCACACGGCACAAGAAGAACCTACGCCTG
CACGTACGGTGCCGACACGCAAGCAGCTTCGAGGAATGGGGGAGGCGCCACCCTGAGGAG
CCCCCCTCCCGCCGTCGCCCCTTCTTCTCTCTGCAGCAGATTGAGGAGCTGAAGCAGCAG
CACAGTGCGGCCCCTGGACCACCTCCCAGCTCCCCAGGACCTGAGATACCCCCAGAG
GCGACACCTTTCCAGTCATCTGAGGCTCCTTCACTGCTCTGTCCTGACACCCTGGGTGGC
GCCACCATCATCTACCAGCAAGGAGCTGAGGAGTCGACAGCAATGGCCACGCAGACAGCC
TTGGATCTTCTGCTGAACATGAGTGCTCAGCGGGAACTGGGGGGCACAGCCCTGCAGGTG
GCTGTGGTGAAGTCGGAGGACGTGGAAGCAGGGTTAGCATCCCCTGGTGGGCAGCCCTCC
CCTGAAGGTGCCACTCCACAGGTGGTCACCCTCCACGTGGCAGAGCCGGGGGGCGGTGCA
GCAGCCGAGAGCCAGCTAGGCCCTCCTGACCTACCGCAGATCACCCTGGCACCTGGTCCA
TTTGGTGGGACTGGCTACAGTGTCATCACAGCACCTATGGAGGAGGGTACATCAGCT
CCTGGCACACCTTACAGCGAGGAGCCCCCAGGAGAGGCAGCCCAGGCTGTGGTTGTGAGT
GACACCCTAAAAGAAGCTGGCACCCACTACATTATGGCTACTGATGGTACCCAGTTGCAC
CACATTGAGCTCACCGCAGATGGCTCCATCTCCTTCCCACCAGATGCTCTGGCCTCT
GGTGCCAAATGGCCCCTGCTGCAGTGTGGGGGGCTGCCCAGAGACGGCCCTGAGCCCCCA
TCTCCAGCCAAGACCCACTGCATAGGGGACTCCCAGAGCTCTGCCTCCCCACCTCCTGCA
ACCAGCAAAGCCCTGGGCCTGGCAGTGCCCCCGTCACCGCCATCTGCAGCCACTGCTGCA
TCAAAGAAGTTTTCCTGCAAGATCTGTGCGGAGGCCTTCCCTGGCCGAGCTGAGATGGAG
AGTCACAAGCGGGCCCATGCTGGGCCTGGTGCCTTCAAGTGCCCTGACTGCCCCTTCAGT
GCCTGCCAGTGGCCCGAGGTCCGGGCACACATGGCACAGCACTCAAGCCTGCGGCCCCAC
CAGTGTAGCCAGTGCAGCTTTGCCTCCAAGAACAAGAAGGACCTGCGTCGACACATGCTG
ACTCACACCAAGGAGAAGCCTTTTGCATGCCACCTCTGCGGGCAGCGTTTCAACCGTAAC
GGGCACCTCAAGTTCCACATCCAGCGGCTGCACAGTCCTGATGGGAGGAAGTCAGGAACC

CCTACAGCCCGGGCCCCTACCCAGACCCCAACCCAGACCATCATCCTGAACAGTGATGAC
GAAACACTGGCCACCCTGCACACTGCACTCCAGTCCAGTCACGGGGTCCTGGTCCCAGAG
CGGCTACAGCAGGCACTGGGCCAGGAACACATCATTGTTGCCCAGGAACAGACAGTGACC
AATCAGGAGGAAGCCACCTACATCCAAGAGATCACCGCAGATGGCCAGACCGTACAG
CACCTGGTGACCTCCGACAACCAGGTGCAGTATATCATCTCCCAGGATGGTGTCCAGCAT
CTGCTCCCCCAGGAATATGTTGTGGTCCCTGAGGGCCATCACATCCAGGTACAGGAGGGC
CAGATCACACACATCCAGTATGAACAAGGAGCCCCGTTCCTTCAGGAGTCCCAGATCCAG
TATGTGCCTGTGTCCCCAGGCCAGCAGCTTGTCACACAGGCTCAACTTGAGGCTGCAGCA
CACTCAGCTGTCACAGCAGTGGCTGATGCTGCCATGGCCCAAGCCCAGGGCCTGTTTGGC
ACAGAGGAGGCAGTGCCTGAACACATTCAACAGCTGCAGCACCAGGGCATCGAGTACGAC
GTCATCACCCTGGCCGAC
>Cercocebus atys XM_012065869.1
ATGGAGGAGAACGAG
GTGGAGAGCAGCAGCGACGCGGCCCCTGGGCCTGGCCGGCCCGAGGAGCCCTCTGAGAGCGGCCTGGATG
TGGGCACCTCGGAAGCCGTGTCGGCCGACAGCAGCGACGCCGCGGCCGCCCCAGGGCAGGCAGAGGCCGA
TGACTCTGGCGTGGGGCAAAGCTCGGACCGCGGTAGCCGCTCTCAGGAGGAGGTATCCGAGAGCAGTTCG
AGCGCAGACCCCTTGCCTAATGGCTACCTCCCTGATTCATCATCTGTGTCCCATGGGCCAGTGGCAGGGG
TGACAGGCGGTCCCCCAGCACTTGTGCACTCTAGTGCACTCCCAGACCCCAACACGCTGGTGTCCGACTG
CACAGCTTCCTCCTCGGACCTGGGCTCAGCCATCGACAAGATCATCGAGTCCACCATCGGGCCCGACCTC
ATCCAGAGCTGCATCACTGTGACCAGTGCCGAGGATGGCGGGGCCGAGACCACGAGGTACCTGATCCTGC
AGGGCCCAGATGATGGAGCCCCCATGACATCACCAATGTCCAGTTCCACCTTGGCCCACAGCCTGGCAGC
CATTGAGGCCCTGGCAGATGGCCCCACATCCACATCCACATGCCTGGAGCCACAGGGT
GGGCCCAGCTCCCCGGTGCAGCTGCCTCCAGCCTCTGGTGCTGAAGAACCAGACCTGCAGAGCCTGGAGG
CCATGATGGAGGTGGTGGTGGTGCAGCAGTTCAAGTGTAAGATGTGCCAGTACCGGAGCAGCACCAAGGC
CACACTGCTGCGCCACATGCGGGAGCGCCACTTCCGTCCAGTAGCAGCAGCATCTGGTAAA
AAAGGACGTCTACGGAAGTGGAGCTCCTCCACCAAGACCCAAGAGGAAGAGGGACCGGAGGAGGAGGATG
ATGACGACATTGTAGACGCTGGAGCCATTGATGACCTGGAGGAGGATAGCGACTATAATCCAGCTGAGGA
TGAGCCCCGAGGCCGGCAGCTTCGGCTCCAGCGCCCCACCCCCAGTACCCCAAGGCCCCGAAGGAGACCT
GGCCGACCCCGGAAGCTGCCCCGCCTGGAGATCTCAGACGATGGTGTGGAAGGAGAGCCTCTAG
TGAGTTCCCAGAGTGGACAGAGCCCTCCAGAGCCACAGGATCCCGAGGCTCCCAGCTCCTCAGGCCCAGG
ACACCTGGTGGCCATGGGCAAGGCAAGCAGGACCCCTGTGGAAGCTGGTGTGAGCCAGTCAGATGCAGAG
AACGCAGCTCCCTCCTGCCCGGATGAGCATGACACTCCACCCCGGCGCCGAGGTCGACCTTCCAGGCGCT
TCCTAGGCAAGAAATACCGCAAGTACTATTACAAGTCGCCCAAGCCGCTTTTGAGGCCCTTCCTGTGCCG
CATCTGTGGTTCTCGCTTTCTGTCCCACGAGGACCTGCGCTTCCATGTCAACTCCCATGAGGCTGGCGAT
CCCCAGCTCTTCAAGTGCCTGCAGTGCAGCTATCGTTCCCGCCGCTGGTCCTCTCTCAAGGAGCACATGT
TCAACCACGTGGGCAGCAAGCCCTACAAGTGTGACGAGTGCAGCTACACCAGTGTCTACCGGAAGGACGT
CATTCGGCACGCCGCTGTGCACAGCCGAGACCGGAAGAAGAGGCCAGATCCGACTCCAAAGCTGAGCTCT
TTCCCCTGCCCTGTGTGTGGCCGTGTGTACCCCATGCAGAAAAGACTCACGCAGCACATGAAGACGCACA
GCACTGAGAAGCCCCATATGTGTGACAAGTGTGGAAAGTCCTTTAAGAAGCGCTACACCTTCAAAATGCA
CCTGCTCACACACATCCAGGCTGTTGCCAACCGCAGGTTCAAGTGTGAGTTCTGTGAGTTCCTTTGTGAA
GACAAGAAGGCACTGCTGAACCACCAGTTGTCCCACGTCAGTGACAAGCCCTTCAAATGCAGCTTTTGTC
CCTACCGCACTTTCCGAGAGGACTTCCTGCTGTCCCATGTGGCTGTTAAGCACACAGGGGCCAAGCCCTT
CGCCTGTGAGTACTGCCACTTCAGCACACGGCACAAGAAGAACCTACGCCTGCACGTACGGTGCCGACAT
GCAAGCAGCTTCGAGGAATGGGGGAGGCGCCACCCTGAGGAGCCCCCCTCCCGCCGTCGCCCCTTCTTCT
CTCTGCAACAGATTGAGGAGCTGAAGCAGCAGCACAGTGTGGCCCCTGGACCACCTCCCAGCTCCCCAGG
ACCTGAGATACCCCCAGAGGCGACACCTTTCCAGTCATCTGAGGCTCCCTCACTGCTCTGTCCTGAC
ACCCTGGGCGGCGCCACCATCATCTACCAGCAAGGAGCCGAGGAGTCGACAGCGATGGCCACGCAGACAG

CCTTGGATCTTCTGCTGAACATGAGTGCCCAGCGGGAACTGGGGGGCACAGCCCTGCAGGTGGCCGTGGT
GAAGTCGGAGGATGTGGAAGCAGGGTTAGCATCCCCTGGTGGGCAGCCCTCCCCTGAAGGTGCCACTCCA
CAGGTGGTCACCCTCCACGTGGCAGAGCCGGGGGGCGGTGCGGCAGCCGAGAGCCAGCTAGGCCCTCCTG
ACCTACCACAGATCACCCTGGCACCTGGTCCATTTGGTGGGACTGGCTACAGTGTCATCACGGCACC
TATGGAGGAGGGGACATCAGCTCCTGGCACACCTTACAGCGAGGAACCCTCAGGAGAGGCAGCCCAGACT
GTGGTTGTGAGTGACACCCTAAAAGAAGCTGGCACCCACTACATCATGGCTACTGATGGTACCCAGTTGC
ACCACATTGAGCTCACCGCAGATGGCTCCATCTCCTTCCCACCAGATGCTCTGGCCTCTGGTGCCAA
ATGGCCCCTGCTGCAGTGTGGGGGGCTGCCTAGAGATGGCCCTGAGCCTCCATCTCCAGCCAACACCCAC
CGTGTAGGGGACCCCCCGAGCTCTGCCTCCCCACCTCCTGCAACCAGCAAAGCCCTGGGCCTGGCAGTGC
CCCCCTCACCGCCGTGTGCAGCCACTGCAGCATCAAAGAAGTTTTCCTGCAAGATCTGTGCCGAGGCCTT
CCCTGGCCGAGCTGAGATGGAGAGTCACAAGCGGGCCCATGCTGGGCCTGGTGCCTTCAAGTGCCCCGAC
TGCCCCTTCAGTGCCCGCCAGTGGCCCGAGGTCCGGGCGCACATGGCGCAGCACTCAAGCCTGCGGCCCC
ACCAGTGTAGCCAGTGCAGTTTCGCCTCCAAGAATAAGAAGGACCTGCGGCGGCACATGCTGACTCACAC
CAAGGAGAAGCCTTTTGCGTGCCACCTCTGCGGGCAGCGTTTCAACCGTAACGGGCACCTCAAGTTCCAT
ATCCAGCGGCTGCACAGTCCTGATGGGAGGAAGTCAGGAACCCCTACAGCCCGGGCTGCTACCCAGACCC
CAACCCAGACCATCATCCTGAACAGTGATGACGAAACACTGGCCACCTTGCACACTGCACTCCAGTCCAG
TCACGGGGTCCTGGGCCCAGAGCGGCTACAGCAGGCGCTGGGCCAGGAACATATCATTGTTGCCCAGGAG
CAGACAGTGACCAATCAGGAGGAAGCCACCTACATCCAAGAGATCACCGCAGATGGCCAGACCGTAC
AGCACCTGGTGACCTCCGACAACCAGGTGCAGTATATCATCTCCCAGGATGGTGTCCAGCACCTGCTCCC
CCAGGAATATGTTGTGGTCCCCGAGGGCCATCACATCCAGGTACAGGAGGGCCAGATCACACACATCCAG
TATGAACAAGGAGCCCCGTTCCTTCAGGAGTCCCAGATCCAGTATGTGCCTGTGTCCCCAGGCCAGCAGC
TTGTCACACAGGCTCAACTTGAGGCTGCAGCACACTCGGCTGTCACAGCAGTGGCTGATGCTGCCATGGC
CCAAGCCCAAGGCCTGTTTGGCACAGAGGAGGCAGTGCCTGAACACATTCAACAGCTGCAGCACCAGGGC
ATCGAGTACGACGTCATCACCCTGGCCGAC
>Mandrillus leucophaeus XM_011983775.1
ATGGAGGAGAACGAGG
TGGAGAGCAGCAGCGACGCGGCCCCTGGGCCTGGCCGGCCCGAGGAGCCCTCTGAGAGCGGCCTGGATGT
GGGCACCTCGGAAGCTGTGTCGGCCGACAGCAGCGACGCCGCGGCCGCCCCGGGGCAGGCAGAGGCCGAT
GACTCTGGCGTGGGGCAAAGCTCGGACCGCGGCAGCCGCTCTCAGGAGGAGGTATCCGAGAGCAGCTCGA
GCGCAGACCCCCCTGCCTAATGGCTACCTCCCTGATTCATCATCTGTGTCCCATGGCCAGTGGCAGGGGT
GACAGGCGGTCCCCCAGCACTTGTGCACTCTAGTGCACTCCCAGACCCCAACATGCTGGTGTCCGACTGC
ACAGCTTCCTCCTCGGACCTGGGCTCAGCCATCGACAAGATCATCGAGTCCACCATCGGGCCCGACCTCA
TCCAGAGCTGCATCACTGTGACCAGTGCCGAGGATGGCGGGGCTGAGACCACGAGGTACCTGATCCTGCA
GGGCCCAGATGATGGAGCCCCCATGACATCACCAATGTCCAGTTCCACCTTGGCCCACAGCCTGGCAGCC
ATTGAGGCCCTGGCAGATGGCCCCACATCCACATCCACATGCCTGGAGCCACAGGGTG
GGCCCAGCTCCCCGGTGCAGCTGCCTCCAGCCTCTGGTGCTGAAGAACCAGACCTGCAGAGCCTGGAGGC
CATGATGGAGGTGGTGGTGGTGCAGCAGTTCAAGTGCAAGATGTGCCAGTACCGGAGCAGCACCAAGGCC
ACACTGCTGCGCCACATGCGGGAGCGCCACTTCCGTCCAGTAGCAGCAGCATCTGGTAAAA
GAGGACGTCTACGGAAGTGGAGCTCCTCCACCAAGACCCAAGAGGAAGAGGGACCGGAGGAGGAGGACGA
TGACGACATTGTAGACGCTGGAGCCATTGATGACCTGGAGGAGGATAGCGACTATAATCCAGCTGAGGAT
GAGCCCCGAGGCCGGCAGCTTCGGCTCCAGCGCCCCACCCCCAGTACCCCAAGGCCCCGAAGGAGACCTG
GCCGGCCCCGGAAGCTGCCCCGCCTGGAGATCTCAGACGATGGTGTGGAAGGAGAGCCTCTAGT
GAGTTCCCAGAGTGGACAGAGCCCTCCAGAGCCACAGGATCCCGAGGCTCCCAGCTCCTCAGGCCCAGGA
CACCTGGTGGCCATGGGCAAGGCAAGCAGGACCCCTGTGGAAGCTGGTGTGAGCCAGTCAGATGCAGAGA
ACGCAGCTCCCTCCTGCCCGGACGAGCATGACACTCCACCCCGGCGCCGAGGTCGACCTTCCAGGCGCTT
CCTAGGCAAGAAATACCGCAAGTACTATTACAAGTCGCCCAAACCGCTTTTGAGGCCCTTCCTGTGCCGC
ATCTGTGGTTCTCGCTTTCTGTCCCACGAGGACCTGCGCTTCCACGTCAACTCCCATGAGGCTGGCGATC

CCCAGCTCTTCAAGTGCCTGCAGTGCAGCTATCGTTCCCGCCGCTGGTCCTCTCTCAAGGAGCACATGTT
CAACCACGTGGGCAGCAAACCCTACAAGTGTGACGAGTGCAGCTACACCAGTGTCTACCGGAAGGACGTC
ATTCGGCACGCCGCTGTGCACAGCCGAGACCGGAAGAAGAGGCCAGATCCGACTCCAAAGCTGAGCTCTT
TCCCCTGCCCTGTGTGTGGCCGTGTGTACCCCATGCAGAAAAGACTCACGCAGCACATGAAGACGCACAG
CACTGAGAAGCCCCATATGTGTGACAAATGTGGAAAGTCCTTTAAGAAGCGCTACACCTTCAAAATGCAC
CTGCTCACACACATCCAGGCTGTTGCCAACCGCAGGTTCAAGTGTGAGTTCTGTGAGTTCCTTTGTGAAG
ACAAGAAGGCACTGCTGAACCACCAGTTGTCCCACGTCAGTGACAAGCCCTTCAAATGCAGCTTTTGTCC
CTACCGCACCTTCCGAGAGGACTTCCTGCTGTCCCATGTGGCTGTTAAGCACACAGGGGCCAAGCCCTTT
GCCTGTGAGTACTGCCACTTCAGCACACGGCACAAGAAGAACCTACGCCTGCACGTACGGTGCCGACATG
CAAGCAGCTTCGAGGAATGGGGGAGGCGCCACCCTGAGGAGCCCCCCTCCCGCCGTCGCCCCTTCTTCTC
TCTGCAGCAGATTGAGGAGCTGAAGCAGCAGCACAGTGTGGCCCCCTGGACCACCTCCCAGCTCCCCAGGA
CCTGAGATACCCCCAGAGGCGACACCTTTCCAGTCATCTGAGGCTCCCTCACTGCTCTGTCCTGACA
CCCTGGGCGGCGCCACCATCATCTACCAGCAAGGAGCCGAGGAGTCGACAGCGATGGCCACGCAGACAGC
CTTGGATCTTCTGCTGAACATGAGTGCCCAGCGGGAACTGGGGGGCACAGCCCTGCAGGTGGCCGTGGTG
AAGTCGGAGGACGTGGAAGCAGGGTTAGCATCCCCTGGTGGGCAGCCCTCCCCTGAAGGTGCCACTCCAC
AGGTGGTCACCCTCCATGTGGCAGAGCCGGGGGGCGGTGCGGCAGCCGAGAGCCAGCTAGGCCCTCCTGA
CCTACCACAGATCACCCTGGCACCTGGTCCATTTGGTGGGACTGGCTACAGTGTCATCACGGCACCT
ATGGAGGAGGGGACATCAGCTCCTGGCACACCTTACAGCGAGGAACCCTCAGGAGAGGCAGCCCAGACTG
TGGTTGTGAGTGACACCCTAAAAGAAGCTGGCACCCACTACATCATGGCTACTGATGGTACCCAGTTGCA
CCACATTGAGCTCACTGCAGATGGCTCCATCTCCTTCCCACCAGATGCTCTGGCCTCTGGTGCCAAA
TGGCCCCTGCTGCAGTGTGGGGGGCTGCCTAGAGATGGTCCTGAGCCTCCATCTCCAGCCAACACCCACC
GTGTAGGGGACCCCCCGAGCTCTGCCTCCCCACCTCCTGCAACCAGCAAAGCCCTGGGCCTGGCAGTGCC
CCCCTCACCGCCGTGTGCAGCCACTGCAGCATCAAAGAAGTTTTCCTGCAAGATCTGTGCCGAGGCCTTC
CCTGGCCGAGCTGAGATGGAGAGTCACAAGCGGGCCCATGCTGGGCCTGGTGCCTTCAAGTGCCCCGACT
GCCCCTTCAGTGCCCGCCAGTGGCCCGAGGTCCGGGCGCACATGGCACAGCACTCAAGCCTGCGGCCCCA
CCAGTGTAGCCAGTGCAGTTTCGCCTCCAAGAATAAGAAGGACCTGCGGCGGCACATGCTGACTCACACC
AAGGAGAAGCCTTTTGCGTGCCACCTCTGCGGGCAGCGTTTCAACCGTAACGGGCACCTCAAGTTCCATA
TCCAGCGGCTGCACAGTCCTGATGGGAGGAAGTCAGGAACCCCTACAGCCCGGGCTGCTACCCAGACCCC
AACCCAGACCATCATCCTGAACAGTGATGACGAAACACTGGCCACCCTGCACACTGCACTCCAGTCCAGT
CACGGGGTCCTGGGCCCAGAGCGGCTACAGCAGGCACTGGGCCAGGAACATATCATTGTTGCCCAGGAGC
AGACAGTGACCAATCAGGAGGAAGCCACCTACATCCAAGAGATCACCGCAGATGGCCAGACCGTACA
GCACCTGGTGACCTCCGACAACCAGGTGCAGTATATCATCTCCCAGGATGGTGTCCAGCACCTGCTCCCC
CAGGAATATGTTGTGGTCCCCGAGGGCCATCACATCCAGGTACAGGAGGGCCAGATCACACACATCCAGT
ATGAACAAGGAGCCCCGTTCCTTCAGGAGTCCCAGATCCAGTATGTGCCTATGTCCCCAGGCCAGCAGCT
TGTCACACAGGCTCAACTTGAGGCTGCAGCACACTCGGCTGTCACAGCAGTGGCTGATGCTGCCATGGCC
CAAGCCCAAGGCCTGTTTGGCACAGAGGAGGCAGTGCCTGAACACATTCAACAGCTGCAGCACCAGGGCA
TCGAGTACGACGTCATCACCCTGGCCGAC
>Chlorocebus sabaeus XM_008015516.1
ATGGAGGAGAACGAGGTGGAGAGCAGCAGCGACGCGG
CCCCTGGGCCTGGCCGGCCCGAGGAGCCCTCTGAGAGCGGCCTGGGTGTGGGCACCTCGGAAGCCGTGTC
GGCCGACAGCAGCGACGCCGCGGCCGCCCCGGGGCAGGCAGAGGCCGATGACTCTGGCGTGGGGCAAAGC
TCGGACCGCGGCAGCGGCTCTCAGGAGGAGGTATCCGAGAGCAGCTCGAGCGCAGACCCCCCTGCCTAATG
GCTACCTCCCTGATTCATCGTCTGTGTCCCATGGGCCAGTGGCAGGGGTGACAGGCGGTCCCCCAGCACT
TGTGCACTCTAGTGCACTCCCAGACCCCAACATGCTGGTGTCCGACTGCACAGCTTCCTCCTCGGACCTG
GGCTCAGCCATCGACAAGATCATCGAGTCCACCATCGGGCCCGACCTCATCCAGAGCTGCATCACTGTGA
CCAGTGCCGAGGATGGCGGGGCCGAGACCACGAGGTACCTGATCCTGCAGGGCCCAGATGATGGAGCCCC
CATGACATCACCAATGTCCAGTTCCACCTTGGCCCACAGCCTGGCAGCCATTGAGGCCCTGGCAGATGGC

CCCACATCCACATCCACATGCCTGGAGCCACAGGGTGGGCCCAGCTCCCCGGTGCAGC
TGCCTCCAGCCTCTGGTGCTGAAGAACCAGACCTGCAGAGCCTGGAGGCCATGATGGAGGTGGTGGT
GCAGCAGTTCAAGTGCAAGATGTGCCAGTACCGGAGCAGCACCAAGGCCACACTACTGCGCCACATGCGG
GAGCGCCACTTCCGTCCAGCAGCAGCAGCATCTGGTAAAAAAGGACGTCTACGGAAGTGGAGCA
CCTCCACCAAGACCCAAGAGGAAGAGGGACCGGAGGAGGAGGACGATGACGACATTGTAGACGCTGGAGC
CATTGATGACCTGGAGGAGGATAGCGACTATAATCCAGCTGAGGATGAGCCCCGAGGCCGGCAGCTTCGG
CTCCAGCGCCCCACCCCCAGTACCCCAAGGCCCCGAAGGAGACCTGGCCGGCCCCGGAAGCTGCCCCGCC
TGGAGATCTCAGACGATGGTGTGGAAGGAGAGCCTCTAGTGAGTTCCCAGAGTGGACAGAGCCC
TCCAGAGCCACAGGATCCTGAGGCTCCCAGCTCCTCAGGCCCAGGACACCTGGTGGCCATGGGCAAGGCA
AACAGGACCCCTGTGGAAGCTGGTGTGAGCCAGTCAGATGCAGAGAACGCAGCTCCCTCCTGCCCGGATG
AGCATGACACTCCACCCCGGCGCCGAGGTCGACCTTCCAGGCGCTTCCTAGGCAAGAAATACCGCAAGTA
CTATTACAAGTCGCCCAAACCGCTTTTGAGGCCCTTCCTGTGCCGCATCTGTGGTTCTCGCTTTCTGTCC
CACGAGGACCTGCGCTTCCACGTCAACTCCCATGAGGCTGGCGATCCCCAGCTCTTCAAGTGCCTGCAGT
GCAGCTATCGTTCCCGCCGCTGGTCCTCTCTCAAGGAGCACATGTTCAACCACGTGGGCAGCAAGCCCTA
CAAGTGTGACGAGTGCAGCTACACCAGTGTCTACCGGAAGGACGTCATTCGGCACGCCGCTGTGCACAGC
CGAGACCGGAAGAAGAGGCCAGATCCGACTCCAAAGCTGAGCTCTTTCCCCTGCCCTGTGTGTGGCCGTG
TGTACCCCATGCAGAAAAGACTTACGCAGCACATGAAGACGCACAGCACTGAGAAGCCCCACATGTGTGA
CAAGTGTGGAAAGTCCTTTAAGAAGCGCTACACCTTCAAAATGCACCTGCTCACACACATCCAGGCTGTT
GCCAACCGCAGGTTCAAGTGTGAGTTCTGTGAGTTCGTTTGTGAAGACAAGAAGGCACTGCTGAACCACC
AGTTGTCCCACGTCAGTGACAAGCCCTTCAAATGCAGCTTTTGTCCCTACCGCACCTTCCGAGAGGACTT
CCTGCTGTCCCATGTGGCTGTCAAGCACACAGGGGCCAAGCCCTTTGCCTGTGAGTACTGCCACTTCAGC
ACACGGCACAAGAAAAACCTACGCCTGCACGTACGGTGCCGACATGCAAGCAGCTTCGAGGAATGGGGGA
GGCGCCACCCTGAGGAGCCCCCCTCCCGCCGTCGCCCCTTCTTCTCTCTGCAGCAGATTGAGGAGCTGAA
GCAGCAGCACAGTGTGGCCCCTGGACCACCTCCCAGCTCCCCAGGACCTGAGATACCCCCAGAGGCG
ACACCTTTCCAGTCATCTGAGGCTCCCTCACTGCTCTGTCCTGACACCCTGGGCGGCGCCACCATCATCT
ACCAGCAAGGAGCCGAGGAGTCGACAGCGATGGCCACGCAGACAGCCTTGGATCTTCTACTGAACATGAG
TGCCCAGCGGGAACTGGGGGGCACAGCCCTGCAGGTGGCTGTGGTGAAGTCGGAGGACGTGGAAGCAGGG
TTAGCATCCCCTGGTGGGCAGCCCTCCCCTGAAGGTGCCACTCCACAGGTGGTCACCCTCCATGTGGCAG
AGCCAGGGGGCGGTGTGGCAGCCGAGAGCCAGCTAGGCCCTCCTGACCTACCACAGATCACCCTGGCACC
TGGTCCATTTGGTGGGACTGGCTACAGTGTCATCACGGCACCTATGGAGGAGGGAACATCAGCTCCT
GGCACACCTTACAGCGAGGAGCCCTCAGGAGAGGCAGCCCAGACTGTGGTTGTGAGTGACACCCTAAAAG
AAGCTGGCACCCACTACATCATGGCTACTGATGGTACCCAGTTGCACCACATTGAGCTCACCGCAGATGG
CTCCATCTCCTTCCCACCAGATGCTCTGGCCTCTGGTGCCAAATGGCCCCTGCTGCAGTGTGGGGGG
CTGCCGAGAGATGGCCCTGAGCCTCCATCTCCAGCCAGCACCCACCGTGTAGGGGACCCCCCGAGCTCTG
CCTCCCCACCTCCTGCAACCAGCAAAGCCCTGGGCCTGGCAGTGCCCCCCTCACCGCCGTGTGCAGCCAC
TGCAGCATCAAAGAAGTTTTCCTGCAAGATCTGTGCCGAGGCCTTCCCTGGCCGAGCTGAGATGGAGAGT
CACAAGCGGGCCCATGCTGGGCCTGGTGCCTTCAAGTGCCCCGACTGCCCCTTCAGTGCCCGCCAGTGGC
CCGAGGTCCGGGCGCACATGGCGCAGCACTCAAGCCTGCGGCCCCACCAGTGTAGCCAGTGCAGTTTCGC
CTCCAAGAATAAGAAGGACCTGCGGCGGCACATGCTGACTCACACCAAGGAGAAGCCGTTTGCATGCCAC
CTCTGCGGGCAGCGTTTCAACCGTAACGGGCACCTCAAGTTCCATATCCAGCGGCTGCACAGTCCCGATG
GGAGGAAGTCAGGAACCCCTACAGCCCGGGCTGCTACCCAGACCCCAACCCAGACCATCATCCTCAACAG
TGATGACGAAACACTGGCCACCCTGCACACCGCACTCCAGTCCAGTCACGGGGTCCTGGGCCCAGAGCGG
CTACAGCAGGCACTGGGCCAGGAACATATCATCGTTGCCCAGGAGCAGACAGTGACCAATCAGGAGGAAG
CCACCTACATCCAAGAGATCACCGCAGATGGCCAGACTGTACAGCACCTGGTGACCTCCGACAACCA
GGTGCAGTATATCATCTCCCAGGATGGTGTCCAGCACCTGCTCCCCCAGGAATATGTTGTGGTCCCCGAG
GGCCATCACATCCAGGTACAGGAGGGCCAGATCACACACATCCAGTATGAACAAGGAGCCCCGTTCCTTC
AGGAGTCCCAGATCCAGTATGTGCCTGTGTCCCCAGGCCAGCAGCTTGTCACACAGGCTCAACTTGAGGC

TGCAGCACACTCGGCTGTCACAGCAGTGGCTGATGCTGCCATGGCCCAAGCCCAAGGCCTGTTTGGCACA
GAGGAGGCAGTGCCTGAACACATTCAACAGCTGCAGCACCAGGGCATCGAGTACGACGTCATCACCCTGG
CCGAC
>Saimiri boliviensis boliviensis XM_003936468.2
ATGGAGGAGAACGAGGTGGAAAGCAGCAGCGACGCGG
CCCCTGGGCCTGGCCGGCCCGAGGAGCCCTCTGAGAGCGGCCTGGGTGTGGGCACCTCGGAAGCCGTGTC
CGCCGACAGCAGCGACGCCGCGGCTGCCCCGGGCCAGGCAGAGGCCGAAGACTCTCGAGTGGGGCAAAGC
TCGGACAGTGGCAGCCGCTCTCAGGAGGAGGTGTCCGGGAGCAGCTCAAGTGCAGACCCCCTGCCTAATG
GTTACCTCCCTGATTCATCGTCTGTGTCCCACGGACCAGTGGCAGGGGTGACAGGCGGTCCCCCAGCACT
TGCGCACTCCAGTGTACTCCCAGACCCCAACATGCTGGTGTCTGACTGCACAGCTTCCTCCTCGGACCTG
GGCTCAGCCATCGACAAGATCATCGAGTCCACCATTGGGCCCGACCTCATGCAGAGCTGTATTACTGTGA
CCAGTGCTGAGGATGGCGGGGCCGAGACCACGCGGTACCTGATCCTGCAGGGCCCAGATGATGGAGCCCC
CATGACATCACCAATGTCCAGTTCCACCTTGGCCCATAGCCTGGCAGCCATTGAGGCCCTGGCCGATGGC
CCCACATCCACATCCACCTGCCTGGAGCCACAGGGTGGGCCCAGCTCCCCGGTGCAGC
TGCTCCCAGCCTCTGGTGCCGAAGAACCGGACCTGCAGAGCCTGGAGGCCATGATGGAGGTGGTGGTGGT
GCAGCAGTTCAAGTGCAAGATGTGCCAGTACCGGAGCAGCACCAAGGCCACGCTGCTGCGCCACATGCGG
GAGCGCCACTTCCGTCCAGTAGCAGCAGGAGTCGGTAAAAAAGGACGTCTACGGAAGTGGA
GCCCCTCCGCCAAGACGCAGCAGGAAGAGGGACCAGAGGAGGAGGAGGATGACGACATTGTAGATGCCGG
AGCCATAGACGACCTGGAGGAGGATAGCGACTATAATCCAGCTGAGGATGAACCCCGAGGCCGGCAGCTT
CGGCTCCAGCGCCCCACCCCCAGTACCCCAAGGCCCCGAAGGAGACCCGGCCGGCCCCGGAAGCTGCCCC
GCCTGGAGATCTCAGACGATGGTGTGGAAGGAGAGCCTCTCGTGAGTTCCCAGAGTGGCCAGAA
CCCTCCAGAGCCACAGGATCCCGAGGCTCCCAGCTCCTCAGGCCCGGGACACCTGGTGGCCATGGGCAAG
GCAAGCAGGACCCCTGTGGAAGCGGGTGTGAGCCAGTCAGACGCAGAGAACGCAGCCCCCTCCCGCCCAG
ATGAGCAGGACACGCCGCCCCGGCGCCGCGGTCGGCCTTCCAGGCGCTTCCTAGGCAAGAAATACCGCAA
GTACTATTACAAGTCACCCAAACCACTTTTGAGGCCCTTCCTGTGCCGCATCTGTGGCTCTCGCTTTCTG
TCCCATGAGGACCTGCGCTTCCACGTCAACTCCCACGAGGCCGGCGATCCCCAGCTCTTCAAGTGCCTGC
AGTGCAGCTATCGCTCTCGTCGCTGGTCCTCGCTCAAGGAGCACATGTTCAACCATGTGGGCAGCAAGCC
CTACAAGTGTGATGAGTGCAGCTACACCAGTGTCTACCGGAAGGACGTCATCAGGCATGCAGCTGTGCAC
AGCCGGGACCGGAAGAAGAGGCCAGATCCGACTCCAAAGCTGAGCTCTTTTCCCTGCCCTGTGTGTGGCC
GTGTGTACCCCATGCAGAAAAGACTCACGCAGCACATGAAGACACACAGCACTGAGAAGCCCCACATGTG
TGACAAGTGTGGAAAGTCCTTTAAGAAGCGCTACACCTTCAAAATGCACTTGCTTACGCACATCCAGGCT
GTCGCCAACCGCAGGTTCAAGTGTGAGTTCTGCGAGTTTGTTTGTGAAGATAAGAAGGCACTGCTGAACC
ACCAGCTGTCCCACGTCAGTGACAAGCCGTTCAAATGCAGCTTTTGTCCCTACCGCACCTTCCGAGAGGA
CTTCTTGCTGTCCCATGTGGCTGTCAAGCACACAGGGGCCAAGCCCTTCGCCTGCGAGTACTGCCACTTC
AGCACACGGCACAAGAAGAATCTCCGCCTGCACGTACGGTGTCGACACGCAAGCAGCTTCGAGGAATGGG
GGCGGCGCCACCCTGAGGAGCCCCCCTCCCGCCGTCGCCCCTTCTTCTCCCTGCAGCAGATTGAGGAGCT
GAAGCAGCAGCACAGTGCGGCCCCCGAACCACCTCCCAGCTCCCCAGGACCTGAGATACCCGCAGAGGCG
ACACCTTTCCAGTCATCTGAGGCTCCCTCACTGCTCTGTCCTGACACCCTGGGTGGCGCCACCATCATCT
ACCAGCAAGGAGCCGAGGAGTCAACCGCGATGGCCACGCAGACAGCCTTGGATCTTCTGCTGAACATGAG
TGCTCAGCGGGAACTGGGGGGCACAGCCCTGCAGGTGGCCGTGGTGAAGTCGGAGGACATGGAAGCGGGG
TTACCATCCCCTGGTGGGCAGCCCTCCCCTGAAGGTACCACTCCACAGGTGGTCACCCTCCATGTGGCAG
AGCCGGGGGGTGGTACAGCAGCCGAGAGCCAGCTAGGCCCTCCTGACCTACCACAGATCACCTTGGCATC
TGGTCCATTTGGTGGGACTGGCTACAGCGTTATCACAGCACCTATGGAGGAGGGGACGTCAGCTCCT
GGCACACCTTACAGCGAGGAGCCCCCAGGAGAGGCAGCCCAGGCTGTGGTTGTGACTGACCCCCTAAAAG
AAGCTGGCACCCACTACATCATGGCTGCTGATGGTACCCAGTTGCACCATGTCGAGCTCTCCGCAGATGG
CTCCATCTCCTTCCCACCAGATGCCCTGGCCTCTGGTGCCAAATGGCCCCTACTGCAGTGTGGAGGG
CTGCCCAGAGATGGCCCTGAGCCCCCATCTCCAGCCAAGACCCACCGGATAGGGGACCCCCAGAGCTCTG

CCTCCCCACCTCCTGCCACCAGCAAAGCCTTGGGCCCAGCAGTGCCCACCTCACCACCATCCGCAGGCAC
TGCAGCATCAAAGAAGTTTTCCTGCAAGATCTGTGCTGAGGCCTTCCCTGGCAGAGCTGAGATGGAGAGT
CACAAGCGGGCCCACGCTGGGCCTGGTGCCTTCAAGTGCCCCGACTGCCCCTTCAGTGCTGGCCAGTGGC
CTGAGGTCCGGGCACACATGGCGCAGCACTCAAGCCTGCGGCCCCACCAGTGTAGCCAGTGCAGCTTTGC
CTCCAAGAACAAGAAGGACCTGCGGCGGCACATGCTGACTCACACCAAGGAGAAGCCTTTTGCGTGCCAC
CTCTGTGGGCAGCGTTTCAACCGTAACGGACACCTGAAGTTCCACATCCAGCGACTGCACAGTCCTGATG
GGAGGAAGTCAGGAACCCCTACATCCCGGGCCGCTACCCAGACCCCAGCCCAGACCATCATTCTCAACAG
TGATGATGAAACACTGGCCACCCTGCACACTGCACTCCAGTCCAGTCACGGGGTCCTGGGCCCAGAGCGG
CTGCAGCAGGCACTGGGCCAGGAGCACATCATTGTCGCCCAGGAACAGACAGTGACCAATCAAGAGGAAG
CCACCTACATCCAAGAGATCACCGCAGATGGCCAGACCGTACAGCACCTGGTAACCTCTGACAACCA
GGTGCAATACATCATCTCCCAGGACGGTGTCCAGCACCTGCTCCCCCAGGAGTACGTTGTGGTCCCTGAG
GGCCATCACATCCAGGTACAGGAGGGCCAGATCACACACATCCAGTATGAACAAGGAGCCCCGTTCCTTC
AGGAGTCCCAGATCCAGTATGTGCCTGTGTCGCCAGGCCAGCAGCTAGTCACACAGGCTCAACTTGAGGC
CGCAGCACACTCAGCTGTCACAGCAGTGGCTGATGCTGCCATGGCTCAAGCCCAGGGCCTGTTTGGCACA
GAGGAGGCAGTGCCTGAACACATTCAGCAGCTGCAGCACCAGGGCATCGAATACGACGTCATCACCCTGG
CCGAC
>Carlito syrichta XM_008057914.2
ATGGAGGAGAACGAAGTGGAGAGCAGTAGCGACGCAG
CCCCCCGGCCCGGCCGGCCCGAGGAGCCCTCTGAGAGTGGCCTGTGTGTGGGCACCTCGGAAGCCGTGTC
CGCCGACAGCAGCGACGCCGCTGCCGGCCCAGGGCAGGCAGAGGCCGACGACTCTGGCGTGGGGCAAAGC
TCGGAAAGTGGCAGCCACTCTCAGGAGGAGGTGTCTGAGAGCAGCTCGAGCACGGACCCCCTGCCTCATG
GCTACCTCCCAGATTCATCGTCTGTGTCCCGTGGGCCCATGGTGGGGGTGACAGGTGGCCCCCCAGCACT
TGTGCACTCCAGCGCACTCCCAGACGCCAACATGCTGGTGTCTGACTGCACGGCTTCCTCCTCGGATCTG
GGCTCGGCCATCGACAAGATCATCGAGTCTACCATTGGACCGGACCTCATCCAGAGCTGTATCACTGTGA
CCAGTGCTGAGGATGGTGGAGCTGAGACCACACGGTACCTGATCCTGCAAGGCCCTGATGATGGAGCCCC
CATGACATCACCAATGTCCAGTTCCACCCTGGCCCATAGCCTGGCAGCCATCGAAGCCCTGGCTGATGGT
CCCACTTCCACATCCACATGCCTGGAGCCCCAGGGTGGACCCAGCTCCCCAGTGCAGC
CACCCCCAGCCTCTGGCGCTGAGGAACCAGACCTGCAGAGCCTGGAGGCCATGATGGAGGTGGTGGTGGT
GCAGCAATTCAAGTGCAAGATGTGCCAGTACCGGAGCAGCACCAAGGCCACACTGCTGCGCCACATGCGG
GAGCGACACTTCCGTCCAGCAGCGGCAGCAGCAGGTAAAAAGGGACGTCTGCGGAAATGGGGTACCT
TGACCAAGACCCAGGAGGAAGAAGGGCCAGAAGAGGAGGACGATGATGACATCGTGGATGCTGGCGCCAT
TGACGACCTGGAGGAGGACAGTGACTATAATCCAGCCGAGGATGAGCCCCGGGGCCGGCCGCTGCGGCTC
CAGCGTCCCACCCCTAGTTCCCCAAGGCCCAGAAGGAGACCTGGCCGACCCCGGAAACTGCCTCACCTGG
AGACCTCGGACGATGATATGGAAGGAGAGCCTCTAGTGAGTTCCCAGAGCGGACAGAGCCCTCC
AGAGCCACAGGACCCCGAGACTCCCAGCTCCTCAGGCCCTGGATACCTGGCGGCCCTGGGCCAGGCTAGC
CAGGCTCCAGTGGAAGCCGGTGTGAGCCAGTCAGATGCAGAGAACGCAGCACCCTCCTGCCAGGATGGGC
CTGATGCCCCACCCCGCCGCCGTGGTCGACCCTCTAGGCGCTTCCTAGGCAAGAAATACCGCAAGTACTA
TTACAAGTCGCCCAAACCGCTTCTGAGGCCCTTCCTGTGCCGCATCTGTGGCTCTCGCTTTCTGTCCCAT
GAGGACCTGCGCTTCCACGTCAACTCCCACGAGGCTGGTGACCCCCAACTCTTCAAGTGCCTGCAGTGCA
GCTACCGTTCCCGCCGCTGGTCCTCACTCAAGGAGCACATGTTCAACCACGTTGGTAGCAAGCCTTACAA
ATGTGACGAGTGCAGTTATACCAGCGTCTACCGGAAGGATGTCATCCGGCATGCAGCTGTGCACAGCCGG
GACCGGAAGAAGAGGCCAGATCCGACCCCAAAGCTGAGCTCTTTCCCCTGCCCTGTATGTGGCCGTGTGT
ACCCTATGCAGAAGAGGCTCACACAGCACATGAAGACACACAGCACTGAGAAGCCTCACATGTGTGACAA
GTGTGGGAAGTCCTTTAAGAAGCGCTACACCTTCAAAATGCACCTGCTCACGCACATTCAGGCTGTGGCC
AACCGCAGGTTCAAGTGCGAGTTCTGCGAGTTTGTTTGTGAGGACAAGAAGGCACTGCTGAACCACCAGC
TGTCCCATGTCAGCGACAAGCCCTTCAAATGCAGCTTTTGTCCCTACCGCACCTTCCGAGAGGACTTCTT
GTTGTCCCACGTGGCCGTCAAGCACACAGGGGCCAAGCCCTTCTCCTGTGAGTACTGCCACTTCAGCACC

CGGCACAAGAAGAACCTCCGGCTGCACGTGCGGTGCCGACACGCCGGCAGCTTTGAGGAGTGGGGGCGGC
GCCACCCCGAGGAGCCCCCCTCCCGCCGCCGCCCCTTCTTCTCTCGGCAGCAGATTGAGGAGCTGAAGCA
GCAGCACAGTGCAGCCCCGGGACCGCCCCCCAGCTCCCTGGGGCCTGAGATGCCCCCAGAGGCAGCA
TCTTTCCAGGCATCTGAGACCCCCTCGCTGCTCTGTCCTGACACCCTGGGTGGCGCCACCATCATCTATC
AGCAAGGAGCTGAGGAGTCAACCACGATGGCCACGCAGACAGCCTTGGATCTGCTGCTGAACATGAGTGC
TCAGCGGGAACTGGGGGGCACAGCCCTGCAGGTGGCCGTGGTGAAGTCGGAGGACATGGAAGCAGAGTTA
GTATCTCCTGGTGGGCAGCCCTCCCCTGAAGGGGCCACTTCACAGGTGGTCACCCTTCACGTGGCTGAGC
CAGGGGGCGGCGTCACAGCTGAGAGCCAGCTAGGCACCCCTGACTCACCGCAGATCACCCTGACACCCGG
TCCGTTTGGTGGGGCTGGCTACAGTGTCATCACAGCGCCTATGGAGGAAGGGACATCAGCTCCTGGG
ACACCTTACAGTGAGGAACCCCCAGGGGAAGCAGCCCAAGCTGTGGTGGTGAGCGACACCCTGAAAGAAG
CGGGCACCCACTACATCATGGCCTCTGACGGGACCCAGCTGCATCACATCGAGCTGACTGCAGATGGCTC
GATCTCTTTCCCACCAGATGCCCTGGCTTCTGGAACCAAGTGGCCCCTGCTGCAGTGTGGGGGCCTG
CCCAGAGACGGCCCTGAGCCTCCATCTCCAGCCAAGACCCACCGGGCAGGGGACCCCCAGAGTTCTGCCT
CCCCCTCTCCTGTAGCCAGCAAAGCCCTGGGCCTGGTCGTGCCCTCCTCACCACTGTCTGCAGCCACTGC
AGCGTCAAAGAAATTTTCCTGCAAGATCTGTGCCGAGGCCTTCCCTGGCCGAGCTGAGATGGAGAGTCAC
AAACGGGCCCATGCTGGGCCTAGTGCCTTCAAGTGTCCCGACTGCTCCTTCAGTGCCCGCCAGTGGCCTG
AGGTCCGGGCCCACATGGCGCAGCACTCGAGTCTGCGGCCCCACCAGTGCAGCCAATGCAGCTTTGCCTC
CAAGAACAAGAAGGACCTGCGGCGGCATGTGCTGACCCACACCAAGGAGAAGCCCTTTGCATGCCACCTC
TGCGGGCAGCGTTTCAACCGCAATGGGCACCTCAAGTTCCACATCCAGCGGCTTCACAGTCCCGATGGGA
AGAAGGCAGGGGCCCCTACTGCCCGGGGCCCAGCCAGAACCCCAACCCAGACCATCATCCTCAATAGTGA
TGACGAGACGCTGGCCACCCTGCACACTGCACTCCAGTCCAGCCATGGGGTCCTGGGCCCAGAGCGGCTA
CAGCAGGCATTGGGCCAGGAACACATTATCGTGGCCCAGGAACAGACAGTGACCAATCAGGAGGAAGCCA
CCTACATCCAGGAGATCACCGCGGATGGCCAGACAGTACAGCACCTGGTGACCTCTGACAACCAGGT
GCAGTACATCATCTCCCAGGATGGTGTCCAACATCTGCTCCCCCAGGAGTATGTTGTGGTCCCCGAAGGC
CATCATATCCAGGTGCAGGAGGGGCAGATCACACACATCCAGTATGAACAAGGGGCCCCATTCCTTCAGG
AGTCCCAGATCCAGTATGTGCCTGTGTCCCCAGGCCAGCAGCTCGTCACACAGGCTCAACTTGAGGCCGC
AGCACATTCAGCCGTCACAGCAGTGGCTGATGCTGCCATGGCCCAAGCACAGGGCCTGTTTGGCACAGAG
GAGACAGTGCCCGAACACATTCAACAGCTGCAACATCAGGGCATCGAGTACGACGTCATCACCCTGGCC
GAC
>Cebus capucinus imitator XM_017541269.1
ATGGAGGAGAACGAGGTGGAGAGCAGCAGCGAC
GCGGCCCCTGGGCCTGGCCGGCCCGAGGAGCCCTCTGAGAGCGGCCTGGGTGTGGGCACCTCGGAAGCCG
TGTCCGCCGACAGCAGCGACGCAGCGGCTGCCCCGGGCCAGGCAGAGGCCGACGACTCTCGAGTGGGGCA
AAGCTCGGACAGTGGCAGCCGCTCTCAGGAGGAGGTGTCCGAGAGCAGCTCAAGCGCAGACCCCCTGCCT
AATGGTTACCTCCCTGATTCATCGTCTGTGTCCCGTGGGCCAGTGGCAGGGGTGACAGGCGGTCCCCCAG
CACTTGTGCACTCCAGTGTACTCCCAGACCCCAACATGCTGGTGTCTGACTGCACAGCTTCCTCCTCGGA
CCTGGGCTCAGCCATCGACAAGATCATCGAGTCCACCATTGGGCCCGACCTCATGCAGAGCTGCATTACT
GTGACCAGTGCCGAGGATGGCGGGGCTGAGACCACGCGATACCTGATCCTGCAGGGCCCAGATGATGGAG
CCCCCATGACGTCACCAATGTCCAGTTCCACCTTGGCCCATAGTCTGGCAGCCATTGAGGCCCTGGCCGA
TGGCCCCACATCCACATCCACATGCATGGAGCCACAGGGTGGGCCCAGCTCCCTGGTG
CAGCTGCTCCCAGCCTCTGGTGCTGAAGAACCGGACCTGCAGAGCCTGGAGGCCATGATGGAGGTGGTGG
TGGTGCAGCAGTTCAAGTGCAAGATGTGCCAGTACCGGAGCAGCACCAAGGCCACGCTGCTGCGCCACAT
GCGGGAGCGCCACTTCCGTCCAGCAGCAGCAGGAGTTGGTAAAAAAGGACGTCTACGGAAG
TGGAGCACCTCCACCAAGACCCAAGAGGAAGAGGGACCAGAGGAGGAGGACGATGACGACATTGTAGATG
CTGGAGCCATTGATGACCTGGAGGAGGATAGTGACTATAATCCAGCTGAGGATGAACCCCGAGGCCGGCA
GCTTCGGCTCCAGCGCCCAACCCCCAGTACCCCAAGGCCCCGAAGGAGACCTGGCCGGCCCCGGAAGCTG
CCCCGCCTGGAGATCTCAGACCATGGTGTGGAAGGAGAGCCTCTAGTGAGTTCCCAGAGTGGCCAGAGCC

CTCCAGAACCACAGGATCCCGAGGCTCCCAGCTCCTCAGGCCCGGGACACCCCGTGGCCATGGGCAAGGC
AAGCAGGACCCCTGTGGAACCGGGTGTGAGCCAGTCAGACGCAGAGAATGCAGCCCCCTCCTGCCCAGAT
GAGCATGACACTCCGCCCCGGCGCCGAGGTCGACCTTCCAGGCGCTTCCTAGGCAAGAAATACCGCAAGT
ACTATTACAAGTCACCCAAACCACTTTTGAGGCCCTTCCTGTGCCGCATCTGTGGCTCTCGATTTCTGTC
CCATGAGGACCTGCGCTTCCATGTCAACTCCCACGAGGCCGGCGATCCCCAGCTCTTCAAGTGTCTGCAG
TGCAGCTATCGCTCCCGTCGCTGGTCCTCACTCAAGGAGCACATGTTCAACCATGTGGGCAGCAAGCCCT
ACAAGTGTGATGAGTGCAGCTACACCAGTGTCTACCGGAAGGACGTCATCAGGCACGCAGCTGTGCACAG
CCGGGACCGGAAGAAGAGGCCAGATCCGACTCCAAAGCTGAGCTCTTTTCCCTGCCCTGTGTGTGGCCGT
GTGTACCCCATGCAGAAAAGACTCACACAGCACATGAAGACACACAGCACTGAGAAGCCCCACATGTGTG
ACAAGTGTGGAAAGTCCTTTAAGAAGCGCTACACCTTCAAAATGCACCTGCTTACGCACATCCAGGCTGT
TGCCAACCGCAGGTTCAAGTGTGAGTTCTGTGAGTTCGTTTGTGAAGATAAGAAGGCACTGCTGAACCAC
CAGCTGTCCCACGTCAGTGACAAGCCCTTCAAATGCAGCTTTTGTCCCTACCGCACCTTCCGAGAGGACT
TCCTGCTGTCCCATGTGGCTGTCAAGCACACAGGGGCCAAGCCCTTCGCCTGTGAGTACTGCCACTTCAG
CACACGGCACAAGAAGAATCTCCGCCTGCACGTACGGTGTCGACACGCAAGCAGCTTCGAGGAATGGGGG
CGGCGCCACCCTGAGGAGCCCCCCTCCCGCCGTCGCCCCTTCTTCTCCCTGCAGCAGATTGAGGAGCTGA
AGCAGCAGCACAGTGCGGCCCCCGAACCACCTCCCAGCTCCCCAGGACCTGAGATACCCCCAGAGGCAAC
ACCTTTCCAGTCATCTGAGGCTCCTTCACTGCTCTGTCCTGACACCCTGGGTGGCGCCACCATCATCTAC
CAGCAAGGAGCCGAGGAGTCGACCGCGCTGGCCACGCAGACGGCCTTGGATCTTCTGCTGAACATGAGCG
CTCGGCGGGAACTGGGGGGCACAGCCCTGCAGGTGGCCGTGGTGAAGTCGGAGAACATGGAAGTGGGGTT
ACCATCCCCTGGTGGGCAGCCCTCCCCTGAAGGTACCACTCCACAGGTGGTCACCCTCCATGTGGCAGAG
CCAGGGGGTGGTACAGTAGCCGAGAGCCAGCAAGGCCCTCCTGACCTACCACAGATCACCTTGGCATCTG
GTGCATTTGGTGGGACTGGCTACAGCGTTATCACAGCACCTATGGAGGAGGGGACGTCAGCTCCTGG
CACACCTTACAGCGAGGAGCCCCCAGGGGAGGCAGCCCAGGCTGTGGTTGTGACTGACCCCCTAAAAGAA
GCTGGCACCCACTACATCATGGCTGCTGATGGTACCCAGTTGCACCACATCGAGCTCTCCGCAGATGGCT
CCATCTCCTTCCCACCAGATTCCTTGGCCTCTGGTCCCAAATGGCCCCTACTGCAGTGTGGGGGGCT
GCCCAGAGACGGCCCTGAGCCCCCATCTCCAGCCAAGACCCACCGGATAGGGGACCCCCAGAGGTCTGCC
TCCCCACCTCCTGCAGCCAGCAAAGCCCTGGGCCCAGCAGTGCCCCCCTCACCACCATCCGCAGGCACTG
CAGCATCAAAGAAGTTTTCCTGCAAGATCTGTGCTGAGGCCTTCCCTGGCAGAGCTGAGATGGAGAGTCA
CAAGCGGGCCCACGCTGGGCCTGGTGCCTTCAAGTGCCCCGACTGTCCCTTCATTGCCCGCCAGTGGCCT
GAGGTTCGGGCACACATGGCACAGCATTCAAGCCTGCGGCCCCACCAGTGTAGCCAATGCAGCTTTGCCT
CCAAGAACAAGAAGGACCTGCGGCGGCACATGCTGACTCACACCAAGGAGAAGCCTTTTGCGTGCCACCT
CTGTGGGCAGCGTTTCAACCGTAACGGGCACCTCAAGTTCCACATCCAGCGACTGCACAGTCCTGATGGG
AGGAAGTCAGGAACCCCTACAGCCCGGGCCGCTACCCAGAACCCAACCCAGACTATCATTCTCAACAGTG
ATGATGAAACACTGGCCACTCTGCATACTGCACTCCAGTCCAGTCACGGAGTCTTAGGCCCAGAGCGACT
GCAGCAGGCACTGGGCCAGGAGCACATCATTGTCGCCCAGGAACAGACAGTGACCAATCAAGAGGAAGCC
ACCTACATCCAAGAGATCACCGCAGATGGCCAGACCGTACAGCACCTGGTAACCTCCGACAACCAGG
TGCAATACATCATCTCCCAGGATGGTGTCCAGCACCTGCTCCCCCAGGAATACGTTGTGGTCCCCGAGGG
CCATCACATCCAGGTACAGGAGGGCCAGATCACACACATCCAGTATGAACAAGGAGCCCCGTTCCTTCAG
GAGTCCCAGATCCAGTATGTGCCTGTGTCGCCAGGCCAGCAGCTAGTCACACAGGCTCAACTTGAGGCCG
CAGCACACTCGGCTGTCACAGCAGTGGCTGATGCTGCCATGGCTCAAGCCCAGGGCCTGTTTGGCACAGA
GGAGGCAGTGCCTGAACACATTCAGCAGCTGCAGCACCAGGGCATCGAATACGACGTCATCACCCTGGCC
GAC
>Microcebus murinus XM_012742899.2
ATGGAGGAGAACGAGG
TGGAGAGCAGTAGCGACGCTGCCCCTCGGCCTGGCCGGCCGGAGGAGCCTTCTGAGAGCGGTCTGGGTGT
GGAGACCTCGGAAGCCGTGTCTGCCGACAGCAGCGACGCGGCGGCCGCCCCGGGGCAGACAGAGGCTGAC
GACTCCGGTGTAGGGCAAAGCTCGGACCTCGGGAGCCGCTCTCAGGAGGAGGTATCCGAGAGCAGCTCAA

GCACGGACCCCCTGCCTCATGGCTACCTCCCTGATTCATCGTCTGTGTCCCATGGGCCAGTGGCAGGGGT
GACAGGCGGCCCCCCAGCTCTAGTGCACTCCAGCGCACTCCCAGACCCCAACATGCTGGTGTCTGACTGC
ACGGCTTCCTCCTCGGACTTGGGTTCAGCCATCGACAAGATCATCGAGTCCACCATCGGCCCAGACCTCA
TCCAGAGCTGCATCACTGTGACCAGTGCTGAGGATGGTGGGGCTGAGACCACGCGGTACCTAATCCTGCA
GGGCCCAGATGACGGTGCCCCTATGACATCGCCGATGTCCAGTTCCACCCTGGCCCACAGCCTGGCAGCC
ATCGAGGCCCTGGCTGATGGCCCCACGTCCACATCCACGTGCCTGGAGCCACAGGGCG
GGCCCAGCTCCCCAGCGCAGCCACCCCCGGCTTCTGATGCCGAGGAACTGGACCTGCAGAGCCTAGAGGC
CATGATGGAGGTGGTGGTGGTACAGCAGTTCAAGTGTAAGATGTGCCAGTACCGGAGCAGCGCCAAGGCC
ACACTGCTGCGCCACATGCGGGAGCGGCACTTCCGTCCGGCAGCGGCACCAGCTGGCAAAAAGGGGCGTC
TGCGGAAGTGGGGCACCTCGACCAAGACCCAGGAGGAAGAGGGGCCAGAGGAGGAGGATGACGACGACAT
TGTAGACGCTGGTGCCATTGACGACCTGGAAGAGGACAGCGACTATAATCCGGCTGAAGATGAGCCCCGG
GGCCGGCAGCTACGGCTCCAGCGCCCCAGCCCCAGTACCCCAAGACCTCGAAGGAGGCCTGGGCGGCCCA
GGAAGCTGCCTCGCCTGGAGACCTCAGACGATGGTGTGGAAGGAGAGCCTCTAGTGAGTTCGCA
GAGTGAACAGAGCCCTCTGGAGCCACAGGATCCTGAAGCTCCCAGCTCCTCAGGCCCAGGGCAGCTAGTG
GGCCTGGGCAAGGCTAGCAGGGCCCCCGTGGAACCTGGTGTGAGCCAGTCAGATGCAGAGAATGCAGCAC
CCTCCTGCCAGGATGAGCCTGACGCCCCGCCCCGCCGCCGTGGTCGACCCTCTAGGCACTTCTTAGGCAA
GAAATACCGCAAGTACTATTACAAGTCGCCCAAACCACTTCTGAGGCCCTTCCTGTGCCGCATCTGTGGC
TCTCGCTTCCTGTCCCACGAGGACCTGCGCTTCCACGTCAGCTCCCACGAGGCCGGCGACCCCCAGCTCT
TCAAGTGCCTGCAGTGCAGCTATCGCTCTCGCCGCTGGTCCTCGCTCAAGGAGCACATGTTCAACCACGT
GGGCAGCAAGCCCTACAAGTGTGATGAGTGCAGCTATACCAGTGTCTACCGGAAGGATGTCATCCGGCAC
GCGGCCGTACATAGCCGGGACCGGAAGAAGAGGCCAGATCCGACCCCAAAGCTGAGCTCTTTCCCCTGCC
CCGTGTGTGGCCGTGTGTACCCCATGCAGAAGAGACTCACACAGCACATGAAGACGCACAGCACTGAGAA
GCCCCACATGTGTGACAAGTGTGGAAAGTCCTTTAAGAAGCGCTACACGTTCAAAATGCACCTGCTCACG
CACATCCAGGCCGTTGCCAACCGCAGGTTCAAGTGTGAGTTCTGCGAGTTTGTTTGTGAGGACAAGAAGG
CACTGTTGAACCACCAGCTGTCCCACGTCAGTGATAAGCCCTTCAAATGCAGCTTTTGTCCCTACCGCAC
CTTCCGAGAGGACTTCCTGCTGTCCCATGTGGCTGTCAAGCACACAGGGGCCAAGCCCTTTGCCTGTGAG
TACTGCCACTTCAGCACACGCCACAAGAAGAATCTACGCCTGCACGTTCGGTGCCGACATGCAAGCAGCT
TCGAGGAATGGGGGCGGCGCCACCCCGAGGAGCCCCCGTCCCGCCGCCGCCCTTTCTTCTCTCTGCAGCA
GATCGAGGAGCTGAGGCAGCAGCACAGTGTGGCCCCTGCGCTGCCTCCCAGCTCCCCAGGACCTGAG
ATACCCCCAGAGGCAGCACCTTTCCAGTCACCTGAGACCCCCCCACTGCTCTGTTCTGACACCCTGGGCG
GCACCACCATCATCTACCAGCAAGGAGCTGAGGAGTCAGCTGCGATGGCCACACAGACAGCCTTGGATCT
GCTGCTGAACATGAGTGCTCAGCGGGCCCTGGGGGGCACAGCCTTGCAGGTGGCTGTGGTGAAGTCGGAG
GACGTGGAAGCAGGGTTGGCATCCCCTAGTGAGCAGCCCTCTCCCGAAGATGCCACACCACAGGTGGTAA
CCCTCCACGTGGCAGAGCCCGGGGGCAATATGGCAGCTGAAGGCCAGCTAGGCACCCCTGAACTACCGCA
GATCACCCTGGCACCAGGTCCATTTGGTGGGGCTGGCTACAGTGTCATCACAGCACCTATGGAGGAG
GGGACATCAGCTCCCGGCACACCTTACAGCGAGGAGCCCCCAGGGGAGGCAGCCCAGGCTGTGGTTGTGA
GTGACACCCTCAAGGAAGCTGGCACCCACTACATAATGGCTGCTGACGGGACCCAGCTGCACCACATCGA
GCTGACTACAGATGGCTCCATCTCCTTCCCTCCGGATGCTCTGGCCTCTGGAGCCAAGTGGCCCCTGCTG
CAGTGTGGAGGGCTGTCCAGAGATGGCCCTGAGCCCCCATCTCCAGCCAAGACCCACCAGGTGGGGGACC
CCCAGAGCTCTACCTCCCCACCTCCTGCAGCCAGCAAAGCCCTGGGCCTGGTCGTGCCCTCCTCACCGCC
ATCTGCAGCCACTGCAGCATCCAAGAAGTTTTCCTGCAAGATCTGTGCCGAGGCCTTCCCTGGCCGAGCA
GAGATGGAGAGTCACAAACGGGCCCACGCGGGGCCCAGTGCCTTCAAGTGCCCGGACTGCCCCTTCAGTG
CTCGCCAGTGGCCTGAAGTCCGGGCCCACATGGCACAGCACTCAAGCCTACGGCCCCACCAGTGCAGCCA
GTGCAGCTTCGCCTCCAAGAACAAGAAGGACCTGCGACGGCACGTGCTGACCCACACCAAGGAGAAGCCT
TTCGCGTGCCACCTCTGCGGGCAGCGTTTCAACCGTAACGGGCACCTCAAGTTCCACATCCAGCGGCTGC
ACAGTCCTGATGGGAGGAAGTCAGGGACCCCTACAGCCCAGGCCCCAACCCGGACTCCCACCCAGACCAT
CATCCTGAACAGTGATGACGAGACACTGGCCACACTACACACTGCACTCCAGTCCAGTCACGGAGTCCTG

GGCCCAGAGCGGCTGCAGCAGGCACTGGGCCAGGAACACATCATCGTGGCCCAGGAACAGACAGTGACTA
ATCAGGAGGAAGCCACCTACATCCAAGAGATCACCGCAGATGGCCAGACAGTACAGCACCTGGTGAC
CTCTGACAACCAGGTACAGTACATCATTTCCCAGGATGGTGTCCAGCACCTGCTCCCCCAGGAATATGTC
GTGGTCCCTGAGGGCCATCACATCCAGGTTCAGGAGGGCCAGATCACACATATCCAGTATGAACAAGGGG
CCCCATTCCTTCAGGAATCCCAGATCCAGTATGTGCCTGTGTCCCCAGGCCAGCAGATTGTCACACAGGC
TCAACTGGAGGCTGCAGCACACTCAGCTGTCACAGCAGTGGCTGACGCTGCCATGGCCCAAGCCCAGGGC
CTGTTTGGCACAGAGGAGGCAGTGCCTGAGCACATTCAGCAGCTGCAACACCAAGGCATCGAGTACGACG
TCATCACCCTGAACGAC
>Propithecus coquereli XM_012640460.1
ATGGAGGAGAACGAGGT
GGAGAGCAGTAGCGACGCGGCCCCTCGGCCTGGCCGGCCGGAGGAGCCCTCGGAGAGCGGCCTGGGTGTG
GAGACCTCGGAAGCCGTGTCAGCCGACAGCAGCGACGCGGCGGCCGCCCCAGGGCAGGCAGAGGCTGACG
ACTCCGGCGTAGGGCAAAGCTCAGACCACGGCAGCCGCTCTCAGGAGGAGGTATCTGAGAGCAGCTCAAG
CACGGACCCCCTGCCTCATGGCTACCTCCCCGATTCATCGTCTGTGTCCCATGGGCCAGTGGCAGGGGTG
ACAGGCGGCCCCCCGGCTCTAGTGCACTCCAGCGCACTCCCAGACCCCAACATGCTGGTGTCTGACTGCA
CGGCTTCCTCCTCGGACTTGGGCTCGGCCATCGACAAGATCATCGAGTCCACCATCGGCCCTGACCTCAT
CCAGAGCTGCATCACTGTGACCAGCGCTGAGGATGGCGGGGCTGAGACCACACGGTACCTAATCCTGCAG
GGCCCAGATGACGGTGCCCCTATGACATCACCGATGTCCAGTTCCACCCTGGCCCACAGCCTGGCGGCCA
TTGAGGCCCTGGCTGATGGCCCCACGTCCACATCCACGTGCCTGGAGCCACAGGGTGG
GCCCAGCTCCCCAGCGCAGCCACCCCCTGCCTCTGATGCTGAGGAACTGGACCTGCAGAGCCTAGAGGCC
ATGATGGAGGTGGTGGTGGTACAGCAATTCAAATGTAAGATGTGCCAGTACCGGAGCAGCGCCAAGGCCA
CGCTGCTGCGCCACATGCGGGAGCGGCACTTCCGTCCAGCAGCAGCAGCAGCTGGCAAAAA
GGGGCGTCTGCGGAAGTGGGGCGCCTCGACCAAGGCCCAGGAGGAAGAGGGGCCAGAGGAGGAGGATGAT
GATGACATTGTAGACGCTGGTGCCATTGACGACCTGGAGGAGGACAGCGACTATAATCCGGCTGAGGACG
AGCCCCGGGGCCGGCAGCTACGGCTCCAGCGCCCCACCCCCAGTACCCCAAGACCCCGAAGGAGGCCTGG
CCGGCCTAGGAAGTTGCCTCGCCTGGAGACCTCAGACGATGGTGTGGAAGGAGAGCCTCTAGTG
AGTTCGCAGAGTGGACGGAGCCCTCTGGAGCCACAGGATCCCGAAGCTCCTAGCTCCTCAGGCCCAGGAC
AGCTAGTGGCCCTGGGCAAGGCTAGCAGGGCCACCGTGGAACCTGGTGTGAGCCAGTCAGATGCAGAGAA
TGCAGCACCCTCCTGCCAGGACGAGCCTGACGCCCCGCCCCGCCGCCGCGGTCGACCCTCTAGGCACTTC
TTGGGCAAGAAATACCGCAAGTACTATTACAAGTCGCCCAAACCACTCCTGAGGCCCTTCCTATGCCGCA
TCTGCGGCTCTCGCTTCCTGTCCCACGAGGACCTGCGCTTCCACGTGAGCTCCCACGAGGCCGGCGACCC
CCAGCTCTTCAAGTGCCTGCAGTGCAGCTATCGTTCCCGCCGCTGGTCCTCGCTCAAGGAGCACATGTTC
AACCACGTGGGCAGCAAGCCCTACAAGTGTGATGAGTGCAGCTACACCAGTGTCTACCGGAAGGACGTCA
TCCGGCACGCGGCCGTACACAGCCGGGACCGGAAGAAGAGGCCAGATCCGACCCCAAAGCTGAGCTCTTT
CCCCTGCCCTGTGTGTGGCCGTGTGTACCCCATGCAGAAGAGACTCACACAGCACATGAAGACGCACAGC
ACTGAGAAGCCCCACATGTGTGACAAGTGTGGAAAGTCCTTTAAGAAGCGCTACACCTTCAAAATGCACC
TGCTCACGCACATCCAGGCTGTTGCCAACCGCAGGTTCAAGTGTGAGTTCTGCGAGTTCGTTTGTGAGGA
CAAGAAGGCACTGCTGAACCACCAGCTGTCCCACGTCAGCGATAAGCCCTTCAAATGCAGCTTTTGTCCC
TACCGCACCTTCCGAGAGGACTTCCTGCTGTCCCACGTGGCTGTCAAGCACACAGGGGCCAAGCCCTTTG
CCTGTGAGTACTGCCACTTCAGCACACGGCACAAGAAGAATCTACGCCTGCACGTACGGTGCCGACATGC
AAGCAGCTTCGAGGAATGGGGGCGGCGCCACCCCGAGGAGCCCCCCTCCCGCCGCCGCCCTTTCTTCTCT
CTGCAGCAGATCGAGGAGCTGAAGCAGCAGCACAGTTCGGGCCCTGAGCTACCTCCCAGCTCTCCAGGAC
CTGAGATACCTCCAGAGGCAGCACCTTTCCAGTCACCTGAGACCCCCCCGCTGCTCTGTTCTGACAC
CCTGGGCGGCACCACCATCATCTACCAGCAAGGAGCTGAGGAATCAGCTGCGATGGCCACACAGACAGCC
TTGGATCTGCTGCTGAACATGAGTGCTCAGCGGGCCCTGGGGGGCACAGCCTTGCAGGTGGCCGTGGTGA
AGTCGGAGGATGTGGAAGCAGGGTTAGCATCCCCTGGTGGGCAGCCCTCCCCCGAAGACGCCACACCACA
GGTGGTAACCCTCCACGTGGCAGAGCCCGGGGGCAGTGTGGCAGCTGAAAGCCAGCTAGGCACCCCTGAA

CTACCACAGATCACCCTGGCACCAGGTCCATTTGGTGGGGCTGGCTACAGTGTCATCACAGCACCTA
TGGAGGAGGGGACATCAGCTCCTGGCACACCTTACAGCGAGGAGCCCCCAGGGGAGGCAGCCCAGGCTGT
GGTTGTGAGCGACACCCTCAAGGAAGCTGGCACCCACTACATAATGGCTGCCGACGGGACCCAGCTGCAC
CACATCGAGCTGACTGCAGATGGCTCCATCTCCTTCCCGCCGGATGCTCTGGCCTCTGGAGCCAAGT
GGCCCCTGTTGCCGTGTGGAGGGCTGCCCAGAGATGGCCCTGAGCCCCCATCTCCAGCCAAGACCCACCA
GGTGGGGGACCCCCAGAGCTCTGCCTCCCCACCTCCTGCAGGCAGCAAAGCCCTGGGCCTGGTCGTGCCC
TCCTCACCACCGTCTGCAGCCACTGCAGCATCAAAGAAGTTTTCCTGCAAGATCTGTGCTGAGGCCTTCC
CTGGCCGAGCAGAGATGGAGAGTCACAAACGGGCCCACGCCGGGCCCAGTGCCTTCAAGTGCCCTGACTG
CCCCTTCAGTGCTCGCCAGTGGCCTGAAGTCCGGGCCCACATGGCACAGCACTCCAGCCTGCGTCCCCAC
CAGTGCAGCCAGTGCAGCTTCGCCTCCAAGAACAAGAAGGACCTGCGGCGGCACGTGCTGACCCACACCA
AGGAAAAGCCTTTCGCGTGCCACCTCTGCGGGCAGCGTTTCAACCGTAACGGGCACCTCAAGTTCCACAT
CCAGCGGCTGCACAGTCCTGATGGGAGGAAGTCAGGGACCCCTACAGCCCGGGCCCCAACCCGGACTCCC
ACCCAGACCATTATCCTGAACAGTGATGACGAGACACTGGCCACACTACACACTGCGCTCCAGTCCAGTC
ACGGAGTCCTGGGCCCAGAGCGGCTACAGCAGACACTGGGCCAGGAACACATCATCGTGGCCCAGGAACA
GACAGTGACCAATCAGGAGGAAGCCACCTACATCCAAGAGATCACCGCAGATGGCCAGACAGTACAG
CACCTGGTGACCTCTGACAACCAGGTACAGTACATCATCTCCCAGGACAGTGTCCAGCACCTGCTCCCCC
AGGAATATGTCGTGGTCCCTGAGGGCCATCACATCCAGGTTCAGGAGGGCCAGATCACACACATCCAGTA
TGAACAAGGAGCCCCATTCCTTCAGGAATCCCAGATCCAGTATGTGCCTGTGTCCCCAGGCCAGCAGATT
GTCACGCAGGCTCAACTGGAGGCTGCAGCACACTCAGCTGTCACAGCAGTGGCTGACGCTGCCATGGCCC
AAGCCCAGGGCCTGTTTGGCACAGAGGAGGCAGTGCCTGAACACATTCAGCAGCTGCAGCACCAGGGCAT
CGAGTACGACGTCATCACCCTGAACGAC
>Otolemur garnettii XM_003787662.3
ATGGAGGAGAGTGAGGTGGAGAGCAGTAGCGACGCGGCCCCT
CGGCCCGGCCAGCCAGAGGAGCCCTCTGAGAGTGGCCTGGGTGTGGGGACTTCGGAAGCCGTGTCTGCGG
ACAGTAGCGACGCGGCAGCAGCCCCAGGACAGGCAGAAGCTGACGACTCCGGCGTGGGGCAAAGCTCAGA
CCGTGGCAGTGGCTCTCAGGTGGAGGTGTCTGAGAGCAGCTCAAGTACAGACTCCCTGCCTCATGGCTAC
CTCCCTGATTCATCGTCTGTGTCCCATGGGCCAGTGGTGGGGGTGACAGGTGGCCCCCAAGCGCTAGTAC
ACTCCAGTGCACTCCCAGACCCCAACATGCTGGTGTCTGACTGCACAGCTTCCTCCTCGGACTTGGGTTC
AGCCATCGACAAGATCATTGAGTCTACCATTGGCCCTGACCTCATCCAGAGCTGTATCACTGTGACCAGT
GCTGAGGATGGCGGGGCTGAGACCACGCGATACCTGATCCTGCAGGGCCCAGATGATGGTGCCCCCATGA
CATCCCCAATGTCCAGTTCCACCCTAGCCCACAGCCTGGCAGCCATTGAGGCCCTGGCTGATGGCCCCAC
CTCCACATCAACGTGCCTGGAGCCACAGGGTGGGCCCAGCTCTCCAGCACAGCCACCC
CCAGCATCTGGTGCCGAGGAGCCGGACCTGCAGAGCCTGGAGGCCATGATGGAGGTGGTGGTGGTGCAGC
AATTCAAGTGTAAGATGTGCCAGTACCGGAGCAGCACCAAGGCCACACTGCTGCGCCACATGCGGGAGCG
GCACTTCCGTCCAGCAGCAGCAGCAGTTGGTAAAAAGGGACGTCTGCGGAAGTGGGGC
ACCTCAACCAAGACCCAGGAGGAAGACGGGCCAGAGGAGGAGGACGATGATGACATTGTTGATGCTGGCG
CCATTGATGACCTAGAGGAGGACAGCGACTATAATCCAGCTGAGGATGAGCCCCGGGGCCGGCAGCTACG
GCTCCAGCGCCCTACCCCCAGTACCCCAAGACCCCGAAGGAGGCCTGGCCGGCCCCGAAAGCTGCCTTGC
TTAGAGACCTCAGACGATGGTGTGGAAGGAGAGCCTCTAGTGAGTTCCCAAAGTGGACAGAGCC
CTCTGGAGCTACAAGATCCTGAAGCTCCTAGCTCCTCAGGTCCTGGACAGCTGGTGGCCCTGGGCAAGGC
TAGCAGGGCCCCTGTGGAACCTGGAGTGAGTCAGTCAGATGCAGAGAATGCAGCACCCTCCTGCCAAGAT
GAGCCTGACGCCCTGCCTCGCCGCCGTGGTCGTCCATCCAGGCACTTCTTAGGCAAGAAATACCGCAAGT
ACTATTACAAGTCGCCCAAACCACTCCTGAGGCCTTTCCTGTGCCGCATCTGTGGCTCTCGCTTCCTGTC
CCACGAGGACCTGCGCTTCCACGTCAGCTCTCATGAGGCCGGTGACCCCCAGCTCTTCAAGTGCCTGCAG
TGCAGCTATCGCTCCCGCCGCTGGTCCTCACTCAAGGAACACATGTTCAACCACGTGGGCAGCAAGCCCT
ACAAGTGTGACGAGTGCAGCTACACCAGTGTCTACCGAAAGGACGTCATCCGGCATGCAGCTGTACACAG
CAGGGACCGGAAGAAGAGGCCAGATCCGACCCCAAAGCTGAGCTCTTTCCCCTGCCCCGTGTGTGGCCGT

GTGTACCCCATGCAAAAGAGACTCACGCAGCACATGAAGACGCACAGCACTGAGAAGCCCCACATGTGTG
ACAAATGTGGAAAGTCATTTAAGAAGCGCTACACCTTCAAAATGCACCTCCTCACGCACATCCAGGCTGT
CGCCAACCGCAGGTTCAAGTGCGAGTTCTGCGAGTTTGTTTGTGAGGACAAGAAGGCACTGCTGAACCAC
CAGCTGTCCCATGTTAGCGATAAGCCGTTCAAATGCAGCTTTTGTCCCTATCGCACCTTCCGAGAGGACT
TCTTGCTGTCCCATGTGGCTGTTAAGCACACAGGGGCCAAGCCCTTTGCCTGTGAGTACTGCCACTTTAG
CACGAGGCACAAGAAGAATCTTCGCCTGCACGTACGGTGCCGACATGCAAGCAGTTTTGAGGAATGGGGG
CGCCGCCACCCGGAGGATCCCCCCTCCCGTCGCCGCCCCTTCTTCTCTCTGCAGCAGATTGAGGAACTGA
AGCAGCAGCATAGTGTGGCCCCTGGGCCACCTCCCAGCCCTCCAGGACCTGAGATACCCCCAGAGGC
AGCACCTTTCCAGTCACCTGAGACCTCCTCGCTGCTCTGTTCTGATTCTCTGGGTGGCACCACCATCATC
TACCAACAAGGAGCTGAGGAGTCAGCTGAGATGGCCACGCAGACAGCCTTGGATCTGCTGCTGAACATGA
GTGCTCAGCGGGCCCTGGGGGGCACAGCCTTGCAGGTGGCCGTGGTGAAGTCGGAGGATGTGGAAACAGA
GTTAACTTCCCCTGGTGGGCAACCCTCTCCCGAAGGTGCCACTCCACAGGTGGTAACCCTCCACATGGCA
GAACCAGGGGGCAGTGTGGCAGCGGAAAGCCAGCTAGGTGCCTCTGAATTACCGCAGATCACCCTGGCAC
CGGGTCCATTCGGTGGGGCTGGCTATAGCGTCATCACAGCACCTATGGAGGAAGGGACATCAGCTCC
TGGCACACCTTACAGTGAAGAGCCCCCAGGGGAAGCAGCCCAGGCTGTTGTTGTGAGTGACACCCTGAAG
GAAGCTGGCACCCACTATATCATGGCTGCTGATGGGACCCAGCTCCACCACATCGAGCTGACTGCAGATG
GCTCTGTTTCCTTCCCAGCAGATGCTCTGGCCTCTGGAGCCAGGTGGCCCCTGCTGCAGTGTGGGGG
GCTGCCCAGAGATGGTCCTGAGCCCCCATCTCCAGCCAAGACCCACCAGCTGGGGGACCCCCAGAGTTCT
GCCTCCCCACCTCTTGCAGCCAGCAAAGCCCTGGGCCTGGTCGTGCCACCCTCATCACCATCTGCTGCCA
CCACAGCATCAAAGAAGTTTTCCTGCAAGATCTGTGCTGAGGCCTTCCCTGGCCGGGCAGAAATGGAAAG
TCACAAACGGGCCCATGCTGGGCCCACTGCTTTCAAGTGCCCTGACTGCCCCTTCAGTGCTCGCCAGTGG
CCTGAGGTCCGGGCCCACATGGCACAGCACTCGAGCCTGCGGCCCCACCAGTGCAGCCAGTGCAGCTTCG
CCTCCAAGAACAAGAAGGACCTGCGGCGGCATGTACTGACCCACACCAAGGAGAAGCCTTTTGCGTGCCA
CCTCTGTGGGCAGCGTTTCAACCGTAATGGGCACCTCAAGTTCCACATCCAGCGGCTGCACAGTCCAGAT
GGGAGGAAGTCAGGGATTCCTACAGTCCGGGCCCCAACCCGGACCCCAACCCAGACCATCATCCTGAACA
GTGATGACGAAACACTGGCCACACTACACACCACACTCCAGTCCAGTCACGGAGTCCTGGGCCCAGAGCG
GCTGCAACAGGCACTGGGCCAAGAACACATCATTGTGGCCCAGGAACAGACAGTGACCAATCACGAGGAA
GCCACCTACATCCAAGAGATCATGGCAGATGGTCAGACAGTACAGCACCTGGTGACCTCTGACAATCAGG
TACAGTACATCATCTCCCCGGATGGTGTTCAGCACCTGCTCCCCCAGGAATACGTTGTGGTCCCAGAGGG
CCATCACATCCAGGTGCAGGAGGGCCAGATCACGCGCATCCAGTATGAACAAGGAGCCCCATTCCTTCAG
GAGTCCCAGATTCAGTACGTGCCTGTGTCTCCAGGCCAGCAGCTTGTCACACAGGCTCAACTGGAAGCTG
CAGCACACTCAGCTGTCACAGCAGTGGCTGATGCTGCCATGGCCCAAGCCCAGGGCCTGTTTGGCACAGA
GGAAGCAGTGCCTGAACACATTCAGCAGCTGCAGCACCAGGGCATCGAGTACGACGTCATCACCCTGACC
GAC

>Macaca fascicularis XM_005569206.2

ATGGAGGAGAACGAGGTGGAGAGCAG
CAGCGACGCGGCCCCTGGGCCTGGCCCCCCCGAGGAGCCCTCTGAGAGCGGCCTGGATGTGGGCACCTCG
GAAGCCGTGTCGGCCGACAGCAGCGACGCCGCGGCCGCCCCGGGGCAGGCAGAGGCCGATGACTCTGGCG
TGGGGCAAAGCTCGGACCGCGGCAGCCGCTCTCAGGAGGAGGTATCCGAGAGCAGCTCGAGCACAGACCC
CCTGCCTAATGGCTACCTCCCTGATTCGTCGTCTGTGTCCCATGGGCCAGTGGCAGGGGTGACAGGCGGT
CCCCCAGCACTTGTGCACTCTAGTGCACTCCCAGACCCCAACATGCTGGTGTCCGACTGCACAGCTTCCT
CCTCGGACCTGGGCTCAGCCATCGACAAGATCATCGAGTCCACCATCGGGCCCGACCTCATCCAGAGCTG
CATCACTGTGACCAGTGCCGAGGATGGCGGGGCCGAGACCACGAGGTACCTGATCCTGCAGGGCCCAGAT
GATGGAGCCCCCATGACATCACCAATGTCCAGTTCCACCTTGGCCCACAGCCTGGCAGCCATTGAGGCCC
TGGCAGATGGCCCCACATCCACATCCACATGCCTGGAGCCACAGGGTGGGCCCAGCTC
CCCGGTGCAGCTGCCTCCAGCCTCTGGTGCTGAAGAACCAGACCTGCAGAGCCTGGAGGCCATGATGGAG
GTGGTGGTGGTGCAGCAGTTCAAGTGCAAGATGTGCCAGTACCGGAGCAGCACCAAGGCCACACTGCTGC

GCCACATGCGGGAGCGCCACTTCCGTCCAGTAGCAGCAGCATCTGGTAAAAAAGGACGTCT
ACGGAAGTGGAGCACCTCCACCAAGACCCAAGAGGAAGAGGGACCGGAGGAGGAGGACGATGACGACATT
GTAGACGCTGGAGCCATTGATGACCTAGAGGAGGATAGCGACTATAATCCAGCTGAGGATGAGCCCCGAG
GCCGGCAGCTTCGGCTCCAGCGCCCCACCCCTAGTACCCCAAGGCCCCGAAGGAGACCTGGCCGACCCCG
GAAGCTGCCCCGCCTGGAGAGCTCAGACGATGGTGTGGAAGGAGAGCCTCTAGTGAGTTCCCAG
AGTGGACAGAGCCCTCCAGAGCCACAGGATCCCGAGGCTCCCAGCTCCTCAGGCCCAGGACACCTGGTGG
CCATGGGCAAGGCAAGCAGGACCCCTGTGGAAGCTGGTGTGAGCCAGTCAGATGCAGAGAATGCAGCTCC
CTCCTGCCCGGATGAGCATGACACTCCACCCCGGCGCCGAGGTCGACCTTCCAGGCGCTTCCTAGGCAAG
AAATACCGCAAGTACTATTACAAGTCGCCCAAACCGCTTTTGAGGCCCTTCCTGTGCCGCATCTGTGGTT
CTCGCTTTCTGTCCCACGAGGACCTGCGCTTCCACGTCAACTCCCATGAGGCTGGCGATCCCCAGCTCTT
CAAGTGCCTGCAGTGCAGCTATCGTTCCCGCCGCTGGTCCTCTCTCAAGGAGCACATGTTCAACCACGTG
GGCAGCAAGCCCTACAAGTGTGACGAGTGCAGCTACACCAGTGTCTACCGGAAGGACGTCATTCGGCACG
CCGCTGTGCACAGCCGAGACCGGAAGAAGAGGCCAGATCCGACTCCAAAGCTGAGCTCTTTCCCCTGCCC
TGTGTGTGGCCGTGTGTACCCCATGCAGAAAAGACTCACGCAGCACATGAAGACGCACAGCACTGAGAAG
CCCCATATGTGTGACAAGTGTGGAAAGTCCTTTAAGAAGCGCTACACCTTCAAAATGCACCTGCTCACAC
ACATCCAGGCTGTTGCCAACCGCAGGTTCAAGTGTGAGTTCTGTGAGTTCCTTTGTGAAGACAAGAAGGC
ACTGCTGAACCACCAGTTGTCCCACGTCAGTGACAAGCCCTTCAAATGCAGCTTTTGTCCCTACCGCACC
TTCCGAGAGGACTTCCTGCTGTCCCATGTGGCTGTTAAGCACACAGGGGCCAAGCCCTTCGCCTGTGAGT
ACTGCCACTTCAGCACACGGCACAAGAAGAACCTACGCCTGCACGTACGGTGCCGACATGCAAGCAGCTT
CGAGGAATGGGGGAGGCGCCACCCTGAGGAGCCCCCCTCCCGCCGTCGCCCCTTCTTCTCTCTGCAGCAG
ATTGAGGAGCTGAAGCAGCAGCACAGTGTGGCCCCTGGACCACCTCCCAGCTCCCCAGGACCTGAGA
TACCCCCAGAGGTGACACCTTTCCAGTCATCTGAGGCTCCCTCACTGCTCTGTCCTGACACCCTGGGCGG
CGCCACCATCATCTACCAGCAAGGAGCCGAGGAGTCGACAGCGATGGCCACGCAGACAGCCTTGGATCTT
CTGCTGAACATGAGTGCCCAGCGGGAACTGGGGGGCACAGCCCTGCAGGTGGCCGTGGTGAAGTCGGAGG
ACATGGAAGCAGGGTTAGCATCCCCTGGTGGGCAGCCCTCCCCTGAAGGTGCCACTCCACAGGTGGTCAC
CCTCCATGTGGCAGAGCCGGGGGGCGGTGCGGCAGCCGAGAGCCAGCTAGGCCCTCCTGACCTACCACAG
ATCACCCTGGCACCTGGTCCATTTGGTGGGACTGGCTACAGTGTCATCACGGCACCTATGGAGGAGG
GGACATCAGCTCCTGGCACACCTTACAGCGAGGAACCCTCAGGAGAGGCAGCCCAGACTGTGGTTGTGAG
TGACACCCTAAAAGAAGCTGGCACCCACTACATCATGGCTACTGATGGTACCCAGTTGCACCACATTGAG
CTCACCGCAGATGGCTCCATCTCCTTCCCACCAGATGCTCTGGCCTCTGGTGCCAAATGGCCCCTGC
TGCAGTGTGGGGGGCTGCCTAGAGATGGCCCTGAGCCTCCATCTCCAGCCAACACCCACCGTGTAGGGGA
CCCCCCGAGCTCTGCCTCCCCACCTCCTGCAACCAGCAAAGCCCTGGGCCTGGCAGTGCCCCCCTCACCG
CCGTGTGCAGCCACTGCAGCATCAAAGAAGTTTTCCTGCAAGATCTGTGCCGAGGCCTTCCCTGGCCGAG
CTGAGATGGAGAGTCACAAGCGGGCCCATGCTGGGCCTGGTGCCTTCAAGTGCCCCGACTGCCCCTTCAG
TGCCCGCCAGTGGCCCGAGGTCCGGGCGCACATGGCGCAGCACTCAAGCCTGCGGCCCCACCAGTGTAGC
CAGTGCAGTTTCGCCTCCAAGAATAAGAAGGACCTGCGGCGGCACATGCTGACTCACACCAAGGAGAAGC
CTTTTGCGTGCCACCTCTGCGGGCAGCGTTTCAACCGTAACGGGCACCTCAAGTTCCATATCCAGCGGCT
GCACAGTCCTGATGGGAGGAAGTCAGGAACCCCTACAGCCCGGGCTGCTACCCAGACCCCAACCCAGACC
ATCATCCTGAACAGTGATGACGAAACACTGGCCACCCTGCACACTGCACTCCAGTCCAGTCACGGGGTCC
TGGGCCCAGAGCGGCTACAGCAGGCACTGGGCCAGGAACATATCATCGTTGCCCAGGAGCAGACAGTGAC
CAATCAGGAGGAAGCCACCTACATCCAAGAGATCACCGCAGATGGCCAGACCGTACAGCACCTGGTG
ACCTCCGACAACCAGGTGCAGTATATCATCTCCCAGGATGGTGTCCAGCACCTGCTCCCCCAGGAATATG
TTGTGGTCCCCGAGGGCCATCACATCCAGGTACAGGAGGGCCAGATCACACACATCCAGTATGAACAAGG
AGCCCCGTTCCTTCAGGAGTCCCAGATCCAGTATGTGCCTGTGTCCCCAGGCCAGCAGCTTGTCACACAG
GCTCAACTTGAGGCTGCAGCACACTCGGCTGTCACAGCAGTGGCTGATGCTGCCATGGCCCAAGCCCAAG
GCCTGTTTGGCACAGAGGAGGCAGTGCCTGAACACATTCAACAGCTGCAGCACCAGGGCATCGAGTACGA
CGTCATCACCCTGGCCGAC

>Rhinopithecus bieti XM_017877918.1
ATGGAGGAGAACGAGGTGGAGAGCAGCAGCG
ACGCGGCCCCTGGGCCTGGCCGGCCCGAGGAGCCCTCTGAGAGCGGCCTGGGTGTGGGCACCTCGGAAGC
CGTGTCGGCCGACAGCAGCGACGCCGCAGCCGCCCCGGGGCAGGCAGAGGCCGATGACTCTGGCGTGGGG
CAAAGCTCGGACCACGGCAGCCGCTCTCAGGAGGAGGTATCCGAGAGCAGCTCGAGCGCAGACCCCCTGC
CTAATGGCTGCCTCCCTGATTCATCGTCTGTGTCCCATGGGCCAGTGGCAGGGGTGACANGGGGGCCCCC
AGCACTTGTGCACTCTAGTGCACTCCCAGACCCCAACATGCTGGTGTCAGACTGCACAGCTTCCTCCTCG
GACCTGGGCTCAGCCATCGACAAGATCATCGAATCCACCATCGGGCCCGACCTCATCCAGAGCTGCATCA
CTGTGACCAGTGCCGAGGATGGCGGGGCGGAGACCACGAGGTACCTGATCCTGCAGGGCCCAGATGATGG
AGCTCCCATGACATCACCAATGTCCAGTTCCACCTTGGCCCACAGCCTGGCAGCCATTGAGGCCCTGGCA
GATGGCCCCACATCCACATCCACATGCCTGGAGCCACAGGGTGGGCCCAGCTCCCCGG
TGCAGCTGCCTCCAGCCTCTGGTGCTGAAGAACCAGACCTGCAGAGCCTGGAGGCCATGATGGAGGTGGT
GGTGGTGCAGCAGTTCAAGTGCAAGATGTGCCAGTACCGGAGCAGCACCAAGGCCACACTGCTGCGCCAC
ATGCGGGAGCGCCACTTCCGTCCAGTAGCAGCAGCATCTGGTAAAAAAGGACGTCTACGGA
AGTGGAGCACCTCCACCAAGACCCAAGAGGAAGAGGGACCGGAGGAGGAGGACGATGACGACATTGTAGA
CGCTGGAGCCATTGATGACCTGGAGGAGGATAGCGACTATAATCCAGCTGAGGATGAGCCCCGAGGCCGG
CAGCTTCGGCTCCAGCGCCCCACCCCCAGTACCCCAAGGCCCCGAAGGAGACCTGGCCGGCCCCGGAAGC
TGCCCCGCCTGGAGATCTCAGAAGATGGTGTGGAAGGAGAGCGTCTAGTGAGTTCCCAGAGTGG
ACAGAGCCCTCCAGAGCCACAGGATCCTGAGGCTCCCAGCTCCTCAGGCCCAGGACACCTGGTGACCATG
GGCAAGGCAAGCAGGACCCCTGTGGAAGCTGGTGTGAGCCAGTCAGATGCAGAGAACGCAGCCCCCTCCT
GCCCGGATGAGCATGACACTCCACCGCGGCGCCGAGGTCGACCTTCCAGGCGCTTCCTAGGCAAGAAATA
CCGCAAGTACTATTACAAGTCGCCCAAACCGCTTTTGAGGCCCTTTCTGTGCCGCATCTGTGGTTCTCGC
TTTCTGTCCCACGAGGACCTGCGCTTCCACGTCAACTCCCATGAGGCTGGCGATCCCCAGCTCTTCAAGT
GCCTGCAGTGCAGCTATCGTTCCCGCCGCTGGTCCTCTCTCAAGGAGCACATGTTCAACCACGTGGGCAG
CAAACCCTACAAGTGTGACGAGTGCAGCTACACCAGTGTCTACCGGAAGGACGTCATTCGGCACGCCGCT
GTGCACAGCCGAGACCGGAAGAAGAGGCCAGATCCGACTCCAAAGCTGAGCTCTTTCCCCTGCCCTGTGT
GTGGCCGTGTGTACCCCATGCAGAAAAGACTCACACAGCACATGAAGACGCACAGCACTGAGAAGCCCCA
CATGTGTGACAAGTGTGGAAAGTCCTTTAAGAAGCGTTACACCTTCAAGATGCACCTGCTCACACACATC
CAGGCTGTTGCCAACCGCAGGTTCAAGTGTGAGTTCTGTGAGTTCGTTTGTGAAGACAAGAAGGCACTGC
TGAACCACCAGTTGTCCCACGTCAGTGACAAGCCCTTCAAATGCAGCTTTTGTCCCTACCGCACCTTCCG
AGAGGACTTCCTGCTGTCCCATGTGGCTGTCAAGCACACAGGGGCCAAGCCCTTCGCCTGTGAGTACTGC
CACTTCAGCACACGGCACAAGAAGAACCTACGCCTGCACGTACGGTGCCGACATGCAAGCAGCTTCGAGG
AATGGGGGAGGCGCCACCCTGAGGAGCCCCCCTCCCGCCGTCGCCCCTTCTTCTCTCTGCAGCAGATTGA
GGAGCTGAAGCAGCAGCACAGTGTGGCCCCTGGACCAGCTCCCAGCTCCCCAGGACCTGAGATACCC
CCAGAGGCGACACCTTTCCAGTCATCCGAGGCTCCCTCACTGCTCTGTCCTGACACCCTGGGCGGTGCCA
CCATCATCTACCAGCAAGGAGCCGAGGAGTCGACAGCGATGGCCACGCAGACAGCCTTGGATCTTCTGCT
GAACATGAGTGCCCAGCGGGAACTGGGGGGCACAGCCCTGCAGGTGGCCGTGGTGAAGTCAGAGGACGTG
GAAGCAGGGTTAGCATCCCCTGGTGGGCAGCCCTCCCCTGAAGGTGCCACTCCACAGGTGGTCACCCTCC
ATGTGGCAGAGCCAGGGGGCGGTGCGGCAGCCGAGAGCCAGCTAGGCCCTCCTGACCTACCACAGATCAC
CCTGGCACCTGGTCCATTTGGTGGGACTGGCTACAGTGTCATCACGGCCCCTATGGAGGAGGGGACA
TCAGCTCCTGGCACACCTTACAGCGAGGAGCCCTCAGGAGAGGCAGCCCAGACTGTGGTTGTGAGTGACA
CCCTAAAAGAAGCTGGCACCCACTACATCATGGCTACTGATGGTACCCAGTTGCACCACATTGAGCTCAC
CGCAGATGGCTCCATCTCCTTCCCACCAGATGCTCTGGCCTCTGGTGCCAAATGGCCCCTGCTGCAG
TGTGGGGGGCTGCCCAGAGATGGCCCTGAGCCTCCATCTCCAGCCAACACCCACCGTGTAGGGGACTCCC
CGAGCTCTGCCTCCCCACCTCCTGCAACCAGCAAAGCCCTGGGCCTGGCAGTGCCCCCCTCACCACCGTG
TGCAGCCACTGCAGCATCAAAGAAGTTTTCCTGCAAGATCTGTGCCGAGGCCTTCCCTGGCCGAGCTGAG
ATGGAGAGTCACAAGCGGGCCCATGCTGGGCCTGGTGCCTTCAAGTGCCCCGACTGCCCCTTCAGTGCCC

GCCAGTGGCCCGAGGTCCGGGCGCACATGGCGCAGCACTCAAGCCTGCGGCCCCACCAGTGTAGCCAGTG
CAGTTTCGCCTCCAAGAATAAGAAGGACCTACGGCGGCACATGCTGACTCACACCAAGGAGAAGCCTTTT
GCGTGCCACCTCTGCGGGCAGCGTTTCAACCGTAACGGGCACCTCAAGTTCCACATCCAGCGGCTGCACA
GTCCTGATGGGAGGAAGTCAGGAACCCCTACAGCCCGGGCTGCTACCCAGACCCCAACCCAGACCATCAT
CCTGAACAGTGATGACGAAACACTGGCCACCCTGCACACTGCACTCCAGTCCAGTCACGGGGTCCTGGGC
CCAGAGCGGCTACAGCAGGCACTGGGCCAGGAACATATCATCGTTGCCCAGGAACAGACAGTGACCAATC
AGGAGGAAGCCACCTACATCCAAGAGATCACCGCAGATGGCCAGACCGTACAGCACCTGGTGACCTC
TGACAACCAGGTGCAGTATATCATCTCCCAGGATGGTGTCCAGCACCTGCTCCCCCAGGAATATGTTGTG
GTGCCCGAGGGCCATCACATCCAGGTACAGGAGGGCCAGATCACACACATCCAGTATGAACAAGGAGCCC
CGTTCCTTCAGGAGTCCCAGATCCAGTATGTGCCTGTGTCCCCAGGCCAGCAGCTTGTCACACAGGCTCA
ACTTGAGGCTGCAGCACACTCGGCTGTCACAGCAGTGGCTGATGCTGCCATGGCCCAAGCCCAAGGCCTG
TTTGGCACAGAGGAGACAGTGCCTGAACACATTCAACAGCTGCAGCACCAGGGCATCGAGTACGACGTCA
TCACCTTGGCCGAC
>Galeopterus variegatus XM_008571581.1
ATGGAGGAGAACGAGGTGGAGAGCAGTAGCGACG
CGGCCCATCGGCCTGGCCGGCCCGAGGAGCCCTCCGAGAGTGGCCTGGGTGTGGGCACCTCGGAAGCCGT
GTCTGCAGACAGCAGCGACGCCGCGGCCGTCCCGGGGCCGGCGGAGGCCGACGACTCTGGCGTGGGGCAA
AGCTCGGATCACAGCAGCCGCTCTCCGGAGGAGGTATCTGAGAGCAGCTCAAGCACAGACCCCCTGCCTC
ATGGCTATCTCCCTGATTCATCTTCTGTGTCTCGTGGGCCAGTGGCCGGTGTGACAGGTGGCCCCCCAGC
ACTGGTGCATTCCAGCGCACTCCCAGACCCCAATATGCTGGTGTCTGACTGCACGGCTTCCTCCTCAGAC
CTGGGCTCGGCCATTGACAAGATCATCGAATCCACCATTGGGCCCGACCTTATCCAGAGCTGCATCACTG
TGACCAGTGCTGAGGATGGCGGGGCTGAGACCACACAATACCTGATCCTGCAGGGTCCAGATGATGGTGC
CCCCATGACATCGCCCATGTCCAGTTCCACCCTGACCCACAGCCTGGCAGCCATTGAGGCCCTGGCCGAC
GGCCCAACGTCCACATCCACGTGCCTGGAACCACATGAGGAGGCACAGAGTGGGCCCAGCTCCCCCGTGC
AGCCACCCCCAGCCACTGGCTCTGAGGAGCCAGACCTGCAGAGCTTGGAGGCCATGATGGAGGTTGTAGT
GGTGCAGCAGTTCAAGTGCAAGATGTGTCAGTACCGGAGCAGCACCAAGGCCACACTGCTGCGCCACATG
AGGGAGCGGCACTTCCGCCCAGTAGCAGCAGCAGCTTGTAAGAAGGGACGGCTACGGAAGTGGGGCAGCT
CAACCAAGACCCAGGAGGAAGAGGGGCCAGAGGAGGAGGAGGATGACGACATCGTAGATGCTGGTGCCAT
TGATGACTTGGAGGAGGACAGCGACTATAATCCAGCTGAAGATGAGCCCCGGGGCCGGCAGCTACGACTC
CAGCACCCCACCCCCAGTACCCCAAGACCCCGAAGGAGACCTGGCCGGCCCCGGAAGCTGCCTCGCCTGG
AGACTTCAGACATCCCAGATGGTGTGGAAGGAGACCCTTTTGTGAGTTCCCAGAGTGGACAGAGCCCTCC
AGAGCCACAGGATCCTGAGGCTCCCACCTCCTCAGGCCCAGGATGCCTGGTGGCCCTGGGCAAAACTGGC
AGGGCCCCTGTGGAACCTGGCGTGAGCCAGTCAGACACAGAGAACACAGTGCCCTCCTACCAGGACGAGC
TGGACACGCCGCCCCGCCGCCGTGGTCGACCTTCCAGGCGCTTCCTAGGCAAGAAATACCGCAAGTACTA
TTACAAGTCACCCAAACCGCTGCTGAGGCCCTTCCTGTGCCGTATCTGTGGCTCTCGCTTTCTGTCCCAC
GAGGACCTGCGCTTCCATGTCAACTCCCATGAGGCCGGTGACCCCCAGCTCTTCAAGTGCCTGCAGTGCA
GCTATCGTTCCCGCCGCTGGTCCTCGCTCAAGGAGCACATGTTCAACCATGTGGGCAGCAAGCCCTACAA
GTGTGATGAGTGCAGCTACACCAGTGTCTACCGGAAGGATGTCATCCGGCATGCGGCTGTGCACAGCCGA
GACCGGAAGAAGAGGCCAGATCCGACCCCAAAGCTAAGCTCTTTCCCCTGCCCCGTGTGTGGCCGTGTGT
ACCCCATGCAGAAGAGACTCACACAGCACATGAAGACGCATAGCACTGAGAAGCCCCACATGTGTGACAA
GTGTGGAAAGTCCTTTAAGAAGCGTTACACCTTCAAAATGCACCTGCTTACGCACATCCAGGCTGTTGCC
AACCGCAGGTTCAAGTGTGAGTTCTGTGAGTTTGTTTGTGAGGACAAGAAGGCACTGCTGAATCACCAGC
TGTCCCATGTCAGCGACAAGCCCTTCAAATGCAGTTTTTGTCCCTACCGCACCTTCCGAGAGGACTTCCT
GCTCTCCCATGTGGCTGTCAAGCACACAGGGGCCAAGCCCTTTGCCTGTGAGTACTGCCACTTCAGCACA
CGGCACAAGAAGAACCTCCGCCTACACGTTCGGTGCCGACACGCAAGCAGCTTTGAGGAATGGGGACGGC
GCCATCCTGAGGAGCCCCCCTCTCGCCGCCGCCCCTTCTTTTCTCTGCAGCAGATTGAAGAGCTGAAGCA
GCAGCACAGTGCGGCCCCTGGACCACCCCCCAGCTCCCCAGAACCTCCTGAGATCCCCCCAGAAGCAGCA

CCTTTCCAGTCACCTGAGACGCCCCCGCTGCTCTGTTCTGACACCCTGGGTGGCGCCACCATCATCTACC
AAGAAGGAGCTGAGGAGTCAGCTGCGATGGCCACACAGACAGCCTTGGATCTGCTGTTGAACATGAGCTC
CCAGCGGGAGCTGGGGGGTGCAGCCCTGCAGGTGGCCGTGGTGAAGTCGGAGGACGTGGAAGCAGAGTTG
GCATCCTCTGGTGGTCAGCCCTCCCCCGAAGGTGCCACTTCACAGGTGGTAACCCTCCATGTGGCAGAGC
CCGGGAGCAGTGTGGCAGCTGAGAGCCAGCTAGGCAACCCTGACCTACAGCAGATCACCCTGGCACCCGG
TCCCTTTGGTGGGGCTGGCTATAGCGTCATCACGGCACCTCCTATGGAGGAGGGGACATCAGCTCCTGGC
ACGCCTTACAGCGAGGAGCCCCCAGGTGAGGCAGCTCAGACTGTGGTTGTGAGTGACACCCTAAAAGAAG
CTGCTACCCACTACATCATGGCTGCTGATGGGACCCAGCTGCACCACATTGAGCTGACTGCAGATGGCTC
CATCTCCTTCCCAAGTCCAGATGCTCTGGCCTCTGGAGCCAAGTGGCCTCTGCTGCAGTGTGGGGGGCTG
CCCAGAGATGGCCCTGAGCCTCCATCTCCAGCCAGGACCCACCAGGTGGGGGACCCCCAGAGTTCTGCCT
CCCCACCTCCTGTAGCCAGCAAAGCCCTGGGCCTGGCAGTGCCGCCCTCACCACCATCAGCAGCGTCAAA
GAAGTTTTCCTGCAAGATCTGTGCAGAGGCCTTTCCTGGCCGAGCTGAGATGGAGAGTCACAAACGGGCC
CATGCTGGGCCTAGTGCCTTCAAATGCCCTGACTGCCCCTTCAGTGCCCGCCAGTGGCCTGAGGTCCGGG
CCCACATGGCGCAGCACTCAAGCCTGCGGCCCCACCAGTGCAACCAGTGCAGCTTCGCCTCCAAGAACAA
GAAGGACCTGCGGCGACACATGCTGACCCACACCAAAGAGAAGCCTTTCGCATGCCATCTCTGCGGGCAA
CGTTTTAACCGTAACGGACACCTCAAGTTCCACATCCAGCGGCTGCACAGTCCTGATGGAAGGAAGTCAG
GGACCTCTACAGCCCGGTCTACAGCCCGGACCCCCACCCAGACCATCATCCTGAACAGCGATGACGAGAC
ATTGGCCACACTACACACTGCACTCCAGTCCAGTCATGGGGTCCTGGGCCCAGAGCGACTACAGCAGGCA
CTGAGCCAGGAACACATTATTGTGGCCCAGGAACAGACAGTGACCAATCAGGAAGAAGCCACCTACATCC
AAGAGATCACCACAGCAGATGGCCAAACAGTACAGCACCTGGTGACCTCTGACAACCAGGTACAGTACAT
CATCTCCCAAGACAGTGTCCAGCACCTGCTCCCCCAGGAATACGTTGTGGTCCCTGAAGGCCATCACATC
CAGGTACAGGAGGGTCAGATCACGCACATCCAGTATGAACAAGGGGCCCCGTTCCTCCAGGAGTCCCAGA
TCCAGTATGTGCCTCAGCAGCTGGTCACACAGGCTCAACTTGAGGCTGCAGCACACTCGGCTGTCACAGC
AGTAGCTGATGCTGCCATGGCCCAAGCCCAGGGCCTATTTGGCACAGAGGAGGCAGTGCCTGAACACATC
CAACAGCTGCAGCACCAGGGCATCGAGTACGATGTCATCACTGTGGGCGATGAC
>Myotis lucifugus XM_006091359.3
ATGGAGGAAAACGA
GGTGGAGAGCAGTAGTGACGCGGGCCCGGGGCCTGGCCAGCCCGAGGAGCCCTCTGAGAGCGGCTTGGGT
GTGGGCACCTCGGAAGCCGTGTCCGCCGACAGCAGCGACGCCGCGGCAGCCCCGGGGCCGGCGGAGGCCG
ACGACTCGGAAGTGGGGCAGAGCTCGGACCGCGGCAGCCGCTCTCGGGAGGAGGTATCTGAGAGCAGCTC
AAGCACCGACCCCCTGCCCCATGGCTACCTCCCTGATTCATCTTCTGTGTCCCATGGGCCAGTGGCAGGG
GTGACGGGTGGCCCCCAAACTCTCGTGCACTCCAGCGCACTCCCAGACCCCAACATGCTGGTGTCCGACT
GCACGGCTTCTTCCTCAGACCTGGGCTCAGCCTTTGACAAGATCATCGAGTCCACCATTGGCCAGACCT
CATCCAGAGCTGCATAACTGTAACCAGTGCTGAGGATGGTGGGGCCGAGACCACACAGTACCTGATCCTG
CAGGGACCAGATGATGGTGCCTCCATAGCATCGCCAATGTCCAGTTCCACCCTAGTCCACAGCCTGGCAG
CCATTGAGGCCCTGGCCGACGGCCCCACATCCACATCCACATGCCTGGAGCCATCTGAGGAGGCACGGGG
TTCACCCAGCTCCCCAGCACAGCCTCCCCCAGCCTCTGGCACCGAGGAGCCAGATCTGCAGAGCCTGGAG
GCCATGATGGAGGTGGTGGTAGTACAGCAGTTCAAGTGCAAGATGTGCCAGTACCGGAGTAGCACTAAGG
CCACACTATTACGCCATATGCGGGAGCGGCACTTCCGTCCAGCAGCAGCAGCAGCAGCAGCTGGTAAGAA
GGGACGTCCACGGAAGTGGGGTACCTCGACCAAGACCCAAGAGGAAGAGGCACCAGAGGAGGAAGACGAT
GATGACATTGTAGATGCCGGTGCCATTGATGACCTAGAGGAGGATAGTGACTACAATCCAGCAGAGGATG
AGCCCCGCGGCCGACAGCTGCGGCCCCAGCGCCCCACTCCCAGCACACTGAGACCCCGAAGGAGGCCTGG
CCGGCCCCGGAAGCTGCCTTGCCTGGAGCCCTCAGACCTCCCAGAGGGTATGGAAGGAGAGCCTCTAGTG
AGTTCCCAGAGTGGACAGAGCCCTCCAGAGCCACAGGAACCCGAGGTACCCAGCTCCTCAGGCCCAGGAC
ACCTGGTGGCCGTGGGTAAGGCCGATAGGGTTCCTGTGGAGCCCAATGTGAGCCAGTCGGACGCAGAGAA
CGCAGCACCCACCTGCCAGGAGGAGCCCGACACCCCACCCCGCCGACGTGGCCGACCCTCCAGGCGCTTC
CTAGGCAAGAAATACCGCAAGTATTATTACAAGTCACCCAAGCCGCTTCTGAGGCCCTTCCTGTGCCGCA

TCTGCGGCTCCCGCTTTCTGTCCCATGAGGACCTGCGCTTTCATGTAAACTCCCACGAGGCCGGAGACCC
CCAGCTCTTCAAGTGCCTTCAGTGCAGCTATCGCTCACGCCGCTGGTCCTCGCTCAAGGAGCACATGTTC
AACCATGTAGGCAGTAAGCCCTACAAGTGTGATGAATGCAGCTACACCAGTGTCTACAGGAAAGACGTCA
TCCGGCATGCAGCCGTGCACAGCCGGGACCGGAAGAAGAGGCCAGATCCCACCCCGAAGCTGAGCTCCTT
CCCCTGCCCCGTGTGTGGCCGTGTCTACCCCATGCAGAAGAGACTCACACAGCACATGAAGACACACAGC
ACTGAGAAGCCCCACATGTGTGACAAGTGTGGAAAGTCCTTTAAGAAGCGCTACACCTTCAAGATGCACC
TTCTCACACATATCCAGGCTGTCGCCAACCGCAGATTCAAGTGTGAGTTCTGCGAGTTTGTTTGTGAAGA
CAAGAAGGCGCTGCTGAACCACCAACTGTCCCATGTCAGCGACAAGCCCTTCAAATGCAGCTTTTGCCCC
TATCGCACCTTCCGCGAGGACTTCCTACTATCTCACGTGGCCGTCAAGCACACAGGGGCCAAGCCCTTTG
CTTGTGAGTACTGCCACTTCAGCACACGGCACAAGAAGAATCTGCGCCTGCACGTACGGTGCCGGCATGC
AAGCAGCTTCGAGGAGTGGGGGCGGCGCCACCCGGAGGAGCCCCCCTCCCGCCGTCGCCCATTCTTCTCT
CTGCAGCAGATTGAGGAGCTGAAGCAGCAGCACAGTGTAGCCCCTGGACCAACCCCCAGCCCCCCAGGAC
CTCGCGAGGTTCCCCCAGAGGTAGCCCCTTTCCAGTCACCTGAGACCCCCCCACTGCTCTGTTCTGACAC
CCTGGGTGGCGCCACCATCATCTACCAGCAAGGAGCTGAGGAGTCAACTGCCATGGCCACACAGACAGCT
TTGGATCTGCTGCTAAACATGAGTGCTCAGCGGGAGCTGGGGGGTGCCGCCCTGCAGGTGGCTGTGGTGA
AGTCAGAGGATGCGGAAGCACGGTTAGCATCCCCTGGTGGGCAGCCCTCCTCAGCAAGTGCAGCTCCACA
AGTGGTAACCCTCCACGTCGCAGAGCCGGGGGGCGGTGTAGCTGCTGAGAGCCAGCTAGGCCCCTCTGAC
CTACAGCAGATCACCCTGGCACCCAGTCCATTCAGTGGGGCTGGGTACAGTGTCATCACAGCACCCCCTA
TGGAAGAGGGGACATCAGCTCCTGGCACACCCTACAGTGAGGAGCCCCCAGGGGAGGCAGCCCAGGCTGT
GGTTGTGAGTGACACCCTGAAAGAAGCTGGCACCCACTACATCATGGCAGCCGATGGGACCCAACTGCAT
CATATCGAGCTGACTGCAGATGGCTCCATCTCCTTCCCAAGTTCTGATGCTCTGGCCTCTGGAGCCAAGT
GGCCTCTGCTGCAGTGTGGGGGCCACCCAGAGACGGCCCTGAGCCCTCATCTCCAGCCAGAACCCACCC
AGTAGAAGACCCCCAGGGCTCTGCCTCCCCACCTCCTGCAACTAGTAAAGCCCTGGGCCTGGTAGTGCCC
CCTTCCCCACCATCTGCAGCGTCAAAGAAGTTTTCCTGCAAGATCTGTGCCGAGGCCTTCTATGGCCGAG
CTGAGATGGAGAGTCACAAACGGGCCCATGCCGGGCCGAGTGCCTTCAAGTGCCCAGACTGCCCTTTCAG
CGCCCGCCAATGGCCTGAGGTCCGGGCCCACATGGTGCAGCACTCAAGCCTGCGGCCCCACCAGTGCAGC
CAGTGCAGCTTTGCCTCCAAAAACAAGAAGGACCTGCGGCGGCACATGCTGACCCACACCAACGAGAAGC
CTTTTTCATGCCACCTCTGCGGGCAGCGTTTTAACCGGAACGGGCACCTCAAGTTCCACATCCAGCGACT
ACATAGTCCTGATGGGAGAAAGGCAGGGACCCCTACTGCCCGGGCCCCAGCTGGGACCCCCACCCAGACC
ATCATCCTGAACAGCGATGACGAGACACTGGCCACACTGCACACCGCACTCCAGTCCAGTCATGGGGTCC
TGGGCCCAGAGCAGCTACAGCAGGCACTGGGCCAGGAACACATCATCGTGGCCCAGGAGCAGACAGTGAC
CAATCAGGAGGAAGCCACTTACATACAAGAGATCACCACAGCAGATGGCCAGACGGTACAGCATCTAGTG
ACCTCCGACAACCAGGTACAATACATCATTTCTCAGGACGGAGTTCAGCACCTGCTCCCTCAGGAATATG
TTGTGGTCCCAGAGGGTCATCACATCCAGGTACAGGAGGGCCAGATCACACACATCCAGTACGAACAAGG
AGCTCCATTCCTTCAGGAGTCCCAGATCCAGTATGTGCCTGTGTCCCCAAGCCAGCAGCTCGTCACACAG
GCCCAGCTAGAGGCTGCAGCACACTCAGCTGTCACAGCGGTGGCTGATGCTGCCATGGCCCAAGCCCAGG
GCCTGTTTGGCACAGAGGAGACAGTGCCCGAACACATCCAACAGCTGCAACACCAGGGCATCGAGTACGA
CGTCATCACCCTGACCGATGAC
>cavia porcellus XM_005002799.3
ATGGAGGAGAACGAGGTAGAAAGCAGTAGCGACGCAGCCC
CTCGGTCTGAGGAGCCTACTGAGAGTGGCCTGAGTGTGGGCACCTCGGAAGCTGTGTCAGCCGACAGCAG
CGACGCTGCGACCGCCCCAGTTCCGGTGGAGGCCGATGTCTCTGGAGTGGTGCAAAGCTCAGACCGTGGC
AGCCACTCTCTGGAGGAAGTATCGGAGAGCGGTTCCAGCACAGACACTCTGCCTCATGGCTACCTCCCGG
ATTCATCTTCTGTGTCTGGTGGACCGACGGCAGGGGTCCCCGGAGGCCCCCCAGCACTGGTGCACTCCAG
TGCTCTCCCGGACCCTAACATGCTGGTGTCTGACTGCGCGGCTTCCTCTTCGGACCTGGGCTCAGCCATC
GACAAGATCATCGAGTCCACCATTGGGCCCGACCTCATCCAGAGTTGTATCACCGTGACTAGTGCTGAAG
ACAGCGGAGCTGAGACCACCCAATACCTGATCCTGCAGGGCCCAGACGATGGTGCCCCCATGACATCTCC

AATGTCCAGTTCCACCCTAGGCCACAGCCTAGCAGCCATCGAGGCCCTGGCTGATGGTCCCACATCTACG
TCCACGTGCCTAGAGCCACCTGAGGAGGCACAGGGTGGGCCCGGCGCCCTGGCACAGCCGCCCCCAGCCT
CTGGCACCGAGGACCTGGACCTGCAGAGCCTGGAAGCCATGATGGAGGTGGTGGTGGTGCAGCAGTTCAA
GTGCAAAATGTGCCAGTACCGGAGCAGCACCAAGGCCACGCTGCTGCGGCACATGCGGGAGCGGCACTTC
CGCCCCGCCACAGCAGCAGGAGCTAACAAGAAAGAACGGCAGCGGAAGTGGGACCCAGCAGCCAAGACCC
GGGAGGAAGAAGGGCCAGAAGAGGAGGAGGAGGATGACATCGTGGATGCTGGTGCCATTGACGACCTGGA
GGAGGACAGTGACTACAATCCTGCCGAGGATGAGCCCCGAGGCCGGCAGCTGCGGGTCCAGCGTCCCACC
CCCAGTACCCCAAGGCCACGAAGGAGACCTGGCCGGCCCCGGAAAGTGCCTCGCCTGGAGACCTCAGACC
TCCCGGATGGTGTGGAAGAGCCTCTGGTGAGTTCTCAGAGTGGAGAGAGCCCCACAGAGCCACAAGATCC
CGAGGCTTCCAGCTCCTCGGGCCCAGGATGCCTGGTAGCCCTGGGCAGAGCCAACAAGGCACCCATGGAG
TCCAGCGTGAGTCAGTCAGACTCAGAGAACACGGCACCCTCCTGTCAAGAAGAGCCCGATGCCCCACCCC
GCCGCCGGGGCCGACCCTCCAGGCGATTCCTGGGCAAGAAATACCGCAAGTACTATTACAAGTCGCCCAA
GCCACTGCTGAGGCCCTATCTGTGCCGCATCTGTGGCTCCCGCTTCCTGTCCCACGAGGACCTGCGCTTC
CATGTCAATTCCCATGAGGCTGGTGACCCCCAGCTCTTCAAGTGCCTACAGTGTAGCTACCGCTCCCGCC
GCTGGTCCTCGCTCAAGGAGCACATGTTCAACCACGTGGGCAGCAAACCCTACAAATGTGACGAATGCAG
CTACACCAGTGTCTACCGGAAGGACGTCATTCGCCACGCTGCTGTGCACAGCCGGGACCGGAAGAAGAGG
CCAGATCCGACCCCAAAGCTGAGTTCTTTCCCCTGCCCTGTGTGTGGCCGCGTGTACCCCATGCAGAAGA
GACTGACGCAGCACATGAAAACTCACAGCACTGAGAAGCCCCACATGTGTGACAAGTGCGGGAAGTCCTT
TAAGAAGCGCTACACCTTCAAAATGCATCTGCTCACACACATCCAGGCTGTTGCCAACCGCAGGTTCAAG
TGCGAGTTCTGTGAGTTTGTTTGTGAGGACAAGAAGGCACTGCTGAATCACCAGCTGTCCCACGTCAGTG
ACAAGCCCTTCAAATGCAGCTTTTGTCCCTACCGTACCTTCCGAGAGGACTTCCTGCTGTCCCATGTGGC
CGTCAAGCACACAGGGGCGAAGCCCTTTGCCTGTGAGTACTGCCACTTCAGCACCAGGCACAAGAAGAAT
CTCCGCCTGCACGTCCGGTGCCGACATGCAAGCAGCTTTGAAGAATGGGGGAGGCGCCACCCTGAGGAGC
CCCCTTCCCGCCGCCGTCCCTTCTTCTCTCTGCAACAAATCGAGGAGCTAAAGCAGCAGCATAGTGCGGC
CCCCGCACCACCCCCCACCTCCCCAGGACCTCCTGAGCTCCCTCCAGAGGCTGCACCTTTCCAGTCACCC
GAGACCCCCCCACTGCTCTGTCCTGACACCCTGGGCGGTGCCACCATCATCTACCAGCAAGGAGCCGAGG
AGTCCACTGCGATGGCTACACAGACAGCCTTGGACCTGCTGCTGAACATGAGCGCTCAGCGGGAGCTGGG
GGGCACAGCCCTGCAGGTAGCTGTGGTGAAATCAGAGGACATGGAAGCAGAGCTGGCGCCCTCTGGTGGG
CAGCCCTCCCCCGAAGACACCACTCCACAGGTGGTAACCCTCCATGTGGCAGAGCCAGGGGACACTGTGG
CAACCGAGAGCCAGCTAGGTCCCCCTGACCTACAGCAGATCACCTTGGCACCTGGTGCATTTGGTGGAGC
TGGCTACAGCGTCATCACAGCACCCCCAATGGAGGAGGGCACATCAGCGCCTGGCACCCCTTACAGTGAG
GAGCCCCCAGGGGAGGCAGCCCAGGCTGTGGTTGTGAGTGACACCCTTAAAGAAGCTGGCACCCACTACA
TCATGGCAGCAGACGGAACCCAGCTGCACCACATCGAGTTGACTGCAGATGGCTCCATCTCCTTTCCCAG
TCCGGATTCCCTGACCTCTGGAGCCAAGTGGCCCTTATTGCAGTGTGGGGGACTGCCCAGAGATGGCCCT
GAGCCCCCATCTCCAGCCAAGACCCACAGGGCGGGAGACCCCCCAAGCTCTGCCTCCCCACCTCCTGCAG
CCAGCAAAACCCTTGGCCTGGTAATGCCACCTTCCCCGCCATCTGCAGCCACTGCAGCATCCAAGAAGTT
TTCCTGCAAGATCTGTGCCGAGGCCTTCCCTGGCCGAGCAGAGATGGAAAGTCACAAACGGGCCCACGCT
GGGCCTAGTGCCTTCAAGTGCCCTGACTGCCCCTTCAGTGCCTGCCAGTGGCCGGAGGTCCGGGCCCACA
TGGCGCAGCACTCGAGCCTGCGGCCTCACCAGTGCAGCCAGTGCAGTTTCGCCTCCAAGAACAAGAAGGA
CTTGCGGCGGCACATGCTGACCCACACCAATGAGAAGCCTTTCTCATGCCACCTCTGTGGGCAGCGTTTC
AACCGCAACGGGCACCTCAAGTTTCACATCCAGCGGCTGCATAGCCCTGATGGAAGGAAAGCGGGGACCC
CTACAGCCCGGGCCCCGGCCCAGACGCCCACTCAGACCATCATCTTAAACAGTGACGAGGAGACACTGGC
CACACTGCACAGTGCCCTCCAGTCCAGTCATGGGGTCCTGGGCCCAGAGCGGCTGCAGCAGGCACTGGGC
CAGGAACATATCATTGTGGCCCAGGAACAGACAGTGACCAATCAGGAAGAAGCCACTTATATCCAAGAGA
TCACCACAGCAGACGGCCAGACGGTACAGCACCTGGTGACCTCTGACAACCAGGTACAGTACATCATCTC
TCAAGATGGTGTCCAGCACCTGCTGCCCCAGGAATATGTCGTGGTCCCTGATGGCCATCACATTCAGGTA
CAGGAGGGCCAGATTACACACATCCAGTATGAACAAGGAGCCCCATTTCTGCAGGAGTCCCAGATCCAGT

ATGTACCTGTGTCCCCAGGCCAGCAGCTTGTCACTCAGGCTCAGCTTGAGGCCGCAGCGCACTCTGCTGT
CACAGCAGTGGCTGATGCTGCCATGGCCCAGGCCCAGGGCTTGTTTGGCACAGAAGAGGCAGTGCCTGAA
CACATTCAGCAGCTGCAGCACCAAGGCATCGAGTACGACGTCATCACCCTGACTGAAGAC
>RABBIT ENSOCUT00000016078
ATGGAGGAGAACGAGGTGGAGAGCAGTAGCGACGCGGCCCCTCGGCCCGGCGGGCCGGAG
GAGCCCTCCGAGAGCGGCCTGGGAGTGGGCACCTCGGAAGCCGTGTCCGCGGACAGCAGC
GACGCGGCGGCCGGCCCGGGGCCGGCGGAGGCCGACGACTCTGGCGTGGGACAGAGCTCG
GACCGCGGCAGCTGTCTGCAGGAGGAGGTATCGGAAAGCAGCTGCAGCGCTGACCCTCTG
CCCCACGGCTACCTCCCCGACTCCTCCTCTGTGTCCCGGGAGCCGGTGGCAGGCGTGGCG
GGCGGCCCCCCGGCGCTGGTGCACTCCAGCGCGCTCCCGGACCCCAGCACGCTGGCCTCC
GACTGCACGGCCTCCTCCTCGGATCTGGGCTCGGCCATCGACAGGATCATCGAGTCCACG
CTTGGGCCCGACGTGATCCAGGGCTGCATCACTGTGACCAGTGCAGAAGATGGGGGAGCT
GAGACCACCCGGTACCTGCTCCTGCAGGGCCCAGACGATGGAGCCCCCATGACATCACCG
ATGTCGACTTCCACCCTGGCCCACAGCCTGGCAGCCATCGAGGCCCTGGCCGACGGCCCC
ACGTCCACGTCCACCTGCCTCGAGCCACCTGAGGAGGTGCGGGGCGGGCCCAGCTCGCCA
GCACAGCCACCCCCGGCCTCTGGCGCGGAGGAGCCAGACCTGCAGAGCCTGGAGGCCATG
ATGGAGGTGGTGGTGGTGCAGCAGTTCAAGTGCAAGATGTGCCAGTACCGGAGCAGCACC
AAGGCCACGCTGCTGCGCCACATGCGCGAGCGCCACTTCCGGCCAGCATCCGCAGCCACA
GCCGCAGCCGGCAAGAAGGGCCGTGTGCGGAAGTGGGGCGCTGTGACCAGGACCCAGGAG
GAGGAGGGGCCGGAGGAGGAGGAGGACGACGACATAGTAGACGCTGGCGCCATTGACGAC
CTAGAGGAGGACAGCGACTACAACCCAGCCGAGGACGAGCCCCGAGGGCGGCAGCTGCGG
CCCCAGCGCCCCAGCCCCAGCACCCCCAGAACCCGAAGGAGACCCGGCAGGCCCCGGAAG
GTGCCCCGCCTGGAGACGTCGGACCTGCCGGACGGTGTGGACGCAGAGCCTCTGGTGAGC
TCCCAGAGTGGACAGAGCCCGCTGGCGCCACAGGACCCCGAGGCTCCCAGCTCCTCGGGC
CCCGGACACCTGGGCGGCCGGGCCCTCCTGGAGCCGGGGGTGAGCCAGTCGGACGCCGAG
AACGCGGCGCCCGGCTGCCCTGACGAGCCCGACGCCCCGCCCCGCCGCCGGGGGCGGCCC
TCCAGGCGCTTCCTGGGCAAGAAGTACCGCAAGTACTACTACAAGTCGCCCAAGCCGCTC
CTAAGGCCCTACCTGTGCCGCATCTGCGGCTCTCGCTTCCTGTCGCACGACGACCTGCGC
TTCCACGTCAACTCCCACGAGTCCGGCGACCCCCAGCTCTTCAAGTGCCTGCAGTGCAGC
TACCGCTCCCGCCGCTGGTCCTCGCTCAAGGAGCACATGTTCAACCACGTGGGCAGCAAA
CCCTACAAGTGTGACGAGTGCAGCTACACCAGTGTCTACCGGAAGGACGTCATCCGGCAC
GCGGCCGTGCACAGCCGGGACCGGAAGAAGAGGCCGGATCCGACCCCGAAGCTGAGCTCC
TTCCCCTGTCCCGTGTGTGGCCGGGTGTACCCCATGCAGAAGAGACTCACGCAGCACCTG
AAGACACACAGCACGGAGAAGCCCCACATGTGCGACAAGTGTGGCAAGTCCTTTAAGAAG
CGCTACACCTTCAAGATGCACCTGCTCACGCACATCCAGGCGGTCGCCAACCGCAGGTTC
AAGTGCGAGTTCTGCGAGTTCGTCTGCGAGGACAAGAAGGCGCTGCTGAACCACCAGCTG
TCACACGTCAGCGACAAGCCCTTCAAGTGCAGCTTCTGTCCCTACCGCACCTTCCGCGAG
GACTTCCTGCTGTCCCACGTGGCGGTCAAGCACACAGGGGCCAAGCCCTTCGCCTGTGAG
TACTGCCACTTCAGCACGCGGCACAAGAAGAACCTGCGTCTCCACGTGCGGTGCCGGCAC
GCGAGCAGCTTCGAGGAGTGGGGGCGGCGCCACCCCGAGGAGCCCCCGTCCCGCCGGCGC
CCCTTCTTCTCTCTGCAGCAGATTGAGGAGCTGAAGCAGCAGCACCGTGCAGCCCCCGCG
CCGCCCCCCAGCCCCCCGGGACCTGCCGAGATCGCTCCAGAGGCAGCACCTCTGCAGTCC
CCGGAGACCCCACCCCTGCTCTGCTCCGACACCCTGGGTGGCGCCACCATCATCTACCAG
CAAGGAGCCGAGGAGTCCACTGCGATGGCCACGCAGACAGCCTTGGATCTGCTGCTGAAC
ATGAGCGCTCAGCGGGAGCTGGGGGGCGCGGCCCTGCAGGTGGCCGTGGTGAAATCGGAG
GATGTGGGAGCAGGGCTGGCATCCCCTGGTGGGGAGCCCTCCCCCGAAGGCACCGCTCCA
CAGGTGGTCACCCTCCACGTGGCAGAGCGGGGGACTGGCGTGGCAGCTGAGAGCCAGCTG

GGCTCCCCTGACCTGCAGCAGATCACCCTGGCCTCCGGTCCCTTTGGTGGGGCTGGCTAC
AGTGTCATCACAGCTCCCCCTATGGAGGAGGGGACATCAGCTCCTGGTACACCCTACAGC
GAGGAGCCTGCAGGGGAGGCAGCCCAGGCTGTGGTTGTGAGCGACACCCTGAAAGAAGCC
AGCACCCACTACATCATGGCCGCCGACGGCACCCAGCTGCACCACATCGAGCTGACTGCA
GATGGCTCCATCTCCTTCCCAAGTCCGGATGCCCTGGCCTCTGGAGCCAAGTGGCCCCTG
CTACAGTGTGGGGGTCTTCCCAGAGACGGCCCTGAGCCCCCTTCTCCGGCCAAGACCCAC
CAGGCGGGGGACTGTGAGAGCTCTGCCTCCCCTCCTCCTGCAGCCAGTAAAGCCCTGGGC
CTGGCGGTGCCCCCCTCGCCCCCGTCTGCAGCCGCCACGGCGTCAAAGAAGTTTTCCTGC
AAGATCTGTGCTGAGGCCTTCCCGGGCCGGGCTGAGATGGAGAGTCACAAACGGGCCCAC
GCCGGGCCCAGTGCCTTCAAGTGTCCGGACTGCCCCTTCAGTGCTCGCCAGTGGCCCGAG
GTCCGGGCCCACATGGCGCAGCACTCCACCCTGCGGCCCCACCAGTGCAGCCAGTGCAGC
TTCGCCTCCAAGAACAAGAAGGACCTGCGGCGGCACGTGCTCACCCACACCAACGAGAAG
CCTTTCGCCTGCCACCTGTGTGGACAGCGCTTCAACCGTAATGGGCACCTTAAGTTTCAC
ATCCAGCGGCTGCACAGTCCTGATGGGAGGAAATCAGGGACCCCAACAGCCAGGACCCCC
ACGCGGACCCCAACACAGACCATCATCCTAAACAGTGACGAGGAGACACTGGCCACACTG
CACACGGCCCTGCAGTCGGGTCACGGAGTCCTGGGCCCAGAGCGGCTACAGCAGGCACTA
GGCCAGGAACACATCATCGTGGCCCAAGAACAGACGGTGACCAATCAGGAGGAAGCAACG
TACATCCAGGAGATCACCACGGCTGACGGCCAGACGGTGCAGCATCTGGTGACCTCCGAC
AACCAGGTGCAGTACATCATCTCCCAGGATGGGGTCCAGCACTTGCTCCCTCAGGAATAC
GTCGTGGTGCCTGAAGGCCATCATATCCAGGTGCAGGAGGGTCAGATCACGCACATCCAG
TATGAACAAGGGACCCCATTCCTTCAAGAGCCCCAGATCCAGTATGTGCCTGTGTCCCCA
GGCCAGCAGCTTGTCACACAGGCACAACTTGAGGCTGCCGCACACTCGGCTGTCACAGCG
GTGGCCGACGCTGCCATGGCTCAAGCCCAGGGCCTGTATGGCACAGAGGAGACAGTGCCT
GAGCACATTCAACAGCTGCAGCACCAGGGCATTGAGTACGATGTCATCACCCTGACGGAC
GAA
>Fukomys damarensis XM_010626203.3
ATGGA
GGAGAACGAGGTAGAGAGCAGTAGCGACGCGGCCCCTAGGCCCGAGGAACCCTCTGAGAGCGGCTTGGGT
GTGGGCACCTCGGAAGCTGTGTCAGCCGACAGCAGCGACACCGCGACAGCCCCAGGTCTAGTGGAGGCCG
ACGTCTCTGGAGTGGTGCAAAGTTCAGACCGTGGCAGCCACACACTGGAGGAGGTATCCGAGAGCAGTTC
AAGCACAGACCCTCTGCCTCATGGCTACCTCCCTGATTCATCTTCTGTGTCTGGCGGGCCAATAGCAGGA
GTCCCTGGAGGTCCCCCAGCACTGGTACACTCCAGTGCTCTCCCAGACCCCAACATGCTGGTGTCTGACT
GTGCGGCTTCCTCCTCGGACCTCGGCTCAGCCATTGACAAGATCATCGAGTCCACCATCGGGCCTGACCT
CATCCAGAGTTGTATAACTGTGACCAGTGCTGAAGATGGCGGGGCTGAGACCACGCGGTACCTGATCCTG
CAGGGTCCAGATGATGGTGCCCCCATGACATCTCCAATGTCGAGTTCCACCTTGGGCCACAGCCTGGCAG
CCATTGAGGCCTTGGCCGATGGCCCCACATCCACATCCACATGCCTGGAGCCACCTGAGGAGGTGCAGGG
CGGGGCCGGCTCCCTGGCACAGCCACCCGCAGCCTCTGGCACCGAGGAGCTGGACCTGCAGAGCCTGGAA
GCCATGATGGAAGTGGTGGTGGTGCAGCAGTTCAAGTGCAAGATGTGCCAGTACCGGAGCAGCACCAAGG
CCACACTGCTGCGGCATATGCGAGAGCGGCACTTCCGCCCTGCAGCAGCAGCAGGAGCAAGAGCTGGTAA
GAAAGAACGACAACGGACGTGGGACACCTCAACCAAGACCCAGGAGGAAGAAGGGCCAGAGGAGGAAGAG
GAGGATGACATTGTAGATGCTGGTGCCATCGATGACCTGGAGGAGGACAGTGACTACAATCCTGCTGAGG
ATGAGCTCCGAGGCCGGCAGCTGCGGCTTCATCGCCCCACCCCTAGTACCCCAAGGCCACGAAGGAGACC
TGGCAGGCCTCGGAAGCTGCCTCGCCTGGAGACCTCAGACCTCCCAGATGGTGTGGAAGAGCATCTAGTA
AGTTCCCAGAGTGGACAAAGCCCTGCAGAGCCGCAAGATCCTGAGGCTTCCAGCTCCTCAGGTCCAGGGT
GCCTAGTGGCCCTGGGCAAGGCCGGCAAGGCCCCCATGGAGCCTGGTGTGAGCCAGTCAGACTCGGAGAA
CACAGCACCCTCCTGCCAAGAGGAGCCTGATGCCCCACCCCGCCGCCGGGGCCGACCCTCCAGGCGATTC
CTGGGCAAGAAATACCGCAAGTACTATTACAAGTCGTCCAAGCCACTTCTGAGGCCCTATCTGTGCCGCA

TCTGTGGCTCCCGCTTCCTGTCCCATGAGGACCTGCGCTTCCATGTCAACTCCCACGAGGCTGGTGACCC
CCAGCTCTTCAAGTGCCTGCAGTGCAGCTATCGCTCCCGCCGCTGGTCCTCACTCAAGGAGCACATGTTC
AACCATGTGGGCAGCAAGCCCTACAAGTGTGATGAATGCAGCTACACCAGTGTCTACCGGAAGGACGTCA
TTCGCCATGCGGCAGTGCACAGCCAAGACCGGAAGAAGAGGCCAGATCCGACACCAAAGCTGAGTTCTTT
CCCCTGCCCTGTGTGTGGCCGTGTGTATCCTATGCAGAAGAGACTGACACAGCACATGAAGACACATAGC
ACTGAGAAGCCCCACATGTGTGACAAGTGTGGAAAGTCCTTTAAGAAGCGCTACACCTTCAAAATGCACC
TCCTCACGCACATCCAGGCAGTCGCCAACCGCAGGTTCAAGTGCGAATTCTGCGAGTTTGTTTGTGAGGA
CAAGAAGGCCCTGCTGAACCATCAGCTGTCCCATATCAGTGACAAGCCCTTCAAATGCAGCTTTTGTCCC
TATCGTACCTTCCGAGAGGACTTCCTGCTGTCCCACGTGGCTGTAAAGCACACAGGCGCCAAGCCCTTCG
CCTGTGAGTACTGCCACTTCAGCACCAGGCACAAGAAGAATCTCCGCCTGCACGTCCGGTGCCGACATGC
AAGCAGCTTTGAGGAATGGGGGCGGCGCCACCCCCAGGAGCCCCCTTCCCGCCGCCGCCCCTTCTTCTCT
CTGCAGCAGATTGAGGAACTGAAGCAGCAGCACAGTGCGGTCCCTGCGGCAACCCCCAGGTCCCCAGGAC
CTCCTGAGGTCCCTGCAGAGGATGCACCTTTCCAGTCACCTGAGACCCCCCCACTACTCTGTTCTGACAC
CCTGGATGGCACCACCATCATCTACCAGCAAGGAGCTGAGGAGTCCACTGCGATGGCTACACAGACAGCC
TTGGACCTGCTGCTCAACATGAGTGCTCAGCGGGAGCTGGGGGGCACAGCGCTGCAGGTAGCCGTGGTGA
AATCAGAGGACATGGAAGCAGAGTTAGCATCTCCTGGTGGGCAGCCCTCCCCTGAAGATACCACTCCACA
GGTGGTAACCCTCCATGTGGCAGAGCCAGGGGACAGTGTGGCACCTGAGAGCCAACTAGGCCCCCCTGAC
TTACAGCAGATCACCTTGGCACCTGGTGCATTTGGTGGAGCTGGGTACAGCGTCATCACAGCACCCCCTA
TGGAGGAGGGGACATCAGCTCCTGGCACACCTTACAGTGAGGAGCCCCCAGGGGAGGCAGCCCAGGCTGT
GGTTGTGAGCGACACCCTCAAAGACGCGGGCACCCACTACATCATGGCAGCTGATGGAACCCAGCTGCAC
CACATTGAGTTGACTGCAGATGGTTCCATCTCCTTCCCCAGTCCGGATTCCCTGACCACTGGAGCCAAGT
GGCCCCTGCTACAGTGTGGGGGAATGCCCAGAGATGGCTGTGTGCCCCCATCTCCAGCCAAGACCCACTG
GGTGGGGGACCCCCCGAGCTCTTCCTCCCCACCTCCTGCAGCCAGCAAAGCCCTGGGCCTGGTAGTGCCC
CTCTCCCCACTGTCTGCAGCCAATGCTGCATCAAAGAAATTTTCCTGCAAGATCTGTGCCGAGGCTTTCC
CTGGCCGAGCAGAGATGGAGAGTCACAAACGGGCCCACGTAGGGCCTAGTGCCTTCAAGTGCCCTGACTG
CCCCTTCGGTGCCCACCAATGGCTGGAGGTCCGGGCTCACATGGCACAGCACTCGAGCCTACGGCCTCAC
CAGTGTAGCCAGTGCAGTTTCGCCTCCAAGAACAAAAAGGACTTGCGGCGGCACATGCTGACCCACACCA
ATGAGAAGCCTTTCTCGTGCCACCTCTGTGGGCAGCGTTTCAACCGCAATGGGCACCTGAAGTTCCACAT
CCAGCGGCTGCACAGCCCCGATGGGAGGAAAGCAGGAACCCCTACAGCCCGGGCCCCAGCCCAGACCCCC
ACGCAGACCATCATCTTGAATAGTGATGAGGAGACACTGGCCACACTGCATACTGCCCTCCAGTCCAGTC
ATGGGGTCCTGGGCCCAGAGCGGTTACAGCAGGCATTGGGCCAGGAACACATCATTGTGGCCCAGGAACA
GACTGTGACCAATCAGGAAGAAGCCACTTATATCCAAGAGATCACCACAGCAGATGGCCAGACAGTACAG
CACCTCGTAACTTCTGACAACCAGGTACAGTATATCATCTCTCAGGACAGTGTCCAGCACCTATTGCCCC
AGGAGTACGTTGTGGTCCCTGACGGCCATCATATCCAGGTACAGGAGGGCCAGATTACACATATCCAGTA
TGAACAAGGAGCCCCATTCCTACAGGAATCCCAGATCCAGTATGTGCCTGTGTCCCCAGGCCAGCAGCTT
GTCACACAGGCTCAGCTTGAGGCTGCAGCACACTCTGCCGTCACAGCAGTGGCTGATGCTGCCATGGCTC
AAGCCCAGGGCCTGTTTGGCACAGAGGAGGCGGTACCTGAACACATTCAACAGCTGCAGCACCAAGGCAT
CGAGTACGACGTCATCACCCTGACCGATGAC
>Ictidomys tridecemlineatus XM_013358602.2
ATGGAGGAGAACGAGGTGGAGAGCAGTAGCGACGCCGCCCCTCGTCCTGGGCGGCC
CGAGGAACCTTCGGAGAGCGGCCTGTGCGTGTGCACTTCAGAAGCCGTGTCTGCCGATAGCAGCGATGCC
GCGTCCGTCCCAGGACCCGGGGAGGCCGACGACTCTGGCGTGGGGCAAAGCTCAGACCGCGGAGGCCGCT
CTCTGGAAGAGGTGTCTGAGAGCAGCTCAAGCACCGACCCTCTGCCTCATGGCTACCTCCCTGATTCGTC
TTCCGTGTCCCGTGGGCCTGTGGCAGGGGTGCCAGGCGGGCCGCCAGCACTGGTGCACTCCAGCGCGCTC
CCCGACCCCAGCATGCTGGTGTCCGACTGCGCAGCCTCCTCCTCAGACCTGGGCTCTGCCATCGACAAGA
TCATCGAGTCCACCATCGGGCCCGACCTCATCCAGAGCTGCATCACTGTGACCAGTGCTGAAGATGCTGG
GGCTGAGGCCACTCGCTATCTGATCCTGCAGGGCCCTGATGATGGCGCCCCTATGACATCACCAATGTCC

AGTTCCACCCTGGCCCGCAGCCTGGCCGCCATCGAGGCCCTGTCAGATGGCCCCACGTCCACATCCACAT
GCCTGGAGCCACCGGAGGAGGTGCAGGGTGGGGCCAGCTCCCTGGCACAGCTGCCCCCGGCCTCTGGCAC
TGAGGAGCTGGACCTCCAGAGCCTGGAGGCCATGATGGAGGTGGTGGTGGTGCAGCAGTTCAAGTGCAGG
ATGTGCCAGTACCGCAGCGGCACCAAGGCCACGCTGCTGCGGCACATGCGGGAGCGGCACTTCCGCCCAG
CAGCAGCAGCAGCAGCAGCCGGTAAGAAGGGACGTCTCCGGAAATGCAGCCCATCGACCAAGAGCCAGGA
GGAGGAGGGGCCCGAGGAGGAGGAGGAGGATGACATTGTAGATGCCGGTGCCATTGATGACCTGGAGGAG
GACAGCGACTACAATCCAGCTGAGGATGAGCCCCGAGGCCGGCAGCTTCGGCTCCAGCGTCCCACACCCA
GTTCCCCAAGACCACGCCGGAGACCTGGCCGGCCCCGGAAGCTGCCTCGCCTGGAGACCTCAGACCTGAC
TGCTGGTGGGGGAGATCCTCTCGTGAGCTCGCAGAGCACCAAGAGCCCTCTGGGGCCACCAGATCCTGAG
GCTCCCAGCTCCTCAGGCCCCGGACACCTGGAGGCCCTGGGCAAGGCCGGCAAGGTCACCATGGAGCCTG
GTGTGAGCCAGTCCGACACAGAGATTGCAGCCCCATCCTGCCAGGAGGAGCCCGATGCCCCACCGCGCCG
CCGTGGTCGACCTTCCCGGCGATTTCTGGGCAAGAAATACCGAAAGTATTATTACAAGTCACCCAAACCG
CTTCTGCGGCCCTATCTGTGCCGCATTTGTGGCTCACGCTTCCTGTCCCATGAGGACCTGCGCTTCCACG
TCAACTCCCACGAGGCTGGTGACCCCCAGCTCTTCAAGTGCCTGCAGTGCAGCTACCGCTCCCGCCGCTG
GTCCTCACTCAAGGAGCACATGTTCAACCACGTGGGCAGCAAGCCCTATAAGTGTGACGAGTGTAGCTAC
ACCAGTGTCTACCGCAAGGACGTCATTCGGCATGCGGCTGTTCACAGCCGAGACAGGAAGAAGAGGCCAG
ACCCGACCCCAAAACTGAGCTCTTTCCCCTGCCCTGTGTGCGGCCGCGTGTACCCCATGCAGAAGAGACT
GACACAGCACATGAAGACGCATAGCACTGAGAAGCCCCACATGTGTGACAAGTGTGGAAAGTCCTTTAAG
AAGCGCTACACCTTCAAAATGCACCTGCTCACACACATCCAGGCTGTCGCCAACCGCAGGTTTAAGTGTG
AGTTCTGTGAGTTCGTCTGTGAGGACAAGAAGGCCCTGCTGAACCATCAGCTCTCCCATGTCAGCGACAA
GCCCTTCAGATGCAGCTTTTGTCCCTACCGTACCTTCCGGGAGGACTTCCTGCTGTCCCACGTGGCTGTC
AAGCACACAGGAGCCAAGCCCTTTGCCTGTGAGTACTGCCACTTCAGCACGCGCCACAAGAAGAACCTGC
GCCTGCACGTGCGCTGCCGCCACGCGAGCAGCTTCGAGGAGTGGGGGCGGCGCCACCCCGAGGAGCCCCC
CTCCCGCCGCCGCCCCTTCTTCTCCCTGCAGCAGATCGAGGAACTGAAGCAGCAGCACAGTGCAGCCCCT
GGGCCACCCCCCGGCTCCCCGGGGCCTCCTGAGATCCCCCCGGAGGCAGCACCTTTCCAGGCTCCTGAGA
CTCCCCCACTTCTCTGTTCTGACACTCTGGGCAGTGCCACCATCATCTACCAGCAAGGAGCTGAGGAGTC
CACGGCAATGGCCACCCAGACGGCTCTGGATCTGCTGCTGAACATGAGTGCCCAGCGGGAGCTGGGGGGC
ACAGCCCTGCAGGTGGCCGTGGTGAAGTCACAGGACGTGGAAGCAGGCTTAGCGTCTTCTGGTGGGCAGC
CCTCCCCCGAAGACACCACTCCACAGGTGGTGACCCTCCATGTGGCAGAGCCTGGGGGCAATGTGGCTGC
TGAGAGCCAGCTAGGCACTCCTGATCTACAGCAGATCACCTTGGCACCTGGTCCATTTGGTGGAGCTGGC
TACAGTGTCATCACAGCACCCACCATGGAGGAGGGGACATCGGCTCCTGGCACACCTTACAGTGAAGAGC
CCCCAGGGGAGGCAGCCCAGGCTGTGGTTGTAAGTGACACCCTCAAAGAAGCTGGCACCCACTACATCAT
GGCTGCTGATGGGACCCAGTTGCACCACATCGAGTTGACTGCAGATGGCTCCATCTCCTTCCCCAGCTCA
GATGCCCTGGCCTCTGGAGCCAAGTGGCCCCTTCTGCAGTGTGGGGGGCTGCCCAGAGATGGTTCCGAGC
CACCGTCTCCAGCCAAGACCCTCCGGGTAGGGGACTCCCAGCATTCTGCCTCCCCACCTCCTGCAGCCAG
CAAAACCCTGGGCTTGGCAGTGCCCCCCTCCCCACCATCTGCAGCCACTGCAGCATCAAAAAAGTTTTCC
TGCAAGATTTGTGCTGAGGCCTTCCCTGGCCGAGCTGAGATGGAGAGTCACAAACGGGCCCATGCTGGGC
CAGGCACCTTCAAGTGCCCTGACTGCCCCTTCAGTGCCCGCCAGTGGCCCGAGGTCCGGGCCCACATGGC
ACAGCACTCGAGCTTGCGGCCCCACCAGTGCAGCCAGTGCAGCTTCGCCTCGAAGAACAAGAAGGACCTG
CGGCGGCACATGCTGACCCACACCAACGAGAAGCCTTTCTCGTGCCATCTCTGTGGGCAGCGTTTCAACC
GTAACGGGCACCTCAAGTTCCACATCCAGCGGCTGCACAGTCCTGACGGGAGGAAGTCTGGCACCCCAAC
AGTCCGCACCCCAGCAAGGACCCCCACCCAGACCATCATCCTGAATAGTGACGACGAGACGCTGGCCACG
CTGCATACTGCCCTCCAGTCCAGTCACGGGGTCCTGGGCCCAGAGAGGCTACAGCAGGCCCTGGGCCAGG
AACACATTATTGTGGCCCAGGAACAGACAGTGACCAATCAGGAGGAAGCCACCTACATCCAAGAGATCAC
CACAGCAGATGGTCAGACGGTACAGCACCTGGTGACCTCGGACAACCAGGTACAGTATATCATCTCTCAG
GATGGTGTCCAGCACCTGCTGCCCCAGGAATATGTTGTGGTCCCTGATGGCCACCACATCCAGGTACAGG
AGGGTCAGATCACACACATCCAGTATGAACAAGGAGCCCCGTTCCTCCAGGAGTCCCAGATCCAGTATGT

GCCTGTATCCCCAGGCCAGCAGCTGGTCACACAGGCTCAGCTTGAGGCTGCAGCCCACTCTGCCGTCACA
GCGGTGGCTGACGCTGCCATGGCCCAAGCCCAGGGCCTGTTTGGCACAGAGGAGGCAGTGCCCGAACACG
TGCAACAGCTGCAGCACCAGGGCATCGAGTACGACGTCATCACCCTGGCCGATGAC
>Chinchilla lanigera XM_005392396.2
ATGGAGGAGAACGAGGTAGAGAGCAGTAGCGACGCTGCCCCTCGGCCTGAGGAGCCCT
CTGAGAGCGGCCTGGGTGTGTGCACCTCGGAAGCTGTGTCAGCCGACAGCAGCGACGCCGCGACCGCCTC
GGGGCCGGTGGAGGCCGATGTCTCTGGAGTGGTGCAAAGCTCAGACCGAGGCAGCCACTCACTGGAGGAG
GTATCGGAGAGCAGCTCAAGCACGGAGCCTCTGCCTCATGGCTACCTCCCGGATTCGTCTTCTGTGTCTG
GTGGGCCGGTGGCAGCAGTCCCTGGAGGCCCCCCAGCACTGGTGCACTCCAGTGCTCTCCCAGACCCCAA
CATGCTGGTGTCTGACTGCACAGCTTCCTCCTCGGACCTGGGCTCGGCCATCGACAAGATCATCGAGTCC
ACCATCGGGCCAGACCTCATCCAGAGTTGTATCACCGTGACCAGTGCTGAAGATGGCGGAGCTGAGACCA
CGCGATACCTAATCCTGCAGGGCCCGGACGATGGTGCCCCCATAACGTCTCCAATGTCCAGTTCCACCCT
GGGCCACAGCCTGGCAGCCATCGAAGCCCTGGCTGATGGCCCTACATCTACATCCACGTGCCTAGAGCCG
CCTGAGGAGGCGCAGGATGGGCCCGGCTCCCTGGCAAGGGCGCCCCCAGCCCCTGGCGCCGAGGAGCTGG
ACCTGCAGAGCCTGGAAGCCATGATGGAGGTGGTGGTGGTGCAGCAGTTCAAGTGCAAGATGTGCCAGTA
CCGGAGCAGCACCAAGGCCACACTGCTGCGGCACATGCGGGAGCGGCACTTCCGCCCTGCAGCAGCAGCA
GCAGCAGGCACTAGTAAGAAAGAACGACAGCGGAAGTGGGACGCCTCTGCCAAGACCCAGGAGGAAGAAG
GGCCGGAGGAGGAGGAGGAAGATGACATTGTGGATGCTGGGGCCATCGATGACCTGGAGGAGGACAGTGA
CTATAATCCTGCTGAGGATGAGCCCCGAGGCCGGCAGCTGCGGCTCCAGCGCCCCGCCCCCAGCACCCCA
AGGCCACGAAGGAGACCTGGCCGGCCCCGAAAGCTGGCTCGCCTGGAAACCTCAGATCTCCCAGATGGTG
TGGAGGAGCCTCTAGTGAGTTCCCAGAGCGGTGAGAGCCCCACAGAGCCGCAAGATCCTGAGGCGTCCAG
CTCCTCGGGCCCAGGATGCCTGGTGGCCCTGGGCAAGGCTGGCAAGGCCCCGGTGGAGTCCGGTGTGAGT
CAGTCAGACTCCGAGAACACAGCACCCTCCTGCCAAGAAGAGCCTGAAGCCCCACCCCGCCGCCGGGGCC
GACCCTCCAGGCGATTCCTGGGCAAGAAATACCGCAAGTACTATTACAAGTCGCCCAAGCCGCTTCTGAG
GCCCTATCTGTGCCGCATCTGTGGCTCCCGCTTCCTGTCCCACGAGGACCTGCGTTTCCATGTCAACTCC
CACGAGTCTGGTGACCCCCAGCTCTTCAAGTGCCTCCAGTGTAGCTACCGCTCCCGCCGCTGGTCCTCGC
TCAAGGAACACATGTTCAACCATGTGGGCAGCAAGCCCTACAAGTGTGACGAATGCAGCTACACCAGTGT
CTACCGGAAGGACGTCATTCGCCATGCAGCAGTGCACAGCCGGGACCGGAAGAAGAGGCCAGATCCGACC
CCAAAGCTGAGTTCTTTCCCCTGCCCTGTGTGTGGCCGTGTGTACCCCATGCAGAAGAGACTGACTCAGC
ACATGAAGACGCACAGCACTGAGAAGCCCCACATGTGTGACAAGTGTGGAAAGTCCTTTAAGAAGCGCTA
CACCTTCAAGATGCACCTGCTCACGCATATCCAGGCTGTTGCCAACCGCAGGTTCAAGTGCGAGTTCTGT
GAGTTTGTTTGTGAGGATAAGAAGGCCCTGCTGAACCACCAGCTGTCCCATGTCAGTGACAAGCCCTTCA
AATGCAGCTTCTGTCCCTACCGCACCTTCCGAGAGGACTTCCTGCTCTCCCACGTGGCTGTCAAGCACAC
AGGGGCCAAGCCCTTTGCCTGTGAGTACTGCCACTTCAGCACCAGGCACAAGAAGAATCTCCGCCTGCAC
GTCCGGTGCCGGCATGCAAGCAACTTTGAGGAATGGGGGCGCCGCCACCCTGAGGACCCTCCCTCCCGCC
GCCGTCCCTTCTTTTCTCTGCAACAAATTGAGGAACTAAAGCAGCAGCATAGTACGACCCCCGCAGCACC
CCCCGCCTCCCCAGGACCTCCTGAGCTCCCTCCAGAGGCTGCACCTTTCCAGTCACCCGAGACCCCTCCA
CTGCTCTGTTCTGACACTCTGGGCAGCGCCACCATCATTTACCAGCAAGGAGCTGAGGAGTCCACCGCAA
TGGCCACACAGACAGCCTTGGACCTGCTGTTGAACATGAGCGCTCAGCGGGAGCTAGGGGGCGCAGCCCT
GCAGGTAGCTGTGGTGAAGTCAGAGGACATGGAAGCAGAGCTAGCATCCTCTGGTGGGCAGCCCTCCCCC
GAAGACACCACTCCACAAGTGGTAACCCTCCATGTGGCTGAGCCAGGAGACACTGTGGCAGCCGAGAGCC
AACTAGGCCCCCCTGACCTGCAGCAGATCACCTTGGCACCTGGTGCATTTGGTGGAGCTGGCTACAGCGT
CATCACAGCACCCCCTATGGAGGAGGGCACATCAGCTCCTGGCACACCTTACAGTGAGGAGCCCCCAGGG
GAGGCAGCCCAGGCTGTGGTTGTGAGTGACACCCTCAAAGAAGCTGGCACCCACTATATCATGGCAGCCG
ATGGAACCCAGCTGCACCACATTGAGTTGACTGCAGATGGCTCCATTTCCTTCCCCAGTCCTGATTCCCT
GACCTCTGGAGCCAAGTGGCCCCTGCTGCAGTGTGGGGGGCTGCCCAGAGATGGCCCCGAGCCCCTATCT
CCAGCCAAGACCCACAGAGTGGGGAACCCCCCGAGCTGTTCCTCCCCACCTCCTACAGCCAGCAAAGCCC

TGGGCCTGGTGGTCCCACCCTCGCCACCATCTGCAACCACTGCAGCATCGAAGAAGTTTTCCTGCAAGAT
CTGTGCCGAGGCCTTCCCTGGCCGGGCAGAGATGGAAAGTCACAAGCGGGCCCACGCTGGGCCTAGTGCC
TTCAAGTGCCCTGACTGCCCCTTCAGTGCCTGCCAGTGGCCGGAGGTCCGGGCCCATATGGCGCAGCACT
CGAGCCTGCGTCCTCACCAGTGCAGCCAGTGTAGTTTCGCCTCCAAGAACAAGAAGGACTTGCGGCGGCA
CATGCTGACCCACACCAATGAGAAGCCTTTCTCGTGCCACCTCTGTGGGCAGCGTTTCAACCGCAATGGG
CACCTCAAGTTTCACATCCAGCGGCTGCACAGCCCCGATGGGAGAAAACCAGGGACCCCTACAGCCCGGG
CCCCAGCCCAGACCCCCACTCAGACCATCATCTTGAATAGTGATGAGGAGACGCTGGCCACACTGCATAC
TGCCCTCCAGTCCAGTCATGGGGTCCTGGGCCCGGAGCGGCTGCAGCAGGCGCTGGGCCAGGAACACATC
ATTGTGGCCCAGGAACAGACAGTGACCAATCAGGAAGAAGCCACCTATATCCAAGAGATCACCACAGCAG
ATGGCCAGACAGTACAGCACCTGGTAACCGCTGACAACCAGGTACAGTATATCATCTCTCAGGACGGTGT
CCAGCACCTGCTGCCTCAGGAATATGTTGTGGTCCCTGATGGCCATCACATCCAGGTACAGGAGGGCCAG
ATTACACACATCCAGTATGAACAAGGAGCCCCGTTCCTGCAGGAGTCCCAGATCCAGTATGTGCCTGTGT
CCCCAGGCCAGCAGCTTGTCACTCAGGCTCAGCTTGAGGCCGCAGCACACTCTGCTGTCACAGTGGCTGA
TGCTGCCATGGCCCAGGCCCAGGGCCTGTTCGGCACAGAAGAGGCAGTGCCTGAACACATCCAACAGCTG
CAGCACCAGGGCATCGAGTATGATGTCATCACCCTGACTGAAGAC
>Marmota marmota marmota XM_015478966.1
ATGGAGGAGAACGAGGTGGAGAGCAGTAGCGACGCCGCCCCTCGTCCTGGGCGGCCC
GAGGAACCTTCGGAGAGCGGCCTGTGCGTGTGCACTTCAGAAGCCGTGTCTGCCGATAGCAGCGACGCCG
CGTCGGTCCCAGGACCCGGGGAGGCCGACGACTCTGGCGTGGGGCAAAGCTCAGACCGCGGTGGCCGCTC
TCTGGAAGAGGTGTCTGAGAGCAGCTCAAGCACCGACCCTCTGCCTCATGGCTACCTCCCTGATTCGTCT
TCCGTGTCCCGTGGGCCTGTGGCAGGGGTGCCAGGCGGGCCCCCAGCACTGGTGCACTCCAGCGCTCTCC
CCGACCCCAGCATGCTGGTGTCCGACTGCGCAGCCTCCTCCTCAGACCTGGGCTCTGCCATTGACAAGAT
CATCGAGTCCACCATCGGGCCCGACCTCATCCAGAGCTGCATCACCGTGACCAGTGCTGAAGATGCTGGG
GCTGAGGCCACTCGCTATCTGATCCTGCAGGGCCCAGATGATGGCGCCCCCATGACATCACCAATGTCCA
GTTCCACCCTGGCCCACAGCCTGGCCGCCATCGAGGCCCTTTCAGATGGCCCCACGTCCACATCCACATG
CCTGGAGCCACCGGAGGAGGTGCAGGGTGGGGCCAGCTCCCTGGCCCAGCTGCCCCCAGCCTCTGGCACT
GAGGAGCTGGACCTCCAGAGCCTGGAGGCCATGATGGAGGTGGTGGTGGTGCAGCAGTTCAAGTGCAGGA
TGTGCCAGTACCGCAGCGGCACCAAGGCCACGCTGCTGCGGCACATGCGGGAACGGCACTTCCGCCCAGC
AGCAGCAGCAGCAGCCGGTAAGAAGGGACGTCTCCGGAAATGCGGCCCATCGACCAAGAGCCAGGAGGAG
GAGGGGCCCGAGGAGGAGGAGGAGGAGGATGACATTGTAGATGCCGGTGCCATTGATGACCTGGAGGAGG
ACAGCGACTACAATCCAGCTGAGGATGAGCCCCGAGGCCGGCAGCTTCGGCTCCAGCGTCCCACACCCAG
TTCCCCAAGACCACGACGGAGACCTGGCCGGCCCCGGAAGCTGCCTCGTCTGGAGACCTCAGACCTGTCT
GCTGCAGGTGGGGGAGATCCTCTAGTGAGCTCGCAGAGCACCAAGAGCCCTCTGGGGCCACCAGATCCTG
AGGCTCCCAGCTCCTCAGGCCCCGGACACCTGGAGGCCCTGGGCAAGGCCGGCAAGGTCACCATGGAGCC
TGGTGTGAGCCAGTCAGACACAGAGATTGCAGCCGCATCCTGCCAGGAGGTGCCCGATGCCCCACCGCGC
CGCCGTGGTCGACCTTCACGGCGATTTCTGGGCAAGAAATACCGAAAGTATTATTACAAGTCGCCCAAAC
CGCTTCTGCGGCCCTATCTGTGCCGCATTTGTGGCTCACGCTTCCTGTCCCATGAGGACCTGCGCTTCCA
CGTCAACTCCCACGAGGCTGGTGACCCCCAGCTCTTCAAGTGCCTGCAGTGCAGCTACCGCTCCCGCCGC
TGGTCCTCACTCAAGGAGCACATGTTCAACCACGTGGGCAGCAAGCCCTACAAGTGTGACGAGTGTAGCT
ACACCAGTGTCTACCGCAAGGACGTCATTCGGCATGCAGCTGTTCACAGCCGAGACAGGAAGAAGAGGCC
AGACCCGACCCCAAAACTGAGCTCTTTCCCCTGCCCTGTGTGCGGCCGCGTGTACCCCATGCAGAAGAGA
CTGACACAGCACATGAAGACGCATAGCACTGAGAAGCCCCACATGTGTGACAAGTGTGGAAAGTCCTTTA
AGAAGCGCTACACCTTCAAAATGCACCTGCTCACACACATCCAGGCTGTCGCCAACCGCAGGTTTAAGTG
TGAGTTCTGTGAGTTCGTCTGTGAGGACAAGAAGGCACTGCTGAACCACCAGCTCTCCCACGTCAGCGAC
AAGCCCTTCAGATGCAGCTTTTGTCCTTACCGTACCTTCCGGGAGGACTTCCTGCTGTCCCACGTGGCTG
TCAAGCACACAGGAGCCAAGCCCTTTGCCTGTGAGTACTGCCACTTCAGCACGCGCCACAAGAAGAACCT
GCGCCTGCATGTGCGCTGCCGCCACGCGAGCAGCTTCGAGGAGTGGGGGCGGCGCCACCCCGAGGAGCCC

CCCTCCCGCCGCCGCCCCTTCTTCTCCCTGCAACAGATTGAGGAACTGAAGCAGCAGCACAGCGCAGCCC
CTGGGCCACCCCCCGGCTCCCCGGGGCCTCCTGAGATCCCCGCAGAGGCAGCACCTTTCCAGATGCCTGA
GACTCCCCCGCTTCTCTGTTCTGACACTCTGGGCAGTGCCACCATCATCTACCAGCAAGGAGCTGAGGAG
TCCACTGCAATGGCCACCCAGACGGCTCTGGATCTGCTGCTGAACATGAGTGCCCAGCGGGAGCTGGGGG
GCACAGCCCTGCAGGTGGCCGTGGTGAAGTCAGAGGACGTGGAAGCAGGGTTAGCGTCTTCTGGTGGGCA
GCCCTCCCCCGAAGACACCACTCCACAGGTGGTCACCCTCCATGTGGCAGAGCCTGGGGGCAGTGTGGCT
GCTGAGAGCCAGCTAGGCACTCCTGATCTACAGCAGATCACCTTGGCACCTGGTCCATTTGGTGGAGCTG
GCTACAGTGTCATCACAGCACCCACCATGGAGGAGGGGACATCGGCTCCTGGCACACCTTACAGTGAAGA
GCCCCCAGGGGAGGCAGCCCAGGCTGTGGTTGTAAGTGACACCCTCAAAGAAGCTGGCACCCACTATATC
ATGGCTGCTGATGGGACCCAGCTGCACCACATCGAGTTGACTGCAGATGGCTCCATCTCCTTCCCCAGCT
CAGATGCCCTGGCCTCTGGAGCCAAGTGGCCCCTTCTGCAGTGTGGGGGGCTGCCCAGAGATGGTCCCGA
GCCACCGTCTCCAGCCAAGACCCTCCGGGTAGGGGACTCCCAGCATTCTGCCTCCCCACCTCCTGCAGCC
AGCAAAACCCTGGGCCTGGCAGTGCCCCCTTCCCCACCATCTGCAGCCACTGCAGCATCAAAAAAGTTTT
CCTGCAAGATTTGTGCTGAGGCCTTCCCTGGCCGAGCTGAGATGGAGAGTCACAAACGGGCCCATGCTGG
GCCTGGCACCTTCAAGTGCCCTGACTGCCCCTTCAGTGCCCGCCAGTGGCCCGAGGTCCGGGCCCACATG
GCACAGCACTCAAGCTTGCGGCCCCACCAGTGCAGCCAGTGCAGCTTCGCCTCGAAGAACAAGAAGGACC
TGAGGCGGCACATGCTGACCCACACCAATGAGAAGCCCTTCTCGTGCCATCTCTGCGGGCAGCGTTTCAA
CCGTAACGGGCACCTCAAGTTCCACATCCAGCGGCTGCACAGTCCTGACGGGAGGAAGTCTGGCACCCCG
ACAGTCCGCACCCCAGCACGGACCCCCACCCAGACCATCATCCTGAATAGTGACGACGAGACACTGGCCA
CGCTGCATACTGCCCTCCAGTCCAGTCACGGGGTCCTGGGCCCAGAGAGGCTACAGCAGGCCCTGGGCCA
GGAACACATTATTGTGGCCCAGGAACAGACGGTGACCAATCAGGAGGAAGCCACCTACATCCAAGAGATC
ACCACAGCCGATGGTCAGACGGTACAGCACCTGGTGACCTCGGATAACCAGGTACAGTATATCATCTCTC
AGGATGGTGTCCAGCACCTGCTGCCCCAGGAATATGTTGTGGTCCCTGATGGCCACCACATCCAGGTACA
GGAGGGCCAGATCACACACATCCAGTATGAACAAGGAGCCCCGTTCCTCCAGGAGTCCCAGATCCAGTAT
GTGCCCGTATCCCCAGGCCAGCAGCTGGTCACACAGGCTCAGCTTGAGGCTGCAGCCCACTCTGCCGTCA
CAGCGGTGGCTGATGCTGCCATGGCCCAAGCCCAGGGCCTGTTTGGCACAGAGGAGGCAGTGCCCGAACA
CGTACAACAGCTGCAGCATCAGGGCATCGAGTACGACGTCATCACCCTGGCCGATGAC

>RAT ENSRNOT00000023521
ATGGAGGAGAACGAGGTGGAGAGCAGTAGCGACGCGGCCCCTCGGCCTGGCCAGCCGGAG
GAGCCTTCTGAGAGCGGCCTGGGTGTGGGCACCTCGGAAGCTGTGTCCGCGGACAGTACG
GACGCTGCGACCGCCCCAGGATTAACGGAGGCCGATGACTCTGGAGTGGGGCAGAGCTCA
GACAGTGGCAGCCGCTCTGTGGAGGAGGTATCCGAGAGCATTTCAACAGAGCCCTTGCCT
CAGGGCTACCTCCCTGATTCATCTTCTGTGTCCCGGGGACCAGTGGCAGAAGTGCCAGGT
GGCCCCCCAGCCCTGGTGCATTCCAGTGCTCTCCCAGACCCCAGCATGCTGGTGTCTGAC
TGCACAGCTTCCTCTTCAGACCTAGGCTCCGCCATTGACAAGATCATTGAATCTACCATT
GGCCCGGACCTCATCCAGAGCTGCATCACTGTGACCAGTGGTGAAGAGGGAGGAGCTGAG
ACCACACAGTACCTGATCCTGCAGGGCCCAGATGATGGTGCTCCCATGGCGTCATCAATG
TCTACTTCCACCTTGGCCAACAGTCTGGCAGCCATTGAAGCCCTGGCTGATGGTCCCACA
TCCACATCTACTTGCCTGGAGCCTGCTGAGCAGCCACCGGGAGAGCCTAGTTCTCTAGCA
CAGCCCCCAGCACCTGTTGTCGAGGAGCTGGACCTGCAGGGCCTGGAGGCCATGATGGAG
GTGGTGGTTGTGCAGCAGTTCAAGTGCAAGATGTGCCAGTACCGGAGCAGCACCAAGGCC
ACCCTGCTCCGCCACATGCGGGAGCGGCACTTCCGCCCAGCAGCACTAGCAGTAGCAGCA
GCAGCAGCTGGTAAAAGGGGACGTGTACGGAAGTGGGGCACTTCCACCAAGACTACAGAG
GAGGAGGGGCCGGAGGAAGAGGAGGAGGACGACGACATTGTGGATGCTGGGGCAATTGAT
GATCTGGAGGAGGACAGTGACTACAACCCAGCTGAGGATGAGCCCCGGGGCCGGCAGCTA
CGGCTTCAGCGTCCCACACCGAGTACCCTGAGACCTCGACGGAGGCCTGGCCGGCCCCGA

AAGTTGCCTCGCCTAGAGACCTCAGACCTCCATGATGGCATAGGAGAGCCGCTAGTGAGT
TCGCAGAGCACACAGAGCCCTCCAGAGCTTCAGGATCTCGAGGCTCCCAGCTCCTCCGAC
CTAAGGGCCCTGGGCAAAGTGGGGAGAGGCCTGGTGGAAACTGGTGTGAGTCAGTCAGAT
GCTGAGAATGCAGCCCCGTCTTGCCAGGATGAAGCTGATGTCCCACCCCGCCGCCGTGGA
AGACCTTCCAGGCGGTTCCTAGGGAAGAAATACCGAAAGTACTACTACAAGTCGCCCAAG
CCACTGCTCAGGCCTTACCTGTGCCGCATATGTGGCTCACGATTCCTGTCCCATGAAGAC
CTTCGCTTCCATGTCAACTCCCATGAGGCTGGTGACCCACAGCTCTTTAAGTGCCTACAA
TGTAGCTACCGCTCCCGCCGCTGGTCCTCACTGAAGGAGCACATGTTCAACCACGTGGGC
AGCAAACCCTACAAGTGTGACGAATGCAGCTACACCAGTGTCTACCGCAAGGATGTTATT
CGGCATGCGGCCGTGCACAGCCAGGACCGAAAGAAGAGGCCGGATCCGACCCCAAAGCTG
AGCTCTTTCCCTTGCCCAGTGTGTGGCCGTGTATACCCCATGCAGAAGAGACTAACACAG
CACATGAAGACTCACAGTACGGAGAAGCCACACATGTGCGATAAGTGTGGAAAGTCCTTT
AAGAAGCGGTACACCTTCAAAATGCACTTGCTCACACACATCCAGGCTGTTGCCAACCGC
AGATTCAAGTGTGAGTTCTGCGAGTTTGTTTGTGAGGACAAGAAAGCACTGTTGAACCAC
CAGCTGTCCCATGTTAGCGACAAGCCCTTCAAATGCAGCTTTTGTCCCTATCGCACCTTC
CGTGAGGACTTCCTGCTGTCTCATGTGGCTGTGAAGCACACAGGAGCCAAGCCCTTCGCC
TGTGAGTACTGCCACTTCAGCACTCGCCACAAGAAGAACCTGCGCCTGCATGTACGGTGC
CGACATGCGAACAGCTTTGAGGAGTGGGGACGGCGCCACCCTGAGGAGCCTCCATCCCGT
CGCCGCCCCTTCTTCTCTCTGCAACAGATAGAAGAGCTGAAGCAGCAGCACAGTGCGGCC
CCTGGCCCTCCCCTCAGCTCAGCAGGCCCCGAGGCCCCCCAAGAACCAGCACCTTTCCAG
CCACCTGAGACTCCCCCACTACTCTGTCCTGATGCCCTAGGTGGTGCCACAATCATCTAC
CAGCAAGGCGCTGAGGAGTCCACTGCAATGGCCACTCAGACAGCCTTGGATCTACTGTTG
AACATGAGCGCCCAACGAGAGCTGGGGGCCACAGCCTTGCAGGTGGCTGTGGTGAAGTCA
GAGGACGTGGAGGCAGAGTTGACATCTACTGCTAGGCAGCCTTCCTCTGAAGACACCACT
CCACGGGTGGTGACACTTCATGTGGCAGAGTCAGGGAGCAGTGTGGCAGCTGAGAGCCAG
CTAGGCCCGTCTGACCTACAGCAGATTGCCTTGCCACCTGGGCCATTCAGTGGGGCCAGC
TACAGTGTCATCACAGCACCCCCCGTGGAGGGGAGGGCATCAGCTTCCGGCCCACCTTAC
AGGGAAGAACCTCCAGGAGAGGCAGCCCAGGCTGTGGTTGTGAACGACACTCTCAAGGAA
GCTGGCACCCACTATATCATGGCAGCTGATGGGACCCAGTTGCACCACATTGAGCTGACT
GCAGATGGCTCCATCTCCTTCCCAAGCCCAGATACTCTGGCCCCTGGAACCAAGTGGCCC
CTGCTGCAGTGTGGAGGGCCACCTAGAGATGGTCCTGAGGTTCTGTCTCCAACGAAGACC
CACCATACGGGAGGCTCCCAGGGCTCTTCCACCCCACCCCCTGCAACCAGCCATGCCCTA
GGCCTGCTAGTACCCCACTCCCCACCGTCTGCAGCAGCTTCATCAACAAAGAAGTTCTCC
TGCAAGGTGTGCTCAGAGGCCTTCCCTAGCCGTGCAGAGATGGAGAGTCACAAGCGGGCC
CATGCTGGGCCTGCTGCCTTCAAGTGCCCTGACTGCCCCTTCAGTGCTCGCCAATGGCCC
GAGGTCCGGGCTCACATGGCACAGCACTCCAGTCTGAGGCCCCACCAGTGCAATCAGTGT
AGCTTCGCCTCCAAGAACAAGAAGGACCTCAGGCGGCACATGCTGACACACACCAATGAG
AAGCCTTTCTCATGCCACGTCTGTGGGCAGCGTTTCAACAGGAACGGGCACCTCAAATTC
CACATCCAGCGGCTACATAGCATCGATGGTAGAAAGACTGGGACTTCTACAGCCCGAGCC
CCAGCCCAGACCATCATCCTCAATAGTGAAGAGGAGACACTGGCCACACTGCACACTGCC
TTCCAGTCGAATCACGGGACTCTGGGGACAGAGAGGCTACAGCAGGCACTGAGCCAGGAG
CATATCATTGTGGCCCAGGAACAGACAGTGGCCAATCAGGAGGAAGCTACCTACATCCAG
GAAATCACGGCAGATGGCCAGACGGTACAGCATCTGGTGACCTCAGACAACCAGGTTCAG
TATATCATCTCTCAGGATGGTGTCCAGCACTTGCTGCCTCAGGAGTACGTTGTGGTCCCT
GATGGCCATCACATCCAGGTCCAGGAGGGCCAGATCACACACATCCAGTATGAGCAGGGC
ACCCCATTCCTACAGGAGTCCCAGATCCAGTATGTACCTGTATCCCCCAGCCAGCAGCTT
GTCACCCAGGCTCAGCTTGAAGCTGCAGCACATTCTGCTGTTACAGCAGTGGCTGATGCT

GCCATGGCCCAAGCCCAGGGCCTGTTTGGCACTGAGGAGGCAGTGCCGGAACACATTCAA
CAGCTGCAGCATCAGGGCATCGAGTACGACGTCATCACCCTCTCGGATGAC
>Mouse ENSMUST00000041361
ATGGAGGAGAACGAGGTGGAGAGCAGTAGCGACGCGGCCCCTCGGCCCGGCCAGCCTGAG
GAGCCTTCTGAGAGCGGCCTGGGTGTGTGCACCTCGGAAGCTGTGTCCGCGGACAGCAGC
GACGCTGCGACCGTCCCAGGATTAACGGAGGCTGACGACTCTGGCGTCGGGCAGAGCTCG
GACGGTGGCAACCACTCTGTGGAGGAGGTATCTGAGAGCATCTCAACAGACCCTTTGCCT
CATGGCTGCCTCCCTGATTCATCTTCTGTGTCCCGGGGACCAGTAGCAGAGATGCCTGGT
GGCCCCCCAGCTCTGGTGCATTCCAGTGTTCTCCCAGACCCCAGCATGCTGGTGTCTGAC
TGCACAGCCTCCTCTTCAGACCTAGGCTCTGCCATTGACAAGATCATCGAATCTACCATT
GGTCCAGACCTCATCCAGAGCTGCATCACTGTGACCAGTGGTGAAGAGGGAGGAGCTGAG
ACCACACAGTACCTGATCCTGCAAGGCCCGGATGACGGCGCTCCCATGGCATCATCCATG
TCTACTTCCACCTTGGCCAACAGTCTGGCAGCCATTGAAGCCCTGGCTGACGGCCCCACA
TCCACATCTGCATGCCTTGAGCCTCCCGAGGAGCCCCAGGGAGATCCCAGCTCTGTAGCG
CAGCAACCCCCAGCTCCTGTCACTGAAGAGCTGGACCTGCAAAGCTTGGAGGCCATGATG
GAGGTGGTGGTTGTGCAACAGTTCAAGTGCAAGATGTGCCAGTACCGGAGCAGCACCAAG
GCAACCCTGCTCCGCCACATGCGGGAACGGCACTTCCGCCCAGCACTAGCAGCAGCAGCA
GCAGCAACTGGTAAAAGGGGCCGTGTACGGAAGTGGGGCACTTCCACCAAGACCACAGAG
GAGGACAGGCCAGAGGAGGAGGAGGAGGACGACGACATCGTGGATGCCGGGGCGATTGAC
GATCTGGAGGAGGACAGTGACTACAATCCAGCTGAGGATGAGCCCCGGGGCCGGCAGCTA
AGGCTCCAGCGCCCCACACCCAGTACCCCGAGACCTCGACGGAGGCCTGGCCGGCCCCGA
AAGCTGCCTCGCCTAGAGACCTCAGACCTTCATGATGGTGTAGGACAGCCTCTAGTGAGT
TCGCAGAGCACACAGAGCCCTCCAGAGTTGCAGGATCTCGAGGCTCCCAGCTCCTCGGGC
CTGAGGGCCCTGGGCAAGGTGGGGAGGGGCCTGGTGGAATCAGGCGTTAGTCAGTCGGAT
GCTGAGAACGCAGCCCCATCGTGCCAGGATGAAGCTGATGCCCCACCCCGCCGCCGCGGA
AGACCTTCCAGGCGGTTCCTAGGGAAGAAATACCGAAAGTACTACTATAAGTCACCCAAG
CCACTGCTCAGGCCTTATCTGTGCCGCATATGTGGCTCACGCTTCCTGTCCCACGAAGAC
CTTCGCTTCCACGTCAACTCCCATGAGGCTGGTGACCCACAGCTCTTCAGGTGCCTGCAG
TGCAGCTACCGCTCCCGCCGCTGGTCCTCGCTCAAGGAGCACATGTTCAACCACGTGGGC
AGCAAGCCCTACAAGTGTGACGAGTGCAGCTACACCAGTGTCTACCGCAAGGATGTTATT
CGCCACGCGGCTGTGCACAGCCAGGACCGAAAGAAGAGGCCGGATCCGACCCCAAAGCTG
AGCTCCTTCCCTTGCCCAGTGTGCGGCCGTGTCTACCCCATGCAGAAGAGGCTAACACAG
CACATGAAGACTCACAGCACCGAGAAGCCACACATGTGCGATAAGTGTGGAAAGTCCTTT
AAGAAGCGCTACACCTTCAAAATGCACCTGCTCACACACATCCAGGCCGTTGCCAACCGC
AGATTCAAGTGTGAGTTCTGCGAGTTTGTTTGTGAGGACAAGAAGGCACTGTTGAACCAC
CAACTGTCCCACGTGAGCGACAAGCCCTTCAAATGCAGCTTTTGTCCCTACCGCACCTTC
CGCGAGGACTTCCTGCTGTCTCATGTGGCTGTCAAGCACACAGGTGCCAAGCCCTTCGCC
TGTGAGTACTGCCACTTCAGCACTCGCCACAAGAAGAACCTGCGCCTCCACGTAAGATGC
CGCCATGCCAACAGCTTTGAGGAGTGGGGACGGCGCCACCCTGAGGAGCCCCCGTCCCGC
CGCCGCCCCTTCTTCTCTCTACAACAGATAGAAGAGCTGAAGCAGCAACACAGCACGGCC
CCTGGCCCTCCCCTCAGCTCACCAGGACCCGAGGCCCCCCAAGAACCAGCACCTTTCCAG
TCACCTGAGACCCCCCCACTACTCTGTCCTGATGCACTAGGTGGCACCACAATCATCTAC
CAGCAAGGAGCCGAGGAGTCCACTGCAGTGGCCACTCAGACAGCCTTGGATCTGCTGTTG
AATATGAGCGCCCAACGAGAACTGGGGGCCACGGCCTTGCAGGTGGCGGTGGTAAAGTCA
GAGGGCATCGAGGCAGAGTTGACGTCTACTGGTGGGCAACCTTCCCCTGAAGACACCACT
CCACGGGTGGTGACACTTCATATGGCAGAATCAGGGAGTAGTGTGGCAGCTGAGAGCCAG
CTAGGCCCATCTGATCTACAGCAGATTGCCTTGCCATCTGGGCCATTTGGTGGGGCCAGC

TACAGTGTCATCACGGCACCCCCAGTGGAGGGGAGGACATCAGCCTCAGGCCCACCTTAC
AGGGAAGAACCTCCCGGGGAGGCAGCCCAGGCTGTGGTTGTGAGCGACACTCTCAAGGAG
GCTGGCACCCACTACATCATGGCAGCTGATGGGACCCAATTGCACCACATTGAGTTGACT
GCAGATGGCTCCATCTCCTTCCCAAGCCCAGATACTCTGGCCCCTGGAACCAAGTGGCCC
CTGCTGCAGTGTGGAGGGCCACCCAGAGATGGTTCTGAGGTTCTGTCTCCAACAAAGACC
CACCATATGGGAGGCTCCCAGGGCTCCTCCACCCCACCCCCTGCAGCCAGCCATACCCTA
GGCCTGGTAGTACCCCAATCCCCACCGTCTGCAGCAGCTTCCTCAACAAAGAAGTTTTCC
TGCAAGGTGTGCTCAGAGGCCTTTCCTAGCCGCGCAGAGATGGAGAGTCACAAGCGGGCC
CATGCTGGGCCTGCTGCCTTCAAGTGCCCTGACTGCCCCTTCAGTGCTCGCCAGTGGCCT
GAGGTCCGGGCTCACATGGCACAGCACTCAAGCCTGAGGCCCCACCAGTGCAATCAGTGT
AGCTTCGCCTCCAAGAACAAGAAGGATCTGAGGCGGCACATGCTAACGCACACCAACGAG
AAGCCATTCTCATGCCACGTCTGCGGGCAGCGTTTCAACAGGAACGGGCACCTCAAATTC
CACATCCAGCGGCTACATAGCATTGATGGGAGAAAGACTGGGACTTCTACAGCCCGAGCC
CCAGCCCAGACCATCATCCTCAATAGTGAAGAGGAGACACTGGCCACACTACACACTGCC
TTCCAGTCCAGTCACGGGGTTCTGGGTACAGAGCGGCTACAGCAGGCACTGAGCCAAGAA
CACATCATTGTGGCCCAGGAACAGACAGTGACCAATCAGGAGGAAGCTACCTACATCCAG
GAAATCACAGCAGATGGCCAGACAGTACAGCATCTGGTGACCTCAGACAACCAGGTTCAG
TATATCATCTCTCAGGATGGTGTCCAGCACTTACTGCCTCAGGAGTACGTTGTGGTCCCT
GATGGCCATCATATCCAGGTCCAGGAAGGCCAGATCACACACATCCAGTATGAGCAAGGT
ACTCCATTCCTGCAGGAGTCCCAGATCCAGTATGTACCTGTCTCCCCCAGCCAGCAGCTT
GTCACCCAGGCTCAGCTGGAAGCTGCCGCACACTCCGCTGTCACAGCGGTGGCTGATGCT
GCCATGGCCCAAGCCCAGGGCCTGTTTGGCACTGAGGAGGCAGTGCCGGAGCAGATCCAC
CAGCTGCAGCATCAGGGCATCGAGTACGACGTCATTACCCTCTCGGATGAC
>Erinaceus europaeus XM_007533055.2
ATGGAGGAGAACGAGGTGGAGA
GCAGCAGCGACGCGGCCCCGGGGCCTGGCCGGCCCGAGGAGCCCTCGGAGAGCGGCCTGGGTGTGGGCAC
CTCGGAAGCCGTGTCGGCGGACAGCAGCGACGCGGCTGCCGACCCCGTGGGGCAGAGCACGGACCGCGGC
GGCGCGCTGGAAGAGGTATCTGAGAGCAGCTCAAGTACAGACCCTCTTCCCCATGGCTACCTCCCTGACT
CATCCTCTGTGTCCCATGGACCAGTTGTGGGGGCAACAGGTGGTCCTCCAGTCCTGGTACACTCCAGCGC
ACTCCCAGACCCCAACATGTTGGTATCGGAGTGCACAGCTTCTTCTTCGGATTTGGGCTCAGCCATCGAT
AAGATCATTGAATCTACCATTGGTCCAGACCTAATCCAGAACTGCATCACTGTGACTAGTGCTGAGGATG
GTGGTGCTGAGACTACACAGTACCTAATCCTACAGGGGCCAGATGATGGTGCCTCCATGGTGTCACCAAT
GTCCAGTTCCACCCTGGCCCATAGTCTGGCAGCCATCGAAGCCCTGGCTGATGGCCCCACATCTACATGC
TTTGAACCATCTGAGGAGGTGCAGGGTAGGCCTAACTCCCCAGCACAACCACCCCCAGGTTCTGGTGCTG
AGGAGCCAGACCTGCAGAGTCTGGAGGCCATGATGGAAGTGGTGGTAGTACAGCAATTCAAGTGCAAGAT
GTGCCAGTACCGGAGCAGCACCAAGGCCACACTGTTGCGCCACATGCGGGAGCGGCACTTCCGTCCAGCA
ATAGCCACAACTACAGCAGCTGGGAAGAAGGGCCGTCCGCGAAAGTGGGGCTCCTCTACCAAGACCCCAG
AGGAAGAGGGGCAGGAAGAGGAAGATGATGACGACATTGTAGATGCTGGAGCCATTGATGACCTAGAGGA
GGACAGCGACTACAATCCATCTGAGGATGAGCCCCGAGGCCGGCAGCTACGGCCCCAACGGCCCACTCCC
AGCACGCCAAGACCTCGAAGAAGACCTGGCCGGCCGCGGAAGATACCTCGGCTGGAAAGCTCAGAGCTCC
TGGATGGTGTGGAAGGAGAGCCTCTAGTGAGTTCTCAGGGTAGACAGAGCCCTCCAGAGCCACAGGACCC
CGAAGTGCCCAGCTCTTCTGAACCAGGAAGCTTGGTAGCCCCTGTGGAACCTAGTGTGAGCCAGTCAGAC
ACAGAAAATGCTGCACCCTCATCACAGGAAGAACTGGACACACCACCCCGTCGCCGTGGCCGACCCTCCA
GACGCTTTCTGGGCAAGAAATACCGCAAGTACTACTACAAGTCTCCCAAACCGCTTCTGAGGCCTTTCCT
GTGCCGCATCTGTGGCTCCCGATTCCTGTCTCATGAGGACCTGCGTTTCCACGTCAACTCCCACGAGGCT
GGGGACCCTCAACTCTTCAAGTGCCTGCAGTGCAGCTATCGCTCCCGCCGCTGGTCCTCACTCAAGGAGC
ACATGTTCAACCACGTGGGCAGTAAGCCCTACAAGTGTGATGAATGCAGCTACACCAGCGTTTACCGAAA

AGATGTCATTCGGCATGCAGCTGTGCACAGCCGGGACCGGAAGAAGAGGCCAGATCCGACCCCAAAGCTG
AGCTCCTTCCCATGTCCTGTGTGTGGGCGCATCTATCCCATGCAGAAGAGACTCACACAGCACATGAAGA
CACATAGCACTGAGAAACCCCACATGTGTGACAAGTGTGGAAAGTCCTTTAAAAAGCGCTACACCTTCAA
GATGCATCTGCTCACGCACATCCAGGCTGTTGCCAACCGCAGGTTCAAGTGTGAGTTCTGTGAGTTTGTT
TGTGAGGACAAGAAGGCACTGCTGAATCACCAGCTGTCCCATGTCAGTGACAAGCCCTTTAAGTGCAGCT
TTTGCCCGTACCGCACCTTCCGAGAGGACTTCCTGCTATCCCATGTGGCTGTCAAACACACAGGGGCGAA
ACCCTTTGCCTGTGAGTACTGTCACTTCAGCACTCGGCACAAGAAGAATCTCCGCCTGCACATACGGTGC
CGACATACAAGCAACTTTGAAGAGTGGGCTCGGCGTCACCCCGAGGAGCCCCCTTCCCGCCGTCGCCCCT
TCTTCTCACTACAGCAGATTGAGGAGCTGAAACAGCAGCACAGCACAGCCCCTGGACCACCCCCCAACTC
CCCAGGTTCTTCTGAGATACCCTCAGAGGCTGCACCTTTCCAGTCAACAGAGACCCCCCCACTACTCTGC
TCTGACACCCTGAGTGGCGCTACTATCATCTACCAGCAAGGAGCTGAGGAGTCTGCTGCCATGGCTACAC
AGACAGCCTTGGATCTGCTGCTGAACATGAGTGCCCAGCGGGAGCTGGGCACTACAGCACTACAGGTGGC
TGTGGTGAAGTCAGACGATGTTGGAGCAAACTTAGCATCTCCTGGTGGGCAGCCCTCCCCAGCAGATGCC
ACTCCACAAGTGGTGACCCTCCATGTGGCAGAGACGGGGGGCAGCATGACAGCTGAAAGCCAGCTAGGCC
CCCCTGAGCTGCAGCAGATCACCCTGGCACCTGCTTCATTTGGTGGAACTGGTTACAGTGTCATCACAGC
ACCACCCATGGAGGAGGGGACTTCAGCTCCTGGCACTCCTTACAGTGATGAGCCCCCAGGGGAGGCAGCC
CAGACTGTGGTTGTGAGTGACAACCTGAAAGAAGCTGGCACCCACTACATCATGGCAGCTGAGGGGACCC
AGCTGCACCACATTGAGGTGACAGCAGATGGTTCCATCTCATTCCCAAGTCCAGATGCTCTGGCTTCTGG
AGCCAAGTGGCCCTTGCTGCAGTGTGGGGGGCTGCCTAGAGACACTGCTGAACCCTCATCTCCAGCCAAG
ACCAACCGGTTAGGGGACCCCCCAGTTCCTACCTCCCCACCTCTTGCTACCAGCAAAGCCCTGGGCCTGG
TAGTGCCCTCCTCACCACCACCTGCAGCCACTGCATCATCAAAGAAGTTTTCCTGCAAGATCTGTGCTGA
GGCTTTCCCAGGTCGAGCTGAGATGGAGAGTCATAAACGGGCCCATGCTGGACCTGGTGCCTTCAAGTGC
CCCGACTGCACCTTCAGTGCCCGCCAGTGGCCCGAGGTCCGGACCCACATGGCACAGCACTCCAGCCTGC
GGCCCCACCAGTGCAACCAGTGCAGCTTTGCCTCCAAGAATAAGAAAGACCTGCGGCGGCACATGCTGAC
CCACACCAATGAGAAGCCTTTTGCATGCCATCTCTGCGGGCAGCGTTTCAACCGGAATGGGCACCTCAAA
TTCCACATCCAGCGGCTACACAGTCCTGATGGGAGAAAGGCAGGAACCCCTATGACCCGGACCCCAGCCC
AGACCCCCACCCAGACCATCATCCTGAACAGTGATGACGAGACCTTGGCTACACTGCACACTGCCCTCCA
GTCCAGCCATGGCGTCCTGGGTCCTGAACGGCTTCAGCAGGCACTGGGCCAGGAACACATCATCGTTGCC
CAGGAGCAGACAGTGACCAATCAGGAGGAAGCTACTTACATCCAAGAAATCACTACAGCAGATGGCCAGA
CTGTACAACATCTGGTGACCTCTGACAACCAGGTACAGTACATCATCTCCCAGGATGGAGTCCAGCATCT
GCTACCCCAGGAATATGTCGTAGTCCCGGAGGGCCATCACATCCAGGTACAAGAAGGCCAGATTACACAC
ATACAGTATGAACAAGGAGGCCCGTTTCTTCAGGAGTCTCAGATCCAGTATGTGCCAGTGTCTCCAGGCC
AGCAACTTGTTACACAAGCCCAGCTTGAGGCTGCAGCACACTCTGCTGTTACAGCGGTGGCTGATGCTGC
CATGGCCCAAGCCCAGGGCCTGTTTGGCACAGAGGAGGCAGTGCCTGAACACATCCAGCAGCTTCAACAC
CAGGGCATCGAGTACGACGTCATCACCCTGACCGATGAC
>Cat XM_011280725.2
ATGGAGGAGAACGAAGTGGAGAGCAGTAGCGACGCGGCCCCTGGGCCTGG
CCGGCCCGAGGAGCCCTCTGAGAGCGGCTTGGGCGTGGGCACCTCTGAAGCCGTGTCGGCCGACAGCAGC
GACGCCGCGGCCGCCCCGGGGCCGGCTGAGGCCGACGACTCTGGCGTGGGGCAGAGCTCCGACCGCGGCA
GCAGCTCTCTGGAGGAAGTATCTGAGAGCAGCTCCAGCACAGACCCCCTGCCCCACGGCTACCTCCCTGA
TTCCTCTTCTGTGTCCCGTGGGCCAGTGGAGGGAGTGACAGGCGGCCCCCCAGCACTGGTGCACTCCAGC
GCTCTCCCAGACGCCAACATGCTGGTGTCCGACTGCACGGCTTCTTCCTCGGACCTGGGCTCAGCCATTG
ACAAGATCATCGAGTCCACCATCGGGCCAGACCTAATCCCAAGCTGCATCACCGTAACCAGTGCTGAGGA
TGGCGGAACCGAGACTGCCCGGTACCTGATCCTGCAGGGCCCGGACGATGGTGCCCCCATGGCTTCGCCG
ATGTCCAGTTCCACCTTGGCCCACAGCCTGGCAGCCATCGAGGCCCTGGCTGACGGCCCCACGTCCACAT
GCCTAGAGCCCCCCGAGGAGGCACGGGGGAGGCCCAGCTCCCCAGCACAGCCGCCCCCAAGCTCTGGTAC
CGAGGAGCCAGACCTGCAGAGCCTGGAGGCCATGATGGAAGTGGTGGTGGTACAGCAGTTCAAGTGCAAG

ATGTGCCAGTACCGGAGCAGCACCAAGGCCACGCTGCTGCGCCACATGCGGGAGCGGCACTTCCGACCAG
CAGCAGCAGCAGCAACCGCAGCCGGTAAGAAGGGGCGTCTGGTAAGGAAGTGGGGTACCTCTGCCAAGAC
CCCAGACGAAGAGGGGCCAGAGGAGGAAGATGATGATGACATTGTCGACGCTGGTGCCATTGACGACCTA
GAGGAGGACAGTGACTACAATCCGGCCGAGGATGAGCCCCGGGGCCGGCAGCTACGGCCCCAGCGCCCTA
CTCCCAGTACCCCAAGACCCCGAAGGAGACCTGGCCGGCCTCGGAAGCTGCCTCGCCTGGAGACCGCGGA
CCTCTCAGATGGTGTGGAAGGAGAGCCTCTAGTGAGTTCCCAGAGCGGACAGAGCCCACCAGAGCCTCAG
GACCCCGAGGCACCCAGCTCCTCTGGCCCGGGATGCCTGGTGGCCCTGGGCAAGGCTGATAGGGGCCCCG
TGGAACCTGGCGTGAGCCAGTCGGATGCAGAGAACGCAGCCCCCTCTTGTCAGGATGAGCCCGATGCCCC
ACCCCGCCGCCGGGGCCGACCCTCCAGGCGCTTCCTCGGCAAGAAATACCGCAAGTATTATTACAAGTCA
CCCAAACCGCTCCTGAGGCCCTTCCTGTGCCGCATCTGCGGCTCCCGCTTCCTGTCCCATGAGGACCTGC
GCTTCCACGTCAACTCCCATGAGGCCGGGGACCCCCAGCTCTTCAAGTGCCTGCAGTGCAGCTATCGCTC
CCGCCGCTGGTCCTCGCTCAAGGAGCACATGTTCAATCACGTGGGTAGTAAGCCCTACAAGTGTGGCGAA
TGCAGCTATGCCAGCGTCTACCGGAAGGACGTGGCCCGGCACGCAGCCGTGCACAGCCGAGACCGGAAGA
AGAGGCCAGATCCGACTCCGAAGCTGAGCTCCTTCCCGTGCCCTGTGTGTGGCCGTGTGTACCCCATGCA
GAAGAGACTCACACAGCACATGAAGACACACAGCACTGAGAAGCCCCACATGTGTGACAAGTGTGGAAAG
TCCTTTAAGAAGCGCTACACCTTTAAGATGCACCTGCTCACACATATCCAGGCTGTCGCCAACCGCAGGT
TCAAGTGCGAGTTCTGTGAATTTGTTTGTGAGGACAAGAAGGCACTGCTGAACCACCAGCTGTCCCACGT
CAGCGACAAGCCATTCAAATGCAGCTTTTGCCCCTACCGCACCTTCCGAGAGGACTTCCTGCTCTCCCAT
GTGGCTGTCAAGCACACAGGGGCCAAGCCCTTCGCCTGTGAGTACTGCCATTTCAGCACCCGGCACAAGA
AGAACCTCCGCCTGCACGTACGGTGTCGGCACGCAAGCAGCTTCGAGGAGTGGGGGCGGCGCCACCCTGA
GGAGCCCCCTTCCCGCCGTCGCCCCTTCTTCTCCCTGCAGCAGATTGAGGAGCTGAAGCAGCAGCACAGC
GCGGCCCCCGGGCCACCCGCCAGCTCCCCAGGGCCTCCTGAGATTCCCCCAGAGGCAGCACCTTTCCAGG
CACCTGAGACCCCCCCACTGCTCTGTTCCGACACCCTTGGTGGCGCCACCATCATCTACCAGCAAGGAGC
TGAGGAGTCAACGGCAATGGCCACGCAGACAGCCTTGGATCTGCTGCTGAACATGAGTGCTCAGCGGGAG
CTGGGGGGCACAGCCCTGCAGGTGGCCGTGGTGAAGTCGGAGGACGTGGAAGCAGAGTTAGCCTCCCCTG
GCGAGCAGCCTTCCCCGGCAGGTGCCGCTCCCCAAGTGGTCACCCTCCACGTGGCAGAGCCGGGGAGCAG
CCTGGCAGCGGAGAGTCAGCTGGGTGCCCCCGACCTGCCCCAGATCACCCTGGCACCCGGTCCATTTGGT
GGGACTGGCTACAGTGTCATCACAGCACCCCCTATGGAGGAGGGGACATCGGCTCCTGGCACACCTTACA
GCGAGGAGCCCCCAGGGGAGGCAGCCCAGGCCGTGGTCGTGAGCGACACCCTGAAAGAAGCTGGCACCCA
CTATATCATGGCAGCCGATGGGACGCAGCTGCACCACATTGAGCTGACTGCAGATGGTTCCATCTCCTTC
CCGAGTCCGGATTCGCTGGCCCCTGGAGCCAAGTGGCCGCTGCTGCAGTGTGGGGGGCTGCCCAGAGATG
GCCCTGAGCCCCCATCTCCAGCCAGGACCCACCGGGTGGGAGACCCCCAGGGCTCTGCCTCCCCACCTCC
TGCAGCCAGCAAAGCCCCGGGCCTGGTAGTACCCCCCTCGCCGCCACCTGCAGGCACGGCATCATCAAAG
AAGTTTTCCTGCAAGATCTGTGCCGAGGCCTTCCCTGGCCGAGCAGAGATGGAGAGTCACAAACGAGCCC
ACGCCGGGCCTAGTGCCTTCAAGTGCCCCGACTGCCCCTTCAGTGCCCGCCAGTGGCCCGAGGTCCGGGC
CCACATGGCACAGCACTCGAGCCTGCGGCCCCACCAGTGCAGCCAGTGCAGCTTTGCCTCCAAGAACAAG
AAGGACCTGCGGCGACACATGCTGACCCACACCAACGAGAAGCCTTTTGCGTGCCATCTCTGCGGGCAGC
GTTTCAACCGGAATGGGCATCTCAAGTTCCATATCCAGCGGCTGCACAGTCCTGATGGGAGAAAGGCGGG
GACACCTACTGCCCGGACCCCAGGCCGGACCCCCACCCAGACCATCATCCTGAACAGTGATGATGACACG
CTGGCCACGCTGCACACTGCACTCCAGTCCAGTCATGGGGTCCTGGGGCCAGAGCGGCTACAGCAGGCAC
TGGGCCAGGAACACATCATTGTCGCCCAAGAGCAGACAGTGACCAATCAGGAAGAAGCCACCTATATCCA
AGAGATCACCACAGCTGATGGCCAGACAGTACAGCATCTGGTTACCTCTGACAACCAGGTACAATACATC
ATCTCCCAGGACGGAGTCCAGCACCTGCTCCCCCAGGAATATGTCGTGGTTCCAGAGGGCCATCACATCC
AGGTACAGGAAGGCCAGATCACACATATCCAGTATGAACAGGGGGCCCCGTTCCTCCAGGAGTCCCAGAT
CCAGTATGTGCCCGTGTCCCCAGGCCAGCAGCTTGTCACACAGGCCCAACTGGAGGCGGCAGCACACTCA
GCAGTCACAGCGGTGGCCGATGCTGCTATGGCCCAAGCCCAGGGCCTGTTTGGCACAGAAGAAGCAGTGC
CTGAACACATCCAACAGCTGCAGCACCAGGGCATCGAGTACGACGTCATCACCCTGACCGATGAC

>Elephant ENSLAFT00000016152
ATGGAGAACGAGGTGGAGAGCAGTAGCGACGCGACCCCTGGGCCTGGCCGGCCCGAGGAA
CCCTCTGAGAGCGGCCTGGGTGTGGGCACCTCGGAAGCCGTGTCGGCTGACAGCAGCGAC
GCTGCAGCCGCCCCGGGACCGGTAGAGGCCGATGACTCCGGGGTGGGGCAAAGCTCGGAC
CGCGGCAGCAGCTCTCTGGAGGAGGTGACTGAGAGCAGCTTGAGCACAGACCCCCTGCCC
CATGGCTATCTCCCAGATTCGTCTTCTGTGTCCCGTGGTCTGGTGGTGGGGGTGACAGGC
GGCCCCCCAGGCCCAGTGCACTCCAGTGCACTCCCAGACCCTGCCATGCTGGTGTCCGAC
TGCACAGCGTCTTCCTCGGACCTGGGCTCAGCCATCGACAAGATCATTGAGTCCACCATC
GGGCCTGACCTCATCCAGAGCTGCATCACAGTGACCAGTGCTGAGAGTGGCGGGGCCGAG
ACCACACGCTTCCTGATCCTGCAGGGCCCAGATGCAGGAGCCCCCATGGTGTCGCCGATG
GCCAATTCCACCCTGGCTCACAGTCTGGCGGCCATCGAGGCCCTGGCTGATGGCCCCACG
TCCACATCCATGTGCCTGGAGCCGCCGGAGGAGGCGCGGGGTGGGCCCAGCTCCCCAACA
GCACAGCCACCTCCAGGCTCTGGTGCCGAGGAGCCAGACCTGCAGAGCCTGGAGGCCATG
ATGGAGGTGGTGGTGGTGCAGCAGTTCAAGTGCAAGATGTGCCAGTACCGGAGCAGCACT
AAGGCCACGCTGCTGCGCCACATGCGGGAACGGCACTTCCGCCCAGCCTCCCCAGCGGCA
GCAGTGGCAGCAGGTAAGAAAGGACGTCTGCGGAAGTGGGGTACCTCAGCCAAGACCCAG
GAGGAGGAGGGGCCTGAGGAGGAGGACGATGATGACATCGTAGATGCTGGCGCCATTGAC
GACCTGGAGGAGGACAGTGACTACAATCCGGCCGAGGACGAGCCTCGAGGCCGGCAGCTG
CGGCCCCAGCGTCCTGCCCCCAGCACTCCAAGACCCCGAAGGAGACCTGGCCGGCCCCGA
AAGCTGCCTCGCCTGGAGACCTCAGACCTCCCAGGTGTGGAAGGAGAGCCCCTGGTGAGT
TCCCAGACTGGACAGAGCCCCCCGGAGCTGCAGGACCCTGAGGCTCCCAGCTCCTCTGGC
CCAGGACACTGGGCGACCCTGGACAAGTCCGGGAGGGTCCCCGGGGAACCCACTGTGAGC
CAGTCGGACGCAGAGAACGCAGCCCCCTCCAGCCAGGACGAACCCGATGCTCCTCCGCGC
CGTCGTGGCCGGCCCTCCCGGCGCTTCCTCGGCAAGAAATATCGAAAGTACTATTATAAG
TCACCTAAACCACTCCTGAGGCCCTTCCTTTGCCGCATCTGCGGCTCCCGCTTCCTGTCC
CACGAGGACCTGCGCTTCCACGTCAACTCCCATGAGGCCGGCGACCCCCAGCTCTTCAAG
TGCCTGCAGTGCAGCTACCGCTCCCGCCGCTGGTCCTCGCTCAAGGAGCACATGTTCAAC
CATGTGGGCAGTAAGCCCTACAAGTGTGACGAGTGCAGCTACACCAGCGTTTACCGGAAG
GACGTCATCCGACATGCGGCCGTGCACAGCCGGGACCGGAAGAAGAGGCCTGATCCGACC
CCAAAGCTGAGCTCTTTCCCCTGTCCTGTGTGTGGCCGAGTCTACCCCATGCAGAAGAGG
CTTACACAGCACATGAAGACACACAGCACTGAGAAGCCCCACATGTGTGACAAGTGCGGA
AAGTCCTTTAAGAAGCGCTACACCTTCAAAATGCACTTGCTGACGCACATCCAAGCTGTT
GCCAACCGCAGGTTCAAGTGCGAATTCTGTGAGTTTGTCTGCGAGGACAAGAAAGCACTG
CTGAACCACCAGCTGTCCCACGTGAGCGACAAGCCCTTCAAATGCGGCTTCTGTCCCTAC
CGCACCTTCCGAGAGGACTTCCTGCTGTCCCACGTGGCTGTCAAGCACACAGGGGCCAAG
CCCTTCGCATGTGAGTACTGCCACTTCAGCACGCGGCACAAGAAGAACCTACGCCTGCAT
GTACGGTGCCGCCATGCAAGCAGCTTCGAGGAGTGGGGGCGGCGCCACCCTGAGGAGCCC
CCATCCCGCCGCCGCCCCTTCTTCTCTCTGCAGCAGATTGAGGAGCTGAAGCAGCAGCAC
AGTTCAGCCCCCGGGCCACCTCCTAGTCCCACAGGACCTCCTGAGGTCCCCCCAGAGGCA
ACACCATTCCAGGCACCTGAGACCCCCCCGCTGCTCTGTTCTGACACCCTGGGTGGCGCC
ACCATCATCTACCAGCAGGGAGCTGAGGAGTCAACGGCAATGGCCACCCAGACAGCTTTG
GATCTGCTGCTGAACATGAGTGCCCAGCGGGAGCTGGGGGGCACCGCCCTGCAGGTGGCT
GTGGTGAAGTCTGAGGACGTGGAAGCAGAGTTAGCGCCCTCTGGTGGGCAGCCCTCCCCA
GCAGGGGCCCCTCAGCAGGTGGTAACCCTCCACGTGGCAGAGCCAGGGGGCAGCGTGGCA
GCCGAGAGCCAGCTAGGCACCCCTGACCTACAGCAGATCACCCTGGCGCCCGGGCCATTT
GGTGGGGCTGGCTACAGCGTCATCACGGCACCCCCCATGGAGGAGGGGACGTCGGCACCA
GGCACACCATACAGCGAAGAACCCCCTGGGGAGGCAGCCCAGGCCGTGGTGGTGAGTGAG

GCCCTGAAAGAAACCGGCACCCACTACATCATGGCAGCCGACGGTACCCAGCTGCATCAC
ATAGAGCTGACTGCCGACGGCTCCATCTCCTTCCCGAGTCCTGAAGCCCTGGCCTCAGGC
ACCAAGTGGCCCCTGCTGCAGTGTGGGGGTCTGTCCAGAGATGGCCCTGAGCCCCCGTCT
CTAGCCAGGACCCACCGAGGCGGGGAAGTCCAGGCCTCCGTCTCCCCTCCTCCGGCAGCC
AGCAAAGCCCTGGGCCTGGTAGTGCCCCCCTCCCCACCATCTGCAGCTGCTGCAGCATCA
AAGAAGTTTTCCTGCAAGATCTGTGCTGAGGCTTTCCCTGGCCGAGCTGAAATGGAGAGT
CACAAACGGGCCCACGCTGGGCCTAGTGCCTTCAAGTGCCCCGACTGCCCATTCAGTGCA
CGCCAATGGCCCGAGGTCCGGGCCCACATGGCACAGCACTCAAGCCTGCGGCCCCACCAG
TGTAGCCAGTGCAGCTTCGCCTCCAAGAACAAGAAGGATCTGCGGCGGCATGTGCTGACC
CACACCAACGAGAAGCCTTTCGAGTGCCACCTCTGTGGGCAGCGCTTCAACCGCAACGGA
CACCTCAAGTTTCACATCCAGCGACTGCACAGCCCCGATGGAAGGAAGTCAGGGGCTCCT
CCAGCCCGGGCCCCAGCCCGGACCCCCACCCAGACCATCATCCTGAACAGCGATGAGACG
CTGACCACGCTGCACACAGCACTCCAGTCCAGTCATGGGGTCCTGGGCCCAGAGCGACTA
CAGCAGGCACTGGGCCAGGAACACATCATTGTGGCCCAGGAGCAGACAGTGACCAACCAG
GAGGAAGCCACCTATATCCAGGAGATCACTACAGCAGATGGCCAGACGGTACAGCACCTG
GTGACCTCTGACAACCAGGTACAGTACATCATTTCCCAGGACGGAGCCCAGCACCTGCTC
CCCCAGGAGTATGTGGTAGTCCCCGAGGGACATCACATCCAGGTACAGGAGGGCCAGATC
ACACATATCCAGTACGAACAAGGGACTCCGTTCCTTCAGGAGTCCCAGATCCAGTATGTA
CCCATGTCGCCAGGCCAGCAGGTTGTTACACAGGCGCAGCTTGAGGCTGCAGCACACTCA
GCTGTCACAGCAGTGGCCGATGCTGCCATGGCCCAAGCCCAGGGCCTTTTCAGCTCGGAG
GAGGCAGTGCCGGAACACATTCAGCAGCTGCAGCACCAGGGCATCGAGTACGACGTCATC
ACCCTGACTGATGAC
>Dog ENSCAFT00000015707
ATGGAGGAGAACGAGGTGGAGAGCAGCAGCGACGCGGCCCCGGGGCCTGGCCGGCCCGAG
GAGCCCTCTGAGAGCGGCCTGGGCGTGGGCACCTCTGAAGCGGTGTCGGCAGACAGCAGC
GACGCCGCGGCCGCTCCGGGGCCGGCGGAGGCCGACGACTCCGGCGTGGGGCAAAGCTCT
GACCGAGGCTGCGGCTCTCTGGAAGAAGTATCTGAGAGCAGCTCGAGCACAGACCCTCTG
CCCCACGGGTACCTGCCTGATTCATCTTCTGTGTCCCGTGGGCCAGTGGCAGGGGTGACA
GGTGGTCCCCCAGCCCTGGTGCACTCCAGCGCACTCCCAGACCCCAACATGCTGGTGTCC
GACTGCACGGCTTCTTCCTCAGACCTGGGCTCAGCCATTGACAAGATCATCGAGTCCACC
ATTGGGCCAGACCTAATCCCGAGCTGTATCACCGTAACCAGTGCTGAGGATGGTGGGGCC
GAGACTGCACGGTACCTGATCCTGCAGGGCCCAGATGATGGTGCCCCCATGGCTTCGCCA
ATGTCCAGTTCCACCTTGGCTCACAGCCTGGCAGCCATCGAGGCCCTGGCTGATGGCCCC
ACATCCACGTGCCTAGAGCCCCCTGAGGAAGCACGGGGTAGGCCCAGCTCCCCGGCGCAG
CCGCCCCCAGGCTCTGGCACCGAGGAGCCAGACCTGCAGAGCCTGGAGGCCATGATGGAG
GTGGTGGTGGTACAGCAATTCAAGTGCAAGATGTGCCAGTACCGGAGCAGCACCAAAGCC
ACGTTGTTGCGCCATATGCGGGAGCGTCACTTCCGACCAGCAGCAGCAGCTGCGGCTGGA
AAGAAGGGGCGTCTGCGGAAATGGGGCACCTCAGCCAAGACCCAGGAGGAAGAGGGGCCA
GAGGAGGAAGATGATGATGACATCATCGACGCCGGCGCCATTGATGACCTAGAGGAGGAC
AGTGACTACAATCCAGCTGAGGATGAGCCCCGGGGCCGGCAGCTACGGCCCCAGCGCCCT
ACTCCCAGTACTCCAAGACCGAGAAGGAGACCTGGCCGGCCTCGGAAACTGCCTCGCCTA
GAGACCTCGGACCTCCCAGATGGTGTGGAAGGGGAACCTCTGGTGAGTTCCCAGAGCGGA
CAGAGCCCTCCAGAGCCCCCAGACCCCGAAGCACCCAGCTCCTCGGGTCCAGGATGCCTG
GCTGCCCTGGGCAAGGCTGATAGGGCCCCTGTGGACCCTGGTGTGAGCCAGTCGGATGCG
GAGAACGCAGCACCCTCCTGCCCAGATGAGCCTGACGCTCTGCCCCGCCGCCGCGGCCGG
CCCTCCAGGCGTTTCCTGGGCAAGAAATACCGCAAGTATTATTACAAGTCTCCCAAACCC
CTCCTGAGACCTTTCCTGTGCCGCATCTGCGGCTCCCGCTTCCTGTCCCACGAGGACCTG

CGCTTCCACGTCAACTCCCACGAGGCCGGGGACCCCCAGCTCTTCAGGTGTCTGCAGTGT
AGCTACCGCTCCCGCCGCTGGTCTTCACTCAAGGAGCACATGTTCAATCACGTGGGCAGT
AAGCCCTACAAGTGTGACGAATGCAGCTATACCAGTGTCTACCGGAAGGATGTGGTCCGG
CACGCAGCTGTGCACAGTCGAGACCGGAAGAAGAGACCAGATCCGACCCCCAAGCTGAGC
TCCTTCCCTTGCCCTGTGTGTGGCCGTGTCTACCCGATGCAGAAGAGACTCACACAGCAC
ATGAAGACACACAGCACGGAGAAGCCCCACATGTGTGACAAGTGTGGAAAGTCCTTTAAG
AAACGCTACACCTTTAAGATGCACCTGCTCACACACATCCAGGCTGTTGCCAACCGCAGG
TTCAAGTGCGAGTTCTGTGAGTTTGTGTGTGAGGACAAGAAAGCACTACTGAACCACCAG
CTGTCTCATGTCAGTGACAAGCCATTCAAATGCAGCTTCTGCCCCTACCGCACCTTCCGG
GAGGACTTCCTGCTCTCCCATGTGGCTGTCAAGCACACAGGGGCCAAGCCCTTTGCCTGT
GAGTACTGCCACTTCAGCACACGACACAAGAAGAATCTGCGCCTGCATGTACGATGCCGA
CATGCCAGCAGCTTTGAGGAATGGGGGCGGCGCCACCCTGAGGAGCCCCCTTCCCGCCGT
CGCCCCTTCTTCTCCCTGCAGCAGATTGAGGAGCTGAAGCAGCAGCATAGTGCAGCCCCT
GGACCACCCCCCAGCTCCCCAGCACCTCCGGAGATCCCCCCAGAGGCAGCACCTTTCCAG
GCACCCGAGACCCCCCCACTGCTCTGTCCTGACACCCTTGGTGGGGCCACCATCATCTAC
CAGCAAGGTGCTGAGGAGTCGACAGCAATGGCCACGCAGACGGCCTTGGATCTGCTGCTG
AACATGAGTGCACAGCGGGAGCTGGGGGGCACAGCCCTGCAGGTGGCCGTGGTGAAGTCA
GAGGACATGGAAGCAGAGTTAGCATGCCCTGGTGGGCAGCCTTCCCCAGCAGGTGCCACT
CCACAAGTGGTAACCCTCCACGTGGCAGAGCCAGGGAGCAGCGTGGCAGCAGAGAGCCAG
CTAGGCGCTCCTGACCTGCAGCAGATCACCCTGGCACCGGGTCCGTTTGGTGGGACTGGC
TACAGCGTCATCACAGCACCCCCTATGGAGGAGGGGACGTCAGCTCCTGGCACGCCTTAC
AGTGAGGAGCCCCCAGGGGAGGCAGCCCAGGCTGTGGTTGTGAGTGACACCCTGAAGGAA
GCTGGCACCCACTATATCATGGCAGCCGATGGGACCCAGCTGCACCACATCGAGCTGACT
GCAGATGGCTCTATCTCCTTCCCAAATGCGGATTCCCTGGCCTCTGGAGCCAAGTGGCCT
CTGCTACAGTGTGGGGGTCTGCCCAGAGACGGCCCTGAACCCCCATCTCCAGCCAGGACC
CACCGGGTGGGAGACTCCCAGGGCTCTGCTTCCCCACCCCCTGTGGCCAACAAAGCCCTG
GGTCTGGGAGTTCCGCCGTCGCCACCATCTGCAGCCACGGCATCATCAAAGAAGTTTTCC
TGCAAGATCTGTGCTGAGGCCTTCCCTGGCCGTGCAGAGATGGAGAGTCATAAACGGGCC
CACGCCGGACCTAGTGCCTTCAAGTGTCCTGACTGTCCCTTCAGTGCCCGCCAGTGGCCT
GAGGTCCGGGCCCACATGGCACAACACTCAAGCCTGCGGCCTCACCAGTGCAGCCAGTGT
AGCTTTGCCTCCAAGAATAAGAAGGACCTGCGGCGGCACATGCTGACCCATACCAACGAG
AAGCCTTTTGCATGCCACCTCTGTGGACAGCGTTTCAACCGGAATGGCCACCTCAAGTTC
CACATCCAGCGGCTACACAGCCCTGATGGGAGAAAGCCAGGGACACCTACTGCCCGGACC
CCAGCCCGGACTCCCACCCAGACCATCATCCTGAACAGTGACGATGAGACGCTGGCCACA
CTGCACACTGCACTCCAGTCCAGCCACGGGGTCCTGGGCTCAGAGCGGCTACAGCAGGCA
CTGGGTCAGGAGCATATCATTGTGGCCCAGGAACAGACAGTGGCCAATCAGGAAGAAGCC
ACCTACATCCAGGAGATCACCACAGCGGATGGCCAAACAGTGCAGCATCTGGTGACCTCT
GACAACCAGGTACAGTACATCATCTCCCAGGATGGGGTCCAGCATCTGCTCCCCCAGGAA
TATGTTGTGGTTCCAGAGGGCCATCACATCCAGGTACAGGAAGGCCAGATCACACACATC
CAGTATGAACAAGGGGCCCCTTTCCTTCAGGAATCCCAGATCCAGTATGTGCCTGTATCC
CCAGGCCAGCAGCTGGTCACACAGGCCCAACTGGAGGCTGCGGCACACTCAGCCGTCACA
GCGGTGGCTGATGCTGCCATGGCCCAAGCCCAGGGCTTATTTGGCACGGAAGAGGCGGTG
CCTGAACACATCCAACAGCTGCAACACCAGGGCATCGAGTATGATGTCATCACCCTGACG
GATGAC
>Cow ENSBTAT00000061303
ATGGAGGAAAACGAGGTGGAGAGCAGTAGCGACGCGGCCCCTAGGCCTGGCCGGCCCGAG
GAGCCCACGGAGAGCGGGTTGGGTGTGGGCACCTCGGAAGCCGTGTCGGCTGACAGCAGC

GACGCCGCGGCCGCCCCGGGGCCAGCGGAGGCCGACGATTCCGGCGTGGGGCAGAGCTCG
GACCGCGGCAGCAGCTCGCTGGAGGAGGTATCTGAGAGCAGCTCCACCACAGACCCCCTG
CCCCATGGCTACCTCCCTGACTCATCTTCTGTTTCCCGTGGGCCAGCGGCAGGGGTGATG
GGTGGCCCCCCAGCCCTGGTGCACTCCAGTGCACTGCCAGACCCCAACATGCTGGTGTCC
GACTGCACGGCTTCTTCCTCGGACCTGGGCTCGGCCATTGACAAGATCATCGAGTCCACC
ATCGGGACCGACCTCATCCGGAGCTGCATCACCGTGACCAGTGCGGAGGGCAGCGGGGCC
GAGGCCACACGGTACCTGATCCTGCAGGGACCAGACGATGGTGCCCCCATGGCGTCACCA
ATGTCCAGTTCTACCCTGGCCCATAGCCTGGCAGCCATCGAGGCCCTGGCTGATGGCCCC
ACATCCACGTCCACGTGCCTGGAGCCACCGGAGGAAGCGCAGGGTGGGCCCAGCTCCCCG
GCGCAGCCACCCCTGGGTTCTGGCACCGAGGAGCCGGACTTGCAGAGCTTGGAGGCCATG
ATGGAGGTGGTGGTGGTGCAGCAGTTCAAGTGCAAGATGTGCCAGTACCGGAGCAGCACC
AAGGCCACGCTGCTACGCCACATGCGGGAGCGGCACTTCCGCCCAGGTGCGTTGATAGCG
GTAGCAGCTGGTAAGAAGGGGCGTCCGCGCAAGTGGGGCAGCTCAGCCCAGCCCCAGGAG
GAAGAGGGCCCAGAGGAGGAAGACGACGATGACATCATAGATGCTGGTGCCATCGATGAC
CTGGAGGAAGACAGCGACTATAACCCTGCAGATGACGAGCCCCGGGGCCGGCAGCTGCGG
GCCCAGCGCCCCACCCCCAGTACCCTGAGACCCCGCAGGAGACCTGGCCGGCCCCGGAAG
CTGCCTCGCCTGGAGACCTTGGATCTCCCAGATGGTGTGGAAGGAGAGCCTTTAGTGAGT
CCACAAAGTGGACAGAGCCCTCTGGAGCCACAAGACCCCGAGGCACCCAGCTCCTCAGGG
CGGGGACGCCTGGTGGCCCTGGGCAAGGCCAACCGGGCCCCCGTGGAACCCAGTGTGAGC
CAGTCAGATGCGAGGAGCGCGGCGGCATCCTACCAGGATGAGGCTGACGCCCTGCCCCGC
CGCCGTGGTCGACCCTCCAGGCGCTTCCTTGGCAAGAAATACCGCAAGTACTATTACAAG
TCACCCAAACCGCTCCTGCGGCCTTTCCTGTGCCGCATCTGCGGCTCCCGCTTCCTGTCC
CACGAGGATCTGCGCTTCCATGTCAACTCCCACGAGGCCGGCAACCCCCAGCTCTTCAGG
TGCCTGCAGTGCAGCTACCGCTCCCGTCGCTGGTCCTCACTCAAGGAGCACATGTTCAAC
CACGTGGGCAGTAAGCCCTACAAGTGTGACAAATGCAGCTACACTAGCGTCTACCGGAAG
GACGTCATTCGGCACGCGGCTGTGCACAGCCGGGATCGGAAAAAGAGGCCAGACCCGACC
CCAAAGCTGAGCTCCTTCCCCTGCCCCGTGTGTGGCCGTGTCTACCCCATGCAAAAGAGA
CTCACTCAGCACATGAAGACACACAGCACCGAGAAGCCCCACATGTGCGACAAGTGTGGA
AAGTCCTTTAAGAAGCGCTACACCTTCAAAATGCACCTGCTCACGCACATTCAGGCTGTC
GCCAACCGCAGGTTCAAGTGCGAGTTCTGCGAGTTTGTTTGCGAGGACAAGAAGGCGCTG
CTCAACCACCAGCTGTCCCACGTCAGCGACAAGCCCTTCAAGTGTAGCTTTTGCCCCTAC
CGCACCTTCCGAGAGGACTTCCTGCTATCCCACGTGGCCGTCAAGCATACAGGGGCCAAG
CCCTTTGCCTGCGAGTACTGCCACTTCAGCACGCGGCACAAGAAGAACCTGCGGCTGCAT
GTGCGGTGCCGGCACGCCAGCAGCTTCGAGGATTGGGGGCGGCGCCACCCCGAGGAGCCC
CCCTCCCGCCGCCGCCCCTTCTTCTCCCTGCAGCAGATCGAGGAGCTGAAGCAGCAGCAC
AGCACGGCCCCTGGGCCCCCCGCCAGCTCCCCAGGACCTCCTGAGATCCCCTCAGAGGCT
GCACCTTCCCAGTCCCCTGAGACCCCCCCACTGCTCTGTTCTGACACCTTGGGTGGCGCC
ACCATCATTTATCAGCCAGGGACTGCGGAGTCTACAGCCGTGGCCACGCAGACAGCCTTG
GACCTGCTGCTGAACATGAGCACTCAGCGGGAGCTGGGTGGCACAGCCCTGCAGGTGGCC
GTGGTGAAGTCAGAGGACGTGGAAGCGGGGTTAGTGTCCTCTGGGGGGCAGCCCTCCCCA
GCAGGCACCACTCCCCAGGTGGTCACCCTCCACATGGCAGACCCAGGAGGCAGCGTGGCC
GCCGAGAGCCAGCTCGCGCCCCCTGACCTGCAGCAGATCACCCTGGCGCCTGGGCCGTTC
GGCGGGGCTGGCTATAGCGTCATTACAGCCCCCACCGTGGAGGAGGGCACTTCGACTCCC
GGCACGCCTTACAGCGAGGAGCCCCCGGGGGAGGCAGCCCAGACCGTGGTCGTGGGTGAC
ACCCTGAAAGAAGCTGGCACGCACTTCATCATGGCAGCCGACGGACCCAGCTGCACCAC
ATCCAGCTGACTGCAGACAGCTCCATCTCCTTCCCAAGTACGGAAGCCTTGGCCTCCGGC
GCCAAGTGGCCCCTCCTGCAGTATGGGGGGCTGCCCAGAGATGGTCCTGAGCCCCCGGCT

CCAGCCAGGACCCACCAGCCAAGGGACCCTCAGGGCTCTGCCTCCCCACCTCCTGCAGCC
AGCAAACCCCTGGGCCTGGTAGTGCCCCCCTCGCCGCCATCTGCAGCTACTGCCTCATCA
AAGAAGTTTTCCTGCAAGATCTGTGCCGAGGCCTTCACCGGCCGAGCAGAGATGGAGAGT
CACAAACGGGCCCACGCCGGGCCGGGAGCCTTCAAGTGCCCTGACTGTCCGTTCAGCGCT
CGCCAGTGGCCCGAGGTCCGGGCCCACATGGCACAGCACTCGAGCCTGCGGCCCCACCAG
TGCAGCCAGTGCAGCTTCGCCTCCAAGAACAAGAAGGACCTGCGGCGGCACATGCTGACC
CACACCAACGAGAAACCCTTCGAGTGCCAGCTCTGCGGGCAGCGCTTCAACCGCAACGGG
CACCTCAAGTTCCACATCCAGCGGCTCCACAGCCCTGACGGGAGGAAGACGGCAGCCCCC
ACTGCACGGGCCCCAGCCCGGCCCCCCACCCAGACCATCATCCTCAACAGTGATGACGAA
ACGCTGGCCACACTGCACACTGCCTTCCAGTCCAGTCACGGAGTCCTGGGTCCGGAGCGG
CTCCAACAGGCACTGGGCCAGGAACACATCTTTGTGGCCCAGGAACAGACAGTGAGCAAT
CCGGAGGAAGCCGCCTACATCCAAGAGATCACCACGGCAGACGGCCAGATGGTCCAGCAC
CTGGTGGCATCTGACAGCCAGGTACAGTACATCATTTCCCCAGACGGAGTCCAGCAGCTC
CTTCCCCAGGAATATGTGGTGGTGCCCGAGGGCCATCACATCCAGGTACAGGAGGGCCAG
ATCACGCACATCCAGTATGAGCAAGGGGCCCCCTTCCTTCAGGAGTCCCAGATCCAATAC
GTGCCTGTGTCCCCGGGCCAGCAGTTGGTCACCCAGGCCCAGCTCGAGGCTGCGGCACAC
TCAGCTGTCACAGCGGTGGCTGACGCTGCCATGGCCCAAGCCCAGGGCCTCTTTGGCACA
GAGGAGGCGGTGCCCGAACACATCCAACAGCTGCAGCACCAGGGCATCGAATACGACGTC
ATCACCCTGAGCGATGAC
>Ailuropoda melanoleuca XM_011235860.1
ATGGAGGAGAACGAGGTGGAGAGCAGTAGCGACGCGGCCCCTGGGCCTGGCCGGCCAGAGGA
GCCCTCTGAGAGCGGTTTGGGCGTGGGCACCTCTGAAGCGGTGTCGGCCGACAGCAGCGACGCTGCGGCC
GCTCCGGGGCCAGCGGAGGCCGACGACTCTGGCGTGGGGCAAAGCTCCGACCGCGGCAGCAGCTCTCTGG
AGGAAGTATCTGAGAGCAGCTCCAGCACAGACCCCTTGCCCCACGGCTACCTGCCTGATTCATCTTCTGT
GTCCCATGGGCCAGTGGCAGGAGTGACAGGTGGCCCCCCAGCCCTGGTGCACTCCAGCGCACTCCCAGAC
CCCAACATGCTGGTGTCGGACTGCACGGCTTCTTCCTCGGACCTGGGCTCGGCCATCGACAAGATCATCG
AATCCACCATCGGGCCGGACTTAATTCCGAGCTGTATCACCGTAACCAGTGCTGAGGATAGCGGGGCCGA
GACGGCACGGTACCTGATCCTGCAGGGCCCAGACGATGGTGCCCCCATGGCTTCGCCGATGTCCAGTTCC
ACCTTGGCCCACAGCCTGGCAGCCATCGAGGCCCTGGCTGACGGCCCCACATCCACGTGCCTGGAGCCAC
CGGAGGAGGCACGGGGTAGGCCCAGCTCCCCAGCACAGCCGCCCCCGGGCTCTGGTGCTGAGGAGCCAGA
CCTGCAGAGCCTGGAGGCCATGATGGAGGTGGTGGTGGTCCAGCAGTTCAAGTGCAAGATGTGCCAGTAC
CGGAGCAGCACCAAGGCCACGCTGTTGCGCCACATGCGGGAGCGGCACTTTCGGCCAGCAGCAGCTACAG
CTGGGAAGAAGGGGCGTCTGCGGAAGTGGGGCACCTCAGCAAAGACCCAGGAGGAAGAGGGACCAGAGGA
GGAAGACGACGATGACATCATCGATGCCGGCGCCATTGACGACCTAGAGGAGGACAGCGACTACAATCCG
GCCGAGGATGAGCCCCGGGGCCGGCAGCTACGGCCCCAGCGCCCTAGTCCCAGTACGCCAAGACCCCGAA
GGAGACCTGGCCGGCCTCGGAAGCTGCCTCGCCTGGAGATCTCGGACCTCCCTGATGGTGTGGAAGGAGA
GCCTCTAGTGAGTTCCCAGAGTGGACAGAGCCCTCCAGAGCCCCCGGACCCCGAGGCACCCAGCTCCTCG
GGCCCAGGATGCCTGGTTGCCGTGGGCAAGGCCCACAGGGCTGCTGTGGACCCCGGTGTGAGCCAGTCAG
ACGTGGAGAACGCGGCCCCCTCCTGCCAGGATGAGACTGCTGCCCCACCCCGACGCCGGGGCCGACCCTC
CAGGCGCTTCCTAGGCAAGAAATACCGCAAGTATTACTACAAGTCCCCCAAACCTCTCCTGAGGCCCTTC
CTGTGCCGCATCTGCGGCTCCCGCTTCCTGTCCCATGAGGACCTGCGCTTCCATGTCAACTCCCACGAGG
CGGGGGACCCCCAGCTCTTCAGGTGCCTACAGTGTAGCTACCGTTCCCGCCGCTGGTCCTCGCTCAAGGA
GCACATGTTCAATCACGTGGGCAGTAAGCCCTACAAGTGTGACGAGTGCAGCTATACCAGCGTCTACCGG
AAGGACGTGGTCCGGCATGCAGCTGTGCACAGTCGAGACCGGAAGAAGAGGCCAGATCCGGCCCCGAAGC
TGAGCTCTTTCCCTTGCCCTGTGTGTGGCCGTGTCTACCCAATGCAGAAGAGACTCACGCAGCACATGAA
GACACACAGCACCGAGAAGCCCCACATGTGTGACAAGTGCGGCAAGTCCTTTAAGAAGCGCTACACCTTT
AAGATGCACCTGCTGACGCACATCCAGGCCGTGGCCAACCGCAGGTTCAAGTGCGAGTTCTGTGAGTTTG

TCTGTGAGGACAAGAAGGCACTCCTGAACCACCAGCTGTCCCATGTCAGCGACAAGCCGTTCAAGTGCAG
CTTTTGCCCCTACCGCACCTTCCGGGAGGACTTCCTGCTCTCCCACGTGGCTGTCAAGCACACAGGGGCT
AAGCCCTTCGCCTGCGAGTACTGCCACTTCAGCACACGGCACAAGAAGAACCTTCGCCTGCACGTGCGGT
GCCGACACGCAAGCAGCTTCGAGGAGTGGGGGCGGCGCCACCCTGAGGAGCCCCCTTCCCGCCGCCGCCC
CTTCTTCTCTCTGCAGCAGATTGAGGAGCTGAAGCAGCAGCACAGTGCAGCCCCTGGACCCCCCTCCGGC
TCCCCGGGGCCTCCTGAGATTCCCCCAGAGGCAGCGCCTTTCCAGGCACCCGAGACCCCCCCACTGCTCT
GTTCCGACACTCTTGGTGGCGCCACCATCATCTACCAGCAAGGCGCCGAGGAGTCCACAGCGATGGCCAC
GCAGACAGCCTTGGACCTGCTGCTGAACATGAGTGCCCAGCGGGAGCTGGGGGGCACAGCCCTGCAGGTG
GCCGTGGTGAAGTCAGAGGACGTGGAAGCAGAGTTAGCGTCCCCTGGGGGGCAGCCTTCCCCAGCAGGTG
CCACTCCGCAAGTGGTAACCCTCCACATGGCAGAGCCAGGGGCCAGTGTGGCCGCAGAGAGCCAGCTGGG
CGCCCCTGACCTGCAGCAGATCACCTTGGCACCTGGGCCATTTGGTGGGACTGGCTACAGCGTCATCACG
GCACCCCCCATGGAAGAGGGGACATCAGTTCCTGGCACACCTTACAGCGAGGAGCCCCCAGGGGAGGCAG
CCCAGGCTGTGGTTGTGAGCGATGCCTTGAAAGAAGCCGGCACCCACTACATCATGGCAGCGGATGGGAC
CCAGCTGCACCACATCGAGCTGACTGCGGATGGCTCCATCTCCTTCCCAAGTCCGGATTCCCTGGCCTCT
GGGGCCAAGTGGCCCCTGCTACAGTGTGGGGGGCTGCCCAGAGATGGCCCTGAGCCCCCATCTCCAGCCA
GGACGCACCGGGTGGGAGACCCCCAGGGCTCTGCCTCCCCACCTCCTGCGGCCAGCAAATCCCTGAGCCT
GGGAGTACCGCCATCGCCGCCGTCGTCCGCAGCCACAGCGTCATCCAAGAAGTTTTCCTGCAAGATCTGT
GCTGAGGCCTTCCCCGGCCGAGCCGAAATGGAGAGTCACAAACGGGCCCACGCCGGGCCTAGTGCCTTCA
AGTGCCCTGACTGCCCCTTCAGTGCCCGCCAGTGGCCCGAGGTCCGGGCCCACATGGCGCAGCACTCGAG
CCTGCGGCCCCACCAATGCAGCCAGTGTAGCTTTGCCTCCAAGAACAAGAAGGACCTGCGACGGCACATG
CTGACCCACACCAACGAGAAGCCCTTTGCGTGCCACCTCTGCGGGCAGCGTTTCAACCGGAACGGGCATC
TCAAGTTCCACATCCAGCGGCTGCACAGTCCTGATGGGAGAAAGACAGGGACACCGACCGCCCGGACCCC
AGCCCGGACCCCCACTCAGACCATCATCCTGAACAGTGACGACGAGACGCTGGCCACACTGCACACTGCA
CTCCAGTCCAGCCATGGGGTCCTGGGCCCAGAGCGGCTGCAGCAGGCCCTGGGCCAGGAACACATTATTG
TGGCCCAAGAGCAGACAGTGACCAATCAGGAAGAAGCCACCTATATCCAAGAGATCACCACTGCGGACGG
CCAGACAGTACAGCATCTGGTGACCTCCGACAACCAGGTACAGTACATCATCTCCCAGGATGGGGTCCAG
CACCTGCTCCCCCAGGAATATGTGGTGGTTCCAGAGGGCCATCATATCCAGGTACAGGAAGGCCAGATCA
CACACATCCAGTACGAACAAGGGGCCCCTTTCCTTCAGGAGTCCCAGATCCAGTATGTGCCTGTGTCCCC
AGGCCAGCAGCTGGTCACACAGGCCCAACTGGAGGCTGCTGCACACTCAGCCGTCACAGCGGTGGCCGAT
GCCGCCATGGCCCAAGCCCAGGGCCTCTTCGGCGCAGAAGAGGCGGTGCCTGAACACATCCAACAGCTGC
AGCACCAGGGCATCGAGTACGACGTCATCACCCTGACCGATGAC
>Pig ENSSSCT00000008135
ATGGAAGAGAACGAGGTGGAGAGCAGTAGCGACGCGGCCCCTGGGCCTGGCCAGCCCGAA
GAGCCCTCTGAGAGCGGCTTGGGTGTGGGCACCTCGGAAGCCGTGTCGGCCGACAGCAGC
GACGCGGCGGCCGCCCCGGGGCCGGCGGAGGCCGACGACTCCCGCGTGGGTCAGAGCTCG
GACCGCGGTAGCAGCTCTCTGGAGGACGTATCCGAGAGCAGCTCCAGCACAGACCCGCTG
CCCCATGGCTACCTCCCTGATTCATCCTCTGTTTCCCACGGGCCAGTGGCCGGGGTGACG
GGTGCCCCCCCCGCCCTGGTGCACTCCAGCGCACTCCCGGACCCCAACATGCTGGTGTCC
GACTGCACGGCTTCCTCCTCGGACCTGGGCTCAGCCATCGAAAAGATCATCGAGTCCACC
ATCGGGCCCGAGCTCATCCAGAGCTGCATCACTGTGACCAGTGCTGAGGATAGTGGGGCC
AAGACGACACAGTACCTGATCCTGCAAGGGCCTGATGATGGTGCCCCCATGGCATCACCG
ATGTCCAGTTCCACCTTGGCCCACAGCCTGGCAGCCATTGAGGCCCTGGCCGATGGGCCC
ACATCCACATCTACGTGCCTGGAGCCACCTGAGGAGGCTCAGGGTGGGCCCAGCTCCCCA
GTGCAGCCACCCCTGGGTTCTGGCACCGAGGAGCCGGACCTGCAGAGCCTGGAGGCCATG
ATGGAGGTGGTGGTGGTGCAGCAGTTCAAGTGCAAGATGTGCCAGTACCGGAGCAGCACC
AAGGCCACGCTGTTGCGCCACATGCGAGAACGGCACTTCCGCCCAGGCCCCACAGCAGCA
GCAGCTGGTAAGAAGGGACGTCTGCGCAAGTGGGGCCCCTCAGCCAAGACCCAGGAGGAA

GAGGGCCCAGAGGAGGAAGATGACGATGACATTGTAGATGCCGGTGCCATCGATGACCTG
GAGGAGGACAGCGACTACAACCCTGCTGAGGACGAGCCCCGGGGCCGGCAGCTGCGGCCC
CAGCGCCCTGCTCCCAGTACTCCAAGACCCCGAAGGAGGCCTGGCCGTCCGCGGAAGCTG
CCCCGCCTGGAGACCTTGGGCCTCCCTGGTGGTGTGGAAGGAGAGCCTCTAGTGAGTTCC
CTGAGTGGACAGAGCCCTCCAGAGCCGCAAGACCCTGAGGCGCCCAGTTCCTCGGGGCCA
AGACGCCTGGTGGCCCTGAGCAAGGCCGACAGACCCCCCGTGGAGCCTGGTGTGAGCCAG
TCAGATGCGGAGAACGCGGCACCCTCCGGCCAGGACGAGCCCGACGCCCCGCCCCGCCGC
CGGGGTCGACCCTCCAGGCGCTTCCTGGGCAAGAAATACCGCAAGTACTATTACAAGTCT
CCCAAACCGCTCCTGCGGCCCTTCCTGTGCCGCATCTGTGGCTCCCGCTTCCTGTCCCAT
GAGGACCTGCGCTTCCACGTCAACTCCCATGAGGCCGGCGACCCCCAGCTCTTCAAGTGC
CTGCAGTGCAGCTACCGCTCCCGCCGCTGGTCCTCGCTCAAGGAGCACATGTTCAACCAC
GTGGGCAGTAAGCCCTACAAGTGTGACGAATGCAGCTACACCAGCGTCTACCGCAAGGAC
GTCATTCGGCACGCGGCCGTGCACAGCCGGGACCGGAAGAAGAGGCCAGATCCGACCCCG
AAGCTGAGCTCCTTCCCCTGTCCTGTGTGTGGCCGTGTGTACCCCATGCAGAAGAGACTC
ACGCAGCACATGAAGACACATAGCACAGAGAAGCCCCACATGTGCGACAAGTGTGGAAAG
TCCTTTAAGAAGCGTTACACCTTTAAGATGCACCTCCTCACGCACATCCAGGCTGTCGCC
AATCGCAGGTTCAAGTGCGAGTTCTGCGAGTTTGTTTGTGAAGACAAGAAAGCGCTGCTG
AATCACCAGCTGTCCCATGTCAGCGACAAGCCCTTCAAATGCAACTTCTGCCCCTATCGC
ACCTTCCGAGAGGACTTCCTGCTGTCCCATGTGGCTGTCAAGCACACAGGGGCCAAGCCC
TTTGCCTGTGAGTACTGCCACTTCAGCACGCGGCACAAGAAGAATCTCCGCCTGCACGTA
CGGTGCCGACATGCAAGCAGCTTTGAGGAGTGGGGGCGGCGCCATCCTGAGGAGCCCCCT
TCCCGCCGTCGCCCCTTCTTCTCTCTGCAGCAGATTGAGGAGCTGAAGCAGCAGCATAGT
GCGGCCCCTGGACCACCGCCTAGCTCCCCAGGACCTCCTGAGATCCCCCCAGAGACAGCA
CCTTTCCAATCACCCGGGAGCCCCCCGCTGCTCTGTTCTGACACCCTGGGTGGTGCCACC
ATCATCTACCAGCAAGGAGCTGAGGAGTCAACGGCCATGGCCACACAGACAGCCTTGGAT
CTGCTGCTGAACATGAGCGCTCAGCGGGAGCTGGGCGGCACGGCCCTGCAGGTGGCCGTG
GTGAAGTCAGAGGACGTGGAAGCAGAGTTGGCAGCCCCTGTCGGGCAGCCCTCCCCAGCA
GGTGCCACTCCCCAAGTGGTAACTCTCCACATGGCAGAGCCGGGGGGTAGCGCAGCCGCT
GAGAGCCAGCTGGGCCCCCCTGACCTGCGGCAGATCACCCTGGCACCCGGGCCATTTGGC
GGGGCTGGCTACAGTGTCATCACAGCACCCACCATGGAGGAGGGTTCATCAGCTCCTGGA
ACACCTTACAGCGAGGAGCCCCCAGGGGAGGCAGCCCAGGCCGTGGTGGTGAATGACACC
CTGAAAGACGCTGGCACCCACTTCATCATGGCAGCCGATGGGACCCAGCTGCACCACATC
GAGCTGACTGCAGATGGTTCCATCTCCTTCCCAAGCCCAGACGCCCTGGCCTCTGGAGCC
AAGTGGCCCCTGCTGCAGTGTGGGGGGCTGCCCAGAGACAGCGGTCCTGAGCCCCCATCT
CCAGCCAGAACCCACGGGGTAGGGGACCCGCAGGGCTCCGCCTCCCCACCTCCTGCAGCC
AGCAAAGCCCTGGGCCTCGTGGCGCCCTCCTCGCCACCATCTTCAGCCACGGCGTCGTCA
AAGAAGTTTTCCTGCAAGATCTGTGCCGAGGCCTTCCCTGGCCGCGCTGAGATGGAGAGT
CACAAGCGGGCCCACGCGGGGCCCAGTGCCTTCAAGTGCCCCGACTGCCCCTTCAGTGCT
CGCCAGTGGCCCGAGGTCCGGGCCCACATGGCACAGCACTCGAGCCTGAGGCCCCACCAG
TGCAGCCAGTGCAGCTTTGCCTCCAAGAACAAGAAGGACCTGCGGCGGCACATGCTGACC
CACACCAACGAGAAGCCCTTTGCCTGCCACCTCTGCGGGCAGCGTTTCAACCGCAACGGG
CACCTCAAGTTCCACATCCAGCGGCTGCACAGTCCTGATGGCAGGAAGGCGGCGACCCCG
AGCGCACGGGCCCCCGCCCGGACCCCCACCCAGACCATCATCCTCAACAGCGATGACGAG
ACACTGGCCACGTTGCACACTGCGCTGCAGTCCAGTCACGGGGTCCTGGGCCCAGAGCGG
CTACAACAGGCACTGGGCCAGGAACACATCATCGTGGCCCAGGAACAGACAGTGACCAAT
CAGGAAGAAGCCACCTACATCCAGGAGATCACGACAGCAGACGGTCAGACGGTGCAGCAC
CTAGTGACAGCCGATAACCAGGTGCAGTACATCATATCCCAGGACGGAGTCCAGCACATG

CTCCCCCAGGAATACGTTGTGGTCCCAGAAGGCCATCACATCCAGGTGCAGGAGGGCCAG
ATCACACACATCCAGTATGAACAAGGGGGCTCGTTCCTTCAGGAGTCCCAGATCCAGTAT
GTGCCTGTGTCCCCGGGCCAACAGCTCGTCACACAGGCCCAGCTTGAGGCTGCAGCCCAC
TCAGCTGTCACAGCGGTGGCCGATGCTGCCATGGCCCAAGCCCAAGGCCTGTTTGGCACA
GAGGAGGCAGTGCCTGAACACATCCAACAGCTGCAGCACCAGGGCATCGAGTACGACGTC
ATCACCCTGACCGATGAC

>Ferret XM_013053232.1
ATGGAGGAGAACGAGGTGGAGAGCAGTAGCGACGCGGCCCCTGGTCCTGGCCGGCCCGAG
GAGCCCTCTGAGAGCGGCCTGGGCGTGGGCACCTCTGAAGCGGTGTCGGCCGACAGCAGC
GACGCCGCTGCCGCTCGGGGGCCGGCGGAGGCCGACGACTCTGGCGTGGGGCAAAGCTCC
GACCGCGGCAGCAGCTCTCTGGAGGAAGTATCTGAGAGCAGCTCGAGCACGGACCCCTTG
CCCCATGGCTACCTGCCTGATTCATCTTCTGTGTCCCATGGGCCAGTGGCAGGGGTGACA
GGTGGCCCCCCAGCCCTGGTGCACTCCAGCGCACTCCCCGACCCCAACATGCTGGTGTCC
GACTGCACGGCTTCTTCCTCAGACCTGGGCTCAGCCATCGACAAGATCATCGAGTCCACC
ATTGGGCCGGACCTAATCCCAAGCTGTATCACCGTAACCAGTGCTGAGGATGGCGGGGCA
GAGACGGCACGGTACCTGATCCTGCAGGGCCCAGACGATGGTGCCCCCATGGCTTCGCCA
ATGTCCAGTTCCACCTTGGCCCACAGCCTGGCAGCCATCGAGGCCCTGGCTGATGGCCCC
ACATCCACGTGCCTGGAGCCACCAGAGGAGACACAGGGTAGACCCGGCTCTCCCGCACAG
CCACCCCCAGGCTCTGGCACTGAGGAGCCAGACCTGCAGAGCCTGGAGGCCATGATGGAG
GTGGTGGTGGTCCAGCAGTTCAAGTGCAAGATGTGCCAGTACCGGAGCAGCACCAAAGCC
ACGCTGTTGCGCCACATGCGGGAGCGGCACTTCCGACCAGCAGCGGCAGCTGCAGCTGGG
AAGAAGGGGCGTCTGCGGAAGTGGGGCACCTCAGCCAAGACCCGGGAGGAAGAGGGGCCA
GAGGAGGAAGATGATGATGACATCGTTGATGCCGGCGCCATCGATGACTTAGAGGAGGAC
AGTGACTACAATCCGGCTGAGGATGAGCCCCGGGGCCGGCAGCTACGGCCCCAGCGCCCA
ACTCCCAGTACGCCAAGACCCCGAAGGAGACCTGGCCGGCCTCGGAAGCTGCCTCGGCTA
GAGACCTTGGACCTCCCAGGTGTGGAAGGAGAGCCTCTAGTGACTTCCCAGAGTGGACAG
AGCCCTCCAGAGCCCCCAGACCCCGAGGCTCCCAGCTCCTCGGGTCCAGGATGCGTGGTT
GCTCTGGGCAAGGCCGACAGGGCCCCCGTGGACCCCGGTGTGAGCCAGTCAGATGCGGAG
AACGCGGCCCCCTCCTGCCAGGATGAGCCTGCTGCCCCGCCCCGCCGCCGTGGCCGGCCC
TCCAGGCGCTTCCTAGGCAAGAAATACCGCAAGTATTACTACAAGTCCCCCAAACCGCTC
CTGAGGCCCTTCCTGTGCCGCATCTGCGGCTCCCGCTTCCTGTCCCATGAGGACCTGCGC
TTCCATGTCAACTCCCACGAGGCCGGGGACCCCCAGCTCTTCAGGTGCCTGCAGTGTAGC
TATCGTTCCCGCCGCTGGTCCTCACTCAAGGAACACATGTTCAATCACGTGGGCAGTAAG
CCCTACAAGTGTGACGAATGCAGCTATACCAGTGTCTACCGGAAGGACGTGGTCCGTCAC
GCAGCTGTGCACAGTCGAGACCGGAAGAAGAGGCCAGACCCGGCCCCAAAGCTGAGCTCC
TTCCCTTGTCCTGTGTGTGGCCGTATCTATCCGATGCAGAAGAGACTCACACAGCACATG
AAGACACACAGCACAGAGAAACCCCACATGTGTGACAAGTGTGGAAAGTCCTTTAAGAAG
CGCTACACCTTCAAGATGCACCTGCTCACACACATCCAAGCTGTTGCCAACCGCAGGTTC
AAGTGCGAGTTCTGTGAGTTTGTTTGTGAGGACAAGAAGGCACTCCTGAACCACCAGCTG
TCCCATGTCAGTGACAAGCCATTCAAGTGCAGCTTTTGCCCCTACCGCACCTTCCGGGAG
GACTTCCTGCTCTCCCACGTGGCTGTTAAGCACACAGGGGCCAAGCCCTTTGCCTGTGAA
TACTGCCACTTCAGCACACGGCACAAGAAGAACCTGCGCCTGCACGTGCGCTGCCGACAC
GCCAGCAGCTTTGAGGAGTGGGGACGGCGCCACCCTGAGGAGCCCCCTTCCCGCCGTCGC
CCCTTCTTCTCTCTGCAGCAGATTGAGGAGCTAAAGCAGCAGCACAGCGCAGCCCCCGGC
CCACCCCCAAGCTCCCCGGGCCCTCCTGAGATTCCCCCAGAAGCAGCACCTTTCCAGGCA
CCCGAGACCCCCCCACTGCTCTGTTCTGACACGCTTGGTGGCGCCACCATCATCTACCAG

CAAGGCGCTGAGGAGTCCACGGCAATGGCCACACAGACGGCCTTGGACCTGCTGCTGAAC
ATGAGCGCCCAGCGGGAGCTGGGGGGCACAGCCCTGCAGGTGGCTGTGGTGAAGTCCGAG
GACGTGGAAGCAGAGTTGGCATCCCCGGGTGGGCAGCCCTCCCCGGCAGGGGCCACTTCG
CAGGTGGTAACTCTCCATGTGGCAGAGCCCGGAGGCAGTGTGGCAGCAGAGAGCCAGCTA
GGCCCCCCTGACCTACAGCAGATCACCCTGGCACCTGGCCCGTTCGGTGGGACTGGCTAC
AGCGTCATCACAGCACCCCCTATGGAGGAGGGGACATCAGCTCCTGGCACGCCTTACAGC
GAGGAGCCCCCAGGGGAGGCAGCCCAGGCTGTGGTGGTGAGTGACACCCTGAAAGAAGCT
GGCACCCACTACATCATGGCAGCTGATGGAACCCAGCTACACCACATCGAGCTGACTGCA
GATGGTTCCATCTCCTTCCCAAGTCCAGATTCCCTGGCCTCCGGAGCCAAGTGGCCCCTG
CTACAGTGTGGGGGGCTGCCCAGAGATGGCCCTGAGCCCCCATCTCCAGCCAGGACTCAC
CGGGTGGGAGACCCCCAGGGCTCTGCCTCCCCACCTCCTGCGGCCAGCAAAGCCCTGAGC
CTGGGAGTACCACCGTCCCCACCGTCGGCAGCCGCGGCCTCATCGAAGAAGTTTTCCTGC
AAGATCTGTGCTGAGGCCTTCCCTGGCCGAGCAGAGATGGAGAGCCACAAACGGGCCCAC
GCTGGGCCTAGTGCCTTCAAGTGCCCCGACTGCTCCTTCAGTGCCCGCCAGTGGCCCGAG
GTCCGGGCCCACATGGCACAGCACTCAAGCCTGCGGCCCCACCAGTGCAGCCAGTGCAGC
TTTGCCTCCAAGAACAAGAAGGACCTGCGGCGGCACATGCTGACCCACACCAATGAGAAA
CCCTTCGCGTGCCACCTCTGCGGGCAGCGCTTTAACCGTAACGGGCATCTCAAGTTCCAC
ATCCAGCGGCTGCACAGTCCTGATGGGAGAAAGACAGGGACACCGACTGCCCGGACCCCA
GCCCGGACCCCCACCCAAACCATCATCCTGAACAGTGACGACGAGACGCTGGCCACACTG
CACACTGCACTCCAGTCCGGCCATGGGGTCCTGGGCCCAGAGCGGCTGCAGCAGGCACTA
GGCCAGGAACACATCATTGTGGCCCAGGAGCAGACCGTGACCAATCAGGAAGAAGCCACC
TACATCCAGGAGATCACCACCGCAGACGGCCAGACGGTACAGCACCTGGTGACCTCTGAC
AATCAGGTACAGTACATCATCTCCCAGGATGGAGTCCAGCACCTGCTCCCCCAGGAATAT
GTGGTGGTTCCAGAGGGCCATCACATCCAGGTACAGGAAGGCCAGATCACACACATCCAG
TATGAACAAGGGGCCCCTTTCCTTCAGGAGTCCCAGATCCAGTATGTGCCTGTGTCCCCA
GGCCAGCAGCTGGTCACACAGGCCCAGCTGGAGGCTGCTGCACACTCAGCTGTCACAGCG
GTGGCCGATGCTGCCATGGCCCAAGCCCAGGGCCTATTTGGTGCAGAAGAGGCGGCGCCT
GAACACATCCAACAGCTGCAGCACCAGGGCATCGAGTACGACGTCATCACCCTGACCGAC
GAC
>Pteropus vampyrus XM_011369316.2
ATGGAGGAGAACGAGGTGGAGAGTAGTAGCGACGCGGCCCCTGGGCCTGACCAGCCGGAGGAGCCC
TCTGAGAGCGGCTTGGGTATGGGCACCTCGGAAGCCGTGTCCGCCGACAGCAGCGACGCCGTGGCCGCCC
CAGGGCCAACAGAGGCCGATGACTCCGGCGTGGGGCAAAGCTCGGATCGCGGCAGCAGCTCTCTGGAGGA
GGTATCTGAGAGCAGCTCAAGTACAGACCCCCTGCCCCATGGCTACCTCCCCGATTCATCTTCTGTATCC
CATGGGCCAGTGGCAGGGGTGACGGAGGGCCCCCCAGCCCTCGTGCACTCCAGCGCACTCCCAGACCCCA
ACATGCTGGTATCCGACTGCACAGCTTCTTCCTCGGACCTGGGTTCAGCCATCGACAAGATCATTGAGTC
CACCATTGGGCCCGACCTCATCCAGAGCTGCATCACTGTGACCAGTGCTGAGGATGGTGGGCCTGAGACT
ACACGGTACCTGATCCTGCAGGGACCAGACGATGGTGCCCCCATGGCATCACCAATGTCCAGTTCCACCC
TGGCCCACAGTCTGGCAGCCATCGAGGCCCTGGCTGATGGCCCCACATCCACATCCACGTGCCTGGAGCC
TCCTGAGGAAGCCCAGGGTGGGCCCAGCTCCCCAGCACAGCCTCCCCCAGCTTCTGGCACTGAAGAGCCC
GACCTACAGAGCCTGGAGGCCATGATGGAGGTGGTGGTAGTGCAGCAATTCAAGTGCAAGATGTGTCAGT
ACCGGAGCAGCACGAAGGCCACACTATTGCGCCACATGCGGGAGAGACACTTCCGCCCAGCAGCAGCAGC
AACTAGTAAGAAGGGACGTCCACGGAAGTGGGGCACCTCCACCAAGACCCAGGAGGAAGAGGCACCAGAG
GAGGAAGACGATGATGACATTGTGGACGCTGGTGCTATTGACGACCTGGAGGAAGACAGTGACTACAATC
CAGCTGAAGATGAGCCCCGGGGCCGGCAGCTACGACCTCAGCGCCCCACTCCCAGTACCCTGAGACCCCG
TAGGAGACCTGGCCGGCCCCGGAAGCTCCCCTGCCTGGAGACAGACCTCCCGGATGGTGTGGAAGGAGAG
CCTCTAGTGACTTCCCAGAGCGGACAGAACCCTCCGGAGCCACAGGACCCTGAGGCACCCAGCTCCTCAG

GCCCAGGACGCCTGGTGGCCTTGGGCAAGACGGGTAGGGTTCCCGTGGAGCCCAGTGTGAGCCAGTCAGA
TGCAGAGAACACAGCACCCTCCTGCCAGGAGGAGCCTGACACCCCACCCCGCCGCCGTGGCCGACCCTCC
AGGCGCTTCCTAGGCAAGAAATACCGCAAGTATTATTATAAGTCACCCAAACCACTTCTGCGGCCCTTTC
TGTGCCGCATCTGTGGCTCCCGCTTCCTATCCCATGAGGACCTGCGCTTCCACGTAAACTCCCATGAGGC
CGGAGACCCCCAGCTCTTCAAGTGCCTGCAGTGCAGCTATCGCTCCCGCCGCTGGTCCTCACTCAAGGAG
CACATGTTCAACCATGTGGGCAGTAAGCCCTACAAGTGTGACGAATGCAGCTATACCAGCGTCTACCGGA
AGGACGTCATCCGGCATGCAGCTGTGCACAGCAGGGACCGGAAGAAGAGGCCAGATCCGACCCCAAAGCT
GAGCTCCTTCCCCTGCCCCGTGTGTGGTCGTGTCTACCCCATGCAGAAGAGACTCACGCAGCACATGAAG
ACACACAGCACTGAGAAGCCCCACATGTGTGACAAGTGCGGAAAGTCCTTTAAGAAGCGCTACACCTTCA
AGATGCACCTGCTCACACACATCCAGGCTGTTGCCAACCGCAGATTCAAGTGTGAGTTCTGCGAGTTTGT
TTGCGAGGACAAGAAGGCACTGCTGAACCACCAGCTGTCCCATGTCAGTGACAAGCCCTTCAAATGCAGC
TTTTGCCCCTATCGCACCTTCCGGGAGGACTTCCTGCTGTCTCACGTGGCTGTCAAGCACACTGGGGCCA
AGCCCTTTGCTTGTGAGTACTGCCACTTCAGCACACGGCACAAGAAGAATCTACGCCTGCACGTCCGGTG
CCGACACGCAAGCAGCTTCGAGGAGTGGGGGCGGCGCCACCCTGAGGAGCCCCCCTCCCGCCGTCGTCCC
TTCTTCTCTCTGCAACAGATTGAAGAGCTGAAGCAACAGCACAGTACAGCCCCTGGACCACCCGCCAGCT
CCCTAGGACCTCCCGAGGTCCCCCCAGAGGCAGCACCTTTACAGCCACCTGAGGCCCCCCCACTGCTCTG
TTCTGACACCCTGGGTGGCGCCACCATCATCTATCAGCAAGGAGCTGAGGAGTCAACCGCTATGGCCACA
CAGACAGCCTTGGATCTGCTGCTGAACATGAGTGCTCAGCGGGAACTGGGGGGCGCCGCCTTGCAGGTGG
CTGTGGTGAAGTCAGAGGATGCGGAAGCAGATTTAGCATCCCCTGGTGGGCAGCCCTCCCCAGCAAGTGC
CACTCCACAAGTGGTAACCCTCCATGTGGCAGAGCCGGGAGGCGGTGTGGCAGCCGAGAGCCAGATAGGC
ACCCCTGACCTACAGCAGATCACCCTGGCACCCGGTCCATTTGGCGCGGCTGGCTACAGCGTCATCACAG
CACCCCCTATGGAAGAGGGGACATCAGCTCCTGGCACACCTTACAGTGAGGAGCCCCCAGGGGAGGCAGC
CCAGGCTGTGGTTGTGAGTGACACCCTGAAGGAATCTGGCGCCCACTACATCATGGCAGCCGACGGGACC
CAGCTGCATCACATCGAGCTGACTGCAGATGGCTCCATTTCCTTCCCAAGTCCTGATGCCCTGCCCTCTG
CAACAAAGTGGCCCCTGCTGCAATGTGGGGGGCCACCCAGAGACGGTCCTGAGCCCCCCTCTCCAGCCAG
GACTCACCCGCTAGGGGACCCTCAGGGCTCTGCCTCCCCACCTCCTACAGCCAGCAAAGCCCTGGGCCTG
GTAGCGCCCCCCTCATCGCCATCTGCAGCCACTGCATCGTCAAAGAAGTTCTCTTGCAAGATTTGTGCAG
AGGCCTTCCCTGGGCGAGCTGAGATGGAGAGTCACAAACGGGCCCACGCCGGGCCGAGTGCCTTCAAGTG
CCCCGACTGCCCCTTCAGCGCCCGCCAGTGGCCCGAGGTCCGGGCCCATATGGCGCAGCACTCGAGCCTG
CGGCCCCACCAGTGCAGCCAGTGCAGCTTTGCTTCCAAGAACAAGAAGGACCTGCGGCGGCACATGCTGA
CCCACACCAATGAGAAGCCTTTTTCATGCCACCTCTGCGGGCAGCGTTTCAACCGGAACGGGCACCTCAA
GTTCCACATCCAGCGGCTACATAGTCCCGATGGGAGAAAGGCAGGGACCCCTACTGCCCGGGCCCCAGCC
CAGACCCCCACCCAGACCATCATCCTGAACAGTGATGACGAGACACTGGCCACACTGCACACTGCACTCC
AGTCCAGTCACGGAGTCCTGGGCCCAGAGCAACTTCAGCAGGCACTGGGCCAGGAACACATTATTGTGGC
CCAGGAGCAGACGGTGACCAATCAGGAGGAAGCCACCTACATTCAGGAGATCACCACAGCAGACGGCCAG
ACAGTACAGCATCTGGTGACCTCTGACAACCAGGTACAGTACATCATCTCTCAGGACGGAGTCCAGCACC
TGCTCCCCCAGGAATATGTTGTAGTCCCAGAGGGCCATCACATCCAGGTACAGGAGGGCCAGATCACACA
CATCCAGTACGAACAAGGAGCCCCATTCCTTCAGGAGTCCCAGATCCAGTATGTGCCTGTGTCTCCAGGC
CAGCAGCTTGTCACACAGGCCCAGCTTGAGGCTGCAGCACACTCAGCTGTAACAGCAGTGGCTGATGCTG
CCATGGCCCAAGCCCAGGGCCTATTTGGCACAGAGGAGGCAGTGCCTGAACACATCCAGCAGCTGCAACA
CCAGGGCATCGAGTACGACGTCATCACCCTGACCGATGAC
>Pteropus alecto XM_006921877.3
ATGGA
GGAGAACGAGGTGGAGAGTAGTAGCGACGCGGCCCCTGGGCCTGACCAGCCGGAGGAGCCCTCTGAGAGC
GGCTTGGGTATGGGCACCTCGGAAGCCGTGTCCGCCGACAGCAGCGACGCCGTGGCCGCCCCAGGGCCAA
CAGAGGCCGATGACTCCGGCGTGGGGCAAAGCTCGGATCGCGGCAGCAGCTCTCTGGAGGAGGTATCTGA
GAGCAGCTCAAGTACAGACCCCCTGCCCCATGGCTACCTCCCCGATTCATCTTCTGTATCCCATGGGCCA

GTGGCAGGGGTGACGGAGGGCCCCCCAGCCCTCGTGCACTCCAGCGCACTCCCAGACCCCAACATGCTGG
TATCCGACTGCACAGCTTCTTCCTCGGACCTGGGTTCAGCCATCGACAAGATCATTGAGTCCACCATTGG
GCCTGACCTCATCCAGAGCTGCATCACTGTGACCAGTGCTGAGGATGGTGGGCCTGAGACTACAAGGTAC
CTGATCCTGCAGGGACCAGACGATGGTGCCCCCATGGCATCACCAATGTCCAGTTCCACCCTGGCCCACA
GTCTGGCAGCCATCGAGGCCCTGGCTGATGGCCCCACATCCACATCCACGTGCCTGGAGCCTCCTGAGGA
AGCCCAGGGTGGGCCCAGCTCCCCAGCACAGCCTCCCCCAGCTTCTGGCACTGAAGAGCCCGACCTACAG
AGCCTGGAGGCCATGATGGAGGTGGTGGTAGTGCAGCAATTCAAGTGCAAGATGTGTCAGTACCGGAGCA
GCACGAAGGCCACACTATTGCGCCACATGCGGGAGAGACACTTCCGCCCAGCAGCAGCAGCAACTAGTAA
GAAGGGACGTCCACGGAAGTGGGGCACCTCCACCAAGACCCAGGACGAAGAGGCACCAGAGGAGGAAGAC
GATGATGACATTGTGGACGCTGGTGCTATTGACGACCTGGAGGAAGACAGTGACTACAATCCAGCTGAAG
ATGAGCCCCGGGGCCGGCAGCTACGACCTCAGCGCCCCACTCCCAGTACCCTGAGACCCCGTAGGAGACC
TGGCCGGCCCCGGAAGCTCCCCTGCCTGGAGACAGACCTCCCGGATGGTGTGGAAGGAGAGCCTCTAGTG
ACTTCCCAGAGTGGACAGAACCCTCCGGAGCCACAGGACCCTGAGGCACCCAGCTCCTCAGGCCCAGGAC
GCCTGGTGGCCTTGGGCAAGACGGGTAGGGTTCCCGTGGAGCCCAGTGTGAGCCAGTCAGATGCAGAGAA
CACAGCACCCTCCTGCCAGGAGGAGCCCGACACCCCACCCCGCCGCCGTGGCCGACCCTCCAGGCGCTTC
CTAGGCAAGAAATACCGCAAGTATTATTATAAGTCACCCAAGCCACTTCTGCGGCCCTTTCTGTGCCGCA
TCTGTGGCTCCCGCTTCCTATCCCATGAGGACCTGCGCTTCCACGTAAACTCCCATGAGGCCGGAGACCC
CCAGCTCTTCAAGTGCCTGCAGTGCAGCTATCGCTCCCGCCGCTGGTCCTCACTCAAGGAGCACATGTTC
AACCATGTGGGCAGTAAGCCCTACAAATGTGACGAATGCAGCTATACCAGCGTCTACCGGAAGGACGTCA
TCCGGCATGCAGCTGTGCACAGCAGGGACCGGAAGAAGAGGCCAGATCCGACCCCAAAGCTGAGCTCCTT
CCCCTGCCCCGTGTGTGGTCGTGTCTACCCCATGCAGAAGAGACTCACGCAGCACATGAAGACACACAGC
ACTGAGAAGCCCCACATGTGTGACAAGTGCGGAAAGTCCTTTAAGAAGCGCTACACCTTCAAGATGCACC
TGCTCACACACATCCAGGCTGTTGCCAACCGCAGATTCAAGTGTGAGTTCTGCGAGTTTGTTTGCGAGGA
CAAGAAGGCACTGCTGAACCACCAGCTGTCCCATGTCAGTGACAAGCCCTTCAAATGCAGCTTTTGCCCC
TATCGCACCTTCCGAGAGGACTTCCTGCTGTCTCACGTGGCTGTCAAGCACACTGGGGCCAAGCCCTTTG
CTTGTGAGTACTGCCACTTCAGCACACGGCACAAGAAGAATCTACGCCTGCACGTCCGGTGCCGACACGC
AAGCAGCTTTGAGGAGTGGGGGCGGCGCCACCCTGAGGAGCCCCCCTCCCGCCGTCGTCCCTTCTTCTCT
CTGCAACAGATTGAAGAGCTGAAGCAACAGCACAGTACAGCCCCTGGACCACCCGCCAGCTCCCTAGGAC
CTCCCGAGGTCCCCCCAGAGGCAGCACCTTTACAGCCACCTGAGGCCCCCCCACTGCTCTGTTCTGACAC
CCTGGGTGGCGCCACCATCATCTATCAGCAAGGAGCTGAGGAGTCAACCGCTATGGCCACACAGACAGCC
TTGGATCTGCTGCTGAACATGAGTGCTCAGCGGGAACTGGGGGGCGCCGCCTTGCAGGTGGCTGTGGTGA
AGTCAGAGGATGCGGAAGGAGATTTAGCATCCCCTGGTGGGCAGCCCTCCCCAGCAAGTGCCACTTCACA
AGTGGTAACCCTCCATGTGGCAGAGCCGGGAGGCGGTGTGGCAGCCGAGAGCCAGATAGGCACCCCTGAC
CTACAGCAGATCACCCTGGCACCCGGTCCATTTGGCGCGGCTGGCTACAGCGTCATCACGGCACCCCCTA
TGGAAGAGGGGACATCGGCTCCTGGCACACCTTACAGTGAGGAGCCCCCAGGGGAGGCAGCCCAGGCTGT
GGTTGTGAGTGACACCCTGAAGGAATCTGGCGCCCACTACATCATGGCAGCCGACGGGACCCAGCTGCAT
CACATCGAGCTGACTGCAGATGGCTCCATTTCCTTCCCAAGTCCTGATGCCCTGCCCTCTGCAACAAAGT
GGCCCCTGCTGCAATGTGGGGGGCCACCCAGAGACGGTCCTGAGCCCCCCTCTCCAGCCAGGACTCACCC
GCTAGGGGACCCTCAGGGCTCTGCCTCCCCACCTCCTACAGCCAGCAAAGCCCTGGGCCTGGTAGCGCCC
CCCTCATCGCCATCTGCAGCCACTGCATCGTCAAAGAAGTTCTCTTGCAAGATCTGTGCAGAGGCCTTCC
CTGGGCGAGCTGAGATGGAGAGTCACAAACGGGCCCACGCTGGCCCGAGTGCCTTCAAGTGCCCTGACTG
CCCCTTCAGCGCCCGCCAGTGGCCCGAGGTCCGGGCCCATATGGCGCAGCACTCGAGCCTGCGGCCCCAC
CAGTGCAGCCAGTGCAGCTTTGCTTCCAAGAACAAGAAGGACCTGCGGCGGCACATGCTGACCCACACCA
ATGAGAAGCCTTTTTCATGCCACCTCTGCGGGCAGCGTTTCAACCGGAACGGGCACCTCAAGTTCCACAT
CCAGCGGCTACATAGTCCCGATGGGAGAAAGGCAGGGACCCCTACTGCCCGGGCCCCAGCCCAGACCCCC
ACCCAGACCATCATCCTGAACAGTGATGACGAGACACTGGCCACACTGCACACCGCACTCCAGTCCAGTC
ACGGAGTCCTGGGCCCAGAGCAACTTCAGCAGGCACTGGGCCAGGAACACATTATTGTGGCCCAGGAGCA

GACGGTGACCAATCAGGAGGAAGCCACCTACATTCAGGAGATCACCACAGCAGACGGCCAGACAGTACAG
CATCTGGTGACCTCTGACAACCAGGTACAGTACATCATCTCTCAGGACGGAGTCCAGCACCTGCTCCCCC
AGGAATATGTTGTAGTCCCAGAGGGCCATCACATCCAGGTACAGGAGGGCCAGATCACACACATCCAGTA
CGAACAAGGAGCCCCATTCCTTCAGGAGTCCCAGATCCAGTATGTGCCTGTGTCTCCAGGCCAGCAGCTT
GTCACACAGGCCCAGCTTGAGGCTGCAGCACACTCAGCTGTAACAGCAGTGGCTGATGCTGCCATGGCCC
AAGCCCAGGGCCTATTTGGCACAGAGGAGGCAGTGCCTGAACACATCCAGCAGCTGCAACACCAGGGCAT
CGAGTACGACGTCATCACCCTGACCGATGAC
>Odobenus rosmarus divergens XM_004405257.2
ATGGAGGAGAACGAGGTGGAGAGCAGTAGCG
ACGCGGCCCCGGGGCCTGGCCGGCCCGAGGAGCCCTCTGAGAGCGGCTTGGGCGTGGGCACCTCTGAAGC
GGTGTCGGCCGACAGCAGCGACGCCGCGGCGGCTCCGGGGTCGGCGGAGGCCGACGACTCTGGCGTGGGG
CAAAGCTCCGACCACGGCAGCAGCTCTCTGGAGGAAGTATCTGAGAGTAGCTCGAGCACAGACCCCTTGC
CCCACGGCTACCTGCCTGATTCATCTTCTCTGTCCCATGGGCCAGTGGCAGGGATGACAGGTGGCCCCCC
AGCCCTGGTGCACTCCAGCGCACTGCCAGACCCCAACATGCTGGTGTCTGACTGCACAGCTTCTTCTTCG
GACCTGGGCTCGGCCATCGATAAGATCATTGAGTCCACCATCGGGCCGGACCTAATCCCGAGCTGTATCA
CCGTAACCAGTGCTGAGGATGGCGGGGCCGAGACGGCACGGTACCTGATCCTGCAGGGCCCAGACGATGG
TGCCCCCATGGCCTCGCCGATGTCCAGTTCCACCTTGGCCCACAGCCTGGCAGCCATCGAGGCCCTGGCT
GATGGCCCCACATCCACGTGCCTAGAGCCACCGGAGGAGGCACGGGGTAGGCCCAGCTCCCCCACGCAGC
CGCCCCCGGGCTCTGGCGCCGAGGAGCCAGACCTGCAGAGCCTGGAGGCCATGATGGAGGTGGTGGTGGT
CCAGCAGTTCAAGTGCAAGATGTGCCAGTACCGGAGCAGCACCAAGGCCACGCTGTTGCGCCACATGCGG
GAACGGCACTTCCGACCAGCTTCTCTGATCTCCGCAGCAGCAGCTGCAGCGGGGAAGAAGGGGCGTCTGC
GGAAGTGGGGCACCTCAGCCAAGACCCAGGAGGAAGAGGGGCCAGAGGAGGAAGATGACGATGACATCGT
CGACGCTGGCGCCATCGACGACCTGGAGGAGGACAGTGACTACAATCCGGCCGAGGATGAGCCCCGGGGC
CGGCAGCTACGTCCCCAGCGCCCTACTCCCAGTACGCCAAGACCCCGAAGGAGACCTGGCCGGCCTCGGA
AGTTGCCTCGCCTAGAGACCTCAGACCTCCCAGATGGTGTGGAAGGAGAGCCTCTAGTGAGTTCCCAGAG
CGGACAGAGCCCTCCAGAGCCCCCGGACCCCGAGGCACCCAGCTCTTCGGGCCCAGGATGCCTGGTTGCC
CTGGGCAAGGCCGCCAGGGCCCCCGTGGACCCCAGCGTGAGCCAGTCAGATGCGGAGAATGCTGCCCCCT
CCTGCCAGGATGAGCCTGCTGCCCCGCCCCGCCGACGGGGCAGACCCTCCAGGCGCTTCCTAGGCAAGAA
ATACCGCAAGTATTACTACAAGTCTCCCAAACCGCTCCTGAGGCCCTTCCTGTGCCGCATTTGCGGCTCC
CGCTTCCTGTCCCACGAGGACCTGCGCTTCCACGTCAACTCCCACGAGGCCGGGGACCCCCAGCTCTTCA
GGTGCCTGCAGTGTAGCTACCGCTCCCGCCGCTGGTCCTCGCTCAAGGAGCACATGTTCAATCACGTGGG
CAGTAAGCCCTACAAGTGTGACGAATGCAGCTATACCAGCGTCTACCGGAAGGACGTGGTCCGACACGCG
GCTGTGCACAGTAGAGACCGGAAGAAGAGACCAGATCCGGCCCCAAAGCTGAGCTCCTTCCCTTGCCCTG
TGTGTGGCCGTGTCTACCCGATGCAGAAGAGACTCACGCAGCACATGAAGACACACAGCACGGAGAAGCC
CCACATGTGTGACAAGTGTGGAAAGTCCTTTAAGAAGCGCTACACCTTTAAAATGCACCTGCTCACGCAC
ATCCAGGCTGTTGCCAACCGCAGGTTCAAGTGTGAGTTCTGTGAATTTGTTTGTGAGGACAAGAAGGCAC
TACTGAACCACCAGCTGTCCCATGTCAGTGACAAGCCATTCAAGTGCAGCTTTTGCCCCTACCGCACCTT
CCGGGAGGACTTCCTGCTCTCTCACGTGGCCGTCAAGCACACAGGGGCCAAGCCCTTTGCCTGTGAGTAC
TGCCACTTCAGCACACGGCACAAGAAGAACCTACGCCTGCACGTGCGATGCCGACACGCAAGCAGCTTTG
AGGAGTGGGGGCGGCGCCACCCTGAGGAGCCGCCTTCCCGCCGTCGCCCCTTCTTCTCTCTGCAGCAGAT
TGAGGAGCTGAAGCAGCAGCACAGTGCAGCCCCTGGACCAGCCTCCTGCTCCCCGGGACCTCCTGAGATT
CCCCCCGAGGCAGCACCTTTCCAGGCACCCGAGACCCCCCCACTGCTCTGTTCTGACACGCTTGGTGCCG
CCACCATCATCTACCAGCAAGGCGCTGAGGAGTCCACGGCAATGGCCACGCAGACGGCCTTGGATCTGCT
GCTGAACATGAGCGCCCAGCGGGAGCTGGGGGACACAGCCCTGCAGGTGGCCGTGGTGAAGTCCGAGGAC
GTGGAAGCAGAGTTAGCATCCCCTGGTGGGCAGCCCTCCCCAGCAGGTGCCACTCCGCAAGTGGTAACCC
TCCACGTGGCCGAGCCGGGCGGCAGCGTGGCAGCAGAGAACCAGCTAGGTGCCCCTGACCTGCAGCAGAT
CACCCTGGCACCTGGTCCATTTGGTGGGACTGGCTACAGCGTCATCACTGCACCCCCTATGGAGGAGGGG

ACGTCAGCTCCTGGCACGCCTTACAGCGAGGAGCCCCCAGGGGAGGCAGCCCAGGCTGTGGTTGTGAGCG
ACACCCTGAAAGAAGCTGGTACCCACTACATCATGGCAGCCGACGGGACCCAGCTGCACCACATTGAGCT
GACTGCAGATGGCTCCATCTCCTTCCCAAGCCCGGATTCCCTGGCCTCTGGAGCCAAGTGGCCCCTACTA
CAGTGTGGGGGGCTGCCGAGAGATGGCCCTGAGCCCCCATCTCCAGCCAGGACCCACCGGGTGGGAGACA
CCCAGGGCTCTGCCTCCCCACCCCCCGCAGCCAGCAAAGCCCTGAGCCTGGGAGTGCCGCCATCGCCACC
GTCCGCAGCCACGGCCTCCTCCAAGAAGTTTTCCTGCAAGATCTGTGCTGAGGCCTTCCCTGGCCGAGCA
GAGATGGAGAGTCACAAACGGGCCCATGCCGGGCCTAGTGCCTTCAAGTGCCCCGACTGCCCCTTCAGTG
CCCGCCAGTGGCCCGAGGTCCGGGCCCACATGGCACAGCACTCGAGCCTGCGGCCCCACCAGTGCAGCCA
GTGTAGCTTTGCCTCCAAGAACAAGAAGGACCTGCGGCGGCACATGTTGACGCACACCAATGAGAAGCCC
TTCGCGTGCCACCTCTGCGGGCAGCGTTTCAACCGGAACGGGCACCTCAAGTTCCACATCCAGCGGCTAC
ACAGTCCTGACGGGAGAAAGACGGGGACCCCGACTGCCCGGACCCCAGCCCGGACCCCCACCCAGACCAT
CATCCTGAATAGTGATGAGGAGACGCTGGCCACACTGCACACTGCACTCCAGTCCAGCCACGGGGTCCTG
GGCCCAGAGCGGCTACAGCAGGCACTGGGCCAGGAACACATCATCGTGGCCCAGGAGCAGACAGTGACCA
ATCAGGAAGAAGCCACCTACATCCAAGAGATCACCACCGCGGACGGCCAGACAGTGCAGCATCTGGTGAC
CTCCGACAACCAGGTACAGTACATCATCTCCCAGGATGGAGTCCAACACCTGCTCCCCCAGGAATATGTG
GTGGTTCCAGAGGGCCATCACATCCAGGTACAGGAGGGCCAGATCACACACATCCAGTACGAACAAGGGG
CCCCTTTCCTTCAGGAGTCCCAGATCCAGTATGTGCCTGTGTCCCCAGGCCAGCAGCTGGTCACACAGGC
CCAACTGGAGGCCGCTGCACACTCAGCTGTCACAGCGGTGGCCGATGCTGCCATGGCCCAAGCCCAGGGC
TTGTTTGGCGCAGAAGAGGCGGTGCCTGAACACATCCAACAGCTGCAGCACCAGGGCATCGAGTACGACG
TCATCACCCTGACCGATGAC
>Equus asinus XM_014859900.1
ATGGAGGAGAACGAGGTGGAGAGCA
GTAGCGACGCGGCCCCTGGGCGGCCCGAGGAGCCCTCTGAGAGCGGCTTGGGGGTGGGCACCTCGGAAGC
CGTGTCGGCCGACAGCAGCGACGCGGCGGCCGCCCCTGGGCCGGCGGAGGCCGACGACTCCGGCGTGGGG
CAAAGCTCGGACCGCGGCAGCAGCTCTCTGGAGGAGGTATCCGAGAGCAGCTCAAGCACGGACCCCCTGC
CCCATGGCTACCTCCCTGATTCATCTTCTGTGTCCCGTGGGCCGGTGGCAGGGGTGACAGGCGGCCTCCC
AGACCCCAACATGCTGGTGTCCGACTGCACAGCTTCTTCCTCGGACCTGGGCTCAGCCATCGACAAGATC
ATCGAGTCCACTATTGGGCCTGACCTCATCCAGAGCTGCATCACTGTGACCAGTGCTGAGGATGGTGCAG
CTGAGACCACACGGTACCTGATCCTGCAGGGACCAGACGATGGTGCCCCCATGGCATCACCGATGTCCAG
TTCCACCCTGGCCCACAGCCTGGCAGCCATCGAGGCCCTGGCGGATGGCCCCACATCCACATCCACATGC
CTAGAGCCACCCGAGGAGGCGCGGGGTGGGCCCAGCTCCCCAGCGCAGCTGCCCCCGGGCTCTGGCACGG
AGGAGCCAGACCTGCAGAGCCTAGAGGCCATGATGGAAGTGGTGGTGGTGCAGCAGTTCAAGTGCAAGAT
GTGCCAGTATCGGAGCAGCACGAAGGCCACACTGTTGCGCCACATGCGGGAGCGGCACTTCCGCCCAGCA
GCAGCAGCAGCAGCAGCAGCTGGTAAGAAGGGACGTCTGCGGAAGTGGGGCACCTCGACCAAAACTCAAG
AGGAAGAGGGGCCTGAGGAGGAAGACGATGATGACATCGTAGACGCCGGTGCCATTGATGACCTGGAGGA
GGACAGCGACTACAATCCGGCTGAGGATGAGCCTCGGGGCCGGCAGCTGCGGCCCCAGCGCCCCACTCCC
AGTACGCCGAGACCCCGAAGGAGACCTGGCCGGCCCCGGAAGCTGCCTCGCCTGGAGACCTTGGACCTCC
CAGATGGTGTGGAAGGAGAGCCTCTGGTGAGCTCCCAGAGTGGACAGAGCCCTCCAGAGCCACAGGACCC
TGAGGCGCCCAGCTCCTCAGGCCCAGGATGCCTGGCAGCCCTGGGCAAGGCCGACAGGGCCCCCATGGAA
CCCGGTGTGAGCCAGTCAGACGCAGACCACGCAGCACCCTCCTGCCAGGATGAACCCGACGCTCTGCCCC
GCCGCCGGGGCCGACCCTCCAGGCGCTTCCTAGGCAAGAAATACCGCAAGTATTATTACAAGTCACCCAA
ACCGCTTCTGAGGCCCTTCCTGTGCCGCATCTGCGGCTCGCGCTTCCTGTCCCACGAGGACCTGCGCTTC
CACGTCAACTCCCATGAGGCAGGAGACCCTCAGCTCTTCAAGTGCCTGCAGTGCAGCTATCGCTCCCGCC
GCTGGTCCTCACTCAAGGAGCACATGTTCAACCACATGGGCAGTAAGCCCTATAAGTGTGACGAATGCAG
CTACACCAGTGTCTACCGGAAGGACGTCATCCGGCACGCGGCGGTGCACAGCCGGGACCGGAAGAAGAGG
CCAGATCCGACCCCGAAGCTGAACTCCTTCCCCTGCCCTGTGTGTGGCCGTGTCTACCCCATGCAGAAGA
GACTCACGCAGCACATGAAGACACACAGCACTGAGAAGCCCCACATGTGTGACAAGTGTGGAAAGTCCTT

TAAGAAGCGTTACACCTTCAAGATGCACCTGCTCACGCACATCCAGGCTGTTGCCAACCGCAGGTTCAAG
TGTGAGTTCTGTGAGTTTGTTTGTGAGGACAAGAAGGCGCTTCTGAACCACCAGCTGTCCCATGTCAGCG
ACAAGCCCTTCAAATGCAGCTTCTGCCCCTACCGCACCTTCCGAGAGGACTTCCTGCTGTCCCACGTGGC
CGTCAAGCACACAGGGGCCAAGCCCTTTGCCTGTGAGTACTGCCACTTCAGCACGCGCCACAAGAAGAAC
CTGCGCCTGCACGTCCGGTGCCGGCACGCCAGCAGCTTCGAGGAGTGGGGGCGGCGCCACCCCGAGGAGC
CCCCGTCCCGCCGCCGCCCCTTCTTCTCGCTGCAGCAGATCGAGGAGCTAAAGCAGCAGCACGGTGCGGC
CCCCGGGCCACCCCCTGGTTCCCCGGGACCGCCCGAGATCCCCCCAGAGGCAGCACCTTTTCAGTCACCC
GAGACTCCCCCACTGCTCTGTTCTGACACCCTGGGTGGCGCCACCATCATCTACCAACAAGGAGCTGAAG
AGTCAACCGCAATGGCCACGCAGACAGCCTTGGATCTGCTGCTGAACATGAGTGCTCAGCGGGAGCTGGG
GGGCACAGCCCTACAGGTGGCCGTGGTCAAGTCGGAGGATGTGGAAGCACAGTTGGCATCCCCTGGTGGG
CAGCCGTCCCCAGCAGGTGCTGCTCCACAAGTGGTCACCCTCCATGTGGCAGAGCCGGGGGGCAGTGTGG
CAGCTGAGAGCCAGCTAGGCCCCCCTGACCTACAGCAGATCACCCTGGCACCCGGTCCATTTGGCGGGGC
TGGCTACAGTGTCATCACAGCACCTCCTGTGGAGGAGGGGACATCAGCTCCTGGCACACCTTACAGCGAG
GAGCCCCCCGGGGAGGCAGCCCAGGCAGTGGTTGTGAGCGACACCCTGAAAGAAGCTGGCACCCACTACA
TCATGGCAGCCGATGGGACCCAGCTGCACCACATCGAACTGACCGCAGATGGCTCCATCTCCTTCCCAAC
TCCAGAAGCCCTGGCCTCTGGAGCCAAGTGGCCCCTGCTGCAGTGTGGGGGGCTGCCCAGAGACGGCCCT
GAGCCCCAATCTCCAGCCAGGACCCACCGGGTAGGGGACCCCCAGGGCTCTGCCTCCCCACCTCCTGCAG
CCAGCAAAGCCCTGGGGCTGGTGGTGCCCCCCTCGCCACCTTCGGCAGCCATGGCATCGTCAAAGAAGTT
TTCCTGCAAGATCTGTGCTGAGGCCTTCCCGGGCCGAGCTGAGATGGAGAGTCACAAACGGGCCCACGCC
GGGCCTAGTGCCTTCAAGTGCCCCGACTGCCCCTTCAGTGCTCGCCAGTGGCCTGAAGTCCGGGCCCACA
TGGCCCAGCACTCGAGCCTGCGGCCCCACCAGTGCAGCCAATGCAGCTTCGCCTCCAAGAACAAGAAAGA
CCTGCGGCGGCACATGCTGACCCACACCAACGAGAAGCCTTTTGCGTGCCACCTCTGTGGGCAGCGTTTC
AACCGGAATGGGCACCTCAAGTTCCACATCCAGCGGCTGCACAGTCCTGATGGGAGAAAGGCAGGGACCC
CTACTGCCCGGGCCCCAGCCCGGACCCCCACCCAAACCATCATCCTGAACAGTGATGATGAGACACTGGC
CACACTGCACACTGCACTCCAGTCCAGTCATGGGGTCCTGGGCCCAGAGCGGCTACAGCAGGCACTGGGC
CAGGAACACATCATCGTGGCCCAGGAGCAGACAGTGACCAATCAGGAGGAAGCCACCTACATCCAAGAGA
TCACCACAGCAGATGGCCAAACAGTGCAGCACCTAGTGACCTCTGACAACCAGGTACAGTACATCATCTC
CCAAGATGGCGTCCAGCACCTACTCCCCCAAGAATATGTCGTGGTCCCAGAAGGTCATCACATCCAGGTA
CAGGAGGGCCAGATCACACACATCCAGTACGAACAAGGAGCCCCATTCCTTCAGGAGTCCCAGATCCAGT
ACGTGCCTGTGTCCCCGGGCCAGCAGCTGGTCACACAGGCCCAGCTTGAGGCTGCAGCACACTCAGCTGT
CACAGCGGTGGCTGATGCTGCTATGGCCCAAGCCCAGGGCCTGTTTGGCACAGAGGAGGCAGTGCCTGAA
CACATCCAACAGCTGCAACACCAGGGCATCGAGTATGATGTTATCACCCTGGCTGATGAC
>Equus caballus XM_001503381.4
ATGGAGGAGAACGAGGTGGAGAGCAGTAGCGACGCGGCC
CCTGGGCGGCCCGAGGAGCCCTCTGAGAGCGGCTTGGGGGTGGGCACCTCGGAAGCCGTGTCGGCCGACA
GCAGCGACGCGGCGGCCGCCCCTGGGCCGGCGGAGGCCGACGACTCCGGCGTGGGGCAAAGCTCGGACCG
CGGCAGCAGCTCTCTGGAGGAGGTATCCGAGAGCAGCTCAAGCACGGACCCCCTGCCCCATGGCTACCTC
CCTGATTCATCTTCTGTGTCCCGTGGGCCTGTGGCAGGCGTGACAGGCGGCCTCCCAGACCCCAACATGC
TGGTGTCCGACTGCACAGCTTCTTCCTCGGACCTGGGCTCAGCCATCGACAAGATCATCGAGTCCACTAT
TGGGCCTGACCTCATCCAGAGCTGCATCACTGTGACCAGTGCTGAGGATGGTGCAGCTGAGACCACACGG
TACCTGATCCTGCAGGGACCAGACGATGGTGCCCCCATGGCATCACCGATGTCCAGTTCCACCCTGGCCC
ACAGCCTGGCAGCCATCGAGGCCCTGGCGGATGGCCCCACATCCACATCCACATGCCTGGAGCCACCCGA
GGAGGCGCGGGGTGGGCCCAGCTCCCCAGCGCAGCTGCCCCCGGGCTCTGGCGCGGAGGAGCCAGACCTG
CAGAGCCTAGAGGCCATGATGGAAGTGGTGGTGGTGCAGCAGTTCAAGTGCAAGATGTGCCAGTATCGGA
GCAGCACGAAGGCCACACTGTTGCGCCACATGCGGGAGCGGCACTTCCGCCCAGCAGCAGCAGCAGCAGC
AGCAGCTGGTAAGAAGGGACGTCTGCGGAAGTGGGGCACCTCGACCAAAACTCAAGAGGAAGAGGGGCCT
GAGGAGGAAGACGATGATGACATCGTAGACGCCGGTGCCATTGATGACCTGGAGGAGGACAGCGACTACA

ATCCGGCTGAGGATGAGCCTCGGGGCCGGCAGCTGCGGCCCCAGCGCCCCACTCCCAGTACGCCGAGACC
CCGAAGGAGACCTGGCCGGCCCCGGAAGCTGCCTCGCCTGGAGACCTTGGACCTCCCAGATGGTGTGGAA
GGAGAGCCTCTGGTGAGCTCCCAGAGTGGACAGAGCCCTCCAGAGCCACAGGACCCTGAGGCGCCCAGCT
CCTCAGGCCCAGGATGCCTGGCAGCCCTGGGCAATGCCGACAGGGCCCCCATGGAACCCGGTGTGAGCCA
GTCAGACGCAGACCACGCAGCACCCTCCTGCCAGGATGAACCCGACGCTCTGCCCCGCCGCCGGGGCCGA
CCCTCCAGGCGCTTCCTAGGCAAGAAATACCGCAAGTATTATTACAAGTCACCCAAACCGCTTCTGAGGC
CCTTCCTGTGCCGCATTTGCGGCTCGCGCTTCCTGTCCCACGAGGACCTGCGCTTCCACGTCAACTCCCA
TGAGGCAGGAGACCCTCAGCTCTTCAAGTGCCTGCAGTGCAGCTATCGCTCCCGCCGCTGGTCCTCACTC
AAGGAGCACATGTTCAACCACATGGGCAGTAAGCCCTACAAGTGTGACGAATGCAGCTACACCAGTGTCT
ACCGGAAGGACGTCATCCGGCACGCGGCGGTGCACAGCCGGGACCGGAAGAAGAGGCCAGATCCGACCCC
GAAGCTGAACTCCTTCCCCTGCCCCGTGTGTGGCCGTGTCTACCCCATGCAGAAGAGACTCACGCAGCAC
ATGAAGACACACAGCACTGAGAAGCCCCACATGTGTGACAAGTGTGGAAAGTCCTTTAAGAAGCGTTACA
CCTTCAAGATGCACCTGCTCACGCACATCCAGGCTGTTGCCAACCGCAGGTTCAAGTGTGAGTTCTGTGA
GTTTGTTTGTGAGGACAAGAAGGCGCTTCTGAACCACCAGCTGTCCCATGTCAGCGACAAGCCCTTCAAA
TGCAGCTTCTGCCCCTACCGCACCTTCCGAGAGGACTTCCTGCTGTCCCACGTGGCCGTCAAGCACACAG
GGGCCAAGCCCTTTGCCTGTGAGTACTGCCACTTTAGCACGCGCCACAAGAAGAACCTGCGCCTGCATGT
CCGGTGCCGGCACGCCAGCAGCTTCGAGGAGTGGGGGCGGCGCCACCCCGAGGAGCCCCCGTCCCGCCGC
CGCCCCTTCTTCTCGCTGCAGCAGATCGAGGAGCTAAAGCAGCAGCACGGTGCGGCCCCCGGGCCACCCC
CCGGTTCTCCGGGACCGCCCGAGATCCCCCCAGAGGCAGCACCTTTCCAGTCACCTGAGACTCCCCCACT
GCTCTGTTCTGACACCCTGGGTGGCGCCACCATCATCTACCAACAAGGAGCTGAAGAGTCAACCGCAATG
GCCACGCAGACAGCCTTGGATCTGCTGCTGAACATGAGCGCTCAGCGGGAGCTGGGGGCACAGCCCTAC
AGGTGGCCGTGGTCAAGTCGGAGGATGTGGAAGCACAGTTGGCATCCCCTGGTGGGCAGCCGTCCCCAGC
AGGTGCTGCTCCACAAGTGGTCACCCTCCATGTGGCAGAGCCAGGGGGCAGTGTGGCAGCTGAGAGCCAG
CTAGGCCCCCCTGACCTACAGCAGATCACCCTGGCACCCGGTCCATTTGGCGGGGCTGGCTACAGTGTCA
TCACAGCACCTCCTGTGGAGGAGGGGACATCAGCTCCTGGCACACCTTACAGCGAGGAGCCCCCCGGGGA
GGCAGCCCAGGCAGTGGTTGTGAGCGACACCCTGAAAGAAGCTGGCACCCACTACATCATGGCAGCCGAT
GGGACCCAGCTGCACCACATCGAACTGACTGCAGATGGCTCCATCTCCTTCCCAACTCCAGAAGCCCTGG
CCTCTGGAGCCAAGTGGCCCCTGCTGCAGTGTGGGGGGCTGCCCAGAGACGGCCCTGAGCCCCAATCTCC
AGCCAGGACCCACCGGGTAGGGGACCCCCAGGGCTCTGCCTCCCCACCTCCTGCAGCCAGCAAACCCCTG
GGGCTGGTGGTGCCCCCCTCGCCACCTTCGGCAGCCATGGCATCGTCAAAGAAGTTTTCCTGCAAGATCT
GTGCTGAGGCCTTCCCGGGCCGAGCTGAGATGGAGAGTCACAAAAGGGCCCACGCCGGGCCTAGTGCCTT
CAAGTGCCCCGACTGCCCCTTCAGTGCTCGCCAGTGGCCTGAAGTCCGGGCCCACATGGCCCAGCACTCG
AGCCTGCGGCCCCACCAGTGCAGCCAATGCAGCTTCGCCTCCAAGAACAAGAAAGACCTGCGGCGGCACA
TGCTGACCCATACCAACGAGAAGCCTTTTGCGTGCCACCTCTGTGGGCAGCGTTTCAACCGGAATGGGCA
CCTCAAGTTCCACATCCAGCGGCTGCACAGTCCTGATGGGAGAAAGGCAGGGACCCCTACTGCCCGGGCC
CCAGCCCGGACCCCCACCCAAACCATCATCCTGAACAGTGATGATGAGACACTGGCCACACTGCACACTG
CACTCCAGTCCAGTCATGGGGTCCTGGGCCCAGAGCGGCTACAGCAGGCACTGGGCCAGGAACACATCAT
CGTGGCCCAGGAGCAGACAGTGACCAATCAGGAGGAAGCCACCTATATCCAAGAGATCACCACAGCAGAT
GGCCAAACAGTGCAGCACCTAGTGACCTCTGACAACCAGGTACAGTACATCATTTCCCAAGATGGCGTCC
AGCACCTACTCCCCCAAGAATATGTCGTGGTCCCAGAAGGTCATCACATCCAGGTACAAGAGGGCCAGAT
CACACACATCCAGTACGAACAAGGAGCCCCATTCCTTCAGGAGTCCCAGATCCAGTACGTGCCTGTGTCC
CCGGGCCAGCAGCTGGTCACACAGGCCCAGCTTGAGGCTGCAGCACACTCAGCTGTCACAGCGGTGGCTG
ATGCTGCTATGGCCCAAGCCCAGGGCCTGTTTGGCACAGAGGAGGCAGTGCCTGAACACATCCAACAGCT
GCAACACCAGGGCATCGAGTATGATGTTATCACCCTGGCTGATGAC
>Orcinus orca XM_012534185.1
ATGGAGGAGAACGAGGTGGAGAGCAGTAGCGACACGGCCCCTGGTCCTGGCCGGCCCGAGGAGCCCTCTG
AGAGCGGCTTGGGTGTGGGCACCTCGGAAGCCGTGTCGGCCGACAGCAGCGACGCCGCGGCCGCTCCGGG

GCCAGCCGAGACCGACGACTCTGGCGTGGGGCAGAGTTCGGACCGCGGCACCAGCTCTCTGGAGGAGGTA
TCCGAGAGCAGCTCCAGCACAGAACCCCTGCCCCATGGCTACCTCCCTGATTCATCTTCTGTTTCCCACG
GGCCAGTGGCAGGGGTGACAGGCGGCCCCCCAGCCCTGGTGCACTCCAGCGCTCTCCCAGACCCCAACAT
GCTGGTGTCCGACTGCACGGCTTCTTCCTCAGACCTCGGCTCAGCCATTGACAAGATCATCGAGTCCACC
ATCGGGCCCGACCTCATCCAGAGCTGCATCACCGTGACCAGTGCTGAGGGCGGTGGGTCCGAGACCACAC
GGTACCTGATCCTGCAGGGACCAGACGATGGGGCCCCCATGGCATCACCGATGTCCAGTTCCACCCTGGC
CCACAGCCTGGCAGCCATCGAAGCTCTAGCCGATGGCCCCACGTCCACATCCACGTGCCTGGAGCCCCCT
GAGGAGGTACAGGGTGGACCCAGCTCCCCAGCGCAGCCATGCCTGGGTTCTGGCGCCGAGGAGCCAGACC
TGCAGAGCCTGGAGGCCATGATGGAGGTGGTGGTGGTGCAGCAGTTCAAGTGCAAGATGTGCCAGTACCG
GAGCAGCGCCAAGGCCACACTGTTGCGCCACATGCGGGAGCGGCACTTCTGCCCAGCCGCCGCCACCGGA
AAAAAGGGACGTCTGCGGAAGTGGGGTGCCTCTGCCAGGACCCAGGAGGAAGAGGGCCCAGAGGAGGAGG
ACGATGATGACATCATAGACGCCGGCGCCATCGATGACCTGGAAGAGGACAGCGACTATAACCCTGCTGA
GGATGAGCCCCGGGGCCGGCAGCTACGGCCCCAGCGCCCCACTCCCAGTACACCGAGACCCCGCAGGAGA
CCTGGCCGGCCTCGGAAGCTGCCTCGCCTGGAGACCTCGGATCTCCTGGATGGTGTGGAAGGAGAGCCTC
TGGTCAGCTCCCAGACTGGACAGAGCCCTCTGGAGCCAGAAGACCCCGAGGCATCCAGCTCCTCAGGCCC
GGGACGCCTGGTGGCCCTGGGCAAGGCCAACCGGCCCCCTGTGGAACCCGGCATGAGCCAGTCAGATGTG
GAGAACGCAGCACCGTCCTGCCAGGACGAGCCCGACGCCCCGCCCCGCCGCCGTGGGCGACCCTCCAGGC
GCTTCCTCGGCAAGAAATACCGCAAGTACTATTACAAGTCGCCCAAACCCCTCCTGCGGCCCTTCTTGTG
CCGCATCTGCGGTTCCCGCTTCCTGTCCCACGAGGACCTGCGCTTCCACGTCAACTCCCACGAGGCTGGC
AACCCCCAGCTCTTCAAGTGCCTGCAGTGCAGCTACCGCTCCCGCCGCTGGTCCTCGCTCAAGGAGCACA
TGTTCAACCACGTGGGCAGTAAGCCCTACAAGTGTGACGAATGCAGCTACACCAGTGTCTACCGGAAGGA
CGTCATCCGGCACGCGGCCGTGCATAGCCGGGACCGGAAGAAGAGGCCAGACCCGACCCCGAAGCTGAGC
TCCTTCCCCTGCCCTGTGTGTGGCCGTGTCTACCCCATGCAGAAGAGACTCACGCAGCACATGAAGACTC
ACAGCACCGAGAAGCCCCACATGTGTGACAAGTGTGGAAAGTCCTTTAAGAAGCGCTACACCTTCAAGAT
GCACCTGCTCACGCATATCCAGGCCGTCGCCAACCGCAGGTTCAAGTGCGAGTTCTGCGAGTTCGTTTGT
GAGGACAAGAAGGCGCTGCTGAACCACCAGCTTTCCCATGTCAGCGACAAGCCCTTCAAGTGCAGCTTTT
GCCCCTACCGCACCTTCCGAGAGGACTTCCTGCTGTCCCACGTGGCCGTCAAGCACACAGGGGCCAAGCC
CTTTGCCTGTGAGTACTGCCACTTCAGCACGCGACACAAGAAGAACCTCCGCTTGCACGTACGGTGCCGA
CATGCAAGCAGCTTTGAGGAGTGGGGGCGGCGCCACCCCGAGGAGCCCCCTTCCCGCCGTCGCCCCTTCT
TCTCTCTGCAACAGATTGAGGAGCTGAAGCAGCAGCATAGCGCAGCCCCTGGACCTCCGCCCAGCTCCCC
AGGACCTCCTGAGATCCCCCCAGAGGCAGCACCTCTCCAGTCACCCGAGACACCCCCGCTGCTGTGTTCT
GACACCCTGGGTGGTGCTACCATCATCTACCAGCAAGGAGCTGAGGAGTCGACGGCCATGGCCACACAGA
CAGCCTTGGACCTGTTGCTGAACATGAGCGCTCGGCGGGAGCTGGGCAGCGCGGCCCTGCAGGTGGCCGT
GGTAAAGTCAGAGGACGTGGAAGCAGGGTTCGCATCCTCTGGTGGGCAGCCCTCCCCAGCAGGTGCCACT
CCCCAAGTGGTAGCCCTCCACATGGCAGAGCCAGGGGGCAGCGTGGCCGCCGAGAGCCAGCTAGGCACCC
CGGACCTACAGCAGATCACCCTGGCGCCTGGGCCATTTGGCGGGGCTGGCTACAGCGTCATCACGGCACC
CACCATGGAGGAGGGCACATCGGCTCCTGGCACACCTTACAGCGAGGAGCCCCCTGGGGAGGCAGCCCAG
GCCGCGGTTGTGAGTGACACCTTGAAAGAAGCTGGTACCCACTTCATCATGGCCGCCGACGGACCCAGC
TGCACCACATCGAGCTGACTGCACATGGCCCCATCTCCTTCCCAAGTCCAGACGCCCTGGCCTCTGGAGC
CAAGTGGCCCTTGCTGCAGTGTGGGGGGCTGCCCAGAGACGGCCCTGAGCCCCCGTCTCCAGCCAGGACC
CGCCGAGTGGGGGACCCCCAGGGCTCTGCCTCCCCACCTCCTGCTGCCAGCAAAGCCCTGGGCCTGGTAG
TGCCCCCCTCGCCACCATCTGCAGCCACTGCGTCGTCAAAGAAGTTTTCCTGCAAGATCTGTGCTGAGGC
CTTCCCCGGCCGAGCTGAGATGGAAAGTCACAAACGGGCCCACGCTGGGCCTAGTGCCTTCAAGTGCCCC
GACTGCCCCTTCAGCGCTCGCCAGTGGCCCGAGGTCCGGGCCCACATGGCACAGCACTCAAGCCTGCGGC
CCCACCAGTGCAACCAGTGCAGCTTCGCTTCCAAGAACAAGAAGGATCTGCGGCGCCACATGCTGACCCA
CACCAACGAGAAGCCCTTCGAGTGCCACCTCTGCGGGCAGCGCTTCAACCGGAACGGGCACCTCAAGTTC
CACATCCAGCGGCTGCACAGTCCCGATGGGAGGAAGGCGGCAACCCCGACCACGCGGGCCCCGGCCCAGA

CCCCCAAGACCCCCACCCAGACCATCATCCTGAACAGTGACGACGAGACGCTGGCCACGCTGCACACTGC
ACTCCAGTCCAGTCACGGGGTCCTGGGCCCAGAGCGGCTACAGCAGGCACTGGGCCAGGAACACATCATT
GTGGCCCAGGAACAGACAGTGACCGATCAGGAGGAAGCCACCTACATCCAAGAAATCACTACAGCAGATG
GCCAGATGGTCCAGCACCTGGTGACATCTGATAACCAGGTACAGTACATCATCTCCCAGGATGGAGTCCA
GCACCTGCTCCCCCAGGAATATGTCGTGGTCCCGGAGGGCCATCACATCCAGGTACAGGAGGGCCAGATC
ACACACATCCAGTATGAACAAGGGGCGCCGTTCCTTCAGGAGTCCCAGATCCAGTATGTGCCGGTGTCCC
CAGGCCAGCAGCTTGTCACACAGGCCCAGCTTGAGGCTGCAGCACACTCAGCTGTCACAGCGGTGGCCGA
TGCTGCCATGGCCCAAGCCCAAGGCCTATTCGGCACGGAGGAGGCAGTGCCTGAACACATCCAACAGCTG
CAACATCAGGGCATCGAGTACGACGTCATCACCCTGACCGATGAC
>Lipotes vexillifer XM_007451630.1
ATGGAGGAGAACGAGGTGGAGAGCAGTAGCGACACGGCCCCTGGTCCTGGCCGGCCCGAGGAGCCCTCTG
AGAGCGGCTTGGGTGTGGGCACCTCGGAAGCCGTGTCGGCCGACAGCAGCGACGCCGCGGCCGCTCCGGG
GCCAGCCGATGCCGACGACTCTGGCGTGGGGCAGAGTTCGGACCGTGGCACCAGCTCTCTGGAGGAGGTA
TCCGAGAGCAGCTCCAGCACAGACCCCCTGCCCCATGGCTACCTCCCTGATTCATCTTCTGTTTCCCACG
GGCCAGTGGCAGGGGTGACAGGCGGCCCCCCAGCCCTGGTGCACTCCAGCGCACTCCCAGACCCCAACAT
GCTGGTATCCGACTGCACGGCTTCTTCCTCAGACCTGGGCTCAGCCATTGACAAGATCATCGAGTCCACC
ATCGGGCCCGACCTCATCCAGAGCTGCATCACCGTGACCAGTGCTGAGGGGAGTGGGTCCGAGACCACAC
GGTACCTGATCCTGCAGGGACCAGACGATGGGGCCCCCATGGCATCACCTATGTCCAGTTCCACCCTGGC
CCACAGCCTGGCAGCCATCGAATCTCTAGCCGATGGCCCCACGTCCACATCCACGTGCCTGGAGCCACCT
GAGGAGGTACAGGGTGGATCCAGCTCCCCAGCGCAGCCATGCCTGGGTTCTGGTGCCGAGGAGCCAGACC
TGCAGAGCCTGGAGGCCATGATGGAGGTGGTGGTGGTGCAGCAGTTCAAGTGCAAGATGTGCCAGTACCG
GAGCAGCGCCAAGGCCACGCTGTTGCGCCACATGCGAGAGCGGCACTTCTGCCCAGGTGCCAAGCTTGTT
GTACCGCTAGCAGCCGCCGCCACCGGAAAAAAGGGACGTCTGCGGAAGTGGGGTGCCTCCACCAGGACCC
AGGAGGAAGAGGGCCCAGAGGAGGAAGACGATGATGACATCATAGACGCCGGTGCCATCGATGACCTGGA
AGAGGACAGCGACTATAACCCTGCTGAGGATGAGCCCCGGGGCCGGCAGCTACGGCCCCAGCGCCCCACT
CCCAGTACACCGAGACCCCGCAGGAGACCTGGCCGGCCCCGGAAGCTGCCTCGCCTGGAGACCTCGGATC
TCCTGGATGGTGTGGAAGGAGAGCCTCTGGTCAGCTCCCAGACTGGGCAGAGCCCTCTGGAGCCACAAGA
CTCCGAGGCACCCAGCTCCTCAGGCCCGGGATGCCTGGTGGCCCTGGGCAAGGCCAACCGGCCCCCTGTG
GAACCCGGCGTGAGCCAGTCAGAAGTGGAGAAGGCAGCACCGTCCTGCCAGGACGAGCCCGACGCCCCGC
CCCGCCGCCGTGGGCGACCCTCCAGGCGCTTCCTCGGCAAGAAATACCGCAAGTACTATTACAAGTCGCC
CAAACCCCTCCTGCGGCCCTTCTTGTGCCGCATCTGCGGTTCCCGCTTCCTGTCCCATGAGGACCTGCGC
TTCCACGTCAACTCCCACGAGGCTGGCAACCCTCAGCTCTTCAAGTGCCTGCAGTGCAGCTACCGCTCCC
GCCGCTGGTCTTCGCTCAAGGAGCACATGTTCAACCACGTGGGCAGTAAGCCCTACAAGTGTGACGAATG
CAGCTACACCAGTGTTTACCGGAAGGACGTCATCCGGCACGCGGCCGTGCATAGCCGGGACCGGAAGAAG
AGGCCAGACCCGACCCCGAAGCTGAGCTCCTTCCCCTGCCCTGTGTGTGGCCGTGTCTACCCCATGCAGA
AGAGACTCACGCAGCACATGAAGACTCACAGCACCGAGAAGCCCCACATGTGTGACAAGTGTGGAAAGTC
CTTTAAGAAGCGCTACACCTTCAAGATGCACCTGCTCACGCATATCCAGGCCGTCGCCAACCGCAGGTTC
AAGTGCGAGTTCTGCGAGTTCGTTTGTGAGGACAAGAAGGCGCGGCTGAACCACCAGCTTTCCCATGTCA
GCGACAAGCCCTTCAAGTGCAGCTTTTGCCCCTACCGCACCTTTCGAGAGGACTTCCTGCTGTCCCACGT
GGCCGTCAAGCACACAGGGGCCAAGCCCTTTGCCTGTGAGTACTGCCACTTCAGCACGCGACACAAGAAG
AACCTCCGCTTGCACGTACGGTGCCGACACGCAAGCAGCTTTGAGGAGTGGGGGCGGCGCCACCCCGAGG
AGCCCCCTTCCCGCCGTCGCCCCTTCTTCTCTCTACAACAGATTGAGGAGCTGAAGCAGCAGCATAGCGC
AGCCCCTGGACCTCCGCCCAGCTCCCCAGGACCTCCTGAGATCCCCCCAGAGGCAGCACCTCTCCAGTCA
CCCGAGACACCCCCGCTGCTCTGTTCTGACACCCTGGGTGGTGCCACCATCATCTACCAGCAAGGAGCTG
AGGAGTCGACGGCCATGGCCACACAGACAGCCTTGGACCTGTTGCTGAACATGAGCACTCGGCGGGAGCT
GGGCAGCGCGGCCCTGCAGGTAGCCGTGGTGAAGTCAGAGGACGTGGAAGCAGGCTTCGCATCCTCTGGT
GGGCAGCCCTCCCCAGCAGGTGCCACTTCCCAAGTGGTAGCCCTCCACATGGCAGAGCCAGGGGGCAGCG

TGGCCGCCGAGAGCCAGCTAGGCGCCCCGGACCTACAGCAGATCACCCTGGCGCCTGGGCCATTCGGCGG
GGCTGGCTACAGCGTCATCACGGCACCCACCATGGAGGAGGGCACATCGGCTCCTGGCACACCTTACAGC
GAGGAGCCCCCCGGGGAGGCAGCCCAGGCCGTGGTTGTGAGTGACACCTTGAAAGAAGCTGGCACCCACT
TCATCATGGCCGCCGACGGGACCCAGCTGCACCACATCGAGCTGACTGCAGATGGCTCCATCTCCTTCCC
AAGTCCAGACGCCCTGGCCTCTGGAGCCAAGTGGCCCTTGCTGCAGTGTGGGGGGCTGCCCAGAGACGGC
CCTGAGCCCCCGTCTCCAGCCAGGACTCGCCGAGCAGGGGACCCCCAGGGCTCTGCCTCCCCACCTCCTG
CTGCCAGCAAAGCCCTGGGCCTGGTAGTGCCCCCCTCGCCACCATCTGCAGCCACTGCGTCGTCAAAGAA
GTTTTCCTGCAAGATCTGCGCCGAGGCCTTCCCTGGCCGAGCTGAGATGGAAAGTCACAAACGGGCCCAC
GCTGGGCCTAGTGCCTTCAAGTGCCCCGACTGCCCCTTCAGTGCTCGCCAGTGGCCCGAGGTCCGGGCCC
ACATGGCGCAGCACTCAAGCCTGCGGCCCCACCAGTGCAACCAGTGCAGCTTCGCTTCCAAGAACAAGAA
GGATCTGCGGCGCCACATGCTGACCCACACCAATGAGAAGCCCTTCGAGTGCCACCTCTGCGGGCAGCGC
TTCAACCGGAACGGGCACCTCAAGTTCCACATCCAGCGGCTGCACAGTCCCGACGGGAGGAAGGCGGCGA
CCCCGACCACGCGGGCCCCGGCCCAGACCCCCAAGACCCCCACCCAGACTATCATCCTGAACAGTGACGA
CGAGACGCTGGCCACGCTGCACACTGCACTCCAGTCCAGTCACGGGGTCCTGGGCCCAGAGCGGCTACAG
CAGGCACTGGGCCAGGAACACATCATTGTGGCCCAGGAACAGACAGTGACCAATCAGGAGGAAGCCACCT
ACATCCAAGAGATCACTACAGCAGATGGCCAGATGGTCCAGCACCTGGTGACATCTGATAACCAGGTACA
GTACATCATCTCCCAGGACGGAGTCCAGCACCTGCTCCCCCAGGAATACGTCGTGGTCCCGGAGGGCCAT
CACATCCAGGTCCAGGAGGGCCAGATCACACACATCCAGTATGAACAAGGGGCGCCGTTCCTTCAGGAGT
CCCAGATCCAGTATGTGCCGGTGTCCCCAGGCCAGCAGCTTGTCACACAGGCCCAGCTTGAGGCTGCAGC
ACACTCAGCTGTCACAGCGGTGGCCGATGCTGCCATGGCCCAAGCCCAAGGCCTGTTCGGCACGGAGGAG
GCAGTGCCTGAACACATCCAACAGCTGCAACATCAGGGCATCGAGTACGACGTCATCACCCTGACCGATG
AC
>Ovis aries musimon XM_012137159.2
ATGGAGGAAAACGAGGTGGAGAGCAGTAGCGACGCGGCGCCTAGGCCTGGCCGGCCAGA
GGAGCCCTCGGAGAGCGGGTTGGGTGTGGGCACCTCGGAAGCCGTGTCGGCCGACAGCAGCGACGCCGCG
GCCGCCCCGGGGCCAGCGGAGGCCGACGATTCCGGCGTGGGGCAGAGCTCGGACCGCGGCAGCAGCTCGC
TGGAGGAGGTATCTGAGAGCAGCTCCAGCACAGACCCCCTACCCCATGGCTACCTCCCTGACTCATCTTC
TGTTTCCCGCGGGCCAGTGGCAGGGGTGACGGGCGGCCCCCCAGCCCTGGTGCACTCCAGTGCACTGCCA
GACCCCAACATGCTGGTGTCCGACTGTACGGCTTCTTCCTCGGACCTGGGCTCGGCCATTGACAAGATCA
TCGAGTCCACCATCGGGCCCGACCTCATCCGGAGCTGCATCACTGTGACCAGTGCGGAGGGCAGCGGGGC
CGAGGCCACACGGTACCTGATCCTGCAGGGACCAGACGATGGCGCCCCCATGGCATCACCGATGTCCAGT
TCCACCCTGGCCCATAGCCTGGCAGCCATCGAGGCCCTGGCCGATGGCCCCACATCCACGTCCACGTGCC
TAGAGCCACCAGAGGATGCGCAGGGTGGGCCCAGCTCCCCGGCGCAGCCCCCCCTGGGTTCTGGCGCCGA
GGAGCCGGACTTGCAGAGCCTGGAGGCCATGATGGAGGTGGTGGTGGTGCAGCAGTTCAAGTGCAAGATG
TGCCAGTACCGGAGCAGCACCAAGGCCACGCTGCTGCGCCACATGCGGGAGCGGCACTTCCGCCCGGCAG
CAGCAGCTGGTAAGAAGGGGCGTCCGCGCAAGTCGGGCAGCTCGGCCCAGCCCCAGGAGGAAGAGGGCCC
AGAGGAGGAAGACGACGACGACATCATAGATGCTGGTGCCATCGATGACCTGGAGGAAGACAGTGACTAT
AACCCTGCAGATGACGAGCCCCGGGGCCGGCAGCTGCGGCCCCAGCGCCCCACCCCCAGTACCCTGAGAC
CCCGCAGGAGACCTGGCCGGCCCCGGAAGCTGCCTCACCTGGAGACCTTGGATCTCCCAGATGGTGTGGA
AGGAGAGCCTTTAGTGAGTTCACAAAGTGGACAGAGCCCTCCGGAGCCACAAGACCCCGAGGCACCCAGC
TCCTCAGGGCGGGGACGCCTGGTGGCCCTGGGCAAGGCCAACCGGGCCCACGTGGAACCCAGTGTGAGCC
AGTCAGATGCAAGGAACGCAGCTTCGTCCTTCCAGGATGAGGCTGACGCCCTGCCCCGCCGTCGTGGTCG
GCCCTCCAGGCGCTTCCTTGGCAAGAAATACCGCAAGTACTATTACAAGTCACCCAAACCGCTCCTGCGG
CCTTTCCTGTGCCGCATCTGCGGCTCCCGCTTCCTGTCCCACGAGGATCTGCGCTTCCACGTTAACTCCC
ATGAGGCCGGCAACCCCCAGCTCTTCAAGTGCCTGCAGTGCAGCTACCGCTCCCGTCGCTGGTCCTCACT
CAAGGAGCACATGTTCAACCACGTGGGCAGTAAGCCCTACAAGTGTGACAAATGCAGCTACACCAGCGTC
TATCGGAAGGACGTCATCCGGCACGCGGCTGTGCACAGCCGGGATCGGAAAAAGAGGCCAGACCCGACCC

CAAAGCTGAGCTCCTTCCCCTGCCCCGTGTGTGGCCGTGTCTACCCCATGCAAAAGAGACTCACTCAGCA
CATGAAGACACACAGCACCGAGAAGCCCCACATGTGCGACAAGTGTGGAAAGTCCTTTAAGAAGCGCTAC
ACCTTCAAGATGCACCTGCTCACGCACATCCAGGCTGTCGCCAACCGCAGGTTCAAATGCGAGTTCTGCG
AGTTTGTTTGCGAGGACAAGAAGGCGCTGCTCAACCACCAGCTGTCCCACGTCAGTGACAAGCCCTTCAA
GTGTAGCTTTTGCCCCTATCGCACCTTCCGAGAGGACTTCCTGCTATCCCACGTGGCTGTCAAGCATACA
GGGGCCAAGCCCTTTGCCTGCGAGTACTGCCACTTCAGCACGCGGCACAAGAAGAACCTGCGGCTGCATG
TGCGCTGCCGGCACGCCAGCAGCTTCGAGGAGTGGGGGCGGCGCCACCCCGAGGAGCCCCCCTCCCGCCG
CCGCCCCTTCTTCTCCCTGCAGCAGATCGAGGAGCTGAAGCAGCAGCACAGCACGGCCCCTGGGCCCCCC
GCCGGCTCCCCAGGACCTCCCGAGATCCCCTCAGAGGCTGCACCTTTCCAGTCCCCTGAGACCCCCCCAC
TGCTCTGTTCCGACACCCTGGGTGGTGCCACCATCATTTATCAGCCAGGGACTGCGGAGTCGACAGCTGT
GGCCACGCAGACAGCCTTGGACCTGCTGCTGAACATGAGCACTCAGCGGGAGCTGAGTGGCACAGCCCTG
CAGGTGGCCGTGGTGAAGTCAGAGGACGTGGACACAGGGTTAGCGTCCTCTGGGGGGCAGCCCTCCCCAG
CAGGCGCCACTCCCCAGGTGGTCACCCTCCACGTGGCAGACCCAGGAGGCAGCGTGGCCGCTGAGAGCCA
GCTTGGGCCCCCTGACCTGCAGCAGATCACCCTGGCACCTGGGCCGTTCAGCGGGGCTGGCTATAGTGTC
ATTTCGGCTCCCACCGTGGAGGAGGGCACTTCGACTCCCGGCACGCCTTACAGCGAGGAGCCCCCGGGGG
AGGCAGCCCAGACCGTGGTCGTGGGTGACACCCTGAAAGAAGCTGGCACGCACTTCATCATGGCAGCCGA
CGGGACCCAGCTGCACCACATCCAGCTGACTGCAGACGGCTCCATCTCCTTCCCAAGTACAGAAGCCTTG
GCCTCCGGTGCCAAGTGGCCCCTCCTGCAGTATGGGGGGCTGCCCAGAGATGGTCCTGAGCCCCCGGCTC
CAGCCAGGACCCACCAGTCAAGGGACCCTCAGGGCTCTGCCTCCCCACCTCCTGCAGCCAGCAAACCTTT
GGGCCTGGTAGTGCCCCCCTCGCCGCCATCTGCAGCTACTGCCTCATCAAAGAAGTTTTCCTGCAAGATC
TGTGCCGAGGCCTTCACCGGCCGAGCAGAGATGGAGAGTCACAAACGGGCCCACGCCGGGCCGGGAGCCT
TCAAGTGCCCTGACTGCCCCTTCAGTGCTCGCCAGTGGCCCGAGGTCCGGGCCCACATGGCACAGCACTC
GAGCCTGCGGCCCCACCAGTGCAGCCAGTGCAGCTTCGCCTCCAAGAACAAGAAGGACCTGCGGCGGCAC
ATGCTGACCCACACCAACGAGAAGCCCTTCGAGTGCCAGCTCTGCGGGCAGCGCTTCAACCGCAACGGGC
ACCTCAAGTTCCACATCCAGCGGCTCCACAGCCCTGACGGGAGGAAGGCGGCAGCGCCCACCGCACGTCC
TCCAGCCGGGCCCCCCACCCAGACCATCATCCTCAACAGTGATGACGAGACGCTGGCCACACTGCACACT
GCCTTCCAGTCCAGTCACGGGGTCCTGGGTCCGGAGCGGCTCCAACAGGCACTGGGCCAAGAACACATCT
TCGTGGCCCAGGAACAGACAGTGAGCAATCCGGAGGAAGCCGCCTATATCCAAGAGATCACCACGGCGGA
CGGCCAGATGGTCCAGCACCTGGTGGCGTCTGATAGCCAGGTACAGTACATCATTTCCCCGGATGGAGTG
CAGCAGCTCCTTCCCCAGGAATATGTGGTGGTGCCCGAGGGCCATCACATCCAGGTACAGGAGGGCCAGA
TCACACACATCCAGTATGAGCAAGGGGCCCCCTTCCTTCAGGAGTCCCAGATCCAGTACGTGCCTGTGTC
CCCGGGCCAGCAGTTGGTCACCCAGGCCCAGCTTGAGGCTGCGGCACACTCAGCTGTCACAGCGGTGGCC
GACGCTGCCATGGCCCAAGCCCAGGGCCTCTTTGGCACAGAGGAGGCTGTGCCTGAACACATTCAACAGC
TGCAGCACCAGGGCATCGAATACGACGTCATCACCCTGAGCGATGAC
>Manis javanica XM_017653428.1
ATGGAGGAGAACGAGGTGGAGA
GCAGTAGCGACGCAGCCCCAGGGCCTGGGCGGCCCGAGGAACCTTCTGAGAGCGGCTTGGGTGTGGGCAG
CTCGGAAGCCGTGTCTGCCGACAGCAGCGACGCCGCAGCCACCCCGGCGCCGGCGGAGGCTGATGACTCC
GGCGTGGGGCAAAGCTCGGACCGTGGCAGCAGCTCTCTGGAGGAGGTATCTGAGAGCAGCTCGAGCACAG
ACACCCTGCCCCATGGCTACCTCCCTGATTCATCATCTGTGTCCCATGGGCCAGTGGCAGGGGTGACAGG
CGGCCCCCCAGCTCTGGTGCACTCCAGTGAACTCCCAGACCCCAACATGCTGGTGTCTGACTGCACAGCT
TCTTCCTCGGACCTGGGCTCGGCCATTGACAAGATCATTGAGTCCACCATCGGGCCGGACCTCATCCAGA
GCTGCATCACCGTAACCAGTGCTGAGGATGGTGGGGCCGGGACCACACGGTACCTGATCCTGCAGGGACC
AGATGATGGTGCCCCCATGACATCACCAATGTCCAGTTCCACCCTGGCTCACAGCCTGGGAGCCATTGAG
GCCCTGGCCGATGGCCCCACATCCACATCCATGTGCCTGGAGCCGCCTGAGGAGGCACTGGGTGGGTCCA
GCTCGCCAGCGCACCCACCCCCAGGCTCTGGGGCTGAGGAGCCAGACCTGCAGAGCCTGGAGGCCATGAT
GGAGGTGGTGGTGGTGCAGCAATTCAAGTGCAAGATGTGCCAGTACCGGAGCAGCACCAAGGCCACACTG

TTGCGCCACATGCGGGAGCGGCACTTCCGACCAGCAGCAGCAAGAGCAGCAGCTGGTAAGAAGGACGTC
TGAGAAAGTGGGGCGTCTCCACCAAGACCCAGGAGGAAGAGGGGCCAGAGGAGGAGGAAGATGATGACAT
CGTAGATGCAGGCGCCATTGACGACCTGGAGGAGGACAGTGACTACAATCCAGCTGAGGATGAGCCTCGG
GGTCGGCAGCTACGACCCCAGCGCCCGGCTCCCAGTACACTGAGACCCCGAAGGAGACCTGGCCGGCCTC
GGAAGCTGCCTCGCCTGGAGGCCTCAGATCTCCCAGATGGTGTGGAAGGAGAGCCTCTAGTGAGTTCTCA
GAGTGGACAGAGCCCTCCAGAGCCACAGGACCCCGAGGCGCCTAGTTCCTCAGGCCCAGGACACCTGGTT
GCCCTGAGCAAGGCCAGTAGGGCCCCTGTGGAACCTGGTGTGAGCCAGTCCGATGCAGAGAATGCAGCAC
CCTCCTGCCAGGATGAGCCTGATGCCCCTCCCCGTCGCCGTGGGCGACCTTCTAGGCGCTTCCTAGGCAA
GAAATACCGCAAGTACTATTACAAGTCACCCAAACCGCTTCTGAGGCCCTTCCTGTGCCGCATCTGCGGC
TCCCGCTTCCTGTCCCATGAGGACCTGCGCTTCCATGTCAACTCCCATGAGGCCGGAGACCCCCAGCTCT
TCAAGTGCCTGCAGTGCAGCTACCGCTCCCGCCGCTGGTCCTCACTCAAGGAGCACATGTTCAACCATGT
GGGCAGTAAGCCCTACAAGTGTGATGAATGCAGCTACACCAGTGTCTACCGGAAGGATGTCATTCGGCAC
GCAGCTGTGCACAGCCGGGACCGGAAGAAAAGACCAGATCCGACCCCGAAGCTGAGCTCCTTCCCCTGCC
CTGTGTGTGGCCGTGTCTACCCCATGCAGAAGAGACTCACACAGCACATGAAGACACATAGCACTGAGAA
ACCCCACATGTGTGATAAGTGTGGAAAGTCCTTTAAGAAGCGCTACACCTTCAAGATGCACCTACTCACA
CACATCCAGGCTGTCGCCAACCGCAGGTTCAAGTGCGAATTCTGTGAGTTTGTTTGTGAGGACAAGAAGG
CCCTGCTGAACCACCAGCTGTCCCATGTCAGTGATAAGCCATTCAAATGCAGCTTTTGCCCCTATCGCAC
TTTCCGAGAGGACTTCCTGTTGTCCCATGTGGCCGTCAAGCACACAGGGGCCAAGCCCTTTGCCTGTGAG
TTCTGCCACTTCAGCACACGGCACAAGAAGAACCTGCGCCTGCATGTGCGATGCCGACATGCAAGCAACT
TCGAGGAATGGGGGCGGCGCCACCCCGAGGAACCCCCTTCCCGCCGTCGCCCCTTCTTCTCTCTGCAGCA
GATTGAGGAGCTGAAGCAGCAGCATAGTGCAGCCCCAGCCCTTGGACCACCCTCTAGCTCCCCCGGAGCT
CCTGAGGTCCCCCCTGAGACAGCCCCTTTCCAGTCACCCGAGGCACCCCCACTGCTGTGTTCTGACACCC
TGAGTGGTGCCACCATCATCTACCAGCAAGGAGCTGAGGAGTCAACTGCAATGGCCACGCAGACAGCCTT
GGATCTGCTGCTGAATATGAGCGCTCAGCGGGAGCTGGGGGGCCCGGCCCTGCAGGTGGCCGTGGTGAAG
TCAGAGGATGTGGGGTCAGAGTTAGCATCCACTGCTGGGCAGCCCTCCCCAGCAGGTGCTTCTCCACAAG
TGGTGACCCTCCACGTGGCAGAGCCTGGGGGCAGTGTGACAGCTGAGAGCCAACTAGGCCCCCCTGACCT
GCAGCAGATCACCCTGGCATCCGGTCCATTTGGTGGGACTGGCTACAGCGTCATCACAGCCCCTCCTATG
GAGGAGGGGACATCAGCTCCTGGCACACCTTACAGCGAGGAGCCCCCAGGGGAGGCAGCCCAGGCTGTGG
TTGTGAGCGACGCCCTTAAAGAAGCTGGCACCCACTACATCATGGCAGCTGACGGGACCCAGTTGCACCA
CATCGAGTTGACCGCAGATGGCTCCATCTCCTTCCCAAGTCCAGATGCCCTGGCCTCTGGAGCCAAATGG
CCCGTGCTGCAGTGTGGAGGGCTGCCCAAAGATGGCCCTGAAGCTGAGGCCCCTTCTCCAGCCAGGACCC
ACCAGGTGGGAGACCCCCAGGGCTCTGCCTCCCCACCTCCTGCAGCCAACAAAGCCCTGGGCCTGGCAGT
GCCTCCCTCGCCACCATCTGCAGCCACTGCGTCATCAAAGAAGTTTTCCTGCAAGATCTGTGCTGAGGCC
TTCCCTGGCCGAGCTGAGATGGAGAGCCACAAACGGGCCCACGCTGGGCCTAGTGCCTTCAAGTGCCCTG
ACTGTCCTTTCAGTGCCCGCCAGTGGCCTGAGGTCCGGGCCCACATGGCACAGCACTCGAGCCTGCGGCC
CCACCAGTGCAGCCAGTGCAGCTTTGCCTCCAAGAACAAGAAGGATCTGCGGCGGCATGTGCTGACCCAC
ACCAATGAGAAGCCTTTCGCATGTCACCTCTGTGGACAGCGTTTCAACCGGAATGGGCACCTCAAGTTCC
ACATCCAACGGCTGCACAGTCCTGATGGGAGAAAAGCAGGGACTCCTGCCGTCCAGGCCCCCACCCAGAC
CCCCACCCAGACCATCATCCTGAACAGTGATGATGACACACTGGCCACACTGCACACTGCACTCCAGTCC
AGTCATGGGGTCCTGGGCCCAGAGAGGCTACAGCAGGCACTGGGCCAGGAACACATCATTGTGGCCCAGG
AGCAGACAGTGACCAATCAGGAGGAGGCCGCCTACATCCAAGAGATCACCACAGCAGATGGCCAGACAGT
GCAGCACCTGGTGACTGCTGACAATCAGGTACAGTACATCATCTCCCAGGATGGAGTCCAACATCTACTC
CCCCAGGAATATGTTGTGGTCCCAGAGGGCCATCATATCCAGGTACAGGAAGGCCAGATAACACACATCC
AGTACGAACAAGGGGCCCCATTCCTTCAGGAATCCCAGATCCAGTATGTGCCTGTGTCCCCAGGCCAGCA
GCTTGTCACACAGGCCCAGCTTGAGGCTGCAGCACACTCAGCCGTCACAGCGGTGGCTGATGCTGCTATG
GCCCAAGCACAGGGCCTGTTTGGCACAGAGGAGGCAGTGCCTGAACACATCCAACAGCTGCAACACCAGG
GCATCGAGTACGACGTCATCACCCTGACCAATGAC

PHC1

>human ENST00000543824.5
ATGGAGACTGAGAGCGAGCAGAACTCCAATTCCACCAATGGGAGTTCTAGCTCAGGGGGC
AGCTCTCGGCCCCAGATAGCTCAAATGTCACTTTATGAACGACAAGCAGTGCAGGCTCTG
CAAGCACTGCAGCGGCAGCCCAATGCAGCTCAGTATTTCCACCAGTTCATGCTCCAGCAG
CAGCTCAGTAATGCCCAGCTGCATAGCCTGGCTGCCGTCCAGCAGGCCACAATTGCTGCC
AGTCGGCAGGCCAGCTCCCCAAACACCAGCACTACACAGCAGCAGACTACCACCACCCAG
GCCTCGATCAATCTGGCCACCACATCGGCCGCCCAGCTCATCAGCCGATCCCAGAGTGTG
AGCTCTCCCAGTGCTACCACCTTGACCCAATCTGTGCTACTGGGGAACACCACCTCCCCA
CCCCTCAACCAGTCTCAGGCCCAGATGTATCTACGGCCACAGCTGGGAAACCTATTGCAG
GTAAACCGAACCCTGGGTCGGAATGTGCCTCTAGCCTCCCAACTCATCCTGATGCCTAAT
GGGGCGGTGGCTGCAGTCCAGCAGGAGGTGCCATCTGCTCAGTCTCCTGGAGTTCATGCA
GATGCAGATCAGGTTCAGAACTTGGCAGTAAGGAATCAACAGGCCTCAGCTCAAGGACCT
CAGATGCAAGGCTCCACTCAGAAGGCCATTCCTCCAGGAGCCTCCCCTGTCTCTAGCCTC
TCCCAGGCCTCTAGCCAGGCCCTAGCGGTGGCACAGGCTTCCTCTGGGGCCACAAACCAG
TCCCTCAACCTTAGTCAAGCTGGTGGAGGCAGTGGGAATAGCATCCCAGGGTCCATGGGT
CCAGGTGGAGGTGGGCAGGCACATGGTGGTTTGGGTCAGTTGCCTTCCTCAGGAATGGGT
GGTGGGAGCTGTCCCAGAAAGGGTACAGGAGTGGTGCAGCCCTTGCCTGCAGCCCAAACA
GTGACTGTGAGCCAGGGCAGCCAGACAGAGGCAGAAAGTGCAGCAGCCAAGAAGGCAGAA
GCAGATGGGAGTGGCCAGCAGAATGTGGGCATGAACCTGACACGGACAGCCACACCTGCG
CCCAGCCAGACACTTATTAGCTCAGCCACCTACACACAGATCCAGCCCCATTCACTGATT
CAGCAACAGCAACAGATCCACCTCCAGCAGAAACAGGTGGTGATCCAGCAGCAGATTGCC
ATCCACCACCAGCAGCAGTTCCAGCACCGGCAGTCCCAGCTCCTTCACACAGCTACACAC
CTCCAGTTGGCGCAGCAGCAGCAGCAGCAACAACAGCAACAGCAGCAACAGCAGCAGCCG
CAAGCCACCACCCTCACTGCCCCTCAGCCACCACAGGTCCCACCTACTCAGCAGGTCCCA
CCTTCCCAGTCCCAGCAGCAAGCCCAAACCCTGGTCGTTCAGCCCATGCTTCAGTCTTCA
CCCTTGTCTCTTCCACCTGATGCAGCCCCTAAGCCACCAATTCCCATCCAATCCAAACCA
CCTGTAGCACCTATCAAGCCGCCTCAGTTAGGGGCCGCTAAGATGTCAGCTGCCCAGCAA
CCACCACCCCATATCCCTGTGCAAGTTGTAGGCACTCGACAGCCAGGTACAGCCCAGGCA
CAGGCTTTGGGGTTGGCACAGCTGGCAGCTGCTGTACCTACTTCCCGGGGGATGCCAGGT
ACAGTGCAGTCTGGTCAGGCCCATTTGGCCTCCTCGCCACCTTCATCCCAGGCTCCTGGT
GCACTGCAGGAGTGCCCTCCCACATTGGCCCCTGGGATGACCCTTGCTCCTGTGCAGGGG
ACAGCACATGTGGTAAAGGGTGGGGCTACCACCTCCTCACCTGTTGTAGCCCAGGTCCCT
GCTGCCTTCTATATGCAGTCTGTGCACTTGCCGGGTAAACCCCAGACATTGGCTGTCAAA
CGCAAGGCTGACTCTGAGGAGGAGAGAGATGATGTCTCCACATTGGGTTCAATGCTTCCT
GCCAAGGCATCTCCAGTAGCAGAAAGCCCAAAAGTCATGGACGAGAAGAGCAGTCTTGGA
GAAAAAGCTGAATCAGTGGCTAATGTGAATGCTAATACTCCAAGCAGTGAACTAGTAGCC
TTGACCCCCGCCCCTTCAGTACCGCCTCCTACACTAGCCATGGTGTCTAGACAAATGGGT
GACTCAAAACCCCCACAGGCCATCGTGAAGCCCCAGATTCTCACCCACATCATTGAAGGC
TTTGTTATCCAGGAAGGAGCAGAACCTTTCCCGGTGGGTTGTTCTCAGTTACTGAAGGAG
TCTGAGAAGCCACTACAGACTGGCCTTCCGACAGGGCTGACTGAGAATCAGTCAGGTGGC
CCTTTGGGAGTGGACAGCCCATCTGCTGAGTTAGATAAGAAGGCGAATCTCCTGAAGTGC
GAGTACTGTGGGAAGTACGCCCCCGCAGAGCAGTTTCGTGGCTCTAAGAGGTTCTGCTCC
ATGACTTGCGCTAAGAGGTACAATGTGAGCTGTAGCCATCAGTTCCGGCTGAAGAGGAAA
AAAATGAAAGAGTTTCAAGAAGCCAACTATGCTCGCGTTCGCAGGCGTGGACCCCGCCGC
AGCTCCTCTGACATTGCCCGTGCCAAGATTCAGGGCAAGTGCCACCGGGGTCAAGAAGAC

TCTAGCCGGGGTTCAGATAATTCCAGTTATGATGAAGCACTCTCTCCAACATCTCCTGGG
CCTTTATCAGTAAGAGCTGGGCATGGAGAACGTGACCTGGGGAATCCCAATACAGCTCCA
CCTACACCGGAATTACATGGCATCAACCCTGTGTTCCTGTCCAGTAATCCCAGCCGTTGG
AGTGTAGAGGAGGTGTACGAGTTTATTGCTTCTCTCCAAGGCTGCCAAGAGATTGCAGAG
GAATTTCGCTCACAGGAGATTGATGGACAGGCCCTTTTATTACTTAAAGAAGAACATCTT
ATGAGTGCCATGAACATCAAGCTGGGCCCTGCCCTCAAGATCTGCGCCAAGATAAATGTC
CTCAAGGAGACC
>marmoset ENSCJAT00000036762.2
ATGGAGACTGAGAGCGAGCAGAACTCCAACTCCACCAATGGGAGTTCCAGCTCAGGGGGC
AGCTCTCGGCCCCAGATAGCTCAAATGTCACTATATGAACGACAAGCAGTGCAGGCTCTA
CAGGCACTGCAGCGGCAGCCCAATGCAGCTCAGTATTTCCACCAGTTCATGCTCCAGCAG
CAGCTCAGTAATGCCCAGTTGCATAGCCTGGCTGCCGTCCAGCAGGCCACAATTGCTGCC
AGTCGGCAAGCCAGCTCCCCAAACACCAGCACTACACAGCAGCAGACTACCACTACCCAG
GCCTCGATCAATCTGGCCACCACATCGGCCGCCCAGCTCATCAGCCGATCCCAGAGTGTG
AGCTCTCCCAGTGCTACCACCTTGACCCAATCTGTGCTACTGGGGAACACCACCTCCCCA
CCCCTCAACCAGTCTCAGGCCCAGATGTATCTACGGCCACAGCTGGGAAACCTATTGCAG
GTAAACCGAACCCTGGGTCGGAATGTGCCTCTAGCCTCCCAACTCATCCTGATGCCTAAT
GGTGCAGTGGCTGCAGTCCAGCAGGAGGTGCCATCTGCTCAGTCTCCTGGAGTTCATGCA
GATGCAGATCAGGTGCAGAACTTGGCAGTAAGGAATCAGCAGGCCTCAGCCCAAGGACCT
CAAATGCAAGGCTCCACTCAGAAGGCCATTCCTCCAGGAGCCTCCCCTGTCTCTAGTCTC
TCCCAGGCCTCTAGCCAGGCCCTCGCTGTGGCACAGGCTTCCTCTGGGGCCTCAAGCCAG
TCCCTCAACCTTAGTCAAGCTGGTGGAGGCAGTGGGAATAGCATCCCAGGGTCCATGGGT
CCAGGTGGAGGTGGCCAGGCACATGGTGGCTTGGGTCAGTTGCCTTCCTCAGGAATGGGT
GGTGGGAGCTGTCCCAGGAAGGGCACAGGAGTGGTGCAGCCCTTGCCTGCAGCCCAAACA
GTGACTGTGAGCCAGGGCAGCCAGACAGAGGCAGAAAGTGCAGCAGCCAAGAAGGCAGAA
GCAGATGGTGGTGGTCAGCAGAATGTGGGCATGAACCTGACCCGGACAGCCACACCTGCG
CCCAGCCAGACACTTATTAGCTCAGCCACCTACACACAGATCCAGCCCCATTCACTGATT
CAGCAACAGCAACAAATTCACCTCCAGCAGAAACAGGTGGTGATCCAGCAGCAGATTGCC
ATCCACCACCAGCAGCAGTTCCAGCACCGTCAGTCCCAGCTCCTTCACACAGCTACACAC
CTCCAATTGGCCCAGCAGCAGCAACAGCAGCAACAGCAGCAACAGCAGCAGCCGCAAGCC
ACAACCCTCACTGCCCCTCAGCCACCACAGGTCCCACCCACTCAGCAGGTCCCACCTTCC
CAGTCTCAGCAGCAAGCCCAAACCTTGGTCGTTCAGCCCATGCTTCAGTCTTCACCCTTG
TCCCTTCCACCTGATGCAGCTCCTAAGCCACCAATTCCCATCCAATCCAAACCACCTGTA
GCACCTATCAAGCCACCTCAGTTAGGGGCTGCTAAGATGTCAGCTACCCAGCAACCACCA
CCCCATATCCCTGTGCAAGTTGTAGGCACTCGACAGCCAGGTACAGCCCAGGCACAGGCT
TTGGGATTGGCACAGCTGGCAGCTGCTATACCCACTTCCCGGGGGATGCCAGGTACAGTG
CAGTCTGGTCAGGCCCATTTGGCCTCCTCGCCACCCTCATCCCAGGCTCCTGGTGCGCTG
CAGGAGTGCCCTCCCACATTGGCCCCTGGGATGACCCTTGCTCCCGTGCAGGGGACAGCA
CATGTAGTAAAGGGTGGGGCTACCACCTCCTCACCTGTTGTAGCCCAGGTCCCTGCTGCC
TTCTATATGCAGTCTGTGCACTTGCCGGGTAAACCCCAGACATTGGCTGTCAAACGCAAG
GCTGACTCTGAGGAGGAGAGAGATGATGTCTCCACATTGGGTTCAGTGTTTCCTGCCAAG
GCATCTCCAGTAGCAGAGAGCCCACAAGTCATGGAGGACAAGAGCAGTCTTGGAGAAAAA
GCTGAACCAGTGGCTAATGTGAATGCTAATACCCCAAGCAGTGAACTAGTAGCCTTGACC
CCCGCCCCATCAGTACCACCTCCTACATTAGCCATGGTGTCTAGACAAATGGGTGATTCA
AAACCCCCACAGGCCATCGTGAAGCCCCAGATTCTCACCCACATCATTGAAGGCTTTGTT
ATCCAGGAAGGAGCAGAACCTTTCCCGGTGGGTTGTTCTCAGTTACTAAAGGAGTCTGAA
AAGCCACTGCAGACTGGCCTTCCGACAGGGCTGACTGAGAATCAGTCAGGTGGCTCTTTG

GGAGTGGACAGCCCATCTGCCGAGTTAGATAAGAAGGCGAATCTCCTGAAGTGCGAGTAC
TGTGGGAAGTACGCCCCTGCAGAGCAATTTCGTGGCTCTAAGAGGTTCTGCTCCATGACT
TGCGCTAAGAGGTACAATGTGAGCTGTAGCCATCAGTTCCGGCTGAAGAGGAAAAAAATG
AAAGAGTTTCAAGAAGCCAACTATGCCCGAGTTCGCAGGCGTGGCCCCGCCGCAGCTCC
TCTGACATTGCCCGTGCCAAGATTCAGGGCAAGTGCCACCGGGGTCAAGAGGACTCTAGC
CGGGGTTCAGATAATTCCAGTTATGATGAAGCACTCTCTCCAACATCTCCTGGGCCTTTA
TCAGTAAGGGCTGGGCATGGAGAACGTGACCTGGGGAACCCCAATACAGCTCCACCTACA
CCGGAATTACATGGCATCAACCCTGTGTTCCTGTCTAGTAATCCCAGCCGTTGGAGTGTA
GAAGAGGTGTATGAGTTTATTGCTTCTCTCCAAGGCTGCCAAGAGATTGCAGAGGAATTT
CGTTCCCAGGAGATTGATGGACAGGCCCTTTTATTACTTAAGGAAGAACATCTTATGAGT
GCCATGAACATCAAGCTGGGTCCTGCCCTCAAGATCTGCGCCAAGATAAATGTCCTCAAG
GAGACC
>vervet AGM ENSCSAT00000007598.1
ATGGAGACTGAGAGCGAGCAGAACTCCAACTCCACCAATGGGAGTTCCAGCTCAGGGGGC
AGCTCTCGGCCCCAGATAGCTCAAATGTCACTATATGAGCGACAAGCAGTGCAGGCTCTG
CAGGCACTGCAGCGGCAGCCCAATGCAGCTCAGTATTTCCACCAGTTCATGCTCCAGCAG
CAACTCAGTAATGCCCAGCTGCATAGCCTGGCTGCTGTCCAGCAGGCCACAATTGCTGCC
AGTCGGCAGGCCAGCTCCCCAAACACCAGCACTACACAGCAGCAGACTACCACCACCCAG
GCCTCGATCAATCTGGCCACCACATCGGCCGCCCAGCTCATCAGCCGATCCCAGAGTGTG
AGCTCTCCCAGTGCTACCACCTTGACCCAATCTGTGCTACTGGGGAACACCACCTCCCCA
CCCCTCAACCAGTCGCAGGCCCAGATGTATCTACGGCCACAGCTGGGAAACCTATTGCAG
GTAAACCGAACCCTGGGTCGGAATGTGCCTCTAGCTTCCCAACTCATCCTGATGCCTAAT
GGGGCAGTGGCTGCAGTCCAGCAGGAGGTGCCATCTGCTCAGTCTCCTGGAGTTCATGCA
GATGCAGATCAGGTGCAGAACTTGGCAGTAAGGAATCAACAGGCCTCAGCTCAAGGACCT
CCAATGCAAGGCTCCACTCAGAAGGCCATTCCTCCGGGAGCCTCCCCTGTCTCTAGCCTC
TCCCAGGCCTCTAGCCAGGCCCTAGCGGTGGCACAGGCTTCCTCTGGGGCCACAAGCCAG
TCCCTCAACCTTAGTCAAGCTGGTGGAGGCAGTGGGAATAACATCCCAGGGTCCATGGGT
CCAGGTGGAGGTGGCCAGGCACATGGTGGTTTGGGCCAGTTGCCTTCCTCAGGAGTGGGT
GGTGGGAGCTGTCCCAGGAAGGGCACAGGAGTGGTGCAGCCCTTGCCTGCAGCCCAAACA
GTGACTGTGAGCCAGGGCAGCCAGACAGAGGCAGAAAGTGCAGCAGCCAAGAAGGCAGAA
GCAGATGGGAGCGGTCAGCAGAATGTGGGCATGAACCTGACACGGACAGCCACACCTGCG
CCCAGCCAGACACTTATTAGCTCAGCCACCTACACACAAATCCAGCCCCATTCACTGATT
CAGCAACAGCAACAGATCCACCTCCAGCAGAAACAGGTGGTGATCCAGCAGCAGATTGCC
ATCCACCACCAGCAGCAGTTCCAGCATCGTCAGTCTCAGCTCCTTCACACAGCTACACAC
CTCCAGTTGGCGCAGCAGCAGCAGCAGCAGCAGCAGCAGCAGCAACAGCAGCAGCCGCAA
GCCACCACTCTCACTGCCCCTCAGCCACCACAGGTCCCACCTACTCAGCAGGTCCCACCT
TCGCAGTCCCAGCAGCAAGCCCAAACCCTGGTCGTTCAGCCCATGCTTCAGTCTTCACCC
TTGTCTCTTCCACCTGATGCAGCCCCTAAGCCACCAATTCCCATCCAATCCAAGCCACCT
GTAGCACCTATCAAGCCACCTCAGTTAGGGACTGCTAAGATGTCAGCTACCCAGCAACCA
CCACCCCATATCCCAGTGCAAGTTGTAGGCACTAGACAGCCAGGTACAGCCCAGGCACAG
GCTTTGGGGTTGGCACAGCTGGCAGCTGCTGTACCTACTTCCCGGGGGATGCCAGGTCCA
GTGCAGTCTGGTCAGGCCCATTTGGCCTCCTCGCCACCTTCATCCCAGGCTCCTGGTGCA
CTGCAGGAGTGCCCTCCCACATTGGCCCCTGGGATGTCCCTTGCTCCTGTGCAGGGGACA
GCACACGTGGTGAAGGGTGGGGCTACCACCTCCTCACCTGTTGTAGCCCAGGTCCCTGCT
GCCTTCTATATGCAGTCTGTGCACTTGCCGGGTAAACCCCAAACATTGCCTGTCAAACGC
AAGGCTGACTCTGAGGAGGAGAGAGATGATGTCTCCACGTTGGGTTCAATGCTTCCTGCC
AAGGCATCTCCAGTAGCGGAGAGCCCAAAAGTCATGGATGAGAAGAGCAGTCTTGGAGAA

AAAGCTGAATCAGTGGCTAATATGAATGCTAATACCCCAGGCAGTGAACTAGTAGCCTTG
ACCCCTGCCCCATCAGTACCGCCTCCTACACTAGCCATGGTGTCTAGACAGATGGGTGAC
TCAAAACCCCCACAGGCCATCGTGAAGCCCCAGATTCTCACCCACATCATTGAAGGCTTT
GTTATCCAGGAAGGAGCAGAACCTTTCCCGGTGGGTTGTTCTCAGTTACTGAAGGAGTCT
GAGAAGCCACTACAGACTGGCCTTCCGACAGGGCTGACTGAGAATCAGTCAGGTGGCCCT
TTGGGAGTAGACAGCCCCTCTGCCGAGTTAGATAAGAAGGCGAATCTCCTGAAGTGCGAG
TACTGTGGGAAGTACGCCCCCGCAGAGCAGTTTCGTGGCTCTAAGAGGTTCTGCTCCATG
ACTTGCGCTAAGAGGTACAATGTGAGCTGTAGCCATCAGTTCCGGCTGAAGAGGAAAAAA
ATGAAAGAGTTTCAAGAAGCCAACTACGCTCGCGTTCGCAGGCGTGGACCCCGTCGCAGC
TCCTCTGACATTGCCCGTGCCAAGATTCAGGGCAAGTGCCACCGGGGTCAAGAAGACTCT
AGCCGAGGTTCAGATAATTCCAGTTATGATGAAGCACTCTCTCCAACATCTCCTGGGCCT
TTATCAGTGAGAGCTGGGCATGGAGAACGTGATCTGGGGAATCCCAATACAGCTCCACCT
ACACCGGAATTACATGGCATCAACCCTGTGTTCCTGTCCAGTAATCCCAGCCGTTGGAGT
GTAGAGGAGGTGTACGAGTTTATTGCTTCTCTCCAAGGCTGCCAAGAGATTGCGGAGGAA
TTTCGCTCACAGGAGATTGATGGACAGGCCCTTTTATTACTTAAAGAAGAACATCTTATG
AGTGCCATGAACATCAAGCTGGGCCCTGCCCTCAAGATCTGCGCCAAGATAAATGTCCTC
AAGGAGACC
>gorilla XM_004052662.1
ATGGAGACTGAGAGCGAGCAGAACTCCAACTCCACCAATGGGAGTTCCAGCTCAGGGGGCAGCTCTCGGCCC
C
AGATAGCTCAAATGTCACTATATGAACGACAAGCAGTGCAGGCTCTGCAGGCACTGCAGCGGCAGCCCAA
TGCAGCTCAGTATTTCCACCAGTTCATGCTCCAGCAGCAGCTCAGTAATGCCCAGCTGCATAGCCTGGCT
GCCGTCCAGCAGGCCACAATTGCTGCCAGTCGGCAGGCCAGCTCCCCAAACACCAGCACTACACAGCAGC
AGACTACCACCACCCAGGCCTCGATCAATCTGGCCACCACATCGGCCGCCCAGCTCATCAGCCGATCCCA
GAGTGTGAGCTCTCCCAGTGCTACCACCTTGACCCAATCTGTGCTACTGGGGAACACCACCTCCCCACCC
CTCAACCAGTCTCAGGCCCAGATGTATCTACGGCCACAGCTGGGAAACCTATTGCAGGTAAACCGAACCC
TGGGTCGGAATGTGCCTCTAGCCTCCCAACTCATCCTGATGCCTAATGGGGCAGTGGCTGCAGTCCAGCA
GGAGGTGCCATCTGCTCAGTCTCCTGGAGTTCATGCAGATGCAGATCAGGTGCAGAACTTGGCAGTAAGG
AATCAACAGGCCTCAGCTCAAGGACCTCAGATGCAAGGCTCCACTCAGAAGGCCATTCCTCCAGGAGCCT
CCCCTGTCTCTAGCCTCTCCCAGGCCTCTAGCCAGGCCCTAGCGGTGGCACAGGCTTCCTCTGGGGCCAC
AAACCAGTCCCTCAACCTTAGTCAAGCTGGTGGAGGCAGTGGGAATAGCATCCCAGGGTCCATGGGTCCA
GGTGGAGGTGGCCAGGCACATGGTGGTTTGGGTCAGTTGCCTTCCTCAGGAATGGGTGGTGGGAGCTGTC
CCAGGAAGGGTACAGGAGTGGTGCAGCCCTTGCCTGCAGCCCAAACAGTGACTGTGAGCCAGGGCAGCCA
GACAGAGGCAGAAAGTGCAGCAGCCAAGAAGGCAGAAGCAGATGGGAGTGGTCAGCAGAATGTGGGCATG
AACCTGACACGGACAGCCACACCTGCGCCCAGCCAGACACTTATTAGCTCAGCCACCTACACACAGATCC
AGCCCCATTCACTGATTCAGCAACAGCAACAGATCCACCTCCAGCAGAAACAGGTGGTGATCCAGCAGCA
GATTGCCATTCACCACCAGCAGCAGTTCCAGCACCGGCAGTCCCAGCTCCTTCACACAGCTACACACCTC
CAGTTGGCGCAGCAGCAGCAGCAGCAACAACAACAACAGCAGCAACAGCAGCAGCCACAAGCCACCACCC
TCACTGCCCCTCAGCCACCACAGGTCCCACCTACTCAGCAGGTCCCACCTTCCCAGTCCCAGCAGCAAGC
CCAAACCCTGGTCGTTCAGCCCATGCTTCAGTCTTCACCCTTGTCTCTTCCACCTGATGCAGCCCCTAAG
CCACCAATTCCCATCCAATCCAAACCACCTGTAGCACCTATCAAGCCACCTCAGTTAGGGGCTGCTAAGA
TGTCAGCTACCCAGCAACCACCACCCCATATCCCTGTGCAAGTTGTAGGCACTCGACAGCCAGGTACAGC
CCAGGCACAGGCTTTGGGGTTGGCACAGCTGGCAGCTGCTGTACCTACTTCCCGGGGGATGCCAGGTACA
GTGCAGTCTGGTCAGGCCCATTTGGCCTCCTCGCCACCTTCATCCCAGGCTCCTGGTGCACTGCAGGAGT
GCCCTCCCACATTGGCCCCTGGGATGACCCTTGCTCCTGTGCAGGGGACAGCACATGTGGTAAAGGGTGG
GGCTACCACCTCCTCACCCGTTGTAGCCCAGGTCCCTGCTGCCTTCTATATGCAGTCTGTGCACTTGCCG
GGTAAACCCCAGACACTGGCTGTCAAACGCAAGGCTGACTCTGAGGAGGAGAGAGATGATGTCTCCACAT

TGGGTTCAATGCTTCCTGCCAAGGCATCTCCAGTAGCAGAAAGCCCAAAAGTCATGGACGAGAAGAGCAG
TCTTGGAGAAAAAGCTGAATCAGTGGCTAATGTGAATGCTAATACTCCAAGCAGTGAACTAGTAGCCTTG
ATCCCCGCCCCTTCAGTACCGCCTCCTACACTAGCCATGGTGTCTAGACAAATGGGTGACTCAAAACCCC
CACAGGCCATCGTGAAGCCCCAGATTCTCACCCACATCATTGAAGGCTTTGTTATCCAGGAAGGAGCAGA
ACCTTTCCCGGTGGGTTGTTCTCAGTTACTGAAGGAGTCTGAGAAGCCACTACAGACTGGCCTTCCGACA
GGGCTGACTGAGAATCAGTCAGGTGGCCCTTTGGGAGTGGACAGCCCATCTGCTGAGTTAGATAAGAAGG
CGAATCTCCTGAAGTGCGAGTACTGTGGGAAGTACGCCCCCGCAGAGCAGTTTCGTGGCTCTAAGAGGTT
CTGCTCCATGACTTGCGCTAAGAGGTACAATGTGAGCTGTAGCCATCAGTTCCGGCTGAAGAGGAAAAAA
ATGAAAGAGTTTCAAGAAGCCAACTATGCTCGCGTTCGCAGGCGTGGACCCCGCCGCAGCTCCTCTGACA
TTGCCCGTGCCAAGATTCAGGGCAAGTGCCACCGGGGTCAAGAAGACTCTAGCCGGGGTTCAGATAATTC
CAGTTATGATGAAGCACTCTCTCCAACATCTCCTGGGCCTTTATCAGTAAGAGCTGGGCATGGAGAACGT
GACCTGGGGAATCCCAATACAGCTCCACCTACACCGGAATTACATGGCATCAACCCTGTGTTCCTGTCCA
GTAATCCCAGCCGTTGGAGTGTAGAGGAGGTGTACGAGTTTATTGCTTCTCTCCAAGGCTGCCAAGAGAT
TGCAGAGGAATTTCGCTCACAGGAGATTGATGGACAGGCCCTTTTATTACTTAAAGAAGAACATCTTATG
AGTGCCATGAACATCAAGCTGGGCCCTGCCCTCAAGATCTGCGCCAAGATAAATGTCCTCAAGGAGACC
>macca malatta NM_001260906.1
ATGGAGACTGAGAGCGAGCAGAACTCCAACTCCACCAATGGGAGTTCCA
GCTCAGGGGGCAGCTCTCGGCCCCAGATAGCTCAAATGTCACTGTATGAGCGACAAGCAGTGCAGGCTCT
GCAGGCACTGCAGCGGCAGCCCAATGCAGCTCAGTATTTCCACCAGTTCATGCTCCAGCAGCAACTCAGT
AATGCCCAGCTGCATAGCCTGGCTGCTGTCCAGCAGGCCACAATTGCTGCCAGTCGGCAGGCCAGCTCCC
CAAACACCAGCACTACACAGCAGCAGACTACCACCACCCAGGCCTCAATCAATCTGGCCACCACATCGGC
CGCCCAGCTCATCAGCCGATCCCAGAGTGTGAGCTCTCCCAGTGCTACCACCTTGACCCAATCTGTGCTA
CTGGGGAACACCACCTCCCCACCCCTCAACCAGTCGCAGGCCCAGATGTATCTACGGCCACAGCTGGGAA
ACCTATTGCAGGTAAACCGAACCCTGGGTCGGAATGTGCCTCTAGCTTCCCAACTCATCCTGATGCCTAA
TGGGGCAGTGGCTGCAGTCCAGCAGGAGGTGCCATCTGCTCAGTCTCCTGGAGTTCATGCAGATGCAGAT
CAGGTGCAGAACTTGGCAGTAAGGAATCAACAGGCCTCAGCTCAAGGACCTCCAATGCAAGGCTCCACTC
AGAAGGCCATTCCTCCGGGAGCCTCCCCTGTCTCTAGCCTCTCCCAGGCCTCTAGCCAGGCCCTAGCGGT
GGCACAGGCTTCCTCTGGGGCCACAAGCCAGTCCCTCAACCTTAGTCAAGCTGGTGGAGGCAGTGGGAAT
AGCATCCCGGGGTCCATGGGTCCAGGTGGAGGTGGCCAGGCACATGGTGGTTTGGGCCAGTTGCCTTCCT
CAGGAGTGGGTGGTGGGAGCTGTCCCAGGAAGGGCACAGGAGTGGTGCAGCCCTTGCCTGCAGCCCAAAC
AGTGACTGTGAGCCAGGGCAGCCAGACAGAGGCAGAAAGTGCAGCAGCCAAGAAGGCAGAAGCAGATGGG
AGCGGTCAGCAGAATGTGGGTATGAACCTGACACGGACAGCCACACCTGCGCCCAGCCAGACACTTATTA
GCTCAGCCACCTACACACAAATCCAGCCCCATTCACTGATTCAGCAACAGCAACAGATCCACCTCCAGCA
GAAACAGGTGGTGATCCAGCAGCAGATTGCCATCCACCACCAGCAGCAGTTCCAGCATCGTCAGTCTCAG
CTCCTTCACACAGCTACACACCTCCAGTTGGCGCAGCAGCAGCAGCAACAACAGCAGCAGCAGCAACAGC
AGCAGCCGCAAGCCACCACTCTCACTGCCCCTCAGCCACCACAGGTCCCACCTACTCAGCAGGTCCCACC
TTCGCAGTCCCAGCAGCAAGCCCAAACCCTGGTCGTTCAGCCCATGCTTCAGTCTTCACCCTTGTCTCTT
CCACCTGATGCAGCCCCTAAGCCACCAATTCCCATCCAATCCAAACCACCTGTAGCACCTATCAAGCCAC
CTCAGTTAGGGACTGCTAAGATGTCAGCTACCCAGCAACCACCACCCCATATCCCAGTGCAAGTTGTAGG
CACTCGACAGCCAGGTACAGCCCAGGCACAGGCTTTGGGCTTGGCACAGCTGGCAGCTGCTGTACCTACT
TCCCGGGGGATGCCAGGTCCAGTGCAGTCTGGTCAGGCCCATTTGGCCTCCTCGCCACCTTCATCCCAGG
CTCCTGGTGCACTGCAGGAGTGCCCTCCCACATTGGCCCCTGGGATGACCCTTGCTCCTGTGCAGGGGAC
AGCACATGTGGTGAAGGGTGGGGCTACCACCTCCTCACCTGTTGTAGCCCAGGTCCCTGCTGCCTTCTAT
ATGCAGTCTGTGCACTTGCCGGGTAAACCCCAAACATTGCCTGTCAAACGCAAGGCTGACTCTGAGGAGG
AGAGAGATGATGTCTCCACGTTGGGTTCAATGCTTCCTGCCAAGGCATCTCCAGTAGCAGAGAGCCCAAA
AGTCATGGATGAGAAGAGCAGTCTTGGAGAAAAAGCTGAATCAGTGGCTAATATGAATGCTAATACCCCA
GGCAGTGAACTAGTAGCCTTGACCCCTGCCCCATCAGTACCGCCTCCTACACTAGCCATGGTGTCTAGAC

AGATGGGTGACTCAAAACCCCCACAGGCCATCGTGAAGCCCCAGATTCTCACCCACATCATTGAAGGCTT
TGTTATCCAGGAAGGAGCAGAACCTTTCCCGGTGGGTTGTTCTCAGTTACTGAAGGAGTCTGAGAAGCCA
CTACAGACTGGCCTTCCGACAGGGCTGACTGAGAATCAGTCAGGTGGCCCTTTGGGAGTAGACAGCCCCT
CTGCCGAGTTAGATAAGAAGGCGAATCTCCTGAAGTGCGAGTACTGTGGGAAGTACGCCCCCGCAGAGCA
GTTTCGTGGCTCTAAGAGGTTCTGCTCCATGACTTGCGCTAAGAGGTACAATGTGAGCTGTAGCCATCAG
TTCCGGCTGAAGAGGAAAAAAATGAAAGAGTTTCAAGAAGCCAACTATGCTCGCGTTCGCAGGCGTGGAC
CCCGCCGCAGCTCCTCTGACATTGCCCGTGCCAAGATTCAGGGCAAGTGCCACCGGGGTCAAGAAGACTC
TAGCCGAGGTTCAGATAATTCCAGTTATGATGAAGCACTCTCTCCAACATCTCCTGGGCCTTTATCAGTA
AGAGCTGCGCATGGAGAACGTGATCTGGGGAATCCCAATACAGCTCCACCTACACCAGAATTACATGGCA
TCAACCCTGTGTTCCTGTCCAGTAATCCCAGCCGTTGGAGTGTAGAGGAGGTGTACGAGTTTATTGCTTC
TCTCCAAGGCTGCCAAGAGATTGCGGAGGAATTTCGCTCACAGGAGATTGATGGACAGGCCCTTTTATTA
CTTAAAGAAGAACATCTTATGAGTGCCATGAACATCAAGCTGGGCCCTGCCCTCAAGATCTGCGCCAAGA
TAAATGTCCTCAAGGAGACC
>Microcebus murinus XM_012777612.1
ATGGAGACTGAGAGTGAGCAGAACTCCAACTCCACTAATGGGAGTTCCAGCTCAGGG
GGCAGCTCTCGGCCCCAGATAGCTCAAATGTCATTGTATGAACGACAAGCAGTGCAGGCTCTGCAGGCAC
TGCAGCGGCAGCCTAATGCGGCTCAGTATTTCCACCAGTTCATGCTCCAGCAGCAGCTCAGTAATGCCCA
GTTGCATAGCCTGGCTGCTGTCCAGCAGGCCACAATTGCTGCCAGTCGGCAGGCCAGCTCCCCAAATACC
AGCAGCAGCCAGCAGCAGACTACCACCACCCAGGCCTCAATCAATCTGGCCACCACGTCAGCTGCCCAGC
TCATCAGCCGATCCCAGAGTGTGAGCTCTCCCAGTGCTACCACCTTGACCCAATCTGTGTTACTGGGGAA
CACCACCTCCCCACCCCTCAACCAGTCCCAGGCCCAGATGTATCTACGGCCACAGCTGGGAAACATATTG
CAGGTAAACCGAACCCTGGGCCGGAATGTGCCTCTAGCCTCCCAACTCATCCTGATGCCCAATGGGGCAG
TGGCTGCAGTCCAGCAGGAGGTACCATCTGCACAGTCTCCTGGAGTTCATGCAGATGCAGATCAGGTGCA
GAACTTGGCAGTGAGGAACCAACAAGCCTCAGCCCAAGGATCCCAAATGCAAGGCTCCACTCAGAAGGCC
ATACCTCCTGGAGCCTCTCCTGTGTCTAGCCTCTCTCAGGCCTCTAGCCAGGCCCTAGCTGTGGCACAGG
CCTCCTCTGGGGCCTCAGGCCAGTCCCTCAACCTTAGTCAAGCTGGTGGAAGTAGTGGGAATAGCATCGC
AGGGTCCATGGGTCCAGGTGGAGGCGGCCAGGCACCTGGGGGTTTGGGTCAGTTGCCTTCCTCAGGAATG
AGTGGTGGGAGCTGTCCCAGGAAGGGCACAGGAGTAGTGCAGCCCCTGCCTGCAGCCCAGACAGTGACTG
TGAGCCAGGGCAGCCAGACAGAGGCAGAAAGTGCAGCAGCCAAGAAGGCAGAAGCAGATGGGAGTGGTCA
GCAGAATGTGGGCATGAACCTGACACGGACAGCCACACCTGCGCCCAGCCAGACACTTATTAGCTCAGCC
ACCTACACACAGATCCAGCCCCATTCCCTGATTCAGCAACAGCAACAGATCCACCTCCAGCAGAAACAGG
TGGTGATCCAGCAGCAGATTGCCATCCACCACCAGCAGCAGTTCCAGCACCGCCAGTCCCAGCTCCTTCA
CACAGCTACACACCTCCAGTTGGCCCAACAGCAGCAGCAGCAGCAGCAGCAGCAGCAGCAGCAGCAGCAG
CAGCAGCAGCAGCAGCAGCAGCAGCAAGCCACAACCCTCACTGCCCCTCAGCCACCACAGGTCCCACCTA
CTCAGCAGGTCCCACCCTCCCAGTCACAGCAGCAAGCCCAAACCCTGGTTGTTCAACCCATGCTTCAGTC
TTCACCCCTGTCCCTTCCACCTGACCCAACCCCCAAGCCACCAATTCCTATCCAATCCAAACCACCTGTG
GCACCTATTAAGCCTCCTCAGTTAGGGGCTGCTAAGATGTCAGCTACCCAGCAACCACCACCTCATATCC
CTGTGCAAGTTGTAGGCACCCGACAGCCAGGTACAGCCCAGGCACAGGCTTTGGGGTTGGCACAGCTGGC
AGCTGCTGTACCTACTTCCCGAGGGATGCCGGGTACAGTGCAGCCTAGTCAGGCCCACTTTGCCCCCTCG
CCACCTTCATCCCAGGCTCCTGGTGCACTGCAGGAGTGCCCTCCTTCTTTGGCCCCTGGGATGACCCTTG
CTCCTGTACAGGGGACAGCACATGTTGTAAAGGGTGGGGCTACCACCTCCTCACCTGTTGTAGCCCAGGT
GCCTGCTTCCTTCTACATGCAGTCTGTACACTTGCCGGGTAAACCCCAGACTCTGGCTGTCAAACGCAAA
GCTGAGTCTGAGGAGGACAGAGATGACGTCTCCACATTGAGTTCAATGCTTTCTGCTAAGGCATCTCCAG
TGGCAGAGAGCCCAAAGGTCATTGAGGAGAAGAGCAGTCTTGGAGAGAAAGCTGAACCAGTGGCCAACGT
GAATGCTAATACCCCAAGCAGTGAACTAGTAGCCTTGACCCCTGCCCCATCAGCACCACCTCCTACACTA
GCCATGGTTTCCAGACAAATGGGTGACTCAAAACCCCCACAGGCCATTGTGAAGCCCCAGATTCTCACCC
ACATCATTGAAGGCTTTGTTATCCAGGAAGGAGCAGAACCTTTCCCGGTGGGTTGTTCTCAGTTACTGAA

GGAGTCTGAGAAGCCACTACAGGCTGGCCTTCCAACAGGGCTGAATGAGAATCAGTCAGGTGGCCCCTTG
GGAGGGGACAGCCCATCTGTTGAGTTAGATAAGAAGGCGAATCTCCTGAAGTGCGAGTACTGTGGGAAGT
ACGCCCCTGCAGAGCAGTTTCGTGGCTCTAAGAGGTTCTGCTCCATGACTTGCGCTAAGAGGTACAATGT
GAGCTGTAGCCATCAGTTCCGGCTGAAGAGGAAAAAAATGAAAGAGTTTCAAGAAGCCAACTATACTCGT
GTTCGCAGGCGGGGACCCCGCCGCAGCTCCTCTGACATTGCCCGTGCTAAGATTCAGGGCAAACGCCACC
GGGGTCAAGAGGACTCTAGCCGGGGTTCAGATAATTCCAGTTATGACGAAGCACTCTCCCCAACATCTCC
CGGGCCTTTATCAGTAAGAGCTGGGCATGGAGAACGTGATCTGGGGAACCCCAGTATAGCTCCACCCACA
CCAGAATTGCATGGCATCAATCCCGTGTTCCTGTCCAGTAATCCCAGCCGTTGGAGTGTAGAGGAGGTGT
ACGAGTTTATCGCTTCTCTCCAAGGCTGCCAAGAGATTGCAGAGGAGTTTCGCTCCCAGGAGATTGATGG
ACAGGCCCTTTTATTACTTAAAGAAGAACATCTTATGAGTGCCATGAACATCAAGCTGGGCCCTGCCCTC
AAGATCTGCGCCAAGATAAACATCCTCAAGGAGACC
>gibbon ENSNLET00000002978.1
ATGGAGACTGAGAGCGAGCAGAACTCCAACTCCACCAATGGGAGTTCCAGCTCAGGGGGC
AGCTCTCGGCCCCAGATAGCTCAAATGTCACTATACGAACGACAAGCAGTGCAGGCTCTG
CAGGCACTGCAGCGGCAGCCCAATGCAGCTCAGTATTTCCACCAGTTCATGCTCCAGCAG
CAGCTCAGTAATGCCCAGCTGCATAGCCTGGCTGCCGTCCAGCAGGCCACAATTGCTGCC
AGTCGGCAGGCCAGCTCCCCAAACACCAGCACTGCACAGCAGCAGACTACCACCACCCAG
GCCTCGATCAATCTGGCCACCACATCGGCCGCCCAGCTCATCAGCCGATCCCAGAGTGTG
AGCTCTCCCAGTGCTACCACCTTGACCCAATCTGTGCTACTGGGGAACACCACCTCCCCA
CCCCTCAACCAGTCTCAGGCCCAGATGTATCTACGGCCACAGCTGGGAAACCTATTGCAG
GTAAACCGAACCCTGGGTCGGAATGTGCCTCTAGCCTCCCAACTCATCCTGATGCCTAAT
GGGGCAGTGGCTGCAGTCCAGCAGGAAGTGCCATCTGCTCAGTCTCCTGGAGTTCATGCA
GATGCAGATCAGGTGCAGAACTTGGCAGTAAGGAATCAACAGGCCTCAGCTCAAGGACCT
CAAATGCAAGGCTCCACTCAGAAGGCCATTCCTCCAGGAGCCTCCCCTGTCTCTAGCCTC
TCCCAGGCCTCTAGCCAGGCCCTGGCGGTGGCACAGGCTTCCTCTGGGGCCACAAACCAG
TCCCTCAACCTTAGTCAAGCTGGTGGAGGCAGTGGGAATAGCATCCCAGGGTCCATGGGT
CCAGGTGGAGGTGGCCAGGCACATGGTGGTTTGGGTCAGTTGCCTTCCTCAGGAATGGGT
GGTGGGAGCTGTCCCAGGAAGGGCACAGGAGTGGTACAGCCCTTGCCTGCAGCCCAAACA
GTGACTGTGAGCCAGGGCAGCCAGACAGAGGCAGAAAGTGCAGCAGCCAAGAAGGCAGAA
GCAGATGGGAGTGGCCAGCAGAATGTGGGCATGAACCTGACACGGACAGCCACACCTGCG
CCCAGCCAGACACTTATTAGCTCAGCCACCTACACACAGATCCAGCCCCATTCACTGATT
CAGCAACAGCAACAGATCCACCTCCAGCAGAAACAGGTGGTGATCCAGCAGCAGATTGCC
ATCCACCACCAGCAGCAGTTCCAGCACCGGCAGTCCCAGCTCCTTCACACAGCTACACAC
CTCCAGTTGGCGCAGCAGCAGCAGCAGCAACAACAGCAACAGCAACAGCAGCAGCAGCCG
CAAGCCACCACCCTCACTGCCCCTCAGCCACCACAGGTCCCACCTACTCAGCAGGTCCCA
CCTTCCCAGTCCCAGCAGCAAGCCCAAACCCTGGTTGTTCAGCCCATGCTTCAGTCTTCA
CCCTTGTCTGTTCCACCTGATGCAGCCCCTAAGCCACCAATTCCCATCCAATCCAAACCA
CCTGTAGCACCTATCAAGCCACCTCAGTTAGGGGCTGCTAAGATGTCAGCTACCCAGCAA
CCACCACCCCATATCCCTGTGCAAGTTGTAGGCACTCGACAGCCAGGTACAGCCCAGGCA
CAGGCTTTGGGGTTGGCACAGCTGGCAGCTGCTGTACCTACTTCCCGGGGGATGCCAGGT
ACAGTGCAGTCTGGTCAGGCCCATTTGGCCTCCTCGCCACCTTCATCCCAGGCTCCTGGT
GCACTGCAGGAGTGCCCTCCCACATTGGCCCCTGGGATGACCCTTGCTCCTGTGCAGGGG
ACAGCACATGTGGTAAAGGGTGGGGCTACCACCTCCTCACCTGTTGTAGCCCAGGTCCCT
GCTGCCTTCTATATGCAGTCTGTGCACTTGCCGGGTAAACCCCAGACACTGGCTGTCAAA
CGCAAGGCTGACTCTGAGGAGGAGAGAGATGATGTCTCCACATTGGGTTCAATGCTTCCT
GCCAAGGCATCTCCAGTAGCAGAGAGCCCAAAAGTCATGGACGAGAAGAGCAGTCTTGGA
GAAAAAGCTGAATCGGTGGCTAATGTGAATGCTAATACCCCAAGCAGTGAACTAGTAGCC

TTGACCCCCGCCCCATCAGTACCGCCTCCTACACTAGCCATGGTGTCTAGACAAATGGGT
GACTCAAAACCCCCACAGGCCATCGTGAAGCCCCAGATCCTCACCCACATCATTGAAGGC
TTTGTTATCCAGGAAGGAGCAGAACCTTTCCCGGTGGGTTGTTCTCAGTTACTGAAGGAG
TCTGAGAAGCCACTACAGACTGGCCTTCCGACAGGGCTGACTGAGAATCAGTCAGGTGGC
CCTTTGGGAGTGGACAGCCCATCTGCCGAGTTAGATAAGAAGGCGAATCTCCTGAAGTGC
GAGTACTGTGGGAAGTACGCCCCCGCAGAGCAGTTTCGTGGCTCTAAGAGGTTCTGCTCC
ATGACTTGCGCTAAGAGGTACAATGTGAGCTGTAGCCATCAGTTCCGGCTGAAGAGGAAA
AAAATGAAAGAGTTTCAAGAAGCCAACTATGCTCGCGTTCGCAGGCGTGGACCCCGCCGC
AGCTCCTCTGACATTGCCCGTGCCAAGATTCAGGGCAAGTGCCACCGGGGTCAAGAAGAC
TCTAGCCGGGGTTCAGATAATTCCAGTTATGATGAAGCACTCTCTCCAACATCTCCTGGG
CCTTTATCAGTAAGAGCTGGGCATGGAGAACGTGACCTGGGGAATCCCAATACAGCTCCA
CCTACACCGGAATTACATGGCATCAACCCTGTGTTCCTGTCCAGTAATCCCAGCCGTTGG
AGTGTAGAGGAGGTGTACGAGTTTATTGCTTCTCTCCAAGGCTGCCAAGAGATTGCAGAG
GAATTTCGCTCACAGGAGATTGATGGACAGGCCCTTTTATTGCTTAAAGAAGAACATCTT
ATGAGTGCCATGAACATCAAGCTGGGCCCTGCTCTCAAGATCTGCGCCAAGATAAATGTC
CTCAAGGAGACC
>bushbaby ENSOGAT00000005608.2
ATGGAGACTGAGAGTGAGCAGAACTCCAACTCCACCAATGGGAGTTCCAGCTCAGGGGGC
AGCTCTCGGCCCCAGATAGCTCAAATGTCACTGTATGAACGACAAGCAGTGCAGGCTCTG
CAGGCACTTCAGCGACAGCCCAATGCAGCTCAGTATTTCCACCAGTTCATGCTCCAGCAG
CAGCTCAGTAATGCCCAGCTGCATAGCTTGGCTGCCGTCCAGCAGGCTACAATTGCTGCC
AGTCGGCAGGCCAGCTCCCCAAACACCAGCACTAGCCAGCAGCAGCCTGCCAGTACCCAG
GCCTCTATCAATCTGGCCACTACGTCGGCCGCCCAGCTCATCAGCCGATCCCAGAGTGTG
AGCTCTCCCAGTGCCACTACCTTGACCCAATCTGTGCTACTGGGGAACACCACCTCTCCA
CCCCTCAACCAGTCCCAGGCCCAGATGTATCTACGGCCACAGCTGGGAAACCTATTGCAG
GTAAACCGAACCCTGGGCCGGAATGTGCCTCTAGCCTCCCAACTCATTCTGATGCCCAAT
GGGGCAGTGGCTGCAGTCCAGCAGGAGGTACCATCTGCTCAGTCTCCTGGAGTTCATGCA
GATGCAGATCAGGTGCAGAACTTGGCAGTGAGGAACCAACAAGCCTCAGCCCAAGGGTCC
CAAATGCAAGGCTCCACTCAGAAGGCCATTCCTCCTGGAGCCTCTCCTGTCTCTAGCCTC
TCCCAGGCCTCTAACCAGACTCTAGCTGTGGCACAGGCCTCTTCTGGGGCCTCAGGCCAA
TCTCTCAACCTTAGTCAAGCTGGTGGAGGCAGTGGGAATAGCATCCCAGGGTCCCTGGGT
TCAGGTGGAGGTGGCCAGGCCCCTGGGGGTTTGGGTCAATTGCCTTCCTCAGGAATGGGT
GGTGGGAGCTGTCCTAGGAAGGGTACAGGAGTAGTGCAGCCCCTGCCTGCAGCCCAAACA
GTGACTGTGAGCCAGGGCAGCCAGACAGAGGCAGATAGTGCAGCAGCCAAGAAGGCAGAA
GCAGATGGGACTGGTCAGCAGAACGTGGGCATGAACCTGACACGGACAGCCACACCTGCG
CCCAGCCAGACGCTTATTAGCTCAGCCACCTACACACAGATCCAGCCCCATTCATTGATA
CAGCAACAGCAACAGATCCACCTCCAGCAGAAACAGGTGGTGATTCAGCAGCAGATTGCC
ATCCACCACCAACAGCAGTTCCAGCACCGTCAATCTCAGCTCCTCCACACAGCTACACAC
CTCCAGTTGGCCCAGCAGCAGCAACAGCAACAACAGCAGCAGCAGCAACAGCAGCAGCAG
CAGCAGCAAGCCACAACCCTTACTGCCCCTCAGCCATCACAAGTCCCACCTACTCAGCAA
GTCCCACCCTCCCAGTCCCAGCAGCAAGCCCAAACCCTGGTGGTTCAACCCATGCTTCAG
TCTTCACCCTTGTCCCTTCCACCTGACCCAACCCCCAAGCCACCAATCCCTATTCAATCC
AAACCATCTGTAGCACCCATCAAGCCTCCTCAATTAGGAGCTGCTAAAATGTCAGCTACC
CAGCAACCACCACCCCACATCCCTGTGCAAGTTGTAGGTACCCGACAGCCAGGTGCAGCT
CAGGCACAGGCTTTGGGGTTGGCACAGCTGGCAGCTGCTGTTCCTACTTCTCGAGGGATG
CCAGGTTCAGTGCAGCCTGGCCAGGCCCATTTGGCTTCCTCCCCACCTTCATCCCAGGCT
CCTGGTGCTCTGCAGGAGTGCCCTCCTGCATTGGCCCCTGGGATGACCCTTGCTCCTGTG

CAGGGGACAGCACATGTAGTAAAGAGTGGAACTACCACCTCCTCACCAGTTGTAGCCCAG
GTCCCTGCTGCCTTCTACATGCAGTCTGTGCATTTGCCGGGTAAACCCCAGACATTGGCT
GTCAAACGTAAAGCTGAGTCTGAGGAGGACAGAGATGAGGTCTCTACATTGAGTTCAATA
CTTCCCGCCAAGGCATCTCCAGTAGCGGAGAGCCCAAAGGTCATGGATGAGAAAAGCAGT
CTTGGAGACAAAGCTGAACCAGTGGCCAGTGTGAATACTAATACCCCAAGCAGTGAACTA
GTAGCCTTGACCCCTGCCCCGTCAGCTCCTACACTAGCCTTGGTGTCCAGACAAATGGGT
GACTCAAAACCCCCACAGGCCATTGTGAAGCCCCAGATTCTCACCCACATCATTGAAGGC
TTTGTTATCCAGGAAGGAGCAGAACCTTTCCCGGTCGGGTGTTCTCAGTTACTGAAGGAG
TCTGAGAAGCCACTACAGGCTGGCCTTCCAACAGGGCTGAATGAGAATCAATCAGGTGGC
CCCTTGGGGGGGGACAGCCCATCTGCTGAGTTAGAGAAGAAGGCGAATCTCCTCAAGTGC
GAGTACTGTGGGAAGTATGCCCCTGCAGAGCAGTTTCGTGGTTCCAAGAGGTTCTGCTCC
ATGACTTGTGCTAAGAGGTACAATGTGAGTTGTAGCCATCAGTTCCGGCTGAAGAGGAAA
AAAATGAAAGAGTTTCAAGAAGCCAACTATGCTCGTGTTCGCAGGCGTGGACCCCGCCGC
AGCTCCTCTGACATTGCCCGTGCTAAGATTCAGGGCAAACGCCACCGGGGTCAAGAGGAC
TCTAGCCGGGGTTCAGATAATTCCAGTTATGATGAAGCACTCTCTCCAACATCTCCTGGG
CCTTTATCAGTAAGAGCTGGGCATGGAGACCGTGACCTGGGTAATCCCAATATGGCTCCA
CCTACACCGGAATTGCACGGCATCAACCCTGTGTTCCTGTCCAGTAATCCCAGCCGTTGG
AGTGTAGAGGAGGTGTATGAGTTTATTGCGTCTCTTCAAGGTACTGAGGGTCTCCAACAG
AACCTTAGAACCCAGGAGATTGATGGACAGGCCCTTTTATTACTTAAAGAAGAACATCTT
ATGAGTGCCATGAACATCAAACTGGGCCCTGCCCTCAAGATCTGCGCCAAGATAAATGTC
CTAAAGGAGACC
>chimpanzee ENSPTRT00000057571.2
ATGGAGACTGAGAGCGAGCAGAACTCCAATTCCACCAATGGGAGTTCCAGCTCAGGGGGC
AGCTCTCGGCCCCAGATAGCTCAAATGTCACTATATGAACGACAAGCAGTGCAGGCTCTG
CAAGCACTGCAGCGGCAGCCCAATGCAGCTCAGTATTTCCACCAGTTCATGCTCCAGCAG
CAGCTCAGTAATGCCCAGCTGCATAGCCTGGCTGCCGTCCAGCAGGCCACAATTGCTGCC
AGTCGGCAGGCCAGCTCCCCAAACACCAGCACTACACAGCAGCAGACTACCACCACCCAG
GCCTCGATGAATCTGGCCACCACATCGGCCGCCCAGCTCATCAGCCGATCCCAGAGTGTG
AGCTCTCCCAGTGCTACCACCTTGACCCAATCTGTGCTACTGGGGAACACCACCTCCCCA
CCCCTCAACCAGTCTCAGGCCCAGATGTATCTACGGCCACAGCTGGGAAACCTATTGCAG
GTAAACCGAACCCTGGGTCGGAATGTGCCTCTAGCCTCCCAACTCATCCTGATGCCTAAT
GGGGCAGTGGCTGCAGTCCAGCAGGAGGTGCCATCTGCTCAGTCTCCTGGAGTTCATGCA
GATGCAGATCAGGTTCAGAACTTGGCAGTAAGGAATCAACAGGCCTCAGCTCAAGGACCT
CAGATGCAAGGCTCCACTCAGAAGGCCATTCCTCCAGGAGCCTCCCCTGTCTCTAGCCTC
TCCCAGGCCTCTAGCCAGGCCCTAGCGGTGGCACAGGCTTCCTCTGGGGCCACAAACCAG
TCCCTCAACCTTAGTCAAGCAGGTGGAGGCAGTGGGAATAGCATCCCGGGGTCCATGGGT
CCAGGTGGAGGTGGCCAGGCACATGGTGGTTTGGGTCAGTTGCCTTCCTCAGGAATGGGT
GGTGGGAGCTGTCCCAGGAAGGGTACAGGAGTGGTGCAGCCCTTGCCTGCAGCCCAAACA
GTGACTGTGAGCCAGGGCAGCCAGACAGAGGCAGAAAGTGCAGCAGCCAAGAAGGCAGAA
GCAGATGGGAGTGGCCAGCAGAATGTGGGCATGAACCTGACACGGACAGCCACACCTGCG
CCCAGCCAGACACTTATTAGCTCAGCCACCTACACACAGATCCAGCCCCATTCACTGATT
CAGCAACAGCAACAGATCCACCTCCAGCAGAAACAGGTGGTGATCCAGCAGCAGATTGCC
ATCCACCACCAGCAGCAGTTCCAGCACCGGCAGTCCCAGCTCCTTCATACAGCTACACAC
CTCCAGTTGGCGCAGCAGCAGCAGCAGCAGCAACAACAGCAACAGCAGCAACAGCAGCAG
CCGCAAGCCACCACCCTCACTGCCCCTCAGCCACCACAGGTCCCACCTACTCAGCAGGTC
CCACCTTCCCAGTCCCAGCAGCAAGCCCAAACCCTGGTCGTTCAGCCCATGCTTCAGTCT
TCACCCTTGTCTCTTCCACCTGATGCAGCCCCTAAGCCACCAATTCCCATCCAATCCAAA

CCACCTGTAGCACCTATCAAGCCACCTCAGTTAGGGGCTGCTAAGATGTCAGCTACCCAG
CAACCACCACCCCATATCCCTGTGCAAGTTGTAGGCACTCGACAGCCAGGTACAGCCCAG
GCACAGGCTTTGGGGTTGGCACAGCTGGCAGCTGCTGTACCTACTTCCCGGGGGATGCCA
GGTACAGTGCAGTCTGGTCAGGCCCATTTGGCCTCCTCGCCACCTTCATCGCAGGCTCCT
GGTGCACTGCAGGAGTGCCCTCCCACATTGGCCCCTGGGATGACCCTTGCTCCTGTGCAG
GGGACAGCACATGTGGTAAAGGGTGGGGCTACCACCTCCTCACCTGTTGTAGCCCAGGTC
CCTGCTGCCTTCTATATGCAGTCTGTGCACTTGCCGGGTAAACCCCAGACATTGGCTGTC
AAACGCAAGGCTGACTCTGAGGATGAGAGAGATGATGTCTCCACATTGGGTTCAATGCTT
CCTGCCAAGGCATCTCCAGTAGCAGAAAGCCCAAAAGTCATGGACGAGAAGAGCAGTCTT
GGAGAAAAAGCTGAATCCGTGGCTAATGTGAATGCTAATACTCCAAGCAGTGAACTAGTA
GCCTTGACCCCCGCCCCTTCAGTACCGCCTCCTACACTAGCCATGGTGTCTAGACAAATG
GGTGACTCAAAACCCCCACAGGCCATCGTGAAGCCCCAGATTCTCACCCACATCATTGAA
GGCTTTGTTATCCAGGAAGGAGCAGAACCTTTCCCGGTGGGTTGTTCTCAGTTACTGAAG
GAGTCTGAGAAGCCACTACAGACTGGCCTTCCGACAGGGCTGACTGAGAATCAGTCAGGT
GGCCCTTTGGGAGTGGACAGCCCATCTGCTGAGTTAGATAAGAAGGCGAATCTCCTGAAG
TGCGAGTACTGTGGGAAGTACGCCCCCGCAGAGCAGTTTCGTGGCTCTAAGAGGTTCTGC
TCCATGACTTGCGCTAAGAGGTACAATGTGAGCTGTAGCCATCAGTTCCGGCTGAAGAGG
AAAAAAATGAAAGAGTTTCAAGAAGCCAACTATGCTCGCGTTCGCAGGCGTGGACCCCGC
CGCAGCTCCTCTGACATTGCCCGTGCCAAGATTCAGGGCAAGTGCCACCGGGGTCAAGAA
GACTCTAGCCGGGGTTCAGATAATTCCAGTTATGATGAAGCACTCTCTCCAACATCTCCT
GGGCCTTTATCAGTAAGAGCTGGGCATGGAGAACGTGACCTGGGGAATCCCAATACAGCT
CCACCTACACCGGAATTACATGGCATCAACCCTGTGTTCCTGTCCAGTAATCCCAGCCGT
TGGAGTGTAGAGGAGGTGTACGAGTTTATTGCTTCTCTCCAAGGCTGCCAAGAGATTGCA
GAGGAATTTCGCTCACAGGAGATTGATGGACAGGCCCTTTTATTACTTAAGGAAGAACAT
CTTATGAGTGCCATGAACATCAAGCTGGGCCCTGCCCTCAAGATCTGCGCCAAGATAAAT
GTCCTCAAGGAGACC
>olive baboon ENSPANT00000007068.2
ATGGAGACTGAG
AGCGAGCAGAACTCCAACTCCACCAATGGGAGTTCCAGCTCAGGGGGCAGCTCTCGGCCC
CAGATAGCTCAAATGTCACTGTATGAGCGACAAGCAGTGCAGGCTCTGCAGGCACTGCAG
CGGCAGCCCAATGCAGCTCAGTATTTCCACCAGTTCATGCTCCAGCAGCAACTCAGTAAT
GCCCAGCTGCATAGCCTGGCTGCTGTCCAGCAGGCCACAATTGCTGCCAGTCGGCAGGCC
AGCTCCCCAAACACCAGCACTACACAGCAGCAGACTACCACCACCCAGGCCTCGATCAAT
CTGGCCACCACATCGGCCGCCCAGCTCATCAGCCGATCCCAGAGTGTGAGCTCTCCCAGT
GCTACCACCTTGACCCAATCTGTGCTACTGGGGAACACCACCTCCCCACCCCTCAACCAG
TCGCAGGCCCAGATGTATCTACGGCCACAGCTGGGAAACCTATTGCAGGTAAACCGAACT
CTGGGTCGGAATGTGCCTCTAGCTTCCCAACTCATCCTGATGCCTAATGGGGCAGTGGCT
GCAGTCCAGCAGGAGGTGCCATCTGCTCAGTCTCCTGGAGTTCATGCAGATGCAGATCAG
GTGCAGAACTTGGCAGTAAGGAATCAACAGGCCTCAGCTCAAGGACCTCCAATGCAAGGC
TCCACTCAGAAGGCCATTCCTCCGGGAGCTTCCCCTGTCTCTAGCCTCTCCCAGGCCTCT
AGCCAGGCCCTAGCGGTGGCACAGGCTTCCTCTGGGGCCACAAGCCAGTCCCTCAACCTT
AGTCAAGCTGGTGGAGGCAGTGGGAATAGCATCCCAGGGTCCATGGGTCCAGGTGGTGGT
GGCCAGGCACATGGTGGTTTGGGCCAGTTGCCTTCCTCAGGAGTGGGTGGTGGGAGCTGT
CCCAGGAAGGGCACAGGAGTGGTGCAGCCCTTGCCTGCAGCCCAAACAGTGACTGTGAGC
CAGGGCAGCCAGACAGAGGCAGAAAGTGCAGCAGCCAAGAAGGCAGAAGCAGATGGGAGC
GGTCAGCAGAATGTGGGTATGAACCTGACACGGACAGCCACACCTGCGCCCAGCCAGACA
CTTATTAGCTCAGCCACCTACACACAAATCCAGCCCCATTCACTGATTCAGCAACAGCAA

CAGATCCACCTCCAGCAGAAACAGGTGGTGATCCAGCAGCAGATTGCCATCCACCACCAG
CAGCAGTTCCAGCATCGTCAGTCTCAGCTACTTCACACAGCTACACACCTCCAGTTGGCG
CAGCAGCAGCAGCAACAACAGCAGCAGCAGCAACAGCAGCAGCCGCAAGCCACCACTCTC
ACTGCCCCTCAGCCACCACAGGTCCCACCTACTCAGCAGGTCCCACCTTCACAGTCCCAG
CAGCAAGCCCAAACCCTGGTCGTTCAGCCCATGCTTCAGTCTTCACCCTTGTCTCTTCCA
CCTGATGCAGCCCCTAAGCCACCAATTCCCATCCAATCCAAACCACCTGTAGCACCTATC
AAGCCACCTCAGTTAGGGACTGCTAAGATGTCAGCTACCCAGCAACCACCACCCCATATC
CCAGTGCAAGTTGTAGGCACTCGACAGCCAGGTACAGCCCAGGCACAGGCTTTGGGGTTG
GCACAGCTGGCAGCTGCTGTACCTACTTCCCGGGGGATGCCAGGTCCAGTGCAGTCTGGT
CAGGCCCATTTGGCCTCCTCGCCACCTTCATCCCAGGCTCCTGGTGCACTGCAGGAGTGC
CCTCCCACATTGGCCCCTGGGATGACCCTTGCTCCTGTGCAGGGGACAGCACATGTGGTG
AAGGGTGGGGCTACCACCTCCTCACCTGTTGTAGCCCAGGTCCCTGCTGCCTTCTATATG
CAGTCTGTGCACTTGCCGGGTAAACCCCAAACATTGCCTGTCAAACGCAAGGCTGACTCT
GAGGAGGAGAGAGATGATGTCTCCACGTTGGGTTCAATGCTTCCTGCCAAGGCATCTCCA
GTAGCAGAGAGCCCAAAAGTCATGGATGAGAAGAGCAGTCTTGGAGAAAAAGCTGAATCA
GTGGCTAATATGAATGCTAATACCCCAGGCAGTGAACTAGTAGCCTTGACCCCTGCCCCA
TCAGTACCGCCTCCTACACTAGCCATGGTGTCTAGACAGATGGGTGACTCAAAACCCCCA
CAGGCCATCGTGAAGCCCCAGATTCTCACCCACATCATTGAAGGCTTTGTTATCCAGGAA
GGAGCAGAACCTTTCCCGGTGGGTTGTTCTCAGTTACTGAAGGAGTCTGAGAAGCCACTA
CAGACTGGCCTTCCGACAGGGCTGACTGAGAATCAGTCAGGTGGCCCTTTGGGAGTAGAC
AGCCCCTCTGCCGAGTTAGATAAGAAGGCGAATCTCCTGAAGTGCGAGTACTGTGGGAAG
TACGCCCCCGCAGAGCAGTTTCGTGGCTCTAAGAGGTTCTGCTCCATGACTTGCGCTAAG
AGGTACAATGTGAGCTGTAGCCATCAGTTCCGGCTGAAGAGGAAAAAAATGAAAGAGTTT
CAAGAAGCCAACTACGCTCGCGTTCGCAGGCGTGGACCCCGCCGCAGCTCCTCTGACATT
GCCCGTGCCAAGATTCAGGGCAAGTGCCACCGGGGTCAAGAAGACTCTAGCCGAGGTTCA
GATAATTCCAGTTATGATGAAGCACTCTCTCCAACATCTCCTGGGCCTTTATCAGTAAGA
GCTGCGCATGGAGAACGTGATCTGGGGAATCCCAATACAGCTCCACCTACACCGGAGTTA
CATGGCATCAACCCTGTGTTCCTGTCCAGTAATCCCAGCCGTTGGAGTGTAGAGGAGGTG
TACGAGTTTATTGCTTCTCTCCAAGGCTGCCAAGAGATTGCGGAGGAATTTCGCTCACAG
GAGATTGATGGACAGGCCCTTTTATTACTTAAAGAAGAACATCTTATGAGTGCCATGAAC
ATCAAGCTGGGCCCTGCCCTCAAGATCTGCGCCAAGATAAATGTCCTCAAGGAGACC
>orangutan ENSPPYT00000005033.2
ATGGAGACTGAG
AGCGAGCAGAACTCCAACTCCACCAATGGGAGTTCCAGCTCAGGGGGCAGCTCTCGGCCC
CAGATAGCTCAAATGTCACTATATGAACGACAAGCAGTGCAGGCTCTGCAGGCACTGCAG
CGGCAGCCCAATGCAGCTCAGTATTTCCACCAGTTCATGCTCCAGCAGCAGCTCAGTAAT
GCCCAGCTGCATAGCCTGGCTGCTGTCCAGCAGGCCACAATTGCTGCCAGTCGGCAGGCC
AGCTCCCCAAACACCAGCACTACACAGCAGCAGACTACCACCACCCAGGCCTCGATCAAT
CTGGCCACCACGTCGGCCGCCCAGCTCATCAGCCGATCCCAGAGTGTGAGCTCTCCCAGT
GCCACCACCTTGACCCAATCTGTGCTACTGGGGAACACCACCTCCCCACCCCTCAACCAG
TCTCAGGCCCAGATGTATCTACGGCCACAGCTGGGAAACCTATTGCAGGTAAACCGAACC
CTGGGTCGGAATGTGCCTCTAGCCTCCCAACTCATCCTGATGCCTAATGGGGCAGTGGCT
GCAGTCCAGCAGGAGGTGCCATCTGCTCAGTCCCCTGGAGTTCATGCAGATGCAGATCAG
GTGCAGAACTTGGCAGTAAGGAATCAACAGGCCTCAGCTCAAGGACCTCAGATGCAAGGC
TCCACTCAGAAGGCCATTCCTCCAGGAGCCTCCCCTGTCTCTAGCCTCTCCCAGGCCTCT
AGCCAGGCCCTAGCAGTGGCACAGGCTTCCTCTGGGGCCACAAACCAGTCCCTCAACCTT
AGTCAAGCTGGTGGAGGCAGCGGGAATAGCATCCCAGGGTCCATGGGTCCAGGTGGAGGT

GGCCAGGCACATGGTGGTTTGGGTCAGTTGCCTTCCTCAGGAATGGGTGGTGGGAGCTGT
CCCAGGAAGGGTACAGGAGTGGTGCAGCCCTTGCCTGCAGCCCAAACAGTGACTGTGAGC
CAGGGCAGCCAGACAGAGGCAGAAAGTGCAGCAGCCAAGAAGGCAGAAGCAGATGGGAGT
GGCCAGCAGAATGTGGGCATGAACCTGACACGGACAGCCACACCTGCGCCGAGCCAGACA
CTTATTAGCTCAGCCACCTACACACAGATCCAGCCCCATTCACTGATTCAGCAACAGCAA
CAGATCCACCTCCAGCAGAAACAGGTGGTGATCCAGCAGCAGATTGCCATCCACCACCAG
CAGCAGTTCCAGCACCGGCAGTCCCAGCTCCTTCACACAGCTACACACCTCCAGTTGGCG
CAGCAGCAGCAGCAGCAGCAACAGCAACAGCAGCAACAGCAGCAGCCGCAAGCCACCACC
CTCACTGCCCCTCAGCCACCACAGGTCCCACCTACTCAGCAGGTCCCACCTTCCCAGTCC
CAGCAGCAAGCCCAAACCCTGGTCGTTCAGCCCATGCTTCAGTCTTCACCCTTGTCTCTT
CCACCTGATGCAGCCCCTAAGCCACCAATTCCCATCCAGTCCAAGCCACCTGTAGCACCT
ATCAAGCCACCTCAGTTAGGGGCTGCTAAGATGTCAGCTACCCAGCAACCACCACCCCAT
ATCCCTGTGCAAGTTGTAGGCACTCGACAGCCAGGTACAGCCCAGGCACAGGCTTTGGGG
TTGGCACAGCTGGCAGCTGCTGTACCTACTTCCCGGGGGATGCCAGGTACAGTGCAGTCT
GGTCAGGCCCATTTGGCCTCCTCGCCACCTTCATCCCAGGCTCCTGGTGCACTGCAGGAG
TGCCCTTCCACATTGGCCCCTGGGATGACCCTTGCTCCTGTGCAGGGGACAGCACATGTG
GTAAAGGGTGGGGCTACCACGTCCTCACCTGTTGTAGCCCAGGTCCCTGCTGCCTTCTAT
ATGCAGTCTGTGCACTTGCCGGGTAAACCCCAGACATTGGCTGTCAAACGCAAGGCTGAC
TCTGAGGAGGAGAGAGATGATGTCTCCACATTGGGTTCAATGCTTCCTGCCAAGGCATCT
CCAGTAGCAGAAAGCCCAAAAGTCATGGATGAGAAGAGCAGTCTTGGAGAAAAAGCCGAA
TCAGTGGCTAATGTGAATGCTAATACCCCAAGCAGTGAACTAGTAGCCTTGACCCCCGCC
CCATCAGTACCGCCTCCTACACTAGCCATGGTGTCTAGACAAATGGGTGACTCAAAACCC
CCACAGGCCATCGTGAAGCCCCAGATTCTCACCCACATCATTGAAGGCTTTGTTATCCAG
GAAGGAGCAGAACCTTTCCCGGTGGGTTGTTCTCAGTTACTGAAGGAGTCTGAGAAGCCA
CTACAGACTGGCCTTCCGACAGGGCTGACTGAGAATCAGTCAGGTGGCCCTTTGGGAGTG
GACAGCCCATCTGCTGAGTTAGATAAGAAGGCGAATCTCCTGAAGTGCGAGTACTGTGGG
AAGTACGCCCCCGCAGAGCAGTTTCGTGGCTCTAAGAGGTTCTGCTCCATGACTTGCGCT
AAGAGGTACAATGTGAGCTGTAGCCATCAGTTCCGGCTGAAGAGGAAAAAAATGAAAGAG
TTTCAAGAAGCCAACTATGCTCGCGTTCGCAGGCGTGGACCCCGCCGCAGCTCCTCTGAC
ATTGCCCGTGCCAAGATTCAGGGCAAGTGCCACCGGGGTCAAGAAGACTCTAGCCGGGGT
TCAGATAATTCCAGTTATGATGAAGCACTCTCTCCAACATCTCCTGGGCCTTTATCAGTA
AGAGCTGGGCATGGAGAACGTGACCTGGGGAATCCCAATACAGCTCCACCTACACCGGAA
TTACATGGCATCAACCCTGTGTTCCTGTCCAGTAATCCCAGCCGTTGGAGTGTAGAGGAG
GTGTACGAGTTTATTGCTTCTCTCCAAGGCTGCCAAGAGATTGCAGAGGAATTTCGCTCA
CAGGAGATTGATGGACAGGCCCTTTTATTACTTAAAGAAGAACATCTTATGAGTGCCATG
AACATCAAGCTGGGCCCTGCCCTCAAGATCTGCGCCAAGATAAATGTCCTCAAGGAGACC

>bonobo XM_003804529.3
ATGGAGACTGAGAGCGAGCAGAACTCCAATTCCACCAATG
GGAGTTCCAGCTCAGGGGGCAGCTCTCGGCCCCAGATAGCTCAAATGTCACTATATGAACGACAAGCAGT
GCAGGCTCTGCAAGCACTGCAGCGGCAGCCCAATGCAGCTCAGTATTTCCACCAGTTCATGCTCCAGCAG
CAGCTCAGTAATGCCCAGCTGCATAGCCTGGCTGCCGTCCAGCAGGCCACAATTGCTGCCAGTCGGCAGG
CCAGCTCCCCAAACACCAGCACTACACAGCAGCAGACTACCACCACCCAGGCCTCGATCAATCTGGCCAC
CACATCGGCCGCCCAGCTCATCAGCCGATCCCAGAGTGTGAGCTCTCCCAGTGCTACCACCTTGACCCAA
TCTGTGCTACTGGGGAACACCACCTCCCCACCCCTCAACCAGTCTCAGGCCCAGATGTATCTACGGCCAC
AGCTGGGAAACCTATTGCAGGTAAACCGAACCCTGGGTCGGAATGTGCCTCTAGCCTCCCAACTCATCCT
GATGCCTAATGGGGCAGTGGCTGCAGTCCAGCAGGAGGTGCCATCTGCTCAGTCTCCTGGAGTTCATGCA

GATGCAGATCAGGTTCAGAACTTGGCAGTAAGGAATCAACAGGCCTCAGCTCAAGGACCTCAGATGCAAG
GCTCCACTCAGAAGGCCATTCCTCCAGGAGCCTCCCCTGTCTCTAGCCTCTCCCAGGCCTCTAGCCAGGC
CCTAGCGGTGGCACAGGCTTCCTCTGGGGCCACAAACCAGTCCCTCAACCTTAGTCAAGCAGGTGGAGGC
AGTGGGAATAGCATCCCGGGGTCCATGGGTCCAGGTGGAGGTGGCCAGGCACATGGTGGTTTGGGTCAGT
TGCCTTCCTCAGGAATGGGTGGTGGGAGCTGTCCCAGGAAGGGTACAGGAGTGGTGCAGCCCTTGCCTGC
AGCCCAAACAGTGACTGTGAGCCAGGGCAGCCAGACAGAGGCAGAAAGTGCAGCAGCCAAGAAGGCAGAA
GCAGATGGGAGTGGCCAGCAGAATGTGGGCATGAACCTGACACGGACAGCCACACCTGCGCCCAGCCAGA
CACTTATTAGCTCAGCCACCTACACACAGATCCAGCCCCATTCACTGATTCAGCAACAGCAACAGATCCA
CCTCCAGCAGAAACAGGTGGTGATCCAGCAGCAGATTGCCATCCACCACCAGCAGCAGTTCCAGCACCGG
CAGTCCCAGCTCCTTCATACAGCTACACACCTCCAGTTGGCGCAGCAGCAGCAGCAGCAGCAACAACAGC
AACAGCAGCAACAGCAGCAGCCGCAAGCCACCACCCTCACTGCCCCTCAGCCACCACAGGTCCCACCTAC
TCAGCAGGTCCCACCTTCCCAGTCCCAGCAGCAAGCCCAAACCCTGGTCGTTCAGCCCATGCTTCAGTCT
TCACCCTTGTCTCTTCCACCTGATGCAGCCCCTAAGCCACCAATTCCCATCCAATCCAAACCACCTGTAG
CACCTATCAAGCCACCTCAGTTAGGGGCTGCTAAGATGTCAGCTACCCAGCAACCACCACCCCATATCCC
TGTGCAAGTTGTAGGCACTCGACAGCCAGGTACAGCCCAGGCACAGGCTTTGGGGTTGGCACAGCTGGCA
GCTGCTGTACCTACTTCCCGGGGGATGCCAGGTACAGTGCAGTCTGGTCAGGCCCATTTGGCCTCCTCGC
CACCTTCATCCCAGGCTCCTGGTGCACTGCAGGAGTGCCCTCCCACATTGGCCCCTGGGATGACCCTTGC
TCCTGTGCAGGGGACAGCACATGTGGTAAAGGGTGGGGCTACCACCTCCTCACCTGTTGTAGCCCAGGTC
CCTGCTGCCTTCTATATGCAGTCTGTGCACTTGCCGGGTAAACCCCAGACATTGGCTGTCAAACGCAAGG
CTGACTCTGAGGAGGAGAGAGATGATGTTTCCACATTGGGTTCAATGCTTCCTGCCAAGGCATCTCCAGT
AGCAGAAAGCCCAAAAGTCATGGACGAGAAGAGCAGTCTTGGAGAAAAAGCTGAATCCGTGGCTAATGTG
AATGCTAATACTCCAAGCAGTGAACTAGTAGCCTTGACCCCCGCCCCTTCAGTACCGCCTCCTACACTAG
CCATGGTGTCTAGACAAATGGGTGACTCAAAACCCCCACAGGCCATCGTGAAGCCCCAGATTCTCACCCA
CATCATTGAAGGCTTTGTTATCCAGGAAGGAGCAGAACCTTTCCCGGTGGGTTGTTCTCAGTTACTGAAG
GAGTCTGAGAAGCCACTACAGACTGGCCTTCCGACAGGGCTGACTGAGAATCAGTCAGGTGGCCCTTTGG
GAGTGGACAGCCCATCTGCTGAGTTAGATAAGAAGGCGAATCTCCTGAAGTGCGAGTACTGTGGGAAGTA
CGCCCCCGCAGAGCAGTTTCGTGGCTCTAAGAGGTTCTGCTCCATGACTTGCGCTAAGAGGTACAATGTG
AGCTGTAGCCATCAGTTCCGGCTGAAGAGGAAAAAAATGAAAGAGTTTCAAGAAGCCAACTATGCTCGCG
TTCGCAGGCGTGGACCCCGCCGCAGCTCCTCTGACATTGCCCGTGCCAAGATTCAGGGCAAGTGCCACCG
GGGTCAAGAAGACTCTAGCCGGGGTTCAGATAATTCCAGTTATGATGAAGCACTCTCTCCAACATCTCCT
GGGCCTTTATCAGTAAGAGCTGGGCATGGAGAACGTGACCTGGGGAATCCCAATACAGCTCCACCTACAC
CGGAATTACATGGCATCAACCCTGTGTTCCTGTCCAGTAATCCCAGCCGTTGGAGTGTAGAGGAGGTGTA
CGAGTTTATTGCTTCTCTCCAAGGCTGCCAAGAGATTGCAGAGGAATTTCGCTCACAGGAGATTGATGGA
CAGGCCCTTTTATTACTTAAGGAAGAACATCTTATGAGTGCCATGAACATCAAGCTGGGCCCTGCCCTCA
AGATCTGCGCCAAGATAAATGTCCTCAAGGAGACC

>Cercocebus atys XM_012064712.1
ATGGAGACTGAGAGCGAGCAGAACTCCAACTCCAC
CAATGGGAGTTCCAGCTCAGGGGGCAGCTCTCGGCCCCAGATAGCTCAAATGTCACTGTATGAGCGACAA
GCAGTGCAGGCTCTGCAGGCACTGCAGCGGCAGCCCAATGCAGCTCAGTATTTCCACCAGTTCATGCTCC
AGCAGCAACTCAGTAATGCCCAGCTGCATAGCCTGGCTGCTGTCCAGCAGGCCACAATTGCTGCCAGTCG
GCAGGCCAGCTCCCCAAACACCAGCACTACACAGCAGCAGACTACCACCACCCAGGCCTCGATCAATCTG
GCCACCACATCGGCCGCCCAGCTCATCAGCCGATCCCAGAGTGTGAGCTCTCCCAGTGCTACCACCTTGA
CCCAATCTGTGCTACTGGGGAACACCACCTCCCCACCCCTCAACCAGTCGCAGGCCCAGATGTATCTACG
GCCACAGCTGGGAAACCTATTGCAGGTAAACCGAACTCTGGGTCGGAATGTGCCTCTAGCTTCCCAACTC
ATCCTGATGCCTAATGGGGCAGTGGCTGCAGTCCAGCAGGAGGTGCCATCTGCTCAGTCTCCTGGAGTTC
ATGCAGATGCAGATCAGGTGCAGAACTTGGCAGTAAGGAATCAACAGGCCTCAGCTCAAGGACCTCCAAT

GCAAGGCTCTACTCAGAAGGCCATTCCACCGGGAGCCTCCCCTGTCTCTAGCCTCTCCCAGGCCTCTAGC
CAGGCCCTAGCGGTGGCACAGGCTTCCTCTGGGGCCACAAGCCAGTCCCTCAACCTTAGTCAAGCTGGTG
GAGGCAGTGGGAATAGCATCCCAGGGTCCATGGGTCCAGGTGGAGGTGGCCAGGCACATGGTGGTTTGGG
CCAGTTGCCTTCCTCAGGAGTGGGTGCTGGTAGCTGTCCCAGGAAGGGCACAGGAGTGGTGCAGCCCTTG
CCTGCAGCCCAAACAGTGACTGTGAGCCAGGGCAGCCAGACAGAGGCAGAAAGTGCAGCAGCCAAGAAGG
CAGAAGCAGATGGGAGCGGTCAGCAGAATGTGGGTATGAACCTGACACGGACAGCCACACCTGCGCCCAG
CCAGACACTTATTAGCTCAGCCACCTACACACAAATCCAGCCCCATTCACTGATTCAGCAACAGCAACAG
ATCCACCTCCAGCAGAAACAGGTGGTGATCCAGCAGCAGATTGCCATCCACCACCAGCAGCAGTTCCAGC
ATCGTCAGTCTCAGCTACTTCACACAGCTACACACCTCCAGTTGGCGCAGCAGCAGCAGCAACAACAGCA
GCAGCAGCAACAGCAGCAGCCGCAAGCCACCACTCTCACTGCCCCTCAGCCACCACAGGTCCCACCTACT
CAGCAGGTCCCACCTTCGCAGTCCCAGCAGCAAGCCCAAACCCTGGTCGTTCAGCCCATGCTTCAGTCTT
CACCCTTGTCTCTTCCACCTGATGCAGCCCCTAAGCCACCAATTCCCATCCAATCCAAACCACCTGTAGC
ACCTATCAAGCCACCTCAGTTAGGGACTGCTAAGATGTCAGCTACCCAGCAACCACCACCCCATATCCCA
GTGCAAGTTGTAGGCACTCGACAGCCAGGTACAGCCCAGGCACAGGCTTTGGGGTTGGCACAGCTGGCAG
CTGCTGTACCTACTTCCCGGGGGATGCCAGGTCCAGTGCAGTCTGGTCAGGCCCATTTGGCCTCCTCGCC
ACCTTCATCCCAGGCTCCTGGTGCACTGCAGGAGTGCCCTCCCACATTGGCCCCTGGGATGACCCTTGCT
CCTGTGCAGGGGACAGCACATGTGGTGAAGGGTGGGGCTACCACCTCCTCACCTGTTGTAGCCCAGGTCC
CTGCTGCCTTCTATATGCAGTCTGTGCACTTGCCGGGTAAACCCCAAACGTTGCCTGTCAAACGCAAGGC
TGACTCTGAGGAGGAGAGAGATGATGTCTCCACGTTGGGTTCAATGCTTCCTGCCAAGGCATCTCCAGTA
GCAGAGAGCCCAAAAGTCATGGATGAGAAGAGCAGTCTTGGAGAAAAAGCTGAATCAGTGGCTAATATGA
ATGCTAATACCCCAGGCAGTGAACTAGTAGCCTTGACCCCTGCCCCATCAGTACCGCCTCCTACACTAGC
CATGGTGTCTAGACAGATGGGTGACTCAAAACCCCCACAGGCCATCGTGAAGCCCCAGATTCTCACCCAC
ATCATTGAAGGCTTTGTTATCCAGGAAGGAGCAGAACCTTTCCCGGTGGGTTGTTCTCAGTTACTGAAGG
AGTCTGAGAAGCCACTACAGACTGGCCTTCCGACAGGGCTGACTGAGAATCAGTCAGGTGGCCCTTTGGG
AGTAGACAGCCCCTCTGCCGAGTTAGATAAGAAGGCGAATCTCCTGAAGTGCGAGTACTGTGGGAAGTAC
GCCCCCGCAGAGCAGTTTCGTGGCTCTAAGAGGTTCTGCTCCATGACTTGCGCTAAGAGGTACAATGTGA
GCTGTAGCCATCAGTTCCGGCTGAAGAGGAAAAAAATGAAAGAGTTTCAAGAAGCCAACTACGCTCGCGT
TCGCAGGCGTGGACCCCGCCGCAGCTCCTCTGACATTGCCCGTGCCAAGATTCAGGGCAAGTGCCACCGG
GGTCAAGAAGACTCTAGCCGAGGTTCAGATAATTCCAGTTATGATGAAGCACTCTCTCCAACATCTCCTG
GGCCTTTATCAGTAAGAGCTGCGCATGGAGAACGTGATCTGGGGAATCCCAATACAGCTCCACCTACACC
GGAATTACATGGCATCAACCCTGTGTTCCTGTCCAGTAATCCCAGCCGTTGGAGTGTAGAGGAGGTGTAC
GAGTTTATTGCTTCTCTCCAAGGCTGCCAAGAGATTGCGGAGGAATTTCGCTCACAGGAGATTGATGGAC
AGGCCCTTTTATTACTTAAAGAAGAACATCTTATGAGTGCCATGAACATCAAGCTGGGCCCTGCCCTCAA
GATCTGCGCCAAGATAAATGTCCTCAAGGAGACC

>Mandrillus leucophaeus XM_011983295.1
ATGGAGACTG
AGAGCGAGCAGAACTCCAACTCCACCAATGGGAGTTCCAGCTCAGGGGGCAGCTCTCGGCCCCAGATAGC
TCAAATGTCACTGTATGAGCGACAAGCAGTGCAGGCTCTGCAGGCACTGCAACGGCAGCCCAATGCAGCT
CAGTATTTCCACCAGTTCATGCTCCAGCAGCAACTCAGTAATGCCCAGCTGCATAGCCTGGCTGCTGTCC
AGCAGGCCACAATTGCTGCTAGTCGGCAGGCCAGCTCCCCAAACACCAGCACTACACAGCAGCAGACTAC
CACCACCCAGGCCTCGATCAATCTGGCCACCACATCGGCCGCCCAGCTCATCAGCCGATCCCAGAGTGTG
AGCTCTCCCAGTGCTACCACCTTGACCCAATCTGTGCTACTGGGGAACACCACCTCCCCACCCCTCAACC
AGTCGCAGGCCCAGATGTATCTACGGCCACAGCTGGGAAACCTATTGCAGGTAAACCGAACCCTGGGTCG
GAATGTGCCTCTAGCTTCCCAACTCATCCTGATGCCTAATGGGGCAGTGGCTGCAGTCCAGCAGGAGGTG
CCATCTGCTCAGTCTCCTGGAGTTCATGCAGATGCAGATCAGGTGCAGAACTTGGCAGTAAGGAATCAAC
AGGCCTCAGCTCAAGGACCTCCAATGCAAGGCTCCACTCAGAAGGCCATTCCTCCGGGAGCCTCCCCTGT

CTCTAGCCTCTCCCAGGCCTCTAGCCAGGCCCTAGCGGTGGCACAGGCTTCCTCTGGGGCCACAAGCCAG
TCCCTCAACCTTAGTCAAGCTGGTGGAGGCAGTGGGAATAGCATCCCAGGGTCCATGGGTCCAGGTGGAG
GTGGCCAGGCACATGGTGGTTTGGGCCAGTTGCCTTCCTCAGGAGTGGGTGGTGGGAGCTGTCCCAGGAA
GGGCACAGGAGTGGTGCAGCCCTTGCCTGCAGCCCAAACAGTGACTGTGAGCCAGGGCAGCCAGACAGAG
GCAGAAAGTGCAGCAGCCAAGAAGGCAGAAGCAGATGGGAGCGGTCAGCAGAATGTGGGTATGAACCTGA
CACGGACAGCCACACCTGCGCCCAGCCAGACACTTATTAGCTCAGCCACCTACACACAAATCCAGCCCCA
TTCACTGATTCAGCAACAGCAACAGATCCACCTCCAGCAGAAACAGGTGGTGATCCAGCAGCAGATTGCC
ATCCACCACCAGCAGCAGTTCCAGCATCGTCAGTCTCAGCTACTTCACACAGCTACACACCTCCAGTTGG
CGCAGCAGCAGCAGCAACAACAGCAGCAGCAGCAACAGCAGCAGCCGCAAGCCACCACTCTCACTGCCCC
TCAGCCACCACAGGTCCCACCTACTCAGCAGGTCCCACCTTCGCAGTCCCAGCAGCAAGCCCAAACCCTG
GTCGTTCAGCCCATGCTTCAGTCTTCACCCTTGTCTCTTCCACCTGATGCAGCCCCTAAGCCACCAATTC
CCATCCAATCCAAACCACCTGTAGCACCTATCAAGCCACCTCAGTTAGGGACTGCTAAGATGTCAGCTAC
CCAGCAACCACCACCCCATATCCCAGTGCAAGTTGTAGGCACTCGACAGCCAGGTACAGCCCAGGCACAG
GCTTTGGGGTTGGCACAGCTGGCAGCTGCTGTACCTACTTCCCGGGGGATGCCAGGTCCAGTGCAGTCTG
GTCAGGCCCATTTGGCCTCCTCGCCACCTTCATCCCAGGCTCCTGGTGCACTGCAGGAGTGCCCTCCCAC
ATTGGCCCCTGGGATGACCCTTGCTCCTGTGCAGGGGACAGCACATGTGGTGAAGGGTGGGGCTACCACC
TCCTCACCTGTTGTAGCCCAGGTCCCTGCTGCCTTCTATATGCAGTCTGTGCACTTGCCGGGTAAACCCC
AAACATTGCCTGTCAAACGCAAGGCTGACTCTGAGGAGGACAGAGATGATGTCTCCACGTTGGGTTCAAT
GCTTCCTGCCAAGGCATCTCCAGTAGCAGAGAGCCCAAAAGTCATGGATGAGAAGAGCAGTCTTGGAGAA
AAAGCTGAATCAGTGGCTAATATGAATGCTAATACCCCAGGCAGTGAACTAGTAGCCTTGACTCCTGCCC
CATCAGTACCGCCTCCTACACTAGCCATGGTATCTAGACAGATGGGTGACTCAAAACCCCCACAGGCCAT
CGTGAAGCCCCAGATTCTCACCCACATCATTGAAGGCTTTGTTATCCAGGAAGGAGCAGAACCTTTCCCG
GTGGGTTGTTCTCAGTTACTGAAGGAGTCTGAGAAGCCACTACAGACTGGCCTTCCGACAGGGCTGACTG
AGAATCAGTCAGGTGGCCCTTTGGGAGTAGACAGCCCCTCTGCCGAGTTAGATAAGAAGGCGAATCTCCT
GAAGTGCGAGTACTGTGGGAAGTACGCCCCCGCAGAGCAGTTTCGTGGCTCTAAGAGGTTCTGCTCCATG
ACTTGCGCTAAGAGGTACAATGTGAGCTGTAGCCATCAGTTCCGGCTGAAGAGGAAAAAAATGAAAGAGT
TTCAAGAAGCCAACTACGCTCGCGTTCGCAGGCGTGGACCCCGCCGCAGCTCCTCTGACATTGCCCGTGC
CAAGATTCAGGGCAAGTGCCACCGGGGTCAAGAAGACTCTAGCCGAGGTTCAGATAATTCCAGTTATGAT
GAAGCACTCTCTCCAACATCTCCTGGGCCTTTATCAGTAAGAGCTGCGCATGGAGAACGTGATCTGGGGA
ATCCCAATACAGCTCCACCTACACCGGAATTACATGGCATCAACCCTGTGTTCCTGTCCAGTAATCCCAG
CCGTTGGAGTGTAGAGGAGGTGTACGAGTTTATTGCTTCTCTCCAAGGCTGCCAAGAGATTGCGGAGGAA
TTTCGCTCACAGGAGATTGATGGACAGGCCCTTTTATTACTTAAAGAAGAACATCTTATGAGTGCCATGA
ACATCAAGCTGGGCCCTGCCCTCAAGATCTGCGCCAAGATAAATGTCCTCAAGGAGACC

>Cebus capucinus imitator XM_017512331.1
ATGGAGACTGAG
AGCGAGCAGAACTCCAACTCCACCAATGGGAGTTCCAGCTCAGGGGGCAGCTCTCGGCCCCAGATAGCTC
AAATGTCACTATATGAACGACAAGCAGTGCAGGCTCTACAGGCACTGCAGCGGCAGCCCAATGCAGCTCA
GTATTTCCACCAGTTCATGCTCCAGCAGCAGCTCAGTAATGCCCAGCTGCATAGCCTGGCTGCCGTCCAG
CAGGCCACAATTGCTGCCAGTCGGCAGGCCAGCTCCCCAAACACCAGCACTACACAGCAGCAGACTACCA
CTACCCAGGCCTCAATCAATCTGGCCACCACATCGGCCGCCCAGCTCATCAGCCGATCCCAGAGTGTGAG
CTCTCCCAGTGCTACCACCTTGACCCAATCTGTGCTACTGGGGAACACCACCTCCCCACCCCTCAACCAG
TCTCAGGCCCAGATGTATCTACGGCCACAGCTGGGAAACCTATTGCAGGTAAACCGAACCCTGGGTCGGA
ATGTGCCTCTGGCCTCCCAGCTCATCCTGATGCCTAATGGTGCAGTGGCTGCAGTCCAGCAGGAGGTGCC
ATCTGCTCAGTCTCCTGGAGTTCATGCAGATGCAGATCAGGTGCAGAACTTGGCAGTAAGGAATCAACAG
GCCTCAGCCCAAGGACCTCAAATGCAAGGCTCAACTCAGAAGGCCATTCCTCCAGGAGCCTCCCCTGTCT
CTGGCCTCTCCCAGGCCTCTAGCCAGGCCCTGGCTGTGGCACAGGCTTCCTCTGGGGCCTCAAGCCAGTC

CCTTAACCTTAGTCAAGCTGGTGGAGGCAGTGGGAATAGCATCCCAGGGTCCATGGGTCCAGGTGGAGGT
GGCCAGGCACATGGTGGCCTGGGTCAGTTGCCTTCCTCAGGAATGGGTGGTGGGAGCTGTCCCAGGAAGG
GCACAGGAGTAGTGCAGCCCTTGCCTGCAGCCCAAACAGTGACTGTGAGCCAGGGCAGCCAGACAGAGGC
AGAAAGTGCAGCAGCCAAGAAGGCAGAAGCAGATGGTGGCGGGCAGCAGAACGTGGGCATGAACCTGACA
CGGACAGCCACGCCTGCGCCTAGCCAGACACTTATTAGCTCAGCCACCTACACACAGATCCAGCCCCATT
CACTGATTCAGCAACAGCAGCAGATCCACCTCCAGCAGAAACAGGTGGTGATCCAGCAGCAGATTGCCAT
CCACCACCAGCAGCAGTTCCAGCACCGTCAGTCCCAGCTCCTTCACACAGCTACACACCTCCAGTTGGCC
CAGCAGCAGCAGCAGCAGCAGCAGCAGCAGCAGCAGCAGCAGCAGCCACAAGCCACAACCCTCACTGCCCCTC
AGCCACCACAGGTCCCACCCACTCAGCAGGTCCCACCTTCCCAGTCTCAGCAGCAAGCCCAAACCCTGGT
CGTTCAGCCCATGCTTCAGTCTTCACCCTTGTCCCTTCCACCTGATGCAGCTCCTAAGCCACCAATTCCC
ATCCAGTCCAAACCACCTGTAGCACCTATCAAGCCACCTCAGTTAGGGGCTGCCAAGATGTCAGCTACCC
AGCAACCACCACCCCATATCCCTGTGCAAGTTGTAGGCACTCGACAGCCAGGCACAGCCCAGGCACAGGC
TTTGGGACTGGCACAGCTGGCAGCTGCTGTACCCAATTCCCGGGGGATGCCAGGTACAGTGCAGTCTGGT
CAGGCCCATTTGGCCTCCTCGCCGCCCTCATCCCAGGCTCCTGGTGCGCTGCAGGAGTGCCCTCCCACAT
TGGCCCCTGGGATGACCCTTGCTCCTGTGCAGGGGACAGCACATGTAGTAAAGGGTGGGGCTACCACCTC
CTCACCTGTTGTAGCCCAGGTCCCTGCTGCCTTCTATATGCAGTCTGTGCACTTGCCGGGTAAACCCCAG
ACATTGGCTGTCAAACGCAAGGCTGACTCTGAGGAGGAGAGAGATGATGTCTCCACATTGGGTTCAATGT
TTCCTGCCAAGGCATCTCCAGTAGCAGAGAGCCCACAAGTCATGGAGGACAAGAGCACTGTTGGAGAAAA
AGCTGAACCAGTGGCTAATGTGAATGCTAATACCCCAAGCAGTGAACTAGTAGCCTTGACCCCTGCCCCA
TCAGTACCACCTCCTACATTAGCCATGGTGTCTAGACAAATGGGTGACTCAAAACCCCCACAGGCCATCG
TGAAGCCCCAGATTCTCACCCACATCATTGAAGGCTTTGTTATCCAGGAAGGAGCAGAACCTTTCCCGGT
GGGTTGTTCTCAGTTACTAAAGGAGTCTGAAAAGCCACTACAGACTGGCCTTCCGACAGGGCTGACTGAG
AATCAGTCAGGTGGCTCTTTGGGAGTGGACAGCCCATCTGCCGAGTTAGATAAGAAGGCGAATCTCCTGA
AGTGCGAGTACTGTGGGAAGTACGCCCCCGCAGAGCAATTTCGTGGCTCTAAGAGGTTCTGCTCCATGAC
TTGCGCTAAGAGGTACAATGTGAGCTGTAGCCATCAGTTCCGGCTGAAGAGGAAAAAAATGAAAGAGTTT
CAAGAAGCCAACTATGCTCGAGTTCGCAGGCGTGGGCCCCGCCGTAGCTCCTCTGACATTGCCCGTGCCA
AGATTCAGGGCAAGTGCCACCGGGGTCAAGAGGACTCTAGCCGGGGTTCAGATAATTCCAGTTATGATGA
AGCACTCTCTCCAACATCTCCTGGGCCTTTATCAGTAAGAGCCGGGCATGGAGAACGTGACCTGGGGAAC
CCCAATACAGCTCCACCTACACCGGAATTACATGGCATCAACCCTGTGTTCCTGTCTAGTAATCCCAGCC
GTTGGAGTGTAGAAGAGGTGTATGAGTTTATTGCTTCTCTCCAAGGCTGCCAAGAGATTGCCGAGGAATT
TCGTTCCCAGGAGATTGATGGACAGGCCCTTTTATTGCTTAAAGAAGAACATCTAATGAGTGCCATGAAC
ATCAAGCTGGGCCCTGCCCTCAAGATCTGCGCCAAGATAAATGTCCTCAAGGAGACC
>Rhinopithecus roxellana XM_010376708.1
ATGGAGACTGAGAGCGAGCAGAACTCCAACTCCA
CCAATGGGAGTTCCAGCTCAGGGGGCAGCTCTCGGCCCCAGATAGCTCAAATGTCACTATATGAACGACA
AGCAGTGCAGGCTCTGCAGGCACTGCAGCGGCAGCCCAATGCAGCTCAGTATTTCCACCAGTTCATGCTC
CAGCAGCAACTCAGTAATGCCCAGCTGCATAGCCTGGCTGCTGTCCAGCAGGCCACAATTGCTGCCAGTC
GGCAGGCCAGCTCCCCAAACACCAGCACTACACAGCAGCAGACTACCACCACCCAGGCCTCGATCAATCT
GGCCACCACATCGGCCGCCCAGCTCATCAGCCGATCCCAGAGTGTGAGCTCTCCCAGTGCTACCACCTTG
ACCCAATCTGTGCTACTGGGGAACACCACCTCCCCACCCCTCAACCAGTCGCAGGCCCAGATGTATCTAC
GGCCACAGCTGGGAAACCTATTGCAGGTAAACCGAACCCTGGGTCGGAATGTGCCTCTAGCTTCCCAACT
CATCCTGATGCCTAATGGGGCAGTGGCTGCAGTCCAGCAGGAGGTACCATCTGCTCAGTCTCCTGGAGTT
CATGCAGATGCAGATCAGGTGCAGAACTTGGCAGTAAGGAATCAACAGGCCTCAGCTCAAGGACCTCAAA
TGCAAGGCTCCACTCAGAAGGCCATTCCTCCGGGAGCCACCCCTGTCTCTAGCCTCTCTCAGGCCTCTAG
CCAGGCCCTAGCGGTGGCACAGGCTTCCTCTGGGGCCACAAGCCAGTCCCTCAACCTTAGTCAAGCTGGT
GGAGGCAGTGGGAATAGCATCCCAGGGTCCATGGGTCCAGGTGGAGGTGGCCAGGCACATGGTGGTTTGG
GCCAGTTGCCTTCCTCAGGAGTGGGTGGTGGGAGCTGTACCAGGAAGGGCACAGGAGTGGTGCAGCCCTT

GCCTGCAGCCCAAACAGTGACTGTGAGCCAGGGCAGCCAGACAGAGGCAGAAAGTGCAGCAGCCAAGAAG
GCAGAAGCAGATGGGAGCGGTCAGCAGAACGTGGGTATGAACCTGACACGGACAGCCACACCTGCGCCCA
GCCAGACACTTATTAGCTCAGCCACCTACACACAGATCCAGCCCCATTCACTGATTCAGCAACAGCAACA
GATTCACCTCCAGCAGAAACAGGTGGTAATCCAGCAGCAGATTGCCATCCACCACCAGCAGCAGTTCCAG
CATCGTCAGTCTCAGCTCCTTCACACAGCTACACACCTCCAGTTGGCGCAGCAGCAGCAGCAGCAACAAC
AGCAGCAGCAGCAGCAACAGCAGCAGCCGCAAGCCACCACTCTCACTGCCCCTCAGCCACCACAGGTCCC
ACCTACTCAGCAGGTCCCACCTTCCCAGTCCCAGCAGCAAGCCCAAACCCTGGTCGTTCAGCCCATGCTT
CAGTCTTCACCCTTGTCTCTTCCACCTGATGCAGCCCCTAAACCACCAATTCCCATCCAATCCAAACCAC
CTGTAGCACCTATTAAGCCACCTCAGTTAGGGACTGCTAAGATGTCAGCTACCCAGCAACCACCACCCCA
TATCCCAGTGCAAGTTGTAGGCACTCGACAGCCAGGTACAGCCCAGGCACAGGCTTTGGGGTTGGCACAG
CTGGCAGCTGCTGTACCTACTTCCCGGGGGATGCCAGGTCCAGTGCAGTCTGGTCAGGCCCATTTGGCCT
CCTCACCACCTTCATCCCAGGCTCCTAGTGCACTGCAGGAGTGCCCTCCCACATTGGCCCCTGGGATGAC
CCTTGCTCCTGTGCAGGGGACAGCACATGTGGTGAAGGGTGGACTACCACCTCCTCACCTGTTGTAGCC
CAGGTCCCTGCTGCCTTCTATATGCAGTCTGTGCACTTGCCGGGTAAACCCCAAACGTTGCCTGTCAAAC
GCAAGGCTGACTCTGAGGAGGAGAGAGATGATGTCTCCACGTTGGGTTCAATGCTTCCTGCCAAGGCATC
TCCAGTAGCAGAGAGCCCAAAAGTCATGGATGAGAAGAGCAGTCTTGGAGAAAAAGCTGAATCAGTGGCC
AGTATGAATGCTAATACCCCAGGCAGTGAACTAGTAGCCTTGACCCCTGCCCCATCAGTACCACCTCCTA
CACTAGCCATGGTGTCTAGACAGACGGGTGACTCAAAACCCCCACAGGCCATCGTGAAGCCCCAGATTCT
CACCCATATCATTGAAGGCTTTGTTATCCAGGAAGGAGCAGAACCTTTCCCGGTGGGTTGTTCTCAGTTA
CTGAAGGAGTCTGAGAAGCCACTACAGACTGGCCTTCCAACAGGGCTGACTGAGAATCAGTCAGGTGGCT
CTTTGGGAGTAGACAGCCCCTCTGCCGAGTTAGATAAGAAGGCGAATCTCCTGAAGTGCGAGTACTGTGG
GAAGTACGCCCCCGCAGAGCAGTTTCGTGGCTCTAAGAGGTTCTGCTCCATGACTTGCGCTAAGAGGTAC
AATGTGAGCTGTAGCCATCAGTTCCGGCTGAAGAGGAAAAAAATGAAAGAGTTTCAAGAAGCCAACTATG
CTCGCGTTCGCAGGCGTGGGCCCCGCCGCAGCTCCTCTGACATTGCCCGTGCCAAGATTCAGGGCAAGTG
CCACCGGGGTCAAGAAGACTCTAGCCGAGGTTCAGATAATTCCAGTTATGATGAAGCACTCTCTCCAACA
TCTCCTGGGCCTTTATCAGTAAGAGCTGGGCATGGAGAACGCGATCTGGGGAATCCCAATACAGCTCCAC
CTACACCTGAATTACATGGCATCAACCCTGTGTTCCTGTCCAGTAATCCCAGCCGTTGGAGTGTAGAGGA
GGTGTACGAGTTTATTGCTTCTCTCCAAGGCTGCCAAGAGATTGCGGAGGAATTTCGCTCACAGGAGATT
GATGGACAGGCCCTTTTATTACTTAAAGAAGAACATCTTATGAGTGCCATGAACATCAAGCTGGGCCCTG
CCCTCAAGATCTGCGCCAAGATAAATGTCCTCAAGGAGACC
>Aotus nancymaae XM_012458395.1
ATGGAGAC
TGAGAGTGAGCAGAACTCCAACTCCACCAATGGGAGTTCCAGCTCAGGGGGCAGCTCTCGGCCCCAGATA
GCTCAAATGTCACTATATGAACGACAAGCAGTGCAGGCTCTGCAGGCACTGCAGCGGCAGCCCAATGCAG
CTCAGTATTTCCACCAGTTCATGCTCCAGCAGCAGCTCAGTAATGCCCAGCTGCATAGCCTGGCTGCCGT
CCAGCAGGCCACAATTGCTGCCAGTCGGCAGGCCAGCTCCCCAAACACCAGCACTACACAGCAGCAGACT
ACCACTACCCAGGCCTCGATCAATCTGGCCACCACATCGGCCGCCCAGCTCATCAGCCGATCTCAGAGTG
TGAGCTCTCCCAGTGCTACCACCTTGACCCAATCTGTGCTACTGGGGAACACCACCTCCCCACCCCTCAA
CCAGTCTCAGGCCCAGATGTATCTACGGCCACAGCTGGGAAACCTATTGCAGGTAAACCGAACCCTGGGT
CGGAATGTGCCTCTAGCCTCCCAACTCATCCTGATGCCTAATGGTGCAGTGGCTGCAGTCCAGCAGGAGG
TGCCATCTGCTCAGTCTCCTGGAGTTCATGCAGATGCAGATCAGGTGCAGAACTTGGCAGTAAGGAATCA
ACAGGCCTCAGCCCAAGGACCTCAAATGCAAGGCTCCACTCAGAAGGCCATTCCTCCAGGAGCCTCCCCT
GTCTCTAGCCTCTCCCAGGCCTCCAGCCAGGCCCTAACTGTGGCACAGGCTTCCTCTGGGGCCTCAAGCC
AGTCCCTCAACCTTAGTCAAGCTGGTGGAGGCAGTGGGAATAGCATCCCAGGGTCCATGGGTCCAGGTGG
AGGTGGCCAGGCACATGGTGGCTTGGGTCAGTTGCCTTCCTCAGGAATGGGTGGTGGGAGCTGTCCCAGG
AAGGGCACAGGAGTGGTGCAGCCCTTGCCTGCAGCCCAAACAGTGACTGTGAGCCAGGGCAGCCAGACAG
AGGCAGAAAGTGCAGCAGCCAAGAAGGCAGAAGCAGATGGTGGTGGTCAGCAGAATGTGGGCATGAACCT

GACACGGACAGCCACACCTGCACCCAGCCAGACACTTATTAGCTCAGCCACCTACACACAGATCCAGCCC
CATTCACTGATTCAGCAACAGCAACAGATCCACCTCCAGCAGAAACAGGTGGTGATCCAGCAGCAGATTG
CCATCCACCACCAGCAGCAGTTCCAGCACCGTCAGTCCCAGCTCCTTCACACAGCCACACACCTCCAGTT
GGCCCAGCAGCAGCAACAGCAGCAACAGCAGCAACAGCAGCAGCCGCAAGCCACAACCCTCACTGCCCCT
CAGCCGCCACAGGTCCCACCCACTCAGCAGGTCCCACCTTCCCAGTCTCAGCAGCAAGCCCAAACCCTGG
TCGTTCAGCCCATGCTTCAGTCTTCACCCTTGTCCCTTCCACCTGATGCAGCTCCTAAGCCACCAATTCC
CATCCAGTCCAAACCACCTGTAGCACCACTCAAGCCACCTCAGTTAGGGGCTGCTAAGATGTCAGCTACC
CAGCAACCACCACCCCATATCCCTGTGCAAGTCGTAGGCACTCGACAGCCAGGTACAGCCCAGGCGCAGG
CTTTGGGATTGGCACAGCTGGCAGCTGCTGTACCCACTTCCCGGGGGATGCCAGGTACAGTGCAGTCTGG
TCAGGCCCATTTGGCCGCCTCGCCGCCCTCATCCCAGGCTCCTGGTGCGCTGCAGGAGTGCCCTCCCACA
TTGGCCTCTGGGATGACCCTTGCTCCTGTGCAGGGGACAGCACATGTAGTAAAGGGTGGGGCTACCACCT
CCTCACCTGTTGTAGCCCAGGTCCCTGCTGCCTTCTATATGCAGTCTGTGCACTTGCCGGGTAAACCCCA
GACATTGGGTGTCAAACGCAAGGCTGACTCTGAGGAGGAGAGAGATGATGTCTCCACATTGGGTTCAATG
TTTCCTGCCAAGGCATCTCCAGTAGCAGAGAGCCCACAAGTCATGGAGGACAAGAGCAGTCTTGGAGAAA
AAGCTGAACCAGTGGCTAATGTGAATGCTAATACCCCAAGCAGTGAACTAGTAGCCTTGACCCCCACCCC
ATCAGTACCACCTCCTACATTAGCCATGGTGTCTAGACAAATGGGTGACTCAAAACCCCCACAGGCCATC
GTGAAGCCCCAGATTCTCACCCACATCATTGAAGGCTTTGTTATCCAGGAAGGAGCAGAACCTTTCCCGG
TGGGTTGTTCTCAGTTACTAAAGGAGTCTGAAAAGCCACTACAGACTGGCCTTCCGACAGGGCTGACTGA
GAATCAGTCAGGTGGCTCTTTGGGAGTGGACAGCCCATCTGCCGAGTTAGATAAGAAGGCGAATCTCCTG
AAGTGCGAGTACTGTGGGAAGTACGCCCCCGCAGAGCAATTTCGTGGCTCTAAGAGGTTCTGCTCCATGA
CTTGCGCTAAGAGGTACAATGTGAGCTGTAGCCATCAGTTCCGGCTGAAGAGGAAAAAAATGAAAGAGTT
TCAAGAAGCCAACTATGCTCGAGTTCGCAGGCGTGGCCCCGCCGCAGCTCCTCTGACATTGCCCGTGCC
AAGATTCAGGGCAAGTGCCACCGGGGTCAAGAGGACTCTAGCCGGGGTTCAGATAATTCCAGTTATGATG
AAGCACTCTCTCCAACATCTCCTGGGCCTTTATCAGTAAGAGCTGGGCATGGAGAACGTGACCTGGGGAA
CCCCAATACAGCTCCACCTACACCGGAATTACATGGCATCAACCCTGTGTTCCTGTCTAGTAATCCCAGC
CGTTGGAGTGTAGAAGAGGTGTATGAGTTTATTGCTTCTCTCCAAGGCTGCCAAGAGATTGCGGAGGAAT
TTCGTTCCCAGGAGATTGACGGACAGGCCCTTTTATTACTTAAAGAAGAACATCTTATGAGTGCCATGAA
CATCAAGCTGGGCCCTGCCCTCAAGATCTGCGCTAAGATAAATGTCCTCAAGGAGACC
>Saimiri boliviensis boliviensis XM_010337395.1
ATGGAGACTGAGAGCGAGCA
GAACTCCAACTCCACCAATGGGAGTTCCAGCTCAGCGGGCAGCTCTCGGCCCCAGATAGCTCAAATGTCA
CTCTATGAACGACAAGCAGTGCAGGCTCTACAGGCACTGCAGCGGCAGCCCAATGCAGCTCAGTATTTCC
ACCAGTTCATGCTCCAGCAGCAGCTCAGTAATGCCCAGCTGCATAGCCTGGCTGCCGTCCAGCAGGCTAC
AATTGCTGCCAGCCGGCAGGCCAGCTCCCCAAACACCAGCACCACACAGCAGCAGACTACCACTACCCAG
GCCTCAATCAATCTGGCCACCACATCGGCCGCCCAGCTCATCAGCCGATCCCAGAGTGTGAGCTCTCCCA
GTGCTACCACCTTGACCCAATCTGTGCTACTGGGGAACACCACCTCCCCACCCCTCAACCAGTCTCAGGC
CCAGATGTATCTACGGCCACAGCTGGGAAACCTATTGCAGGTAAACCGAACCCTGGGTCGGAATGTCCCT
CTGGCCTCCCAGCTCATCCTGATGCCTAATGGTGCAGTGGCTGCAGTCCAACAGGAGGTGCCGTCTGCTC
AGTCTCCTGGAGTTCATGCAGATGCAGATCAGGTGCAGAACTTGGCAGTAAGGAATCAACAGGCCTCAGC
CCAAGGACCTCAAATGCAAGGCTCCACTCAGAAGGCCATTCCTCCAGGAGCCTCCCCTGTCTCTAGCCTC
TCCCAGGCCTCTAGCCAGGCCCTAGCTGTGGCACAGGCTTCCTCTGGGGCCTCAAGCCAATCCCTCAACC
TTAGTCAAGCTGGTGGAGGCAGTGGGAATAGCATCCCAGGGTCCATGGGTCCAGGTGGAGGTGGGCAGGC
ACATGGTGGCTTGGGTCAGTTGCCTTCCTCAGGAATGGGTGGTGGGAGCTGTTCCAGGAAGGGCACAGGA
GTGGTGCAGCCCTTGCCTGCAGCCCAGACAGTGACTGTGAGCCAGGGCAGCCAGACAGAGGCAGAAAGTG
CAGCAGCCAAGAAGGCAGAAGCAGATGGTGGCGGTCAGCAGAACGTGGGCATGAACCTGACACGGACAGC
CACACCTGCGCCCAGCCAGACACTTATTAGCTCAGCCACCTACACACAGATCCAGCCCCACTCACTGATT
CAGCAACAGCAACAGATCCACCTCCAGCAGAAACAGGTGGTGATCCAGCAGCAGATTGCCATCCACCACC

AGCAGCAGTTCCAGCACCGTCAGTCCCAGCTCCTTCACACAGCTACACACCTCCAGCTGGCCCAGCAGCA
GCAACAGCAGCAGCAGCAGCAGCAGCAACAGCAGCAGCCGCCAGCCACAACCCTCACTGCCCCTCAGCCA
CCACAGGTCCCACCCACTCAGCAGGTCCCACCATCCCAGTCTCAGCAGCAAGCTCAAACCCTGGTGGTTC
AGCCCATGCTTCAGTCTTCACCCTTGTCCCTTCCACCTGATGCAGCTCCTAAGCCACCGATTCCCATCCA
GTCCAAACCACCTGTAGCACCTATCAAGCCACCTCAGTTAGGGGCTGCTAAGATGTCAGCTACCCAGCAG
CCACCACCCCACATCCCTGTGCAAGTTGTAGGCACTCGACAGCCAGGTACAGCCCAGGCACAGGCTTTGG
GATTGGCACAGCTGGCAGCTGCTGTACCCACTTCCCGGGGGATGCCGGGTACAGTGCAGTCTGGTCAGGC
CCATTTGGCTTCCTCGCCGCCCTCATCCCAGGCTCCTGGTGCGCTGCAGGAGTGCCCTCCCACATTGGCC
CCTGGGATGACCCTTGCTCCTGTGCAGGGGACAGCACATGTAGTAAAGGGTGGGGTTACCACCTCCTCAC
CTGTTGTAGCCCAGGTCCCTGCTGCCTTCTATATGCAGTCTGTGCACTTGCCGGGTAAACCCCAGACATT
GGCTGTCAAACGCAAGGCTGACTCTGAGGAGGAGAGAGATGATGTTTCCACGTTGGGTTCAATGTTTCCT
GCCAAGGCATCTCCAGTAGCAGAGAGCCCACAAGTCCTGGAGGACAAGAGCAGTCTTGGAGAAAAACCTG
AACCAGTGACTAATGTGAATGCTAATACCCCATGCAGTGAACTAGTAGCCTTGACCCCCGCCCCATCAGT
ACCACCTCCTACATTAGCCATGGTGTCTAGACAAATGGGTGACTCAAAACCCCCACAGGCCATCGTGAAG
CCCCAGATTCTCACCCATATCATTGAAGGCTTTGTCATCCAGGAAGGAGCAGAACCTTTCCCGGTGGGTT
GTTCTCAGTTACTAAAGGAGTCTGAAAAGCCACTACAGACTGGCCTTCCGACAGGGCTGACTGAGAATCA
GTCAGGTGGCTCTTTGGGAGTGGACAGCCCATCTGCTGAGTTAGATAAGAAGGCGAATCTCCTGAAGTGC
GAGTACTGTGGGAAGTACGCCCCCGCCGAGCAGTTTCGCGGCTCTAAGAGGTTCTGCTCCATGACTTGCG
CTAAGAGGTACAATGTGAGCTGTAGCCATCAGTTCCGGCTGAAGAGGAAAAAAATGAAAGAGTTTCAAGA
AGCCAACTATGCTCGAGTTCGCAGGCGTGGCCCCGCCGCAGCTCCTCTGACATTGCCCGTGCCAAGATT
CAGGGCAAGTGCCACCGGGGTCAAGAGGACTCTAGCCGGGGTTCAGATAATTCCAGTTATGATGAAGCAC
TCTCTCCAACATCTCCTGGGCCTTTATCAGTAAGAGCTGGGCATGGAGAACGCGACCTGGGGAACCCCAA
TACAGCTCCACCTACACCGGAATTACATGGCATCAACCCTGTGTTCCTGTCTAGTAATCCCAGCCGCTGG
AGTGTAGAAGAGGTGTATGAGTTTATTGCTTCTCTCCAAGGCTGCCAAGAGATTGCAGAGGAATTTCGTT
CCCAGGAGATTGATGGACAGGCCCTTTTATTACTTAAAGAAGAACATCTAATGAGTGCCATGAACATCAA
GCTGGGCCCTGCCCTCAAGATCTGCGCCAAGATAAATGTCCTCAAGGAGACC
>Propithecus coquereli XM_012637879.1
ATGGAGACTGAGAGTGAGCAGAACTCCAACT
CCACTAATGGGAGTTCCAGCTCAGGGGGCAGCTCTCGGCCCCAGATAGCTCAAATGTCACTGTATGAACG
ACAAGCAGTACAGGCTCTGCAGGCATTGCAGCGGCAGCCCAATGCGGCTCAGTATTTCCACCAGTTCATG
CTCCAGCAGCAGCTCAGTAATGCCCAGCTGCATAGCCTGGCTGCAGTCCAGCAGGCCACAATTGCTGCCA
GTCGGCAGGCCAGCTCCCCAAACACCAGCACCAGCCAGCAGCAGACTACCACCACCCAGGCCTCAATCAA
TCTGGCCACCACGTCAGCTGCCCAGCTCATCAGCCGATCCCAGAGTGTGAGCTCTCCCAGTGCTACCACC
TTGACCCAATCTGTGCTACTGGGGAACACCACCTCCCCACCCCTCAACCAGTCTCAGGCCCAGATGTATC
TGCGGCCACAGCTGGGAAACATATTGCAGGTAAACCGAACCCTGGGCCGGAATGTGCCTCTAGCCTCCCA
ACTCATCCTGATGCCCAATGGGGCAGTGGCTGCAGTCCAGCAGGAGGTCCCATCTGCACAGTCTCCTGGA
GTTCATGCAGATGCAGATCAGGTGCAGAACTTGGCAGTGAGGAACCAACAAGCCTCAGCCCAAGGATCCC
AAATGCAAGGCTCCACTCAGAAGGCCATTCCTCCTGGAGCCTCTCCTGTGTCTAGCCTCTCTCAGGCCTC
TAGCCAGGCCCTAGCTGTGGCACAGGCCTCCTCTGGGGCCTCAGGCCAGTCCCTCAACCTAAGTCAAGCT
GGTGGAGGAAGTGGGAATAGCATCGCAGGGTCCATGGGTCCAGGTGGAGGTGGCCAGGCCCCTGGGGGTT
TGAGTCAGTTGCCTTCCTCAGGAATGGGCGGTGGGAGCTGTCCCAGGAAGGGCACAGGAGTAGTGCAGCC
CCTGCCTGCAGCCCAGACAGTGACTGTGAGCCAGGGCAGCCAGACAGAGGCAGAAAGTGCAGCAGCCAAG
AAGGCAGAAGCAGATGGGAGTGGTCAGCAGAACGTGGGCATGAACCTCACACGGACAGCTACACCTGCGC
CTAGCCAGACGCTTATTAGCTCAGCCACCTACACACAGATCCAGCCCCATTCCCTGATTCAGCAACAGCA
ACAGATCCACCTCCAGCAGAAACAGGTGGTGATCCAGCAGCAGATTGCCATCCACCACCAGCAGCAGTTC
CAGCACCGCCAGTCCCAGCTCCTTCACACAGCTACACACCTCCAGTTGGCCCAACAGCAGCAGCAACAGC
AGCAGCAGCAACAGCAGCAGCAGCAGCAGCAGCAGCAAGCCACAACCCTCACTGCCCCTCAGCCACC

ACAGGTCCCACCTACTCAGCAGGTCCCACCCTCCCAGTCACAGCAGCAAGCCCAAACCCTGGTTGTTCAA
CCCATGCTTCAGTCTTCACCTCTGTCCCTTCCACCTGACCCAACCCCCAAGCCACCAATTCCTATCCAGT
CCAAACCACCTGTGGCACCTATTAAGCCTCCTCAGTTAGGGGCTGCTAAGATGTCAGCTACCCAGCAACC
ACCACCCCATATCCCTGTGCAAGTTGTAGGTACCCGACAGCCAGGTACAGCCCAGGCACAGGCTTTGGGG
TTGGCACAGCTGGCAGCTGCTGTACCTACTTCCCGGGGGATGCCGGGTACAGTGCAGCCTAGTCAGGCCC
ATTTTGCCCCCTCGCCACCTTCATCCCAGGCTCCTGGTGCACTGCAGGAGTGCCCTGCTACGTTGGCCCC
TGGGATGACCCTTGCTCCTGTACAGGGGACAGCACATGTTGTAAAGGGTGGGGCTACCACCTCCTCACCC
GTCGTAGCCCAGGTCCCTGCTTCCTTCTACATGCAGTCTGTACACTTGCCGGGTAAACCCCAGCCTTTGG
CTGTCAAACGCAAAGCTGAGTCTGAGGAGGACAGAGATGATGTCTCCACATTGAGTTCAATGCTTCCTGC
TAAGGCATCTCCAGTGGCAGAGAGCCCAAAGGTCATGGAGGAGAAGAGCAGTCTTGGAGAGAAAGCTGAA
CCAGTGGCCAACGTGAATGCTAACACCCCAAGCAGTGAACTAGTAGCCTTGACCTCTGCCCCATCAGCAC
CACCTCCTACACTAGCCATGGTTTCCAGACAAATGGGTGACTCAAAACCCCCACAGGCCATTGTGAAGCC
CCAGATTCTCACCCACATCATTGAAGGCTTTGTTATCCAGGAAGGAGCAGAACCTTTCCCGGTGGGTTGT
TCTCAGTTACTGAAGGAGTCTGAGAAGCCACTACAGGCTGGCCTTCCAACGGGGCTGAATGAGAATCAGT
CAGGTGGCCCCTTGGGAGGGGACAGCCCATCTGCTGAGTTAGATAAGAAGGCGAATCTCTTGAAGTGCGA
GTACTGTGGGAAGTACGCCCCTGCAGAGCAGTTCCGTGGCTCGAAGAGGTTCTGCTCCATGACTTGCGCT
AAGAGGTACAATGTGAGCTGTAGCCATCAGTTCCGGCTGAAGAGGAAAAAAATGAAAGAGTTTCAAGAAG
CCAACTATACTCGTGTTCGCAGGCGTGGACCCCGCCGCAGCTCCTCTGACATTGCCCGCGCTAAGATTCA
GGGCAAACGCCACCGGGGTCAAGAGGACTCTAGCCGGGGTTCAGATAATTCCAGTTATGATGAAGCACTC
TCCCCAACATCTCCCGGGCCTTTATCAGTAAGAGCTGGGCATGGAGAACGTGATCTGGGGAACCCCAATA
TAGCTCCATCTACACCGGAATTGCATGGCATCAATCCCGTGTTCCTGTCCAGTAATCCCAGCCGTTGGAG
TGTAGAGGAGGTGTATGAGTTTATCGCTTCTCTCCAAGGCTGCCAAGAGATTGCAGAGGAGTTTCGCTCC
CAGGAGATTGATGGACAGGCCCTTTTATTACTTAAAGAAGAACATCTTATGAGTGCCATGAACATCAAGC
TGGGCCCTGCCCTCAAGATCTGCGCCAAGATAAACATCCTCAAGGAGACC

>Carlito syrichta XM_008059434.1
ATGGAGACTGAGAGTGAGCAGAACTCCAACTCCACC
AATGGGAGTTCCAGCTCAGGGGGCAGCTCTCGACCCCAGATAGCTCAAATGTCACTGTATGAACGACAAG
CAGTGCAGGCTCTGCAGGCCCTCCAGCGGCAGCCCAACGCAGCACAGTATTTCCACCAGTTCATGCTCCA
GCAGCAACTCAGCAATGCCCAGCTGCATAGCCTGGCTGCTGTCCAGCAGGCCACAATTGCTGCCAGTCGG
CAGGCCAGCTCCCCAAACACCAGCACTGCACAGCAGCAGACCACCACCACTCAGGCCTCAATAAATCTGG
CCACCACGTCGGCCGCCCAACTCATCAGCCGATCCCAGAGTGTGAGCTCTCCTAGTGCCACCACCTTGAC
CCAGTCTGTGCTACTGGGGAACACCACCTCCCCACCCCTCAACCAGTCCCAGGCCCAGATGTATCTACGG
CCACAGCTGGGGAACCTGTTGCAGGTAAACCGAACGCTGGGCCGGAATGTGCCTCTAGCCTCCCAACTCA
TCCTGATGCCTAATGGGGCAGTGGCTGCAGTCCAGCAGGAAGTACCATCTGCTCAGTCTCCTGGAATTCA
TTCAGATGCAGACCAGGTGCAGAACTTGGCAGTGAGGAACCAACAAGCCTCAGCCCAAGGACCCCAAATG
CAAGGCTCCACTCAGAAGGCCATTCCTCCTGGAGCGTCCCCTGTCTCTAGCCTCTCTCAGGCCTCTAGCC
AGGCCCTGGCTGTAGCACAGGCTTCCTCTGGGGCCTCAGCCCAGTCCCTCAACCTTAGCCAAGCTGGCGG
AGGCAGCGGGAATAGCATCCCAGGGTCCATGGGTCCAGGTGGGGGTGGCCAGGCACCTGGGGGTTTGGGT
CAGTTGCCTTCCTCAGGAATTGGTGGTGGGAGCTGTCCCAGGAAGGGCACAGGAGTTGTGCAGCCATTGC
CTGCGGCACAAACAGTGACTGTGAGTCAGGGCAGCCAGACAGAGGCAGAAAGCGCAGCAGCCAAGAAGGC
CGAAGCAGATGGGAGTGGTCAGCAGAATGTGGGCATGAACTTGACACGGACAGCTACACCTGCACCCAGC
CAGACGCTTATTAGTTCAGCCACCTACACGCAGATCCAGCCGCATTCACTGATTCAGCAACAGCAGCAGA
TCCACCTTCAGCAGAAACAGGTGGTGATCCAGCAGCAGATTGCCATCCACCACCAGCAGCAGTTCCAGCA
CCGGCAGTCCCAGCTCCTCCACACAGCCACACACCTCCAGCTGGCCCAGCAGCAGCAGCAGCAGCCACCA
CCACCGCCGCCGCCGCAGGCCACAGCCCTCCCTGCCCCTCCGCCCCCACAGGTCCCACCTGCCCAGGTCC
AGCAACAAGCCCAAACTCTGGTGGTTCAGCCCATGCTTCAGTCTTCACCGCTGTCCCTTCCCCCTGACCC

AGCCCCCAAGCCACCAGTCCCCATCCAATCCAAATCTCCCGCAGCACCGCTCAAGCCTCCTCAGTTGGGG
GCTGCCAAGATGTCTGCTGCCCCGCAACCACCTCCTCATATCCCCGTGCAAGTAGTAGGCACCCGACAGC
CAGGTACAGCCCAGGCACAGGCTCTGGGGCTGGCACAGCTGGCAGCTGCTGTCCCCACTTCCCGAGGGAT
GCCAGGTGCAGTGCAGCCTGGTCAGGCCCATTTGGCTGCCTCGCCACCTTCATCCCAGGCTCCTGGTGCA
CTGCAGGAATGCCCTCCTACCTTGGCCCCTGGGATGACCCTGGCTCCTGTGCAGGGGACGGCACATGTGG
TCAAAGGGGGGACTACCACCTCCTCGCCCGTTGTAGCCCAGGTCCCTGCTGCCTTCTACATGCAGTCTGT
GCACTTGCCGGGTAAACCCCAGACATTGGCCGTCAAACGCAAGGCTGAGTCTGAGGAGGAGAGAGATGAT
GTCTCCACATTGGGCTCAATGCTTCCTGCCAAGGCATCGCCAGTAGCAGAGAGCCCAAGGTCTGTGGAGG
AGAAGAGCAGTCTTGGAGAGAAAGCTGAACCAGTGGCCAATGTGAATGCTAACACCCCAAGCAGTGACCT
AGTAGCCTTGACCCCTGCTCCATCCGTACCACCTCCTACACTAGCCATGGTGTCCAGGCAAATGGGTGAC
TCAAAACCCCCACAGGCCATTGTGAAGCCCCAGATTCTCACCCACATCATTGAAGGCTTTGTTATTCAAG
AAGGAGCAGAACCTTTCCCGGTGGGTTGTTCTCAGTTACTGAAGGAGTCTGAGAAGCCACTGCAGACTGG
CCTTTCGACAGGGCTGGCCGAGAATCAGTCAGGTGGCCCCTTGGGAGGAGACAGCCCCTCTACAGAGTTA
GACAAGAAGGCGAACCTCCTGAAGTGTGAGTACTGCGGCAAGTACGCCCCTGCAGAGCAGTTTCGTGGCT
CCAAGAGGTTCTGCTCCATGACTTGCGCTAAGAGGTACAATGTGAGTTGTAGCCATCAGTTCCGGCTGAA
GAGGAAAAAGATGAAAGAATTTCAAGAAGCCAACTATGCTCGCGTTCGCAGGCGTGGCCCCCGCCGCAGC
TCCTCTGACATTGCCCGTGCCAAGATTCAGGGCAAACGCCACCGGGGTCAAGAGGACTCTAGCCGGGGTT
CAGATAATTCCAGTTATGATGAAGCACTCTCCCCAACTTCTCCTGGGCCTTTATCAGTAAGAGCTGGGCA
TGGAGACCGTGACCTGGGGAACCCCAATACTGCTCCACCTACACCGGAACTACACGGCATCAACCCTGTG
TTCCTGTCCAGTAATCCCAGCCGCTGGAGTGTAGAGGAGGTGTATGAGTTCATCGCTTCTCTCCATGGCT
GCCAAGAGATTGCAGAGGAGTTTCGCTCCCAGGAGATTGATGGACAGGCTCTTTTATTACTTAAAGAAGA
ACATCTTATGAGTGCCATGAACATCAAGCTGGGCCCTGCCCTCAAGATCTGCGCCAAGATAAATGTCCTC
AAGGAAACC
>Galeopterus variegatus XM_008590854.1
ATGGAGACTGAGAGCGAGC
AGAACTCCAATTCCACCAATGGGAGTTCCAGCTCAGGGGGCAGCTCTCGGCCCCAGATAGCTCAAATGTC
ACTGTATGAACGACAAGCAGTGCAGGCTCTGCAGGCACTGCAGCGGCAGCCCAATGCAGCTCAGTATTTC
CACCAGTTCATGCTCCAGCAGCAGCTCAGTAATGCCCAGCTGCATAGCCTGGCTGCCGTCCAGCAGGCCACA
ATTGCTGCCAGTCGGCA
GGCCAGCTCCCCAAACACCAGCACTACACAGCAGCAGACTACCACCACCCAGGCCTCAATCAATCTGGCC
ACCACGTCGGCTGCCCAGCTCATCAGCCGATCCCAGAGTGTGAGCTCTCCCAGTGCTACCACCTTGACGC
AATCTGTGCTACTGGGCAACACCACCTCTCCACCTCTCAACCAGTCTCAGGCCCAGATGTATCTACGGCC
ACAGCTGGGAAACCTGTTGCAGGTAAACCGGACCCTGGGCCGGAATGTGCCTCTCGCCTCCCAACTCATT
CTGATGCCTAACGGGGCGGTGGCTGCAGTGCAGCAGGAGGTACCATCTGCTCCGTCCCCTGGAGTTCATG
CAGATACAGATCAGGTGCAGAACTTGGCAATGAGGAACCAACAGGCCCCAGCCCAAGGACCCCAAATGCA
AGGCTCCACTCAGAAGGCTATTCCTCCTGGAGCCTCCCCTGTCTCTAGCCTCTCCCAGGCCTCTAGCCAG
GCCCTAGCTATGGCACAGGCTTCCTCTGGGGCCTCAGGCCAGTCCCTCAACCTTAGTCAAGCTGGTGGAG
GTAGTGGGAATAGCATCCCTGGGTCCATGGGTCCAGGTGGAGGTGGCCAGGCACCCGGAGGTTTGGGTCA
ATTGCCTTCCTCAGGAATGGGTGGTGGGAGCTGTCCCAGAAAGGGCACAGGTGTGGTGCAGCCCTTGCCT
ACAGCCCAGACAGTGACTGTGAGCCAGGGCAGCCAGACAGAGGCAGAAAGTGCAGCAGCCAAAAAGGCAG
AAGCAGATGGGAGTGGACAGCAGAATGTGGGCATGAACTTGACACGGACAGCTACACCCGCCCCCAGTCA
GACACTTATTAGCTCAGCCACCTACACACAGATCCAGCCCCATTCACTGATTCAGCAACAGCAGCAGATC
CATCTCCAGCAGAAGCAGGTGGTGATCCAGCAGCAGATCGCCATCCACCACCAGCAACAGTTCCAGCACC
GTCAGTCCCAGCTGCTTCACACAGCCACACACCTCCAGCTGGCCCAGCAGCAGCAGCAGCAGCAGCAGCA
GCAGCAGCAACAGCAGCAGCAAGCCACAACCCTCACTGCCCCTCAGCCACCACAGGTCCCACCTACTCAG
CAGGTCCCACCTTCCCAGTCCCAGCAGCAAGCCCAAACCCTGGTTGTTCAACCCATGCTTCAGTCTTCAC
CCCTGTCCCTTCCACCTGACCCAACCCCCAAGCCACCCATCCCCATCCAATCCAAACCACCTGTAACGCC

TATCAAGCCTCCTCAGTTAGGGGCTGCTAAGATGTCTGCTACCCAGCAACCACCACCCCATATCCCTGTG
CAAGTTGTGGGCACCCGCCAGCCAGGTACAGCCCAGGCACAGGCTTTGGGGTTGGCACAGCTGGCAGCTG
CTGTACCTACTTCCCGGGGGATGCCAGGTACAGTGCAGCCTGGTCAGACCCATTTGGCCTCCTCACCACC
TTCATCCCAGGCTCCTGGTGCACTACAGGAGTGCCCTCCTACACTGGCCCCTGGGATGACCCTTGCTCCT
GTGCAGGGGACAGCACATGTGGTCAAGGGTGGGGCTACCACCTCCTCACCTGTTGTAGCCCAGGTCCCTG
CTGCCTTCTACATGCAGTCTGTGCACCTGCCGGGTAAACCCCAGACAGTGGCTGTCAAACGCAAGGCTGA
GTCTGAGGAGGAGAGAGACGATGTCTCCACATTGGGGTCAATGCTTCCTGCCAAGGCCTCTCCAGTAGCA
GAGAGCCCAAAGGTCATGGAGGAGAAGAGCAGTCTTGGAGAGAAAGCTGAACCAGTGGTCAGTGTGAATG
CTAATACCCCAAGCAGTGAAGTAGTAGCCTTGACCCCTGCCCCATCAGCACCACCTCCTACGTTAGCCAT
GGTGTCCAGACAAATGGGTGATTCGAAACCCCCACAAGCCATTGTGAAGCCCCAGATTCTCACCCACATC
ATTGAAGGCTTTGTTATCCAGGAAGGAGCAGAACCTTTTCCGGTGGGTTGTTCTCAGTTGCTGAAGGAGT
CTGAGAAGCCACTACAGACTGGCCTCCCAACAGTGCTGAATGAGAATCAGTCAGGTGGCCCTTTGGGAGG
GGACAGCCCATCTGCTGAGTTGGATAAGAAGGCAAATCTCCTGAAGTGCGAGTACTGTGGGAAGTACGCC
CCCGCAGAGCAGTTTCGTGGCTCTAAGAGGTTCTGCTCCATGACTTGTGCTAAGAGGTACAATGTGAGCT
GTAGCCACCAGTTCCGGCTGAAGAGGAAAAAAATTAAAGAGTTTCAAGAGGCCAACTATGCTCGAGTTCG
CAGGCGTGGGCCCCGTCGCAGCTCCTCTGATATTGCTCGTGCCAAGATCCAGGGCAAGCGCCACCGGGGT
CAAGAGGACTCTAGCCGGGGTTCAGATAATTCCAGTTATGATGAAGCACTCTCCCCAACATCTCCTGGGC
CTTTATCAGTAAGAGCTGGGCATGGAGAACGTGACCTGGGGAACCCCAATACAGTTCCATCTACACCAGA
ATTACATGGCATCAACCCTGTGTTCCTGTCCAGTAATCCTAGCCGCTGGAGTGTAGAGGAGGTGTATGAG
TTTATTGCTTCTCTCCAAGGCTGCCAAGAGATTGCAGAGGAGTTCCGCTCCCAGGAGATTGATGGACAGG
CCCTTTTATTACTTAAGGAAGAACATCTTATGAGTGCCATGAATATCAAGTTGGGCCCTGCCCTCAAGAT
CTGCGCCAAGATAAATGTCCTCAAGGAGACC
>Cavia porcellus XM_003470494.3
ATGGAGACTGAGAGCGAGCAGAACACTGGTTCCACC
AATGGGAACTCCAGCTCTGGGGGCAGCTCTCGGCCCCAGATAGCGCAGATGTCCCTGTATGAGCGGCAGG
CGGTTCAGGCTCTGCAGGCTCTACAGCGGCAGCCCAATGCGGCTCAGTATTTCCACCAGTTCATGCTTCA
GCAGCAACTCAGCAATGCCCAGCTACATAGCCTGGCTGCAGTCCAGCAGGCCACGATTGCTGCCAGTCGG
CAGGCCAGCTCTCCGAACACCAGCACCACGCAGCAGCAGGCGACCACCACCCAGGCCTCAATCAACCTGG
CCACCACCTCAGCTGCCCAGCTAATCAGTCGATCCCAGAGTGTGAGCTCCCCCAGTGCCACCACCTTGAC
CCAGTCTGTGTTACTCGGGAACACCACCTCTCCACCCCTCAACCAGTCCCAGGCCCAGATGTATCTGCGG
CCACAGCTGGGAAACCTATTGCAGGTGAACCGGACCCTGGGCCGAAATGTGCCTCTGGCTTCCCAGCTGA
TCCTGATGCCCAACGGGGCAGTGGCTGCGGTCCAGCAGGAGGTGCCCTCGGCTCAGTCCCCCGGAGTTCA
CGCGGATGCGGATCAGGTGCAGAACTTGGCAGTGAGGAACCAGCAGGCCTCTGCTCAAGGGGCCCCGATG
CCAGGCTCCTCTCAGAAGGCCATTCCTCCTGGAGCCTCTCCAGTCTCAGGCATCTCTCAGGCCTCGAACC
AGGCACTTGCTGTGGCACAGGCTTCTTCCGGGGCCTCAGGCCAGTCCCTCAATCTCAGTCAGGCTGGCGG
AAGCAGCGGGAGTAGCCTCACAGGGTCCCTGGGTCCAGGTGGAGGTGGCCAGGCACCTGGGGGCTTGGGT
CAGTTGCCTTCTTCAGGGATGGGTGGGGCAGGCTGTCCCAGGAAGGGCACAGGAGTAGTCCCACCCCTGC
CGGCAGCCCAGACAGTGACTGTGAGCCAGGGCAGCCAGACAGAGGCGGAAAGTGCGGCGGCCAAGAAGGC
AGATGCAGATGGCAGTGGTCAGAACGTGGGCATGAACCTGACACGGACAGCCACCCCTGCCCCCAGCCAG
ACTCTTATTAGCTCAGCTACCTATACACAGATCCAGCCCCACTCGCTAATTCAGCAGCAGCAGCAGATCC
ACCTCCAGCAGAAGCAGGTGGTGATCCAGCAGCAGATCGCCATCCACCACCAGCAGCAGTTCCAGCACCG
TCAGTCCCAGCTGCTGCACACAGCAACACATCTCCAGCTGGCCCAGCAGCAGCAACAGCAGCAGCAGCAG
CAGCAGCAGCAGCAGCAGCAGCAGCAGCAGCAGCAGCAACAGCAAGCTACAACCCTCACTGCCCCCCAGC
CACCACAGATTCCCCCTACTCAGCAGGTCCCGCCCTCCCAGTCCCAACAGCAAGCCCAGACCCTTGTGGT
TCAACCCATGCTTCAGTCTTCACCGCTGTCTCTTCCGCCAGACCCAACCCCCAAGCCACCCATCCCTATC
CAGTCCAAGCCACCCATAGCGCCTATCAAACCTCCTCAGTTAGGGGCTACGAAGATGTCAGCTCCCCAGC
AACCACCACCCCACATCCCTGTGCAAGTTGTGGGTACCCGGCAGCCAGGTACAGCCCAGGCACAGGCTCT

GGGCTTGGCACAGCTGGCAGCCGCTGCCCCTGCCTCCCGGGGGATGCCAAGTGCAGTGCAGCCTGGCCAG
GCCCACTTGGCCTCTTCCCCACCTTCATCTCAGGCTCCTGGCACACTGCAAGAGTGTCCTCCTACATTGG
CCCCTGGGATGACCCTTGCTGCTGTGCAGGGAACAGCACATGTGGTAAAGGGTGTGTCCACCACATCTTC
ACCTGTGGTTGCGCAGGTCCCTGCTGCCTTCTACATGCAGTCTGTACACCTGCCGGGTAAACCCCAGACA
CTGGCTGTGAAGCGCAAAGCTGAGTGTGAGGAGGAGAGAGATGAGGTCTCCGCATTGAGCTCAATGCTCC
CTGCCAAGGCATCTCCAGCTGCAGAGAGCCCAAAGGTCATGGAGGAAAAGACCAGTCTTGCGGAGAAAGC
TGACTCCGGGGCCAGTGTGAATACCAACCCCCCCAGCAGTGAGCTTGTAGTTTTGGGCCCTGCCCCGGCA
GCACCGCCTCCTTCATTGGCCATGGTGTCCAGACAAATGGGAGACTCGAAACCTCCACAGGCCATCGTGA
AGCCTCAGATTCTCACTCACATCATTGAGGGCTTCGTTATCCAGGAAGGAGCGGAACCTTTCCCGGTGGG
TTGTTCTCAGTTACTAAAGGAGTCTGAGAAGCCACTACAGACTGGACTCCCAACAGGGCTGAGTGAAAGT
CAGTCGAGTGGCCCCTTGGGAGGGGACAGCCCTTCTGCCGATTTAGATAAGAAGGCAAACCTCCTGAAGT
GCGAGTACTGTGGGAAGTACGCCCCTGCAGAGCAGTTCCGAGGCTCTAAGAGGTTCTGCTCTATGACTTG
TGCCAAGAGGTACAATGTGAGCTGTAGCCACCAGTTTCGGCTCAAGAGGAAAAAAATGAAGGAGTTTCAA
GAAGCCAACTATGCTCGAGTTCGTAGGCGTGGGCCTCGCCGTAGCTCCTCTGATATCGCCCGAGCTAAGA
TCCAGGGCAAGCGCCACCGGGGTCAAGAGGATTCCAGCCGGGGTTCAGATAACTCCAGTTACGATGAAGC
ACTGTCCCCTACATCTCCTGGGCCATTGTCAGTACGAGCTGGGCATGGAGAACGTGACCTAGGGAGTACC
ATTGCAGCGCCACCCACACCGGAATTACACGGCATAAACCCTGTGTTCTTGTCCAGTAATCCAAGTCGCT
GGAGTGTAGAGGAGGTGTATGAGTTCATAGCTTCTCTCCAAGGCTGCCAAGAGATCGCAGAGGAGTTCCG
TTCCCAGGAGATTGATGGACAGGCCCTTTTATTACTTAAAGAAGAACATCTTATGAGTGCCATGAATATC
AAATTGGGTCCTGCCCTTAAGATCTGTGCCAAGATAAATGTCCTCAAGGACACC
>Ictidomys tridecemlineatus ENSSTOT00000013210.2
ATGGAGACTGAGAGCGAGCAGAACTCCAGCTCCACCAATGGGAGTTCCAGCTCAGGGGGC
AGCTCTCGGCCCCAGATAGCTCAAATGTCCCTATATGAACGACAAGCAGTGCAGGCTCTG
CAGGCATTGCAGCGGCAGCCCAATGCAGCTCAGTATTTCCACCAGTTCATGCTCCAGCAG
CAGCTCAGTAATGCCCAACTGCATAGCTTGGCTGCCGTCCAGCAGGCCACGATTGCTGCT
AGTCGGCAAGCCAGCTCCCCAAACACCAGCACTGCACAGCAGCAGACTACCACCACTCAG
GCCTCAATTAATCTGGCCACCACGTCAGCTGCCCAGCTTATTAGCCGATCACAGAGTGTG
AGCTCTCCCAGTGCCACCACCTTGACTCAATCTGTGCTGCTGGGGAATACTACTTCCCCA
CCCCTCAACCAGTCCCAGGCCCAGATGTATCTGCGGCCACAGCTGGGAAACCTATTGCAG
GTAAACCGGACCCTGGGCCGGAATGTGCCTCTAGCCTCCCAACTCATCCTGATGCCTAAT
GGGGCAGTGGCTGCAGTCCAGCAGGAGGTGCCATCCGCTCAGTCTCCTGGAGTTCATGCA
GATGCAGATCAGGTGCAGAACTTGGCAGTGAGGAATCAACAGGCCTCAGCTCAAGGACCC
CAAATGCAAGGTTCCACTCAGAAGGCCATACCTCCTGGAGCCTCCCCTGTCTCTAGCCTT
TCTCAGGCCTCTAGCCAGGCCCTAGCTGTGGCACAGCCTTCTCCAGGGGCCTCAGGCCAA
TCTCTCAACCTCAGTCAAGCTGGTGGAGGCAGTGGAAACAGCCTCTCAGGGCCAATGGGT
CCAGGTGGAGGTGGCCAAGCTCCAGGGGGTTTGGGTCAGTTGCCTTCTTCAGGAATGGGT
GGTGGAAGCTGTCCCAGGAAGGGCACAGGAGTGGTTCAGCCCTTGCCTGCTGCCCAGACG
GTGACTGTGAGCCAGGGCAGCCAGACAGAGGCAGAAAGTGCAGCAGCCAAGAAAGCAGAT
GCTGATGGGAGTGGTCAACAGAATGTGGGCATGAACTTGACACGGACAGCTACACCTGCA
CCCAGCCAGACCCTCATTAGCTCAGCCACGTACACGCAGATCCAGCCCCATTCTCTGATT
CAGCAGCAGCAGCAGATCCACCTCCAGCAGAAGCAGGTAGTGATCCAACAGCAGATTGCC
ATTCATCACCAGCAACAGTTCCAACACCGTCAGTCCCAGCTACTTCACACAGCTACACAC
CTCCAGTTGGCTCAGCAGCAGCAGCAACAGCAACAACAACAGCAGCAGCAGCAGCAGCAG
CAGCAGCAGCAACAGCAGCAGCAGCAGCAAGCCACAACCCTCACTGCCCCTCAGCCACCA
CAGGTCCCACCTACTCAGCAGGTCCCACCGTCCCAGTCCCAGCAGCAAGCTCAAACCCTT
GTAGTTCAGCCCATGCTTCAGTCTTCACCCCTGTCCCTTCCACCTGACCCAACCTCCAAG
CCACCCATTCCCATCCAGTCCAAACCACCTGTAGCACCTATTAAACCTCCTCAGTTAAGT

GCTGCTAAGATGGCAGCTACCCAGCAACCACCACCCCACATCCCCGTGCAAGTTGTGGGT
ACCCGGCAACCAGGTACAGCCCAGGCACAGGCTTTGGGGTTGGCACAGCTGGCAGCTGCT
GTACCTACTTCCCGGGGGCTGCCAGGTACAGTGCAGCCTGGCCAGGCCCATTTGGCCTCC
TCACCACCTTCATCCCAGGCTCCTGGTGCACTGCAGGAGTGTTCTCCTACGTTGGCCCCT
GGGATGACCCTTGCTTCTGTGCAGGGGACAGCACATGTGGTTAAGGGTGGGGCTACTAGC
TCTTCACCTGTTGTAGCCCAGGTTCCTGCTGCCTTCTACATGCAGTCTGTGCATCTACCG
AGTAAACCTCAGACACTGGCTGTGAAACGCAAAGCTGAGTCTGAGGAAGAGAGAGATGAT
ATCTCGACTTTGGGTTCAATACTTCCCCCCAAGGCTTCTCCAGCAGCAGAGAGCCCAAAA
GTCATGGAGGAGAAGAGTAGTCTTGGAGAGAAAGCTGAACCTGTGGCCAGTGTGAATGCT
AGTACCCCAAGCAATGAACTGGTAGCCTTGACTCCTGCCCCATCAGCACCACCTCCTACG
CTAGCCATGGTGTCCAGACAAATGGGAGACTCCAAACCCCCACAGGCCATTGTGAAGCCC
CAGATTCTCACCCACATCATTGAAGGCTTTGTTATCCAGGAAGGAGCGGAACCTTTCCCG
GTGGGTTGTTCTCAGTTATTGAAGGAGTCTGAGAAGCCACTACAGACTGGCCTTCAAACC
GGACTGAATGAGAGTCAGTCAAGTGGACCCTTGGGAGGGGACAGCCCTTCTGCTGAGTTA
GATAAGAAGGCGAATCTCCTGAAGTGCGAGTACTGTGGGAAGTATGCCCCTGCAGAGCAA
TTTCGTGGCTCTAAGAGGTTTTGCTCCATGACTTGTGCAAAGAGGTACAATGTGAGCTGT
AGCCATCAGTTCCGGTTGAAGAGGAAAAAAATGAAAGAGTTTCAGGAAGCCAACTATGCT
CGTGTTCGTAGGCGTGGACCCCGCCGCAGCTCCTCTGACATTGCCCGTGCCAAGATCCAG
GGCAAGCGTCACCGGGGTCAAGAGGACTCTAGCCGGGGTTCAGATAACTCTAGTTATGAT
GAAGCGCTCTCTCCAACATCTCCTGGGCCATTGTCAGTAAGAGCTGGACATGGAGATCGT
GACCTTGGGAACACCATTACAGCTCCCCCCACACCAGAATTACATGGCATCAACCCTGTG
TTCCTGTCCAGCAATCCTAGTCGTTGGAGTGTAGAGGAGGTGTATGAGTTTATCGCTTCT
CTCCAAGGCTGCCAAGAAATTGCAGAAGAGTTTCGTTCCCAGGAGATTGATGGACAGGCT
CTTTTATTACTTAAAGAAGAACATCTTATGAGTGCCATGAACATCAAATTGGGCCCTGCC
CTCAAGATCTGCGCCAAGATAAATGTCCTGAAGGAGACC
>mouse ENSMUST00000160696.7
ATGGAAACGGAGAGTGAGCAGAACTCTAGTTCGACCAATGGGAGTTCCAGTTCAGGGGCC
AGCTCTCGGCCCCAGATAGCACAGATGTCCCTGTATGAACGGCAAGCAGTGCAGGCTCTG
CAAGCACTGCAGCGGCAACCTAATGCGGCTCAATATTTCCACCAGTTCATGCTCCAGCAA
CAGCTCAGCAATGCCCAACTGCACAGCCTGGCAGCTGTCCAGCAGGCCACGATTGCTGCC
AGTCGACAGGCTAGCTCCCCAAACAGCAGCACTGCACAGCAGCAGACCGCTACCACCCAG
GCCTCGATGAATCTCGCGACCACGTCAGCTGCTCAGCTCATCAGCCGATCGCAGAGTGTG
AGCTCCCCCAGTGCTACCACCCTGACGCAGTCGGTGCTGCTGGGGAACACCACTTCCCCA
CCTCTCAACCAGTCCCAGGCCCAGATGTATCTGCGGCCACAGCTGGGCAACCTATTGCAG
GTTAACCGGACCTTGGGCCGGAATGTGCCTCTTGCCTCCCAGCTCATCCTGATGCCTAAT
GGGGCAGTGGCTGCAGTCCAGCAGGAGGTGCCACCCGCTCAGTCTCCTGGAGTTCATGCA
GATGCAGATCAGGTGCAGAACTTGGCAGTGAGGAACCAACAGGCTTCAGCTCAAGGACCC
CAAATGCCAGGCTCCACTCAGAAGGCCATCCCTCCTGGAGCTTCTCCTGTTTCTGGCCTC
TCCCAGACTTCTAGCCAGGCCCTAGCCGTGGCACAGGCTTCTTCTGGGGCCTCAGGCCAG
TCACTCAACCTTAGTCAAGCCGGTGGAGGCAGTGGGAATAGCCTCCCAGGCTCCATGGGT
CCAGGTGGAGGTGGCCAGGCGCCTGGGGGTTTAGGTCAGTTGCCTTCTTCAGGATTGACT
GGTGGAAGCTGTCCCCGGAAGGGCACAGGAGTGGTGCAGCCCTTACCTGCAGCTCAAACA
GTGACTGTGAGCCAAGGAAGCCAAACAGAAGCAGAAAGTGCAGCAGCCAAGAAGGCAGAA
GCAGATGGGAGTGGCCAGCAGAGTGTGGGCATGAACCTGACTCGGACAGCCACACCTGCC
CCCAGCCAGACGCTTATTAGCTCAGCCACATACACACAGATCCAGCCCCATTCACTGATT
CAGCAACAGCAGCAGATCCACCTCCAGCAGAAGCAAGTGGTGATCCAGCAGCAGATTGCC
ATCCACCACCAGCAACAGTTCCAACACCGCCAGTCCCAGCTGCTTCACACAGCCACACAC

CTCCAGTTGGCCCAGCAGCAGCAGCAGCAACAGCAGCAACAGCAGCAACAACAGCAACAG
CAGCAGCAGCAGCAGCAAGGAACAACCCTCACTGCCCCTCAGCCACCCCAGGTCCCACCT
ACTCAGCAGGTCCCACCTTCCCAATCACAACAGCAAGCCCAGACTCTGGTGGTCCAACCC
ATGCTTCAGTCTTCACCCCTGACTCTTCCACCTGAACCAACCTCTAAACCACCCATCCCC
ATTCAGTCCAAACCTCCTGTAGCGCCTATCAAACCTCCTCAACTAGGAGCTGCTAAGATG
TCAGCTACACAGCAGCCACCACCACATATCCCCGTGCAAGTTGTCGGTACCCGGCAGCCA
GGTTCAGCCCAAGCACAGGCTTTGGGATTAGCACAGCTGGCTGCTGCTGTACCTACTCCC
CGTGGAATAACAGGAGCAGTCCAGCCTGGCCAGGCTCATCTGGCCTCCTCGCCACCTTCA
TCACAGGCGGCTCCTGGTGCACTTCAAGAATGTCCTCCTGCATTGGCTGCTGGGATGACC
CTTGCTCCTGTGCAGGGGACAGCACATGTGGTAAAGGGTGGGCCTACAGCCTCCTCTCCT
GTTGTGGCCCAGGTCCCTGCTGCTTTCTACATGCAGTCGGTGCACCTGCCGGGTAAAGCC
CAGACCTTGGCTGTGAAACGCAAAGCTGAGTCTGAGGAGGAGAGAGATGACCTCTCCGCA
TTGGCCTCTGTGCTTCCTACAAAGGCCTCCCCAGCAGCAGAGAGCCCAAAGGTAATAGAG
GAGAAGAATAGTCTTGGAGAGAAAGCTGAACCAGTGGCCAGTTTGAATGCTAATCCTCCA
AACAGTGATCTAGTAGCCTTGGCCCCTACCCCATCAGCACCACCTCCTACTCTGGCCTTG
GTGTCCAGACAGATGGGTGACTCAAAACCCCCACAGGCCATTGTGAAGCCCCAGATTCTC
ACGCACATCATTGAAGGCTTTGTTATCCAGGAAGGAGCAGAGCCTTTTCCGGTGGGATGT
TCTCAGTTCCTGAAAGAAACCAAGAAGCCACTGCAGGCTGGCCTTCCGACCGGGCTGAAT
GAGAGTCAGCCAAGTGGCCCTTTGGGGGGAGACAGCCCCTCTGTAGAGTTAGAGAAGAAG
GCAAATCTCCTCAAGTGTGAGTACTGTGGCAAGTACGCCCCAGCAGAGCAGTTTCGAGGC
TCTAAGAGGTTCTGCTCCATGACGTGTGCAAAGAGGTACAATGTGAGCTGCAGCCACCAG
TTCCGCTTGAAGAGGAAAAAAATGAAAGAGTTTCAGGAAGCCAGCTATGCACGTGTTCGG
AGGCGTGGACCCCGCCGCAGCTCCTCTGATATTGCTCGTGCTAAGATCCAGGGCAAGCGC
CATCGGGGTCAAGAGGACTCTAGCCGGGGATCAGATAATTCCAGCTATGATGAAGCACTC
TCTCCAACATCTCCTGGGCCACTGTCAGTAAGAGCCGGGCACGGAGAACGTGATCTAGGA
AACACCATTACAACCCCATCCACGCCAGAGTTACAGGGCATCAATCCCGTGTTCTTGTCC
AGTAATCCTAGCCAATGGAGCGTCGAGGAGGTCTACGAGTTTATTGCTTCTCTGCAAGGC
TGCCAAGAGATTGCAGAAGAGTTTCGTTCCCAGGAGATTGATGGACAGGCCCTTTTATTA
CTTAAAGAAGAACATCTGATGAGTGCCATGAACATCAAATTAGGTCCTGCTCTCAAGATC
TGTGCCAAGATCAATGTCCTCAAGGAGACC
>Oryctolagus cuniculus XM_002712768.3
ATGGAGACCGAGAGCGAGCAGAACTCCAACTCCACCA
ATGGCAGTTCCAGCTCTGGGGGCAGCTCTCGGCCCCAGATAGCTCAGATGTCCCTGTATGAACGACAAGC
AGTGCAGGCTCTGCAGGCACTACAGCGGCAGCCCAATGCGGCGCAGTACTTCCACCAGTTCATGCTCCAG
CAGCAGCTCAGTAATGCCCAGCTGCACAGCCTGGCTGCCGTCCAGCAGGCCACCATCGCTGCCAGTCGGC
AGGCCAGCTCCCCAAACACCAGCACCACCCAGCAGCAGGCTACCACCACCCAGGCCTCAATCAATCTGGC
CACCACGTCGGCTGCACAGCTCATCAGCCGATCCCAGAGTGTGAGCTCTCCCAGTGCCACCACCTTGACC
CAGTCTGTGCTACTGGGGAACACCACCTCCCCACCCCTCAACCAGTCCCAAGCCCAGATGTATCTACGGC
CACAGCTGGGAAACCTGTTGCAGGTAAACCGGACCCTGGGCCGGAATGTGCCTCTAGCCTCCCAACTCAT
CCTGATGCCTAATGGGGCAGTGGCTGCAGTCCAGCAGGAGGTGCCATCTGCTCAGTCTCCCGGAGTCCAT
GCAGATGCCGATCAGGTTCAGAACTTGGCAGTGAGGAACCAGCAGACCTCAGCCCAAGGACCCCAGATGC
AAGGCTCCACTCAGAAGGCCATTCCTCCCGGAGCCTCCCCTGTCTCTAGCCTCTCCCAGGCCTCTAACCA
GGCCTTGGCTGTGGCACAGGCCTCGTCTGGGGCCTCAGGCCAGTCCCTCAACCTTAGTCAAGCTGGTGGA
GGCAGTGGGAGTAGCATGCCAGGGTCCTTGGGTCCAGGTGGAGGTGGCCAGGCGCCTGCAGGTTTGGGTC
CATTGCCCTCCTCCGGAATGGGTGGTGGAAGCTGTCCCAGGAAGGGCACAGGAGTGGTGCAGCCCTTGCC
TGCAGCCCAAACAGTGACTGTGAGCCAAGGCAGCCAGACAGAGGCAGAAACTGCAGCGGCCAAGAAGGCA
GAAGCAGACGGGAGTGGTCAGCAGAACGTAGGCATGAACCTGACACGGACAGCTACACCTGCTCCCAGTC

AGACACTTATTAGCTCAGCCACGTACACGCAGATCCAGCCCCACTCACTGATTCAGCAGCAGCAGCAGAT
CCACCTGCAGCAGAAGCAGGTGGTGATCCAGCAGCAGATTGCCATTCACCACCAGCAGCAGTTCCAGCAC
CGCCAGTCCCAGCTCCTGCACACAGCGACACACCTCCAGCTGGCCCAGCAGCAGCAGCAGCAGCAGCAAC
AGCAGCAGCAGCAGCAAACCACAACCCTCACTGCCCCCCAGCCACCACAGGTCCCACCTACTCAGCAGGT
CCCCCCTTCTCAGTCCCAGCAACAAGCCCAAACCCTTGTTGTTCAACCGATGCTTCAGTCCTCACCGCTG
TCCCTCCCACCTGACCCAGCCCCTAAGCCACCCATTCCCATCCAGTCCAAACCACCTGCAGCACCTATCA
AACCTCCTCAGTTGGGGGCTGCTAAGATGTCAGCTACCCAACAACCACCCCCCCATATCCCTGTGCAAGT
TGTGGGCACCCGGCAGCCAGGTACAGCCCAGGCACAGGCTTTGGGGTTGGCACAGCTGGCAGCTGCTGTG
CCTACTTCTCGGGGGATGCCAGGTGCAGTGCAGCCTGGTCAAACCCACTTGGCCTCCTCGCCACCTTCAT
CCCAGGCTCCTGGTGCACTGCAGGAGTGCCCTCCTACATTGGCCCCTGGGATGACCCTTGCTCCTGTGCA
AGGGACAGCACATGTGGTAAAGGGTGGTGCTACTACCACCTCACCTGTTGTAGCCCAGGTCCCTGCTGCC
TTCTACATGCAGTCTGTGCACCTGCCGGGTAAACCTCAGACGTTGTCTGTTAAACGCAAGGCTGAGTCTG
AGGAGGAGAGAGACGATGTGTCCACACTGGGTTCAATACTTCCCGCCAAGGCATCTCCAGCCGCAGAGAG
CCCAAAGATCATGGAGGAGAAGAGCAATCTTGGAGAGAAAGCTGAACCAGTGGCCAATGTGAATGCTAAC
ACCCCAAGCAGTGAACTAGTAGCCTTGACCCCTACCCCATCAGCACCACCTCCTACGCTTGCCATGGTCT
CCAGACAAATGGGTGACTCAAAACCCCCACAGGCCATTGTGAAACCTCAGATTCTCACGCACATCATTGA
AGGCTTTGTTATCCAGGAAGGAGCAGAACCTTTCCCGGTGGGCTGTTCTCAGTTACTGAAGGAGTCCGAG
AAGCCACTGCAGACCAGCCTTCCAGCGGGGCTCAGTGAGAATCCTTCAAGTGACCCCTTGGGAGGGGACA
GCCCCTCTGCTGAGCTGGAGAAGAAGGCGAGCCTCCTGAAGTGCGAGTACTGTGGGAAGTACGCCCCTGC
AGAGCAGTTCCGTGGCTCCAAGAGATTCTGCTCCATGACTTGTGCTAAGAGGTACAATGTGAGCTGTAGC
CACCAGTTCCGGCTGAAGAGGAAAAAAATGAAAGAGTTTCAAGAAGCCAACTATGCTCGTGTGCGCCGGC
GTGGACCCCGACGCAGCTCCTCTGACATTGCCCGGGCCAAGATCCAGGGCAAACGCCACCGGGGTCAAGA
GGACTCCAGCCGGGGTTCAGATAATTCCAGTTACGATGAAGCACTCTCCCCAACATCTCCTGGGCCATTA
TCAGTACGCGCTGGGCATGGAGAACGTGACCTGGGGAATCCCATTACAGCTCCACCTACACCGGAATTAC
ATGGCATCAACCCTGTGTTCCTGTCCAGTAATCCCAGCCGTTGGAGTGTAGAGGAGGTGTATGAGTTCAT
TGCTTCTCTCCAAGGTTGCCAAGAGATTGCAGAGGAATTTCGCTCCCAGGAGATTGATGGACAGGCCCTT
TTATTACTTAAGGAAGAGCATCTTATGAGTGCCATGAACATCAAATTGGGCCCTGCCCTCAAGATCTGCG
CCAAGATAAACGTCCTTAAGGAGACC
>rat ENSRNOT00000020529.5
ATGGAAACGGAGAGTGAGCAGAACTCGAGTTCGACCAAT
GGGAGTTCCAGCTCAGGGGCCAGCTCTCGGCCCCAGATAGCACAAATGTCCCTGTATGAG
CGGCAGGCAGTGCAGGCTCTGCAAGCCCTGCAGCGGCAGCCTAATGCAGCTCAGTACTTC
CACCAGTTCATGCTCCAGCAGCAGCTCAGTAATGCCCAGCTGCACAGCCTGGCAGCCGTT
CAGCAGGCCACCATTGCTGCCAGTCGTCAGGCTAGCTCTCCAAACAGCAGCACTGCACAG
CAGCAGACCGCCACCACACAGGCCTCGATCAATCTAGCAACCACGTCAGCTGCTCAGCTC
ATCAGCCGATCCCAGAGTGTGAGCTCCCCCAGTGCTACCACCCTGACTCAGTCTGTGTTA
CTGGGGAACACCACTTCCCCACCTCTCAACCAGTCCCAGGCCCAGATGTATCTGCGGCCA
CAGCTGGGCAACCTATTGCAGGTTAACCGGACCTTGGGCCGGAATGTGCCTCTAGCCTCC
CAGCTCATCCTGATGCCTAATGGGGCAGTGGCTGCAGTCCAGCAGGAGGTGCCACCCGCT
CAGTCTCCTGGAGTTCATGCAGATGCAGATCAGGTGCAGAACTTGGCAGTGAGGAACCAA
CAGGCCTCAGCTCAAGGACCCCAAATACCAGGCTCTACTCAGAAGGCCATCCCTCCTGGA
GCGTCTCCTGTTTCTGGCCTCTCCCAGGCCTCAAGCCAGGCTCTAGCTGTGGCACAGGCT
TCTGGGGCCTCAGGCCAGTCACTCAACCTCAGTCAAGCTGGTGGAGGCAGTGGGAATAGC
CTCCCAGGCTCCATGGGTCCGGGTGGAGGTGGCCAGGCGCCTGGGGGTTTGGGTCAGCTG
CCTTCTTCAGGACTAGCTGGTGGAAACTGTCCCCGGAAGGGTACAGGAGTGGTGCAGCCC
TTACCTGCGGCTCAAACAGTGACTGTGAGCCAGGGAAGCCAAACAGAGGCAGAGAGTGCA
GCGGCCAAGAAGGCAGAAGGAGATGGGAGTAGCCAGCAGAGTGTGGGCATGAACCTGACT

CGGACAGCCACACCTGCCCCCAGCCAGACGCTTATTAGCTCAGCCACATACACACAGATC
CAGCCCCATTCCCTGATTCAGCAACAGCAGCAGATCCACCTCCAGCAGAAGCAAGTGGTG
ATCCAGCAGCAGATTGCCATCCACCACCAGCAACAGTTCCAACACCGCCAGTCCCAGCTA
CTTCACACAGCCACGCACCTCCAGTTGGCCCAGCAGCAGCAGCAGCAGCAGCAACAGCAG
CAGCAGCAGCAGCAGCAGCAGCAGCAGCAGCAGCAGCAGCAGCAGCAGCAACAGCAAGCA
ACAACTCTCGCTGCCCCTCAGCCACCCCAGGTCCCACCTACTCAGCAGGTCCCACCTTCC
CAGTCGCAACAGCAAGCCCAGACTCTGGTGGTCCAACCCATGCTTCAGTCTTCACCCCTG
TCTCTTCCACCTGAACCAACTCCCAAACCACCTATTCCCATTCAGTCCAAACCTCCTGTA
GCTCCTATCAAACCTCCTCAACTAGGAGCTGCTAAGATGTCAGCTACACAGCAGCCACCA
CCACATATCCCTGTGCAAGTTGTCGGTACCCGGCAGCCAGGTACAGCCCAGGCACAGGCT
TTGGGATTAGCGCAACTGGCAGCTGCTGTACCTACTTCCCGGGGGATGCCAGGGGCAGTC
CAGCCTGGCCAGGCCCACCTGGCCTCCTCGCAACCTTCATCCCAGGCTCCTGGTGCACTT
CAAGAATGTCCTCCTGCATTGGCTACTGGGATGACCCTTGCTCCTGTGCAGGGAACGGCA
CATGTGGTAAAGGGTGGGCCTACAGCCTCCTCTCCTGTTGTGGCTCAGGTCCCTGCTGCT
TTCTACATGCAGTCGGTGCACCTGCCGGGTAAAGCCCAGACGTTGGCTGTGAAACGCAAA
GCTGAGTCTGAGGAGGAGAGAGAAGACCTCTCTGCATTGGGCTCTGTGCTTCCTACAAAG
GCATCACCAGCAGCAGAGAGCCCAAAGGTAATAGAGGAGAAGAACAGTCTTGTAGAGAAA
GCTGAACCAGTGGCCAGTTTGAATGCTAATCCTCCAAACAGTGATCTAGTAGCCTTGGCC
CCTACCCCATCAGCACCACCTCCTACTCTGGCCTTGGTGTCCAGACAGATGGGGGACTCA
AAACCCCCACAGGCCATTGTGAAGCCCCAGATTCTCACGCACATCATTGAAGGCTTTGTT
ATCCAGGAAGGAGCAGAGCCTTTCCCGGTGGGATGTTCTCAGTTCCTGAAAGAAACGGAG
AAGCCACTGCCGGCTGGCCTTCCAACAGGGCTGAATGAGAGTCAGTCAGGTGGCCCTTTA
GGGGGTGATAGCCCCTGTGTAGACTTAGATAAGAAGGCAAATCTCCTCAAGTGTGAATAC
TGTGGGAAGTACGCCCCAGCAGAGCAGTTTCGAGGCTCTAAAAGGTTCTGCTCCATGACA
TGTGCAAAGAGGTACAATGTGAGCTGTAGTCACCAGTTCCGCTTGAAGAGGAAAAAAATG
AAAGAGTTTCAGGAAGCCAGCTATGCACGTGTTCGGAGGCGTGGACCCCGCCGCAGCTCC
TCTGACATTGCTCGTGCTAAGATCCAGGGCAAGCGCCATCGGGGTCAAGAGGATTCTAGC
CGGGGCTCAGATAATTCCAGCTATGATGAAGCACTCTCTCCGACATCTCCTGGGCCACTG
TCAGTAAGAGCTGGGCATGGAGAACGTGATCTAGGAAACACCATTACAACCCCGTCCACA
CCAGAGTTACAGGGCATCAACCCTGTGTTCTTGTCCAGTAATCCTAGCCAATGGAGTGTC
GAGGAGGTCTACGAGTTTATTGCTTCTCTGCAAGGTTGCCAAGAGATTGCAGAAGAGTTT
CGTTCCCAGGAGATTGATGGACAGGCCCTTTTATTACTTAAAGAAGAACATCTTATGAGT
GCCATGAACATCAAATTAGGTCCTGCTCTCAAGATTTGTGCCAAGATCAATGTCCTCAAG
GAGACC
>Marmota marmota marmota XM_015506576.1
ATGGAGACTGAGAGCGAGCAGAACTCCAGCTCCACCA
ATGGGAGTTCCAGCTCAGGGGGCAGCTCTCGGCCCCAGATAGCTCAAATGTCCCTGTATGAACGACAAGC
AGTGCAGGCTCTGCAGGCATTGCAGCGGCAGCCCAATGCAGCTCAGTATTTCCACCAGTTCATGCTCCAG
CAGCAGCTCAGTAATGCCCAGCTGCATAGTTTGGCTGCTGTCCAGCAGGCCACGATTGCTGCTAGTCGGC
AAGCCAGCTCCCCAAACACCAGCACTGCACAGCAGCAGACTACCACCACTCAGGCCTCAATTAACCTGGC
CACCACGTCAGCTGCCCAGCTTATCAGCCGATCACAGAGTGTGAGCTCTCCCAGTGCCACCACCTTGACT
CAATCCGTGTTGCTGGGGAATACTACTTCCCCACCTCTCAACCAGTCCCAGGCCCAGATGTATCTGCGGC
CACAGCTGGGAAACCTATTGCAGGTAAACCGGACCCTGGGCCGGAATGTGCCTCTAGCCTCCCAACTCAT
CCTGATGCCTAATGGGGCAGTGGCTGCAGTCCAGCAGGAGGTGCCATCCGCTCAGTCTCCTGGAGTTCAT
GCAGATGCAGATCAGGTGCAGAACTTGGCAGTGAGGAATCAACAGGCCTCAGCTCAAGGACCCCAAATGC
AAGGCTCCACTCAGAAGGCCATACCTCCTGGAGCCTCCCCTGTCTCTAGCCTTTCTCAGGCCTCTAGCCA
GGCCCTAGCTGTGGCACAGGCTTCTCCAGGGGCCTCAGGCCAATCTCTCAACCTCAGTCAAGCTAGTGGA

GGCAGTGGAAACAGCCTCTCAGGGCCAATGGGTCCAGGTGGAGGTGGCCAAGCTCCAGGGGGTTTGGGTC
AGTTGCCTTCTTCAGGAATGGGTGGTGGAAGCTGTCCCAGGAAGGGCACAGGAGTGGTTCAGCCCTTGCC
TGCGGCCCAAACGGTGACTGTGAGCCAGGGCAGCCAGACAGAGGCAGAAAGTGCAGCAGCCAAGAAAGCA
GATGCTGATGGGAGTGGTCAACAGAATGTGGGCATGAACTTGACACGGACAGCTACACCTGCGCCCAGCC
AGACCCTCATTAGCTCAGCCACGTACACGCAGATCCAGCCCCATTCTCTGATTCAGCAGCAGCAGCAGAT
CCACCTCCAGCAGAAGCAGGTAGTGATCCAACAGCAGATTGCCATTCATCACCAGCAACAGTTCCAACAC
CGGCAGTCCCAGCTACTTCACACAGCTACACATCTCCAGTTGGCTCAGCAGCAGCAGCAACAGCAACAAC
AACAGCAGCAGCAACAACAACAACAGCAGCAGCAGCAACAGCAGCAGCAGCAGCAAGCCACAACCCTCAC
TGCCCCTCAGCCACCACAGGTCCCACCTACTCAGCAGGTCCCACCATCCCAGTCCCAGCAGCAAGCTCAA
ACCCTTGTAGTTCAGCCCATGCTTCAGTCTTCACCCCTGTCCCTTCCACCTGACCCAACCCCCAAGCCAC
CCATTCCCATCCAGTCCAAACCACCTGTAGCACCTATTAAACCTCCTCAGTTAGGTGCTGCTAAGATGTC
AGCTACCCAGCAACCACCACCCCATATCCCCGTGCAAGTTGTGGGTACCCGGCAACCAGGTACAGCCCAG
GCACAGGCTTTGGGGTTGGCACAGCTGGCAGCTGCTGTACCTACTTCCCGGGGGATGCCAGGTACAGTGC
AGCCTGGCCAGGCCCATTTGGCCTCCTCACCACCTTCATCCCAGGCTCCTGGTGCACTGCAGGAGTGTTC
TCCTACGTTGGCCCCCGGGATGACCCTTGCTTCTGTGCAGGGGACAGCACATGTGGTTAAGGGTGGGGCT
ACTAGCTCTTCACCTGTTGTAGCCCAGGTTCCTGCTGCCTTCTACATGCAGTCTGTGCATCTACCGGGTA
AACCTCAGACACTGGCTGTGAAACGCAAAGCTGAGTCTGAGGAAGAGAGAGATGATATCTCCACTTTGGG
TTCAATACTTCCTGCCAAGGCTTCTCCAGCAGCAGAGAGCCCAAAAGTCATGGAGGAGAAGAGTAGTCTT
GGAGAGAAAGCTGAACCTGTGGCCAATGTGAATGCTAGTACCCCAAGCAATGAACTGGTAGCCTTGACTC
CTGCCCCATCAGCACCACCTCCTACACTAGCCATGGTGTCCAGACAAATGGGAGACTCCAAACCCCCACA
GGCCATTGTGAAGCCCCAGATTCTCACCCACATCATTGAAGGCTTTGTTATCCAGGAAGGAGCGGAACCT
TTCCCGGTGGGTTGTTCTCAGTTACTGAAGGAGTCTGAGAAGCCACTACAGACTGGCCTCCAAACTGGAC
TGAATGAGAGTCAGTCAAGTGGACCCTTGGGAGGGGACAGCCCTTCTGCTGAGTTAGATAAGAAGGCGAA
TCTCCTGAAGTGCGAGTACTGTGGGAAGTATGCCCCTGCAGAGCAATTTCGTGGCTCTAAGAGGTTTTGC
TCCATGACTTGTGCAAAGAGGTACAATGTGAGCTGTAGCCATCAGTTCCGGTTGAAGAGGAAAAAAATGA
AAGAGTTTCAGGAAGCCAACTATGCTCGTGTTCGTAGGCGTGGACCCCGCCGCAGCTCCTCTGACATTGC
CCGTGCCAAGATCCAGGGCAAGCGTCACCGGGGTCAAGAGGACTCTAGCCGGGGTTCAGATAATTCCAGT
TATGATGAAGCGCTCTCCCCAACATCTCCTGGGCCATTGTCAGTAAGAGCTGGACATGGAGATCGTGACC
TTGGGAACACCATTACAGCTCCCCCCACACCAGAATTACAAGGCATCAACCCTGTGTTCCTGTCCAGCAA
TCCCAGTCGTTGGAGTGTAGAGGAGGTGTATGAGTTTATCGCTTCTCTCCAAGGCTGCCAAGAAATTGCA
GAAGAGTTTCGTTCCCAGGAGATTGATGGACAGGCCCTTTTATTACTTAAAGAAGAACATCTTATGAGTG
CCATGAACATCAAATTGGGCCCTGCCCTCAAGATCTGTGCCAAGATAAATGTCTTGAAGGAGACC
>Chinchilla lanigera XM_005378897.2
ATGGAGACTGAGAGCGAGCAGAACACGGGTTCCACCAACGGGAGTTCCAG
CTCCGGGGGCAGCTCTCGGCCCCAGATAGCGCAGATGTCCCTGTATGAGCGGCAGGCGGTTCAGGCTCTG
CAGGCTCTGCAGCGGCAGCCCAATGCGGCTCAGTATTTCCACCAGTTCATGCTCCAGCAGCAGCTCAGCA
ATGCCCAGCTGCACAGCCTCGCTGCAGTCCAGCAGGCCACGATTGCTGCCAGTCGGCAGGCCAGCTCTCC
GAACACCAGCACCGCGCAGCAGCAGGCGCCCACCACCCAGGCCTCAATCAACCTGGCCACCACCTCCGCT
GCCCAGCTCATCAGCCGATCCCAGAGCGTGAGCTCCCCCAGTGCCACCACCTTGACCCAGTCTGTGCTAC
TGGGGAACACTACCTCCCCACCCCTCAACCAGTCCCAGGCCCAGATGTACCTGCGGCCACAGCTGGGAAA
CCTATTGCAGGTGAACCGGACCCTGGGCCGGAATGTGCCTCTGGCTTCCCAGCTGATCCTGATGCCCAAC
GGGGCGGTGGCCGCTGTCCAGCAGGAGGTGCCCTCGGCTCAGTCTCCCGGAGTTCACACGGATGCAGATC
AGGTGCAGAACTTGGCAGTGAGGAACCAACAGGCCTCTGCTCAAGGGGCCCCAATGCCAGGCTCCTCCCA
GAAGGGCATTCCTCCTGGAGCCTCTCCTGTCTCCGGCCTCCCGCAGGCCTCAAACCAGGCCCTTGCTGTG
GCACAGGCTTCTTCGGGAGCCTCAGGCCAGTCCCTCAACCTCAGTCAGGCTGGTGGAAGCAGTGGGAGTA
GCCTCGCAGGGTCCCTGGGTCCAGGTGGAGGTGGCCAGGCACCTGGGGGTTTGGGTCAGTTGCCTTCCTC
AGGAATGGGCGGGGCAGGCTGTCCCAGGAAGGGCACAGGAGTGGTCCCACCCCTGCCGGCAGCCCAGACA

GTGACTGTGAGCCAGGGCAGCCAGACAGAGGTGGAAAGTGCAGCAGCCAAGAAGGCAGATGCAGATGGGA
GTGGTCAGAATGTGGGCATGAACCTGACGCGGACGGCCACCCCTGCCCCCAGCCAGACTCTTATTAGCTC
AGCTACATACACACAGATCCAGCCCCATTCACTGATTCAGCAGCAGCAGCAGATCCACCTCCAGCAGAAG
CAGGTGGTGATCCAGCAGCAGATCGCCATCCACCACCAGCAGCAGTTCCAGCACCGTCAGTCCCAGCTGC
TGCACACAGCCACACACCTGCAGTTGGCCCAGCAGCAGCAGCAGCAGCAGCAGCAGCAACAGCAACAGCA
GCAGCAGCAGCAGCAGCAGCAACAACAGCAGCAGCAGCAACAAGCCACAACCCTCACTGCCCCTCAACCA
CCACAGATCCCTCCTACTCAGCAGGTCCCACCTTCCCAGTCCCAGCAGCAAGCCCAGACCCTTGTGGTTC
AACCTATGCTTCAGTCTTCACCGCTGTCTCTTCCACCGGACCCAACCCCCAAGCCGCCCATCCCCATCCA
GTCCAAGCCGCCCTTAGCGCCTATCAAACCTCCTCAGTTAGGGGCTACAAAGATGTCAGCTACCCAGCAA
CCACCACCCCATATCCCTGTGCAAGTTGTGGGTACCCGGCAGCCAGGTACAGCCCAGGCACAGGCTTTGG
GCTTGGCACAGCTTGCAGCCACTGCCCCTTCTTCCCGGGGGATGCCAAGTTCAGTGCAGCCTGGCCAGGC
CCACTTGGCCTCCTCCCCACCTTCATCTCAGGCTCCCGGCACACTGCAGGAGTGTCCTCCTACATTGGCT
CCTGGGATGACCCTTGCTCCTGTGCAGGGGACAGCACATGTTGTAAAGGGTGCGGCCACCACATCGTCAC
CTGTGGTTGCCCAGGTCCCCGCTGCCTTCTACATGCAGTCTGTGCACCTACCGGGTAAGCCCCAGACACT
GGCCGTGAAGCGCAAAGCTGAGTCGGAGGAGGAGAGAGACGAGGTTTCCACGTTGGGCTCAATGCTGCCT
ACCAAGGCGTCTTCAGCTGCAGAGAGCCCAAAGGTCATGGAGGAGAAGAGCAGTTTTGCGGAGAAAGCTG
ACCCCGGGGCCAGCATGAATGCCAACCCCCCCAGCAGTGAGCTCGTAGCCCTGGCCCCCACCCCGGGAGC
ACCACCTCCTTCGCTGGCCATGGTGTCCAGACAAATGGGAGACTCGAAGCCCCCGCAGGCCATCGTGAAG
CCTCAAATTCTCACCCACATCATTGAGGGCTTTGTTATCCAGGAAGGAGCAGAACCTTTTCCGGTGGGCT
GTTCTCAGTTACTAAAGGAGTCTGAGAAGCCGCTACAGACCGGCCTCCCAACAGGCCTGAGTGAAAGTCA
GTCAAGTGGCCCCTTAGGAGGGGACAGCCCTTCTGCTGATTTAGATAAGAAGGCAAACCTCCTGAAGTGC
GAGTACTGTGGGAAGTACGCCCCTGCAGAACAGTTCCGAGGCTCTAAGAGGTTCTGCTCCATGACTTGTG
CCAAGAGATACAATGTGAGCTGCAGCCACCAGTTCCGGCTCAAGAGGAAAAAAATGAAAGAGTTTCAAGA
GGCCAACTACGCTCGAGTTCGTAGGCGTGGCCCCGCCGCAGCTCCTCTGACATCGCCCGAGCCAAGATC
CAGGGCAAGCGCCACCGGGGTCAAGAGGATTCCAGCCGGGGTTCAGATAACTCCAGTTATGACGAGGCAC
TCTCCCCTACATCTCCTGGGCCACTGTCAGTACGAGCTGGGCATGGAGAACGTGACCTAGGGAATACCAT
TACGGCACCACCAACGCCAGAGTTACACGGCATAAACCCTGTGTTCCTGTCCAGTAATCCGAGCCGTTGG
AGTGTAGAGGAGGTGTATGAGTTCATAGCTTCTCTCCAAGGCTGCCAAGAGATCGCAGAGGAGTTCCGTT
CCCAGGAGATTGATGGACAGGCTCTTTTATTACTTAAAGAAGAACATCTTATGAGTGCCATGAACATCAA
ACTGGGCCCTGCCCTTAAGATCTGCGCCAAGATAAATGTCCTCAAGGAGACC
>Fukomys damarensis XM_010607807.1
ATGGAGACTGAGAGCGAGCAGAACTCCGGCTCCACCAATGGGGGTTCCAGCTC
TGGGGGCAGCTCTCGGCCCCAGATCGCGCAGATGTCCCTGTACGAGCGGCAGGCGGTCCAGGCTTTGCAG
GCGCTGCAGCGGCAGCCCAATGCGGCTCAGTATTTCCACCAGTTCATGCTTCAACAGCAGCTCAGCAATG
CCCAGCTGCACAGCCTGGCTGCGGTCCAGCAGGCCACGATTGCTGCCAGTCGTCAGGCCAGCTCTCCGAA
CACCAGCACTGCCCAGCCACAGCAGGCGACCACCACGCAGGCCTCCATCAATCTGGCCACCACCTCGGCC
GCGCAGCTCATCAGTCGATCCCAGAGCGTGAGCTCTCCCAGCGCCACCACCTTGACCCAGTCTGTGTTAC
TGGGGAACACCAGCTCCCCACCACTCAACCAGTCCCAGGCGCAGATGTACCTGCGGCCACAGCTGGGAAA
CCTATTGCAGGTAAACCGGACTCTGGGCCGGAATGTGCCTCTGGCCTCCCAGCTGATCCTGATGCCCAAC
GGGGCAGTGGCTGCGGTCCAGCAGGAGGTGCCCTCTGCTCAGTCCCCCGGAGTTCACGCAGATGTGGATC
AGGTGCAGAACTTGGCAGTGAGGAACCAACAGGCCTCTGCTCAAGGAGCCCCGATGCCAGGCTCCTCCCA
GAAGGCCATTCCTCCAGGAGCCTCTCCTGTCTCTGGCTTCTCCCAGACCTCAAACCAGGCACTCGCTGTG
GCACAGGCTTCTTCGGGGGCCTCAGGCCAGTCCCTCAACCTCAGTCAGGCTGGTGGAAGCAGTGGGAGTA
GCCTTGCAGGGTCCCTGGGTCCAGGTGGAGGTAGCCAGGCGCCTGGGGGTTTGGGTCAGTTGCCTTCCTC
AGCAATGGGCGGAGCAGGCTGTCCCAGGAAGGGCACAGGAGTTGTCCCACCCCTGCCAGCAGCCCAGACA
GTGACTGTGAGCCAGGGCAGCCAGACAGAGGTGGAAAGCGCAGCAGCCAAGAAGGCAGATACAGATGGGA
GTGGTCAGAATGTGGGCATGAACCTGACGCGGACAGCCACCCCTGCCCCCAGCCAGACGCTTATTAGCTC

AGCCACATACACACAGATCCAGCCCCACTCGCTGATTCAGCAGCAGCAGCAGATCCACCTCCAGCAGAAG
CAGGTGGTGATCCAGCAGCAGATCGCCATCCACCACCAGCAGCAGTTCCAGCACCGTCAGTCCCAGCTGC
TGCACACAGCCACACACCTCCAGTTGGCCCAGCAGCAGCAGCAGCAGCACCAGCAGCAGCAGCAGCAGCA
GCAGCACCAGCACCAGCAGCAGCAGCAACAGCAGCAGCCACAGCCGCAGCAACAGGCCACCACCCTCTCT
GCCCCTCAGCCGCCACAGGTCCCCCCTACTCAGCAGGTCCCACCTTCCCAGTCCCAGCAGCAAGCCCAGA
CCCTTGTGGTTCAACCCATGCTTCAGTCTTCACCGCTCTCTCTTCCGCCGGACCCAACCCCCAAGCCACC
TATCCCCATCCAGTCCAAGCCGCTCGTAGCGCCTATCAAACCTCCTCAGTTAGGAGCTACGAAGATGTCA
GCTACACAGCAACCGCCACCTCATATCCCTGTGCAAGTCGTAGGTACCCGGCAGCCAGGTACAGCCCAGG
CCCAGGCTCTGGGCTTGGCACAGCTGGCAGCCGCTGCCCCTGCTTCCCGGGGGATGCCAAGTACGGTGCA
GCCTGGCCAGGCCCACTTGGCCTCCTCGCCGCCTTCATCTCAGGCTCCTGGCGCACTGCAGGAGTGTCCC
CCTGTGTTGGCTCCTGGGATGACCCTTGCTCCTGTGCAGGGGACAGCACATGTGGTAAAGGGTGCGGCCA
CCACGTCTTCACCTGTGGTTGCCCAGGTCCCCGCTGCCTTCTACATGCAGTCTGTACACCTGCCGGGTAA
GCCCCAGACACTGGCTGTGAAACGCAAAGCTGAGTCTGAGGAGAGAGACGAGGTCTCCACCTTGGCTCCC
ATGCTTCCTGCCAAGGTGTCTCCAGCCACAGAGAGCCCAAAGGTCGTGGAGGAGAAGAGCAGTCTTTTGG
AGAGAGCTGACCCAGGTGCCAGTGTGAATGCCAACACCCCCAGTAGTGAGCTTGTAGCCCTGGCCCCGGC
CCCAGGAGCACCACCTCCATCGCTGGCCATGGTGTCCAGACAAGTGGGAGACTCGAAACCCCCGCAGGCC
ATCGTGAAGCCTCAGATTCTTACACACATCATTGAGGGCTTTGTCATCCAAGAAGGAGCGGAACCTTTCC
CGGTGGGTTGTTCTCAGTTATTAAAGGAGTCTGAGAAGCCACTACAGACTGGCCTCCCAACAGGGCTGAG
TGAAAGTCAGTCAAGTGGCCCCTTGGGAGGGGACAGTCCTTCTGCCGATTTAGATAAGAAGGCAAACCTC
CTGAAGTGCGAGTACTGTGGGAAGTACGCCCCTGCAGAGCAGTTCCGAGGCTCTAAGAGGTTCTGCTCCA
TGACTTGTGCCAAGAGGTACAATGTGAGCTGCAGCCACCAGTTCCGGCTGAAGAGGAAAAAAATGAAAGA
GTTTCAAGAAGCCAACTATGCTCGAGTTCGTAGGCGTGGACCCCGCCGCAGCTCCTCTGACATCGCCCGA
GCCAAGATCCAGGGCAAGCGTCACCGGGGTCAAGAGGATTCCAGCCGGGGTTCAGATAATTCCAGTTATG
ACGAAGCACTCTCCCCTACGTCTCCTGGGCCTTTGTCAGTACGAGCTGGGCATGGAGACCGTGACCTAGG
GACCACCATTACGGCGCCTCCGACACCTGAATTACACGGCATAAACCCTGTGTTCCTGTCCAGTAATCCC
AGCCGCTGGAGCGTAGAGGAAGTGTATGAGTTCATTGCTTCTCTCCAAGGCTGCCAAGAGATCGCAGAGG
AGTTCCGTTCCCAGGAGATTGATGGACAGGCTCTTTTATTACTTAAGGAAGAACATCTTATGAGTGCCAT
GAACATCAAATTGGGCCCTGCCCTTAAGATCTGTGCTAAGATAAACGTTCTTAAGGAGACT
>Ailuropoda melanoleuca XM_002929196.2
ATGGAGACCGAGAG
TGAGCAGAACTCCAACTCCACTAATGGGAGCTCCAGCTCTGGGGGCAGCTCTCGGCCCCAGATAGCTCAG
ATGTCACTCTATGAACGCCAAGCAGTGCAGGCTCTGCAGGCACTGCAGCGTCAGCCCAACGCAGCTCAGT
ATTTCCACCAGTTCATGCTCCAGCAGCAGCTCAGCAATGCTCAGCTGCATAGCCTGGCTGCGGTCCAGCA
GGCCACGATTGCTGCCAGTCGGCAGGCCAGCTCCCCAAACACCAGCAGTGCACAGCAGCAGACTACCACC
ACCCAGGCCTCAATCAATCTGGCCACCACGTCGGCCGCCCAGCTCATCAGCCGATCCCAGAGTGTGAGCT
CTCCCAGTGCTACCACTTTGACCCAATCTGTGCTACTGGGGAACACCACCTCCCCGCCTCTCAACCAATC
CCAGGCCCAGATGTATCTACGGCCACAGCTGGGAAACCTATTGCAGGTAAACCGGACCCTGGGCCGGAAT
GTGCCTCTAGCCTCCCAACTCATCCTGATGCCTAACGGGGCGGTGGCTGCGGTCCAGCAGGAGGTGCCAT
CTGCTCAGTCTCCGGGAGTTCATACAGATGCAGATCAGGTGCAGAATTTGGCAGTGAGGAACCAACAGGC
CTCAGCCCAAGGACCCCAAATGCAGGGCTCTACTCAGAAGGCCATTCCTCCTGGAGCCTCCCCTGTCTCT
AGCCTCTCCCAGGCCTCTAGCCAGGCCCTCGCTGTGGCTCAGGCTTCCTCTGGGGCCTCGGGCCAGTCTC
TCAACCTCAGTCAAGCTGGTGCGGGCAGTGGGAGTAGCATCCCAGGGTCCATGGGGCCAGGCGGAGGTGG
CCAGGCACCTGGGGGCTTGGGTCAGTTGCCTTCCTCAGGAATGGGTGGGGGTGGGAGCTGTCCTAGGAAG
GGCACAGGAGTGGTGCAGCCCTTGCCTGCAGCCCAAACAGTGACTGTGAGTCAGGGAAGCCAGACAGAAG
CAGAAAGTGCAGCGGCCAAGAAGGCAGAAGCAGATGGGGCTGGTCAGCAGAACGTGGGCATGAACCTGAC
ACGGACAGCTACGCCTGCTCCCAGCCAGACCCTTATTAGCTCAGCCACCTACACACAGATCCAGCCCCAT
TCCCTGATTCAGCAACAGCAGCAGATCCACCTCCAGCAGAAACAGGTGGTAATCCAGCAGCAGATCGCCA

TCCACCACCAGCAGCAGTTCCAGCACCGGCAGTCCCAGCTCCTCCACACAGCCACACACCTCCAGCTGGC
CCAGCAGCAGCAGCAGCAACAGCAGCAGCAGCCGCCGCCGCCGCCTCCGCCCCCGCCGCCAGCCACCACC
CTCACTGCCCCTCAGCCACCGCAGGTCCCACCCACTCAGCAGGTCCCGCCTTCCCAGTCCCAGCAGCAAG
CCCAGACCCTGGTCGTTCAACCCATGCTTCAGTCTTCACCCTTGTCCCTACCCGACCCAACCCCCAAGCC
ACCCATCCCCATCCAGTCCAAACCACCTGTGGCACCTATCAAGCCTCCTCAGTTAGGAGCTGCTAAGATG
TCAGCTACCCAGCAACCACCACCCCACATCCCTGTGCAAGTTGTGGGCACCCGACAGCCGGGGACGGCGC
AGGCACAGGCTTTGGGGTTGGCACAGCTGGCGGCTGCCGTACCTACTTCCCGGGGGATGCCCAGTACAGT
GCAGCCCGGTCAGGCCCACTTGGCCTCCTCACCACCTTCATCCCAGGCTCCCGGGGCGCTGCAAGAGTGC
CCTCCCCCTTTGGCGTCGGGGATGAGCCTGGCTCCCGTGCAGGGGACGGCCCATGTGGTAAAGGGTGGGG
CTACGACCTCCTCACCTGTCGTGGCCCAGGTACCTGCTGCCTTCTACATGCAGTCTGTGCACCTGCCGAG
CAAACCCCAGACACTGGCTGTCAAACGCAAGGCAGAGTCTGAGGAGGAGAGAGACGATGTCTCGACATTG
GGTTCAATGCTTCCAGCCAAGGCATCTCCAGTAGCTGAGAGCCCAAAGGCCGTGGAGGAGAAGAGCAGTC
TTGGAGAGAAAGCTGAACCAGTGACTGGTGTGAATGCTAACACTCCAAGCAGTGAACTAGTAGCCTTGGC
TCCTGCCCCATCCGCACCCCCTCCTACACTAGCCATGGTGTCCAGACAAATGGGTGACTCAAAACCCCCA
CAGGCCATCGTGAAGCCCCAGATTCTCACCCACATCATTGAAGGCTTCGTTATCCAGGAAGGAGCAGAGC
CTTTCCCGGTGGGTTGTTCTCAATTACTGAAAGAGTCTGAGAAGCCACTACAGACTGGCCTCCCGACGGG
GCTGAATGAGAATCAGTCAGGGGGACCCTTGGGAGGGGACAGCCCATCTGCTGAGCTAGACAAGAAGGCG
AATCTCCTCAAGTGTGAGTACTGCGGGAAGTACGCTCCTGCCGAGCAGTTTCGCGGCTCCAAGAGGTTCT
GCTCAATGACCTGCGCTAAGAGGTATAACGTGAGCTGTAGTCACCAGTTCCGGCTGAAAAGGAAAAAAAT
GAAAGAGTTCCAAGAAGCCAACTATGCTCGCGTTCGTCGGCGTGGACCCCGCCGCAGCTCCTCTGACATC
GCCCGTGCCAAGATCCAGGGCAAGCGCCACCGGGGTCAAGAGGACTCTAGCCGGGGTTCAGATAATTCCA
GTTATGATGAAGCACTCTCTCCAACATCTCCTGGGCCTTTATCTGTGAGAACTGGGCATGGAGAACGTGA
CCTGGGGAACCCCAATACAGCGCCACCAACACCGGAATTACATGGCATCAACCCCGTGTTCCTGTCCAGC
AATCCCAGCCGCTGGAGCGTAGAAGAGGTCTATGAGTTTATCGCTTCTCTACAAGGCTGCCAAGAGATTG
CAGAGGAGTTTCGTTCCCAGGAGATTGATGGACAGGCCCTCTTATTACTTAAAGAGGAACATCTTATGAG
TGCCATGAACATCAAGTTGGGCCCTGCCCTGAAGATCTGCGCCAAGATAAACGTGCTCAAGGAGACC

>cow ENSBTAT00000024230.5
ATGGAGACTGAGAGTGAACAGAACTCCAACTCCACCAATGGGAGTTCTGGCTCTGGGGGCAGC
TCTCGGCCCCAGATCGCTCAAATGTCACTGTATGAACGGCAGGCTGTGCAGGCTCTGCAG
GCACTGCAGCGTCAGCCCAACGCGGCTCAGTATTTCCACCAGTTCATGCTCCAGCAGCAG
CTCAGCAATGCCCAGCTGCATAGCCTGGCTGCCGTCCAGCAGGCTACAATTGCTGCCAGT
CGGCAGGCCAGCTCCCCAAACACCAGCACTGCACAGCAGCAGACCACCACCACCCAGGCC
TCCATCAATCTGGCCACCACATCGGCTGCCCAGCTCATCAGCCGGTCCCAGAGTGTGAGC
TCTCCCAGTGCTACCACTTTGACCCAGTCTGTGCTACTGGGGAACACCACCTCCCCACCC
CTCAACCAGTCCCAGGCCCAGATGTATCTACGGCCACAGCTGGGAAACCTATTGCAGGTA
AACCGGACCCTGGGCCGGAATGTGCCTCTAGCCTCCCAACTCATCCTGATGCCTAACGGG
GCAGTGGCTGCAGTCCAGCAGGAGGCGCCATCTGCTCAGTCTCCTGGAGTCCACACAGAT
GCTGATCAGGTGCAGAACTTGGCCGTCAGGAACCAGCAGGCCTCTGCCCAAGGACCCCAA
ATGCAAGGCTCTGCCCAGAAGGCCATCCCTCCTGGGGCCTCCCCTGTCTCCAGCCTCTCC
CAGGCCTCTAGCCAGGCCCTTGCGGTGGCTCAGGCTTCCTCTGGGGCCTCAGGCCAGTCC
CTCAACCTCAGTCAAGCTGGTGGAGGCAGTGGGACTAGCATCCCAGGGTCCATGGGTCCT
GGAGGAGGCGGCCAGGCTCCTGGGGGCTTGGGGCAGTTACCTTCCTCAGGAATGGGTGGT
GGTGGGAGCTGCCCCAGGAAGGGCACTGGAGTGGTGCAGCCCTTGCCTGCAGCCCAGGCA
GTGACCGTGAGTCAGGGCAGCCAGACAGAAGCAGAAAGCGCAGCAGCCAAGAAAGCAGAA
GCAGACGGAACTGGTCAGCAGAACGTGGGCATGAATCTCACCCGGACCGCCACTCCTGCT
CCCAGCCAGACCCTCATTAGCTCAGCCACCTACACGCAGATCCAGCCCCACTCCCTGATT

CAGCAGCAGCAGCAGATCCACCTCCAGCAGAAGCAGGTGGTGATCCAGCAGCAGATCGCC
ATCCACCACCAGCAGCAGTTCCCGCACCGCCAGTCCCAGCTTCTGCACACAGCCACGCAC
CTCCAGCTGGCCCAGCAGCAGCAGCAGCAGCAGCAGCAACAGCAGGCCACCACCCTCACT
GCCCCGCAGCCTCCGCCGGGTCCCCCGACCCAGCCGGTCCCGCCGTCCCCATCCCAGCAG
CCAGCCCAGACCCTGGTCGTCCAGCCCATGCTGCAGTCTTCACCGCTGTCCCTGCCGCCG
GACCCCGCCCCCAAGCCCCCTATTCCCATCCAGTCCAAACCGCCGGCAGCCCCCCTCAAG
CCCCCTCAGCTAGGGGCTGCCAAGATGTCTGCCGCCCAGCAGCCCCCACCCCACATCCCT
GTGCAGGTGGTGGGCACCCGGCAGCCAGGTACAGCCCAGGCCCAGGCTTTGGGGCTGGCC
CAGCTGGCAGCTGCAGTCCCCACTTCCCGGGGGATGCCAGGCACAGTGCCGCCAGGCCAG
GCCCACTTGGCCTCCTCGCCGCCTTCATCCCAGGCCCCCGGTGCGCTGCAGGAGTGCCCA
CCCACGTTGGCCTCAGGGATGACCCTGGCCCCTGTGCAGGGGACGGCGCATGTGGTGAAG
GGAGGGGCGACCACCTCCTCACCGGTGGTCGCGCAGGTCCCTGCCGCCTTCTACATGCAG
TCTGTGCACCTGCCGGGCAAACCCCAGACACTGGCTGTAAAGCGCAAGGCTGAGTCTGAG
GAGGAGCGAGATGATGTCTCCACACTGAGTTCAATGATTCCCGCGAAGACATCGCCAGTG
GTAGAGAGCCCAAAGGCCATGGAGGAGAAGGGTGGTCTTGGAGAGAAAGCTGAGCCAGTG
ACCAGCGCGAACACTAACACCCTGAGCAGTGACATTGTAGCTTTGGCTCCTGCCCCATCA
GCGCCCCCTCCTTCGCTGGCCATGGTGTCCAGACAGATGGGTGACTCCAAGCCCCCCCAG
GCCATTGTGAAGCCCCAGATCCTCACTCACATCATTGAAGGCTTCGTTATCCAGGAAGGA
GCAGAGCCTTTCCCGGTGGGCTGTTCTCAGTTATTGAAAGAGTCTGAGAAGCCCCTGCAG
ACTGGCCTCACCACAGGACTGAATGAGAATCAGTCGGGTGGTCCCTTGGGTGGGGACAGC
CCATCTGCTGAGCTAGATAAGAAGGCAAACCTCCTCAAGTGCGAGTACTGTGGGAAGTAC
GCCCCCGCCGAGCAATTCCGCGGCTCCAAGAGATTCTGCTCCATGACTTGTGCTAAGAGG
TATAATGTGAGCTGTAGCCACCAATTCCGGCTGAAGAGGAAAAAAATGAAAGAATTTCAG
GAAGCCAACTATGCCCGTGTTCGCAGGCGCGGACCCCGCCGCAGCTCCTCGGACATCGCC
CGTGCCAAGATCCAGGGCAAGCGCCACCGAGGTCAAGAGGACTCTAGCCGGGGTTCAGAT
AATTCCAGTTACGATGAAGCACTCTCTCCAACATCTCCTGGGCCTTTATCCGTGAGAGCA
GGGCATGGAGAACGTGATCTGGGGAATCCCAACACAGCTCCACCCACCCCAGAATTACAT
GGCATCAACCCTGTGTTCCTATCCAGTAATCCTAGCCGCTGGAGCGTAGAGGAAGTCTAT
GAGTTCATTGCTTCTCTACAAGGCTGCCAAGAGATTGCAGAAGAGTTTCGTTCCCAGGAG
ATCGATGGACAGGCCCTCTTATTACTTAAAGAGGAACATCTCATGAGTGCCATGAACATC
AAGCTGGGTCCTGCCCTCAAGATCTGTGCTAAGATAAATGTCCTCAAGGAGACC

>Equus asinus XM_014850959.1
ATGGAGACTGAGAGTGAGCAGAACTCTAGCTCCACCA
ATGGGAGTTCCAGCTCTGGGGGCAGCTCTCGGCCCCAGATAGCTCAGATGTCACTGTATGAACGACAAGC
AGTGCAGGCCCTGCAGGCGCTGCAGCGTCAGCCTAATGCGGCTCAGTATTTCCACCAGTTCATGCTTCAG
CAGCAGCTCAGCAATGCCCAGCTGCATAGCCTGGCTGCCGTCCAGCAGGCCACAATTGCTGCCAGTCGGC
AGGCCAGCTCTCCAAACACCAGCACCGCACAGCAGCAGACTACCACCACCCAGGCCTCAATCAATCTGGC
CACCACGTCGGCCGCGCAGCTCATCAGCCGATCCCAGAGTGTGAGCTCTCCCAGTGCTACCACTTTGACC
CAATCTGTGCTACTGGGGAACACCACCTCCCCACCCCTCAACCAGTCCCAGGCCCAGATGTATCTACGGC
CACAGCTGGGAAACCTATTGCAGGTAAACCGGACCCTGGGCCGGAATGTGCCTCTAGCCTCCCAGCTCAT
CCTGATGCCTAACGGGGCAGTGGCTGCAGTCCAGCAGGAGGTGCCATCTGCTCAGTCTCCCGGAGTTCAT
ACAGATGCAGATCAGGTGCAGAACTTGGCAGTGAGGAACCAACAGGCCTCAGCCCAAGGACCCCAAATGC
AAGGCTCTACTCAGAAGGCCATTCCTCCTGGAGCCTCCCCTGTCTCTAGCCTGTCCCAGGCCTCTAGCCA
GGCCCTAGCTGTGGCGCAGGCTTCCTCTGGGGCCTCAGGCCAGTCCCTCAACCTTAGTCAGGCTGGTGGA
GGCAGTGGTAATAGCATCCCAGGGCCCATGGGTCCAGGTGGAGGTGGCCAGGCACCTGGGGGCTTGGGGC
AGTTACCTTCCTCAGGAATGGGTGGTGGTGGGAGCTGTCCCAGGAAGGGCACAGGAGTGGTGCAGCCCTT

GCCTGCAGCCCAAACAGTGACTGTGAGTCAGGGCAGCCAGACAGAAGCAGAAAGTGCAGCGGCCAAGAAG
GCGGAAGCAGATGGGTCTGGTCAGCAGAATGTGGGCATGAACCTGACACGGACAGCTACACCTGCTCCCA
GCCAGACCCTTATTAGCTCAGCCACCTACACACAGATCCAGCCCCATTCTCTGATTCAGCAACAGCAACA
GATCCACCTCCAGCAGAAACAGGTGGTAATCCAGCAGCAGATTGCCATCCACCACCAGCAACAGTTCCAG
CACCGTCAATCCCAGCTACTCCACACAGCCACACACCTCCAGTTGGCGCAGCAGCAGCAGCAGCAGCAGC
AGCAGCAGCAGCAGCAGCAAGCCACAACTCTCACTGCCCCTCAAACACCACAGGTCCCACCTACTCAGCA
GGTCCCACCTTCCCAGTCCCAGCAGCAAGCCCAAACCCTGGTTGTTCAACCTATGCTTCAGTCTTCACCC
ATGTCCCTCCCACCTGATCCAACCCCCAAGCCGCCCATCCCCATCCAGTCCAAACCACCTGTAGCACCTA
TCAAGCCTCCTCAGTTAGGGGCTGCTAAGATGTCAGCTACCCAGCAACCACCACCCCATATTCCTGTGCA
AGTTGTGGGCACCCGACAGCCAGGTACAGCCCAGGCACAGGCTTTGGGGTTGGCACAGCTGGCAGCCGCT
GTACCTACTTCCCGGGGGATGCCAGGTACAGTGCAGCCTGGTCAGGCCCATTTGGCCTCCTCGCCACCTT
CATCCCAGACTCCTGGTGCACTGCAGGAGTGCCCTCCCACATTGGCCTCTGGCATGACCCTTGCTCCTGT
GCAGGGGACAGCACATGTGGTAAAGGGTGGGGCTACCACCTCTTCACCTGTTGTAGCCCAGGTCCCTGCT
GCCTTCTACATGCAGTCTGTGCACCTGCCGGGCAAACCTCAGACACTGGCTGTCAAACGCAAGGCTGAGT
CTGAGGAGGAGAGAGATGACGTTTCCACACTGGGTTCAATGCTTCCTGCCAAGGCATCTCCAGTAGCAGA
GAGCCCAAAGGTCATGGAGGAGAAGAGTAGTCTTGGAGAGAAAGCTGAACCAGTGACCAGTGTGAATGCT
AATACCCCAAGCAGTGAACTAGTAGCCTTGGCTCCTGCCCCATCAGCGCCACCTCCTACGCTAGCCATGG
TGTCCAGACAGATGGGTGACTCAAGACCCCCACAGGCCATTGTGAAGCCTCAGATTCTCACCCACATCAT
TGAAGGCTTCGTTATCCAGGAAGGAGCAGAACCTTTCCCGGTGGGTTGTTCTCAGTTACTGAAAGAGTCT
GAGAAGCCACTACAGACGGGCCTCTCGACAGGGCTGAATGAGAATCAGTCAGGTGGCCCCTTGGGAGGGG
ACAGCCCATCTGCTGAGCTGGATAAGAAGGCGAATCTCCTGAAGTGTGAGTACTGTGGGAAGTATGCCCC
TGCAGAGCAGTTTCGCGGCTCCAAGAGATTCTGCTCCATGACTTGCGCTAAGAGGTATAATGTGAGCTGT
AGCCACCAGTTCCGGCTCAAGAGGAAAAAAATGAAAGAGTTTCAAGAAGCCAACTACGCTCGTGTTCGTC
GGCGTGGACCCCGCCGCAGCTCCTCTGACATTGCCCGTGCCAAGATCCAGGGCAAGCGCCACCGGGGTCA
AGAGGACTCTAGCCGGGGTTCAGATAATTCCAGTTACGATGAAGCACTCTCTCCAACATCTCCTGGGCCT
TTATCAGTGAGAGCTGGGCATGGAGAACGTGACCTGGGGAACCCCAATACAGCTCCGCCTACACCAGAAT
TACATGGCATCAACCCTGTGTTCCTGTCCAGTAATCCGAGCCGTTGGAGTGTGGAGGAGGTGTATGAGTT
TATCGCTTCTCTACAAGGCTGCCAAGAGATTGCAGAGGAGTTTCGTTCCCAGGAGATTGATGGACAGGCC
CTTTTATTACTTAAAGAGGAACATCTTATGAGTGCCATGAACATCAAGCTGGGCCCTGCCCTCAAGATCT
GCGCCAAGATAAATGTCCTCAAGGAGACC
>Equus caballus XM_005610913.1
ATGGAGACTGAGAGTGAGCAGAACTC
TAGCTCCACCAATGGGAGTTCCAGCTCTGGGGGCAGCTCTCGGCCCCAGATAGCTCAGATGTCACTGTAT
GAACGACAAGCAGTGCAGGCCCTGCAGGCGCTGCAGCGTCAGCCTAATGCGGCTCAGTATTTCCACCAGT
TCATGCTTCAGCAGCAGCTCAGCAATGCCCAGCTGCATAGCCTGGCTGCCGTCCAGCAGGCCACAATTGC
TGCCAGTCGGCAGGCCAGCTCTCCAAACACCAGCACCGCACAGCAGCAGACTACCACCACCCAGGCCTCA
ATCAATCTGGCCACCACGTCGGCCGCGCAGCTCATCAGCCGATCCCAGAGTGTGAGCTCTCCCAGTGCTA
CCACTTTGACCCAATCTGTGCTACTGGGGAACACCACCTCCCCACCCCTCAACCAGTCCCAGGCCCAGAT
GTATCTACGGCCACAGCTGGGAAACCTATTGCAGGTAAACCGGACCCTGGGCCGGAATGTGCCTCTAGCC
TCCCAGCTCATCCTGATGCCTAACGGGGCAGTGGCTGCAGTCCAGCAGGAGGTGCCATCTGCTCAGTCTC
CCGGAGTTCATACAGATGCAGATCAGGTGCAGAACTTGGCAGTGAGGAACCAACAGGCCTCAGCCCAAGG
ACCCCAAATGCAAGGCTCTACTCAGAAGGCCATTCCTCCTGGAGCCTCCCCTGTCTCTAGCCTGTCCCAG
GCCTCTAGCCAGGCCCTAGCTGTGGCGCAGGCTTCCTCTGGGGCCTCAGGCCAGTCCCTCAACCTTAGTC
AGGCTGGTGGAGGCAGTGGTAATAGCATCCCAGGGCCCATGGGTCCAGGTGGAGGTGGCCAGGCACCTGG
GGGCTTGGGGCAGTTACCTTCCTCAGGAATGGGTGGTGGTGGGAGCTGTCCCAGGAAGGGCACAGGAGTG
GTGCAGCCCTTGCCTGCAGCCCAAACAGTGACTGTGAGTCAGGGCAGCCAGACAGAAGCAGAAAGTGCAG
CGGCCAAGAAGGCGGAAGCAGATGGGTCTGGTCAGCAGAATGTGGGCATGAACCTGACACGGACAGCTAC

ACCTGCTCCCAGCCAGACCCTTATTAGCTCAGCCACCTACACACAGATCCAGCCCCATTCTCTGATTCAG
CAACAGCAACAGATCCACCTCCAGCAGAAACAGGTGGTAATCCAGCAGCAGATTGCCATCCACCACCAGC
AACAGTTCCAGCACCGTCAATCCCAGCTACTCCACACAGCCACACACCTCCAGTTGGCGCAGCAGCAGCA
GCAGCAGCAGCAGCAGCAGCCACAGCAGCAAGCCACAACTCTCACTGCCCCTCAAACACCACAGGTCCCA
CCTACTCAGCAGGTCCCACCTTCCCAGTCCCAGCAGCAAGCCCAAACCCTGGTTGTTCAACCTATGCTTC
AGTCTTCACCCATGTCCCTCCCACCTGATCCAACCCCCAAGCCACCCATCCCCATCCAGTCCAAACCACC
TGTAGCACCTATCAAGCCTCCTCAGTTAGGGGCTGCTAAGATGTCAGCTACCCAGCAACCACCACCCCAT
ATTCCTGTGCAAGTTGTGGGCACCCGACAGCCAGGTACAGCCCAGGCACAGGCTTTGGGGTTGGCACAGC
TGGCAGCCGCTGTACCTACTTCCCGGGGGATGCCAGGTACAGTGCAGCCTGGTCAGGCCCATTTGGCCTC
CTCGCCACCTTCATCCCAGACTCCTGGTGCACTGCAGGAGTGCCCTCCCACATTGGCCTCTGGCATGACC
CTTGCTCCTGTGCAGGGGACAGCACATGTGGTAAAGGGTGGGGCTACCACCTCTTCACCTGTTGTAGCCC
AGGTCCCTGCTGCCTTCTACATGCAGTCTGTGCACCTGCCGGGCAAACCTCAGACACTGGCTGTCAAACG
CAAGGCTGAGTCTGAGGAGGAGAGAGATGACGTTTCCACACTGGGTTCAATGCTTCCTGCCAAGGCATCT
CCAGTAGCAGAGAGCCCAAAAGTCATGGAGGAGAAGAGTAGTCTTGGAGAGAAAGCTGAACCAGTGACCA
GTGTGAATGCTAATACCCCAAGCAGTGAACTAGTAGCCTTGGCTCCTGCCCCATCAGCGCCACCTCCTAC
GCTAGCCATGGTGTCCAGACAGATGGGTGACTCAAGACCCCCACAGGCCATTGTGAAGCCTCAGATTCTC
ACCCACATCATTGAAGGCTTCGTTATCCAGGAAGGAGCAGAACCTTTCCCGGTGGGTTGTTCTCAGTTAC
TGAAAGAGTCTGAGAAGCCACTACAGACGGGCCTCTCGACAGGGCTGAATGAGAATCAGTCAGGTGGCCC
CTTGGGAGGGGACAGCCCATCTGCTGAGCTGGATAAGAAGGCGAATCTCCTGAAGTGTGAGTACTGTGGG
AAGTACGCCCCTGCAGAGCGGTTTCGCGGCTCCAAGAGATTCTGCTCCATGACTTGCGCTAAGAGGTATA
ATGTGAGCTGTAGCCACCAGTTCCGGCTCAAGAGGAAAAAAATGAAAGAGTTTCAAGAAGCCAACTACGC
TCGTGTTCGTCGGCGTGGACCCCGCCGCAGCTCCTCTGACATTGCCCGTGCCAAGATCCAGGGCAAGCGC
CACCGGGGTCAAGAGGACTCTAGCCGGGGTTCAGATAATTCCAGTTACGATGAAGCACTCTCTCCAACAT
CTCCTGGGCCTTTATCAGTGAGAGCTGGGCATGGAGAACGTGACCTGGGGAACCCCAATACAGCTCCGCC
TACACCAGAATTACATGGCATCAACCCTGTGTTCCTGTCCAGTAATCCGAGCCGTTGGAGTGTGGAGGAG
GTGTATGAGTTTATCGCTTCTCTACAAGGCTGCCAAGAGATTGCAGAGGAGTTTCGTTCCCAGGAGATTG
ATGGACAGGCCCTTTTATTACTTAAAGAGGAACATCTTATGAGTGCCATGAACATCAAGCTGGGCCCTGC
CCTCAAGATCTGCGCCAAGATAAATGTCCTCAAGGAGACC
>cat ENSFCAT00000015281.3
ATGGAGACGGAGAGTGAGCAGAACTCTAACTCCACCAATGGGAGCTCCAGCTCTGGGGGC
AGCTCTCGGCCCCAGATAGCTCAGATGTCGCTGTATGAACGGCAAGCAGTGCAGGCTCTG
CAGGCACTGCAGCGTCAGCCCAACGCGGCTCAGTATTTCCACCAGTTCATGCTCCAGCAG
CAGCTCAGCAATGCCCAGCTGCATAGCCTGGCCGCCGTCCAGCAGGCCACGATCGCTGCC
AGTCGGCAGGCCAGCTCCCCAAACAGCACCACACAGCAGCAGACTACCACCACCCAGGCC
TCAATCAATCTGGCCACCACGTCGGCCGCCCAGCTCATCAGCCGATCGCAGAGTGTGAGC
TCTCCCAGTGCTACCACTTTGACCCAATCTGTGCTACTGGGGAACACCACCTCCCCACCT
CTCAACCAGTCTCAGGCCCAGATGTATCTACGGCCACAGCTGGGAAACCTATTGCAGGTA
AACCGGACCCTGGGCCGGAATGTGCCTCTAGCCTCCCAGCTCATCCTGATGCCTAACGGG
GCGGTGGCTGCAGTCCAGCAGGAGGTGCCATCGGCTCAGTCTCCGGGAGTCCATGCAGAT
GCAGATCAGGTGCAGAATTTGGCAGTGAGGAACCAACAGGCCTCAGCCCAAGGACCCCAA
ATGCAAGGCTCTGCTCAGAAGGCCGTTCCTCCTGGAGCCTCCCCTGTGTCTAGCCTCTCC
CAGGCCTCTAGCCAGGCCCTAGCTGTGGCTCAGGCATCCTCTGGGGCTTCAGGCCAGTCC
CTGAACCTCAGTCAAGCTGGTGGAGGCAGCGGAAGTAGCATCCCAGGGTCCATGGGTCCA
GGTGGAGGTGGCCAGGCACCTGGGGGCTTGGGTCAGTTGCCTTCCTCAGGAATGGGTGGT
GGTGGGAGCTGTCCCAGGAAGGGCACAGGAGTGGTGCAGCCCTTGCCTGCAGCCCAGACA
GTGACTGTGAGTCAGGGAAGCCAGACGGAAGCAGAAAGTGCAGCGGCCAAGAAGGCAGAA
GCAGATGGGGCTGGTCAGCAGAACGTGGGCATGAACCTGACACGGACAGCTACACCTGCT

CCCAGCCAGACCCTTATTAGCTCAGCCACCTACACGCAGATCCAGCCCCACTCCCTGATC
CAGCAGCAGCAGCAGATCCACCTCCAGCAGAAACAGGTGGTGATCCAGCAGCAGATCGCC
ATCCACCACCAGCAGCAGTTCCAGCACCGCCAGTCCCAGCTCCTTCACACCGCCACGCAC
CTCCAGTTGGCCCAGCAGCAGCAGCAACAGCAGCAGCCGCCGCCGCCCCACCGCCGCCG
CCGCCGCCCCCGCCGCCGGCCACGACTCTCACTGCCCCTCAGCCGCCGCAGGTCCCGCCT
ACTCAGCAGGTCCCGCCTTCCCAGTCCCAGCAGCAAGCCCAGACCCTGGTGGTTCAGCCC
ATGCTTCAGTCTTCACCCTTGTCCCTCCCGCCCGACCCGACCCCCAAGCCGCCCATCCCC
ATCCAGTCCAAGCCGCCGGGAGCACCTATCAAGCCTCCTCAGTTAGGGGCTGCGAAGATG
GCGGCCACCCAGCAACCACCACCCCATATCCCCGTGCAAGTCGTGGGCACCCGGCAGCCG
GGGACAGCCCAGGCGCAGGCCCTGGGGTTGGCACAGCTGGCGGCCGCCGTACCTACTTCC
CGGGGGATGCCCAGCACAGCGCAGCCTGGTCAGGCCCACTTGGCCTCCTCGCCACCTTCG
TCCCAGGCTCCTGGCGCGCCGCAAGAGTGCCCTCCCGCCTTGGCCTCCGGGATGACCCTT
GCTCCTGTGCAGGGGACGGCGCACGTGGTAAAGGGCGGGGCTACCACCTCCTCACCTGTC
GTAGCCCAGGTGCCTGCCGCCTTCTACATGCAGTCCGTGCACCTGCCGAGCAAACCCCAG
ACGTTGGCTGTCAAACGGAAGGCAGAATCTGAGGAGGAGAGAGACGATGTCTCCACATTG
GGTTCAATGCTTCCTGCCAAGGCATCTCCAGCAGCAGAGAGCCCAAAGGCCCTGGAGGAG
AAGAGCAGTCTTGGAGAGAAAGCTGAACCAGTGACCGGTGTGAATGCTAATACTCCAAGC
AGTGAACTAGTAGCCTTGGCTCCTGCCCCGTCAGCACCCCCTCCTACACTAGCCATGGTG
TCCAGACAGATGGGTGACTCAAAACCCCCACAGGCCATCGTGAAGCCCCAGATTCTCACC
CACATCATTGAAGGCTTCGTTATCCAGGAAGGAGCAGAGCCTTTCCCGGTGGGTTGTTCT
CAGTTACTGAAAGAGTCTGAGAAGCCGCTGCAGACTGGCCTCCCGACGGGGCTGAATGAG
AATCAGTCAGGTGGCCCCTTGGGAGGGGACAGCCCATCGGCTGAGCTGGATAAGAAGGCG
AATCTCCTGAAGTGTGAGTACTGCGGGAAGTATGCTCCCGCAGAGCAGTTTCGCGGCTCC
AAGAGGTTCTGCTCCATGACTTGCGCTAAGAGGTATAACGTGAGCTGTAGTCACCAGTTC
CGGCTGAAGAGGAAAAAAATGAAAGAGTTCCAAGAAGCCAACTATGCTCGTGTTCGTCGG
CGTGGACCCCGCCGTAGCTCCTCCGACATCGCCCGCGCCAAGATCCAGGGCAAGCGCCAC
CGGGGTCAAGAGGACTCCAGCAGGGGTTCGGATAATTCCAGTTACGACGAAGCACTCTCT
CCGACATCTCCTGGGCCTTTATCTGTGAGAACTGGGCATGGAGAACGTGACCTGGGGACC
CCCAATACGGTACCACCGACGCCGGAATTACATGGCATCAACCCTGTGTTCCTGTCCAGC
AATCCGAGCCGCTGGAGCGTAGAAGAGGTGTATGAGTTCATCGCTTCTCTACAAGGCTGC
CAAGAGATTGCAGAGGAGTTCCGTTCCCAGGAGATTGATGGACAGGCCCTTTTATTACTT
AAAGAGGAACATCTCATGAGTGCCATGAACATCAAGTTGGGCCCTGCCCTCAAGATCTGC
GCCAAGATAAACGTCCTCAAGGAGACC
>elephant ENSLAFT00000034253.1
ATGGAGACTGAGAGTGAGCAGAACTCCAGCTCCACCAATGGGAGTTCCAGCTCAGGGGGC
AGCTCAAGGCCCCAGATAGCTCAAATGTCACTGTACGAACGACAAGCAGTGCAGGCTCTG
CAGGCGCTGCAGCGGCAGCCCAACGCGGCTCAGTATTTTCACCAGTTCATGCTCCAGCAG
CAGCTCAGCAATGCCCAGCTGCATAGTCTGGCTGCCGTCCAGCAGGCTACCATTGCTGCT
AGTCGGCAGGCCAGCTCCCCAAACACCAGCACTACACAGCAGCAGTCTACCACCACCCAG
GCCTCAATTAATCTGGCCACCACGTCGGCCGCCCAGCTCATCAGCCGATCCCAGAGTGTG
AGCTCTCCTAGTGCCACCACCTTGACCCAATCTGTGCTTCTGGGGAACACCACCTCCCCA
CCCCTCAACCAGTCCCAGGCCCAGATGTATCTACGGCCACAGCTGGGAAACCTGTTGCAG
GTAAACCGGACCCTGGGCAGGAATGTGCCTCTAGCCTCCCAACTCATCCTAATGCCCAAC
GGGGCTGTGGCTGCAGTCCAGCAGGAGGTGCCATCTGCTCAGTCTCCTGGAGTTCATGCA
GATGCAGATCAGGTGCAGAACTTGGCAGTGAGGAACCAACAGGCCTCAGCCCAAGGACCC
CAAATGCAAGGCTCAACTCAGAAGGCCATTCCTCCTGGAGCCACCCCTACCTCTAGCCTT
TCTCAGGCCTCTAGCCAGGCCCTTGCTGTGGCTCAGGCTTCTTCTGGGGCTACAGGCCAG

TCCCTCAACCTTAGCCAAGCTGGTGGAGGCAGTGGGAATAGCATCCCAGGGTCCATGGGT
CCAGGTGGAGGTGGCCAGGCACCTGGGGGCTTGGGTCAGTTGCCTTCCTCAGGAATGGGT
GGTGGTGGGAGCTGTCCCAGGAAAGGCACAGGAGTGGTGCAGCCCTTGCCTGCAGCCCAA
GCAGTGACTGTGAGCCAGGGCAGCCAGACAGAGGCAGAAAGTGCAGCGGCCAAGAAAGCA
GAAGCCGAGGGGAGTGGTCAGCAGAATGTGGGCATGAACCTGACGCGGACAGCAACACCT
GCTCCCAGTCAGACCCTTATTAGCTCAGCCACCTATACACAGATTCAACCCCATTCACTG
ATTCAGCAGCAACAACAGATCCACCTACAGCAGAAACAGGTGGTGATCCAGCAGCAGATC
GCCATCCACCACCAGCAGCAGTTCCAGCACCGCCAATCTCAGCTCCTCCACACAGCCACC
CACCTCCAGTTGGCCCAGCAGCAACAGCAGCAGCAGCAGCAGCAGCAGCAGCAGCAACAG
CAACAGCAGCAGCAGCAGCAGCAGCAAACCACGACCCTCACTGCCCCTCAGCCACCACAG
GTCCCACCTACTCAGCAGGTCCCACCTTCCCAGTCCCAGCAGCAAGCCCAAACCCTGGTT
GTTCAACCCATGCTTCAGTCTTCTCCCCTGTCCCTCCCGCCTGACCCAACCCCCAAGCCA
CCCATCCCCATCCAATCCAAACCACCTGTAGCGCCTCTTAAGCCTCCTCAGTTAGGGGCT
GCTAAGATGTCAGCTACCCAGCAACCACCACCTCACATCCCTGTGCAAGTGGTAGGCACC
AGACAGCCAGGTACAGCCCAGGCACAGGCTCTGGGGTTGGCACAGCTGGCAGCTGCTGCA
CCTACTTCTCGGGGGTTGCCGGGTACAGCACAGCCAGCGGGTCAGGCCCATTTGGCCGCC
TCACCACCTTCATCCCAGGCTCCTGGTACACTTCAGGAGTGCTCTCCCACGTTGGCCCCT
GGGATGACCCTTGCCCCTGTGCAGGGGACAGCACATGTAGTAAAGGGTGGGGCAACCACC
TCCTCACCTGTTGTAGCTCAGGTCCCTGCTGCCTTCTACATGCAGTCTGTGCACCTGCCG
GGCAAACCCCAGACGTTGGCTGTCAAACGCAAGGCTGAGTCTGAAGAAGAGAGAGAAGAT
GTCTCTGCACTGGGTTCAATGCTTCCTGCCAAGGCATCTCCAGTAGCAGAGAGCCCAAAG
GCCATGGAGGAGAAGAGCAGTCTTGGAGAGAAAGCTGAACCAGTGGCCGCTGTGAATGCT
AATACCCCAAGCAGTGAACTAGTAACCTTGACCCCTGCCCCATCAGCACCACCTCCTACG
CTAGCCATGGTGTCCAGACAAATGAGTGACTCAAAACCCCCACAGGCCATCGTGAAGCCC
CAGATTCTCACTCACATCATTGAAGGCTTTGTTATCCAGGAAGGAGCAGAGCCTTTCCCG
GTGGGTTGTTCTCAGTTACTGAAAGAGTCTGAGAAGGCACTACAGACTGGTCTCCCGACA
GGGCTGAATGAGAATCAGCCAGGTGGCCCCTTGGGAGGGAACAGCCCATCTGCTGAACTA
GATAAGAAGGCAAATCTCCTGAAGTGTGAGTACTGTGGGAAGTACGCCCCTGCGGAGCAG
TTTCGTGGCTCCAAGAGGTTCTGTTCCATGACTTGCGCTAAGAGGTACAATGTGAGCTGT
AGCCACCAGTTCCGACTGAAGAGGAAAAAAATGAAAGAGTTTCAAGAAGCGAACTATGCC
CGCGTTCGCCGGCGTGGGCCCCGCCGCAGCTCCTCTGACATTGCCCGTGCCAAGATCCAG
GGCAAGCGCCACCGGGGTCAAGAGGACTCTAGTCGGGGTTCAGATAATTCCAGTTATGAT
GAAGCACTCTCACCAACATCTCCTGGGCCTTTATCAGTGAGAGCTGGGCATGGAGAACGT
GACCTGGGGAACCCCAACACAGCTCCTCCAACACCAGAATTACATGGCATCAACCCTGTG
TTCCTGTCCAGTAACCCTAGCCGTTGGAGTGTAGAGGAGGTGTATGAGTTTATTGCTTCT
CTACAAGGCTGCCAAGAGATTGCAGAGGAGTTTCGTTCCCAGGAGATTGATGGACAGGCC
CTTTTATTACTTAAAGAAGAACATCTTATGAGTGCCATGAACATAAAGCTGGGTCCTGCT
CTCAAGATCTGCGCCAAGATAAATGTCCTCAAGGAGACC

>ferret ENSMPUT00000017684.1
ATGGAGACTGAGAGTGAGCAGAACTCCAACTCCACCAATGGGAGCTCCAGCTCTGGGGGC
AGCTCTCGGCCCCAGATAGCTCAGATGTCACTGTATGAACGGCAGGCGGTGCAGGCTTTG
CAGGCACTGCAGCGTCAGCCCAATGCGGCTCAGTATTTCCACCAGTTCATGCTCCAGCAG
CAGCTCAGCAATGCCCAGCTGCATAGCCTGGCTGCGGTCCAGCAGGCCACGATTGCTGCC
AGTCGGCAGGCCAGCTCCCCAAACACCAGCACTGCCCAGCAGCAGACTACTACCACCCAG
GCCTCAATCAATCTGGCCACCACGTCAGGCGCCCAGCTCATCAGCCGATCGCAGAGTGTG
AGCTCTCCCAGTGCTACCACTTTGACCCAATCTGTGCTACTGGGGAACACCACCTCCCCA

CCTCTCAACCAGTCCCAGGCCCAGATGTATCTACGGCCACAGCTGGGAAACCTATTGCAG
GTAAACCGGACCCTGGGCCGGAATGTGCCTCTAGCCTCCCAACTCATCCTGATGCCTAAC
GGGGCGGTGGCTGCGGTCCAGCAGGAGGTGCCATCTGCTCCATCTCCGGGAGTTCATACA
GATGCAGATCAGGTGCAGAATTTGGCAGTGAGGAACCAACAGGCCTCAGCTCAAGGACCT
CAAATGCAAGGCTCTACTCAGAAGGCCATTCCTCCTGGAGCCTCCCCTGTTTCTAGCCTC
TCCCAGGCCTCTAGCCAGGCCCTTGCTGTGGCTCAGGCTTCCTCTGGGGCCTCAGGCCAG
TCCCTCAACCTCAGTCAAGCTGGCACAGGCAGTGGGAGTAGCATCCCAGGGTCCATGGGG
CCAGGTGGTGGTGGCCAGGCACCCGGGGGCTTGGGTCCATTGCCTTCCTCAGGAATGGGT
GGGGGTGGGAGCTGTCCCAGGAAGGGCACAGGAGTGGTGCAGCCCTTGCCTGCCGCCCAA
ACAGTGACTGTGAGTCAGGGAAGCCAGACAGAAGCAGAAAGTGCAGCAGCTAAGAAGGCA
GAAGCAGATGGGGCTGGTCAGCAGAATGTGGGCATGAACCTGACACGGACAGCTACACCT
GCTCCCAGCCAGACCCTTATTAGCTCAGCCACCTACACGCAGATCCAGCCCCACTCCCTG
ATTCAGCAACAGCAGCAGATCCACCTCCAGCAGAAACAGGTGGTGATCCAGCAGCAGATC
GCCATCCACCACCAGCAGCAGTTCCAGCACCGGCAGTCCCAGCTCCTCCACACAGCCACA
CACCTCCAGCTGGCCCAGCAGCAGCAGCAGCAGCAGCAGCAGCCGCCGCCGCCGCCCCCG
CCGCCAGCTGCCACACTCACTGCCCCTCAGCCACCGCAGGTCCCACCTACTCAGCAGGTC
CCTCCTTCCCAGTCCCAGCAGCAAGCCCAGACCCTGGTGGTCCAACCCATGCTTCAGTCT
TCACCCTTGTCCCTCCCGCCTGACCCAACCCCCAAGCCACCCATCCCCATCCAGTCCAAA
CCACCTGTAGCACCTATCAAGCCTCCTCAGTTAGGCGCTGCTAAGATGTCAGCTACCCAG
CAACCACCACCCCATATCCCTGTGCAAGTGGTGGGCACCCGGCAGCCTGGGACGGCCCAG
GCACAGGCTCTGGGGTTGGCACAGCTGGCCGCTGCTGTCCCTACTTCCCGGGGCATGCCC
AATACAGTGCAGCCCAGTCAGGCCCATTTGGCCTCCTCGCCACCTTCATCCCAGGCTGCC
GGGGCCCTGCAAGAGTGCCCTCCCACGCTGGCGTCTGGGATGAGCCTCGCTCCTGTGCAG
GGGACAGCGCATGTGGTAAAGGGTGGGGCTACGACCTCCTCACCTGTTGTGGCCCAGGTA
CCTGCTGCCTTCTACATGCAGTCTGTGCACCTGCCGAGCAAACCCCAGACATTGGCTGTC
AAACGCAAGGCAGAGTCTGAGGAGGAGAGAGATGATATCTCCACGTTGGGTTCAATGCTT
CCTGCCAAGGCATCTCCAGTAGCTGAAAGTCCAAAGGCCATGGAGGAGAAGAGCAGTCTT
GGAGAGAAAGCTGAACCAGTGACTGGTGTGAATGCTAGTACTCCAAGCAGTGAACTAGTA
GCCTTGGCTCCTGCCCCAGCTGCACCGCCCCCTACACTAGCCATGGTGTCCAGACAGATG
GGTGACTCAAAACCCCCACAGGCCATCGTGAAGCCCCAGATTCTCACCCACATCATTGAA
GGCTTCGTTATCCAGGAAGGAGCAGAGCCTTTCCCGGTGGGTTGTTCTCAGTTACTGAAA
GAGTCTGAGAAGCCACTACAGACTGGCCTGCCAGGGCTGAATGAGAATCAGTCAGGGGGC
CCCTTGGGAGGGGATAGCCCATCTGCTGAGCTAGATAAGAAGGCGAACCTCCTGAAGTGT
GAGTACTGCGGGAAGTATGCTCCTGCTGAGCAGTTTCGCGGCTCCAAGAGGTTCTGCTCA
ATGACCTGCGCTAAGAGGTATAATGTGAGCTGTAGTCACCAGTTCCGGCTGAAGAGGAAA
AAAATGAAAGAGTTCCAAGAAGCCAACTATGCTCGCGTTCGTCGGCGGGGACCCCGCCGC
AGCTCCTCAGACATCGCTCGTGCCAAGATCCAGGGCAAGCGCCACCGGGGTCAAGAGGAC
TCTAGCCGGGGTTCAGATAATTCCAGTTATGATGAAGCACTCTCTCCAACATCTCCTGGG
CCTTTATCTGTGCGAACTGGGCATGGAGAACGTGACCTGGGGAACCCCAATACGGCACCA
CCAACGCCAGAATTACATGGCATCAACCCTGTGTTCCTGTCCAGCAATCCCAGCCGTTGG
AGTGTAGAAGAGGTGTATGAGTTTATCGCTTCTCTACAAGGCTGCCAAGAGATTGCAGAG
GAGTTTCGTTCCCAGGAGATTGATGGACAGGCCCTTTTATTACTTAAAGAGGAGCATCTT
ATGAGTGCCATGAACATCAAGTTGGGCCCTGCCCTCAAGATCTGCGCCAAGATAAACGTC
CTCAAGGAGACC
>myotis lucifugis ENSMLUT00000011139.2
ATGGAGACTGAGAGTGAGCAG
AACTCCAACTCCACCAATGGGAGTTCCAGCTCTGGGGGCAGCTCTCGGCCCCAAATAGCT

CAAATGTCACTGTATGAACGGCAAGCAGTGCAGGCTCTGCAGGCACTGCAGCGTCAGCCC
AATGCGGCTCAGTATTTCCACCAGTTCATGCTCCAGCAGCAGCTCAGCAATGCCCAGCTG
CATAGCCTGGCTGCCGTCCAGCAGGCCACCATTGCTGCCAGTCGGCAAGCCAGCTCCCCA
AACACCAGCACCGCGCAGCAGCAGACTGCCACCACCCAGGCCTCAATCAATCTGGCCACC
ACGTCGGCCGCCCAGCTCATCAGCCGGTCCCAGAGTGTGAGCTCTCCCAGTGCTACCACT
TTGACCCAGTCTGTGCTACTGGGGAACACCACCTCCCCACCCCTCAACCAGTCCCAGGCT
CAGATGTATCTACGGCCACAGCTGGGAAACCTATTGCAGGTAAACCGGACCCTGGGCCGG
AATGTGCCTCTAGCCTCCCAGCTCATCCTGATGCCTAACGGGGCAGTGGCTGCAGTCCAG
CAGGAGGTGCCATCTGCTCAGTCTCCGGGAGTTCATGCAGATGCAGATCAGGTGCAGAAC
TTGGCAGTGAGGAACCAACAAGCCTCAGCCCAAGGCCCTCAAATGCAAGGCTCTACTCAG
AAGGCCATTCCTCCTGGAGCATCCCCTGTCTCTAACCTCTCCCAGGGTTCCAGCCAGGCC
CTAGCTGTGGCTCAGGCTTCCCCTGGGGCCTCAGGCCAGTCCCTCAACCTTAGTCAAGCT
GGTGGAGGCAGTGGAAATAGCATCCCAGGGTCCATGGGTCCAGGTGGAAGTGGCCAGGCA
CCTGGAGGCTTGGGTCAGTTGCCTACCTCAGGAATGGGGGGTGGTGGGAGCTGTCCCAGG
AAGGGCACAGGAGTGGTGCAGCCCTTGCCTGCAGCCCAAACAGTGACTGTGAGTCAGGGC
AGCCAGACAGAGGCAGAAAGTGCGGCAGCTAAGAAGGCAGAAGCGGATGGGACTGGTCCG
CAGAACGTGGGCATGAACCTGACACGGACAGCTACACCTGCTCCCAGCCAGACCCTTATT
AGCTCAGCCACCTACACACAGATCCAGCCCCATTCCCTGATTCAGCAACAGCAACAGATC
CACCTCCAGCAGAAACAAGTGGTGATCCAACAGCAGATTGCCATCCACCACCAGCAACAG
TTCCAGCACCGTCAATCGCAGCTACTCCACACAGCCACACACCTCCAGTTGGCCCAGCAG
CAGCAGCAGCAGCAGCAACAGCAGCAGCAGCAGCAGCAGCAGCAGCAAGCCACAACTCTC
TCTGCCCCTCAGCCACCACAGGTCCCACCTACTCAGCAGGTCCCACCTTCCCAGTCCCAG
CAGCAAGCCCAAACCCTGGTTGTTCAACCCATGCTTCAATCTTCACCCTTGTCCCTCCCA
CCTGACCCAACTCCCAAGCCACCCATCCCTCTCCAATCCAAACCACCTATAGCACCGATC
AAGCCTCCTCAGTTAGGACCTGCTAAGATGTCAGCTTCCCAGCAACCACCACCCCATATC
CCTGTGCAAGTTGTGGGCACCCGACAGCCAGGTACAGCCCAGGCACAGGCTTTGGGGTTG
GCACAGCTGGCAGCTGCTGTGCCGGCTTCCCGGGGGATGCCAGGTACAATGCAGCCTGGT
CAGGCCCATTTGGCCTCTTCGCCACCATCATCCCAGGCTCCTGGTGCACTGCAGGAGTGC
CCTTCCACACTGACCTCTGGGATGACCCTTGCTCCTGTGCAGGGGACAGCACATGTAGTA
AAGGGTGGGGCTACCGCCTCCTCACCTGTTGTAGCCCAGGTCCCTGCTGCCTTCTACATG
CAGTCTGTGCACCTGCCGGGCAAGCCCCAGACATTGGGTGTGAAACGCAAGGCTGAGTCT
GAGGAGGAAAGAGATGATGTCTCCACATTGGGTTCAATGCTTCCTGCCAAGGCATCTCCG
GTAGCAGAGAGCCCAAAGGCCATGGAGGAGAAGAGCAGTTTTGGAGAGAAAGTTGAATCA
GTGACCAATGTGAATGCTAATACCCCAAACAGTGAACTAATATCCTTGGCTCCTGCCCCA
TCAGCACCACCTCCTACACTAGCCATGGTGTCCAGACAGATGGGCGACTCAAAACCCCCA
CAGGCCATTGTGAAGCCCCAGATTCTCACCCACATCATTGAAGGCTTCGTTATCCAGGAA
GGAGCAGAACCTTTCCCGGTGGGTTGTTCTCAGTTACTGAAAGAGTCTGAGAAGCCTCTA
CAGACTGGCCTCCTGACAGGGCTGAATGAGAACCAGTCATGTGGCCCCTTGGGAGGGGAC
AGCCCATCTGCTGAGCTAGATAAGAAGGCGAATCTCCTGAAGTGCGAATACTGTGGGAAG
TACGCCCCTGCAGACCAGTTTCGAGGCTCCAAGAGATTCTGCTCCATGACTTGTGCCAAG
AGGTATAATGTGAGCTGTAGCCACCAGTTTCGGCTGAAGAGGAAAAAAATGAAAGAGTTT
CAAGAAGCCAATTATGCTCGTGTTCGTCGGCGTGGACCCCGCCGCAGCTCCTCTGACATC
GCCCGTGCCAAGATCCAGGGAAAGCGACACCGGGGTCAAGAGGACTCTAGCCGGGGTTCA
GATAATTCCAGTTATGATGAAGCACTCTCTCCAACATCTCCTGGGCCTTTATCAGTGAGA
GTGGGGCATGGAGATCGAGACCTGGGGAACCCCAATACAGCTCCACCTACCCCAGAATTA
CACGGCATCAACCCTGTGTTCCTGTCCAGCAATCCCAGCCGTTGGAGTGTAGAGGAGGTG
TATGAGTTTATCGCTTCTCTACAAGGCTGTCAAGAGATTGCAGAGGAGTTTCGTTCCCAG

GAGATTGATGGACAGGCCCTTTTATTACTTAAAGAGGAACATCTTATGAGTGCCATGAAC
ATCAAGCTGGGCCCAGCCCTTAAGATCTGCGCCAAGATCAACGTCCTCAAGGAGACC
>Pteropus vampyrus XM_011365765.1
ATGGAGACTGAGAGCGAGCAGAACTCTAACTCCACCAATGGGAGTTCCAGCTCTGGGGGCAGCTCTCGGCCC
CAG
ATAGCTCAGATGTCACTGTATGAACGGCAAGCAGTGCAGGCTCTGCAGGCACTGCAGCGTCAGCCCAACG
CGGCTCAGTATTTCCACCAGTTCATGCTCCAGCAGCAGCTCAGCAATGCCCAGCTGCATAGCCTGGCTGC
CGTCCAGCAGGCCACAATTGCTGCCAGTCGGCAGGCCAGCTCCCCAAACACCAGCACTGCACAGCAGCAG
ACCGCCACCACCCAGGCCTCAATCAATCTGGCCACCACGTCGGCCGCCCAGCTCATCAGCCGATCCCAGA
GTGTGAGCTCTCCCAGTGCTACCACTTTGACCCAATCTGTGCTACTGGGGAACACCACCTCCCCACCCCT
CAACCAGTCCCAGGCCCAGATGTATCTACGGCCACAGCTGGGAAACCTATTGCAGGTAAACCGGACCCTG
GGCCGGAATGTGCCTCTAGCCTCCCAACTCATCCTGATGCCTAACGGGGCAGTGGCTGCAGTCCAGCAGG
AGGTGCCATCTGCTCAGTCTCCAGGAGTTCATACAGATGCAGATCAGGTGCAGAACTTGGCTGTGAGAAA
CCAACAGGCTTCTGCCCAAGGACCTCAAATGCAAGGCTCTACTCAGAAGGCCATTCCTCCCGGAGCCTCC
CCTGTCTCTGGCCTCTCCCAAGCCTCTAGCCAGGCCCTAGCTGTGGCTCAGGCTTCCTCTGCGGCCTCAG
GCCAGTCCCTCAACCTTAGTCAAGCTGGTGGAGGCAGTGGGAATAGCATCCCAGGGTCTATGGGTCCAGG
TGGAAGTGGCCAGGCACCTGGGGGCATGGGTCAGTTGCCTTCCTCAGGAATGGGTGGTGGTGGGAGCTGT
CCCAGGAAGGGCACAGGAGTGGTGCAGCCCTTGCCTGCAGCCCAAACAGTGACTGTGAGTCAGGGAAGCC
AGACTGAAGCAGAAAGTGCGGCAGCCAAGAAGGCAGAAGCAGATGGGACTGGTCAGCAGAACGTGGGCAT
GAACCTGACACGGACAGCTACACCTGCTCCTAGCCAGACCCTTATTAGCTCAGCCACCTACACACAGATC
CAGCCCCATTCCCTGATTCAGCAACAGCAACAGATCCACCTGCAGCAGAAACAGGTGGTGATCCAGCAGC
AGATTGCCATCCACCATCAGCAACAGTTCCAGCACCGTCAATCTCAGCTACTCCACACAGCCACACACCT
CCAGTTGGCCCAGCAGCAGCAACAGCAGCAGCAGCAGCAGCAGCAGCAGCAACAGCAACAGCAGCAGCAG
CAGCAGCAGCAGCAACAGCAGCAGCAGCAGCAGCAAGCCACAGCTCTCTCTGCCCCTCAGCCACCACAGG
TCCCACCGACTCAGCAGGTCCCACCTTCCCAGTCCCAGCAGCAAGCCCAAACCCTGGTTGTTCAACCCAT
GCTTCAGTCTTCACCTTTGTCCCTCCCACCCGACCCAACTCCCAAGCCACCCATTCCCATCCAATCCAAA
CCACCTGGAGCACCGATCAAACCTCCTCAGTTGGGGGCTGCTAAGATGTCAGCTACCCAGCAACCACCAC
CCCATATCCCTGTGCAAGTTGTGGGCACCCGACAGCCAGGTACAGCCCAGGCACAGGCTTTGGGGTTGGC
ACAGCTGGCAGCTGCTGTACCTACTCCCCGGGGGATGCCAGGTACAATGCAGCCTGGCCAGTCCCATTTG
GCCTCCTCCTCGCCACCTTCATCCCAGGCTCCTGGTACACTGCAGGAGTGCCCTCCCACATTGGCCTCTG
GGATGACCCTTGCTCCTGTGCAGGGGACAGCACATGTGGTAAAGGGTGGGGCTACCACCTCCTCACCTGT
TGTAGCCCAGGTCCCTGCTGCCTTCTACATGCAGTCTGTGCACCTGCCGGGCAAACCCCAGACATTGGGT
GTCAAACGCAAGGCAGAGTCTGAGGAGGAGAGAGACGATGTCTCTACAATGGGTTCAATGCTTCCTGCCA
AGGCATCTCCAGTAGCAGAGAGCCCAAAGGCCATGGAGGAGAAGAGCAGTCTTGGAGAGAAAGGTGAACC
AATGACCAATGTGAATGCTAATACCCCAAGCGGTGAACTCGTATCCTTGGCTCCTGCCCCATCGGCACCA
CCTCCTACTCTAGCAATGGTGTCCAGACAGATGGGTGACTCAAAACCCCCACAGGCCATTGTGAAGCCCC
AGATTCTCACCCACATCATTGAAGGCTTTGTTATCCAGGAAGGAGCAGAACCTTTCCCGGTGGGTTGTTC
TCAGTTACTGAAAGAGTCTGAGAAGCCACTGCAGACTGGCCTCCCGACAGGTCTGAATGAGAATCAGTCA
GGTGGCCCCTTGGGAGGGGACAGCCCATCTGTTGAGCTAGATAAGAAGGCGAATCTCCTGAAGTGCGAGT
ACTGTGGGAAGTACGCCCCTGCAGAGCAGTTTCGCGGCTCCAAGAGATTCTGCTCCATGACTTGTGCTAA
GAGGTATAATGTGAGCTGTAGCCACCAGTTCCGGCTGAAGAGGAAAAAAATGAAAGAGTTTCAAGAAGTC
AACTATGCTCGTGCCCGCCGGCGCGGACCCCGGCGCAGCTCCTCTGACATCGCCCGAGCCAAGATTCAGG
GCAAGCGCCACCGGGGTCAAGAGGACTCTAGCCGGGGTTCAGATAATTCCAGTTATGATGAAGCACTCTC
TCCAACATCTCCTGGGCCTTTGTCAGTGAGAGCTGGCCATGGAGAACGAGACCTGGGGAACCCCAATACA
GCCCCGCCCACCCCAGAGTTACATGGCATCAACCCTGTGTTCCTGTCCAGTAATCCCAGCCGTTGGAGTG
TAGAGGAGGTGTATGAGTTTATTGCTTCTCTACAAGGCTGCCAAGAGATCGCAGAGGAGTTTCGTTCCCA
GGAGATTGATGGACAGGCCCTTTTATTACTTAAAGAGGAACATCTTATGAGTGCCATGAACATCAAGCTG

GGCCCTGCCCTCAAGATCTGCGCCAAGATAAATGTCCTCAAGGAGACC
>Pteropus alecto XM_015591491.1
ATGGAGACTGAGAGCGAGCAGAACTCTAACTCCACCAATGGGAGTTCCAGCTCTGGGGGCAGCT
CTCGGCCCCAGATAGCTCAGATGTCACTATATGAACGGCAAGCAGTGCAGGCTCTGCAGGCACTGCAGCG
TCAGCCCAACGCGGCTCAGTATTTCCACCAGTTCATGCTCCAGCAGCAGCTCAGCAATGCCCAGCTGCAT
AGCCTGGCTGCCGTCCAGCAGGCCACAATTGCTGCCAGTCGGCAGGCCAGCTCCCCAAACACCAGCACTG
CACAGCAGCAGACCGCCACCACCCAGGCCTCAATCAATCTGGCCACCACGTCGGCCGCCCAGCTCATCAG
CCGATCCCAGAGTGTGAGCTCTCCCAGTGCTACCACTTTGACCCAATCTGTGCTACTGGGGAACACCACC
TCCCCACCCCTCAACCAGTCCCAGGCCCAGATGTATCTACGGCCACAGCTGGGAAACCTATTGCAGGTAA
ACCGGACCCTGGGCCGGAATGTGCCTCTAGCCTCCCAACTCATCCTGATGCCTAACGGGGCAGTGGCTGC
AGTCCAGCAGGAGGTGCCATCTGCTCAGTCTCCAGGAGTTCATACAGATGCAGATCAGGTGCAGAACTTG
GCTGTGAGAAACCAACAGGCTTCTGCCCAAGGACCTCAAATGCAAGGCTCTACTCAGAAGGCCATTCCTC
CCGGAGCCTCCCCTGTCTCTGGCCTCTCCCAAGCCTCTGGCCAGGCCCTAGCTGTGGCTCAGGCTTCCTC
TGCGGCCTCAGGCCAGTCCCTCAACCTTAGTCAAGCTGGTGGAGGCAGTGGGAATAGCATCCCAGGGTCT
ATGGGTCCAGGTGGAAGTGGCCAGGCACCTGGGGGCATGGGTCAGTTGCCTTCCTCAGGAATGGGTGGTG
GTGGGAGCTGTCCCAGGAAGGGCACAGGAGTGGTGCAGCCCTTGCCTGCAGCCCAAACAGTGACTGTGAG
TCAGGGAAGCCAGACTGAAGCAGAAAGTGCGGCAGCCAAGAAGGCAGAAGCAGATGGGACTGGTCAGCAG
AACGTGGGCATGAACCTGACACGGACAGCTACACCTGCTCCTAGCCAGACCCTTATTAGCTCAGCCACCT
ACACACAGATCCAGCCCCATTCCCTGATTCAGCAACAGCAACAGATCCACCTGCAGCAGAAACAGGTGGT
GATCCAGCAGCAGATTGCCATCCACCATCAGCAACAGTTCCAGCACCGTCAGTCTCAGCTACTCCACACA
GCCACACACCTCCAGTTGGCCCAGCAGCAGCAACAGCAGCAGCAGCAGCAGCAACAGCAGCAGCAGC
AGCAGCAGCAGCAGCAGCAGCAGCAGCAGCAGCAACAGCAGCAGCAGCAGCAGCAGCAGCAGCAGCAGCA
AGCCACAGCTCTCTCTGCCCCTCAGCCACCACAGGTCCCACCTACTCAGCAGGTCCCACCTTCCCAGTCC
CAGCAGCAAGCCCAAACCCTGGTTGTTCAACCCATGCTTCAGTCTTCACCTCTGTCTCTCCCACCCGACC
CAACTCCCAAGCCACCCATTCCCATCCAATCCAAACCACCTGGAGCACCGATCAAACCTCCTCAGTTGGG
GGCTGCTAAGATGTCAGCTACCCAGCAACCACCACCCCATATCCCTGTGCAAGTTGTGGGCACCCGACAG
CCAGGTACAGCCCAGGCACAGGCTTTGGGGTTGGCACAGCTGGCAGCTGCTGTACCTACTCCCCGGGGGA
TGCCAGGTACAATGCAGCCTGGCCAGTCCCATTTGGCCTCCTCCTCGCCACCTTCATCCCAGGCTCCTGG
TACACTGCAGGAGTGCCCTCCCACATTGGCCTCTGGGATGACCCTTGCTCCTGTGCAGGGGACAGCACAT
GTGGTAAAGGGTGGGGCTACCACCTCCTCACCTGTTGTAGCCCAGGTCCCTGCTGCCTTCTACATGCAGT
CTGTGCACCTGCCGGGCAAACCCCAGACATTGGGTGTCAAACGCAAGGCAGAGTCTGAGGAGGAGAGAGA
CGATGTCTCTACAATGGGTTCAATGCTTCCTGCCAAGGCATCTCCAGTAGCAGAGAGCCCAAAGGCCATG
GAGGAGAAGAGCAGTCTTGGAGAGAAAGGTGAACCAATGACCAATGTGAATGCTAATACCCCAAGCGGTG
AACTCGTATCCTTGGCTCCTGCCCCATCGGCACCACCTCCTACTCTAGCAATGGTGTCCAGACAGATGGG
TGACTCAAAACCCCCACAGGCCATTGTGAAGCCCCAGATTCTCACCCACATCATTGAAGGCTTTGTTATC
CAGGAAGGAGCAGAACCTTTCCCGGTGGGTTGTTCTCAGTTACTGAAAGAATCTGAGAAGCCACTGCAGA
CTGGCCTCCCGACAGGTCTGAATGAGAATCAGTCAGGTGGCCCCTTGGGAGGGGACAGCCCATCTGTTGA
GCTAGATAAGAAGGCGAATCTCCTGAAGTGCGAGTACTGTGGGAAGTACGCCCCTGCAGAGCAGTTTCGC
GGCTCCAAGAGATTCTGCTCCATGACTTGTGCTAAGAGGTATAATGTGAGCTGTAGCCACCAGTTCCGGC
TGAAGAGGAAAAAAATGAAAGAGTTTCAAGAAGTCAACTATGCTCGTGCCCGCCGGCGCGGACCCCGGCG
CAGCTCCTCTGACATCGCCCGAGCCAAGATTCAGGGCAAGCGCCACCGGGGTCAAGAGGACTCTAGCCGG
GGTTCAGATAATTCCAGTTATGATGAAGCACTCTCTCCAACATCTCCTGGGCCTTTGTCAGTGAGAGCTG
GCCATGGAGAACGAGACCTGGGGAACCCCAATACAGCCCCGCCTACCCCAGAGTTACATGGCATCAACCC
TGTGTTCCTGTCCAGTAATCCTAGCCGTTGGAGTGTAGAGGAGGTGTATGAGTTTATTGCTTCTCTACAA
GGCTGCCAAGAGATCGCAGAGGAGTTTCGTTCCCAGGAGATTGATGGACAGGCCCTTTTATTACTTAAAG
AGGAACATCTTATGAGTGCCATGAACATCAAGCTGGGCCCTGCCCTCAAGATCTGCGCCAAGATAAATGT
CCTCAAGGAGACC

>sheep ENSOART00000001639.1
ATGGAGACTGAGAGTGAACAGAACTCCAAC
TCCACTAATGGGAGTTCTGGCTCTGGGGGCAGCTCTCGGCCCCAGATCGCTCAAATGTCA
CTGTATGAACGGCAGGCCGTGCAGGCTCTGCAGGCACTGCAGCGTCAGCCCAACGCGGCT
CAGTATTTCCACCAGTTCATGCTCCAGCAGCAGCTCAGCAATGCCCAGCTGCATAGCCTG
GCTGCCGTCCAGCAGGCCACAATTGCTGCCAGTCGGCAGGCCAGCTCCCCAAACACCAGC
ACTGCACAGCAGCAGACCACCACCACCCAGGCCTCTATCAATCTGGCCACCACATCGGCC
GCCCAGCTCATCAGCCGGTCCCAGAGTGTGAGCTCTCCCAGTGCTACCACTTTGACCCAG
TCTGTGCTACTGGGGAACACCACCTCCCCACCCCTCAACCAGTCCCAGGCCCAGATGTAT
CTACGGCCACAGCTGGGAAACCTATTGCAGGTAAACCGGACCCTGGGCCGGAATGTGCCT
CTAGCCTCCCAACTCATCCTGATGCCTAACGGGGCAGTGGCTGCAGTCCAGCAGGAGGCG
CCATCTGCTCAGTCTCCTGGAGTCCACACAGATGCTGATCAGGTGCAGAACTTGGCCGTC
AGGAACCAGCAGGCCTCCGCCCAAGGACCCCAAATGCAAGGCTCTGCTCAGAAGGCCATC
CCTCCTGGGGCCTCCTCTGTCTCCAGCCTCTCCCAGGCCTCTAGCCAGGCCCTTGCGGTG
GCTCAGGCTTCCTCTGGGGCCTCAGGCCAGTCCCTCAACCTTAGTCAAGCTGGTGGAGGC
AGTGGGACTAACATCCCAGGGTCCATGGGTCCTGGAGGAGGTGGCCAGGCTCCTGGGGGA
TTGGGGCAGTTGCCTTCCTCAGGAATGGGTGGTGGTGGGAGCTGCCCCAGGAAGGGCACC
GGAGTGGTGCAGCCCTTGCCTGCAGCCCAGGCCGTGACTGTGAGTCAGGGCAGCCAGACA
GAAGCCGAAAGCGCAGCAGCCAAGAAAGCAGAAGCAGACGGGACCGGTCAGCAGAGCGTG
GGCATGAACCTCACCCGGACCGCCACTCCTGCTCCCAGCCAGACCCTCATTAGCTCAGCT
ACCTACACGCAGATCCAGCCCCACTCCCTGATTCAGCAGCAGCAGCAGATCCACCTCCAG
CAGAAGCAGGTGGTGATCCAGCAGCAGATCGCTATCCACCACCAGCAGCAGTTCCCGCAC
CGCCAGTCCCAGCTTCTACACACGGCCACGCACCTCCAGCTGGCCCAGCAGCAGCAGCAG
CAGCAGCAGCAACAGCAGGCCACCACCCTCACTGCCCCGCAGCCTCCGCCGGGTCCCCCG
ACCCAGCCGGTTCCGCCGTCCCCGCCCCAGCAGCCAGCCCCCCTGGTTGTCCAGCCCATG
CTGCCGTCTTCCCCCCTGTCCCTGCCGCCAGACCCCGCCCCCAAGCCCCCCATCCCCATC
CAGTCCAAACCGCCGGCAGCCCCTCTCAAGCCCCCTCAGCTAGGGGCTGCCAAGATGTCT
GCCGCCCAGCAGCCCCCACCCCACATCCCTGTGCAGGTGGTGGGCACCCGGCAGCCGGGT
ACAGCCCAGGCCCAGGCTTTGGGGCTGGCCCAGCTGGCAGCTGCCGTCCCCACTTCCCGG
GGGATGCCAGGCACAGTGCCGCCAGGCCAGGCCCACTTGGCCTCCTCGCCGCCTTCATCC
CAGGCCCCGGGTGCGCTGCAGGAGTGCCCGCCCACGTTGGCCTCAGGGATGACCCTGGCC
CCCGTGCAGGGGACGGCGCATGTGGTGAAGGGAGGGGCAACCACCTCCTCACCGGTGGTC
GCGCAGGTCCCTGCCGCCTTCTACATGCAGTCTGTGCACCTGCCGGGCAAACCCCAGACA
TTGGCTGTAAAGCGCAAGGCTGAGTCTGAGGAGGAGCGAGATGACGTCTCCACACTGAGT
TCAATGATTCCTGCGAAGACATCTCCAGTGGTAGAGAGCCCGAAGGCCATGGAGGAGAAG
GGCGGTCTTGGAGAGAAAGCTGAGCCAGTGACCAGCACGAATGCTAACACCCTGAGCAGT
GACATTGTAGCTTTGGCTCCCGCCCCATCAGCGCCCCCTCCTTCGCTGGCCATGGTGTCC
AGACAGATGGGTGACTCCAAGCCCCCCCAGGCCATTGTGAAGCCCCAGATCCTCACCCAC
ATCATCGAAGGCTTCGTTATCCAGGAAGGAGCAGAGCCTTTCCCGGTGGGTTGTTCTCAG
TTATTGAAAGAGTCTGAGAAGCCCCTGCAGACTGGCCTCACCACAGGACTGAATGAGAAT
CAGTCAGGTGGTCCCTTGGGTGGGGACAGCCCATCTGCTGAGCTAGATAAGAAGGCAAAC
CTCCTGAAGTGCGAGTACTGTGGGAAGTACGCCCCCGCCGAGCAGTTCCGCGGCTCCAAG
AGATTCTGCTCCATGACTTGCGCTAAGAGGTATAATGTGAGCTGTAGCCACCAATTCCGG
CTGAAGAGGAAAAAAATGAAAGAATTTCAGGAAGCCAACTACGCCCGTGTTCGCCGGCGC
GGACCCCGCCGTGGCTCCTCGGACATCGCCCGTGCCAAGATCCAGGGCAAGCGCCACCGA
GGTCAAGAGGACTCTAGCCGGGGTTCAGATAATTCCAGTTACGATGAAGCACTCTCTCCA
ACATCTCCCGGGCCTTTATCCGTGAGAGCAGGGCATGGAGAACGTGACCTGGGGAATCCC

AACACAGCTCCACCCACCCCAGAATTACATGGCATCAACCCTGTGTTCCTATCCAGTAAT
CCTAGCCGCTGGAGCGTAGAGGAGGTCTATGAGTTCATTGCTTCTCTACAAGGTTGCCAA
GAGATTGCAGAGGAGTTTCGTTCCCAGGAGATTGATGGACAGGCCCTCTTATTACTTAAA
GAGGAACATCTCATGAGTGCCATGAACATCAAGCTGGGTCCTGCCCTCAAGATCTGTGCT
AAGATAAATGTCCTCAAGGAGACC
>pig ENSSSCT00000000717.2
ATGGAGACTGAGAGCGAGCAGAACTCCAGCTCCACCAATGGGAGTTCCAGCTCTGGGGGC
AGCTCTCGGCCCCAGATAGCTCAGATGTCACTGTATGAACGGCAGGCAGTGCAGGCTCTG
CAAGCACTGCAGCGTCAGCCCAATGCGGCTCAGTATTTCCACCAGTTTATGCTCCAGCAG
CAGCTCAGCAATGCCCAGCTGCATAGCCTGGCTGCCGTCCAGCAGGCTACAATTGCTGCC
AGCCGACAGGCCAGCTCCCCAAACACCAGCACTGCACAGCAGCAGAGCACCACCACCCAG
GCCTCCATCAATCTGGCCACGACGTCCGCTGCCCAGCTCATCAGCCGATCGCAGAGCGTG
AGCTCTCCCAGTGCCACCACTTTGACCCAGTCTGTGCTCCTGGGGAACACCACCTCCCCA
CCCCTCAACCAGTCTCAGGCCCAGATGTATCTACGGCCACAGCTGGGAAACCTGTTGCAG
GTAAACCGGACCCTGGGCCGGAATGTGCCTCTAGCCTCCCAACTCATCCTGATGCCTAAC
GGGGCGGTGGCCGCAGTCCAGCAGGAGGCGCCATCTGCTCAGTCTCCTGGAGTTCACACA
GATGCCGATCAGGTGCAGAACTTGGCAGTCAGGAACCAACAGGCCTCAGCCCAAGGACCC
CAAATGCAAGGCTCTGCTCAGAAGGCTGTCCCTCCTGGAGCCTCTCCTGTCTCTAGCCTC
TCCCAGGCCTCCAGTCAGGCCCTCGCTGTGGCTCAGGCTTCCTCTGGGGCCTCAGGCCAG
TCCCTCAACCTTAGTCAAGCTGGTGGAGGCAGTGGGAATAGCCTCCCAGGGTCCATGGGT
CCAGGTGGAGGTGGCCAGGCCGCTGGGGGCTTGGGTCAGCTGCCTTCCTCAGGAATGGGT
GGTGGCGGGAGCTGTTCCAGGAAGGGCACAGGAGTGGTGCAGCCCTTGCCTGCAGCCCAG
ACAGTGACTGTGAGTCAGGGCAGCCAGACAGAAGCAGAAAGTGCAGCGGCCAAGAAGGCA
GAAGCAGATGGGACTGGTCAGCAGAATGTGGGCATGAACCTGACCCGGACAGCCACGCCT
GCTCCCAGCCAGACCCTGATTAGCTCAGCCACCTACACCCAGATCCAGCCCCATTCCCTG
ATCCAGCAGCAGCAGCAGATCCACCTGCAGCAGAAGCAGGTGGTGATCCAGCAGCAGATA
GCCATCCACCACCAGCAGCAGTTCCAGCACCGCCAGTCGCAGCTGCTGCACACCGCCACG
CACCTCCAGCTGGCCCAGCAGCAGCAGCAGCAGCAGCAGCAGCAGCAGCAACAGCAGGCC
ACAACGCTCACTGCCCCCCCACCTCCGCAGGCCCCCCCTACGCAGCAGGTCCCACCTTCC
CAGTCCCAGCAGCAAGCCCAGACTCTGGTTGTCCAACCCATGCTTCAGTCCTCACCCTTG
TCCCTCCCGCCCGACCCAACCCCCAAGCCACCCATCCCCATCCAGTCCAAACCGCCCATG
GCCCCCATCAAGCCTCCTCAGCTAGGGGCTGCCAAGATGTCAGCCCCCCAGCCCCCCCCA
CCCCACATCCCCGTGCAAGTCGTGGGCACCCGACAGCCGGGGGCCACCCAGGCCCAGGCT
CTGGGGTTGGCACAGCTGGCAGCTGCCGTCCCTACCTCCCGGGGGGTGCCAGGCACAGTG
CAGCCTGGCCAGGCCCACCTGGCCTCCTCGCCACCTTCATCCCAGGCGCCCGGGGCGCTT
CAGGAGTGCCCTCCCACACTGGCCTCTGGGATGACCCTTGCTCCTGTCCAGGGGACAGCA
CATGTGGTGAAGGGTGGGGCTACCACCTCCTCACCTGTTGTAGCCCAGGTCCCTGCCGCC
TTCTACATGCAGTCTGTGCACCTGCCGGGCAAACCGCAGACATTGGCTCTGAAACGCAAG
GCCGAGTCTGAGGAGGAGAGAGACGACGTCGCCACGTTGAGCTCAGTGCTTCCCGCGAAG
ACATCCCCCGCCGTCGAGAGCCCGGAGGCCTTGGAGGAGAAGGGCGGCCTCGGGGAGAAA
GCTGAACCAGTGCCCAGCGTGAATGCGAATGCCCCGAGCAGTGATGTGGTAGCCTTGGCC
CCGGCCCCGGCAGCACCACCCCCTACACTGGCCATGGTGTCCAGACAGATGGGGGACTCA
AAACCCCCGCAGGCCATCGTGAAGCCCCAGATTCTCACCCACATCATTGAAGGCTTTGTT
ATCCAGGAAGGAGCAGAACCTTTCCCGGTGGGTTGTTCTCAGTTTCTGAAAGAGTCTGAG
AAGCCACTGCCGACTGGCCTTGCGACAGGGCTGAATGAGAATCAGTCAGGTGGCCCCTTG
GGTGGGGACAGCCCATCTGCTGAGCTGGATAAGAAGGCCAACCTCCTGAGGTGCGAGTAC
TGTGGCAAGTATGCCCCCGAGCAGTTTCGTGGCTCCAAGAGATTCTGCTCTATGACCTGC

GCGAAGAGGTATAATGTGAGCTGCAGCCACCAGTTCCGGCTGAAGAGGAAAAAAATGAAA
GAATTCCAAGAAGCCAACTATGCTCGCGTTCGCCGGCGCGGACCCCGCCGCAGCTCCTCT
GACATCGCCCGGGCCAAGATCCAGGGCAAGCGCCACCGGGGTCAGGAGGACTCTAGCCGG
GGCTCAGATAACTCCAGTTACGATGAAGCACTCTCTCCAACATCTCCTGGGCCTTTGTCT
GTGAGAGCAGGGCACGGAGAACGTGACCTGGGGAACCCCAATACAGCCCCGCCCATACCG
GAATTACACGGCATCAACCCTGTGTTCCTGTCCAGCAATCCCAGCCGCTGGAGCGTAGAG
GAGGTGTACGAGTTTATTGCTTCTCTACAAGGCTGTCAAGAGATTGCAGAGGAATTTCGT
TCCCAGGAGATTGATGGACAGGCCCTTTTATTACTTAAAGAGGAACATCTTATGAGTGCC
ATGAACATCAAGCTAGGCCCTGCCCTCAAGATCTGCGCCAAGATAAATGTCCTCAAGGAG
ACC

>Orcinus orca XM_012537227.1
ATGGAGACTGAGAGTGAGCAGAGCTCCAACTCCACCAATGGAAGCTCCGGCTCTG
GGGGCAGCTCTCGGCCCCAGATAGCTCAGATGTCACTGTACGAACGGCAAGCAGTGCAGGCTCTGCAGGC
ACTGCAGCGTCAGCCCAACGCGGCTCAGTATTTCCACCAGTTCATGCTCCAGCAGCAGCTCAGCAATGCC
CAGCTGCACAGCCTGGCTGCCGTCCAGCAGGCCACGATTGCTGCCAGTCGGCAGGCCAGCTCCCCAAACA
CCAGCCCTGCCCAGCAGCAGACCACCACCACCCAGGCCTCCATCAATCTGGCCACCACGTCGGCCGCCCA
GCTCATCAGCCGATCCCAGAGTGTGAGCTCTCCCAGTGCTACCACTTTGACCCAGTCTGTGCTACTGGGG
AACACCACCTCCCCACCCCTCAACCAGTCCCAGGCCCAGATGTATCTACGGCCACAGCTGGGAAACCTGT
TGCAGGTAAACCGGACCCTGGGCCGGAATGTGCCTCTAGCCTCCCAGCTCATCCTGATGCCCAACGGGGC
GGTGGCCGCGGTCCAGCAGGAGGCGCCATCTGCTCAGCCTCCGGGAGTTCACGCAGATGCCGATCAGGTG
CAGAACTTGGCAGTCAGGAACCAACAGGCCTCAGCCCAAGGACCCCAAATGCAAGGCTCTGCTCAGAAGG
CCATTCCTCCTGGAGCCTCCCCTGTCTCCAGCCTCTCCCAGGCCTCTAGCCAGGCCCTCGCTGTGGCTCA
GGCTTCCTCTGGGGCCTCAGGCCAGTCCCTCAACCTTAGTCAAGCTGGTGGAGGCAGTGGGAATAGCATC
CCAGGGTCCATGGGTCCAGGTGGCCAGACACCTGGGGGCTTGGGTCAGTTGCCTTCCTCAGGAATGGGTG
GTGGTGGGAGCTGTCCCAGGAAGGGCACAGGAGTGGTGCAGCCCTTGCCTGCAGCCCAGGCAGTGACTGT
GAGTCAGGGCAGCCAGACAGAAGCAGAAAGTGCAGCGGCCAAGAAGGCAGAAGCAGATGGGACTGGTCAG
CAGAGCGTGGGCATGAACCTGACACGGACAGCCACACCTGCTCCCAGCCAGACCCTTATTAGCTCAGCCA
CCTACACGCAGATCCAGCCCCACTCCCTGATCCAGCAGCAGCAGCAGATCCACCTGCAGCAGAAGCAGGT
GGTGATCCAGCAGCAGATCGCCATCCACCACCAGCAGCAGTTCCCGCACCGCCAGGCGCAGCTGCTGCAC
ACGGCCACCCACCTCCAGCTGGCCCAGCAGCAGCAGCAGCAGCAGCAGCAGCAGCAGCAGCAGCAGGCCG
CGCCCCTCCCTGCCCCCCCACCCCCGCAGGGCCCCCCCGCCCAGCAGGCCCCGCCTTCGCAGTCCCAGCA
GCCAGCCCAGACTCTGGTTGTCCAGCCCATGCTGCAGTCTTCGCCCTTGTCGCTCCCTCCTGACCCAGCC
CCCAAGCCCCCCGTCCCCATCCAGTCCAAGCCACCTGTGGCCCCTGTCAAGCCTCCTCAGCTGGGGGCTG
CCAAGATGTCAGCCACCCAGCAACCTCCGCCCCACATCCCCGTGCAAGTCGTAGGCACCCGGCAGCCAGG
TACAGCCCAGGCCCAGGCTTTGGGGCTGGCCCAGCTGGCAGCCGCCGTGCCTGCTTCCCGGGGGATGGCA
GGCACAGTGCAGCCTGGCCAGGCCCACGTGGCCTCTTCGCCACCTTCATCCCAGGCCCCTGGTGCGCTGC
AGGAGGGCCCGCCCGCGTTGGCTTCGGGGATGACCCTGGCTCCTGTGCAGGGGACGGCACATGTGGTGAA
GGGGGCGGCTACCGCCTCCTCGCCTGTTGTGGCCCAGGTCCCTGCCGCCTTCTACATGCAGTCTGTGCAC
CTGCCGGGCAAACCCCAGACATTGCCTGTCAAACGCAAGGCTGAGTCAGAGGAGGAGAGAGACGATATCT
CCACGTTGAGTTCAATGCTTCCTGCGAAGGCGTCTCCGGTAGTAGAGAGCCCGAAGGTCATGGAGGAGAA
GGGCGGTGTTGGAGACAAAGCTGAACCAGTGACCAGTGTGAATGCTGGTACCCCAAGCAGTGATGTAGTA
GCCTTGGCTCCTGCCCCGTCAGCACCGCCTCCCACGCTGGCCATGGTGTCCAGACAGATGGGTGACTCCA
AGCCCCCACAGGCCATTGTGAAGCCCCAGATTCTCACCCACATCATCGAAGGCTTCGTTATCCAGGAAGG
AGCAGAACCTTTTCCGGTGGGTTGTTCTCAGTTACTGAAAGAGTCTGAGAAGCCACTGCAGACTGGCCTC
GTGACAGGGCTGAATGAGAATCAGTCGGGTGGCCCCTTAGGTGGGGACAGCCCATCTGCTGAGCTCGATA
AGAAGGCGAACCTCCTGAAGTGCGAGTACTGTGGGAAGTACGCCCCTGCAGAGCAGTTTCGCGGCTCCAA

GAGATTCTGCTCCATGACTTGCGCAAAGAGGTATAATGTGAGCTGTAGCCACCAATTCCGGCTGAAGAGG
AAAAAAATGAAAGAGTTTCAAGAAGCCAACTATGCCCGCGTTCGTCGGCGCGGACCCCGCCGCAGCTCCT
CCGACATCGCCCGTGCCAAGATCCAGGGCAAGCGTCACCGGGGTCAAGAGGACTCTAGCCGGGGTTCAGA
TAATTCCAGTTACGATGAAGCACTCTCTCCAACATCTCCTGGGCCTTTATCCGTGCGAGCAGGGCATGGA
GAACGTGACCTGGGGAACCCCAATACAGCTCCGCCTACACCAGAATTACATGGCATCAACCCTGTGTTCC
TGTCCAGTAATCCTAGCCGTTGGAGCGTAGAGGAGGTGTATGAGTTTATTGCTTCTCTACAAGGCTGCCA
AGAGATTGCAGAGGAGTTTCGTTCCCAGGAGATTGATGGACAGGCCCTTTTATTACTTAAAGAGGAACAT
CTTATGAGTGCCATGAACATCAAGCTGGGCCCTGCCCTCAAGATCTGCGCCAAGATAAACGTCCTCAAGG
AGACC

>Lipotes vexillifer XM_007469360.1
ATGGAGACTGAGAGTGAGCAGAGCTCCAACTCCACCAATGGGAGCTCCGGCTCTGGGGGCAGC
TCTCGGCCCCAGATAGCTCAGATGTCACTGTACGAACGGCAAGCAGTGCAGGCTCTGCAGGCACTGCAGC
GTCAGCCCAACGCGGCTCAGTATTTCCACCAGTTCATGCTCCAGCAGCAGCTCAGCAATGCCCAGCTGCA
TAGCCTGGCTGCCGTCCAGCAGGCCACGATTGCTGCCAGTCGGCAGGCCAGCTCCCCAAACACCAGCCCT
GCCCAGCAGCAGACCACCACCACCCAGGCCTCTATCAATCTGGCCACCACGTCGGCCGCCCAGCTCATCA
GCCGATCCCAGAGTGTGAGCTCTCCCAGTGCTACCACTTTGACCCAATCTGTGCTACTGGGGAACACCAC
CTCCCCACCCCTCAACCAGTCCCAGGCCCAGATGTATCTACGGCCACAGCTGGGAAACCTGTTGCAGGTA
AACCGGACCCTGGGCCGGAATGTGCCTCTAGCCTCCCAGCTCATCCTGATGCCCAACGGGGCGGTGGCCG
CGGTCCAGCAGGAGGCGCCATCTGCTCAGCCTCCGGGAGTTCACGCAGATGCTGATCAGGTGCAGAACTT
GGCAGTCAGGAACCAACAGGCCTCAACCCAAGGACCCCAAATGCAAGGCTCTGCTCAGAAGGCCATTCCT
CCTGGAGCCTCCCCTGTCTCCAGCCTCTCCCAGGCCTCTAGCCAGGCCCTCACTGTGGCTCAGGCTTCCT
CTGGGGCCTCAGGCCAGTCCCTCAACCTCAGTCAAGCTGGTGGAGGCAGTGGGAATAGCATCCCAGGGTC
CATGGGTCCAGGTGGCCAGACACCTGGGGGCTTGGGTCAGTTGCCTTCCTCAGGAATGGGTGGTGGTGGG
AGCTGTCCCAGGAAGGGCACAGGAGTGGTGCAGCCCTTGCCTGCTGCCCAGGCAGTGACTGTGAGTCAGG
GCAGCCAGACAGAAGCAGAAAGTGCAGCGGCCAAGAAGGCAGAAGCAGATGGGACTGGTCAGCAGAGCGT
GGGCATGAACCTGACACGGACAGCCACACCTGCTCCCAGCCAGACCCTTATTAGCTCAGCCACCTACACG
CAGATCCAGCCCCACTCCCTGATCCAGCAGCAGCAGCAGATCCACCTGCAGCAGAAGCAGGTGGTGATCC
AGCAGCAGATCGCCATCCACCACCAGCAGCAGTTCCCGCACCGCCAGGCGCAGCTGCTGCACACGGCCAC
CCACCTCCAGCTGGCCCAGCAGCAGCAGCAGCAGCAGCAGCAGCAGCAGCAGCAGCAGGCCGCGCCCCTC
CCTGCCCCCCAACCCCCGCAGGGCCCACCCGCCCAGCAGGCCCCGCCTTCGCAGTCCCAGCAGCCAGCCC
AGACTCTGGTTGTCCAGCCCATGCTGCAGTCTTCACCCTTGTCGCTCCCTCCTGACCCAGCCCCCAAGCC
CCCCGTCCCCATCCAGTCCAAGCCACCTGTGGCCCCTGTCAAGCCTCCTCAGCTGGGGGCTGCCAAGATG
TCAGCCACCCAGCAACCTCCGCCCCACATCCCCGTGCAAGTCGTGGGCACCCGACAGCCAGGTACAGCCC
AGGCCCAGGCTTTGGGGCTGGCCCAGCTGGCAGCTGCCGTGCCTGCTTCCCGGGGAATGCCAGGCACAGT
GCAGCCTGGCCAGGCCCACTTGGCCTCCTCGCCGCCTTCATCCCAGGCCCCTGGTGCGCTGCAGGAGTGC
CCGCCCACATTGGCCTCGGGGATGACCCTGGCTCCTGTGCAGGGGACGGCACACGTGGTGAAGGGGGCAG
CTACCGCCTCCTCACCTGTTGTGGCCCAGGTCCCTGCCGCCTTCTACATGCAGTCTGTGCACCTGCCGGG
CAAACCCCAGACATTGCCCGTCAAACGCAAGGCTGAGTCAGAGGAGGAGAGAGACGATATCTCCACATTG
AGTTCAATGCTCCCTGCGAAGGCATCTCCGGTAGTAGAGAGCCCGAAGGCCACGGAGGAGAAGGGCGGTC
TCGGAGACAAAGCTGAACCAGTGACCAGTGTGAATGCTGGTACCCCGAGCAGTGATGTAGTAGCCTTGGC
TCCTGCCCCACCAGCACCACCTCCCACGCTGGCCATGGTGTCCAGACAGATGGGTGACTCCAAGCCCCCA
CAGGCCATTGTGAAGCCCCAGATTCTCACCCACATCATCGAAGGCTTCGTTATCCAGGAAGGAGCAGAAC
CTTTTCCGGTGGGTTGTTCTCAGTTACTGAAAGAGTCTGAGAAGCCACTGCAGACTGGCCTCGTGACAGG
GCTGAATGAGAATCAGTCGGGTGGCCCCTTAGGTGGGGACAGCCCATCTGCTGAGCTAGATAAGAAGGCG
AACCTCCTGAAGTGCGAGTACTGTGGGAAGTACGCCCCTGCAGAGCAGTTTCGCGGCTCCAAGAGATTCT
GCTCCATGACTTGCGCGAAGAGGTATAATGTGAGCTGTAGCCACCAATTCCGGCTGAAGAGGAAAAAAAT

GAAAGAGTTTCAAGAAGCCAACTATGCCCGCGTTCGCCGGCGCGGACCCCGCCGCAGCTCCTCCGACATC
GCCCGTGCCAAGATCCAGGGCAAGCGTCACCGGGGTCAAGAGGACTCTAGCCGGGGTTCAGATAATTCCA
GTTACGATGAAGCACTCTCTCCAACATCTCCTGGGCCTTTATCCGTGCGAGCAGGGCATGGAGAACGTGA
CCTGGGGAACCCCAATACAGCTCCGCCTACACCAGAATTACATGGCATCAACCCTGTGTTCCTGTCCAGT
AATCCTAGCCGTTGGAGCGTAGAGGAGGTGTATGAGTTTATTGCTTCTCTACAAGGCTGCCAAGAGATTG
CAGAGGAGTTTCGTTCCCAGGAGATTGATGGACAGGCCCTTTTATTACTTAAAGAGGAACATCTTATGAG
TGCCATGAACATCAAGCTGGGCCCTGCCCTCAAGATCTGCGCCAAGATAAACGTCCTCAAGGAGACC

>Erinaceus europaeus XM_007537630.2
ATGGAGACTGAGAGCGAGCAGAACTCTGGCTC
CACCAGCGGGGGTTCCAGCTCCGGAGGCAGCTCTCGGCCCCAGATAGCACAGATGTCCCTGTATGAGCGC
CAGGCAGTGCAGGCTTTGCAGGCCCTGCAACGTCAGCCCAATGCAGCTCAGTATTTCCACCAGTTCATGC
TCCAGCAGCAGCTCAGCAATGCCCAGCTGCACAGCCTGGCCGCCGTCCAGCAGGCCACAATTGCTGCCAG
TCGGCAGGCCAGCTCCCCAAACACCAGCACAGCCCAGCAGCAGACCACCACTACCCAGGCCTCAATCAAT
CTGGCCACCACGTCGGCTGCCCAGCTCATCAGCCGATCCCAGAGTGTGAGCTCTCCCAGTGCTGCCACTC
TGACCCAGTCTGTGCTTCTAGGGAACACCACCTCTCCGCCCCTCAACCAGTCCCAGGCCCAGATGTATCT
ACGGCCACAGCTGGGAAACCTATTGCAGGTAAACCGGACCCTGGGCCGGAATGTGCCTCTAACCTCCCAA
CTCATCCTGATGCCTAACGGGGCGGTGGCTGCAGTCCAGCAGGAGGTGCCATCTGCTCAGTCTCCGGGAG
TCCATGCAGACGCAGATCAGGTGCAAAACTTGGCAGTGAGGAATCAACAGGCCTCAGCCCAAGGACCCCA
AATGCAAGGCTCCACTCAGAAGGCCATTCCTCCTGGAGCCTCCCCTGTGTCTAGTCTCTCACAGGCCTCT
GGTCAGGCCTTAGCTGTGGCTCAGGCTTCCTCTGGGGCCGCAGGCCAGTCCCTGAACCTTAGTCAGGCTG
GTGGAGGCAGTGGGAATAGTATTGCAGGACCCCTGGGGCCAGGAGGAGGTGGCCAGCCACCGGGGGCTTT
GGGTCAGTTGCCTTCCTCAGGACTGGGTGGTAGTGGGAGTTGTCCCAGGAAGGGCACAGGAGTAGTACAG
CCCTTACCTGCAGCCCAGACAGTGACTGTGAGCCAGGGCAGCCAGACAGAAGCAGAAAGTGCAGCGGCCA
AGAAGGCAGAAGCAGATGGGACAGGTCAGCAGAACGTGGGCATGAACCTGACCCGTACAGCAACACCTGC
TCCCAGCCAGACCCTCATTAGTTCAGCCACATACACACAGATCCAGCCTCACTCGCTGATCCAGCAGCAG
CAACAGATCCACCTCCAGCAGAAACAGGTGGTGATCCAGCAGCAGATCGCCATCCACCACCAGCAGCAGT
TCCAGCACCGGCAGTCGCAGCTCCTGCATACGGCCACGCACCTCCAGCTGGCACAGCAGCAGCAGCAACA
GCAGCAGCAGCAAACCCCCACGCTCACCGCCCCTCAGCCCCCACAGGTCCCACCCACTCAGCAGGTCCCA
CCTTCCCAGACCCAGCAGCAAGCCCAGACCTTGGTTGTTCAGCCTATGCTACAATCGTCACCCTTGTCGC
TCCCACCTGATCCCACTCCCAAGCCAACCATTCCAATCCAGTCCAAACCACCTATAGCACCCATCAAGCC
TCCTCAGTTAGGGGCCGCGAAGATGTCCTCGGCCCAGCAGCCGCCACCCCACATCCCTGTGCAAGTTGTG
GGAAGCAGGCAGCCAGGCAGTGCCCAGGCACAGGCTTTGGGCTTAGCTCAGCTGGCAACAGCCATGCCTG
CTTCCCGGGGCATGGCAGGCACAGTGCAGCCTGGCCAGGCCCATTTGGCCTCCTCACCACCAGTGTCCCA
GGCTCCGGGCACACTGCAGGAGTGCCCCCCCACAATGGCCACAGGGATGACCCTTGGGCCAGTTCAGGGG
ACAGCCCACGTGGTGAAGAGTGGGGCTACCACCTCGCCTGTTGTTGCCCAGGTCCCTGCAGCCTTCTACA
TGCAGTCTGTGCACCTGCCGAGCAAACCCCAGATGTTGGCTGTCAAACGCAAAGCTGAGTCTGAGGAGGA
GAGAGACGATGTCTCCACATTGGGGTCAATGCTCCCTGCCAAGGCATCTCCAGTAGCAGAGAGCCCAAAG
ATCATGGAAGAGAAGAGCAGTCTTGGAGAGAAAGTTGAACCAGTGACCAGTGTAAATAATACTCAAAGCA
GTGAACTAGTAGCCTTGGCCCCAGCTCCATCAGTCCCACCTCCCACACTCACCATGATTTCCAGGCAGAT
AGGTGATTCCAAACCCCCCCAGGCCATTGTGAAGCCCCAGATCCTCACCCACATCATTGAAGGCTTTGTT
ATCCAGGAAGGAGCGGAACCTTTCCCTGTGGGTTGTTCTCAGATACTCAAGGAGTCTGAGAAGCCACTAC
AGATGGGCCTCTCCACAGGACCGAGTGAGAATCAGCCAGGTGTCCGAACAGACAGCCCACCTGCAGAATT
AGAGAAGAAGGCCAGCCTCCTCAAGTGTGAGTACTGCGGCAAGTACGCCCCTGCGGAGCAATTTCGTGGT
TCCAAGAGATTCTGCTCTATGACCTGTGCTAAGAGGTATAATGTGAGCTGTAGCCACCAGTTCCGACTGA
AGAGGAAAAAAATGAAGGAGTTTCAGGAAGCCAATTATGCCCGAGTTCGCCGGCGTGGACCTCGCCGCAG

CTCCTCAGACATTGCCCGTGCCAAGATCCAGGGCAAACGCCACCGGGGTCAAGAGGACTCAAGCCGGGGT
TCAGATAATTCCAGTTACGATGAAGCACTCTCTCCAACATCTCCTGGGCCTTTGTCAGTGAGAGCTGGAC
ATGGAGATCGAGACCTGGGGAACCCCAACACAGCTCCACCGACGCCAGAATTACATGGGATCAATCCTGT
GTTCCTCTCTAGTAATCCCAGCCGTTGGAGTGTAGAGGAGGTGTATGAGTTTATTGCTTCCCTACAAGGC
TGCCAAGAGATTGCAGAAGAGTTTCGCTCCCAGGAAATTGATGGACAGGCCCTTTTATTACTTAAAGAAG
AACATCTCATGAGTGCCATGAACATCAAACTGGGCCCTGCCCTCAAGATCTGCGCCAAGATAAATGTCCT
CAAGGAGACC
>dog XM_014108505.1
ATGGAGACTGAGAGTGAGCAGAACTCCAACTCCACCAATGGGAGCTCCAGCTCTGGGGGCAGC
TCTCGGCCCCAGATAGCTCAGATGTCACTGTATGAACGGCAAGCGGTGCAGGCTCTACAGGCACTGCAGC
GTCAGCCCAATGCAGCTCAGTATTTCCACCAGTTCATGCTCCAGCAGCAGCTCAGCAATGCCCAACTGCA
TAGCCTGGCTGCGGTCCAGCAGGCCACGATTGCTGCCAGTCGCCAGGCCAGCTCTCCAAACACCAGCACT
GCACAGCAGCAGACTACCACCAGCCAGGCCTCGATCAATCTGGCCACCACGTCGGCCGCCCAGCTCATCA
GCCGATCCCAGAGTGTGAGCTCTCCCAGTGCTACCACTTTGACCCAATCTGTGCTACTGGGAAACACCAC
CTCCCCACCTCTCAACCAGTCCCAGGCCCAGATGTATCTACGGCCACAGCTGGGAAACCTATTGCAGGTA
AACCGGACCCTGGGCCGGAATGTGCCTCTAGCCTCCCAACTCATCCTGATGCCTAACGGGGCAGTGGCTG
CAGTCCAGCAGGAGGTGCCATCTGCTCAGTCTCCGGGAGTTCATACAGATGCAGACCAGGTGCAGAATTT
GGCAGTGAGGAACCAACAGGCCTCGGCCCAAGGACCCCAAATGCAAGGCTCTGCTCCGAAGGCCATTCCT
CCTGGAGCCTCCCCTGTCTCCAGCCTCTCCCAGGCCTCCAGCCAGGCCCTTGCTGTAGCTCAGGCGTCCT
CTGGGGCCTCAGGCCAGTCCCTCAACCTCAGTCAAGCTGGTGGAGGCAGTGGGAGTAGCATCCCAGGGTC
CCTGGGTCCCGGTGGAGGTGGCCAGGCCCCTGGCGGCTTGGGCCAGTTGCCTTCCTCAGGAATGGGTGGG
GGTGGGAGCTGTCCCAGGAAGGGCACAGGAGTGGTGCAGCCCTTACCTGCAGCCCAAGCAGTGACTGTGA
GTCAGGGAAGCCAGACAGAAGCAGAGAGTGCAGCAGCCAAGAAGGCAGAAGCAGATGGGGCTGGTCAGCA
GAACGTGGGCATGAACCTGACACGGACAGCTACCCCTGCTCCCAGCCAGACCCTTATTAGCTCAGCCACC
TACACGCAGATCCAGCCCCATTCCTTGATTCAGCAACAGCAGCAGATCCACCTCCAGCAGAAGCAGGTGG
TAATCCAGCAGCAGATCGCCATCCACCACCAGCAGCAGTTCCAGCACCGGCAGTCCCAGCTCCTCCACAC
AGCCACACACCTCCAGTTGGCCCAGCAGCAGCAGCAACAGCAGCAGCAGCCAGCACCGCCGCCGCCACCA
CCACCACCGCCACCACCGGCCACAACCCTCACTGCCCCTCAGCCACCGCAGGTCCCACCTACTCAGCAGG
TCCCACCTTCCCAGTCTCAGCAGCAAGCCCAGACCCTGGTTGTGCAACCCATGCTGCAGTCTTCGCCCTT
GTCCCTCCCACCTGACCCAACCCCTAAGCCACCCCTCCCCATCCAGTCCAAGCCACCTGTAGCACCTATC
AAGCCGCCTCAGTTAGGGGCTGCTAAGATGTCAGCCACCCAGCAGCCACCACCCCATATCCCTGTGCAAG
TCGTGGGCACCCGGCAGCCAGGGACAGCCCAGGCACAGGCTTTGGGCTTGGCGCAGCTGGCGGCTGCCGT
ACCTACGTCCCGGGGGATGCCCAATACGGTACAGCCCGGGCAGGCCCATTTGGCCTCCTCACCGCCTTCG
TCCCAGGCTCCTGGTGCGCTGCAGGAGTGCCCCCCAACGCTAGCCTCTGGGATGAGCCTGGCTCCGGTGC
AGGGGACCGCACATGTGGTAAAGGGGGCTACAACATCCTCCCCTGTTGTAGCCCAGGTACCTGCTGCCTT
CTACATGCAGTCTGTGCACCTGCCGAGCAAACCACAGACGTTGGCTGTCAAACGCAAGGCAGAGTCTGAA
GAGGAGAGAGACGATGTTTCCACACTGGGTTCAATGCTTCCTGCCAAGGTGTCTCCAGTAGCCGAGAGCC
CAAAGGCCATGGAGGAGAAGAACAGTCTTGGAGAGAAAGCTGAACCAGTGACTGGTATGAACGCTAATAC
TCCAAGCAGTGAACTAGTAGCCTTGGCTCCTGCCCCATCCGCACCGCCTCCTACACTAGCCATGGTGTCC
AGACAAATGGGTGACTCGAAACCCCCACAGGCCATCGTGAAGCCTCAGATTCTCACCCACATCATTGAAG
GCTTCGTTATCCAGGAAGGAGCAGAGCCTTTCCCGGTGGGTTGTTCTCAGTTACTGAAAGAGTCTGAGAA
GCCATTACAGACTGGCCTCCCGACAGGGCTGAATGAGAATCAGTCGGTGGGCCCCTTGGGAGGGAACAGC
CCATCTGCTGAGCTCGATAAGAAGGCGAACCTCCTGAAGTGTGAGTACTGCGGAAAGTATGCTCCTGCTG
AGCAGTTTCGAGGCTCCAAGAGATTCTGCTCCATGACCTGCGCTAAGAGGTATAATGTGAGCTGTAGTCA
CCAGTTCCGGGTAAAGAGGAAAAAAATAAAAGATTTCCAAGAATCCAACTATGCTCGTGTTCGTCGGCGT
GGGCCTCGCCGCAGCTCCTCCGACATCGCCCGTGCCAAGATCCAGGGCAAGCGCCACCGGGGTCAAGAGG
ACTCTAGCCGGGGTTCAGATAATTCCAGTTACGATGAAGCACTCTCTCCAACATCTCCTGGGCCTTTATC

TGTGAGAACTGGGCATGGAGAACGTGACCTGGGGAACCCCAATACAGCGCCACTGACACCAGAATTACAT
GGCATCAACCCTGTGTTCCTGTCCAGCAATCCCAGCCGTTGGAGTGTAGAAGAGGTGTATGAGTTTATTG
CTTCTCTACAAGGCTGCCAAGAGATTGCAGAGGAGTTTCGTTCCCAGGAGATTGATGGACAGGCCCTTTT
ATTACTTAAAGAGGAACATCTTATGAGTGCCATGAACATCAAGTTGGGCCCCGCCCTCAAGATCTGCGCC
AAGATAAATGTCCTCAAGGAGACC
>Manis javanica XM_017658843.1
ATGGAGACTG
AGAGTGAGCAGAACTCCAGCTCCACCAATGGGAGTTCCAGCTCCGGGGGCAGCTCTCGGCCGCAGATAGC
GCAAATGTCGCTGTATGAACGCCAAGCGGTGCAGGCTCTTCAGGCACTGCAGCGTCAGCCCAATGCAGCT
CAGTATTTCCACCAGTTCATGCTCCAGCAGCAGCTCAGCAATGCCCAGCTGCATAGCCTGGCTGCTGTCC
AGCAGGCCACAATTGCAGCCAGTCGGCAGGCCAGCTCCCCGAACACCAGCACTGCCCCGCCGCAGACTCC
CACCACCCAGGCCTCAATCAATCTGGCCACCACGTCGGCCGCCCAGCTCATCAGCCGATCCCAGAGTGTG
AGCTCTCCCAGTGCTACCACCTTGACTCAGTCTGTGCTACTGGGGAACACCACCTCCCCACCCCTCAACC
AGTCCCAGGCCCAGATGTATCTACGGCCACAGCTGGGAAACCTGTTGCAGGTAAACCGGACCCTGGGCCG
GAATGTGCCTCTAGCCTCCCAGCTCATCCTGATGCCTAACGGGGCAGTGGCTGCAGTCCAGCAGGAGGTA
CCATCCGCTCAGTCTCCCGGAGTTCATACAGATACAGATCAGGTGCAGAACTTGGCAGTGAGGAACCAAC
AGGCCTCAGCCCAAGGACCCCAAATGCAAGGCTCTACCCAGAAGGCCATTCCTCCTGGAGCCAGCCCTGT
CTCCAGCCTCTCCCAAGCCTCCAGCCAGGCCCTGGCTGTGGCTCAGGCTTCTTCTGGGACCTCAGGCCAG
TCCCTCAACCTTAGTCAAGCTGGTGGAGGCAGTGGGAGTAGCATCCCGGGGTCCATGGGTCCAGGTGGAG
GTGGCCAGCCACCTGGGGGCTTGGGTCAGTTGCCTTCCTCAGGAATGGGTGGCGGGAGCTGTCCCAGGAA
GGGCACAGGAGTGGTGCAGCCTTTGCCTGCAGCCCAGACAGTGACTGTGAGTCAGGGCAGCCAGACAGAG
GCAGAAAGTGCAGCAGCCAAGAAGGCGGACACAGATGGGCCTGCTCAGCAGAATGTGGGCATGAACCTGA
CACGGACAGCTACACCTGCTCCCAGCCAGACCCTGATTAGCTCAGCCACCTACACACAGATCCAGCCCCA
CTCCCTGATTCAGCAGCAGCAGATCCACCTCCAGCAGAAGCAGGTGGTGATCCAGCAGCAGATCGCCATC
CACCACCAGCAGCAGTTCCAGCACCGCCAGTCGCAGCTGCTGCACACGGCCACGCACCTCCAGCTGGCCC
AGCAGCCGCCTCCGCAAGCCACAGCCCTGTCTGCCCCTCAGCCTCCGCCAGTCCCACCTGCCCAGCCTGT
CCCGCCTTCCCAGTCCCAGCAGCAGGCCCAGACCCTGGTGGTCCAGCCCATGCTTCAGTCTTCACATTTG
TCCCTCCCACCTGACCCAGCCCCCAAGCCACCCATCCCCATCCAGTCCAAACCACCAGTAGCACCCATCA
AGCCTCCTCAGTTAGGGGCTGCTAAGATGTCAGCTACCCAGCAGCCACCTCCGCATATTCCTGTGCAAGT
TGTGGGCACCCGACAGCCAGGTACAGCCCAGGCCCAGGCTTTGGGACTGGCACAGCTGGCAGCTGCTGTT
CCTTCTTCCCGGGGAATGCCGGGCGCACTGCAGCCTGGTCAGGCCCATCTGGCTGCCTCGCCACCTTCAT
CCCAGGCTCCTGGTGCACTGCAGGAGTGCCCTCCTACATTGGCCTCTGGGATGACCCTTGGCCCTGTGCA
AGGGACAGCACATGTGGTAAAGGGGGGGACTACCAGCTCCTCACCTGCTGTAGCCCAGGTCCCCGCTGCC
TTCTACATGCAGTCTGTGCACCTGCCGGGCAAGCCCCAGACATTGGCTGTCAAACGCAAGGCAGAGTCTG
AGGAGGAGAGAGATGATGTCTCCACATTAGGTTCAATGCTTCCTGCCAAGGCATCTCCAGTGGCAGAGAG
CCCCAAAGCGATGGAGGAGAAGAGCAGCCTTGGAGAGAGAGCCGAACCCTTGAGCAGTGTGAATGCTAAC
ACCCCAAGCAGTGACCTAGTAGCCTTGGCTCCTGCCCCATCAGCACCGCCTCCTACACTAGCCATGGTGT
CCAGACAGATGGGTGACTCAAAACCCCCACAGGCCATTGTGAAGCCCCAGATTCTCACCCACATCATTGA
AGGCTTCGTTATCCAGGAAGGAGCAGAACCTTTCCCGGTGGGTTGTTCTCAGTTACTGAAAGAGTCTGAG
AAGCCACTGCAGACTGGCCTCCCAACAGGGCTGAAGGAGAATCAGTCAGGTGGCCTCTTGGGAGGGGACA
GCCCATCTGCTGAGCTAGATAAGAAGGCGAATCTCCTGAAGTGTGAGTACTGTGGGAAGTACGCCCCTGC
AGAGCAGTTTCGTGGCTCCAAGAGATTCTGCTCCATGACTTGTGCCAAGAGGTATAATGTGAGCTGTAGC
CATCAGTTCCGGCTGAAGAGGAAAAAAATGAAAGAATTTCAAGAAGCCAACTATGCTCGAGTTCGCCGAC
GTGGACCCCGCCGCAGCTCCTCTGACATCGCCCGTGCCAAGATCCAGGGCAAACGCCACCGGGGTCAAGA
GGACTCAAGCCGGGGCTCAGATAATTCCAGTTACGATGAAGCACTCTCTCCAACATCTCCGGGGCCTTTG
TCCGTGAGAGCGGGGCATGGAGAACGTGACCTGGGGAACCCCAGTACAGCTCCACCAACACCGGAATTAC
ATGGCATCAACCCTGTGTTCCTGTCTAGTAATCCGAGCCGCTGGAGTGTAGAGGAGGTGTATGAGTTTAT

CGCCTCTCTACAAGGCTGCCAAGAGATTGCAGAGGAGTTTCGTTCCCAGGAGATTGATGGACAGGCCCTT
TTATTACTTAAAGAAGAACATCTTATGAGTGCCATGAATATCAAGCTGGGCCCTGCCCTCAAGATCTGCG
CCAAGATAAATGTCCTCAAGGAAACC

CDK6

>human ENST00000424848.2
ATGGAGAAGGACGGCCTGTGCCGCGCTGACCAGCAGTACGAATGCGTGGCGGAGATCGGG
GAGGGCGCCTATGGGAAGGTGTTCAAGGCCCGCGACTTGAAGAACGGAGGCCGTTTCGTG
GCGTTGAAGCGCGTGCGGGTGCAGACCGGCGAGGAGGGCATGCCGCTCTCCACCATCCGC
GAGGTGGCGGTGCTGAGGCACCTGGAGACCTTCGAGCACCCCAACGTGGTCAGGTTGTTT
GATGTGTGCACAGTGTCACGAACAGACAGAGAAACCAAACTAACTTTAGTGTTTGAACAT
GTCGATCAAGACTTGACCACTTACTTGGATAAAGTTCCAGAGCCTGGAGTGCCCACTGAA
ACCATAAAGGATATGATGTTTCAGCTTCTCCGAGGTCTGGACTTTCTTCATTCACACCGA
GTAGTGCATCGCGATCTAAAACCACAGAACATTCTGGTGACCAGCAGCGGACAAATAAAA
CTCGCTGACTTCGGCCTTGCCCGCATCTATAGTTTCCAGATGGCTCTAACCTCAGTGGTC
GTCACGCTGTGGTACAGAGCACCCGAAGTCTTGCTCCAGTCCAGCTACGCCACCCCCGTG
GATCTCTGGAGTGTTGGCTGCATATTTGCAGAAATGTTTCGTAGAAAGCCTCTTTTTCGT
GGAAGTTCAGATGTTGATCAACTAGGAAAAATCTTGGACGTGATTGGACTCCCAGGAGAA
GAAGACTGGCCTAGAGATGTTGCCCTTCCCAGGCAGGCTTTTCATTCAAAATCTGCCCAA
CCAATTGAGAAGTTTGTAACAGATATCGATGAACTAGGCAAAGACCTACTTCTGAAGTGT
TTGACATTTAACCCAGCCAAAAGAATATCTGCCTACAGTGCCCTGTCTCACCCATACTTC
CAGGACCTGGAAAGGTGCAAAGAAAACCTGGATTCCCACCTGCCGCCCAGCCAGAACACC
TCGGAGCTGAATACAGCC
>marmoset ENSCJAT00000027002.2
ATGGAGAAGGACGGCCTGAGCCGCGCGGACCAGCAGTACGAATGCGTAGCGGAGATCGGG
GAGGGCGCCTATGGGAAGGTGTTCAAGGCCCGAGACTTGAAGAACGGAGGCCGTTTCGTG
GCGTTGAAGCGCGTGCGGGTGCAGACCGGCGAGGAGGGCATGCCGCTCTCCACCATCCGC
GAGGTGGCGGTGCTGAGGCACCTGGAGACCTTCGAACACCCCAACGTGGTCAGGTTGTTT
GATGTGTGCACAGTGTCACGAACTGACAGAGAAACCAAACTAACTTTAGTGTTTGAACAT
GTCGATCAAGACTTGACCACTTACTTGGATAAAGTTCCAGAGCCTGGAGTGCCCACTGAA
ACCATAAAGGATATGATGTTTCAGCTTCTCCGAGGTCTGGACTTTCTTCATTCACACCGA
GTAGTGCATCGCGATCTAAAACCACAAAACATTCTGGTGACCAGCAATGGACAAATAAAA
CTCGCTGACTTCGGCCTTGCCCGCATCTACAGTTTCCAGATGGCTCTAACTTCAGTGGTC
GTCACGCTGTGGTACAGAGCGCCCGAAGTCTTGCTCCAGTCCAGCTACGCCACCCCCGTG
GATCTCTGGAGTGTTGGCTGCATATTTGCAGAAATGTTTCGTAGAAAGCCTCTTTTTCGT
GGAAGTTCAGATGTCGATCAACTAGGAAAAATCTTGGACGTAATTGGACTCCCAGGAGAA
GAAGACTGGCCTAGAGATGTTGCCCTTCCCAGGCAGGCTTTTCATGCAAAATCTGCCCAA
CCAATTGAGAAGTTTGTGACAGATATTGATGAACTAGGCAAAGACCTACTTCTGAAGTGT
TTGACATTTAACCCAGCCAAAAGAATATCTGCCTACAGCGCCCTGTCTCACCCATACTTC
CAGGACCTGGAGAGGGGCAGAGAAAACCTGGATTCCCACCTGCCTCCCAGCCAGAACACC
TCCGAGATGAATACAGCC
>vervet agm ENSCSAT00000010706.1
ATGGAGAAGGACGGCCTGTGCCGCGCTGACCAGCAGTACGAATGCGTGGCGGAGATCGGG
GAGGGCGCCTATGGGAAGGTGTTCAAGGCCCGCGACTTGAAGAACGGAGGCCGTTTCGTG
GCGTTGAAGCGCGTGCGGGTGCAGACGGGCGAGGAGGGCATGCCGCTCTCCACCATCCGC
GAGGTGGCGGTGCTGAGGCACCTGGAGACCTTCGAGCACCCCAACGTGGTCAGGTTGTTT
GATGTGTGCACAGTGTCACGAACAGACAGAGAAACCAAACTAACTTTAGTGTTTGAACAT

GTCGATCAAGACTTGACCACTTACTTGGATAAAGTTCCAGAGCCTGGAGTGCCCACTGAA
ACCATAAAGGATATGATGTTTCAGCTTCTCCGAGGTCTGGACTTTCTTCATTCACACCGA
GTAGTGCATCGCGATCTAAAACCACAGAACATTCTGGTGACCAGCAGTGGACAAATAAAA
CTCGCTGACTTCGGCCTTGCCCGCATCTATAGTTTCCAGATGGCTCTAACCTCAGTGGTC
GTCACGCTGTGGTACAGAGCTCCCGAAGTCTTGCTCCAGTCCAGCTACGCCACCCCTGTG
GATCTATGGAGTGTTGGCTGCATATTTGCAGAAATGTTTCGTAGAAAGCCTCTTTTTCGT
GGAAGTTCAGATGTCGATCAACTAGGAAAAATCTTGGACGTAATTGGACTCCCAGGAGAA
GAAGACTGGCCTAGAGATGTTGCCCTTCCCAGGCAGGCTTTTCATTCAAAATCTGCCCAA
CCAATTGAGAAGTTTGTAACAGATATCGATGAACTAGGCAAAGACCTACTTCTGAAGTGT
TTGACATTTAACCCAGCCAAAAGAATATCAGCCTACAGTGCCCTGTCTCACCCATACTTC
CAGGACCTGGAGAGGTGCAAAGAAAACCTGGATTCCCACCTGCCGCCCAGCCAGAACACC
TCGGAGCTGAATACAGCC
>macaque ENSMMUT00000012156.3
ATGGAGAAGGACGGCCTGTGCCGCGCTGACCAGCAGTACGAATGCGTGGCGGAGATCGGG
GAGGGCGCCTATGGGAAGGTGTTCAAGGCCCGCGACTTGAAGAACGGAGGCCGTTTCGTG
GCGTTGAAGCGCGTGCGGGTGCAGACGGGCGAGGAGGGCATGCCGCTCTCCACCATCCGC
GAGGTGGCGGTGCTGAGGCACCTGGAGACCTTCGAGCACCCCAACGTGGTCAGGTTGTTT
GATGTGTGCACAGTGTCACGAACAGACAGAGAAACCAAACTAACTTTAGTGTTTGAACAT
GTCGATCAAGACTTGACCACTTACTTGGATAAAGTTCCAGAGCCTGGAGTGCCCACTGAA
ACCATAAAGGATATGATGTTTCAGCTTCTCCGAGGTCTGGACTTTCTTCATTCACACCGA
GTAGTGCATCGCGATCTAAAACCACAGAACATTCTGGTGACCAGCAGCGGACAAATAAAA
CTCGCTGACTTCGGCCTTGCCCGCATCTATAGTTTCCAGATGGCTCTAACCTCAGTGGTC
GTCACGCTGTGGTACAGAGCTCCCGAAGTCTTGCTGCAGTCCAGCTACGCCACCCCCGTG
GATCTATGGAGTGTTGGCTGCATATTTGCAGAAATGTTTCGTAGAAAGCCTCTTTTTCGT
GGAAGTTCAGATGTCGATCAACTAGGAAAAATCTTGGACGTAATTGGACTCCCAGGAGAA
GAAGACTGGCCTAGAGATGTTGCCCTTCCCAGGCAGGCTTTTCATTCAAAATCTGCCCAA
CCAATTGAGAAGTTTGTAACAGATATCGATGAACTAGGCAAAGACCTACTTCTGAAGTGT
TTGACATTTAACCCAGCCAAAAGAATATCAGCCTACAGTGCCCTGTCTCACCCATACTTC
CAGGACCTGGAGAGGTGCAAAGAAAACCTGGATTCCCACCTGCCGCCCAGCCAGAACACC
TCGGAGCTGAATACAGCC
>microcebus murinus ENSMICT00000004216.2
ATGGAGAAGGACGGCCTGAGCCGCGCCGACCAGCAGTACGAGTGCGTGGCGGAGATCGGG
GAAGGCGCCTATGGCAAGGTGTTCAAGGCCCGCGACCTGAAGAACGGAGGCCGTTTCGTG
GCTCTGAAGCGCGTGCGGGTGCAGACGGGCGAGGAAGGCATGCCGCTCTCCACCATCCGC
GAGGTGGCGGTGCTGAGGCACCTGGAGACCTTCGAGCACCCCAACGTGGTCAGGTTGTTT
GATGTGTGCACAGTGTCACGAACAGACCGAGAAACCAAACTAACATTAGTCTTTGAACAT
GTTGATCAAGACTTGACCACTTACTTGGATAAAGTTCCAGAGCCTGGAGTGCCCACTGAA
ACCATAAAGGATATGATGTTCCAGCTTCTCCGAGGGCTGGACTTTCTTCATTCACACCGA
GTAGTGCACCGTGATCTAAAACCACAGAACATCCTAGTGACCAGCAGTGGACAAATAAAA
CTGGCTGACTTCGGCCTTGCCCGCATCTACAGTTTTCAGATGGCTCTTACCTCAGTGGTC
GTCACACTGTGGTACAGAGCTCCGGAAGTCTTGCTCCAGTCCAGCTATGCCACCCCCGTG
GATCTCTGGAGTGTTGGCTGCATATTTGCAGAAATGTTTCGTAGAAAGCCTCTTTTTCGT
GGAAGTTCAGATGTCGATCAACTAGGAAAAATCTTGGACGTAATTGGACTCCCAGGAGAA
GAGGACTGGCCTAGAGATGTTGCCCTTCCCAGGCAAGCGTTTCATTCAAAGTCTGCCCAA
CCGATTGAGAAGTTTGTAACGGATATTGATGAACTAGGCAAAGACCTACTTCTGAAGTGT
TTGACATTTAATCCAGCCAAAAGAATATCCGCCTACAGTGCCCTGTCTCACCCATACTTC
CAGGACCTGGAGAGGTGCAAGGAGAACCTGGATTCCCACCTGCCACCTAGCCAGAACCCC

TCGGAGCTGAACACAGCC
>gibbon ENSNLET00000032687.1
ATGGAGAAGGACGGCCTGTGTCGCGCTGACCAGCAGTACGAATGCGTGGCGGAGATCGGG
GAGGGCGCCTATGGGAAGGTGTTCAAGGCCCGCGACTTGAAGAACGGAGGCCGTTTCGTG
GCATTGAAGCGCGTGCGGGTGCAGACGGGCGAGGAGGGCATGCCGCTCTCCACCATCCGC
GAGGTGGCGGTGCTGAGGCACCTGGAGACCTTCGAGCACCCCAACGTGGTCAGGTTGTTT
GATGTGTGCACAGTGTCACGAACAGACAGAGAAACCAAACTAACTTTAGTGTTTGAACAT
GTTGATCAAGACTTGACCACTTACTTGGATAAAGTTCCAGAGCCTGGAGTGCCCACTGAA
ACCATAAAGGATATGATGTTTCAGCTTCTCCGAGGTCTGGACTTTCTTCATTCACACCGA
GTAGTGCATCGCGATCTAAAACCACAGAACATTCTGGTGACCAGCAGCGGACAAATAAAA
CTCGCTGACTTCGGCCTTGCCCGCATCTATAGTTTCCAGATGGCTCTAACCTCAGTGGTC
GTCACGCTGTGGTACAGAGCACCCGAAGTCTTGCTCCAGTCCAGCTACGCCACCCCCGTG
GATCTCTGGAGTGTTGGCTGCATATTTGCAGAAATGTTTCGTAGAAAGCCTCTTTTTCGT
GGAAGTTCAGATGTTGATCAACTAGGAAAAATCTTGGACGTAATTGGACTCCCAGGAGAA
GATGACTGGCCTAGAGATGTTGCCCTTCCCAGGCAGGCTTTTCATTCAAAATCTGCCCAA
CCAATTGAGAAGTTTGTAACAGATATCGATGAACTAGGCAAAGACTTACTTCTGAAGTGT
TTGACATTTAACCCAGCCAAAAGAATATCTGCCTACAGTGCCCTGTCTCACCCATACTTC
CAGGACCTGGAAAGGTGCAGAGAAAACCTGGATTCCCATCTGCCGCCCAGCCAGAACACC
TCGGAGCTGAATACAGCC
>bushbaby ENSOGAT00000027280.1
ATGGAGAAGGACGGCCTGAGCCGCGCTGACCAGCAGTACGAGTGCGTGGCGGAGATCGGG
GAGGGCGCCTACGGAAAGGTGTTCAAGGCCCGCGACTTGAAGAACGGAGGCCGTTTCGTG
GCGCTGAAACGCGTGCGGGTGCAGACGGGCGAGGAGGGCATGCCGCTCTCCACTATCCGC
GAGGTGGCGGTGCTGAGGCACCTGGAGACCTTTGAGCACCCCAACGTGGTCAGGTTGTTT
GATGTGTGCACAGTGTCACGAACAGATCGAGAAACCAAACTAACATTAGTGTTTGAACAT
GTTGATCAAGACTTGACCACTTACTTGGATAAAGTTCCAGAGCCTGGAGTGCCCACTGAA
ACCATAAAGGATATGATGTTCCAGCTTCTCCGAGGCCTGGACTTTCTTCATTCACACCGA
GTAGTGCATCGTGATCTAAAACCACAAAATATTCTGGTGACCAGCAGTGGACAAATAAAA
CTGGCTGACTTTGGCCTTGCCCGCATCTACAGTTTTCAGATGGCTCTTACCTCAGTGGTC
GTCACGCTGTGGTACAGAGCTCCAGAAGTCTTGCTCCAGTCCAGCTATGCCACCCCTGTG
GATCTCTGGAGTGTTGGCTGCATATTTGCAGAAATGTTTCGTAGAAAGCCTCTTTTTCGT
GGAAGTTCAGATGTTGATCAACTAGGAAAAATCTTGGATGTAATTGGACTCCCGGGAGAA
GAGGACTGGCCTAGAGATGTTGCCCTTCCCAGGCAAGCTTTTCATTCAAAATCTGCCCAA
CCAATTGAGAAGTTTGTAACGGATATTGATGAGCTAGGCAAAGACCTACTTCTGAAATGT
TTGACATTTAATCCAGCCAAAAGAATATCCGCCTACAGTGCCCTGTCTCACCCATACTTC
CAGGACCTGGAGAGGCGTAAGGAGAACCTGGATTCCCACCTGCCACCCAGCCAGAACACC
TCAGAGCTGAACACAGCT
>chimpanzee ENSPTRT00000035908.5
ATGGAGAAGGACGGCCTGTGCCGCGCTGACCAGCAGTACGAATGCGTGGCGGAGATCGGG
GAGGGCGCCTATGGGAAGGTGTTCAAGGCCCGCGACTTGAAGAACGGAGGCCGTTTCGTG
GCGTTGAAGCGCGTGCGGGTGCAGACCGGCGAGGAGGGCATGCCGCTCTCCACCATCCGC
GAGGTGGCGGTGCTGAGGCACCTGGAGACCTTCGAGCACCCCAACGTGGTCAGGTTGTTT
GATGTGTGCACAGTGTCACGAACAGACAGAGAAACCAAACTAACTTTAGTGTTTGAACAT
GTCGATCAAGACTTGACCACTTACTTGGATAAAGTTCCAGAGCCTGGAGTGCCCACTGAA
ACCATAAAGGATATGATGTTTCAGCTTCTCCGAGGTCTGGACTTTCTTCATTCACACCGA
GTAGTGCATCGCGATCTAAAACCACAGAACATTCTGGTGACCAGCAGCGGACAAATAAAA
CTCGCTGACTTCGGCCTTGCCCGCATCTATAGTTTCCAGATGGCTCTAACCTCAGTGGTC

GTCACGCTGTGGTACAGAGCACCCGAAGTCTTGCTCCAGTCCAGCTACGCCACCCCCGTG
GATCTCTGGAGTGTTGGCTGCATATTTGCAGAAATGTTTCGTAGAAAGCCTCTTTTTCGT
GGAAGTTCAGATGTTGATCAACTAGGAAAAATCTTGGACGTAATTGGACTCCCAGGAGAA
GAAGACTGGCCTAGAGATGTTGCCCTTCCCAGGCAGGCTTTTCATTCAAAATCTGCCCAA
CCAATTGAGAAGTTTGTAACAGATATTGATGAACTAGGCAAAGACCTACTTCTGAAGTGT
TTGACATTTAACCCAGCCAAAAGAATATCTGCCTACAGTGCCCTGTCTCACCCATACTTC
CAGGACCTGGAAAGGTGCAAAGAAAACCTGGATTCCCACCTGCCGCCCAGCCAGAACACC
TCGGAGCTGAATACAGCC
>olive baboon ENSPANT00000026400.1
ATGGAGAAGGACGGCCTGTGCCGCGCTGACCAGCAGTACGAATGCGTGGCGGAGATCGGG
GAGGGCGCCTATGGGAAGGTGTTCAAGGCCCGCGACTTGAAGAACGGAGGCCGTTTCGTG
GCGTTGAAGCGCGTGCGGGTGCAGACGGGCGAGGAGGGCATGCCGCTTTCCACCATCCGC
GAGGTGGCGGTGCTGAGGCACCTGGAGACCTTCGAGCACCCCAACGTGGTCAGGTTGTTT
GATGTGTGCACAGTGTCACGAACAGACAGAGAAACCAAACTAACTTTAGTGTTTGAACAT
GTCGATCAAGACTTGACCACTTACTTGGATAAAGTTCCAGAGCCTGGAGTGCCCACTGAA
ACCATAAAGGATATGATGTTTCAGCTTCTCCGAGGTCTGGACTTTCTTCATTCACACCGA
GTAGTGCATCGCGATCTAAAACCACAGAACATTCTGGTGACCAGCAGCGGACAAATAAAA
CTCGCTGACTTCGGCCTTGCCCGCATCTATAGTTTCCAGATGGCTCTAACCTCAGTGGTC
GTCACGCTGTGGTACAGAGCTCCCGAAGTCTTGCTCCAGTCCAGCTACGCCACCCCCGTG
GATCTTTGGAGTGTTGGCTGCATATTTGCAGAAATGTTTCGTAGAAAGCCTCTTTTTCGT
GGAAGTTCAGATGTCGATCAACTAGGAAAAATCTTGGACGTAATTGGACTCCCAGGAGAA
GAAGACTGGCCTAGAGATGTTGCCCTTCCCAGGCAGGCTTTTCATTCAAAATCTGCCCAA
CCAATTGAGAAGTTTGTAACAGATATCGATGAACTAGGCAAAGACCTACTTCTGAAGTGT
TTGACATTTAACCCAGCCAAAAGAATATCAGCCTACAGTGCCCTGTCTCACCCATACTTC
CAGGACCTGGAGAGGTGCAAAGAAAACCTGGATTCCCACCTGCCGCCCAGCCAGAACACC
TCGGAGCTGAATACAGCC
>orangutan ENSPPYT00000020758.1
ATGGAGAAGGACGGCCTGTGCCGCGCTGACCAGCAGTACGAATGCGTGGCGGAGATCGGG
GAGGGCGCCTATGGAAAGGTGTTCAAGGCCCGCGACTTGAAGAACGGAGGCCGTTTCGTG
GCGTTGAAGCGTGTGCGGGTGCAGACGGGCGAGGAGGGCATGCCGCTCTCCACCATCCGC
GAGGTGGCGGTGCTGAGGCACCTGGAGACCTTCGAGCACCCCAACGTGGTCAGGTTGTTT
GATGTGTGCACAGTGTCACGAACAGACAGAGAAACCAAACTAACTTTAGTGTTTGAACAT
GTCGATCAAGACTTGACCACTTACTTGGATAAAGTTCCAGAGCCTGGAGTGCCCACTGAA
ACCATAAAGGATATGATGTTTCAGCTTCTCCGAGGTCTGGACTTTCTTCATTCACACCGA
GTAGTGCATCGCGATCTAAAACCACAGAACATTCTGGTGACCAGCAGCGGACAAATAAAA
CTCGCTGACTTCGGCCTTGCCCGCATCTATAGTTTCCAGATGGCTCTAACCTCAGTGGTC
GTCACGCTGTGGTACAGAGCACCCGAAGTCTTGCTCCAGTCCAGCTACGCCACCCCCGTG
GATCTCTGGAGTGTTGGCTGCATATTTGCAGAAATGTTTCGTAGAAAGCCTCTTTTTCGT
GGAAGTTCAGATGTTGATCAACTAGGAAAAATCTTGGACGTAATTGGACTCCCAGGAGAA
GAAGACTGGCCTAGAGATGTTGCCCTTCCCAGGCAGGCTTTTCATTCAAAATCTGCCCAA
CCAATTGAGAAGTTTGTAACAGATATCGATGAACTAGGCAAAGACCTACTTCTGAAGTGT
TTGACATTTAACCCAGCCAAAAGAATATCTGCCTACAGTGCCCTGTCTCACCCATACTTC
CAGGACCTGGAAAGGTGCAAAGAAAACCTGGATTCCCACCTGCCGCCCAGCCAGAACACC
TCGGAGCTGAATACAGCC

>Cebus capucinus imitator XM_017536420.1
ATGGAGAAGGACGGCCTGAGCCGCGCGGACCAGCAGTACGAATGCGTGGCAGAGATCGGGGAGGGCGC

CTATGGGAAGGTGTTCAAGGCCCGAGACTTGAAGAACGGAGGCCGTTTCGTGGCATTGAAGCGCGTGCGG
GTGCAGACCGGCGAGGAGGGCATGCCGCTCTCCACCATCCGCGAGGTGGCGGTGCTGAGGCACCTGGAGA
CCTTCGAGCACCCCAACGTGGTCAGGTTGTTTGATGTGTGCACAGTGTCACGAACTGACAGAGAAACCAA
ACTAACTTTAGTGTTTGAACATGTTGATCAAGACTTGACCACTTACTTGGATAAAGTTCCAGAGCCTGGA
GTGCCCACTGAAACCATAAAGGATATGATGTTTCAGCTTCTCCGAGGTCTGGACTTTCTTCATTCACACC
GAGTAGTGCATCGCGATCTAAAACCACAAAACATTCTGGTGACCAGCAACGGACAAATAAAACTCGCTGA
CTTTGGCCTTGCCCGCATCTACAGTTTCCAGATGGCTCTAACTTCAGTGGTCGTCACGCTGTGGTACAGA
GCGCCCGAAGTCTTGCTCCAGTCCAGCTACGCCACCCCCGTGGATCTCTGGAGTGTTGGCTGCATATTTG
CAGAAATGTTTCGTAGAAAGCCTCTTTTTCGTGGAAGTTCAGATGTCGATCAACTAGGAAAAATCTTGGA
CGTAATTGGACTCCCAGGAGAAGAAGACTGGCCTAGAGATGTTGCCCTTCCCAGGCAGGCTTTTCATGCA
AAATCTGCCCAACCAATTGAGAAGTTTGTGACAGATATCGATGAACTAGGCAAAGACCTACTTCTGAAGT
GTTTGACATTTAACCCAGCCAAAAGAATATCTGCCTACAGCGCCCTGTCTCACCCATACTTCCAGGACCT
GGAGAGGTGCAGAGAAAACCTGGATTCCCACCTGCCTCCCAGCCAGAACACCTCGGAGATGAATACAGCC

>Aotus nancymaae XM_012450814.1
ATGGAGAAGGACGGCCTGAGCCGCGCGGACCAGCAGTA
CGAATGCGTGGCGGAGATCGGGGAGGGCGCCTATGGGAAGGTGTTCAAGGCCCGAGACTTGAAGAACGGA
GGCCGTTTCGTGGCGTTGAAGCGCGTGCGGGTGCAGACCGGCGAGGAGGGCATGCCGCTCTCCACCATCC
GCGAGGTGGCGGTGCTGAGGCACCTGGAGACCTTCGAGCACCCCAACGTGGTCAGGTTGTTTGATGTGTG
CACAGTGTCACGAACTGACAGAGAAACCAAACTAACTTTAGTGTTTGAACATGTCGATCAAGACTTGACC
ACTTACTTGGATAAAGTTCCAGAGCCTGGAGTGCCCACTGAAACCATAAAGGATATGATGTTTCAGCTTC
TCCGAGGTCTGGACTTTCTTCATTCACACCGAGTAGTGCATCGCGATCTAAAACCACAAAACATTCTGGT
GACCAGCAACGGACAAATAAAACTCGCTGACTTCGGCCTTGCCCGTATCTACAGTTTCCAGATGGCTCTA
ACTTCAGTGGTCGTCACGCTGTGGTACAGAGCGCCCGAAGTCTTGCTCCAGTCCAGCTACGCCACCCCCG
TGGATCTCTGGAGTGTTGGCTGCATATTTGCAGAAATGTTTCGTAGAAAGCCTCTTTTTCGTGGAAGTTC
AGATGTCGATCAACTAGGAAAAATCTTGGACGTAATTGGACTCCCAGGAGAAGAAGACTGGCCTAGAGAT
GTTGCCCTTCCCAGGCAGGCTTTTCATGCAAAATCTGCCCAACCAATTGAGAAGTTTGTGACAGATATCG
ATGAACTAGGCAAAGACCTACTTCTGAAGTGTTTGACATTTAACCCAGCCAAAAGAATATCTGCCTACAG
CGCCCTGTCTCACCCATACTTCCAGGACCTGGAGAGGTGCAGAGAAAACCTGGATACCCACCTGCCTCCC
AGCCAGAACACCTCAGAGATGAATACAGCC
>Saimiri boliviensis boliviensis XM_010345323.1
ATGGAGAAGGACGGCCTGAGCCGCGCGGACCAGCAGTACGAATGCGTGGCGGAGATCGGGGAGGGCG
CCTATGGAAAGGTGTTCAAGGCCCGAGACTTGAAGAACGGAGGCCGTTTCGTGGCGTTGAAGCGCGTGCG
GGTGCAGACCGGCGAGGAGGGCATGCCTCTCTCCACCATCCGCGAGGTGGCAGTGCTGAGGCACCTGGAG
ACCTTCGAGCACCCCAACGTGGTCAGGTTGTTTGATGTGTGCACAGTGTCACGAACTGACAGAGAAACCA
AACTAACTTTAGTGTTTGAACATGTCGATCAAGACTTGACCACTTACTTGGATAAAGTTCCAGAGCCTGG
AGTGCCCACTGAAACTATAAAGGATATGATGTTTCAGCTTCTCCGAGGTCTGGACTTTCTTCATTCACAC
CGAGTAGTGCATCGTGATCTAAAACCACAAAACATTCTGGTGACCAGCAACGGACAAATAAAACTCGCTG
ACTTTGGCCTTGCCCGCATCTACAGTTTCCAGATGGCTCTAACTTCAGTGGTCGTCACGCTGTGGTACAG
AGCGCCCGAAGTCTTGCTCCAGTCCAGCTACGCCACCCCCGTGGATCTCTGGAGTGTTGGCTGCATATTT
GCAGAAATGTTTCGTAGAAAGCCTCTTTTTCGTGGAAGTTCAGATGTCGATCAACTAGGAAAAATCTTGG
ACGTAATTGGACTCCCAGGAGAAGAAGACTGGCCTAGAGATGTTGCCCTTCCCAGGCAGGCTTTTCATGC
AAAATCTGCCCAACCAATTGAGAAGTTTGTGACAGATATCGATGAACTAGGCAAAGACCTACTTCTGAAG
TGTTTGACATTTAACCCAGCCAAAAGAATATCTGCCTACAGCGCCCTGTCTCACCCATACTTCCAGGACC
TGGAGAGGTGCAGAGAAAGCCTGGATTCCCACCTGCCTCCCAGCCAGAACACCTCGGAGATGAATACAGC
C
>Pan paniscus XM_003809731.3

ATGGAGAAGGACGGCCTGTGCCGCGCTGACCAGCAGTACGAATGCGTGGCGGAGATCGG
GGAGGGCGCCTATGGGAAGGTGTTCAAGGCCCGCGACTTGAAGAACGGAGGCCGTTTCGTGGCGTTGAAG
CGCGTGCGGGTGCAGACCGGCGAGGAGGGCATGCCGCTCTCCACCATCCGCGAGGTGGCGGTGCTGAGGC
ACCTGGAGACCTTCGAGCACCCCAACGTGGTCAGGTTGTTTGATGTGTGCACAGTGTCACGAACAGACAG
AGAAACCAAACTAACTTTAGTGTTTGAACATGTCGATCAAGACTTGACCACTTACTTGGATAAAGTTCCA
GAGCCTGGAGTGCCCACTGAAACCATAAAGGATATGATGTTTCAGCTTCTCCGAGGTCTGGACTTTCTTC
ATTCACACCGAGTAGTGCATCGCGATCTAAAACCACAGAACATTCTGGTGACCAGCAGCGGACAAATAAA
ACTCGCTGACTTCGGCCTTGCCCGCATCTATAGTTTCCAGATGGCTCTAACCTCAGTGGTCGTCACGCTG
TGGTACAGAGCACCCGAAGTCTTGCTCCAGTCCAGCTACGCCACCCCCGTGGATCTCTGGAGTGTTGGCT
GCATATTTGCAGAAATGTTTCGTAGAAAGCCTCTTTTTCGTGGAAGTTCAGATGTTGATCAACTAGGAAA
AATCTTGGACGTAATTGGACTCCCAGGAGAAGAAGACTGGCCTAGAGATGTTGCCCTTCCCAGGCAGGCT
TTTCATTCAAAATCTGCCCAACCAATTGAGAAGTTTGTAACAGATATTGATGAACTAGGCAAAGACCTAC
TTCTGAAGTGTTTGACATTTAACCCAGCCAAAAGAATATCTGCCTACAGTGCCCTGTCTCACCCATACTT
CCAGGACCTGGAAAGGTGCAAAGAAAACCTGGATTCCCACCTGCCGCCCAGCCAGAACACCTCGGAGCTG
AATACAGCC
>Cercocebus atys XM_012032059.1
ATGGAGAAGGACGGCCTGTGCCGCGCTGACCAGCAGTACGAATGCGTGGCGGAGATCGGGGAGGGCGCC
TATGGGAAGGTGTTCAAGGCCCGCGACTTGAAGAACGGAGGCCGTTTCGTGGCGTTGAAGCGCGTGCGGG
TGCAGACGGGCGAGGAGGGCATGCCGCTTTCCACCATCCGCGAGGTGGCGGTGCTGAGGCACCTGGAGAC
CTTCGAGCACCCCAACGTGGTCAGGTTGTTTGATGTGTGCACAGTGTCACGAACAGACAGAGAAACCAAA
CTAACTTTAGTGTTTGAACATGTCGATCAAGACTTGACCACTTACTTGGATAAAGTTCCAGAGCCTGGAG
TGCCCACTGAAACCATAAAGGATATGATGTTTCAGCTTCTCCGAGGTCTGGACTTTCTTCATTCACACCG
AGTAGTGCATCGCGATCTAAAACCACAGAACATTCTGGTGACCAGCAGTGGACAAATAAAACTCGCTGAC
TTCGGCCTTGCCCGCATCTATAGTTTCCAGATGGCTCTAACCTCAGTGGTCGTCACGCTGTGGTACAGAG
CTCCCGAAGTCTTGCTCCAGTCCAGCTACGCCACCCCCGTGGATCTTTGGAGTGTTGGCTGCATATTTGC
AGAAATGTTTCGTAGAAAGCCTCTTTTTCGTGGAAGTTCAGATGTCGATCAACTAGGAAAAATCTTGGAC
GTAATTGGACTCCCAGGAGAAGAAGACTGGCCTAGAGATGTTGCCCTTCCCAGGCAGGCTTTTCATTCAA
AATCTGCCCAACCAATTGAGAAGTTTGTAACAGATATCGATGAACTAGGCAAAGACCTACTTCTGAAGTG
TTTGACATTTAACCCAGCCAAAAGAATATCAGCCTACAGTGCCCTGTCTCACCCATACTTCCAGGACCTG
GAGAGGTGCAAAGAAAACCTGGATTCCCACCTGCCGCCCAGCCAGAACACCTCGGAGCTGAATACAGCC
>Mandrillus leucophaeus XM_011998272.1
ATGGAGA
AGGACGGCCTGTGCCGCGCTGACCAGCAGTACGAATGCGTGGCGGAGATCGGGGAGGGCGCCTATGGGAA
GGTGTTCAAGGCCCGCGACTTGAAGAACGGAGGCCGTTTCGTGGCGTTGAAGCGCGTGCGGGTGCAGACG
GGCGAGGAGGGCATGCCGCTTTCCACCATCCGCGAGGTGGCGGTGCTGAGGCACCTGGAGACCTTCGAGC
ACCCCAACGTGGTCAGGTTGTTTGATGTGTGCACAGTGTCACGAACAGACAGAGAAACCAAACTAACTTT
AGTGTTTGAACATGTCGATCAAGACTTGACCACTTACTTGGATAAAGTTCCAGAGCCTGGAGTGCCCACT
GAAACCATAAAGGATATGATGTTTCAGCTTCTCCGAGGTCTGGACTTTCTTCATTCACACCGAGTAGTGC
ATCGCGATCTAAAACCACAGAACATTCTGGTGACCAGCAGCGGACAAATAAAACTCGCTGACTTCGGCCT
TGCCCGCATCTATAGTTTCCAGATGGCTCTAACCTCAGTGGTCGTCACGCTGTGGTACAGAGCTCCCGAA
GTCTTGCTCCAGTCCAGCTACGCCACCCCCGTGGATCTTTGGAGTGTTGGCTGCATATTTGCAGAAATGT
TTCGTAGAAAGCCTCTTTTTCGTGGAAGTTCAGATGTCGATCAACTAGGAAAAATCTTGGACGTAATTGG
ACTCCCAGGAGAAGAAGACTGGCCTAGAGATGTTGCCCTTCCCAGGCAGGCTTTTCATTCAAAATCTGCC
CAACCGATTGAGAAGTTTGTAACAGATATCGATGAACTAGGCAAAGACCTACTTCTGAAGTGTTTGACAT
TTAACCCAGCCAAAAGAATATCAGCCTACAGTGCCCTGTCTCACCCATACTTCCAGGACCTGGAGAGGTG
CAAAGAAAACCTGGATTCCCACCTGCCACCCAGCCAGAACACCTCAGAGCTGAATACAGCC

>Rhinopithecus roxellana XM_010389487.1
ATGGAGAAGGACGGCCTGTGCC
GCGCTGACCAGCAGTACGAATGCGTGGCGGAGATCGGGGAGGGCGCCTATGGGAAGGTGTTCAAGGCCCG
CGACTTGAAGAACGGAGGCCGTTTCGTGGCGTTGAAGCGCGTGCGGGTGCAGACGGGCGAGGAGGGCATG
CCGCTCTCTACCATCCGCGAGGTGGCGGTGCTGAGGCACCTGGAGACCTTCGAGCACCCCAACGTGGTCA
GGTTGTTTGATGTGTGCACAGTGTCACGAACAGACAGAGAAACCAAACTAACTTTAGTGTTTGAACATGT
TGATCAAGACTTGACCACTTACTTGGATAAAGTTCCAGAGCCTGGAGTGCCCACTGAAACCATAAAGGAT
ATGATGTTTCAGCTTCTCCGAGGTCTGGACTTTCTTCATTCACACCGAGTAGTGCATCGCGATCTAAAAC
CACAGAACATTCTGGTGACCAGCAGCGGACAAATAAAACTCGCTGACTTCGGCCTTGCCCGCATCTATAG
TTTCCAGATGGCTCTTACCTCAGTGGTCGTCACACTGTGGTACAGAGCTCCCGAAGTCTTGCTCCAGTCC
AGCTACGCCACCCCCGTGGATCTATGGAGTGTTGGCTGCATATTTGCAGAAATGTTTCGTAGAAAGCCTC
TTTTTCGTGGAAGTTCAGATGTCGATCAACTAGGAAAAATCTTGGACGTAATTGGACTCCCAGGAGAAGA
AGACTGGCCTAGAGATGTTGCCCTTCCCAGGCAGGCTTTTCATTCAAAATCTGCCCAACCAATTGAGAAG
TTTGTAACAGATATCGATGAACTAGGCAAAGATCTACTTCTGAAGTGTTTGACATTTAACCCAGCCAAAA
GAATATCAGCCTACAGTGCCCTGTCTCACCCATACTTCCAGGACCTGGAGAGGTGCAAAGAAAACCTGGA
TTCCCACCTGCCGCCCAGCCAGAACACCTCGGAGCTGAATACAGCC
>Galeopterus variegatus XM_008572294.1
ATGGAGAAGGACGGCCTGAGCCGCGCCGACCAGCAG
TACGAATGCGTGGCGGAGATCGGAGAGGGCGCCTATGGGAAGGTGTTCAAGGCCCGCGACCTGAAGAACG
GAGGCCGTTTCGTGGCGCTGAAGCGCGTGCGGGTGCAGACGGGCGAGGAGGGCATGCCGCTCTCCACCAT
CCGCGAGGTGGCGGTGCTGAGGCACCTGGAGACCTTCGAGCACCCCAACGTGGTCAGATTGTTTGATGTG
TGCACCGTGTCACGAACAGACAGAGAAACCAAACTAACATTGGTATTTGAACATGTTGATCAAGACTTGA
CCACTTACTTGGATAAAGTTCCAGAGCCTGGAGTGCCTACTGAAACCATAAAGGATATGATGTTCCAGCT
TCTCCGAGGTCTGGACTTTCTTCATTCTCACCGAGTAGTGCATCGCGATCTAAAACCACAGAATATTCTG
GTGACCAGCAGTGGACAAATCAAACTGGCTGACTTCGGCCTTGCCCGCATCTACAGCTTTCAGATGGCTC
TTACCTCAGTGGTCGTCACGCTGTGGTACAGAGCTCCAGAGGTCTTGCTCCAGTCCAGCTACGCCACCCC
CGTGGATCTCTGGAGTGTTGGCTGCATATTTGCAGAAATGTTTCGTAGAAAGCCTCTTTTTCGTGGAAGT
TCAGATGTCGATCAACTAGGAAAAATCTTGGACGTAATTGGACTCCCAGGAGAAGAGGACTGGCCTAGAG
ATGTTGCCCTTCCCAGGCAGGCTTTTCATTCAAAATCTGCCCAACCAATTGAGAAGTTTGTAACAGATAT
TGATGAACTAGGCAAAGACCTACTTCTGAAGTGCTTGACATTTAATCCAGCCAAAAGAATATCTGCCTAC
AGCGCCCTGTCTCACCCGTATTTCCAGGACCTGGAGAGGTGCAAAGAGAACCTGGATTCCCACCTGCCGC
CCAGCCAGAACACCTCGGAGCTGAACACAGCC
>cavia porcellus ENSCPOT00000004865.2
ATGGAGAAGGACGGCCTGAGCCGCGCCGACCAGCAGTATGAGTGCGTGGCCGAGATCGGG
GAGGGCGCCTATGGGAAAGTGTTCAAGGCCCGTGACCTGAAGAACGGAGGCCGATTCGTG
GCACTGAAGCGCGTGCGGGTGCAGACCGGCGAGGAGGGCATGCCGCTCTCCACCATCCGC
GAGGTGGCGGTGCTGAGGCACCTGGAGACCTTCGAGCACCCCAACGTGGTCAGGTTGTTT
GATGTGTGCACAGTGTCTCGGACAGACAGAGAAACCAAACTGACACTGGTATTTGAACAT
GTTGATCAAGACCTGACCACTTACTTGGACAAGGTTCCTGAACCTGGAGTGCCCACTGAA
ACCATAAAGGATATGATGTTTCAGCTTCTTCGAGGTTTGGACTTCCTTCATTCTCACCGA
GTGGTACATCGTGATCTAAAACCACAGAACATTCTGGTGACCAGCAATGGACAAATAAAG
CTGGCTGACTTTGGCCTTGCCCGCATCTATAGTTTTCAAATGGCTCTTACTTCAGTGGTT
GTCACACTGTGGTACCGCGCTCCTGAAGTCTTGCTCCAGTCCAGCTACGCCACCCCTGTG
GACCTCTGGAGTGTTGGCTGCATATTTGCAGAAATGTTTCGTAGAAAGCCTCTTTTTCGT
GGAAGTTCAGATGTGGATCAACTAGGAAAAATCTTGGATGTAATTGGACTCCCAGGAGAA
GAAGACTGGCCTAGAGATGTGGCCCTGCCCAGGCAGGCTTTTCATTCCAAACCTGCCCAA
CCCATTGAGAAGTTTGTAACAGATATTGATGAGCTAGGCAAAGACCTACTTCTGAAGTGC

TTGACATTTAATCCAGCCAAGAGAATATCTGCCTACAGTGCCCTGTCTCACCCATACTTC
CAAGACCTGGAGAGGTGCAAGGAGAACCTGGATTCCCACCTGCCTCCCAGCCAGAACGCC
TCAGAGCTGAACACAGCC
>Ictidomys tridecemlineatus XM_005340537.2
ATGGAGAAGGACGGCCTGAGCCGTGCGGACCAGCAGTACGAGTGCGTGGCGGAGATCGGGGAGG
GCGCCTATGGGAAGGTGTTCAAAGCCCGCGACCTGAAGAACGGAGGCCGGTTCGTGGCACTGAAGCGCGT
GCGGGTGCAGACGGGCGAGGAGGGCATGCCGCTCTCCACCATCCGCGAGGTGGCGGTGCTGAGGCACCTG
GAGACCTTCGAGCACCCCAACGTGGTCAGGCTGTTTGATGTGTGCACAGTGTCACGAACAGACAGAGAAA
CCAAACTAACACTAGTGTTTGAACACGTTGATCAAGACTTGACCACTTATCTGGATAAAGTTCCGGAGCC
TGGAGTGCCCACAGAAACCATAAAGGATATGATGTTTCAGCTTCTCCAAGGTCTGGACTTTCTTCACTCT
CACCGAGTAGTGCACCGTGATCTAAAACCACAGAATATTCTGGTGACCAGCAGTGGACAAATAAAACTGG
CTGACTTTGGCCTTGCCCGCATTTATAGTTTTCAGATGGCCCTTACCTCAGTGGTCGTCACGCTGTGGTA
CCGAGCTCCGGAGGTCTTGCTCCAGTCCAGCTATGCCACCCCCGTGGATCTCTGGAGTGTTGGCTGCATA
TTTGCAGAAATGTTTCGTAGAAAGCCTCTTTTTCGTGGAAGTTCTGATGTCGATCAACTAGGAAAAATCT
TGGATGTAATTGGACTCCCAGGAGAAGAGGACTGGCCCAGAGACGTTGCCCTTCCCAGGCAGGCTTTTCA
TTCCAAATCTGCCCAACCCATTGAGAAGTTTGTATCAGATATCGACGAACTTGGCAAAGACCTGCTTCTG
AAGTGCCTGACATTTAATCCAGCCAAAAGAATATCTGCCTACAGCGCCCTGTCTCACCCATACTTCCAAG
ACCTGGAGAGGTGCAAGGAGAACCTGGATTCCCACCTGCCGCCCAGCCAGAACCCCTCAGAGCTGAATAC
AGCC
>mouse ENSMUST00000042410.4
ATGGAGAAGGACAGCCTGAGTCGCGCCGATCAGCAGTATGAGTGCGTGGCGGAGATCGGC
GAAGGCGCCTATGGGAAGGTGTTCAAGGCCCGCGACCTGAAGAACGGCGGCCGCTTCGTG
GCTCTGAAGCGCGTGCGAGTGCAGACCAGTGAGGAGGGCATGCCGCTCTCCACCATCCGC
GAGGTGGCGGTGCTGAGGCACCTGGAGACCTTCGAGCACCCCAACGTGGTCAGGTTGTTT
GATGTGTGCACAGTGTCACGGACGGACAGAGAAACCAAGCTTACACTAGTGTTTGAGCAT
GTTGATCAAGACTTGACCACTTACTTGGATAAAGTTCCAGAGCCCGGCGTACCCACAGAA
ACCATAAAGGATATGATGTTTCAGCTTCTCCGAGGTCTGGACTTTCTTCATTCTCACAGA
GTAGTGCATCGTGATCTGAAACCGCAGAACATTCTGGTGACCAGCAGTGGACAGATAAAG
CTGGCTGACTTTGGCCTTGCCCGCATCTATAGTTTTCAGATGGCCCTTACCTCGGTGGTC
GTCACGCTGTGGTACCGAGCCCCAGAAGTCCTGCTCCAGTCCAGCTATGCCACCCCTGTG
GACCTCTGGAGTGTCGGTTGCATCTTTGCAGAAATGTTTCGCAGAAAGCCTCTTTTTCGT
GGAAGTTCAGACGTGGATCAACTAGGAAAAATCTTGGACATCATTGGACTCCCAGGAGAG
GAAGACTGGCCTAGGGACGTGGCCCTTCCCCGGCAGGCTTTTCATTCCAAATCTGCTCAA
CCCATCGAGAAGTTTGTGACAGATATTGACGAACTAGGCAAAGACCTACTTCTGAAATGC
CTGACGTTTAATCCAGCTAAAAGGATATCCGCCTACGGCGCCCTGAATCACCCGTACTTC
CAAGATCTGGAGAGATACAAGGACAACCTGAACTCTCACCTGCCATCCAACCAGAGCACC
TCGGAGCTGAACACAGCC
>Oryctolagus cuniculus ENSOCUT00000011075.3
ATGGAGAAGGACGGCCTGAGCCGCGCCGACCAGCAGTACGAGTGCGTGGCGGAGATCGGG
GAGGGCGCCTACGGGAAGGTGTTCAAGGCCCGCGACCTCAAGAACGGAGGCCGGTTCGTG
GCGCTGAAGCGCGTGCGGGTGCAGACGGGCGAGGAAGGCATGCCGCTCTCCACCATCCGC
GAGGTGGCGGTGCTGAGGCACCTGGAGACCTTCGAGCACCCCAACGTGGTCAGGCTGTTC
GATGTATGCACAGTGTCCCGGACAGACAGAGAGACAAAGCTAACACTAGTGTTTGAACAT
GTTGATCAAGACTTGACCACTTACCTGGATAAAGTTCCGGAGCCTGGAGTGCCCACTGAA
ACTATAAAGGATATGATGTTTCAGCTCCTGCGAGGTCTGGACTTTCTTCATTCTCACCGA
GTAGTGCATCGTGATCTAAAACCACAGAACATTCTGGTGACCAGCAGTGGACAAATAAAA
CTGGCTGACTTCGGCCTTGCGCGCATCTACAGTTTTCAGATGGCTCTTACTTCAGTGGTC

GTCACGCTGTGGTACCGAGCTCCAGAAGTCCTGCTGCAGTCCAGCTACGCCACGCCCGTG
GATCTCTGGAGTGTCGGCTGCATATTTGCAGAAATGTTTCGTAGAAAGCCTCTCTTTCGT
GGAAGTTCAGATGTGGATCAGCTAGGAAAAATCTTGGACGTCATTGGACTCCCAGGAGAA
GAGGACTGGCCCAGAGATGTTGCCCTTCCCAGGCAAGCGTTTCATTCCAAATCTGCCCAG
CCGATTGAGAAGTTCGTAACAGATATCGATGAACTAGGCAAAGACCTGCTTCTGAAGTGC
TTGACCTTCAATCCAGCCAAAAGAATATCTGCCTACAGCGCCCTGGCCCACCCGTACTTC
CAAGACCTGGAGAGGGGCAAGGAGAGCTTGGATCCCCACCTACCGCCCAGCCAGAGCGCC
TCAGAGCTGAACACAGCC
>rat ENSRNOT00000012597.6
ATGGAGAAGGACAGCCTGAGTCGCGCCGACCAGCAGTATGAGTGCGTGGCGGAGATCGGG
GAAGGCGCCTACGGGAAGGTGTTCAAGGCCCGCGACCTGAAGAACGGCGGCCGCTTCGTG
GCTCTGAAGCGCGTGCGAGTGCAGACCGGAGAGGAGGGCATGCCGCTCTCCACCATCCGC
GAGGTGGCGGTGCTGAGGCACCTGGAGACCTTTGAGCACCCCAACGTGGTCAGGTTGTTT
GACGTGTGCACAGTGTCACGGACAGACAGAGAAACTAAACTTACGCTAGTGTTTGAGCAT
GTTGATCAAGACTTGACCACTTACTTGGATAAAGTTCCAGAACCCGGTGTGCCCACAGAG
ACCATAAAGGATATGATGTTTCAGCTTCTCCGAGGTCTGGACTTCCTCCATTCTCACAGA
GTAGTGCATCGTGACCTGAAGCCACAGAACATTCTGGTGACCAGCAGTGGACAAATAAAA
CTGGCTGACTTCGGCCTTGCCCGCATCTACAGTTTTCAGATGGCCCTTACCTCGGTGGTC
GTCACGCTGTGGTACCGAGCCCCGGAAGTCCTGCTCCAGTCCAGCTACGCCACCCCCGTG
GACCTCTGGAGTGTTGGCTGCATCTTTGCAGAACTGTTTCGCAGAAAGCCTCTTTTTCGT
GGAAGTTCAGACGTGGATCAACTAGGGAAAATCTTGGACGTCATCGGACTCCCAGGAGAA
GAAGACTGGCCTAGGGATGTTGCTCTTCCCAGACAGGCTTTTCACTCCAAATCTGCCCAA
CCCATCGAGAAGTTTGTGACAGACATCGACGAGCTAGGCAAAGACCTCCTTCTGAAATGC
TTGACGTTTAATCCAGCTAAAAGAATATCCGCTTATGGCGCCCTGAATCACCCGTACTTC
CAAGACCTGGAGAGATACAAGGACAACCTGCATTCTCACCTGTCGTCCAGCCAGAGCACC
TCGGAGCTGAACACAGCC
>Marmota marmota marmota XM_015506732.1
ATGGAGAAGGACGGCCTGAGCCGCGCGGACCA
GCAGTACGAGTGCGTGGCGGAGATCGGGGAGGGCGCCTATGGGAAGGTGTTCAAAGCCCGCGACCTGAAG
AACGGAGGCCGGTTCGTGGCACTGAAGCGCGTGCGGGTGCAGACGGGCGAGGAGGGCATGCCGCTCTCCA
CCATCCGCGAGGTGGCGGTGCTGAGGCACCTGGAGACCTTCGAGCACCCCAACGTGGTCAGGCTGTTTGA
TGTGTGCACAGTGTCACGAACAGACAGAGAAACCAAACTAACACTAGTGTTTGAACACGTTGATCAAGAC
TTGACCACTTATCTGGATAAAGTTCCGGAGCCTGGAGTGCCCACAGAAACCATAAAGGATATGATGTTTC
AGCTTCTCCGAGGTCTGGACTTTCTTCACTCTCACCGAGTAGTGCACCGTGATCTAAAACCACAGAACAT
TCTGGTGACCAGCAGTGGACAAATAAAACTGGCTGACTTTGGCCTTGCCCGCATTTATAGTTTTCAGATG
GCCCTTACCTCAGTGGTCGTCACGCTGTGGTACAGAGCTCCAGAGGTCTTGCTCCAGTCCAGCTACGCCA
CCCCCGTGGATCTCTGGAGTGTTGGCTGCATATTTGCAGAAATGTTTCGTAGAAAGCCTCTTTTTCGTGG
GAGTTCTGATGTCGATCAACTAGGAAAAATCTTGGATGTAATTGGACTCCCAGGAGAAGAGGACTGGCCT
AGAGACGTTGCCCTGCCCAGGCAGGCTTTTCATTCCAAATCTGCCCAACCCATTGAGAAGTTTGTATCAG
ATATCGACGAACTTGGCAAAGACCTGCTTCTGAAGTGCCTGACATTTAATCCAGCCAAAAGAATATCTGC
CTACAGCGCCCTGTCTCACCCATACTTCCAAGACCTGGAGAGGTGCAAGGAGAACCTGAATTCCCACCTG
CCGCCCAGCCAGAACCCCTCAGAGCTGAATACAGCC
>Chinchilla lanigera XM_005388024.2
ATGGAGAAGGACGGCCTGA
GCCGCGCCGACCAGCAGTACGAATGCGTGGCGGAGATCGGGGAGGGCGCTTATGGGAAGGTGTTCAAGGC
CCGTGACCTGAAGAACGGAGGCCGGTTCGTGGCTCTGAAGCGCGTGCGGGTGCAGACCGGCGAGGAGGGC
ATGCCGCTCTCCACTATCCGCGAGGTGGCGGTGCTGAGGCACCTGGAGACCTTCGAGCACCCCAACGTGG

TCAGGTTGTTTGATGTGTGCACAGTGTCTCGGACAGATAGAGAGACAAAACTAACACTAGTGTTCGAACA
TGTTGATCAAGACCTGACCACTTACTTGGACAAAGTTCCCGAGCCTGGAGTGCCCACGGAAACCATAAAG
GATATGATGTTTCAGCTTCTTCGAGGTCTGGACTTCCTTCATTCTCACCGAGTGGTGCATCGTGATCTAA
AACCACAGAACATTCTGGTGACCAGCAGTGGACAAATAAAGCTGGCTGACTTTGGCCTTGCCCGCATCTA
TAGTTTTCAGATGGCTCTTACTTCAGTGGTTGTCACACTGTGGTACCGCGCTCCCGAAGTCTTGCTGCAG
TCCAGCTACGCCACCCCCGTGGATCTCTGGAGTGTCGGCTGCATATTTGCAGAAATGTTTCGTAGAAAGC
CTCTTTTTCGTGGAAGTTCAGATGTGGATCAACTAGGAAAAATCTTGGATGTAATTGGACTCCCAGGAGA
AGAGGACTGGCCTAGAGATGTGGCCCTTCCCAGGCAGGCTTTTCATTCCAAACCTGCCCAGCCCATTGAG
AAGTTTGTAACAGATATTGACGAGCTAGGCAAAGACCTACTTCTGAAGTGCTTGACATTTAATCCAGCCA
AGAGAATATCCGCCTACAGTGCCCTGTCTCACCCGTACTTCCAAGACCTGGAGAGGTGCAAGGAGAACCT
GGATTCCCCCCTGCCACCCAGCCAGAACGCCTCAGAGCTGAACACAGCC
>panda ENSAMET00000019565.1
ATGGAGAAGGACGGCCTGAGCCGCGCGGACCAGCAGTACGAGTGCGTGGCGGAGATCGGG
GAGGGCGCCTATGGGAAGGTGTTCAAGGCCCGGGACCTGAAGAACGGAGGCCGTTTCGTG
GCGCTGAAGCGCGTGCGGGTGCAGACGGGTGAGGAGGGCATGCCGCTCTCTACCATCCGC
GAGGTGGCGGTGCTGAGGCACCTGGAGACCTTCGAGCACCCCAACGTGGTCAGGTTGTTT
GATGTATGCACAGTGTCACGAACAGACAGAGAAACCAAACTAACGTTAGTGTTTGAACAT
GTTGATCAAGACTTGACCACTTATTTGGATAAAGTCCCGGAGCCTGGAGTGCCCACCGAA
ACCATAAAGGATATGATGTTTCAGCTTCTCCGAGGTCTAGACTTTCTTCATTCTCACCGA
GTGGTGCATCGTGATCTCAAACCACAGAACATTCTGGTGACCAGCAGTGGACAAATAAAG
CTGGCTGACTTCGGCCTTGCCCGCATCTACAGTTTTCAGATGGCTCTTACCTCAGTGGTC
GTCACGCTGTGGTACAGAGCTCCAGAAGTCTTGCTTCAGTCCAGCTACGCCACCCCCGTG
GACCTCTGGAGTGTTGGCTGCATATTTGCAGAAATGTTTCGTAGAAAGCCTCTTTTTCGT
GGAAGTTCAGATGTCGATCAGCTTGGAAAAATCTTGGATGTAATTGGACTCCCAGGAGAA
GAGGACTGGCCAAGGGATGTCGCCCTTCCCAGACAGGCTTTTCACTCCAAATCTCCTCAA
CCGATTGAGAAGTTCGTAACAGATATTGACGAGCAAGGCAAGGACCTCCTTCTGAAGTGC
TTAACATTTAATCCAGCCAAAAGAATATCTGCCTACAGTGCTCTGTCTCACCCGTACTTC
CATGACCTGGAGAGGTGCAAAGAGAACGTGGATGCCCATCTGCCGCCCAGCCAGAACAGC
TCGGAGATGAATACAGCC
>cow ENSBTAT00000061349.2
ATGGAGAAGGACGGCCTGAGCCGTGCCGACCAGCAGTATGAGTGCGTGGCGGAGATCGGG
GAGGGCGCCTACGGGAAGGTGTTCAAGGCCCGGGACCTGAAGAACGGAGGCCGTTTCGTG
GCGCTGAAGCGCGTGCGAGTGCAGACTGGCGAGGAGGGCATGCCGCTCTCCACCATCCGC
GAGGTGGCGGTGCTGAGGCACCTGGAGACCTTCGAGCACCCCAACGTGGTCAGGTTGTTT
GACGTGTGTACAGTGTCACGAACAGACAGAGAAACCAAACTAACGTTAGTGTTTGAACAC
GTTGATCAAGACTTGACCACTTATTTGGATAAAGTGCCAGAACCTGGAGTGCCCACTGAA
ACCATAAAGGATATGATGTTTCAGCTTCTCCGAGGCCTGGACTTTCTTCATTCTCACCGA
GTGGTGCATCGTGATCTAAAACCACAGAACATTCTGGTGACCAGCAGTGGACAAATAAAG
CTGGCTGACTTCGGCCTTGCTCGCATCTACAGTTTTCAGATGGCTCTTACCTCAGTGGTC
GTCACGCTGTGGTACCGAGCTCCGGAAGTCCTGCTTCAGTCCAGCTACGCCACCCCTGTG
GATCTCTGGAGCGTTGGCTGCATCTTTGCAGAGATGTTTCGTAGAAAGCCGCTTTTTCGT
GGAAGTTCAGATGTCGACCAACTAGGAAAAATCTTGGACGTAATTGGACTCCCGGGAGAA
GAGGACTGGCCTAGAGATGTTGCCCTACCCAGGCTGGCTTTTCACTCAAAATCCCCTCAA
CCAATTGAGAAGTTTGTAACAGACATTGATGAACAAGGCAAAGACCTGCTTCTGAAGTGC
TTGACATTTAATCCAGCCAAAAGAATATCAGCATACAGTGCCCTGTCTCACCCATACTTC
CACGATCTGGAGAGGCGCAAGGAAAACCTGGATTCCCACCTGCCGCCCAGCCAGAACAGC
TCGGAAATGAATACCGCT

```
>dog ENSCAFT00000003170.3
ATGGAGAAGGACGGCCTGAGCCGCGCGGACCAGCAGTACGAGTGCGTGGCGGAGATCGGG
GAGGGTGCCTACGGGAAGGTGTTCAAGGCCCGGGACCTGAAGAACGGAGGCCGTTTCGTG
GCGCTGAAGCGCGTGCGGGTGCAGACGGGCGAAGAGGGCATGCCGCTCTCCACCATCCCC
GCGAGTGGCGTGCTGAGGCACCTGGAGACCTTCGAGCACCCCAGCGTGGTCAGATTGTTT
GATGTATGCACAGTGTCACGAACAGACAGAGAAACGAAACTAACATTAGTGTTTGAACAT
GTTGATCAAGACTTGACCACTTATTTGGATAAAGTCCCAGAGCCGGGAGTGCCCACTGAA
ACCATAAAGGATATGATGTTTCAGCTTCTCCGAGGCCTGGACTTTCTTCATTCTCACCGA
GTAGTGCATCGCGATTTAAAACCACAGAACATTTTAGTGACCAGCAGTGGACAAATAAAA
CTGGCTGACTTCGGCCTTGCCCGCATCTACAGTTTTCAGATGGCTCTTACTTCAGTGGTC
GTCACACTGTGGTACAGAGCTCCAGAAGTCCTGCTTCAGTCCAGCTATGCCACCCCCGTG
GACCTCTGGAGTGTTGGCTGCATATTTGCAGAAATGTTTCGTAGAAAGCCTCTTTTTCGT
GGAAGTTCAGATGTCGATCAACTTGGAAAAATCTTGGACGTAATTGGACTCCCAGGAGAA
GAGGACTGGCCTAGAGATGTCGCCCTACCCAGGCAGGCTTTCCACTCAAAATCTCCTCAA
CCGATTGAGAAGTTTGTGACAGACATTGATGAACAAGGCAAAGACCTCCTTCTGAAGTGC
TTAACATTTAATCCAGCCAAAAGAATATCTGCCTACAGTGCCCTGTCTCACCCATACTTC
CATGACCTGGAGAGGTGCAAGGAAAACTTGGATTCCCATCTGCCGCCCAGCCAGAACAGC
TCAGAGATGAACACAGCC
>Erinaceus europaeus XM_007522491.2
ATGGAGAAGGACGGTCTGAGAGCCGACCAGCAGTACGAGTGCGTGGCGGAGATCG
GGGAGGGCGCCTATGGGAAGGTGTTCAAGGCCCGGGACCTGAAGAACGGAGGCCGATTCGTGGCGTTGAA
GCGCGTGCGGGTGCAGACGGGCGAGGAAGGCATGCCGCTCTCCACCATCCGCGAGGTGGCGGTGCTGAGG
CACCTGGAAACCTTCGAGCACCCCAACGTGGTCAGGTTGTTTGATGTGTGCACTGTGTCACGAACAGATA
GAGAAACCAAACTAACATTAGTGTTTGAACATGTTGACCAAGACTTGACCACTTACTTGGATAAGGTTCC
AGAACCTGGAGTGCCAAATGAAACTATAAAGGATATGATGTTTCAGCTTCTCCGAGGTTTGGATTTTCTT
CATTCTCACCGAGTAGTGCATCGTGATCTGAAACCACAGAATATTCTAGTAACCAGCAGTGGACAAATAA
AGCTGGCTGATTTCGGCCTCGCCCGTATCTACAGTTTTCAGATGGCTCTTACATCAGTGGTTGTCACACT
TTGGTACAGAGCACCAGAAGTCCTGCTTCAGTCCAGCTACGCCACCCCCGTGGATCTCTGGAGTGTTGGC
TGCATATTTGCAGAAATGTTCCGTAGAAAGCCTCTTTTTCGTGGAAGTTCAGATGTGGATCAACTAGGAA
AAATCTTGGATGTAATTGGACTCCCAGGAGAAGAGGACTGGCCTAGAGATGTTGCTCTTCCCAGACAGGC
TTTTCATTCCAAGTCTCCTCAACCTATTGAGAAGTTTGTAGCAGATATTGATGAACTAGGCAAAGACCTA
CTTCTGAAGTGCTTGACATTTAATCCAGCCAAAAGAATATCAGCCTACAGTGCCCTGTCTCACCCATACT
TCCATGACTTGGAGAGGTTCAAGGAAAACCTGGATTCCCATCTGCCACCCAGCCAGAACAGCTCAGAGAT
GACTACAGCT
>cat ENSFCAT00000002744.3
GACGGCCTGAGCCGCGCGGACCAGCAGTACGAGTGCGTGGCGGAGATCGGGGAGGGCGCC
TATGGAAAGGTGTTCAAGGCCCGGGACCTGAAGAACGGAGGCCGGTTCGTGGCGCTGAAG
CGCGTGCGGGTGCAGACAGGCGAGGAGGGCATGCCGCTCTCCACCATCCGCGAGGTGGCG
GTGCTGAGGCACCTGGAGACCTTCGAGCACCCCAACGTGGTCAGGTTGTTTGATGTATGC
ACAGTGTCACGAACAGACAGAGAAACCAAACTAACATTAGTGTTTGAACATGTTGATCAA
GACCTGACCACTTATTTGGATAAAGTCCCGGAGCCTGGAGTGCCCACTGAAACCATAAAG
GATATGATGTTTCAGCTTCTCCGAGGTCTGGACTTTCTTCATTCTCACCGAGTAGTGCAT
CGTGATCTAAAACCGCAGAACATTCTGGTGACCAGCAGCGGACAAATAAAACTGGCTGAC
TTTGGCCTTGCCCGCATCTACAGTTTTCAGATGGCTCTTACCTCAGTGGTCGTCACGCTG
TGGTACAGAGCTCCAGAAGTCTTGCTTCAGTCCAGCTACGCCACCCCCGTGGACCTCTGG
AGTGTTGGCTGCATATTTGCAGAAATGTTTCGTAGAAAGCCTCTTTTTCGTGGAAGTTCA
GACGTTGATCAACTTGGAAAAATCTTGGATGTAATTGGACTCCCAGGAGAAGAAGACTGG
```

CCCAGAGATGTCGCCCTTCCCAGACAGGCTTTTCACTCAAAATCTCCTCAACCAATTGAG
AAGTTCGTAACAGATATTGATGAACAAGGCAAAGACCTACTCCTGAAGTGCCTAACCTTT
AATCCGGCCAAAAGAATATCTGCCTACAGTGCCCTGTCTCACCCCTACTTCCATGATCTG
GAGAGGTGCAAGGAGAATCTGGATTCCCGTCTGCCTCCCAGCCAGAACAGCTCAGAGATG
AATACAGCC
>elephant ENSLAFT00000018528.2
ATGGAGAAGGACGGCCTGAGTCGCGCGGACCAGCAATACGAGTGCGTGGCGGAGATCGGG
GAGGGCGCCTATGGGAAGGTGTTCAAGGCCCGGGACTTGAAGAACGGAGGCCGTTTCGTG
GCGCTGAAGCGTGTGCGGGTGCAGACTGGCGAAGAGGGCATGCCGCTCTCCACCATCCGC
GAGGTGGCGGTGCTGAGGCACCTGGAGACCTTCGAGCACCCCAACGTGGTCAGGTTGTTT
GATGTATGCACAGTATCACGAACAGACAGAGAAACGAAACTAACATTAGTGTTTGAACAT
GTTGATCAAGACTTGACCACTTACTTGGATAAGGTTCCGGAGCCTGGAGTGCCCACTGAA
ACCATTAAGGATATGATGCTTCAGCTTCTCCGAGGTCTGGACTTTCTTCATTCTCACCGA
GTAGTGCATCGCGATCTAAAACCACAGAACATTCTGGTGACCAGCAGTGGACAAATAAAA
CTGGCTGACTTCGGCCTGGCCCGGATCTACAGTTTTCAGATGGCGCTTACGTCAGTGGTC
GTCACGCTGTGGTACAGAGCTCCAGAAGTCTTGCTTCAGTCCAGCTACGCCACCCCTGTG
GATCTCTGGAGTGTTGGCTGCATATTTGCAGAAATGTTTCGTAGAAAGCCTCTTTTTCGT
GGAAGTTCAGATGTTGATCAACTAGGAAAAATCCTGGATGTAATTGGACTCCCAGGAGAA
GAGGACTGGCCTAGAGATGTTGCTCTTCCCAGGCAGGCTTTCCATTCAAAATCTCCCCAA
CCAATTGAGAAGTTTGTAACAGATATTGATGAACTAGGCAAAGACCTACTTCTGAAGTGT
CTGACATTCAATCCAGCGAAAAGAATATCTGCCTACAGTGCCCTGTCTCACCCATACTTC
CACGACCTGGAGAGGTGCAAGGAGAACCTGGATTCACATCTGCCGCCTGGCCAGAACACC
TCGGAGCTGAACACAGCC

>ferret ENSMPUT00000008398.1
ATGGAGAAGGACGGCCTGAGCCGCGCGGACCAGCAGTACGAGTACGTGGCGGAGATCGGG
GAGGGCGCCTATGGGAAGGTGTTCAAGGCCCGGGACCTGAAGAACGGAGGCCGTTTCGTG
GCGCTGAAGCGCGTGCGGGTGCAGACGGGCGAGGAGGGCATGCCGCTCTCTACGATCCGC
GAGGTGGCGGTGCTGAGGCACCTGGAGACCTTCGAGCACCCCAACGTGGTCAGGTTGTTT
GATGTATGCACAGTGTCACGAACAGACAGAGAAACCAAACTGACATTAGTGTTTGAACAC
GTCGATCAAGACTTGACCACTTACTTGGATAAAGTCCCGGAGCCTGGTGTGCCCACTGAA
ACCATAAAGGACATGATGTTTCAGCTTCTCCGAGGTCTAGATTTTCTTCATTCTCACCGA
GTAGTGCATCGTGACCTAAAACCGCAGAACATTCTGGTGACCAGCAGCGGACAAATAAAG
CTGGCTGACTTCGGCCTTGCCCGCATCTACAGTTTCCAGATGGCTCTTACCTCAGTGGTC
GTCACGCTGTGGTACAGAGCTCCAGAAGTCCTGCTTCAGTCCAGCTACGCCACCCCCGTG
GACCTCTGGAGTGTTGGCTGCATATTTGCAGAAATGTTTCGTAGAAAGCCTCTTTTTCGG
GGAAGTTCGGATGTCGATCAGCTTGGAAAAATCTTGGACGTAATTGGACTCCCAGGAGAA
GAAGACTGGCCTAGAGATGTCGCCCTTCCCAGGCAGGCATTTCACGCAAAAGCTCCTCAG
CCGATTGAGAAGTTTGTAACAGATATTGATGAACAAGGCAAAGACCTACTTCTGAAGTGC
TTAACATTCAATCCAGCCAAAAGAATATCAGCCTACAGTGCCCTGTCTCACCCATACTTC
CATGACCTGGAGAGACGCAAGGAGAACCTGGACGCCCGTCTGCCACCCAACCAGAACAGC
TCAGACATGAACACAGCC
>myotis lucifugus ENSMLUT00000011872.2
ATGGAGAAGGACGGGCTGAGCCGCGCGGACCAGCAGTACGAGTGCGTGGCGGAGATCGGG
GAGGGCGCCTACGGGAAGGTGTTCAAGGCCCGGGACCTGAAGAACGGAGGCCGTTTCGTG
GCGCTGAAGCGCGTGCGGGTGCAGACGGGCGAAGAGGGCATGCCGCTCTCCACCATCCGC
GAGGTGGCGGTGCTGAGGCACCTGGAGACCTTCGAGCACCCCAACGTGGTCAGGTTATTC

GATGTGTGCACGGTGTCACGAACAGACAGAGAAACTAAACTAACATTAGTGTTTGAACAT
GTTGATCAAGACTTGACCACTTATTTGAATAAAGTTCCAGAGCCCGGAGTGCCCACTGAA
ACCATAAAGGATATGATGTTTCAGCTTCTCCGAGGTCTGGACTTTCTTCATTCTCACCGA
GTAGTGCATCGTGATCTAAAACCACAGAACATTCTGGTGACCAGCAGCGGACAAATAAAA
CTGGCTGACTTCGGCCTTGCCCGCATCTACAGTTTTCAGATGGCTCTGACCTCAGTGGTC
GTCACGCTGTGGTACCGGGCCCCCGAAGTCCTGCTGCAGTCCAGCTACGCCACCCCCGTG
GACCTCTGGAGTGTCGGCTGCATATTCGCAGAAATGTTTCGTAGAAAGCCTCTTTTTCGT
GGAAGTTCAGATGTGGATCAACTAGGAAAAATCTTGGACGTAATTGGACTCCCAGGAGAA
GAGGACTGGCCTAGAGATGTGGCCCTTCCCAGGCAGGCTTTTCACTCAAAATCTCCTCAA
CCTATTGAGAACTTTGTAAGCGACATTGATGAACAAGGCAAAGACCTCCTTCTGAAGTGC
TTGACATTTAATCCAGCCAAAAGAATATCTGCCTACGGTGCCCTGTCTCACCCATACTTC
CACGACCTGGAGAGGCGCAAGGAGAGCCTGGATTCCCACCTGCCGCCCAGCCAGAACAGT
TCGGAGATGAATATAGCC
>Pteropus alecto XM_006911896.2
ATGGAGAAGGACAGCCTGAG
CCGCGCGGACCAGCAGTACGAGTGCGTGGCGGAGATCGGGGAGGGCGCCTATGGGAAGGTGTTCAAGGCC
CGGGACCTGAAGAACGGAGGCCGTTTCGTGGCGCTGAAGCGCGTGCGGGTGCAGACGGGCGAGGAGGGCA
TGCCGCTCTCCACCATCCGCGAGGTGGCGGTGCTGAGGCACCTGGAGACTTTCGAGCACCCCAACGTGGT
CAGGTTATTCGATGTGTGTACAGTGTCACGAACAGACAGAGAAACCAAACTAACATTAGTGTTTGAACAT
GTTGATCAAGACTTGACCACTTATTTGGATAAAGTCCCAGAGCCTGGAGTGCCCACTGAAACCATAAAGG
ACATGATGTTTCAGCTTCTCCGAGGTCTGGACTTTCTTCATTCTCACCGAGTCGTGCATCGTGATCTAAA
ACCACAGAACATTCTCGTGACCAGCAGTGGACAAATAAAACTGGCTGACTTCGGCCTTGCCCGCATCTAC
AGTTTTCAGATGGCTCTAACCTCAGTGGTCGTCACGCTGTGGTACCGAGCTCCAGAAGTTTTGCTTCAGT
CCAGCTACGCCACCCCCGTGGACCTCTGGAGCGTTGGCTGCATATTTGCAGAAATGTTTCGTAGAAAGCC
TCTTTTCCGTGGCAGTTCAGATGTCGATCAACTAGGAAAAATCTTGGATGTAATCGGACTCCCAGGAGAG
GAGGACTGGCCTAGAGATGTTGCCCTTCCCAGGCAGGCTTTTCACTCAAAAACTCCTCAACCTATTGAGA
AGTTTGTAGCAGATATTGATGAACAAGGCAAAGACCTACTTCTGAAGTGCTTGACATTTAATCCAGCCAA
AAGAATATCTGCCTACAGTGCCCTGTCTCACCCATACTTCCACGACCTGGAGAGGTGCAAGGAGAACCTG
GATTCCCATCTGCCACCCAGCCAGAACAGCTCGGAGATGAATACAGCC
>Pteropus vampyrus XM_011363661.1
ATGGAGAAGGACAGCCTGAGCCGCGCGGACCAG
CAGTACGAGTGCGTGGCGGAGATCGGGGAGGGCGCCTATGGGAAGGTGTTCAAGGCCCGGGACCTGAAGA
ACGGAGGCCGTTTCGTGGCGCTGAAGCGCGTGCGGGTGCAGACGGGCGAGGAGGGCATGCCGCTCTCCAC
CATCCGCGAGGTGGCGGTGCTGAGGCACCTGGAGACTTTCGAGCACCCCAACGTGGTCAGGTTATTCGAT
GTGTGTACAGTGTCACGAACAGACAGAGAAACCAAACTAACATTAGTGTTTGAACATGTTGATCAAGACT
TGACCACTTATTTGGATAAAGTCCCAGAGCCTGGAGTGCCCACTGAAACCATAAAGGACATGATGTTTCA
GCTTCTCCGAGGTCTGGACTTTCTTCATTCTCACCGAGTCGTGCATCGTGATCTAAAACCACAGAACATT
CTCGTGACCAGCAGTGGACAAATAAAACTGGCTGACTTCGGCCTTGCCCGCATCTACAGTTTTCAGATGG
CTCTAACCTCAGTGGTCGTCACGCTGTGGTACCGAGCTCCAGAAGTTTTGCTTCAGTCCAGCTACGCCAC
CCCCGTGGACCTCTGGAGCGTTGGCTGCATATTTGCAGAAATGTTTCGTAGAAAGCCTCTTTTCCGTGGC
AGTTCAGATGTCGATCAACTAGGAAAAATCTTGGATGTAATCGGACTCCCAGGAGAGGAGGACTGGCCTA
GAGATGTTGCCCTTCCCAGGCAGGCTTTTCACTCAAAAACTCCTCAACCTATTGAGAAGTTTGTAGCAGA
TATTGATGAACAAGGCAAAGACCTACTTCTGAAGTGCTTGACATTTAATCCAGCCAAAAGAATATCTGCC
TACAGTGCCCTGTCTCACCCATACTTCCACGACCTGGAGAGGTGCAAGGAGAACCTGGATTCCCATCTGC
CACCCAGCCAGAACAGCTCGGAGATGAATACGGCC

>ovis aries musimon XM_015107543.1

ATGGAGAAGGACGGCCTGAGCCGTGCTGACCAGCAGTATGAGTGCGTGGCGGAGAT
CGGGGAGGGCGCCTACGGGAAGGTGTTCAAGGCCCGGGACCTGAAGAACGGAGGCCGTTTCGTGGCGCTG
AAGCGTGTGAGAGTGCAGACTGGCGAGGAGGGCATGCCGCTCTCCACCATCCGCGAGGTGGCGGTGCTGA
GGCACCTGGAGACCTTCGAGCACCCCAACGTGGTCAGGTTGTTTGACGTGTGTACAGTGTCACGAACAGA
CAGAGAAACCAAACTAACGTTAGTGTTTGAACACGTTGATCAAGACTTGACCACTTACTTGGATAAAGTG
CCAGAACCTGGAGTGCCCACTGAAACCATCAAGGATATGATGTTTCAGCTTCTCCGAGGCCTGGACTTTC
TTCATTCTCACCGAGTGGTGCATCGTGATCTAAAACCACAGAACATTCTGGTGACCAGCAGTGGACAAAT
AAAGCTGGCTGACTTCGGCCTTGCTCGCATCTACAGTTTTCAGATGGCTCTTACCTCAGTGGTTGTCACG
CTGTGGTACCGAGCACCGGAAGTCCTGCTGCAGTCCAGCTACGCCACCCCTGTGGACCTCTGGAGCGTTG
GCTGCATATTTGCAGAGATGTTTCGTAGAAAGCCTCTTTTTCGTGGAAGTTCAGATGTCGACCAGCTAGG
AAAAATCTTGGACGTGATTGGACTCCCAGGAGAAGAGGACTGGCCTAGAGATGTTGCCCTACCCAGGCAG
GCTTTTCACTCAAAATCTCCTCAACCAATTGAGAAGTTTGTAACAGACATTGATGAACAAGGCAAAGACC
TACTTCTGAAGTGCTTGACGTTTAATCCAGCCAAAAGAATATCAGCATACAGTGCCCTGTCTCACCCATA
CTTCCACGATCTGGAGAGGTGCAAGGAAAACCTGGATTCCCACCTGCCACCCAGCCAGAACAGCTCAGAA
ATGAATACCGCC
>Sus scrofa XM_013979690.1
ATGGAGAAGGACAGCCTGAGCCGTGCCGACCAGCAGTACGAGTGCGTGGCGGAGATCGGGGAGGGCGC
CTATGGGAAGGTGTTCAAGGCCCGGGACCTGAAGAACGGAGGCCGTTTCGTGGCGCTAAAGCGCGTGCGA
GTGCAGACTGGCGAGGAGGGCATGCCGCTCTCCACCATCCGCGAGGTGGCGGTGCTGAGGCACCTGGAGA
CCTTCGAGCACCCCAACGTGGTCAGGTTGTTTGATGTGTGCACAGTGTCACGAACAGACAGAGAAACCAA
ACTAACATTAGTGTTTGAACATGTTGATCAAGACTTGACCACTTATTTGGATAAAGTTCCAGAGCCTGGA
GTGCCCACTGAAACCATAAAGGATATGATGTTTCAGCTTCTCCGAGGTCTGGACTTTCTTCATTCTCACC
GAGTAGTACATCGTGATCTAAAACCACAGAACATTCTGGTGACCAGCAGTGGACAAATAAAGCTGGCTGA
CTTTGGTCTTGCCCGCATCTACAGTTTTCAGATGGCTCTTACCTCAGTGGTCGTCACGCTGTGGTACAGA
GCGCCAGAAGTCTTGCTTCAGTCCAGCTACGCCACCCCTGTGGACCTCTGGAGTGTTGGCTGCATATTTG
CAGAAATGTTTCGTAGAAAGCCGCTTTTTCGTGGAAGTTCAGATGTCGATCAACTAGGAAAAATCTTGGA
CGTGATTGGACTCCCAGGAGAAGAGGACTGGCCTCGAGATGTTGCCCTTCCCAGGCAGGCTTTCCACTCC
AAATCTCCCCAGCCAATTGAGAAGTTTGTAGCAGATATTGATGAACAAGGCAAAGACCTGCTTCTGAAGT
GCTTGACATTTAATCCAGCCAAAAGAATATCAGCCTACAGTGCCCTGTCTCACCCATACTTCCACGACCT
GGAGAGGTGCAAGGAAAACCTGGATTCCCATCTGCCACCCAGCCAGAACAGCTCAGAAATGAATACAGCC
>Odobenus rosmarus divergens XM_004394091.2
ATGGAGAAGGACGGCCTGAGCCGCGCGGACCAGCAGTACGAGTGCGT
GGCGGAGATTGGGGAGGGCGCCTATGGGAAGGTGTTCAAGGCCCGGGACCTGAAGAACGGAGGCCGTTTC
GTGGCACTGAAACGCGTGCGGGTGCAGACGGGCGAGGAGGGCATGCCGCTCTCTACCATCCGCGAGGTGG
CGGTGCTGAGGCACCTGGAGACCTTCGAGCACCCCAACGTGGTCAGGTTGTTTGATGTATGCACAGTGTC
ACGGACAGACAGAGAAACCAAACTAACATTAGTGTTTGAACATGTTGATCAAGACTTGACCACTTACTTG
GATAAAGTCCCGGAGCCTGGAGTGCCCACTGAAACCATAAAGGATATGATGTTTCAGCTTCTCCGAGGTC
TAGACTTCCTTCATTCTCACCGCGTAGTGCATCGTGATCTAAAACCACAGAACATTCTGGTGACCAGCAG
TGGACAAATAAAGCTGGCTGACTTCGGCCTTGCCCGCATCTACAGTTTTCAGATGGCTCTTACCTCAGTG
GTCGTCACGCTGTGGTACAGAGCTCCAGAAGTCTTGCTTCAGTCCAGCTACGCCACCCCTGTGGACCTCT
GGAGTGTTGGCTGCATATTTGCAGAAATGTTTCGTAGAAAGCCTCTTTTTCGTGGAAGTTCAGATGTCGA
TCAGCTTGGAAAAATCTTGGACGTCATCGGACTCCCGGGAGAGGAAGACTGGCCTCGAGATGTCGCCCTT
CCCAGGCAGGCTTTTCACTCAAAATCTCCTCAACCGATTGAGAAGTTTGTAACAGATATCGATGAACAAG
GCAAAGACCTACTTCTGAAGTGCTTAACATTTAATCCAGCCAAAAGGATATCCGCCTACAGTGCCCTGTC
TCACCCATACTTCCATGACCTGGAGAGGTGCAAGGAGAACCTGGATGCCCATCTCCCTCCCAGCCAGAAC
AGCTCAGAGATGAATACAGCC
>Orcinus orca XM_004265601.1

ATGGAGAAGGACGGCCTGAGCCGTGCCGACCAGCAGTACGAA
TGCGTGGCGGAGATCGGGGAGGGCGCCTATGGGAAGGTGTTCAAGGCCCGGGACCTGAAGAACGGAGGCC
GTTTCGTGGCGCTGAAGCGCGTGCGAGTGCAGACCGGCGAGGAGGGCATGCCGCTCTCTACCATCCGCGA
GGTGGCGGTGCTGAGGCACCTGGAGACCTTCGAGCACCCCAACGTGGTCAGGTTGTTTGATGTGTGCACA
GTGTCACGAACAGACAGAGAAACCAAACTAACATTAGTGTTTGAACATGTTGATCAAGACTTGACCACTT
ATTTGGATAAAGTTCCAGAGCCTGGAGTGCCCACTGAAACCATAAAGGATATGATGTTTCAGCTTCTCCG
GGGTCTGGACTTTCTTCATTCTCACCGAGTGGTACATCGTGATCTAAAACCACAGAACATTCTGGTGACC
AGCAGTGGACAAATAAAACTGGCTGACTTCGGCCTTGCCCGCATCTACAGTTTTCAGATGGCTCTTACCT
CAGTGGTCGTCACGCTGTGGTACAGAGCTCCAGAAGTCTTGCTTCAGTCCAGCTACGCCACCCCTGTGGA
CCTCTGGAGTGTTGGTTGCATATTTGCAGAAATGTTTCGTAGAAAGCCGCTTTTTCGTGGAAGTTCAGAT
GTCGATCAGCTAGGAAAAATCTTGGATGTAATTGGACTCCCGGGAGAAGAGGACTGGCCTAGAGATGTTG
CCCTCCCCAGGCAGGCTTTTCACTCAAAATCTCCTCAACCAATTGAGAAGTTTGTAACAGATATTGATGA
ACAAGGCAAAGACCTACTTCTGAAGTGCTTGACATTTAATCCAGCCAAAAGAATATCGGCCTACAGTGCC
CTGTCTCACCCATACTTCCAGGACCTGGAGAGGTGCAAGGAAAACCTGGATGCCCACCTGCCGCCCAGCC
AGAACAGCTCAGAAATGAATACAGCC

>Lipotes vexillifer XM_007461886.1
ATGGAGAAGGACGGCCTGAGCCGTGCCGACCAGCAGTACGAATGCGTGGCGGAGATCGGGGAGGGCGCCT
ATGGGAAGGTGTTCAAGGCCCGGGACCTGAAGAACGGAGGCCGTTTCGTGGCGCTGAAGCGCGTGCGAGT
GCAGACCGGCGAGGAGGGCATGCCGCTTTCTACCATCCGCGAGGTGGCGGTGCTGAGGCACCTGGAGACC
TTCGAGCACCCCAACGTGGTCAGGTTGTTTGATGTGTGCACAGTGTCACGAACAGACAGAGAAACCAAAC
TAACATTAGTGTTTGAACATGTTGATCAAGACTTGACCACTTATTTGGATAAAGTTCCAGAGCCTGGAGT
GCCCACTGAAACCATAAAGGATATGATGTTTCAGCTTCTCCGAGGTCTGGACTTTCTTCATTCTCACCGA
GTGGTACATCGTGATCTAAAACCACAGAACATTCTGGTGACCAGCAGTGGACAAATAAAACTGGCTGACT
TCGGCCTTGCCCGCATCTACAGTTTTCAGATGGCTCTTACCTCAGTGGTCGTCACGCTGTGGTACAGAGC
TCCAGAAGTCTTGCTTCAGTCCAGCTACGCCACCCCTGTGGACCTCTGGAGTGTTGGTTGCATATTTGCA
GAAATGTTTCGTAGAAAGCCGCTTTTTCGTGGAAGTTCAGATGTCGATCAACTAGGAAAAATCTTGGATG
TAATTGGACTCCCGGGAGAAGAGGACTGGCCTAGAGATGTTGCCCTCCCCAGGCAGGCTTTTCACTCAAA
ATCTCCTCAACCAATTGAGAAGTTTGTAACAGATATTGATGAACAAGGCAAAGACCTACTTCTGAAGTGC
TTGACATTTAATCCAGCCAAAAGAATATCGGCCTACAGTGCCCTGTCTCACCCATACTTCCACGACCTGG
AGAGGTGCAAGGAAAACCTGGATGCCCACCTGCCGCCCAGCCAGAACAGCTCAGAAATGAATACAGCC

>Equus caballus XM_001493503.5
ATGGAGAAGGACGGCCTGAGCCGCGCCGACCAGCAGTAC
GAGTGCGTGGCGGAGATCGGGGAGGGCGCCTATGGGAAGGTGTTCAAGGCCCGGGATCTGAAGAACGGAG
GCCGTTTCGTGGCACTGAAGCGCGTGCGGGTGCAGACGGGCGAGGAGGGCATGCCGCTTTCCACCATCCG
CGAGGTGGCGGTGCTGAGGCACCTGGAGACCTTCGAGCACCCCAACGTGGTCAGGTTGTTTGATGTGTGC
ACAGTGTCACGAACAGACAGAGAAACCAAACTAACATTAGTGTTTGAACATGTTGATCAAGACTTGACCA
CTTATTTGGATAAAGTTCCAGAGCCTGGAGTGCCCACTGAAACCATAAAGGATATGATGTTTCAGCTTCT
CCGAGGTCTGGACTTTCTTCATTCTCACCGAGTAGTGCATCGTGATCTAAAACCACAGAACATCCTGGTG
ACCAGCAGTGGACAAATAAAACTGGCTGACTTCGGCCTTGCCCGCATCTATAGTTTTCAGATGGCTCTTA
CCTCAGTGGTCGTCACGCTGTGGTACAGAGCTCCAGAAGTCTTGCTTCAGTCCAGCTACGCCACCCCTGT
GGATCTCTGGAGTGTTGGCTGCATATTTGCAGAAATGTTTCGTAGAAAGCCTCTTTTTCGTGGAAGTTCA
GATGTCGATCAACTAGGAAAAATCTTGGACGTAATTGGACTCCCAGGAGAAGAAGACTGGCCTAGAGATG
TTGCCCTTCCCCGGCAGGCGTTTCACTCAAAATCTCCTCAACCGATTGAGAAGTTTGTAACAGATATTGA
TGAACAAGGCAAAGACCTACTTCTGAAGTGCTTGACATTTAATCCAGCCAAAAGAATATCTGCCTACAGT
GCCCTGTCTCACCCATACTTCCACGACCTGGAGAGGTGCAAGGAGAACCTGGATTCCCATCTGCCGCCCA

GCCAGAACAGCTCGGAGATGAATACAGCC
>Equus asinus XM_014842958.1
ATGGAGAAGGACGGCCTGAGCCGCGCCGACCAGCAGTACG
AGTGCGTGGCGGAGATCGGGGAGGGCGCCTATGGGAAGGTGTTCAAGGCCCGGGACCTGAAGAACGGAGG
CCGTTTCGTGGCACTGAAGCGCGTGCGGGTGCAGACGGGCGAGGAGGGCATGCCGCTTTCCACCATCCGC
GAGGTGGCGGTGCTGAGGCACCTGGAGACCTTCGAGCACCCCAACGTGGTCAGGTTGTTTGATGTGTGCA
CAGTGTCACGAACAGACAGAGAAACCAAACTAACATTAGTGTTTGAACATGTTGATCAAGACTTGACCAC
TTATTTGGATAAAGTTCCAGAGCCTGGAGTGCCCACTGAAACCATAAAGGATATGATGTTTCAGCTTCTC
CGAGGTCTGGACTTTCTTCATTCTCACCGAGTAGTGCATCGTGATCTAAAACCACAGAACATCCTGGTGA
CCAGCAGTGGACAAATAAAACTGGCTGACTTCGGCCTTGCCCGCATCTATAGTTTTCAGATGGCTCTTAC
CTCAGTGGTCGTCACGCTGTGGTACAGAGCTCCAGAAGTCTTGCTTCAGTCCAGCTACGCCACCCCTGTG
GATCTCTGGAGTGTTGGCTGCATATTTGCAGAAATGTTTCGTAGAAAGCCTCTTTTTCGTGGAAGTTCAG
ATGTCGATCAACTAGGAAAAATCTTGGACGTAATTGGACTCCCAGGAGAAGAAGACTGGCCTAGAGATGT
TGCCCTTCCCCGGCAGGCGTTTCACTCAAAATCTCCTCAACCGATTGAGAAGTTTGTAACAGATATTGAT
GAACAAGGCAAAGACCTACTTCTGAAGTGCTTGACATTTAATCCAGCCAAAAGAATATCTGCCTACAGTG
CCCTGTCTCACCCATACTTCCACGACCTGGAGAGGTGCAAGGAGAACCTGGATTCCCATCTGCCGCCCAG
CCAGAACAGCTCGGAGATGAATACAGCC
>Manis javanica XM_017652847.1
ATGGAGAAGGACAGCCTGAGCCGCGCCGACCAGCAGTACGAGTGCGTGGCGGAGATCGGC
GAGGGCGCGTATGGGAAGGTGTTCAAGGCCCGGGACCTGAAGAACGGAGGCCGTTTCGTGGCGCTGAAGC
GCGTGCGGGTGCAGACGGGCGAGGAGGGCATGCCGCTCTCCACCATCCGCGAGGTGGCGGTGCTGAGGCA
CCTGGAGACCTTCGAGCACCCCAACGTGGTCAGGTTGTTTGATGTGTGCACAGTGTCACGAACAGACAGA
GAAATCAAACTAACATTAGTGTTTGAACATGTTGATCAAGACTTGACCACTTACTTGGATAAAGTTCCAG
AACCGGGAGTGCCCACTGAAACCATAAAGGATATGATGTTTCAACTTCTCCGAGGTCTGGACTTTCTTCA
TTCTCACCGGGTAGTGCATCGTGATCTAAAACCACAGAACATTCTGGTGACCAGCAGTGGACAAATAAAA
CTGGCTGACTTCGGCCTTGCCCGCATCTACAGTTTTCAGATGGCTCTTACCTCAGTGGTCGTCACACTGT
GGTACAGAGCTCCAGAAGTCTTGCTTCAGTCCAGCTACGCCACCCCCGTGGATCTCTGGAGTGTTGGCTG
CATATTTGCAGAAATGTTTCGTAGAAAGCCTCTTTTTCGTGGAAGTTCAGATGTTGATCAACTAGGAAAA
ATCCTGGATGTAATTGGACTCCCAGGAGAAGAGGACTGGCCTAGAGATGTGGCCCTTCCCAGGCAGGCTT
TTCACTCAAAATCTCCTCAACCAATTGAGAAGTTTGTAACAGATATTGATGAACAAGGCAGAGACCTACT
TCTGAAGTGCTTGACATTTAATCCAACCAAAAGAATATCTGCCTACAGTGCCCTGTCTCACCCATACTTC
CACGACCTGGAGAGGTTCAAGGAGAACCTGGATTCCCATCTGCCGCCCAGCCAAAACAGCTCTGAGATGA
ATACAGCC

SASS6
>human ENST00000287482.5
ATGAGCCAAGTGCTGTTCCACCAACTAGTCCCGTTGCAGGTGAAATGCAAAGACTGTGAG
GAGAGGAGAGTAAGTATAAGAATGAGCATTGAACTACAATCAGTTTCTAATCCAGTTCAC
AGAAAGGACTTAGTTATTCGTCTGACTGATGACACGGATCCATTTTTTTATATAACCTT
GTTATATCTGAGGAAGATTTTCAAAGTTTAAAATTCCAGCAAGGTCTTCTGGTAGACTTC
TTAGCTTTCCCACAAAAATTTATAGATCTCCTTCAGCAATGTACTCAAGAACATGCCAAA
GAAATTCCAAGGTTTTTGCTACAGTTAGTTTCTCCAGCAGCTATTTTGGATAACTCACCT
GCATTTTTAAATGTGGTAGAGACAAATCCTTTTAAGCATCTTACACACCTCTCACTAAAA
CTTTTACCTGGAAATGATGTGGAGATAAAGAAATTTCTCGCAGGCTGTTTGAAATGTAGC
AAGGAAGAAAAATTATCATTGATGCAATCACTAGATGATGCTACTAAGCAACTGGACTTT
ACACGAAAGACATTAGCAGAAAAAAAACAAGAATTAGATAAGTTACGGAATGAATGGGCG
TCACATACAGCAGCCTTGACAAACAAGCATTCTCAGGAACTGACAAATGAAAAGGAAAAA

GCCTTGCAGGCACAGGTTCAATATCAACAGCAGCATGAACAACAGAAAAAAGATTTAGAA
ATCCTCCATCAACAAAACATCCACCAGCTACAAAACAGACTGTCTGAGTTAGAAGCGGCT
AATAAAGACTTAACCGAAAGAAAATATAAAGGAGACTCCACTATTAGAGAACTTAAAGCA
AAACTTTCTGGTGTTGAAGAGGAGCTACAGCGGACTAAGCAAGAAGTCCTCTCTTTGCGA
AGAGAGAATTCTACACTAGATGTTGAATGCCACGAGAAAGAAAAGCACGTTAATCAGCTA
CAAACAAAAGTGGCAGTTTTAGAACAGGAAATCAAGGATAAGGACCAGCTTGTTTTAAGA
ACAAAAGAGGCATTTGATACAATCCAGGAACAAAAGGTGGTTTTAGAAGAAAATGGTGAG
AAAAATCAAGTACAACTAGGAAAGCTTGAAGCTACAATAAAATCATTATCTGCAGAACTT
CTGAAGGCAAATGAAATTATCAAGAAGTTACAAGGGGATCTGAAAACTTTAATGGGTAAG
TTGAAATTGAAGAATACAGTTACTATTCAGCAAGAAAAACTCTTGGCTGAGAAGGAGGAA
AAATTACAAAAGGAACAAAAGGAATTACAAGATGTTGGACAGTCTCTTCGAATTAAAGAG
CAAGAGGTATGCAAATTACAAGAACAATTAGAAGCTACAGTTAAAAAACTTGAAGAAAGC
AAACAACTTCTAAAAAATAATGAAAAGTTAATCACGTGGTTAAATAAAGAACTAAATGAA
AATCAGCTAGTGAGAAAGCAAGATGTATTGGGACCTTCTACTACTCCGCCTGCACATTCC
AGCAGCAACACAATCAGAAGTGGAATTTCTCCTAACCTGAATGTGGTTGATGGTAGACTG
ACTTACCCAACCTGTGGGATTGGTTATCCTGTCTCCTCTGCATTTGCATTCCAGAATACC
TTCCCTCATTCGATATCTGCCAAAAATACCAGCCACCCTGGTTCAGGAACAAAGGTTCAG
TTTAATTTGCAGTTTACAAAACCAAATGCATCACTAGGAGATGTTCAGTCAGGAGCAACT
ATTAGTATGCCTTGCTCAACTGATAAGGAAAATGGTGAAAATGTAGGGTTGGAATCCAAA
TACCTGAAGAAAAGGGAAGATAGCATTCCTTTACGCGGACTCAGCCAGAACCTATTTAGT
AATTCAGACCATCAGAGAGATGGCACTTTAGGAGCATTACATACATCTTCCAAACCCACA
GCGCTCCCCTCTGCGTCTTCAGCCTATTTCCCTGGGCAGTTACCAAACAGT
>Callithrix jacchus XM_002751130.3
ATGAGCCAAGTTCTG
TTCCACCAACTAGTCCCTTTGCAGGTGAAATGCAAAGACTGCGAGGAGAGGAGAGTAAGTATAAGAATGA
CCATTGAACTACAATCAGTTTCTAATCCAGTTCACAGAAAGGACTTAGTTATTCGTCTGACTGATGACAC
GGATCCATTTTTTCTATATAACCTTGTTATATCTGAGGAAGATTTTCAAAGTTTAAAATTCCAGCAAGGT
CTTCTGGTAGACTTCTTAGATTTCCCACAAAAATTTATAGATCTCCTTCAGCAATGTACTCAAGAACATG
CCAAAGAAATTCCAAGGTTTTTGCTACAGTTAGTTTCTCCAGCAGCTATTTTGGATAACTCACCTGCATT
TTTAAATGTGGTAGAGACAAATCCTTTTAAGCATCTTACACACCTCTCATTAAAACTTTTACCTGGAAAT
GATGTGGCGATAAAGAAATTTCTAGCAGGCTGTTTGAAATGTAGCAAGGAAGAAAAATTATCATTGATGC
AATCACTAGATGATGTTACTAAGCAACTGGACTTTACACAAAAGACATTAACAGAAAAAACACAAGAATT
GGATAAGTTACGGAATGAATGGGCATCACACACAGCAGCCTTGACAAACAAGCATTCTCAGGAACTGACA
AATGAAAAGGAAAAAGCCTTGCAGGCACAGGTTCAGTATCAACAGCAGCATGAAAAACAGAAAAAAGATT
TAGAAATCCTCCATCAGCAAAACATCCAGCAGCTGCAACACAGACTGTCTGATTTAGAAGCAGCTAATAA
AGACCTAACTGAAAGAAAATATAAAGGAGACTCCACTATTAGAGAACTTAAAGCAAAACTTTCTGGTGTT
GAGGAGGAGCTGCAGCGGACTAAGCAAGAGGTCCTCTCTTTGCGAAGAGAGAATTCTACATTAGATGCTG
AATGCCATGAGAAAGAAAAGCATATTAATCAGCTACAAACAAAAGTAGCAGTTTTAGAACAGGAAATCAA
GGATAAGGACCAGCTTGTTTTAAGAACAAAAGAAGCATTTGATACAATCCAGGAACAAAAGGTGGTTTTA
GAAGAAAATGGTGAGAAAAATCAAGTACAACTAGGAAAGCTTGAAGCTACAATAAAATCATTATCTGCAG
AACTTCTGAAGGCAAATGAAATTATCAAGAAGTCACAAGGGGATCTGAAAACTTTAATGGGTAAGTTGAA
ATTGAAGAATACAGTTACTATTCAGCAAGAAAAACTCTTGGCTGAGAAGGAGGAAAAATTACAAAAGGAG
CAAAAGGAATTACAAGATGTCGGGCAGTCTCTTCGAATTAAAGAGCAAGAGGTATGCAAATTACAAGAAC
AGTTAGAAGCTACAGTTAAAAAACTTGAAGAAAGCAAACAACTTCTAAAAGATAATGAAAAGTTAATCAC
ATGGTTAAATAAAGAACTAAATGTAAATCAGCTAATGAGAAAGCAAGGTGTATTGGGACTTTCTACTACT
CCGCCTGTCCATTCTAGCAGCAATGCAATCAGAAGTGGAATTTCTCCTAACCTGAATATGGTTGATGGTA
GACTGACTTACCCAACTTGTGGGATTGGTTATCCTGTCTCCTCTGCATTTGCATTCCAGAATACCTTCCC

TCATTCGATATCTGCCAAAAATACAAGCCACCCTGGTTCAGGAACAAAGGTTCAGTTTAATTTGCAATTT
AAAAAACCAAATGCTTCGCTAGGAGATGTTCAGTCAGAAGCAGTTATTAGTATGCCTTGCTCAACTGATA
AGGAAAATAGTGAAAATTTAGGGTTGGAATCCAAATACCTGAAGATAAGGGAAGATAGCATTCCTTTACG
AGGACTCAGTCAGAACCTATTTAATAATTCAGACCATCAGAAAGATGGCACGTTAGGAGCATTACAGACA
TCTTCCAAACCCACAGTGCTCTCCTCCGCATCTTCAGCATATTTCCCTGGGCAGTTACCCAACAGT
>vervet agm ENSCSAT00000017028.1
ATGAGCCAAGTTCTGTTCCACCAACTAGTCCCGTTGCAGGTGAAATGCAAAGACTGCGAG
GAGAGGAGAGTAAGTATAAGAATGAGCATTGAACTACAATCAGTTTCTAATCCAGTTCAC
AGAAAGGACTTAGTTATTCGTCTGACTGATGACACGGATCCATTTTTTTTATATAACCTT
GTTATATCTGAGGAAGATTTTCAAAGTTTAAAATTCCAGCAAGGCCTTCTGGTAGACTTC
TTAGCTTTCCCACAAAAATTTATAGATCTCCTTCAGCAATGTACTCAAGAACATGCCAAA
GAAATTCCAAGATTTTTGCTACAGTTAGTTTCTCCAGCAGCTATTTTGGATAATTCACCT
GCATTTTTAAATGTGGTAGAGACAAATCCTTTTAAGCATCTTACTCACCTCTCACTAAAA
CTTTTACCTGGAAATGATGTGGAGATAAAGAAATTTCTAGCAGGCTGTTTGAAATGTAGC
AAGGAAGAAAAATTATCATTGATGCAATCACTAGATGATGTTACTAGGCAACTGGACTTT
ACACAAAAGACATTAGCAGAAAAAAACCAAGAATTAGATAAGTTACGGAATGAATGGGCA
TCACATACAGCAGCGTTGACAAACAAGCATTCTCAGGAACTGACAAATGAAAAGGAAAAA
GCCTTGCAGGCACAGGTTCAATATCAACGGCAGCATGAACAACAGAAAAAAGATTTAGAA
ATCCTCCATCAACAAAATATCCACCAGATACAAAACAGACTGTCTGAGTTAGAAGCAGCT
AATAAAGACTTAACTGAAAGAAAATATAAAGGAGACTCCACTATTAGAGAACTTAAAGCA
AAACTTTCTGGTGTTGAAGAGGAGCTGCAGCGGACTAAGCAAGAAGTCCTCTCTTTGAGA
AGAGAGAATTCTACACTAGATGCTGAATGCCACGAGAAAGAAAAGCATATTAATCAGCTA
CAAACAAAAGTGGCAGTTTTAGAACAGGAAATCAAGGATAAGGACCAGCTTGTTTTAAGA
ACAAAAGAAGCATTTGATACAATCCAGGAACAAAAGGTGGTTTTAGAAGAAAATGGTGAG
AAAAATCAAGTACAACTAGGAAAACTTGAAGCTACAATAAAATCATTATCTGCAGAACTT
CTAAAGGCAAATGAAATTATCAAGAAGTTACAGGGGGATCTGAAAACTTTAATGGGTAAG
TTGAAATTGAAGAATACAGTTACTATTCAGCAAGAAAAACTCCTGGCTGAGAAGGAGGAA
AAATTACAAAAGGAACAAAAGGAATTACAAGATGTTGAACAGTCTCTTCGAATTAAAGAG
CAAGAGGTATGCAAATTACAAGAACAATTAGAAGCTACAGTTAAAAAACTTGAAGAAAGC
AAACAACTTCTAAAAAATAATGAAAAGTTAATCACGTGGTTAAATAAAGAACTAAATGAA
AATCAGCTAGTGAGAAAGCAAGATGTATTGGGACCTTCTACTACTCCGCCTACACATTCT
AGCAGCAACACAATCAGAAGTGGAATTTCTCCTAACCTGAATGTGGTTGATGGTAGACTG
ACTTACCCAACCTGTGGGATTGGTTATCCTGTCTCCTCTGCATTTGCATTCCAGAATACC
TTCCCTCATTCGATATCTGCCAAAAATACCAGCCACCCTGGTTCAGGAACAAAGGTTCAG
TTTAATTTGCAGTTTACAAAACCAAATGCAGCACTAGGAGACGTTCAGTCAGGAGCAACT
ATTAGTATGCCTTGCTCAACTGATAAGGAAAATAGTGAAAATTTAGGGTTGGAATCCAAA
TACCTGAAGAAAAGGGAAGATAGCATTCCTTTACGCGGACTCAGCCAGAACCTATTTAGT
AATTCAGACCATCAGAGAGATGGCACTTTAGGAGCATTACAGGTGTCTTCCAAACCCACA
GTGCTCCCCTCGGCATCTTCAGCATATTTCCCTGGGCAGCTACCAAACAGT

>macaque ENSMMUT00000006015.3
ATGAGCCAAGTTCTGTTCCACCAACTAGTCCCGTTGCAGGTGAAATGCAAAGACTGCGAG
GAGAGGAGAGTAAGTATAAGAATGAGCATTGAACTACAATCAGTTTCTAATCCAGTTCAC
AGAAAGGACTTAGTTATTCGTCTGACTGATGACACGGATCCATTTTTTTTATATAACCTT
GTTATATCTGAGGAAGATTTTCAAAGTTTAAAATTCCAGCAAGGTCTTCTGGTAGACTTC
TTAGCTTTCCCACAAAAATTTATAGATCTCCTTCAGCAATGTACTCAAGAACATGCCAAA
GAAATTCCAAGGTTTTTGCTACAGTTAGTTTCTCCAGCAGCTATTTTGGATAACTCACCC

GCATTTTTAAATGTGGTAGAGACAAATCCTTTTAAGCATCTTACTCACCTCTCACTAAAA
CTTTTACCTGGAAATGATGTGGAGATAAAGAAATTTCTAGCAGGCTGTTTGAAATGTAGC
AAGGAAGAAAAATTATCATTGATGCAATCACTAGATGATGCTACTAGGCAACTGGACTTT
ACACAAAAGACATTAGCAGAAAAAAACCAAGAATTAGATAAGTTACGGAATGAATGGGCA
TCACATACAGCAGCCTTGACAAACAAGCATTCTCAGGAACTGACAAATGAAAAGGAAAAA
GCCTTGCAGGCACAGGTTCAATATCAACGGCAACATGAACAACAGAAAAAAGATTTAGAA
ATCCTCCATCAACAAAATATCCACCAGCTACAAAACAGACTGTCTGAGTTAGAAGCAGCT
AATAAAGACTTAACTGAAAGAAAATATAAAGGAGACTCCACTATTAGAGAACTTAAAGCA
AAACTTTCTGGTGTTGAAGAGGAGTTGCAGCGGACTAAGCAAGAAGTCCTCTCTTTGCGA
AGAGAGAATTCTACACTAGATGCTGAATGCCACGAGAAAGAAAAGCATATTAATCAGCTA
CAAACAAAAGTGGCAGTTTTAGAACAGGAAATCAAGGATAAGGACCAGCTTGTTTTAAGA
ACAAAAGAAGCATTTGATACAATCCAGGAACAAAAGGTGGTTTTAGAAGAAAATGGTGAG
AAAAATCAAGTACAACTAGGAAAGCTTGAAGCTACAATAAAATCATTATCTGCAGAACTT
CTAAAGGCAAATGAAATTATCAAGAAGTTACAGGGGGATCTGAAAACTTTAATGGGTAAG
TTGAAATTGAAGAATACAGTTACTATTCAGCAAGAAAAACTCCTGGCTGAGAAGGAGGAA
AAATTACAAAAGGAACAAAAGGAATTACAAGATGTTGAACAGTCTCTTCGAATTAAAGAG
CAAGAGGTATGCAAATTACAGGAACAATTAGAAGCTACAGTTAAAAAACTTGAAGAAAGC
AAACAACTTCTAAAAAATAATGAAAAGTTAATCACGTGGTTAAATAAAGAACTAAATGAA
AATCAGCTAGTGAGAAAGCAAGATGTATTGGGACCTTCTACTACTACGCCTGCACATTCT
AGCAGCAACACAATCAGAAGTGGAATTTCTCCTAACCTGAATGTGGTTGATGGTAGACTG
ACTTACCCAACCTGTGGGATTGGTTATCCTGTCTCCTCTGCATTTGCATTCCAGAATACC
TTCCCTCATTCGATATCTGCCAAAAATACCAGCCACCCTGGTTCAGGAACAAAGGTTCAG
TTTAATTTGCAGTTTACAAAACCAAATGCATCACTAGGAGACGTTCAGTCAGGAGCAACT
ATTAGTATGCCTTGCTCAACTGATAAGGAAAATAGTGAAAATTTAGGGTTGGAATCCAAA
TACCTGAAGAAAAGGGAAGATAGTATTCCTTTACGCGGACTCAGCCAGAACCTATTTAGT
AATTCAGACCATCAGAGAGATGGCACTTTAGGAGCATTACAGGTGTCTTCCAAACCCACA
GTGCTCCCCTCGGCATCTTCAGCATATTTCCCTGGGCAGCTACCAAACAGT
>mouse lemur ENSMICT00000036029.1
ATGAGCCAAGTTCTGTTCCAGCAACTAGTCCCATTGCAGGTGAAATGCAAAGACTGCGAA
GAGAGGAGAGTAAGTGTTAGAGTGAGCATTGAACTACAATCAGTTTCTAATCCAGTTCAC
AGAAAGGACTTAGTTATCCGTCTGACTGATGACATGGATCCATTTTTTCTATATAACCTT
GTTATATCTGAGGAAGATTTTCAAAGTTTAAAATTCCAGCAAGGTCTTCTGGTAGACTTC
TTAGCTTTCCCACAAAAATTTATAGATCTCCTTCAGCAGTGTACTCAAGAACACACCAAA
GAAATTCCAAGGTTTTTACTACAGTTAGTTTCTCCAGCAGCTATTTTGGATAACTCACCT
GTATCTTTAAATGTGGTAGAAACAAACCCTTTTAAGCATCTTACACACCTCTCACTAAAA
CTTTTACCTGGAAATGATGTGGAGATAAAGAAATTTCTAGCAGGCTGTTTGAAATGTAGC
AAGGAAGAAAAATTATCATTGACACAATCACTAGATGATGTTACTAGGAAACTGAACTTC
ACGCAAAAGACATTATCAGAAAAAATCCAAGAATTAGATAAGTTACGGAACGAATGGGCA
TCACACACGGCAGCATTGTCAAATAAGCACTCTCAGGAACTAACAAATGAGAAGGAAAAA
GCCTTGCAGGCACAGGTTCAATATCAACAGCAGCATGAACAACAGAAAAAAGATCTAGAA
ATTCTCCATCAACAAAACATCCATCAGCTACAAAACAGAGTGTCTGAGTTAGAAGCAGCT
AATAAAGACCTAACTGAAAGAAAATATAAAGGAGACTCCACTATTAGAGAACTTAAAGCA
AAACTTTCTGGTGTTGAAGAGGAGTTGCAGCGAACTAAGCAAGAAGTCCTGTCTTTGCGA
AGAGAGAATTCTACACTAGATGCTGAATGCCATGAGAAAGAAAAGCATATTAATCAGCTA
CAAACAAAAGTGGCAGTTTTAGAACAGGAAATCAAGGATAAGGACCAGCTTGTTTTAAGA
ACAAAAGAAGCATTTGATACAATCCAGGAACAGAAGGTGGCCTTAGAAGAAAATGGGGAG
AAAAATCAAGTACAGCTAGGAAAACTTGAAGCTACAATAAAATCATTATCAGCAGAACTT

CTGAAGGCAAATGAAATTATCAAGAAGTTACAAGGAGATCTGAAAACTCTAATGGGTAAG
TTGAAACTGAAGAATACAGTTACTATTCAGCAGGAAAAACTCTTGGCTGAGAAAGAGGAA
AAATTACAAAAGGAGCAAAAGGAATTACAAGATGTTGGACAGTCTCTTCGAATTAAAGAG
CAAGAGGTGTGCAAGTTACAAGAACAATTAGAAGCTACAGTTCAAAAACTTGAAGAAAGC
AAACAGCTTTTAAAAAATAATGAAAAGTTAATCACATGGTTAAATAAAGAATTAAATGAA
AATCAGCTGGTGAGAAAGCAAGACGTATTGGTACCTTCTACTACTCCACCTGCACATTCT
AGCAGCAACACAATCAGAAGTGGAATTTCTCCTAACTTGAATGTGGTTGATGGTAGACTA
ACTTACCCAACTTGTGGGATTGGCTATCCTGTCTCCTCTGCATTTGCATTTCAAAATACC
TTTCCTCATTCCATATCTGCCAAAAATGCCCTCCACTCAGTTTCAGGACCAAAGGTTCAG
TTTAACTTGCAATTTACAAAACCAAATACATCGCTAGGAGATGTTCAGTCAGGAGCAACT
ATGCCTTGCTCAGCTGATAAGGAAAATGGTGAAAATTTAGGGCTGGAATCCAAATACCTG
AAGAAAAGGGAAGATAGCATTCCTTTACGTGGACTCAGCCAGAATCTATTTAGTAATTCA
GACCATCAGAAAGATGGCACTCTGGGAGCACTACAGACATCTTCCAAACCCACAGTGCTC
CCTTCTACATCTTCAGCATATTTCCCTGGGCAGTTACCAAACAGT
>Nomascus leucogenys XM_003260110.3
ATGAGCCAAGTTCTGTTCCACCAACTAGTCCCGTTGCAGGTGAAATGCAAAGACTGCGAGGAGAGGAGA
GTAAGTATAAGAATGAGCATTGAACTACAATCAGTTTCTAATCCAGTTCACAGAAAGGACTTAGTTATTC
GTCTGACTGATGACACGGATCCATTTTTTTATATAACCTTGTTATATCTGAGGAAGATTTTCAAAGTTT
AAAATTCCAGCAAGGTCTTCTGGTAGACTTCTTAGCTTTCCCACAAAAATTTATAGATCTCCTTCAGCAA
TGTACTCAAGAACATGCCAAAGAAATTCCAAGGTTTTTGCTGCAGTTAGTTTCTCCAGCAGCTATTTTGG
ATAACTCACCTGCATTTTTAAATGTGGTAGAGACAAATCCTTTTAAGCATCTTACACACCTCTCACTAAA
ACTGTTACCTGGAAATGATGTGGAGATAAAGAAATTTCTCGCAGGCTGTTTGAAATGTAGCAAGGAAGAA
AAATTATCATTGATGCAATCACTAGATGATGTTACTAGGCAACTGGACTTTACACAAAAGACATTAGCAG
AAAAAAAACAAGAATTAGATAAGTTACGGAATGAATGGGCATCACATACAGCAGCCTTAACAAACAAGCA
TTCTCAGGAACTGACAAATGAAAAGGAAAAAGCCTTGCAGGCACAGGTTCAATATCAACAGCAGCATGAA
CAACAGAAAAAAGATTTAGAAATCCTCCATCAACAAAACATCCACCAGCTGCAAAACAGACTGTCTGAGT
TAGAAGCAGCTAATAAAGACTTAACCGAAAGAAAATATAAAGGAGACTCCACTATTAGAGAACTTAAAGC
AAAACTTTCTGGTGTTGAAGAGGAGCTACAGCGGACTAAGCAAGAAGTCCTCTCTTTGCGAAGAGAGAAT
TCTACACTAGATGTTGAATGCCACGAGAAAGAAAAGCACATTAATCAGCTACAAACAAAAGTGGCAGTTT
TAGAACAGGAAATCAAGGATAAGGACCAGCTTGTTTTAAGAACAAAAGAAGCATTTGATACAATCCAGGA
ACAAAAGGTGGTTTTAGAAGAAAATGGTGAGAAAAATCAAGTACAACTAGGAAAGCTTGAAGCTACAATA
AAATCATTATCTGCAGAACTTCTGAAGGCAAATGAAATTATCAAGAAGTTACAAGGGGATCTGAAAACTT
TAATGAGTAAGTTGAAATTGAAGAATACAGTTACTATTCAGCAAGAAAAACTCTTGGCTGAGAAGGAGGA
AAAGTTACAAAAGGAACAAAAGGAATTACAAGATGTCGGACAGTCTCTTCGAATTAAAGAGCAAGAGGTA
TGCAAATTACAAGAACAATTAGAAGCTACAGTTAAAAAACTTGAAGAAAGCAAACAACTTCTAAAAAATA
ATGAAAAGTTAATCACGTGGTTAAATAAAGAACTAAATGAAAATCAGCTAGTGAGAAAGCAAGATGTATT
GGGACCTTCTACTACTACGCCTGCACATTCTAGCAGCAACACAATCAGAAGTGGAATTTCTCCTAACCTG
AATGTGGTTGATGGTAGACTGACTTACCCAACCTGTGGGATTGGTTATCCTGTCTCCTCTGCATTTGCAT
TCCAGAATACCTTCCCTCATTCGATATCTGCCAAAAATACCAGCCACCCTGGTTCAGGAACAAAGGTTCA
GTTTAATTTGCAGTTTACAAAACCAAATGCATCACTAGGAGATGTTCAGTCAGGAGCAACTATTAGTATG
CCTTGCTCAACTGATAAGGAAAATGGTGAAAATTTAGGGTTGGAATCCAAATACCTGAAGAAAAGGGAAG
ACAGCATTCCTTTACGTGGACTCAGCCAGAACCTATTTAGTAATTCAGACCATCAGAGAGATGGCACTTT
AGGAGCATTACATACATCTTCCAAACCCACAGCGCTCCCCTCTGCATCTTCAGCCTATTTCCCTGGGCAG
TTACCAAACAGT
>bushbaby ENSOGAT00000005619.2
ATGAGCCAAGTTCTGTTCCAGCAACTCGTCCCGTTGCAGGTGAAATGCAAAGACTGCGAA
GAGAGGAGAGTAAGTGTTAGAGTGAGCATTGAACTGCAATCAGTTTCCAATCCGATTCAC

AGAAAGGACTTAGTTATCCGTCTGACTGATGACACAGATCCATTTTTTCTATATAATCTT
GTTATATCTGAAGAAGATTTTCAAAGTTTAAAATTCCAGCAAGGTCTTCTGGTAGACTTC
TTAGCTTTCCCACAAAAATTTATAGATCTCCTTCAGCAGTGTACTCAGGAATACGCCAAA
GAAATTCCAAGGTTTTTGCTACAGTTAGTTTCTTCAGCAGCTATTTTGGATAACTCACCT
GTATTTTTAAATGTGGTAGAAACAAATCCTTTTAAACATCTTACACACCTCTCACTAAAA
CTTTTACCTGGAAATGATGTGGAGATAAAGAAATTTCTAGCTGACTGTTTGAAATGTAGC
AAGGAGGAAAAATTATCATTGATACAATCACTAGATGATGTAACTAGACAACTGAACTTC
ACACAGAAGACATTATCAGAAAAAATCCAAGAATTAGATAAGTTACGGAATGAATGGGCA
TCACACACAGCAGCATTGACAAATAAGCACTCTCAGGAACTGACAAATGAGAAGGAAAAA
GCCTTGCAGGCACAGGTTCAATATCAACAGCAGCATGAGCAACAGAAAAAAGATTTAGAA
ATCCTGCATCAACAGAACATCCACCAGCTACAGAACAGATTGTCTGAGTTAGAAGCAGCT
AACAAAGACCTGACCGAGAGGAAATATAAAGGAGACTCCACTATTAGAGAACTTAAAGCA
AAACTTTCTGGTATTGAAGAGGAGCTGCAGCGAACTAAGCAAGAAGTCATCTCTTTGCGA
AGAGAAAATTCTACCCTCGATGCTGAATGCCATGAGAAAGAAAAGCAGATTAATCAGCTA
CAAACAAAGGTGGCAGTTTTAGAACAGGAAATCAAGGATAAGGACCAACTTGTCTTAAGA
ACAAAAGAAGCATTTGATACAATCCAGGAACAGAAGGTGGCCTTAGAAGAAAATGGGGAG
AAAAATCAAGTACAACTAGGAAAACTTGAAGCTACAATAAAATCATTATCAGCAGAACTT
CTAAAGGCAAATGAAATTATTAAGAAGTTACAAGGGGATCTGAAAACTCTAATGGGTAAG
TTGAAACTGAAGAATACAGTTACTATTCAGCAGGAAAAACTCTTGGCTGAGAAGGAGGAA
AAATTACAAAAGGAGCAAAAGGAATTACAAGATGTTGGACAGTCTCTTCGAATTAAAGAG
CAAGAGGTGTGCAAGTTACAAGACCAATTAGAAGCTACAGTTCAAAAACTTGAAGAAAGC
AAACAGCTTCTAAAAAATAATGAAAAATTAATCACGTGGTTAAACAAAGAGCTCAATGAG
AATCAGTTAGTGAGAAAGCAAGACACCTTGGGACCATCTACCACCCCACCTTCCCATTCT
AGCAGCAACACGATCCGAAGTGGAATTTCTCCTAACTTGAATGTGGTTGATGGCAGACTG
ACTTACCCCACTTGTGGGATTGGCTATCCTGTCTCCTCTGCATTTGCATTCCAGAATACC
TTTCCTCATTCAGTATCTGCCAAAAATACCGTCCACCCGATTACAGGACCAAAGGTTCAG
TTTAACTTGCAGTTTACAAAACCAAATACATCAGTAGGAGATGTTCAATCAGGAGCAACT
TGCTCAGCAGATAAGGAAAATGGTGAAAACTTAGGGCTGGAATCCAAATACCTGAAGAAA
AGGGAAGACAGCATTCCTTTACGCGGACTCAGCCAGAATCTGTTCAGTAATTCAGACCAT
CAGAAAGATGGCACTCTGGGAGCACTGCCGACAGCCTCCAAACCCACAGGGCTCCCCTCT
GCGTCTTCAGCGTATTTCCCTGGGCAGTTACCAAACAGT
>chimpanzee ENSPTRT00000001874.4
ATGAGCCAAGTTCTGTTCCACCAACTAGTCCCGTTGCAGGTGAAATGCAAAGACTGTGAG
GAGAGGAGAGTAAGTATAAGAATGAGCATTGAACTACAATCAGTTTCTAATCCAGTTCAC
AGAAAGGACTTAGTTATTCGTCTGACTGATGACACGGATCCATTTTTTTATATAACCTT
GTTATATCTGAGGAAGATTTTCAAAGTTTAAAATTCCAGCAAGGTCTTCTGGTAGACTTC
TTAGCTTTCCCACAAAAATTTATAGATCTCCTTCAGCAATGTACTCAAGAACATGCCAAA
GAAATTCCAAGGTTTTTGCTACAGTTAGTTTCTCCAGCAGCTATTTTGGATAACTCACCT
GCATTTTTAAATGTGGTAGAGACAAATCCTTTTAAGCATCTTACACACCTCTCACTAAAA
CTTTTACCTGGAAATGATGTGGAGATAAAGAAATTTCTCGCAGGCTGTTTGAAATGTAGC
AAGGAAGAAAAATTATCATTGATGCAATCACTAGATGATGCTACTAAGCAACTGGACTTT
ACACGAAAGACATTAGCAGAAAAAAAACAAGAATTAGATAAGTTACGGAATGAATGGGCG
TCACATACAGCAGCCTTGACAAACAAGCATTCTCAGGAACTGACAAATGAAAAGGAAAAA
GCCTTGCAGGCACAGGTTCAATATCAACAGCAGCATGAACAACAGAAAAAAGATTTAGAA
ATCCTCCATCAACAAAACATCCACCAGCTACAAAACAGACTGTCTGAGTTAGAAGCGGCT
AATAAAGACTTAACCGAAAGAAAATATAAAGGAGACTCCACTATTAGAGAACTTAAAGCA
AAACTTTCTGGTGTTGAAGAGGAGCTACAGCGGACTAAGCAAGAAGTCCTCTCTTTGCGA

AGAGAGAATTCTACACTAGATGTTGAATGCCACGAGAAAGAAAAGCACGTTAATCAGCTG
CAAACAAAAGTGGCAGTTTTAGAACAGGAAATCAAGGATAAGGACCAGCTTGTTTTAAGA
ACAAAAGAGGCATTTGATACAATCCAGGAACAAAAGGTGGTTTTAGAAGAAAATGGTGAG
AAAAATCAAGTACAACTAGGAAAGCTTGAAGCTACAATAAAATCATTATCTGCAGAACTT
CTGAAGGCAAATGAAATTATCAAGAAGTTACAAGGGGATCTGAAAACTTTAATGGGTAAG
TTGAAATTGAAGAATACAGTTACTATTCAGCAAGAAAAACTCTTGGCTGAGAAGGAGGAA
AAATTACAAAAGGAACAAAAGGAATTACAAGATGTTGGACAGTCTCTTCGAATTAAAGAA
CAAGAGGCATGCAAATTACAAGAACAATTAGAAGCTACAGTTAAAAAACTTGAAGAAAGC
AAACAACTTCTAAAAAATAATGAAAAGTTAATCACGTGGTTAAATAAAGAACTAAATGAA
AATCAGCTAGTGAGAAAGCAAGATGTATTGGGACCTTCTACTACTCCGCCTGCACATTCC
AGCAGCAACACAATCAGAAGTGGAATTTCTCCTAACCTGAATGTGGTTGATGGTAGACTG
ACTTACCCAACCTGTGGGATTGGTTATCCTGTCTCCTCTGCATTTGCATTCCAGAATACC
TTCCCTCATTCGATATCTGCCAAAAATACCAGCCACCCTGGTTCAGGAACAAAGGTTCAG
TTTAATTTGCAGTTTACAAAACCAAATGCATCACTAGGAGATGTTCAGTCAGGAGCAACT
ATTAGTATGCCTTGCTCAACTGATAAGGAAAATGGTGAAAATGTAGGGTTGGAATCCAAA
TACCTGAAGAAAAGGGAAGATAGCATTCCTTTACGCGGACTCAGCCAGAACCTATTTAGT
AATTCAGACCATCAGAGAGATGGCACTTTAGGAGCATTACATACATCTTCCAAACCCACA
GCGCTCCCCTCTGCGTCTTCAGCCTATTTCCCTGGGCAGTTACCAAACAGT
>Pan paniscus XM_003808468.2
ATGAGCCAAGTTCTGTTCCACCAACTAGTCCCGTTGCAGGTGAAATGCAAAGACTGTGAGGAGAGGAGAGTA
AG
TATAAGAATGAGCATTGAACTACAATCAGTTTCTAATCCAGTTCACAGAAAGGACTTAGTTATTCGTCTG
ACTGATGACACGGATCCATTTTTTTTATATAACCTTGTTATATCTGAGGAAGATTTTCAAAGTTTAAAAT
TCCAGCAAGGTCTTCTGGTAGACTTCTTAGCTTTCCCACAAAAATTTATAGATCTCCTTCAGCAATGTAC
TCAAGAACATGCCAAAGAAATTCCAAGGTTTTTGCTACAGTTAGTTTCTCCAGCAGCTATTTTGGATAAC
TCACCTGCATTTTTAAATGTGGTAGAGACAAATCCTTTTAAGCATCTTACACACCTCTCACTAAAACTTT
TACCTGGAAATGATGTGGAGATAAAGAAATTTCTCGCAGGCTGTTTGAAATGTAGCAAGGAAGAAAAATT
ATCATTGATGCAATCACTAGATGATGCTACTAAGCAACTGGACTTTACACGAAAGACATTAGCAGAAAAA
AAACAAGAATTAGATAAGTTACGGAATGAATGGGCGTCACATACAGCAGCCTTGACAAACAAGCATTCTC
AGGAACTGACAAATGAAAAGGAAAAAGCCTTGCAGGCACAGGTTCAATATCAACAGCAGCATGAACAACA
GAAAAAAGATTTAGAAATCCTCCATCAACAAAACATCCACCAGCTACAAAACAGACTGTCTGAGTTAGAA
GCGGCTAATAAAGACTTAACCGAAAGAAAATATAAAGGAGACTCCACTATTAGAGAACTTAAAGCAAAAC
TTTCTGGTGTTGAAGAGGAGCTACAGCGGACTAAGCAAGAAGTCCTCTCTTTGCGAAGAGAGAATTCTAC
ACTAGATGTTGAATGCCACGAGAAAGAAAAGCACGTTAATCAGCTGCAAACAAAAGTGGCAGTTTTAGAA
CAGGAAATCAAGGATAAGGACCAGCTTGTTTTAAGAACAAAAGAGGCATTTGATACAATCCAGGAACAAA
AGGTGGTTTTAGAAGAAAATGGTGAGAAAAATCAAGTACAACTAGGAAAGCTTGAAGCTACAATAAAATC
ATTATCTGCAGAACTTCTGAAGGCAAATGAAATTATCAAGAAGTTACAAGGGGATCTGAAAACTTTAATG
GGTAAGTTGAAATTGAAGAATACAGTTACTATTCAGCAAGAAAAACTCTTGGCTGAGAAGGAGGAAAAAT
TACAAAAGGAACAAAAGGAATTACAAGATGTTGGACAGTCTCTTCGAATTAAAGAGCAAGAGGTATGCAA
ATTACAAGAACAATTAGAAGCTACAGTTAAAAAACTTGAAGAAAGCAAACAACTTCTAAAAAATAATGAA
AAGTTAATCACGTGGTTAAATAAAGAACTAAATGAAAATCAGCTAGTGAGAAAGCAAGATGTATTGGGAC
CTTCTACTACTCCGCCTGCACATTCCAGCAGCAACACAATCAGAAGTGGAATTTCTCCTAACCTGAATGT
GGTTGATGGTAGACTGACTTACCCAACCTGTGGGATTGGTTATCCTGTCTCCTCTGCATTTGCATTCCAG
AATACCTTCCCTCATTCGATATCTGCCAAAAATACCAGCCACCCTGGTTCAGGAACAAAGGTTCAGTTTA
ATTTGCAGTTTACAAAACCAAATGCATCACTAGGAGATGTTCAGTCAGGAGCAACTATTAGTATGCCTTG
CTCAACTGATAAGGAAAATGGTGAAAATGTAGGGTTGGAATCCAAATACCTGAAGAAAAGGGAAGATAGC
ATTCCTTTACGCGGACTCAGCCAGAACCTATTTAGTAATTCAGACCATCAGAGAGATGGCACTTTAGGAG

CATTACATACATCTTCCAAACCCACAGCGCTCCCCTCTGCGTCTTCAGCCTATTTCCCTGGGCAGTTACC
AAACAGT
>olive baboon ENSPANT00000006786.2
ATGAGCCAAGTTCTGTTCCACCAACTAGTCCCGTTGCAGGTGAAATGCAAAGACTGCGAG
GAGAGGAGAGTAAGTATAAGAATGAGCATTGAACTACAATCAGTTTCTAATCCAGTTCAC
AGAAAGGACTTAGTTATTCGTCTGACTGATGACACGGATCCATTTTTTTTATATAACCTT
GTTATATCTGAGGAAGATTTTCAAAGTTTAAAATTCCAGCAAGGTCTTCTGGTAGACTTC
TTAGCTTTCCCACAAAAATTTATAGATCTCCTTCAGCAATGTACTCAAGAACATGCCAAA
GAAATTCCAAGATTTTTGCTACAGTTAGTTTCTCCAGCAGCTATTTTGGATAACTCACCC
GCATTTTTAAATGTGGTAGAGACAAATCCTTTTAAGCATCTTACTCACCTCTCACTAAAA
CTTTTACCTGGAAATGATGTGGAGATAAAGAAATTTCTAGCAGGCTGTTTAAAATGTAGC
AAGGAAGAAAAATTATCATTGATGCAATCACTAGATGATGCTACTAGGCAACTGGACTTT
ACACAAAAGACATTAGCAGAAAAAAAACAAGAATTAGATAAGTTACGGAATGAATGGGCA
TCACATACAGCAGCCTTGACAAACAAGCATTCTCAGGAACTGACAAATGAAAAGGAAAAA
GCGTTGCAGGCACAGGTTCAATATCAACGGCAGCATGAACAACAGAAAAAAGATTTAGAA
ATCCTCCATCAACAAAATATCCACCAGCTACAAAACAGACTGTCTGAGTTAGAAGCAGCT
AATAAAGACTTAACTGAAAGAAAATATAAAGGAGACTCCACTATTAGAGAACTTAAAGCA
AAACTTTCTGGTGTTGAAGAGGAGCTGCAGCGGACTAAGCAAGAAGTCCTCTCTTTGCGA
AGAGAGAATTCTACACTAGATGCTGAATGCCACGAGAAAGAAAAGCATATTAATCAGCTA
CAAACAAAAGTGGCAGTTTTAGAACAGGAAATCAAGGATAAGGACCAGCTTGTTTTAAGA
ACAAAAGAAGCATTTGATACAATCCAGGAACAAAAGGTGGTTTTAGAAGAAAATGGTGAG
AAAAATCAAGTACAACTAGGAAAGCTTGAAGCTACAATAAAATCATTATCTGCAGAACTT
CTAAAGGCAAATGAAATTATCAAGAAGTTACAGGGGGATCTGAAAACTTTAATGGGTAAG
TTGAAATTGAAGAATACAGTTACTATTCAGCAAGAAAAACTCCTGGCTGAGAAGGAGGAA
AAATTACAAAAGGAACAAAAGGAATTACAAGATGTTGAACAGTCTCTTCGAATTAAAGAG
CAAGAGGTATGCAAATTACAAGAACAATTAGAAGCTACAGTTAAAAAACTTGAAGAAAGC
AAACAACTTCTAAAAAATAATGAAAAGTTAATCACGTGGTTAAATAAAGAACTAAATGAA
AATCAGCTAGTGAGAAAGCAAGATGTATTGGGACCTTCTACTACTCCGCCTGCACTTTCT
AGCAGCAACACAATCAGAAGTGGAATTTCTCCTAACCTGAATGTGGTTGATGGTAGACTG
ACTTACCCAACCTGTGGGATTGGTTATCCTGTCTCCTCTGCATTTGCATTCCAGAATACC
TTCCCTCATTCGATATCTGCCAAAAATACCAGCCACCCTGGTTCAGGAACAAAGGTTCAG
TTTAATTTGCAGTTTACAAAACCAAATGCATCACTAGGAGACGTTCAGTCAGGAGCAACT
ATTAGTATGCCTTGCTCAACTGATAAGGAAAATAGTGAAAATTTAGGGTTGGAATCCAAA
TACCTGAAGAAAAGGGAAGATAGCATTCCTTTACGCGGACTCAGCCAGAACCTATTTAGT
AGTTCAGACCATCAGAGAGATGGCACTTTAGGAGCATTACAGGTGTCTTCCAAACCCACA
GTGCTCCCCTCGGCATCTTCAGCATATTTCCCTGGGCAGCTACCAAACAGT
>orangutan ENSPPYT00000001343.2
ATGAGCCAAGTTCTGTTCCACCAACTAGTCCCGTTGCAGGTGAAATGCAAAGACTGCGAG
GAGAGGAGAGTAAGTATAAGAATGAGCATTGAACTACAATCAGTTTCTAATCCAGTTCAC
AGAAAGGACTTAGTTATTCGTCTGACTGATGACACGGATCCTTTTTTTTTATATAACCTT
GTTATATCTGAGGAAGATTTTCAAAGTTTAAAATTCCAGCAGGGTCTTCTGGTAGACTTC
TTAGCTTTCCCACAAAAATTTATAGATCTCCTTCAACAATGTACTCAAGAACATGCCAAA
GAAATTCCAAGGTTTTTGCTACAGTTAGTTTCTCCAGCAGCTATTTTGGATAACTCACCT
GCATTTTTAAATGTGGTAGAGACAAATCCTTTTAAGCATCTTACACACCTCTCACTAAAA
CTTTTACCTGGAAATGATGTGGAGATAAAGAAATTTCTCGCAGGCTGTTTGAAATGTAGT
AAGGAAGAAAAATTATCATTGATGCAATCACTAGATGATGCTACTAGGCAACTGGACTTT
ACACAAAAGACATTAGCAGAAAAAAAACAAGAATTAGATAAGTTACGGAACGAATGGGCG

TCACATACAGCAGCCTTGACAAACAAGCATTCTCAGGAACTGACAAATGAAAAGGAAAAA
GCTTTGCAGGCACAGGTTCAATATCAACAGCAGCATGAACAGCAGAAAAAAGATTTAGAA
ATCCTCCATCAACAAAACATCCACCAGCTACAAAACAGACTGTCTGAGTTAGAAGCAGCT
AATAAAGACTTAACCGAAAGAAAATATAAAGGAGACTCCACTATTAGAGAACTTAAAGCA
AAACTTTCTGGTGTTGAAGAGGAGCTACAGCGGACTAAGCAAGAAGTCCTTTCTTTGCGA
AGAGAGAATTCTACACTAGATGTTGAATGCCATGAGAAAGAAAAGCACATTAATCAGCTA
CAAACAAAAGTGGCAGTTTTAGAACAGGAAATCAAGGATAAGGACCAGCTTGTTTTAAGA
ACAAAAGAGGCATTTGATACAATCCAGGAACAAAAGGTGGTTTTAGAAGAAAATGGTGAG
AAAAATCAAGTACAACTAGGAAAGCTTGAAGCTACAATAAAATCATTATCTGCAGAACTT
CTGAAGGCAAATGAAATTATCAAGAAGTTACAAGGGGATCTGAAAACTTTAATGGGTAAG
TTGAAATTGAAGAATACAGTTACTATTCAGCAAGAAAAACTCTTGGCTGAGAAGGAGGAA
AAATTACAAAAGGAACAAAAGGAATTACAAGATGTTGGACAGTCTCTTCGAATTAAAGAG
CAAGAGGTATGCAAATTACAAGAACAATTAGAAGCTACAGTTAAAAAACTTGAAGAAAGC
AAACAACTTCTAAAAAATAATGAAAAGTTAATCACGTGGTTAAATAAAGAACTAAATGAA
AATCAGCTAGTGAGAAAGCAAGATGTATTGGGACCTTCTACTACTCCGCCTGCACATTCC
AGCAGCAACACAATCAGAAGTGGAATTTCTCCTAACCTGAATGTGGTTGATGGTAGACTG
ACTTACCCAGCCTGTGGGATTGGTTATCCTGTCTCCTCTGCATTTGCATTCCAGAATACC
TTCCCTCATTCGATATCTGCCAAAAATACCAGCCACCCTGGTTCAGCAACAAAGGTTCAG
TTTAATTTGCAGTTTACAAAACCAAATGCATCACTAGGAGATGTTCAGTCAGGAGCAACT
ATTAGTATGCCTTGCTCAACTGATAAGGAAAATGGTGAAAATTTAGGGTTGGAATCCAAA
TACCTGAAGAAAAGGGAAGATAGCATTCCTTTACGCGGACTCAGCCAGAACCTATTTAGT
AATTCAGACCATCAGAGAGATGGCACTTTAGGAGCATTACATACATCTTCCAAACCCACA
GTGCTCCCTTCTGCATCTTCAGCCTATTTCCCTGGGCAGTTACCAAACAAT
>Carlito syrichta XM_008064712.1
ATGAGCCAAGTGCTGTTCCAGCAACCCGTCCCGCTGCAGGTGAAATGCAAGGACTGCGAGGAGAGGAG
AGTAAGTGTTAGAGTGAGCATCGAGCTACAATCAGTTTCTAATCCAGTTCACAGAAAGGATCTAGTTATT
CGTCTGACTGATGACACGGATCCGTTTTTTCTGTATAACCTTGTTATATCTGAGGAAGATTTTCAAAGCT
TAAAAGTCCAGCAAGGCCTTCTGGTGGACTTCTTAGCTTTCCCACAGAAGTTCATAGATCTTCTTCAACA
GTGCACTCAAGAGCACGCCAAGGAAATTCCAAGGTTTTTGCTACAGTTAGTTTGTCCAGCAACAATTTTG
GATAACTCACCTGCATTTTTAAATGTGGTAGAAACAAATCCCTTTAAGCATCTTACACACCTCTCACTAA
AACTTTTACCTGGAAATGATGTGGAAATAAAGAAATTTCTAGCAGGCTGCCTGAAATGTAGCAAGGAAGA
AAAATTAGCATTGATGCAATCACTAGATGATGTTACTAGGCAACTGAACTTCACACAAAAGACATTATCA
GAAAAAACCCAAGAATTAGACAAGTTACGGAATGAATGGGTGTCACACACAGCAGCGTTGACAAATAAGC
ATTCTCAGGAACTGACGAGTGAGAAGGAGAAAGCCTTGCAGGCACAGGTACAATATCAACAACAGCATGA
ACAGCAGAATAAAGATTTAGAAATCCTCCATCAACAAAACATCCACCAGCTACAAAACAGACTGTCTGAG
TTGGAAACTGCTAATAAAGACCTGACGGAAAGAAAATATAAAGGAGATGCCACTGTCAGAGAGCTGAAGA
CCAAGCTCTCGGGTGTGGAGGAGGAGCTGCAGCGGACCAAGCAAGAAGTCCTTTCACTGCGAAGAGAGAA
CTCTGCACTAGATGCTGAGTGCCACGAGAAAGAAAAGCACATTAATCAGCTGCAGACAAAGGTGGCCGTT
TTAGAACAGGAAATCAAGGATAAGGACCAGCTTGTTTTAAGAACAAAAGAGGCTTTTGATACAATCCAGG
AACAGAAGGTGGCTTTAGAAGAAAACGGTGAGAAAAACCAAGTGCAACTCGGAAAACTTGAAGCTACAAT
AAAATCATTGTCAGCAGAACTTCTGAAGGCAAATGAAATCATCAAGAAGTTGCAAGGGGATCTGAAAATG
CTCCTGGGTAAACTGAAGCTGAAGAACACAGTCACCATTCAGCAGGAAAAGCTCCTGGCTGAGAAGGAGG
AGAAATTGCAGAAGGGACAGAAGGAGCTGCAGGATGTTGGCCAGGCCCTTCATGTCAAGGAGCAGGAGGT
ATGCAAATTACAAGAGCAGTTAGAAGCTACAGTTCAGAAACTTGAAGAAAGCAAACAGCTTCTAAAAAAT
AATGAAAAGTTAATCACATGGTTAAATAAAGAGCTCAATGAAAATCAGGTCATGAGGAAGCAGGATGCGT
TGGTGCCTTCCATGACTCCACCTGCACATGCCAGCAGCACCGCGGTCAGAAGTGGGATTTCTCCTAACAT
GAACATGGTTGATGGTAGGCTGACTTACCCAACTTGTGGGATTGGTTATCCTGTCTCTTCTGCATTTGCA

TTCCAGAATACCTTCCCTCATTTGATAACTGCCAAAAATACTGTCCACTCTGTTTCAGGACCAAAGGTTC
AGTTTAACCTGCAGTTCACCAAACCAAACACATCGCTTGGAGACATCCAGTCAGGTGCACCTATAAGTGC
GCCATGCTCAGCTGACAAGGAGAATGGTGAAAATTTAGGGCTGGAATCTAAGTACCTGAAGAAGAGGGAA
GATAGCATTCCATTACGAGGACTCAGCCAGAACCTCTTCAGCTCGGACCTTCAGAAAGACGGCAGCCTCG
GAGCCGCGCCCACGGCGTCCAAGCCCATGGCACTCGCGGCCACCTCGTCAGCCTACTTCCCTGGGCAGTT
GCCGAGCAGT
>Cercocebus atys XM_012069381.1
ATGAGCCAAGTTCTGTTCCACCAACTAGTCCCGTTGCAGGTGAAATGCAAAGACTGCGAGGAGAGGAG
AGTAAGTATAAGAATGAGCATTGAACTACAATCAGTTTCTAATCCAGTTCACAGAAAGGACTTAGTTATT
CGTCTGACTGATGACACGGATCCATTTTTTTATATAACCTTGTTATATCTGAGGAAGATTTTCAAAGTT
TAAAATTCCAGCAAGGTCTTCTGGTAGACTTCTTAGCTTTCCCACAAAAATTTATAGATCTCCTTCAGCA
ATGTACTCAAGAACATGCCAAAGAAATTCCAAGATTTTTGCTACAGTTAGTTTCTCCAGCAGCTATTTTG
GATAACTCACCCGCATTTTTAAATGTGGTAGAGACAAATCCTTTTAAGCATCTTACTCACCTCTCACTAA
AACTTTTACCTGGAAATGATGTGGAGATAAAGAAATTTCTAGCAGGCTGTTTGAAATGTAGCAAGGAAGA
AAAATTATCATTGATGCAATCACTAGATGATGCTACTAGGCAACTGGACTTTACACAAAAGACATTAGCA
GAAAAAAAACAAGAATTAGATAAGTTACGGAATGAATGGGCATCACATACAGCAGCCTTGACAAACAAGC
ATTCTCAGGAACTGACAAATGAAAAGGAAAAAGCCTTGCAGGCACAGGTTCAATATCAACGGCAGCATGA
ACAACAGAAAAAAGATTTAGAAATCCTCCATCAACAAAATATCCACCAGCTACAAAACAGACTGTCTGAG
TTAGAAGCAGCTAATAAAGACTTAACTGAAAGAAAATATAAAGGAGACTCCACTATTAGAGAACTTAAAG
CAAAACTTTCTGGTGTTGAAGAGGAGCTGCAGCGGACTAAGCAAGAAGTCCTCTCTTTGCGAAGAGAGAA
TTCTACACTAGATGCTGAATGCCACGAGAAAGAAAAGCATATTAATCAGCTACAAACAAAAGTGGCAGTT
TTAGAACAGGAAATCAAGGATAAGGACCAGCTTGTTTTAAGAACAAAAGAAGCATTTGATACAATCCAGG
AACAAAAGGTGGTTTTAGAAGAAAATGGTGAGAAAAATCAAGTACAACTAGGAAAGCTTGAAGCTACAAT
AAAATCATTATCTGCAGAACTTCTAAAGGCAAATGAAATTATCAAGAAGTTACAGGGGGATCTGAAAACT
TTAATGGGTAAGTTGAAATTGAAGAATACAGTTACTATTCAGCAAGAAAAACTCCTGGCTGAGAAGGAGG
AAAAATTACAAAAGGAACAAAAGGAATTACAAGATGTTGAACAGTCTCTTCGAATTAAAGAGCAAGAGGT
ATGCAAATTACAAGAACAATTAGAAGCTACAGTTAAAAAACTTGAAGAAAGCAAACAACTTCTAAAAAAT
AATGAAAAGTTAATCACGTGGTTAAATAAAGAACTAAATGAAAATCAGCTAGTGAGAAAGCAAGATGTAT
TGGGACCTTCTACTACTCCGCCTGCACATTCTAGCAGCAACACAATCAGAAGTGGAATTTCTCCTAACCT
GAATGTGGTTGATGGTAGACTGACTTACCCAACCTGTGGGATTGGTTATCCTGTCTCCTCTGCATTTGCA
TTCCAGAATACCTTCCCTCATTCGATATCTGCCAAAAATACCAGCCACCCTGGTTCAGGAACAAAGGTTC
AGTTTAATTTGCAGTTTACAAAACCAAATGCATCACTAGGAGACGTTCAGTCAGGAGCAACTATTAGTAT
GCCTTGCTCAACTGATAAGGAAAATAGTGAAAATTTAGGGTTGGAATCCAAATACCTGAAGAAAAGGGAA
GACAGCATTCCTTTACGCGGACTCAGCCAGAACCTATTTAGTAGTTCAGACCATCAGAGAGATGGCACTT
TAGGAGCATTACAGGTGTCTTCCAAACCCACAGTGCTCCCCTCGGCATCTTCAGCATATTTCCCTGGGCA
GCTACCAAACAGT

>Mandrillus leucophaeus XM_011992525.1
ATGAGCCAAGTTCTGTTCCACCAACTAGTCCCGTTGCAGGTGAAATGCAAAGACTGCGAGGAGAGGAGA
GTAAGTATAAGAATGAGCATTGAACTACAATCAGTTTCTAATCCAGTTCACAGAAAGGACTTAGTTATTC
GTCTGACTGATGACACGGATCCATTTTTTTATATAACCTTGTTATATCTGAGGAAGATTTTCAAAGTTT
AAAATTCCAGCAAGGTCTTCTGGTAGACTTCTTAGCTTTCCCACAAAAATTTATAGATCTCCTTCAGCAA
TGTACTCAAGAACATGCCAAAGAAATTCCAAGATTTTTGCTACAGTTAGTTTCTCCAGCAGCTATTTTGG
ATAACTCACCCGCATTTTTAAATGTGGTAGAGACAAATCCTTTTAAGCATCTTACTCACCTCTCACTCAA
ACTTTTACCTGGAAATGATGTGGAGATAAAGAAATTTCTAGCAGGCTGTTTGAAATGTAGCAAGGAAGAA
AAATTATCATTGATGCAATCACTAGATGATGCTACTAGGCAACTGGACTTTACACAAAAGACATTAGCAG
AAAAAAAACAAGAATTAGATAAGTTACGGAATGAATGGGCATCACATACAGCAGCCTTGACAAACAAGCA

TTCTCAGGAACTGACAAATGAAAAGGAAAAAGCCTTGCAGGCACAGGTTCAATATCAACGGCAGCATGAA
CAACAGAAAAAAGATTTAGAAATCCTCCATCAACAAAATATCCACCAGCTACAAAACAGACTGTCTGAGT
TAGAAGCAGCTAATAAAGACTTAACTGAAAGAAAATATAAAGGAGACTCCACTATTAGAGAACTTAAAGC
AAAACTTTCTGGTGTTGAAGAGGAGCTGCAGCGGACTAAGCAAGAAGTCCTCTCTTTGCGAAGAGAGAAT
TCTACACTAGATGCTGAATGCCACGAGAAAGAAAAGCATATTAATCAGCTACAAACAAAAGTGGCAGTTT
TAGAACAGGAAATCAAGGATAAGGACCAGCTTGTTTTAAGAACAAAAGAAGCATTTGATACAATCCAGGA
ACAAAAGGTGGTTTTAGAAGAAAATGGTGAGAAAAATCAAGTACAACTAGGAAAGCTTGAAGCTACAATA
AAATCATTATCTGCAGAACTTCTAAAGGCAAATGAAATTATCAAGAAGTTACAGGGGGATCTGAAAACTT
TAATGGGTAAGTTGAAATTGAAGAATACAGTTACTATTCAGCAAGAAAAACTCCTGGCTGAGAAGGAGGA
AAAATTACAAAAGGAACAAAAGGAATTACAAGATGTTGAACAGTCTCTTCGAATTAAAGAGCAAGAGGTA
TGCAAATTACAAGAACAATTAGAAGCTACAGTTAAAAAACTTGAAGAAAGCAAACAACTTCTAAAAAATA
ATGAAAAGTTAATCACGTGGTTAAATAAAGAACTAAATGAAAATCAGCTAGTGAGAAAGCAAGATGTATT
GGGACCTTCTACTACTACGCCTGCACATTCTAGCAGCAACACAATCAGAAGTGGAATTTCTCCTAACCTG
AATGTGGTTGATGGTAGACTGACTTACCCAACCTGTGGGATTGGTTATCCTGTCTCCTCTGCATTTGCAT
TCCAGAATACCTTCCCTCATTCGATATCTGCCAAAAATACCAGCCACCCTGGTTCAGGAACAAAGGTTCA
GTTTAATTTGCAGTTTACAAAACCAAATGCATCACTAGGAGACGTTCAGTCAGGAGCAACTATTAGTATG
CCTTGCTCAACTGATAAGGAAAATAGTGAAAATTTAGGGTTGGAATCCAAATACCTGAAGAAAAGGGAAG
ATAGCATTCCTTTACGCGGACTCAGCCAGAACCTATTTAGTAGTTCAGACCATCAGAGAGATGGCTCTTT
AGGAGCATTACAGGTGTCTTCCAAACCCACAGTGCTCCCCTCGGCATCTTCAGCATATTTCCCTGGGCAG
CTACCAAACAGT

>Rhinopithecus roxellana XM_010377227.1
ATGAGCCAAGTTCTGTTCCACCAACTAGTCCCGTTGCAGGTGAAATGCAAAGACTGCGAGGAGAGGAGAGTA
AG
TATAAGAATGAGCATTGAACTACAATCAGTTTCTAATCCAGTTCACAGAAAGGACTTAGTTATTCGTCTG
ACTGATGACATGGATCCATTTTTTTATATAACCTTGTTATATCTGAGGAAGATTTTCAAAGTTTAAAAT
TCCAGCAAGGTCTTCTGGTAGACTTCTTAGCTTTCCCACAAAAATTTATAGATCTCCTTCAGCAGTGTAC
TCAAGAACATGCCAAAGAAATTCCAAGGTTTTTACTACAGTTAGTTTCTCCAGCAGCTATGTTGGATAAC
TCACCTGCATTTTTAAATGTGGTAGAGACAAATCCTTTTAAGCATCTTACCCACCTCTCACTAAAACTTT
TACCTGGAAATGATGTGGAGATAAAGAAATTTCTAGCAGGCTGTCTGAAATGTAGCAAGGAAGAAAAATT
ATCATTGATGCAATCACTAGATGATGTTACTAGGCAACTGGACTTTACACAAAAGACATTAGCAGAAAAA
AACCAAGAATTAGATAAGTTACGGAATGAATGGGCGTCACACACAGCAGCCTTGACAAACAAGCATTCTC
AGGAACTGACAAATGAAAAGGAAAAAGCCTTGCAGGTACAAGTTCAATATCAACGGCAGCATGAACAACA
GAAAAAAGATTTAGAAATCCTCCATCAACAAAATATCCACCAGCTACAAAACAGACTGTCTGAGTTAGAA
GCAGCTAATAAAGACTTAACTGAAAGAAAATATAAAGGAGACTCCACTATTAGAGAACTTAAAGCAAAAC
TTTCTAGTGTTGAAGAGGAGCTGCAGCGGACTAAGCAAGAAGTCCTCTCTTTGCGAAGAGAGAATTCTAC
ACTAGATGCTGAATGCCACGAGAAAGAAAAGCATATTAATCAGCTACAAACAAAAGTGGCAGTTTTAGAA
CAGGAAATCAAGGATAAGGACCAGCTTGTTTTAAGAACAAAAGAAGCATTTGATACAATCCAGGAACAAA
AGGTGGTTTTAGAAGAAAATGGTGAGAAAAATCAAGTACAACTAGGAAAGCTTGAAGCTACAATAAAATC
ATTATCTGCAGAACTTCTAAAGGCAAATGAAATTATCAAGAAGTTACAGGGGGATCTGAAAACCTTAATG
GGTAAGTTGAAATTGAAGAATACAGTTACTATTCAGCAAGAAAAACTCCTGGCTGAGAAGGAGGAAAAAT
TACAAAAGGAACAAAAGGAATTACAAGATGTTGAACAGTCTCTTCGAATTAAAGAGCAAGAGGTATGCAA
ATTACAAGAACAATTAGAAGCTACAGTTAAAAAACTTGAAGAAAGCAAACAACTTCTAAAAAATAATGAA
AAATTAATCACATGGTTAAATAAAGAACTAAATGAAAATCAGCTAGTGAGAAAGCAAGATGTATTGGGAC
CTTCTACTACTCCGCCTGCACATTCTAGCAGCAACACAATCAGAAGTGGAATTTCTCCTAACCTGAATGT
GGTTGATGGTAGACTGACTTACCCAACCTGTGGGATTGGTTATCCTGTCTCCTCTGCATTTGCATTCCAG
AATACCTTCCCTCATTCGCTATCTGCCAAAAATACCAGCCACCCTGGTTCAGGAACAAAGGTTCAGTTTA

ATTTGCAGTTTACAAAACCAAATGCATCACTAGGAGACGTTCAGTCAGGAGCAACTATTAGTATGCCTTG
CTCAACTGATAAGGAAAATGGTGAAAATTTAGGGTTGGAATCCAAATACCTGAAGAAAAGGGAAGATAGC
ATTCCTTTACGCGGACTGAGCCAGAACCTATTTAGTAATTCAGACCATCAGAGAGATGGCACTTTAGGAG
CATTACAGGCATCTTCCAAACCCACAGTGCTCCCCTCGGCATCTTCAGCATATTTCCCTGGGCAGCTACC
AAACAGT
>Aotus nancymaae XM_012458448.1
ATGAGCCAAGTTCTCTTCCACCAACTAGTCCCGTTGCAGGTGAAATGCAAAGACTGCGAGGAGAGGAGAGTA
AGTATAAGAATGACCATTGAACTACAATCAGTTTCTAATCCAGTTCACAGAAAGGACTTAGTTATTCGTC
TGACTGATGACACGGATCCATTTTTTTATATAACCTTGTTATATCTGAGGAAGATTTTCAAAGTTTAAA
ATTCCAGCAAGGTCTTCTGGTAGACTTCTTAGCTTTCCCACAAAAATTTATAGATCTCCTTCAGCAATGT
ACTCAAGAACATGCCAAGGAAATTCCAAGGTTTTTGCTACAGTTAGTTTCTCCAGCAGCTATTTTGGATA
ACTCACCTGCATTTTTAAATGTGGTAGAGACAAATCCTTTTAAGCATCTTACACACCTCTCACTAAAACT
TTTACCTGGAAATGATGTGGAGATAAAGAAATTTCTAGCAGGCTGTTTGAAATGTAGCAAGGAAGAAAAA
TTATCACTGATGCAATCACTAGATGATGTTACTAAGCAACTGGACTTTACACAAAAGACATTAACAGAAA
AAAAACAAGAATTGGATAAGTTACGGAATGAATGGGCATCACACACAGCAACCTTGACAAACAAGCATTC
TCAGGAACTGACAAATGAAAAGGAAAAAGCCTTGCAGGCACAGGTTCAGTATCAACAGCAGCATGAACAA
CAGAAAAAAGATTTAGAAATCCTCCATCATCAAAACATCCAGCAGCTGCAACACAGACTATCTGATTTAG
AAGCAGCTAATAAAGACCTAACTGAAAGAAAATATAAAGGAGACTCCACTATTAGAGAACTTAAAGCAAA
ACTTTCTGGTGTTGAAGAGGATCTACAGCGGACTAAGCAAGAGGTCCTCTCTTTGCGAAGAGAGAATTCT
ACATTAGATGCTGAATGCCATGAGAAAGAAAAGCATATTAATCAGCTACAAACAAAAGTAGCAGTTTTAG
AACAGGAAATCAAGGATAAGGACCAGCTTGTTTTAAGAACAAAAGAAGCATTTGATACAATCCAGGAACA
AAAGGTGGTTTTAGAAGAAAGTGGTGAGAAAAATCAAGTACAACTAGGAAAGCTTGAAGCTACAATAAAA
TCATTATCTGCAGAACTTCTGAAGGCAAATGAAATTATCAAGAAGTTACAAGGGGATCTGAAAACTTTAA
TGGGTAAGTTGAAATTGAAGAATACAGTTACTATTCAGCAAGAAAAACTCTTGGCTGAGAAGGAGGAAAA
ATTACAAAAGGAGCAAAAGGAATTACAAGATGTCGGGCAGTCTCTTCGAATTAAAGAGCAAGAGGTATGC
AAATTACAAGAACAGTTAGAAGCTACAGTTAAAAAACTTGAAGAAAGCAAACAACTTCTAAAAAAATAATG
AAAAGTTAATCACATGGTTAAATAAAGAACTAAATGAAAATCAGCTAGTGAGAAAGCAAGATGTATTGGG
ACCTTCTTCTACTCCACCTGCCTATTCTAGCAGCAATGCAATCAGAAGTGGAATATCTCCTAACCTGAAT
ATGGCTGATGGTAGACTGACTTACCCAACTTGTGGGATTGGTTATCCTGTCTCCTCTGCATTTGCATTCC
AGAATACCTTCCCTCATTCGATATCTGCCAAAAATACCAGCCACCCTGGTTCAGGAACAAAGGTTCAGTT
TAATTTGCAATTTACAAAACCAAAGGCATCACTAGGAGATGTTCAGTCAGAAGCAGTTATTAGTATGCCT
TGCTCAACTGATAAGGAAAATAGTGAAAATTTAGGGTTGGAATCCAAATACCTGAAGAAAAGGGAAGATA
GCATTCCTTTACGAGGACTCAGTCAGAACCTATTTAATAATTCAGACCATCAGAAAGATGGCACGTTAGG
AGCATTACAGACATCTTCCAAACCCACAGTGCTCTCCTCTGCATCTTCAGCATATTTCCCTGGGCAGTTA
CCCAACAGT
>Saimiri boliviensis boliviensis XM_003933261.2
ATGAGCCAAGTTCTGTTCCACCAGCTAGTCCCGTTGCAGGTGAAATGCAAAGACTGCGAG
GAGAGGAGAGTAAGTATAAGAATGACCATTGAACTACAATCAGTTTCTAATCCAGTTCACAGAAAGGACT
TAGTTATTCGTCTGACTGATGACATGGATCCATTTTTTTATATAACCTTGTTATATCTGAGGAAGATTT
TCAAAGTTTAAAATTCCAGCAAGGTCTTCTGGTAGACTTCTTAGCTTTCCCACAAAAATTTATAGATCTC
CTTCAGCAATGTACTCAAGAACATGCCAAAGAAATTCCAAGGTTTTTGCTACAGTTAGTTTCTCCAGCAG
CTATTTTGGATAACTCACCGGCATTTTTAAATGTGGTAGAGACAAATCCTTTTAAGCATCTTACACACCT
CTCACTAAAACTTTTACCTGGAAATGATGTGGAGATAAAGAAATTTCTAGCAGGCTGTTTGAAATGTAGC
AAGGAAGAAAAATTATCACTGATGCAATCACTAGATGATGTTACTAGGCAACTGGACTTTACACAGCAGA
CATTAATAGAAAAAAAACAAGAATTGGATAAGTTACGGAATGAATGGGCATCACACACAGCAGCCTTGAC
AAACAAGCATTCTCAGGAACTGACAAATGAAAAGGAAAAAGCCTTGCAGGCACAGGTTCAGTATCAACAG
CAGCATGAACAACAGAAAAAAGATTTAGAAACCCTCCATCAGCAAAACATCCAGCAGCTGCAACACAGAC

TATCTGATTTAGAAGCAGCTAATAAAGACCTAACTGAAAGAAAATATAAAGGAGACTCCACTATTAGGGA
ACTTAAAGCAAAACTTTCTGGTGTTGAAGAGGACCTGCAGCGGACTAAGCAAGAGGTCCTCTCTTTGCGA
AGAGAGAATTCTACATTAGATGCTGAATGCCATGAGAAAGAAAAGCATATTAATCAGCTACAAACAAAAG
TAGCAGTTTTAGAACAGGAAATCAAGGATAAGGACCAGCTTGTTTTAAGAACAAAAGAAGCATTTGATAC
AATCCAGGAACAAAAGGTGGTTTTAGAAGAAAATGGTGAGAAAAATCAAGTACAACTAGGAAAGCTTGAA
GCTACAATAAAATCATTATCTGCAGAACTTCTGAAGGCAAATGAAATTATCAAGAAATTACAAGGGGATC
TGAAAACTTTAATGGGTAAGTTGAAATTGAAGAATACAGTTACTATTCAGCAAGAAAAACTCTTGGCTGA
GAAGGAGGAAAAATTACAAAAGGAGCAAAAGGAGTTACAAGATGTCGGGCAGTCTCTTCGAATTAAAGAG
CAAGAGGTATGCAAATTACAAGAACAGTTAGAAGCTACAGTTAAAAAACTTGAAGAAAGCAAACAACTTC
TAAAAAATAATGAAAAGTTAATCACATGGTTAAATAAAGAACTAAATGAAAATCAGCTAGTGAGAAAGCA
AGATGTATTGGGACCTTCTACTACTACGCCTGCCCATTCTAGCAGCAATGCAATCAGAAGTGGAATTTCT
CCTAACCTGAATATGGTTGATGGTAGACTGACTTACCCAACTTGTGGGATTGGTTATCCTGTCTCCTCTG
CATTTGCATTCCAGAAAACCTTCCCTCATTCGATATCAGCCAAAAATACCAGCCACCCTGGTTCAGGAAC
AAAGGTTCAGTTTAATATGCAATTTACAAAACCAAATGCATTGCTAGGAGATGTTCAGTCAGAAGCAGTT
ATTAGTATGCCTTGCTCAACTGATAAGGAAAATAGTGAAAATTTGGGGTTGGAATCCAAATACCTGAAGA
AAAGGGAAGATAGCATTCCTTTACGAGGACTCAGTCAGAACCTATTTAATAATTCAGACCAACAGAAAGA
TGGCACATTAGGAGCATTACAGACATCTTCCAAACCCACTGTGCTCTCCTCTGCATCTTCAGCATATTTC
CCTGGGCAGTTACCAAACAGT
>Propithecus coquereli XM_012654907.1
ATGAGCCAAGTTCTACTCCAGCAACTAGTCCCGTTGCAGGTGAAATGCAAAGACTGCGAAGAGAGGAGA
GTAAGTGTTAGAGTGAGCGTTGAACTACAATCAGTTTCTAATCCAGTTCACAGAAAGGACTTAGTTATCC
GTCTGACTGATGACATGGATCCATTTTTTCTATATAACCTTGTTATATCTGAGGAAGATTTTCAAAGCTT
AAAATTCCAGCAAGGTCTTCTGGTAGACTTCTTAGCTTTCCCACAAAAATTTATAGATCTCCTTCAGCAG
TGTACTCAAGAACACACCAAAGAAATTCCAAGGTTTTTACTACAGTTAGTTTCTCCAGCAGCTATTTTGG
ATAACTCACCTGTATTTTTAAATGTGGTAGAAACAAATCCTTTTAAGCATCTTACACACCTCTCACTAAA
ACTTTTACCTGGAAATGATGTGGAGATAAAGAAATTTCTAGCAGGCTGTTTGAAATGTAGCAAGGAAGAA
AAATTGTCATTGACACAATCATTAGATGATGTCACTAGGCAACTGAACTTCACACAAAAGACATTATCAG
AAAAAATCCAAGAATTAGATAAGTTACGGAACGAATGGGCATCACACACAGCAGCATTGACAAATAAGCA
CTCTCAGGAACTAACAAACGAGAAGGAAAAAGCCTTGCAGGCACAGGTTCAATATCAACAGCAGCATGAA
CAACAGAAAAAAGATTTAGAAATTCTCCATCAACAAAACATCCACCAGCTACAAAACAGAGTGTCTGAGT
TAGAAGCAACTAATAAAGACCTAACCGAAAGAAAATATAAAGGAGACTCCACTATTAGAGAACTTAAAGC
AAAACTTTCTGGCGTCGAAGAGGAGTTGCAGCGAACTAAGCAAGAAGTCCTCTCTTTGCGAAGAGAAAAT
TCTACACTAGATGCCGAATGCCACGAGAAAGAAAAGCATATTAATCAGCTACAAACAAAAGTGGCAGTTT
TAGAACAGGAAATCAAGGATAAGGACCAGCTTGTTTTAAGAACAAAAGAAGCATTTGATACAATCCAGGA
GCAGAAGGTGGCCTTAGAAGAAAATGGGGAGAAAAATCAAGTACAGCTAGGAAAACTTGAAGCTACAATA
AAATCATTATCAGCAGAACTTCTGAAGGCAAATGAAATTATCAAGAAGTTACAAGGGGATCTGAAAACTC
TAATGGGTAAGTTGAAACTGAAGAATACAGTTACTATTCAGCAGGAAAAACTCTTGGCTGAGAAGGAGGA
AAAATTACAAAAGGAGCAAAAGGAATTACAAGATATTGGACAGTCTCTTCGAATTAAAGAGCAAGAGGTA
TGTAAGTTACAAGAACAATTAGAAGCTACAGTTCAAAAACTTGAAGAAAGCAAACAGCTTTTGAAAAATA
ATGAAAAGTTAATCACATGGTTAAATAAAGAACTAAATGAAAATCAGCTAGTGAGAAAGCAAGATGTATT
GGGACCTTCTACTACCCCACCTGCACATTCTAGCAGCAACACAATCAGAAGTGGAATTTCTCCTAACTTG
AATGTGGTTGATGGTAGACTAACTTACCCAACTTGTGGGATTGGCTATCCTGTCTCCTCTGCATTTGCAT
TCCAAAATACCTTTCCTCATTCGATATCTGCCAAAAATACCATCCACCCGGTTTCAGGACCAAAGGTTCA
GTTTAACTTGCAATTTACAAAACCAAATACATCGCTAGGAGATGTTCAGTCAGGAGCAACTATTAGTATG
CCTTGCTCAGCTGATAAGGAAAATGGTGAAAATTTAGGGCTGGAATCCAAATACCTGAAGAAAAGGGAAG
ATAGCATTCCTTTACGTGGACTCAGCCAGAATCTATTTAGTAATTCAGACCATCAGAAAGATGGCCCTCT
AGGAGCATTACAGACATCTTCCAAACCCACAGTGCTCCCTTCTACATCTTCAGCATATTTCCCTGGGCAG

TTACCAAACAGT
>Cebus capucinus imitator XM_017547614.1
ATGAGCCAAGTTCTGTTCCACCAACTAGTCCCGTTGCAGGTGAAATGCAAAGACTGCGAGGAGAGGAGAGTA
AG
TATAAGAATGACCATTGAACTACAATCAGTTTCTAATCCAGTTCACAGAAAGGACTTAGTTATTCGTCTG
ACTGATGACACGGATCCATTTTTTTATATAACCTTGTTATATCTGAGGAAGATTTTCAAAGTTTAAAAT
TCCAGCAAGGTCTTCTGGTAGACTTCTTAGCTTTCCCACAAAAATTTATAGATCTCCTTCAGCAGTGTAC
TCAAGAACATGCCAAAGAAATTCCAAGGTTTTTGCTACAGTTAGTTTCTCCAGCAGCTATTTTGGATAAC
TCACCTGCATTTTTAAATGTGGTAGAGACAAATCCTTTTAAGCATCTTACACACCTCTCACTAAAACTTT
TACCTGGAAATGATGTGGAGATAAAGAAATTTCTAGCAGGCTGTTTGAAATGTAGCAAGGAAGAAAAATT
ATCATTGACGCAATCACTAGATGATGTTACTAGGCAACTGGACTTTACACAAAAGATATTAACAGAAAAA
AACCAAGAATTGGATAAGTTACGGAATGAATGGGCATCACACACAGCAGCCTTGACAAACAAGCATTCTC
AGGAACTGACAAATGAAAAGGAAAAAGCCTTGCAGGCACAGGCTCAGTATCAACAGCAGCATGAACAACA
GAAAAAAGATTTAGAAATCCTCCATCAACAAAACATCCAGCAGCTGCAACACAGACTATCTGATTTAGAA
GCAGCTAATAAAGACCTAACTGAAAGAAAATATAAAGGAGACTCCACTATTAGAGAACTTAAAGCAAAAC
TTTCTGGTGTTGAAGAGGAGCTGCAGCGGACTAAGCAAGAGGTTCTCTCTTTGCGAAGAGAGAATTCTAC
ATTAGATGCTGAATGCCATGAGAAAGAAAAGCATATTAATCAGCTACAAACAAAAGTAGCAGTTTTAGAA
CAGGAAATCAAGGATAAGGACCAGCTTGTTTTAAGAACAAAAGAAGCATTTGATACAATCCAGGAACAAA
AGGTGGTTTTAGAAGAAAATGGTGAGAAAAATCAAGTACAACTAGGAAAGCTTGAAGCTACAATAAAATC
ATTATCTGCAGAACTTCTGAAGGCAAATGAAATTATCAAGAAGTTACAAGGGGATCTGAAAACTTTAATG
GGTAAGTTGAAATTGAAGAATACAGTTACTATTCAGCAAGAAAAGCTCTTGGCTGAGAAGGAGGAAAAAT
TACAAAAGGAGCAAAAGGAATTACAAGATGTCGGGCAGTCTCTTCGAATTAAAGAGCAAGAGGTATGCAA
ATTACAAGAACAGTTAGAAGCTACAGTTAAAAAACTTGAAGAAAGCAAACAACTTCTAAAAAATAATGAA
AAGTTAATCACATGGTTAAATAAAGAACTAAACGAAAATCAGCTAGTGAGAAAGCAAGATGTATTGGGAC
CTTCTACTACTCCACTTGCCCATTCTAGCAGCAATGCAATCAGAAGTGGAATTTCTCCTAACCTGAATAT
GGTTGATGGTAGACTGACTTACCCAACCTGTGGGATTGGTTATCCTGTCTCCTCTGCATATGCATTCCAG
AAAACCTTCCCTCATTCGATATCTGCCAAAAATACCAGCCACCCTGGTTCAGGAACAAAGGTTCAGTTTA
ATTTGCAATTTACAAAACCAAACGCATCGCTAGGAGATGTTCAGTCAGAAGCAGTTATTAGTATGCCTTG
CTCAACTGATAAGGAAAATAGTGAAAATTTAGGGTTGGAATCCAAATACCTGAAGAAAAGAGAAGATAGC
ATTCCTTTACGAGGACTCAGTCAGAACCTATTTAATAATTCAGACCATCAGAAAGATGGCACGTTAGGAG
CATTACAGACATCTTCCAAACCCACAGTGCTCTCCTCTGCATCTTCAGCATATTTCCCTGGGCAGTTACC
AAACAGT
>Galeopterus variegatus XM_008579569.1
ATGAGCGAAGTTCTGTTCCAGCAACTAGTCCCGTTGCAGGTGAAATGCAAAGACTGCGAGGAGAGGAG
AGTAAGTGTTAGAGTGAGCATTGAACTACAATCAGTTTCTAATCCAGTTCACAGAAAGGATTTAGTTATC
CGTCTGACTGATGATACGGATCCATTTTTCCTGTATAACCTTGTTATATCTGAGGAAGATTTTCAAAGTT
TAAAATTCCAGCAAGGTCTTCTGGTAGACTTCTTAGCTTTTCCACAAAAATTTATAGATCTCCTTCAGCA
ATGTACTCAAGAACATGCCAAAGAAATTCCAAGGTTTTTGCTACAGTTAGTTTCTCCAGCAGCTATTTTG
GATAACTCACCTGCATTTTTAAATGTGATAGAGACAAATCCTTTTAAGCATCTTACACACCTTTCACTAA
AACTTTTACCTGGGAGTGATGTGGAGATAAAGAAATTTCTAGCAGGCTGTTTAAAATGTAGCAAGGAAGA
AAAATTATCTTTGACGCATTCACTAGATGAGGTTACTAGGCAACTGAACTTCACACAAAAGACATTATCA
GAAAAGATCCAAGAATTAGATAAGTTACGGAATGAATGGGCATCACACACAGCAGCACTGACAAATAAGC
ATTCTCAGGAACTGACAAATGAGAAGGAAAAAGCCTTGCAGGCACAAGTTCAATATCAACAGCAGCATGA
ACAACAGAAAAAAGATTTAGAAGTCGTCCATCAACAAAATATTCACCAGCTACAAAACAGGTTGTCTGAA
TTAGAAGCAGCTAATAAAGACCTAACTGAAAGGAAATATAAAGGAGACTCCACTATTAGAGAACTTAAAG
CAAAACTTTCTGGTGTCGAAGAGGAGCTACAGCGGACTAAGCAAGAAGTACTCTCTTTGCGAAGAGAGAA
TTCTACACTAGATGCTGAATGCCATGAGAAAGAAAAGCATATTAATCAGCTACAGACAAAAGTGGCAGTT

TTAGAACAGGAAATCAAAGATAAGGATCAGCTTGTTTTAAGAACAAAAGAAGCATTTGATACAATCCAGG
AGCAAAAGGTGGTTTTAGAAGAAAATGGTGAGAAAAATCAGGTACAACTAGGAAAACTTGAAGCTACAAT
AAAATCATTATCAGCAGAACTTCTGAAGGCAAATGAAATTATAAAGAAGTTACAAGGGGATCTGAAAACT
CTAATGGGTAAATTGAAACTGAAGAATACAGTTACTGTTCAGCAAGAAAAACTCTTGGCTGAGAAGGAGG
AAAAATTACAAAAGGAACAAAAGGAATTACAAGATGTTGGACAGTCTCTCCGAATTAAAGAGCAAGAGGT
ATGCAAATTACAAGAACAATTAGAAGCTACAGTTCAAAAACTTGAAGAAAGCAAACAGCTTCTAAAAAAT
AATGAAAAGTTAATCACATGGTTAAATAAAGAGCTAAATGAAAATCAGCTAGTGAGAAAGCAAGATGTAT
TGGGACCTTCTACCACTCCACCTGCACATTCTAGCAGCAATACAATCAGAAGTGGAATTTCTCCTAACCT
GAATGTGATTGATGGTAGACTGACTTACCCAACTTGTGGGATTGGTTATCCTGTCTCCTCTGCGTTTACG
TACCAGAACACCTTTCCTCATCCAATGTCTTCCAAAAATAACATCCAACCGGTTTCAGGACCAAAGGTTC
AGTTTAATTTGCAGTTTACAAAACCAAATACACTGCTAGGAGATGTCCAGTCAGGAGCAACTATGAGTAT
ACCTTGCTCAAATGATAAGGAAAATGGTGAAAATTTAGGGCTGGAATCCAAATACCTGAAGAAAAGGGAA
GATAGTATTCCTTTACGTGGTCTCAGCCAGAATCTATTTAGTAACTCAGACTACCAGAAAGATGGCACTC
TCGGAGCATTACAAACTTCCAAACCCACAGTGCACCCTGCTACATCTTCAGCATATTTCCCTGGGCAGTT
ACCAAACAGT
>Ictidomys tridecemlineatus XM_005339681.2
ATGAGCCAAGTTCTGTTCCAGCAACTAGTTCCGCTGCAGATGAAATGCAAAGACTGCGAGGAAAGGAGAATA
AAT
GTTAGAGTAAGCATTGAACTGCAATCAGTTTCTAATCCAGTTCATAGAAAGGATTTGGTTATTCGTCTGA
CTGATGATACAGATCCATTTTTTCTGTATAATCTTATTATATCTGAGGAAGATTTTCAAAGTTTAAAATT
CCAGCAAGGTCTTCTGGTAGACTTCTTAGCTTTCCCACAAAAATTTATAGATCTCCTTCAACAGTGTACT
CAAGAACATGTTAAAGAAATTCCAAGGTTTTTGCTACAGTTAGTTTCTCCAGCAGCTATTTTGGATAATT
CACCTGCATTTTTAAATGTGATAGAGACAAATCCTTTTAAGCATCTTACACATCTCTCACTAAAACTCTT
ACCTGGAAATGATGTGGAAATAAAGAAATTCCTAGCAGGTTGTTTGAAATGTAGTAAGGAAGAAATATTA
TCATTGACACAATCACTAGATGATGTTACTAGACAACTGAACTTGACACAAAAGACATTATCAGAAAAAA
TACAAGAATTAGAAAAGTTACGGAATGAATGGGAATCACACACAGCATCATTGACAAATAAGCATTCTCA
GGAACTGACGAATGAGAAGGAAAAGGCTTTACAGGTACAAGTTCAATATCAACAGCAGCATGAACAACAG
AAAAAAGATTTAGAAATCCTCCATCAACGAAATATCCACCAGCTACAGAACAGATTGTCAGAGTTAGAGG
CACTTAATAAAGACCTAACTGAAAGGAAATATAAAGGAGACTCCAGTATTAGAGAACTTAAAGCAAAACT
TTCTGGTGTTGAAGAGGAGCTGCAACGAACTAAGCAAGAAGTCCTCTCTTTGCAAAGAGAGAATTCTACG
TTAGATGCTGAATGCCATGAGAAAGAAAAGCATATTAATCAGCTACAAACAAAAGTGGCAGTTTTAGAAC
AAGAAATCAAGGATAAAGACCAACTTGTTTTAAGAACAAAAGAAGCATTTGATACAATCCAGGAACAAAA
GGTGGCTTTAGAAGAAAGTGGTGAGAAGAATCAGGTACAACTGGGAAAACTTGAAACTACGATAAAATCA
TTATCAGCAGAACTTCTGAAGGCAAATGAAATTATTAAGAAGTTACAAGGAGATTTGAAAACCCTAATGG
GCAAATTGAAACTGAAGAATACAGTTACTGTTCAGCAAGAAAAACTCTTGGCTGAGAAGGAGGAAAAATT
ACAAAAGGAACAAAAGGAACTACAAGATGTTGGACAGTCTCTTCGAGTTAAGGAGCAAGAGGTATGCAAA
TTACAAGAACAGTTAGAAGCTACAGTTCAAAAACTTGAGGAAAGCAAACAACTTCTAAAAAATAATGAAA
AGTTAATCACATGGTTAAATAAAGAACTAAATGAAAATCAGCTAGTGAGAAAGCAAGATGTGTTGGGACC
TTCTACCACTCCTCCTGCACATTCTAGCAACAACACAATCAGAAGTGGAATTTCTCCTAACCTGAATGTG
GTTGATAGTAGGCTGACTTACCCAAGCTGTGGGATTCCTTATCCTGTCTCCTCTGCATTTGCATTCCAGA
ATACTTTTCCTCATCTGTTATCTGCCAAAAATACCATCCACCCAGTTTCAGGACCGAAGGTTCAGTTTAA
CTTGCAGTTTACAAAACCAAATATATCACTAGGAGATGTTCAGTCAGGAGCAACCATTAATGTGCCTTGC
TCAACTGATAAGGAAAATGGTGAAAATTTAGGATTGGAATCTAAATACTTGAAGAAAAGAGAAGATAGCA
TTCCTTTACGTGGACTGAGCCAGAATCTGTTTAGTAATTCAGATCATCAGAAAGATAGCAATCTAGGAGT
GATGCAGACATCTTCCAAACCCACAGTGCTCCCCTCTACATCTTCGGCCTATTTTCCTGGGCAGTTACCA
AACAGT
>mouse ENSMUST00000198311.4

ATGAGTCAAGTTCTGTTCCAGCAGCTTGTTCCCTTGCTAGTGAAATGCAAAGACTGCGAG
GAGAGGAGAGGGAGCGTTAGAGTGAGCATTGAGCTGCAGTCCCTTTCGAATCCAGTACAC
AGGAAGGATTTAGTCATCCGTCTGACTGATGATACAGATCCGTTTTTTCTGTATAACCTT
GTTATATCTGAGGAAGATTTTCAAAGTTTAAAATTGCAGCAAGGTCTCCTGGTGGACTTC
TTAGCTTTCCCACAGAAGTTTATAGACCTCCTTCAGCAGTGTATGCAAGAACACGCGAAA
GAGACTCCAAGGTTCCTGCTGCAGCTTCTTTCTTCAGCCACTCTTTTGGAGAACTCACCG
GTCCTTTTAAACGTAGTGGAGACAAATCCTTTTAAGCATCTTATTCACCTGTCACTAAAG
CTTTTACCTGGAAATGATGTAGAAATAAAGAAATTTCTAGCAGGATGTTTGAAATGTAGC
AAGGAAGAAAAATTATCACTGACTAGATCACTAGATGATGTTACCAGGCAACTGCACATT
ACACAAGAGACGTTGTCGGAAAAAATGCAAGAATTAGATAAGCTGCGGAGTGAATGGGCC
TCGCACACAGCATCGCTGACGAATAAGCACTCTCAGGAGTTAACAGCTGAGAAGGAGAAG
GCCTTGCAGACCCAAGTTCAGTGCCAACAGCAGCACGAGCAACAGAAAAAGGAACTGGAA
ACCCTCCATCAACGGAATATCCACCAGCTACAAAGCAGATTGTCCGAGTTAGAGGCAGCT
AATAAAGAGCTCACCGAGAGGAAGTATAAAGGAGACTCGACTGTCCGAGAGCTAAAGGCG
AAGCTGGCTGGCGTGGAAGAGGAGCTGCAGCGGGCCAAGCAAGAGGTCCTTTCTCTGAGA
AGAGAGAATTGTACTCTGGATACTGAATGCCACGAGAAAGAAAAGCACATCAACCAGCTA
CAAACAAAAGTGGCCGTTTTAGAACAGGAGATCAAAGATAAAGACCAGCTTGTTCTGAGA
ACAAAAGAAGCATTCGATACAATCCAAGAGCAAAAGGTGGCTTTAGAAGAAAATGGTGAG
AAAAATCAGATACAACTGGGAAAACTTGAAGCTACAATAAAATCATTATCAGCAGAACTT
CTAAAGGCAAATGAGATCATCAAGAAGTTACAAGGAGATCTTAAAACTCTGATGGGTAAA
TTGAAACTGAAGAATACAGTTACTATTCAGCAAGAAAAACTATTGGCTGAGAAAGAAGAA
ATGCTACAAAAGGAGCGAAAGGAATCACAGGACGCTGGGCAGTTTCTTCGTGCCAAAGAG
CAAGAGGTATGCAGATTACAGGAACAATTAGAAACTACAGTTCAGAAACTCGAAGAAAGT
AAACAACTTTTGAAAAATAATGAAAAATTAATCACGTGGCTAAACAAAGAGCTAAATGAA
AATCAGCTGGTAAGAAAACAGGACACATTGGGAACCTCTGCCACCCCACATTCTACTAGC
AACAGCACCATCAGAAGTGGGCTCTCTCCCAATCTGAATGTGGTTGATAGACTAAATTAC
CCAAGCTGTGGAATTGGCTATCCTGTCTCCTCTGCATTGACATTCCAGAATGCTTTTCCT
CATGTAGTAGCTGCCAAAAACACCAGCCACCCTATCTCTGGACCAAAGGTTCACTTTAAC
CTGCAGCTTACAAAACCAAGCGCTTCGATAGATGGGCAGCCGGGAGCCGCTGTTAACAGG
CCTTGTTCAAATGATAAGGAAAATGGTGAAACTTTAGGATTGGAATCCAAATACTTGAAG
AGAAGAGAAGCTAGCATTCCTTTACGCGGACTTAGCCAGAATCTGTTGAGTGACTCAGAC
CATCAGAAAGACGGCATGCTGGGAGCGTTCCAGCTATCTTCCAAACCCACCGTTCTCCCC
TCCTCGTCTTCAGCCTACTTCCCTGGGCAGTTACCAAGTAGT
>rabbit ENSOCUT00000007827.2
ATGAGCCAAGTTCTCTTCCAGCAGCTGGTTCCGTTGCTGGTGAAATGCAAAGACTGCGAG
GAGAGGAGAGTAAGTGTTAGAGTCAGCATTGAACTACAATCAGTTTCTAATCCAGTTCAC
AGAAAGGATTTAGTTATCCGTCTGACTGATGACACGGATCCATTTTTTCTGTATAATCTT
ATTATATCCGAGGAAGATTTTCAAAGTTTAAAATTCCAGCAAGGGCTTCTGGTGGACTTC
TTAGCTTTCCCACAAAAATTTATAGATCTCCTTCAGCAGTGTGCTCAAGAACATGCCAAA
GAAATTCCAAGGTTTTTGCTACAGTTAGTTTCTCCAGCAGCTAATCTGGATAACTCACCT
GTATTTTTAAATGTGGTAGAGACAAATCCTTTTAAGCATCTTACACACCTCTCACTAAAA
CTTTTACCTGGAAATGATGTGGAGATAAAGAAATTTTTAGCAAGCTGTTTAAAATGTAGC
AAGGAAGAAAAAATATCATTGACGCAATCACTAGAGGATGTTACTAGGCAACTGAACTTA
ACGCAAAAGACATTATCAGAAAAAATTCAAGAATTAGATAAGCTACGGAATGAATGGACA
TCACACACAGCAGCGTTGACAAATAAGCATTCTCAGGAACTGACAAATGAGAAGGAAAAA
GCTTTACAGGCACAGGTTCAGTATCAGCAACAGCATGAACAACAGAAAAAGGATTTAGAA
ATCCTCCATCAGCGAAACATCCACCAACTACAGAACAGATTGTCTGAGTTAGAAGCAACT

AACAAAGACCTAACTGAAAGGAAATATAAAGGAGACGCCACTATCAGAGAACTTAAAGCA
AAACTTTCTGGTGTTGAAGAGGAGCTGCAGCGGACTAAGCAAGAAGTTCTCTCTTTGAGA
AGAGAGAATTCTACACTAGATTCTGAATGCCATGAGAAAGAAAAGCATATTAATCAGCTT
CAAACAAAAGTGGCAGTTTTAGAACAGGAAATCAAGGATAAAGACCAGCTTGTTTTAAGA
ACAAAAGAAGCATTTGATACAATCCAGGAACAAAAGGTGGCTTTAGAAGAAAATGGTGAG
AAAAATCAGGTACAGCTGGGAAAACTTGAAGCTACAATAAAATCATTGTCAGCAGAACTT
CTGAAGGCGAATGAAATTATCAAGAAGTTACAAGGGGATCTAAAAACTCTAATGGGTAAG
TTGAAACTGAAGAATACAGTTACTATTCAGCAAGAAAAACTCTTGGCTGAGAAGGAGGAA
AAATTACAAAAGGAACATAAAGAATTAGAAGAGGTTGGACAGTCTCTCCGAGTTAAAGAG
CAAGAGGTATGCAAATTACAAGAACAATTAGAAGCTACAGTTCAAAAACTTGAAGAAAGC
AAACAGCTTTTAAAAAATAATGAAAAATTAATCACATGGTTAAATAAAGAGCTAAATGAA
AATCAGTTAGTGAGAAAGCAAGATGTATTGGGATCTTCTACAACTCCACCTGTACATTCT
AATAGCAGTACAATCAGAAGTGGAATTTCTCCTAACCTGAATGTGGTTGATGGTAGACTT
ACCTACCCAAGCTGTGGGATTGGTTATCCTGTCTCTTCTGCATTTACATTTCAGAATGCC
TTTCCTCATCCAATATCTGCCAAAAATACCATCCACCCAGTTTCAGGACCAAAGGTACAG
TTTAACTTGCAGTTTACCAAACCAAATACATCGCTAGGAGATGTTCAGTCAGGAGCAACC
ATTAGTGTGCCTTGTTCAACTGATAAGGAAAATGGTGAAAATCTAGGGCTGGAATCCAAA
TACTTGAAGAAAAGGGAAGATAGCATTCCTTTACGTGGACTCAGCCAGAATCTATTTAGC
AGTTCAGACCATCAGAAAGATGGCACTCTGGGAGCATTACAGACATCTTCCAAACCTGCA
GTCCTCCCTTCCACGTCTTCAGCCTATTTCCCTGGGCAGATACCCAACAGT
>Rattus norvegicus XM_006233227.2
ATGAGTCAAGTTCTGTTCCAGCAACTTGTTCCCTTGCT
AGTGAAATGCAAAGACTGCGAGGAGAGGAGAGGGAGCGTTAGAGTGAGCATTGAACTACAGTCACTGTCT
AATCCAGTACACAGAAAGGATCTAGTCATCCGTCTGACTGATGATACAGATCCATTTTTTCTGTATAACC
TTGTTATATCCGAGGAAGACTTTCAAAGTTTAAAATTGCAGCAAGGTCTCCTGGTGGACTTCTTATCTTT
TCCACAGAAGTTTATAGACCTCCTTCAGCAATGTATGCAGGAGCATGTGAAAGAGACTCCAAGGTTTCTG
CTGCAGCTCACGTCTTCAGCTGCTCTTTTGGATAACTCGCCGGTGTTTTTAAATGTAGTGGAGACAAATC
CTTTTAAGCATCTTATTCACCTGTCACTAAAGCTTTTACCTGGAAATGATGTAGAAATAAAGAAATTTCT
AGCAGGATGCTTGAAATGTAGCAAGGAAGAAAAATTATCACTGACTAAATCACTAGATGATGTTACCAGG
CAACTGCACATTACACAAGAGACACTCTCGGAAAAGATGCAGGAACTAGATAAGTTGCGGAGTGAGTGGG
CCTCGCACACAGCAGCGCTGACCAATAAGCACTCCCAGGAGTTAACAAACGAGAAGGAGAAAGCCTTGCA
GACACAAGTTCAGTACCAACAGCAGCATGAACAACAGAAAAAGGATCTGGAAGCCCTCCATCAACGAAAT
ATCCACCAGCTACAAAACAGATTGTCTGAGTTAGAGGCAGCTAATAAAGAGCTCACCGAGAGGAAGTATA
AAGGAGACTCCACTGTCAGAGAACTAAAAGCGAAGCTTGCTGGTGTGGAAGAGGAGCTGCAGCGGACCAA
GCAAGAGGTCCTTTCTCTGAGAAGAGAGAATTGTACTCTGGATACTGAATGCCACGAGAAAGAAAAGCAT
ATTAACCAGCTACAAACAAAAGTGGCCGTTTTAGAACAGGAGATCAAGGATAAAGACCAGCTTGTTCTGA
GAACAAAAGAAGCATTCGATACAATCCAAGAGCAAAAGGTGGCTTTAGAAGAAAATGGTGAGAAAAATCA
GATACAATTGGGAAAACTTGAAGCTACAATAAAATCATTATCAGCAGAACTTCTAAAGGCGAATGAGATT
ATCAAGAAGTTACAAGGAGATCTTAAAACTCTGATGGGTAAATTGAAACTGAAGAATACAGTTACTATTC
AGCAAGAAAAGCTATTGGCTGAGAAAGAAGAAATGCTACAAAAGGAGCGAAAGGAATCACAGGATGCTGG
GCAGTCTCTTCGGGCCAAAGAGCAAGAGGTATGCAGATTACAAGAACAATTAGAAACTACAGTTCAGAAA
CTTGAAGAAAGTAAACAACTTTTGAAAAATAATGAAAAATTAATCACATGGCTAAACAAAGAACTAAATG
AAAACCAGCTGGGAAGAAAGCAAGACACACTGGGGGCCTCTGCCACCCCACCCACACATTCTGCTAGCAA
CACCGTCAGAAGTGGGCTTTCTCCCAGCCTGAATGTGGTTGATAGTAGACTAAATTACCCAAGCTGTGGA
ATTGGCTATCCTGTCTCGTCTGCGTTCACATTCCAGAATGCTTTTCCTCATGCAGTAGTTGCCAAAAACA
CCAGCCATCCAGTCTCTGGACCAAAGGTGCAGTTCAGCTTGCAGCTGACAAAGCAAAGCACGTCGTCGGG
AGATGGGCAGCCGGGAGCGGCTGTTAACAGGTCTGCTTCAGCTGACAAGGAGAATGGTGAAAATTTAGGA

TTGGAATCCAAATACTTGAAGAAAAGAGAAGATAGCATTCCTTTACGTGGACTCAGCCAGAATCTGTTTA
GTAATTCAGACCATCAGAAAGATGGCATGCTGGGAGCGCTGCAGACATCTTCCAAACCCACAGTCCTGCC
CCCATCTTCATCGTATTTTCCTGGGCCATTACCAAGTAGT
>Fukomys damarensis XM_010639069.1
ATGAGCCAAGTCCTGTTCCAGCAGCTAATTCCGT
TGCAGGTGAAATGCAGAGACAGCGAGGAGAGGAGAATAAGTGTTAGAGTGAGCATTGAACTACAATCAGT
TTCTAATCCAGTTCACAGAAAGGATTTAGTTATTCGTCTGACTGATGATACAGATCCATTTTTTTTGTAT
AACCTTGTTATATCTGAGGAAGATTTTCAAAGTTTAAAATTCCAACAAGGTCTTCTGGTAGACTTCTTAG
CTTTCCCACAAAAATTTATAGATCTCCTTCAGCAGTGTGCTCAAGAACATGCCAAAGAAATCCCAAGGTT
TTTACTACAGTTAGTTTCTTCAGCAGCTATTTTGGATAACTCACCTGCGTTTTTAAATGTGGTAGAGACA
AATCCTTTTAAGCATCTTACACACCTCTCACTAAAACTTTTACCTGGAAATGATACGGAGATAAAGAAAT
TTCTAGCAGGCTGTTTGAGATGTAGCAAGGAAGAAAAATTATTATTAACACAATCACTAGATGATGTTAC
TAGGCAACTGAACCTCACAAAAAAGACATTATCAGAAAAAAATACAAGAATTAGACAAATTACGGAATGAA
TGGGTATCACACACAGCCTCACTAACAAATAAGCATTCTCAGGAACTGACAAATGAGAAGGAAAAAGCAT
TACAGGCACAGGCTCAGTATCAACAACAGCATGAACAACAGAAAAAAGATTTAGAAACCCTCCATCAGCG
AAACATCCACCAGCTACAAAACAGATTGTCTGAATTGGAAGCAGCTAATAAAGATCTAACTGAGAGGAAA
TATAAAGGAGACTCCACTACTAGAGAACTTAAAGCTAAACTTTCTGGTGTTGAAGAGGAGCTGCAGCGAG
CTAAGCAGGAAGTTCTCTCTTTGAGAAGAGAAAATTCTACTTTAGATGCTGAATGTCATGAGAAAGAAAA
GCATATTAATCAGCTACAAACAAAAGTGGCAGTTTTAGAACAAGAAATTAAGGATAAAGACCAGCTTGTT
GTAAGAACAAAAGAAGCATTTGATACAATCCAGGAACAAAAGGTGGCATTAGAAGAAAATAGTGAGAAAA
ATCAGGTTCAACTGGGAAAACTTGAAGCTACAATAAAATCATTATCAGCAGAACTTCTGAAGGCAAATGA
AATTATCAAGAAGTTACAAGGGGATTTGAAAACTCTGATGGGTAAGTTGAAACTGAAGAATACAGTTACT
ATTCAGCAAGAAAAACTATTGGCTGAGAAGGAGGAAAAATTACAAAAGGAACAAAAGGAATTACAAGATG
TTGGACAGACTCTCAGAGCTAAGGAACAAGAGGTATGCAAATTACAAGGACAATTAGAAGCTACAGTTCA
AAAACTTGAGGAAAGCAAACAGCTTCTAAAAAATAATGAAAAGTTAATCACATGGTTAAATAAAGAACTA
AATGAAAATCACCTAGTGAGAAAGCAAGATGGATTGGGACCTTCTACCACTCCACCTGCACATTCCAGCA
GCAACACAATCAGAAGTGGAATTTCTCCTAACCCGAATGTGATTGATAATAGACTTGCTTACCCAGGCTG
TGGGATTGGTTATCCTGTGTCTTCTGCATTTGCATTCCAGAATACCTTTCCTCATCCAATACCTGCCAAA
AATATCATCCACCCAGTTTCAGGACCAAAGGTTCAGTTTAACTTACAGTTTACAAAACCGAGTACATCAC
TAGGAGATACTCACTCAGGGGCAGCTGTTGGTGTTCCATGCTCCACTGACAAGGAAAATGGAGAAAATTT
AGGGCTGGAATCCAAATACCTGAAGAAAAGAGAAGATAGTATTCCTTTACGTGGTCTCAGCCAGAATCTA
TTTAGTAATGCAGACCATCAAAAAGATGGCACTTTAGGAGCACTGCAGACTTCTTCCAAACCCACAGTGC
TCCCTTCCACATCTTCAGCCTACTTCCCTGGACAGTTGTCAAGCAGT
>Chinchilla lanigera XM_005389097.2
ATGAGCCAGGTCCTGTTCCAGCAGCGGATCCCGCTGCAGGTGAAATGCAAAGAGTG
CGAGGAGAGGAGAATCAGTGTTAGAGTGAGCATTGAACTACAATCAATTTCTAATCCAGTTCACAGAAAG
GATTTAGTTATTCGTCTGACAGATGATACAGATCCATTTTTTTTGTATAACCTTGTTATATCTGAGGAAG
ATTTTCAAAGTTTAAAATTCCAGCAAGGTCTTCTGGTAGACTTCCTAGCTTTCCCACAAAAATTTATAGA
TCTCCTTCAGCAGTGTGCTCAAGAGCATACCAAAGAAATCCCAAGGTTTTTGCTGCAGTTAGTTTCTCCA
GCGGCTGTTCTGGATAACTCACCCGCATTTTTAAATGTGGTGGAGACAAACCCTTTTAAGCATCTTATAC
ATCTCTCACTAAAACTTTTACCTGGAAATGATGTGGAGATAAAGAAATTTCTAGCAGGCTGTTTGAAATG
TACCAAGGAAGAAAAATTATTATTGACACAATCGCTAGATGATGTTACCAGACAGCTGAACCTTACGAAA
AAGACGTTATCGGAAAAAGTTCAAGAATTAGATAAGCTCCGGAATGAATGGGCATCACACACGGCCTCAC
TGACAAGTAAGCATTCTCAGGAACTGACAAATGAGAGGGAAAAGGCATTACAGGCACAGGTCCAGTACCA
ACAGCAGCATGAGCAACAGAAAAAAGATTTAGAGACCCTCCATCAGCAAAACATTCACCAGCTGCAGAGC
AGACTGTCTGAGTTAGAAGCAGCTAATAAAGACCTCACTGAGAGGAGATACAAAGGAGACTCCGCTACTA
GAGAACTGAAAGCAAAGCTCTCTGGTGTTGAAGAGGAGCTACAGCGTACTAAGCAAGAAGTCCTTTCTTT

GCGAAGAGAGAATTGTACACTAGATGCTGAATGTCATGAGAAAGAAAAGCATATTAATCAGCTACAAACA
AAAGTGGCAGTTTTAGAACAAGAAATTAAGGATAAAGACCAGCTTGTTGTGAGAACAAAAGAAGCATTTG
ATACAATCCAGGAACAAAAGGTGGCATTAGAAGAGAATGGTGAGAAAAATCAGGTTCAACTGGGAAAACT
TGAAGCCACAATAAAATCATTATCAGCAGAACTTCTGAAGGCAAATGAAATTATCAAAAAGTTGCAAGGG
GATCTGAAAACTGTGATGGGTAAGTTGAAATTGAAGAATACAGTTACTATTCAGCAAGAAAAACTGTTGG
CTGAGAAGGAGGAAAAGTTACAAAAGGAGCAAAAGGAATTACAGGACATTGGACAGTCTCTCCGAGCTAA
AGAGCAAGAGGTATGCAGATTACAAGAACAGTTAGAAGCCACAGTTCAAAAACTTGAAGAAAGTAAACAA
CTTCTAAAAAACAATGAGAAGTTAATCACGTGGTTAAATAAAGAATTAAATGAAAATCAGCTTGTGAGAA
AGCAAGATGTATTGGGGCCTTCCCCCACTCCGCCTGCACATTCCAGCAGCAACGCGGTCAGAAGTGGAAT
TTCTCCTAACCTGAATATGATTGATAACAGACTTGCTTACCCCAGCTGTGGGATTGGCTATCCCGTCTCC
TCTGCATTTGCATTCCAAAATACCTTTTCTCACCCGGTACCTACCAAAAATGCCATCCACCCAGTTTCAG
CACCAAAGGTTCAGTTTAACTTGCAGTTTACAAAACCAAGTACATCACTAGGGGAGACTCACTCGGGAGC
AGCAGCAGTTCCATGCTCAACTGACAAGGAGAATGGTGAAAATTCAGGGCTGGAATCCAAATATTTGAAG
AAAAGAGAAGATAGCATTCCTTTGCGTGGGCTCAGCCAGAATCTATTTAGTAATGCAGAGCATCAGAAAG
ACGGCACTCTCGGAGCGTCGCAGGCCGCTTCCAAACCCACAGCGTTCCCGTCCGCGTCTTCGGCCTATTT
CCCCGGACAGCTGTCAAGCAGT
>Marmota marmota marmota XM_015502607.1
ATGAGCCAAGTTCTGTTCCAGCAACTAGT
TCCGCTGCAGATGAAATGCAAAGACTGCGAGGAAAGGAGAATAAATGTTAGAGTAAGCATTGAACTGCAA
TCAGTTTCTAATCCAGTTCATAGAAAGGATTTGGTTATTCGTCTGACTGATGATACAGATCCATTTTTTC
TGTATAATCTTATTATATCTGAGGAAGATTTTCAAAGTTTAAAATTCCAGCAAGGTCTTCTGGTAGACTT
CTTAGCTTTCCCACAAAAATTTATAGATCTCCTTCAACAGTGTACTCAAGAACATGTTAAAGAAATTCCA
AGGTTTTTGCTACAGTTAGTTTCTCCAGCAGCTATTTTGGATAATTCACCTGCATTTTTAAATGTGATAG
AGACAAATCCTTTTAAGCATCTTACACATCTCTCACTAAAACTCTTACCTGGAAATGATGTGGAAATAAA
GAAATTCCTAGCAGGTTGTTTGAAATGTAGTAAGGAAGAAATATTATCATTGACACAATCACTAGATGAT
GTTACTAGACAACTGAACTTGACACAAAAGACATTATCAGAAAAAATACAAGAATTAGAAAAGTTACGGA
ATGAATGGGAATCACACACAGCATCATTGACAAATAAGCATTCTCAAGAACTGACGAATGAGAAGGAAAA
GGCTTTACAGGTACAGGTTCAATATCAACAGCAGCATGAACAACAGAAAAAAGATTTAGAAATCCTCCAT
CAACGAAATATCCACCAGCTACAGAACAGATTGTCAGAGTTAGAGGCACTTAATAAAGACCTAACTGAAA
GGAAATATAAAGGAGACTCCAATATTAGAGAACTTAAAGCAAAACTTTCTGGTGTTGAAGAGGAGCTGCA
ACGAACTAAGCAAGAAGTCCTCTCTTTGCAAAGAGAGAATTCTACATTAGATGCTGAATGCCATGAGAAA
GAAAAGCATATTAATCAGCTACAAACAAAAGTGGCAGTTTTAGAACAAGAAATCAAGGATAAAGATCAAC
TTGTTTTAAGAACAAAAGAAGCATTTGATACAATCCAGGAACAAAAGGTGGCTTTAGAAGAAAGTGGTGA
GAAGAATCAGGTACAACTGGGAAAACTTGAAACTACGATAAAATCATTATCAGCAGAACTTCTGAAGGCA
AATGAAATTATCAAGAAGTTACAGGGGGATTTGAAAACCCTAATGGGCAAATTGAAACTGAAGAATACAG
TTACTGTTCAGCAAGAAAAACTCTTGGCTGAGAAGGAGGAAAAATTACAAAAGGAACAAAAGGAACTACA
AGATGTTGGACAGTCTCTTCGAGTTAAGGAGCAAGAGGTATGCAAATTACAAGAACAATTAGAGGCTACA
GTTCAAAAACTTGAGGAAAGCAAACAGCTTCTAAAAAATAATGAAAAGTTAATCACATGGTTAAATAAAG
AACTAAATGAAAATCAGCTAGTAAGAAAGCAAGATGTGTTGGGACCTTCTACCACTCCTCCTGCACATTC
CAGCAACAACACAATCAGAAGTGGAATTTCTCCTAACCTGAATGTGGTTGATAGTAGGCTGACTTACCCA
AGCTGTGGGATTCCTTATCCTGTCTCCTCTGCATTTGCATTCCAGAATACTTTTCCTCATCTGTTATCTA
CCAAAAATACCATCCACCCAGTTTCAGGACCAAAGGTTCAGTTTAACTTGCAGTTTACAAAACCAAATAT
ATCACTAGGAGATGTTCAGTCAGGAGCAACTGTTAATGTGCCTTGCTCAACTGATAAGGAAAATGGTGAA
AATTTAGGATTGGAATCTAAATACTTGAAGAAAAGAGAAGATAGCATTCCTTTACGTGGACTAAGCCAGA
ATCTGTTTAGTAATTCAGATCATCAGAAAGATGGCAGTCTAGGAGTGATGCAGACATCTTCCAAACCTGC
AGTGCTCCCCTCTACATCTTCAGCCTATTTTCCTGGGCAGTTACCAAACAGT
>panda ENSAMET00000009837.1

ATGAGCCAGGTTCTGTTCCAGGAGCTAGTCCCGCTGCAGGTGAAATGCAAAGACTGCGAG
GAGAGGAGAGTAAGTGTTAGAGTGAGCATTGAACTACAATCAGTTTCCAATCCAGTTCAC
AGAAAGGATTTAGTTATCCGTCTGACTGATGATAGTGATCCATTTTTTCTGTATAACCTT
GTTATATCTGAGGAGGATTTTCAGAGTTTAAAATTCCAGCAAGGCCTTCTGGTAGACTTC
TTAGCTTTCCCACAGAAATTTATAGATCTTCTTCATCAGTGTACTCAAGAACATGCCAAA
GAAATTCCAAGGTTTTTGCTGCAGTTAGTTTCTCCAGCACCTGTTTTGGATAATTCACCT
GCCTTTTTAAATGTGGTAGAGACAAATCCTTTTAAGCATCTTACACACCTGTCACTAAAA
CTTTTACCTGGAAATGATGTGGAAATAAAGAAGTTTTTAGCCAGCTGTTTGAAATGTAGC
AAGGAAGAAAAATTATCATTGGTGCAATCACTGGATGATGTTACTAGGCAACTGAACTTC
ACACAAAAGACATTATCAGAAAAAATGCAAGAATTAGATAAGTTGCAGAATGAATGGGCA
TCACACACAGCTGCATTGTCTAGTAAACATTCCCAGGAACTGACAAATGAGAAAGAAAAG
GCCTTGCAGGCACAGGTACAATACCAACAGCAGCATGAACAACAGAAAAAAGATTTAGAA
ATCCTCCATCAACGAAACGTCCAGCAACTACAAAACAAATTATCTGAGTTAGAAGCAGCT
AATAAAGACCTAACCGAAAGGAAATATAAAGGGGACTCGACCATCAGAGAACTTAAAACA
AAACTGTCTGGTGTTGAAGAGGACCTCCAGCGGGCTAAGCAAGAAGTCCTGTCTTTGCGA
AGAGAGAATTCTACACTAGATGCCGAATGCCATGAAAAAGAAAAGCATATTAATCAGCTA
CAAACAAAAGTGGCTGTTTTAGAACAGGAAATCAAGGATAAGGACCAGCTTGTTTTAAGA
ACAAAAGAAGCATTTGATACAATCCAGGAACAAAAGGTGGCTTTAGAAGAGAATGGGGAG
AAAAATCAGGTACAACTAGGAAAACTTGAAGCTACGATAAAATCATTATCGGCGGAACTT
CTTAAGGCAAATGAAATTATCAAGAAGTTACAGGGGGATCTGAAAACTTTAATGGGTAAA
TTGAAACTGAAGAATACAGTAACTATTCAGCAAGAGAAACTCTTGGCTGAGAAGGAAGAC
AAATTACAAAAGGAACAGAAGGAATTACAAGATGTTGGACAGTCTCTCCGAATTAAAGAG
CAAGAGGTGTGCAAATTACAAGAACAGTTAGAAGCTACCGTTCAAAAGCTTGAAGAAAGC
AAACAACTTCTAAAAAATAATGAAAAGTTAATCACATGGTTAAATAAAGAACTAAATGAA
AATCAGGTAGTCAGAAAGCAAGATATATTGGGACCTTCCAGCACTCCACCTGTACATTCT
ACCAGTACCACAATCAGAAGTGGGATTTCTCCTAACTCTAATGTGGTTGATGGTAGACTT
ACTTACCCAACTTGTGGTATTGGTTATCCTGTCTCCTCTGCATTTGCATTCCAGAATACT
TTTCCTCATCCTATATCTGCCAAAAATACCATCCACCCAATTTCAGGATCAAAGGTTCAG
TTTAACTTGCAGTTTACAAAACCAAATACATCACTAGGAGATGTTCATTCAGGAACAACT
ATTAGTGTGCCTTGCTCAACTGATAAGGAAAATGGTGCAAATTTAGGGCTGGAATCCAAA
TACCTGAAGAAAAGGGAAGATAGCATTCCTTTACGTGGGCTCAGCCAAAACCTGTTCAAT
AACCCAGACCATCAAAAAGATGGCACTTTGGGAGCATTACAGACATCTTCCAAACCTGCA
GTGCTCCCTCCTGCATCTTCCGCGTATTTCCCCGGGCAGTTAACAAACAGT
>cow ENSBTAT00000020951.5
ATGAGCCAGATTTTGTTCCAGCAACTCGTCCCGTTGCAGGTGAAATGCAGAGACTGCGAG
GAGAGGAGAGTGAGTATTAGACTGAGCATTGAACTACAATCAGTTTCTAACCCCGTTCAC
AGAAAGGATTTAGTTATCCGTCTGACTGATGATGCGGATCCATTTTTTCTGTATAACCTT
GTTATTTCAGAGGAAGATTTTCAGAGTTTAAAATTCCAGCAAGGTCTTCTGGTAGACTTC
TTAGCTTTCCCACAAAAATTCATAGATCTCCTCCAGCAGTGTACTCAAGAATACGCGAAA
GAAATTCCAAGGTTTTTGCTGCAGTTAGTTTCTACAGAAGCTATTTTGGATAACTCACCT
GCTCTTTTAAATGTGGTAGAGACAAATCCTTTTAAGCATCTGACACACCTCTCACTAAAA
CTTTTACCTGGAAATGATGTGGAAATAAAGAAGTTTCTAGCAGACTGTTTGAAATGTAGT
AAGGAAGAAAAATTATCATTAACACAATCGCTGGATGATGTTACCAGGCAACTGAATTTC
ACCCAAAAGACATTATCAGAAAAGATCCAAGAATTAGATAAGTTACGGAATGAATGGGCG
TCGCACACAGCAACATTGTCAAATAAGCATTCTCAGGAACTAACGAATGAGAAGGAAAAA
GCTTTGCAGGCCCAGGTTCAATACCAACAGCAACATGAACAACAGAAGAAAGATTTAGAA
ATCCTTCATCAACGGAACATGCAGCAGCTACAGAACAGATTGTCTGAGTTAGAAACGGCT

AATAAAGACCTCACTGAAAGGAAATACAAAGGAGACTCCACCATCAGAGAACTTAAAGCA
AAGCTGTCTGGTGTTGAAGAGGAGCTCCAGCGGGCTAAGCAAGAAGTCCTCTCTTTGAGA
AGAGAAAATTCTACACTCGATGCTGAATGCCATGAAAAAGAAAAGCATATTAATCAGCTA
CAAACAAAAGTGGCTGTTTTAGAACAGGAAATCAAGGATAAGGACCAGCTTGTTTTAAGA
ACAAAAGAAGCGTTTGATACAATCCAGGAACAAAAGGTGGCTCTGGAAGAAAATGGTGAG
AAAAATCAGGTACAACTAGGAAAACTTGAAGCTACAATAAAATCATTATCAGCTGAACTT
CTTAAGGCAAATGAAATTATCAAAAAACTGCAAGGGGATCTGAAGACTTTAATGGGTAAG
TTGAAATTGAAGAATACAGTAACTATTCAGCAAGAGAAACTCTTGGCTGAAAAGGAAGAA
AAATTACAAAAGGAACAAAAAGAATTACAAGATGTTGGACAGTCTCTCCGAATTAAAGAA
CAAGAGGTATGCAAATTACAAGAACAATTAGAAGCTACAGTTCAAAAACTTGAAGAAAGC
AAACAACTTCTAAAAAATAATGAAAAGTTAATCACATGGTTAAATAAAGAATTAAATGAA
AATCAGCTAGTGAGAAAGCAAGATGTATTGGGACCTTCTACCACTCCACCTGTACATTCC
AGTAGCAACACCATCAGAAGTGGAATTTCTCCTACCTCAAATGTGGTTGAGGGTAGACTG
ACTTACCCAGCTTGTGGGATTGGTTATCCTGTCTCCTCTGCGTTTGCATTCCAGAATACC
TTTTCTCATCCTATATCTGCCAAAAATAGCATCCACACAGTTTCAGGACCAAAGGTTCAG
TTTAACTTGCAGTTTACAAAACCAAATCCATCACTAGGAGATGTTCAGTCAGGAACAACT
ATTAGTATGCCCTGCTCAACTGATAAGGAAAATGGTGAAAATTTAGGACTGGAATCCATA
TATCTGAAGAAAAGGGAAGATAGCATTCCTTTGCGTGGACTCAGCCAAAATCTATTTAAT
AATCCAGACCATCAGAAAGATGGCACTTTAGGAGCATTACAGACATCTTCAAAACTCACA
GTGCTCCCTTCTGCATCTTCAGCTTACTTTCCCGGGCAGCTACAGAACAGT
>dog ENSCAFT00000031863.4
ATGAGCCAGGTTCTGTTCCAAGAGCCGGTCCCGCTGCAGGTGAAATGCAAAGACTGC
GAGGAGAGGAGAATAACTGTTAGAGTGAGCATTGAACTACAATCAGTTTCCAATCCAGTT
CACAGAAAGGATTTAGTTATCCGTCTGACTGATGAAACGGATCCATTTTTTCTATATAAC
CTTGTTATATCTGAGGAAGATTTTCAGAGTTTAAAATTCCAGCAAGGCCTTCTGGTCGAC
TTCTTAGCTTTCCCACAAAAATTTATAGATCTCCTTCAGCAGTGTACTCAAGAACATGCC
AAAGAAATTCCAAGGTTTTTGTTACAGTTAGTTTCTCCAGCACCTATTTTGGATAACTCA
CCTGCCTTTTTAAATGTGGTAGAGACAAATCCTTTTAAGCATCTCACACACCTCTCACTA
AAACTTTTACCTGGAAATGATGTGGAAATAAAGAAGTTTCTGGCCGGCTGTTTGAAATGT
AGTAAGGAAGAAAAATTATCATTGATGCAATCACTAGATGATGTTACTAGGCAACTGAAT
TTCACACAAAAGACATTATCAGAAAAAATCCAAGAATTAGATAAGTTACAGAATGAATGG
GCATCACACACAGCTGCATTGTCAAATAAGCATTCCCAGGAACTGACAAATGAGAAAGAA
AAGGCCTTGCAGGCACAGGTACAGTACCAACAGCAGCATGAACAACAGAAAAAAGATTTA
GAAATCCTCCATCAACGAAATATCCAGCAACTACAAAACAAATTATCTGAGTTAGAAGCA
ACTAATAAAGACCTAACGGAAAGGAAATATAAAGGAGACTCTACCATCAGAGAACTGAAA
ACAAAACTGTCTGGTGTAGAAGAGGAACTCCAGCGGGCTAAGCAAGAAGTCCTCTCTTTG
CGAAGAGAGAATTCTACACTAGATGCCGAATGCCATGAAAAAGAAAAGCATATTAATCAG
CTACAAACAAAAGTGGCTGTTTTAGAACAGGAAATCAAGGATAAGGACCAGCTTGTTTTA
AGAACAAAAGAAGCATTTGATACAATCCAGGAACAAAAGGTGGCTTTAGAAGAGAATGGT
GAGAAAAATCAGGTACAGCTAGGAAAACTTGAAGCTACAATAAAATCATTATCAGCAGAA
CTTCTTAAGGCAAATGAAATTATCAAGAAGTTACAGGGGGATCTGAAAACTTTAATGGGT
AAATTAAAACTGAAGAATACAGTAACTGTTCAACAAGAGAAACTCTTGGCTGAGAAGGAA
GACAAATTACAAAAGGAACAAAAGGAATTACAAGATGTTGGACAGGCTCTCCGAATTAAA
GAGCAAGAGGTATGCAAATTACAAGAACAGTTAGAAGCTACTGTTCAAAAGCTTGAAGAA
AGCAAACAGCTTCTAAAAAATAATGAGAAGTTAATCACATGGTTAAATAAAGAACTAAAT
GAAAATCAGCTAGTGAGAAAACAAGATGTATTGGGACCTTCTACCACTCCACCTGTGCAT
TCTAGCAGTAACACAATTAGAAGTGGAATTTCTTCTAACTCTAATGTGGTTGATGGTAGA

CTGACTTACCCAAGTTTTGGGATTGGTTATCCTATCTCCTCTGCATATGCATTCCAGAAT
AACTTTCCTCATCCTGTATCTGCCAAAAATACCAACCACCCAATTTCAGGACCAAAGGTT
CATTTTAACTTGCAGTTTACAAAACCAAATACGTCACTAGGAGATGTTCAGTCAGGAACA
ACCATTAGTATGCCTTGCTCAACTGATAAGGAAAATGGTGCAAATTTAGGGCTGGAATCC
AAATACCTGAAGAAAAGGGAAGATAGCATTCCTTTACGTGGGCTCAGCCAAAACCTGTTC
AATAATCCAGACCATCAAAAAGATGGCACTTTAGGAGCCTTACAGACCTCTTCAAAACCC
GCAGTGCTCCCTTCTCCATCTTCAGCATACTTCCCTGGGCAGTTAACAAACAGT
>erinaceus europaeus XM_007528586.2
ATGA
GCCAGGTTCTGTTCCAGCAGCTAGTCCCGTTGCAGGTGAAGTGCAAAGACTGCGAGGAGAGGAGAGTAAG
TGTTAGAGTGAGCATTGAACTACAATCAGTTTCTAATCCAGTTCACAGAAAGGATTTAATTATTCGTCTG
ACTGATGACTCTGATCCATTTTTTCTGTATAACCTCGTCATATCTGAGGAAGATTTTCAGAGCTTAAAGT
TCCAGCAGGGTCTTCTGGTAGACTTCTTAGCTTTCCCACAAAAGTTTATTGATCTCCTTCAGCAGTGCAC
TCAGGAATACACCAAAGAAATTCCAAGGTTTTTGCTACAGTTAGTTTCTCCAACAACTATTTTGGATAGC
TCACCTGCCTTTTTAAATGTGGTAGAGACAAACCCTTTTAAACACCTTACGCACCTATCGTTAAAACTTT
TACCTGGAAATGATGTGGAAATAAAGAAATTTCTAGCAGGCTGTTTGAAATGTAGCAAGGAAGAAAAATT
ATCATTGACGCAGTCACTAGATGATGTTACTAGGCAACTGAATTTTACCCAAAAGACATTATCAGAAAAA
ATCCAAGAATTAGATAAATTAAGGAATGAATGGGCATCACACACAGCAGCATTATCAAATAAACATTCTC
AGGAACTGACAAATGAGAAGGAAAAAGCCTTGCAGGCACAGGTGCAATACCAACAGCAGCATGAACAACA
GAAAAAAGATTTAGAAGCCCTCCACCAACGAAACATTCAGCAGCTACAAAATAGACTGTCTGAGTTAGAA
GCAACCAATAAAGACCTAACTGAAAGGAAATATAAAGGAGACTCTACCATCAGAGAGCTTAAAGCCAAAT
TATCTGGCATTGAAGAGGAGGTCCAGAGGGCCAAGCAAGAAGTATTGTCTTTGCGAAGAGAGAATTCTAC
ACTAGATGTTGAATGTCATGAGAAAGAAAAGCTTATTAATCAGCTACAAACAAAAGTGGCTGTCTTAGAA
CAGGAAATCAAGGATAAGGACCAACTTGTTCTAAGAACAAAAGAAGCATTTGATACCATCCAGGAACAAA
AGATTGCGTTAGAAGAAAATGGTGAGAAAAATCAGGTACAGCTAGGAAAACTTGAAGCCACTATAAAATC
GTTATCAGCAGAACTTCTTAAGGCAAATGAAATTATCAAGAAGCTACAAGGGGATCTGAAAACTTTAATG
GGTAAATTGAAATTGAAGAATACAGTTACTATTCAGCAAGAGAAGCTCTTGGCTGAGAAAGAAGAAAAAT
TACAAAAGGAACAAAAAGAATTACAAGATGTTGGACAGTCTCTGCGAATGAAAGAGCAAGAGGTGTGCAA
ATTACAAGAACAATTAGAAGCTACAGTTCAAAAACTTGAAGAAAGCAAACAGCTTCTAAAAAATAATGAA
AAGTTAATTACATGGTTAAATAAAGAACTAAATGAAAATCAAGTAGTGAGAAAGCAAGATTTATTGGGAT
CTTCTGCCACTCCACCTGTACATTCTAGTAGCAATACAATCAGAAGTGGAATTTCTCCTAACCCTAATGT
GGTTGATGGTAGACTGACATACCCAGCCTGTGGGATTGGTTATCCTGTTTCTTCTGCGTATGCATTCCAA
AATACCTTCCCTCATTTTATACCTGCCAAAAATACCATCCATCCAGTTTCAGGACCAAAGGTTCAGTTTA
ACTTGCAATTTACAAAAGCAAATACATCCTCAGATGTTCAGTCAGGAACAGCAATCAGTATGCCTTCCTC
AACTGATAAAGAAAGTGGTGAAAATTTAGGACTGGAATCCAAATACCTGAAAAAAAGGGAAGATAGCATT
CCTTTACGCGGACTCAGTCAAAATCTATTTAATAATCCAGATCATCACAAAGATGGCCCTTTGGGGACAT
CACAGACATCAAAACCAGCAGGACTGTCTTCTGCATCGTCAGCCTATTTCCCTGGGCAGCTTCCCAACAG
T
>Felis catus XM_003990361.3
ATGAGC
CAGGTTCTGTTCCAGGAACTAGTCCCGCTGCAGGTGAAATGCAAAGACTGCGAGGAGAGGAGAGTAAGTG
TTAGAGTGAGCATTGAACTACAATCAGTTTCTAATCCAGTTCACAGAAAGGATTTAGTTATCCGTCTGAC
TGATGATACCGATCCATTTTTTCTGTATAACCTTGTTATATCTGAGGAAGATTTTCAGAGTTTAAAATTC
CAGCAAGGCCTTCTGGTAGACTTCTTAGCTTTCCCACAAAAATTTATAGATCTTCTTCAGCAATGTACTC
AAGAACATGCCAAAGAAATTCCAAGGTTTTTGCTGCAGTTAGTTTCTCCAGCACCTATTTTGGATAACTC
ACCTGCCTTTTTAAATGTGGTAGAGACAAATCCTTTTAAGCATCTTACACACCTCTCACTAAAACTTTTA
CCTGGAAATGATGTGGAAATAAAGAAGTTTTTAGCCAGCTGTTTGAAAAGTAGCAAGGAAGAAAAATTAT

CATTGATGCAATCACTAGATGATGTTACTAGGCAACTGAAATTCACACAAAAGACCTTATCAGAAAAAGT
GCAAGAATTAGATAAGTTACAGAATGAATGGGCATCACACACAGCTGCATTGTCAAATAAACATTCCCAG
GAACTAACAAATGAGAAAGAAAAGGCCTTGCAGGCACAGGTACAGTGTCAACAGCAGCATGAACAACAGA
AAAAAGATTTAGAAATCCTCCATCAACGAAACATCCAGCAGCTACAAAACAAATTATCTGAGTTAGAAGC
GGCTAATAAGGACCTAATGGAAAGAAAATATAAAGGAGACTCTACCATCAGAGAACTGAAAACAAAACTC
TCTGGTGTGGAAGAGGAGCTCCAGCGGGCTAAGCAAGAAGTTCTCTCTTTGCGAAGAGAAAATTCTACCC
TAGATGCTGAATGCCATGAAAAAGAAAAGCATATTAATCAGCTACAAACAAAAGTGGCTGTTTTAGAACA
GGAAATCAAGGATAAGGACCAGCTTGTTTTAAGAACAAAAGAAGCATTTGATACAATCCAGGAACAAAAG
GTGGCTTTAGAAGAAAATGGTGAGAAAAATCAGGTACAACTAGGAAAACTTGAAGCTACAATAAAATCAT
TATCAGCGGAACTTCTTAAGGCAAATGAAATTATCAAGAAGTTGCAGGGAGATCTGAAAACTTTAATGGG
TAAATTGAAACTGAAGAATACAGTAACTATTCAGCAAGAGAAACTCTTAGCTGAGAAGGAAGAAAAATTA
CAAAAGGAACAAAAGGAATTACAAGATGTTGGACAGTCTCTCCGAATTAAAGAGCAAGAGGTATGCAAAT
TACAAGAACAATTAGAAGCTACAGTTCAAAAACTTGAAGAAAGCAAACAGCTTCTAAAAAATAATGAAAA
GTTAATCACATGGTTAAATAAAGAACTAAATGAAAATCAGCTAGTGAGAAAGCAAGATGTATTGGGACCG
TCCACCACTCCGCCTGTACATTCGAGCAGCAACGCAATCAGAAGTGGGATTTCTCCTAATTCTAATGTGG
TTGATGGTAGACTTACCTACCCAGCCTGTGGGATTGGTTATCCTGTCTCCTCTGCATTTGCATTCCAGAA
TACCTTTCCTCATCCTATATCTGCCAAAAATAACATCCATCCAGTTTCAGGACCAAAGGTTCAGTTTAAC
TTGCAGTTTACAAAACCAAATACGTCACTAGGAGATGTTCAAACAGGAACAACTATTAGTATGCCTTGCT
CAACTGATAAGGAAAATGGTGCAAATTTAGGGCTGGAATCCAAATATCTGAAGAAAAGGGAAGATAGCAT
TCCTTTACGCGGACTCAGCCAAAATCTGTTCAGTAATCCAGACCATCAGAAAGATGGCACTTTCGGAGCA
GTACAGACATCTTCAAAGCCCCCTGTGCTCCCTTCTGCACCGTCAGCCTATTTCCCTGGCAGTTAGCAA
ACAGT
>elephant ENSLAFT00000002382.3
ATGAGCCAGGTTCTCTTCCAGCAACTGGTCCCGTTGCAGGTGAAATGCAAAGACTGCGAA
GAAAGGAGAGTAAGTGTTAGAGTGAGCATTGAACTACAATCAGTTTCTAACCCAGTTCAC
AGAAAGGATTTAGTTATTCGTCTGACTGACGATACTGATCCCTTTTTCCTGTATAACCTC
GTTATATCTGAGGAAGATTTTCAGAGTTTGAAATTCCAGCAAGGTCTTCTGGTAGACTTC
TTAGCTTTCCCACAAAAATTTGTAGATCTCCTTGAGCAGTGTACTCAAGAACACGTCAAA
GAAATTCCAAGGTTTTTGCTACAGTTAGTGTCTCCAGCAGCCATTTTGGATAACTCACCT
GCATTTTTAAATGTGGTGGAGACAAATCCTTTTAAGCATCTTACTCACCTTTCACTAAAA
CTTTTACCTGGAAATGATGTGGAAATAAAGAAATTTCTAGCAGGATGTTTGAAATGTAGC
AAGGAAGAAAAATTATCATTGACGCAATCACTAGATGATGTTACTAGGCAACTGAACTTG
ACACAAAAGACATTATCAGAAAAAATCCAGGAACTAGATAAGCTACGGAATGAATGGGCA
TCACACACAGCAGCATTGACAAATAAGCATTCTCTGGAACTAACAAATGAGAAGGAAAAG
GCCTTACAGGCACAGGTTCAATGTCAACAGCAGCACGAACAACAGAAAAAAGATTTAGAA
ATCCTCCACCAGCGAAACATCAGCCAGCTACAAAACAGACTGTCTGAGTTAGAAGCGGCT
AATAAGGACCTAACTGAAAGGAAATATAAAGGAGACTCCACTATCAGAGAACTTAAAGCA
AAACTTTCTGGCGTTGAAGAGGAGCTCCAGCGGGCCAAGCAAGAAGTCCTGTCTTTGCGG
AGGGAGAATTCTGCGCTGGATGCTGAATGCCATGAGAAAGAGAAGCATAATAATCAGCTC
CAAACAAAAGCGGCTGTTTTAGAGCAGGAGATCAAGGATAAGGACCAGCTTGTTTTAAGG
ACAAAAGAAGCATTCGATACAATCCAGGAACAAAAGGTGGCTTTAGAAGAAAATGGAGAG
AAAAGTCAGGTGCAACTAGAAAAACTTGAAGCTACGATAAAATCATTATCAGCAGAACTT
CTTAAGGCAAATGAAATTATCAAGAAGTTACAAGGGGATCTCAAAACTTTAATGGGTAAG
TTGAAACTGAAGAATACAGTTACTATTCAGCAAGAAAAACTCTTGGCTGAGAAGGAAGAA
AAATTACAAAAGGAACAAAAGGAATTACAAGATGTTGGACAGTCTCTCCGAATTAAAGAG
CAAGAGGTATGCAGATTACAAGAACAGTTAGAAGCTACAGTTCAAAAACTTGAAGAAAGC
AAACAGCTTCTGAAAAATAATGAGCAGTTAATCACATGGTTAAATAAAGAACTAAATGAA

AATCAGCTAGTGAGAAAGCAGGATGCATTGGGACCTTCTACCACTCCACTTGGACATTCT
AGCAGCAACACAATCAGAAGTGGGATTTCTCCTAACCCTAATGTGGTTGATGGTAGACTG
ACTTACCCAGCTTGTGGGATTGGTTATCCTGGCTCCTCTGCGTTTGTATTCCAGAATGCC
TTTGCACATCCAATATCTGCCAAAAATGCAGTCCACCCAGTTTCAGGACCAAAGGTCCAC
TTTAACTTACAATTTACAAAGCCAAATACATCTGTGGGCGATGTTCAGTCTGGAGGAGCT
ATGAATATGCCTAGCTCATCTGATAAGGAAAATGGTGAAAACTTGGGGCTGGAATCCAAA
TATCTGAAGAAAAGGGAAGATAGCATTCCTTTACGTGGGCTCAGCCAGAATCTGTTTAAT
AATCCAGACCATCAGAAAGATGGCGCTTTAGGCACGTTACAGACGTCTTCAAAACCTGCA
GTGCTCCCTCCTGCGTCTTCAGCGTATTTCCCTGGACAGTTACCAAACAGT

>ferret ENSMPUT00000012275.1
ATGAGCCAGGTTCTGTTCCAGGAGCTAGTCCCACTGCAGGTAAAATGCAAAGACTGCGAG
GAGAGGAGAGTAAGTGTTAGAGTGAGCATTGAACTACAGTCAGTTTCCAATCCAGTTCAC
AGAAAGGATTTAGTTATCCGTCTGACTGATGATACTGATCCATTTTTTCTATATAACCTT
GTTATTTCCGAGGAAGATTTTCAGAGTTTAAAATTCCAGCAAGGCCTTCTGGTAGACTTC
TTAGCTTTCCCACAGAAATTTATAGATCTCCTTCAGCAGTGTACTCAAGAACAAGCCAAA
GAAATTCCAAGGTTTTTGCTACAGTTAGTTTCTCCATCACCTATTTTGGATAACTCACCT
GCCTTTTTAAATGTGGTAGAGACAAATCCTTTTAAGCATCTTACACACCTCTCATTAAAA
CTTTTACCTGGAAGTGATGTGGAAATAAAGAAGTTTTTAGCCAGCTGTTTGAAATGTAGC
AAGGAAGAAAAATTATCATTGATTCAATCACTAGATGATGTTACTAGGCAACTGAATTTC
ACACAAAAGACATTATCAGAAAAAATCCAAGAATTAGATAAGTTGCAGAATGAATGGGCA
TCACACACAGCTGCATTGTCAAATAAACATTCCCAGGAACTGACGAATGAGAAAGAAAAG
GCCTTGCAGGCACAGGTCCATTACCAACAGCAGCATGAACAACAGAAAAAAGATTTAGAA
ATCCTCCATCAACGAAACATCCAGCAACTACAAAACAAATTATCTGAGTTAGAAACAGCT
AATAAAGACCTAACCGAAAGGAAATATAAAGGAGACTCTACCATCAGAGAACTTAAAACA
AAACTGTCTGGTGTTGAAGAGGAGCTCCAGCGGGCTAAGCAAGAAGTCCTCTCTTTGCGA
AGAGAGAATTCTACACTAGACGCAGAATGCCACGAGAAAGAAAAGCATATTAATCAGTTA
CAAACAAAAGTGGCTGTTTTAGAACAGGAAATCAAGGATAAGGACCAACTTGTTTTAAGA
ACAAAAGAAGCATTTGATACAATCCAGGAACAAAAGGTGGCTTTAGAAGAGAATGGTGAG
AAAAATCAGGTACAACTAGGAAAACTTGAAGCTACGATAAAATCCTTATCAGCGGAACTT
CTTAAGGCAAATGAAATTATCAAGAAGCTACAGGGGGATCTGAAAACTTTAATGGGTAAA
TTGAAACTGAAGAATACAGTAACTATTCAGCAAGAGAAGCTTTTGGCTGAGAAGGAAGAC
AAATTACAAAAGGAACAAAAAGAATTACAAGATGTTGGACAGTCTCTCCGAATTAAAGAG
CAAGAGGTGTGCAAATTACAAGAACAGTTAGAAGCTACCGTTCAAAAGCTTGAAGAAAGC
AAACAGCTTCTAAAAAATAATGAAAAATTAATCACATGGTTAAATAAAGAACTAAATGAA
AATCAGCTAGTGAGAAAGCAAGATGGATTGGGACCTTCTACCACTCCACCTGTACATTCT
AGCAGCAACATAATCAGAAGTGGGATTTCTCCTAACTCAAATGTGGTTGATGGTAGACTT
CCTTACCCAACTTGTGGGATTGGTTATCCTGTCTCCTCTGCATTTGCATTCCAAAATACC
TTTCCTCATCCTATATCTGCCAAAAATACCATCCACCCAGTTTCTGGACCAAAGGTCCAG
TTTAACTTGCAGTTTACAAAACCAAATACACCTCTAGGAGAAGTTCAGTCAGGAACAACT
GTTAGTATGCCTTGCTCAACTGATAAGGAAAATGGTGCAAATGTAGGGCTGGAATCCAAA
TACCTGAAGAAAAGGGAAGACAGCATTCCTTTACGTGGGCTCAGCCAAAACCTGTTCAAT
AATCCAGACCATCAAAAAGATGGCACTTTAGGAGCATTACAGACATCTTCCAAACCTGCA
GTGCTCCCTTCTGCATCTTCAGCGTATTTCCCTGGGCAGTTAACAAACAGT
>myotis lucifagus ENSMLUT00000002118.1
ATGAGCCAGGTTCTGTTCCAGCAGCTCGTCCCGCTGCAGGTGAAGTGCAGAGACTGCGAG
GAGAGGAGAGTCAGTGTTAGAGTGAGCATTGAGCTGCAAGCAGTGTCTAATCCGGTTCAC

AGAAAGGATTTAGTTATCCGTCTGACCGATGACACTGATCCATTTTTCCTCTATAACCTT
GTTATATCGGAGGAAGATTTTCAGAGTTTAAAATTCCAGCAAGGTCTTCTGGTAGACTTT
TTAGCTTTCCCGCAGAAATTTATAGATCTGCTTCAGCAGTGTACTCAGGAGCATGCCAAG
GAAACCCCAAGGTTCCTGCTGCAGTTAGTGTCTCCAGCGGCTGTGTTGGATAACTCTCCT
GTCCTTCTAAATGTGGTAGAGACAAATCCTTTTAAGCATCTTACACACCTCTCACTAAAA
CTTCTACCTGGCAGTGATGTGGAGATAAAGAAGTTTCTAGCAGGCTGTTTGAAATGTAGC
AAGGAAGAAAAATTATCATTGACACAATCGCTAGAGGATGTTACTAGGCAACTGAATTTC
ACACAAAAGACATTATCAGAAAAAGTTCAAGAATTAGATAAGTTACGGAATGAATGGGCA
TCCCACACAGCAGAATTGTCGAGTAAGCATTCTCAGGAGCTGACCAGTGAGAGGGAAAAG
GCCTTGCAGGCGCAGGTGCAGTACCAACAGCAGCGTGAGCAGCAGAAGAAAGAGCTAGAA
ATCCTCCATCAGCGCAACACCCAGCAGCTGCAGAGCAGGCTGTCCGAGCTAGAAGCAGCT
AACAAAGACCTCACCGAGAGGAGATACAAAGGCGACTCCACCATCAGAGAGCTTAAAGCA
AAGCTGGCTGGAGTTGAGGAGGAGCTGCAGCGGGCCAAGCAGGAAGTGCTGTCTCTGCGA
AGAGAGAACTCGACGCTCGATGCCGAGTGCCACGAGAAGGAGAAGCATGCTAATCAGCTG
CAGACAAAAGTGGCTGTTCTAGAGCAGGAGATCAAGGACAAGGACCAGCTGGTCCTCAGA
ACAAAAGAAGCATTTGATACAATCCAGGAACAGAAGGTGGCTTTAGAAGAACACCGTGAG
AAAAATCAGGTACAAATAGGAAAACTTGAATCAACCATAAAATCATTATCAGCGGAACTG
CTTAAGGCAAATGAAATTATCAAGAAGTTACAAGGGGATCTGAAAACTTTGATGGGGAAA
TTGAAACTGAAAAATACAGTCACCATTCAGCAAGAGAAACTCTTGGCCGAAAAGGAAGAA
AAATTACAAAAGGAACAAAAGGAATTACAAGAAGTTGGGCAGTCTCTCCGAAGTAAAGAG
CAGGAGGTATGCAAATTACAAGAACAATTAGAAGCTACAGTTCAAAAACTTGAGGAAAGC
AAACAGCTTCTAAAAAATAATGAAAAGTTGATCACATGGTTAAATAAAGAACTAAATGAT
AATCAGCTAGTGAGAAAGCAGGACGTGCTGGGACCTTCTGCCACTCCACCGGTACATGCT
AGCAGCACGATCAGAAGTGGACTGTCTCCTAGCTCTAACATGGTTGATGGTAGGCTGACT
TACCCAACTTGTGGCATCGGGTATCCGGTCTCCTCTGCATTTGGTTTCCAGAACACCTTC
CCTCATCCCGTGTCTGCCAAAAATGCCATCCACCCGGCTTCAGGACCAAAGGTTCAGTTT
AACTTGCAGTTAACAAAACCAAACCCGTCACTAGGGGATATTCAGTCAGGAACAACTCTC
AGTGTGCCCGGCTCCGCTGATAAGGAAAATGGTGAAAATGTAGGGCTGGAGCCCAAGTAT
CTGAAGAAAAGGGAGGACAGCATTCCTTTGCGGGGGCTCAGCCAGAATCTCTTCAGCACT
GCAGACCATCAGAAAGATGGCCCGTTAGGAGCGCTGCAGACATCCTGCAAACCCGCCTCG
GTCCCTTCCTCCTCCTCTGCGTACTTCCCCGGGCAGCTGCCTCACAGC
>pteropus alecto XM_006919559.2
ATGAGCCAAGTTCTGTTCCAGCAGCTAGTCCCGTT
GCAGGTGAAGTGCAAAGACTGCGAAGAGAGGAGAGTAAGTGTTAGAGTGAGCATTGAACTACAATCAGTT
TCTAATCCAGTTCACAGAAAGGATTTAGTTATTCGTCTGACTGATGATACTGATCCATTTTTCCTGTATA
TCCTTGTCATATCTGAGGAAGATTTTCAGAGTTTAAAATTCCAGCAGGGTCTTCTGGTAGACTTTTTAGC
TTTCCCACAAAAATTTATAGATCTCCTTCAGCAATGTACTCAAGAACATGCCAAAGAAATTCCAAGGTTT
TTGTTACAGTTAGTTTCTCCTGCAGCTGTTTTGGATAACTCTCCTGCCTTTTTAAATGTGGTAGAGACAA
ATCCTTTTAAGCATCTTACACATCTCTCACTAAAACTTTTACCTGGAAGCGATGTGGAAATAAAGAAGTT
TCTAGCAGGCTGTTTGAAATGTAGTAAGGAAGAAAAATTATCATTGACGCAATCACTAGAGGATGTCACT
AGGCAACTGAATTTCGCACAAAAGACATTATCAGAAAAAATCCAAGAATTAGATAAGTTACGGAATGAAT
GGGCATCACACACAGCAGCATTGTCAAATAAGCATTCTCAGGAACTGACAAACGAGAAGGAAAAAGCCTT
GCAGGCACAAGTTCAATACCAACAGCAGCATGAACAACAGAAAAAAGATTTAGAAATCCTGCATCAACGA
AACGTCCAGCAGCTACAGAACAGATTGTCTGAGTTAGAAGCAACTAATAAAGACCTAACTGAAAGGAAAT
ATAAAGGAGACTCCTCCATCAGAGAACTAAAGGCAAAACTGTCTGGTGTTGAAGAGGAGCTCCAGCGGAC
TAAACAAGAAGTTCTCTCCTTGCGAAGAGAGAATTCTACACTAGATGCTGAATGTCATGAAAAAGAAAAG
CATATTAATCAGCTGCAAACAAAAGTGGCTGTTTTAGAACAAGAAATTAAGGATAAGGACCAGCTTGTAT

TAAGAACAAAAGAAGCATTTGATACAATCCAGGAACAAAAGGTGGCTTTAGAAGAAAATGGTGAGAAAAA
TCAGGTACAACTTGGAAAACTTGAAGCTACCATAAAATCATTATCAACGGAACTTCTTAAGGCAAATGAA
ATTATCAAGAAGTTACAAGGGGATCTGAAAACTTTAATGGGTAAATTGAAACTGAAGAATACAGTTACCA
TTCAGCAAGAGAAACTCTTGGCTGAGAAGGAAGAAAAATTACAAAAGGAACAAAAGGAATTACAAGATGT
TGGACAGTCTCTTAGAAATAAAGAGCAAGAGGTATGCAGATTACAAGAACAATTAGAAGCTACAGTTCAA
AAACTTGAAGAAAGCAAACAGCTTCTAAAAAATAATGAAAAGTTAATCACATGGTTAAATAAAGAACTAA
ATGAAAATCAGCTAGTGAGAAAGCAAGATGTGTTGGGACCTTCTGCCACTCCACCTGTGCATTCTAGTAG
CAACGCAATTAGAAGTGGAATTTCTCCTAACCCTAATGTGGTTGATGGTAGACTGGCTTACCCAGCTTGT
GGGATTGGTTATCCTGTCTCCTCTGCATTTGCTTTCCAGAATACCTTTCCTCATCCTTTATCTGCCAAAA
ATGCCATCCACTCAGTTTCAGGACCAAAGGTTCAGTTTAACTTGCAGTTTACAAAACCAAATACATCACC
AGGAGATGTTCAGTCAGGAACAACTGTTAGTATGCCTCGCTCAACTGATAAGGAAAATGGTGAAAATTTA
GGGCTGGAATCCAAATATCTGAAGAAAAGGGAAGATAGCATTCCTTTACGTGGACTCAGCCAAAATCTCT
TCAGTAACCCAGACCATCAGAAAGGTGGCCCGTTGGGAGCGTTACAGGCACCCTCAAAGCCAGCGGCGCT
GCCTTCCACATCTTCAGCGTATTTCCCGGGACAGTTACCCAACAGT
>pteropus vampyrus XM_011368393.1
ATGAGCCAAGTTCTGTTCC
AGCAGCTAGTCCCGTTGCAGGTGAAGTGCAAAGACTGCGAAGAGAGGAGAGTAAGTGTTAGAGTGAGCAT
TGAACTACAATCAGTTTCTAATCCAGTTCACAGAAAGGATTTAGTTATCCGTCTGACTGATGATACTGAT
CCATTTTTCCTGTATATCCTTGTCATATCTGAGGAAGATTTTCAGAGTTTAAAATTCCAGCAGGGTCTTC
TGGTAGACTTTTTAGCTTTCCCACAAAAATTTATAGATCTCCTTCAGCAATGTACTCAAGAACATGCCAA
AGAAATTCCAAGGTTTTTGTTACAGTTAGTTTCTCCCGCAGCTGTTTTGGATAACTCTCCTGCCTTTTTA
AATGTGGTAGAGACAAATCCTTTTAAGCATCTTACACATCTCTCACTAAAACTTTTACCTGGAAGCGATG
TGGAAATAAAGAAGTTTCTAGCAGGCTGTTTGAAATGTAGTAAGGAAGAAAAATTATCATTGACGCAATC
ACTAGAGGATGTCACTAGGCAACTGAATTTCGCACAAAAGACATTATCAGAAAAAATCCAAGAATTAGAT
AAGTTACGGAATGAATGGGCATCACACACAGCAGCATTGTCAAATAAGCATTCTCAGGAACTGACAAATG
AGAAGGAAAAAGCCTTGCAGGCACAAGTTCAATACCAACAGCAGCATGAACAACAGAAAAAAGATTTAGA
AATCCTGCATCAACGAAACGTCCAGCAGCTACAGAACAGATTGTCTGAGTTAGAAGCAACTAATAAAGAC
CTAACTGAAAGGAAATATAAAGGAGACTCCTCCATCAGAGAACTAAAGGCAAAACTGTCTGGTGTTGAAG
AGGAGCTCCAGCGGACTAAACAAGAAGTTCTCTCCTTGCGAAGAGAGAATTCTACACTAGATGCTGAATG
TCATGAAAAAGAAAAGCATATTAATCAGCTGCAAACAAAAGTGGCTGTTTTAGAACAAGAAATTAAGGAT
AAGGACCAGCTTGTATTAAGAACAAAAGAAGCATTTGATACAATCCAGGAACAAAAGGTGGCTTTAGAAG
AAAATGGTGAGAAAAATCAGGTACAACTTGGAAAACTCGAAGCTACCATAAAATCATTATCAACGGAACT
TCTTAAGGCAAATGAAATTATCAAGAAGTTACAAGGGGATCTGAAAACTTTAATGGGTAAATTGAAACTG
AAGAATACAGTTACCATTCAGCAAGAGAAACTCTTGGCTGAGAAGGAAGAAAAATTACAAAAGGAACAAA
AGGAATTACAAGATGTTGGACAGTCTCTTAGAAATAAAGAGCAAGAGGTATGCAGATTACAAGAACAATT
AGAAGCTACAGTTCAAAAACTTGAAGAAAGCAAACAGCTTCTAAAAAATAATGAAAAGTTAATCACATGG
TTAAATAAAGAACTAAATGAAAATCAGCTAGTGAGAAAGCAAGATGTGTTGGGACCTTCTGCCACTCCAC
CTGTGCATTCTAGTAGCAACGCAATTAGAAGTGGAATTTCTCCTAACCCTAATGTGGTTGATGGTAGACT
GGCTTACCCAGCTTGTGGGATTGGTTATCCTGTCTCCTCTGCATTTGCTTTCCAGAATACCTTTCCTCAT
CCTTTATCTGCCAAAAATGCCATCCACTCAGTTTCAGGACCAAAGGTTCAGTTTAACTTGCAGTTTACAA
AACCAAATACATCACCAGGAGATGTTCAGTCAGGAACAACTGTTAGTATGCCTCGCTCAACTGATAAGGA
AAATGGTGAAAATTTAGGGCTGGAATCCAAATATCTGAAGAAAAGGGAAGATAGCATTCCTTTACGTGGA
CTCAGCCAAAATCTCTTCAGTAACCCAGACCATCAGAAAGGTGGCCCGTTGGGAGCATTACAGGCACCCT
CAAAGCCAGCGGCGCTGCCTTCCACATCTTCAGCGTATTTCCCGGGACAGTTACCCAACAGT
>Ovis aries XM_004002230.3
ATGAGCCAGATTTTGTTCCAGCAGCTCGTCC
CGTTGCAGGTGAAATGCAGAGACTGCGAGGAGAGGAGAGTGAGTATTAGACTGAGCATTGAACTGCAATC

AGTTTCTAACCCTGTTCACAGAAAGGATTTAGTTATCCGTCTGACTGATGATGCGGATCCATTTTTTCTG
TATAACCTTGTTATTTCAGAGGAAGATTTTCAGAGTTTAAAATTCCAGCAAGGTCTTCTGGTAGACTTCT
TAGCTTTCCCACAAAAATTCATAGATCTCCTTCAGCAGTGTACTCAAGAATATGGGAAAGAAATTCCAAG
GTTTTTGCTGCAGTTAGTTTCAACAGAAGCTATTTTGGATAACTCACCTGCTCTTTTAAATGTGATAGAG
ACAAATCCTTTTAAGCATCTGACACACCTCTCACTAAAACTTTTACCTGGAAATGATGTGGAAATAAAGA
AATTTCTAGCAGACTGTTTGAAATGTAGTAAGGAAGAAAAATTATCATTAACGCAATCATTGGATGATGT
TACCAGGCAACTGAATTTCACCCAAAAGACATTATCAGAAAAGATCCAAGAATTAGATAAGTTACGGAAT
GAATGGGCATCGCACACAGCAACATTGTCAAATAAGCATTCTCAGGAACTGACGAATGAGAAGGAGAAAG
CTTTGCAGGCCCAGGTTCAATACCAACAGCAACATGAACAACAGAAAAAAGATTTAGAAATCCTTCATCA
ACGGAACATCCAGCAGCTACAGAACAGATTGTCTGAGTTAGAAACGGCTAATAAAGACCTCACTGAAAGG
AAATATAAAGGAGACTCCACCATCAGAGAACTTAAAGCAAAGCTGTCTGGTGTTGAAGAGGAGCTCCAGC
GGGCTAAGCAAGAAGTCCTCTCTTTGAGAAGAGAAAATTCTACTCTTGATGCTGAATGCCATGAAAAAGA
AAAGCATATTAATCAGCTACAAACAAAAGTGGCTGTTTTAGAACAGGAAATCAAGGATAAGGACCAGCTT
GTTTTAAGAACAAAAGAAGCGTTTGATACAATCCAGGAACAAAAGGTGGCTCTGGAAGAAAATGGTGAGA
AAAATCAGGTACAACTAGGAAAACTTGAAGCTACAATAAAATCATTATCAGCTGAACTTCTTAAGGCAAA
TGAAATTATCAAAAAACTGCAAGGGGATCTGAAGACTTTAATGGGTAAGTTGAAATTGAAGAATACAGTA
ACTATTCAGCAGGAGAAACTCTTGGCTGAAAAGGAAGAAAAATTACAAAAGGAACAAAAAGAATTACAAG
ATGTTGGACAGTCTCTCCGAATTAAAGAACAAGAGGTATGCAAATTACAAGAACAATTAGAAGCTACAGT
TCAAAAACTTGAAGAAAGCAAACAACTTCTAAAAAATAATGAAAAGTTGATCACGTGGTTAAATAAAGAA
TTAAATGAAAATCAGCTGGTGAGAAAGCACGATGTACTGGGACCTTCTACAGCTCCACCTGTACATTCCA
GCAGCAACACCATCAGAAGTGGAACTTCTCCTACCTCTAATGTGGTTGAGGGTAGACTGACTTACCCAAT
TTGTGGGATTGGTTATCCTGTCTCCTCTGCGTTTGCATTCCAGAATAACTTTTCTCATCCTATATCTGCC
AAAAATACCATCCACCCAGTTTCAGGACCAAAGGTTCAGTTTAACTTGCAGTTTACAAAACCAAATCCAT
CACTAGGAGATGTTCAGTCAGGAACAACTGTTAGTATGCCCTGCTCAACTGACAAGGAAAATGGTGAAAA
TTTAGGACTGGAATCCAAATATCTGAAGAAAAGGGAAGATAGCATTCCTTTGCGTGGACTCAGCCAAAAT
CTATTTAATAATCCAGACCATCAGAAAGATGGCACTTTAGGAGCACTACAGACATCTTCAAAACCCACAG
TGCTCCCTTCTACATCTTCAGCCTATTTTCCTGGGCAGCTCCCGAACAGT

>Odobenus rosmarus divergens XM_004401476.2
ATGAGCCAGGTTCTGTTCCAGGAGCTA
GTCCCGCTGCAGGTGAAATGCAAAGACTGTGAGGAGAGGAGAGTGAATGTTAGAGTGAGCATTGAACTAC
AATCAGTTTCCAATCCAGTTCACAGAAAGGATTTAGTTATCCGTCTGACTGATGATACTGATCCATTTTT
TCTGTATAACCTTGTTATATCTGAGGAAGATTTTCAGAGCTTAAAATTCCAGCAAGGCCTTTTGGTAGAC
TTCTTAGCTTTCCCACAAAAATTTATAGATCTCCTTCAGCAGTGTACTCAAGAACATGCCAAAGAAATTC
CAAGGTTTTTGCTACAGTTAGTTTGTCCAGCACCTCTTTTGGATAACTCACCTGCCTTTTTAAATGTGGT
AGAGACAAATCCTTTTAAGCATCTTACACACCTCTCGCTAAAACTTTTACCTGGAAATGATGTGGAAATA
AAGAAGTTTTTAGCTGGCTGTTTGAAATGTAGCAAGGAAGAAAAATTATCATTGATGCAATCACTAGATG
ATGTTACTAGGCAACTGAATTTCACACAAAAGACATTATCAGAAAAAATCCAAGAATTAGATAAGTTGCA
GAATGAATGGGCATCACATACAGCTGCATTGTCAAATAAACATTCCCAGGAACTGACAAATGAGAAAGAA
AAGGCCTTGCAGGCACAGGTACAGTACCAACAGCAGCATGAGCAACAGAAAAAAGATGTAGAAATCCTCC
ATCAACGAAACATCCAGCAACTACAAAACAGATTATCTGAGTTGGAAGCCGCTAATAAAGACCTAACCGA
AAGGAAATATAAAGGAGACTCTACCATCAGAGAACTTAAAACAAAACTGTCTAGTGTTGAAGAGGAGCTC
CAGCGGGCTAAGCAAGAAGTCCTCTCTTTGCGGAGAGAGAATTCTACACTAGATGCCGAATGCCACGAAA
AAGAAAAGCATATTAATCAGCTACAAACAAAAGTGGCTGTTTTAGAACAGGAAATCAAGGATAAGGACCA
GCTTGTTTTAAGAACAAAAGAAGCATTTGATACAATCCAGGAACAAAAGGTTGCTTTAGAAGAGAATGGT
GAGAAAAATCAGGTACAACTAGGAAAACTTGAAGCTACGATCCAATCATTATCAGCGGAACTTCTTAAGG
CAAATGAAATTATCAAGAAGTTACAGGGGGATCTGAAAACTTTAATGGGTAAATTAAAACTGAAGAATAC

AGTAACTATTCAGCAAGAGAAACTCTTGGCTGAGAAGGAAGACAAATTACAAAAGGAACAAAAGGAATTA
CAAGATGTTGGACAGTCTCTCCGAATTAAAGAGCAAGAGGTGTGCAAATTACAAGAACAGTTAGAAGCTA
CCGTTCAAAAGCTTGAAGAAAGCAAACAACTTCTAAAAAATAATGAAAAGTTAATCACATGGCTAAATAA
AGAACTAAATGAAAATCAGCTAGTGAGAAAGCAAGATGTATTGGGACCTTCTACCACTCCACCTGTACAT
TCTAGCAGCAACACAATCAGAAGTGGGATTTCTCCTAACTCTAATGTGGTTGATGGTAGACTGACTTACC
CAACTTGTGGGATTGGTTATCCTGTCTCCTCTGCATTTGCATTCCAGAATACCTTTCCTCATCCTATATC
TACCAAAAATACCATCCACCCAATTTCAGGACCAAAGGTTCAGTTTAACCTGCAGTTTACAAAACCAAAT
ACATCACTAGGAGATGTTCAGTCAGGAACAACTATTAGTATGCCTTGCTCAACTGATAAGGAAAATGGTG
CAAATTTAGGGCTGGAATCCAAATACCTGAAGAAAAGGGAAGATAGCATTCCTTTACGCGGGCTCAGCCA
AAACCTGTTCAATAATCCAGACCGTCAAAAAGATGGCACTTTAGGAGCATTACAGACATCTTCCAAACCC
GCAGTGCTCCCTTCTGCATCTTCAGCGTATTTCCCTGGACAGTTAACAAACAGT
>equus asinus XM_014851503.1
ATGAGCCAGGTTCTGTTC
CAGGAACTAGTCCCGCTGCAGGTGAAATGCAAAGACTGCGAGGAGAGGAGAGTAAGTGTTAGAGTGAGCG
TTGAACTACAATCAGTTTCTAATCCAGTTCACAGAAAGGATTTAGTTATCCGTCTGACTGATGATACTGA
CCCATTTTTTCTGTATAACCTTGTTATATCTGAGGAGGATTTTCAGAGTTTAAAATTCCAGCAAGGTCTT
CTGGTAGACTTCTTAGCTTTCCCACAAAAATTTATAGATCTCCTTCAGCAATGTACTCAAGAACATACCA
AAGAAATTCCAAGGTTTTTGCTACAGTTAGTTTCTCCAGCTGCTATTTTGGATAACTCACCTGCCTTTTT
AAATGTGGTAGAGACAAATCCTTTTAAACATCTTACACACCTCTCACTGAAAGTTTTACCTGGAAATGAT
GTGGAAATAAAGAAGTTTCTAGCAGGCTGTTTGAAATGTAGCAAGGAAGAAAAACTATCATTGGCACAAT
CACTAGAGGATGTTACTAGGCAACTGAATTTCACACAAAAGACATTATCAGAAAAAATCCAAGAATTAGA
TAAGTTACGGAATGAATGGGCATCACACACAACAGCATTGTCAAATAAGCATTCTCAGGAACTGACAAAT
GAGAAGGAAAAAGCCTTACAGGCACAGGTTCAGTACCAACAGCAGCATGAACAACAGAAGAAAGATTTAG
AAATGCTCCACCAGCGGAACATCCAGCAGTTACAGAGCAGATTGTCTGAGTTGGAAGCAGCTAATAAAGA
CCTAACCGAAAGGAAGTATAAAGGAGACTCCACCATCAGAGAACTTAAAGCAAAACTATCTGGTGTTGAA
GAGGAGCTTCAGCGGGCTAAGCAAGAAGTCCTCTCTTTGCGAAGAGAGAATTCTACACTAGATGCTGAAT
GCCATGAAAAAGAAAAGCATATTAATCAGCTACAAACAAAAGTGGCTGTTTTAGAACAGGAAATCAAGGA
TAAGGACCAGCTTGTTTTAAGAACAAAGGAAGCATTTGATACAATCCAGGAACAAAAGGTGGCTTTAGAA
GAAAATGGTGAGAAAAATCAGGTACAACTAGGAAAACTTGAAGCTACAATAAAATCACTATCTGCTGAAC
TTCTTAAGGCAAATGAAATTATCAAGAAATTACAAGGGGATCTGAAGACTTTAATGGGTAAATTGAAACT
GAAGAATACAGTAACTATTCAGCAAGAGAAACTCCTGGCTGAGAAGGAAGAAAAATTACAAAAGGAACAA
AAGGAATTACAAGATGTTGGACAGTCTCTCAGAATTAAAGAGCAAGAGATATGCAAATTACAAGAACAGT
TAGAAGCCACGGTTCAAAAACTTGAAGAAAGCAAACAGCTTCTAAAAAATAATGAAAAGTTAATCACATG
GTTAAATAAAGAACTAAATGAAAATCAGCTAGTGAGAAAGCAAGATGTATTGGGACCTTCTGCCACTCCA
CCCGTACATTCTAGTAGCAACACAATCAGAAGTGGAATTTCTCCTAACTCTAATGTGGTTGATGGTAGAC
TGACTTACCCATCTTGTGGGATGGGTTATCCTGTCTCCTCTGCATTTGCATTCCAGAATACCTTTCCTCA
TCCTATATCTGCCAAAAATACCATCCACCCAGTTTCAGGACCAAAGGTTCATTTTAACTTGCAGTTTACA
AAACCAAACACATCACTAGGAGATGTTCAGTCAGGAACAACTGTTAATATGCCTTGCTCAACTGACAAGG
AAAATGGTGAAAATTTAGGGCTGGAATCCAAATATCTGAAGAAAAGGGAAGGTAGCATTCCTTTACGTGG
GCTCAGCCAGAATCTATTTAATAATGCAGACCAGCAGAAAGATGGCACTGTAGGAGGAGCATTACAGACA
GCTTCGAAACCCGCAGTGCTCCCCCCGGCTGCTTCAGCCTATTTCCCTGGGCAGTTACCAAACAGT
>equus caballus XM_001488828.3
ATGAGCCAGGTTCTGTTCCAGGAA
CTAGTCCCGCTGCAGGTGAAATGCAAAGACTGCGAGGAGAGGAGAGTAAGTGTTAGAGTGAGCGTTGAAC
TACAATCAGTTTCTAATCCAGTTCACAGAAAGGATTTAGTTATCCGTCTGACTGATGATACTGATCCATT
TTTTCTGTATAACCTTGTTATATCTGAGGAGGATTTTCAGAGTTTAAAATTCCAGCAAGGTCTTCTGGTA
GACTTCTTAGCTTTCCCACAAAAATTTATAGATCTCCTTCAGCAATGTACTCAAGAACATACCAAAGAAG

TTCCAAGGTTTTTGCTACAGTTAGTTTCTCCAGCTGCTATTTTGGATAACTCACCTGCCTTTTTAAATGT
GGTAGAGACAAATCCTTTTAAACATCTTACACCTCTCACTGAAAGTTTTACCTGGAAATGATGTGGAA
ATAAAGAAGTTTCTAGCAGGCTGTTTGAAATGTAGCAAGGAAGAAAAACTATCATTGGCACAATCACTAG
AGGATGTTACTAGGCAACTGAATTTCACACAAAAGACATTATCAGAAAAAATCCAAGAATTAGATAAGTT
ACGGAATGAATGGGCATCGCACACAACAGCATTGTCAAATAAGCATTCTCAGGAACTGACAAATGAGAAG
GAAAAAGCCTTACAGGCACAGGTTCAGTACCAACAGCAGCATGAACAACAGAAGAAAGATTTAGAAATGC
TCCACCAGCGGAACATCCAGCAGTTGCAGAGCAGATTGTCTGAGTTGGAAGCAGCTAATAAAGACCTAAC
CGAAAGGAAGTATAAAGGAGACTCCACCATCAGAGAACTTAAAGCAAAACTATCTGGTGTTGAAGAGGAG
CTTCAGCGGGCTAAGCAAGAAGTCCTCTCTTTGCGAAGAGAGAATTCTACACTAGATGCTGAATGCCATG
AAAAAGAAAAGCATATTAATCAGCTACAAACAAAAGTGGCTGTTTTAGAACAAGAAATCAAGGATAAGGA
CCAGCTTGTTTTAAGAACAAAGGAAGCATTTGATACAATCCAGGAACAAAAGGTGGCTTTAGAAGAAAAT
GGTGAGAAAAATCAGGTACAACTAGGAAAACTTGAAGCTACAATAAAATCATTATCTGCTGAACTTCTTA
AGGCAAATGAAATTATCAAGAAATTACAAGGGGATCTGAAGACTTTAATGGGTAAATTGAAACTGAAGAA
TACAGTAACTATTCAGCAAGAGAAACTCCTGGCTGAGAAGGAAGAAAAATTACAAAAGGAACAAAAGGAA
TTACAAGATGTTGGACAGTCTCTCAGAATTAAAGAGCAAGAGATATGCAAATTACAAGAACAGTTAGAAG
CCACGGTTCAAAAACTTGAAGAAAGCAAACACCTTCTAAAAAATAATGAAAAGTTAATCACGTGGTTAAA
TAAAGAACTAAATGAAAATCAGCTAGTGAGAAAGCAAGATGTATTGGGACCTTCTGCCACTCCACCCGTA
CATTCTAGTAGCAACACAATCAGAAGTGGAATTTCTCCTAACTCTAATGTGGTTGATGGTAGACTGACTT
ACCCATCTTGTGGGATGGGTTATCCTGTCTCCTCTGCATTTGCATTCCAGAATACCTTTCCTCATCCTAT
ATCTGCCAAAAATACCATCCACCCAGTTTCAGGACCAAAGGTTCATTTTAACTTGCAGTTTACAAAACCA
AACACATCACTAGGAGATGTTCAGTCAGGAACAACTGTTAATATGCCTTGCTCAACTGACAAGGAAAATG
GTGAAAATTTAGGGCTGGAATCCAAATATCTGAAGAAAAGGGAAGGTAGCATTCCTTTACGTGGGCTCAG
CCAGAATCTATTTAATAATGCAGACCAGCAGAAAGATGGCACTGTAGGAGGAGCATTACAGACAGCTTCG
AAACCCGCAGTGCTCCCCCCGGCTGCTTCAGCCTATTTCCCTGGGCAGTTACCAAACAGT
>orcinus orca XM_004263075.1
ATGAGCCAGGTTCTC
TTCCAGCACCTCGTCCCGTTGCAAGTGAAATGCAAAGACTGCGAGGAGAGGAGAGTGAGTATTAGACTGA
GCATTGAACTACAATCAGTTTCTAATCCAGTTCACAGAAAGGATTTAGTTATCCGTCTGACTGATGATAC
TGATCCATTTTTTCTGTATAACCTTGTTATATCAGAGGAAGATTTTCAGAGTTTAAAATTCCAGCAAGGT
CTTCTGATAGACTTCTCAGCTTTCCCACAAAAATTCATAGATCTCCTTCAGCAGTGTACTCAAGAACATG
CCAAAGAAATTCCAAGGTTTTTGCTACAGTTAGTTTCTCCAGAAGCTATTTTGGATAACTCACCTGCTTT
TTTAAATGTGGTAGAGACAAATCCTTTTAAGCATCTTACACACCTTTCATTAAAACTGTTACCTGGAAAT
GATGTGGAAATAAAGAAGTTTCTAGCAGACTGTTTGAAATGTAGCAAGGAAGAAAAATTATCATTGACAC
AATCACTAGATGATGTTACCAGGCAACTGAATTTCACACAAAAGACATTATCAGAAAAAATCCAAGAATT
AGATAAGTTACGGAATGAATGGGCATCACACACAGCAACATTGTCAAATAAGCATTCTCAAGAACTGACA
AATGAGAAGGAAAAAGCTTTGCAGGCACAGGTTCGATACCAACAGCAGCATGAACAACAGAAAAAAGATT
TAGAAATCCTCCATCAACGAAACATCCAGCAGCTACAAAACAGATTGTCTGAGTTAGAATCTGCTAATAA
AGACCTCACTGAAAGGAAATATAAAGGAGACTCTACCATCAGAGAACTTAAAACAAAACTGTCTGGTGTT
GAAGAGGAGCTCCAGCGGGCTAAGCAAGAAGTCCTCTCTTTGCGAAGAGAGAATTCTACACTTGATGCTG
AGTGCCATGAAAAAGAAAAGCATATTAATCATCTACAAACAAAAGTGGCTGTTTTAGAACAGGAAATCAA
GGATAAGGATCAGCTTGTTTTAAGAACAAAAGAAGCATTTGATACAATCCAGGAACAAAAGGTGGCTTTA
GAAGAAAATGGTGAGAAAAATCAGGTACAACTAGGAAAACTTGAAGCTACAATAAAATCATTATCAGCTG
AACTTCTTAAGGCAAATGAAATTATCAAAAAGTTGCAAGGGGATCTGAAGACCTTAATGGGTAAATTGAA
ACTGAAGAATACAGTAACTATTCAGCAAGAGAAACTCTTGGCTGAGAAGGAAGAAAAATTACAAAAGGAA
CAAAAAGAATTACAAGATGTTGTACAGTCTCTCCGAATTAAAGAACAAGAGGTATGCAAATTACAAGAAC
AATTAGAAGCTACAGTTCAAAAACTTGAAGAAAGCAAACAGCTTCTAAAAAATAATGAGAAGTTAATCAC
ATGGTTAAATAAAGAATTAAATGAAAATCAGCTAGTGAGAAAGCAAGATGTATTGGGACCTTCAACCACT

CCACCTGTACATTCTAGTAGCAACACCATCAGAAGTGGAATTTCTCCTAACTCTAATGTGGTTGATAGTA
GACTGACTTACCCAACTTGTGGGATTGGTTATCCTGTCTCCTCTGCATTTGCATTCCAGAATACCTTTCC
TCATCCTATAGCTGCCAAAAATAGCATCCACCCAGTTTCAGGACCAAAGGTGCAGTTTAACTTGCAGTTT
ACAAAACCAAGTACATCACTAGGAGATGTTCAATCAGGAACAACTGTTAGTATGCCTTGCTCAACTGATA
AGGAAAATGCTGAAAATTTAGGGCTGGAATCCAAATATCTGAAGAAAAGGGAAGACAGCATTCCTTTACG
TGGGCTCAACCAAAATCTATTCAATAATCCAGACCATCAAAAAGACGGCACTTTAGGAGCATTACAGACA
TCTTCCAAACCCGTAGTCCTCCCTTCTACATCTTCAGCATATTTCCCTGGGCAGCTACCAAACAGT
>Lipotes vexillifer XM_007449607.1
ATGAGCCAGGTTCTGTTCCAGCAGCTCGTCCCGTTGCAAGTGAAATGCA
GAGACTGCGAGGAGAGGAGAGTGAGTATTAGACTGAGCATTGAACTACAATCAGTTTCTAATCCAGTTCA
CAGAAAGGATTTAGTTATCCGTCTGACTGATGATACTGATCCATTTTTTCTGTATAACCTTGTTATATCA
GAGGAAGATTTTCAGAGTTTAAAATTCCAGCAAGGTCTTCTGATAGACTTCTCAGCTTTCCCACAAAAAT
TCATAGATCTCCTTCAGCAGTGTACTCAAGAACATGCCAAAGAAATTCCAAGGTTTTTGCTACAGTTAGT
CTCTCCAGAAGCTATTTTGGATAACTCACCTGCTTTTTTAAATGTGGTAGAGACAAATCCTTTTAAGCAT
CTTACACACCTTTCATTAAAACTGTTACCTGGAAATGATGTGGAAATAAAGAAGTTTCTAGCAGACTGTT
TGAAATGTAGCAAGGAAGAAAAATTATCATTGACACAATCACTAGATGATGTTACCAGGCAACTGAATTT
CACACAAAAGACATTATCAGAAAAAATCCAAGAATTAGATAAGTTACGGAATGAATGGGCATCACACACA
GCAACATTGTCAAATAAGCATTCTCAAGAACTGACGAATGAGAAGGAAAAGGCTTTGCAGGCACAGGTTC
GATACCAACAGCAGCATGAACAACAGAAAAAAGATTTAGAAATCCTCCATCAACGAAACATCCAGCAGCT
ACAAAACAGATTGTCTGAGTTAGAATCTGCTAATAAAGACCTCACTGAAAGGAAATATAAAGGAGACTCT
GCCATCAGAGAACTTAAAACAAAACTGTCTGGTGTTGAAGAGGAGCTCCAGCGGGCTAAGCAAGAAGTCC
TCTCTTTGCGAAGAGAGAATTCTACACTTGATACTGAGTGCCATGAAAAAGAAAAGCATATTAATCATCT
ACAAACAAAAGTGGCTGTTTTAGAACAGGAAATCAAGGATAAGGATCAGCTTGTTTTAAGAACAAAAGAA
GCATTTGATACAATCCAGGAACAAAAGGTGGCTTTAGAAGAAAATGGTGAGAAAAATCAGGTACAACTGG
GAAAACTTGAAGCTACAATAAAATCATTATCAGCTGAACTTCTTAAGGCAAATGAAATTATCAAAAAGTT
GCAAGGGGATCTGAAGACCTTAATGGGTAAATTGAAACTGAAGAATACAGTAACTATTCAGCAGGAGAAA
CTCTTGGCTGAGAAGGAAGAAAAATTACAAAAGGAACAAAAAGAATTGCAAGATGTTGTACAGTCTCTCC
GAATTAAAGAGCAAGAGGTATGCAAATTACAAGAACAATTAGAAGCTACAGTTCAAAAACTTGAAGAAAG
CAAACAGCTTCTAAAAAATAATGAGAAGTTAATCACATGGTTAAATAAAGAGTTAAATGAAAATCAGCTA
GTGAGAAAGCAAGATGTATTGGGACCTTCAACCACTCCACCTGTACATTCTAGTAGCAACACCATCAGAA
GTGGAATTTCTCCTAACTCTAATATGGTTGATAGTAGACTGACTTACCCAACTTGTGGGATTGGTTATCC
TGTCTCCTCTGCATTTGCATTCCAGAATACCTTTCCTCATCCTATAGCTGCCAGAAATACCATCCACCCA
GTTTCAGGACCAAAGGTGCAGTTTAACTTGCAGTTTACAAAACCAAGTACATCACTAGATGTTCAATCAG
GAACAACTGTTAGTATGCCTTGCTCAACTGATAAGGAAAATGGTGAAAATTTAGGGCTGGAATCCAAATA
TCTGAAGAAAAGGGAAGACAGCATTCCTTTACGTGGGCTTAACCAAAATCTATTTAATAATCCAGACCAT
CAAAAAGACGGCACTTTAGGAGCATTACAGACATCTTCCAAACCCGTAGTGCTCCCTTCTACATCTTCAG
CATATTTCCCTGGGCAGCTACCAAACAGT
>Manis javanica XM_017672517.1
ATGAGCCAGGTTCTGTTTCAGCAGC
TAGTCCCGTTGCAGGTGAAATGCAAAGACTGCGAAGAGAGGAGAGTAAGTGTTAGAGTAAGCATTGAACT
ACAATCAGTTTCTAATCCAGTTCATAGAAAGGATTTAGTTATTCGTCTGACTGATGATACTGACCCATTT
TTTCTGTATAACCTTGTTATATCTGAGGAAGATTTTCAGAGTTTAAAATTCCAACAAGGTCTTCTGGTGG
ACTTCTTAGCTTTCCCACAAAAATTTGTAGATCTCCTTCAGCAGTGTACTCAAGAACATGTCAAAGAAAT
TCCAAGGTTTTTGTTACAGTTAGTTTCTCCAGCAACTATTTTGGATAACTCACCTGCCTTTTTAAATGTG
GTAGAGACAAATCCTTTCAAGCATCTTACGCATCTCTCACTAAAACTTTTACCTGGGAATGATGTGGAAA
TAAAGAAGTTTCTAGCAAGCTGTTTGAAATGTAGCAAGGAAGAAAAAATATCATTGACGCAATCACTAGA
TGATGTTACTAGGCAACTAAATTTCACACAAAAGACATTATCAGAGAAAATCCAAGAATTAGATAAATTA

CAGAATGAATGGTCATCACACACAGCAGCATTGTCAAATAAGCATTCTCAGGAACTGACAAATGTGAAGG
AAAAAGCTTTGCAGGCACAGGTTCAATACCAACAGCAGTATGAACAACAAAAGAAAGATTTAGAAGTCCT
CCATCAACGAAACATCCAGCAGCTACAAAACAGATTGTCTGAGTTAGAAGCGGCTAATAAAGACTTAACT
GAAAGGAAATATAAAGGAGACTCTACTGTCAGAGAACTTAAATCAAAACTGTCTGGTGTTGAAGAGGAGC
TCCAGCGGGCTAAGCAAGAAGTCCTCTCTTTGCGAAGAGAGAATTCTACACTAGATGCTGAATGCCATGA
AAAGGAAAAGCTTATTAATCAGCTACAAACAAAAGTGGCTGTTTTAGAACAGGAAATCAAGGATAAGGAC
CAGCTTGTTTTAAGAACAAAAGAAGCATTTGATACAATTCAAGAACAAAAGGTGGCTTTAGAAGAAAATG
GTGAGAAAAATCAGGTTCAACTAGGAAAACTTGAAGCTACAATAAAATCATTGTCAGCAGAACTTCTCAA
GGCAAATGAAATTATCAAGAAGTTACAAGGGAATCTGAAAACTTTAATGAGTAAATTGAAACTGAAGAAT
ACAGTAACAATTCAGCAAGAGAAACTCTTGGCTGAGAAGGAAGAAAAATTACAAAAGGAACAAAAGGAAT
TACAAGATGTTGGACAGTCTCTCCGAATTAAAGAGCAAGAGGTATGCAAATTGCAGGAACAATTAGAAGC
TACAGTTCAAAAACTAGAAGAAAGCAAACAGCTTCTAAAAAATAATGAAAAATTGATCACATGGTTAAAT
AAAGAACTAAATGAAAATCAGCTGGTGAGAAAGCCAGATGTGTTGGGATCTTCTACCACTCCGCCTGTAC
ACTCTAGTAGCAACACAGTCAGAAGTGGAATTTCTCCTAATTCTAATGTGGTTGATGGTAGACTGACTTA
CCCAACCTGTGGGATTGGTTATCCTTTCTCATCTGCATTTGCATTCCAGAATACATTTCCTCATCCTTTA
TCTGCTAAAAATACTATCCACCCGGTTTCAGGACCAAAGGTTCAGTTTAACTTGCAGTTTACAAAACCAA
ACACATCACTAGGAGATACTCACTCGGGAACAACTAGTAATGTGCTTTGCTCAACTGATAAGGAAAATGG
TGAAAATTTAGGGCTGGAATCCAAGTATCTGAAGAAAAGGGAAGATAGCATTCCTTTACGTGGACTCAGC
CAAAATCTATTTAATAATGCAGACCATCAAAAAGATGGCATTGTAGGAGCATCACAGACATCTTCAAAAC
CCACAGTCCTCCCTTCTGCATCTTCAGCTTATTTCCCTGGGCAGTTATCAAACAGT

MFSD2A

>human ENST00000372811.9
ATGGCCAAAGGAGAAGGCGCCGAGAGCGGCTCCGCGGCGGGGCTGCTACCCACCAGCATC
CTCCAAAGCACTGAACGCCCGGCCCAGGTGAAGAAAGAACCGAAAAAGAAGAAACAACAG
TTGTCTGTTTGCAACAAGCTTTGCTATGCACTTGGGGGAGCCCCCTACCAGGTGACGGGC
TGTGCCCTGGGTTTCTTCCTTCAGATCTACCTATTGGATGTGGCTCAGGTGGGCCCTTTC
TCTGCCTCCATCATCCTGTTTGTGGGCCGAGCCTGGGATGCCATCACAGACCCCCTGGTG
GGCCTCTGCATCAGCAAATCCCCCTGGACCTGCCTGGGTCGCCTTATGCCCTGGATCATC
TTCTCCACGCCCCTGGCCGTCATTGCCTACTTCCTCATCTGGTTCGTGCCCGACTTCCCA
CACGGCCAGACCTATTGGTACCTGCTTTTCTATTGCCTCTTTGAAACAATGGTCACGTGT
TTCCATGTTCCCTACTCGGCTCTCACCATGTTCATCAGCACCGAGCAGACTGAGCGGGAT
TCTGCCACCGCCTATCGGATGACTGTGGAAGTGCTGGGCACAGTGCTGGGCACGGCGATC
CAGGGACAAATCGTGGGCCAAGCAGACACGCCTTGTTTCCAGGACCTCAATAGCTCTACA
GTAGCTTCACAAAGTGCCAACCATACACATGGCACCACCTCACACAGGGAAACGCAAAAG
GCATACCTGCTGGCAGCGGGGGTCATTGTCTGTATCTATATAATCTGTGCTGTCATCCTG
ATCCTGGGCGTGCGGGAGCAGAGAGAACCCTATGAAGCCCAGCAGTCTGAGCCAATCGCC
TACTTCCGGGGCCTACGGCTGGTCATGAGCCACGGCCCATACATCAAACTTATTACTGGC
TTCCTCTTCACCTCCTTGGCTTTCATGCTGGTGGAGGGGAACTTTGTCTTGTTTTGCACC
TACACCTTGGGCTTCCGCAATGAATTCCAGAATCTACTCCTGGCCATCATGCTCTCGGCC
ACTTTAACCATTCCCATCTGGCAGTGGTTCTTGACCCGGTTTGGCAAGAAGACAGCTGTA
TATGTTGGGATCTCATCAGCAGTGCCATTTCTCATCTTGGTGGCCCTCATGGAGAGTAAC
CTCATCATTACATATGCGGTAGCTGTGGCAGCTGGCATCAGTGTGGCAGCTGCCTTCTTA
CTACCCTGGTCCATGCTGCCTGATGTCATTGACGACTTCCATCTGAAGCAGCCCCACTTC
CATGGAACCGAGCCCATCTTCTTCTCCTTCTATGTCTTCTTCACCAAGTTTGCCTCTGGA
GTGTCACTGGGCATTTCTACCCTCAGTCTGGACTTTGCAGGGTACCAGACCCGTGGCTGC
TCGCAGCCGGAACGTGTCAAGTTTACACTGAACATGCTCGTGACCATGGCTCCCATAGTT

CTCATCCTGCTGGGCCTGCTGCTCTTCAAAATGTACCCCATTGATGAGGAGAGGCGGCGG
CAGAATAAGAAGGCCCTGCAGGCACTGAGGGACGAGGCCAGCAGCTCTGGCTGCTCAGAA
ACAGACTCCACAGAGCTGGCTAGCATCCTC
>marmoset ENSCJAT00000005231.2
ATGGCCAAAGGAGAAGGCGCCGAGAGCGGCTCCGCGGCGGGGCTGCTGCCCACCAGCATC
CTACAAGCCAGTGAACGCCCTGCCCAGGTGAAGAAGGAACCAAAAAAGAAGAAACAACAG
TTGTCCGTGTGCAACAAGCTTTGCTATGCAGTTGGGGGAGCCCCTTACCAGGTGACGGGC
TGTGCCCTGGGTTTCTTCCTTCAGATCTACCTATTGGATGTGGCTCAGGTGGGCCCTTTCTCTGCCTCTATT
ATCCTGTTT
GTGGGCCGAGCCTGGGATGCCATCACAGACCCCCTGGTGGGCTTCTGCATCAGCAAATCC
TCCTGGACCTGCCTGGGTCGTCTTATGCCCTGGATCATCTTCTCCACACCCCTGGCCGTC
ATAGCCTACTTCCTCATCTGGTTCGTGCCTGACTTCCCACAGGGCCAGACCTATTGGTAC
CTTTTCTATTGCCTCTTTGAGACGCTGGTCACGTGTTTCCACGTTCCCTACTCGGCTCTT
ACCATGTTCATCAGCACCGAGCAGACTGAGCGGGATTCGGCCACCGCGTATCGGATGACT
GTGGAAGTGCTGGGCACAGTGCTGGGCACGGCGATCCAGGGGCAAATTGTGGGCCAAGCA
GACATGCCTTGTTTCCAGGACCACAATAGCTCTGCAGAGGCTTCACAAAGTACCAACCGT
ACACATTACATCACCTCACACAGAGAAACGCAAAATGCATACCTGCTGGCAGCGGGGGTC
ATTGCCTCTATCTATGTCATCTGTGCTGTCATCCTGACCCTGGGTGTGCAGGAGCGGAGA
GAACCCTATGAGGCCCAGCAGACTGAGCCGATCGCCTACTTCCAGGGCCTCCGGCTGGTC
ATGAGCCACGGCCCATACATCAAGTTGATTACCGGCTTCCTCTTCACCTCCTTGGCTTTC
ATGCTGGTGGAGGGGAACTTTGTCTTGTTTTGCACCTATACCTTGGGCTTCCGCAACGAA
TTCCAGAATCTACTCCTGGCCATCATGCTCTCCGCTACCTTCACCATTCCCATCTGGCAG
TGGTTCCTGACCCGGTTTGGCAAGAAGACAGCTGTCTATGTTGGGATCTCATCAGCAGTA
CCATTTCTCATCTTGGTGGCCCTTATGGAGAGTAACCTCATTGTCACATATGTGGTAGCT
GTGGCAGCTGGCATCAGTGTGGCAGCTGCCTTCTTATTACCCTGGTCCATGCTGCCTGAC
GTCATTGATGACTTCCATCTGAAGCAGCCCCACTTCCATGGAACCGAGCCCATCTTCTTC
TCCTTCTATGTCTTCTTTACCAAGTTTGCCTCTGGAGTGTCACTGGGCATTTCCACCCTC
AGTCTCGACTTTGCAGGGTACCAGACCCTTAGCTGCTCCCAGCCGGAACGTGTTAAGTTT
ACACTGAACATGCTCGTGACCATGGCACCCATAGTTCTCATCCTGCTGGGCCTGCTGCTC
TTCAAACTGTACCCCATTGATGAGGAGAGGCGGCGGCAGAATAAGAAAGCCTTGCAGGCA
CTGAGGGATGAAGCCAGCAGCTCTGGCTGCTCAGAAACAGACTCCACAGAGCTGGCTAGC
ATCCTC
>vervet agm ENSCSAT00000017394.1
ATGGCCAAAGGAGAAGGCGCCGAGAGCGGCTCCGCGGCAGGGCTGCTGCCCACCAGCATC
CTCCAAGCCACTGAACGCCCGGCCCAGGTGAAGAAAGAACCGAAAAAGAAGAAACAACAG
TTGTCTGTTTGCAGCAAGCTTTGCTATGCACTTGGGGGAGCCCCCTACCAGGTCACAGGC
TGTGCCCTGGGTTTCTTCCTTCAGATCTACCTATTGGATGTGGCTCAGGTGGGCCCTTTT
TCTGCCTCCATCATCCTGTTTGTGGGCCGAGCCTGGGATGCCATCACAGACCCCCTGGTG
GGCTTCTGCATCAGCAAATCCTCCTGGACCTGCCTGGGTCGCCTTATGCCTTGGATCATC
TTCTCCACGCCCCTGGCCGTCATCTCCTACTTCCTCATCTGGTTCGTGCCCGACTTCCCA
CACGGCCAGACCTATTGGTACCTGCTTTTCTATTGCCTCTTTGAGACGATGGTCACGTGT
TTCCATGTTCCCTACTCGGCTCTCACCATGTTCATCAGCACCGAGCAGACTGAGCGGGAT
TCTGCCACCGCCTATCGGATGACTGTGGAAGTGCTGGGCACAGTGCTGGGCACGGCAATC
CAGGGGCAAATCGTGGGCCAAGCAGACACGCCTTGTTTCCAGGACCTCAATGGCTCTACA
GTGGCTTCACAAAGTGCCAACCATACACATGGCACCACCTCACACAGAGAAACGCAAAAG
GCATACCTGCTGGCAGCGGGGGTTATCATCTGTATCTATGTAATCTGTGCTGTCATCCTG
ACCCTGGGCGTGCGGGAGCAGAGAGAACCCTATGAAGCCCAGCAGGCTGAGCCGATCGCC

TACTTCCAGGGCCTACGGCTGGTTATGAGCCATGGCCCATACGTCAAGCTTATTACCGGC
TTCCTCTTCACCTCCTTGGCTTTCATGCTGGTGGAGGGGAACTTTGTCTTGTTTTGCACC
TATACCTTGGGCTTCCGCAATGAATTCCAGAATCTACTCCTGGCCATCATGCTCTCAGCC
ACTTTCACCATTCCCATCTGGCAGTGGTTCCTGACCCGGTTTGGCAAGAAGACAGCTGTG
TATATTGGGATCTCATCAGCAGTGCCATTTCTCGTCTTGGTGGCCCTCATGGAGAGTAAC
CTCATCATCACATATGTGGTAGCTGTGGCAGCTGGCATCAGTGTGGCAGCTGCCTTCTTA
CTACCCTGGTCCATGCTGCCTGACGTCATTGATGACTTCCATCTGAAGCAGCCTCACTTC
CACGGAACCGAGCCCATCTTCTTCTCCTTCTATGTCTTCTTCACCAAGTTTGCCTCTGGA
GTGTCACTGGGCATTTCCACCCTCAGTCTGGACTTTGCAGGGTACCAGACCCGTGGCTGC
TCGCAGCCGGAACGTGTCAAGTTTACACTGAACATGCTCGTGACCATGGCTCCCATAGTT
CTCATCCTGCTGGGCCTGCTGCTCTTCAAACTGTACCCCATAGATGAGGAGAGGCGGCGG
CAGAATAAGAAGGCCCTACAGGCACTGAGGGACGAGGCCAGCAGCTCTGGCTGCTCAGAA
ACAGACTCCACAGAGCTGGCTAGCATCCTC
>gorilla XM_004025524.1
ATGGCCAAAGGAGAAGGCGCCGAGAGCGG
CTCCGCGGCGGGGCTGCTGCCCACCAGCATCCTCCAAAGCACTGAACGCCCGGCCCAGGTGAAGAAAGAA
CCGAAAAAGAAGAAACAACAGTTGTCTGTTTGCAACAAGCTTTGCTATGCACTTGGGGGAGCCCCCTACC
AGGTGACGGGCTGTGCCCTGGGTTTCTTCCTTCAGATCTACCTATTGGATGTGGCTCAGGTGGGCCCTTT
CTCTGCCTCCATCATCCTGTTTGTGGGCCGAGCCTGGGATGCCATCACAGACCCCCTGGTGGGCCTCTGC
ATCAGCAAATCCCCCTGGACCTGCCTGGGTCGCCTTATGCCCTGGATCATCTTCTCCACGCCCCTGGCCG
TCATTGCCTACTTCCTCATCTGGTTTGTGCCCGACTTCCCACACGGCCAGACCTATTGGTACCTGCTTTT
CTATTGCCTCTTTGAGACAATGGTCACGTGTTTCCATGTTCCCTACTCGGCTCTCACCATGTTCATCAGC
ACCGAGCAGACTGAGCGGGATTCTGCCACCGCCTATCGGATGACTGTGGAAGTGCTGGGCACAGTACTGG
GCACGGCGATCCAGGGGCAAATCGTGGGCCAAGCAGACACGCCTTGTTTCCAGGACCTCAATAGCTCTAC
AGTAGCTTCACAAAGTGCCAACCATACACATGGCACCACCTCACACAGGGAAACGCAAAAGGCATACCTG
CTGGCAGCGGGGGTCATTGTCTGTATCTATATAATCTGTGCTGTCATCCTGATCCTGGGCGTGCGGGAGC
AGAGAGAACCCTATGAAGCCCAGCAGGCTGAGCCGATCGCCTACTTCCGGGGCCTACGGCTGGTCATGAG
CCACGGCCCATACATCAAACTTATTACCGGCTTCCTCTTCACCTCCTTGGCTTTCATGCTGGTGGAGGGG
AACTTTGTCTTGTTTTGCACCTACACCTTGGGCTTCCGCAATGAATTCCAGAATCTACTCCTGGCCATCA
TGCTCTCGGCCACTTTAACCATTCCCATCTGGCAGTGGTTCTTGACCCGGTTTGGCAAGAAGACAGCTGT
ATATGTTGGGATCTCATCAGCAGTGCCATTTCTCATCTTGGTGGCCCTCATGGAGAGTAACCTCATCATT
ACATATGCGGTAGCTGTGGCAGCTGGCATCAGTGTGGCAGCTGCCTTCTTACTACCCTGGTCCATGCTGC
CTGATGTCATTGACGACTTCCATCTGAAGCAGCCCCACTTCCATGGAACCGAGCCCATCTTCTTCTCCTT
CTATGTCTTCTTCACCAAATTTGCCTCTGGAGTGTCACTGGGCATTTCTACCCTCAGTCTGGACTTTGCA
GGGTACCAGACCCGTGGCTGCTCGCAGCCGGAACGTGTCAAGTTTACGCTGAACATGCTCGTGACCATGG
CTCCCATAGTTCTCATCCTGCTGGGCCTGCTGCTCTTCAAAATGTACCCCATTGATGAGGAGAGGCGGCG
GCAGAATAAGAAGGCCCTGCAGGCACTGAGGGACGAGGCCAGCAGCTCTGGCTGCTCAGAAACAGACTCC
ACAGAGCTGGCTAGCATCCTC
>bonobo XM_003815415.2
ATGGCCAAAGGAGAAGGCGCCGAGAGCGG
CTCCGCGGCGGGGCTGCTGCCCACCAGCATCCTCCAAAGCACTGAACGCCCGGCCCAGGTGAAGAAAGAA
CCGAAAAAGAAGAAACAACAGTTGTCTGTTTGCAACAAGCTTTGCTATGCACTTGGGGGAGCCCCCTACC
AGGTGACGGGCTGTGCCCTGGGTTTCTTCCTTCAGATCTACCTACTGGATGTGGCTCAGGTGGGCCCTTT
CTCTGCCTCCATCATCCTGTTTGTGGGCCGAGCCTGGGATGCCATCACAGACCCCCTGGTGGGCCTCTGC
ATCAGCAAATCCCCCTGGACCTGCCTGGGTCGCCTTATGCCCTGGATCATCTTCTCCACGCCCCTGGCTG
TCATTGCCTACTTCCTCATCTGGTTCGTGCCCGACTTCCCACACGGCCAGACCTATTGGTACCTGCTTTT
CTATTGCCTCTTTGAGACAATGGTCACGTGTTTCCATGTTCCCTACTCGGCTCTCACCATGTTCATCAGC

ACCGAGCAGACTGAACGGGATTCTGCCACCGCCTATCGGATGACTGTGGAAGTGCTGGGCACAGTGCTGG
GCACGGCGATCCAGGGGCAAATCGTGGGCCAAGCAGACACGCCTTGTTTCCAGGACCTCAATAGCTCTAC
AGTAGCTTCACAAAGTGCCAACCATACACATGGCACCACCTCACACAGGGAAACGCAAAAGGCATACCTG
CTGGCAGCGGGGGTCATTGTCTGTATCTATATAATCTGTGCTGTCATCCTGATCCTGGGCGTGCGGGAGC
AGAGAGAACCCTATGAAGCCCAGCAGGCTGAGCCGATTGCCTACTTCCGGGGCCTACGGCTGGTCATGAG
CCACGGCCCATACATCAAACTTATTACCGGCTTCCTCTTCACCTCCTTGGCTTTCATGCTGGTGGAGGGG
AACTTTGTCTTGTTTTGCACCTACACCTTGGGCTTCCGCAATGAATTCCAGAATCTACTCCTGGCCATCA
TGCTCTCGGCCACTTTAACCATTCCCATCTGGCAGTGGTTCTTGACCCGGTTTGGCAAGAAGACAGCTGT
ATATGTTGGGATCTCATCAGCAGTGCCATTTCTCATCTTGGTGGCCCTCATGGAGAGTAACCTCATCATT
ACATATGCGGTAGCTGTGGCAGCTGGCATCAGTGTGGCAGCTGCCTTCTTACTACCCTGGTCCATGCTGC
CTGATGTCATTGACGACTTCCATCTGAAGCAGCCCCACTTCCATGGAACCGAGCCCATCTTCTTCTCCTT
CTATGTCTTCTTCACCAAGTTTGCCTCTGGAGTGTCACTGGGCATTTCTACCCTCAGTCTGGACTTTGCA
GGGTACCAGACCCGTGGCTGCTCGCAGCCGGAACCTGTCAAGTTTACACTGAACATGCTCGTGACCATGG
CTCCCATAGTTCTCATCCTGCTGGGCCTGCTGCTCTTCAAAATGTACCCCATTGATGAGGAGAGGCGGCG
GCAGAATAAGAAGGCCCTGCAGGCACTGAGGGACGAGGCCAGCAGCTCTGGCTGCTCAGAAACAGACTCC
ACAGAGCTGGCTAGCATCCTC
>Macaca fascicularis NM_001284595.1
ATGGCCAAAGGAGAAGGCGCCGAGAGCGGCTCCGCGGCAGGGCTGCTGCCCACCAGCATCCTCCAAG
CCACTGAACGCCCGGCCCAGGTGAAGAAAGAACCGAAAAAGAAGAAACAACAGTTGTCTGTTTGCAGCAA
GCTTTGCTATGCACTTGGGGGAGCCCCCTACCAGGTGACAGGCTGTGCCCTGGGTTTCTTCCTTCAGATC
TACCTATTGGATGTGGCTCAGGTGGGCCCTTTTTCTGCCTCCATCATCCTGTTTGTGGGCCGAGCCTGGG
ATGCCATCACAGACCCCCTGGTGGGCTTCTGCATCAGCAAATCCTCCTGGACCTGCCTGGGTCGCCTTAT
GCCTTGGATCATCTTCTCCACGCCCCTGGCCGTCATCTCCTACTTCCTCATCTGGTTCGTGCCCGACTTC
CCACACGGCCAGACCTATTGGTACCTGCTTTTCTATTGCCTCTTTGAGACGATGGTCACGTGTTTCCATG
TTCCCTACTCGGCTCTCACCATGTTCATCAGCACCGAGCAGACTGAGCGGGATTCTGCCACCGCCTATCG
GATGACTGTGGAAGTGCTGGGCACAGTGCTGGGCACGGCGATCCAGGGGCAAATCGTGGGCCAAGCAGAC
ACGCCTTGTTTCCAGGACCTCAATGGCTCTACAGTGGCTTCACAAAGTGCCAACCATACACATGGCACCA
CCTCACACAGAGAAACGCAAAAGGCATACCTGCTGGCAGCGGGGGTTATTGTCTGTATCTATGTAATCTG
TGCTGTCATCCTGACCCTGGGCGTGCGGGAGCAGAGAGAACCCTATGAAGCCCAGCAGGCTGAGCCGATC
GCCTACTTCCAGGGCCTACGGCTGGTCATGAGCCATGGCCCATACGTCAAGCTTATTACCGGCTTCCTCT
TCACCTCCTTGGCTTTCATGCTGGTGGAGGGGAACTTTGTCTTGTTTTGCACCTACACCTTGGGTTTCCG
CAATGAATTCCAGAATCTACTCCTGGCCATCATGCTCTCAGCCACTTTCACCATTCCCATCTGGCAGTGG
TTCCTGACCCGGTTTGGCAAGAAGACAGCTGTATATATTGGGATCTCATCAGCAGTGCCATTTCTCGTCT
TGGTGGCCCTCATGGAGAGTAACCTCATCATCACATATGTGGTAGCTGTGGCAGCTGGCATCAGTGTGGC
AGCTGCCTTCTTACTACCCTGGTCCATGCTGCCTGACGTCATTGACGACTTCCATCTGAAGCAGCCTCAC
TTCCACGGAACCGAGCCCATCTTCTTCTCCTTCTATGTCTTCTTCACCAAGTTTGCCTCTGGAGTGTCAC
TGGGCATTTCCACCCTCAGTCTGGACTTTGCAGGGTACCAGACCCGTGGCTGCTCGCAGCCAGAACGTGT
CAAGTTTACACTGAACATGCTCGTGACCATGGCTCCCATAGTTCCCATCCTGCTGGGCCTGCTGCTCTTC
AAACTGTACCCCATCGATGAGGAGAGGCGGCGGCAGAATAAGAAGGCCCTGCAGGCACTGAGGGACGAGG
CCAGCAGCTCTGGCTGCTCAGAAACAGACTCCACAGAGCTGGCTAGCATCCTC
>mouse lemur ENSMICT00000016325.2
ATGGCCAAAGGAGAGGGCTCCGAGAGCGGCTCCGCGGCGGGACTGCTGCCCACCGGCATC
CTCCAAGCGGGTGAACGCCCGGCCCAGGTGAAGAAGGAACCAAAGAAGAAGCAACAGTTG
TCCATTTGTAACAAGCTTTGCTATGCAGTTGGAGGGGCCCCCTACCAGGTGACAGGTTGT
GCCCTGGGTTTCTTCCTTCAGATCTATCTACTGGACGTCGCTCAGGTGGACCCTTTCCCT
GCCTCCATCATCCTATTTGTGGGCCGAGCCTGGGATGCCTTCACAGACCCCCTGGTGGGC
TTCTGCATTAGCAAATCCTCCTGGACCCGCCTGGGCCGTCTCATGCCCTGGGTCATCTTC

TCCACACCCCTGGCCATCATCGCCTACTTCCTCATCTGGTTCGTGCCCGACTTCCCGCAG
GTCCAGCCCCTCTGGTACCTGCTTTTCTATTGCCTCTTTGAGACACTGGTCACGTGTTTC
CACGTGCCCTACTCGGCCCTCACCATGTTCATCAGCACAGAGCAGAGTGAGCGGGATTCT
GCCACAGCCTACCGGATGACCGTGGAAGTGCTGGGCACGGTACTGGGCACAGCGATCCAG
GGGCAAATCGTGGGCCAAGCGGATACCCCTTGTCTCCAGGACCCCAACGGCTCCACGGTG
GCCGCGGACAGTGCCAATCGCACGCAGAGCACCACCTCACTCAGAGACACGCAAAACGCA
TACCTGCTGGCAGCAGGCGTCATTGCCTCCATCTATGTCATCTGTGCTGTCATCCTGACC
CTGGGAGTGCGGGAGCAGAGAGAACCCTATGAGGCGCAGCAGGCCGAGCCGATATCCTTC
TTCCGGGGCCTCCGCCTGGTCATGAGCCATGGCCCTTACATCAAGCTCATCGCTGGCTTC
CTCTTCACCTCCCTGGCTTTCATGCTGGTGGAGGGCAACTTTGCCTTGTTTTGCACCTAC
ACTTTGGGCTTCCGCAATGAATTCCAGAATCTACTCCTGGCCATCATGCTCTCGGCCACA
TTCACCATTCCCATCTGGCAGTGGTTCCTAACCCGGTTTGGCAAGAAGACGGCTGTATAC
ATTGGGATCTCATCAGCAGTGCCATTTCTCATCTTGGTGGCCCTCATGGAGAGTAACCTA
ATCGTCACGTACGTGGTAGCCGTGGCAGCTGGCATCAGTGTGGCAGCTGCCTTCTTACTA
CCCTGGTCCATGCTGCCCGATGTCATTGACGACTTCCACCTAAAGCAGCCCCACTCCCAT
GGAACCGAGCCCATCTTCTTCTCCTTCTATGTCTTCTTCACCAAGTTCGCCTCCGGAGTC
TCCCTGGGCATCTCCACCCTCAGTCTCGACTTTGCCGGGTACCAGACCCGTGGCTGCTCC
CAGCCGGAACCTGTCAGGTTCACGCTGAAGATGCTGGTGACCATGGCTCCCATAGTCCTC
ATCCTGCTGGGCCTGCTGCTCTTCAAGCTGTACCCTATCGACGAGGAGAAGCGGCGGCAG
AACAAGAAGGCCCTGCAGGCTCTGAGGGATGAAGCCAGCAGCTCGGGCTGCTCTGACACA
GACTCCACAGAGCTGGCCAGCATCCTC
>bushbaby XM_003798986.1
ATGGCCAAAGGAGAAGGCGCCGAGAGCGGC
TCCGCGGCAGGGCTGCTGCCCACGGGCATCCTCCAAGCGGGTGAACGTCCAGCCCAGGCGAAGAAGGAAC
CCAAGAAGAAACAACAGTTGACCATTTGCAACAAGCTTTGCTATGCAGTTGGAGGGGCCCCCTACCAGGT
GACAGGTTGTGCCCTGGGTTTCTTCCTTCAGATCTACCTATTGGATGTGGCTCAGGTGGACCCTTTCCCT
GCCTCCATCATCCTATTTGTGGGCCGGGCCTGGGATGCCTTCACAGACCCCCTGGTGGGCTTCTGCATTA
GCAAATCCTCCTGGACCCGCCTGGGCCGACTCATGCCCTGGATCATCTTCTCCACGCCCCTGGCCATCAT
CGCCTACTTCCTCATTTGGTTCGTGCCTGACTTCCCGCAGGTCCAGCCCCTTTGGTACCTGCTTTTCTAT
TGCCTCTTTGAGACGCTGGTCACGTGTTTCCACGTTCCCTACTCGGCCCTCACCATGTTCATCAGCACAG
AGCAGAGTGAGCGGGATTCTGCCACAGCCTACCGGATGACCGTGGAGGTGCTGGGCACAGTGCTGGGCAC
AGCGATCCAGGGGCAAATCGTGGGCCAAGCAGATACGCCTTGTCTCCAGGGCCCCAACAGCTCTGCAGTG
GCCTTGGGAAGTGCCAATCACACACACAGCACCACCTCACTCAAAGACACGCAAAATGCGTACTTGCTGG
CAGCAGGAGTCATTGCCTCTATCTACGTCATCTGTGCTGTCATCCTGGTCCTGGGCGTGCGGGAGCAAAG
AGAACCCTATGAGACGCAGCAGGCTGAGCAGATGTCCTTCTTCAGGGGCCTCCGCCTTGTCATGAGCCAC
GGCCCATACATCAAGCTTATTGCTGGCTTCCTCTTCACCTCCTTGGCCTTCATGCTGGTGGAAGGGAACT
TCGCCTTGTTTTGCACCTACACCTTGGGCTTCCGCAATGAATTCCAGAATCTACTCCTGGCCATCATGCT
CTCGGCCACGTTCACTATTCCCATCTGGCAGTGGTTCCTAACCCGGTTTGGCAAGAAGACGGCTGTATAC
ATTGGGATCTCATCAGCAGTGCCATTTCTCATCTTGGTGGCCCTCATGGAGCGTAACCTAATCATCACAT
ACGTGGTAGCTGTGGCAGCTGGCATCAGCGTGGCAGCTGCTTTCTTATTACCCTGGTCCATGCTGCCTGA
CGTCATTGATGACTTCCACCTGAAGCAGCCCCACTCCCATGGAACTGAGCCCATCTTCTTTTCCTTCTAT
GTCTTCTTCACCAAGTTCGCCTCTGGAATCTCACTGGGCATCTCCACCCTCAGTCTCGACTTTGCTGGGT
ACCGGACCCGCAGCTGCTCCCAGCCAGCACGCGTCAGGTTTACACTGAAGATGCTGGTGACCATGGCGCC
CATAGTCCTCATCCTGCTGGGCCTGCTGCTCTTCAAGCTGTACCCTATTGATGAGGAGAAGCGACGGCAG
AACAAGAAGGCCCTGCAGGCTCTGAGGGATGAAGCCAGCAGCTCGGGCTGCTCTGACACAGACTCCACAG
AGCTGGCCAGCATCCTC
>chimpanzee NM_001280162.1

ATGGCCAAAGGAGAAGGCGCCGAGAGCGGCTCCGCGGCGGGGCTGCTGCCCACCAGCATCCTCCAAA
GCACTGAACGCCCGGCCCAGGTGAAGAAAGAACCGAAAAAGAAGAAACAACAGTTGTCTGTTTGCAACAA
GCTTTGCTATGCACTTGGGGGAGCCCCCTACCAGGTGACGGGCTGTGCCCTGGGTTTCTTCCTTCAGATC
TACCTACTGGATGTGGCTCAGGTGGGCCCTTTCTCTGCCTCCATCATCCTGTTTGTGGCCGAGCCTGGG
ATGCCATCACAGACCCCCTGGTGGGCCTCTGCATCAGCAAATCCCCCTGGACCTGCCTGGGTCGCCTTAT
GCCCTGGATCATCTTCTCCACGCCCCTGGCCGTCATTGCCTACTTCCTCATCTGGTTCGTGCCCGACTTC
CCACACGGCCAGACCTATTGGTACCTGCTTTTCTATTGCCTCTTTGAGACAATGGTCACGTGTTTCCATG
TTCCCTACTCGGCTCTCACCATGTTCATCAGCACCGAGCAGACTGAACGGGATTCTGCCACCGCCTATCG
GATGACTGTGGAAGTGTTGGGCACAGTGCTGGGCACGGCGATCCAGGGGCAAATCGTGGGCCAAGCAGAC
ACGCCTTGTTTCCAGGACCTCAATAGCTCTACAGTAGCTTCACAAAGTGCCAACCATACACATGGCACCA
CCTCACACAGGGAAACGCAAAAGGCATACCTGCTGGCAGCGGGGGTCATTGTCTGTATCTATATAATCTG
TGCTGTCATCCTGATCCTGGGCGTGCGGGAGCAGAGAGAACCCTATGAAGCCCAGCAGGCTGAACCGATC
GCATACTTCCGGGGCCTACGGCTGGTCATGAGACACGGCCCATACATCAAACTTATTACCGGCTTCCTCT
TCACCTCCTTGGCTTTCATGCTGGTGGAGGGGAACTTTGTCTTGTTTTGCACCTACACCTTGGGCTTCCG
CAATGAATTCCAGAATCTACTCCTGGCCATCATGCTCTCGGCCACTTTAACCATTCCCATCTGGCAGTGG
TTCTTGACCCGGTTTGGCAAGAAGACAGCTGTATATGTTGGGATCTCATCAGCAGTGCCATTTCTCATCT
TGGTGGCCCTCATGGAGAGTAACCTCATCATTACATATGCGGTAGCTGTGGCAGCTGGCATCAGTGTGGC
AGCTGCCTTCTTACTACCCTGGTCCATGCTGCCTGATGTCATTGACGACTTCCATCAGAAGCAGCCCCAC
TTCCATGGAACCGAGCCCATCTTCTTCTCCTTTTATGTCTTCTTCACCAAGTTTGCCTCTGGAGTGTCAC
TGGGCATTTCTACCCTCAGTCTGGACTTTGCAGGGTACCAGACCCGTGGCTGCTCGCAGCCGGAACGTGT
CAAGTTTACACTGAACATGCTCGTGACCATGGCTCCCATAGTTCTCATCCTGCTGGGCCTGCTGCTCTTC
AAAATGTACCCCATTGATGAGGAGAGGCGGCGGCAGAATAAGAAGGCCCTGCAGGCACTGAGGGACGAGG
CCAGCAGCTCTGGCTGCTCAGAAACAGACTCCACAGAGCTAGCTAGCATCCTC
>Papio anubis XM_003891646.2
ATGGCCAAAGGAGAAGGCGCCGAGAG
CGGCTCCGCGGCAGGGCTGCTGCCCACCAGCATCCTCCAAGCCACTGAACGCCCGGCCCAGGTGAAGAAA
GAACCGAAAAAGAAGAAACAACAGTTGTCTGTTTGCAGCAAGCTTTGCTATGCACTTGGGGGAGCCCCCT
ACCAGGTGACAGGCTGTGCCCTGGGTTTCTTCCTTCAGATCTACCTATTGGATGTGGCTCAGGTGGGCCC
TTTTTCTGCCTCCATCATCCTGTTTGTGGCCGAGCCTGGGATGCCATCACAGACCCCCTGGTGGGCTTC
TGCATCAGCAAATCCTCCTGGACCTGCCTGGGTCGCCTTATGCCTTGGATCATCTTCTCCACGCCCCTGG
CCGTCATCTCCTACTTCCTCATCTGGTTCGTGCCCGACTTCCCACACGGCCAGACCTATTGGTACCTGCT
TTTCTATTGCCTCTTTGAGACGATGGTCACGTGTTTCCATGTTCCCTACTCGGCTCTCACCATGTTCATC
AGCACCGAGCAGACTGAGCGGGATTCTGCCACCGCCTATCGGATGACTGTGGAAGTGCTGGGCACAGTGC
TGGGCACGGCGATCCAGGGGCAAATCGTGGGCCAAGCAGACACGCCTTGTTTCCAGGACCTCAATGGCTC
TACAGTGGCTTCACAAAGTGCCAACCATACACATGGCACCACCTCACACAGAGAAACGCAAAAGGCATAC
CTGCTGGCAGCGGGGGTTATTGTCTGTATCTATGTAATCTGTGCTGTCATCCTGACCCTGGGCGTGCGGG
AGCAGAGAGAACCCTATGAAGCCCAGCAGGCTGAGCCGATCGCCTACTTCCAGGGCCTACGGCTGGTCAT
GAGCCATGGCCCATACGTCAAGCTTATTACCGGCTTCCTCTTCACCTCCTTGGCTTTCATGCTGGTGGAG
GGGAACTTTGTCTTGTTTTGCACCTACACCTTGGGCTTCCGCAATGAATTCCAGAATCTACTCCTGGCCA
TCATGCTCTCAGCCACTTTCACCATTCCCATCTGGCAGTGGTTCCTGACCCGGTTTGGCAAGAAGACAGC
TGTATATATTGGGATCTCATCAGCAGTGCCATTTCTCGTCTTGGTGGCCCTCATGGAGAGTAACCTCATC
ATCACATATGTGGTAGCTGTGGCAGCTGGCATCAGTGTGGCAGCTGCCTTCTTACTACCTTGGTCCATGC
TGCCTGACGTCATTGACGACTTCCATCTGAAGCAGCCTCACTTCCACGGAACCGAGCCCATCTTCTTCTC
CTTCTATGTCTTCTTCACCAAGTTTGCCTCTGGAGTGTCACTGGGCATTTCCACCCTCAGTCTGGACTTT
GCAGGGTACCAGACCCGTGGCTGCTCGCAGCCAGAACGTGTCAAGTTTACACTGAACATGCTCGTGACCA
TGGCTCCCATAGTTCTCATCCTGCTGGGCCTGCTGCTCTTCAAACTGTACCCCATAGATGAGGAGAGGCG
GCGGCAGAATAAGAAGGCCCTGCAGGCACTGAGGGACGAGGCCAGCAGCTCTGGCTGCTCAGAAACAGAC

TCCACAGAGCTGGCTAGCATCCTC
>orangutan ENSPPYT00000001786.2
ATGGCCAAAGGAGAAGGCGCCGAGAGCGGCTCCGCGGCGGGGCTGCTGCCCACCAGCATC
CTCCAAACCACTGAACGCCCGGCCCAGGTGAAGAAAGAACCGAAAAAGAAGAAACAACAG
TTGTCTGTTTGCAACAAGCTTTGCTATGCACTTGGGGGAGCCCCCTACCAGGTGACGGGC
TGTGCCCTGGGTTTCTTCCTTCAGATCTACCTATTGGATGTGGCTCAGGTGGGCCCTTTC
TCTGCCTCCATCATCCTGTTTGTGGGCCGAGCCTGGGATGCCATCACAGACCCCCTGGTG
GGCCTCTGCATCAGCAAATCCCCCTGGACCTGCCTGGGTCGCCTTATGCCCTGGATCATC
TTCTCCACACCCCTGGCCGTCATTGCCTACTTCCTTATCTGGTTCGTGCCTGACTTCCCA
GATGGCCAGACCTATTGGTACCTGCTTTTCTATTGCCTCTTTGAGACGATGGTCACGTGT
TTCCATGTTCCCTACTCGGCTCTCACCATGTTCATCAGCACCGAGCAGACTGAGCGGGAT
TCTGCCACCGCCTATCGGATGACTGTGGAAGTGCTGGGCACAGTGCTGGGTACGGCGATC
CAGGGGCAAATCGTGGGCCAAGCAGACACGCCTTGTTTCCAGGACCTCAATAGCTCTACA
GTAGCTTCACAAAGTGCCAACCATACACATGGCACCACCTCACACAGGGAAACGCAAAAG
GCATACCTGCTGGCAGCGGGGGTCATTGTCTGTATCTATATAATCTGTGCTGTCATCCTG
ATCCTGGGCGTGCGGGAGCAGAGAGAACCCTACGAAGCCCAGCAGGCTGAGCCGATCGCC
TACTTCCGGGGCCTACGGCTGGTCATGAGCCACGGCCCATACATCAAGCTTATTACCGGC
TTCCTCTTCACCTCCTTGGCTTTCATGCTGGTGGAGGGGAACTTTGTCTTGTTTTGCACC
TACACCTTGGGCTTCCGCAATGAATTCCAGAATCTACTCCTGGCCATCATGCTCTCGGCC
ACTTTCACCATTCCCATCTGGCAGTGGTTCTTGACCCGGTTTGGCAAGAAGACAGCTGTA
TATGTTGGGATCTCATCAGCAGTGCCATTTCTCATCTTGGTGGCCCTCATGGAGAGTAAC
CTCATCATTACATATGTGGTAGCTGTGGCAGCTGGCATCAGTGTGGCAGCTGCCTTCTTA
CTACCCTGGTCCATGTTGCCTGATGTCATTGACGACTTCCATCTGAAGCAGCCCCACTTC
CATGGAACCGAGCCCATCTTCTTCTCCTTCTATGTCTTCTTCACCAAGTTTGCCTCTGGA
GTGTCACTGGGCATTTCCACCCTCAGTCTGGACTTTGCAGGGTACCAGACCCGCGGCTGC
TCGCAGCCGGAACGTGTCAAGTTTACACTGAACATGCTCGTGACCATGGCTCCCATAGTT
CTCATCCTGCTGGGCCTACTGCTCTTCAAAATGTACCCCATTGATGAGGAGAGGCGGCGG
CAGAATAAGAAGGCCCTGCAGGCACTGAGGGATGAGGCCAGCAGCTCTGGCTGCTCAGAA
ACAGACTCCACAGAGCTGGCTAGCATCCTC
>Rhinopithecus roxellana XM_010354204.1
ATGGCCAAAGGAGAAGGCGCCGAGAGCGGCT
CCGCGGCAGGGCTGCTGCCCACCAGCATCCTCCAAGCCACTGAACGCCCGGCCCAGGTGAAGAAAGAACC
GAAAAAGAAGAAACAACAGTTGTCTGTTTGCAGCAAGCTTTGCTATGCACTTGGGGGAGCCCCCTACCAG
GTGACAGGCTGTGCCCTGGGTTTCTTCCTTCAGATCTACCTATTGGATGTGGCTCAGGTGGGCCCTTTTT
CTGCCTCCATCATCCTGTTTGTGGGCCGAGCCTGGGATGCCATCACAGACCCCCTGGTGGGCTTCTGCAT
CAGCAAATCCTCCTGGACCTGCCTGGGTCGCCTTATGCCCTGGATCATCTTCTCCACGCCCCTGGCCGTC
ATCTCCTACTTCCTCATCTGGTTCGTGCCCGACTTCCCACACGGCCAGACCTATTGGTACCTGCTTTTCT
ATTGCCTCTTTGAGACGATGGTCACGTGTTTCCATGTTCCCTACTCGGCTCTCACCATGTTCATTAGCAC
CGAGCAGACTGAGCGGGATTCTGCCACCGCCTATCGGATGACTGTGGAAGTGCTGGGCACAGTGCTGGGC
ACGGCGATCCAGGGGCAGATCGTGGGCCAAGCAGACACGCCTTGTTTCCAGGACCTCAATAACTCTACAG
TGGCTTCACAAAGTGCCAACCATACACATGGCACCACCTCACACAGAGAAACGCAAAAGGCATACCTGCT
GGCAGCGGGGGTTATTGTCTGTATCTATGTAATCTGTGCTGTCATCCTGACCCTGGGCGTGCGGGAGCAG
AGAGAACCCTATGAAGCCCAGCAGGCTGAGCCGATCGCCTACTTCCGGGGCCTACGGCTGGTCATGAGCC
ACGGCCCATACATCAAGCTTATTACCGGCTTCCTCTTCACCTCCTTGGCTTTCATGCTGGTGGAGGGGAA
CTTTGTCTTGTTTTGCACCTACACCTTGGGCTTCCGCAATGAATTCCAGAATCTACTCCTGGCCATCATG
CTCTCAGCCACTTTCACCATTCCCATCTGGCAGTGGTTCCTGACCCGGTTTGGCAAGAAGACAGCTGTAT
ATATTGGGATCTCATCAGCAGTGCCATTTCTCGTCTTGGTGGCCCTCATGGAGAGTAACCTCATCATCAC

ATATGTGGTAGCTGTGGCAGCTGGCATCAGTGTGGCAGCTGCCTTCTTACTACCCTGGTCCATGCTGCCT
GACGTCATTGACGACTTCCATCTGAAGCAACCTCACTTCCACGGAACCGAGCCCATCTTCTTCTCCTTCT
ATGTCTTCTTCACCAAGTTTGCCTCTGGAGTGTCACTGGGCATTTCCACCCTCAGTCTGGACTTTGCAGG
GTACCAGACCCGTGGCTGCTCGCAGCCAGAACGTGTCAAGTTTACACTGAACATGCTCGTGACCATGGCT
CCCATAGTTCTCATCCTGCTGGGCCTGTTGCTCTTCAAACTGTACCCCATCGATGAGGAGAGGCGGCGGC
AGAACAAGAAGGCCCTGCAGGCACTGAGGGACGAGGCCAGCAGCTCTGGCTGCTCAGAAACAGACTCCAC
AGAGCTGGCTAGCATCCTC

>Mandrillus leucophaeus XM_011977662.1
ATGGCCAAAGGAGAAGGCGCCGAGAGCGGCT
CCGCGGCAGGGCTGCTGCCCACCAGCATCCTCCAAGCCACTGAACGCCCGGCCCAGGTGAAGAAAGAACC
GAAAAAGAAGAAACAACAGTTGTCTGTTTGCAGCAAGCTTTGCTATGCACTTGGGGGAGCCCCCTACCAG
GTGACAGGCTGTGCCCTGGGTTTCTTCCTTCAGATCTACCTATTGGATGTGGCTCAGGTGGGCCCTTTTT
CTGCCTCCATCATCCTGTTTGTGGGCCGAGCCTGGGATGCCATCACAGACCCCCTGGTGGGCTTCTGCAT
CAGCAAATCCTCCTGGACCTGCCTGGGTCGCCTTATGCCTTGGATCATCTTCTCCACGCCCCTGGCCGTC
ATCTCCTACTTCCTCATCTGGTTCGTGCCCGACTTCCCACACGGCCAGACCTATTGGTACCTGCTTTTCT
ATTGCCTCTTTGAGACGATGGTCACGTGTTTCCATGTTCCCTACTCGGCTCTCACCATGTTCATCAGCAC
CGAGCAGACTGAGCGGGATTCTGCCACCGCCTATCGGATGACTGTGGAAGTGCTGGGCACAGTGCTGGGC
ACGGCGATCCAGGGGCAAATCGTGGGCCAAGCAGACACGCCTTGTTTCCAGGACCTCAATGGCTCTACAG
TGGCTTCACAAAGTGCCAACCATACACATGGCACCACCTCACACAGAGAAACGCAAAAGGCATACCTGCT
GGCAGCGGGGGTTATTGTCTGTATCTATGTAATCTGTGCTGTCATCCTGACCCTGGGCGTGCGGGAGCAG
AGAGAACCCTATGAACCCCAGCAGGCTGAGCCGATCGCCTACTTCCAGGGCCTACGGCTGGTCATGAGCC
ATGGCCCATACGTCAAGCTTATTACCGGCTTCCTCTTCACCTCCTTGGCTTTCATGCTGGTGGAGGGGAA
CTTTGTCTTGTTTTGCACCTACACCTTGGGCTTCCGCAATGAATTCCAGAATCTACTCCTGGCCATCATG
CTCTCAGCCACTTTCACCATTCCCATCTGGCAGTGGTTCCTGACCCGGTTTGGCAAGAAGACAGCTGTAT
ATATTGGGATCTCATCAGCAGTGCCATTTCTCGTCTTGGTGGCCCTCATGGAGAGTAACCTCATCATCAC
ATATGTGGTAGCTGTGGCAGCTGGCATCAGTGTGGCAGCTGCCTTCTTACTACCTTGGTCCATGCTGCCT
GACGTCATTGATGACTTCCATCTGAAGCAGCCTCACTTCCACGGAACCGAGCCCATCTTCTTCTCCTTCT
ATGTCTTCTTCACCAAGTTTGCCTCTGGAGTGTCACTGGGCATTTCCACCCTCAGTCTGGACTTTGCAGG
GTACCAGACCCGTGGCTGCTCGCAGCCGGAACGTGTCAAGTTTACACTGAACATGCTCGTGACCATGGCT
CCCATAGTTCTCATCCTGCTGGGCCTGCTGCTCTTCAAACTGTACCCCATAGATGAGGAGAGGCGGCGGC
AGAATAAGAAGGCCCTGCAGGCACTGAGGGACGAGGCCAGCAGCTCTGGCTGCTCAGAAACAGACTCCAC
AGAGCTGGCTAGCATCCTC
>Saimiri boliviensis boliviensis XM_010347555.1
ATGGCCAAAGGAGAAGGCGCCGAGAGCGGCTCCGCGGCGGGGCTGCT
GCCCAACAGCATCCTCCAAGCCAGTGAACGCCCTGTCCAGGTGAAGGAACCGAAAAAGAAGAAACAACAG
TTGTCCGTGTGCAACAAGCTTTGCTATGCAGTTGGGGGAGCCCCCTACCAGGTGACGGGCTGTGCCCTGG
GTTTCTTCCTTCAGATCTACCTATTGGATGTGGCTCAGGTGAGCCCTTTCTCTGCCTCTATCATTCTGTT
TGTGGGCCGAGCCTGGGATGCCATCACAGACCCCCTGGTGGGCTTCTGCATCAGCAAATCCTCCTGGACC
TGCCTGGGTCGCCTTATGCCCTGGATCATCTTCTCCACACCCCTGGCCGTCATAGCCTACTTCCTCATCT
GGTTCGTGCCTGACTTCCCACAGGGCCAGACCTATTGGTACCTGCTTTTCTATTGCCTCTTTGAGACGCT
GGTCACGTGTTTCCACGTTCCCTACTCGGCTCTTACCATGTTCATCAGCACCGAGCAGACTGAGCGGGAT
TCGGCCACCGCCTATCGGATGACTGTGGAAGTGCTGGGCACAGTGCTGGGCACAGCGATTCAGGGGCAAA
TCGTGGGCCAAGCAGACATGCCTTGTTTCCAGGACCACAATAGCTCTGCAGAGGCTTCACAAAGTACCAA
CCGTACACATTACATCACCTCACACAGAGAAACGCAAAATGCATACCTGCTGGCAGCGGGGGTCATTGCC
TCTATCTATGTCGTCTGTGCTGTCATCCTGACCCTGGGCGTGCGGGAGCGGAGAGAACCCTATGAGGCCC
AGCAGGCTGAGCCGATCGCCTACTTCCAGGGCCTACGGCTGGTCATGAGCCACGGCCCATACATCAAGCT

GATTACTGGCTTCCTTTTCACCTCCTTGGCTTTCATGCTGGTGGAGGGGAACTTTGTCTTGTTTTGCACC
TATACCTTGGGCTTCCGCAACGAATTCCAGAATCTACTCCTGGCCATCATGCTCTCCGCCACCTTCACCA
TTCCCATCTGGCAGTGGTTCCTGACCCGGTTTGGCAAGAAGACAGCTGTCTATGTTGGGATCTCATCAGC
AGTGCCATTTCTCGTCTTGGTGGCCCTTATGGAGAGTAATCTCATCGTCACATATGTGGTAGCTGTGGCG
GCTGGCATCAGTGTGGCAGCTGCCTTCTTATTACCCTGGTCCATGCTGCCTGACGTCATTGATGACTTCC
ATCTGAAGCAGCCCCATTTCCATGGAACCGAGCCCATCTTCTTCTCCTTCTATGTCTTCTTCACCAAGTT
TGCCTCTGGAGTGTCACTGGGCATTTCCACCCTCAGTCTCCACTTTGCAGGGTACCAGACCCTTAGCTGC
TCCCAGCCGGAACGTGTTAAGTTTACACTGAACATGCTTGTGACCATGGCGCCCATAGTTCTCATCCTGC
TGGGCCTGCTGCTCTTCAAACTGTACCCCATTGATGAGGAGAGGCGGCAGCAGAATAAAAAAGCCCTGCA
GGCGCTGAGGGATGAAGCCAGCAGCTCTGGCTGCTCAGAAACAGACTCCACAGAGCTGGCTAGCATCCTC

>Cebus capucinus imitator XM_017507968.1
ATGGCCAAAGGAGAAGGCGCCGAGAGCGGCTCTGCGGCTGGGCTGCTGCCCACCAGCATCC
TCCAAGCCAGTGAACGTCCTGCCCAGGTGAAGGAACCGAAAAAGAAGAAACAACAGTTGTCCGTGTGCAA
CAAGCTTTGCTATGCAGTTGGGGGAGCCCCCTACCAGGTGACAGGCTGTGCCCTGGGTTTCTTCCTTCAG
ATCTACCTATTGGATGTGGCTCAGGTTGGCCCTTTCTCTGCCTCTATCATTCTGTTTGTGGGCCGAGCCT
GGGATGCCATCACAGACCCCCTGGTGGGCTTCTGCATCAGCAAATCCTCCTGGACCTGCCTGGGTCGCCT
TATGCCCTGGATCATCTTCTCCACACCCCTGGCAGTCATAGCCTACTTCCTCATCTGGTTCGTGCCTGAC
ATCCCACAGGGCCAGACCTATTGGTACCTGCTTTTCTATTGCCTCTTTGAGACGCTGGTCACGTGTTTCC
ACGTTCCCTACTCGGCTCTTACCATGTTCATCAGCACCGAGCAGACTGAGCGGGATTCGGCCACCGCCTA
TCGGATGACTGTGGAAGTGCTGGGCACAGTGCTGGGCACGGCGATCCAGGGGCAAATTGTGGGCCAAGCA
GACATGCCTTGTTTCCAGGACCACAATAGCTCTGCAGAGGCTTCACAAAGTACCAACGGTACACATTACA
TCACCTCACACAGAGAAACGCAAAATGCATACCTGCTGGCAGCGGGGGTCATTGCCTTTATCTATGTCAT
CTGTGCTGTCATCTTGACCCTGGGCGTGCGGGAGCGGAGAGAACCCTATGAAGCCAAGCAGGCTGAGCCG
ATCGCCTACTTCCAGGGCCTCCGGCTGGTCATGAGCCACGGCCCATACATCAAGCTGATTACTGGCTTCC
TCTTCACCTCCTTGGCTTTCATGCTGGTGGAGGGGAACTTTGTCTTGTTTTGCACCTATACCTTGGGCTT
CCGCAACGAATTCCAGAATCTACTCCTGGCCATCATGCTCTCCGCCACCTTCACCATTCCCGTCTGGCAG
TGGTTCCTGACCCGGTTTGGCAAGAAGACAGCTGTCTATGTTGGGATCTCATCAGCAGTGCCATTTCTCA
TCTTGGTGGCCCTTATGGAGAGTAACCTCATCGTCACATATGTGGTAGCTGTGGCAGCTGGCATCAGTGT
GGCAGCTGCCTTCTTATTACCCTGGTCCATGCTGCCTGACGTCATTGACGACTTCCATCTGAAGCAGCCC
CACTTCCATGGAACCGAGCCCATCTTCTTCTCCTTCTATGTCTTCTTCACCAAGTTTGCCTCTGGAGTGT
CACTGGGCATTTCCACCCTCAGTCTCCACTTTGCAGGGTACAAGACCCTTAGCTGCTCCCAGCCAGAACA
TGTTAAGTTTACACTGAACATGCTTGTGACCATGGCGCCCATAGTTCTCATCCTGCTGGGCCTGCTGCTC
TTCAAACTGTACCCCATTGATGAGGAGAGGCGGCGGCAGAATAAAAAAGCCCTGCAGGCACTGAGGGATG
AAGCCAGCAGCTCTGGCTGCTCAGAAACAGACTCCACAGAGCTGGCTAGCATCCTC
>Aotus nancymaae XM_012439135.1
ATGGCCAAAGGAGAAGGCGCCGAGAGCGGC
TCCGCGGCGGGGCTGCTGCCCACCAGCATCCTCCAAGCCAGTGAACGTCCTGCCCAGGTGAAGAAGGAAC
CGAAAAAGAAGAAACAACAGTTGTCCGTGTGCAACAAGCTTTGCTATGCCGTTGGGGGAGCCCCCTACCA
GGTGACGGGCTGTGCCCTGGGTTTCTTCCTTCAGATCTACCTATTGGATGTGGCTCAGGTGGGCCCTTTC
TCTGCCTCTATCATCCTGTTTGTGGGCCGAGCCTGGGATGCCATCACAGACCCCCTGGTGGGCTTCTGCA
TCAGCAAATCCTCCTGGACCTGCCTGGGTCGCCTTATGCCCTGGATCATCTTCTCCACACCCCTGGCCGT
CATAGCCTACTTCCTCATCTGGTTCGTGCCTGACTTCCCACAGGGCCAGACCTATTGGTACCTGCTTTTC
TATTGCCTCTTTGAGACACTGGTCACGTGTTTCCACGTTCCCTACTCGGCTCTTACCATGTTCATCAGCA
CCGAGCAGACTGAGCGGGATTCAGCCACCGCCTATCGGATGACTGTGGAAGTGCTGGGCACAGTGCTGGG
CACGGCGATCCAGGGGCAAATCGTGGGCCAAGCAGACATGCCTTGTTTCCAGGACCACAATAGCTCTGCA
GAGGCTTCACAAAGTACCAACAGTACACATTACATCACCTCACACAGAGAAACGCAAAATGCATACCTGC

TGGCAGCGGGGGTCATTGCCTCTATCTATGTCATCTGTGCTGTCATCCTGACCCTGGGTGTGCGGGAGCA
GAGAGAACCCTATGAGGCCCAGCAGGCTGAGCCGATCGCCTACTTCCAGGGCCTCCGGCTGGTCATGAGC
CACGGCCCATACATCAAGCTGATTACCGGCTTCCTCTTCACCTCCTTGGCTTTCATGCTGGTGGAGGGAA
ACTTTGTCTTGTTTTGCACTTACACCTTGGGCTTCCGCAACGAATTCCAGAATCTACTCCTGGCCATCAT
GCTCTCCGCCACCTTCACCATTCCCATCTGGCAGTGGTTCCTGACCCGGTTTGGCAAGAAGACGGCTGTC
TATGTTGGGATCTCATCAGCAGTGCCATTTCTCATCTTGGTGGCCCTTATGGAGAGTAACCTCATCGTCA
CATATGTGGTAGCTGTGGCGGCTGGCATCAGTGTGGCAGCTGCCTTCTTATTACCCTGGTCCATGCTGCC
TGACGTCATTGATGACTTCCATCTGAAGCAGCCCCACTTCCGTGGAACCGAGCCCATCTTCTTCTCCTTC
TATGTCTTCTTCACCAAGTTTGCCTCTGGAGTGTCACTGGGCATTTCCACCCTCAGTCTTGACTTTGCAG
GGTACCAGACCCTTAGCTGCTCCCAGCCGGAACGTGTTAAGTTTACACTGAACATGCTTGTGACCATGGC
GCCCATAGTTCTCATCCTGCTGGGCCTGCTGCTCTTCAAACTGTACCCCATTGATGAGGAGAGGCGGCGG
CAGAATAAGAAAGCCCTGCAGGCACTGAGGGATGAAGCCAGCAGCTCTGGCTGCTCAGAAACAGACTCCA
CAGAGCTGGCTAGCATCCTT
>Cercocebus atys XM_012079323.1
ATGGCCAAAGGAGAAGGCGCCGAGAGCGGCTCCGCG
GCAGGGCTGCTGCCCACCAGCATCCTCCAAGCCACTGAACGCCCGGCCCAGGTGAAGAAAGAACCGAAAA
AGAAGAAACAACAGTTGTCTGTTTGCAGCAAACTTTGCTATGCACTTGGGGGAGCCCCCTACCAGGTGAC
AGGCTGTGCCCTGGGTTTCTTCCTTCAGATCTACCTATTGGATGTGGCTCAGGTGGGCCCTTTTTCTGCC
TCCATCATCCTGTTTGTGGGCCGAGCCTGGGATGCCATCACAGACCCCCTGGTGGGCTTCTGCATCAGCA
AATCCTCCTGGACCTGCCTGGGTCGCCTTATGCCTTGGATCATCTTCTCCACGCCCCTGGCCGTCATCTC
CTACTTCCTCATCTGGTTCGTGCCCGACTTCCCACACGGCCAGACCTATTGGTACCTGCTTTTCTATTGC
CTCTTTGAGACGATGGTCACGTGTTTCCATGTTCCCTACTCGGCTCTCACCATGTTCATCAGCACCGAGC
AGACTGAGCGGGATTCTGCCACCGCCTATCGGATGACTGTGGAAGTGCTGGGCACAGTGCTGGGCACGGC
GATCCAGGGGCAAATCGTGGGCCAAGCAGACACGCCTTGTTTCCAGGACCTCAATGGCTCTACAGTGGCT
TCACAAAGTGCCAACCATACACATGGCACCACCTCACACAGAGAAACGCAAAAGGCATACCTGCTGGCAG
CGGGGGTTATTGTCTGTATCTATGTAATCTGTGCTGTCATCCTGACCCTGGGCGTGCGGGAGCAGAGAGA
ACCCTATGAAGCCCAGCAGGCTGAGCCGATCGCCTACTTCCAGGGCCTACGACTGGTCATGAGCCATGGC
CCATACGTCAAGCTTATTACCGGCTTCCTCTTCACCTCCTTGGCTTTCATGCTGGTGGAGGGGAACTTTG
TCTTGTTTTGCACCTACACCTTGGGCTTCCGCAATGAATTCCAGAATCTACTCCTGGCCATCATGCTCTC
AGCCACTTTCACCATTCCCATCTGGCAGTGGTTCCTGACCCGGTTTGGCAAGAAGACAGCTGTATATATT
GGGATCTCATCAGCAGTGCCATTTCTCGTCTTGGTGGCCCTCATGGAGAGTAACCTCATCATCACATATG
TGGTAGCTGTGGCAGCTGGCATCAGTGTGGCAGCTGCCTTCTTACTACCTTGGTCCATGCTGCCTGACGT
CATTGATGACTTCCATCTGAAGCAGCCTCACTTCCACGGAACCGAGCCCATCTTCTTCTCCTTCTATGTC
TTCTTCACCAAGTTTGCCTCTGGAGTGTCACTGGGCATTTCCACCCTCAGTCTGGACTTTGCAGGGTACC
AGACCCGTGGCTGCTCGCAGCCGGAACGTGTCAAGTTTACACTGAACATGCTCGTGACCATGGCTCCCAT
AGTTCTCATCCTGCTGGGCCTGCTGCTCTTCAAACTGTACCCCATAGATGAGGAGAGGCGGCGGCAGAAT
AAGAAGGCCCTGCAGGCACTGAGGGACGAGGCCAGCAGCTCTGGCTGCTCAGAAACAGACTCCACAGAGC
TGGCTAGCATCCTC
>Propithecus coquereli XM_012646720.1
ATGGCCAAAGGAGAGGGCGCCGAGAGCGGCTCCGCGGC
GGGACTCCTGCCCCCCGGCATCCTCCAAGCGGGCGAACGCCCGGCCCAGGTGAAGAAGGAACCAAAGAAG
AAACAACAGCTGTCCATTTGCAACAAGCTGTGCTATGCAGTTGGAGGGGCCCCCTACCAGGTGACAGGCT
GTGTCCTGGGTTTTTTCCTTCAGATCTACCTACTGGACGTGGCTCAGGTGGACCCTTTCCCTGCCTCCAT
CATCCTATTTGTGGGCCGAGCCTGGGATGCCTTCACAGACCCCCTGGTGGGCTTCTGCATTAGCAAATCC
TCATGGACCCGCCTGGGCCGCCTCATGCCCTGGATCATCTTCTCCACGCCCCTGGCCATCATTGCCTACT
TCCTCATTTGGTTCGTGCCCGACTTCCCGCAGGTCCAGCCCCTCTGGTACCTGCTTTTCTATTGCCTCTT
TGAGACACTGGTCACGTGTTTCCACGTGCCCTACTCAGCCCTCACCATGTTCATCAGCACAGAGCAGAGC

GAGCGGGATTCTGCCACAGCCTACCGGATGACCGTGGAGGTGCTGGGCACAGTGCTGGGCACAGCGATCC
AGGGGCAAATCGTGGGCCAAGCGGATACACCTTGTCTCCAGGACCCCAACGGCTCCGCAGTGGCCTCGGA
CAGTGCCAATCGCACACAGAGCATCACCTCACTCAGAGACACGCAAAACGCTTACCTGCTGGCAGCAGGG
GTCATTGCCTCCATCTATGTCATCTGTGCTGTCATCCTGACCCTGGGAGTGCGGGAGCAGAGAGAACCCT
ATGAGACACAGCAGGCTGAGCCAGTATCCTTCTTCCGGGGCCTCCGCCTGGTCATGAGCCATGGCCCGTA
CATCAAGCTCATCACTGGCTTCCTCTTCACCTCCCTGGCTTTCATGCTGGTGGAGGGCAACTTTGCCTTG
TTTTGCACCTACACCTTGGGCTTCCGCAACGAATTCCAGAATCTACTCCTGGCCATCATGCTCTCAGCCA
CATTCACCATTCCCATCTGGCAGTGGTTCCTAACCCGGTTTGGCAAGAAGACGGCTGTATACATTGGGAT
CTCATCAGCAGTGCCATTTCTCATCTTGGTGGCCCTCATGGAGAGTAACCTAATCGTCACGTACGTGGTA
GCTGTGGCAGCTGGCATCAGTGTGGCAGCTGCCTTCTTACTACCCTGGTCCATGCTGCCCGACGTCATTG
ACGACTTCCACCTGAAGCAGCCCCACTCCCATGGAACCGAGCCCATCTTCTTCTCCTTCTATGTCTTCTT
CACCAAGTTCGCCTCCGGAGTCTCACTGGGCATCTCCACCCTCAGTCTCGACTTTGCCGGCTACCAGACC
CGTGGCTGCTCCCAGCCGGCACGTGTCAGGTTTACACTGAAGATGCTGGTGACCATGGCTCCCATAGTCC
TCATCCTGCTGGGCTTGCTGCTCTTCAAGCTGTACCCTATCGACGAGGAGAAGCGGCGGCAGAACAAGAA
GGCCCTGCAGGCTCTGAGGGATGAAGCCAGCAGCTCGGGCTGCTCTGACACAGACTCCACAGAGCTGGCC
AGCATCCTC
>Galeopterus variegatus XM_008580248.1
ATGGCCAAAGGAGAGGGCGCCGAGAGCAGCTCCGCGGCG
GGGCTACTGCCCACGGGCATCCTCCAAGCCAGTGAACGCCCTGCCCAGGCGAAGAAGGAACCAAAGAAGA
AACAACAGTTGTCAATTTGCAACAAGCTTTGCTATGCAGTTGGGGGAGCCCCATACCAGGTGACGGGCTG
TGCCCTGGGGTTCTTCCTGCAGATCTACCTATTGGATGTGGCTCAGGTGGACACTTTCTCTGCTTCCATC
ATCCTGTTTGTAGGCCGAGCCTGGGATGCCTTCACGGACCCTCTGGTGGGCTTCTGCATTAGCAAATCCT
CCTGGACCCGCCTGGGCCGCCTCATGCCCTGGATCATCTTCTCAACTCCCCTGGCCATCATCGCCTACTT
CCTCATCTGGTTTGTGCCCGACTTCCCACGGGGCCAGGCCCTTTGGTACCTGCTTTTCTATTGCCTGTTT
GAGACACTGGTCACGTGTTTCCACGTTCCCTACTCAGCCCTCACCATGTTCATCAGCACAGAGCAGAGCG
AGCGGGATTCTGCCACTGCCTATCGAATGACTGTGGAGGTGCTGGGCACAGTGCTGGGTACAGCGATCCA
GGGGCAAATTGTGGGCCAAGCGGATACGCCTTGCCTCCAGGACCCTAATGGCTCTGCAGTGGCTCTGGAA
AGTGCCAATCACACATACAGTACCACCTCACTCAGAAAAACGCAAAATGCATACCTGCTGGCAGCTGGTG
TCATCGCCTCCATCTATGTCATTTGTGCTGTCATCCTGACTGTGGGTGTGCGGGAGCAGAGAGAACCCTA
TGAGACACAGCAGGCTGAGCCGATGCCCTTCTTCCGGAGCCTCCGGCAGGTCATGAGCCATGGCCCATAC
ATCAAGCTCATTGCCGGCTTCCTCTTCACTTCCCTGGCTTTCATGCTGGTGGAGGGGAACTTCGCCTTGT
TTTGCACCTACACCTTGGGCTTCCGCAACGAATTCCAGAATCTACTCCTGGCCATCATGCTCTCAGCCAC
ATTCACCATCCCCATCTGGCAGTGGTTCCTAACCCGGTTTGGCAAGAAGACAGCTGTGTACATTGGGATC
TCATCAGCAGTGCCATTTCTCATCTTGGTGGCCCTCATGGAGAGCAACCTAATCGTCACGTATGTGGTAG
CTGTGGCAGCTGGCATCAGTGTGGCAGCTGCCTTCTTACTACCTTGGTCTATGCTGCCCGATGTCATTGA
TGACTTCCACCTGAAGCACCCCCACTCCCATGGAACTGAGCCCATCTTCTTCTCCTTCTATGTCTTCTTC
ACCAAGTTCGCCTCTGGAGTCTCCCTGGGTATCTCCACCCTCAGTCTCGACTTTGCCGGGTACCAGACTG
GGGGCTGCTCCCAGCCAGCACGTGTCAAGTTCACGCTGAAGATGCTAGTGACCATGACTCCCATCGTCCT
CATCCTGCTGGGCCTGCTGCTCTTCAAGCTGTACCCCATTGATGAAGAGAAGCGGCGGCAGAATAAGAAG
GCCCTGCAGGCTTTGAGGGATGAAGCCAGCAGCTCGGGGTGCTCTGACACAGACTCCACAGAGCTGGCCA
GCATCCTC
>Cavia porcellus XM_003462274.3
ATGGCCAAAGGAGAGGGTTCCGAGAGCGGCTCCGCGGCGGGGCTGCT
GCCCCCCAGCATCCTCCAAGCCAGCGAACGCCCGGCCCAGGTGAAGAAGGAACCGAAGAAGAAGCAACAG
TTGTCAGTTTGCAACAAGCTCTGCTATGCAGTTGGAGGGGCCCCCTACCAGGTGACTGGATGTGCCCTGG
GCTTCTTCCTGCAGATCTACCTGTTGGACGTAGCTCTGGTGGATCCTTTCTCTGCCTCCATCATCCTGTT
CGTGGGCCGAGCCTGGGATGCCTTCACGGACCCTCTGGTGGGCTTCTTCATTAGCAAATCCTCCTGGACC

CGCCTGGGTCGCCTTATGCCCTGGATCATCTTCTCCACACCCCTGGCCGTGGTCGCCTACTTCCTCATCT
GGTTCGTGCCTGACTTCCCTCGGGGCCAGGCCCTTTGGTACCTGATTTTCTATTGCCTCTTTGAGACACT
GGTCACGTGCTTCCATGTCCCCTACTCGGCCCTCACCATGTTCATCAGTACTGAGCAGAGTGAGCGGGAC
TCGGCCACTGCCTATCGGATGACCGTGGAGGTGCTGGGCACAGTACTGGGCACAGCAATCCAGGGACAAA
TAGTAGGCCAAGCAAATGCACCTTGTCTCCAGGATCCTGGTGACTCTGCACTAATCTCAGAAAGTGGGAA
TCGCACACACAGCATCTCTTCACTCAGACAAACGCAAAATGCATACCTGCTGGCAGCGGGAGTCATTGCC
TCCATTTACATCGTCTGTGCTGTCGTCCTGACCCTGGGTGTGCGGGAGCAGAGAGAGCCCTGTGAGACCC
AGCAGGCGGAGCCAATTCCTTTTTTCCGGGGCCTTCGGCTGGTCATGAGTCACGGCCCGTACGTCAAGCT
CATTGCTGCCTTCCTCTTTACCTCGCTGGCTTTCATGCTAGTGGAAGGGAACTTTGCCTTGTTTTGCACC
TATACCCTGGGCTTCCGCAATGAGTTCCAGAACCTACTCCTGGCCATCATGCTCTCAGCCACGTTCACCA
TCCCCCTCTGGCAGTGGTTCCTGACCCGCTTTGGCAAGAAGACAGCTGTGTACACTGGGATCTCATCAGC
AGTGCCATTTCTCATCTTGGTGGCCCTCATAGAGCGTAACCTAATCGTCACGTACGTGGTGGCCATAGCA
GCTGGCGTCAGTGTAGCAGCAGCCTTCTTACTGCCCTGGTCCATGCTGCCCGACGTCATTGATGACTTCC
ACCTGAAGCACCCTAACTCCCATGGCACGGAGCCCATCTTCTTCTCTTTCTATGTCTTCTTCACCAAGTT
CGCCTCTGGAGTTTCACTGGGCATCTCCACTCTCAGTCTCGACTTTGCCGGGTACCAGTCGCGGGGCTGC
TCCCAGCCATCACGTGTCAAGTTCACTCTGAAGATGCTGGTGGCCCTGACGCCCATAGCCCTCATTGTGC
TGGGTCTGATGCTCTTCAAGCTGTACCCCATCGATGAGGAAAAGAGGCGGCAGAATAAGAAGGCCCTGCA
GGCTCTGCGGGATGAAGCCAGCAGCTCAGGCTGCTCCGATGCAGACTCCACAGAGCTGGCCAGCATTCTC

>Ictidomys tridecemlineatus ENSSTOT00000005049.2
ATGGCCAAAGGAGAGGGCGCCGAGAGCGGCTCCGCGGCGGGGCTGCTGCCCACCGGCATT
CTCCAAGCGGGTGAACGCCCGGCCCAGATGAAGAAGGAGCCAAAGAAGAAGCAGCAGTTG
TCCATTTGCAACAAACTTTGCTACGCTGTTGGGGGGGCCCCCTACCAGGTGACGGGCTGT
GCTCTAGGGTTCTTCCTGCAGATCTACCTATTGGACGTGGCTCAGGTGAACCCTTTCCCT
GCTTCCATCATCCTGTTCGTGGGTCGAGCCTGGGATGCCTTCACTGATCCTTTGGTGGGC
TTCTGCATTAGCAAATCTTCCTGGACCCGCCTAGGCCGCCTCATGCCCTGGATCATCTTC
TCCACACCCCTGGCCATCATCACCTACTTCCTCATCTGGTTTGTGCCTGACTTCCCACAG
GGCCAAGAGCTTTGGTACCTGCTTTTCTATTGCCTCTTTGAGACTCTAGTCACGTGTTTC
CACGTTCCCTACTCAGCCCTCACCATGTTCATCAGCACTGAGCAGAGCGAGAGGGACTCT
GCCACTGCCTATCGGATGACTGTGGAGGTGCTGGGCACAGTGCTGGGCACAGCGATCCAG
GGGCAAATTGTGGGCCAAGCAAACGCACCTTGTCTCCAGTACCTCAATGGCTCTGCAGTG
GCTTCAGAAAGTGCCAATCGCACATACAGCACCTCCTCACTCAGAGAAACGCAAAATGCA
TACCTGCTGGCAGCAGGGGTCATTGCCTCCATTTACGTCATCTGTGCTGTCATCCTGACC
CTGGGCGTACGGGAGCAGAGAGAACCCTATGAGACCCAGCAGGCTGAGCCGATGTCCTTT
TTCCGGGGCCTCCGCCAGGTCATGAGCCATGGGCCATATATCAAGCTCATTGCTGGCTTC
CTCTTTACCTCTCTGGCTTTCATGCTGGTGGAGGGGAACTTTGCCTTGTTTTGCACCTAC
ACCTTGGGCTTCCGCAATGAATTCCAGAACCTACTGCTGGCCATCATGCTTTCTGCCACA
TTTACCATTCCCATCTGGCAGTGGTTCCTAACCCGGTTTGGCAAGAAGACAGCTGTATAT
GTTGGGATCTCATCAGCAGTACCATTTCTCATCTTGGTGGCCCTTATGGAGAGTAACCTA
ATCATCACGTATGTGGTGGCTATCGCAGCCGGCATCAGTGTGGCAGCTGCCTTCCTATTA
CCCTGGTCCATGTTGCCTGATGTCATTGACGACTTCCACCTGAAGCAGCCTCACTCCCAT
GGCACTGAACCCATCTTCTTCTCCTTCTATGTTTTTTTCACCAAGTTTGCCTCTGGAGTC
TCACTGGGTATCTCCACCCTCAGTCTCGACTTTGCTGGGTACAAGACACGAGGGTGCTCA
CAGCCGGCAAGTGTCAAGTTTACACTGAAGATGTTGGTGACCATGGCTCCTATAGTCCTC
ATCCTGCTGGGCCTGTTGCTCTTCAAGCTGTATCCCATTGATGAGGAGAAGCGGCGGCAG
AATAAGAAGGCCCTACAGGCTCTGCGGGATGAAGCCAGCAGCTCAGGCTATTCTGACACA
GACTCCACAGAGCTGGCCAGCATCCTC

>mouse ENSMUST00000030408.11
ATGGCCAAAGGAGAAGGCGCCGAGAGCGGTTCCGCGGCGGGGCTGCTCCCCACCAGCATC
CTCCAAGCCAGTGAACGGCCGGTCCAGGTGAAGAAGGAACCAAAAAAGAAGCAGCAACTG
TCCATTTGCAACAAGCTTTGCTATGCAGTTGGAGGGGCCCCGTACCAGTTGACCGGCTGC
GCACTGGGATTCTTCCTGCAGATCTACCTATTGGATGTGGCTAAGGTGGAACCACTTCCT
GCTTCCATTATCCTTTTTGTGGGCCGAGCCTGGGATGCCTTCACTGACCCTCTGGTGGGC
TTCTGCATTAGCAAGTCCTCCTGGACCCGCCTGGGCCGCCTCATGCCCTGGATCATCTTC
TCCACTCCCCTGGCCATCATTGCTTACTTCCTCATCTGGTTTGTGCCTGACTTCCCATCA
GGGACCTTCCTTTGGTACCTGCTTTTCTATTGCCTCTTTGAGACA
CTGGTCACGTGCTTTCATGTTCCCTACTCAGCGCTCACCATGTTCATCAGCACGGAGCAG
AGTGAGCGTGACTCAGCCACGGCATACAGAATGACTGTGGAGGTGCTGGGCACAGTGATA
GGCACAGCGATTCAAGGACAAATTGTGGGCCAAGCCAAGGCACCTTGTCTCCAGGACCAG
AATGGCTCTGTGGTGGTCTCAGAAGTTGCCAATCGCACCCAGAGTACTGCCTCCCTCAAA
GACACGCAAAATGCTTACCTGCTGGCAGCAGGGATCATCGCCTCCATCTACGTCCTCTGT
GCCTTCATTCTGATCCTAGGCGTGCGGGAGCAGAGAGAACTCTACGAGTCCCAGCAGGCT
GAGTCAATGCCCTTCTTTCAGGGCCTCCGGCTGGTCATGGGTCATGGCCCCTATGTCAAG
CTCATTGCCGGCTTCCTTTTTACCTCCCTGGCTTTCATGCTGGTGGAGGGTAACTTTGCC
TTGTTCTGCACCTATACCTTGGACTTCCGAAATGAGTTCCAGAACCTCCTCCTGGCCATC
ATGCTCTCGGCCACATTCACCATCCCTATCTGGCAGTGGTTCCTAACCCGGTTTGGCAAG
AAGACAGCTGTATACATCGGGATCTCTTCTGCAGTTCCTTTTCTCATCTTGGTGGCCCTC
ATGGAGCGTAATCTAATCGTCACTTACGTGGTGGCCGTAGCAGCTGGCGTCAGTGTAGCA
GCTGCCTTCCTACTACCATGGTCCATGCTGCCTGACGTTATCGATGACTTCCACCTGAAA
CACCCTCACTCCCCTGGCACCGAGCCCATATTCTTCTCCTTCTATGTCTTCTTCACCAAG
TTTGCCTCTGGAGTCTCACTGGGTGTCTCTACCCTCAGTCTCGACTTTGCCAACTACCAG
AGGCAGGGATGCTCCCAGCCAGAACAGGTCAAGTTTACACTGAAGATGCTGGTGACCATG
GCTCCTATCATCCTCATCTTGCTGGGCCTGCTGCTCTTCAAGCTCTACCCCATTGATGAG
GAGAAGCGGCGACAGAATAAGAAAGCTCTGCAGGCTCTACGAGAAGAAGCCAGCAGCTCA
GGTTGCTCGGATACAGACTCCACAGAGCTGGCCAGTATTCTC
>rabbit ENSOCUT00000027629.2
ATGGCCAAAGGAGAAGGTGCCGAGAGCGGCTCCGCGGCGGGGCCTCTCCCGAGCTCTCAC
CCAGTAGGCAGGTGGGTCCCAGCCTCAGCTAGGAAGGAACCAAAGAAGAAACAGCAGTTG
TCCGTTTGCAACAAGCTCTGCTATGCAGTTGGAGGCGCCCCTTACCAGGTGACAGGCTGT
GCCCTGGGCTTCTTCCTACAGATATACCTGCTGGATGTGGCTCAGGTGGACCCTTTCTCT
GCCTCCATCATCTTGTTCGTGGGCCGAGCCTGGGATGCCTTCACGGACCCTCTGGTGGGC
TTCTGCATTAGCAAATCCTCCTGGACCCGCCTGGGCCGCCTCATGCCCTGGATCATCTTC
TCCACGCCCTTGGCCATCATTGCCTACTTCCTCATCTGGTTCGTGCCCGACTTCCCACAG
GGCCAGACCCTTTGGTACCTGCTTTTCTATTGCCTCTTTGAGACACTGGTCACGTGTTTC
CACGTCCCCTACTCGGCTCTCACCATGTTCATCAGCGTAGAGCAGAGCGAGCGGGATTCA
GCCACCGCCTACCGGATGACAGTGGAGGTGCTGGGCACAGTGCTGGGCACAGCAATCCAG
GGGCAAATTGTGGGCAAAGTGAATTCGCCTTGTCTCCAGGAGCCCAATGGCTCTGCAGTG
GCCTTGGAAAGTGCCAACTGGACGCACAGCACCACCTCGCTCAGAGAAACGCAAAATGCG
TACCTGCTGGCAGCAGGGGTCATCGCCTCCATCTATGTCATCTGTGCTGTCATCCTGATC
CTGGGCGTGCGGGAGCAGCGAGAACCCTGTGAGGCCCAGCAGACTGAGCCCATGTCCTTC
TTCCGGGGCCTCCGGCTGGTCATGAGCCATGGCCCGTATGTCAAGCTTATCGCTGGCTTC
CTCTTCACCTCCCTGGCTTTCATGCTGGTGGAGGGGAACTTTGCTCTGTTTTGCACATAC
TCCTTAGGCTTCCGCAATGAATTCCAGAACCTACTCCTGGCCATCATGCTCTCGGCCACC
TTCACCATTCCCATCTGGCAGTGGTTCCTGACCCGGTTTGGCAAGAAGACGGCCGTATAT

GTGGGGATCTCATCAGCAGTGCCATTTCTCATCTTGGTGGCCGTCAGGGAGAGTAACCTA
ATCGTCACATATGTGGTGGCCGTGGCAGCTGGCGTCAGTGTGGCAGCTGCCTTCTTACTA
CCTTGGTCCATGCTGCCTGACGTTATTGATGACTTCCACCTGAAGCAGCCCCGCTCCCAG
GGCACCGAGCCCATCTTCTTCTCCTTCTACGTCTTCTTCACTAAGTTCGCCTCCGGCGTG
TCGCTGGGTATCTCCACCCTAAGCCTCGACTTTGCTGGGTACCAGACACGCGGCTGCTCC
CAGCCGAGGCGCGTCAAGTTTACGCTGAACATGCTGGTGACCATGACGCCCATAGCCCTC
ATCCTGGTGGGCCTGCTGCTCTTCAAGCTCTACCCCATCGACGAGGACAGGCGGCGGCAG
AACAAGAAGGCGCTGCAGGCTCTGAGGGACGAAGCCAGCAGCTCCGGCTGCTCTGACACA
GACTCCACAGAGCTGGCCAGCATCCTC
>rat ENSRNOT00000019080.7
ATGGCCAAAGGAGAGGGCGCCGAGAGCGGTTCCGCGGCGGGGCTGCTCCCCACCGGCATC
CTCCAAGCCAGCGAACGACCAGCCCAGGTGAAGAAGGAACCAAAAAAGAAGCAGCAACTG
TCCATTTGCAATAAGCTTTGCTATGCGGTTGGAGGGGCCCCATACCAGTTGACCGGCTGC
GCGCTGGGATTCTTCCTGCAGATCTACCTGTTGGATGTGGCTAAGGTGGAGCCACTCCCT
GCTTCCATTATCCTTTTCGTGGGCCGAGCGTGGGATGCCTTCACTGACCCTCTCGTGGGC
TTCTGCATCAGCAAGTCCTCCTGGACCCGCCTGGGCCGCCTCATGCCCTGGATCATCTTC
TCCACTCCTCTGGCCATCATTGCTTACTTTCTCATCTGGTTTGTGCCTGACTTCCCATCA
GGGACGTTCCTTTGGTACCTGCTTTTCTATTGCCTCTTTGAGACA
CTGGTCACGTGCTTCCACGTTCCCTACTCAGCGCTCACCATGTTCATCAGTACCGAGCAG
AGTGAGCGTGACTCCGCCACTGCCTATAGGATGACTGTGGAGGTTCTGGGCACAGTGATA
GGCACAGCGATCCAAGGACAAATTGTGGGCCAAGCCAAGGCACCTTGTCTCCAGGACCAG
AATGGCTCTGCAGTGGCCTCAGAAGTTGTCAATCGTACCCAGAGCACTGCCTCCCTCAAA
GAAACGCAAAATGCATACCTGCTGGCAGCAGGGATCATCGCCTCCATCTACGTCATCTGT
GCCGTCATTCTGATTTTAGGCGTGCGGGAGCAGAGAGAACCCTACGAGGCCCAGCAGGCC
GAGTCAATGCCCTTCTTTCAGGGCCTCCGGCTGGTTATGAGTCATGGCCCCTATGTCAAG
CTCATTGCCGGCTTCCTTTTTACCTCCCTGGCTTTCATGCTGGTAGAAGGTAATTTTGCC
TTGTTCTGCACCTACACCTTGGGCTTCCGGAACGAGTTCCAGAACCTCCTCCTGGCCATC
ATGCTCTCGGCCACATTCACCATCCCCATCTGGCAATGGTTCCTAACCCGGTTTGGCAAG
AAGACGGCTGTATACGTTGGGATCTCTTCTGCAGTTCCTTTTCTCATCTTGGTGGCCCTC
ATGAAGAGTAATCTAATCGTCACTTACGTGGTGGCCATAGCAGCTGGTGTCAGTGTAGCA
GCTGCCTTCTTACTACCATGGTCCATGCTGCCTGATGTTATCGATGACTTCCACCTGAAA
CACCCTCACTCCCCTGGCACGGAGCCCATCTTCTTCTCCTTCTATGTCTTCTTCACCAAG
TTTGCCTCTGGAGTCTCACTGGGTGTCTCTACCCTCAGTCTCGACTTTGCTAAGTACCAG
ACGCAGGGGTGCTCCCAGCCAGAAGAGGTCAAGTTTACACTGAAGATGCTGGTGACCATG
GCTCCTATCATCCTCATCCTGCTGGGCCTGCTGCTCTTCAAGCTGTACCCCATTGATGAG
GAGAAGCGGCGACAGAACAAGAAAGCTCTGCAGGCTTTACGAGATGAAGCCAGCAGCTCA
GGCTGCTCGGATACAGACTCCACAGAGCTGGCCAGCATTCTC
>Marmota marmota marmota XM_015485214.1
ATGGCCAAAGGAGAGGGCGCCGAGAGCGGCTCCGCGGCGGG
GCTGCTGCCCACCAGCATTCTCCAAGCGGGTGAACGCCCGGCCCAGGTGAAGAAGGAGCCAAAGAAGAAG
AAGCAGTTGTCCATTTGCAACAAACTTTGCTATGCTGTTGGGGGGGCCCCCTACCAGGTGACGGGCTGTG
CTCTAGGGTTCTTCCTGCAGATCTACCTATTGGACGTGGCTCAGGTGAACCCTTTCCCTGCTTCCATCAT
CCTGTTTGTGGGTCGAGCCTGGGATGCCTTCACTGATCCTCTGGTGGGCTTCTGCATTAGCAAATCTTCC
TGGACCCGCCTAGGCCGCCTCATGCCCTGGATCATCTTCTCCACACCCCTGGCCATCATCACCTACTTCC
TCATCTGGTTTGTGCCTGACTTCCCACAGGGCCAAGAGCTTTGGTACCTGCTTTTCTATTGCCTCTTTGA
GACTCTAGTCACGTGTTTCCACGTTCCCTACTCAGCCCTCACCATGTTCATCAGCACTGAGCAGAGCGAG
AGGGACTCTGCCACTGCCTATCGGATGACTGTGGAGGTGCTGGGCACAGTGCTGGGCACAGCGATCCAGG

GGCAAATTGTGGGCCAAGCAAATGCACCTTGTCTCCAGTACCTCAATGGCTCTGCAGTGGCTTCAGAAAG
TGCCAATCGCACATACAGCACCACCTCACTCAGAGAAACGCAAAATGCATACCTGCTGGCAGCAGGGGTC
ATTGCCTCTATTTATGTCATCTGTGCTGTCATCCTGACCCTGGGCGTACGGGAGCAGAGAGAACCCTATG
AGACCCAGCAGGCTGAGCCGATGTCCTTTTTCCGGGGCCTCCGCCAGGTCATGAGCCATGGGCCATATAT
CAAGCTCATTGCTGGCTTCCTCTTTACCTCTCTGGCTTTCATGCTGGTGGAGGGGAACTTTGCCTTGTTT
TGCACCTACACCTTGGGCTTCCGCAATGAATTCCAGAACCTACTGCTGGCCATCATGCTTTCTGCCACAT
TTACCATTCCCATCTGGCAGTGGTTCCTAACCCGGTTTGGCAAGAAGACAGCTGTATACGTTGGGATCTC
ATCAGCAGTGCCATTTCTCATCTTGGTGGCCCTTATGGAGAGTAACCTAATCGTCACGTATGTGGTGGCT
ATCGCAGCCGGCATCAGTGTGGCAGCTGCCTTCCTATTACCCTGGTCCATGTTGCCTGATGTCATTGACG
ACTTCCACCTGAAGCAGCCTCACTCCCATGGCACTGAGCCCATCTTCTTCTCCTTCTATGTTTTTTTCAC
CAAGTTTGCCTCTGGAGTCTCACTGGGTATCTCCACCCTCAGTCTCGACTTTGCTGGGTACAAGACACGA
GGGTGCTCACAGCCGGCAAGTGTCAAGTTTACACTGAAGATGTTGGTGACCATGGCTCCTATAGTCCTCA
TCCTGCTGGGCCTGTTGCTCTTCAAGCTGTATCCCATTGATGAGGAGAAGCGGCGGCAGAATAAGAAGGC
CCTGCAGGCTCTGCGGGATGAAGCCAGCAGCTCAGGCTATTCTGACACAGACTCCACAGAGCTGGCCAGC
ATCCTC
>Chinchilla lanigera XM_005395270.1
ATGGCCAAAGGAGAGGGCTCCGAGAGCGGCTCCGCGGCGGGGC
TGCTGTCCCCCAGCATCCTCCAAGCCGGCGAACGCCAGGCCCAGGTGAAGAAGGAACCAAAGAAGAAACA
GCAGTTGTCAGTTTGCAACAAGCTCTGCTATGCAGTTGGAGGGGCCCCCTACCAGGTGACAGGCTGTGCC
CTGGGCTTCTTCCTGCAGATCTACCTGTTGGACGTAGCTCTGGTGGACCCTTTCTCTGCCTCCATCATCC
TGTTTGTGGGCCGAGCCTGGGATGCCTTCACGGACCCTCTGGTGGGCTTCTGCATTAGCAAATCCTCCTG
GACCCGCCTGGGTCGCCTCATGCCCTGGATCATCTTCTCCACACCACTGGCTGTCGTCGCCTACTTCCTC
ATCTGGTTCGTGCCCGACTTCCCACGGGGGCAGGCCCTTTGGTACCTGATTTTCTATTGCCTCTTTGAGA
CATTGGTCACGTGCTTCCATGTTCCCTACTCGGCACTTACCATGTTCATCAGCACTGAGCAGAGTGAGCG
GGACTCAGCCACGGCCTATCGGATGACCGTGGAGGTTCTGGGCACAGTGCTGGGCACAGCGATCCAGGGA
CAGATCGTAGGCCAAGCGAATGCGCCTTGTCTCCAGGACCCCAGTGACTCTGCACTGGCCTCAGAAAGCA
GCAATCGCACGCACAGCATCTCCTCACTCAGACAAACGCAAAATGCATACCTGCTCGCAGCGGGGGTCAT
TGGCTCCATCTACGTGGTCTGTGCTGTCATCCTGACCCTGGGCGTGCGGGAGCGGAGAGAGCCCTGTGAG
ACCCAGCAGGCTGAGCCAGTGCCCTTCTTCCGGGGCCTTCGGCTGGTCATGAGCCATGGCCCATACGTCA
AGCTCATTGCTGCCTTCCTCTTTACCTCCCTGGCGTTCATGCTGGTGGAAGGGAACTTTGCTCTGTTTTG
CACCTACACCCTGGGCTTCCGCAATGAGTTCCAGAATCTGCTCCTGGCCATCATGCTCTCAGCCACATTC
ACCATTCCCCTCTGGCAGTGGTTTCTGACCCGCTTTGGCAAGAAGACAGCTGTGTACATTGGGATCTCAT
CAGCAGTGCCGTTTCTCATCTTGGTGGCCCTCATCGAGAGTAATTTAATCATCACATATGTGGTGGCTGT
AGCAGCTGGCATCAGTGTAGCAGCAGCCTTCCTACTACCCTGGTCCATGCTGCCTGATGTCATCGATGAC
TTCCACCTGAAGCACCCCCACTCCCACGGCACAGAGCCCATCTTCTTCTCCTTCTATGTCTTCTTCACCA
AGTTCGCCTCTGGAGTCTCGATGGGCATCTCCACCCTCAGTCTCGACTTTGCTGGGTACCGGTCGCGGGG
CTGCTCCCAGCCGGCACGCGTCAAGTTCACGCTGAAGATGCTGGTGGCCCTGACGCCCATAACCCTCATC
CTGCTGGGCCTGCTGCTCTTCAAGCTATACCCTATTGACGAGGAGAAGCGGCGGCAGAACAAGAAGGCCC
TGCAGGCTCTGCGGGATGAAGCCAGCAGCTCAGGCTGCTCTGACACAGACTCCACAGAGCTGGCCAGCAT
CCTC
>Fukomys damarensis XM_010625897.1
ATGGCCAAAGGAGAGGGTTCCGAGAGCGGCTCCGCGGCGGGGTTGCTGTCCCCCAGTATCCTCCAAGCCGGC
G
AACACCCGGCCCAGGTGAAGGAACCAAAGAAGAAACAGCAGTTGTCAGTTTGCAACAAGCTTTGCTATGC
AGTTGGAGGGGCCCCCTACCAGGTGACAGGCTGTGCCCTAGGCTTCTTCCTGCAGATCTACCTGTTGGAT
GTAGCTCTGGTGGACCCTTTCTCTGCCTCCATCATCCTGTTTGTGGGCCGAGCCTGGGACGCCTTCACAG
ACCCTCTGGTGGGCTTCTGCATTAGCAAATCCTCTTGGACCCGCCTGGGTCGCCTCATGCCCTGGATCAT

CTTCTCCACGCCCCTGGCCGTGGTCGCCTACTTCCTCATCTGGTTCGTGCCCGACTTCTCGCGGGGCCAG
GCCCTTTGGTACCTGGTTTTCTATTGCCTCTTTGAGACACTGGTCACGTGCTTCCACGTTCCCTACTCGG
CTCTCACCATGTTCATCAGCACCGAGCAGAGTGAGCGGGACTCCGCCACTGCCTATCGGATGACTGTGGA
GGTGCTGGGCACGGTGCTCGGCACAGCAATCCAGGGACAAATCGTAGGCCAGGCGAATTCACCCTGTGTC
CAGGACCCCAGTGACTCCATACTGGCCTCAGAAAGCGGCAATCGCACACACAGCATCTCTTCACTCAGAC
AAACGCAAAATGCGTACCTGCTGGCAGCGGGGGTCATATCCTCCATCTACGTCATCTGTGCTGTCATCCT
GACCCTGGGTGTGCGGGAGCGGAGAGAGCCCTATGAGGCCCAGCAGGCTGAGCCGGTTCCCTTCTTCCGG
GGTCTTCGTCTGGTCATGAGCCATGGCCCGTACATCAAGCTCATTGCTGCCTTCCTCTTTACCTCCCTGG
CTTTCATGCTGGTGGAAGGGAACTTTGCCCTGTTTTGCACCTACACCTTGGGCTTCCGCAATGAGTTCCA
GAATCTACTCCTGGCCATCATGCTCTCAGCCACATTCACCATTCCCATCTGGCAGTGGTTCCTGACCCGC
TTTGGCAAGAAGACGGCTGTGTACATTGGCATCTCATCAGCGCTGCCATTTCTCATGTTGGTGGCCCTCA
TGGAGAGTAACCTAATCGTCACATACGTGGTGGCCATAGCAGCTGGTATCAGCGTGGCGGCAGCTTTCTT
ACTACCCTGGTCCATGCTGCCCGACGTCATCGATGACTTCCACCTGAAGCAGCCCCACTCCCATGGCACT
GAGCCCATCTTCTTCTCCTTCTACGTCTTCTTCACCAAGTTCGCCTCGGGAGTCTCGCTGGGTATTTCCA
CCCTCAGTCTCGACTTTGCCGGGTACCGGTCGCGGGGCTGCTCCCAGCCAACTCGTGTCAAGTTTACACT
GAAGATGCTGGTGACCATGACTCCCATCATCCTCATCCTGCTGGGCCTGCTGCTCTTCAAGCTATACCCT
ATTGACGAGGAGAAGCGGCGACAGAACAAGAAGGCCCTGCAGGCTCTTCGGGATGAAGCCAGCAGCTCAG
GCTGCTCTGACACAGACTCCACAGAGCTGGCCAGTATCCTC

>cow XM_005204713.3
ATGGCCAAAGGAGAGGGCGCCGAGAGCGGCTCCGCCGCGGGACTTCTGCC
CACTGGCATCCTCCAAGCCGGTGAACGCCCGGTCCAGGTGAAGAAGGAACCAAAGAAGAAAAAACAGTTG
TCCATTTGCAACAAACTATGCTATGCAGTTGGGGGAGCCCCGTATCAGGTGACGGGATGTGCCCTGGGGT
TCTTCCTGCAGATCTACCTGTTGGATGTAGCTCAGGTGGATCCTTTCTCTGCTTCCATCATCCTATTCGT
GGGCCGAGCTTGGGATGCCATCACAGACCCCCTGGTGGGCTTCTGCATTAGCAAATCTCCCTGGACCCGC
CTGGGCCGCCTCATGCCCTGGATCACCTTCTCCACCCCCCTGGCCATCATCGCCTACTTCCTCATCTGGT
TCGTGCCCGACTTCCACCAGGGCCAGACCCTGTGGTACTTGCTTTTCTACTGCCTCTTTGAAACGCTGGT
CACGTGTTTCCACGTTCCCTACTCAGCTCTCACCATGTTCATCAGCACGGAGCAGAGTGAGCGTGATTCT
GCCACTGCCTATCGGATGACCGTGGAGGTGTTAGGTACAGTGCTGGGCACAGCAATCCAGGGGCAGATCG
TGGGCCAAGCAGATTCGCCTTGTATCCCAGATGCCAATGCTTCTACAGTCAATCGCACGCAGAGTTCCAC
CTCGATCAAAGAAACGCAAAATGCATACCTGCTGGCAGCAGGAGTCATTGCCTCCATCTATGTCATCTGT
GCTGTCATCTTGATCCTGGGCGTGCGGGAACAGAGAGAATCCTACGAGACTCAGCAGACCAAGCAGATGC
CCTTCTTTCGGGGCCTCCGGCTGGTCATGAGCCATGGGCCGTACATCAAGCTTATTGCCGGCTTCCTCTT
CACCTCCTTGGCTTTCATGCTGGTGGAGGGGAACTTTGCCTTGTTTTGTACCTACACCTTGGGCTTTCGG
AACGAATTCCAGAATCTGCTCCTGGCAATCATGTTCTCAGCCACAGTCACCATCCCCATCTGGCAGTGGT
TCCTAACCCGGTTTGGCAAAAAGACAGCTGTGTACATTGGAATCTCGTCAGCAGTGCCATTTCTCATCTT
GGTGGCCCTCATGGAGAGTAATCTGATTGTCACATACGTGGTGGCTGTGGCAGCTGGCATCAGCGTTGCA
GCTGCCTTCTTACTACCATGGTCCATGCTGCCCGACGTCATTGATGACTTCCACTTGAAGCAGCCCCACA
TCCACGGGACCGAGCCCATCTTCTTCTCCTTCTACGTCTTCTTCACCAAGTTTGCCTCTGGAGTCTCTCT
GGGCATCTCCACCCTCAGTCTGGACTTCACCGGGTACCAGACCCGTGGCTGCTCCCAGCCGGCACGTGTC
AAGTTCACGCTGAAGATGCTGGTGACCATGGCTCCCATAGTCCTCATCCTCATAGGCCTGCTGCTCTTCA
AGCTGTACCCCATTGATGAGGAGAAGCGGCGGCAGAACAAGAAGGCCCTGCAGGCTCTGAGGGAAGAGGC
CAGCAGCTCAGGCTGCTCTGACACAGACTCTACAGAACTGGCCAGCATCCTC
>dog ENSCAFT00000004852.3
ATGGCCAAGGGAGAGGGCGCCGAGAGCGGCTCCGCGGCGGGGCTGCTGCCCACCGGCATC
CTCCCAGCCGCCGAACGGCCGGCGCAGGTCAAGAAGGAACCGAAGAAGAAACAACAGTTG
TCCATTTGTAACAAGCTTTGCTATGCAGTTGGAGGAGCCCCCTACCAGGTGACGGGCTGT

GCCCTGGGGTTCTTCCTGCAGATCTACCTGTTGGATGTGGCTCAGGTGGAACCTTTCTTT
GCCTCCATCATCCTATTTGTGGGCCGAGCGTGGGATGCCTTCACAGACCCCCTGGTGGGC
TTCTGCATTAGCAAATCTTCCTGGACCCGCCTGGGCCGCCTCATGCCCTGGATCATCTTC
TCCACACCCCTGGCCATCATTGCTTACTTCCTCATCTGGTTCGTGCCTGATTTCCCACGG
GGCCAGGCCCTGTGGTACCTGCTTTTCTACTGCCTGTTTGAAACGCTGGTCACGTGTTTC
CACGTTCCCTACTCAGCTCTCACGATGTTCATCAGCACAGAGCAGAGTGAGCGGGATTCC
GCTACTGCATATCGGATGACTGTGGAGGTGCTGGGCACAGTGTTGGGCACAGCGATCCAG
GGGCAAATCGTGGGCCAAGCAGACACGCCTTGCCTCCAGGACCCCAGTGACTCTGCACTG
GCCATGGAAGGTGCCAATCACACACAGAGCACCACATCACTCAAAGAAACGCAAAACGCA
TACCTGCTGGCAGCAGGGGTCATTGCCTCCATCTATGTCATCTGTGCTGTCATCCTAACC
CTGGGTGTGCGGGAGCAGAGAGAACCCTATGAGACTCAGCAGGCTGAGCCGATGTCCTTT
TTTCGGGGCCTGCGGCTGGTCATGAGCCATGGCCCATACGTCAAGCTTATTGCTGGCTTC
CTCTTCACCTCCCTGGCTTTTATGCTGGTGGAGGGGAACTTCGCCTTGTTTTGCACCTAC
ACCTTGGGCTTCCGAAATGAATTCCAGAATCTGCTCCTGGCCATCATGCTCTCGGCCACA
TTCACCATTCCCATCTGGCAGTGGTTTCTAACCCGATTTGGCAAGAAGACGGCTGTATAC
GTTGGGATCTCATCAGCAGTGCCATTTCTCATCTTGGTGGCCTTCATGGAGAGTAACCTG
ATTGTCACCTATGTGGTAGCTGTGGCAGCTGGCATCAGTGTAGCAGCGGCCTTCTTACTA
CCCTGGTCCATGCTGCCTGATGTCATTGACGACTTCCACTTGAAGCAGCCCCAGTCTCAC
GGCACTGAGCCCATCTTCTTCTCCTTCTATGTCTTTTTCACCAAGTTTGCTTCCGGAGTC
TCACTGGGCATCTCCACCCTGAGTCTTGACTTCGCCGGGTACCAGACCCGTGGCTGCTCA
CAGCCGGCACGTGTCAAGTTCACCCTGAAGTTGCTGGTGACCATAGCTCCCATAGTCCTC
ATCCTGCTAGGCCTGCTGCTCTTCAAGCTGTACCCCATTGACGAGGAGAAGCGGCGGCAG
AACAAGAAGGCCCTGCAGGCTCTGAGGGAAGAGGCCAGCAGCTCCGGCTGCTCTGACACA
GACTCTACAGAGCTGGCCAGCATCCTC
>Erinaceus europaeus XM_007527080.2
ATGGCCAAAGGAGAGGGCGCCGAGAGCGGGCCCGCGGCGGGACTGCTGCCCACCGGCATCCTCCAAAGTGGT
GAA
CGCCCGGCCCAGGTGAAGAAAGAGACAAAGAAGAAACAACAGTTGTCAATTTGCAACAAGCTGTGCTATG
CAGTTGGGGGAGCCCCCTACCAAGTGACAGGCTGTGCCCTAGGATTCTTCCTACAGATCTACCTGTTGGA
TGTGGCTCGGATGCACCCTTTCTCTGCCTCTATCATC
CTATTTGTGGGCCGAGCTTGGGATGCCGTCACAGACCCACTGGTGGGCTTCTGCATTAGCAAATCATCCT
GGACCCGCCTGGGCCGCCTCATGCCCTGGATCATCTTCTCCACACCCCTGGCCATCATTGCCTACTTCCT
TATCTGGTACGTGCCTGACTTCCCACAGGACCAGACCCTGTGGTATCTGCTTTTCTACTGCCTCTTTGAA
ACACTGGTCACGTGTTTCCATGTTCCCTACTCAGCTCTCACCATGTTCATCAGCACCGAGCAGAGTGAGA
GGGACTCTGCTACTGCATATCGGATGACTGTGGAGGTACTGGGCACAGTGCTGGGCACCGCAATCCAGGG
GCAAATCGTGGGCCAAGCGAATACACCTTGCCAGAACTCAAATGGCTCTACAGTGACCTCGGAAGGTATC
AATCGCACACACACAGCACCACCTCCCTCAAAGAAACGCAAAATGCATATCTGCTGGCAGCAGGAGTGATTG
CCTCCATCTATGTTTTCTGTGCTGTCATCCTGATCCTGGGTGTGCGGGAACAGAGAGAACCCTATGAAGC
TCAGAGGGCTGAGTCAATGTCCTTCTTTCGGGGTCTCCGGTTGGTCATGAGCCACGGCCCATATATCAAA
CTTATCGCTGGCTTTCTCTTCACCTCCCTAGCTTTCATGCTGGTAGAGGGGAACTTCGCCTTGTTCTGCA
CCTACACCTTAGGATTCCGCAATGAATTTCAGAATCTGCTCCTGGCCATCATGCTCTCAGCCACATTCAC
CATTCCCATCTGGCAATGGTTCCTAACCCGGTTTGGCAAGAAGACAGCTGTATATGTTGGGATCTCCTCA
GCAGTGCCTTTTCTGGTCTTGGTGGCCCTCATCGAGAGTAACCTGATTGTCACATACATAGTCGCTGTGG
CTGCCGGCATCAGTGTAGCAGCTGCCTTCTTACTACCCTGGTCCATGCTGCCCGATGTCATTGATGACTT
CCACTTGAAGCAGCCGCAGTCCCATGGAACCGAGCCCATCTTCTTCTCCTTCTACGTCTTCTTCACCAAG
TTTGCCTCTGGTGTCTCCCTGGGCATCTCCACCCTCAGTCTTGACTTTGCCGGATACCAGACCGGTAGCT
GCTCCCAGCCGGCAGGTGTCAAGTTTACTTTGAAGATGTTGGTGACCATGACTCCCATAGTCCTCATCCT

GCTGGGCCTGCTGCTCTTCAAACTATACCCTATTGATGAGGAGAAACGGCGGCAGAACAAGAAGGCCCTG
CAGGCTCTGAGGGAAGAGGCCAGTAGCTCGGGCTGCTCGGACACGGACTCTACAGAGCTAGCAAGCATTC
TC
>elephant ENSLAFT00000003074.3
ATGGCCAAAGGAGAGGGCGCCGAGGGCGGCTCGGCGGCGGGGCTGCTGCCCACGAGCATC
CTGCAAACCGGTGAACGCCCGGCCCACGTGAAAAAGGAACCGAAGAAGAAGCAACAGTTG
TCCATTTGCAACAAGCTTTGCTATGCTGTTGGGGGGGCCCCCTACCAGGTGACAGGCTGT
GCCCTGGGGTTCTTCCTGCAGATCTACCTGTTGATGTGGCTCAGGGTGGACCCTTTCTCT
GCCTCCATCATCCTGTTTGTGGGCCGTGCCTGGGATGCTATCACAGATCCCCTAGTGGGC
TTCTGCATTAGTAAATCCTCCTGGACCCGCCTGGGCCGTCTTATGCCCTGGATCATCTTC
TCCACACCCCCGGCCATCATTGCCTACTTCCTCATCTGGTTCGTGCCTGACTTCCCACAG
GGCCAGGCTCTGTGGTACCTGCTTTTCTACTGCCTCTTTGAGACACTGGTCACGTGTTTC
CACGTTCCCTACTCAGCTCTCACCATGTTCATCAGCACAGAGCAGAGTGAGCGGGATTCC
GCCACTGCATACCGGATGACTGTGGAGGTGTTGGGCACAGTGCTGGGCACAGCGATCCAG
GGGCAAATCGTGGGCCAAGTGGATACACCTTGTCTCCAGGACCCCAATGACTTTGCGATG
GCCTCAGAAGGTGTCAATCGCACACACAGCACCACTTCACTCAAAGAAACGCAAAATGCA
TACTTGCTGGCAGCAGGGGTCATTTCCTCCCTCTATGTCATCTGTGCTGTCATCCTGACC
CTGGGTGTGCGGGAACAGAGAGAACCCTACAAGACCCAGCAGACTGAGCACACGTCCTTC
TTCCGCGGCCTTCGGCTGGTCATGAGCCATGGCCCATATGTCAGGCTCATCGCTGGCTTC
CTCTTCACCTCCCTGGCCTTCATGCTGGTAGAGGGGAACTTCGCCTTGTTTTGCACCTAC
ACCTTGGGCTTCCGCAATGAATTCCAGAATCTACTCTTGGCCGTCATGCTCTCGGCCACA
TTTACCATTCCCGTCTGGCAGTGGTTCCTAACACGTTTTGGCAAGAAGACAGCTGTGTAC
ATTGGGATCTCATCAGCAGTGCCATTTCTTATCTTGGTGGCCCTCATGGACAGTAACCTG
ATTGTCACTTACGTGGTAGCTGTGGCAGCCGGCATCAGCGTGGCAGCTGCCTTCTTGTTG
CCCTGGTCCATGCTGCCCGACGTCATTGATGACTTCCATCTGAAGCAGCCCCACTCCCAT
GGAACTGAGCCCATCTTCTTCTCCTTCTACGTCTTCTTCACCAAGTTTGCCTCTGGAGTC
TCGCTGGGCATCTCCACCCTCAGTCTCGATTTTGCCGGATACCAGACCCGTGGTTGCTCC
CAGCCGGCACGGGTCAAGTTTACACTGAAGATGCTGGTGACCATGGCTCCCATAGTCCTC
ATCCTGCTGGGCCTGCTGCTCTTCAAGCTGTACCCCATTGACGAGGAGAAGCGGAGGCAG
AACAAGAAGGCCCTGCAGGCTCTGAGGGACGAGGCCGGCAGCTCGGGCTGCTCTGACACA
GACTCCACAGAGCTGGCCAGCATCCTC
>ferret ENSMPUT00000014400.1
ATGGCCAAAGGAGAGGGCGCCGAGGGCGGCTCCGCGGCGGGGCTGCTACCCACCGGCATC
CTCCCAGCCGCTGAACGGCCGGCCCAGGTGAAGAAGGAACCCAAGAAGAAACAACAGTTG
TCCATTTGCAACAAGCTTTGCTATGCAGTTGGGGGGGCCCCCTACCAGGTGACAGGCTGT
GCCCTGGGGTTCTTCCTGCAGATCTACCTGTTGGATGTGGCTCAGGTGGACCCTTTCTCT
GCCTCCATCATCCTGTTTGTGGGCCGAGCTTGGGATGCCGTCACAGACCCCTTGGTGGGC
TTCTGCATTAGCAAATCTTCCTGGACCCGCCTGGGCCGCCTCATGCCCTGGATCATCTTC
TCCACACCCCTGGCCATCATTGCTTACTTCCTCATCTGGTTCGTGCCTGACTTCCCACAG
GGCCAGGCCCTGTGGTACCTGCTTTTCTATTGCCTGTTTGAAACACTGGTCACGTGTTTC
CACGTTCCCTACTCAGCTCTCACGATGTTCATCAGCACAGAGCAGAGTGAGCGGGATTCT
GCCACTGCATATCGGATGACTGTGGAGGTAATGGGCACAGTGCTGGGCACGGCGATCCAG
GGGCAAATTGTGGGACAAGCGGATACGCCTTGCCTCCAGGACCCCGCTGACTCCGCACTG
GCCACGGAAGGTGCCAATCGGACACAGAGCACCACCTCCCTGAGAGAAACGCAAAATGCG
TACCTGTTAGCGGCAGGGGTCATTGCTTCCATCTATGTCATCTGTGCTGTCATCCTGACC
CTGGGCGTGAGGGAGCAGAGAGAACCCTATGAGACTCAGCAGGCTGAGCCAATATCCTTT
TTTCGGGGCCTCCGGCTGGTCATGGGCTATGGCCCCTACGTCAAGCTTATTGCCGGCTTC

CTCTTCACCTCCCTGGCTTTTATGCTGGTGGAGGGGAACTTCGCCTTGTTTTGCACCTAC
ACCCTGGGCTTCCGAAACGAATTCCAGAATCTGCTCCTGGCCATCATGCTCTCGGCCACA
TTCACCATTCCCATCTGGCAGTGGTTCCTAACCCGATTTGGCAAGAAGACGGCCGTCAAC
ATTGGGATCTCATCAGCAGTGCCATTTCTCATCTTGGTGGCCTTCATGGAGAGCAACCTG
ATTGTCACCTACGTGGTAGCTGTGGCAGCTGGCATCAGTGTAGCTGCGGCCTTCTTATTA
CCCTGGTCCATGCTGCCTGATGTCATCGACGACTTCCATCTGAAGCAGCCCCAGTTTCAT
GGAACCGAGCCCATCTTCTTCTCCTTTTACGTCTTCTTCACCAAGTTCGCCTCCGGAGTC
TCCCTAGGCATCTCCACCCTCAGTCTTGACTTTGCCGGGTACCAGAGCCGTGGCTGCTCC
CAGCCGGCACGTGTCAAATTCACCCTGAAGATGCTGGTGACCATAGCTCCCATAGCCCTC
ATCCTGCTAGGCCTGCTGCTCTTCACACTGTACCCCATCGATGAGGAGAAGCGGCGGCAG
AACAAGAAGGCCCTGCAGGCTCTGAGGGAAGAGGCCAGCAGCTCTGGCTGCTCAGACACA
GACTCTACAGAGCTGGCCAGCATCCTC
>myotis lucifagus ENSMLUT00000000338.2
ATGGCCAAAGGAGAGGGCGCCGAGAGCGGCTCCGCCACGGGGCTGCTGCCTACAAGCATC
CTCCAAGTTGGTGAACGCCCGGCCCAGGTGAAGAAGGAACCAAAGAAGAAACAACAGTTG
TCCATTTGCAACAAGCTTTGCTATGCAGTTGGGGGGGCCCCCTACCAGATGACGGGCTGT
GCCCTGGGGTTCTTCCTACAGATCTACCTGTTGGATGTGGCTCAGGTGGACCCATTCTCT
GCCTCTATCATCCTATTTGTGGGCCGAGCTTGGGATGCCTTCACAGACCCCCTGGTGGGC
TTCTGCATAAGCAAATCTTCCTGGACCTGTCTGGGCCGTCTTATGCCCTGGATCATCTTC
TCCACACCCCTGGCCATCATTGCCTACTTCCTCATATGGTTCGTGCCTGACTTCCCACAG
GGCCAGATCCTATGGTACCTGCTTTTCTATTGCCTCTTTGAGACACTAGTCACGTGTTTC
CACGTTCCCTACTCAGCTCTCACCATGTTCATCAGCACAGAACAGAGTGAGCGGGATTCG
GCCACTGCTTACCGGATGACTGTGGAGGTACTGGGCACAGTGCTGGGCACAGCGATCCAA
GGGCAAATCGTAGGCCAAGCAAATATGCCTTGTCTTGAGGACCCCAAAGATTCAGCAGTG
GCCACGGAAGATGCCAGTCATACACACAGCACCACCTCACTCAGAGAAACGAAAAATGCA
TACCTGCTGGCGGCAGGGGTCATAGCCTCCATCTATGTCATCTGCGCTGTCATCCTGGTC
CTGGGCGTGAGGGAACAGAGAGAAGCCTATGAGACTCAGCAGGCTGAGCCAATACCCTTC
TTTCGGGGCCTCCGACTGGTCATGAGCCATGGCCCATATATTAAGCTTATTGCTGGCTTC
CTCTTCACCTCCCTGGCTTTCATGCTGGTGGAGGGGAACTTCGCCTTGTTTTGCACCTAC
ACCTTGGGCTTCCGAAACGAATTCCAGAATCTGCTCCTGGCCATCATGCTCTCGGCCACA
TTCACCATTCCCTTCTGGCAGTGGTTTCTAACCCAGTTTGGCAAGAAGACGGCTGTATAT
ATTGGGATCTCATCAGCAGTACCATTTCTCATCCTGGTGGCCCTCATAGAGAGTAACCTG
ATTGTCACGTATGTGGTAGCTGTGGCAGCTGGCATCAGTGTAGCAGCTGCCTTCTTACTA
CCCTGGTCCATGCTGCCCGATGTCATTGACGACTTCCACTTGAAGCACCCCCATTCCCAT
GGAACTGAGCCCATCTTCTTCTCCTTCTATGTCTTCTTCACCAAGTTCGCCTCTGGAGTC
TCACTGGGCGTCTCTACCCTCAGTCTTGACTTTGCCGGATACCAGACCCGTGGCTGCTCC
CAGCCAGCGCCCGTCAGGTTTACATTGAAGATTCTGGTGACAATGGCTCCCATAGTCCTC
ATCCTACTAGGCCTGCTGCTCTTTGAGCTGTACCCCATTGATGAGGAGAAGCGGCGGCAG
AACAAGAAGGCCCTCCAGGCTCTGAGGGAAGAGGCCAGCAGCTCAGGCTGCTCTGACACA
GACTCTACAGAGTTGGCTAGCATCCTC
>sheep XM_004001833.3
ATGGCCAAA
GGAGAGAGCGCCGAGAGCGGCTCCGCCGCGGGACTTCTGCCCACCGGCATCCTCCAAGCCGGTGAACGCC
CGGTCCAGGTGAAGAAGGAACCAAAGAAGAAAAAACAGTTGTCCATTTGCAACAAACTTTGCTATGCAGT
TGGGGGAGCCCCATACCAGGTGACGGGATGTGCCCTGGGGTTCTTCCTGCAGATCTACCTGTTGGATGTA
GCTCAGGTGGATCCTTTCTCTGCTTCCATCATCCTGTTCGTGGGCCGAGCTTGGGATGCCATCACAGACC
CCCTGGTGGGCTTCTGCATTAGCAAATCTCCCTGGACCCCTCTGGGCCGCCTCATGCCCTGGATCACCTT

CTCCACACCCCTGGCCGTCATCGCCTACTTCCTCATCTGGTTCGTGCCCGGCTTCCACCAGGGCCAGACC
CTGTGGTACTTGCTTTTCTACTGCCTCTTTGAAACGCTGGTCACGTGTTTCCACGTTCCCTACTCAGCTC
TCACCATGTTCATCAGCACGGAGCAGAGTGAGCGCGATTCTGCCACTGCCTATCGGATGACCGTGGAGGT
ATTAGGTACAGTGCTGGGCACAGCAATCCAGGGGCAGATCGTGGGCCAAGCAGATTCGCCTTGTATCCCG
GATGGCAATGCTTCTACAGTCAATCGTACGCAGAGCTCCACTTCAATCAAAGAAACACAAAATGCATACC
TGCTGGCGGCAGGAGTCATCGCCTCCATCTATGTCATCTGTGCGGTCATCCTGACCCTGGGCGTGCGGGA
ACAGAGAGAATCCTACGAGACTCAGCAGACCAAGCAGATGCCCTTCTTTCGGGGCCTCCGGCTGGTCATG
AGCCATGGGCCGTACATCAAGCTCATCGCCGGCTTCCTCTTCACCTCCTTGGCTTTCATGCTGGTGGAGG
GGAACTTTGCCTTGTTTTGTACCTATACCTTGGGCTTTCGGAACGAATTCCAGAATCTGCTCCTGGCAAT
CATGTTCTCAGCCACAGTCACCATCCCCATCTGGCAGTGGTTCCTAACCCGGTTTGGCAAAAAGACAGCT
GTATACATTGGAATCTCGTCAGCAGTGCCATTTCTCATCTTGGTGGCCCTCATGGAGAGTAACCTGATTG
TCACATACGTGGTGGCTGTGGCAGCTGGCATCAGTGTTGCAGCTGCCTTCTTACTACCGTGGTCCATGCT
GCCCGACGTCATTGATGACTTCCACTTGAAGCAGCCCCACATCCATGGGACCGAGCCCATCTTCTTCTCC
TTCTACGTCTTCTTCACCAAGTTTGCCTCTGGAGTCTCTCTGGGCATCTCCACCCTCAGTCTGGACTTCA
CTGGGTACCAGACCCGTGGCTGCTCCCAGCCGGCACGTGTCAAGTTCACGCTGAAGATGCTGGTGACCGT
GGCTCCCATAGTCCTCATCCTCATGGGCCTGCTGCTCTTCAAGCTATACCCCATTGACGAGGAGAGGCGG
CGGCAGAACAAGAAGGCCCTGCAGGCTCTGAGGGAAGAGGCCAGCAGCTCAGGCTGCTCTGACACAGACT
CTACAGAACTGGCCAGCATCCTC
>pteropus vampyrus ENSPVAT00000003351.1
ATGGCCAAAGGAGAGGGCGCCGAGAGCGGCTCCGCGGCGGGGCTGCTGCCCACAGGCGTC
CTTAAAGCTGGTGAACATCCGGCCCAGGTGATGAAGGAACCAAAGAAGAAACAACAGTTG
TCCATTTGCAACAAGCTTTGCTATGCAGTTGGGGGGGCCCCATACCAGGTGACGGGCTGT
GCCCTAGGGTTCTTCCTGCAGATCTACCTGTTGGATGTGGCTCAGT
TAGACCCATTCTCTGCCTCCATCATCCTATTCGTGGGCCGAGCTTGGGATGCCTTCACA
GACCCCCTGGTGGGCTTCTGCATTAGCAAATCCTCCTGGACCCGGCTGGGCCGCCTCATG
CCCTGGATTGTCTTCTCCACACCCCTGGCCATCATTGCCTACTTCCTCATCTGGTTCGTC
CCTGACTTCCCACAGGGCCAGGCCCTGTGGTACCTGCTTTTCTATTGCCTCTTTGAGACA
CTGGTCACATGTTTCCACGTTCCCTACTCAGCTCTCACCATGTTCATCAGCACAGAGCAG
AGTGAGCGGGATTCAGCCACTGCCTATCGGATGACTGTGGAGGTATTGGGCACAGTGCTG
GGAACAGCAATCCAGGGGCAAATTGTAGGCCAAGCGGATACGCCTTGTCTCCAGGTCCCC
AATGGTTCAGAAGTGGCCTCGGAAGGTGCCAATCGCACACACAGCTCCACTTCACTCAGA
GGAACGCAAAATGCATACCTGCTGGCAGCAGGGGTCATTGCCTCCATCTATGTCATCTGT
GCTGTCATCCTGGTCCTGGGTGTGCGAGAGCAGAGAGAACCCTATGAGACTCAGCAGGCT
GAGCCAATGTCCTTCTTTCGGGGCCTCCGGCTGGTCATGAGCCACGGCCCATATATCAAG
CTTATCGCTGGCTTCCTCTTCACCTCCCTGGCATTCATGCTGGTGGAAGGGAACTTTGCT
TTATTTTGTACCTACACCTTGGGCTTCCGCAACGAATTCCAGAATCTGCTCCTGGCCATC
ATGCTCTCAGCCACATTCACCATTCCTCTTTGGCAGTGGTTCCTAACTCGGTTTGGCAAG
AAAACGGCTGTATACATCGGGATCTCATCAGCAGTGCCGTTTCTCATCTTGGTGGCCCTC
AAGGAGAGCAACCTGATTGTCACATACGTAGTAGCCGTGGCAGCTGGCATCAGTGTAGCA
GCCGCCTTCTTACTACCCTGGTCCATGCTGCCCGACGTCATTGACGACTTCCTCTTGAAG
CAGCCTCACTCCCATGGAACCGAGCCCATCTTCTTCTCCTTCTATGTTTTTTTCACCAAG
TTTGCCTCTGGAGTCTCACTGGGCATCTCTACTCTCAGTCTTGACTTTGCTGGGTACCAG
ACCCGTGGCTGCTCCCAGCCAGCACGTGTCAGGTTTACACTGAAGATGCTGGTGACAATG
GCTCCCATAGTCCTCATCCTGCTAGGCCTGCTGCTCTTCAAGCTATACCCCATTGATGAA
GAGAAGCGGCGGCAGAACAAGAAGGCACTGCAGGCTCTGAGGGAAGAGGCCAGCAGCTCA
GGCTGCTCTGACACAGACTCTACAGAGCTGGCCAGCATCCTC

>pig ENSSSCT00000004065.2
ATGGCTAAAGGAGAGGGCGCCGAGAGCGGCTCCGCGGCGGGGCTGCTGCCCACCGGCATC
CTCCAAGCGGGTGAACGTCCGGTCCAGGTGAAGAAGGAGCCGAAGAAGCAAAAACAGTTG
TCCGTTTGCAACAAGCTTTGCTATGCAGTTGGGGGGGCCCCCTACCAGGTGACGGGATGT
GCCCTGGGGTTCTTCCTGCAGATCTACCTGTTGGATGTAGCTCAGGTGGATCCTTTCTCC
GCCTCCATCATCCTATTTGTGGGCCGAGCTTGGGATGCCATCACAGACCCCCTGGTGGGC
TTCTGCATTAGCAAATCCTCCTGGACCCGCCTGGGCCGCCTCATGCCCTGGATCATCTTC
TCCACACCCCTGGCCATCATCGCCTACTTCCTCATCTGGTTCGTGCCTGACTTCAAGCAG
GGCCAGGCCCTGTGGTACTTGCTTTTCTACTGCCTCTTTGAGACACTGGTCACGTGTTTC
CACGTTCCCTACTCAGCTCTCACCATGTTCATCAGCACGGAGCAGAGCGAGCGGGATTCT
GCCACTGCGTATCGGATGACCGTGGAGGTATTAGGGACAGTGCTGGGCACGGCCATCCAG
GGGCAGATCGTGGGCCAAGCAGATACGCCTTGTATCCAGGATGTTAATGCTTCTACAGTA
GCCTCGGAAGGTGTCAATCTCACACAGAGTGCCACCTCGCTCAAAGAAACGCAAAATGCA
TACCTGCTGGCGGCAGGGGTCATTGCCTCCATCTATGTCATCTGTGCCATCATCCTGATC
CTGGGCGTGCGGGAGCAGAGAGAACCATACGAGACTCAGCAGACCAAGCCGATGTCCTTC
TTTCCGGGCCTCCGGCTGGTCATGAGCCATGGCCCCTACATCAAGCTTATTGCCGGCTTC
CTCTTTACCTCCTTGGCTTTCATGCTGGTGGAGGGGAACTTCGCCCTGTTCTGCACATAC
ACCTTGGGCTTTCGCAATGAATTCCAGAATCTGCTCCTGGCCATCATGCTCTCAGCCACA
ATCACCATTCCCATCTGGCAGTGGTTCCTAACCCGGTTTGGCAAGAAGATGGCTGTGTAC
ATCGGGATCTCATCAGCAGTGCCATTTCTCATCTTGGTGGCCCTCATGGAAAGTAACCTG
ATCGTCACATACGTGGTAGCAGTAGCAGCCGGCATCAGCGTAGCAGCTGCTTTCTTACTA
CCCTGGTCCATGCTCCCCGACGTCATTGACGACTTCCACCTGAAGCAGCCCCACATCCAC
GGGACGGAGCCCATCTTCTTCTCCTTCTACGTCTTCTTTACCAAGTTTGCCTCTGGAGTC
TCCCTGGGCATCTCCACCCTCAGCCTGGACTTCACTGGGTACCAGACCCGAGGCTGCTCC
CAGCCGGCACGGGTCAAGTTCACGCTGAAGATGCTGGTGACCATGGCTCCCATAGTCCTC
ATCCTGCTGGGCCTGTTGCTCTTCAAGCTGTACCCCATTGATGAGGAGAAGCGGCGGCAG
AACAAGAAGGCCCTGCAGGCTCTGAGGGAAGAGGCCAGCAGCTCAGGCTGCTCTGAAACA
GACTCTACAGAACTGGCCAGCATCCTC
>Equus asinus XM_014828616.1
ATGGCCAAAGGAGAGGGCGCCGAGAGCGGCTC
CGCGGCGGGGCTGCTGCCCGCGGGCATTATACAAGCGGGTGAACGTCCGGCCCAGGTGAAGAAGGAACCG
AAGAAGAAACAACAGTTGTCCGTTTGCAGCAAGCTTTGCTATGCAGTTGGAGGGGCCCCCTACCAGGTGA
CAGGCTGTGCCCTGGGGTTCTTCCTGCAGATCTACCTGTTGGATGTGGCTCAGGTGGACCCTTTCTCTGC
CTCCATCATCCTATTTGTGGGCCGGGCTTGGGATGCCATCACAGATCCCCTGGTGGGTTTCTTCATTAGC
AAGTCTTCGTGGACCCGCCTGGGCCGCCTCATGCCCTGGATCATCTTCTCCACGCCCCTGGCCGTCGTTG
CCTACTTCCTCATCTGGTTCGTGCCTGACTTCCCACAGGGCCAGAGCCTGTGGTACCTGCTTTTCTATTG
CCTCTTTGAGACACTGGTCACATGTTTCCACGTTCCCTACTCAGCTCTCACCATGTTCATCAGCACAGAA
CAGAGTGAGCGGGATTCTGCCACTGCATATCGGATGACTGTGGAGGTGCTGGGCACAGTGCTGGGAACAG
CGATCCAGGGGCAAATCGTGGGCCAAGCGGATACACCTTGTCTCCAGGACCGCAACATTTCTGCAGTGGC
CTTGGAAGGTGCCAATCGCACACACAGCACCACCTCACTCAGAGAAACGCAAAATGCATACCTGCTGGCA
GCAGGGGTCATTGCCACCATCTATATCATCTGTGCTGTCATCCTGATCCTGGGCGTGCGGGAGCAAAGAG
AACCCTATGAGACTCAGCAGGCTGAGCCGATGTCCTTCTTTCGGGGCCTCCGGCTGGTCATGAGCCACGG
CCCGTACGTCAAGCTTATTGCCGGCTTCCTCTTCACCTCCCTGGCTTTCATGCTGGTGGAAGGGAACTTC
GCCTTGTTTTGCACCTACACCCTGGGCTTCCGCAATGAATTCCAGAATCTGCTCCTGGCCATCATGCTCT
CGGCCACATTCACCATCCCCATCTGGCAATGGTTCCTAACCCGGTTTGGCAAAAAGACGGCTGTATACAT
TGGGATCTCTTCAGCAGTGCCATTTCTCACCTTGGTGGCCCTCATGGAGCGTAACCTGATCATCACATAC
GTGGTAGCTGTGGCAGCTGGCATCAGTGTAGCAGCTGCCTTCTTACTACCCTGGTCCATGCTGCCCGACG

TCATTGACGACTTCCACTTGAAGCAGCCCCACTCCCGTGGAACCGAGCCCATCTTCTTCTCCTTCTATGT
CTTCTTCACCAAGTTCGCCTCCGGAGTCTCTCTGGGCATCTCCACCCTCAGTCTTGACTTTGCCGGCTAC
CAAACCCGTGGCTGCACCCAGCCGAGGCGCGTCAAGTTTACGCTGAAGATGCTGGTGACCATGACTCCCA
TAGTCCTCATCCTGCTGGGCCTGCTGCTCTTCAAACTGTACCCCATTGACGAGGAGAAGCGGCGACAGAA
CAAGAAAGCCCTGCAGGCTCTGCGGGAAGAGGCCAGCAGCTCGGGCTGCTCTGACACAGACTCTACAGAG
CTGGCCAGCATCCTC
>Equus caballus XM_001503368.3
ATGGCCAAAGGAGAGGGCTCCGAGAGCGG
CTCCGCGGCGGGGCTGCTGCCCGCGGGCATTATACAAGCGGGTGAACGTCCGGCCCAGGTGAAGAAGGAA
CCGAAGAAGAAACAACAGTTGTCCGTTTGCAGCAAGCTTTGCTATGCAGTTGGAGGGGCCCCCTACCAGG
TGACAGGCTGTGCCCTGGGGTTCTTCCTGCAGATCTACCTGTTGGATGTGGCTCAGGTGGACCCTTTCTC
TGCCTCCATCATCCTATTTGTGGGCCGGGCTTGGGATGCCATCACAGATCCCCTGGTGGGTTTCTTCATT
AGCAAGTCTTCGTGGACCCGCCTGGGCCGCCTCATGCCCTGGATCATCTTCTCCACGCCCCTGGCCGTCG
TTGCCTACTTCCTCATCTGGTTCGTGCCTGACTTCCCACAGGGCCAGAGCCTGTGGTACCTGCTTTTCTA
TTGCCTCTTTGAGACACTGGTCACATGTTTCCACGTTCCCTACTCAGCTCTCACCATGTTCATCAGCACA
GAGCAGAGTGAGCGGGATTCTGCCACTGCATATCGGATGACTGTGGAGGTGCTGGGCACAGTGCTGGGAA
CAGCGATCCAGGGGCAAATCGTGGGCCAAGCGGATACACCTTGTCTCCAGGACCGCAACATTTCTGCAGT
GGCCTTGGAAGGTGCCAATCGCACACACAGCACCACCTCACTCAGAGAAACGCAAAATGCATACCTGCTG
GCAGCAGGGGTCATTGCCACCATCTATATCATCTGTGCTGTCATCCTGATCCTGGGCGTGCGGGAGCAGA
GAGAACCCTATGAGACTCAGCAGGCTGAGCCGATGTCCTTCTTTCGGGGCCTCCGGCTGGTCATGAGCCA
CGGCCCGTACGTCAAGCTTATTGCCGGCTTCCTCTTCACCTCCCTGGCTTTCATGCTGGTGGAAGGGAAC
TTCGCCTTGTTTTGCACCTACACCCTGGGCTTCCGCAATGAATTCCAGAATCTGCTCCTGGCCATCATGC
TCTCGGCCACATTCACCATCCCCATCTGGCAATGGTTCCTAACCCGGTTTGGCAAAAAGACGGCTGTATA
CGTTGGGATCTCTTCAGCAGTGCCATTTCTCACCTTGGTGGCCCTCATGGAGCGTAACCTGATCATCACA
TACGTGGTAGCTGTGGCAGCTGGCATCAGTGTAGCAGCTGCCTTCTTACTACCCTGGTCCATGCTGCCCG
ACGTCATTGACGACTTCCACTTGAAGCAGCCCCACTCCCGTGGAACCGAGCCCATCTTCTTCTCCTTCTA
TGTCTTCTTCACCAAGTTCGCCTCCGGAGTCTCACTGGGCATCTCCACCCTCAGTCTTGACTTTGCCGGC
TACCAAACCCGTGGCTGCACCCAGCCGAGGCGCGTCAAGTTTACGCTGAAGATGCTGGTGACCATGACTC
CCATAGTCCTCATCCTGCTGGGCCTGCTGCTCTTCAAACTGTACCCCATTGACGAGGAGAAGCGGCGACA
GAACAAGAAAGCCCTGCAGGCTCTGCGGGAAGAGGCCAGCAGCTCGGGCTGCTCTGACACAGACTCTACA
GAGCTGGCCAGCATCCTC
>pteropus alecto XM_006903964.2
ATGGCCAAAGGAGAGGGCGCCGAGAGCGGCTCCGCGGCGGGGCTGCTGCCCACAGGCGTCCTT
AAAGCTGGTGAACATCCGGCCCAGGTGATGATGGAACCAAAGAAGAAACAACAGTTGTCCATTTGCAACA
AGCTTTGCTATGCAGTTGGGGGGGCCCCATACCAGGTGACGGGCTGTGCCCTAGGGTTCTTCCTGCAGAT
CTACCTGTTGGATGTGGCTCAGGTAGACCCATTCTCTGCCTCCATCATCCTATTCGTGGGCCGAGCTTGG
GATGCCTTCACAGACCCCCTGGTGGGCTTCTGCATTAGCAAATCCTCCTGGACCCGGCTGGGCCGCCTCA
TGCCCTGGATTGTCTTCTCCACACCCCTGGCCATCATTGCCTACTTCCTCATCTGGTTCGTCCCTGACTT
CCCACAGGGCCAGGCCCTGTGGTACCTGCTTTTCTATTGCCTCTTTGAGACACTGGTCACATGTTTCCAC
GTTCCCTACTCAGCTCTCACCATGTTCATCAGCACAGAGCAGAGTGAGCGGGATTCAGCCACTGCCTATC
GGATGACTGTGGAGGTATTGGGCACAGTGCTGGGAACAGCAATCCAGGGGCAAATTGTAGGCCAAGCGGA
TACGCCTTGTCTCCAGGTCCCCAATGGTTCAGAAGTGGCCTCGGAAGGTGCCAATCGCACACACAGCTCC
ACTTCACTCAGAGAAACGCAAAATGCATACCTGCTGGCAGCAGGGGTCATTGCCTCCATCTATGTCATCT
GTGCTGTCATCCTGGTCCTGGGTGTGCGAGAGCAGAGAGAACCCTATGAGACTCAGCAGGCTGAGCCAAT
GTCCTTCTTTCGGGGCCTCCGGCTGGTCATGAGCCACGGCCCATATATCAAGCTTATCGCTGGCTTCCTC
TTCACCTCCCTGGCATTCATGCTGGTGGAAGGGAACTTTGCTTTATTTTGCACCTACACCTTGGGCTTCC
GCAACGAATTCCAGAATCTGCTCCTGGCCATCATGCTCTCAGCCACATTCACCATTCCTCTCTGGCAGTG

GTTCCTAACTCGGTTTGGCAAGAAAACGGCTGTATACATCGGGATCTCATCAGCAGTGCCGTTTCTCATC
TTGGTGGCCCTCAAGGAGAGCAACCTGATTGTCACATACGTAGTAGCCGTGGCAGCTGGCATCAGTGTAG
CAGCCGCCTTCTTACTACCCTGGTCCATGCTGCCCGACGTCATTGACGACTTCCTCTTGAAGCAGCCTCA
CTCCCATGGAACCGAGCCCATCTTTTTCTCCTTCTACGTTTTTTTCACCAAGTTTGCCTCTGGAGTCTCA
CTGGGCATCTCTACTCTCAGTCTTGACTTTGCTGGGTACCAGACCCGTGGCTGCTCCCAGCCAGCACGTG
TCAGGTTTACACTGAAGATGCTGGTGACAATGGCTCCCATAGTCCTCATCTTGCTAGGCCTGCTGCTCTT
CAAGCTATACCCCATTGATGAGGAGAAGCGGCGGCAGAACAAGAAGGCACTGCAGGCTCTGAGGGAAGAG
GCCAGCAGCTCAGGCTGCTCTGACACAGACTCTACAGAGCTGGCCAGCATCCTC
>Lipotes vexillifer XM_007453760.1
ATGGCCAAAGGAGAGGGCGCCGA
GAGCGGCTCCGCCGCGGGGCTGCTGCCTACGGGCATCCTCCAAGCCGGTGAACGCCCGGTCCAGGTGAAG
AAGGAATCGAAGAAGAAAAACCAGTTGTCCATTTGCAACAAACTTTGCTATGCAGTTGGGGGGGCCCCCT
ACCAGGTGACGGGTTGTGCCCTGGGGTTCTTCCTGCAGATCTACCTGTTGGATGTAGCTCAGGTGGATCC
TTTCTCTGCTTCCATCATCCTGTTTGTGGGCCGAGCTTGGGATGCCATCACAGACCCCCTGGTGGGCTTC
TGCATTAGCAAATCCCCCTGGACCCGTCTAGGCCGCCTCATGCCCTGGATCATCTTCTCCACACCCCTGG
CCATCATCGCCTACTTCCTCATCTGGTTTGTGCCTGACTTCCAACAGGGCCAGACCCTGTGGTACTTGCT
TTTCTACTGCCTCTTTGAGACGCTGGTCACGTGTTTCCACGTTCCCTACTCAGCTCTCACCATGTTCATC
AGCACGGAACAGAGTGAGCGGGATTCTGCCACTGCGTATCGGATGACCGTGGAGGTGTTAGGTACAGTGC
TGGGCACAGCAATCCAGGGGCAGATTGTGGGCCAAGCACATACACCTTGTGTCCAGGATGCCAATGCTTC
TACGGTAGGCTTGGAAGGTGTCAATCGCACGCAGAGCACCACCTCGCTCAGAGAAACGCAAAACGCATAC
CTGCTGGCGGCAGGGGTCATTGCCTCCATCTATGTCATCTGCGCTGTCATCCTGACCCTGGGCGTGCGGG
AGCAGAGAGAACCCTACGAGACTCAGCAGGCCAAGCCGATGTCCTTCTTTCGGGGCCTCCGGCTGGTCAT
GAGCCATGGCCCGTACATCAAACTTATTGCCGGCTTCCTCTTCACCTCCTTGGCTTTCATGCTGGTGGAG
GGGAACTTCGCTCTGTTTTGCACCTACACCTTGGGTTTTCGCAACGAATTCCAGAATCTGCTCCTGGCCA
TCATGTTCTCGGCCACAGTCACCATCCCCATCTGGCAGTGGTTCCTAACCCGGTTTGGCAAGAAGATGGC
TGTGTACATTGGGATCTCATCAGCAGTGCCATTTCTCATCTTGGTGGCCCTCATGGAGAGTAACCTTATT
GTCACATATGTGGTAGCTGTGGCAGCTGGCATCAGTGTAGCAGCTGCCTTCTTACTACCCTGGTCCATGC
TGCCTGACGTCATTGATGACTTCCGCTTGAGGCAGCCCCGTAGCCATGGGACCGAGCCCATCTTCTTCTC
CTTCTACGTCTTCTTCACCAAGTTTGCCTCCGGAGTCTCCCTGGGCATCTCCACCCTCAGTCTGGACTTT
ACTGGGTACCAGACCCGTGGCTGCTCCCAGCCAGCACGTGTCAAGCTCACACTGAAGATGCTGGTGACCA
TGGCGCCCATAGTCCTCATCCTCATAGGCCTGCTGCTCTTTAAGCTGTACCCCATCGACGAGGAGAGGCG
GCGGCAGAACAAGAAGGCCCTGCAGGCTCTGAGGGAAGAGGCCAGCAGCTCGGGCTGCTCTGACACAGAC
TCTACAGAACTAGCCAGAATCCTC
>Odobenus rosmarus divergens XM_004415367.2
ATGGCCAAAGGAGAGGGCGCGGAGAGCGGCTCCGCGG
CGGGGCTGCTGCCCACCGGCATCCTCCCAGTCGCTGAACGGCCGGCCCAGGTGAAGAAGGAACCCAAGAA
GAAACAACAGTTGTCCATTTGCAACAAGCTTTGCTATGCAGTTGGGGGGGCCCCCTACCAGGTGACAGGC
TGTGCCCTGGGGTTCTTCCTGCAGATCTACCTGTTGGATGTGGCTCAGGTGGACCCTTTCTCTGCCTCCA
TCATCCTGTTTGTGGGCCGAGCTTGGGATGCCTTCACAGACCCCCTGGTGGGCTTCTGCATTAGCAAATC
TTCCTGGACCCGCCTGGGCCGCCTCATGCCCTGGATCATCTTCTCCACACCCCTGGCCATCATTGCTTAC
TTCCTCATCTGGTTCGTGCCTGACTTCCCACAGGGCCAGGCCCTGTGGTACCTGCTTTTCTATTGCCTGT
TTGAAACACTGGTCACGTGTTTCCACGTTCCCTACTCAGCTCTCACGATGTTCATCAGCACAGAGCAGAG
TGAGCGGGATTCTGCCACTGCATATCGGATGACTGTGGAGGTACTTGGCACAGTGCTGGGCACAGCGATC
CAGGGGCAAATCGTGGGCCAAGTGGATACGCCTTGCCTCCAGGACCCCAAGGACTCTGCACTGGCTACGG
AAGGTGCCAATCGGACACAGAGCACCACGTCACTCAAAGAAACGCAAAATGCATACCTGCTGGCAGCAGG
GGTCATTGCCTCCATCTATGTCGTCTGTGCTGTCATCCTGACCCTGGGCGTGCGGGAGCAGAGAGAACCC
TATGAGACTCAGCAGGCTGAGCCGATGTCCTTTTTTCGGGGCCTCCGGCTGGTCATGAGCCATGGCCCAT

ATGTCAAACTTATTGCTGGCTTCCTCTTTACCTCCCTGGCTTTCATGCTGGTGGAGGGGAACTTCGCCTT
GTTTTGCACCTACACCTTGGGCTTCCGAAATGAATTCCAGAATCTGCTCCTGGCCATCATGCTCTCGGCC
ACGTTCACCATTCCCATCTGGCAGTGGTTCCTAACCCGATTTGGCAAGAAGACGGCGGTATACGTTGGGA
TCTCATCAGCAGTGCCATTTCTCATCTTGGTGGCCTTCACGGAGAGTAACCTGATTGTCACCTACGTGGT
AGCTGTGGCAGCTGGCATCAGTGTGGCAGCGGCCTTCTTACTACCCTGGTCCATGCTGCCTGATGTCATC
GACGACTTCCACCTGAAGCAGCCCCAGTCTCATGGAACTGAGCCCATCTTCTTCTCCTTCTATGTCTTCT
TCACCAAGTTCGCCTCCGGAGTCTCCCTGGGCATCTCCACCCTCAGTCTTGACTTTGCCGGGTACGAGAC
CCGTGGCTGCTCACAGCCGGCACGTGTCAAGTTCACCCTGAAGATGCTGGTGACCATAGCTCCCATAGCC
CTCATCCTGCTAGGCCTGCTGCTCTTCAAGCTGTACCCCATCGATGAGGAGAAGCGGCGGCAGAACAAGA
AGGCCCTGCAGGCTCTGAGGGAGGAGGCCAGCAGCTCTGGCTGTTCTGACACAGACTCTACGGAGCTGGC
CAGCATCCTC

>orcinus orca XM_004266404.2
ATGGCCAAAGGAGAGGGCGCCGAGAGCGGCTCCGCCGCGGGGCTG
CTGCCCACGGGCATCCTCCAAGCCGGTGAACGCCCGGTCCAGGTGAAGAAGGAGTCGAAGAAGAAAAACC
AGTTGTCCATTTGCAACAAACTTTGCTATGCAGTTGGGGGGACACCCTACCAGGTGACGGGTTGTGCCCT
GGGGTTCTTCCTGCAGATCTACCTGTTGGATGTAGCTCAGGTGGATCCTTTCTCTGCTTCCATCATCCTG
TTTGTGGGCCGAGCTTGGGATGCCATCACAGACCCCCTGGTGGGCTTCTGCATTAGCAAATCCCCCTGGA
CCCGCCTAGGCCGCCTCATGCCCTGGATCATCTTCTCCACGCCCCTGGCCATCATCGCCTACTTCCTCAT
CTGGTTCGTGCCTGACTTCCAACAGGGCCAGACCCTGTGGTACTTGCTTTTCTACTGCCTCTTTGAGACG
CTGGTCACGTGTTTCCACGTTCCCTACTCAGCTCTCACCATGTTCATCAGCACGGAACAGAGTGAGCGGG
ATTCTGCCACTGCGTATCGGATGACCGTGGAGGTATTAGGTACAGTGCTGGGCACAGCAATCCAGGGGCA
GATTGTGGGCCAAGCACATACACCTTGTGTCCAGGACGCCAATGCTTCTACAGTAGCCTTGGAAGGTGTC
AATCGCACGCAGAGCGCCACCTCGCTCAGAGAAACGCAAAACGCATACCTGCTGGCGGCAGGGGTCATTG
CCTCCATCTATGTCATCTGCGCTGTCATCCTGACCCTGGGCGTGCGGGAGCAGAGAGAACCCTACGAGAC
TCAGCAGGCCAAGCCGATGCCCTTCTTTCGGGGCCTCCGGCTGGTCATGAGCCATGGCCCGTACATCAAG
CTTATTGCCGGCTTCCTCTTCACCTCCTTGGCTTTCATGCTGGTGGAGGGGAACTTCGCTCTGTTTTGCA
CCTACACCTTGGGTTTTCGTAACGAATTCCAGAATCTGCTCCTGGCCATCATGTTCTCGGCCACAGTCAC
CATCCCCATCTGGCAGTGGTTCCTAACCCGGTTTGGCAAGAAGATGGCTGTGTACATTGGGATCTCATCA
GCAGTGCCATTTCTCATCTTGGTGGCCCTCATGGAGAGTAACCTGATTGTCACATATGTGGTAGCTGTGG
CAGCTGGCGTCAGTGTAGCAGCTGCCTTCTTACTACCCTGGTCCATGCTGCCTGACGTCATTGATGACTT
CCACTTGAAGCAGCCCCATATCCATGGGACCGAGCCCATCTTCTTCTCCTTCTATGTCTTCTTCACCAAG
TTTGCCTCCGGAGTCTCCCTGGGCATCTCCACCCTCAGTCTGGACTTTACTGGGTACCAGACCCGTGGCT
GCTCCCAGCCAGCACGTGTCAAGTTCACACTGAAGATGCTGGTGACCATGGCTCCCATAGTCCTCATCCT
CATAGGCCTGCTGCTCTTTAAGCTGTACCCCATTGACGAGGAGAAGCGGCGGCAGAACAAGAAGGCCCTG
CAGGGCCTGAGGGAAGAGGCCAGCAGCTCGGGGTGCTCTGACACAGACTCTACAGAACTAGCCAGAATCC
TC
>Manis javanica XM_017666029.1
ATGGCCAAAGGAGAGGGCGCCGAGAGCGGCTCCGCGGCGGGGCTGC
TGCCCACCGGCATCCTCCAAGCCGGTGAACGCCCGTCCCAGGTGAAGAAGGAACTGAAGAAGAAACAACA
GCTGTCCATTTGCAACAAGCTTTGCTATGCAGTTGGTGGGGCCCCTTACCAGGTGACTGGCTGTGCCCTG
GGGTTCTTCCTGCAGATCTACTTGTTGGATGTGGCTCAGGTGGACCCTTTCTCTGCCTCCATCATCCTTT
TTGTGGGCCGAGCTTGGGATGCCTTCACAGACCCTCTGGTGGGCTTCTGCATTAGTAAATCCTCCTGGAC
CCGCCTGGGCTGCCTCATGCCCTGGATCATCTTCTCCACGCCTCTGGCCGTCATAGCCTACTTCCTCATC
TGGTTTGTGCCTGACTTCCCACAGGGCCAGGCCCTGTGGTACCTGCTGTTCTATTGCCTTTTTGAGACAC
TGGTCACGTGTTTCCACGTTCCCTACTCAGCTCTCACCATGTTCATCAGCACAGAGCAGAGTGAGCGGGA
TTCTGCCACTGCATATCGGATGACTGTGGAAGTGTTGGGCACAGTGCTGGGCACAGCGATCCAGGGGCAA

ATTGTGGGACAAGCCGATACACCTTGTCTCCAGGACCCCAACGGTTCTGCAGTGGCCTTGGAAGTTGCCA
ATCACACACATGGCACCACCTCACTTAGAGAAACGCAAAATGCATACCTGCTGGCAGCAGGGGTCATCGC
CTCCATCTATGTCATCTGTGCTGTCATCCTAACCCTGGGCGTGCGGGAGCAGAGAGAACCCTATGAAACT
CAGCAGGCTGAGCCAATGCCCTTCTTTCGGGGCCTCCGGCTGGTCATGAGCCATGGCCCATACATCAAGC
TTATTGCAGGCTTCCTCTTCACCTCCCTGGCTTTCATGCTGGTGGAGGGAAACTTCGCCTTGTTTTGCAC
CTATACCTTGGGCTTCCGAAATGAATTCCAGAACCTGCTTCTGGCCATCATGCTCTCGGCCACATTCACC
ATTCCCATCTGGCAGTGGTTCCTAACCCGGTTTGGCAAGAAGACGGCTGTATACTTTGGGATCTCATCTG
CAGTACCATTTCTCATCTTGGTGGCCCTCAAGAAGAGTAACCTGATCGTCACATATGTGGTAGCTGTGGC
GGCTGGCATCAGTGTAGCTGCTGCCTTCTTACTACCCTGGTCCATGCTGCCCGACGTCATTGATGACTTC
CATTTGAAGCAGCCCCGCTCTCGTGGAACTGAGCCCATCTTTTTCTCCTTCTATGTCTTCTTCACCAAGT
TCGCCTCTGGAGTCTCACTGGGCATCTCCACCCTCAGTCTTGACTTTGCCGGGTACCAGACCCGTGGCTG
CTCCCAGCCGGCACGTGTTAAGTTCACCCTGAAGATGCTGGTGACCATGGTTCCCATAGTCCTCATCCTG
CTAGGCCTGCTGCTCTTCAAGCTGTACCCGATTGATGAGGAGAAGCGGCGGCAAAACAAGAAAGCCCTAC
AGGCTCTGAGGGAGGAGGCCAGCAGCTCGGGCTGCTCTGACACAGACTCTACAGAGCTGGCCAGCATCCT
C

CIT

>human ENST00000392521.6
ATGTTGAAGTTCAAATATGGAGCGCGGAATCCTTTGGATGCTGGTGCTGCTGAACCCATT
GCCAGCCGGGCCTCCAGGCTGAATCTGTTCTTCCAGGGGAAACCACCCTTTATGACTCAA
CAGCAGATGTCTCCTCTTTCCCGAGAAGGGATATTAGATGCCCTCTTTGTTCTCTTTGAA
GAATGCAGTCAGCCTGCTCTGATGAAGATTAAGCACGTGAGCAACTTTGTCCGGAAGTAT
TCCGACACCATAGCTGAGTTACAGGAGCTCCAGCCTTCGGCAAAGGACTTCGAAGTCAGA
AGTCTTGTAGGTTGTGGTCACTTTGCTGAAGTGCAGGTGGTAAGAGAGAAAGCAACCGGG
GACATCTATGCTATGAAAGTGATGAAGAAGAAGGCTTTATTGGCCCAGGAGCAGGTTTCA
TTTTTTGAGGAAGAGCGGAACATATTATCTCGAAGCACAAGCCCGTGGATCCCCCAATTA
CAGTATGCCTTTCAGGACAAAAATCACCTTTATCTGGTCATGGAATATCAGCCTGGAGGG
GACTTGCTGTCACTTTTGAATAGATATGAGGACCAGTTAGATGAAAACCTGATACAGTTT
TACCTAGCTGAGCTGATTTTGGCTGTTCACAGCGTTCATCTGATGGGATACGTGCATCGA
GACATCAAGCCTGAGAACATTCTCGTTGACCGCACAGGACACATCAAGCTGGTGGATTTT
GGATCTGCCGCGAAAATGAATTCAAACAAGATGGTGAATGCCAAACTCCCGATTGGGACC
CCAGATTACATGGCTCCTGAAGTGCTGACTGTGATGAACGGGGATGGAAAAGGCACCTAC
GGCCTGGACTGTGACTGGTGGTCAGTGGGCGTGATTGCCTATGAGATGATTTATGGGAGA
TCCCCCTTCGCAGAGGGAACCTCTGCCAGAACCTTCAATAACATTATGAATTTCCAGCGG
TTTTTGAAATTTCCAGATGACCCCAAAGTGAGCAGTGACTTTCTTGATCTGATTCAAAGC
TTGTTGTGCGGCCAGAAAGAGAGACTGAAGTTTGAAGGTCTTTGCTGCCATCCTTTCTTC
TCTAAAATTGACTGGAACAACATTCGTAACTCTCCTCCCCCCTTCGTTCCCACCCTCAAG
TCTGACGATGACACCTCCAATTTTGATGAACCAGAGAAGAATTCGTGGGTTTCATCCTCT
CCGTGCCAGCTGAGCCCCTCAGGCTTCTCGGGTGAAGAACTGCCGTTTGTGGGGTTTTCG
TACAGCAAGGCACTGGGGATTCTTGGTAGATCTGAGTCTGTTGTGTCGGGTCTGGACTCC
CCTGCCAAGACTAGCTCCATGGAAAAGAAACTTCTCATCAAAAGCAAAGAGCTACAAGAC
TCTCAGGACAAGTGTCACAAGATGGAGCAGGAAATGACCCGGTTACATCGGAGAGTGTCA
GAGGTGGAGGCTGTGCTTAGTCAGAAGGAGGTGGAGCTGAAGGCCTCTGAGACTCAGAGA
TCCCTCCTGGAGCAGGACCTTGCTACCTACATCACAGAATGCAGTAGCTTAAAGCGAAGT
TTGGAGCAAGCACGGATGGAGGTGTCCCAGGAGGATGACAAAGCACTGCAGCTTCTCCAT
GATATCAGAGAGCAGAGCCGGAAGCTCCAAGAAATCAAAGAGCAGGAGTACCAGGCTCAA
GTGGAAGAAATGAGGTTGATGATGAATCAGTTGGAAGAGGATCTTGTCTCAGCAAGAAGA

CGGAGTGATCTCTACGAATCTGAGCTGAGAGAGTCTCGGCTTGCTGCTGAAGAATTCAAG
CGGAAAGCGACAGAATGTCAGCATAAACTGTTGAAGGCTAAGGATCAAGGGAAGCCTGAA
GTGGGAGAATATGCGAAACTGGAGAAGATCAATGCTGAGCAGCAGCTCAAAATTCAGGAG
CTCCAAGAGAAACTGGAGAAGGCTGTAAAAGCCAGCACGGAGGCCACCGAGCTGCTGCAG
AATATCCGCCAGGCAAAGGAGCGAGCCGAGAGGGAGCTGGAGAAGCTGCAGAACCGAGAG
GATTCTTCTGAAGGCATCAGAAAGAAGCTGGTGGAAGCTGAGGAACGCCGCCATTCTCTG
GAGAACAAGGTAAAGAGACTAGAGACCATGGAGCGTAGAGAAAACAGACTGAAGGATGAC
ATCCAGACAAAATCCCAACAGATCCAGCAGATGGCTGATAAAATTCTGGAGCTCGAAGAG
AAACATCGGGAGGCCCAAGTCTCAGCCCAGCACCTAGAAGTGCACCTGAAACAGAAAGAG
CAGCACTATGAGGAAAAGATTAAAGTGTTGGACAATCAGATAAAGAAAGACCTGGCTGAC
AAGGAGACACTGGAGAACATGATGCAGAGACACGAGGAGGAGGCCCATGAGAAGGGCAAA
ATTCTCAGCGAACAGAAGGCGATGATCAATGCTATGGATTCCAAGATCAGATCCCTGGAA
CAGAGGATTGTGGAACTGTCTGAAGCCAATAAACTTGCAGCAAATAGCAGTCTTTTTACC
CAAAGGAACATGAAGGCCCAAGAAGAGATGATTTCTGAACTCAGGCAACAGAAATTTTAC
CTGGAGACACAGGCTGGGAAGTTGGAGGCCCAGAACCGAAAACTGGAGGAGCAGCTGGAG
AAGATCAGCCACCAAGACCACAGTGACAAGAATCGGCTGCTGGAACTGGAGACAAGATTG
CGGGAGGTCAGTCTAGAGCACGAGGAGCAGAAACTGGAGCTCAAGCGCCAGCTCACAGAG
CTACAGCTCTCCCTGCAGGAGCGCGAGTCACAGTTGACAGCCCTGCAGGCTGCACGGGCG
GCCCTGGAGAGCCAGCTTCGCCAGGCGAAGACAGAGCTGGAAGAGACCACAGCAGAAGCT
GAAGAGGAGATCCAGGCACTCACGGCACATAGAGATGAAATCCAGCGCAAATTTGATGCT
CTTCGTAACAGCTGTACTGTAATCACAGACCTGGAGGAGCAGCTAAACCAGCTGACCGAG
GACAACGCTGAACTCAACAACCAAAACTTCTACTTGTCCAAACAACTCGATGAGGCTTCT
GGCGCCAACGACGAGATTGTACAACTGCGAAGTGAAGTGGACCATCTCCGCCGGGAGATC
ACGGAACGAGAGATGCAGCTTACCAGCCAGAAGCAAACGATGGAGGCTCTGAAGACCACG
TGCACCATGCTGGAGGAACAGGTCATGGATTTGGAGGCCCTAAACGATGAGCTGCTAGAA
AAAGAGCGGCAGTGGGAGGCCTGGAGGAGCGTCCTGGGTGATGAGAAATCCCAGTTTGAG
TGTCGGGTTCGAGAGCTGCAGAGAATGCTGGACACCGAGAAACAGAGCAGGGCGAGAGCC
GATCAGCGGATCACCGAGTCTCGCCAGGTGGTGGAGCTGGCAGTGAAGGAGCACAAGGCT
GAGATTCTCGCTCTGCAGCAGGCTCTCAAAGAGCAGAAGCTGAAGGCCGAGAGCCTCTCT
GACAAGCTCAATGACCTGGAGAAGAAGCATGCTATGCTTGAAATGAATGCCCGAAGCTTA
CAGCAGAAGCTGGAGACTGAACGAGAGCTCAAACAGAGGCTTCTGGAAGAGCAAGCCAAA
TTACAGCAGCAGATGGACCTGCAGAAAAATCACATTTTCCGTCTGACTCAAGGACTGCAA
GAAGCTCTAGATCGGGCTGATCTACTGAAGACAGAAAGAAGTGACTTGGAGTATCAGCTG
GAAAACATTCAGGTTCTCTATTCTCATGAAAAGGTGAAAATGGAAGGCACTATTTCTCAA
CAAACCAAACTCATTGATTTTCTGCAAGCCAAAATGGACCAACCTGCTAAAAAGAAAAAG
GGTTTATTTAGTCGACGGAAAGAGGACCCTGCTTTACCCACACAGGTTCCTCTGCAGTAC
AATGAGCTGAAGCTGGCCCTGGAGAAGGAGAAAGCTCGCTGTGCAGAGCTAGAGGAAGCC
CTTCAGAAGACCCGCATCGAGCTCCGGTCCGCCCGGGAGGAAGCTGCCCACCGCAAAGCA
ACGGACCACCCACACCCATCCACGCCAGCCACCGCGAGGCAGCAGATCGCCATGTCCGCC
ATCGTGCGGTCGCCAGAGCACCAGCCCAGTGCCATGAGCCTGCTGGCCCCGCCATCCAGC
CGCAGAAAGGAGTCTTCAACTCCAGAGGAATTTAGTCGGCGTCTTAAGGAACGCATGCAC
CACAATATTCCTCACCGATTCAACGTAGGACTGAACATGCGAGCCACAAAGTGTGCTGTG
TGTCTGGATACCGTGCACTTTGGACGCCAGGCATCCAAATGTCTCGAATGTCAGGTGATG
TGTCACCCCAAGTGCTCCACGTGCTTGCCAGCCACCTGCGGCTTGCCTGCTGAATATGCC
ACACACTTCACCGAGGCCTTCTGCCGTGACAAAATGAACTCCCCAGGTCTCCAGACCAAG
GAGCCCAGCAGCAGCTTGCACCTGGAAGGGTGGATGAAGGTGCCCAGGAATAACAAACGA
GGACAGCAAGGCTGGGACAGGAAGTACATTGTCCTGGAGGGATCAAAAGTCCTCATTTAT

GACAATGAAGCCAGAGAAGCTGGACAGAGGCCGGTGGAAGAATTTGAGCTGTGCCTTCCC
GACGGGGATGTATCTATTCATGGTGCCGTTGGTGCTTCCGAACTCGCAAATACAGCCAAA
GCAGATGTCCCATACATACTGAAGATGGAATCTCACCCGCACACCACCTGCTGGCCCGGG
AGAACCCTCTACTTGCTAGCTCCCAGCTTCCCTGACAAACAGCGCTGGGTCACCGCCTTA
GAATCAGTTGTCGCAGGTGGGAGAGTTTCTAGGGAAAAAGCAGAAGCTGATGCTAAACTG
CTTGGAAACTCCCTGCTGAAACTGGAAGGTGATGACCGTCTAGACATGAACTGCACGCTG
CCCTTCAGTGACCAGGTGGTGTTGGTGGGCACCGAGGAAGGGCTCTACGCCCTGAATGTC
TTGAAAAACTCCCTAACCCATGTCCCAGGAATTGGAGCAGTCTTCCAAATTTATATTATC
AAGGACCTGGAGAAGCTACTCATGATAGCAGGAGAAGAGCGGGCACTGTGTCTTGTGGAC
GTGAAGAAAGTGAAACAGTCCCTGGCCCAGTCCCACCTGCCTGCCCAGCCCGACATCTCA
CCCAACATTTTTGAAGCTGTCAAGGGCTGCCACTTGTTTGGGGCAGGCAAGATTGAGAAC
GGGCTCTGCATCTGTGCAGCCATGCCCAGCAAAGTCGTCATTCTCCGCTACAACGAAAAC
CTCAGCAAATACTGCATCCGGAAAGAGATAGAGACCTCAGAGCCCTGCAGCTGTATCCAC
TTCACCAATTACAGTATCCTCATTGGAACCAATAAATTCTACGAAATCGACATGAAGCAG
TACACGCTCGAGGAATTCCTGGATAAGAATGACCATTCCTTGGCACCTGCTGTGTTTGCC
GCCTCTTCCAACAGCTTCCCTGTCTCAATCGTGCAGGTGAACAGCGCAGGGCAGCGAGAG
GAGTACTTGCTGTGTTTCCACGAATTTGGAGTGTTCGTGGATTCTTACGGAAGACGTAGC
CGCACAGACGATCTCAAGTGGAGTCGCTTACCTTTGGCCTTTGCCTACAGAGAACCCTAT
CTGTTTGTGACCCACTTCAACTCACTCGAAGTAATTGAGATCCAGGCACGCTCCTCAGCA
GGGACCCCTGCCCGAGCGTACCTGGACATCCCGAACCCGCGCTACCTGGGCCCTGCCATT
TCCTCAGGAGCGATTTACTTGGCGTCCTCATACCAGGATAAATTAAGGGTCATTTGCTGC
AAGGGAAACCTCGTGAAGGAGTCCGGCACTGAACACCACCGGGGCCCGTCCACCTCCCGC
AGCAGCCCCAACAAGCGAGGCCCACCCACGTACAACGAGCACATCACCAAGCGCGTGGCC
TCCAGCCCAGCGCCGCCCGAAGGCCCCAGCCACCCGCGAGAGCCAAGCACACCCCACCGC
TACCGCGAGGGGCGGACCGAGCTGCGCAGGGACAAGTCTCCTGGCCGCCCCCTGGAGCGA
GAGAAGTCCCCCGGCCGGATGCTCAGCACGCGGAGAGAGCGGTCCCCCGGGAGGCTGTTT
GAAGACAGCAGCAGGGGCCGGCTGCCTGCGGGAGCCGTGAGGACCCCGCTGTCCCAGGTG
AACAAGGTCTGGGACCAGTCTTCAGTA
>chimpanzee NM_001329948.1
ATGTTGAAGTTCAAATATGGAGCGCGGAATCCTCTGGATGCTGGTGCTGCTGAACCCATTGCCAGCCGGG
CCTCCAGGCTGAATCTGTTCTTCCAGGGGAAACCACCCTTTATGACTCAACAGCAGATGTCTCCTCTTTC
CCGAGAAGGGATATTAGATGCCCTCTTTGTTCTCTTTGAAGAATGCAGTCAGCCTGCTCTGATGAAGATT
AAGCATGTGAGCAACTTTGTCCGGAAGTATTCCGACACCATAGCTGAGTTACAGGAGCTCCAGCCTTCGG
CAAAGGACTTCGAAGTCAGAAGTCTTGTAGGTTGTGGTCACTTTGCTGAAGTGCAGGTGGTAAGAGAGAA
AGCAACCGGGGACATCTATGCTATGAAAGTGATGAAGAAGAAGGCTTTATTGGCCCAGGAGCAGGTTTCA
TTTTTTGAGGAAGAGCGGAACATATTATCTCGAAGCACAAGCCCGTGGATCCCCCAATTACAGTATGCCT
TTCAGGACAAAAATCACCTTTATCTGGTCATGGAATATCAGCCTGGAGGGGACTTGCTGTCACTTTTGAA
TAGATATGAGGACCAGTTAGATGAAAACCTGATACAATTTTACCTAGCTGAGCTGATTTTGGCTGTTCAC
AGCGTTCATCTGATGGGATACGTGCATCGAGACATCAAGCCTGAGAACATTCTCATTGACCGCACAGGAC
ACATCAAGCTGGTGGATTTTGGATCTGCCGCGAAAATGAATTCAAACAAGATGGTGAATGCCAAACTCCC
GATTGGGACCCCAGATTACATGGCTCCTGAAGTGCTGACTGTGATGAACGGGGATGGAAAAGGCACCTAC
GGCCTGGACTGTGACTGGTGGTCAGTGGGCGTGATTGCCTATGAGATGATTTATGGGAGATCCCCCTTCG
CAGAGGGAACCTCTGCCAGATCCTTCAATAACATCATGAATTTCCAGCGGTTTTTGAAATTTCCAGATGA
CCCCAAAGTGAGCGGTGACTTTCTTGATCTGGTTCAAAGCTTGTTGTGTGGCCAGAAAGAGAGACTGAAG
TTTGAAGGTCTTTGCTGCCATCCTTTCTTCTCTAAAATCGACTGGAACAACATTCGTAACTCTCCTCCCC
CCTTCGTTCCCACCCTCAAGTCTGACGATGACACCTCCAATTTTGATGAACCAGAGAAGAATTCGTGGGT
TTCATCCTCTCCGTGCCAGCTGAGCCCCTCAGGTTTCTCGGGTGAAGAACTGCCGTTTGTGGGGTTTTCG

TACAGCAAGGCACTGGGGATTCTTGGTAGATCTGAGTCTGTTGTGTCGGGTCTGGACTCCCCTGCCAAGA
CTAGCTCCATGGAAAAGAAACTTCTCATCAAAAGCAAAGAGCTACAAGACTCTCAGGACAAGTGTCACAA
GATGGAGCAGGAAATGACCCGGTTACATCGGAGAGTGTCAGAGGTGGAGGCTGTGCTTAGTCAGAAGGAG
GTGGAGCTGAAGGCCTCTGAGACTCAGAGATCCCTCCTGGAGCAGGACCTTGCTACCTACATCACAGAAT
GCAGTAGCTTAAAGCGAAGTTTGGAGCAAGCACGGATGGAGGTGTCCCAGGAGGATGACAAAGCACTGCA
GCTTCTCCATGATATCAGAGAGCAGAGCCGGAAGCTCCAAGAAATCAAAGAGCAGGAGTACCAGGCTCAA
GTGGAAGAAATGAGGTTGATGATGAATCAGTTGGAAGAGGATCTTGTCTCAGCAAGAAGACGGAGTGATC
TCTACGAATCTGAGCTGAGAGAGTCTCGGCTTGCTGCTGAAGAATTCAAGCGGAAAGCGACAGAATGTCA
GCATAAACTGTTGAAGGCTAAGGATCAAGGGAAGCCTGAAGTGGGAGAATATGCGAAACTGGAGAAGATC
AATGCTGAGCAGCAGCTCAAAATTCAGGAGCTCCAAGAGAAACTGGAGAAGGCTGTAAAAGCCAGCACGG
AGGCCACCGAGCTGCTGCAGAATATCCGCCAGGCAAAGGAGCGAGCCGAGAGGGAGCTGGAGAAGCTGCA
GAACCGAGAGGATTCTTCTGAAGGCATCAGAAAGAAGCTGGTGGAAGCTGAGGAACGCCGCCATTCTCTG
GAGAACAAGGTAAAGAGACTAGAGACCATGGAGCGTAGAGAAAACAGACTGAAGGATGACATCCAGACAA
AATCCCAACAGATCCAGCAGATGGCTGATAAAATTCTGGAGCTCGAAGAGAAACATCGGGAGGCCCAAGT
CTCAGCCCAGCACCTAGAAGTGCACCTGAAACAGAAAGAGCAGCACTATGAGGAAAAGATTAAAGTGTTG
GACAATCAGATAAAGAAAGACCTGGCCGACAAGGAGACGCTGGAGAACATGATGCAGAGACACGAGGAGG
AGGCCCATGAGAAGGGCAAAATTCTCAGCGAACAGAAGGCGATGATCAATGCTATGGATTCCAAGATCAG
ATCCCTGGAACAGAGGATTGTGGAACTGTCTGAAGCCAATAAACTTGCAGCAAATAGCAGTCTTTTTACC
CAAAGGAACATGAAGGCCCAAGAAGAGATGATTTCTGAACTCAGGCAACAGAAATTTTACCTGGAGACAC
AGGCTGGGAAGTTGGAGGCCCAGAACCGAAAACTGGAGGAGCAGCTGGAGAAGATCAGCCACCAAGACCA
CAGTGACAAGAATCGGCTGCTGGAACTGGAGACAAGATTGCGGGAGGTCAGTCTAGAGCATGAGGAGCAG
AAACTGGAGCTCAAGCGCCAGCTCACAGAGCTACAGCTCTCCCTGCAGGAGCGCGAGTCACAGTTGACAG
CCCTGCAGGCTGCACGGGCGGCCCTGGAGAGCCAGCTTCGCCAGGCGAAGACAGAGCTGGAAGAGACCAC
AGCAGAAGCCGAAGAGGAGATCCAGGCGCTCACGGCACATAGAGATGAAATCCAGCGCAAATTTGATGCT
CTTCGTAACAGCTGTACTGTAATCACAGACCTGGAGGAGCAGCTAAACCAGCTGACCGAGGACAACGCTG
AACTCAACAACCAAAACTTCTACTTGTCCAAACAACTCGATGAGGCTTCTGGCGCCAACGACGAGATTGT
ACAACTGCGAAGTGAAGTGGACCATCTCCGCCGGGAGATCACGGAACGAGAGATGCAGCTTACCAGCCAG
AAGCAAACGATGGAGGCTCTGAAGACCACGTGCACCATGCTGGAGGAACAGGTCATGGATTTGGAGGCCC
TAAACGATGAGCTGCTAGAAAAAGAGCGGCAGTGGGAGGCCTGGAGGAGCGTCCTGGGTGATGAGAAATC
CCAGTTTGAGTGTCGGGTTCGAGAGCTGCAGAGGATGCTGGACACCGAGAAACAGAGCAGGGCGAGAGCC
GATCAGCGGATCACCGAGTCTCGCCAGGTGGTGGAGCTGGCAGTGAAGGAGCACAAGGCTGAGATTCTCG
CTCTGCAGCAGGCTCTCAAAGAGCAGAAGCTGAAGGCCGAGAGCCTCTCTGACAAGCTCAATGACCTGGA
GAAGAAGCATGCTATGCTTGAAATGAATGCCCGAAGCTTACAGCAGAAGCTGGAGACTGAACGAGAGCTC
AAACAGAGGCTTCTGGAAGAGCAAGCCAAATTACAGCAGCAGATGGACCTGCAGAAAAATCACATTTTCC
GTCTGACTCAAGGACTGCAAGAAGCTCTAGATCGGGCTGATCTACTGAAGACAGAAAGAAGTGACTTGGA
GTATCAGCTGGAAAACATTCAGGTTCTCTATTCTCATGAAAAGGTGAAAATGGAAGGGACTATTTCTCAA
CAAACCAAACTCATTGATTTTCTGCAAGCCAAAATGGACCAACCTGCTAAAAAGAAAAAGGGTTTATTTA
GTCGACGGAAAGAGGACCCTGCTTTACCCACACAGGTTCCTCTGCAGTACAATGAGCTGAAGCTGGCCCT
GGAGAAGGAGAAAGCTCGCTGTGCAGAGCTAGAGGAAGCCCTTCAGAAGACCCGCATCGAGCTCCGGTCC
GCCCGGGAGGAAGCTGCCCACCGCAAAGCAACGGACCACCCACACCCATCCACGCCAGCCACCGCGAGGC
AGCAGATCGCCATGTCCGCCATCGTGCGGTCGCCAGAGCACCAGCCCAGTGCCATGAGCCTGCTGGCCCC
GCCATCCAGCCGCAGAAAGGAGTCTTCAACTCCAGAGGAATTTAGTCGGCGTCTTAAGGAACGCATGCAC
CACAATATTCCTCACCGATTCAACGTAGGACTGAACATGCGAGCCACAAAGTGTGCTGTGTGTCTGGATA
CCGTGCACTTTGGACGCCAGGCATCCAAATGTCTCGAATGTCAGGTGATGTGTCACCCCAAGTGCTCCAC
GTGCTTGCCAGCCACCTGCGGCTTGCCTGCTGAATATGCCACACACTTCACCGAGGCCTTCTGCCGTGAC
AAAATGAACTCCCCAGGTCTCCAGACCAAGGAGCCCAGCAGCAGCTTGCACCTGGAAGGGTGGATGAAGG
TGCCCAGGAATAACAAACGAGGACAGCAAGGCTGGGACAGGAAGTACATTGTCCTGGAGGGATCAAAAGT

CCTCATTTATGACAATGAAGCCAGAGAAGCTGGACAGAGGCCGGTGGAAGAATTTGAGCTGTGCCTTCCC
GACGGGGATGTATCTATTCATGGTGCCGTTGGTGCTTCCGAACTCGCAAATACAGCCAAAGCAGATGTCC
CATACATACTGAAGATGGAATCTCACCCGCACACCACCTGCTGGCCCGGGAGAACCCTCTACTTGCTAGC
TCCCAGCTTCCCTGACAAACAGCGCTGGGTCACCGCCTTAGAATCAGTTGTCGCAGGTGGGAGAGTTTCT
AGGGAAAAAGCAGAAGCTGATGCTAAACTGCTTGGAAACTCCCTGCTGAAACTGGAAGGTGATGACCGTC
TAGACATGAACTGCACACTGCCCTTCAGTGACCAGGTGGTGTTGGTGGGCACCGAGGAAGGGCTCTACGC
CCTGAATGTCTTGAAAAACTCCCTAACCCATGTCCCAGGAATTGGAGCAGTCTTCCAAATTTATATTATC
AAGGACCTGGAGAAGCTACTCATGATAGCAGGAGAAGAGCGGGCACTGTGTCTTGTGGACGTGAAGAAAG
TGAAACAGTCCCTGGCCCAGTCCCACCTGCCTGCCCAGCCCGACATCTCACCCAACATTTTTGAAGCTGT
CAAGGGCTGCCACTTGTTTGGGGCAGGCAAGATTGAGAACGGGCTCTGCATCTGTGCAGCCATGCCCAGC
AAAGTCGTCATTCTCCGCTACAACGAAAACCTCAGCAAATACTGCATCCGGAAAGAGATAGAGACCTCAG
AGCCCTGCAGCTGTATCCACTTCACCAATTACAGTATCCTCATTGGAACCAATAAATTCTACGAAATTGA
CATGAAGCAGTACACGCTCGAGGAATTCCTGGATAAGAATGACCATTCCTTGGCACCTGCTGTGTTTGCC
GCCTCTTCCAACAGCTTCCCTGTCTCAATCGTGCAGGTGAACAGCGCAGGGCAGCGAGAGGAGTACTTGC
TGTGTTTCCACGAATTTGGAGTGTTCGTGGATTCTTACGGAAGACGTAGCCGCACAGACGATCTCAAGTG
GAGTCGCTTACCTTTGGCCTTTGCCTACAGAGAACCCTATCTGTTTGTGACCCACTTCAACTCACTCGAA
GTAATTGAGATCCAGGCACGCTCCTCAGCAGGGACCCCTGCCCGAGCGTACCTGGACATCCCGAACCCGC
GCTACCTGGGCCCTGCCATTTCCTCAGGAGCGATTTACTTGGCGTCCTCATACCAGGATAAATTAAGGGT
CATTTGCTGCAAGGGAAACCTCGTGAAAGAGTCCGGCACTGAACACCACCGGGGCCCGTCCACCTCCCGC
AGCAGCCCCAACAAGCGAGGCCCGCCCACGTACAACGAGCACATCACCAAGCGCGTGGCCTCCAGCCCAG
CGCCGCCCGAAGGCCCCAGCCACCCGCGAGAGCCAAGCACACCCCACCGCTACCGCGAGGGGCGGACCGA
GCTGCGCAGGGACAAGTCTCCTGGCCGCCCCCTGGAGCGAGAGAAGTCCCCGGGCCGGATGCTCAGCACG
CGGAGAGAGCGGTCCCCCGGGAGGCTGTTTGAAGACAGCAGCAGGGGCCGGCTGCCTGCGGGAGCCGTGA
GGACCCCGCTGTCCCAGGTGAACAAGGTCTGGGACCAGTCTTCAGTA
>pan paniscus XM_003832401.2
ATGTTGAAGTTCAAATATGGAGCGCGGAATCCTCTGGATGCTGGTGCTGCTGAACCCATTGCCAGCCGGG
CCTCCAGGCTGAATCTGTTCTTCCAGGGGAAACCACCCTTTATGACTCAACAGCAGATGTCTCCTCTTTC
CCGAGAAGGGATATTAGATGCCCTCTTTGTTCTCTTTGAAGAATGCAGTCAGCCTGCTCTGATGAAGATT
AAGCATGTGAGCAACTTTGTCCGGAAGTATTCCGACACCATAGCTGAGTTACAGGAGCTCCAGCCTTCGG
CAAAGGACTTCGAAGTCAGAAGTCTTGTAGGTTGTGGTCACTTTGCTGAAGTGCAGGTGGTAAGAGAGAA
AGCAACCGGGGACATCTATGCTATGAAAGTGATGAAGAAGAAGGCTTTATTGGCCCAGGAGCAGGTTTCA
TTTTTTGAGGAAGAGCGGAACATATTATCTCGAAGCACAAGCCCGTGGATCCCCCAATTACAGTATGCCT
TTCAGGACAAAAATCACCTTTATCTGGTCATGGAATATCAGCCTGGAGGGGACTTGCTGTCACTTTTGAA
TAGATATGAGGACCAGTTAGATGAAAACCTGATACAATTTTACCTAGCTGAGCTGATTTTGGCTGTTCAC
AGCGTTCATCTGATGGGATACGTGCATCGAGACATCAAGCCTGAGAACATTCTCATTGACCGCACAGGAC
ACATCAAGCTGGTGGATTTTGGATCTGCCGCGAAAATGAATTCAAACAAGATGGTGAATGCCAAACTCCC
GATTGGGACCCCAGATTACATGGCTCCTGAAGTGCTGACTGTGATGAACGGGGATGGAAAAGGCACCTAC
GGCCTGGACTGTGACTGGTGGTCAGTGGGCGTGATTGCCTATGAGATGATTTATGGGAGATCCCCCTTCG
CAGAGGGAACCTCTGCCAGAACCTTCAATAACATCATGAATTTCCAGCGGTTTTTGAAATTTCCAGATGA
CCCCAAAGTGAGCGGTGACTTTCTTGATCTGATTCAAAGCTTGTTGTGTGGCCAGAAAGAGAGACTGAAG
TTTGAAGGTCTTTGCTGCCATCCTTTCTTCTCTAAAATCGACTGGAACAACATTCGTAACTCTCCTCCCC
CCTTCGTTCCCACCCTCAAGTCTGACGATGACACCTCCAATTTTGATGAACCAGAGAAGAATTCGTGGGT
TTCATCCTCTCCGTGCCAGCTGAGCCCCTCAGGTTTCTCGGGTGAAGAACTGCCGTTTGTGGGGTTTTCG
TACAGCAAGGCACTGGGGATTCTTGGTAGATCTGAGTCTGTTGTGTCGGGTCTGGACTCCCCTGCCAAGA
CTAGCTCCATGGAAAAGAAACTTCTCATCAAAAGCAAAGAGCTACAAGACTCTCAGGACAAGTGTCACAA
GATGGAGCAGGAAATGACCCGGTTACATCGGAGAGTGTCAGAGGTGGAGGCTGTGCTTAGTCAGAAGGAG
GTGGAGCTGAAGGCCTCTGAGACTCAGAGATCCCTCCTGGAGCAGGACCTTGCTACCTACATCACAGAAT

GCAGTAGCTTAAAGCGAAGTTTGGAGCAAGCACGGATGGAGGTGTCCCAGGAGGATGACAAAGCACTGCA
GCTTCTCCATGATATCAGAGAGCAGAGCCGGAAGCTCCAAGAAATCAAAGAGCAGGAGTACCAGGCTCAA
GTGGAAGAAATGAGGTTGATGATGAATCAGTTGGAAGAGGATCTTGTCTCAGCAAGAAGACGGAGTGATC
TCTACGAATCTGAGCTGAGAGAGTCTCGGCTTGCTGCTGAAGAATTCAAGCGGAAAGCGACAGAATGTCA
GCATAAACTGTTGAAGGCTAAGGATCAAGGGAAGCCTGAAGTGGGAGAATATGCGAAACTGGAGAAGATC
AATGCTGAGCAGCAGCTCAAAATTCAGGAGCTCCAAGAGAAACTGGAGAAGGCTGTAAAAGCCAGCACGG
AGGCCACCGAGCTGCTGCAGAATATCCGCCAGGCAAAGGAGCGAGCCGAGAGGGAGCTGGAGAAGCTGCA
GAACCGAGAGGATTCTTCTGAAGGCATCAGAAAGAAGCTGGTGGAAGCTGAGGAACGCCGCCATTCTCTG
GAGAACAAGGTAAAGAGACTAGAGACCATGGAGCGTAGAGAAAACAGACTGAAGGATGACATCCAGACAA
AATCCCAACAGATCCAGCAGATGGCTGATAAAATTCTGGAGCTCGAAGAGAAACATCGGGAGGCCCAAGT
CTCAGCCCAGCACCTAGAAGTGCACCTGAAACAGAAAGAGCAGCACTATGAGGAAAAGATTAAAGTGTTG
GACAATCAGATAAAGAAAGACCTGGCCGACAAGGAGACGCTGGAGAACATGATGCAGAGACACGAGGAGG
AGGCCCATGAGAAGGGCAAAATTCTCAGCGAACAGAAGGCGATGATCAATGCTATGGATTCCAAGATCAG
ATCCCTGGAACAGAGGATTGTGGAACTGTCTGAAGCCAATAAACTTGCAGCAAATAGCAGTCTTTTTACC
CAAAGGAACATGAAGGCCCAAGAAGAGATGATTTCTGAACTCAGGCAACAGAAATTTTACCTGGAGACAC
AGGCTGGGAAGTTGGAGGCCCAGAACCGAAAACTGGAGGAGCAGCTGGAGAAGATCAGCCACCAAGACCA
CAGTGACAAGAATCGGCTGCTGGAACTGGAGACAAGATTGCGGGAGGTCAGTCTAGAGCATGAGGAGCAG
AAACTGGAGCTCAAGCGCCAGCTCACAGAGCTACAGCTCTCCCTGCAGGAGCGCGAGTCACAGTTGACAG
CCCTGCAGGCTGCACGGGCGGCCCTGGAGAGCCAGCTTCGCCAGGCGAAGACAGAGCTGGAAGAGACCAC
AGCAGAAGCCGAAGAGGAGATCCAGGCGCTCACGGCACATAGAGATGAAATCCAGCGCAAATTTGATGCT
CTTCGTAACAGCTGTACTGTAATCACAGACCTGGAGGAGCAGCTAAACCAGCTGACCGAGGACAACGCTG
AACTCAACAACCAAAACTTCTACTTGTCCAAACAACTCGATGAGGCTTCTGGCGCCAACGACGAGATTGT
ACAACTGCGAAGTGAAGTGGACCATCTCCGCCGGGAGATCACGGAACGAGAGATGCAGCTTACCAGCCAG
AAGCAAACGATGGAGGCTCTGAAGACCACGTGCACCATGCTGGAGGAACAGGTCATGGATTTGGAGGCCC
TAAACGATGAGCTGCTAGAAAAAGAGCGGCAGTGGGAGGCCTGGAGGAGCGTCCTGGGTGATGAGAAATC
CCAGTTTGAGTGTCGGGTTCGAGAGCTGCAGAGGATGCTGGACACCGAGAAACAGAGCAGGGCGAGAGCC
GATCAGCGGATCACCGAGTCTCGCCAGGTGGTGGAGCTGGCAGTGAAGGAGCACAAGGCTGAGATTCTCG
CTCTGCAGCAGGCTCTCAAAGAGCAGAAGCTGAAGGCCGAGAGCCTCTCTGACAAGCTCAATGACCTGGA
GAAGAAGCATGCTATGCTTGAAATGAATGCCCGAAGCTTACAGCAGAAGCTGGAGACTGAACGAGAGCTC
AAACAGAGGCTTCTGGAAGAGCAAGCCAAATTACAGCAGCAGATGGACCTGCAGAAAAATCACATTTTCC
GTCTGACTCAAGGACTGCAAGAAGCTCTAGATCGGGCTGATCTACTGAAGACAGAAAGAAGTGACTTGGA
GTATCAGCTGGAAAACATTCAGGTTCTCTATTCTCATGAAAAGGTGAAAATGGAGGGGACTATTTCTCAA
CAAACCAAACTCATTGATTTTCTGCAAGCCAAAATGGACCAACCTGCTAAAAAGAAAAAGGGTTTATTTA
GTCGACGGAAAGAGGACCCTGCTTTACCCACACAGGTTCCTCTGCAGTACAATGAGCTGAAGCTGGCCCT
GGAGAAGGAGAAAGCTCGCTGTGCAGAGCTAGAGGAAGCCCTTCAGAAGACCCGCATCGAGCTCCGGTCC
GCCCGGGAGGAAGCTGCCCACCGCAAAGCAACGGACCACCCACACCCATCCACGCCAGCCACCGCGAGGC
AGCAGATCGCCATGTCCGCCATCGTGCGGTCGCCAGAGCACCAGCCCAGTGCCATGAGCCTGCTGGCCCC
GCCATCCAGCCGCAGAAAGGAGTCTTCAACTCCAGAGGAATTTAGTCGGCGTCTTAAGGAACGCATGCAC
CACAATATTCCTCACCGATTCAACGTAGGACTGAACATGCGAGCCACAAAGTGTGCTGTGTGTCTGGATA
CCGTGCACTTTGGACGCCAGGCATCCAAATGTCTCGAATGTCAGGTGATGTGTCACCCCAAGTGCTCCAC
GTGCTTGCCAGCCACCTGCGGCTTGCCTGCTGAATATGCCACACACTTCACCGAGGCCTTCTGCCGTGAC
AAAATGAACTCCCCAGGTCTCCAGACCAAGGAGCCCAGCAGCAGCTTGCACCTGGAAGGGTGGATGAAGG
TGCCCAGGAATAACAAACGAGGACAGCAAGGCTGGGACAGGAAGTACATTGTCCTGGAGGGATCAAAAGT
CCTCATTTATGACAATGAAGCCAGAGAAGCTGGACAGAGGCCGGTGGAAGAATTTGAGCTGTGCCTTCCC
GACGGGGATGTATCTATTCATGGTGCCGTTGGTGCTTCCGAACTCGCAAATACAGCCAAAGCAGATGTCC
CATACATACTGAAGATGGAATCTCACCCGCACACCACCTGCTGGCCCGGGAGAACCCTCTACTTGCTAGC
TCCCAGCTTCCCTGACAAACAGCGCTGGGTCACCGCCTTAGAATCAGTTGTCGCAGGTGGGAGAGTTTCT

AGGGAAAAAGCAGAAGCTGATGCTAAACTGCTTGGAAACTCCCTGCTGAAACTGGAAGGTGATGACCGTC
TAGACATGAACTGCACACTGCCCTTCAGTGACCAGGTGGTGTTGGTGGGCACCGAGGAAGGGCTCTACGC
CCTGAATGTCTTGAAAAACTCCCTAACCCATGTCCCAGGAATTGGAGCAGTCTTCCAAATTTATATTATC
AAGGACCTGGAGAAGCTACTCATGATAGCAGGAGAAGAGCGGGCACTGTGTCTTGTGGACGTGAAGAAAG
TGAAACAGTCCCTGGCCCAGTCCCACCTGCCTGCCCAGCCCGACATCTCACCCAACATTTTTGAAGCTGT
CAAGGGCTGCCACTTGTTTGGGGCAGGCAAGATTGAGAACGGGCTCTGCATCTGTGCAGCCATGCCCAGC
AAAGTCGTCATTCTCCGTTACAACGAAAACCTCAGCAAATACTGCATCCGGAAAGAGATAGAGACCTCAG
AGCCCTGCAGCTGTATCCACTTCACCAATTACAGTATCCTCATTGGAACCAATAAATTCTACGAAATCGA
CATGAAGCAGTACACGCTCGAGGAATTCCTGGATAAGAATGACCATTCCTTGGCACCTGCTGTGTTTGCC
GCCTCTTCCAACAGCTTCCCTGTCTCAATCGTGCAGGTGAACAGCGCAGGGCAGCGAGAGGAGTACTTGC
TGTGTTTCCACGAATTTGGAGTGTTCGTGGATTCTTACGGAAGACGTAGCCGCACAGACGATCTCAAGTG
GAGTCGCTTACCTTTGGCCTTTGCCTACAGAGAACCCTATCTGTTTGTGACCCACTTCAACTCACTCGAA
GTAATTGAGATCCAGGCACGCTCCTCAGCAGGGACCCCTGCCCGAGCGTACCTGGACATCCCGAACCCAC
GCTACCTGGGCCCTGCCATTTCCTCCGGAGCGATTTACTTGGCGTCCTCATACCAGGATAAATTAAGGGT
CATTTGCTGCAAGGGAAACCTCGTGAAGGAGTCCGGCACTGAACACCACCGGGGCCCGTCCACCTCCCGC
AGCAGCCCCAACAAGCGAGGCCCGCCCACGTACAACGAGCACATCACCAAGCGCGTGGCCTCCAGCCCAG
CGCCGCCCGAAGGCCCCAGCCACCCGCGAGAGCCAAGCACACCCCACCGCTACCGCGAGGGGCGGACCGA
GCTGCGCAGGGACAAGTCTCCTGGCCGCCCCCTGGAGCGAGAGAAGTCCCCGGGCCGGATGCTCAGCACG
CGGAGAGAGCGGTCCCCCGGGAGGCTGTTTGAAGACAGCAGCAGGGGCCGGCTGCCTGCGGGAGCCGTGA
GGACCCCGCTGTCCCAGGTGAACAAGGTCTGGGACCAGTCTTCAGTA
>macaque ENSMMUT00000027913.3
ATGTTGAAGTTCAAATATGGAGCGCGGAATCCTTTGGATGCTGGTGCTGCTGAACCCATT
GCCAGCCGGGCCTCCAGGCTGAATCTATTCTTCCAGGGGAAACCACCCTTTATGACTCAA
CAGCAGATGTCTCCTCTTTCCCGAGAAGGGATATTAGATGCCCTCTTTGTTCTCTTTGAA
GAATGCAGTCAGCCTGCTCTGATGAAGATTAAGCATGTGAGCAACTTTGTCCGGAAGTAT
TCCGACACCATAGCTGAGTTACAGGAGCTCCAGCCTTCGACAAAGGACTTTGAAGTCAGA
AGTCTTGTAGGTTGTGGTCACTTTGCTGAAGTGCAGGTGGTGAGAGAGAAGGCAACCGGG
GACATCCATGCTATGAAAGTGATGAAGAAGAAGGCTTTATTGGCCCAGGAGCAGGTTTCA
TTTTTTGAGGAAGAGCGTAACATACTATCTCGAAGCACAAGCCCGTGGATCCCCCAATTA
CAGTATGCCTTTCAGGACAAAAATCACCTTTATCTGGTTATGGAATATCAGCCTGGAGGG
GACTTGCTGTCACTTTTGAATAGATATGAGGACCAGTTAGATGAAAACCTGATTCAGTTT
TACCTAGCTGAACTGATTTTGGCTGTTCACAGTGTTCATCAGATGGGATATGTGCATCGA
GACATCAAGCCCGAGAACATTCTCATTGACCGCACAGGACACATCAAGCTGGTGGATTTT
GGATCTGCAGCAAAAATGAATTCAAACAAGATGGTGAATGCCAGACTCCCAATTGGGACC
CCGGATTACATGGCTCCTGAAGTGCTGACTGTGATGAATGCGGACGGAAAAGGCACCTAC
GGCCTGGACTGTGACTGGTGGTCAGTGGGCGTGATTGCCTATGAGATGATTTATGGGAGA
TCCCCCTTCGCAGAGGGAACCTCTGCCAGAACCTTCAATAACATCATGAATTTCCAGCGG
TTTTTGAAGTTTCCAGATGACCCCAAAGTGAGCAGTGACTTTCTTGATCTGATTCAAAGT
TTGTTGTGTGGCCAGAAAGAGAGACTGAAGTTTGAAGGTCTCTGCTGCCATCCTTTCTTC
TCTAAAATCGACTGGAATAACATTCGTAACTCTCCTCCCCCCTTCGTTCCCACCCTCAAG
TCTGACGATGACACCTCCAATTTTGATGAACCAGAGAAGAATTCGTGGGTTTCATCCTCT
CCGTGCCAGCTGAGCCCCTCAGGTTTCTTGGGTGAAGAACTGCCGTTTGTGGGGTTTTCG
TACAGCAAGGCACTGGGGATTCTTGGTAGATCTGAGTCTGTTGTGTCGGGTCTGGACTCC
CCTGCCAAGACTAGCTCCATGGAAAAGAAACTTCTCATCAAAAGCAAAGAGCTACAAGAC
TCTCAGGACAAGTGTCACAAGATGGAGCAGGAAATGACCCGGTTACATCGGAGAGTGTCA
GAGGTGGAGGCTGTGCTTAGTCAGAAGGAGGTGGAGCTGAAGGCCTCTGAGACTCAGAGA
TCCCTCCTGGAGCAGGACCTTGCTACCTACATCACAGAATGCAGTAGCTTAAAGCGAAGT

TTGGAGCAAGCACGGATGGAGGTGTCCCAGGAGGATGACAAAGCACTGCAGCTTCTCCAT
GATATCAGAGAGCAGAGCCGGAAGCTCCAAGAAATCAAAGAGCAGGAGTACCAGGCTCAA
GTGGAAGAAATGAGGTTAATGATGAATCAGTTGGAAGAGGACCTTGTCTCAGCAAGAAGA
CGGAGTGATCTCTACGAATCTGAGCTGAGAGAGTCTCGGCTCGCTGCTGAAGAATTCAAG
CGGAAAGCGACAGAATGTCAGCATAAACTGTTGAAGGCTAAGGATCAAGGGAAGCCTGAA
GTGGGAGAATATGCGAAACTGGAGAAGATCAATGCTGAGCAGCAGCTCAAAATTCAGGAG
CTCCAAGAGAAACTGGAGAAGGCTGTAAAAGCCAGCACCGAGGCCACCGAGCTGCTGCAG
AATATCCGCCAGGCAAAGGAGCGAGCCGAGAGGGAGCTGGAGAAGCTGCAGAACCGAGAG
GATTCTTCTGAAGGCATTAGAAAGAAGCTGGTGGAAGCCGAGGAACGTCGCCATTCTCTG
GAGAACAAGGTAAAGAGACTAGAGACCATGGAGCGTAGAGAAAACAGACTGAAGGATGAC
ATCCAGACAAAATCCCAACAGATCCAGCAGATGGCTGATAAAATTCTGGAACTTGAAGAG
AAACATCGGGAGGCCCAAGTCTCAGCCCAGCACCTAGAAGTGCACCTGAAACAGAAAGAG
CAGCACTATGAGGAAAAGATTAAAGTGTTGGACAATCAGATAAAGAAAGACCTGGCCGAC
AAGGAGACGCTGGAGAACATGATGCAGAGACACGAGGAGGAGGCCCATGAGAAGGGCAAA
ATTCTCAGCGAACAGAAGGCGATGATCAATGCTATGGATTCCAAGATCAGATCCCTGGAA
CAGAGGATTGTGGAACTGTCTGAAGCCAATAAACTTGCAGCAAATAGCAGTCTTTTTACC
CAAAGGAACATGAAGGCTCAAGAAGAGATGATTTCTGAACTCAGGCAACAGAAATTTTAC
CTGGAGACCCAGGCTGGGAAATTGGAGGCCCAGAACCGAAAGCTGGAGGAGCAGCTGGAG
AAGATCAGCCACCAAGACCACAGTGACAAGAATCGGCTGCTGGAACTGGAGACAAGACTG
CGGGAGGTCAGTCTAGAGCACGAGGAGCAGAAACTGGAGCTCAAGCGCCAGCTCACAGAG
CTACAGCTCTCCCTGCAGGAGCGTGAGTCACAGTTGACAGCCCTGCAGGCTGCCCGGGCG
GCGCTGGAAAGCCAGCTTCGCCAGGCAAAGACAGAGCTGGAAGAGACCACAGCAGAAGCC
GAAGAGGAGATCCAGGCGCTCACGGCACATAGAGATGAAATCCAGCGCAAATTTGATGCT
CTTCGTAACAGCTGTACTGTAATCACAGACCTGGAGGAGCAGCTAAACCAGCTGACCGAG
GATAACGCTGAACTCAACAACCAAAACTTCTACTTGTCCAAACAACTCGATGAGGCTTCC
GGCGCCAACGATGAGATTGTACAACTGCGAAGTGAAGTGGACCACCTTCGCCGGGAGATC
ACAGAACGAGAGATGCAGCTTACCAGCCAGAAGCAAACGATGGAGGCTCTGAAGACCACG
TGTACCATGCTGGAGGAACAGGTCATGGATTTGGAGGCCCTAAACGACGAGCTGCTAGAA
AAAGAGCGGCAGTGGGAGGCCTGGAGGAGCGTCCTGGGTGACGAGAAATCCCAGTTTGAG
TGTCGGGTTCGAGAGCTGCAGAGGATGTTGGACACCGAGAAACAGAGCAGGGCGAGAGCC
GATCAGCGGATCACCGAGTCTCGCCAGGTGGTGGAGCTGGCAGTGAAGGAGCACAAGGCG
GAGATTCTCGCTCTGCAGCAGGCTCTCAAAGAGCAGAAGCTGAAGGCCGAGAGCCTCTCT
GACAAGCTCAATGACCTGGAGAAGAAGCATGCTATGCTTGAAATGAATGCCCGAAGCTTA
CAGCAGAAGCTGGAGACTGAACGAGAGCTCAAACAGAGGCTTCTGGAAGAGCAAGCCAAA
TTACAGCAGCAGATGGACCTGCAGAAAAATCACATTTTCCGTCTGACTCAAGGACTGCAA
GAAGCTCTAGATCGGGCTGATCTGCTGAAGACAGAAAGAAGTGACCTGGAGTATCAGCTG
GAAAACATTCAGGTTCTCTATTCTCATGAAAAGGTGAAAATGGAAGGCACTATTTCTCAA
CAAACCAAACTCATCGATTTTCTGCAAGCCAAAATGGACCAACCTGCTAAAAAGAAAAAG
GGTTTATTTAGTCGACGGAAAGAGGACCCTGCTTTGCCCACACAGGTTCCTCTGCAGTAC
AATGAGCTGAAGCTGGCCCTGGAGAAGGAGAAAGCTCGCTGTGCAGAGCTAGAGGAAGCC
CTTCAGAAGACCCGCATCGAGCTCCGGTCCGCCCGGGAAGAAGCTGCCCACCGCAAAGCG
ACGGACCACCCACACCCATCCACGCCAGCCACCGCGAGGCAGCAGATCGCCATGTCCGCC
ATCGTGCGGTCACCAGAGCACCAGCCCAGTGCCATGAGCCTGCTGGCCCCGCCATCCAGC
CGCAGAAAGGAGTCTTCAACTCCAGAGGAATTTAGTCGGCGTCTTAAGGAACGCATGCAC
CACAATATTCCTCACCGATTCAACGTAGGACTGAACATGCGAGCCACAAAGTGTGCTGTG
TGTCTGGATACCGTGCACTTTGGACGCCAAGCATCCAAATGTCTCGAATGTCAGGTGATG
TGTCACCCCAAGTGCTCCACGTGCTTGCCAGCCACCTGCGGCCTGCCTGCCGAATACGCC

ACACACTTCACTGAGGCCTTCTGCCGCGACAAAATGAACTCCCCAGGTCTCCAGACCAAG
GAGCCCAGCAGCAGCTTGCACCTGGAAGGGTGGATGAAGGTGCCCAGGAATAACAAACGA
GGACAGCAAGGCTGGGACAGGAAGTACATTGTCCTGGAGGGATCAAAAGTCCTTATTTAT
GACAATGAAGCCAGAGAAGCTGGACAGAGGCCGGTGGAAGAATTTGAGCTGTGCCTTCCC
GACGGGGATGTATCTATTCATGGTGCCGTTGGTGCTTCCGAACTCGCAAATACAGCCAAA
GCAGATGTCCCATACATACTGAAGATGGAATCTCACCCGCACACCACCTGCTGGCCCGGG
AGAACCCTCTACTTGCTAGCTCCCAGCTTCCCTGACAAACAGCGCTGGGTCACCGCCTTA
GAATCAGTTGTCGCAGGTGGGAGAGTTTCTAGGGAAAAAGCAGAAGCTGATGCTAAATTG
CTTGGAAACTCCCTGCTGAAACTGGAAGGTGATGACCGTCTAGACATGAACTGCACACTG
CCCTTCAGTGACCAGGTGGTGTTGGTGGGCACCGAGGAAGGGCTCTACGCCCTGAATGTC
TTGAAAAACTCCCTAACGCACGTCCCAGGAATTGGAGCAGTCTTCCAAATTTATATTATC
AAGGACCTGGAGAAGCTACTCATGATAGCAGGAGAAGAGCGGGCACTGTGTCTTGTGGAC
GTGAAGAAAGTGAAGCAGTCCCTGGCGCAGTCCCACCTGCCCGCCCAGCCCGACATCTCA
CCCAACATTTTTGAAGCTGTCAAGGGCTGCCACTTGTTTGGGGCTGGCAAGATTGAGAAC
GGGCTCTGCATCTGTGCAGCCATGCCCAGCAAAGTCGTGATTCTCCGCTACAACGAAAAC
CTCAGCAAATACTGCATTCGGAAAGAGATAGAGACCTCAGAGCCTTGCAGCTGTATCCAC
TTCACCAATTACAGTATCCTCATTGGAACCAATAAATTCTACGAAATCGACATGAAGCAG
TACACGCTGGAGGAATTCCTGGATAAGAATGACCATTCCTTGGCACCTGCTGTGTTTGCC
GCCTCTTCCAACAGCTTCCCTGTCTCGATCGTGCAGGTGAACAGTGCAGGGCAGCGAGAG
GAGTACTTGCTGTGTTTCCACGAGTTTGGAGTGTTCGTGGATTCTTACGGAAGACGTAGC
CGCACAGACGACCTCAAGTGGAGTCGCTTACCTTTGGCCTTTGCCTACAGAGAACCCTAT
CTGTTTGTGACCCACTTCAACTCACTCGAAGTAATTGAGATCCAGGCACGCTCCTCGGCA
GGGACCCCTGCCCGAGCGTACCTGGACATCCCGAACCCACGCTACCTGGGCCCTGCCATT
TCCTCAGGAGCGATTTACTTGGCATCCTCATACCAGGATAAATTAAGGGTCATTTGCTGC
AAGGGAAACCTCGTGAAGGAGTCTGGCACTGAACACCACCGGGGCCCGTCCACCTCCCGC
AGCAGCCCCAACAAGCGAGGCCCGCCCACGTACAACGAGCACATCACCAAGCGCGTGGCC
TCCAGCCCGGCGCCGCCCGAAGGCCCCAGCCACCCCCGAGAGCCAAGCACACCCCACCGC
TACCGCGAGGGGCGGACCGAGCTGCGCAGGGACAAGTCTCCTGGCCGCCCCCTGGAGCGG
GAGAAGTCCCCGGGCCGGATGCTCAGCACACGGAGAGAGCGGTCCCCTGGGAGGCTGTTT
GAAGACAGCAGCAGGGGCCGGCTGCCTGCGGGAGCCGTGAGGACCCCGCTGTCCCAGGTG
AACAAGGTCTGGGACCAGTCTTCAGTA
>gorilla XM_004053983.2
ATGTTGAAGTTCAAATATGGAGCGCGGAATCCTTTGGATGCTGGTGCTGCTGAACCCATTGCCAGCCGGG
CCTCCAGGCTGAATCTGTTCTTCCAGGGGAAACCACCCTTTATGACTCAACAGCAGATGTCTCCTCTTTC
CCGAGAAGGGATATTAGATGCCCTCTTTGTTCTCTTTGAAGAATGCAGTCAGCCTGCTCTGATGAAGATT
AAGCATGTGAGCAACTTTGTCCGGAAGTATTCCGACACCATAGCTGAGTTACAGGAGCTCCAGCCTTCGG
CAAAGGACTTCGAAGTCAGAAGTCTTGTAGGTTGTGGTCACTTTGCTGAAGTGCAGGTGGTAAGAGAGAA
AGCAACCGGGGACATCTATGCTATGAAAGTGATGAAGAAGAAGGCTTTATTGGCCCAGGAGCAGGTTTCA
TTTTTTGAGGAAGAGCGGAACATATTATCTCGAAGCACAAGCCCGTGGATCCCCCAATTACAGTATGCCT
TTCAGGACAAAAATCACCTTTATCTGGTCATGGAATATCAGCCTGGAGGGGACTTGCTGTCACTTTTGAA
TAGATATGAGGACCAGTTAGATGAAAACCTGATACAGTTTTACCTAGCTGAGCTGATTTTGGCTGTTCAC
AGCGTTCATCTGATGGGATACGTGCATCGAGACATCAAGCCTGAGAACATTCTCATTGACCGCACAGGAC
ACATCAAGCTGGTGGATTTTGGATCTGCCGCGAAAATGAATTCAAACAAGATGGTGAATGCCAAACTCCC
GATTGGGACCCCAGATTACATGGCTCCTGAAGTGCTGACTGTGATGAACGGGGATGGAAAAGGCACCTAC
GGCCTGGACTGTGACTGGTGGTCAGTGGGCGTGATTGCCTATGAGATGATTTATGGGAGATCCCCCTTCG
CAGAGGGAACCTCTGCCAGAACCTTCAATAACATCATGAATTTCCAGCGGTTTTTGAAATTTCCAGATGA
CCCCAAAGTGAGCAGTGACTTTCTTGATCTGATTCAAAGCTTGTTGTGTGGCCAGAAAGAGAGACTGAAG

TTTGAAGGTCTTTGCTGCCATCCTTTCTTCTCTAAAATCGACTGGAACAACATTCGTAACTCTCCTCCCC
CCTTCGTTCCCACCCTCAAGTCTGACGATGACACCTCCAATTTTGATGAACCAGAGAAGAATTCGTGGGT
TTCATCCTCTCCGTGCCAGCTGAGCCCCTCAGGTTTCTCGGGTGAAGAACTGCCGTTTGTGGGGTTTTCG
TACAGCAAGGCACTGGGGATTCTTGGTAGATCTGAGTCTGTTGTGTCGGGTCTGGACTCCCCTGTCAAGA
CTAGCTCCATGGAAAAGAAACTTCTCATCAAAAGCAAAGAGCTACAAGACTCTCAGGACAAGTGTCACAA
GATGGAGCAGGAAATGACCCGGTTACATCGGAGAGTGTCAGAGGTGGAGGCTGTGCTTAGTCAGAAGGAG
GTGGAGCTGAAGGCCTCTGAGACTCAGAGATCCCTCCTGGAGCAGGACCTTGCTACCTACATCACAGAAT
GCAGTAGCTTAAAGCGAAGTTTGGAGCAAGCACGGATGGAGGTGTCCCAGGAGGATGACAAAGCACTGCA
GCTTCTCCATGATATCAGAGAGCAGAGCCGGAAGCTCCAAGAAATCAAAGAGCAGGAGTACCAGGCTCAA
GTGGAAGAAATGAGGTTGATGATGAATCAGTTGGAAGAGGACCTTGTCTCAGCAAGAAGACGGAGTGATC
TCTACGAATCTGAGCTGAGAGAGTCTCGGCTTGCTGCTGAAGAATTCAAGCGGAAAGCGACAGAATGTCA
GCATAAACTGTTGAAGGCTAAGGATCAAGGGAAGCCTGAAGTGGGAGAATATGCGAAACTGGAGAAGATC
AATACTGAGCAGCAGCTCAAAATTCAGGAGCTCCAAGAGAAACTGGAGAAGGCTGTGAAAGCCAGCACAG
AGGCCACCGAGCTGCTGCAGAATATCCGCCAGGCCAAGGAGCGAGCCGAGAGGGAGCTGGAGAAGCTGCA
GAACCGAGAGGATTCTTCTGAAGGCATCAGAAAGAAGCTGGTGGAAGCTGAGGAACGCCGCCATTCTCTG
GAGAACAAGGTAAAGAGACTAGAGACCATGGAGCGTAGAGAAAACAGACTGAAGGATGACATCCAGACAA
AATCCCAACAGATCCAGCAGATGGCTGATAAAATTCTGGAGCTCGAAGAGAAACATCGGGAGGCCCAAGT
CTCAGCCCAGCATCTAGAAGTGCACCTGAAACAGAAAGAGCAGCACTATGAGGAAAAGATTAAAGTGTTG
GACAATCAGATAAAGAAAGACCTGGCCGACAAGGAGACGCTGGAGAACATGATGCAGAGACACGAGGAGG
AGGCCCATGAGAAGGGCAAAATTCTCAGCGAACAGAAGGCGATGATCAATGCTATGGATTCCAAGATCAG
ATCCCTGGAACAGAGGATTGTGGAACTGTCTGAAGCCAATAAACTTGCAGCAAATAGCAGTCTTTTTACC
CAAAGGAACATGAAGGCCCAAGAAGAGATGATTTCTGAACTCAGGCAACAGAAATTTTACCTGGAGACAC
AAGCTGGGAAGTTGGAGGCCCAGAACCGAAAACTGGAGGAGCAGCTGGAGAAGATCAGCCACCAAGACCA
CAGTGACAAGAATCGGCTGCTGGAACTGGAGACAAGATTGCGGGAGGTCAGTCTAGAGCACGAGGAGCAG
AAACTGGAGCTCAAGCGCCAGCTCACAGAGCTACAGCTCTCCCTGCAGGAGCGCGAGTCACAGTTGACAG
CCCTGCAGGCTGCACGGGCGGCCCTGGAGAGCCAGCTTCGCCAGGCGAAGACAGAGCTGGAAGAGACCAC
AGCAGAAGCCGAAGAGGAGATCCAGGCGCTCACGGCACATAGAGATGAAATCCAGCGCAAATTTGATGCT
CTTCGTAACAGCTGTACTGTAATCACAGACCTGGAGGAGCAGCTAAACCAGCTGACCGAGGACAATGCTG
AACTCAACAACCAAAACTTCTACTTGTCCAAACAACTCGATGAGGCTTCTGGCGCCAACGATGAGATTGT
ACAACTGCGAAGTGAAGTGGACCATCTCCGCCGGGAGATCACGGAACGAGAGATGCAGCTTACCAGCCAG
AAGCAAACGATGGAGGCTCTGAAGACCACGTGCACCATGCTGGAGGAACAGGTCATGGATTTGGAGGCCC
TAAACGATGAGCTGCTAGAAAAAGAGCGGCAGTGGGAGGCCTGGAGGAGCGTCCTGGGTGATGAGAAATC
CCAGTTTGAGTGTCGGGTTCGAGAGCTGCAGAGGATGCTGGACACCGAGAAACAGAGCAGGGCGAGAGCC
GATCAGCGGATCACCGAGTCTCGCCAGGTGGTGGAGCTGGCAGTGAAGGAGCACAAGGCTGAGATTCTTG
CTCTGCAGCAGGCTCTCAAAGAGCAGAAGCTGAAGGCCGAGAGCCTCTCTGACAAGCTCAATGACCTGGA
GAAGAAGCATGCTATGCTTGAAATGAATGCCCGAAGCTTACAGCAGAAGCTGGAGACTGAACGAGAGCTC
AAACAGAGGCTTCTGGAAGAGCAAGCCAAATTACAGCAGCAGATGGACCTGCAGAAAAATCACATTTTCC
GTCTGACTCAAGGACTGCAAGAAGCTCTAGATCGGGCTGATCTACTGAAGACAGAAAGAAGTGACTTGGA
GTATCAGCTGGAAAACATTCAGGTTCTCTATTCTCATGAAAAGGTGAAAATGGAAGGCACTATTTCTCAA
CAAACCAAACTCATTGATTTTCTGCAAGCCAAAATGGACCAACCTGCTAAAAAGAAAAAGGGTTTATTTA
GTCGACGGAAAGAGGACCCTGCTTTACCCACACAGGTTCCTCTGCAGTACAATGAGCTGAAGCTGGCCCT
GGAGAAGGAGAAAGCTCGCTGTGCAGAGCTAGAGGAAGCCCTTCAGAAGACCCGCATCGAGCTCCGGTCC
GCCCGGGAGGAAGCTGCCCACCGCAAAGCAACGGACCACCCACACCCATCCACTCCAGCCACCGCGAGGC
AGCAGATCGCCATGTCCGCCATCGTGCGGTCGCCAGAGCACCAGCCCAGTGCCATGAGCCTGCTGGCCCC
GCCATCCAGCCGCAGAAAGGAGTCTTCAACTCCAGAGGAATTTAGTCGGCGTCTTAAGGAACGCATGCAC
CACAATATTCCTCACCGATTCAACGTAGGACTGAACATGCGAGCCACAAAGTGTGCTGTGTGTCTGGATA
CCGTGCACTTTGGACGCCAGGCATCCAAATGTCTCGAATGTCAGGTGATGTGTCACCCCAAGTGCTCCAC

GTGCTTGCCAGCCACCTGCGGCTTGCCTGCTGAATATGCCACACACTTCACCGAGGCCTTCTGCCGTGAC
AAAATGAACTCCCCAGGTCTCCAGACCAAGGAGCCCAGCAGCAGCTTGCACCTGGAAGGGTGGATGAAGG
TGCCCAGGAATAACAAACGAGGACAGCAAGGCTGGGACAGGAAGTACATTGTCCTGGAGGGATCAAAAGT
CCTCATTTATGACAATGAAGCCAGAGAAGCTGGACAGAGGCCGGTGGAAGAATTTGAGCTGTGCCTTCCC
GACGGGGATGTATCTATTCATGGTGCCGTTGGTGCTTCCGAACTGGCAAATACAGCCAAAGCAGATGTCC
CATACATACTGAAGATGGAATCTCACCCGCACACCACCTGCTGGCCCGGGAGAACCCTCTACTTGCTAGC
TCCCAGCTTCCCTGACAAACAGCGCTGGGTCACCGCCTTAGAATCAGTTGTCGCAGGTGGGAGAGTTTCT
AGGGAAAAAGCAGAAGCTGATGCTAAACTGCTTGGAAACTCCCTGCTGAAACTGGAAGGTGATGACCGTC
TAGACATGAACTGCACACTGCCCTTCAGTGACCAGGTGGTGTTGGTGGGCACCGAGGAAGGGCTCTACGC
CCTGAATGTCTTGAAAAACTCCCTAACCCATGTCCCAGGAATTGGAGCAGTCTTCCAAATTTATATTATC
AAGGACCTGGAGAAGCTACTCATGATAGCAGGAGAAGAGCGGGCACTGTGTCTTGTGGACGTGAAGAAAG
TGAAACAGTCCCTGGCGCAGTCCCACCTGCCCGCCCAGCCTGACATCTCACCCAACATTTTTGAAGCTGT
CAAGGGCTGCCACTTGTTTGGGGCAGGCAAGATTGAGAATGGGCTCTGCATCTGTGCAGCCATGCCTAGC
AAAGTCGTCATTCTCCGCTACAACGAAAACCTCAGCAAATACTGCATCCGGAAAGAGATAGAGACCTCAG
AGCCCTGCAGCTGTATCCACTTCACCAATTACAGTATCCTCATTGGAACCAATAAATTCTATGAAATCGA
CATGAAGCAGTACACGCTCGAGGAATTTCTGGATAAGAATGACCATTCCTTGGCACCTGCTGTGTTTGCC
GCCTCTTCCAACAGCTTCCCTGTCTCAATCGTGCAGGTGAACAGTGCAGGGCAGCGAGAGGAGTACTTGC
TGTGTTTCCACGAATTTGGAGTGTTTGTGGATTCTTACGGAAGACGTAGCCGCACAGACGATCTCAAGTG
GAGTCGCTTACCTTTGGCCTTTGCCTACAGAGAACCCTATCTGTTTGTGACCCACTTCAACTCACTCGAA
GTAATTGAGATCCAGGCACGCTCCTCAGCAGGGACCCCTGCCCGAGCATACCTGGACATCCCAAACCCGC
GCTACCTGGGCCCTGCCATTTCCTCAGGAGCGATTTACTTGGCGTCCTCATACCAGGATAAATTAAGGGT
CATTTGCTGCAAGGGAAACCTCGTGAAGGAGTCCGGCACTGAACACCACCGGGGCCCGTCCACCTCCCGC
AGCAGCCCCAACAAGCGAGGCCCGCCCACGTACAACGAGCACATCACCAAGCGCGTGGCCTCCAGCCCAG
CGCCGCCCGAAGGCCCCAGCCACCCGCGAGAGCCAAGCACACCCCACCGCTACCGCGAGGGGCGGACTGA
GCTGCGCAGGGACAAGTCTCCTGGCCGCCCCCTGGAGCGAGAGAAGTCCCCGGGCCGGATGCTCAGCACG
CGGAGAGAGCGGTCCCCCGGGAGGCTGTTTGAAGACAGCAGCAGGGGCCGGCTGCCTGCGGGAGCCGTGA
GGACCCCGCTGTCCCAGGTGAACAAGGTCTGGGACCAGTCTTCAGTA
>Pongo abelii XM_003907237.4
ATGTTGAAGTTCAAATATGGAGCGCGGAATCCTTTGGATGCTGGTGCTGCTGAACCCATTGCCAGCCGGG
CCTCCAGGCTGAATCTGTTCTTCCAGGGGAAACCACCCTTTATGACTCAACAGCAGATGTCTCCTCTTTC
CCGAGAAGGGATATTAGATGCCCTCTTTGTTCTCTTTGAAGAATGCAGTCAGCCTGCTCTGATGAAGATT
AAGCATGTGAGCAACTTTGTCCGGAAGTATTCCGACACCATAGCTGAGTTACAGGAGCTCCAGCCTTCGG
CAAAGGACTTCGAAGTCAGAAGTCTTGTAGGTTGTGGTCACTTTGCTGAAGTGCAGGTGGTAAGAGAGAA
AGCAACCGGGGACATCTATGCTATGAAAGTGATGAAGAAGAAGGCTTTATTGGCCCAGGAGCAGGTTTCA
TTTTTTGAGGAAGAGCGGAACATATTATCTCGAAGCACAAGCCCGTGGATCCCCCAATTACAGTATGCCT
TTCAGGACAAAAATCACCTTTATCTGGTCATGGAATATCAGCCTGGAGGGGACTTGCTGTCACTTTTGAA
TAGATATGAGGACCAGTTAGATAAAAACCTGATACAGTTTTACCTAGCTGAGCTGATTTTGGCTGTTCAC
AGCGTTCATCTGATGGGATACGTGCATCGAGACATCAAGCCTGAGAACATTCTCATTGACCGCACAGGAC
ACATCAAGCTGGTGGATTTTGGATCTGCCGCGAAAATGAATTCAAACAAGATGGTGAATGCCAAACTCCC
TATTGGGACCCCAGATTACATGGCTCCTGAAGTGCTGACTGTGATGAACGGGGACGGAAAAGGCACCTAC
AGCCTGGACTGTGACTGGTGGTCAGTGGGCGTGATTGCCTATGAGATGATTTATGGAGATCCCCCTTCA
CAGAGGGAACCTCTGCCAGAACCTTCAATAACATCATGAATTTCCAGCGGTTTTTGAGGTTTCCAGATGA
CCCCAAAGTGAGCAGTGACTTTCTTGATCTGATTCAAAGCTTGTTGTGTGGCCAGAAAGAGAGACTGAAG
TTTGAAGGTCTTTGCTGCCATCCTTTCTTCTCTAAAATTGACTGGAATAACATTCGTAACTCTCCTCCCC
CCTTCGTTCCCACCCTCAAGTCTGACGATGACACCTCCAATTTTGATGAACCAGAGAAGAATTCGTGGGT
TTCATCCTCTCCGTGCCAGCTGAGCCCCTCAGGTTTCTCGGGTGAAGAACTGCCGTTTGTGGGGTTTTCG
TACAGCAAGGCACTGGGGATTCTTGGTAGATCTGAGTCTGTTGTGTCGGGTCTGGACTCCCCTGCCAAGA

CTAGCTCCATGGAAAAGAAACTTCTCATCAAAAGCAAAGAGCTACAAGACTCTCAGGACAAGTGTCACAA
GATGGAGCAGGAAATGACCCGGTTACATCGGAGAGTGTCAGAGGTGGAGGCTGTGCTTAGTCAGAAGGAG
GTGGAGCTGAAGGCCTCTGAGACTCAGAGATCCCTCCTGGAGCAGGACCTTGCTACCTACATCACAGAAT
GCAGTAGCTTAAAGCGAAGTTTGGAGCAAGCACGGATGGAGGTGTCCCAGGAGGATGACAAAGCACTGCA
GCTTCTCCATGATATCAGAGAGCAGAGCCGGAAGCTCCAAGAAATCAAAGAGCAGGAGTACCAGGCTCAA
GTGGAAGAAATGAGGTTGATGATGAATCAGTTGGAAGAGGACCTTGTCTCAGCAAGAAGACGGAGTGATC
TCTATGAATCTGAGCTGAGAGAGTCTCGGCTTGCTGCTGAAGAATTCAAGCGGAAAGCGACAGAATGTCA
GCATAAACTGTTGAAGGCTAAGGATCAAGGGAAGCCTGAAGTGGGAGAATATGTGAAACTGGAGAAGATC
AATGCTGAGCAGCAGCTCAAAATTCAGGAGCTCCAAGAGAAACTGGAGAAGGCTGTAAAAGCCAGCACGG
AGGCCACTGAGCTGCTGCAGAATATCCGCCAGGCAAAGGAGCGAGCCGAGAGGGAGCTGGAGAAGCTGCA
GAACCGAGAGGATTCTTCTGAAGGCATCAGAAAGAAGCTGGTGGAAGCTGAGGAACGCCGCCATTCTCTG
GAGAACAAGGTAAAGAGACTAGAGACCATGGAGCGTAGAGAAAACAGACTGAAGGATGACATCCAGACAA
AATCCCAACAGATCCAGCAGATGGCTGATAAAATTCTGGAGCTCGAAGAGAAACATCGGGAGGCCCAAGT
CTCAGCCCAGCACCTAGAAGTGCACCTGAAACAGAAAGAGCAGCACTATGAGGAAAAGATTAAAGTGTTG
GACAATCAGATAAAGAAAGACCTGGCCGACAAGGAGACGCTTGAGAACATGATGCAGAGACACGAAGAGG
AGGCCCATGAGAAGGGCAAAATTCTCAGCGAACAGAAGGCGATGATCAATGCTATGGATTCCAAGATCAG
ATCCCTGGAACAGAGGATTGTGGAACTGTCTGAAGCCAATAAACTTGCAGCAAATAGCAGTCTTTTTACC
CAAAGGAACATGAAGGCCCAAGAAGAGATGATTTCTGAACTCAGGCAACAGAAATTTTACCTGGAGACAC
AGGCTGGGAAGTTGGAGGCCCAGAACCGAAAGCTGGAGGAGCAGCTGGAGAAGATCAGCCACCAAGACCA
CAGTGACAAGAATCGGCTGCTGGAACTGGAAACAAGATTGCGGGAGGTCAGTCTAGAGCACGAGGAGCAG
AAACTGGAGCTCAAGCGCCAGCTCACAGAGCTACAGCTCTCCCTGCAGGAGCGCGAGTCACAGTTGACAG
CCCTGCAGGCTGCACGGGCGGCCCTGGAGAGCCAGCTTCGCCAGGCGAAGACAGAGCTGGAAGAGACCAC
AGCAGAAGCCGAAGAGGAGATCCAGGCGCTCACGGCACATAGAGATGAAATCCAGCGCAAATTTGATGCT
CTTCGTAACAGCTGTACTGTAATCACAGACCTGGAGGAGCAGCTAAACCAGCTGACCGAGGACAACGCTG
AACTCAACAACCAAAACTTCTACTTGTCCAAACAACTCGATGAGGCTTCTGGCGCCAACGATGAGATTGT
ACAACTGCGAAGTGAAGTGGACCATCTCCGCCGGGAGATCACAGAACGAGAGATGCAGCTTACCAGCCAG
AAGCAAACGATGGAGGCTCTGAAGACCACGTGCACCATGCTGGAGGAACAGGTCATGGATTTGGAGGCCC
TAAACGATGAGCTGCTAGAAAAAGAGCGGCAGTGGGAGGCCTGGAGGAGTGTCCTGGGTGATGAGAAATC
CCAGTTTGAGTGTCGGGTTCGAGAGCTGCAGAGGATGCTGGACACCGAGAAACAGAGCAGGGCGAGAGCC
GATCAGCGGATCACCGAGTCTCGCCAGGTGGTGGAGCTGGCAGTGAAGGAGCACAAGGCCGAGATTCTCG
CTCTGCAGCAGGCTCTCAAAGAGCAGAAGCTGAAGGCCGAGAGCCTCTCTGACAAGCTCAATGACCTGGA
GAAGAAGCACGCTATGCTTGAAATGAATGCCCGAAGCTTACAGCAGAAGCTGGAGACTGAACGAGAGCTC
AAACAGAGGCTTCTGGAAGAGCAAGCCAAATTACAGCAGCAGATGGACCTGCAGAAAAATCACATTTTCC
GTCTGACTCAAGGACTGCAAGAAGCTCTAGATCGGGCTGATCTACTGAAGACAGAAAGAAGTGACCTGGA
ATATCAGCTGGAAAACATTCAGGTTCTCTATTCTCATGAAAAGGTGAAAATGGAAGGCACTATTTCTCAA
CAAACCAAACTCATTGATTTTCTGCAAGCCAAAATGGACCAACCTGCTAAAAAGAAAAAGGGTTTATTTA
GTCGACGGAAAGAGGACCCTGCTTTGCCCACACAGGTTCCTCTGCAGTACAATGAGCTGAAGCTGGCCCT
GGAGAAGGAGAAAGCTCGCTGTGCAGAGCTAGAGGAAGCCCTTCAGAAGACCCGCATCGAGCTCCGGTCC
GCCCGGGAGGAAGCTGCCCACCGCAAAGCGACGGACCACCCACACCCATCCACGCCAGCCACCGCGAGGC
AGCAGATCGCCATGTCCGCCATTGTGCGGTCGCCAGAGCACCAGCCCAGTGCCATGAGCCTGCTGGCCCC
GCCATCCAGCCGCAGAAAGGAGTCTTCAACTCCAGAGGAATTTAGTCGGCGTCTTAAGGAACGCATGCAT
CACAATATTCCTCACCGATTCAACGTAGGACTGAACATGCGAGCCACAAAGTGTGCTGTGTGTCTGGATA
CCGTGCACTTTGGACGCCAGGCATCCAAATGTCTCGAATGTCAGGTGATGTGTCACCCCAAGTGCTCCAC
GTGCTTGCCAGCCACCTGCGGCTTGCCTGCTGAATACGCCACACACTTCACCGAGGCCTTCTGCCGCGAC
AAAATGAACTCCCCAGGTCTCCAGACCAAGGAGCCCAGCAGCAGCTTGCACCTGGAAGGGTGGATGAAGG
TGCCCAGGAATAACAAACGAGGACAGCAAGGCTGGGACAGGAAGTACATTGTCCTGGAGGGATCAAAAGT
CCTCATTTACGACAATGAAGCCAGAGAAGCTGGACAGAGGCCGGTGGAAGAATTTGAGCTGTGCCTTCCC

GACGGGGATGTATCTATTCATGGTGCCGTTGGTGCTTCCGAACTCGCAAATACAGCCAAAGCAGATGTCC
CATACATACTGAAGATGGAATCTCACCCGCACACCACCTGCTGGCCCGGGAGAACCCTCTACTTGCTAGC
TCCCAGCTTCCCTGACAAACAGCGCTGGGTCACCGCCTTAGAATCAGTTGTCGCAGGTGGGAGAGTTTCT
AGGGAAAAAGCAGAAGCCGATGCTAAACTGCTTGGAAACTCCCTGCTGAAACTGGAAGGTGATGACCGTC
TAGACATGAACTGCACATTGCCCTTCAGTGACCAGGTGGTGTTGGTGGGCACCGAGGAAGGTCTCTACGC
CCTGAATGTCTTGAAAAACTCCCTAACCCATGTCCCAGGAATTGGAGCAGTCTTCCAAATTTATATTATC
AAGGACCTGGAGAAGCTACTCATGATAGCAGGAGAAGAGCGGGCACTGTGTCTTGTGGACGTGAAGAAAG
TGAAACAGTCCCTGGCGCAGTCCCACCTGCCCGCCCAGCCCGACATCTCACCCAACATTTTTGAAGCTGT
CAAGGGCTGCCACTTGTTTGGGGCAGGCAAGATTGAGAACGGGCTCTGCATCTGTGCAGCCATGCCCAGC
AAAGTCGTCATTCTCCGCTACAACGAAAACCTCAGCAAATACTGCATCCGGAAAGAGATAGAGACCTCAG
AGCCCTGCAGCTGTATCCACTTCACCAATTACAGTATCCTCATTGGAACCAATAAATTCTACGAAATCGA
CATGAAGCAGTACACGCTCGAGGAATTCCTGGATAAGAATGACCATTCCTTGGCACCTGCTGTGTTTGCC
GCCTCTTCCAACAGCTTCCCTGTCTCGATCGTGCAGGTGAACAGCGCAGGGCAGCGAGAGGAGTACTTGC
TGTGTTTCCACGAATTTGGAGTGTTCGTGGATTCTTACGGAAGACGCAGCCGCACAGACGATCTCAAGTG
GAGTCGCTTACCTTTGGCCTTTGCCTACAGAGAACCCTATCTGTTTGTGACCCACTTCAACTCACTCGAA
GTAATTGAGATCCAGGCACGCTCCTCGGCAGGGACCCCTGCCCGAGCGTACCTGGACATCCCGAACCCGC
GCTACCTGGGCCCTGCCATTTCCTCAGGAGCGATTTACTTGGCGTCCTCATACCAGGATAAATTAAGGGT
CATTTGCTGCAAGGGAAACCTCGTGAAGGAGTCCGGCACTGAACACCACCGAGGCCCGTCCACCTCCCGC
AGCAGCCCCAACAAGCGAGGTCCGCCCACGTACAATGAGCACATCACCAAGCGCGTGGCCTCCAGCCCAG
CGCCGCCCGAAGGCCCCAGCCACCCGCGAGAGCCAAGCACACCCCACCGCTACCGCGAGGGGCGGACCGA
GCTGCGCAGGGACAAGTCTCCTGGCCGCCCCCTGGAGCGAGAGAAGTCCCCGGGCCGGATGCTCAGCACG
CGGAGAGAGCGGTCCCCCGGAAGGCTGTTTGAAGACAGCAGCAGGGGCCGGCTGCCTGCGGGAGCCGTGA
GGACCCCGCTGTCCCAGGTGAACAAGGTCTGGGACCAGTCTTCAGTA
>marmoset ENSCJAT00000017650.1
ATGCTGAAGTTCAAATATGGAGCGAGGAATCCTTTGGATGCTGGTGCTGCTGAACCCATT
GCCAGCCGGGCCTCCAGGCTGAATCTGTTCTTCCAGGGGAAACCACCCTTTATGACTCAA
CAGCAGATGTCTCCTCTTTCCCGAGAAGGGATATTAGATGCCCTCTTTGTTCTCTTTGAA
GAGTGCAGTCAGCCTGCTCTGATGAAGATTAAGCATGTGAGCAACTTTGTCCGGAAGTAT
TCCGACACCATAGCTGAGTTACAGGAGCTCCAGCCTTCGGCAAAGGACTTTGAAGTCAGA
AGTCTTGTAGGTTGTGGTCACTTTGCTGAAGTGCAGGTGGTAAGAGAGAAAGCAACTGGG
GACATCTATGCCATGAAAGTGATGAAGAAGAAGGCCTTATTGGCCCAGGAGCAGGTTTCG
TTTTTTGAGGAAGAACGAAACATATTATCTCGAAGCACAAGCCCTTGGATCCCCCAATTA
CAGTATGCCTTTCAGGACAAAAATCACTTGTATCTGGTCATGGAATATCAGCCTGGAGGG
GACTTGCTGTCACTTTTGAATAGATTTGAGGACCAATTAGATGAAAATCTGATTCAGTTT
TACCTGGCTGAACTGATTTTGGCTGTTCACAGCGTTCATCAGATGGGATATGTGCATCGA
GACATCAAGCCCGAGAACATTCTCATTGACCGCACAGGACACATCAAGCTGGTGGATTTT
GGATCAGCTGCGAAAATGAATTCAAACAAGATGGTGAGTGCCAGACTCCCAGTTGGGACC
CCAGATTACATGGCTCCTGAAGTGCTGACTGTGATGAATGGGGATGGAAAAGGCACCTAC
GGCCTTGACTGTGACTGGTGGTCGGTGGGCGTGATTGCCTATGAGATGATTTATGGGAGA
TCCCCCTTTGCAGAGGGAACCTACGCCAGAACCTTCAATAACATCATGAATTTCCAGCGG
TTTTTGAAGTTTCCAGATGACCCCAAAGTGAGCAGTGACTTTCTTGATCTGATTCAAAGT
TTGTTGTGTGGCCAGAAAGAGAGGCTGAAGTTTGAAGGCCTTTGCTGCCATCCTTTCTTC
TCGAAAATTGACTGGAATAACATTCATAACTCTCCTCCCCCCTTCGTTCCCACCCTCAAG
TCTGACGATGACACCTCCAATTTTGATGAACCAGAGAAGAATTCGTGGGTTTCATCCTCT
CCGTGCCAGCTGAGCCCCTCAGGTTTCTCGGGTGAAGAACTGCCGTTTGTGGGGTTTTCC
TACAGCAAGGCACTGGGGATTCTTGGTAGATCTGAGTCTGTTGTGTCGGGTCTGGACTCC
CCTGCCAAGACTAGCTCCATGGAAAAGAAACTTCTCATCAAAAGCAAAGAGCTACAAGAC

TCTCAGGACAAGTGTCACAAGATGGAGCAGGAAATGACCCGGTTACATCGGAGAGTGTCA
GAGGTGGAGGCTGTGCTTAGTCAGAAGGAGGTGGAGCTGAAGGCCTCTGAGACTCAGAGA
TCCCTCCTGGAGCAGGACCTTGCTACCTACATCACAGAATGCAGTAGCTTAAAGCGAAGT
TTGGAGCAAGCACGGATGGAGGTGTCCCAGGAGGATGACAAAGCACTGCAGCTTCTCCAT
GATATCAGAGAGCAGAGCCGGAAGCTCCAAGAAATCAAAGAGCAGGAGTACCAGGCTCAA
GTGGAAGAAATGAGGTTGATGATGAATCAGTTGGAAGAGGACCTTGTCTCAGCAAGAAGA
CGGAGTGATCTCTACGAATCAGAGCTGAGAGAGTCTCGGCTCGCTGCCGAAGAATTCAAG
CGGAAAGCAACAGAATGTCAGCATAAACTACTGAAGGCTAAGGATCAAGGGAAGCCGGAG
GTGGGAGAATATGCTAAGCTGGAGAAGATCAATGCTGAGCAGCAGCTCAAAATTCAGGAG
CTCCAAGAGAAACTGGAGAAGGCTGTAAAAGCCAGCACAGAGGCCACCGAGCTGCTGCAG
AATATTCGCCAGGCAAAGGAGCGAGCTGAGAGGGAGCTGGAGAAACTGCAGAACCGAGAG
GATTCTTCTGAAGGCATCAGAAAGAAGCTGGTGGAAGCCGAGGAACGCCGCCATTCTCTG
GAGAACAAGGTAAAGAGACTAGAGACCATGGAGCGTAGAGAAAACAGACTGAAGGATGAC
ATCCAGACAAAATCCCAACAGATCCAGCAGATGGCTGATAAAATTCTGGAGCTCGAAGAG
AAGCATCGGGAGGCCCAAGTCTCAGCCCAGCACCTAGAAGTGCACCTGAAACAGAAAGAG
CAGCACTACGAGGAAAAAATTAAAGTGTTGGACAATCAGATAAAGAAAGACCTGGCCGAC
AAGGAGACGCTGGAGAACATGATGCAGAGACACGAGGAGGAGGCCCATGAGAAGGGCAAA
ATTCTCAGCGAACAGAAGGCGATGATCAATGCTATGGATTCCAAGATCAGATCCCTGGAA
CAGAGGATTGTGGAATTGTCTGAAGCCAATAAACTTGCAGCAAACAGCAGCCTTTTTACC
CAAAGGAACATGAAGGCCCAAGAAGAGATGATTTCTGAACTCAGGCAACAGAAATTTTAC
CTGGAGACACAGGCTGGGAAATTGGAGGCCCAGAACCGAAAGCTGGAGGAGCAGCTGGAG
AAGATTAGCCACCAAGATCATAGTGACAAGAATCGGCTGCTGGAACTGGAGACAAGATTG
CGGGAGGTCAGTCTAGAGCATGAGGAACAAAAGCTGGAGCTCAAGCGCCAGCTCACAGAG
CTACAGCTCTCCCTGCAGGAGCGCGAGTCGCAGTTGACAGCGCTGCAGGCTGCCCGGGCG
GCCTTGGAGAGCCAGCTTCGCCAGGCGAAGACAGAGCTGGAAGAGACCACAGCGGAAGCT
GAAGAGGAGATCCAGGCACTCACGGCACATAGAGATGAAATCCAGCGCAAATTTGATGCT
CTTCGTAACAGCTGTACTGTCATCACAGACCTGGAGGAGCAGCTAAACCAGCTGACTGAG
GACAATGCTGAACTCAACAACCAAAACTTCTATTTGTCCAAACAACTCGATGAGGCTTCT
GGCGCCAACGATGAGATTGTACAACTGCGAAGTGAAGTGGACCACCTCCGCCGGGAGATC
ACAGAACGAGAGATGCAACTCACCAGCCAGAAGCAAACGATGGAGGCTCTGAAGACCACA
TGCACCATGCTGGAGGAACAGGTCATGGATTTGGAAGCCCTAAACGACGAGCTGCTAGAA
AAAGAGCGGCAGTGGGAGGCCTGGAGGAGCGTCCTGGGTGACGAGAAATCCCAGTTTGAG
TGTCGGGTTCGAGAGCTGCAGAGGATGTTGGACACCGAGAAACAGAGCAGGGCGAGAGCT
GATCAGCGGATCACCGAGTCTCGCCAGGTGGTGGAGCTGGCAGTGAAGGAGCACAAGGCC
GAGATTCTTGCTCTGCAGCAGGCCCTCAAAGAGCAGAAGCTGAAGGCTGAGAGCCTCTCT
GACAAGCTCAATGACCTGGAGAAGAAGCATGCTATGCTTGAAATGAATGCCCGAAGCTTA
CAGCAGAAGCTGGAGACGGAACGAGAGCTCAAACAGAGGCTTCTGGAGGAGCAAGCCAAA
TTACAGCAGCAGATGGACCTGCAGAAAAACCACATTTTCCGTCTGACTCAAGGACTGCAA
GAAGCTCTAGATCGGGCTGATCTGCTGAAGACAGAAAGAAGTGACCTGGAGTATCAGCTA
GAAAACATTCAGGTTCTCTATTCTCATGAAAAGGTGAAAATGGAAGGCACTATTTCTCAA
CAAACCAAACTCATTGATTTTCTGCAAGCCAAAATGGACCAACCTGCTAAAAAGAAAAAG
GGTTTATTTAGTCGACGGAAAGAGGACCCTGCTTTGCCCACACAGGTTCCTCTGCAGTAC
AATGAGCTGAAGCTGGCCCTGGAGAAGGAGAAAGCTCGCTGTGCAGAGCTAGAGGAAGCC
CTTCAGAAGACCCGCATTGAGCTCCGGTCTGCCCGGGAGGAAGCTGCCCACCGCAAAGCG
ACGGACCACCCACACCCATCCACGCCAGCCACCGCGAGGCAGCAGATCGCCATGTCCGCC
ATCGTGCGGTCGCCCGAGCACCAGCCCAGTGCCATGAGCCTGCTGGCCCCGCCGTCGAGC
CGGAGAAAGGAGTCTTCAACTCCAGAGGAATTTAGTCGGCGTCTCAAGGAACGCATGCAC

CACAATATTCCTCACCGATTCAATGTAGGACTGAACATGCGAGCCACAAAGTGTGCTGTT
TGTTTGGATACCGTGCACTTTGGACGCCAGGCATCCAAATGTCTTGAATGTCAAGTGATG
TGTCACCCCAAGTGCTCCACGTGCTTGCCAGCCACCTGCGGCCTGCCTGCTGAGTACGCC
ACACACTTCACCGAGGCCTGCCGTGACAAAATGAACTCCCCGGGTCTCCAGACCAAGGAG
CCTGGCAGCAGCTTGCACCTGGAAGGGTGGATGAAGGTGCCCAGGAATAACAAACGAGGA
CAGCAAGGCTGGGACAGGAAGTACATTGTCCTGGAGGGATCAAAAGTCCTCATTTATGAC
AGTGAAGCCAGAGAAGCTGGACAGAGGCCGGTGGAAGAATTTGAGCTGTGCCTTCCTGAT
GGGGATGTATCTATTCATGGTGCCGTTGGTGCTTCCGAACTCGCAAATACAGCCAAAGCA
GATGTCCCATACATACTGAAGATGGAATCTCACCCGCACACCACCTGCTGGCCTGGGAGA
ACCCTCTACTTGCTAGCTCCTAGCTTCCCCGACAAACAGCGCTGGGTCACCGCCTTAGAA
TCAGTTGTCGCAGGTGGGAGAGTTTCTAGGGAAAAAGCAGAAGCTGACGCTAAATTGCTT
GGAAACTCCCTGCTGAAACTGGAAGGTGATGACCGTCTAGACATGAACTGCACACTGCCC
TTCAGTGACCAGGTGGTGTTGGTGGGCACCGAGGAAGGGCTCTATGCCCTGAATGTCTTG
AAAAACTCCCTAACCCACGTCCCAGGAATTGGAGCAGTCTTCCAAATTTATATTATCAAG
GACCTGGAGAAGCTACTCATGATAGCAGGAGAAGAACGGGCACTGTGTCTTGTTGATGTG
AAGAAGGTGAAGCAGTCCCTAGCGCAGTCCCACCTTCCAGCCCAGCCAGACATCTCACCC
AACATTTTCGAGGCTGTGAAGGGCTGCCACTTGTTTGGGGCTGGCAAGATTGAGAACGGG
CTCTGCATCTGTGCAGCCATGCCCAGCAAAGTCGTCATTCTCCGCTACAATGAAAACCTC
AGCAAATATTGCATTCGGAAAGAGATAGAGACCTCAGAGCCCTGCAGCTGTATCCACTTC
ACCAATTACAGTATCCTCATCGGAACCAATAAATTCTATGAAATCGACATGAAGCAGTAC
ACACTTGAGGAATTCCTGGATAAGAATGATCATTCCTTGGCACCTGCTGTGTTTGCTGCC
TCTTCCAACAGCTTCCCTGTCTCGATCGTGCAGGTGAACGGCGCAGGGCAGCGAGAGGAG
TACCTGCTGTGTTTCCACGAATTTGGAGTGTTCGTGGATTCTTACGGAAGACGTAGCCGG
ACAGATGATCTCAAGTGGAGTCGCTTACCTTTGGCCTTCGCCTACAGAGAACCCTATCTC
TTTGTGACCCACTTCAACTCACTCGAAGTAATTGAGATCCAGGCACGCTCCTCTGCAGGG
ACCCCTGCCCGAGCGTATCTGGACATCCCAAACCCGCGCTACCTGGGCCCTGCGATCTCC
TCGGGAGCGATTTACCTGGCGTCCTCATACCAGGATAAATTACGGGTCATTTGCTGCAAG
GGAAACCTCGTGAAGGAGTCCGGCACTGAACACCACCGGGGCCCTTCCACCTCCCGCAGC
AGCCCCAACAAGCGAGGCCCGCCCACATACAATGAGCACATCACCAAGCGTGTGGCCTCC
AGCCCAGCGCCGCCCGAAGGCCCCAGCCACCCCCGAGAGCCAAGCACACCCCACCGGTAC
CGTGAGGGGCGGACCGAGCTGCGCAGGGACAAGTCTCCTGGCCGCCCCCTGGAGCGGGAG
AAGTCCCCGGGCCGGATGCTCAGCACGCGGAGAGAGCGGTCTCCTGGGAGGCTGTTTGAA
GACAGCAGCAGGGGCCGGCTGCCTGCGGGAGCTGTGAGGACCCCGCTGTCCCAGGTTAAC
AAGGTCTGGGACCAGTCTTCAGTA
>Papio anubis XM_003907237.4
ATGTTGAAGTTCAAATATGGAGCGCGGAATCCTTTGGATGCTGGTGCTGCTGAACCCATTGCCAGCCGGG
CCTCCAGGCTGAATCTATTCTTCCAGGGGAAACCACCCTTTATGACTCAACAGCAGATGTCTCCTCTTTC
CCGAGAAGGGATATTAGATGCCCTCTTTGTTCTCTTTGAAGAATGCAGTCAGCCTGCTCTGATGAAGATT
AAGCATGTGAGCAACTTTGTCCGGAAGTATTCCGACACCATAGCTGAGTTACAGGAGCTCCAGCCTTCGG
CAAAGGACTTTGAAGTCAGAAGTCTTGTAGGTTGTGGTCACTTTGCTGAAGTGCAGGTGGTGAGAGAGAA
GGCAACCGGGGACATCCATGCTATGAAAGTGATGAAGAAGAAGGCTTTATTGGCCCAGGAGCAGGTTTCA
TTTTTTGAGGAAGAGCGGAACATACTATCTCGAAGCACAAGCCCGTGGATCCCCCAATTACAGTATGCCT
TTCAGGACAAAAATCACCTTTATCTGGTCATGGAATATCAGCCTGGAGGGGACTTGCTGTCACTTTTGAA
TAGATATGAGGACCAGTTAGATGAAAACCTGATTCAGTTTTACCTAGCTGAACTGATTTTGGCTGTTCAC
AGTGTTCATCAGATGGGATATGTGCATCGAGACATCAAGCCCGAGAACATTCTCATTGACCGCACAGGAC
ACATCAAGCTGGTGGATTTTGGATCTGCTGCAAAAATGAATTCAAACAAGATGGTGAATGCCAGACTCCC
AATTGGGACCCCGGATTACATGGCTCCTGAAGTGCTGACTGTGATGAATGCGGACGGAAAAGGCACCTAC

GGCCTGGAATGTGACTGGTGGTCAGTGGGCGTGATTGCCTATGAGATGATTTATGGAGATCCCCCTTCG
CAGAGGGAACCTCTGCCAGAACCTTCAATAACATCATGAATTTCCAGCGGTTTTTGAAGTTTCCAGATGA
CCCCAAAGTGAGCAGTGACTTTCTTGATCTGATTCAAAGTTTGTTGTGTGGCCAGAAAGAGAGACTGAAG
TTTGAAGGTCTCTGCTGCCATCCTTTCTTCTCTAAAATCGACTGGAATAACATTCGTAACTCTCCTCCCC
CCTTCGTTCCCACCCTCAAGTCTGACGATGACACCTCCAATTTTGATGAACCAGAGAAGAATTCGTGGGT
TTCATCCTCTCCGTGCCAGCTGAGCCCCTCAGGTTTCTTGGGTGAAGAACTGCCGTTTGTGGGGTTTTCG
TACAGCAAGGCACTGGGGATTCTTGGTAGATCTGAGTCTGTTGTGTCGGGTCTGGACTCCCCTGCCAAGA
CTAGCTCCATGGAAAAGAAACTTCTCATCAAAAGCAAAGAGCTACAAGACTCTCAGGACAAGTGTCACAA
GATGGAGCAGGAAATGACCCGGTTACATCGGAGAGTGTCAGAGGTGGAGGCTGTGCTTAGTCAGAAGGAG
GTGGAGCTGAAGGCCTCTGAGACTCAGAGATCCCTCCTGGAGCAGGACCTTGCTACCTACATCACAGAAT
GCAGTAGCTTAAAGCGAAGTTTGGAGCAAGCACGGATGGAGGTGTCCCAGGAGGATGACAAAGCACTGCA
GCTTCTCCATGATATCAGAGAGCAGAGCCGGAAGCTCCAAGAAATCAAAGAGCAGGAGTACCAGGCTCAA
GTGGAAGAAATGAGGTTGATGATGAATCAGTTGGAAGAGGACCTTGTCTCAGCAAGAAGACGGAGTGATC
TCTACGAATCTGAGCTGAGAGAGTCTCGGCTCGCTGCTGAAGAATTCAAGCGGAAAGCGACAGAATGTCA
GCATAAACTGTTGAAGGCTAAGGATCAAGGGAAGCCTGAAGTGGGAGAATATGCGAAACTGGAGAAGATC
AATGCTGAGCAGCAGCTCAAAATTCAGGAGCTCCAAGAGAAACTGGAGAAGGCTGTAAAAGCCAGCACCG
AGGCCACCGAGCTGCTGCAAAATATCCGCCAGGCAAAGGAGCGAGCCGAGAGGGAGCTGGAGAAGCTGCA
GAACCGAGAGGATTCTTCTGAAGGCATTAGAAAGAAGCTGGTGGAAGCCGAGGAACGTCGCCATTCTCTG
GAGAACAAGGTAAAGAGACTAGAGACCATGGAGCGTAGAGAAAACAGACTGAAGGATGACATCCAGACAA
AATCCCAACAGATCCAGCAGATGGCTGATAAAATTCTGGAACTCGAAGAGAAACATCGGGAGGCCCAAGT
CTCAGCCCAGCACCTAGAAGTGCACCTGAAACAGAAAGAGCAGCACTATGAGGAAAAGATTAAAGTGTTG
GACAATCAGATAAAGAAAGACCTGGCCGACAAGGAGACGCTGGAGAACATGATGCAGAGACACGAGGAGG
AGGCCCATGAGAAGGGCAAAATTCTCAGCGAACAGAAGGCGATGATCAATGCTATGGATTCCAAGATCAG
ATCCCTGGAACAGAGGATTGTGGAACTGTCTGAAGCCAATAAACTTGCAGCAAATAGCAGTCTTTTTACC
CAAAGGAACATGAAGGCTCAAGAAGAGATGATTTCTGAACTCAGGCAACAGAAATTTTACCTGGAGACCC
AGGCTGGGAAATTGGAGGCCCAGAACCGAAAGCTGGAGGAGCAGCTGGAGAAGATCAGCCACCAAGACCA
CAGTGACAAGAATCGGCTGCTGGAACTGGAGACAAGACTGCGGGAGGTCAGTCTAGAGCACGAGGAGCAG
AAACTGGAGCTCAAGCGCCAGCTCACAGAGCTACAGCTCTCCCTGCAGGAGCGTGAGTCACAGTTGACAG
CCCTGCAGGCTGCCCGGGCGGCCCTGGAAAGCCAGCTTCGCCAGGCAAAGACAGAGCTGGAAGAGACCAC
AGCAGAAGCCGAAGAGGAGATCCAGGCGCTCACGGCACATAGAGATGAAATCCAGCGCAAATTTGATGCT
CTTCGTAACAGCTGTACTGTAATCACAGACCTGGAGGAGCAGCTAAACCAGCTGACCGAGGATAACGCTG
AACTCAACAACCAAAACTTCTACTTGTCCAAACAACTTGATGAGGCTTCCGGCGCCAACGATGAGATTGT
ACAACTGCGAAGTGAAGTGGACCACCTTCGCCGGGAGATCACAGAACGAGAGATGCAGCTTACCAGCCAG
AAGCAAACGATGGAGGCTCTGAAGACCACGTGTACCATGCTGGAGGAACAGGTCATGGATTTGGAGGCCC
TAAACGACGAGCTGCTAGAAAAAGAGCGGCAGTGGGAGGCCTGGAGGAGTGTCCTGGGTGACGAGAAATC
CCAGTTTGAGTGTCGGGTTCGAGAGCTGCAGAGGATGTTGGACACCGAGAAACAGAGCAGGGCGAGAGCC
GATCAGCGGATCACCGAGTCTCGCCAGGTGGTGGAGCTGGCAGTGAAGGAGCACAAGGCGGAGATTCTCG
CTCTGCAGCAGGCTCTCAAAGAGCAGAAGCTGAAGGCCGAGAGCCTCTCTGACAAGCTCAATGACCTGGA
GAAGAAGCATGCTATGCTTGAAATGAATGCCCGAAGCTTACAGCAGAAGCTGGAGACTGAACGAGAGCTC
AAACAGAGGCTTCTGGAAGAGCAAGCCAAATTACAGCAGCAGATGGACCTGCAGAAAAATCACATTTTCC
GTCTGACTCAAGGACTGCAAGAAGCTCTAGATCGGGCTGATCTGCTGAAGACAGAAAGAAGTGACCTGGA
GTATCAGCTGGAAAACATTCAGGTTCTCTATTCTCATGAAAAGGTGAAAATGGAAGGCACTATTTCTCAA
CAAACCAAACTCATCGATTTTCTGCAAGCCAAAATGGACCAACCTGCTAAAAAGAAAAAGGGTTTATTTA
GTCGACGGAAAGAGGACCCTGCTTTGCCCACACAGGTTCCTCTGCAGTACAATGAACTGAAGCTGGCCCT
GGAGAAGGAGAAAGCTCGCTGTGCAGAGCTAGAGGAAGCCCTTCAGAAGACCCGCATCGAGCTTCGGTCC
GCCCGGGAGGAAGCTGCCCACCGCAAAGCGACGGACCACCCACACCCATCCACGCCAGCCACCGCGAGGC
AGCAGATCGCCATGTCCGCCATCGTGCGGTCACCAGAGCACCAGCCCAGTGCCATGAGCCTGCTGGCCCC

GCCATCCAGCCGCAGAAAGGAGTCTTCAACTCCAGAGGAATTTAGTCGGCGTCTTAAGGAACGCATGCAC
CACAATATTCCTCACCGATTCAACGTAGGACTGAACATGCGAGCCACAAAGTGTGCTGTGTGTCTGGATA
CCGTGCACTTTGGACGCCAAGCATCCAAATGTCTCGAATGTCAGGTGATGTGTCACCCCAAGTGCTCCAC
GTGCTTGCCAGCCACCTGCGGCCTGCCTGCCGAATACGCCACACACTTCACTGAGGCCTTCTGCCGCGAC
AAAATGAACTCCCCGGGTCTCCAGACCAAGGAGCCCAGCAGCAGCTTGCACCTGGAAGGGTGGATGAAGG
TGCCCAGGAATAACAAACGAGGACAGCAAGGCTGGGACAGGAAGTACATTGTCCTGGAGGGATCAAAAGT
CCTTATTTATGACAATGAAGCCAGAGAAGCTGGACAGAGGCCGGTGGAAGAATTTGAGCTGTGCCTTCCC
GACGGGGATGTATCTATTCATGGTGCCGTTGGTGCTTCCGAACTCGCAAATACAGCCAAAGCAGATGTCC
CATACATACTGAAGATGGAATCTCACCCGCACACCACCTGCTGGCCCGGGAGAACCCTCTACTTGCTAGC
TCCCAGCTTCCCTGACAAACAGCGCTGGGTCACCGCCTTAGAATCAGTTGTCGCAGGTGGGAGAGTTTCT
AGGGAAAAAGCAGAAGCCGATGCTAAATTGCTTGGAAACTCCCTGCTGAAACTGGAAGGTGATGACCGTC
TAGACATGAACTGCACACTGCCCTTCAGTGACCAGGTGGTGTTGGTGGGCACTGAGGAAGGGCTCTACGC
CCTGAATGTCTTGAAAAACTCCCTAACGCACGTCCCAGGAATTGGAGCAGTCTTCCAAATTTATATTATC
AAGGACCTGGAGAAGCTACTCATGATAGCAGGAGAAGAGCGGGCACTGTGTCTTGTGGACGTGAAGAAAG
TGAAGCAGTCCCTGGCGCAGTCCCACCTGCCCGCCCAGCCCGACATCTCACCCAACATTTTTGAAGCTGT
CAAGGGCTGCCACTTGTTTGGGGCTGGCAAGATTGAGAACGGGCTCTGCATCTGTGCAGCCATGCCCAGC
AAAGTCGTCATTCTCCGCTACAATGAAAACCTCAGCAAATACTGCATTCGGAAAGAGATAGAGACCTCAG
AGCCTTGCAGCTGTATCCACTTCACCAATTACAGTATCCTCATTGGAACCAATAAATTCTACGAAATCGA
CATGAAGCAGTACACCCTGGAGGAATTCCTGGATAAGAATGACCATTCCTTGGCACCTGCTGTGTTTGCC
GCCTCTTCCAACAGCTTCCCTGTCTCTATCGTGCAGGTGAACAGTGCAGGGCAGCGAGAGGAGTACTTGC
TATGTTTCCACGAGTTTGGAGTGTTCGTGGATTCTTACGGAAGACGTAGCCGCACAGATGACCTCAAGTG
GAGTCGCTTACCTTTGGCCTTTGCCTACAGAGAACCCTATCTGTTTGTGACACACTTCAACTCACTCGAA
GTAATTGAGATCCAGGCACGCTCCTCGGCAGGGACCCCTGCCCGAGCGTACCTGGACATCCCGAACCCAC
GCTACCTGGGCCCTGCCATTTCCTCGGGAGCGATTTACTTGGCATCCTCATACCAGGATAAATTAAGGGT
CATTTGCTGCAAGGGAAACCTCGTGAAGGAGTCTGGCACTGAACACCACCGGGGCCCGTCCACCTCCCGC
AGCAGCCCCAACAAGCGAGGCCCGCCCACGTACAACGAGCACATCACCAAGCGCGTGGCCTCCAGCCCGG
CGCCGCCCGAAGGCCCCAGCCACCCCCGAGAGCCAAGCACACCCCACCGCTACCGCGAGGGGCGGACCGA
GCTGCGCAGGGACAAGTCTCCTGGCCGCCCCCTGGAGCGGGAGAAGTCCCCGGGCCGGATGCTCAGCACG
CGGAGAGAGCGGTCCCCTGGGAGGCTGTTTGAAGACAGCAGCAGGGGCCGGCTGCCTGCGGGAGCCGTGA
GGACCCCGCTGTCCCAGGTGAACAAGGTCTGGGACCAGTCTTCAGTA
>Chlorocebus sabaeus XM_008004908.1
ATGTTAAAGTTCAAATATGGAGCGCGGAATCCTTTGGATGCTGGTGCTGCTGAACCCATTGCC
AGCCGGGCCTCCAGGCTGAATCTATTCTTCCAGGGGAAACCACCCTTTATGACTCAACAGCAGATGTCTC
CTCTTTCCCGAGAAGGGATATTAGATGCCCTCTTTGTTCTCTTTGAAGAATGCAGTCAGCCTGCTCTGAT
GAAGATTAAGCATGTGAGCAACTTTGTCCGGAAGTATTCCGACACCATAGCTGAGTTACAGGAGCTCCAG
CCTTCGGCAAAGGACTTTGAAGTCAGAAGTCTTGTAGGTTGTGGTCACTTTGCTGAAGTGCAGGTGGTGA
GAGAGAAAGCAACCGGGGACATCCATGCTATGAAAGTGATGAAGAAGAAGGCTTTATTGGCCCAGGAGCA
GGTTTCATTTTTTGAGGAAGAGCGGAACATACTATCTCGAAGCACAAGCCCGTGGATCCCCCAATTACAG
TATGCCTTTCAGGACAAAAATCACCTTTATCTGGTCATGGAATATCAGCCTGGAGGGGACTTGCTGTCAC
TTTTGAATAGGTATGAGGACCAGTTAGATGAAAACCTGATTCAGTTTTACCTAGCTGAACTGATTTTGGC
TGTTCACAGTGTTCATCAGATGGGATATGTGCATCGAGACATCAAGCCCGAGAACATTCTCATTGACCGC
ACAGGACACATCAAGCTGGTGGATTTTGGATCTGCTGCAAAAATGAATTCAAACAAGATGGTGAATGCCA
GACTCCCAATTGGGACCCCGGATTACATGGCTCCTGAAGTGCTGACTGTGATGAATGCGGACGGAAAAGG
CACCTACGGCCTGGACTGTGACTGGTGGTCAGTGGGCGTGATTGCCTATGAGATGATTTATGGGAGATCC
CCCTTCGCAGAGGGAACCTCTGCCAGAACCTTCAATAACATCATGAATTTCCAGCGGTTTTTGAAGTTTC
CAGATGACCCCAAAGTGAGCAGTGACTTTCTTGATCTGATTCAAAGTTTGTTGTGTGGCCAGAAAGAGAG
ACTGAAGTTTGAAGGTCTTTGCTGCCATCCTTTCTTCTCTAAAATCGACTGGAATAACATTCGTAACTCT

CCTCCCCCCTTCGTTCCCACCCTCAAGTCTGACGATGACACCTCCAATTTTGATGAACCAGAGAAGAATT
CGTGGGTTTCATCCTCTCCGTGCCAGCTGAGCCCCTCAGGTTTCTCGGGTGAAGAACTGCCGTTTGTGGG
GTTTTCGTACAGCAAGGCACTGGGGATTCTTGGTAGATCTGAGTCTGTTGTGTCGGGTCTGGACTCCCCT
GCCAAGACTAGCTCCATGGAAAAGAAACTTCTCATCAAAAGCAAAGAGCTACAAGACTCTCAGGACAAGT
GTCACAAGATGGAGCAGGAAATGACCCGGTTACATCGGAGAGTGTCAGAGGTGGAGGCTGTGCTTAGTCA
GAAGGAGGTGGAGCTGAAGGCCTCTGAGACTCAGAGATCCCTCCTGGAGCAGGACCTTGCTACCTACATC
ACAGAATGCAGTAGCTTAAAGCGAAGTTTGGAGCAAGCACGGATGGAGGTGTCCCAGGAGGATGACAAAG
CACTGCAGCTTCTCCATGATATCAGAGAGCAGAGCCGGAAGCTCCAAGAAATCAAAGAGCAGGAGTACCA
GGCTCAAGTGGAAGAAATGAGGTTGATGATGAATCAGTTGGAAGAGGACCTTGTCTCAGCAAGAAGACGG
AGTGATCTCTACGAATCTGAGCTGAGAGAGTCTCGGCTCGCTGCTGAAGAATTCAAGCGGAAAGCGACAG
AATGTCAGCATAAACTGTTGAAGGCTAAGGATCAAGGGAAGCCTGAAGTGGGAGAATATACGAAACTGGA
GAAGATCAATGCTGAGCAGCAGCTCAAAATTCAGGAGCTCCAAGAGAAACTGGAGAAGGCTGTAAAAGCC
AGCACCGAGGCCACTGAGCTGCTGCAGAATATCCGCCAGGCAAAGGAGCGAGCCGAGAGGGAGCTGGAGA
AGCTGCAGAACCGAGAGGATTCTTCTGAAGGCATCAGAAAGAAGCTGGTGGAAGCCGAGGAACGTCGCCA
TTCTCTGGAGAACAAGGTAAAGAGACTAGAGACCATGGAGCGTAGAGAAAACAGACTGAAGGATGACATC
CAGACAAAATCCCAACAGATCCAGCAGATGGCTGATAAAATTCTGGAACTCGAAGAGAAACATCGGGAGG
CCCAAGTCTCAGCCCAGCACCTAGAAGTGCACCTGAAACAGAAAGAGCAGCACTACGAGGAAAAGATTAA
AGTGTTGGACAATCAGATAAAGAAAGACCTGGCCGACAAGGAGACGCTGGAGAACATGATGCAGAGACAC
GAGGAGGAGGCCCATGAGAAGGGCAAAATTCTCAGCGAACAGAAGGCGATGATCAATGCTATGGATTCCA
AGATCAGATCCCTGGAACAGAGGATTGTGGAACTATCTGAAGCCAATAAACTTGCAGCAAATAGCAGTCT
TTTTACCCAAAGGAACATGAAGGCTCAAGAAGAGATGATTTCTGAACTCAGGCAACAGAAATTTTACCTG
GAGACCCAGGCTGGGAAATTGGAGGCCCAGAACCGAAAGCTGGAGGAGCAGCTGGAGAAGATCAGCCACC
AAGACCACAGTGACAAGAATCGGCTGCTGGAACTGGAGACAAGACTGCGGGAGGTCAGTCTAGAGCACGA
GGAGCAGAAACTGGAGCTCAAGCGCCAGCTCACAGAGCTACAGCTCTCCCTGCAGGAGCGTGAGTCACAG
TTGACAGCCCTGCAGGCTGCCCGGGCGGCCCTGGAAAGCCAGCTTCGCCAGGCAAAGACAGAGCTGGAAG
AGACCACGGCAGAAGCTGAAGAGGAGATCCAGGCGCTCACGGCACATAGAGATGAAATCCAGCGCAAATT
TGATGCTCTTCGTAACAGCTGTACTGTAATCACAGACCTGGAGGAGCAGCTAAACCAGCTGACCGAGGAT
AACGCCGAACTCAACAACCAAAACTTCTACTTGTCCAAACAACTCGATGAGGCTTCCGGCGCCAACGATG
AGATTGTACAACTGCGAAGTGAAGTGGACCACCTTCGCCGGGAGATCACAGAACGAGAGATGCAGCTTAC
CAGCCAGAAGCAAACGATGGAGGCTCTGAAGACCACGTGTACCATGCTGGAGGAACAGGTCATGGATTTG
GAGGCCCTAAACGACGAGCTGCTAGAAAAAGAGCGGCAGTGGGAGGCCTGGAGGAGCGTCCTGGGTGACG
AGAAATCCCAGTTTGAGTGTCGGGTTCGAGAGCTGCAGAGGATGTTGGACACCGAGAAACAGAGCAGGGC
GAGAGCCGATCAGCGGATCACCGAGTCTCGCCAGGTTGTGGAGCTGGCAGTGAAGGAGCACAAGGCGGAG
ATTCTCGCTCTGCAGCAGGCTCTCAAAGAGCAGAAGCTGAAGGCCGAGAGCCTCTCTGACAAGCTCAATG
ACCTGGAGAAGAAGCATGCTATGCTTGAAATGAATGCCCGAAGCTTACAGCAGAAGCTGGAGACTGAACG
AGAGCTCAAACAGAGGCTTCTGGAAGAGCAAGCCAAATTACAGCAGCAGATGGACCTGCAGAAAAATCAC
ATTTTCCGTCTGACTCAAGGACTGCAAGAAGCTCTAGATCGGGCTGATCTGCTGAAGACAGAAAGAAGTG
ACCTGGAGTATCAGCTGGAAAACATTCAGGTTCTCTATTCTCATGAAAAGGTGAAAATGGAAGGCACTAT
TTCTCAACAAACCAAACTCATCGATTTTCTGCAAGCCAAAATGGACCAACCTGCTAAAAAGAAAAAGGGT
TTATTTAGTCGACGGAAAGAGGACCCTGCTTTGCCCACACAGGTTCCTCTGCAGTACAATGAGCTGAAGC
TGGCCCTGGAGAAGGAGAAAGCTCGCTGTGCAGAGCTAGAGGAAGCCCTTCAGAAGACCCGCATCGAGCT
CCGGTCCGCCCGGGAGGAAGCTGCCCACCGCAAAGCGACGGACCACCCACACCCATCCACGCCAGCCACC
GCGAGGCAGCAGATCGCCATGTCCGCCATCGTGCGGTCACCAGAGCACCAGCCCAGTGCCATGAGCCTGC
TGGCCCCGCCATCCAGCCGCAGAAAGGAGTCTTCAACTCCAGAGGAATTTAGTCGGCGTCTTAAGGAACG
CATGCACCACAATATTCCTCACCGATTCAACGTAGGACTGAACATGCGAGCCACAAAGTGTGCTGTGTGT
CTGGATACCGTGCACTTTGGACGCCAAGCATCCAAATGTCTCGAATGTCAGGTGATGTGTCACCCCAAGT
GCTCCACGTGCTTGCCAGCCACCTGCGGCCTGCCTGCCGAATACGCCACACACTTCACTGAGGCCTTCTG

CCGCGACAAAATGAACTCCCCGGGTCTCCAGACCAAGGAGCCCAGCAGCAGCTTGCACCTGGAAGGGTGG
ATGAAGGTGCCCAGGAATAACAAACGAGGACAGCAAGGCTGGGACAGGAAGTACATTGTCCTGGAGGGAT
CAAAAGTCCTTATTTATGACAATGAAGCCAGAGAAGCTGGACAGAGGCCGGTGGAAGAATTTGAGCTGTG
CCTTCCCGACGGGGATGTATCTATTCATGGTGCCGTTGGTGCTTCCGAACTCGCAAATACAGCCAAAGCA
GATGTCCCATACATACTGAAGATGGAATCTCACCCGCACACCACCTGCTGGCCCGGGAGAACCCTCTACT
TGCTAGCTCCCAGCTTCCCTGACAAACAGCGCTGGGTCACCGCCTTAGAATCAGTTGTCGCAGGTGGGAG
AGTTTCTAGGGAAAAAGCAGAAGCCGATGCTAAATTGCTTGGAAACTCCCTGCTGAAACTGGAAGGTGAT
GACCGTCTAGACATGAACTGCACACTGCCCTTCAGTGACCAGGTGGTGTTGGTGGGCACCGAGGAAGGGC
TCTACGCCCTGAATGTCTTGAAAAACTCCCTAACGCACGTCCCAGGAATTGGAGCAGTCTTCCAAATTTA
TATTATCAAGGACCTGGAGAAGCTACTCATGATAGCAGGAGAAGAGCGGGCACTGTGTCTTGTGGACGTG
AAGAAAGTGAAACAGTCCCTGGCGCAGTCCCACCTGCCCGCCCAGCCCGACATCTCACCCAACATTTTTG
AAGCTGTCAAGGGCTGCCACTTGTTTGGGGCTGGCAAGATTGAGAACGGGCTCTGCATCTGTGCAGCCAT
GCCCAGCAAAGTCGTCATTCTCCGCTACAACGAAAACCTCAGCAAATACTGCATTCGGAAAGAGATAGAG
ACCTCAGAGCCTTGCAGCTGTATCCACTTCACCAATTACAGTATCCTCATTGGAACCAATAAATTCTACG
AAATCGACATGAAGCAGTACACGCTGGAGGAATTCCTGGATAAGAATGACCATTCCTTGGCACCTGCTGT
GTTTGCCGCCTCTTCCAACAGCTTCCCTGTCTCGATCGTGCAGGTGAACAGTGCAGGGCAGCGAGAGGAG
TACTTGCTGTGTTTCCACGAGTTTGGAGTGTTCGTGGATTCTTATGGAAGACGTAGCCGCACAGACGACC
TCAAGTGGAGTCGCTTACCTTTGGCCTTTGCCTACAGAGAACCCTATCTGTTTGTGACCCACTTCAACTC
ACTCGAAGTAATTGAGATCCAGGCACGCTCCTCGGCAGGGACCCCTGCCCGAGCGTACCTGGACATCCCG
AACCCACGCTACCTGGGCCCTGCCATTTCCTCGGGAGCGATTTACTTGGCATCCTCTTACCAGGATAAAT
TAAGGGTCATTTGCTGCAAGGGAAACCTCGTGAAGGAGTCTGGCACTGAACACCACCGGGGCCCGTCCAC
CTCCCGCAGCAGCCCCAACAAGCGAGGCCCGCCCACGTACAACGAGCACATCACCAAGCGCGTGGCCTCC
AGCCCGGCGCCGCCCGAAGGCCCCAGCCACCCCCGAGAGCCAAGCACACCCCACCGCTACCGCGAGGGGC
GGACCGAGCTGCGCAGGGACAAGTCTCCTGGCCGCCCCCTGGAGCGGGAGAAGTCCCCGGCCGGATGCT
CAGCACGCGGAGAGAGCGGTCCCCCGGGAGACTGTTTGAAGACAGCAGCAGGGGCCGGCTGCCTGCGGGA
GCCGTGAGGACCCCGCTGTCCCAGGTGAACAAGGTCTGGGACCAGTCTTCAGTA
>Rhinopithecus roxellana XM_010368276.1
ATGTTGAAGTTCAAATATGGAGCGCGGAATCCTTTGGATGCTGGTGCTGCTGAACCCATTGCCAGCCGGG
CCTCCAGGCTGAATCTGTTCTTCCAGGGGAAACCACCCTTTATGACTCAACAGCAGATGTCTCCTCTTTC
CCGAGAAGGGATATTAGATGCCCTCTTTGTTCTCTTTGAAGAGTGCAGTCAGCCTGCTCTGATGAAGATT
AAGCATGTGAGCAACTTTGTCCGGAAGTATTCCGACACCATAGCTGAGTTACAGGAGCTCCAGCCTTCAG
CAAAGGACTTTGAAGTCAGAAGTCTTGTAGGTTGTGGTCACTTTGCTGAAGTGCAGGTGGTGAGAGAGAA
GGCAACCGGGGACATCCATGCTATGAAAGTGATGAAGAAGAAGGCTTTATTGGCCCAGGAGCAGGTTTCA
TTTTTTGAGGAAGAGCGGAACATACTCTCTCGAAGCACAAGCCCGTGGATCCCCCAATTACAGTATGCCT
TTCAGGACAAAAATCACCTTTATCTGGTCATGGAATATCAGCCTGGAGGGGACTTGCTGTCACTTTTGAA
TAGATATGAGGACCAGTTAGATGAAAATCTGATTCAGTTTTACCTAGCTGAACTGATTTTGGCTGTTCAC
AGTGTTCATCAGATGGGATATGTGCATCGAGACATCAAGCCCGAGAACATTCTCATTGACCGCACAGGAC
ACATCAAGCTGGTGGATTTTGGATCCGCTGCAAAAATGAATTCAAACAAGATGGTGAATGCCAAACTCCC
AATTGGGACCCCGGATTACATGGCTCCTGAAGTGCTGACTGTGATGAATGCGGACGGAAAAGGCACCTAC
GGCCTGGACTGTGACTGGTGGTCAGTGGGCGTGATTGCCTATGAGATGATTTATGGGAGATCCCCCTTCT
CAGAGGGAACCTCTGCCAGAACCTTCAATAACATCATGAATTTCCAGCGGTTTTTGAAGTTTCCAGATGA
CCCCAAAGTGAGCAGTGACTTTCTTGATCTGATTCAAAGTTTGTTGTGTGGCCAGAAAGAGAGACTGAAG
TTTGAAGGTCTTTGCTGCCATCCTTTCTTCTCTAAAATCGACTGGAATAACATTCGTAACTCTCCTCCCC
CCTTCGTTCCCACCCTCAAGTCTGACGATGACACCTCCAATTTTGATGAACCAGAGAAGAATTCGTGGGT
TTCATCCTCTCGGTGCCAGCTGAGCCCCTCAGGTTTCTCGGGTGAAGAACTGCCGTTTGTGGGGTTTTCG
TACAGCAAGGCACTGGGGATTCTTGGTAGATCTGAGTCTGTCCTGTCGGGTCTGGACTCCCCTGCCAAGA
CTAGCTCCATGGAAAAGAAACTTCTCATCAAAAGCAAAGAGCTACAAGACTCTCAGGACAAGTGTCACAA

GATGGAGCAGGAAATGACCCGGTTACATCGGAGAGTGTCAGAGGTGGAGGCTGTGCTTAGTCAGAAGGAG
GTGGAGCTGAAGGCCTCTGAGACTCAGAGATCCCTCCTGGAGCAGGACCTTGCTACCTACATCACAGAAT
GCAGTAGCTTAAAGCGAAGTTTGGAGCAAGCACGGATGGAGGTGTCCCAGGAGGATGACAAAGCACTCCA
GCTTCTCCATGATATCAGAGAGCAGAGCCGGAAGCTCCAAGAAATCAAAGAGCAGGAGTACCAGGCTCAA
GTGGAAGAAATGAGGTTGATGATGAATCAGTTGGAAGAGGACCTTGTCTCAGCAAGAAGACGGAGTGATC
TCTACGAATCTGAGCTGAGAGAGTCTCGGCTCGCTGCTGAAGAATTCAAGCGGAAAGCGACAGAATGTCA
GCATAAACTGTTGAAGGCTAAGGATCAAGGGAAGCCTGAAGTGGGAGAATATGCGAAACTGGAGAAGATC
AATGCTGAGCAGCAGCTCAAAATTCAGGAGCTCCAAGAGAAACTGGAGAAGGCTGTAAAAGCCAGCACCG
AGGCCACCGAGCTGCTGCAGAATATCCGCCAGGCAAAGGAGCGAGCCGAGAGGGAGCTGGAGAAGCTGCA
GAACCGAGAGGATTCTTCTGAAGGCATCAGAAAGAAGCTGGTAGAAGCCGAGGAACGTCGCCATTCTCTG
GAGAACAAGGTAAAGAGACTAGAGACCATGGAGCGTAGAGAAAACAGACTGAAGGATGACATCCAGACAA
AATCCCAACAGATCCAGCAGATGGCTGATAAAATTCTGGAACTCGAAGAGAAACATCGGGAGGCCCAAGT
CTCAGCCCAGCACCTAGAAGTGCACCTGAAACAGAAAGAGCAGCACTACGAGGAAAAGATTAAAGTGTTG
GACAATCAGATAAAGAAAGACCTGGCCGACAAGGAGACGCTGGAGAACATGATGCAGAGACACGAGGAGG
AGGCCCATGAGAAGGGCAAAATTCTCAGTGAACAGAAGGCGATGATCAATGCTATGGATTCCAAGATCAG
ATCCCTGGAACAGAGGATTGTGGAACTGTCTGAAGCCAATAAACTTGCAGCAAATAGCAGTCTTTTTACC
CAAAGGAACATGAAGGCTCAAGAAGAGATGATTTCTGAACTCAGGCAACAGAAATTTTACCTGGAGACCC
AGGCTGGGAAATTGGAGGCCCAGAACCGAAAGCTGGAGGAGCAGCTGGAGAAGATCAGCCACCAAGACCA
CAGTGACAAGAATCGGCTGCTGGAACTGGAGACAAGACTGCGGGAGGTCAGTCTAGAGCACGAGGAGCAG
AAACTGGAGCTCAAGCGCCAGCTCACAGAGCTACAGCTCTCCCTGCAGGAGCGTGAGTCACAGTTGACAG
CCCTGCAGGCTGCCCGGGCGGCCCTGGAAAGCCAGCTTCGCCAGGCGAAGACAGAGCTGGAAGAGACCAC
AGCAGAAGCCGAAGAGGAGATCCAGGCGCTCACGGCACATAGAGATGAAATCCAGCGCAAATTTGATGCT
CTTCGTAACAGCTGTACTGTAATCACAGACCTGGAGGAGCAGCTAAACCAGCTGACCGAAGATAATGCTG
AACTCAACAACCAAAACTTCTACTTGTCCAAACAACTCGATGAGGCTTCCGGCGCCAACGATGAGATTGT
ACAACTGCGAAGTGAAGTGGACCACCTTCGCCGGGAGATCACAGAACGAGAGATGCAGCTTACCAGCCAG
AAGCAAACGATGGAGGCTCTGAAGACCACGTGTACCATGCTGGAGGAACAGGTCATGGATTTGGAGGCCC
TAAATGACGAGCTGCTAGAAAAAGAGCGGCAGTGGGAGGCCTGGAGGAGCGTCCTGGGTGACGAGAAATC
CCAGTTTGAGTGTCGGGTTCGAGAGCTGCAGAGGATGTTGGACACCGAGAAACAGAGCAGGGCGAGAGCC
GATCAGCGGATCACCGAGTCTCGCCAGGTGGTGGAGCTGGCAGTGAAGGAGCACAAGGCGGAGATTCTTG
CTCTGCAGCAGGCTCTCAAAGAGCAGAAGCTGAAGGCCGAGAGCCTCTCTGACAAGCTCAATGACCTGGA
GAAGAAGCATGCTATGCTTGAAATGAATGCCCGAAGCTTACAGCAGAAGCTGGAGACTGAACGAGAGCTC
AAACAGAGGCTTCTGGAAGAGCAAGCCAAATTACAGCAGCAGATGGACCTGCAGAAAAATCACATTTTCC
GTCTGACTCAAGGACTGCAAGAAGCTCTAGATCGGGCTGATCTGCTGAAGACAGAAAGAAGTGACCTGGA
GTATCAGCTGGAAAACATTCAGGTTCTCTATTCTCATGAAAAGGTGAAAATGGAAGGCACTATTTCTCAA
CAAACCAAACTCATCGATTTTCTGCAAGCCAAAATGGACCAACCTGCTAAAAAGAAAAAGGGTTTATTTA
GTCGACGGAAAGAGGACCCTGCTTTGCCCACACAGGTTCCTCTGCAGTACAATGAGCTGAAGCTGGCCCT
GGAAAAGGAGAAAGCTCGCTGTGCAGAGCTAGAGGAAGCCCTTCAGAAGACCCGCATCGAGCTCCGGTCC
GCCCGGGAGGAAGCTGCCCACCGCAAAGCGACGGACCACCCACACCCATCCACGCCAGCCACCGCGAGGC
AGCAGATCGCCATGTCCGCCATCGTGCGGTCACCAGAGCACCAGCCCAGTGCCATGAGCCTGCTGGCCCC
GCCATCCAGCCGCAGAAAGGAGTCTTCAACTCCAGAGGAATTTAGTCGGCGTCTTAAGGAACGCATGCAC
CACAATATTCCTCACCGATTCAACGTAGGACTGAACATGCGAGCCACAAAGTGTGCTGTGTGTCTGGATA
CCGTGCACTTTGGACGCCAAGCATCCAAATGTCTCGAATGTCAGGTGATGTGTCACCCCAAGTGCTCCAC
GTGCTTGCCAGCCACCTGCGGCCTGCCTGCCGAATACGCCACACACTTCACTGAGGCCTTCTGCCGTGAC
AAAATGAACTCCCCGGGTCTCCAGACCAAGGAGCCCAGCAGCAGCTTGCACCTGGAAGGGTGGATGAAGG
TGCCCAGGAATAACAAACGAGGACAGCAAGGCTGGGACAGGAAGTACATTGTCCTGGAGGGATCTAAAGT
CCTCATTTATGACAATGAAGCCAGAGAAGCTGGACAGAGGCCGGTGGAAGAATTTGAGCTGTGCCTTCCC
GACGGGGATGTATCTATTCATGGTGCCGTTGGTGCTTCCGAACTCGCAAATACAGCCAAAGCAGATGTCC

CATACATACTGAAGATGGAATCTCACCCGCACACCACCTGCTGGCCCGGGAGAACCCTCTACTTGCTAGC
TCCCAGCTTCCCTGACAAACAGCGCTGGGTCACCGCCTTAGAATCAGTTGTCGCAGGTGGGAGAGTTTCT
AGGGAAAAAGCAGAAGCCGATGCTAAATTGCTTGGAAACTCCCTGCTGAAACTGGAAGGTGATGACCGTC
TAGACATGAACTGCACACTGCCCTTCAGTGACCAGGTGGTGTTGGTGGGCACCGAGGAAGGGCTCTACGC
CCTGAATGTCTTGAAAAACTCCCTAACGCATGTCCCAGGAATTGGAGCAGTCTTCCAAATTTATATTATC
AAGGATCTGGAGAAGCTACTCATGATAGCAGGAGAAGAGCGGGCACTGTGCCTTGTGGACGTGAAGAAAG
TAAAACAGTCCCTGGCGCAGTCCCACCTGCCTGCCCAGCCCGACATCTCACCCAACATTTTTGAAGCTGT
CAAGGGCTGCCACTTGTTTGGGGCTGGCAAGATTGAGAACGGGCTCTGCATCTGTGCAGCCATGCCCAGC
AAAGTCGTCATTCTCCGCTACAACGAAAACCTCAGCAAATACTGCATTCGGAAAGAGATAGAGACCTCAG
AGCCTTGCAGCTGTATCCACTTCACCAATTACAGTATCCTCATTGGAACCAATAAATTCTACGAAATCGA
CATGAAGCAGTACACGCTGGAGGAATTCCTGGATAAGAATGACCATTCCTTGGCACCTGCTGTGTTTGCC
GCCTCTTCCAACAGCTTCCCTGTCTCGATCGTGCAGGTGAACAGTGCAGGGCAGCGAGAGGAGTACTTGC
TGTGTTTCCACGAGTTTGGAGTGTTCGTGGATTCTTACGGAAGACGTAGCCGCACAGACGACCTCAAGTG
GAGTCGCTTACCTTTGGCCTTTGCCTACAGAGAACCCTATCTGTTTGTGACCCACTTCAACTCACTCGAA
GTAATTGAGATCCAGGCACGCTCCTCGGCAGGGACCCCTGCCCGAGCGTACCTGGACATCCCGAACCCAC
GCTACCTGGGCCCTGCCATTTCCTCGGGAGCGATTTACTTGGCATCCTCATACCAGGATAAATTAAGGGT
CATTTGCTGCAAGGGAAACCTCGTGAAGGAGTCTGGCACTGACCACCACCGGGGCCCGTCCACCTCCCGC
AGCAGCCCCAACAAGCGAGGCCCGCCCACGTACAACGAGCACATCACCAAGCGCGTGGCCTCCAGCCCGG
CGCCGCCCGAAGGCCCCAGCCACCCCCGAGAGCCAAGCACACCGCACCGCTACCGCGAGGGGCGGACCGA
GCTGCGCAGGGACAAGTCTCCTGGCCGCCCCCTGGAGCGGGAGAAGTCCCCGGGCCGGATGCTCAGCACG
CGGAGAGAGCGGTCCCCCGGGAGGCTGTTTGAAGACAGCAGCAGGGGCCGGCTGCCTGCGGGAGCCGTGA
GGACCCCGCTGTCCCAGGTGAACAAGGTCTGGGACCAGTCTTCAGTA
>Saimiri boliviensis boliviensis XM_003932193.2
ATGCTGAAGTTCAAATATGGAGTGCGGAATCCTTTGGATGCCGGTGCTGCTGAACCCATTGCCAGCCGGG
CCTCCAGGCTGAATCTGTTCTTCCAGGGGAAACCACCCTTTATGACTCAACAGCAGATGTCTCCTCTTTC
CCGAGAAGGGATATTAGATGCCCTCTTTGTTCTCTTTGAAGAGTGCAGTCAGCCTGCTCTGATGAAGATT
AAGCATGTGAGCAACTTTGTCCGGAAGTATTCCGACACCATAGCTGAGTTACAGGAGCTCCAGCCTTCGG
CAAAGGACTTTGAAATCAGAAGTCTTGTAGGTTGTGGTCACTTTGCTGAAGTGCAGGTGGTAAGAGAGAA
AGCAACTGGGGACATCTATGCCATGAAAGTGATGAAGAAGAAGGCCTTATTGGCCCAGGAGCAGGTTTCG
TTTTTTGAGGAAGAACGAAATATATTATCTCGAAGCACAAGCCCTTGGATCCCCCAATTACAGTATGCCT
TTCAGGACAAAAATCACCTGTATCTGGTCATGGAATATCAGCCTGGAGGGGACTTGCTGTCACTTTTGAA
TAGATATGAGGACCAATTAGATGAAAATCTGATTCAGTTTTACCTGGCTGAACTGATTTTGGCTGTTCAC
AGTGTTCATCAGATGGGATACGTGCATCGAGACATCAAGCCCGAGAACATTCTCATTGACCGCACAGGAC
ACATCAAGCTGGTGGATTTTGGATCAGCTGCGAAAATGAATTCAAACAAGATGGTGAGTGCTAGACTCCC
AGTTGGGACCCCAGATTACATGGCTCCTGAAGTGCTGACTGTGATGAATGGGGATGGAAAAGGCACCTAT
GGCCTAGACTGTGACTGGTGGTCGGTGGGCGTGATTGCCTATGAGATGATTTATGGGAGATCCCCCTTCG
CAGAGGGAACTTCCGCCAGAACCTTCAATAACATCATGAACTTCCAGCGGTTTTTGAAGTTTCCAGATGA
CCCTAAAGTGAGCAGTGACTCTCTTGATCTGATTCAAAGTTTGTTGTGTGGCCAGAAAGAGAGGCTGAAG
TTTGAAGGCCTTTGCTGCCATCCTTTCTTCTCTAAAATTGACTGGAATAACATTCGTAACTCTCCTCCCC
CCTTCGTTCCCACCCTCAAGTCTGACGATGACACCTCCAATTTTGATGAACCAGAGAAGAATTCGTGGGT
TTCATCCTCTCCGTGCCAGCTGAGCCCCTCAGGTTTCTCGGGTGAAGAACTGCCGTTTGTGGGGTTTTCG
TACAGCAAGGCACTGGGAATTCTTGGTAGATCTGAGTCTGTTGTGTCGGGTCTGGACTCCCCTGCCAAGA
CTAGCTCCATGGAAAAGAAACTTCTCATCAAAAGCAAAGAGCTACAAGACTCTCAGGACAAGTGTCACAA
GATGGAGCAGGAAATGACCCGGTTACATCGGAGAGTGTCAGAGGTGGAGGCTGTGCTTAGTCAGAAGGAG
GTGGAGCTGAAGGCCTCTGAGACTCAGAGATCCCTCCTGGAGCAGGACCTTGCTACCTACATCACAGAAT
GCAGTAGCTTAAAGCGAAGTTTGGAGCAAGCACGGATGGAGGTGTCCCAGGAGGATGACAAAGCACTGCA
GCTTCTCCATGATATCAGAGAGCAGAGCCGGAAGCTCCAAGAAATCAAAGAGCAGGAGTACCAGGCTCAA

GTGGAAGAAATGAGGTTGATGATGAATCAGTTGGAAGAGGACCTTGTCTCAGCAAGAAGACGGAGTGATC
TCTACGAATCGGAGCTGAGAGAGTCTCGGCTTGCCGCCGAAGAATTCAAGCGGAAAGCGACAGAATGTCA
GCATAAACTATTGAAGGCTAAGGATCAAGGGAAGCCGGAGGTGGGAGAATATGCTAAGCTGGAGAAGATC
AATGCTGAGCAGCAGCTCAAAATTCAGGAGCTCCAAGAGAAACTGGAGAAGGCTGTAAAAGCCAGCACAG
AGGCCACCGAGCTGCTGCAGAATATCCGCCAGGCAAAGGAGCGAGCTGAGAGGGAGCTGGAGAAACTGCA
GAACCGAGAGGACTCTTCTGAAGGCATCAGAAAGAAGCTGGTGGAAGCCGAGGAACGCCGCCATTCTCTG
GAGAACAAGGTAAAGAGACTAGAGACCATGGAGCGTAGAGAAAACAGACTGAAGGATGACATCCAGACAA
AATCCCAACAGATCCAGCAGATGGCTGATAAAATTCTGGAGCTTGAAGAGAAGCATCGGGAGGCCCAAGT
CTCAGCCCAGCACCTAGAAGTGCACCTGAAACAGAAGGAGCAGCACTATGAGGAAAAAATTAAAGTGTTG
GACAATCAGATAAAGAAAGACCTGGCCGACAAGGAGACCCTGGAGAACATGATGCAGAGACACGAGGAGG
AGGCCCACGAGAAGGGCAAGATTCTCAGCGAACAGAAGGCGATGATCAATGCTATGGATTCCAAGATCAG
ATCCCTGGAACAGAGGATTGTGGAACTGTCTGAAGCCAATAAACTTGCAGCAAACAGCAGCCTTTTTACC
CAAAGGAACATGAAGGCCCAAGAAGAGATGATTTCTGAACTCAGGCAACAGAAATTTTACCTGGAGACAC
AGGCTGGGAAATTGGAAGCCCAGAACCGAAAGCTGGAGGAGCAGCTGGAGAAGATCAGCCACCAAGACCA
TAGTGACAAGAATCGGCTGCTGGAACTGGAGACAAGACTGCGGGAGGTCAGTCTAGAGCATGAGGAGCAA
AAACTGGAGCTCAAGCGGCAGCTCACAGAGCTACAGCTCTCCCTGCAGGAGCGCGAGTCGCAGTTGACAG
CGCTGCAGGCTGCCCGGGCGGCCTTGGAGAGCCAGCTTCGCCAGGCGAAGACAGAGCTGGAAGAGACCAC
AGCGGAAGCCGAAGAGGAGATCCAGGCCCTCACGGCACATAGAGATGAAATCCAGCGCAAATTTGATGCT
CTTCGTAACAGCTGTACTGTAATCACAGACCTGGAGGAGCAGTTAAACCAGCTGACCGAGGACAACGCTG
AACTCAACAACCAAAACTTCTATTTGTCCAAACAACTCGATGAGGCTTCTGGCGCCAATGATGAGATTGT
ACAACTGCGAAGTGAAGTGGACCACCTCCGCCGGGAGATCACGGAACGAGAGATGCAACTCACCAGTCAG
AAGCAAACGATGGAGGCTCTGAAGACCACATGCACCATGCTGGAGGAACAGGTCATGGATTTGGAGGCCC
TAAACGACGAGCTGCTAGAAAAAGAGCGGCAGTGGGAGGCCTGGAGGAGCGTCCTGGGTGACGAGAAATC
CCAGTTTGAGTGTCGGGTTCGAGAGCTGCAGAGGATGTTGGACACCGAGAAACAGAGCAGGGCGAGGGCC
GATCAGCGGATCACCGAGTCTCGCCAGGTGGTGGAGCTGGCAGTGAAGGAACACAAGGCCGAGATTCTTG
CTCTGCAGCAGGCTCTCAAAGAGCAGAAGCTGAAGGCTGAGAGCCTCTCTGACAAGCTCAATGACCTGGA
GAAGAAGCATGCTATGCTTGAAATGAATGCCCGAAGCTTACAGCAGAAGCTGGAGACTGAACGAGAGCTC
AAGCAGAGGCTTCTGGAGGAGCAAGCCAAATTACAGCAGCAGATGGACCTGCAGAAAAACCACATTTTCC
GTCTGACTCAAGGACTGCAAGAAGCTCTAGATCGGGCTGATCTGCTGAAGACAGAAAGAAGTGACCTGGA
GTATCAGCTAGAGAACATTCAGGTTCTCTATTCTCATGAAAAGGTAAAAATGGAAGGCACTATTTCTCAA
CAAACCAAACTCATTGATTTTCTGCAAGCCAAAATGGACCAACCTGCTAAAAAGAAAAAGGGTTTATTTA
GTCGACGGAAAGAGGACCCTGCTTTGCCCACACAGGTTCCTCTGCAGTACAATGAGCTGAAGCTGGCCCT
GGAGAAGGAGAAAGCTCGCTGTGCAGAGCTAGAGGAAGCCCTTCAGAAGACCCGCATTGAGCTCCGGTCT
GCCCGGGAGGAAGCTGCCCACCGCAAAGCGACAGACCACCCACACCCATCCACGCCAGCCACCGCGAGGC
AGCAGATCGCCATGTCCGCCATCGTGCGGTCTCCAGAGCACCAGCCCAGTGCCATGAGCCTGCTGGCCCC
GCCGTCCAGCCGCAGAAAGGAGTCTTCAACTCCAGAGGAATTTAGTCGGCGTCTGAAGGAACGCATGCAC
CACAATATTCCTCACCGCTTCAACGTAGGACTGAACATGCGAGCCACAAAGTGTGCTGTTTGTTTGGATA
CTGTGCACTTTGGACGCCAGGCATCCAAATGTCTTGAATGTCAGGTGATGTGTCACCCCAAGTGCTCCAC
GTGCTTGCCAGCCACATGTGGCCTGCCTGCCGAATATGCCACACACTTCACCGAGGCCTTCTGCCGCGAC
AAAATGAACTCCCCGGGTCTCCAGACCAAGGAGCCCAGCAGCAGCTTGCACCTGGAAGGGTGGATGAAAG
TGCCCAGGAATAACAAACGAGGGCAGCAAGGCTGGGACAGGAAGTACATTGTCTTGGAGGGATCAAAAGT
CCTCATTTATGACAGTGAAGCCAGAGAAGCTGGACAGAGGCCGGTGGAAGAATTTGAGCTGTGCCTTCCC
GACGGGGATGTATCTATTCATGGTGCCGTTGGTGCTTCCGAACTCGCAAATACAGCCAAAGCAGATGTCC
CATACATACTGAAGATGGAATCTCACCCGCACACCACCTGCTGGCCCGGGAGAACCCTCTACTTGCTAGC
TCCTAGCTTCCCCGACAAACAGCGCTGGGTCACCGCCTTAGAATCAGTTGTCGCAGGTGGGAGAGTTTCT
AGGGAAAAAGCAGAAGCCGATGCTAAATTACTTGGAAACTCCCTGCTGAAACTGGAAGGTGATGACCGTC
TAGACATGAACTGCACACTGCCCTTCAGTGACCAGGTGGTGTTGGTGGGCACCGAGGAAGGGCTCTACGC

CCTGAATGTCTTGAAAAACTCCCTAACCCACGTCCCAGGAATTGGAGCAGTCTTCCAAATTTATATTATC
AAGGACCTGGAGAAGCTACTCATGATAGCAGGAGAAGAACGGGCACTGTGTCTTGTGGACGTGAAGAAGG
TGAAACAGTCCCTAGCGCAGTCCCACCTTCCCGCCCAGCCAGACATCTCACCCAACATTTTCGAAGCTGT
GAAGGGTTGCCACTTGTTTGGGGCTGGCAAGATTGAGAACGGGCTCTGCATCTGTGCAGCCATGCCCAGC
AAAGTCGTCATTCTCCGCTACAATGAAAACCTCAGCAAATACTGCATTCGGAAAGAGATAGAGACCTCAG
AGCCCTGCAGCTGTATCCACTTCACCAATTACAGTATCCTCATCGGAACCAATAAATTCTATGAAATCGA
CATGAAGCAGTACACACTTGAGGAATTCCTGGATAAGAATGATCATTCCTTGGCACCTGCTGTGTTTGCT
GCCTCTTCCAACAGCTTCCCTGTCTCGATAGTGCAGGTGAACAGTGCAGGGCAGCGAGAGGAGTACCTGC
TGTGTTTCCACGAATTTGGAGTGTTCGTGGATTCTTACGGAAGACGTAGCCGGACAGACGATCTCAAGTG
GAGTCGCTTACCTTTGGCCTTCGCCTACAGAGAACCCTATCTGTTTGTGACCCACTTCAACTCACTCGAA
GTAATTGAGATCCAGGCACGATCCTCGGCAGGGCCCCCTGCCCGAGCGTATCTGGACATTCCGAACCCAC
GCTACCTGGGCCCTGCGATCTCCTCGGGAGCGATTTACCTGGCGTCCTCATACCAGGACAAATTACGGGT
CATTTGCTGCAAGGGAAACCTCGTGAAGGAGTCTGGCACTGAACACCACCGGGGCCCTTCCACCTCCCGC
AGCAGCCCCAACAAGCGAGGCCCACCCACATACAACGAGCACATCACCAAGCGAGTGGCCTCCAGCCCCG
CGCCGCCCGAAGGCCCCAGCCACCCCCGAGAGCCAAGCACACCCCACCGCTACCGTGAGGGGCGGACTGA
GCTGCGCAGGGACAAGTCTCCTGGCCGCCCCCTGGAGCGGGAGAAGTCCCCGGGCCGGATGCTCAGCACA
CGGAGAGAGCGGTCCCCCGGGAGGCTGTTTGAAGACAGCAGCAGGGGCCGGCTGCCTGCAGGAGCCGTGA
GGACCCCGCTGTCCCAGGTTAACAAGGTCTGGGACCAGTCTTCAGTA
>Cebus capucinus imitator XM_017510213.1
ATGCTGAAGTTCAAATATGGAGCGCGGAATCCTTTGGATGCTGGTGCTGCTGAACCCATTGCCAGCCGGG
CCTCCAGGCTGAATCTGTTCTTCCAGGGGAAACCACCCTTTATGACTCAACAGCAGATGTCTCCTCTTTC
CCGAGAAGGGATATTAGATGCCCTCTTTGTTCTCTTTGAAGAGTGCAGTCAGCCCGCTCTGATGAAGATT
AAGCATGTGAGCAACTTTGTCCGGAAGTATTCCGACACCATAGCTGAGTTACAGGAGCTCCAGCCTTCGG
CAAAGGACTTTGAAGTCAGAAGTCTTGTAGGTTGTGGTCACTTTGCTGAAGTGCAGGTGGTAAGAGAGAA
AGCAACTGGGGACATCTATGCCATGAAAGTGATGAAGAAGAAGGCCTTATTGGCCCAGGAGCAGGTTTCA
TTTTTTGAGGAAGAACGAAACATATTATCTCGAAGCACAAGCCCTTGGATCCCCCAATTACAGTATGCCT
TTCAGGACAAAAATCACCTGTATCTGGTCATGGAATATCAGCCTGGAGGGGACTTGCTGTCACTTTTGAA
TAGATATGAGGACCAATTAGATGAAAATCTGATTCAGTTTTACCTGACTGAACTGATTTTGGCTGTTCAC
AGCGTTCATCAGATGGGATACGTGCATCGAGACATCAAGCCCGAGAACATTCTCATTGACCGCACAGGAC
ACATCAAGCTGGTGGATTTTGGATCAGCTGCAAAAATGAATTCAAACAAGACGGTGAGTGCCAGACTCCC
AGTTGGGACCCCAGATTATATGGCTCCTGAAGTATTGACTGTGATGAATGGGGATGGAAAAGGCATCTAC
GGCCTAGATTGTGACTGGTGGTCGGTGGGCGTGATTGCCTATGAGATGATTTATGGGAGATCCCCCTTCG
CAGAGGGAACCTCCGCCAGAACCTTCAATAACATCATGAATTTCCAGCGGTTTTTGAAGTTTCCAGATGA
CCCCAAAGTGAGCAGTAACTTTCTTGATCTGATTCAAAGTTTGTTGTGTGGCCAGAAAGAGAGGCTGAAG
TTTGAAGGCCTTTGCTGCCATCCTTTCTTCTCTAAAATCGACTGGAATAACATTCGTAACTCTCCTCCCC
CCTTCGTTCCCACCCTCAAGTCTGACGATGACACCTCCAATTTTGATGAACCAGAGAAGAATTCGTGGGT
TTCATCCTCTCCGTGCCAGCTGAGCCCCTCAGGTTTCTCGGGTGAAGAACTGCCGTTTGTGGGGTTTTCG
TACAGCAAGGCACTGGGGATTCTTGGTAGATCTGAGTCTATTGTGTCGGGTCTGGACTCCCCTGCCAAGA
CTAGCTCCATGGAAAAGAAACTTCTCATCAAAAGCAAAGAGCTACAAGACTCTCAGGACAAGTGTCACAA
GATGGAGCAGGAAATGACCCGGTTACATCGGAGAGTGTCAGAGGTGGAGGCTGTGCTTAGTCAGAAGGAG
GTGGAGCTGAAGGCCTCTGAGACTCAGAGATCCCTCCTGGAGCAGGACCTTGCTACCTACATCACAGAAT
GCAGTAGCTTAAAGCGAAGTTTGGAGCAAGCACGGATGGAGGTGTCCCAGGAGGATGACAAAGCACTGCA
GCTTCTCCATGATATCAGAGAGCAGAGCCGGAAGCTCCAAGAAATCAAAGAGCAGGAGTACCAGGCTCAA
GTGGAAGAAATGAGGTTGATGATGAATCAGTTGGAAGAGGACCTTGTCTCGGCAAGAAGACGGAGTGATC
TCTACGAATCGGAGCTGAGAGAGTCTCGGCTCGCCGCCGAAGAATTCAAGCGGAAAGCGACAGAATGTCA
GCATAAACTATTGAAGGCTAAGGATCAAGGGAAGCCGGAGGTGGGAGAATATGCTAAGCTGGAGAAGATC
AATGCTGAGCAGCAGCTCAAAATTCAGGAGCTCCAGGAGAAACTGGAGAAGGCTGTAAAAGCCAGCACAG

AGGCCACCGAGCTGCTGCAGAATATCCGCCAGGCAAAGGAACGAGCTGAGAGGGAGCTGGAGAAGCTGCA
GAACCGGGAGGATTCTTCTGAAGGCATCAGAAAGAAGCTGGTGGAAGCCGAGGAACGCCGCCATTCTCTG
GAGAACAAGGTAAAGAGACTAGAGACCATGGAGCGTAGAGAAAACAGACTGAAGGATGACATCCAGACAA
AATCCCAACAGATCCAGCAGATGGCTGATAAAATTCTGGAGCTCGAAGAGAAGCATCGGGAGGCCCAAGT
CTCAGCCCAGCACCTAGAAGTGCACCTGAAACAGAAAGAGCAGCACTACGAGGAAAAAATTAAAGTGTTG
GACAATCAGATAAAGAAAGACCTGGCCGACAAGGAGACGCTGGAGAACATGATGCAGAGACACGAGGAGG
AGGCCCATGAGAAGGGCAAAATTCTCAGCGAACAGAAGGCGATGATCAATGCTATGGATTCCAAGATCAG
ATCCCTGGAACAGAGGATTGTGGAACTGTCTGAAGCCAATAAACTTGCAGCAAACAGCAGCCTTTTTACC
CAAAGGAACATGAAGGCCCAAGAAGAGATGATTTCTGAACTCAGGCAACAGAAATTTTACCTGGAGACAC
AGGCTGGGAAATTGGAGGCCCAGAACCGAAAGCTGGAGGAGCAGCTGGAGAAGATCAGCCACCAAGACCA
TAGTGACAAGAATCGGCTGCTGGAACTGGAGACACGATTGCGGGAGGTCAGTCTAGAGCATGAGGAGCAA
AAACTGGAGCTCAAGCGCCAGCTCACAGAGCTACAGCTCTCCCTGCAGGAGCGCGAGTCGCAGTTGACAG
CGCTGCAGGCCGCCCGGGCGGCCTTGGAAAGCCAGCTTCGCCAGGCGAAGACAGAGCTGGAAGAGACCAC
AGCGGAAGCAGAAGAGGAGATCCAGGCACTCACGGCACATAGAGATGAAATCCAGCGCAAATTTGATGCT
CTTCGTAACAGCTGTACTGTAATCACAGACCTGGAGGAGCAGCTAAACCAGCTGACTGAGGACAATGCTG
AACTCAACAACCAAAACTTCTATTTGTCCAAACAACTTGATGAGGCTTCCGGTGCCAACGATGAGATTGT
ACAACTGCGAAGTGAAGTGGACCACCTCCGCCGGGAGATTACAGAACGAGAGATGCAACTCACCAGCCAG
AAGCAAACGATGGAGGCTCTGAAGACCACATGCACCATGCTGGAGGAACAGGTCATGGATTTGGAGGCCC
TAAACGACGAGCTGCTAGAAAAAGAGCGGCAGTGGGAGGCCTGGAGGAGCGTCCTGGGTGACGAGAAATC
CCAGTTTGAGTGTCGGGTTCGAGAGCTGCAGAGGATGTTGGACACCGAGAAACAGAGCAGGGCGAGAGCT
GATCAGCGGATCACCGAGTCTCGCCAGGTGGTGGAGCTGGCAGTGAAAGAGCACAAGGCCGAGATTCTTG
CTCTGCAGCAGGCTCTCAAAGAGCAGAAGCTGAAGGCTGAGAGCCTCTCTGACAAGCTCAATGACCTGGA
GAAGAAGCATGCTATGCTTGAAATGAATGCCCGAAGCTTACAGCAGAAGCTGGAGACTGAACGAGAGCTC
AAGCAGAGGCTTCTGGAGGAGCAAGCCAAATTACAGCAGCAGATGGACCTGCAGAAAAACCACATTTTCC
GTCTGACTCAAGGACTGCAAGAAGCTCTAGATCGGGCTGATCTGCTGAAGACAGAAAGAAGTGACCTGGA
GTATCAGCTAGAGAACATTCAGGTTCTCTATTCTCATGAAAAGGTGAAAATGGAAGGCACTATTTCTCAA
CAAACCAAACTCATTGATTTTCTGCAAGCCAAAATGGACCAACCTGCTAAAAAGAAAAAGGGTTTATTTA
GTCGACGGAAAGAGGACCCTGCTTTGCCCACACAGGTTCCTCTGCAGTACAATGAGCTGAAGCTGGCCCT
GGAGAAGGAGAAAGCTCGCTGTGCAGAGCTAGAGGAAGCCCTTCAGAAGACCCGCATTGAGCTCCGTTCT
GCCCGGGAGGAAGCTGCCCACCGCAAAGCGACGGACCACCCACACCCATCCACGCCAGCCACTGCAAGGC
AGCAGATCGCCATGTCTGCCATCGTGCGGTCGCCAGAGCACCAGCCCAGTGCCATGAGCCTGCTGGCCCC
GCCGTCCAGCCGCAGAAAGGAGTCTTCAACTCCAGAGGAATTTAGTCGGCGTCTTAAGGAACGCATGCAC
CACAATATTCCTCACCGATTCAACGTAGGACTGAACATGCGGGCCACAAAGTGTGCTGTTTGTTTGGATA
CCGTGCACTTTGGACGCCAGGCATCCAAATGTCTTGAATGTCAGGTGATGTGTCACCCCAAGTGCTCCAC
GTGCTTGCCAGCCACCTGCGGACTGCCTGCTGAATATGCCACACACTTCACCGAGGCCTTCTGCCGCGAC
AAAATGAACTCTCCGGGTCTCCAGACCAAGGAGCCCAGCAGCAGCTTGCACCTGGAAGGGTGGATGAAGG
TGCCCAGGAATAACAAACGAGGACAGCAAGGCTGGGACAGGAAGTACATTGTCCTGGAGGGATCAAAAGT
CCTCATTTATGACAGTGAAACCAGAGAAGCTGGACAGAGGCCGGTGGAAGAATTTGAGCTGTGCCTTCCC
GACGGGGATGTATCTATTCACGGTGCCGTTGGTGCTTCTGAACTGGCAAATACAGCCAAAGCAGATGTCC
CATACATACTGAAGATGGAATCTCACCCGCACACCACCTGCTGGCCCGGGAGAACCCTCTACTTGCTAGC
TCCTAGCTTCCCCGACAAACAGCGCTGGGTCACCGCCTTAGAATCAGTTGTCGCAGGTGGGAGAGTTTCT
AGGGAAAAAGCAGAAGCCGATGCTAAATTGCTTGGAAACTCCCTGCTGAAACTGGAAGGTGATGACCGTC
TAGACATGAACTGCACACTGCCCTTCAGTGACCAGGTGGTGTTGGTGGGCACCGAGGAAGGGCTCTATGC
CCTGAATGTCTTGAAAAACTCCCTAACCCACGTCCCAGGAATTGGAGCAGTCTTCCAAATTTATATTATC
AAGGACCTGGAGAAGCTACTCATGATAGCAGGAGAAGAACGGGCACTGTGTCTTGTGGACGTGAAGAAGG
TGAAACAGTCCCTAGCGCAGTCCCACCTTCCCGCCCAGCCAGACATCTCACCCAACATTTTCGAAGCTGT
GAAGGGCTGCCACTTGTTTGGGGCTGGCAAGATTGAGAATGGGCTCTGCATCTGTGCAGCAATGCCCAGC

AAAGTTGTCATTCTCCGCTACAACGAAAACCTCAGCAAATACTGCATTCGGAAAGAGATAGAGACCTCAG
AGCCCTGCAGCTGTATCCACTTCACCAATTACAGTATCCTCATCGGAACCAATAAATTCTATGAAATCGA
CATGAAGCAGTACACACTTGAGGAATTCCTGGATAAGAATGATCATTCCTTGGCACCTGCTGTGTTTGCT
GCCTCTTCCAACAGCTTCCCTGTCTCGATCGTGCAGGTGAACGGCGCAGGGCAGAGAGAGGAGTACCTGC
TGTGTTTCCACGAATTTGGAGTGTTCGTGGATTCTTATGGAAGACGTAGCCGGACAGACGATCTCAAGTG
GAGTCGCTTACCTTTGGCCTTCGCCTACAGAGAACCCTATCTGTTTGTGACCCACTTCAACTCACTCGAA
GTAATTGAGATCCAGGCACGCTCCTCGGCAGGGACCCCTGCCCGAGCGTATCTGGACATTCCGAACCCGC
GCTACCTGGGCCCTGCGATCTCCTCGGGAGCGATTTACCTGGCGTCCTCATACCAGGATAAATTACGGGT
CATTTGCTGCAAGGGAAACCTCGTGAAGGAGTCCGGAACTGAACACCACCGGGGCCCTTCCACCTCCCGC
AGCAGCCCCAACAAGCGAGGCCCGCCCACATACAACGAGCACATCACCAAGCGTGTGGCCTCCAGCCCAG
CGCCGCCCGAAGGCCCCAGCCACCCCCGAGAGCCAAGCACACCCCACCGCTACCGTGAGGGGCGGACCGA
GCTGCGCAGGGACAAGTCTCCCGGCCGCCCCCTGGAGCGGGAGAAGTCCCCCGGCCGGATGCTCAGCACG
CGGAGAGAGCGGTCCCCCGGGAGGCTGTTTGAAGACAGCAGCAGGGGCCGGCTGCCTGCGGGAGCTGTGA
GGACCCCACTGTCCCAGGTTAACAAGGTCTGGGACCAGTCTTCAGTA
>Otolemur garnettii XM_003789925.1
ATGTTGAAATTCAAGTATGGAGCACGGAATCCACTGGATGCTGGTGCGGTGGAACCCATTGCTAGCCGGG
CCTCCAGGCTGAATCTTTTCTTCCAGGGGAAACCACCCTTTATGACTCAACAGCAGATGTCTCTTCTTTC
CCGAGAAGGGATATTAGATGCCCTCTTTGTTCTCTTTGAAGAATGCAGTCAGCCTGCTCTGATGAAGATT
AAGCATGTGAGCAACTTTGTCCGGAAGTATTCTGATACCATAGCTGAGTTACAGGAGCTCCAGCCTTCGG
CAAAGGACTTTGAAATCAGAAGTCTTGTAGGTTGTGGTCACTTTGCTGAGGTGCAGGTGGTAAGAGAGAA
AGCAACTGGGGACATCTATGCCATGAAAGTCATGAAGAAGAAGGCCTTATTGGCCCAGGAGCAGGTTTCA
TTTTTTGAGGAAGAACGGAACATATTATCACGAAGTACAAGTCCTTGGATTCCCCAATTACAATATGCCT
TTCAGGACAAAAATAACCTGTATCTGGTCATGGAATATCAGCCAGGAGGGGACTTGCTGTCACTATTGAA
CAGATATGAGGACCAATTAGATGAAAATATGATTCAGTTTTACCTAGCCGAACTGATTTTGGCTGTTCAC
AGTGTCCATCAGATGGGATATGTACATCGAGACATCAAACCGGAGAACATTCTCATTGACCGAACGGGGC
ACATCAAGCTGGTGGATTTTGGATCAGCTTCTAAAATGAATTCAAACAAGATGGTGAATGCCAGACTCCC
AATTGGGACTCCAGATTACATGGCTCCTGAAGTGTTAACCGTGATGAATGGGGACGGGAAGGGAACCTAC
AGCCTAGACTGTGACTGGTGGTCAGTGGGGGTGATTGCCTATGAGATGATTTATGGGCGATCCCCTTTCA
CAGATGGAACTTCAGCCAGAACCTTCAATAACATCATGAATTTCCAAAGGTTTTTGAAGTTTCCAGATGA
TCCCAAAGTTAGCAGTGAATTTCTTGATCTGATTCAAAGTTTGTTGTGTGGACAGAAAGAGAGACTGAAG
TTTGAAGGGCTTTGCTGCCATCCTTTCTTCTCCAAAATTGACTGGAATAACATCCGTAATTCGCCTCCCC
CCTTCGTTCCCACCCTCAAGTCTGATGATGACACCTCCAATTTTGATGAACCAGAGAAGAATTCGTGGGT
TTCATCCTCTCCGTGCCAGCTGAGCCCCTCGGGTTTCTCAGGCGAAGAACTGCCGTTTGTAGGGTTTTCG
TACAGCAAGGCACTGGGGATTCTTGGTAGATCTGAGTCTGTTGTGTCGGGTCTGGACTCCCCTGCCAAGA
CTAGCTCCATGGAAAAGAAACTTCTCATCAAAAGCAAAGAGCTACAAGACTCTCAGGACAAGTGTCACAA
GATGGAGCAGGAAATGACCCGGTTACATCGGAGAGTATCAGAGGTGGAGGCTGTGCTTAGTCAGAAGGAG
GTGGAGCTGAAGGCCTCTGAGACTCAGAGATCTCTCCTGGAGCAGGACCTTGCTACCTACATCACAGAAT
GCAGTAGCTTAAAGCGAAGTTTGGAGCAAGCTCGGATGGAGGTGTCCCAGGAGGATGACAAAGCGCTGCA
GCTTCTCCATGATATCAGAGAGCAGAGCCGGAAGCTCCAAGAAATCAAAGAGCAGGAGTACCAGGCTCAA
GTGGAAGAAATGAGGTTGATGATGAATCAATTGGAAGAGGACCTTGTCTCAGCCAGAAGACGGAGTGATC
TCTACGAGTCTGAGCTGAGAGAGTCTCGCCTGGCTGCGGAAGAGTTCAAGCGGAAAGCGACAGAATGTCA
GCATAAGCTGTTGAAGTCAAAGGATCAAGGGAAGCCTGAAGTGGGAGAATATGCCAAACTGGAGAAGATC
AATGCTGAGCAGCAGCTCAAAATTCAAGAGCTCCAAGAGAAGCTGGAAAAGGCTGTAAAAGCCAGCACAG
AGGCCACCGAGCTGCTGCAGAACATCCGCCAGGCAAAGGAGCGAGCTGAGAGGGAGCTGGAGAAGCTGCA
GAATCGTGAAGATTCTTCTGAAGGCATCAGAAAGAAGCTGGCAGAAGCCGAGGAACGCCGCCATTCTCTG
GAGAACAAGGTAAAGAGACTAGAGACCATGGAGCGTAGAGAAAACAGACTGAAGGATGACATCCAGACAA
AATCCCAACAGATCCAGCAGATGGCTGATAAAATTCTGGAGCTGGAGGAGAAACACCGGGAGGCCCAAGT

CTCAGCCCAGCACCTAGAGGTGCACCTGAAACAGAAAGAGCAACACTACGAGGAAAAAATTAAAGTGTTG
GACAGTCAGATAAAGAAAGACCTGGCTGATAAGGAGAATCTCGAGAATATGATGCAGAGACATGAGGAGG
AGGCCCATGAGAAAGGCAAAATTCTCAGCGAGCAGAAGGCGATGATCAATGCTATGGATTCCAAGATCAG
GTCCCTGGAACAGAGGATTGTGGAACTGTCTGAAGCCAACAAACTTGCGGCAAACAGCAGTCTTTTTACC
CAAAGGAACATGAAGGCCCAAGAAGAGATGATTTCTGAACTCAGGCAACAGAAATTTTACCTGGAGACCC
AGGCTGGGAAATTGGAGGCCCAGAACCGAAAGCTGGAAGAGCAGCTGGAGAAAATGAGCCACCAAGACCA
CAGTGACAAGAATCGGCTACTAGAACTGGAGACAAGGTTGAGGGAGGTCAGTCTAGAGCATGAGGAGCAG
AAACTGGAGCTTAAGCGCCAGCTGACAGAGCTACAGCTGTCCCTGCAGGAGCGGGAGTCACAACTGACAG
CCCTGCAGGCTGCTCGGGCGGCCCTGGAGAGCCAGCTTCGCCAGGCAAAGACAGAGCTGGAGGAGACAAC
AGCAGAAGCTGAAGAGGAGATCCAGGCACTCACGGCACATAGAGATGAAATCCAGCGCAAATTTGATGCC
CTTCGTAACAGCTGTACTGTGATCACAGACCTGGAGGAGCAGCTAAACCAACTGACTGAGGACAATGCTG
AACTCAACAACCAAAACTTCTACCTGTCCAAACAACTTGATGAAGCGTCTGGAGCCAACGATGAGATAGT
ACAACTGCGGAGTGAAGTAGACCACCTTCGCCGTGAGATCACAGAGAGGGAGATGCAGCTCACCAGCCAG
AAGCAAACCATGGAGGCTCTGAAGACCACTTGTACAATGCTGGAAGAACAGGTCATGGACTTGGAGGCCT
TGAATGATGAACTGCTGGAAAAAGAACGGCAATGGGAAGCTTGGAGGAGCGTCCTCGGTGATGAGAAGTC
CCAGTTCGAGTGTCGGGTTCGAGAGTTACAGAGGATGCTGGACACTGAGAAACAGAGCAGGGCAAGAGCC
GATCAGCGGATCACCGAGTCTCGGCAGGTGGTGGAACTGGCGGTGAAGGAACACAAGGCTGAGATTCTCG
CCCTGCAGCAGGCTCTCAAAGAACAGAAGCTGAAAGCTGAGAGCCTCTCTGATAAGCTCAATGACCTGGA
GAAGAAGCATGCCATGCTTGAAATGAATGCCCGAAGCTTGCAGCAGAAACTGGAGACTGAACGGGAGCTC
AAACAAAGGCTTCTGGAAGAGCAAGCCAAATTACAGCAACAGATGGACCTGCAGAAGAATCACATCTTCC
GTCTGACACAAGGACTGCAAGAAGCTCTAGATCGAGCTGATCTGCTGAAGACAGAAAGAAGCGATCTGGA
GTATCAGCTAGAAAACATTCAGGTTCTTTATTCTCATGAAAAGGTGAAAATGGAAGGCACCATTTCTCAA
CAAACCAAACTCATTGATTTTCTGCAAGCCAAAATGGATCAACCTGCTAAAAAGAAAAAGGGTTTATTTA
GTCGACGGAAAGAGGACCCTGCTTTGCCCACACAGGTTCCTCTGCAGTACAATGAGCTGAAGCTGGCCCT
GGAGAAGGAGAAAGCTCGCTGTGCAGAGCTAGAGGAAGCCCTTCAGAAGACCCGCATTGAGCTCCGGTCG
GCCCGGGAGGAAGCTGCCCACCGAAAAGCCACAGACCACCCGCACCCATCCACGCCAGCCACCGCGAGGC
AGCAGATTGCCATGTCTGCCATCGTGCGGTCACCTGAGCACCAGCCCAGTGCCATGAGCCTGCTGGCCCC
ACCATCCAGCCGCAGAAAGGAGTCTTCAACTCCAGAGGAATTTAGTCGGCGTCTTAAGGAGCGCATGCAC
CACAATATTCCTCACCGATTTAACGTAGGACTAAACATGCGAGCCACAAAGTGTGCTGTGTGTCTGGATA
CCGTGCACTTCGGACGCCAGGCATCCAAATGTCTTGAATGTCAGGTGATGTGTCATCCCAAGTGCTCCAC
ATGCTTGCCAGCCACCTGTGGCCTGCCTGCTGAATATGCCACACACTTCACTGAGGCCTTCTGCCGTGAC
AAAATGAACTCCCCGGGTCTCCAGACCAAGGAGCCCAGCAGCAGCTTACATCTGGAAGGGTGGATGAAGG
TGCCCAGGAATAACAAGCGAGGACAGCAAGGGTGGGACAGGAAGTACATTGTCCTGGAGGGATCAAAAGT
TCTCATTTATGATAATGAAGCCAGAGAAGCTGGACAGAGGCCGGTGGAAGAATTTGAGCTGTGCCTTCCC
GACGGGGATGTATCTATTCATGGTGCCGTTGGTGCTTCCGAACTTGCAAATACAGCCAAAGCAGATGTCC
CATACATACTGAAGATGGAATCTCACCCGCACACCACCTGCTGGCCTGGGAGAACCCTCTACTTGTTAGC
TCCCAGCTTCCCTGACAAACAGCGCTGGGTCACCGCCTTAGAATCTGTTGTCGCAGGTGGGAGAGTTTCT
AGGGAAAAAGCAGAAGCTGATGCTAAATTACTTGGAAACTCCCTGCTGAAATTGGAAGGTGATGACCGTC
TAGACATGAACTGCACACTGCCCTTCAGTGACCAGGTGGTGTTGGTGGGCACTGAGGAAGGGCTCTATGC
ACTGAACGTCTTGAAAAACTCCCTTACCCACGTCCCAGGAATTGGAGCAGTCTTCCAAATTTATATTATC
AAGGACCTGGAGAAGCTACTCATGATAGCAGGAGAAGAGCGGGCGCTGTGTCTCGTGGACATAAAGAAAG
TGAAACAGTCCTTGGCACAGTCTCACCTTCCTGCCCAGCCGGACATTTCGCCCAACATTTTCGAAGCTGT
CAAGGGCTGCCACCTCTTTGCTGCTGGCAAGATTGAGAATGGGCTCTGCATCTGTGCAGCCATGCCTAAC
AAAGTCGTTATTCTTCGCTACAACGAAAACCTCAGCAAATACTGCATTCGGAAGGAGATAGAGACCTCAG
AGCCCTGCAGCTGTATCCACTTCACCAATTACAGTATCCTCATTGGAACCAATAAATTCTACGAAATCGA
CATGAAGCAGTACACACTCGAGGAATTCCTGGATAAGAATGACCATTCCTTGGCACCTGCCGTATTTGCC
TCCTCTTCCAACAGTTTCCCTGTCTCCATCGTGCAGGTGAACAGTGCAGGCCAGCGAGAGGAGTATTTGC

TGTGTTTCCACGAATTTGGGGTGTTTGTGGATTCTTACGGAAGACGAAGCCGCACAGACGATCTCAAGTG
GAGTCGCTTACCTTTGGCCTTCGCCTACAGAGAACCTTATCTGTTTGTGACCCACTTCAACTCACTCGAA
GTAATTGAGATCCAGGCCCGCTCGTCACTGGGGACCCCTGCCCGAGCATATTTGGAAATCCCGAATCCAC
GCTACCTAGGCCCTGCAATTTCCTCGGGAGCAATTTACCTGGCATCCTCGTACCAGGATAAATTAAGGGT
CATCTGCTGCAAAGGAAACCTTGTGAAGGAGTCGGGCACTGACCACCATCGGGTCCCTTCCACCTCCCGC
AGCAGCCCCAACAAGCGAGGCCCGCCCACGTACAATGAGCACATCACCAAGCGTGTGGCCTCCAGCCCAG
CGCCACCTGAAGGGCCCAGCCACCCCCGAGAGCCAAGCACACCCCACCGCTACCGTGAGGGGCGGACAGA
GCTGCGCAGGGACAAGTCTCCTGGCCGCCCCCTGGAGCGGGAGAAGTCCCCGGGCCGCGTGCTGAGCACA
CGGAGGGAGCGGTCCCCCGGGAGGCTATTTGAAGACAGCAGCAGGGGCCGGCTGCCTGCAGGAGCCGTGA
GGACCCCACTGTCCCAGGTCAATAAGGTCTGGGACCAGTCTTCAGTA
>Microcebus murinus XM_012756541.1
ATGTTGAAGTTCAAGTATGGAGCACGGAATCTACTGGATGCTGGTGCCGTGGAACCCATTGCCAGCCGGG
CCTCCAGGCTGAATCTTTTCTTCCAGGGGAAACCACCCTTTATGACTCAACAGCAGATGTCTCTTCTTTC
CCGAGAAGGGATATTAGATGCCCTCTTTGTTCTCTTTGAAGAATGCAGTCAGCCTGCTCTGATGAAGATT
AAGCATGTGAGCAACTTCGTCCGGAAGTATTCTGACACCGTAGCCGAGTTGCAGGAGCTCCAGCCTTCGG
CAAAGGACTTTGAAGTCAGAAGTCTCGTAGGTTGTGGTCACTTTGCTGATGTGCAGGTGGTAAGAGAGAA
AGCAACTGGGGACATCTATGCCATGAAAGTCATGAAGAAGAAGGCCTTATTGGCCCAGGAGCAGGTTTCA
TTTTTTGAGGAAGAACGGAACATTTTATCTCGAAGCACAAGCCCTTGGATCCCCCAATTACAGTATGCCT
TTCAGGACAAAAATAACCTGTATCTGGTCATGGAATATCAGCCTGGAGGGGACTTGTTGTCACTATTGAA
CAGATACGAGGATCAATTAGATGAAAATATGATTCAGTTTTACCTAGCCGAACTGATTTTGGCTGTTCAC
AGTGTTCATCAGATGGGATATGTACATCGAGACATCAAGCCTGAGAACATTCTCATTGACCGAACGGGAC
ACATCAAACTGGTGGATTTTGGATCAGCTGCTAAAATGAATTCAAACAAGATGGTTATTGCCAAACTCCC
GATCGGGACCCCAGATTACATGGCTCCTGAAGTGTTGACCGTGATGAATGGGGACGCGAAGGGCACCTAC
AGCCTGGACTGTGACTGGTGGTCGGTGGGCGTGATCGCCTACGAGATGATTTACGGGCGATCCCCCTTCA
CGGAGGGAACTTCAGCCAGAACCTTCAGTAACATCATGAATTTCCAGCGGTTTTTGAAGTTTCCAGATGA
CCCCAAAGTTAGCAGCGAATTCCTTGATCTGATTCAAAGTTTGTTGTGCGGCCAGAAAGAGAGACTGAAG
TTCGAAGGTCTTTGCTGCCATCCTTTCTTCTCTAAAATTGACTGGAATAACATCCGTAACTCTCCTCCCC
CCTTCGTTCCCACCCTCAAGTCTGACGATGACACCTCCAATTTTGATGAACCAGAGAAGAATTCGTGGGT
TTCATCCTCTTCATGCCAGCTGAGCCCCTCGGGTTTCTCAGGCGAAGAACTGCCGTTTGTGGGGTTTTCG
TACAGCAAGGCACTGGGGATTCTTGGTAGATCTGAGTCTGTTGTTTCGGGTCTGGACTCCCCTGCCAAGA
CTAGCTCCATGGAAAAGAAACTTCTCATCAAAAGCAAAGAGCTACAAGACTCTCAGGACAAGTGTCACAA
GATGGAGCAGGAAATGACCCGGTTACATCGGAGAGTGTCAGAGGTGGAGGCTGTGCTTAGTCAGAAGGAG
GTGGAGCTGAAGGCCTCTGAGACTCAGAGATCCCTCCTGGAGCAGGACCTTGCTACCTACATCACAGAAT
GCAGTAGCTTAAAGCGAAGTTTGGAGCAAGCCCGGATGGAGGTGTCCCAGGAGGATGACAAAGCACTGCA
GCTTCTCCATGATATCAGAGAGCAGAGCCGGAAGCTCCAAGAAATCAAAGAGCAGGAATACCAGGCTCAG
GTGGAGGAAATGAGGCTGATGATGAACCAGTTGGAAGAGGACCTTGTCTCTGCAAGAAGACGGAGTGATC
TCTACGAGTCTGAGCTGAGAGAGTCTCGGCTCGCCGCGGAAGAATTCAAGCGGAAAGCTACAGAATGTCA
GCATAAGCTGTTGAAGTCTAAGGATCAAGGGAAGCCTGAAGTAGGAGAATATTCCAAACTGGAAAAGATC
AATGCTGAGCAGCAGCTCAAAATTCAGGAGCTCCAAGAGAAGCTGGAAAAGGCCGTAAAAGCCAGCACAG
AGGCCACCGAGCTGCTGCAGAACATCCGCCAGGCCAAGGAGCGCGCCGAGAGGGAGCTGGAGAAGCTGCA
GAACCGTGAAGACTCTTCCGAAGGCATAAGAAAGAAGCTGGCGGAAGCCGAGGAACGCCGCCATTCTCTG
GAGAACAAGGTAAAGAGACTAGAGACCATGGAGCGTAGAGAAAACAGACTGAAGGATGACATCCAGACAA
AATCCCAACAGATCCAGCAGATGGCTGATAAAATTCTGGAGCTGGAGGAGAAACACCGGGAGGCCCAAGT
CTCAGCCCAGCACCTAGAGGTGCACCTGAAACAGAAAGAGCAACACTATGAGGAAAAAATTAAGGTATTG
GACAATCAGATAAAGAAAGACCTGGCCGATAAGGAGACCCTGGAGAATATGATGCAGAGGCACGAGGAGG
AGGCCCACGAGAAAGGCAAAATTCTCAGCGAGCAGAAGGCGATGATCAATGCTATGGATTCCAAGATCAG
ATCCCTGGAACAGAGGATTGTGGAACTGTCTGAAGCCAACAAACTCGCGGCAAACAGCAGCCTTTTTACC

CAAAGGAACATGAAGGCCCAAGAAGAGATGATTTCTGAACTTAGGCAACAGAAATTTTACCTGGAGACAC
AGGCCGGGAAATTGGAGGCCCAGAACCGAAAGCTGGAAGAGCAGCTGGAGAAAATCAGCCACCAAGATCA
CAGTGACAAGAATCGGCTCCTGGAACTGGAGACAAGGTTGAGGGAGGTCAGCCTAGAGCACGAGGAGCAG
AAACTAGAGCTGAAGCGCCAGTTGACGGAGCTGCAGCTGTCCCTGCAGGAGCGTGAGTCGCAGCTGACGG
CCCTGCAGGCTGCTCGGGCAGCCCTGGAGAGCCAGCTTCGCCAGGCCAAGACAGAGCTGGAGGAGACAAC
AGCGGAAGCTGAAGAGGAGATCCAGGCGCTCACGGCACATAGAGATGAAATCCAGCGCAAATTTGATGCC
CTTCGTAACAGCTGTACTGTAATCACAGACCTGGAGGAGCAGCTAAACCAGCTGACCGAGGACAACGCTG
AACTCAACAACCAAAACTTCTACTTGTCCAAACAACTCGACGAGGCTTCCGGCGCCAACGATGAGATAGT
GCAACTGCGAAGTGAAGTAGACCACCTCCGCCGTGAGATCACAGAGAGGGAGATGCAGCTCACCAGCCAG
AAGCAAACGATGGAGGCTCTGAAGACCACTTGCACGATGCTGGAAGAACAGGTCATGGATTTGGAGGCCC
TAAACGACGAGCTGCTGGAAAAAGAGCGGCAGTGGGAGGCCTGGAGGAGCGTCCTCGGTGATGAGAAGTC
CCAGTTCGAGTGTCGGGTTCGAGAGTTACAGAGGATGCTGGACACTGAGAAACAGAGCAGGGCAAGAGCC
GACCAGCGGATCACCGAGTCCCGCCAGGTGGTGGAGCTGGCAGTGAAGGAACACAAGGCCGAGATTCTCG
CCCTGCAGCAGGCTCTCAAAGAACAGAAGCTGAAAGCTGAGAGTCTCTCCGACAAGCTCAATGACCTGGA
GAAGAAGCATGCCATGCTAGAAATGAACGCCCGAAGCTTACAGCAGAAACTAGAGACTGAACGAGAGCTC
AAACAAAGGCTTCTGGAAGAGCAAGCCAAATTACAGCAGCAGATGGACTTGCAGAAGAATCACATTTTCC
GTCTGACACAAGGGCTGCAAGAAGCTCTAGATCGAGCTGATCTGCTGAAGACAGAAAGAAGTGATCTGGA
GTATCAGCTAGAAAACATTCAGGTTCTCTATTCTCATGAAAAGGTGAAAATGGAAGGCACCATTTCTCAA
CAAACCAAACTCATTGATTTTCTGCAAGCCAAAATGGACCAACCTGCTAAAAAGAAAAAGGGTTTATTTA
GTCGACGGAAAGAGGACCCTGCTTTGCCCACACAGGTTCCTCTGCAATACAATGAATTGAAGCTGGCCCT
GGAGAAGGAGAAAGCTCGCTGTGCAGAGCTAGAGGAAGCCCTTCAGAAGACCCGCATTGAGCTCCGGTCC
GCCCGGGAGGAAGCTGCCCACCGAAAAGCCACGGACCACCCTCACCCGTCCACGCCAGCCACCGCGAGGC
AGCAGATCGCCATGTCTGCCATTGTGCGGTCACCTGAGCACCAGCCTAGTGCCATGAGCCTGCTGGCCCC
GCCGTCCAGCCGCAGAAAGGAGTCTTCAACTCCAGAGGAATTTAGTCGGCGTCTGAAGGAGCGCATGCAT
CACAATATTCCTCACCGATTCAACGTGGGACTGAACATGAGAGCCACAAAGTGTGCTGTGTGTCTGGATA
CCGTGCACTTCGGACGGCAGGCATCCAAATGCCTCGAATGTCAGGTGATGTGCCATCCCAAGTGCTCCAC
ATGCTTGCCAGCCACCTGTGGCCTGCCTGCCGAATATGCCACACACTTCACCGAGGCCTTCTGCCGTGAC
AAAATGAACTCCCCAGGACTCCAGACCAAGGAGCCCAGCAGCAGCTTGCACCTGGAAGGGTGGATGAAGG
TGCCCAGGAATAACAAACGAGGACAGCAAGGCTGGGACCGGAAGTACATTGTCCTGGAGGGATCAAAAGT
CCTCATTTATGACAATGAAGCCCGAGAAGCTGGACAGAGGCCGGTGGAAGAATTTGAGCTGTGCCTTCCC
GACGGGGATGTATCTATTCATGGTGCCGTTGGTGCTTCTGAACTCGCAAATACAGCCAAAGCAGATGTCC
CATACATACTGAAGATGGAATCTCACCCGCACACCACCTGCTGGCCCGGGAGAACCCTCTACTTGCTAGC
TCCCAGCTTCCCCGACAAACAGCGCTGGGTCACCGCCTTAGAATCCGTTGTCGCAGGTGGGAGAGTTTCT
AGGGAAAAAGCAGAAGCCGATGCTAAATTACTTGGAAACTCCCTGCTGAAACTGGAAGGTGATGACCGTC
TAGACATGAACTGCACACTGCCCTTCAGTGACCAGGTGGTGTTGGTGGGCACCGAGGAAGGGCTCTATGC
ACTGAATGTCTTGAAAAACTCCCTAACCCATGTCCCAGGAATCGGAGCAGTCTTCCAAATTTACATCATC
AAGGACCTGGAGAAGCTACTCATGATAGCAGGAGAAGAGCGGGCGCTGTGTCTCGTGGACGTGAAGAAAG
TGAAACAGTCCCTGGCACAGTCCCACCTTCCTGCCCAGCCGGACATCTCGCCCAACATCTTCGAAGCTGT
CAAGGGCTGCCACTTGTTTGCCGCCGGCAAGATTGAGAACGGGCTCTGCATCTGCGCGGCCATGCCCAGC
AAAGTCGTTATTCTCCGCTACAACGAAAACCTCAGCAAATACTGCATCCGGAAAGAGATAGAGACCTCAG
AGCCCTGCAGCTGTATCCACTTCACCAATTACAGTATCCTCATTGGAACCAATAAATTCTACGAAATCGA
CATGAAGCAGTACACACTTGAGGAATTCCTGGATAAGAATGACCATTCCCTGGCGCCTGCCGTGTTTGCC
GCCTCTTCCAACAGCTTCCCTGTCTCGATCGTGCAGGTGAACGGCGCAGGGCAGCGGGAGGAGTATTTGC
TGTGCTTCCACGAATTTGGGGTGTTCGTGGATTCCTACGGAAGACGGAGCCGCACGGACGATCTCAAGTG
GAGTCGCTTACCTTTGGCCTTTGCCTACAGAGAACCCTATTTGTTTGTGACCCACTTCAACTCACTCGAA
GTAATCGAGATCCAGGCCCGCTCATCACTGGGGACTCCTGCCCGAGCATACTTGGAAATCCCGAATCCAC
GCTACCTGGGCCCCGCGATTTCCTCGGGAGCAATTTACCTGGCGTCCTCGTACCAGGATAAATTAAGGGT

CATCTGCTGCAAAGGAAACCTCGTGAAGGAGTCCGGCACTGACCACCACCGGGTCCCTTCCACCTCCCGC
AGCAGCCCCAATAAGCGAGGCCCACCCACGTACAACGAGCACATCACCAAGCGCGTGGCCTCCAGCCCGG
CGCCACCGGAAGGCCCCAGCCACCCCCGAGAGCCAAGCACACCCCACCGCTACCGCGAGGGGCGGACAGA
GCTGCGCAGGGACAAGTCTCCTGGCCGCCCCCTGGAGCGGGAGAAGTCCCCGGGCCGCATGCTCAGCACG
CGGAGGGAGCGGTCCCCCGGGAGGCTGTTCGAAGACAGCAGCAGGGGCCGGCTGCCCGCGGGAGCCGTGA
GGACCCCGCTGTCCCAGGTCAACAAGGTCTGGGACCAGTCTTCGGTA
>Carlito syrichta XM_021718196.1
ATGTTGAAGTTCAAGTATGGAGCGTGGAATCCACTGGATGCTGGTGCTGCTGAACCCATTGCCAGCCGGG
CCTCCAGGCTGAATCTATTCTTCCAGGGGAAACCACCCTTTATGACTCAACAGCAGATGTCTCCTCTTTC
CCGAGAAGGGATATTAGATGCCCTCTTTGTTCTCTTTGAAGAATGCAGTCAGCCTGATCTGATGAAGATT
AAGCATGTGAGCAACTTTGTCCGGAAATATTCTGAAACCATAGCTGAGTTACAGGAGCTCCAGCCTTCCG
CAAGGGACTTTGAGATCAGAAGTCTAGTAGGTTGTGGTCACTTTGCTGAAGTGCAGGTGGTAAGAGAGAA
AGCAACTGGGGACATTTATGCCATGAAAGTCATGAAGAAGAAGGCCTTATTGGCCCAGGAGCAGGTTTCA
TTTTTTGAGGAAGAACGGAACATATTATCTCGAAGCACAAGCCCTTGGATCCCCCAATTACAGTATGCCT
TTCAGGACAAAAATAACCTTTATCTGGTCATGGAATATCAGCCTGGAGGGGACTTGCTGTCACTTTTGAA
TAGATATGAGGACCAATTAGATGAAAATATGATTCAGTTTTACCTAGCCGAACTGATTTTGGCTGTTCAC
AGCGTTCATCAAATGGGATATGTACATCGAGACATCAAGCCTGAGAACATTCTCATTGACCGAACAGGAC
ACATCAAGCTGGTGGATTTTGGATCAGTAGCTAAAATGAACTCAAACACGATGGTGAGTGCCAAACTCCC
GATTGGGACCCCAGATTACATGGCTCCTGAAGTGTTGACTGTGATGAACGGGGATGGAAAAGGCATCTAT
GGTCTAGACTGTGACTGGTGGTCGGTGGGCGTGATTGCTTATGAGATGATTTATGGGAGATCCCCATTCA
CAGAGGGAACCGCAGCCAGAACCTTCAATAACATCATGAATTTCCAGAGGTTTTTGAAGTTTCCAGATGA
CCCCAAAGTTAGCAATGAATTTCTTGATCTGATTCAAAGTTTGTTGTGTGGCCAGAAAGAGAGACTGAAG
TTTGAAGGTCTTTGCTACCATCCTTTCTTCTCTAAAATTGACTGGAATAACATTCGTAACTCTCCTCCCC
CCTTCGTTCCCACCCTCAAGTCTGATGATGACACCTCCAATTTTGATGAACCAGAGAAGAATTCGTGGGT
TTCATCCTCTCCGTGCCAGCTGAGCCCCTCAGGTTTCTCGGGCGAAGAACTGCCGTTTGTGGGGTTTTCA
TATAGCAAGGCACTGAGGATTCTTGGTAGATCGGAGTCTGTTGTGTCAGGTCTGGACTCCCCTGCCAAGA
CTAGCTCCATGGAAAAGAAACTTCTCATCAAAAGCAAAGAGCTACAAGACTCTCAGGACAAGTGTCACAA
GATGGAGCAGGAAATGACCCGGTTACATCGGAGAGTGTCAGAGGTGGAGGCTGTGCTTAGTCAGAAGGAG
GTGGAGCTGAAGGCCTCTGAGACTCAGAGATCCCTCCTGGAGCAGGACCTTGCTACCTACATCACAGAAT
GCAGTAGCTTAAAGCGAAGTTTGGAGCAAGCACGGATGGAGGTGTCTCAGGAGGATGACAAAGCACTGCA
GCTTCTCCATGATATCAGAGAGCAGAGCCGGAAGCTCCAAGAAATCAAAGAGCAGGAGTACCAGGCTCAA
GTGGAAGAAATGAGGTTGATGATGAATCAGTTGGAAGAGGACCTTGTCTCAGCGAGAAGACGGAGTGATC
TCTACGAATCTGAGCTGAGAGAGTCTCGGCTTGCTGCTGAAGAATTTAAGCGAAAAGCGACAGAATGTCA
GCATAAACTGTTGAAGGCCAAGGATCAAGGGAAGCCTGAAGTAGGAGAATATTCTAAACTGGAAAAGATC
AATGCTGAGCAGCAGCTTAAAATCCAGGAGCTCCAAGAGAAGCTGGAAAAGGCTGTAAAAGCCAGCACAG
AGGCCACCGAGCTGCTGCAGAATATCCGTCAGGCAAAGGAGCGGGCCGAGAGGGAGCTGGAGAAGCTGCA
GAACCGGGAAGATTCTTCTGAAGGCATAAGAAAGAAACTGGTGGAAGCTGAGGAACGCCGCCATTCTCTG
GAGAACAAGGTAAAGAGACTAGAGACCATGGAACGTAGAGAAAACAGACTGAAGGATGACATCCAGACAA
AATCCCAACAGATCCAGCAGATGGCTGATAAAATTCTGGAATTGGAAGAGAAACACCGGGAGGCTCAAGT
CTCTGCCCAACACCTAGAAGTGCACCTGAAACAGAAAGAGCAGCACTACGAGGAAAAAATTAAGGTGTTG
GACAATCAGATAAAGAAAGACCTGGCTGATAAGGAGACTCTGGAGAATATGATGCAGAGACACGAGGAGG
AAGCCCATGAGAAAGGCAAAATTCTCAGCGAGCAGAAGGCGATGATCAATGCTATGGATTCCAAGATTAG
ATCTCTGGAACAGAGGATTGTGGAGCTCTCTGAAGCCAATAAACTTGCAGCAAACAGCAGTCTTTTTACC
CAAAGGAACATGAAGGCCCAAGAAGAGATGATTTCTGAACTCAGGCAACAGAAATTTTACCTGGAGACAC
AGGCTGGAAAATTGGAGGCCCAGAACCGAAAGCTGGAAGAACAGCTGGAAAAAATCAGCCACCAAGACCA
CAGTGACAAGAATCGGCTGCTGGAACTGGAGACAAGGTTGAGGGAGGTCAGTCTAGAACATGAGGAGCAG
AAATTAGAGCTCAAGCGCCAGCTCACAGAGCTGCAGCTCTCCCTGCAGGAGCGTGAGTCACAGCTGACAG

CTCTGCAGGCTGCTCGGGCGGCCCTGGAGAGCCAGCTTCGCCAGGCGAAGACGGAACTGGAAGAGACAAC
AGCAGAAGCTGAAGAGGAGATCCAGGCGCTCACGGCACATAGAGATGAAATCCAGCGCAAATTTGATGCC
CTTCGTAACAGCTGTACTGTAATCACAGACCTGGAAGAGCAGCTAAACCAACTGACTGAGGACAATGCTG
AACTCAACAACCAAAACTTCTACCTGTCCAAACAACTTGATGAGGCTTCTGGCGCCAACGATGAGATAGT
CCAACTTCGAAGTGAAGTGGACCACCTCCGCCGGGAGATCACAGAAAGGGAGATGCAACTCACCAGCCAG
AAGCAGACAATGGAGGCTCTGAAGACCACATGCACAATGTTGGAAGAACAGGTCATGGATTTGGAGGCCT
TGAACGATGAACTCTTGGAAAAAGAACGGCAGTGGGAGGCCTGGAGGAGCGTTCTCGGTGATGAGAAGTC
CCAGTTTGAGTGTCGAGTTCGAGAGTTACAGAGGATGCTGGACACTGAGAAACAGAGCAGGGCCAGAGCT
GATCAGCGGATAACTGAGTCTCGCCAGGTGGTGGAGCTGGCAGTAAAGGAGCACAAGGCTGAGATTCTTG
CTTTGCAGCAGGCTCTCAAAGAACAGAAGCTGAAAGCCGAAAGCCTCTCTGACAAGCTCAATGACCTGGA
GAAGAAGCATGCCATGCTGGAGATGAACGCCCGAAGCTTACAGCAGAAACTGGAGACTGAACGAGAGCTC
AAACAAAGGCTTCTGGAGGAGCAAGCCAAATTACAGCAGCAGATGGACCTGCAGAAGAATCACATTTTCC
GTCTGACTCAAGGGCTGCAAGAAGCTTTAGATCGGGCTGATCTGCTGAAGACAGAAAGAAGTGACCTGGA
GTATCAGCTAGAAAACATTCAGGTTCTCTACTCTCATGAAAAAGTGAAAATGGAAGGCACTATTTCTCAG
CAAACCAAACTTATTGATTTTCTACAAGCCAAAATGGACCAACCTGCTAAAAAGAAAAAGGGTTTATTTA
GTCGACGGAAAGAGGACCCTGCTTTGCCCACACAGGTTCCTCTGCAGTACAATGAGCTGAAGCTGGCCCT
GGAGAAGGAGAAAGCTCGCTGTGCAGAGCTAGAGGAAGCCCTTCAGAAGACCCGCATCGAGCTCCGGTCC
GCCCGGGAGGAAGCCGCCCACCGCAAAGCCACGGACCACCCGCACCCATCCACGCCAGCCACTGCAAGGC
AGCAGATTGCCATGTCCGCCATCGTGCGGTCGCCTGAGCACCAGCCCAGTGCCATGAGCCTGCTGGCCCC
GCCGTCCAGCCGCAGAAAGGAGTCTTCAACTCCAGAGGAATTTAGTCGGCGTCTTAAGGAACGCATGCAC
CACAATATTCCTCACCGATTTAACGTAGGACTGAACATGCGAGCCACAAAGTGTGCCGTGTGTCTGGATA
CTGTGCACTTTGGACGCCAGGCATCCAAATGTCTTGAATGTCAGGTGATGTGTCATCCCAAGTGCTCCAC
GTGCTTGCCAGCCACCTGCGGCCTGCCTGCTGAATATGCCACACACTTCACTGACGCCTTCTGCCGTGAC
AAAATGAACTCCCCAGGTCTCCAGACCAAGGAGCCCAGCAGCAGCTTGCACCTGGAAGGGTGGATGAAGG
TGCCCAGGAATAACAAACGAGGACAGCAAGGCTGGGACAGGAAGTACATTGTCCTGGAGGGATCCAAAGT
CCTCATTTATGACAATGAAGCCAGAGAAGCTGGACAGAGGCCGGTGGAAGAATTTGAGCTGTGCCTTCCC
GATGGGGATGTATCTATTCATGGTGCCGTTGGTGCTTCTGAACTCGCAAATACAGCCAAAGCAGATGTCC
CATACATACTGAAGATGGAGTCTCACCCGCACACCACCTGCTGGCCCGGGAGAACCTTCTACTTGCTAGC
TCCCAGCTTCCCCGACAAACAGCGCTGGGTCACCGCCTTAGAATCAGTTGTCGCAGGTGGGAGAGTTTCT
AGGGAAAAAGCAGAAGCCGATGCTAAATTGCTTGGAAACTCCCTGCTGAAACTGGAAGGTGATGACCGTC
TAGACATGAACTGCACACTGCCCTTCAGTGACCAGGTGGTGTTGGTGGGCACTGAGGAAGGGCTCTATGC
ACTGAATGTCTTGAAAAACTCCCTAACCCACGTCCCAGGAATTGGAGCAGTCTTCCAAATTTATATTATC
AAGGACCTGGAGAAGCTACTCATGATAGCAGGAGAAGAGCGGGCCCTGTGTCTCGTGGACGTGAAGAAAG
TGAAACAGTCCCTAGCACAGTCTCATCTTCCCGCCCAGCCAGACATCTCACCCAATGTTTTTGAAGCTGT
CAAGGGCTGCCACTTGTTTGGGGCTGGCAAGATTGAGAATGGGCTTTGTATCTGCGCAGCCATGCCCAGC
AAAGTTGTCATTCTCCGCTACAACGAAAACCTCAGCAAATACTGCATTCGGAAAGAGATAGAGACCTCAG
AGCCCTGCAGCTGTATCCACTTCACCAATTACAGTATCCTCATTGGAACCAATAAATTCTACGAAATTGA
CATGAAGCAGTACACGCTCGAGGAATTCTTGGATAAGAACGACCATTCCTTGGCACCTGCTGTGTTTGCT
GCTTCTTCCAACAGTTTCCCTGTCTCGATCGTGCAGGTGAACAGCACGGGGCAGCGAGAAGAGTACTTGC
TGTGCTTCCATGAATTTGGGGTGTTCGTGGATTCTTATGGAAGACGTAGCCGCACAGATGATCTCAAGTG
GAGTCGCTTACCTTTGGCCTTTGCCTACAGAGAGCCCTATCTGTTTGTGACCCACTTCAACTCACTTGAA
GTAATTGAGATCCAGGCACGCTCCTCGCTGGGGACCCCTGCCCGAGCTTATCTGGAAATCCCGAACCCAC
GCTACCTGGGCCCCGCAATTTCCTCAGGAGCAATCTACCTGGCATCCTCATACCAAGATAAGTTAAGAGT
TATTTGCTGCAAAGGAAACCTCGTGAAGGAGTCTGGCACTGAACACCACCGGGTCCCTTCCACCTCCCGC
AGCAGCCCCAACAAGCGAGGCCCACCCACATACAACGAGCACATCACCAAGCGTGTGGCCTCCAGCCCAG
CCCCGCCCGAAGGTCCCAGCCATCCCCGAGAGCCAAGCACACCCCACCGCTACCGTGAGGGGCGGACAGA
GCTGCGCAGGGACAAGTCTCCAGGCCGCCCCCTGGAGCGGGAGAAGTCCCCGGGCCGGATGCTCAGCACG

CGGAGGGAGCGGTCCCCCGGGAGGCTGTTTGAAGACAGCAGCAGGGGCCGGCTGCCAGCGGGAGCTGTGA
GGACCCCACTGTCCCAGGTTAACAAGGTCTGGGACCAGTCTTCAGTA
>Ictidomys tridecemlineatus XM_005336820.2
ATGCTGAAGTTCAAGTATGGATCACGGAATACACAGGATGCTGGTGCCGTTGAGCCCATTGCCAGCCGGG
CCTCCAGGCTGAATCTCTTCTTCCAGGGGAAACCACCCTTTATGACTCAACAGCAGATGTCTCCTCTTTC
CCGAGAAGGGATATTAGATGCCCTCTTTGTTCTCTTTGAAGAATGCAGTCAGCCTGCTCTGATGAAGATT
AAGCACGTGAGCAACTTCGTCCGGAAGTATTCTGACATCATAGCTGAGTTACAGGAGCTCCAGCCTTCGG
CAAAGGACTTTGAAGTCAGAAGTCTTGTAGGTTGTGGCCACTTTGCTGAAGTGCAGGTGGTAAGAGAGAG
AGCAACTGGGGACATCTATGCCATGAAAATCATGAAGAAGAAGGCCTTGTTGGCCCAGGAGCAGGTTTCA
TTTTTTGAGGAAGAACGGAACATATTATCTCGGAGTACAAGCCCTTGGATCCCCCAATTACAGTATGCCT
TCCAGGATAAAAATAACCTTTACCTGGTCATGGAATATCAGCCTGGAGGGGATTTGCTGTCACTTTTGAA
TAGATATGAGGACCAATTAGATGAAAACATGATTCAATTTTACCTAGCTGAACTGATTTTGGCTGTTCAC
AGTGTTCATCAGATGGGATATGTACACCGCGACATCAAGCCAGAGAATGTTCTCATTGACCGAACAGGAC
ACATCAAGTTGGTGGATTTTGGATCAGCTGCTAAAATGAATTCAAATCAAATGGTGAACTCCAAACTCCC
GATTGGCACCCCAGATTACATGGCTCCTGAAGTGCTGACCATTATGAATGGGGACGGGAAAGGTGTCTAC
GGTCCAGACTGTGACTGGTGGTCCGTGGGAGTCATTGCCTACGAAATGGTTTATGGGAGAACCCCGTTCA
CGGAGGGAACCTCAGCCAGAACCTTCAATAACATCATGAACTTCCAGCGGTTTTTGAAATTTCCGGATGA
TCCCAAAGTGAGCAGTGGATTCCTTGATCTCATTCAAAGTTTGTTGTGTGGCCAGAGAGAGAGGTTGAAG
TTTGAGGGCCTCTGTTGCCACCCTTTCTTCTCTAAAATCGACTGGAATGACATTCGTAACTCGCCTCCCC
CCTTCGTCCCCACCCTCAAGTCTGACGATGACACCTCCAATTTTGATGAACCAGAGAAGAATTCGTGGGT
TTCATCCTCTCTGTGCCAGCTGAGCCCCTCGGGTTTCTCGGGTGAAGAACTGCCGTTTGTGGGGTTTTCG
TACAGCAAGGCACTGGGGATTCTTGGTAGATCTGAGTCTGTTGTGTCGGGTCTGGACTCCCCTGCCAAGA
CTAGCTCCATGGAAAAGAAACTTCTCATCAAAAGCAAAGAGCTGCAAGACTCTCAGGACAAGTGTCACAA
GATGGAGCAGGAAATGACCCGGTTACATCGGAGAGTGTCAGAGGTGGAGGCTGTGCTTAGTCAGAAGGAG
GTGGAGCTGAAGGCCTCTGAGACTCAGAGATCCCTCCTGGAGCAGGACCTTGCCACCTACATCACAGAAT
GCAGTAGCTTAAAGCGAAGTTTGGAGCAAGCACGGATGGAGGTGTCCCAGGAGGATGACAAAGCACTGCA
GCTTCTCCATGACATCAGAGAGCAGAGCCGGAAACTCCAAGAAATCAAAGAGCAGGAGTACCAGGCTCAA
GTGGAAGAAATGAGGCTGATGATGAATCAGTTGGAAGAGGATCTTGTCTCAGCAAGAAGACGGAGCGATC
TGTACGAATCTGAGCTGAGAGAGTCTCGGCTTGCTGCCGAAGAGTTCAAGCGGAAAGCGACAGAATGTCA
GCATAAACTGATGAAGGCTAAGGATCAAGGGAAGCCTGAAGCAGGAGAATATTCCAAACTTGAGAAGATC
AATGCCGAGCAGCAGCTCAAAATCCGGGAGCTCCAAGAAAAGCTGGAAAAGGCGGTAAAAGCCAGCACAG
AGGCCACTGAGCTGCTGCAGAACATCCGCCAGGCCAAGGAGCGAGCCGAGAGGGAGCTGGAGAAGCTGCA
GAACCGAGAAGACTCTTCTGAAGGCATAAGAAAGAAGCTGGTGGAAGCTGAGGAACGCCGCCATTCTCTG
GAGAACAAGGTAAAGAGACTAGAGACCATGGAGCGTAGAGAAAACAGACTGAAGGATGACATCCAGACAA
AATCCCAACAGATCCAGCAGATGGCTGATAAAATTCTGGAGCTGGAGGAGAAACACCGGGAGGCCCAAGT
CTCAGCCCAGCACCTAGAAGTACACCTGAAGCAGAAAGAACAGCACTACGAGGAAAAAATTAAAGTGTTG
GACAATCAGATAAAGAAAGACTTGGCAGATAAGGAGAGCCTGGAGAACTTGATGCAGAGACACGAGGAGG
AGGCCCATGAGAAGGGCAAGATTCTCAGCGAGCAGAAGGCGATGATCAATGCTATGGATTCCAAGATCAG
ATCCCTGGAACAGAGGATTGTGGAACTGTCTGAAGCCAATAAACTGGCAGCAAATAGCAGTCTCTTCACC
CAAAGGAACATGAAGGCCCAGGAAGAGATGATTTCAGAACTCAGGCAGCAGAAGTTTTACCTGGAGACGC
AGGCTGGGAAATTGGAGGCCCAGAACCGAAAGCTGGAAGAACAGCTGGAGAAAATCAGCCACCAAGATCA
CAGTGACAAGAGTCGGCTGCTGGAGCTGGAGACCAGGCTGAGGGGGGTCAGCCTGGAGCATGAGGAGCAG
AAACTGGAACTGAAGCGCCAGCTCACGGAGCTGCAGCTGTCCTTGCAGGAGCGCGAGTCCCAGCTGACAG
CCCTGCAGGCCGCCCGGGCTGCCCTGGAGAGCCAGCTTCGGCAAGCTAAGACAGAGCTGGAGGAGACTAC
AGCAGAAGCGGAAGAGGAGATCCAGGCTCTCACGGCGCATAGAGATGAAATCCAGCGCAAATTTGATGCC
CTTCGTAACAGCTGTACTGTCATCACAGACCTGGAGGAGCAGCTAAACCAGCTGACTGAGGACAACGCTG
AACTCAACAACCAAAACTTCTATCTGTCCAAACAACTCGATGAGGCATCTGGTGCCAATGATGAGATAGT

CCAGCTTCGAAGTGAGGTGGACCATCTTCGCCGCGAGATCACCGAGAGGGAGATGCAGCTCACCAGCCAG
AAGCAAACGATGGAGGCTCTGAAGACCACGTGCACGATGCTGGAGGAGCAGGTCATGGATTTGGAGGCCC
TGAATGATGAGCTGCTGGAGAAGGAGCGCCAGTGGGAGGCCTGGAGGAGTGTCCTTGGTGACGAGAAGTC
CCAGTTTGAGTGTCGGGTTCGAGAGTTACAGAGGATGCTGGACACGGAGAAGCAGAGCAGGGCGAGAGCT
GACCAGCGGATCACTGAGTCTCGCCAGGTGGTGGAGTTGGCAGTGAAAGAGCACAAGGCTGAGATTCTCG
CTCTGCAACAGGCTCTCAAAGAACAGAAGCTGAAAGCTGAGAGCCTCTCTGACAAGCTCAATGACCTGGA
GAAGAAGCACGCCATGCTTGAAATGAATGCCCGAAGCTTGCAGCAGAAACTGGAGACAGAACGAGAGCTC
AAACAGAGGCTTCTGGAAGAGCAAGCCAAGCTACAGCAGCAGATGGACATGCAGAAGAACCACATTTTCC
GTCTGACTCAAGGGCTGCAAGAAGCTTTGGATAGGGCCGATCTGCTGAAGACAGAAAGAAGCGATCTAGA
GTATCAGCTGGAAAACATTCAGGTTCTCTACTCTCATGAAAAGGTGAAGATGGAAGGCACTATTTCTCAA
CAAACCAAACTCATTGATTTTCTGCAAGCCAAAATGGATCAACCTGCTAAAAAGAAAAAGGGTTTATTTA
GTCGACGGAAAGAGGACCCTGCTTTACCCACACAGGTTCCTCTGCAGTACAATGAGCTGAAGCTGGCCCT
GGAGAAGGAAAAAGCTCGCTGTGCAGAGCTGGAGGAAGCCCTTCAGAAGACCCGCATTGAGCTCCGATCT
GCCCGGGAGGAAGCTGCCCACCGCAAAGCCACAGACCACCCACATCCTTCTACTCCAGCCACAGCGAGGC
AGCAGATTGCCATGTCTGCCATTGTGCGGTCACCTGAGCACCAGCCCAGTGCCATGAGCCTGCTTGCCCC
ACCGTCTAGCCGCAGAAAGGAGTCTTCAACCCCAGAAGAATATAGCCGGCGTCTTAAGGAGCGCATGCAC
CACAATATTCCTCACCGATTTAACGTAGGACTGAACATGCGAGCCACAAAGTGTGCTGTGTGTCTGGATA
CTGTACACTTCGGACGCCAGGCATCCAAATGTCTCGAATGTCAGGTGATGTGTCACCCCAAGTGCTCCAC
GTGCTTGCCAGCCACCTGCGGCCTGCCCGCCGAATACGCCACACACTTCACCGAGGCCTTTTGCCGTGAC
AAAATGAACTCCCCGGGTCTGCAGACCAAGGACCCCAGCAGCAGCCTGCACCTGGAAGGGTGGATGAAAG
TGCCCAGGAATAACAAACGAGGACAGCAGGGCTGGGACAGGAAGTACATTGTCCTGGAGGGATCCAAAGT
CCTCATTTATGACAATGAAGCCAGAGAAGCTGGACAGAGGCCGGTGGAAGAATTTGAGCTGTGCCTTCCC
GACGGGGATGTATCTATTCATGGTGCCGTTGGTGCTTCCGAACTCGCAAATACAGCCAAAGCAGATGTCC
CATACATTCTGAAGATGGAATCTCACCCGCACACCACGTGCTGGCCCGGGAGGACCCTCTACCTATTAGC
TCCCAGCTTTCCTGACAAACAGCGCTGGGTCACCGCTTTAGAATCAGTTGTCGCAGGTGGGAGAGTTTCT
AGGGAGAAGGCAGAAGCTGATGCCAAATTGCTTGGAAACTCCCTGCTGAAACTGGAAGGTGATGACCGGC
TGGACATGAACTGCACACTGCCCTTCAGTGACCAGGTGGTGTTGGTGGGCACCGAGGAAGGGCTGTATGC
ACTGAATGTCTTGAAAAACTCCTTAACCCACGTCCCAGGAGTTGGAGCAGTCTTCCAAATTTATATCATC
AAGGACCTGGAGAAGCTACTCATGATAGCAGGAGAAGAGCGGGCCCTGTGTCTGGTGGACGTGAAGAAAG
TGAAGCAGTCCCTGGCGCAGTCGCACCTTCCCGCCCAGCCAGACATCTCGCCCAACATCTTCGAAGCTGT
GAAGGGCTGCCACTTGTTTGCTGCTGGCAAGATTGAGAATGGGCTCTGTATCTGTGCAGCCATGCCCAAC
AAAGTCGTCATCCTTCGCTACAACGAAAACCTCAGCAAGTACTGCATTCGGAAGGAGATCGAGACCTCAG
AGCCCTGCAGCTGCATCCACTTCACCAATTACAGTATCCTCATCGGAACCAACAAATTCTACGAAATTGA
CATGAAGCAGTACACGCTAGAGGAATTCCTGGACAAGAACGACCACTCCTTGGCACCTGCTGTGTTTGCT
TCCTCTTCCAACAGCTTCCCCGTCTCCATCGTGCAGGTGAACGGGGCAGGGCAGCGAGAGGAGTACCTGC
TGTGCTTCCACGAATTTGGGGTGTTCGTGGATTCTTACGGAAGACGTAGCCGCACAGATGATCTCAAGTG
GAGCCGCTTACCCTTGGCCTTTGCCTACAGAGAACCCTATCTGTTTGTGACCCACTTCAACTCACTGGAA
GTCATTGAGATCCAGGCACGCTCCTCGCTGGGGACCCCTGCCCGAGCGTATCTGGAAATCCCGAACCCAC
GTTACCTGGGCCCTGCGATTTCCTCAGGAGCGATTTACCTGGCCTCCTCGTATCAGGATAAATTAAGGGT
CATATGCTGCAAAGGAAACCTTGTGAAGGAGTCCGGCACCGACCACCACCGGGTCCCCTCCACCTCCCGC
AGCAGCCCCAACAAGCGAGGCCCACCGACGTACAACGAGCACATCACCAAGCGCGTGGCCTCCAGCCCGG
CACCACCCGAAGGCCCCAGCCACCCCCGAGAGCCAAGCACACCCCACCGCTACCGCGAGGGGCGGACAGA
GCTACGCAGGGACAAGTCTCCTGGCCGCCCCCTGGAGCGCGAGAAGTCCCCAGGCCGCATGCTTAGCACG
CGGAGGGAGCGGTCCCCTGGGAGGCTGTTTGAAGACAGCAGCAGGGGCCGGCTGCCTGTGGGAGCTGTGA
GGACCCCACTGTCCCAGGTCAACAAGGTCTGGGACCAGTCCTCAGTA
>Rattus norvegicus XM_006249471.3
ATGTTGAAGTTCAAGTATGGAGTGCGGAACCCGTCCGAGGCCAGTGCCCCCGAGCCCATTGCCAGTCGGG

CCTCCAGGCTAAATCTCTTCTTCCAGGGGAAACCGCCCCTCATGACTCAACAGCAGATGTCTGCTCTTTC
CCGGGAAGGGGTGTTAGATGCCCTCTTTGTTCTCTTGGAAGAGTGCAGTCAGCCTGCCCTCATGAAGATA
AAGCACGTGAGCAGCTTCGTCCGGAAGTATTCTGACACGATAGCCGAGTTACGGGAGCTCCAGCCGTCGG
TGAGGGACTTCGAAGTGCGAAGTCTTGTGGGCTGCGGTCACTTCGCCGAAGTGCAGGTGGTTAGAGAGAA
GGCCACCGGGGATGTCTACGCCATGAAGATCATGAAGAAGGCGGCTTTGCGGGCCCAGGAGCAGGTTTCA
TTTTTCGAGGAGGAGAGGAACATATTATCCCAGAGCACGAGCCCTTGGATCCCCCAATTACAGTACGCCT
TTCAGGACAAAAATAACCTTTACCTGGTCATGGAGTATCAGCCTGGAGGGGATTTGCTGTCTCTTCTGAA
TAGATATGAGGACCAGTTAGATGAAAACATGATTCAGTTTTACCTTGCTGAGCTGATCTTGGCTGTCCAC
AGCGTGCACCAGATGGGATATGTGCATCGAGACATCAAGCCGGAGAACATCCTCATTGACCGAACGGGAC
ACATCAAGCTGGTGGATTTTGGATCAGCCGCTAAGATGAATTCAAATAAGGTGGATGCCAAACTCCCTAT
TGGGACCCCAGATTACATGGCTCCGGAAGTGTTGACTGTGATGAACGAGGACCGAAGGGGCACCTACGGC
CTGGACTGTGACTGGTGGTCTGTCGGAGTGGTTGCCTATGAGATGCTTTACGGGAAGACCCCATTCACGG
AGGGAACCTCCGCCCGGACCTTCAACAACATCATGAACTTCCAGCGGTTTTTGAAGTTCCCGGATGACCC
CAAAGTTAGCAGTGAGCTCCTTGATCTGATTCAGAGTTTGCTGTGTGTCCAGAAAGAGAGACTGAAGTTC
GAGGGTCTCTGTTGCCACCCTTTCTTTGCCAGAACCGACTGGAATAACATCCGGAACTCTCCTCCCCCCT
TCGTTCCCACCCTCAAGTCTGACGATGACACCTCCAATTTTGATGAACCAGAGAAGAATTCGTGGGTTTC
ATCCTCTCCGTGCCAGCTGAGCCCTTCGGGTTTCTCGGGCGAAGAGCTGCCCTTTGTGGGGTTTTCATAC
AGCAAGGCACTGGGGTATCTTGGTAGATCTGAGTCTGTTGTGTCGGGTCTGGACTCCCCTGCCAAGATTA
GCTCCATGGAAAAGAAACTTCTCATCAAAAGCAAAGAGCTGCAAGACTCTCAGGACAAGTGTCACAAGAT
GGAGCAGGAAATGGCCCGGTTGCATCGCAGAGTGTCAGAGGTGGAGGCTGTGCTTAGTCAGAAGGAGGTG
GAGCTGAAGGCCTCTGAGACTCAGAGATCCCTCCTGGAGCAGGACCTTGCCACCTACATCACAGAATGCA
GTAGCTTAAAGCGAAGTCTGGAGCAAGCGCGGATGGAGGTGTCTCAGGAGGATGACAAAGCTCTTCAGCT
TCTCCACGACATCAGAGAGCAGAGCCGGAAGCTCCAGGAGATCAAGGAGCAGGAGTACCAGGCTCAGGTG
GAGGAGATGAGGCTGATGATGAATCAGCTGGAAGAGGACCTCGTGTCAGCCCGAAGACGCAGCGATCTCT
ACGAGTCTGAGCTGAGGGAGTCTCGGCTCGCCGCCGAGGAATTCAAGCGGAAGGCAAACGAATGTCAGCA
TAAACTGATGAAGGCTAAGGACCTAGGGAAGCCTGAAGTGGGAGAATGTTCCAGACTGGAGAAGATCAAT
GCTGAGCAGCAGCTGAAGATTCAGGAGCTCCAGGAGAAGCTGGAGAAGGCTGTAAAAGCCAGCACCGAGG
CCACAGAGCTCCTGCAGAACATCCGGCAGGCAAAGGAGCGAGCTGAGAGGGAGCTGGAGAAGCTGCACAA
CAGGGAAGATTCTTCCGAAGGCATCAAAAAGAAACTGGTGGAAGCTGAGGAACGCCGCCACTCCCTGGAG
AACAAGGTAAAGAGACTAGAGACCATGGAGCGTAGAGAGAACAGACTGAAGGATGACATCCAGACAAAGT
CCGAACAGATCCAGCAGATGGCTGATAAAATTCTGGAGCTGGAGGAGAAGCATCGGGAGGCTCAGGTCTC
AGCTCAACACCTAGAAGTACACCTGAAGCAGAAGGAACAGCACTATGAGGAAAAGATCAAAGTGTTGGAC
AATCAGATAAAGAAGGACTTGGCCGACAAGGAGAGCCTAGAGACCATGATGCAGAGGCACGAAGAGGAGG
CCCACGAGAAGGGCAAGATCCTCAGCGAGCAGAAGGCGATGATCAACGCAATGGATTCCAAGATCAGATC
CCTGGAGCAGAGGATTGTGGAGCTGTCAGAAGCCAACAAGCTTGCGGCAAACAGCAGTCTCTTCACCCAG
AGGAACATGAAGGCCCAGGAAGAGATGATCTCAGAACTCAGGCAGCAGAAATTTTACCTGGAGACACAGG
CCGGGAAGCTGGAGGCCCAGAACCGAAAACTGGAAGAGCAACTAGAGAAGATCAGCCACCAAGATCACAG
TGACAAGAATCGGCTGCTGGAGCTGGAGACAAGGTTGAGAGAGGTCAGCCTGGAGCATGAGGAGCAGAAG
CTGGAGCTGAAGCGGCAGCTCACGGAGCTGCAGCTGTCCCTGCAGGAGCGTGAGTCCCAGCTGACGGCCC
TGCAGGCCGCCCGGGCTGCCCTTGAGAGCCAGCTCCGCCAGGCGAAGACGGAGCTGGAGGAGACAACTGC
AGAAGCGGAGGAGGAGATCCAGGCACTCACGGCACATCGAGATGAAATCCAGCGCAAATTCGATGCCCTT
CGCAACAGCTGTACTGTCATCACCGACCTGGAGGAGCAGCTGAACCAGCTCACCGAGGACAACGCCGAGC
TCAACAACCAAAACTTCTACCTGTCCAAACAACTCGATGAGGCTTCGGGTGCCAACGATGAGATTGTGCA
GCTGCGAAGCGAGGTGGACCATCTCCGCCGTGAGATCACGGAGCGGGAGATGCAGCTCACCAGCCAGAAG
CAAACAATGGAGGCTCTGAAGACGACCTGCACCATGCTGGAGGAGCAGGTCATGGACCTGGAGGCCCTGA
ACGACGAGCTGCTGGAGAAGGAGCGCCAGTGGGAGGCCTGGAGGAGCGTCCTCGGGGACGAGAAGTCTCA
GTTTGAGTGTCGAGTTCGAGAGCTACAGAGGATGCTGGACACCGAGAAGCAGAGCAGGGCTAGAGCTGAT

CAGCGGATCACGGAGTCTCGCCAGGTGGTGGAGCTGGCAGTGAAGGAGCACAAAGCCGAGATTCTCGCGC
TGCAGCAGGCTCTCAAAGAGCAGAAGCTGAAAGCTGAGAGCCTGTCGGACAAGCTCAACGACCTGGAGAA
GAAGCACGCCATGCTTGAGATGAATGCCCGGAGCTTACAGCAGAAACTGGAGACAGAGCGGGAGCTCAAA
CAGAGGCTTCTGGAGGAGCAAGCCAAGTTGCAGCAGCAGATGGACCTGCAGAAGAACCACATCTTCAGAC
TGACACAAGGGCTGCAGGAGGCGCTGGACCGGGCGGATCTGCTGAAGACCGAAAGGAGCGACCTGGAATA
TCAGCTGGAAAACATTCAGGTTCTCTACTCACACGAGAAGGTGAAAATGGAAGGCACTATTTCTCAACAA
ACCAAACTCATTGATTTCCTGCAAGCCAAAATGGACCAGCCCGCTAAAAAGAAAAAGGGTTTATTTAGTC
GACGGAAAGAGGACCCTGCTTTGCCCACACAGGTTCCTCTGCAGTACAATGAGCTGAAGCTCGCCCTGGA
AAAGGAGAAAGCGCGCTGTGCGGAGCTGGAGGAAGCCCTTCAGAAGACCCGCATCGAACTCCGGTCCGCA
CGGGAGGAAGCTGCTCACCGCAAAGCCACGGACCACCCGCACCCATCTACGCCAGCCACTGCGAGGCAGC
AGATCGCCATGTCCGCCATCGTGCGGTCGCCTGAGCACCAGCCCAGTGCCATGAGCCTGCTCGCCCCACC
ATCCAGCCGCAGAAAGGAGGCGTCAACTCCAGAGGAGTTCAGCCGGCGTCTTAAAGAACGTATGCACCAC
AACATTCCTCACCGGTTTAACGTGGGCCTGAATATGCGAGCCACCAAGTGCGCCGTGTGTCTGGATACTG
TGCACTTTGGACGCCAGGCATCCAAATGTCTCGAATGTCAGGTGATGTGCCATCCCAAGTGTTCCACCTG
CTTGCCCGCCACCTGTGGCCTACCTGCCGAATATGCCACACACTTCACCGAGGCCTTCTGCCGAGACAAA
GTGAGCTCCCCGGGGCTCCAGAGCAAGGAGCCCAGCAGCAGCTTGCACCTGGAAGGGTGGATGAAAGTGC
CCAGGAATAACAAACGAGGACAGCAAGGCTGGGACAGAAAGTACATTGTCTTAGAGGGTTCAAAAGTCCT
CATCTATGACAACGAAGCCAGAGAAGCTGGACAGAGGCCGGTGGAAGAATTTGAGCTGTGCCTTCCCGAT
GGGGATGTATCTATTCATGGTGCCGTTGGTGCTTCTGAACTTGCAAATACAGCCAAAGCAGATGTCCCAT
ACATACTGAAGATGGAGTCCCATCCGCATACCACCTGCTGGCCTGGGAGAACCCTCTACCTGCTGGCACC
CAGCTTTCCTGACAAGCAGCGCTGGGTCACAGCCCTAGAGTCTGTCGTCGCAGGTGGGAGAGTTTCTAGG
GAAAAGGCAGAGGCCGATGCTAAATTACTTGGAAACTCTCTGCTGAAGCTGGAAGGTGACGACCGGCTTG
ACATGAACTGTACCCTGCCCTTCAGCGACCAGGTGGTGCTGGTGGGCACCGAGGAGGGCCTCTATGCGCT
GAACGTCTTGAAAAACTCCTTAACCCACATCCCAGGAATCGGAGCAGTCTTCCAGATTTACATCATCAAG
GACCTGGAGAAGCTGCTCATGATAGCAGGGGAAGAGCGGGCTCTGTGTTTGGTGGACGTGAAGAAGGTGA
AGCAGTCGTTGGCACAGTCACACCTTCCTGCCCAGCCTGACGTCTCCCCCAACATATTTGAAGCCGTCAA
AGGCTGCCACCTGTTTGCTGCTGGCAAGATCGAGAACAGCCTCTGCATCTGTGCCGCCATGCCGAGCAAA
GTCGTCATCCTGCGCTACAACGACAACCTCAGCAAGTTCTGCATCCGCAAGGAGATTGAGACCTCTGAGC
CCTGCAGCTGTATCCACTTCACCAATTACAGTATCCTCATCGGAACCAACAAGTTCTATGAGATCGACAT
GAAACAGTACACGCTTGAGGAGTTCCTGGACAAGAATGACCATTCCTTGGCACCTGCTGTGTTCGCATCC
TCGACCAACAGCTTCCCCGTCTCCATCGTGCAGGCAAACAGCACCGGGCAGCGGGAAGAGTACCTGCTGT
GTTTCCACGAATTTGGGGTGTTCGTGGATTCTTATGGAAGACGTAGCCGCACAGACGATCTCAAGTGGAG
TCGCTTACCTCTGGCCTTTGCCTACAGAGAGCCTTATCTGTTTGTGACTCACTTTAACTCCCTGGAAGTA
ATTGAGATCCAGGCACGTTCCTCACTGGGGACCCCTGCCCGAGCCTATCTGGAAATCCCAAACCCTCGCT
ACCTGGGCCCCGCAATTTCCTCCGGAGCAATTTACCTGGCCTCCTCATACCAGGACAAGTTAAGGGTCAT
ATGCTGCAAAGGAAACCTCGTGAAGGAGTCTGGCACTGAGCAGCACCGCGTGCCCTCCACATCCCGCAGC
AGCCCTAACAAGCGAGGCCCACCAACATACAACGAGCACATCACCAAACGTGTGGCCTCCAGCCCAGCAC
CACCAGAAGGCCCCAGCCATCCCCGAGAGCCAAGCACACCCCACCGCTACCGAGACAGAGAGGGCCGGAC
AGAGCTGCGCAGGGACAAGTCTCCAGGCCGTCCTCTGGAGCGGGAGAAGTCGCCAGGCCGGATGCTCAGC
ACGAGGAGAGAGCGGTCCCCGGGGAGACTGTTTGAAGACAGCAGCAGGGGCCGGCTGCCTGCGGGAGCCG
TGAGGACCCCACTGTCCCAGGTTAACAAGGTCTGGGACCAGTCTTCAGTA
>Fukomys damarensis XM_010604069.2
ATGTTGAAGTTTAAATATGGTGCGCGAAATCCACTGGACGCTGGTGCTGTTGAGCCCATTGCCAGCCGGG
CCTCCAGGCTGAATCTCTTCTTCCAGGGGAAACCACCCTTTATGACTCACCAGCAGATGTCTGCTCTTTC
CAGAGAAGGGATATTAGATGCCCTGTTTGTTCTCTTTGAAGAATGCAGCCAGCCTGCTCTGATGAAGATT
AAACACGTGAGCAACTTTGTCCGGAAGTATTCCGACACCATTGCTGAGTTACAGGAGCTCCAGCCTTCTG
CAAAGGACTTTGAAGTCAGAAGTCTTGTAGGTTGTGGTCACTTTGCTGAAGTACAGGTGGTAAGAGAGAG

AGCAACCGGGGACATCTATGCCATGAAAATCATGAAGAAGAAGGCCTTGTTGGCCCAGGAACAGGTTTCA
TTTTTTGAAGAAGAACGGAACATACTATCTCGAAGCACAAGCCCTTGGATTCCCCAATTACAGTATGCCT
TTCAGGACAAAAATAACCTTTACCTGGTCATGGAATATCAGCCTGGAGGGGATTTGCTGTCACTTTTGAA
TAGATATGAGGACCATTTAGATGAAAATATAATTCAGTTTTACCTAGCTGAACTGATTTTAGCTGTTCAC
AGTGTTCATCAGATGGGATATGTACATCGAGACATCAAGCCTGAGAATGTTCTCATTGACCGAACGGGAC
ACATCAAGCTGGTGGATTTTGGATCAGTTGCTAAAATGAATTCAAATAAAATGGTGAATGCCAAGCTCCC
AATTGGAACCCCAGATTATATGGCTCCTGAGGTGCTGACTGTGATGAACGGGGATGGAAGAGCCGTCTAT
GGCCTTGACTGTGACTGGTGGTCAGTGGGGGTTATTGCTTACGAAATGGGTTATGGAGGACCCCGTTCA
CCGAGGGAACCTCTGCCAGGACCTTCAATAATATCATGAATTTCCAGCGGTTTTTGAAGTTTCCAGATGA
CCCAAAAGTTAGCAGTGAATACCTTGATTTGATTCAGAGTTTGTTGTGTGGCCAGAAAGAGAGACTGAAG
TTTGAGGGCCTTTGCTGCCATCCTTTTTTCTCTAAGATGGACTGGAATGACATTCGTAACTCTCCTCCCC
CCTTCGTTCCCACCCTCAAGTCTGACGATGACACCTCCAATTTTGATGAACCAGAGAAGAATTCGTGGGT
TTCATCCTCTCCGTGCCAGCTGAGCCCCTCGGGTTTCTCTGGTGAAGAACTGCCGTTTGTGGGGTTTTCG
TACAGCAAGGCACTGGGGATTCTTGGTAGATCTGAGTCTGTTGTATCAAGTCTGGACTCCCCTGCCAAGA
CTAGCTCCATGGAAAAGAAACTTCTCATCAAAAGCAAAGAGCTGCAAGACTCCCAGGACAAGTGTCACAA
GATGGAGCAGGAAATGACCCGGTTACATCGGAGAGTGTCAGAGGTGGAGGCTGTGCTTAGTCAGAAGGAG
GTGGAGCTGAAGGCCTCTGAGACTCAGAGATCCCTCCTGGAGCAGGACCTTGCTACCTACATCACAGAAT
GCAGTAGCTTAAAGCGAAGTCTGGAGCAAGCACGGATGGAGGTGTCCCAGGAGGACGACAAAGCCCTGCA
GCTCCTCCATGACATCAGAGAGCAGAGCAGGAAGCTCCAAGAAATCAAAGAGCAGGAATACCAGGCTCAA
GTGGAAGAAATGAGGTTGATGATGAACCAGTTAGAAGAGGACCTTGTCTCAGCCAGAAGACGGAGTGATC
TCTATGAATCTGAGCTGAGGGAATCCCGGCTTGCTGCTGAAGAATTCAAGCGGAAAGCCACAGAATGCCA
GCATAAACTGATAAAGGCTAAAGATCAAGGGAAACCTGAAGTAGGAGAATATTCCAAACTTGAGAAGATC
AATGCTGAGCAGCAGCTCAAAATCCAGGAGCTCCAAGAGAAGCTGGAAAAGGCAGTAAAAGCCAGCACAG
AAGCCACCGAGCTGCTGCAGAATATCCGCCAGGCAAAGGAGCGAGCTGAGAGGGAGCTTGAGAAGCTGCA
GAACCGAGAAGATTCTTCGGAAGGCATAAGAAAGAAGCTGGTTGAAGCTGAGGAACGTCGCCATTCTCTG
GAGAACAAGGTAAAGAGACTAGAGACCATGGAGCGTAGAGAAAACAGACTGAAGGATGACATCCAGACAA
AATCCCAACAGATCCAGCAGATGGCTGATAAAATTCTGGAGTTGGAGGAGAAACACCGGGAGGCCCAAGT
TTCAGCCCAGCACCTAGAAGTACACTTGAAGCAGAAAGAGCAGCACTACGAGGAGAAAATTAAAGTGTTA
GACAATCAGATAAAGAAGGACCTGGCTGACAAGGAGAGCCTGGAGAACCTGATGCAGAGACACGAGGAGG
AGGCCCATGAGAAGGGCAAGATTCTCAGCGAGCAGAAGGCGATGATCAATGCCATGGATTCTAAGATCAG
ATCCCTGGAACAGAGAATCGTGGAACTTTCTGAAGCCAATAAACTTGCAGCAAATAGCAGCCTCTTTACC
CAAAGGAACATGAAGGCCCAGGAAGAAATGATTTCAGAACTCAGGCAACAGAAATTTTACCTCGAGACAC
AGGCTGGGAAATTGGAGGCCCAGAACCGAAAGCTGGAAGAGCAGCTGGAGAAAATCAGCCACCAGGATCA
CAGTGACAAGAATCGGCTGCTGGAGCTAGAGACAAGATTGAGGGAGGTCAGTCTGGAGCACGAGGAACAG
AAACTGGAGCTGAAGCGCCAGCTCACAGAGCTGCAGCTGTCTCTGCAAGAGCGGGAGTCCCAGCTGACTG
CCCTGCAGGCTGCGCGGGCTGCCCTGGAGAGCCAGCTTCGCCAGGCGAAGACAGAGCTGGAGGAGACCAC
TGCGGAAGCTGAGGAGGAGATTCAGGCTCTCACGGCACATAGAGATGAAATCCAGCGCAAATTTGATGCC
CTTCGTAACAGCTGTACTGTCATCACAGACCTGGAGGAGCAGCTAAATCAGCTCACCGAAGACAACGCTG
AGCTCAATAACCAAAACTTCTACTTGTCCAAACAACTCGATGAGGCTTCAGGTGCCAACGACGAGATAGT
TCAGCTGCGAAGTGAAGTAGACCATCTCCGCCGTGAGATCACAGAAAGGGAGATGCAGCTCACCAGCCAG
AAGCAAACGATGGAGGCTCTGAAGACCACGTGCACAATGCTAGAAGAACAGGTCATGGATCTGGAGGCCC
TGAATGATGAACTGCTAGAAAAAGAGCGTCAGTGGGAGGCCTGGAGGAGTGTCCTTGGTGACGAGAAGTC
CCAGTTTGAGTGTCGGGTTCGAGAGTTACAGCGAATGCTGGACACTGAGAAACAGAGCAGGGCGCGAGCT
GATCAGCGGATCACTGAGTCTCGCCAGGTGGTGGAGCTGGCAGTGAAGGAACACAAGGCCGAGATTCTCG
CGCTGCAACAGGCTCTCAAGGAACAGAAGCTGAAAGCTGAGAGCCTCTCTGACAAGCTCAATGACCTGGA
AAAGAAGCATGCCATGCTTGAAATGAACGCCCGAAGCCTACAGCAGAAACTGGAGACTGAGCGAGAACTC
AAACAAAGACTTCTGGAAGAGCAAGCCAAGTTACAGCAGCAGATGGACCTACAGAAGAACCACATTTTCC

GTCTCACTCAAGGGCTTCAAGAAGCTCTTGATCGGGCTGATCTGTTGAAGACAGAAAGAAGTGATCTCGA
GTATCAGCTGGAAAATATTCAGGTTCTCTATTCTCATGAAAAGGTGAAAATGGAAGGCACTATTTCTCAA
CAAACCAAACTCATTGATTTTCTGCAAGCCAAAATGGACCAACCTGCTAAAAAGAAAAAGGGTTTATTTA
GTCGACGGAAAGAGGACCCTGCTTTGCCCACACAGGTTCCTCTGCAGTACAATGAGCTGAAGCTGGCCCT
GGAGAAGGAGAAAGCTCGCTGTGCAGAGCTAGAGGAGGCCCTTCAGAAGACCCGCATTGAGCTCCGCTCC
GCCCGAGAGGAAGCTGCCCATCGCAAAGCCACGGACCACCCTCACCCGTCTACGCCAGCCACTGCAAGGC
AGCAAATCGCCATGTCTGCCATTGTGCGGTCTCCTGAGCACCAGCCCAGTGCCATGAGCCTGCTCGCACC
GCCTTCCAGCCGCAGAAAAGAGTCTTCAACTCCAGAGGAATTTAGTCGACGTCTTAAGGAGCGCATGCAC
CACAATATTCCTCATCGATTTAATGTAGGACTGAACATGCGAGCCACAAAGTGTGCTGTGTGTCTGGATA
CTGTACACTTTGGACGCCAGGCATCCAAATGTCTTGAATGTCAGGTGATGTGTCATCCCAAGTGCTCCAC
CTGCTTGCCCGCCACCTGTGGCCTGCCTGCTGAATACGCCACACACTTCACTGAGGCCTTCTGCCGTGAC
AAAATGAACTCCCCAGGTCTCCAGAGCAAGGAACCCAGCAGCAGCCTGCACCTGGAAGGGTGGATGAAGG
TGCCCAGGAATAATAAACGAGGACAGCAAGGTTGGGACAGGAAATACATTGTGTTGGAGGGATCAAAAGT
CCTCATTTATGACAGTGAAGCCAGAGAAGCTGGACAGAGACCGGTGGAAGAATTTGAGCTCTGCCTTCCC
GACGGGGACGTATCTATTCATGGTGCCGTTGGTGCTTCTGAACTCGCAAACACAGCCAAAGCAGATGTCC
CATATATACTGAAGATGGAGTCTCACCCGCACACCACCTGCTGGCCCGGGAGAACCCTCTACTTGCTAGC
TCCCAGCTTTCCTGACAAACAACGCTGGGTCACCGCCTTAGAATCAGTTGTTGCAGGTGGGAGAGTTTCT
AGGGAAAAGGCAGAAGCCGATGCTAAATTGCTTGGAAACTCCCTGCTGAAACTGGAAGGTGATGACCGGC
TAGACATGAACTGCACACTGCCCTTCAGCGATCAGGTGGTGTTGGTGGGCACTGAGGAAGGGCTGTATGC
ACTGAATGTCTTGAAAAACTCCTTAACCCACGTCCCAGGAATTGGAGCAGTCTTCCAAATTTATATCATC
AAGGACCTGGAGAAGCTACTCATGATAGCAGGAGAAGAGCGGGCCTTGTGTCTGGTGGATGTGAAGAAAG
TAAAGCAGTCTCTGGCACAGTCGCACCTTCCTGCCCAGCCAGACATCTCACCTAACATTTTCGAAGCTGT
CAAGGGCTGCCACCTGTTTGCTGCTGGCAAGATTGAGAACAGCCTCTGCATCTGTGCGGCCACGCCCAGC
AAAGTTGTCATCCTCCGCTACAACGAAAACCTCGGCAAGTATTGCATTCGGAAGGAGATAGAGACCTCAG
AGCCCTGCAGCTGTATCCACTTTACCAATTACAGTATCCTCATTGGAACCAACAAATTCTACGAAATCGA
CATGAAGCAGTACACGCTGGAGGAATTCCTAGACAAGAATGACCACTCCTTGGCCCCTGCTGTGTTTGCC
TCCTCGTCCAACAGCTTCCCTGTCTCGGTCATTCAGGTGAACAGCGCGGGGCAGCGAGAGGAGTACCTGC
TCTGCTTCCACGAATTTGGGGTATTCGTGGATTCTTACGGGAGACGTAGCCGCACAGATGATCTCAAGTG
GAGTCGCTTGCCTTTGGCCTTTGCCTACAGAGAACCCTATCTGTTTGTGACCCACTTCAACTCACTTGAA
GTCATTGAGATCCAGGCACGCTCCTCCCTGGGGACCCCTGCCCGAGCCTATTTGGAAATCCCGAACCCAC
GCTACTTGGGTCCTGCAATTTCCTCGGGAGCAATTTACCTGGCCTCCTCATACCAGGATAAATTAAGGGT
CATTTGCTGCAAAGGAAACCTCGTGAAGGAGTCCAGCACTGAGCACCACCGGGTCCCCTCCACCTCCCGC
AGCAGCCCCAACAAGCGAGGCCCACCGACGTACAACGAGCACATTACCAAGCGCGTGGCCTCCAGCCCAG
CACCACCGGAAGGCCCCAGCCACTCCCGAGAGCCAAGCACACCCCACCGCTACCGTGAGGGGCGAACAGA
GCTGCGTAGGGACAAGTCTCCCGGCCGTCCCCTGGAGCGCGAGAAGTCACCAGGCCGGATGCTGAGCACG
CGGAGGGAACGGTCCCCTGGGAGGCTGTTTGAAGACAGCAGCAGGGGACGGCTGCCCGCGGGAGCTGTGA
GGACCCCGCTGTCCCAGGTTAACAAGGTCTGGGACCAGTCTTCAGTA
>Oryctolagus cuniculus XM_017349605.1
ATGCTGAAGTTCAAGTATGGAGCGCGGCACCCACCGGACGCTGCTGCTGCTGCGGAGCCCATCGCCAGCC
GGGCCTCCAGGCTGCATCATTTCTTCCAGGGGAAGCCACCCTTCATGACTCAGCAGCAGATGTCTCCTCT
TTCCCGAGAAGGGATATTGGATGCCCTCTTTGTTCTCTTTGAAGAATGCAGTCAGCCTGCTCTCATGAAA
ATTAAGCACGTGAGCAACTTCGTCCGGAAGTACTCCGACACCATCGCTGAGTTACAGGAGCTCCAGCCTT
CGGTGAGAGACTTTGAAGTCAGAAGTCTTGTAGGTTGTGGGCACTTTGCTGAAGTGCAGGTGGTGAGAGA
GAGAGCAACCGGGGACATCTATGCCATGAAAGTCATGAAGAAGAAGGCCTTGTTGGCCCAGGAACAGGTT
TCATTTTTTGAAGAAGAACGAAACATACTATCTCGGAGCACAAGTCCCTGGATCCCCCAGTTGCAGTATG
CCTTCCAGGACAAAGATAACCTTTACCTGGTCATGGAATATCAGCCAGGAGGGGATTTGCTGTCACTTTT
GAATAGATATGAGGACCAATTAGATGAAAACATGATTCAGTTCTACTTGGCTGAACTGATCTTGGCTGTC

CACAGCGTTCATCAGATGGGATATGTACATCGAGACATCAAGCCCGAGAACATTCTCATTGACCGAACAG
GACACATCAAGCTGGTGGATTTTGGATCGGCTGCTAAGATGAATTCAAATAAGATGGTGAATGCCAGGCT
CCCGATTGGGACCCCCGATTACATGGCTCCTGAAGTGCTGACCGTGATGAACGGGGATGGGAAAGGCACC
TACAGCCTGGACTGTGACTGGTGGTCGGTGGGAGTCATCGCTTACGAGATGGTTTATGGGAGGTCCCCAT
TCACGGAGGGAACCTCAGCCAGGACCTTCAATAACATCATGAATTTCCAGCGGTTTTTGAAGTTTCCAGA
GGACCCCAAAGTCAGCAGTGAATTTCTTGATCTGATTCAAAGTTTGCTGTGTGGCCAGAAAGAGAGACTG
AAGTTCGAAGGTCTTTGCTGCCATCCTTTCTTCTCTAAAATTGATTGGAATAAAATACGTGAGTCTCCAC
CCCCCTTCGTTCCCACCCTCCAGTCTGACGATGACACCTCCAATTTTGATGAACCAGAGAAGAATTCGTG
GGCTTCCTCGTCTCCGTGCCAGCTGAGCCCCTCAGGTTTCTCGGGCGAAGAACTGCCGTTCGTGGGGTTT
GCGTACAGCAAGGCGCTGGGGATTCTTGGTAGATCTGAGTCTGTCGTGTCGAGTCTGGACTCCCCTGCCA
AGACTAGCTCGATGGAAAAGAAACTTCTCATCAAAAGCAAAGAGCTGCAGGACTCTCAGGACAAGTGTCA
CAAGATGGAGCAGGAAATGACCCGGTTACATCGGAGAGTGTCAGAGGTGGAGGCTGTGCTTAGTCAGAAG
GAGGTGGAGCTGAAGGCCTCTGAGACTCAGAGATCCCTCCTGGAGCAGGACCTTGCTACCTACATCACAG
AATGCAGTAGCTTAAAGCGAAGTTTGGAGCAAGCGCGGATGGAGGTGTCCCAGGAGGATGACAAAGCACT
GCAGCTTCTCCATGATATCAGGGAGCAGAGCCGGAAGCTCCAGGAGATCAAAGAGCAGGAGTACCAGGCT
CAGGTGGAGGAGATGAGGTTGATGATGAATCAGTTGGAAGAGGACCTCGTGTCTGCGAGACGACGCAGTG
ACCTCTACGAATCCGAGCTGAGAGAGTCTCGGCTCGCCGCCGAGGAGTTCAAGCGGAAAGCGACAGAGTG
CCAGCACAAACTGATGAAGGCTAAGGATCAAGGGAAGCCTGAAGTGGGTGAATATTCCAAACTGGAGAAG
ATCAATGCTGAACAGCAGCTCAAAATTCAGGAACTCCAAGAGAAGCTGGAGAAGGCTGTCAAAGCCAGCA
CAGAGGCCACCGAGCTGCTGCAGAACATCCGGCAGGCAAAGGAGCGCGCCGAGAGGGAGCTGGAGAAGCT
GCAGAACCGGGAGGACTCTTCCGAAGGCATCAGAAAGAAGCTGGTGGAAGCCGAGGAACGCCGCCATTCT
CTGGAGAACAAGGTTAAGAGGCTAGAGACCATGGAGCGTAGAGAAAACAGACTGAAGGATGACATCCAGA
CAAAATCCCAACAGATCCAGCAGATGGCTGATAAAATCCTGGAGCTGGAGGAGAAACACCGGGAAGCCCA
GGTCTCAGCGCAGCACCTGGAAGTACACCTGAAACAGAAAGAGCAGCACTACGAGGAAAAAATCAAAGTG
TTGGACAATCAGATAAAGAAAGACCTGGCTGATAAGGAGAGTCTGGAGAACCTGATGCAGAGACACGAAG
AGGAGGCCCACGAGAAAGGCAAAATCCTCAGCGAGCAGAAGGCGATGATCAATGCCATGGATTCCAAGAT
CAGATCCCTGGAACAGAGGATCGTGGAGCTGTCTGAAGCCAATAAACTTGCAGCAAATAGCAGTCTTTTT
ACCCAAAGGAACATGAAGGCCCAGGAAGAGATGATTTCCGAACTCAGGCAACAGAAATTTTACCTGGAGA
CGCAGGCTGGGAAACTGGAGGCCCAGAACCGGAAGCTGGAAGAACAGCTGGAGAAAATCAGCCACCAGGA
CCACAGCGACAAGAACCGGCTGCTGGAGCTGGAGACGCGGTTGCGGGAGGTCAGCCTGGAGCACGAGGAG
CAGAAGCTGGAGCTGAAGCGCCAGCTCACGGAGCTGCAGCTCTCCCTGCAGGAGCGCGAGTCCCAGCTGA
CGGCGCTGCAGGCGGCGCGGGCGGCGCTGGAGAGCCAACTGCGCCAGGCCAAGACCGAGCTGGAGGAGAC
GACCGCGGAAGCGGAGGAGGAGATCCAGGCGCTCACGGCACATAGAGATGAAATCCAGCGCAAATTTGAT
GCCCTTCGTAACAGCTGTACTGTCATCACAGACCTGGAGGAGCAGCTGAACCAGCTGACTGAGGACAACG
CCGAACTCAACAACCAGAACTTCTACTTGTCCAAACAACTCGATGAGGCTTCCGGTGCCAACGACGAGAT
TGTCCAGCTGCGGAGTGAGGTGGACCATCTGCGCCGCGAGATCACAGAGAGGGAGATGCAGCTCACCAGC
CAGAAGCAAACGATGGAGGCGCTGAAGACGACCTGCACGATGCTGGAAGAACAGGTCATGGATCTGGAGG
CCCTGAACGACGAGCTGCTGGAGAAGGAGCGGCAGTGGGAGGCCTGGAGGAGCGTCCTCGGCGACGAGAA
GTCCCAGTTCGAGTGTCGAGTTCGAGAGCTGCAGCGCATGCTGGACACTGAGAAGCAGAGCAGGGCGAGG
GCCGACCAGCGGATCACCGAGTCTCGCCAGGTGGTGGAGCTGGCGGTGAAGGAGCACAAGGCCGAGATCC
TCGCTCTGCAGCAGGCTCTCAAGGAACAGAAGCTGAAAGCCGAGAGCCTCTCCGACAAGCTCAATGACCT
GGAGAAAAAGCATGCCATGCTTGAAATGAATGCTCGGAGCTTACAACAGAAACTGGAGACTGAACGCGAG
CTCAAACAGAGACTCCTGGAAGAGCAAGCCAAGTTACAGCAGCAGATGGACCTGCAGAAGAACCACATCT
TCCGTCTGACTCAGGGGCTGCAGGAAGCCCTGGACCGGGCTGATTTGCTGAAGACAGAAAGGAGTGACCT
GGAGTATCAGCTGGAGAACATCCAGGTTCTGTATTCTCACGAAAAGGTGAAAATGGAAGGCACGATTTCT
CAACAAACCAAACTCATTGATTTTCTGCAAGCCAAAATGGACCAACCCGCTAAAAAGAAAAAGGGTTTAT
TTAGTCGACGGAAAGAGGACCCTGCTTTGCCCACACAGGTTCCTCTGCAGTACAATGAGCTGAAGCTGGC

CCTGGAGAAGGAGAAAGCTCGCTGTGCCGAGCTGGAGGAAGCCCTGCAGAAGACCCGCATCGAGCTGCGC
TCTGCCCGGGAGGAAGCTGCCCACCGGAAAGCCGCAGAACACCCGCACCCGTCCACACCGGCCACCGCGA
GGCAGCAGATCGCCATGTCCGCCATCGTGCGGTCGCCCGAGCACCAGCCCAGTGCCATGAGCCTGCTGGC
CCCGCCGTCCAGCCGCAGAAAGGAGTCTTCCACTCCAGAGGAATTTAGCCGGCGTCTTAAGGAGCGCATG
CACCACAATATCCCTCACCGGTTTAACGTGGGCCTGAACATGCGAGCCACCAAGTGCGCCGTGTGTCTGG
ACACAGTGCACTTCGGACGCCAAGCATCCAAATGTCTCGAGTGCCAGGTGATGTGTCATCCCAAGTGCTC
CACCTGCTTGCCTGCCACCTGCGGCCTGCCCGCCGAATACGCCACGCACTTCACCGAGGCCTTCTGCCGT
GACAAAATGAACTCCCCGGGTCTCCAGGCCAAGGAGCCCAGCAGCAGCCTGCACCTGGAGGGGTGGATGA
AGGTGCCCAGGAATAACAAGCGAGGACAGCAAGGCTGGGACAGGAAGTACATTGTCCTGGAGGGATCCAA
AGTCCTCATTTACGACAGCGAAGCCCGAGAAGCTGGACAGAGGCCGGTGGAAGAATTTGAGCTGTGCCTT
CCCGACGGGGACGTTTCTATCCACGGGGCCGTTGGTGCTTCTGAACTCGCAAACACAGCCAAAGCAGATG
TCCCGTATATCCTGAAGATGGAGTCTCACCCGCACACCACCTGTTGGCCCGGGAGGACCCTCTACTTGCT
AGCTCCCAGCTTCCCTGACAAGCAGCGCTGGGTCACTGCCCTGGAGTCGGTGGTCGCAGGTGGGAGAGTT
TCTAGGGAGAAGGCCGAAGCCGATGCCAAATGGCTGGGGAACTCCCTGCTGAAACTGGAAGGTGACGACC
GGCTGGACATGAACTGCACGCTGCCCTTCAGCGACCAGGTGGTGTTGGTGGGCACCGAGGAAGGACTGTA
TGCCCTGAATGTCTTGAAAAACTCCCTGACCCACGTCCCAGGAATCGGAGCGGTCTTCCAGATCTATATC
ATCAAGGACCTGGAGAAGCTTCTCATGATAGCAGGAGAAGAGCGGGCCCTGTGTCTCGTGGACGTGAAGA
AAGTGAAGCAGTCCCTGGCGCAGGCTCACCTGCCCGCCCAGCCCGACATCTCGCCCAACGTCTTCGAAGC
CGTCAAGGGCTGCCACTTGTTTGCTGCAGGCAAGATTGACAACGGGCTCTGCATCTGTGCAGCCATGCCC
AGCAAAGTCGTCATCCTCCGCTACAACGAGAACCTCAGCAAGTACTGCATCCGCAAAGAGATCGAGACCT
CAGAGCCCTGCAGCTGTATCCACTTCACCAATTACAGTATCCTCATCGGAACCAACAAATTCTACGAGAT
CGACATGAAACAGTACACGCTCGAGGAATTCCTGGATAAGAACGACCACTCCTTGGCGCCCGCTGTGTTT
GCCTCCTCGTCCAACAGCTTCCCTGTCTCGATCGTGCAGGTGAACGGCGCAGGGCAGCGAGAGGAGTACT
TGCTGTGCTTCCACGAATTTGGGGTGTTCGTGGATTCTTACGGAAGGCGTAGCCGCACGGATGACCTCAA
GTGGAGCCGCTTGCCCTTGGCCTTCGCCTACAGAGAACCCTACCTGTTTGTGACCCACTTCAACTCACTG
GAAGTTATTGAGATCCAGGCGCGCTCCTCGCTGGGGACCCCTGCTCGAGCGTACCTGGAAATCCCAAACC
CACGCTACCTGGGCCCCGCCATCTCCTCGGGAGCCATTTACCTGGCGTCCTCGTACCAGGATAAAGTCAG
GGTCATCTGCTGCAAAGGAAACCTCGTGAAGGAGCCCGGCACTGACCACCACCGGGTCCCCTCCACCTCC
CGCAGCAGCCCCAACAAGCGAGGCCCGCCGACGTACAACGAGCACATCACCAAGCGCGTGGCCTCCAGCC
CGGCGCCACCCGAAGGCCCCAGCCACCCCCGAGAGCCAAGCACACCCCACCGCTACCGCGAGGGGCGGAC
AGAGCTGCGTAGAGACAAGTCTCCCGGCCGTCCCCTGGAGCGCGAGAAGTCCCCGGGCCGCATGCTGAGC
ACGCGGAGGGAGCGGTCCCCCGGGAGGCTGTTCGAAGACAGCAGCAGGGGCCGGCTGCCGGTGGGAGCCG
TGAGGACCCCACTGTCCCAGGTTAACAAGGTCTGGGACCAGTCTTCAGTA
>Chinchilla lanigera XM_005396521.2
ATGTTGAAGTTTAAATATGGAGCGCGGAATCCACCGGATGCTGGTGCTGTTGAGCCCATTGCCAGCCGGG
CCTCCAGGCTGAATCTGTTCTTCCAGGGGAAACCACCCTTTATGACTCAACAGCAGATGTCTGCTCTTTC
CAGAGAAGGGATATTAGATGCCCTGTTTGTTCTCTTTGAAGAATGCAGCCAGCCTGCTCTGATGAAGATT
AAGCACGTGAGCAACTTCGTGCGGAAGTATTCCGACACCATAGCTGAGTTACAGGAGCTCCAGCCTTCGG
CAAAGGACTTTGAAGTGAAAAGTCTTGTAGGTTGTGGTCACTTTGCTGAAGTACAGGTGGTAAGAGAGAG
AGCAACTGGGGACATCTATGCCATGAAGATCATGAAGAAGAAGGCCTTGTTGGCCCAAGAACAGGTTTCA
TTTTTTGAGGAAGAACGGAACATATTATCTCGGAGCACAAGCCCTTGGATTCCCCAATTACAGTATGCCT
TTCAGGACAAAAATAATCTTTACCTGGTCATGGAATATCAGCCTGGAGGGGATTTGCTTTCACTTTTGAA
TAGATATGAGGACCAATTAGATGAAAATATGATTCAGTTTTACCTAGCTGAACTGATTTTAGCTGTTCAC
AGTGTTCATCAGATGGGATATGTACATCGAGATATCAAGCCTGAGAACGTTCTCATTGACCGAATGGGAC
ACATCAAGCTGGTGGATTTTGGATCAGCTGCTAAAATGAATTCAAGTAAAATGGTGAATGCCAAGCTCCC
AATTGGAACCCCAGATTACATGGCTCCTGAAGTGTTGACTGTGATGAACGGGGATGGAAAAGGCACCTAC
GGCCTCGACTGTGACTGGTGGTCAGTGGGCGTTATTGCTTATGAAATGGTTTATGGGCGAACCCCATTCA

CAGAGGGAACCTCTGCCAGGACCTTCAATAATATCATGAATTTCCAGCGGTTTTTGAAGTTTCCGGATGA
CCCAAAAGTTAGCAGTGAATACCTTGATCTGATTCAAAGTTTGCTGTGTGGCCAGAAGGAGAGACTGAAA
TTTGAGGGGCTTTGCTGCCACCCTTTTTTCTCTAAGGTGGACTGGAATGACATTCGTAACTCTCCTCCCC
CCTTCGTTCCCACCCTCAAGTCTGACGATGACACCTCCAATTTTGATGAACCAGAGAAGAATTCGTGGGT
TTCATCCTCTCCGTGCCAGCTGAGCCCCTCGGGTTTCTCGGGCGAAGAACTGCCGTTTGTGGGGTTTTCG
TACAGCAAGGCACTGGGGATTCTTGGTAGATCTGAGTCTGTTGTATTGAGTCTGGACTCCCCTGCCAAGA
CTAGCTCCATGGAAAAGAAACTTCTCATCAAAAGCAAAGAGCTGCAAGACTCTCAGGACAAGTGTCACAA
GATGGAGCAGGAAATGACCCGGTTACATCGGAGAGTGTCAGAGGTGGAGGCTGTGCTTAGTCAGAAGGAG
GTGGAGCTGAAGGCCTCTGAGACTCAGAGATCCCTCCTGGAGCAGGACCTTGCCACCTACATCACAGAAT
GCAGTAGCTTAAAGCGAAGTTTGGAGCAAGCACGGATGGAGGTGTCCCAGGAGGATGACAAAGCACTGCA
GCTCCTTCATGACATCAGAGAACAGAGCCGGAAGCTCCAAGAAATCAAAGAGCAGGAATACCAGGCTCAA
GTGGAAGAAATGAGGTTGATGATGAATCAGTTGGAAGAGGACCTTGTCTCAGCCAGAAGACGGAGTGATC
TCTATGAATCCGAGCTGAGAGAGTCTCGGCTCGCTGCTGAAGAATTCAAGCGGAAGGCCACAGAATGTCA
GCATAAACTGATAAAGGCTAAAGATCAAGGAAAACCCGAAGTGGGAGAATATTCCAAACTTGAGAAGATC
AATGCTGAGCAGCAGCTCAAAATCCAAGAGCTCCAAGAGAAGCTGGAAAAGGCAGTGAAAGCCAGCGCAG
AGGCCACCGAGCTGCTGCAGAATATCCGTCAGGCAAAGGAGCGCGCGGAGCGGGAGCTGGAGAAGCTGCA
GAACCGAGAAGATTCCTCAGAAGGCATAAGAAAGAAGCTGGTTGAAGCTGAGGAACGCCGACATTCTCTG
GAGAACAAGGTAAAGAGACTAGAGACCATGGAGCGTAGAGAAAACAGACTGAAGGATGACATCCAGACAA
AATCCCAACAGATCCAGCAGATGGCTGATAAAATTCTGGAGCTGGAGGAGAAACACCGGGAGGCCCAAGT
GTCAGCGCAGCACCTAGAAGTACACTTGAAGCAGAAAGAGCAGCACTACGAGGAGAAAATTAAAGTGTTG
GACAATCAGATAAAGAAGGACCTGGCGGACAAAGAGAGCCTCGAGAACCTGATGCAGAGACACGAAGAGG
AGGCCCACGAGAAGGGCAAGATTCTCAGCGAGCAGAAGGCGATGATCAATGCCATGGATTCCAAGATCAG
ATCTCTAGAACAGAGAATTGTGGAACTTTCTGAAGCCAATAAACTTGCAGCAAACAGCAGCCTCTTTACC
CAAAGGAACATGAAGGCCCAGGAAGAGATGATTTCAGAACTCAGGCAACAGAAATTTTATCTGGAGACCC
AGGCTGGGAAACTGGAGGCCCAGAACCGAAAGCTGGAAGAGCAGCTGGAGAAAATCAGCCACCAAGATCA
CAGTGACAAGAATCGGCTGCTGGAGCTAGAGACAAGATTGCGGGAGGTCAGTCTGGAGCATGAAGAGCAG
AAACTGGAGCTGAAGCGCCAGCTCACAGAGCTGCAGCTGTCCTTGCAAGAGCGTGAATCCCAGCTCACTG
CCCTGCAGGCTGCCCGGGCTGCCCTGGAGAGCCAGCTTCGCCAGGCAAAGACAGAGCTGGAGGAGACGAC
TGCGGAAGCTGAAGAGGAGATTCAGGCTCTCACGGCACATAGAGATGAAATCCAGCGAAAATTTGATGCC
CTTCGTAACAGCTGCACTGTCATCACAGACCTGGAAGAGCAGCTAAACCAACTCACCGAAGACAACGCTG
AACTCAACAACCAAAACTTCTACTTGTCCAAACAACTCGATGAGGCTTCGGGCGCTAATGATGAGATAGT
TCAGCTGCGAAGCGAAGTAGACCATCTCCGGCGTGAGATCACAGAGAGGGAGATGCAGCTCACCAGCCAG
AAGCAAACGATGGAGGCTCTGAAGACCACATGCACGATGCTAGAAGAACAAGTTATGGATCTGGAGGCCC
TAAATGATGAACTACTCGAAAAAGAGCGCCAGTGGGAGGCCTGGAGGAGCGTCCTCGGTGACGAGAAGTC
CCAGTTTGAGTGTCGGGTTCGAGAGTTACAGAGGATGCTGGACACTGAGAAACAGAGCAGGGCGCGAGCT
GATCAGCGGATCACTGAGTCTCGCCAGGTGGTGGAGCTGGCAGTGAAGGAACACAAGGCTGAGATTCTCG
CTCTGCAGCAGGCTCTCAAGGAACAGAAGCTGAAAGCTGAGAGCCTCTCTGACAAGCTTAATGACCTGGA
AAAGAAGCATGCTATGCTTGAAATGAATGCCCGAAGCCTACAGCAGAAACTGGAGACTGAGCGAGAGCTC
AAACAAAGGCTTCTGGAAGAGCAAGCCAAATTACAGCAGCAGATGGACCTACAGAAGAACCACATTTTCC
GTCTCACACAAGGGCTTCAAGAAGCTCTTGATCGGGCTGATCTGTTGAAGACAGAAAGAAGTGATCTGGA
GTATCAGCTGGAAAATATTCAGGTTCTCTATTCTCATGAAAAGGTGAAAATGGAAGGTACTATTTCTCAA
CAAACCAAACTCATTGATTTCCTGCAAGCCAAAATGGACCAACCTGCTAAAAAGAAAAAGGGTTTATTTA
GTCGACGGAAAGAGGACCCTGCTTTGCTCACACAGGTTCCTCTGCAGTACAATGAGCTGAAGCTGGCCCT
GGAGAAGGAGAAAGCTCGCTGTGCAGAGCTAGAGGAAGCCCTGCAGAAGACCCGCATTGAGCTCCGCTCC
GCCCGAGAGGAAGCTGCGCACCGCAAAGCCACGGACCACCCTCACCCGTCTACGCCAGCCACTGCGAGGC
AGCAAATTGCCATGTCTGCCATTGTGCGGTCGCCTGAGCACCAGCCCAGTGCCATGAGCCTGCTTGCACC
ACCATCCAGCCGCAGAAAAGAGTCTTCAACTCCAGAAGAATTTAGTCGGCGTCTGAAGGAGCGCATGCAC

CACAATATTCCTCACCGATTTAATGTAGGACTGAACATGCGAGCCACGAAGTGCGCTGTGTGTCTGGATA
CCGTGCACTTTGGACGCCAGGCATCCAAATGTCTCGAATGTCAGGTGATGTGTCATCCCAAGTGCTCCAC
CTGCTTGCCAGCCACCTGTGGCCTGCCTGCCGAATATGCCACGCACTTCACTGAGGCCTTTTGCCGTGAC
AAAATGAACTCCCCAGGTCTCCAGAGCAAGGAACCCAGCAGCAGCCTGCACCTGGAAGGGTGGATGAAGG
TGCCCAGGAATAACAAACGAGGACAGCAAGGCTGGGACAGGAAATACATTGTGTTGGAGGGATCAAAAGT
CCTCATTTATGACAATGAAGCCAGAGAAGCTGGACAGAGACCGGTGGAAGAATTTGAGCTCTGCCTTCCC
GACGGGGACGTATCTATTCATGGTGCCGTTGGTGCTTCTGAACTCGCAAACACAGCCAAAGCAGATGTCC
CATACATACTGAAGATGGAGTCTCACCCACACACCACCTGCTGGCCGGGGAGAACCCTCTACTTGCTAGC
TCCCAGCTTTCCCGACAAACAGCGCTGGGTCACCGCCTTAGAATCAGTTGTTGCAGGTGGGAGAGTTTCT
AGGGAAAAGGCAGAAGCCGATGCTAAATTGCTTGGAAACTCCCTGCTGAAACTGGAAGGTGATGACCGGC
TAGACATGAACTGCACACTGCCCTTCAGTGACCAGGTGGTGTTGGTGGGCACCGAGGAAGGGCTCTATGC
ACTGAATGTCTTGAAAAACTCCTTAACCCATGTCCCAGGAATTGGAGCAGTCTTCCAAATTTATATCGTC
AAGGACCTGGAGAAACTACTCATGATAGCAGGAGAAGAGCGGGCCCTGTGTCTGGTAGATGTGAAGAAAG
TGAAGCAGTCTCTGGCACAGTCGCACCTTCCTGCCCAGCCAGACATCTCACCTAACGTTTTCGAAGCTGT
CAAGGGCTGCCACCTGTTCGGTGCTGGCAAGATTGAGAACAGCCTCTGCATCTGTGCAGCCATGCCCAGC
AAAGTCGTCATCCTTCGCTACAACGAAAACCTCGGCAAGTATTGCATTCGGAAGGAGATAGAGACCTCAG
AGCCCTGCAGCTGTATCCACTTTACCAATTACAGTATCCTCATTGGAACCAACAAATTCTACGAAATCGA
CATGAAGCAGTACACCCTGGAGGAATTCCTAGACAAGAATGACCACTCCTTGGCACCTGCTGTGTTTGCC
TCCTCGTCCAACAGCTTTCCTGTCTCAATCGTTCAGGTGAACGGCTCAGGGCAGCGAGAGGAGTACCTGC
TGTGCTTCCACGAATTTGGGGTATTCGTGGATTCTTACGGAAGACGTAGCCGCACAGATGATCTCAAGTG
GAGTCGCTTGCCTTTGGCCTTCGCCTACAGAGAACCCTATCTGTTTGTGACCCACTTCAACTCACTTGAA
GTCATTGAGATCCAGGCCCGTTCCTCACTAGGGACCCCTGCCCGAGCCTATTTGGAAATCCCAAACCCGC
GCTACCTGGGCCCAGCAATTTCTTCGGGAGCAATTTACCTGGCCTCCTCATACCAGGATAAATTAAGGGT
CATTTGCTGCAAAGGAAACCTTGTGAAGGAGACCGGCACTGAGCACCACCGGGTCCCCTCCACCTCCCGC
AGCAGCCCCAACAAACGAGGCCCACCGACCTACAATGAGCACATCACCAAGCGCGTGGCCTCCAGCCCAG
CACCGCCTGAAGGCCCCAGCCACCCCCGAGAGCCAAGCACACCCCACCGCTACCGTGAGGGGCGAACGGA
GCTGCGTAGGGACAAGTCTCCCGGCCGCCCCCTGGAGCGTGAGAAGTCCCCGGGCCGGATGCTGAGCACA
CGGAGGGAGCGGTCCCCTGGGAGGCTGTTCGAAGACAGTAGCAGGGGCCGGCTGCCTGTGGGAGCTGTGA
GGACCCCATTGTCCCAGGTTAACAAGGTCTGGGACCAGTCTTCAGTA
>Mus musculus ENSMUST00000102560.6
ATGTTGAAGTTCAAGTATGGTGTGCGGAACCCGCCGGAGGCCAGTGCCTCCGAGCCCATT
GCCAGTCGGGCCTCCAGGCTAAATCTCTTCTTCCAGGGGAAACCGCCCCTCATGACTCAA
CAGCAGATGTCTGCTCTTTCCCGGGAAGGGATGCTAGACGCCCTCTTCGCTCTCTTTGAA
GAGTGCAGCCAACCCGCCCTGATGAAGATGAAGCACGTGAGCAGCTTTGTCCAGAAGTAT
TCCGACACCATAGCCGAGTTGCGGGAGCTGCAGCCGTCGGCGAGAGACTTCGAAGTTCGA
AGCCTTGTGGGCTGTGGTCACTTCGCTGAAGTGCAGGTGGTTAGAGAGAAGGCGACCGGG
GACGTCTATGCCATGAAAATCATGAAGAAGAAGGCTTTGCTGGCCCAGGAACAGGTTTCA
TTTTTCGAGGAGGAGAGGAACATATTATCTCGGAGCACGAGTCCTTGGATCCCCCAGTTA
CAGTACGCCTTTCAGGACAAAAATAACCTTTACCTGGTCATGGAATATCAGCCTGGAGGG
GATTTGCTGTCGCTTCTGAACAGATACGAGGACCAATTAGATGAGAGCATGATCCAGTTT
TACCTTGCTGAGCTGATTTTGGCTGTCCACAGCGTGCACCAGATGGGATATGTGCATCGA
GACATCAAGCCCGAGAACATCCTCATCGACCGGACGGGACACATCAAGCTGGTGGATTTT
GGATCAGCCGCTAAGATGAATTCAAATAAGGTGGATGCCAAACTCCCCATTGGGACCCCG
GATTACATGGCTCCGGAAGTGTTGACCGTGATGAACGAGGACCGAAGGGGCACATACGGC
TTGGACTGTGACTGGTGGTCTGTCGGAGTTGTTGCTTATGAGATGGTTTATGGGAAGACC
CCATTCACAGAGGGAACCTCCGCCCGGACCTTCAACAACATCATGAACTTCCAGCGGTTT
TTGAAGTTCCCAGATGACCCCAAAGTTAGCAGTGAGCTCCTTGATCTGCTTCAGAGTCTG

CTGTGTGTCCAGAAAGAGAGACTGAAGTTCGAGGGTCTCTGCTGCCACCCTTTCTTTGCC
AGAACGGACTGGAACAACATCCGTAACTCTCCTCCCCCCTTCGTCCCCACCCTCAAGTCT
GACGATGACACCTCCAATTTTGATGAACCAGAGAAGAATTCGTGGGTTTCATCCTCTGTG
TGCCAGCTGAGCCCCTCGGGCTTCTCAGGCGAAGAGCTGCCGTTTGTGGGATTTTCGTAC
AGCAAGGCACTGGGGTATCTTGGTAGATCTGAGTCTGTCGTGTCGAGTCTGGACTCCCCT
GCCAAGGTTAGCTCCATGGAAAAGAAACTTCTCATCAAAAGCAAAGAGCTCCAAGACTCC
CAGGACAAGTGTCACAAGATGGAGCAGGAAATGACCCGGTTACATCGCAGAGTGTCAGAG
GTGGAGGCTGTGCTTAGTCAGAAGGAGGTGGAGCTGAAGGCCTCTGAGACTCAGAGATCC
CTCCTGGAGCAGGACCTTGCTACCTACATCACAGAATGCAGTAGCTTAAAGCGAAGTTTG
GAGCAAGCGCGGATGGAGGTGTCCCAGGAGGATGACAAAGCTCTGCAGCTTCTCCACGAC
ATCCGAGAGCAGAGCCGGAAGCTCCAGGAGATCAAGGAGCAGGAGTACCAGGCTCAGGTG
GAGGAGATGAGGCTGATGATGAATCAGCTGGAAGAAGACCTGGTGTCAGCCCGCAGACGC
AGCGATCTCTACGAGTCTGAGCTGAGGGAGTCTCGGCTTGCCGCCGAGGAATTCAAGCGG
AAGGCAAACGAATGTCAGCACAAACTGATGAAGGCTAAGGACCAAGGGAAGCCTGAAGTG
GGAGAATATTCCAAACTGGAGAAGATCAATGCTGAGCAGCAGCTGAAGATCCAGGAGCTC
CAGGAGAAGCTGGAGAAGGCTGTAAAAGCCAGCACAGAGGCCACCGAGCTCCTGCAGAAT
ATCCGCCAGGCAAAGGAGCGAGCAGAGCGGGAGCTAGAGAAGCTACACAACCGGGAAGAC
TCCTCCGAGGGCATCAAAAAGAAGCTGGTGGAAGCCGAGGAACGCCGCCACTCCCTGGAG
AACAAGGTAAAGAGACTAGAGACCATGGAGCGTAGAGAGAACAGACTGAAGGATGACATC
CAGACAAAGTCCGAACAGATCCAGCAGATGGCTGATAAAATTCTGGAGCTGGAGGAGAAA
CATCGGGAGGCTCAGGTCTCAGCTCAACATCTAGAAGTACACTTGAAACAGAAAGAACAG
CACTACGAGGAAAAGATCAAGGTATTGGACAATCAGATAAAGAAGGACCTGGCCGACAAG
GAGAGCCTGGAGAACATGATGCAGAGACACGAGGAGGAGGCCCACGAGAAGGGCAAGATC
CTCAGCGAGCAGAAGGCGATGATCAACGCGATGGATTCCAAGATCCGATCCCTGGAGCAG
AGGATCGTGGAGCTGTCGGAAGCCAACAAGCTTGCGGCAAACAGCAGTCTCTTCACCCAG
AGGAACATGAAGGCCCAGGAAGAGATGATCTCAGAACTCAGGCAGCAGAAATTTTACCTG
GAGACGCAGGCCGGAAAGCTGGAGGCCCAGAACCGAAAGCTGGAAGAGCAACTGGAGAAG
ATCAGCCACCAAGATCACAGTGACAAGAGTCGGCTGCTGGAGCTGGAAACAAGGCTGAGG
GAGGTCAGCCTGGAGCACGAGGAGCAGAAGCTGGAGCTGAAGCGGCAGCTCACGGAGCTG
CAGCTGTCCCTGCAGGAGCGCGAGTCCCAGCTGACGGCCCTGCAGGCCGCCCGGGCAGCG
CTGGAGAGCCAGCTCCGCCAGGCGAAGACAGAGCTGGAGGAGACAACCGCGGAAGCGGAG
GAGGAGATCCAGGCGCTCACGGCACATCGAGATGAGATCCAGCGCAAATTCGATGCCCTT
CGCAACAGCTGCACGGTCATCACCGACCTGGAGGAGCAGCTGAACCAGCTCACCGAGGAC
AACGCCGAGCTCAACAACCAAAACTTCTACCTGTCCAAACAACTCGATGAGGCTTCCGGG
GCCAATGACGAGATTGTGCAGCTGCGGAGTGAGGTGGACCATCTCCGCCGTGAGATCACG
GAGCGGGAGATGCAGCTCACCAGCCAGAAGCAAACGATGGAGGCTCTGAAGACGACATGC
ACCATGCTGGAGGAGCAGGTCCTGGACCTGGAGGCCCTGAACGACGAGCTGCTGGAGAAG
GAGCGCCAGTGGGAGGCCTGGCGGAGCGTCCTCGGCGACGAGAAGTCCCAGTTCGAGTGT
CGAGTTCGAGAGCTACAGAGGATGCTGGACACCGAGAAGCAGAGCAGGGCTAGGGCCGAT
CAGCGGATCACCGAGTCGCGCCAGGTGGTGGAGTTGGCGGTGAAGGAACACAAGGCCGAG
ATTCTTGCTCTGCAGCAGGCTCTCAAGGAGCAGAAGCTCAAAGCCGAGAGCCTGTCGGAC
AAGCTCAACGACCTGGAGAAGAAGCACGCCATGCTGGAGATGAACGCCCGGAGCTTACAG
CAGAAACTAGAGACAGAGCGGGAGCTCAAACAGAGGCTTCTGGAGGAGCAAGCCAAATTG
CAGCAGCAGATGGACCTGCAGAAGAACCACATCTTCAGACTGACGCAAGGGCTGCAGGAG
GCGCTGGACCGGGCGGATCTGCTGAAGACAGAAAGGAGCGACCTGGAGTACCAGCTGGAA
AACATTCAGGTTCTCTACTCTCACGAGAAAGTGAAAATGGAAGGCACAATCTCTCAGCAA
ACCAAACTCATTGATTTCCTGCAAGCCAAAATGGACCAGCCTGCTAAAAAGAAAAAGGGT

TTATTTAGTCGACGGAAAGAGGACCCTGCTTTGCCCACACAGGTTCCTCTGCAGTACAAT
GAGCTGAAGCTAGCCCTGGAAAAGGAGAAAGCCCGATGCGCGGAGCTGGAGGAGGCCCTT
CAGAAGACCCGCATCGAACTCCGCTCTGCGCGGGAGGAAGCTGCCCACCGCAAAGCCACG
GACCACCCGCACCCGTCTACGCCAGCCACTGCGAGGCAGCAGATCGCCATGTCTGCCATT
GTGCGGTCGCCCGAGCACCAGCCCAGTGCCATGAGCCTGCTTGCCCCACCATCCAGCCGC
AGAAAGGAGTCGTCAACTCCAGAGGAATTCAGCCGGCGTCTGAAAGAGCGCATGCACCAC
AACATCCCTCACCGGTTTAATGTGGGCCTGAACATGAGAGCCACCAAGTGCGCCGTGTGT
CTGGATACTGTGCACTTTGGACGCCAGGCATCCAAATGCCTCGAATGTCAAGTGATGTGT
CACCCCAAATGCTCCACCTGCTTGCCCGCTACCTGTGGCCTGCCAGCTGAATATGCCACA
CACTTCACTGAGGCCTTCTGCCGGGACAAAATGAACTCCCCGGGGCTCCAGAGCAAGGAG
CCTGGCAGCAGCTTGCACCTGGAAGGGTGGATGAAAGTGCCCAGAAATAACAAACGGGGA
CAGCAAGGCTGGGACAGGAAGTACATTGTTCTGGAGGGGTCAAAAGTCCTCATCTATGAC
AATGAAGCCAGAGAAGCTGGACAGAGGCCGGTGGAAGAATTTGAGCTGTGCCTTCCTGAC
GGGGACGTATCTATTCATGGTGCCGTTGGTGCTTCAGAACTTGCAAATACCGCCAAAGCA
GATGTCCCATACATCCTGAAGATGGAGTCTCACCCACACACCACCTGCTGGCCTGGGAGA
ACCCTCTACTTGCTAGCACCCAGCTTCCCCGACAAGCAGCGCTGGGTCACCGCCTTAGAA
TCTGTCGTCGCAGGTGGGAGAGTTTCTAGGGAAAAGGCCGAAGCCGATGCTAAATTACTT
GGAAACTCTCTGCTGAAACTGGAGGGCGATGACCGGCTTGACATGAACTGCACCCTGCCC
TTCAGTGACCAGGTAGTGCTGGTGGGCACGGAGGAAGGCCTCTACGCCCTGAATGTCTTG
AAAAACTCCTTAACCCACATCCCAGGGATTGGCGCAGTCTTCCAAATTTACATCATCAAG
GACCTGGAGAAGCTGCTCATGATAGCAGGGGAAGAGCGGGCTCTGTGCCTGGTGGACGTG
AAGAAGGTGAAGCAGTCCCTGGCGCAGTCACACCTGCCTGCCCAGCCCGACGTCTCCCCC
AACATATTCGAAGCCGTCAAAGGCTGCCACTTGTTCGCTGCTGGCAAGATCGAGAACAGC
CTGTGCATCTGCGCCGCTATGCCAAGCAAAGTCGTCATCCTCCGCTACAATGACAACCTC
AGCAAGTACTGCATCCGCAAGGAGATCGAGACCTCAGAGCCCTGCAGCTGTATCCACTTC
ACCAACTACAGCATCCTCATTGGGACCAACAAATTCTATGAGATCGACATGAAGCAGTAC
ACGCTTGACGAGTTCCTGGACAAGAACGACCATTCCTTGGCACCTGCTGTGTTCGCCTCC
TCGTCCAACAGCTTCCCTGTCTCCATTGTGCAGGCGAACAGCGCCGGGCAGCGAGAAGAA
TACCTGCTGTGCTTCCACGAATTTGGGGTGTTCGTGGATTCTTACGGAAGACGTAGCCGC
ACAGATGATCTCAAGTGGAGTCGCTTACCTCTGGCCTTCGCCTACAGAGAACCTTATCTG
TTTGTGACTCACTTCAACTCCCTGGAAGTCATTGAGATCCAGGCCAGATCCTCACTGGGG
AGCCCTGCCCGAGCATATCTGGAAATTCCAAACCCTCGCTACCTGGGCCCCGCGATTTCC
TCCGGAGCGATTTACCTGGCCTCCTCATACCAGGACAAGTTAAGGGTCATATGCTGCAAA
GGAAACCTCGTGAAGGAGTCAGGCACTGAGCAGCACCGGGTGCCCTCCACCTCCCGCAGC
AGCCCCAACAAGCGAGGCCCACCAACATACAACGAGCACATCACCAAACGCGTGGCCTCC
AGCCCGGCGCCACCGGAAGGCCCCAGCCACCCCCGAGAGCCAAGCACACCGCACCGCTAC
CGAGACAGAGAGGGTCGGACAGAGCTGCGCAGGGACAAGTCTCCAGGCCGCCCTCTGGAG
CGGGAGAAGTCCCCAGGCCGAATGCTCAGCACTAGGAGAGAGCGGTCCCCAGGGAGACTG
TTTGAAGACAGCAGCAGGGGCCGGCTGCCTGCAGGAGCAGTGAGGACCCCACTGTCCCAG
GTTAACAAGGTCTGGGACCAGTCTTCAGTA
>Marmota marmota marmota XM_015505204.1
ATGCTGAAGTTCAAGTATGGATCACGGAATACACAGGATGCTGGTGCCGTTGAGCCCATTGCCAGCCGGG
CCTCCAGGCTGAATCTCTTCTTCCAGGGGAAACCACCCTTTATGACTCAACAGCAGATGTCTCCTCTTTC
CCGAGAAGGGATATTAGATGCCCTCTTTGTTCTCTTTGAAGAATGCAGTCAGCCTGCTCTGATGAAGATT
AAGCACGTGAGCAACTTCGTCCGGAAGTATTCTGACACCATAGCTGAGTTACAGGAGCTCCAGCCTTCGG
CAAAGGACTTTGAAGTCAGAAGTCTTGTAGGTTGTGGCCACTTTGCTGAAGTGCAGGTGGTAAGAGAGAG
AGCAACTGGGGACATCTATGCCATGAAAATCATGAAGAAGAAGGCCTTGTTGGCCCAGGAGCAGGTTTCA

TTTTTTGAGGAAGAACGGAACATATTATCTCGGAGCACAAGCCCTTGGATCCCCCAATTACAGTATGCCT
TCCAGGATAAAAATAACCTTTACCTGGTCATGGAATATCAGCCTGGAGGGGATTTGCTGTCACTTTTGAA
TAGATATGAGGACCAATTAGATGAAAATATGATTCAGTTTTACCTAGCTGAACTGATTTTGGCTGTTCAC
AGTGTTCATCAGATGGGATATGTACACCGCGACATCAAGCCAGAGAATGTTCTCATTGACCGAACAGGAC
ACATCAAGTTGGTGGATTTTGGATCAGCTGCTAAAATGAATTCAAATCAAATGGTGAACTCCAAACTCCC
AATTGGGACCCCAGATTACATGGCTCCTGAAGTGCTGACCATTATGAATGGGGACGGAAAGGTGTCTAC
GGTACAGACTGTGACTGGTGGTCCGTGGGAGTCATTGCTTACGAAATGGTTTATGGGAGAACCCCATTCA
CGGAGGGAACCTCAGCCAGAACCTTCAATAACATCATGAACTTCCAGCGGTTTTTGAAATTTCCGGATGA
CCCCAAAGTGAGCAGTGGATTCCTTGATCTCATTCAAAGTTTGTTGTGTGGCCAGAGAGAGAGGTTGAAG
TTTGAGGGCCTCTGTTGCCACCCTTTCTTCTCTAAAATCGACTGGAATGACATTCGTAACTCGCCTCCCC
CCTTCGTCCCCACCCTCAAGTCTGATGATGACACCTCCAATTTTGATGAACCAGAGAAGAATTCGTGGGT
TTCATCCTCTCCGTGCCAGCTGAGCCCCTCGGGTTTCTCGGGCGAAGAACTGCCGTTTGTGGGGTTTTCA
TACAGCAAGGCACTGGGGATTCTTGGTAGATCTGAGTCTGTTGTGTCGGGTCTGGACTCCCCTGCCAAGA
CTAGCTCCATGGAAAAGAAACTTCTCATCAAAAGCAAAGAGCTGCAAGACTCTCAGGACAAGTGTCACAA
GATGGAGCAGGAAATGACCCGGTTACATCGGAGAGTGTCAGAGGTGGAGGCTGTGCTTAGTCAGAAGGAG
GTGGAGCTGAAGGCCTCTGAGACTCAGAGATCCCTCCTGGAGCAGGACCTTGCCACCTACATCACAGAAT
GCAGTAGCTTAAAGCGAAGTTTGGAGCAAGCACGGATGGAGGTGTCCCAGGAGGATGACAAAGCACTGCA
GCTTCTCCATGACATCAGAGAGCAGAGCCGGAAGCTCCAAGAAATCAAAGAGCAGGAGTACCAGGCTCAA
GTGGAAGAAATGAGGCTGATGATGAATCAGTTGGAAGAGGATCTTGTCTCAGCAAGAAGACGAAGCGATC
TGTACGAATCTGAGCTGAGAGAGTCTCGGCTTGCCGCCGAAGAGTTCAAGCGGAAAGCGACAGAATGTCA
GCATAAACTGATGAAGGCTAAGGATCAAGGGAAGCCTGAAGCAGGAGAATATTCCAAACTTGAGAAGATC
AATGCCGAGCAGCAGCTCAAAATCCGGGAGCTCCAAGAAAAGCTGGAAAAGGCGGTAAAAGCCAGCACAG
AGGCCACCGAGCTGCTGCAGAACATCCGCCAGGCCAAGGAGCGAGCCGAGAGGGAGCTGGAGAAGCTGCA
GAACCGAGAAGACTCTTCTGAAGGCATAAGAAAGAAGCTGGTGGAAGCCGAGGAACGCCGCCATTCTCTG
GAGAACAAGGTAAAGAGACTAGAGACCATGGAGCGTAGAGAAAACAGACTGAAGGATGACATCCAGACAA
AATCCCAACAGATCCAGCAGATGGCTGATAAAATTCTGGAGCTGGAGGAGAAACACCGGGAGGCCCAGGT
CTCAGCCCAGCACCTAGAAGTACACCTGAAGCAGAAAGAACAGCACTACGAGGAAAAAATTAAAGTGTTG
GACAATCAGATAAAGAAAGACTTGGCAGATAAGGAGAGCCTGGAGAACCTGATGCAGAGACACGAGGAGG
AGGCCCACGAGAAGGGCAAGATTCTCAGCGAGCAGAAGGCGATGATCAACGCTATGGATTCCAAGATCAG
ATCCCTGGAACAGAGGATTGTGGAACTGTCTGAAGCCAATAAACTGGCAGCAAACAGCAGTCTCTTCACC
CAAAGGAACATGAAGGCCCAGGAAGAGATGATTTCAGAACTCAGGCAGCAGAAGTTTTACCTGGAGACGC
AGGCTGGGAAATTGGAGGCCCAGAACCGAAAGCTGGAAGAACAGCTGGAGAAAATCAGCCACCAAGATCA
CAGTGACAAGAGTCGGCTGCTGGAGCTGGAGACCAGGCTGAGGGAGGTCAGCCTGGAGCACGAGGAGCAG
AAACTGGAGCTGAAGCGCCAGCTCACGGAGCTGCAGCTGTCCCTGCAGGAGCGCGAGTCCCAGCTGACAG
CCCTGCAGGCCGCCCGGGCTGCCCTGGAGAGCCAGCTTCGGCAAGCTAAGACAGAGCTGGAGGAGACCAC
AGCAGAAGCGGAAGAGGAGATCCAGGCTCTCACGGCGCATAGAGATGAAATCCAGCGCAAATTTGATGCC
CTTCGTAACAGCTGTACTGTCATCACAGACCTGGAGGAGCAGCTAAACCAGCTGACTGAGGACAACGCTG
AACTCAACAACCAAAACTTCTACCTGTCCAAACAACTCGATGAGGCATCTGGTGCCAACGATGAGATAGT
CCAGCTGCGAAGTGAGGTGGACCATCTTCGCCGCGAGATCACCGAGAGGGAGATGCAGCTCACCAGCCAG
AAGCAAACGATGGAGGCTCTGAAGACCACGTGCACGATGCTGGAGGAGCAGGTCATGGATTTGGAGGCCC
TGAATGATGAGCTGTTGGAGAAGGAGCGCCAGTGGGAGGCCTGGAGGAGTGTCCTTGGTGACGAGAAGTC
CCAGTTTGAGTGTCGGGTTCGAGAGTTACAGAGGATGCTGGACACGGAGAAGCAGAGCAGGGCAAGAGCT
GACCAGCGGATCACTGAGTCTCGCCAGGTGGTGGAGTTGGCAGTGAAGGAGCACAAGGCTGAGATTCTCG
CTCTGCAACAGGCTCTCAAAGAACAGAAGCTGAAAGCTGAGAGCCTCTCTGACAAGCTCAATGACCTGGA
GAAGAAGCACGCCATGCTTGAAATGAATGCCCGAAGCTTGCAGCAGAAACTGGAGACAGAACGAGAGCTC
AAACAGAGGCTTCTGGAAGAGCAAGCCAAGCTACAGCAGCAGATGGACATGCAGAAGAACCACATTTTCC
GTCTGACTCAAGGGCTGCAAGAAGCTTTGGATAGGGCCGATCTGCTGAAGACAGAAAGAAGCGATCTGGA

GTATCAGCTGGAAAACATTCAGGTTCTCTACTCTCATGAAAAGGTGAAGATGGAAGGCACTATTTCTCAA
CAAACCAAACTCATTGATTTTCTGCAAGCCAAAATGGACCAACCTGCTAAAAAGAAAAAGGGTTTATTTA
GTCGACGGAAAGAGGACCCTGCTTTACCCACACAGGTTCCTCTGCAGTACAATGAGCTGAAGCTGGCCCT
GGAGAAGGAAAAAGCTCGCTGTGCAGAGCTGGAGGAAGCCCTTCAGAAGACCCGCATTGAGCTCCGATCT
GCCCGGGAGGAAGCTGCCCACCGCAAAGCCACAGACCACCCACATCCTTCTACTCCAGCCACAGCGAGGC
AGCAGATTGCCATGTCCGCCATTGTGCGGTCACCTGAGCACCAGCCCAGTGCCATGAGCCTGCTTGCCCC
ACCGTCTAGCCGCAGAAAGGAGTCTTCAACCCCAGAAGAATATAGCCGGCGTCTTAAGGAGCGCATGCAC
CACAATATTCCTCACCGATTTAACGTAGGACTGAACATGCGAGCCACAAAGTGTGCTGTGTGTCTGGATA
CTGTACACTTCGGACGTCAGGCATCCAAATGTCTCGAATGTCAGGTGATGTGTCACCCCAAGTGCTCCAC
GTGCTTGCCAGCCACCTGCGGCCTGCCCGCCGAATACGCCACACACTTCACCGAGGCCTTCTGCCGTGAC
AAAATGAACTCCCCGGGTCTGCAGACCAAGGACCCCAGCAGCAGCCTGCACCTGGAAGGGTGGATGAAAG
TGCCCAGGAATAACAAACGAGGACAGCAGGGCTGGGACAGGAAGTACATTGTCCTGGAGGGATCCAAAGT
CCTCATTTATGACAATGAAGCCAGAGAAGCTGGACAGAGGCCGGTGGAAGAATTTGAGCTGTGCCTTCCC
GACGGGGATGTATCTATTCATGGTGCCGTTGGTGCTTCCGAACTCGCAAATACAGCCAAAGCAGATGTCC
CATACATACTGAAGATGGAATCTCACCCGCACACCACGTGCTGGCCCGGGAGGACCCTCTACCTGTTAGC
TCCCAGCTTTCCTGACAAACAGCGCTGGGTCACCGCCTTAGAATCAGTTGTCGCAGGTGGGAGAGTTTCT
AGGGAGAAGGCAGAAGCCGATGCCAAATTGCTTGGAAACTCCCTGCTGAAACTGGAAGGTGATGACCGGC
TGGACATGAACTGCACACTGCCCTTCAGTGACCAGGTGGTGTTGGTGGGCACCGAGGAAGGGCTGTATGC
ACTGAATGTCTTGAAAAACTCCTTAACCCATGTGCCAGGAATTGGAGCAGTCTTCCAAATTTATATCATC
AAGGACCTGGAGAAGCTACTCATGATAGCAGGAGAAGAGCGGGCCCTGTGTCTGGTGGACGTGAAGAAAG
TGAAGCAGTCCCTGGCGCAGTCGCACCTTCCCGCCCAGCCAGACATCTCGCCCAACATCTTCGAAGCTGT
GAAGGGCTGCCACTTGTTTGCTGCTGGCAAGATTGAGAATGGGCTCTGTATCTGTGCAGCCATGCCCAAC
AAAGTCGTCATCCTCCGCTACAACGAAAACCTCAGCAAGTACTGCATTCGGAAGGAGATCGAGACCTCAG
AGCCCTGCAGCTGCATCCACTTCACCAATTATAGTATCCTCATCGGAACCAACAAATTCTACGAAATCGA
CATGAAGCAGTACACGCTAGAGGAATTCCTGGACAAGAACGACCACTCCTTGGCGCCTGCTGTGTTTGCT
TCCTCTTCCAACAGCTTCCCCGTCTCCATCGTGCAGGTGAACGGGGCAGGGCAGCGAGAGGAGTACCTGC
TGTGCTTCCACGAATTTGGGGTGTTCGTGGATTCTTACGGAAGACGTAGCCGCACAGATGATCTCAAGTG
GAGCCGCTTACCCTTGGCCTTTGCCTACAGAGAACCCTATCTGTTTGTGACCCACTTCAACTCACTGGAA
GTCATTGAAATCCAGGCACGCTCCTCGCTGGGGACCCCTGCCCGAGCGTATCTGGAAATCCCGAACCCAC
GCTACCTGGGCCCTGCGATTTCCTCAGGAGCGATTTACCTGGCCTCCTCGTATCAGGATAAATTAAGGGT
CATATGCTGCAAAGGAAACCTTGTGAAGGAGTCTGGCACCGACCACCACCGGGTCCCCTCCACCTCCCGC
AGCCCCAACAAGCGAGGCCCACCGACGTACAACGAGCACATCACCAAGCGCGTGGCCTCCAGTCCGGCGC
CACCCGAAGGCCCCAGCCACCCCCGAGAGCCAAGCACACCCCACCGCTACCGCGAGGGGCGGACAGAGCT
ACGCAGGGACAAGTCTCCCGGCCGCCCCCTGGAGCGCGAGAAGTCCCCAGGCCGCATGCTTAGCACGCGG
AGGGAGCGGTCCCCTGGGAGGCTGTTTGAAGACAGCAGCAGGGGCCGGCTGCCTGTGGGAGCCGTGAGGA
CCCCACTGTCGCAGGTCAACAAGGTCTGGGACCAGTCCTCAGTA
>Equus asinus XM_014862433.1
ATGTTGAAGTTCAAGTATGGGGCTCGGAACCTGCTGGACGCTGGTGCTGCTGAACCTATCGCCAGCCGGG
CCTCCAGGCTGAATCATTTCTTCCAGGGGAAACCACCCTTTATGAGTCAACAGCAGATGTCTCCTCTTTC
CCGAGAAGGAATATTAGATGCCCTCTTTGTTCTCTTTGAAGAATGCAGTCAGCCTGCTCTGATGAAGATT
AAACATGTGAGCAACTTTGTCCGGAAGTATTCTGACACCATAGCTGAGTTACAGGAGCTCCAGCCTTCGG
CAAAGGACTTTGAAGTCAGAAGTCTCGTAGGTTCTGGTCACTTTGCCGAAGTGCAGGTGGTAAGAGAGCG
AGCAACCGGGGATATCTATGCCATGAAAGTCATGAAGAAGCAGGCCTTGTTGGCCCAGGAGCAGGTTTCA
TTTTTTGAGGAAGAACGGAACATATTATCTCGGAGCACAAGCCCTTGGATCCCCCAATTACAGTATGCCT
TTCAGGACAAAAATAACCTCTATCTGGTCATGGAATATCAGCCTGGAGGGGACTTGCTGTCACTTTTGAA
TAGATATGAGGACCAATTAGATGAAAATATGATTCAGTTTTACCTAGCTGAACTGATTTTGGCTGTTCAC
AGCGTTCATCAGATGGGATATGTACATCGAGACATCAAGCCTGAGAACATTCTCATTGACCGAATGGGAC

ACATCAAGCTGGTGGATTTTGGATCAGCTGCTAAAATGAACTCAAATAAAATGGTGAGTGCCAGACTCCC
AGTCGGGACCCCAGATTACATGGCCCCTGAAGTACTGACTGTGATGAACGGGGACGGAAAAGGCGTCTAC
AGCCTAGGCTGTGACTGGTGGTCGGTGGGAGTCATAGCTTATGAGATGGTTTATGGAAGGTCCCCATTCA
CTGAGGGGACCTCAGCCAGAACCTTCAATAACATCATGAATTTCCAGCGGTTTTTGAAGTTTCCAGATGA
CCCCAAAGTTAGCAGTGAATTACTCGATCTGATTCAGAGTTTGTTGTGTGGCCAGAAAGAGAGACTGAAG
TTTGAAGGCCTTTGCTGCCATCCTTTCTTCTCTAGAATCGACTGGAATAGCATTCGTAACTCTCCTCCCC
CGTTCGTTCCCACCCTCAAGTCTGATGATGACACCTCCAATTTTGATGAACCAGAGAAGAATTCGTGGGT
TTCATCCTCTCCGTGCCAGCTGAACCCCTCAGGTTTCTCGGGCGAAGAACTGCCGTTTGTGGGGTTTTCG
TATAGCAAGGCACTGGGGATTCTTGGTAGATCTGAGTCTGTTGTGTCAAGTCTGGACTCCCCTGCCAAGA
CTAGCTCCATGGAAAAGAAACTTCTCATCAAAAGCAAAGAGCTGCAAGACTCCCAGGACAAGTGTCACAA
GATGGAGCAGGAAATGACCCGGTTACATCGGAGAGTGTCAGAGGTGGAGGCTGTGCTTAGTCAGAAGGAG
GTGGAACTGAAGGCCTCTGAGACTCAGAGATCCCTCCTGGAGCAGGACCTTGCTACCTACATCACAGAAT
GCAGTAGCTTAAAGCGAAGTTTGGAGCAAGCACGGATGGAGGTGTCCCAGGAGGATGACAAAGCACTGCA
GCTTCTCCATGATATCAGAGAGCAGAGCCGGAAGCTCCAAGAAATCAAAGAGCAGGAGTACCAGGCTCAA
GTGGAAGAAATGAGGTTGATGATGAATCAGTTGGAAGAAGATCTTGTTTCGGCAAGAAGACGGAGTGATC
TCTATGAATCTGAGCTGAGAGAGTCTCGGCTTGCCGCCGAAGAGTTCAAGCGGAAAGCGACAGAATGTCA
ACATAAACTGATGAAGGCTAAGGATCAAGGGAAGCCTGAAGTGGGAGAATATTCCAAACTGGAGAAGATC
AATGCTGAGCAGCAGCTTAAAATTCAGGAGCTCCAAGAGAAGCTGGAAAAGGCTGTCAAAGCCAGCACTG
AGGCCACCGAGCTGCTGCAGAATATCCGCCAGGCGAAGGAGCGAGCCGAGCGTGAGTTGGAGAAGCTGCA
GAACCGCGAGGATTCATCTGAAGGCATAAAAAAGAAGCTCGTTGAAGCCGAGGAACGCCGCCATTCTCTG
GAGAACAAGGTAAAGAGGCTAGAGACCATGGAGCGTAGAGAAAACAGACTGAAGGATGACATCCAGACAA
AATCCCAACAGATCCAGCAGATGGCTGATAAAATTCTGGAGCTGGAGGAGAAGCACCGGGAGGCCCAGGT
CTCAGCCCAGCACCTAGAGGTGCACCTGAAACAGAAAGAGCAGCACTACGAGGAAAAAATTAAAGTGTTG
GACAATCAGATAAAGAAAGATCTGGCTGATAAGGAGACTCTGGAGAACCTGATGCAGAGACACGAGGAGG
AGGCCCATGAGAAAGGCAAAATTCTCAGCGAGCAGAAGGCGATGATCAATGCCATGGATTCCAAGATCAG
ATCCCTGGAACAGAGGATTGTGGAATTGTCGGAAGCCAATAAACTTGCGGCCAACAGCAGTCTTTTTACC
CAGAGGAACATGAAGGCCCAGGAAGAGATGATTTCAGAACTCAGGCAACAAAAATTTTACCTAGAGACTC
AGGCTGGGAAGTTGGAGGCCCAGAACCGAAAGCTGGAGGAGCAGTTGGAAAAAATCAGCCACCAAGACCA
CAGCGACAAGAATCGTCTGCTGGAGTTGGAGACAAGGTTGAGGGAGGTCAGTCTGGAGCATGAGGAGCAG
AAGCTGGAGCTAAAGCGTCAGCTCACGGAGCTGCAGCTTTCCCTGCAAGAGCGTGAGTCCCAGCTGACAG
CCCTGCAGGCCGCGCGGGCGGCCCTGGAGAGCCAGCTTCGCCAGGCGAAGACGGAGCTGGAAGAGACAAC
AGCAGAAGCAGAAGAAGAGATCCAGGCACTCACGGCACATAGAGATGAAATCCAGCGCAAATTTGATGCC
CTTCGTAACAGCTGTACTGTCATCACAGACCTGGAGGAGCAGCTGAACCAGCTGACCGAGGACAATGCTG
AGCTCAACAACCAAAATTTCTACTTGTCCAAACAACTCGATGAGGCTTCCGGTGCCAACGACGAGATAGT
ACAGCTGCGAAGTGAAGTGGACCATCTCCGCCGTGAGATCACAGAGAGGGAGATGCAGCTCACCAGCCAG
AAGCAAACTATGGAGGCTCTCAAGACCACCTGCACGATGCTGGAAGAACAGGTCATGGATTTGGAGGCCC
TGAACGACGAGCTGCTGGAAAAAGAGCGGCAGTGGGAGGCGTGGAGGAGCGTCCTTGGCGATGAGAAGTC
CCAGTTTGAGTGTCGGGTTCGAGAGTTACAGAGGATGCTGGACACTGAAAAGCAGAGCAGGGCAAGAGCT
GATCAGCGCATCACTGAGTCCCGCCAGGTGGTGGAGCTGGCAGTGAAGGAACACAAGGCCGAGATTCTCG
CTCTGCAGCAGGCTCTCAAAGAACAGAAGCTGAAAGCTGAGAGCCTCTCTGACAAGCTCAACGACCTGGA
GAAGAAACACGCCATGCTGGAAATGAACGCCCGAAGTTTACAACAGAAACTGGAGACCGAAAGAGAGCTC
AAACAAAGGCTTCTGGAAGAGCAAGCCAAATTACAGCAGCAGATGGACCTGCAGAAGAATCATATTTTCC
GGCTGACTCAAGGGCTGCAAGAAGCTCTAGATCGAGCTGATCTGCTGAAGACAGAAAGGAGTGATCTGGA
ATATCAGCTGGAAAACATTCAGGTTCTGTATTCTCATGAAAAGGTGAAAATGGAAGGCACTATTTCTCAA
CAAACCAAACTCATTGATTTTCTGCAAGCCAAAATGGACCAACCTGCTAAAAAGAAAAAGGGTTTATTTA
GTCGACGGAAAGAGGACCCTGCTTTGCCCACACAGGTTCCTCTGCAGTACAATGAGCTGAAGGTGGCCCT
GGAGAAGGAGAAAGCTCGCTGTGCAGAGCTGGAGGAAGCCCTTCAGAAGACCCGCATTGAGCTCCGGTCC

GCCCGGGAGGAAGCTGCCCACCGGAAAGCCACAGACCACCCGCACCCATCTACGCCAGCCACTGCGAGGC
AGCAGATCGCCATGTCTGCCATCGTGCGGTCACCCGAGCACCAGCCCAGTGCCATGAGCCTGCTCGCCCC
GCCTTCCAGCCGCAGAAAGGAGTCTTCAACTCCAGAGGAATTCAGTCGGCGTCTTAAGGAGCGCATGCAC
CACAATATTCCTCACCGATTTAATGTAGGACTGAACATGCGAGCCACAAAGTGCGCTGTGTGTCTGGATA
CTGTGCACTTTGGACGCCAGGCATCCAAATGTCTCGAATGTCAGGTGATGTGTCATCCCAAGTGCTCCAC
GTGCTTGCCAGCCACTTGTGGCCTGCCAGCTGAATATGCCACGCACTTCACTGAGGCCTTCTGCCGTGAC
AAAATGAACTCCCCGGGTCTGCAGACCAAGGAGCCCAGCAGCAGCTTGCACCTGGAAGGGTGGATGAAGG
TTCCCAGGAATAACAAACGAGGACAGCAAGGCTGGGACAGGAAGTACATCGTCCTGGAGGGATCCAAAGT
CCTCATTTATGACAACGAAGCCAGAGAAGCTGGACAGAGGCCGGTGGAAGAATTTGAGCTGTGCCTTCCC
GATGGGGATGTATCTATTCATGGCGCCGTCGGTGCTTCTGAACTTGCAAATACAGCCAAAGCAGATGTCC
CATACATCCTGAAGATGGAATCTCACCCTCATACCACCTGCTGGCCCGGGAGAACCCTCTACTTGCTAGC
TCCCAGCTTCCCCGACAAACAGCGCTGGGTCACCGCCTTAGAATCAGTTGTCGCAGGTGGGAGAGTGTCT
AGGGAAAAAGCAGAAGCTGATGCTAAATTGCTTGGAAACTCCCTGCTGAAACTGGAAGGTGATGACCGTC
TGGACATGAACTGCACACTGCCCTTCAGCGACCAGGTGGTGTTGGTGGGCACCGAGGAAGGGCTCTATGC
ACTGAATGTCTTGAAAAACTCCCTCACGCATGTGCCAGGAATTGGAGCGGTCTTCCAAATTTACATCATC
AAGGACCTGGAGAAGCTACTCATGATAGCAGGGGAAGAGCGGGCCCTGTGTCTTGTTGACGTGAAGAAGG
TGAAGCAGTCTCTGGCACAGTCGCACCTTCCCGCCCAGCCCGACATCTCGCCCAACATCTTCGAAGCTGT
CAAAGGCTGCCACTTGTTCGCTGCTGGCAAGATTGAGAACGGGCTCTGCATCTGTGCAGCCATGCCCAGC
AAAGTTGTCATTCTCCGCTACAATGAAAACCTCAGCAAGTACTGCATTCGGAAAGAGATAGAGACCTCAG
AGCCCTGCAGCTGCATCCACTTCACCAATTACAGTATCCTCATCGGAACCAATAAATTCTACGAAATTGA
CATGAAGCAGTACACACTTGAGGAATTCCTGGATAAGAACGACCATTCCTTGGCGCCGGCTGTGTTTGCC
TCCTCTTCCAACAGCTTTCCTGTCTCGATCGTGCAGGTGAACAGCGCAGGGCAGCGGGAGGAGTACCTGC
TCTGCTTCCACGAATTCGGGGTGTTCGTGGATTCTTACGGAAGACGTAGCCGCACAGATGATCTCAAGTG
GAGTCGCTTACCTTTGGCCTTTGCCTACAGAGAACCCTATCTGTTTGTGACCCACTTCAACTCACTTGAA
GTAATTGAGATCCAGGCACGCTCCTCTCTGGGGACCCCCGCCCGAGCATATTTGGAAATCCCGAACCCAC
GCTACCTGGGCCCTGCAATTTCCTCAGGAGCGATTTACCTGGCGTCCTCATACCAGGATAAATTAAGGGT
CATTTGCTGCAAAGGAAACCTTGTGAAGGAGACTGGCACTGACCACCACCGGGGCCCCTCCACCTCCCGC
AGCAGCCCCAACAAGCGAGGCCCGCCAACGTACAACGAGCACATCACCAAGCGCGTGGCCTCCAGCCCAG
CACCACCCGAAGGCCCCAGCCACCCGCGAGAGCCAAGCACACCCCACCGCTACCGAGAGGGGCGGACGGA
GCTGCGCAGAGACAAGTCTCCTGGCCGCCCTCTGGAGCGCGAGAAGTCCCCGGGCCGGATGCTCAGCACG
CGGAGGGAACGGTCCCCTGGGAGGCTCTTTGAAGACAGCAGCAGGGGCCGGCTGCCTGTGGGAGCCGTGC
GAACCCCACTGTCCCAGGTCAACAAGGTGTGGGACCAGTCTTCAGTA
>Equus przewalskii XM_008507336.1
ATGTTGAAGTTCAAGTATGGGGCTCGGAACCTGCTGGACGCTGGTGCTGCTGAACCTATCGCTAGCCGGG
CCTCCAGGCTGAATCATTTCTTCCAGGGGAAACCACCCTTTATGAGTCAACAGCAGATGTCTCTTTCCCG
AGAAGGAATATTAGATGCCCTCTTTGTTCTCTTTGAAGAATGCAGTCAGCCTGCTCTGATGAAGATTAAA
CACGTGAGCAACTTTGTCCGGAAGTATTCTGACACCATAGCTGAGTTACAGGAGCTCCAGCCTTCGGCAA
AGGACTTTGAAGTCAGAAGTCTCGTAGGTTCTGGTCACTTTGCCGAAGTGCAGGTGGTAAGAGAGCGAGC
AACCGGGGATATCTATGCCATGAAAGTCATGAAGAAGCAGGCCTTGTTGGCCCAGGAGCAGGTTTCATTT
TTTGAGGAAGAACGGAACATATTATCTCGGAGCACAAGCCCTTGGATCCCCCAATTACAGTATGCCTTTC
AGGACAAAAATAACCTCTATCTGGTCATGGAATATCAGCCTGGAGGGGACTTGCTGTCACTTTTGAATAG
ATATGAGGACCAATTAGATGAAAATATGATTCAGTTTTACCTAGCTGAACTGATTTTGGCTGTTCACAGC
GTTCATCAGATGGGATATGTACATCGAGACATCAAGCCTGAGAACATTCTCATTGACCGAATGGGACACA
TCAAGCTGGTGGATTTTGGATCAGCTGCTAAAATGAACTCAAATAAAATGGTGAGTGCCAGACTCCCAGT
CGGGACCCCAGATTACATGGCCCCTGAAGTACTGACTGTGATGAACGGGGATGGAAAAGGCGTCTACAGC
CTAGGCTGTGACTGGTGGTCGGTGGGAGTCATAGCTTATGAGATGGTTTATGGAAGGTCCCCATTCACTG
AGGGGACCTCAGCCAGAACCTTCAATAACATCATGAATTTCCAGCGGTTTTTGAAGTTTCCAGATGACCC

CAAAGTTAGCAGTGAATTACTTGATCTGATTCAGAGTTTGTTGTGTGGCCAGAAAGAGAGACTGAAGTTT
GAAGGCCTTTGCTGCCATCCTTTCTTCTCTAGAATCGACTGGAATAGCATTCGTAACTCTCCTCCCCCGT
TCGTTCCCACCCTCAAGTCTGATGATGACACCTCCAATTTTGATGAACCAGAGAAGAATTCGTGGGTTTC
ATCCTCTCCGTGCCAGCTGAACCCCTCAGGTTTCTCGGGCGAAGAACTGCCGTTTGTGGGGTTTTCGTAT
AGCAAGGCACTGGGGATTCTTGGTAGATCTGAGTCTGTTGTGTCAAGTCTGGACTCCCCTGCCAAGACTA
GCTCCATGGAAAAGAAACTTCTCATCAAAAGCAAAGAGCTGCAAGACTCCCAGGACAAGTGTCACAAGAT
GGAGCAGGAAATGACCCGGTTACATCGGAGAGTGTCAGAGGTGGAGGCTGTGCTTAGTCAGAAGGAGGTG
GAACTGAAGGCCTCTGAGACTCAGAGATCCCTCCTGGAGCAGGACCTTGCTACCTACATCACAGAATGCA
GTAGCTTAAAGCGAAGTTTGGAGCAAGCACGGATGGAGGTGTCCCAGGAGGATGACAAAGCACTGCAGCT
TCTCCATGATATCAGAGAGCAGAGCCGGAAGCTCCAAGAAATCAAAGAGCAGGAGTACCAGGCTCAAGTG
GAAGAAATGAGGTTGATGATGAATCAGTTGGAAGAGGATCTTGTTTCGGCAAGAAGACGGAGTGATCTCT
ATGAATCTGAGCTGAGAGAGTCTCGGCTTGCCGCCGAAGAGTTCAAGCGGAAAGCGACAGAATGTCAGCA
TAAACTGATGAAGGCTAAGGATCAAGGGAAGCCTGAAGTGGGAGAATATTCCAAACTGGAGAAGATCAAT
GCTGAGCAGCAGCTTAAAATTCAGGAGCTCCAAGAGAAGCTGGAAAAGGCTGTCAAAGCCAGCACTGAGG
CCACCGAGCTGCTGCAGAACATCCGCCAGGCGAAGGAGCGAGCCGAGCGTGAGCTGGAGAAGCTGCAGAA
CCGCGAGGATTCATCTGAAGGCATAAAAAAGAAGCTGGTTGAAGCCGAGGAACGCCGCCATTCTCTGGAG
AACAAGGTAAAGAGGCTAGAGACCATGGAGCGTAGAGAAAACAGACTGAAGGATGACATCCAGACAAAAT
CCCAACAGATCCAGCAGATGGCTGATAAAATTCTGGAGCTGGAGGAGAAGCACCGGGAGGCCCAGGTCTC
AGCCCAGCACTTAGAGGTGCACCTGAAACAGAAAGAGCAGCACTACGAGGAAAAAATTAAAGTGTTGGAC
AATCAGATAAAGAAAGATCTGGCTGATAAGGAGACTCTGGAGAACCTGATGCAGAGACACGAGGAGGAGG
CCCATGAGAAAGGCAAAATTCTCAGCGAGCAGAAGGCGATGATCAATGCCATGGATTCCAAGATCAGATC
CCTGGAACAGAGGATTGTGGAATTGTCGGAAGCCAATAAACTTGCGGCCAACAGCAGTCTTTTTACCCAG
AGGAACATGAAGGCCCAGGAAGAGATGATTTCAGAACTCAGGCAACAAAAATTTTACCTGGAGACTCAGG
CTGGGAAGTTGGAGGCCCAGAACCGAAAGCTGGAGGAGCAGTTGGAAAAAATCAGCCACCAAGACCACAG
CGACAAGAATCGTCTGCTGGAGTTGGAGACGAGGTTAAGGGAGGTCAGTCTGGAGCATGAGGAGCAGAAG
CTGGAGCTAAAGCGTCAGCTCACGGAGCTGCAGCTCTCCCTGCAAGAGCGTGAGTCCCAGCTGACAGCCC
TGCAGGCCGCGCGGGCGGCCCTGGAGAGCCAGCTTCGCCAGGCAAAGACGGAGCTGGAAGAGACAACAGC
AGAAGCAGAAGAAGAGATCCAGGCACTCACGGCACATAGAGATGAAATCCAGCGCAAATTTGATGCCCTT
CGTAACAGCTGTACTGTCATCACAGACCTGGAGGAGCAGCTGAACCAGCTGACCGAGGACAATGCTGAGC
TCAACAACCAAAATTTCTACTTGTCCAAACAACTCGATGAGGCTTCCGGTGCCAACGACGAGATAGTACA
GCTGCGAAGTGAAGTGGACCATCTCCGCCGTGAGATCACAGAGAGAGAGATGCAGCTCACCAGCCAGAAG
CAAACTATGGAGGCTCTCAAGACCACCTGCACGATGCTGGAAGAACAGGTCATGGATTTGGAGGCCCTGA
ACGACGAGCTTCTGGAAAAAGAGCGGCAGTGGGAGGCGTGGAGGAGCGTCCTTGGCGATGAGAAGTCCCA
GTTTGAGTGTCGGGTTCGAGAGTTACAGAGGATGCTGGACACTGAAAAGCAGAGCAGGGCAAGAGCTGAT
CAGCGCATCACTGAGTCGCGCCAGGTGGTGGAGCTGGCAGTGAAGGAACACAAGGCCGAGATTCTCGCTC
TGCAGCAGGCTCTCAAAGAACAGAAGCTGAAAGCTGAGAGCCTCTCTGACAAGCTCAACGACCTGGAGAA
GAAACACGCCATGCTGGAAATGAATGCCCGAAGTTTACAACAGAAACTGGAGACCGAAAGAGAGCTCAAA
CAAAGGCTTCTGGAAGAGCAAGCCAAATTACAGCAGCAGATGGACCTGCAGAAGAATCATATTTTCCGGC
TGACTCAAGGGCTGCAAGAAGCTCTAGATCGAGCTGATCTGCTGAAGACAGAAAGGAGTGATCTGGAATA
TCAGCTGGAAAACATTCAGGTTCTGTATTCTCATGAAAAGGTGAAAATGGAAGGCACTATTTCTCAGCAA
ACCAAACTCATTGATTTTCTGCAAGCCAAAATGGACCAACCTGCTAAAAAGAAAAAGGGTTTATTTAGTC
GACGGAAAGAGGACCCTGCTTTGCCCACACAGGTTCCTCTGCAGTACAATGAGCTGAAGGTGGCCCTGGA
GAAGGAGAAAGCTCGCTGTGCAGAGCTGGAGGAAGCCCTTCAGAAGACCCGCATTGAGCTCCGGTCCGCC
CGGGAGGAAGCTGCCCACCGGAAAGCCACAGACCACCCGCACCCATCTACGCCAGCCACTGCGAGGCAGC
AGATCGCCATGTCCGCCATCGTGCGGTCACCCGAGCACCAGCCCAGTGCCATGAGCCTGCTCGCCCCGCC
TTCCAGCCGCAGAAAGGAGTCTTCAACTCCAGAGGAATTCAGTCGGCGTCTTAAGGAGCGCATGCACCAC
AATATTCCTCACCGATTTAATGTAGGACTGAACATGCGAGCCACAAAGTGTGCTGTGTGTCTGGATACTG

TGCACTTTGGACGCCAGGCATCCAAATGTCTCGAATGTCAGGTGATGTGTCATCCCAAGTGCTCCACGTG
CTTGCCAGCCACTTGTGGCCTGCCAGCCGAATATGCCACGCATTTCACTGAGGCCTTCTGCCGTGACAAA
ATGAACTCCCCGGGTCTGCAGACCAAGGAGCCCAGCAGCAGCTTGCACCTGGAAGGGTGGATGAAGGTTC
CCAGGAATAACAAACGAGGACAGCAAGGCTGGGACAGGAAGTACATCGTCCTGGAGGGATCCAAAGTCCT
CATTTATGACAACGAAGCCAGAGAAGCTGGACAGAGGCCGGTGGAAGAATTTGAGCTGTGCCTTCCCGAT
GGGGACGTATCTATTCATGGCGCCGTCGGTGCTTCTGAACTTGCAAATACAGCCAAAGCAGATGTCCCAT
ACATCCTGAAGATGGAATCTCACCCTCATACCACCTGCTGGCCCGGGAGAACCCTCTACTTGCTAGCTCC
CAGCTTCCCCGACAAACAGCGCTGGGTCACCGCCTTAGAATCAGTTGTCGCAGGTGGGAGAGTGTCTAGG
GAAAAAGCAGAAGCTGATGCTAAATTGCTTGGAAACTCCCTGCTGAAACTGGAAGGTGATGACCGTCTGG
ACATGAACTGCACACTGCCCTTCAGCGACCAGGTGGTGTTGGTGGGCACCGAGGAAGGGCTCTATGCACT
GAATGTCTTGAAAAACTCCCTCACGCATGTGCCAGGAATTGGAGCGGTCTTCCAAATTTACATCATCAAG
GACCTGGAGAAGCTACTCATGATAGCAGGGGAAGAGCGGGCCCTGTGTCTTGTTGACGTGAAGAAGGTGA
AGCAGTCTCTGGCACAGTCGCACCTTCCCGCCCAGCCCGACATCTCGCCCAACATCTTCGAAGCTGTCAA
AGGCTGCCACTTGTTCGCTGCTGGCAAGATTGAGAACGGGCTCTGCATCTGTGCAGCCATGCCCAGCAAA
GTTGTCATTCTCCGCTACAATGAAAACCTCAGCAAGTACTGCATTCGGAAAGAGATAGAGACCTCAGAGC
CCTGCAGCTGCATCCACTTCACCAATTACAGTATCCTCATCGGAACCAATAAATTCTACGAAATTGACAT
GAAGCAGTACACACTTGAGGAATTCCTGGATAAGAACGACCATTCCTTGGCGCCGGCTGTGTTTGCCTCC
TCTTCCAACAGCTTTCCTGTCTCGATCGTGCAGGTGAACAGCGCAGGGCAGCGGGAGGAGTACCTGCTCT
GCTTCCACGAATTCGGGGTGTTCGTGGATTCTTACGGAAGACGTAGCCGCACAGACGATCTCAAGTGGAG
TCGCTTACCTTTGGCCTTTGCCTACAGAGAACCCTATCTGTTTGTGACCCACTTCAACTCACTTGAAGTA
ATCGAGATCCAGGCACGCTCCTCTCTGGGGACCCCCGCCCGAGCATATTTGGAAATCCCGAACCCACGCT
ACCTGGGCCCTGCAATTTCCTCAGGAGCGATTTACCTGGCGTCCTCATACCAGGATAAATTAAGGGTCAT
TTGCTGCAAAGGAAACCTTGTGAAGGAGACTGGCACTGACCACCACCGGGGCCCCTCCACCTCCCGCAGC
AGCCCCAACAAGCGAGGCCCGCCAACATACAACGAGCACATCACCAAGCGCGTGGCCTCCAGCCCAGCAC
CACCCGAAGGCCCCAGCCACCCGCGAGAGCCAAGCACACCCCACCGCTACCGAGAGGGGCGGACGGAGCT
GCGCAGAGACAAGTCTCCTGGCCGCCCTCTGGAGCGCGAGAAGTCCCCGGGCCGGATGCTCAGCACGCGG
AGGGAACGGTCCCCCGGGAGGCTCTTTGAAGACAGCAGCAGGGGCCGGCTGCCTGTGGGAGCCGTGCGAA
CCCCACTGTCCCAGGTCAACAAGGTGTGGGACCAGTCTTCAGTA
>Sus scrofa XM_021073543.1
ATGTTGAAGTTCAAGTATGGAGCACGGAATCCGCTGGATGCCGGAGCTGCTGAACCCATTGCCAGCAGGG
CCTCCAGGCTGAATCTTTTCTTCCAGGGGAAACCACCCTTCATGACTCAACAACAGATGTCTCCTCTTTC
CCGAGAAGGGATATTAGATGCCCTCTTTGTTCTCTTTAAAGAATGCAGTCAGCCGGCTCTGATGAAGATT
AAGCACGTGAGCAGCTTTGTCCAGAAGTATGCTGATGTCATAGCTGAGTTGCAGGAGCTCCAGCCTTCAG
CAAAGGACTTCGAAGTGAGAAGTCTCGTGGGTTGCGGTCACTTTGCCGAAGTGCAAGTGGTCCGAGAGAA
GGCGACCGGGGACATCTATGCCATGAAAGTCATGAAGAAGAAGGCCTTGTTGGCCCAGGAGCAGGTTTCA
TTTTTTGAGGAAGAACAGAACATCTTATCCCGGAGCACAAGCCCTTGGATCCCCCAGTTACAGTATGCCT
TTCAGGACAAACATAACCTTTATCTGGTCATGGAATATCAGCCTGGAGGGGATTTGCTGTCACTTTTGAA
TAGATATGAGGACCAATTAGATGAAAATATGATACAGTTTTACCTAGCTGAAATGATTTTGGCTGTTCAC
AGCGTTCATCAGATGGGATATGTACATCGAGACATCAAGCCTGAGAACATTCTCATCGACCGAACGGGAC
ACATCAAGCTGGTGGACTTTGGATCAGCTGCTAAGATGAACTCAAATAAGATGGTGAATGCCAAACTCCC
GGTTGGGACCCCAGATTACATGGCCCCTGAAGTGCTGACTGTGATGAATGGGGATGGAAAAGGTGCCTAT
GGCCTAGACTGTGATTGGTGGTCAGTGGGAGTTATTGCTTATGAGATGGTTTATGGAAGGTCCCCATTCA
CTGAGGGAACCTCAGCCAGAACCTTCAATAACATCATGAATTTCCAGCGGTTTTTGAAGTTTCCAGATGA
CCCCAAAGTTAGCAGTGAATTGCTTGATCTGATCCAAAGTTTATTGTGTGGCCAGAAAGAGAGACTGAAG
TTTGAAGGCCTTTGCTGTCATCCTTTCTTCTCTAAAATCGACTGGAATAACATTCGTAACTCTCCTCCCC
CCTTCGTTCCCACCCTCAAGTCTGATGATGACACCTCCAATTTTGATGAACCAGAGAAGAATTCGTGGGT
TTCATCCTCTCCGTGCCAGCTGAACCCCTCGGGTTTCTCGGGCGAAGAACTGCCGTTTGTGGGGTTTTCC

TATAGCAAGGCACTGGGAATTCTTGGTAGATCTGAGTCTGTTGTGTCAAGTCTGGACTCCCCTGCCAAGA
CTAGCTCCATGGAAAAGAAACTTCTCATCAAAAGCAAAGAGCTGCAAGACTCCCAGGACAAGTGTCACAA
GATGGAGCAGGAAATGACCCGGTTACATCGGAGAGTGTCAGAGGTGGAGGCTGTGCTTAGTCAGAAGGAG
GTGGAGCTGAAGGCCTCTGAGACTCAGAGATCCCTCCTGGAGCAGGACCTTGCTACCTACATCACAGAAT
GCAGTAGCTTAAAGCGAAGTTTGGAGCAAGCTCGGATGGAGGTGTCCCAGGAGGACGACAAAGCACTGCA
GCTTCTCCACGATATCAGAGAGCAGAGCCGGAAGCTCCAAGAAATCAAAGAGCAGGAGTACCAGGCTCAA
GTGGAAGAAATGAGGTTGATGATGAATCAGTTGGAAGAGGACCTCGTTTCTGCAAGAAGACGGAGTGATC
TCTACGAATCGGAGCTGAGAGAGTCCCGGCTGGCGGCTGAAGAGTTCAAGCGCAAAGCAACGGAATGCCA
GCATAAACTGATGAAGGCTAAAGATCAAGGGAAGCCTGAAGTGGGAGAATATTCCAAACTGGAAAAGATC
AATGCAGAGCAGCAGCTAAAAATTCAAGAGCTCCAAGAGAAGCTGGAAAAGGCCGTGAAAGCCAGCACCG
AGGCCACTGAGCTACTGCAGAACATCCGCCAGGCAAAGGAACGGGCGGAGCGGGAGCTGGAGAAGCTGCA
TAACCGCGAGGACTCGTCCGAAGGCATCAGAAAGAAGCTGGTGGAAGCCGAGGAACGCCGCCATTCTCTG
GAGAACAAGGTAAAGAGGCTAGAGACCATGGAGCGTAGAGAAAACAGACTGAAGGATGACATCCAGACAA
AGTCCCAACAGATCCAGCAGATGGCTGATAAAATTCTGGAGCTGGAGGAGAAGCACCGGGAAGCTCAGGT
CTCAGCCCAGCACCTGGAGGTGCACCTGAAACAGAAAGAACAGCACTATGAGGAAAAAATTAAAGTGTTG
GACAACCAGATAAAGAAAGACCTCGCCGATAAGGAGACTCTGGAGAATCTGATGCAGAGACACGAAGAGG
AGGCCCACGAGAAGGGCAAAATTCTCAGCGAGCAGAAGGCGATGATCAATGCCATGGATTCCAAGATCAG
ATCCCTGGAGCAGAGGATAGTGGAATTGTCAGAAGCCAATAAACTGGCAGCCAACAGCAGCCTCTTCACC
CAGAGGAACATGAAGGCCCAGGAAGAGATGATCTCAGAACTCAGGCAACAGAAGTTTTACCTGGAGACGC
AGGCTGGGAAGTTGGAGGCCCAGAACCGGAAGCTGGAGGAGCAGCTGGAAAAAATCAGCCACCAGGACCA
CAGCGACAAGAACCGCCTGCTGGAGCTGGAGACCCGGCTGAGGGAGGTCAGTCTGGAGCATGAAGAGCAG
AAGCTGGAGCTCAAGCGCCAGCTCACAGAGCTGCAGCTCTCCCTGCAAGAACGTGAGTCCCAGCTGACAG
CCCTACAGGCAGCCCGGGCAGCCCTGGAGAGCCAGCTACGCCAGGCAAAGACAGAGCTGGAAGAGACAAC
AGCGGAAGCAGAAGAAGAGATCCAGGCGCTCACGGCACATAGAGATGAAATCCAGCGCAAATTTGATGCC
CTTCGTAACAGCTGTACTGTAATCACAGACCTGGAGGAGCAGCTCAACCAGCTGACGGAGGACAATGCCG
AGCTCAACAACCAAAACTTCTACTTGTCCAAACAACTCGACGAGGCCTCGGGTGCCAACGACGAGATTGT
ACAGCTGAGAAGTGAAGTGGACCACCTCCGCCGCGAGATCACAGAGAGGGAAATGCAGCTCACCAGCCAG
AAGCAAACGATGGAGGCTCTGAAGACCACGTGCACGATGCTGGAGGAGCAGGTCATGGATCTGGAGGCCC
TGAACGATGAGCTGCTGGAGAAGGAGAGGCAGTGGGAGGCATGGAGGAGCGTTCTTGGTGACGAGAAGTC
CCAGTTTGAGTGTCGGGTTCGAGAGCTGCAGAGGATGCTGGACACTGAGAAGCAGAGCAGGGCGAGGGCT
GATCAGCGGATCACCGAGTCTCGCCAGGTGGTGGAGCTGGCGGTCAAGGAGCACAAGGCCGAGATTCTGG
CCCTGCAGCAGGCTCTCAAGGAACAGAAGCTGAAAGCTGAGAGCCTCTCCGACAAGCTCAATGACCTGGA
GAAGAAACACGCCATGCTTGAAATGAATGCCCGAAGTTTACAGCAGAAGCTGGAGACGGAACGAGAGCTC
AAACAAAGGCTTCTGGAAGAGCAAGCCAAACTGCAGCAGCAGATGGACCTGCAGAAGAATCATATTTTCC
GTCTGACTCAAGGGCTGCAAGAAGCTCTTGATCGGGCTGACCTGCTGAAGACAGAAAGGAGTGATCTGGA
ATATCAGCTAGAAAACATCCAGGTTCTCTATTCTCATGAAAAGGTGAAAATGGAAGGCACTATTTCTCAA
CAAACCAAACTCATCGATTTTCTGCAAGCCAAAATGGACCAACCTGCTAAGAAGAAAAAGGGTTTATTTA
GTCGACGGAAAGAGGACCCTGCTTTGCCCACACAGGTTCCTCTGCAGTACAATGAGCTGAAGGTGGCTCT
GGAGAAGGAGAAAGCTCGCTGTGCGGAGCTCGAGGAAGCCCTGCAGAAGACACGCATCGAGCTCCGCTCC
GCCCGGGAGGAAGCTGCCCACCGAAAGGCCACGGACCACCCGCACCCGTCCACTCCGGCCACCGCCAGGC
AGCAGATCGCCATGTCCGCCATCGTGCGCTCCCCCGAGCACCAGCCCAGCGCCATGAGCCTGCTCGCCCC
GCCGTCCAGCCGCAGAAAGGAGTCCTCAACTCCAGAGGAGTTCAGTCGGCGACTCAAGGAGCGCATGCAC
CACAACATTCCTCACCGATTCAATGTAGGACTGAACATGCGAGCCACAAAGTGTGCTGTGTGTCTGGATA
CTGTGCACTTCGGACGCCAGGCATCCAAGTGCCTCGAATGTCAGGTCATGTGTCACCCCAAGTGCTCCAC
GTGCTTGCCAGCTACCTGTGGCCTGCCAGCCGAATATGCCACGCACTTCACCGAGGCCTTCTGCCGTGAC
AAAATGAACTCCCCAGGTCTCCAGGCCAAGGAGCCCAGCAGCAGCTTGCACCTGGAAGGGTGGATGAAGG
TGCCCAGGAATAACAAACGAGGGCAGCAGGGCTGGGACAGGAAGTACATCGTCCTGGAGGGCTCGAAAGT

CCTCATTTACGACAGCGAAGCCAGAGAAGCTGGACAGAGGCCGGTGGAAGAATTTGAGCTGTGCCTTCCC
GACGGGGACGTGTCTATTCATGGCGCCGTTGGTGCTTCTGAACTTGCCAATACAGCCAAAGCAGATGTCC
CATACGTACTGAAGATGGAGTCTCACCCGCATACCACCTGTTGGCCCGGGAGAACCCTCTACTTGCTAGC
GCCCAGCTTTCCTGACAAACAGCGCTGGGTCACCGCCTTAGAATCGGTTGTCGCAGGTGGGAGAGTGTCT
AGGGAAAAGGCAGAAGCTGATGCTAAATTGCTTGGAAACTCACTGCTGAAGCTGGAAGGCGATGACCGTC
TGGACATGAACTGCACACTGCCCTTCAGTGACCAGGTGGTGCTGGTGGGCACAGAGGAGGGGCTCTACGC
GCTGAATGTCTTGAAAAACTCCCTCACCCACGTCCCCGGAATCGGCGCAGTCTTCCAAATTTATATCATC
AAGGACCTAGAGAAGCTCCTCATGATAGCAGGAGAGGAGCGGGCCCTGTGTCTCGTGGACGTGAAGAAAG
TGAAACAGTCCCTGGCACAGTCTCACCTTCCCGCCCAGCCAGACATCTCGCCCAACATCTTCGAAGCTGT
CAAGGGCTGCCACTTGTTTGCTGCCGGCAAGATTGAGAGCGGGCTCTGCATCTGCGCAGCCATGCCCAAC
AAAGTTGTCATTCTCCGCTACAACGAAAACCTCAGCAAGTACTGCATTCGGAAAGAGATCGAGACCTCGG
AGCCCTGCAGCTGCATCCACTTCACCAACTACAGTATCCTCATCGGAACCAATAAATTCTACGAGATTGA
CATGAAGCAGTATACCCTCGAGGAATTCCTGGATAAGAATGACCACTCCTTGGCACCTGCCGTGTTTGCC
TCCTCTTCCAACAGTTTCCCCGTGTCCATCATGCAGGTCAACGGCCCAGGGCAGCGGGAGGAGTTCCTGC
TCTGCTTCCATGAATTCGGGGTGTTCGTGGATTCTTATGGAAGACGGAGCCGCACAGACGATCTCAAGTG
GAGTCGCTTACCTTTGGCCTTTGCCTACAGAGAACCCTATCTGTTTGTGACCCACTTCAACTCACTCGAA
GTAATTGAGATCCAGGCACGCTCCTCTCTGGGGACCCCTGCCCGAGCATATCTGGAAATCCCGAACCCAC
GCTACCTGGGCCCTGCAATTTCCTCAGGAGCGATTTACCTGGCATCCTCGTACCAGGATAAATTAAGGAT
CATTTGCTGCAAAGGAAACCTCGTGAAGGAGTCTGGCACTGACCACCACCGGGGCCCGTCCACCTCCCGC
AGCAGCCCTAACAAGCGAGGCCCGCCGACCTACAACGAGCACATCACCAAGCGTGTGGCCTCCAGCCCGG
CGCCCCCCGAAGGCCCCAGCCACCCAAGAGAGCCAAGCACACCCCACCGCTATCGCGAGGGGCGGACAGA
GCTGCGCAGGGACAAGTCCCCGGGCCGCCCCCTGGAACGGGAGAAGTCCCCAGGCCGGATGCTCAGCACG
CGGCGGGAGCGCTCCCCGGGACGGCTGTTCGAGGACAGCAGCAGGGGCCGGCTGCCTGTGGGGGCTGTGA
GGACCCCGCTGTCCCAGGTCAACAAGGTCTGGGACCAGTCTTCAGTA
>Myotis lucifugus XM_006096555.2
ATGTTGAAGTTCAAGTATGGAGCACGGAGTCAGCTGGACGCTGGCGCTGCTGAGCCCATTGCCGCCCGGG
CCTCCAGGCTGAATCTGTTCTTCCAGGGGAAACCGCCCTTGATGACTCAACAGCAGATGTCTCCTCTTTC
CCGAGAAGGGATATTAGATGCCCTCTTTGTTCTCTTTGAAGAATGCAGCCAGCCTGCTCTGATGAAGATG
AAGCATGTGAGCAACTTCGTCCGCAAGTATTCCAACACCATCGCTGAGCTGCAGGAGCTCCAGCCTTCTG
CGAAGGACTTTGAAGTCAGAAGTCTGGTGGGTTGTGGACACTTTGCTGAAGTGCAGGTGGTAAGAGAGAG
AGCCACCGGGGACATCTATGCTATGAAAGTGATGAAGAAGAAGGCCGTGTTGGCCCAGGAGCAGGTTTCA
TTTTTTGAGGAAGAACGGAACATATTATCTCAGAGCACAAGTCCTTGGATCCCCCATTTACAGTATGCCT
TTCAGGACAAAAATAACCTTTATCTGGTCATGGAATATCAGCCTGGAGGGGACTTGCTGTCACTTTTGAA
TAGATATGAGGACCAATTAGATGAAAACATGATTCAGTTTTACCTTGCTGAACTGATTTTGGCTGTTCAC
AGCGTTCATCTGATGGGATATGTACATCGAGACCTCAAGCCTGAGAATATTCTCATTGATCGGACGGGAC
ACATCAAGCTGGTGGATTTTGGATCAGCTGCTAAAATGAACTCAAATAAGATGGTGAATGCCAAACTCCC
GATTGGGACCCCAGATTACATGGCCCCGGAAGTGTTGACAGTGATGAATGGGGACGGAAAAGGTGTCTAC
AGCCTCGACTGTGACTGGTGGTCAGTGGGAGTTATTGCTTATGAGATGGTGTATGGAAGGTCTCCGTTCA
CTGAGGGAACTTCGGCCAGAACCTTTAATAACATCATGAATTTCCAGCGGTTTTTGAAGTTTCCAGATGA
CCCCAAAGTTAGCAGTGAATTACTGGATCTGATTCAAAGCTTGTTGTGTGGCCAGAAAGAGAGACTGAAG
TTTGAAGGCCTTTGCTGCCATCCTTTTTTCTCAAAAATCGACTGGAATAACATTCGTAACTCTCCTCCCC
CCTTCGTTCCCACCCTCAAGTCTGATGATGACACCTCCAATTTTGATGAACCAGAGAAGAATTCGTGGGT
TTCATCCTCTCCGTGCCAGCTGAACCCCTCAGGCTTCTCCGGCGAAGAACTGCCGTTTGTGGGGTTTTCA
TATAGCAAGGCACTGGGGATTCTTGGTAGATCTGAGTCTGTTGTGTCGGGCCTGGACTCCCCTGCCAAGA
CTAGCTCCATGGAAAAGAAACTTCTCATCAAAAGCAAAGAGCTGCAAGACTCCCAGGACAAGTGTCACAA
GATGGAGCAGGAAATGACCCGGTTACATCGGAGAGTGTCAGAGGTGGAGGCTGTGCTTAGTCAGAAGGAG
GTGGAGCTGAAGGCCTCTGAGACTCAGAGATCCCTCCTGGAGCAGGACCTTGCTACCTACATCACAGAAT

GCAGTAGCTTAAAGCGAAGTTTGGAGCAAGCACGGATGGAGGTGTCCCAGGAGGATGACAAAGCACTGCA
GCTTCTCCATGATATCAGAGAGCAGAGCCGGAAGCTCCAGGAAATCAAAGAGCAGGAGTACCAAGCTCAA
GTGGAAGAAATGAGGTTAATGATGAACCAGTTGGAAGAGGACCTGGTTTCGGCAAGAAGACGGAGTGACC
TCTATGAATCGGAGCTGAGAGAGTCCAGGCTCGCCGCCGAAGAGTTCAAGCGGAAAGCGACAGAAAGTCA
GCATAAACTGATGAAGGCGAAGGATCAAGGGAAGCCTGAAGTGGGAGAATATTCCAAACTGGAGAAGATC
AATGCTGAGCAGCAGCTCAAAATTCAAGAGCTCCAAGAAAAGCTGGAGAAGGCTGTAAAAGCCAGCACCG
AGGCCACCGAGCTGCTGCAGAATATCCGCCAGGCGAAGGAGCGAGCCGAGCGCGAGCTGGAGAAGCTGCA
GAACCGCGAGGATTCTTCCGAAGGCATAAAAAAGAAGCTGATGGAAGCCGAGGAACGTCGCCATTCTCTG
GAGAACAAGGTAAAGAGGCTAGAGACCATGGAGCGTAGAGAAAACAGACTGAAGGATGACATCCAGACAA
AATCCCAACAGATCCAGCAGATGGCTGATAAAATTCTGGAGCTGGAGGAGAAGCACCGGGAGGCCCAGAT
CTCAGCCCAGCACCTAGAGGTCCACCTGAAGCAGAAAGAGCAGCACTATGAGGAAAAAATTAAAGTGTTG
GACAATCAGATAAAGAAAGACCTGGCTGATAAGGAGAGCCTGGAGAATATGATGCAGAGACACGAGGAG
AGGCCCATGAGAAAGGCAAAATTCTCAGCGAGCAGAAGGCGATGATCAATGCTATGGATTCCAAGATCAG
ATCCCTGGAACAGAGGATTGTGGAGCTGTCAGAAGCCAATAAACTTGCTGCAAACAGCAGTCTTTTTACC
CAGAGGAACATGAAGGCCCAGGAAGAGATGATTTCAGAACTCAGGCAGCAGAAGTTTTACCTGGAGACGC
AGGCTGGGAAATTGGAGGCCCAGAACCGGAAGCTGGAGGAACAGCTGGAAAAAATCAGCCACCAAGACCA
CAGCGATAAGAATCGTCTGCTGGAGCTGGAGACACGGTTGAGGGAGGTCAGCCTCGAGCACGAGGAGCAG
AAACTGGAGCTAAAGCGCCAGCTCACAGAGCTGCAGCTCTCCCTGCAGGAGCGCGAGGCCCAGCTGACAG
CCCTGCAGGCTGCCCGGGCGGCCCTGGAGAGCCAGCTCCGCCAGGCCAAGACGGAGCTAGAAGAGACGAC
TGCAGAAGCAGAAGAGGAGATCCAGGCGCTCACGGCACATAGAGATGAAATCCAGCGCAAATTTGATGCC
CTTCGTAACAGCTGTACTGTAATCACAGACCTGGAGGAGCAGCTAAACCAGCTAACGGAGGACAACGCTG
AGCTCAACAACCAAAACTTCTACTTGTCCAAACAACTCGATGAGGCTTCCGGCGCCAACGATGAGATAGT
TCAGCTGCGAAGTGAGGTGGACCATCTCCGCCGCGAGATCTCGGAGAGAGAGATGCAACTCACCAGCCAG
AAGCAGACGATGGAGGCTCTGAAGACCACCTGCACGATGCTGGAAGAACAGGTCATGGACCTGGAGGCCC
TGAACGACGAGCTGCTGGAAAAGGAGCGGCAGTGGGAGGCGTGGAGGAGCGTCCTCGGTGACGAGAAGTC
CCAGTTTGAGTGCCGGGTCCGAGAGTTGCAGAGGATGCTGGACACTGAGAAGCAGAGCAGAGCAAGAGCC
GACCAGCGGATCACTGAGTCGCGCCAGGTGGTCGAGCTGGCCGTGAAGGAGCACAAGGCCGAGATTCTCG
CCCTGCAGCAGGCTCTCAAAGAGCAGAAGCTGAAAGCCGAGAGCCTCTCCGACAAGCTCAGTGACCTGGA
GAAGAAACACGCCATGCTTGAAATGAACGCCCGGAGTTTGCAGCAGAAACTGGAGACCGAGCGTGAGCTC
AAGCAGAGGCTCCTGGAGGAGCAAGCCAAACTACAGCAGCAGATGGACCTGCAGAAGAGTCACATTTTCC
GTCTGACTCAAGGGTTGCAAGAAGCTCTAGATCGGGCTGATCTGCTGAAGACAGAAAGGAGTGATCTGGA
ATATCAGTTGGAAAATATTCAGGTTCTCTATTCTCATGAAAAGGTGAAAATGGAGGGCACTATTTCTCAA
CAAACCAAACTTATTGATTTTCTGCAAGCCAAAATGGACCAACCTGCTAAAAAGAAAAAGGGTTTATTTA
GTCGACGGAAAGAGGACCCTGCTTTGCCCACACAGGTTCCTCTGCAGTACAATGAGCTGAAGGTGGCTCT
GGAGAAGGAGAAAGCTCGCTGTGCCGAGCTAGAGGAAGCCCTTCAGAAGACCCGCATCGAGCTCCGCTCC
GCCCGCGAGGAAGCTGCTCACCGGAAAGCCACCGACCATCCGCACCCATCTACACCAGCCACTGCGAGGC
AGCAGATTGCCATGTCTGCTATCGTGCGGTCGCCCGAGCACCAGCCCAGTGCCATGAGCCTGCTCGCCCC
GCCGTCCAGCCGTAGAAAGGAGTCTTCAACTCCAGAGGAGTTCAGCCGGCGTCTTAAGGAACGCATGCAC
CACAATATTCCTCACCGGTTTAACGTGGGACTGAACATGCGAGCCACAAAGTGCGCTGTGTGTCTGGATA
CTGTGCACTTTGGACGCCAGGCATCCAAATGTCTTGAATGTCAGGTGATGTGTCATCCCAAGTGCTCCAC
GTGCTTGCCGGCGACCTGTGGCCTGCCAGCCGAATATGCCACACACTTCACCGAGGCCTTCTGCCGCGAC
AAAATGAACTCCCCGGGCCTCCAGGCCAAGGAGCCCGGCAGCAGCTTGCACCTGGAAGGTTGGATGAAGG
TGCCCAGGAATAACAAACGAGGACAGCAAGGCTGGGACAGGAAGTACATTGTCCTGGAGGGATCCAAAGT
CCTCATTTATGACAACGAAGCCAGAGAAGCTGGACAGAGGCCGGTGGAAGAATTTGAGCTGTGCCTTCCC
GACGGGGATGTATCTATTCATGGCGCCGTTGGTGCTTCTGAACTCGCAAATACAGCCAAAGCAGATGTCC
CATACATCCTGAAGATGGAGTCCCACCCGCATACCACCTGCTGGCCCGGGAGAACCCTCTACTTGCTAGC
TCCCAGCTTCCCCGACAAGCAGCGCTGGGTCACCGCCTTAGAATCAGTTGTCGCAGGTGGGAGAGTTTCT

AGGGAAAAGGCAGAAGCCGATGCTAAATTGCTTGGAAACTCCCTGCTGAAACTGGAAGGTGATGACCGGC
TAGACATGAACTGCACACTGCCCTTCAGCGACCAGGTGGTGTTGGTAGGCACCGAGGAAGGGCTCTATGC
ACTGAATGTCTTGAAAAACTCCCTCACCCACGTCCCGGGAATCGGAGCAGTCTTCCAAATTTATATCATC
AAGGACCTGGAGAAGCTCCTCATGATAGCAGGAGAAGAGCGAGCCCTCTGTCTTGTTGACGTGAAGAAAG
TGAAACAGTCCCTGGCACAGTCTCACCTTCCTGCCCAGCCAGACATCTCGCCCAACGTTTTTGAAGCTGT
CAAGGGCTGCCACTTGTTTGCTGCTGGCAAGATTGAGAACGGGCTCTGCATCTGTGCAGCCATGCCCAGC
AAAGTCGTCGTTCTCCGCTACAACGAAAACCTCAGCAAGTACTGCATTCGGAAGGAGATAGAGACCTCAG
AGCCCTGCAGCTGTATCCACTTCACCAACTACAGCATCCTCATCGGAACCAATAAATTCTACGAAATTGA
CATGAAGCAGTACACGCTTGAGGAATTCCTGGATAAGAACGACCATTCCTTGGCGCCTGCTGTGTTTGCC
TCCTCTTCCCACAGTTTCCCTGTCTCGATAATGCAGGTGAACAGTGCCGGGCAGCGGGAGGAGTACCTGC
TCTGCTTCCACGAATTTGGGGTGTTTGTGGATTCTTACGGGAGACGTAGCCGCACAGACGATCTCAAGTG
GAGTCGCTTACCTTTGGCCTTCGCCTACAGAGAACCCTATCTGTTTGTGACCCACTTCAACTCACTAGAA
GTGATTGAGATCCAGGCACGCTCCGCTCTGGGGACCCCTGCTCGAGCGTATTTGGAAATCCCAAACCCAC
GCTACCTGGGCCCTGCAATTTCCTCGGGAGCGATTTACCTGGCGTCCTCATACCAGGATAAACTGAGGAT
CATTTGCTGCAAAGGAAACCTCGTGAAGGAGTCTGGAACTGACCACCACCGGGGTCCCTCCACCTCCCGC
AGCAGCCCCAACAAGCGAGGCCCGCCAACGTACAACGAGCACATCACCAAGCGTGTGGCCTCCAGCCCGG
CGCCACCCGAAGGCCCCAGCCACCCGCGAGAGCCAAGCACACCCCACCGCTACCGAGAGGGGCGGACCGA
GCTGCGCAGGGACAAGTCTCCTGGCCGCCCCCTGGAGCGGGAGAAGTCCCCAGGCCGCATGCTCAGCACG
CGGAGGGAGCGGTCCCCTGGGAGGCTGTTTGAAGACAGCAGCAGGGGCCGGCTTCCTGCGGGAGCTGTGA
GGACGCCCCTGTCCCAGGTCAACAAGGTCTGGGACCAGTCTTCAGTA
>Pteropus alecto XM_006908606.2
ATGTTGAAGTTCAAGTATGGGGCGAGGAATCTGCTGGATGCCGGTGCAGCTGAGCCCATTGGCAGCCGGG
CCTCCAGGCTGAATCTTTTCTTCCAGGGAAAACCACCCCTTATGACTCAACAACAGATGTCTCCTCTTTC
CCGAGAAGGGGTATTAGATGCCCTCTTTGTTCTCTTTGAAGAATGCAGTCAGCCTGCGCTGATGAAGATT
AAGCACGTGAGCAGCTTTGTCCGAAAGTATTCCGACACTGTAGCTGAGTTACAGGAGCTCCAGCCTTCAG
TAAAGGACTTTGAGGTCAGAAGTCTGGTAGGTTGTGGACACTTTGCAGAAGTGCATGTGGTAAGAGAGAG
AGCTACTGGGGACATCTATGCCATGAAAGTCATGAAGAAGAAGGCCTTATTGGCCCAGGAACAGGTTTCG
TTTTTTGAGGAAGAACGGAATATATTATCTCGGAGCACAAGTCCTTGGATCCCCCAATTACAGTATGCTT
TTCAGGACAAAAATAACCTTTATCTGGTTATGGAATATCAGCCTGGAGGGGACTTGCTGTCACTTTTGAA
TAGATATGAGGACCAATTAGATGAAAATATGATTCAGTTTTACCTAGCCGAACTGATTTTGGCCGTTCAC
AGCGTTCATCAGATGGGATACGTACATCGAGACATCAAGCCCGAGAACATCCTCATTGATCGAACAGGAC
ACATCAAACTAGTCGATTTTGGATCAGCTGCGAAAATGAACTCAAATAAGATGGTGAATGCCAGACTCCC
GATTGGGACCCCAGATTACATGGCCCCTGAAGTGTTGACCGTGATGAATGGGGATGGGAAAGGTGTCTAC
AGTCCTGACTGCGACTGGTGGTCAGTGGGAGTTATCGCGTATGAGATGGTTTATGGAAGGTCCCCATTCA
CTGAGGGAACCTCAGCCAGAACCTTTAACAACATCATGAATTTCCAGCGGTTTTTGAAGTTTCCAGATGA
CCCCAAAGTTAGCAGTGAATTGCTGGATCTGATTCAAAGTTTGTTGTGTGGCCAGAAAGAGAGACTGAAG
TTTGAAGGCCTTTGCTGCCATCCTTTCTTCTCAAAAATCGACTGGGATAACATTCGTAACTCTCCTCCCC
CCTTCGTTCCCACCCTCAAGTCTGATGATGACACCTCCAATTTTGATGAACCAGAGAAGAATTCGTGGGT
TTCATCCTCTCCGTGCCAGCTGAACCCCTCAGGTTTCTCCGGCGAAGAACTGCCGTTTGTGGGGTTTTCG
TATAGCAAGGCACTGGGGATTCTTGGTAGATCTGAGTCTGTTGTGTCGGGCCTGGACTCCCCTGCCAAGA
CTAGCTCCATGGAAAAGAAACTTCTCATCAAAAGCAAAGAGCTGCAAGACTCCCAGGACAAGTGTCACAA
GATGGAACAGGAAATGACCCGGTTACATCGGAGAGTGTCAGAGGTGGAGGCTGTGCTTAGTCAGAAGGAG
GTGGAGCTGAAGGCCTCTGAGACTCAGAGATCCCTCCTGGAGCAGGACCTTGCTACCTACATCACAGAAT
GCAGTAGCTTAAAGCGAAGTTTGGAGCAAGCACGGATGGAGGTGTCCCAGGAGGATGACAAAGCACTGCA
GCTTCTCCACGATATCAGAGAGCAGAGCCGGAAGCTCCAAGAAATCAAAGAGCAGGAGTACCAGGCTCAA
GTGGAAGAAATGAGGTTGATGATGAATCAGTTGGAAGAGGACCTGGTTTCAGCAAGAAGACGGAGTGATC
TCTACGAATCCGAGCTGAGAGAGTCTCGGCTCGCCGCTGAAGAGTTCAAGCGGAAAGCCACAGAGTGTCA

GCATAAACTGATGAAGGCTAAGGATCAAGGGAAGCCCGAAGTGGGAGAATATTCCAAGCTGGAGAAGATT
AATGCTGAGCAGCAGCTCAAAATTCAGGAGCTCCAAGAGAAGCTGGAAAAGGCTGTAAAAGCCAGCGCAG
AGGCCACCGAGCTGCTACAGAATATCCGCCAGGCAAAGGAGCGAGCCGAACGTGAGCTGGAGAAGCTGCA
GAACCGCGAGGATTCCTCTGAGGGCATAAAAAAGAAGCTAGTTGAGGCCGAGGAACGCCGCCATTCTCTG
GAGAACAAGGTAAAGAGGCTAGAGACCATGGAGCGTAGAGAAAACAGACTGAAGGATGACATCCAGACAA
AATCCCAACAGATCCAGCAGATGGCTGATAAAATTCTGGAGCTGGAGGAGAAGCACCGAGAGGCCCAGCT
CTCAGCCCAGCACCTAGAGGTGCACCTGAAACAGAAAGAGCAGCACTACGAGGAAAAAATTAAAGTCTTG
GACAATCAGATAAAGAAGGACCTGGCCGATAAAGAGACTCTGGAGAATATGATGCAGCGACACGAAGAGG
AGGCCCACGAGAAAGGCAAAATTCTCAGCGAGCAGAAGGCGATGATCAATGCTATGGATTCCAAGATCAG
ATCCCTGGAACAGAGGATTGTGGAATTGTCAGAAGCCAATAAACTTGCAGCCAACAGCAGCCTTTTTACC
CAGAGGAACATGAAGGCTCAGGAAGAGATGATTTCAGAACTCAGGCAACAGAAGTTTTACCTGGAGACAC
AGGCTGGGAAATTGGAGGCCCAGAACCGGAAGCTGGAGGAGCAGCTGGAAAAAATCAGCCACCAAGACCA
CAGCGACAAGAATCGTCTACTGGAGCTGGAGACAAGGCTGAGGGAGGTCAGTCTAGAGCACGAGGAGCAG
AAATTGGAGCTAAAGCGCCAGCTCACAGAGCTGCAGCTCTCCCTGCAGGAGCGTGAGTCCCAGCTGACAG
CCCTGCAGGCTGCCCGGGCAGCCCTGGAGAGCCAGCTTCGCCAGGCCAAGACCGAGCTGGAAGAGACGAC
AGCAGAGGCAGAAGAAGAGATCCAGGCACTCACGGCACATAGAGATGAAATCCAGCGCAAATTTGATGCC
CTTCGTAACAGCTGTACTGTAATCACAGACCTGGAGGAGCAGCTAAACCAGCTGACCGAGGACAACGCTG
AGCTCAACAACCAAAATTTCTACTTGTCCAAACAACTCGATGAGGCTTCTGGCGCCAACGATGAGATAGT
GCAGCTGCGAAGTGAAGTGGACCATCTCCGCCGTGAGATCACGGAGAGAGAGATGCAGCTCACCAGCCAG
AAGCAAACGATGGAGGCGCTGAAGACCACCTGCACGATGCTGGAAGAACAGGTCATGGACTTGGAGGCCC
TGAACGACGAGCTGCTGGAAAAGGAGCGGCAGTGGGAGGCGTGGAGGAGCGTCCTCGGCGACGAGAAGTC
CCAGTTCGAGTGTCGGGTTCGAGAGTTACAGAGGATGCTGGACACCGAGAAACAGAGCAGGGCAAGAGCC
GATCAGCGGATCACCGAGTCGCGCCAGGTGGTTGAGCTGGCAGTGAAGGAACACAAGGCTGAGATTCTCG
CTCTGCAGCAGGCTCTCAAAGAACAGAAGCTGAAAGCCGAGAGCCTCTCTGACAAGCTCAACGACCTGGA
GAAGAAACATGCCATGCTTGAAATGAACGCCCGCAGTTTACAACAGAAACTGGAGACCGAACGAGAGCTC
AAACAAAGACTTCTGGAAGAGCAAGCCAAATTACAGCAGCAGATGGACCTGCAGAAGAATCATATTTTCC
GTCTGACACAAGGGCTACAGGAAGCTCTAGATCGGGCTGACCTGCTGAAGACAGAAAGGAGCGATCTGGA
ATATCAGCTAGAAAACATTCAGGTTCTCTATTCTCATGAAAAGGTGAAAATGGAAGGCACTATTTCTCAA
CAAACCAAACTCATTGATTTTCTGCAAGCCAAAATGGACCAACCTGCTAAAAAGAAAAAGGGTTTATTTA
GTCGACGGAAAGAGGACCCTGCTTTGCCCACACAGGTTCCTCTGCAGTACAATGAGCTGAAGGTGGCTCT
GGAGAAGGAGAAAGCTCGCTGTGCCGAGCTAGAGGAAGCCCTTCAGAAGACCCGCATCGAGCTCCGTTCC
GCCCGGGAGGAAGCTGCTCACCGGAAGGCCACAGACCACCCCCACCCGTCCACACCAGCCACTGCGAGGC
AGCAGATCGCCATGTCTGCCATCGTGCGGTCGCCTGAGCACCAGCCCAGTGCCATGAGCCTGCTCGCCCC
GCCATCCAGCCGCAGAAAGGAGTCTTCAACTCCAGAGGAATTTAGTCGGCGTCTTAAGGAGCGCATGCAC
CACAATATTCCCCACCGATTTAACGTAGGACTGAACATGCGAGCCACGAAGTGTGCCGTGTGTCTGGATA
CTGTGCACTTTGGCCGCCAGGCATCCAAATGTCTAGAATGTCAGGTGATGTGTCATCCCAAGTGCTCCAC
GTGCTTGCCAGCTACCTGTGGCCTGCCAGCTGAATATGCCACGCACTTCACTGAGGCCTTCTGCCGTGAC
AAAATGAACTCCCCGGGTCTCCAGACCAAGGAGCCCAGCAGCAGCTTGCATCTGGAAGGGTGGATGAAGG
TGCCCAGGAATAACAAACGGGGACAGCAAGGCTGGGACAGGAAGTACATCGTCCTGGAGGGGTCAAAAGT
CCTCATTTATGACAACGAAGCCAGAGAAGCTGGACAGAGGCCGGTGGAAGAATTTGAGCTGTGCCTTCCC
GACGGGGACGTATCTATTCATGGCGCCGTTGGTGCTTCTGAACTCGCAAACACAGCCAAAGCAGATGTCC
CATACATACTGAAGATGGAATCTCACCCGCACACCACCTGCTGGCCCGGGAGAACCCTCTACTTGCTAGC
TCCCAGCTTCCCCGACAAACAGCGCTGGGTCACCGCCTTAGAATCAGTTGTCGCAGGTGGGAGAGTGTCT
AGGGAAAAAGCAGAAGCCGATGCTAAATTGCTTGGAAACTCCCTGCTGAAACTGGAAGGCGATGACCGTC
TAGACATGAACTGCACACTGCCCTTCAGTGACCAGGTGGTGTTGGTGGGCACCGAGGAAGGGCTCTATGC
ACTGAATGTCTTGAAAAACTCCCTCACCCACGTCCCAGGAATTGGAGCAGTCTTCCAAATTTATATCATC
AAGGACCTGGAGAAGCTACTCATGATAGCAGGAGAAGAGCGGGCCTTGTGTCTTGTTGACGTGAAGAAAG

TGAAACAGTCCCTGGCACAGTCTCACCTTCCTGCTCAGCCAGACATCTCGCCCAACATTTTTGAAGCTGT
CAAGGGCTGCCACTTGTTTGCTGCTGGCAAGATTGAGAACGGGCTCTGCATCTGTGCAGCCATGCCCAAC
AAAGTCGTCATCCTCCGCTACAATGAAAACCTCAGCAAATATTGCATTCGGAAAGAGATAGAGACCTCAG
AGCCCTGCAGCTGTATCCACTTCACCAACTACAGTATCCTCATCGGAACCAATAAATTCTACGAAATTGA
CATGAAGCAGTACACGCTTGAGGAATTCCTGGACAAGAATGACCACTCTTTGGCGCCTGCTGTGTTTGCC
TCCTCTTCCAACAGTTTCCCTGTATCGATCGTGCAGGTGAATGGCACAGGACAGCGGGAGGAGTACCTGC
TCTGCTTCCACGAATTTGGGGTGTTCGTGGATTCTTACGGAAGACGTAGCCGCACAGACGATCTCAAGTG
GAGTCGCTTACCTTTGGCCTTCGCCTACAGGGAACCCTATCTGTTTGTGACCCACTTCAACTCACTCGAA
GTGATTGAGATCCAGGCACGCTCCTCTCTGGGGACCCCTGCCCGAGCGTATTTGGAAATCCCAAACCCAC
GCTACCTGGGCCCTGCAATTTCCTCGGGAGCGATTTACCTGGCGTCCTCGTACCAGGATAAATTAAGGGT
CATTTGCTGCAAAGGCAACCTCGTGAAGGAGTCCGGCACTGACCACCACCGGGGTCCCTCCACCTCCCGC
AGCAGCCCCAACAAGCGCGGCCCGCCAACGTACAACGAGCACATCACCAAGCGCGTGGCCTCCAGCCCGG
CGCCGCCCGAAGGCCCCAGCCACCCGAGAGAGCCAAGCACACCCCACCGCTACCGAGAGGGGCGGACGGA
GCTGCGCAGGGACAAGTCGCCCGGCCGCCCCCTGGAGCGGGAGAAGTCCCCAGGCCGGATGCTTAGCACT
CGGAGGGAGCGGTCCCCCGGGAGGCTGTTCGAAGACAGCAGCAGGGGCCGGCTGCCCGTGGGAGCTGTGA
GGACACCCCTGTCCCAGGTCAACAAGGTCTGGGACCAGTCTTCAGTA
>Canis lupus familiaris XM_014107963.2
ATGTTGAAGTTCAAGTATGGAGCACGGAATCAAATGGACGCTGGTGCTGCTGAACCCATTGCCAGCCGGG
CCTCCAGGCTGAATCATTTCTTCCAGGGGAAACCACCCTTTCTGACTCAACAGCAGATGTCTCCTCTTTC
CCGAGAAGGGATATTAGATGCCCTCTTCGTTCTCTTTGGAGAATGCAGTCAGCCTGCTCTGATGAAGATC
AAGCACGTGAGCAACTTTGTCCGGAAGTATTCCGACACCATAGCTGAATTACAGGAGCTCCAGCCTTCAG
CAAAGGACTTTGAAGTCCGAAGTCTTGTGGGTTGTGGTCACTTTGCTGAAGTGCAGGTGGTAAGAGAGCG
AGCCACCGGAGATATCTATGCCATGAAAGTCATGAAGAAGAAGACCTTGTTGGCCCAAGAGCAGGTTTCA
TTTTTTGAGGAAGAACGGAACATATTATCTCAGAGCATGAGCCCTTGGATCCCCCAGCTACAGTATGCCT
TTCAGGACAAAAATAACCTTTATCTGGTCATGGAGTATCAGCCTGGAGGGGACTTGCTGTCACTTTTGAA
TAGATATGAGGACCAATTAGATGAAAATATGATTCAGTTTTACCTAGCCGAACTGATTTTGGCTGTTCAC
AGCATTCATCAGATGGGATATGTACATCGAGACATCAAGCCTGAGAACATTCTCATTGACCGAACAGGAC
ACATCAAGCTTGTGGATTTTGGATCAGCGGCTAAAATGAACTCAAATAAGATGGTGAGCGCCAAACTCCC
GATTGGGACTCCAGATTACATGGCCCCTGAAGTGTTGACCGTGATGAATGGGGACGGGAAAGGCATCTAT
AGTCTAGACTGTGATTGGTGGTCAGTGGGAGTGATTGCATATGAAATGGTTTATGGAAGGTCCCCATTCA
CAGAGGGAACTTCGGCCAGAACCTTCAGTAACATCATGAATTTCCAGCGGTTTTTGAAGTTTCCAGATGA
CCCCAAAGTTAGTGGTGAATTACTTGATCTGATTCAGAGTTTGCTGTGTGGCCAGAGAGAGAGACTGAAG
TTTGAAGGCCTGTGCTGCCACCCTTTCTTCTCTAAAATTGACTGGAATAACATTCGTAACTCTCCTCCCC
CCTTCGTTCCCACCCTCAAGTCTGATGATGACACCTCCAATTTTGATGAACCAGAGAAGAATTCGTGGGT
TTCATCCTCTCCGCGCCAGCTGAACCTCTCAGGTTTCTCGGGCGAAGAACTGCCGTTTGTGGGGTTTTCG
TATAGCAAGGCACTGGGGATTCTTGGTAGATCTGAGTCTATCGTGTCAAGTCTGGACTCCCCTGCCAAGA
CTAGCTCCATGGAAAAGAAACTTCTCATCAAAAGCAAAGAGCTGCAAGACTCCCAGGACAAGTGTCACAA
GATGGAGCAGGAAATGACCCGGTTACATCGGAGAGTATCAGAGGTGGAGGCTGTGCTTAGTCAGAAGGAG
GTGGAGCTGAAGGCCTCTGAGACTCAGAGATCCCTCCTGGAGCAGGACCTTGCTACCTACATCACAGAAT
GCAGTAGCTTAAAGCGAAGTTTGGAGCAAGCACGGATGGAGGTGTCCCAGGAGGATGACAAAGCACTGCA
GCTTCTCCATGATATCAGAGAGCAGAGCCGGAAGCTCCAGGAAATCAAAGAGCAGGAGTACCAGGCTCAA
GTGGAAGAAATGAGGTTGATGATGAATCAGTTGGAAGAGGACCTTGTTTCAGCAAGGAGACGGAGTGATC
TCTATGAATCGGAGTTGAGAGAGTCTCGGCTCGCTGCTGAAGAATTCAAGCGGAAAGCAACAGAATGTCA
GCATAAACTAATGAAGGCTAAGGATCAAGGGAAGCCTGAAGTGGGAGAATATTCCAAACTGGAGAAGATC
AATGCTGAGCAACAGCTCAAAATCCAGGAGCTCCAGGAGAAGCTGGAAAAGGCTGTAAAAGCCAGTACGG
AGGCCACTGAACTGCTGCAGAATATCCGCCAGGCAAAGGAGAGAGCCGAGCGTGAGCTGGAGAAGCTGCA
GAACCGGGAGGACTCCTCTGAAGGCATAAAAAAGAAGCTGGTGGAGGCTGAGGAACGCCGCCATTCTCTG

GAGAACAAGGTAAAGAGGCTAGAGACCATGGAGCGTAGAGAAAACAGACTGAAGGATGACATCCAGACAA
AATCCCAACAGATCCAGCAGATGGCTGATAAAATTCTGGAGCTGGAAGAGAAGCACCGCGAGGCCCAGGT
CTCAGCCCAGCACCTAGAGGTGCACCTGAAGCAGAAAGAACAGCACTACGAGGAAAAAATTAAAGTGTTG
GACAGCCAGATAAAGAAAGACCTGGCCGATAAAGAGACTCTGGAGAATCTGATGCAGAGACACGAAGAGG
AGGCCCATGAGAAGGGCAAAATTCTCAGCGAGCAGAAGGCGATGATCAATGCTATGGATTCCAAGATCAG
ATCCCTGGAACAGAGGATCGTGGAACTCTCAGAAGCCAACAAACTGGCGGCAAACAGCAGTCTTTTTACT
CAGAGGAACATGAAGGCCCAGGAAGAGATGATTTCAGAACTCAGGCAACAGAAGTTCTACCTGGAGACAC
AGGCTGGGAAATTGGAGGCTCAGAACCGAAAGCTGGAGGAGCAGCTGGAGAAAATAAGCCACCAAGACCA
CAGTGACAAGAATCGGCTGCTGGAGCTGGAGACGAGGTTGAGGGAGGTCAGTCTAGAGCACGAGGAGCAG
AAACTAGAGCTGAAGCGCCAGCTCACAGAGCTACAGCTCTCCCTGCAAGAGCGTGAGTCCCAGCTGACAG
CCCTGCAGGCTGCCCGGGCAGCCCTGGAGAGCCAGCTTCGCCAGGCCAAGACGGAACTAGAAGAGACGAC
GGCAGAGGCAGAAGAAGAGATCCAGGCGCTCACGGCACATAGAGATGAAATCCAGCGCAAATTTGATGCC
CTTCGTAACAGCTGTACTGTAATCACAGACCTGGAGGAGCAGCTAAACCAGCTGACTGAGGACAACGCTG
AGCTCAACAACCAAAATTTCTATTTGTCCAAACAACTCGACGAGGCTTCCGGTGCCAACGATGAGATAGT
ACAGCTACGGAGTGAAGTGGACCATCTCCGCCGTGAGATTACAGAGAGGGAGATGCAGCTCACCAGCCAG
AAGCAAACGATGGAGGCGCTGAAGACTACTTGCACGATGCTGGAAGAACAGGTCATGGACCTGGAGGCCC
TGAATGATGAGCTGCTGGAAAAAGAGCGGCAGTGGGAGGCATGGAGGAGTGTCCTTGGTGACGAGAAGTC
CCAATTTGAGTGTCGGGTTCGAGAGTTACAGAGAATGCTGGACACTGAGAAGCAGAGCAGAGCGAGGGCC
GACCAGCGGATCACAGAATCACGCCAGGTGGTAGAGCTGGCGGTGAAGGAGCACAAGGCTGAGATTCTTG
CTCTGCAGCAGGCCCTCAAAGAACAGAAGCTGAAAGCTGAGAGCCTCTCTGATAAGCTCAATGACCTGGA
GAAGAAACATGCCATGCTTGAAATGAACGCCCGAAGTTTACAGCAGAAACTGGAGACTGAACGAGAGCTC
AAACAGAGGCTTCTGGAAGAGCAAGCCAAATTACAGCAGCAGATGGACCTGCAGAAGAATCACATCTTCC
GTTTGACTCAAGGGCTGCAAGAAGCTCTGGACCGGGCTGATCTGCTGAAGACAGAAAGGAGCGATCTAGA
ATACCAGCTAGAAAACATTCAGGTTCTCTACTCTCATGAAAAGGTGAAAATGGAAGGTACTATTTCTCAA
CAAACCAAACTCATTGACTTTCTGCAAGCCAAAATGGACCAGCCCGCTAAGAAGAAAAAGGGTTTATTTA
GTCGACGGAAAGAGGACCCTGCTTTGCCCACACAGGTTCCTCTGCAGTACAATGAGCTGAAGGTGGCTCT
GGAGAAGGAGAAAGCTCGCTGTGCAGAGCTAGAGGAAGCCCTCCAGAAGACCCGCATCGAGCTCCGGTCT
GCCCGGGAGGAAGCTGCCCACCGGAAGGCCACAGACCATCCGCACCCATCTACGCCAGCCACCGCGAGGC
AGCAGATTGCCATGTCTGCCATCGTGCGGTCACCTGAGCACCAGCCCAGTGCCATGAGCCTGCTTGCCCC
ACCATCCAGCCGCAGAAAGGAGTCATCAACTCCAGAGGAATTCAGTCGGCGTCTTAAGGAGCGCATGCAC
CACAATATTCCTCACCGATTTAATGTAGGATTGAACATGCGAGCCACAAAGTGTGCTGTGTGTCTGGATA
CTGTGCACTTTGGGCGCCAGGCATCCAAATGTCTTGAATGTCAGGTGATGTGTCATCCCAAGTGCTCCAC
GTGCTTGCCAGCCACCTGTGGCCTGCCAGCCGAGTATGCCACACACTTCACTGAGGCCTTCTGTCGTGAC
AAAATGAACTCCCCGGGGCTTCAGACCAAGGAGCCCAGCAGCGGCTTACACCTGGAAGGGTGGATGAAAG
TGCCCAGGAATAACAAACGAGGACAGCAAGGTTGGGACAGGAAGTACATTGTCCTGGAGGGATCCAAAGT
CCTCATTTATGACAATGAAGCCAGAGAAGCTGGACAGAGGCCGGTGGAAGAATTTGAGCTGTGCCTTCCC
GACGGGGATGTATCTATTCATGGCGCCGTTGGTGCTTCTGAGCTTGCAAATACAGCCAAAGCAGATGTCC
CTTACGTGCTGAAGATGGAGTCTCACCCACACACCACCTGCTGGCCTGGGAGAACGCTCTATTTGCTGGC
TCCCAGCTTCCCTGACAAACAGCGCTGGGTCACCGCCTTAGAGTCCGTTGTCGCAGGTGGGAGAGTTTCT
AGGGAAAAAGCAGAAGCCGATGCTAAATTGCTTGGAAACTCCCTGCTGAAACTGGAAGGTGATGACCGTC
TAGACATGAACTGCACACTGCCTTTCAGCGACCAGGTGGTGCTGGTGGGCACTGAGGAAGGGCTCTACGC
GCTGAATGTCCTGAAAAACTCTCTCACCCATGTCCCAGGAATTGGAGCGGTCTTCCAAATTTATATCATC
AAGGACCTGGAGAAGCTCCTCATGATAGCAGGAGAAGAGCGGGCTCTGTGTCTCATTGACGTGAAGAAAG
TTAAGCAGTCCCTAGCACAGGCTCACCTTCCTGCCCAGCCTGACATCTCACCCAACATTTTCGAAGCCGT
GAAGGGCTGCCACTTGTTTGCTGCTGGCAAGATTGAGAATGGGCTTTGCATTTGTGCAGCCATGCCCAGC
AAAGTTGTCATTCTCCGCTACAACGAAAACCTCAGCAAGTACTGCATTCGGAAAGAGATAGAGACTTCAG
AGCCCTGCAGCTGCATTCACTTCACCAATTACAGTATCCTCATTGGAACCAATAAATTCTACGAAATTGA

CATGAAGCAGTACACGCTCGAGGAATTCCTGGATAAGAATGACCATTCCTTGGCGCCTGCTGTGTTTGCC
TCTTCTTCCAACAGTTTCCCTGTCTCGATCGTGCAGGTGAACAGTGCAGGGCAGCGGGAGGAATACCTGC
TCTGCTTCCACGAATTTGGGGTGTTCGTGGACTCTTACGGAAGACGTAGCCGCACAGACGATCTCAAGTG
GAGTCGCTTACCTTTGGCCTTTGCCTACAGAGAACCCTATCTCTTTGTGACCCACTTCAACTCACTTGAA
GTAATTGAGATCCAGGCACGCTCCTCTCTGGGGACTCCTGCCCGAGCATATTTGGAAATTCCAAACCCAC
GCTACTTGGGCCCTGCAATATCCTCAGGAGCAATTTACCTGGCGTCCTCATACCAGGATAAATTAAGGGT
CATTTGCTGCAAAGGAAACCTCGTGAAGGAGTCCGGCACTGACCACCACCGGGGCCCCTCCACCTCCCGC
AGCCCCAACAAGCGAGGCCCGCCAACGTACAACGAGCACATCACCAAGCGTGTGGCCTCCAGCCCAGCGC
CACCCGAAGGCCCTGGCCACCCTCGAGAGCCAAGCACACCCCACCGCTACCGAGAGGGGCGGACAGAGCT
GCGCAGGGACAAGTCTCCGGGCCGCCCCCTGGAGCGGGAGAAGTCCCCAGGGCGGATGCTCAGCACGCGG
AGGGAGCGCTCCCCTGGGAGGCTGTTTGAAGACAGCAGCCGGGGCCGCCTGCCTGTGGGAGCCGTCAGGA
CCCCACTGTCCCAGGTCAACAAGGTCTGGGACCAGTCTTCAGTG
>Ovis aries XM_004017373.3
ATGTTGAAGTTCAAGTATGGAGCACGGAATCTGCTGGACGCTGGTGCTATTGAATCCATTGCCAGCCGGG
CCTCCAGGCTGAATCTTTTCTTCCAGGGGAAACCACCCTCCATGACTCAACAGCAGATGTCTCTTCTTTC
CCGAGAAGCGATGTTAGATGCCCTCTTTGTTCTCTTTAAAGAATGCAGTCAGCCGGCTCTGATGAAGATT
AAGCACGTGAGCAACTTTGTCCAGAAGTATTCTGATATTATAGCTGAGTTGCAAGAGCTCCAGCCTTCAG
TGAAGGACTTTGAAGTGAGAAGTGTCATAGGCTGCGGTCACTTTGCCGAAGTACAAGTGGTCAGAGAGAA
AGCGACTGGGGATGTCTATGCCATGAAAGTCATGAAGAAGAAGGCCTTGTTGGCCCAGGAGCAGGTCTCA
TATTTTGAGGAAGAACAGAACATATTATCTCGGAGTACAAGTCCTTGGATCCCCCAGTTGCAGTACGCCT
TTCAGGACAAAAATAACCTTTATCTGGTCATGGAATATCAGCCTGGAGGGGATTTGCTGTCACTTTTGAA
TAGATATGAGGACCAATTGGATGAAAATATGATTCAGTTTTACCTAGCTGAACTGATTTTGGCTGTCCAC
AGTGTTCATCAGATGGGATATGTACATCGAGACATCAAGCCTGAGAACATCCTCATTGACCGAACGGGAC
ACATTAAGCTGGTGGATTTTGGATCAGCTGCTAAAATGAACTCAAATAAGATGGTAAATGCCAAACTCCC
GGTTGGGACTCCAGATTACATGGCCCCTGAAGTGTTGACTGTGATGAATGGGGATGGAAAAGGTGCCTAT
AGCCTAGACTGTGATTGGTGGTCAGTGGGAGTTATTGCTTATGAGATGGTTTATGGAAGATGCCCATTCA
CTGAAGGAACCTCAGCCAAAACCTTCAATAACATCATGAATTTCCAGCGGTTCTTGAAGTTTCCAGATGA
CCCCAAAGTTAGCAGTGAATTGCTTGATCTAATCCAGAGTTTGTTGTGTGGCCAGAAAGAGAGACTGAAG
TTTGAGGGCCTTTGCTGTCATCCTTTCTTCTCCAAAATCGACTGGAGTAACATTCGTAACTCTCCTCCCC
CCTTCGTTCCCACCCTCAAGTCTGATGATGACACCTCCAATTTTGATGAACCAGAGAAGAATTCGTGGGT
TTCATCCTCTCCGTGCCAGCTGAACCCCTCAGGTTTCTCAGGCGAAGAACTGCCATTTGTGGGGTTTTCG
TATAGCAAGGCACTGGGAATTCTTGGTAGATCTGAGTCTGTTGTGTCAAATCTGGACTCCCCTGCCAAGA
CTAGCTCCATGGAAAAGAAACTTCTTATCAAAAGCAAAGAGCTGCAAGACTCCCAGGACAAGTGTCACAA
GATGGAGCAGGAAATGACCCGGTTACATCGGAGAGTGTCAGAGGTGGAGGCTGTGCTTAGTCAGAAGGAG
GTGGAGCTGAAGGCCTCTGAGACTCAGAGATCCCTCCTGGAGCAGGACCTTGCTACCTACATCACAGAAT
GCAGTAGCTTAAAGCGAAGTCTGGAGCAAGCACGGATGGAGGTGTCCCAAGAGGACGACAAAGCACTGCA
GCTCCTCCATGATATCAGAGAGCAGAGCCGGAAGCTCCAAGAAATCAAAGAGCAGGAGTACCAGGCTCAA
GTGGAAGAAATGAGGTTGATGATGAGTCAATTGGAAGAGGACCTGGTTTCGGCGAGAAGGCGGAGTGACC
TCTATGAATCGGAGCTCAGAGAGTCTCGGCTGGCTGCTGAAGAGTTTAAGCGGAAAGCAACAGAATGTCA
GCATAAACTGATGAAGGCTAAAGATCAAGGGAAGCCTGAAGTGGGAGAATATGCCAAACTGGAGAAGATC
AATGCTGAGCAGCAGCTCAAAATTCAGGAGCTCCAAGAGAAGCTAGAAAAGGCTGTGAAAGCGAGCACAG
AGGCCACCGAGCTGCTACAGAACATCCGTCAGGCGAAGGAGCGGGCTGAGCGTGAGCTGGAGAAGCTGCA
CAACCGTGAGGACTCCTCCGAAGGCATCAAGAAGAAGCTGGTGGAGGCCGAGGAACGCCGACACTCTCTG
GAGAACAAGGTAAAGAGGCTAGAGACCATGGAGCGTAGAGAAAACAGACTGAAGGATGACATCCAGACAA
AGTCCCAACAGATCCAGCAGATGGCTGATAAAATTCTGGAGCTGGAGGAGAAGCACAGAGAAGCCCAGGT
CTCAGCCCAGCATCTGGAGGTGCACCTGAAACAGAAAGAACAGCACTATGAGGAAAAAATTAAAGTGTTG
GACAATCAGATAAAGAAAGACCTAGCAGACAAGGAGACACTGGAGAACCTGATGCAAAGACACGAAGAGG

AGGCCCATGAAAAAGGCAAAATTCTCAGCGAGCAGAAGGCGATGATCAATGCCATGGATTCCAAGATCAG
ATCCCTGGAACAGAGGATAGTTGAATTGTCGGAAGCCAATAAACTTGCGGCCAACAGCAGTCTCTTTACC
CAGAGGAACATGAAGGCCCAGGAAGAGATGATTTCAGAACTCAGGCAACAGAAGTTTTACCTGGAGACGC
AAGCTGGGAAATTGGAGGCCCAGAACCGGAAGCTGGAGGAGCAGCTGGAAAAAATAAGCCACCAAGACCA
CAGTGACAAGAACCGTCTGCTGGAGCTGGAGACCAGGCTGAGGGAGGTCAGTCTAGAGCATGAAGAACAG
AAACTGGAGCTCAAACGCCAGCTCACAGAGCTGCAGCTCTCCCTGCAGGAGCGTGAATCCCAGCTGACAG
CCCTGCAGGCAGCACGGGCGGCCCTGGAGAGCCAGCTCCGCCAGGCCAAGACGGAGCTGGAAGAGACGAC
AGCAGAAGCAGAAGAAGAGATCCAGGCGCTTACGGCACATAGAGATGAAATCCAGCGCAAATTTGATGCC
CTTCGTAACAGCTGTACTGTGATCACAGACTTGGAGGAGCAGCTAAACCAGCTGACAGAGGACAACGCTG
AGCTCAATAACCAAAACTTCTACTTGTCTAAACAACTTGATGAGGCCTCTGGTGCCAACGACGAGATTGT
GCAGCTGCGAAGTGAAGTAGACCATCTCCGCCGCGAGATCACTGAGAGGGAGATGCAGCTCACTAGCCAG
AAGCAAACGATGGAGGCGCTGAAGACCACGTGCACAATGCTGGAGGAGCAGGTCATGGACCTGGAGGCCC
TGAACGACGAGCTTCTGGAGAAAGAGCGGCAGTGGGAGGCCTGGAGGAGCGTCCTTGGTGACGAGAAGTC
CCAATTTGAGTGTCGGGTTCGAGAGTTGCAGAGAATGCTGGACACCGAGAAGCAGAGCAGGGCAAGGGCT
GACCAGCGGATCACTGAGTCGCGCCAGGTGGTCGAGCTGGCAGTAAAGGAGCACAAGGCCGAGATCCTGG
CCTTGCAGCAGGCTCTCAAGGAGCAGAAGCTGAAGGCCGAGAGCCTCTCTGACAAGCTCAATGATCTGGA
GAAGAAGCATGCCATGCTTGAAATGAACGCCCGAAGTTTACAACAGAAGCTGGAGACGGAACGAGAGCTC
AAACAAAGGCTTCTGGAAGAGCAAGCCAAGTTACAGCAGCAGATGGACCTGCAGAAGAATCATATTTTCC
GTCTGACTCAAGGGCTGCAAGAAGCTCTGGATCGGGCTGATCTGCTGAAGACAGAAAGGAGTGACCTGGA
ATATCAGCTGGAAAACATTCAGGTTCTCTATTCTCATGAGAAGGTGAAGATGGAAGGCACTATTTCCCAG
CAAACCAAACTCATCGATTTTCTGCAAGCCAAAATGGACCAGCCCGCCAAAAAGAAAAAGGGTTTATTTA
GTCGACGGAAAGAGGACCCTGCTTTGCCCACACAGGTTCCTCTGCAGTACAATGAGCTGAAGGTGGCCCT
GGAGAAGGAGAAAGCTCGCTGCGCGGAGCTCGAGGAAGCCCTGCAGAAGACCCGCATCGAGCTCCGCTCC
GCCCGGGAGGAAGCTGCCCACCGGAAAGCCGCAGACCACCCACACCCATCTACGCCGGCTACCGCCAGGC
AGCAGATTGCCATGTCCGCCATTGTGCGGTCACCCGAGCACCAGCCCAGCGCCCTGAGCCTGCTTGCCCC
GCCGTCCAGCCGCAGGAAGGAGTCTTCCACTCCAGAGGAATTCAGCCGGCGTCTTAAGGAGCGCATGCAC
CACAACATCCCTCACCGGTTCAACGTGGGACTGAACATGCGAGCCACGAAGTGCGCCGTGTGTCTGGATA
CCGTGCACTTCGGACGTCAGGCATCCAAGTGTCTCGAATGTCAGGTCATGTGCCATCCCAAGTGCTCCAC
GTGCTTGCCAGCCACCTGTGGCCTGCCAGCCGAATATGCCACACACTTCACTGAGGCCTTCTGCCGCGAC
AAGATGAACTCCCCGGGCCTGCAGTCCAAGGAACCCAGCAGCAGTTTGCACCTGGAAGGGTGGATGAAGG
TGCCCAGGAATAACAAGCGAGGACAGCAAGGCTGGGACAGGAAGTACATCGTCCTGGAGGGCTCCAAAGT
CCTCATTTACGACAACGAAGCCAGAGAAGCTGGACAGAGGCCGGTGGAAGAATTTGAGCTGTGCCTTCCC
GATGGGGACGTGTCTATTCATGGCGCCGTTGGTGCTTCTGAACTTGCAAACACAGCCAAAGCAGATGTCC
CCTATGTCCTGAAGATGGAGTCTCACCCCCATACCACCTGCTGGCCTGGGAGGACCCTCTACTTGCTGGC
TCCCAGCTTTCCTGACAAGCAGCGCTGGGTCACCGCCTTAGAGTCAGTTGTGGCAGGTGGGAGAGTGTCT
AGGGAAAAGGCGGAAGCCGACGCTAAATTGCTCGGAAACTCCCTGCTGAAACTGGAAGGTGATGACCGTC
TGGACATGAACTGTACACTGCCCTTCAGCGACCAGGTGGTGCTGGTGGGCACCGAGGAAGGGCTGTATGC
GCTGAATGTCTTGAAGAACTCCCTCACCCACGTCCCAGGAATCGGAGCAGTCTTCCAGATTTACATCATC
AAGGACCTGGAGAAGCTCCTCATGATCGCAGGAGAGGAGCGGGCCCTGTGTCTCGTGGATGTGAAGAAGG
TGAAGCAGTCCCTCGCGCAGTCTCACCTCCCCGCCCAACCGGACATCTCGCCCAACATCTTTGAAGCGGT
CAAGGGCTGCCACTTATTTGCTGCTGGCAAGATTGAGAGCGGGCTCTGCATCTGTGCAGCCATGCCCAGC
AAAGTTGTCATTCTCCGCTACAATGAAAACCTCAGCAAGTACTGCATTCGGAAAGAGATTGAGACCTCGG
AGCCCTGCAGCTGTATCCACTTTACCAACTATAGTATCCTCATCGGAACCAATAAATTCTACGAAATCGA
CATGAAGCAGTACACGCTGGAGGAATTCCTGGATAAGAACGACCATTCCTTGGCGCCTGCCGTGTTTGCC
TCCTCCTCCAACAGTTTCCCCGTGTCCATCATGCAGGTGAACGGCGCGGGGCAGCGGGAGGAGTTCCTGC
TCTGTTTCCACGAATTCGGGGTGTTCGTGGATTCTTACGGAAGACGCAGCCGCACGGATGATCTCAAGTG
GAGTCGCTTACCTCTGGCCTTTGCCTACAGAGAACCCTATCTGTTTGTGACTCACTTCAACTCACTCGAA

GTAATTGAGATCCAGGCCCGCTCCTCTCTGGGGACCCCCGCCCGAGCATATTTGGAAATCCCCAACCCAC
GCTACCTGGGCCCTGCAATCTCCTCAGGAGCCATCTACCTGGCGTCCTCATATCAGGATAAGTTAAGGGT
CATTTGCTGCAAAGGAAACCTGGTGAAGGAGACCGGCACTGACCACCACCGTGGCCCGTCCACCTCCCGC
AGCCCCAACAAGCGTGGCCCGCCGACGTACAACGAGCACATCACCAAGCGCGTGGCCTCAAGCCCGGCGC
CTCCTGAAGGCCCCAGCCACCCTCGAGAGCCAAGCACACCCCACCGCTACCGCGAGGGGCGGACTGAGCT
GCGCAGGGACAAGTCTCCGGGCCGCCCCCTGGAGCGCGAGAAGTCCCCCGGCCGGATGCTGAGCACGCGG
AGGGAGCGCTCCCCCGGGCGGCTGTTTGAGGACAGCAGCAGGGGCCGGCTGCCCGTGGGGGCTGTGAGGA
CGCCGCTGTCCCAGGTCAACAAGGTCTGGGACCAGTCTTCAGTA
>Ailuropoda melanoleuca ENSAMET00000017603.1
ATGCTGAAGTTCAAATATGGAGCACGGAATCTGCTGGACGCTGGTGCTGCTGAACCCATT
GCCAGCCGAGCCTCCAGGCTGAATCATTTCTTCCAGGGAAAACCACCCTTTCTGACTCAA
CAGCAGATGTCTTCTCTTTCCCGAGAAGGGATATTAGATGCCCTCTTCGTTCTCTTCGGA
GAATGCAGTCAGCCTGCTCTGATGAAGATCAAGCACGTGAGCAACTTTGTCCGGAAGTAT
TCCGACACCATAGCTGAGTTACAGGAGCTCCAGCCTTCAGCCAAAGACTTTGAAGTCCGA
AGTCTTGTGGGTTGTGGTCACTTTGCCGAAGTGCAGGTGGTAAGGGAGCGAGCGACTGGG
GACATCTATGCCATGAAAATCATGAAGAAGAAGACCTTGTTGGCCCAAGAGCAGGTTGCA
TTTTTTGAGGAAGAACGGAACATATTATCTCAGAGCACAAGCCCTTGGATCCCCCAGTTA
CAGTATGCCTTTCAGGACAAAAATAACCTTTATCTGGTCATGGAATATCAGCCTGGAGGG
GACTTGCTGTCACTTTTGAATAGATACGAGGACCAATTAGATGAAAATATGATTCAGTTT
TACCTAGCCGAACTGATTTTGGCTGTTCACAGCATTCATGAGATGGGTTATGTACATCGA
GACGTCAAACCTGAGAACATTCTCATTGACCGAACAGGACACATCAAGCTTGTGGATTTT
GGATCAGTGGCTAAAATGAACTCAAATAAGATGGTGAACGCCAGACTCCCAATTGGGACC
CCAGATTACATGGCCCCTGAAGTGTTGACCGTGATGAATGGGGAAGGGAAAGGCGTCTAC
AGTCTCGACTGTGACTGGTGGTCGGTGGGAGTGATCGCCTATGAGATGGTTTATGGAAGG
TCCCCATTCACTGAGGGGACCTCAGCCAGAACCTTCAATAACATCATGAATTTCCAGAGG
TTTTTGAAGTTTCCTGATGACCCCAAAGTTAGTGGCGAATTACTTGATCTGATTCAGAGT
TTGCTGTGTGGCCAGAAAGAGAGACTGAAGTTTGAAGGCCTTTGCTGCCACCCTTTCTTC
TCCAAAATTGACTGGAATAACATCCGTAACTCTCCTCCCCCCTTCGTTCCCACCCTCAAG
TCTGATGATGACACCTCCAATTTTGATGAACCAGAGAAGAATTCGTGGGTTTCATCCTCT
CCGTGCCAGCTGAACCTCTCAGGTTTCTCGGGCGAAGAACTGCCGTTTGTGGGGTTTTCG
TATAGCAAGGCACTGGGGATTCTTGGTAGATCTGAGTCTGTCGTGTCAAGTCTGGACTCC
CCTGCCAAGACTAGCTCCATGGAAAAGAAACTTCTCATCAAAAGCAAAGAGCTGCAAGAC
TCCCAGGACAAGTGTCACAAGATGGAGCAGGAAATGACCCGGTTACATCGGAGAGTGTCA
GAGGTGGAGGCTGTGCTTAGTCAGAAGGAGGTGGAGCTGAAGGCCTCTGAGACTCAGAGA
TCCCTCCTGGAGCAGGACCTTGCTACCTACATCACAGAATGCAGTAGCTTAAAGCGCAGT
TTGGAGCAAGCACGGATGGAGGTGTCCCAGGAGGATGACAAAGCACTCCAGCTGCTCCAT
GATATCCGGGAGCAGAGCCGGAAGCTCCAGGAAATCAAAGAGCAGGAGTACCAGGCTCAA
GTGGAAGAAATGAGGCTGATGATGAATCAGTTGGAAGAGGACCTTGTTTCGGCAAGGAGA
CGGAGTGATCTGTATGAATCGGAGTTGAGAGAGTCTCGGCTTGCTGCTGAAGAGTTCAAG
CGGAAAGCGACAGAATGTCAACATAAACTAATGAAGGCTAAGGATCAAGGGAAGCCTGAA
GTGGGAGAATATTCCAAACTGGAGAAGATCAATGCTGAGCAACAGCTCAAAATTCAGGAG
CTCCAAGAGAAGCTGGAAAAGGCTGTCAAAGCCAGCACAGAGGCCACCGAACTGCTGCAG
AATATCCGCCAGGCAAAGGAGCGAGCCGAGCGTGAGCTGGAGAAGCTGCAGAACCGGGAG
GACTCCTCTGAAGGCATAAAAAAGAAGCTGGTGGAGGCTGAGGAACGCCGCCATTCTCTG
GAGAACAAGGTAAAGAGGCTAGAGACCATGGAGCGTAGAGAAAACAGACTGAAGGATGAC
ATCCAGACAAAATCCCAACAGATCCAGCAGATGGCTGATAAAATTCTGGAGCTGGAGGAG
AAGCACCGTGAGGCCCAGGTCTCAGCCCAGCACCTAGAGGTGCACCTGAAACAGAAAGAA

CAGCACTACGAGGAAAAAATTAAAGTGTTGGACAACCAGATAAAGAAAGACCTGGCCGAT
AAAGAGACTCTGGAGAATCTGATGCAGAGACACGAAGAGGAGGCCCACGAGAAGGGCAAA
ATTCTCAGCGAGCAGAAGGCGATGATCAATGCCATGGATTCCAAGATCAGGTCCCTGGAA
CAGAGGATCGTGGAACTCTCAGAAGCCAACAAACTTGCAGCAAACAGCAGTCTTTTTACC
CAGAGGAACATGAAGGCCCAGGAAGAGATGATTTCAGAACTCAGGCAACAGAAGTTCTAC
CTGGAGACACAGGCAGGGAAGCTAGAGGCCCAGAACCGAAAACTGGAGGAGCAGCTGGAG
AAAATCAGCCACCAAGACCACAGTGACAAGAATCGGCTGCTGGAGCTGGAGACAAGGTTG
AGGGAGGTCAGTCTAGAGCACGAGGAGCAGAAACTGGAGCTAAAGCGCCAGCTCACAGAG
CTGCAGCTCTCGCTGCAAGAGCGCGAGTCCCAGCTGACGGCCCTGCAGGCTGCCCGGGCA
GCCCTGGAGAGCCAGCTGCGCCAGGCCAAGACGGAACTGGAAGAGACCACGGCAGAGGCA
GAAGAAGAGATCCAGGCACTCACGGCACATAGAGATGAAATCCAGCGCAAATTTGACGCC
CTTCGTAACAGCTGTACTGTAATCACAGACCTGGAGGAGCAGCTGAACCAGCTGACCGAG
GACAACGCTGAGCTCAACAACCAAAATTTCTACTTGTCCAAACAACTCGACGAGGCTTCC
GGCGCCAACGATGAGATAGTACAGCTACGGAGTGAAGTGGACCATCTCCGCCGTGAGATT
ACGGAGAGGGAGATGCAGCTCACCAGCCAGAAGCAAACGATGGAGGCTCTGAAGACCACT
TGCACGATGCTGGAAGAACAGGTCATGGACCTGGAGGCCCTGAACGATGAGCTGCTGGAA
AAAGAGCGGCAGTGGGAGGCGTGGAGGAGCGTCCTTGGCGACGAGAAGTCCCAGTTTGAG
TGTCGGGTTCGAGAGTTACAGAGGATGCTGGACACCGAGAAGCAGAGCAGGGCACGGGCC
GACCAGCGGATCACCGAGTCACGCCAGGTTGTGGAGCTGGCGGTGAAGGAGCACAAGGCT
GAGATCCTTGCTCTGCAGCAGGCCCTCAAAGAGCAGAAGCTGAAAGCTGAGAGCCTCTCT
GACAAGCTCAATGACCTGGAGAAGAAACATGCCATGCTTGAAATGAACGCTCGCAGTTTA
CAGCAGAAACTGGAGACTGAACGGGAGCTCAAACAGAGGCTTCTGGAAGAGCAAGCCAAA
TTACAGCAGCAGATGGACCTACAGAAGAGTCACATCTTCCGTTTGACTCAAGGGCTGCAG
GAAGCTCTGGACCGGGCTGATCTGCTGAAGACCGAAAGGAGTGATCTGGAATACCAGCTA
GAAAACATTCAGGTTCTCTATTCTCATGAAAAGGTGAAAATGGAAGGTACTATTTCTCAA
CAAACCAAACTCATTGACTTTCTGCAAGCCAAAATGGACCAGCCCGCTAAGAAGAAAAAG
GGTTTATTTAGTCGACGGAAAGAGGACCCTGCTTTGCCCACACAGGTTCCTCTGCAGTAC
AATGAGCTGAAAGTGGCTCTGGAGAAGGAGAAAGCTCGCTGTGCAGAGCTAGAGGAAGCC
CTTCAGAAGACCCGCATCGAGCTCCGGTCTGCCCGGGAGGAAGCTGCCCACCGGAAGGCC
ACAGACCATCCGCACCCATCTACGCCAGCCACCGCGAGGCAGCAGATCGCCATGTCCGCC
ATCGTGCGGTCACCTGAGCACCAGCCCAGTGCCATGAGCCTGCTTGCCCCGCCATCCAGC
CGCAGAAAGGAGTCATCAACTCCAGAGGAATTCAGTCGGCGTCTTAAGGAGCGCATGCAC
CACAATATTCCTCACCGATTTAACGTAGGATTGAACATGCGAGCCACGAAGTGTGCTGTG
TGTCTGGATACTGTGCACTTTGGGCGCCAGGCATCCAAATGTCTTGAATGTCAGGTGATG
TGTCATCCCAAGTGCTCCACGTGCTTGCCCGCCACCTGCGGCCTGCCAGCCGAGTATGCC
ACACACTTCACTGAGGCCTTCTGCCGTGACAAAATGAACTCCCCGGGTCTCCAGAGCAAG
GAGCCCAGCAGCGGCTTGCACCTGGAAGGGTGGATGAAGGTGCCCAGGAATAACAAACGA
GGACAGCAAGGCTGGGACAGGAAGTACATTGTCCTGGAGGGATCCAAAGTCCTCATTTAT
GACAATGAAGCCAGAGAAGCTGGACAGAGGCCTGTGGAAGAATTTGAGCTGTGCCTTCCC
GACGGGGATGTATCTATTCATGGCGCCGTTGGTGCTTCTGAGCTTGCAAATACAGCCAAA
GCAGATGTCCCCTACATCCTGAAGATGGAGTCTCACCCGCACACCACGTGCTGGCCCGGG
AGAACGCTCTATTTGCTGGCTCCCAGCTTCCCTGACAAACAGCGCTGGGTCACCGCCTTA
GAGTCTGTTGTCGCAGGTGGGAGAGTTTCTAGGGAAAAAGCAGAAGCTGATGCTAAATTG
CTTGGAAACTCCCTGCTGAAACTGGAAGGTGATGACCGCCTGGACATGAACTGCACGCTG
CCCTTCAGCGACCAGGTGGTGTTGGTGGGCACTGAGGAAGGGCTCTACGCACTGAATGTC
TTGAAAAATTCCCTCACCCATGTCCCAGGAATTGGAGCGGTCTTCCAAATTTATATCATC
AAGGACCTGGAGAAGCTCCTCATGATAGCAGGTGGAGAAGAGCGGGCTCTGTGCCTTGTG

GACGTGAAGAAAGTGAAGCAGTCCCTCGCACAGGCTCACCTTCCCGCCCAGCCCGACATC
TCCCCCAACATTTTCGAAGCCGTGAAGGGCTGCCACTTGTTTGCTGCTGGCAAGATTGAG
AACGGGCTCTGCATCTGTGCAGCCATGCCCAGCAAAGTTGTCATTCTCCGCTACAACGAA
AACCTCAGCAAGTACTGCATTCGGAAAGAGATAGAGACCTCAGAGCCCTGCAGCTGCATC
CACTTCACCAATTACAGTATCCTCATTGGAACCAATAAGTTCTACGAAATTGACATGAAG
CAGTACACGCTTGAGGAATTCCTGGATAAGAATGACCATTCCTTGGCACCTGCTGTGTTT
GCCTCCTCTTCCAACAGTTTTCCTGTCTCGATAGTGCAGGTGAACGGCTCAGGGCAGCGG
GAGGAGTACCTGCTCTGCTTCCACGAATTTGGGGTGTTCGTGGATTCTTACGGAAGACGT
AGCCGCACAGACGATCTCAAGTGGAGTCGCTTACCTTTGGCCTTTGCCTACAGAGAACCC
TATCTGTTTGTGACCCACTTCAACTCACTCGAAGTCATTGAGATCCAGGCACGCTCCTCT
CTGGGGACTCCTGCCCGAGCGTATTTGGAAATCCCGAACCCACGCTACTTGGGCCCTGCA
ATTTCCTCAGGAGCGATTTACCTGGCATCCTCATACCAGGATAAATTAAGGGTCATTTGC
TGCAAAGGAAACCTCGTGAAGGAGTCCGGCACTGACCATCACCGGGGCCCCTCCACCTCC
CGCAGCTGCAGCCCCAACAAGCGAGGCCCGCCAACGTACAACGAGCACATCACCAAGCGT
GTGGCCTCTAGCCCGGCACCCCCGGAAGGCCCCAGCCACCCGCGAGAGCCAAGCACACCC
CACCGCTACCGAGAGGGGCGGACAGAGCTGCGCAGGGACAAGTCTCCCGGCCGCCCCCTG
GAACGGGAGAAGTCCCCAGGCCGGATGCTCAGCACGCGGAGGGAACGCTCCCCTGGGAGG
CTGTTCGAAGACAGCAGCCGGGGCCGGCTGCCCGTGGGAGCCGTGAGGACCCCACTGTCC
CAGGTCAACAAGGTCTGGGACCAGTCTTCAGTG
>Orcinus orca XM_004276745.2
ATGTTGAAGTTCAAGTATGGAGCACGGAATCTGCCGGACGCTGGTGCTGCAGAATCCATTGCCAGCCGGG
CCTCCAGGTTGAATCTTTTCTTCCAGGGGAAACCACCCTTCATGACTCAACAGCAGATGTCTCCTCTTTC
CCGAGAAGGGATATTAGATGCCCTCTTTGTTCTCTTTAAAGAATGCAGTCAGCCTGCTCTGATGAAGATT
AAGCACGTGAGCAACTTTGTCCAGAAGTATTCTGACATCGTAGCTGAGTTGCAGGAGCTCCAGCCTTCAG
CAAAGGACTTTGAAGGGAGAAGTGTGGTAGGTTGCGGTAACTTTGCCGAAGTGCAGGTGGTCAGAGAGAA
AGCGACCGGGGACATCTATGCCATGAAAGTCATGAAGAAGAAGGCTTTGTTGGCCCAGGAGCAGGTTTCA
TATTTTGAGGAAGAACAGAACATATTATCTCAGAGCACAAGCCCTTGGATCCCCCGGTTGCAGTATGCCT
TTCAAGACAAAAATAATCTTTATCTGGTCATGGAATATCAGCCTGGAGGGGATTTGCTGTCACTTTTGAA
CAGATATGAGGACCAATTAGATGAAAATATGATTCAGTTTTACCTAGCTGAACTCATTTTGGCTGTTCAC
AGCGTTCATCAGATGGGATATGTACATCGAGACATCAAGCCCGAGAACATCCTCATTGACCGAATGGGAC
ACATCAAGCTGGTGGATTTTGGATCAGCTGCTAAAATGAACTCAAATAAGATGGTGAATGCCAAACTCCC
AGTTGGGACCCCAGATTACATGGCCCCTGAAGTGTTGACCGTCATGAATGGGGATGGAAAAGGTGCCTAT
GGCCTAGACTGTGATTGGTGGTCAGTGGGAGTTATTGCTTATGAGATGGTTTATGGAAGGTCCCCATTCA
CTGAGGGAACCTCAGCCAGAACCTTCAGTAACATCATGAATTTCCAGCGGTTTTTGAAGTTTCCAGATGA
TCCCAAAGTTAGCAGTGAATTACTTGATCTGATCCAAAGTTTGTTGTGTGGCCAGAAAGAGAGACTGAAG
TTTGAAGGCCTTTGCTGTCATCCTTTCTTCTCTAAAATCAACTGGAGTGACATTCGAAACTCTCCTCCCC
CCTTCGTTCCCACCCTCAAGTCTGATGATGACACCTCCAATTTTGATGAACCAGAGAAGAATTCGTGGGT
TTCATCCTCTCCGTGCCAGCTGAACCCCTCAGGTTTCTCAGGAGAAGAACTGCCGTTTGTGGGGTTTTCG
TATAGCAAGGCACTGGGAATTCTTGGTAGATCTGAGTCTGTTGTGTCAAGTCTGGACTCCCCTGCCAAGA
CTAGCTCCATGGAAAAGAAACTTCTCATCAAAAGCAAAGAGCTGCAGGACTCCCAGGACAAGTGTCACAA
GATGGAGCAGGAAATGACCCGGTTACATCGGAGAGTGTCAGAGGTGGAGGCTGTGCTTAGTCAGAAGGAG
GTGGAGCTGAAGGCCTCTGAGACTCAGAGATCCCTCCTGGAGCAGGACCTTGCTACCTACATCACAGAGT
GCAGTAGCTTAAAGCGAAGTTTGGAGCAAGCACGGATGGAGGTGTCCCAGGAGGACGACAAAGCACTGCA
GCTTCTCCACGATATCAGAGAGCAGAGCCGGAAGCTCCAAGAAATCAAAGAGCAGGAGTACCAGGCTCAA
GTGGAAGAAATGAGGTTAATGATGAATCAGTTGGAAGAGGACCTGGTTTCGGCCAGAAGACGGAGTGATC
TCTATGAATCGGAGCTCAGAGAGTCTCGGCTGGCTGCCGAAGAGTTCAAGCGGAAAGCAACAGAATGTCA
GCATAAATTGATGAAGACTAAAGATCAAGGGAAGCCTGAAGTGGGAGAATATGCCAAACTGGAGAAGATC

AATGCTGAGCAGCAGCTCAAAATTCAGGAGCTCCAAGAGAAGCTGGAAAAGGCCGTGAAAGCCAGCACGG
AGGCCACCGAGCTTCTGCAGAACATCCGCCAGGCGAAGGAGCGGGCTGAGCGCGAGCTGGAGAAGCTGCA
CAACCGCGAGGACTCTTCCGAAGGCATCAAGAAGAAGCTGGTGGAAGCCGAGGAACGCCGCCACTCTCTG
GAGAACAAGGTAAAGAGGCTAGAGACCATGGAGCGTAGAGAAAACAGACTGAAGGATGACATCCAGACAA
AGTCCCAACAGATCCAGCAGATGGCTGATAAAATTCTGGAACTGGAGGAGAAGCACCGAGAAGCCCAGGT
CTCAGCCCAGCACCTGGAGGTGCACCTGAAACAGAAAGAACAGCACTACGAGGAAAAAATTAAAGTGTTG
GACAATCAGATAAAGAAAGACCTAGCCGACAAGGAGACTTTGGAGAATCTGATGCAGAGACACGAAGAGG
AGGCCCACGAGAAAGGCAAGATTCTCAGCGAGCAGAAGGCGATGATCAACGCCATGGATTCCAAGATCAG
ATCCCTGGAACAGAGGATAGTGGAACTGTCGGAAGCCAATAAACTTGCGGCAAACAGCAGTCTCTTTACC
CAGAGGAACATGAAGGCCCAGGAAGAAATGATTTCAGAACTCAGGCAGCAGAAGTTTTACCTGGAGACAC
AGGCTGGGAAACTGGAGGCCCAGAACCGGAAGCTGGAGGAGCAGCTGGAAAAAATCAGCCACCAAGACCA
CAGCGACAAGAATCGTCTGCTGGAGCTGGAGACCAGGCTGAGGGAGGTCAGTCTAGAGCATGAGGAGCAG
AAACTGGAGCTCAAGCGTCAGCTCACGGAGCTGCAGCTCTCTCTGCAGGAGCGCGAGTCTCAGCTGACAG
CCCTGCAGGCAGCCCGGGCAGCTCTGGAGAGCCAGCTCCGCCAGGCAAAGACAGAGCTGGAAGAGACAAC
AGCGGAAGCAGAAGAAGAGATCCAGGCGCTCACGGCACATAGAGATGAAATCCAGCGCAAATTTGATGCC
CTTCGTAACAGCTGTACTGTAATCACAGACCTGGAGGAGCAGCTAAACCAGCTGACGGAGGACAATGCTG
AGCTCAACAACCAAAATTTCTACTTGTCCAAACAACTTGATGAGGCCTCTGGCGCCAGTGATGAGATCGT
ACAGCTGCGAAGTGAAGTAGACCATCTCCGCCGCGAGATCACGGAGAGGGAGATGCAGCTCACCAGCCAG
AAGCAAACGATGGAGGCTCTGAAGACTACCTGCACGATGCTGGAGGAGCAGGTCATGGACCTGGAAGCCC
TGAACGACGAGCTGCTGGAAAAAGAGCGGCAGTGGGAGGCCTGGAGGAGCGTCCTTGGTGACGAGAAGTC
CCAGTTTGAGTGTCGGGTTCGAGAATTACAGAGGATGCTGGACACTGAGAAGCAGAGCAGGGCGAGGGCC
GATCAGCGGATCACCGAGTCGCGCCAGGTGGTCGAGCTAGCAGTGAAGGAGCACAAGGCTGAGATTCTGG
CCCTGCAGCAGGCTCTCAAGGAACAGAAGCTGAAGGCCGAGAGTCTCTCCGACAAGCTCAATGACCTGGA
GAAGAAACACGCCATGCTTGAAATGAATGCCCGAAGTTTACAACAGAAGCTGGAGACGGAACGAGAGCTC
AAACAAAGGCTTCTGGAAGAGCAAGCCAAGTTACAGCAGCAGATGGACATGCAAAAGAGTCATATTTTCC
GTCTGACTCAAGGGCTACAGGAAGCTCTAGATCGGGCTGATCTGCTGAAGACAGAAAGGAGTGATCTGGA
ATATCAGCTAGAGAACATTCAGGTTCTCTATTCTCATGAAAAGGTGAAAATGGAAGGCACTATTTCGCAA
CAAACCAAACTCATCGATTTTCTGCAAGCCAAAATGGACCAACCTGCTAAAAAGAAAAAGGGTTTATTTA
GTCGACGGAAAGAGGACCCTGCTTTGCCCACACAGGTTCCTCTGCAGTACAATGAGCTGAAGGTGGCTCT
GGAGAAGGAGAAAGCTCGCTGCGCGGAGCTCGAGGAAGCCCTGCAGAAGACCCGCATCGAGCTCCGCTCC
GCCCGGGAGGAAGCAGCCCACCGGAAAGCCGCAGACCACCCGCACCCGTCCACGCCGGCCACCGCCAGGC
AGCAGATCGCCATGTCCGCCATTGTGCGGTCGCCCGAGCACCAGCCCAGCGCCCTGAGCCTGCTCGCCCC
GCCGTCCAGCCGCAGGAAGGAGTCTTCGACTCCAGAGGAATTCAGCCGACGTCTTAAGGAGCGCATGCAC
CACAACATTCCTCACCGGTTCAATGTAGGACTGAACATGCGAGCCACGCAGTGCGCTGTGTGTCTGGATA
CCGTGCACTTTGGGCGCCAGGCATCCAAGTGTCTTGAATGTCAGGTCATGTGTCATCCCAAGTGCTCCAC
GTGCTTGCCGGCCACCTGTGGCCTGCCAGCCGAATATGCCACGCACTTCACTGAGGCCTTCTGCCGTGAC
AAAATGAACTCCCCGGGTCTCCAGACCAAGGAGCCCAGCAGCGGCTTGCACCTGGAAGGGTGGATGAAGG
TGCCCAGGAATAACAAACGAGGACAGCAAGGCTGGGACAGGAAGTACATCGTCCTGGAGGGCTCGAAAGT
CCTCATTTACGACAGTGAAGCCAGAGAAGCTGGACAGAGGCCGGCGGAAGAATTTGAGCTGTGCCTTCCC
GACGGGGACGTGTCTATTCATGGCGCCGTTGGTGCTTCTGAACTCGCAAACACAGCCAAAGCAGATGTCC
CCTACGTGCTGAAGATGGAATCTCACCCGCACACCACCTGCTGGCCCGGGAGAACCCTCTACTTGCTGGC
TCCCAGCTTTCCCGACAAGCAGCGCTGGGTCACCGCCTTAGAGTCAGTTGTCGCAGGTGGGAGAGTGTCT
AGGGAAAAGGCAGAAGCCGATGCTAAATTGCTTGGAAACTCCCTGCTGAAACTGGAAGGCGATGACCGGC
TCGACATGAACTGCACGCTGCCCTTCAGCGACCAGGTGGTGCTGGTGGGCACCGAGGAAGGGCTGTACGC
GCTGAATGTCTTGAAGAACTCCCTCACCCACGTCCCAGGGATCGGAGCGGTCTTCCAGATTTACATCATC
AAGGACCTGGAGAAGCTCCTCATGATTGCAGGAGAAGAGCGGGCCCTGTGTCTCGTGGACGTGAAGAAAG
TGAAACAGTCCCTCGCACAGTCTCACCTTCCCGCCCAGCCGGACATCTCACCCAACATCTTCGAAGCCGT

CAAGGGCTGCCACTTGTTTGCTGCCGGCAAGATTGAGAGCGGGCTCTGCATCTGTGCGGCCATGCCCAAC
AAAGTTGTCATTCTCCGCTACAACGAAAACCTCAGCAAGTACTGCATTCGGAAAGAGATCGAGACCTCGG
AGCCCTGCAGCTGTGTCCACTTCACCAACTACAGTATCCTCATCGGAACCAATAAATTCTACGAGATTGA
CATGAAGCAGTACACGCTGGAGGAATTCCTGGATAAGAACGACCATTCCTTGGCGCCTGCCGTGTTTGCC
TCCTCTTCCCACAGTTTCCCCGTGTCCATCATGCAGGTGAACGGCGCGGGGCAGCGGGAGGAGTTCCTGC
TCTGCTTCCATGAGTTTGGGGTGTTCGTGGATTCTTACGGAAGACGCAGCCGCACAGATGATCTCAAGTG
GAGTCGCTTACCTTTGGCCTTCGCCTACAGAGAACCTTATCTGTTTGTGACCCACTTCAACTCACTCGAA
GTAATTGAGATCCAGGCCCGCTCCTCTCTAGGGACCCCTGCCCGAGCGTACTTGGAAATCCCGAACCCAC
GCTACCTGGGCCCTGCAATTTCCTCGGGAGCTATTTACCTGGCGTCCTCGTATCAGGATAAATTAAGGGT
CATCTGCTGCAAAGGAAACCTCGTGAAGGAGACCGGCACTGACCAGCACCGGGGCCCGTCCACCTCCCGC
AGCCCCAACAAGCGAGGCCCGCCGACGTACAACGAGCACATCACTAAGCGTGTGGCCTCCAGCCCGGCGC
CCCCTGAAGGCCCCAGCCACCCGCGAGAGCCCGGCACGCCCCACCGCTACCGCGAGGGGCGGACGGAGCT
GCGCAGGGACAAGTCTCCGGGCCGCCCCCTGGAGCGGGAGAAGTCCCCCGGCCGGATGCTCAGCACGCGC
AGGGAGCGCTCCCCCGGGAGGCTGTTTGAGGACGGCAGCAGGGGCCGGCTGCCCGTGGGAGCCGTGAGGA
CCCCGCTGTCCCAGGTCAACAAGGTCTGGGACCAGTCTTCAGTA
>Mustela putorius furo XM_013056605.1
ATGCTGAAGTTCAAATATGGAGCACGGAATTTGCTGGACGCTGGTGCTGCTGAATCCATTGCCACCCGGG
CCTCCAGGCTGAATCATTTCTTCCAGGGGAAACCACCCTTTCTGACTCAACAGCAGATGTCTCCGCTTTC
CCGAGAAGGGATATTAGATGCCCTCTTCGTTCTCTTCGGAGAATGCAGTCAGCCTGCTCTGATGAAGATC
AAGCATGTGAGCAACTTTGTCCGGAAGTATTCCGACACCATAGCTGAATTACAGGAGCTCCAGCCTTCAG
CCAAGGACTTTGAAGTCCGAAGTCTTGTGGGCTGTGGTCACTTTGCTGAAGTGCAGGTGGTAAGGGAGCG
AGCCACTGGGGACATCTATGCCATGAAAGTCATGAAGAAGGAGACCTTGTTGGCCCAGGAGCAGGTTTCA
TTTTTTGAGGAAGAACAGAACATATTATCTCGGAGCACAAGCCCTTGGATCCCCCAGTTACAGTACGCCT
TTCAGGACAAAAATAACCTCTATCTGGTCATGGAATATCAGCCTGGAGGCGACTTGCTGTCACTTTTGAA
TAGATATGAGGACCAATTAGATGAAAATATGATTCAGTTTTACCTAGCCGAACTGATTTTGGCTGTTCAC
AGTGTTCATCAGATGGGATATGTACATCGAGACATCAAACCCGAGAATATTCTCATTGACCGAACAGGAC
ACATCAAGCTTGTGGATTTCGGATCAGCGGCTAAAATGAACTCAAATAAGATGGTGAATGCCAAGCTCCC
AATTGGGACTCCAGATTACATGGCCCCTGAAGTGTTGACTGTGCTGAATGGGGATGGGAAAGGCACCTAC
AGTCTCGACTGTGACTGGTGGTCGGTGGGTGTGATCGCCTACGAGATGGTTTACGGAAGGTCCCCGTTCA
CTGAGGGGACCTCAGCCAGAACCTTCAATAACATCATGAATTTCCAGCGGTTTTTGAAGTTTCCCGATGA
CCCCAAAGTTAGCAGTGAATTACTTGATCTGATTCAGAGTTTGCTGTGTGGCCAGAAAGAGAGACTGAAG
TTTGAAGGCCTTTGCTGCCACCCTTTCTTCTCTAAAATTGACTGGGATAACATCCGTAACTCTCCTCCCC
CCTTCGTTCCCACCCTCAAGTCTGATGATGACACCTCCAATTTTGATGAACCAGAGAAGAATTCGTGGGT
TTCATCCTCTCCGTGCCAGCTGAACCTCTCAGGTTTCTCGGGCGAAGAACTGCCGTTTGTGGGGTTTTCG
TATAGCAAGGCACTGGGGATTCTTGGTAGATCTGAGTCTGTCGTGTCAAGTCTGGACTCCCCTGCCAAGA
CTAGCTCCATGGAAAAGAAACTTCTCATCAAAAGCAAAGAGCTGCAAGACTCCCAGGACAAGTGTCACAA
GATGGAGCAGGAAATGACCCGGTTACATCGGAGAGTGTCAGAGGTGGAGGCTGTTCTTAGTCAGAAGGAG
GTGGAGCTGAAGGCCTCTGAGACTCAGAGATCCCTCCTGGAGCAGGACCTTGCCACCTACATCACAGAAT
GCAGTAGCTTAAAGCGAAGTTTGGAGCAAGCACGGATGGAGGTGTCCCAGGAGGATGACAAAGCACTGCA
GCTTCTCCATGATATCCGAGAGCAGAGCCGGAAGCTCCAGGAAATCAAAGAGCAGGAGTACCAGGCTCAA
GTGGAAGAAATGAGGTTGATGATGAATCAGTTGGAAGAGGACCTTGTTTCGGCAAGGAGACGGAGTGATC
TCTATGAATCGGAGTTGAGAGAGTCTCGGCTCGCTGCTGAAGAGTTCAAGCGGAAAGCGACAGAATGTCA
GCATAAACTAATGAAGGCTAAGGATCAAGGGAAGCCTGAAGTGGGGGATTATTCCAAACTGGAGAAGATC
AATGCTGAGCAACAGCTCAAAATTCAGGAGCTCCAAGAGAAGCTGGAAAAGGCTGTAAAAGCCAGCACAG
AGGCCACCGAGCTGCTGCAGAATATCCGCCAGGCCAAGGAGCGGGCCGAGCGTGAGCTGGAGAAGCTGCA
GAACCGGGAGGACTCCTCCGAAGGCATAAAAAAGAAGCTGGTGGAGGCCGAGGAACGCCGCCATTCTCTG
GAGAACAAGGTAAAGAGGCTAGAGACCATGGAGCGTAGAGAAAACAGACTGAAGGATGACATCCAGACAA

AATCCCAACAGATCCAGCAGATGGCTGATAAAATTCTGGAGCTGGAGGAGAAGCACCGCGAGGCCCAGGT
CTCCGCCCAGCACCTAGAGGTGCACCTGAAACAGAAAGAACAGCACTACGAGGAAAAAATTAAAGTGTTG
GACAACCAGATAAAGAAAGACCTGGCCGATAAAGAGACTCTGGAGAATCTGATGCAGAGACACGAAGAGG
AGGCCCACGAGAAGGGCAAAATTCTCAGTGAGCAGAAGGCGATGATCAATGCTATGGATTCCAAGATCAG
ATCCCTGGAACAGAGGATTGTGGAACTCTCAGAAGCCAACAAACTTGCGGCGAACAGCAGTCTTTTCACC
CAGAGGAACATGAAGGCCCAGGAAGAGATGATTTCAGAACTCAGGCAACAGAAGTTCTACCTGGAGACAC
AGGCAGGGAAGCTGGAGGCCCAGAACCGAAAGCTGGAGGAGCAGCTGGAGAAAATCAGCCACCAAGACCA
CAGTGACAAGAATCGGCTGCTGGAGCTGGAGACAAGGTTGAGGGAGGTCAGTCTAGAGCACGAGGAGCAG
AAGCTGGAGCTAAAGCGCCAGCTCACAGAGCTGCAGCTCTCGCTGCAGGAGCGCGAGTCCCAGCTGACGG
CCCTGCAGGCGGCCCGGGCAGCCCTGGAGAGCCAGCTGCGCCAGGCCAAGACGGAACTGGAAGAGACAAC
AGCGGAAGCGGAAGAGGAGATCCAGGCACTCACGGCACATAGAGATGAAATCCAACGCAAATTTGATGCC
CTTCGTAACAGCTGTACTGTAATCACAGACCTGGAGGAACAGCTAAACCAGCTCACCGAGGACAATGCTG
AGCTCAACAACCAAAATTTCTACTTGTCCAAACAACTCGACGAGGCTTCCGGCGCCAACGATGAGATAGC
ACAGCTCCGTAGCGAAGTGGATCATCTTCGCCGTGAGATCACGGAGAGGGAGATGCAGCTCACCAGCCAG
AAGCAAACGATGGAGGCTCTGAAGACCACTTGCACGATGCTGGAAGAACAGGTCATGGACCTGGAAGCTC
TGAATGATGAGCTGCTGGAAAAAGAGCGGCAGTGGGAGGCGTGGAGGAGCGTCCTTGGCGACGAGAAGTC
CCAGTTTGAGTGTCGGGTTCGAGAGTTACAGAGGATGCTGGACACCGAAAAGCAAAGCAGGGCGCGGGCC
GACCAGCGGATCACCGAGTCCCGCCAGGTGGTGGAGCTGGCCGTGAAGGAGCACAAGGCTGAGATCCTCG
CTCTGCAGCAGGCCCTCAAGGAGCAGAAGCTGAAGGCTGAGAGCCTCTCCGACAAGCTCAATGACCTGGA
GAAGAAACATGCCATGCTTGAAATGAACGCCCGCAGTTTACAGCAGAAACTGGAGACTGAACGGGAGCTC
AAACAGAGGCTTCTGGAAGAGCAAGCCAAATTACAGCAGCAGATGGACATGCAGAAGAGCCACATCTTCC
GTCTGACTCAAGGGCTGCAAGAAGCTCTGGACCGGGCGGATCTGCTGAAGACGGAAAGGAGCGATCTGGA
ATACCAGCTAGAAAACATTCAGGTTCTCTATTCTCACGAAAAGGTGAAAATGGAAGGTACTATTTCTCAA
CAAACCAAACTCATTGACTTTCTGCAAGCCAAAATGGACCAGCCCGCTAAGAAGAAAAAGGGTTTATTTA
GTCGACGGAAAGAGGACCCTGCTTTGCCCACACAGGTTCCTCTGCAGTACAATGAGCTGAAGGTGGCTCT
GGAGAAGGAGAAAGCTCGCTGCGCAGAGCTAGAGGAAGCCCTTCAGAAGACCCGCATCGAGCTCCGGTCT
GCCCGGGAGGAAGCTGCCCACCGGAAGGCCACAGACCATCCACACCCATCTACGCCAGCCACCGCGAGGC
AGCAGATCGCCATGTCCGCCATCGTGCGGTCACCCGAGCACCAGCCCAGTGCCATGAGCCTGCTGGCCCC
GCCATCTAGCCGCAGAAAGGAGTCGTCCACTCCAGAGGAATTCAGTCGGCGTCTTAAGGAGCGCATGCAC
CACAATATTCCTCACCGATTTAACGTAGGACTGAATATGCGAGCCACAAAGTGTGCTGTGTGTCTGGATA
CTGTGCACTTTGGGCGCCAGGCATCCAAATGTCTTGAATGTCAGGTGATGTGCCATCCCAAGTGCTCCAC
GTGCTTGCCCGCCACCTGTGGCCTGCCAGCCGAGTATGCCACACACTTTACCGAGGCCTTCTGCCGTGAC
AAAATGAACTCCCCAGGTCTCCAGTCCAAGGAGCCCAGCAGCGGCTTGCACCTGGAAGGGTGGATGAAGG
TGCCCAGGAATAACAAGCGAGGACAGCAGGGCTGGGACAGGAAGTACATTGTCCTGGAGGGATCCAAAGT
CCTCATTTATGACAATGAAGCCAGAGAAGCTGGACAGAGGCCGGTGGAAGAATTTGAGCTGTGCCTTCCC
GACGGGGATGTATCTATTCATGGCGCCGTTGGTGCTTCTGAGCTTGCAAATACAGCCAAAGCAGATGTTC
CCTACGTACTGAAGATGGAGTCTCACCCACACACCACCTGCTGGCCCGGGAGGACGCTCTACTTGCTGGC
TCCCAGCTTCCCCGACAAACAGCGCTGGGTCACCGCCTTAGAGTCTGTTGTCGCAGGTGGGAGAGTTTCT
AGGGAAAAAGCAGAAGCCGATGCTAAATTGCTCGGAAACTCTCTGTTGAAACTGGAAGGTGATGACCGTC
TGGACATGAACTGCACGCTGCCCTTCAGCGACCAGGTGGTGTTGGTGGGCACTGAGGAAGGGCTGTACGC
TCTGAATGTCTTGAAAAACTCCCTCACCCACGTCCCAGGAATCGGAGCAGTCTTCCAAATTTATATCATC
AAGGACCTGGAGAAGCTCCTCATGATAGCAGGAGAAGAACGGGCTCTGTGCCTTGTTGACGTGAAGAAAG
TGAAGCAGTCCCTCGCACAGGCTCACCTTCCTGCCCAGCCCGACATCTCCCCCAACGTGTTCGAGGCCGT
TAAGGGCTGCCACTTGTTTGCTGCTGGCAAGATTGAGAACGGGCTCTGCATCTGTGCAGCCATGCCCAGC
AAAGTTGTCATTCTCCGCTACAACGAAAACCTCAGCAAGTACTGCATTCGCAAAGAGATAGAGACCTCAG
AGCCCTGCAGCTGCATCCACTTCACCAATTACAGTATCCTCATTGGAACCAATAAATTCTACGAAATTGA
CATGAAGCAGTACACGCTTGAGGAATTCCTGGATAAGAATGACCATTCCTTGGCGCCTGCTGTGTTTGCC

TCCTCTTCCAACAGTTTCCCTGTCTCGATCATGCAGGTGAACGGCGCAGGGCAGCGGGAGGAGTACCTGC
TCTGCTTCCACGAATTTGGGGTGTTCGTGGATTCTTACGGAAGACGTAGCCGCACAGACGATCTCAAGTG
GAGTCGCTTACCTCTGGCCTTCGCCTACAGAGAACCCTATCTGTTTGTGACCCACTTCAACTCACTTGAA
GTCATTGAGATCCAGGCACGCTCCTCTCTGGGGACTCCTGCCCGAGCGTATTTGGAAATCCCAAACCCAC
GCTACTTGGGCCCTGCAATTTCCTCAGGAGCGATTTACCTGGCCTCGTCATACCAGGATAAATTAAGGGT
CATTTGCTGCAAAGGAAACCTCGTGAAGGAGTCCGGCACTGACCACCACCGGGGCCCGTCCACCTCCCGC
AGCCCCAACAAGCGAGGCCCACCAACATACAACGAGCACATCACCAAGCGTGTGGCCTCTAGCCCGGCAC
CCCCACCGGAAGGCCCCAGCCACCCGCGAGAGCCAAGCACACCCCACCGCTACCGAGAGGGGCGGACAGA
GCTGCGCAGGGACAAGTCTCCGGGCCGCCCCCTGGAGCGGGAGAAGTCGCCAGGCCGGATGCTCAGCACG
AGGAGGGAACGCTCCCCTGGGAGGCTGTTTGAAGACAGCAGCCGGGGCCGGCTGCCTGTGGGAGCTGTGA
GGACCCCACTGTCCCAGGTCAATAAGGTCTGGGACCAGTCTTCAGTG
>Felis catus XM_019814614.1
ATGTTGAAGTTCAAATATGGAGCAAGGAATCTGCTGGACGCTGGTGCTGCTGAACCCATTGCCAGCCGGG
CCTCCAGGTTGAATCATTTCTTCCAGGGGAAACCACCCTTTCTGACTCAACAGCAGATGTCTCCTCTTTC
CCGAGAAGGGATATTAGATGCCCTCTTCGTTCTCTTTGAAGAATGCAGTCAGCCTGCTCTGATGAAGATT
AAGCACGTGAGCAACTTTGTCCGCAAATATTCCGACACCATCGCTGAGTTACAGGAGCTCCAGCCTTCAG
CAAAAGACTTTGAAGTCCGAAGTCTCGTGGGCTGTGGTCACTTTGCTGAAGTGCAGGTGGTAAGAGAGCG
AGCAACTGGCGACGTCTATGCCATGAAGGTCATGAAGAAGACGGCCTTGTTGGCCCAAGAGCAGGTTTCA
TTTTTTGAGGAAGAACGGAACATATTATCTCAGAGCACAAGCCCTTGGATTCCCCAGTTACAGTATGCCT
TTCAGGACAAAAATAACCTTTATCTGGTCATGGAATATCAGCCTGGAGGGGACTTGCTGTCACTTTTGAA
CAGATACGAGGACCAATTAGATGAAAATACGATTCAGTTTTACCTAGCCGAATTGATTTTGGCTGTTCAC
AGCATTCATCAGATGGGATATGTGCATCGAGACGTCAAGCCTGAGAACATACTCATTGACCGAATAGGAC
ACATCAAGCTTGTGGATTTTGGATCAGCGGCTAAAATGAGCTCGAATAAGATGGTGAACGCAAAACTCCC
GATTGGGACCCCAGATTACATGGCCCCTGAAGTGTTGACTGTGATGAATGGGGACGGAAAAGGCGTCTAT
AGTCTGGATTGTGACTGGTGGTCAGTGGGAGTTATTGCTTATGAGATGGTTTATGGAAGATCCCCATTCA
CTGAGGGAACGTCAGCCAGAACCTTCAATAACATCATGAATTTCCAGCGGTTTTTGAAGTTTCCAGATGA
TCCAAAAGTTAGCAGCGAATTACTTGATCTGATTCAGAGTTTGTTGTGTGGCCAGAAAGAGAGACTGAAG
TTTGAAGGCCTTTGCTGCCACCCTTTCTTCTCCAAAATCGACTGGAATAACATTCGTAACTCTCCTCCCC
CCTTCGTTCCCACCCTCAAGTCTGATGATGACACCTCCAATTTTGATGAACCAGAGAAGAATTCGTGGGT
TTCATCCTCTCCGCGCCAGCTGAACCTCTCCGGTTTCTCGGGCGAAGAACTGCCGTTTGTGGGGTTTTCG
TATAGCAAGGCACTGGGAATTCTTGGTAGATCTGAGTCTGTTGTGTCAAGTCTGGACTCCCCTGCCAAGA
CTAGCTCCATGGAAAAGAAACTTCTCATCAAAAGCAAAGAGCTGCAAGACTCCCAGGACAAGTGCCACAA
GATGGAGCAGGAAATGACCCGGTTACATCGGAGAGTGTCAGAGGTGGAGGCTGTGCTTAGTCAGAAGGAG
GTGGAGCTGAAGGCCTCTGAGACTCAGAGATCCCTCCTGGAGCAGGACCTTGCTACCTACATCACAGAAT
GCAGTAGCTTAAAGCGAAGTTTGGAGCAAGCACGGATGGAGGTGTCCCAGGAGGATGACAAAGCGCTTCA
GCTTCTCCATGACATCAGAGAGCAGAGCCGGAAGCTCCAGGAAATCAAAGAGCAGGAGTACCAGGCTCAA
GTGGAAGAAATGAGGTTAATGATGAATCAGCTGGAAGAGGACCTCGTTTCAGCGAGGAGACGGAGTGATC
TCTATGAATCGGAGTTGAGAGAGTCTCGGCTCGCTGCTGAAGAGTTCAAGCGGAAAGCCACAGAATGTCA
GCATAAACTGATGAAGGCTAAGGATCAAGGGAAGCCTGAAGTGGGAGAATATTCCAAACTAGAGAAGATC
AATGCTGAGCAACAGCTCAAAATTCAGGAGCTCCAAGACAAGCTGGAAAAGGCTGTAAAAGCCAGCACAG
AGGCCACTGAGCTGCTGCAGAATATCCGCCAGGCAAAGGAGCGAGCCGAGCGTGAGCTGGAGAAGCTGCA
GAACCGTGAGGACTCCTCTGAAGGCATAAAAAAGAAGCTGGTGGAGGCCGAGGAACGCCGCCATTCTCTG
GAGAACAAGGTAAAGAGGCTAGAGACCATGGAGCGTAGAGAAAACAGACTGAAGGATGACATCCAGACAA
AATCCCAACAGATCCAGCAGATGGCTGATAAAATTCTGGAGCTGGAGGAGAAGCACCGCGAGGCCCAGGT
CTCAGCCCAGCACCTAGAGGTGCACCTGAAACAGAAAGAACAGCACTACGAAGAAAAAATTAAAGTGTTG
GACAACCAGATAAAGAAAGACCTGGCCGATAAAGAGACTCTGGAGAATCTGATGCAGAGACACGAAGAGG
AGGCCCACGAGAAGGGCAAAATTCTCAGTGAGCAGAAGGCGATGATCAATGCTATGGATTCCAAGATCAG

ATCCCTGGAACAGAGGATTGTGGAACTCTCAGAAGCCAATAAACTTGCAGCAAACAGCAGTCTTTTTACC
CAGAGGAACATGAAGGCCCAGGAAGAGATGATTTCAGAACTCAGGCAGCAGAAGTTCTACCTGGAGACAC
AGGCAGGGAAACTGGAGGCCCAGAACCGAAAGCTGGAGGAGCAGCTGGAGAAAATCAGCCACCAAGACCA
CAGCGACAAGAACCGGCTGCTGGAGCTGGAGACGAGGCTGAGGGAGGTCAGTCTAGAGCACGAGGAGCAG
AAACTGGAGCTGAAGCGTCAGCTCACAGAGCTGCAACTCTCCCTGCAAGAGCGCGAGTCCCAGCTGACGG
CCCTGCAGGCGGCCCGGGCGGCCCTGGAGAGCCAGCTTCGCCAGGCCAAGACCGAGCTGGAGGAGACGAC
GGCGGAGGCAGAAGAAGAGATCCAGGCGCTCACGGCACATAGAGATGAAATCCAGCGCAAATTTGACGCC
CTTCGTAACAGCTGTACTGTAATCACAGACCTGGAGGAGCAGCTAAACCAGCTGACCGAGGACAACGCTG
AGCTCAACAACCAAAATTTCTACTTGTCCAAACAACTCGACGAGGCTTCTGGCGCCAACGATGAGATAGT
ACAGCTACGAAGCGAAGTGGACCATCTCCGCCGCGAGATTACGGAGAGAGAGATGCAGCTCACCAGCCAG
AAGCAAACGATGGAGGCTCTGAAGACTACTTGCACGATGCTGGAAGAACAGGTCATGGACCTGGAGGCCC
TAAACGACGAACTGCTGGAAAAAGAGCGTCAGTGGGAGGCGTGGAGGAGTGTCCTTGGTGACGAGAAGTC
CCAGTTTGAGTGTCGGGTTCGAGAGTTACAGAGGATGCTGGACACCGAGAAGCAGAGCAGGGCGAGGGCC
GATCAGCGGATCACTGAGTCCCGCCAGGTGGTGGAGCTGGCGGTGAAGGAGCACAAGGCTGAGATTCTCG
CTCTGCAGCAGGCCCTCAAAGAGCAGAAGCTGAAAGCCGAGAGCCTCTCTGACAAGCTCAATGATCTGGA
GAAGAAACATGCCATGCTTGAAATGAACGCCCGAAGTTTACAGCAGAAACTGGAGACTGAACGGGAGCTC
AAACAGAGGCTTCTGGAAGAGCAAGCCAAATTACAGCAGCAGATGGATCTGCAGAAGAATCACATCTTCC
GTCTGACTCAAGGGCTGCAGGAAGCCCTAGACCGGGCTGATCTGCTGAAGACGGAAAGGAGCGATCTGGA
GTACCAGCTGGAAAACATTCAGGTTCTCTATTCTCATGAAAAAGTGAAAATGGAAGGTACTATTTCTCAA
CAAACCAAACTCATTGATTTTCTGCAAGCCAAAATGGACCAGCCCGCTAAAAAGAAAAAGGGTTTATTTA
GTCGACGGAAAGAGGACCCTGCTTTGCCCACACAGGTTCCTCTGCAGTACAATGAGCTGAAGGTGGCTCT
GGAGAAGGAGAAAGCTCGCTGTGCAGAGCTGGAGGAAGCCCTTCAGAAGACCCGCATCGAGCTCCGGTCT
GCCCGGGAGGAAGCTGCCCACCGGAAGGCCACAGACCACCCGCACCCATCTACGCCAGCCACCGCGAGGC
AGCAGATCGCCATGTCCGCCATCGTGCGGTCGCCCGAGCACCAGCCCAGTGCCATGAGCCTGCTCGCCCC
GCCATCCAGCCGCAGAAAGGAGTCATCGACTCCAGAGGAATTCAGTCGGCGTCTTAAGGAGCGCATGCAC
CACAATATTCCTCACCGATTTAACGTAGGATTGAACATGCGAGCCACAAAGTGCGCTGTGTGTCTGGATA
CTGTGCACTTTGGGCGCCAGGCATCCAAATGTCTTGAATGTCAGGTGATGTGTCATCCCAAGTGCTCTAC
GTGCTTGCCAGCCACCTGTGGCCTGCCAGCCGAGTATGCCACACACTTCACTGAGGCCTTCTGCCGTGAC
AAAATGAACTCCCCGGGTCTCCAGACCAAGGAGCCCAGCAGCGGCCTGCACCTGGAAGGGTGGATGAAAG
TGCCCAGGAATAACAAACGAGGACAGCAAGGCTGGGACAGGAAGTACATTGTCCTGGAGGGATCTAAAGT
CCTCATTTATGACAGCGAAGCCAGAGAAGCTGGACAGAGGCCGGTGGAAGAATTTGAGCTGTGCCTTCCC
GACGGGGATGTATCTATTCATGGCGCCGTTGGTGCTTCTGAGCTTGCAAATACAGCCAAAGCAGATGTCC
CCTACATACTGAAGATGGAGTCTCACCCACACACCACCTGCTGGCCTGGGAGAACGCTCTACTTGCTGGC
TCCCAGCTTCCCCGACAAACAGCGCTGGGTCACCGCCTTAGAGTCTGTGGTCGCAGGTGGGAGAGTTTCT
AGGGAAAAAGCAGAAGCTGATGCTAAATTGCTTGGAAACTCCCTGCTGAAACTGGAAGGTGATGACCGTC
TAGACATGAACTGCACACTGCCCTTCAGCGACCAGGTGGTGTTGGTAGGCACTGAGGAAGGGCTCTACGC
TCTGAATGTCTTGAAAAACTCCCTCACCCATGTCCCAGGAATCGGAGCAGTCTTCCAAATTTATATCATC
AAGGACCTGGAGAAGCTACTCATGATAGCAGGAGAAGAGCGAGCTCTGTGCCTTGTTGACGTGAAAAAAG
TGAAGCAGTCTCTGGCGCAGGCTCACCTTCCCGCCCAGCCCGACATCTCACCCAACGTTTTTGAAGCCGT
GAAGGGCTGTCACTTGTTTGCTGCTGGCAAGATTGAGAACGGGCTCTGCATCTGTGCAGCCATGCCCAGC
AAAGTCATCATTCTCCGCTACAATGAAAACCTCAGCAAGTACTGCATTCGGAAAGAGATAGAGACCTCAG
AGCCCTGCAGCTGCATCCACTTCACCAATTACAGTATCCTTATTGGAACCAATAAATTCTACGAAATTGA
CATGAAGCAGTACACGCTTGAGGAATTCCTGGATAAGAATGACCATTCCTTGGCGCCTGCTGTGTTTGCC
TCCTCTTCCAATAGTTTCCCTGTCTCGATCGTGCAGGTGAACGGCGCAGGGCAGCGGGAGGAGTACCTGC
TCTGCTTCCACGAATTTGGGGTGTTCGTGGATTCTTATGGAAGACGTAGCCGCACAGATGATCTCAAGTG
GAGTCGCTTACCTTTGGCCTTTGCCTACAGAGAACCCTATCTGTTTGTGACCCACTTCAACTCACTAGAA
GTAATTGAGATCCAGGCACGCTCTTCTCTGGGGACCCCTGCCCGAGCGTATTTGGAAATCCCGAACCCAC

GCTACTTGGGCCCTGCAATTTCCTCAGGAGCGATTTACCTGGCGTCCTCATACCAGGATAAATTAAGGGT
TATTTGCTGCAAAGGAAACCTTGTGAAGGAGTCCGGCACTGACCACCACCGGGGCCCCTCCACCTCCCGC
AGCCCCAACAAGCGAGGCCCGCCAACATACAACGAGCACATCACCAAGCGTGTGGCCTCTAGCCCGGCGC
CACCGGAAGGCCCCAGCCACCCACGAGAGCCAAGCACACCCCACCGCTACCGAGAGGGGCGGACAGAGCT
GCGCAGGGACAAGTCTCCTGGCCGCCCCCTGGAGCGAGAGAAGTCCCCAGGCCGGATGCTCAGCACACGG
AGGGAACGCTCACCTGGGAGGCTGTTTGAAGACAGCAGCCGGGGCCGGCTGCCTGCGGGAGCCGTGAGGA
CCCCACTGTCCCAGGTCAACAAGGTCTGGGACCAGTCTTCAGTA
>Erinaceus europaeus XM_007528615.2
ATGCTGAAGTTTAAGTATGGAACACGGAATCCACAGGATGTCGGGGCTGCTGAGCCCATCACTAGCCGAG
CCTCCAGACTGAATCTTTTCTTCCAGGGGAAACCACCCTTCATGACTCAACAGCAGGTGTCTCCTCTCTC
CCGGGAAGGGGTGTTAGACGCCCTCTTTGTTCTCTTTGAAGAGTGCAGTCAGCCTGCGATGATGAAGATG
AAGCACGTGAACAACTTTGTCCGGAAGTATTCTGACACCATAGCTGAGCTTCAGGGGCTCCAGCCCTCGG
CAAAGGATTTTGAAGTCCGTAGTCTTGTAGGCTGTGGTCACTTTGCAGAAGTGCAGGTGGTCAGAGAGAA
AGCCACGGGGGACATCTACGCCATGAAAGTCATGAAGAAGAAGGCCTTGTTGGCCCAGGAGCAGGTCTCA
TTTTTTGAGGAAGAACGGAACATATTATCTAGGAGCACAAGCCCTTGGATCCCCCAGTTACAATACGCTT
TTCAGGACAAGAGTAACCTTTATCTGGTCATGGAATACCAGCCTGGAGGGGACTTGCTGTCACTTTTGAA
TAGATACGAAGACCAATTAGATGAAAATATGATTCAGTTTTACCTTGCCGAGCTGATTTTGGCTGTTCAC
AGCATTCATCAGATGGGATATGTGCATCGAGACATCAAACCAGAGAACATTCTCATTGACCGAACAGGAC
ACCTCAAGCTGGTGGATTTTGGATCAGCTGCTAAGCTCAACTCAAATAAAATGGTGACCGCCAAACTCCC
AATCGGGACGCCAGACTACATGGCCCCTGAAGTGCTGACTGTGATGAACGGGGATGGCAAAGGCGCTGCC
TATGGCCCGGACTGTGACTGGTGGTCGGTGGGCGTGATCACCTATGAGATGATTTATGGCCGGACCCCAT
TCACGGAGGGGACCTCTGCCAGGACCTTTAATAACATCATGAACTTCCAGCGGTTTTTGAAGTTTCCAGA
TGATCCTAAAGTTAGCAGCACATTACTTGATCTGATTCAAAGTTTGTTGTGTGGCCAGAAAGAGAGACTG
AAGTTTGAAGGCCTTTGCTGCCATCCTTTCTTCTCTAAAATCGACTGGAATAACATTCGAAACACTCCTC
CTCCCTTCGTTCCCACCCTCAAGTCTGACGATGACACCTCCAATTTTGATGAACCAGAGAAGAATTTGTG
GGTTTCCTCCTCTGCATGCCAGCTGAGCCCCTCGGGTTTCTCGGGCGAAGAACTGCCGTTTGTGGGCTTT
TCATACAGCAAGGCACTGGGGATTCTTGGTAGATCTGAGTCTGTTGTGTCGGGTCTGGACTCCCCTGCCA
AGACTAGCTCCATGGAAAAGAAACTCCTCATCAAAAGCAAAGAGCTGCAAGACTCCCAGGACAAGTGTCA
CAAGATGGAGCAGGAAATGACCCGGTTACATCGGAGAGTGTCAGAGGTGGAGGCTGTGCTTAGTCAGAAG
GAGGTGGAGCTGAAGGCCTCTGAGACTCAGAGATCCCTCCTGGAGCAGGACCTTGCTACCTACATCACAG
AATGCAGTAGCTTAAAGCGAAGTTTGGAGCAGGCACGGATGGAGGTGTCTCAGGAAGATGACAAAGCACT
GCAGCTCCTCCATGATATCAGGGAGCAGAGCCGGAAGCTCCAGGAAATCAAAGAGCAGGAGTACCAGGCT
CAGGTGGAAGAAATGAGGTTGATGATGAATCAGCTAGAAGAGGACCTCGTTTCGGCCAGAAGACGGAGTG
ATCTCTACGAATCTGAGCTGAGAGAGTCCCGGCTCGCTGCCGAAGAGTTCAAACGGAAAGCGACAGAATG
CCAGCATAAGCTGGTGAAGGCGGCTAAGGATCCAGGGAAACCTGAAGTGGGAGAATATTCCAAGCTGGAG
AAGATCAATGCTGAGCAGCAGATCAAAATCCAGGAGCTCCAAGAGAAGCTAGAAAAGGCCGTCAAAGCCA
GCACGGAGGCCGCTGAGCTGCTGCAGAATATTCGCCAGGCCAAAGAGCGAGCGGAGCGTGAACTGGAGAA
GCTGCAGAACCGGGAGGACTCTTCGGAAGGCATCAAAAAGAAGCTGGTGGAAGCCGAGGAACGCCGCCAT
TCTCTGGAGAACAAGGTAAAGAGGCTAGAGACCATGGAGCGTAGAGAAAACAGACTGAAGGATGACATCC
AGACAAAATCCCAACAGATCCAGCAGATGGCTGATAAAATTCTGGAGCTGGAGGAGAAACACCGAGAGGC
CCAGGTCTCCGCCCAGCACCTAGAGGTGCACCTGAAACAGAAAGAGCAGCACTATGAGGAAAAAATCAAA
GTGTTGGACAGTCAGATAAAGAAAGACCTGGCCGACAAGGAGAGCTTGGAAAACCTGATGCAGAGGCACG
AGGAGGAGGCCCATGAGAAGGGCAAAATTCTCAGCGAGCAGAAGGCGATGATTAATGCAATGGATTCCAA
GATCAGATCCCTGGAGCAGAGGATTGTGGAACTATCAGAAGCCAATAAACTCGCAGCAAACAGCAGTCTT
TTTACCCAGAGGAACATGAAGGCCCAGGAGGAGATGATCTCAGAGCTCAGGCAACAGAAATTCTACCTGG
AGACACAGGCTGGGAAGCTGGAGGCCCAGAACCGGAAGCTAGAGGAACAGCTGGAAAAAATCAGCCACCA
AGACCACAGTGACAAAAGCCGGCTGCTGGAGCTGGAGACGAGGCTGAGGGAGGTCAGTCTGGAGCATGAG

GAGCAGAAGCTGGAGCTGAAGCGCCAGCTCACAGAGCTGCAGCTCTCCTTGCAGGAGCGCGAGTCCCAGC
TGACGGCCCTGCAGTCTGCCCGGGCTGCGCTGGAGGGGCAGCTCCGCCAGGCCAAGACAGAGCTGGAGGA
GACGACAGCAGAAGCAGAGGAGGAGATCCAGGCGCTCACGGCACATAGAGATGAGATCCAGCGCAAGTTT
GATGCCCTTCGTAACAGCTGTACCGTGATCACAGACCTGGAGGAGCAGCTAAACCAGCTGACCGAGGACA
ACGCTGAGCTCAACAACCAAAACTTCTACTTGTCCAAACAACTCGACGAGGCTTCCGGCGCCAACGACGA
GATCGTGCAGCTGCGAAGCGAAGTGGACCACCTCCGCCGGGAGATCACCGAGCGAGAGATGCAGCTCACC
AGCCAGAAGCAAACGATGGAGGCGCTGAAGACCACCTGCACGATGCTGGAGGAGCAGGTCATGGACCTGG
AGGCCCTGAACGATGAGCTGCTGGAGAAGGAGCGGCAGTGGGAGGCCTGGAGGAGTGTCCTGGGTGACGA
GAAGTCCCAGTTTGAGTGCCGGGTTCGAGAGCTGCAGAGGATGCTAGACACCGAGAAGCAGAGCAGGGCG
AGGGCTGACCAGCGCATCACCGAGTCTCGCCAGGTGGTCGAGCTGGCAGTGAAGGAGCACAAAGCCGAGA
TCCTGGCTCTGCAGCAGGCTCTCAAAGAGCAGAAGCTGAAAGCCGAGAGCCTTTCCGACAAGCTTAATGA
CCTGGAGAAGAAGCACGCCATGCTTGAAATGAACGCCCGGAGCTTGCAGCAGAAGCTGGAGACTGAGCGA
GAGCTCAAACAGAGGCTTCTGGAAGAGCAAGCCAAACTACAGCAGCAAATGGACCTACAGAAGAGTCACA
TTTTCCGTCTCACTCAAGGGCTGCAGGAAGCTCTAGACCGGGCTGATCTGCTGAAGACAGAGAGGAGTGA
CCTGGAATATCAGCTTGAAAACATTCAGGTTCTCTATTCTCATGAAAAGGTGAAAATGGAAGGCACTATT
TCTCAACAAACCAAACTCATTGATTTTCTACAAGCCAAAATGGACCAACCCGCCAAAAAGAAAAAGGGTT
TATTTAGTCGACGGAAAGAGGACCCTGCTTTGCCCACACAGGTTCCTCTGCAGTACAATGAGCTGAAGGC
GGCTCTGGAGAAGGAGAAAGCACGCTGTGCAGAGCTTGAGGAAGCCCTGCAGAAGACCCGCATCGAGCTC
CGGTCTGCCCGGGAGGAAGCTGCCCACCGGAAAGCCACAGAACACCCACACCCATCCACGCCAGCCACCG
CGAGGCAGCAGATTGCCATGTCTGCCATCGTGCGGTCCCCCGAGCACCAGCCCAGTGCTATGAGCCTGCT
CGCCCCGCCGTCCGGCCGCAGAAAGGAGTCTTCAACTCCAGAGGAATTCAGCCGGCGTCTCAAGGAGCGG
ATGCACCACAACATTCCTCACCGGTTCAATGTGGGACTGAACATGCGGGCCACCAAGTGCGCCGTGTGCC
TGGACACGGTGCACTTCGGACGCCAGGCCTCCAAATGCCTCGAATGCCAGGTGATGTGTCATCCCAAGTG
CTCCACGTGCTTGCCAGCGACCTGCGGCCTGCCCGCTGAATACGCTACACACTTCACCGAGGCCTTCTGC
CGTGACAAAATGAACTCCCCGGGTCTGCAGACCAAGGAGCCCAGCAGCGGCTTGCACCTGGAAGGGTGGA
TGAAGGTGCCCAGGAACAACAAACGAGGACAGCAAGGCTGGGACAGGAAGTACATCGTCCTGGAGGGCTC
CAAAGTCCTCATCTATGACAGTGAAGCCAGAGAAGCTGGACAGAGGCCGGTGGAAGAATTTGAGCTGTGC
CTTCCCGACGGGGATGTCTCTATTCATGGCGCTGTCGGTGCTTCTGAACTCGCAAACACAGCCAAAGCAG
ATGTTCCATACATACTGAAGATGGAATCCCACCCACACACCACCTGCTGGCCCGGGAGAAGCCTCTACCT
GCTTGCCCCCAGCTTCCCCGACAAACAGCGCTGGGTCACCGCCTTAGAGTCAGTCGTGGCAGGTGGACGA
GTTTCTAGGGAAAAAGCAGAGGCCGACGCTAAACTGCTTGGGAACTCCCTACTGAAACTGGAAGGCGATG
ACCGTTTGGACATGAACTGCACACTGCCCTTCAGCGACCAGGTGGTGTTGGTGGGCACTGAGGAAGGGCT
GTATGCTCTGAATGTCTTGAAAAACTCCCTCACCCACGTCCCAGGGATTGGAGCCGTCTTCCAGATTTAC
ATCATCAAGGACCTGGAGAAGCTACTCATGATAGCAGGAGAGGAGCGGGCCCTGTGTCTTGTTGATGTGA
AGAAAGTCAAACAGTCCCTCGCCCAGTCACACCTGCCAGCTCAGCCTGACATCTCACCCAACGTTTTTGA
AGCTGTCAAGGGCTGCCACTTGTTTGCTGCTGGCAAGATTGAGAGTGGGCTTTGCATCTGTGCGGCCACG
CCCAGCAAAGTCGTCATTCTCCGCTACAATGAAAACCTCAGCAAGTACTGCATTCGGAAAGAGATAGAGA
CCTCAGAGCCCTGCAGCTGCATCCACTTCACCAACTACAGCGTCCTCATCGGGACCAACAAATTCTACGA
AATCGACATGAAACAGTACACGCTTGAGGAATTCCTAGACAAGAACGACCACTCCTTGGCACCTGCGGTG
TTCGCCTCCTCTTCCAACAGCTTCCTGGTCTCCATCGTGCAGGTGAACGGCGCGGGGCAGCGTGAGGAGT
ACCTGCTCTGCTTTCACGAATTTGGGGTGTTTGTGGATTCTTACGGAAGACGCAGCCGCACAGACGATCT
CAAGTGGAGCCGCTTACCTCTGGCCTTCGCCTACAGAGAGCCCTATCTGTTTGTGACCCACTTCAACTCA
CTGGAAGTCATTGAGATCCAGGCCCGCTCCTCTCTGGGGACCCCTGCCCGAGCGTACCTGGAGATCCCCA
ACCCTCGCTACCTGGGCCCTGCCATCTCCTCGGGTGCCATCTACCTGGCATCCTCATACCAGGATAAATT
AAGGATCATCTGCTGCAAGGGGAACCTGGTGAAAGAGTCTGGCACCGACCACCACCGGGGCCCCTCCACC
TCCCGCAGCCCCAACAAGCGTGGCCCTCCCACGTACAACGAGCACATCACCAAGCGTGTGGCCTCCAGCC
CAGCGCCCCCTGAAGGCCCCAGCCACCCGCGAGAGCCAAGCACACCCCACCGCTACCGAGAGGGGCGGAC

GGAGCTGCGGAGGGACAAGTCTCCTGGCCGCCCCCTGGAGCGGGAGAAGTCCCCCGGCCGGGTGCTGAGC
ACGCGCAGGGAGCGCTCCCCCGGGAGGCTGTTTGAGGACAGCAGCAGGGGCCGGCTGCCCGCGGGAGCCG
TCAGGACCCCCCTGTCCCAGGTCAATAAGGTCTGGGACCAGTCTTCGGTA
>Manis javanica XM_017678052.1
ATGCTGAAGTTCAAGTATGGAGCGCGGAATCTGCAGGATGCTGGTGCAGCCGATCCCATTGCCAGCCGGG
CCTCCAGGCTGAATCATTTCTTCCAGGGGAAACCACCCTCCATGACTCAACAGCAGATGTCTCCTCTTTC
CCGAGAAGGGGTATTAGATGCCCTCTTTGTTCTCTTTGAAGAATGCAGTCAGCCTGCTCTGATGAAGATG
AAGCATGTGAGCAACTTTGTCTGCAAGTATTCTGACGCCATAGCTCAGTTACAGGGGCTCCAGCCTTCAG
CAAAGGACTTTGAAGTCAGAAGTCTTGTAGGTTGTGGTCACTTTGCCGAAGTGCGGGTGGTAAGCGAGAG
AGCAACTGGGGACATCTATGCCATGAAAGTCATGAAGAAGAAGGCCTTGCTGGCTCAGGAGCAGGTTTCG
TTTTTTGAGGAGGAACAGAACATATTATCCCAGAGCACAAGTTCCTGGATTCCCCAGTTACAGTATGCCT
TTCAGGACAAAAATAACCTTTATCTGGTCATGGAATATCTGCCTGGAGGGGACTTGCTGTCACTTTTGAA
TAGATATGAGGACCAATTAGATGAGAATATGATTCAGTTTTACCTAGCCGAACTGATTTTGGCTGTCCAC
AGTGTTCATCAGATGGGCTATGTACATCGAGACATCAAGCCTGAGAACATTCTCATTGACCGAACGGGAC
ACATCAAGCTGGTGGATTTTGGATCGGCTGCTAAAATGAACTCAAATAAGATGGTGAATGCCAAACTCCC
GATTGGGACCCCAGATTACATGGCCCCTGAGGTGTTGACCGTGATGAATGGGGATGGAAAAGGTGTCTAC
AGTCTAGACTGTGACTGGTGGTCAGTGGGAGTGATTGCTTATGAGATGGTTTATGGAAGGTCACCATTCA
CCGAAGGAACCTCAGCCAGGACCTTCAATAACATCATGAATTTCCAGCGGTTTTTGAAGTTTCCAGATGA
CCCCAAAGTTAGTAGTGAACTACTTGACCTGATTCAGAGTTTGTTGTGTGGCCAGAAAGAAAGACTGAAG
TTTGAAGGCCTTTGCTGCCATCCTTTCTTCTCTAAAATCGACTGGAATGACATTCATAACTCTCCTCCCC
CCTTCGTTCCCACCCTCAAGTCTGATGATGACACCTCCAATTTTGATGAACCAGAGAAGAATTCGTGGGT
TTCATCCTCTCCGTGCCAGCTGAACCTCTCAGGTTTCTCTGGGGAAGAACTGCCGTTTGTGGGGTTTTCG
TACAGCAAGGCACTGGGGATTCTTGGTAGATCTGAGTCTATTGTGTCAAGTCTGGACTCCCCTGCCAAGA
CTAGCTCCATGGAAAAGAAACTTCTCATCAAAAGCAAAGAACTGCAAGACTCCCAGGACAAGTGTCACAA
GATGGAGCAGGAAATGACCCGGTTACATCGGAGAGTGTCAGAGGTGGAGGCCGTGCTTAGTCAGAAGGAG
GTGGAGCTGAAGGCCTCTGAGACTCAGAGATCCCTCCTGGAGCAGGACCTTGCTACCTACATCACAGAAT
GCAGTAGCTTAAAGCGAAGTTTGGAGCAAGCGCGGATGGAGGTGTCCCAGGAGGATGACAAAGCACTGCA
GCTTCTCCATGATATCAGAGAGCAGAGCCGGAAGCTCCAAGAAATCAAAGAGCAGGAGTACCAGGCTCAA
GTGGAAGAAATGAGATTGATGATGAATCAGTTGGAGGAGGACCTGGTTTCTGCAAGAAGACGGAGCGATC
TCTATGAATCAGAGCTGAGAGAGTCCCGGCTCGCCGCTGAAGAGTTCAAGCGGAAAGCCACAGAATGTCA
GCATAAACTGATGAAGGCTAAGGATCAAGGGAAGCCTGAAGTAGGAGAATATTCCAAACTGGAGAAGATC
AATGCTGAGCAGCAGCTCAAAATTCAGGAACTCCAAGAGAAACTGGAAAAGGCAGTAAAGGCCAGCACAG
AGGCCACGGAGCTGCTGCAGAATATCCGCCAGGCAAAGGAACGAGCTGAGCGTGAGCTAGAGAAGCTGCA
GAACCGCGAAGATTCTTCTGAGGGCATAAAAAAGAAGCTGGCTGAAGCCGAGGAACGCCGCCATTCTCTG
GAGAACAAGGTAAAGAGGCTAGAGACCATGGAGCGTAGAGAAAACAGACTGAAGGATGACATCCAGACAA
AATCCCAACAGATCCAGCAGATGGCTGATAAAATTCTGGAGCTGGAGGAGAAGCATCGGGAGGCCCAGGT
CTCAGCCCAGCACCTGGAGGTGCACCTGAAACAGAAGGAGCAGCACTATGAGGAAAAAATTAAAGTGTTG
GACAACCAGATAAAGAAAGACCTGGCTGATAAGGAGAGTCTGGAGAATCTGATGCAGAGACACGAAGAGG
AGGCCCATGAGAAGGGCAAAATTCTCAGCGAGCAGAAGGCGATGATCAATGCTATGGATTCTAAGATCCG
ATCCCTGGAACAGAGGATCGTTGAACTGTCAGAAGCCAATAAACTTGCAGCAAACAGCAGTCTTTTTACG
CAGAGGAACATGAAGGCCCAAGAAGAGATGATTTCAGAACTCAGGCAACAAAAGTTTTACCTGGAGACGC
AGGCCGGGAAATTGGAGGCCCAGAACCGAAAGCTGGAGGAGCAGCTGGAAAAAATCAGCCACCAAGACCA
CAGCGATAAGAATCGGCTGCTGGAGCTGGAGACAAGGTTGAGGGAGGTTAGTCTCGAGCACGAGGAGCAG
AAACTGGAGCTGAAGCGCCAGCTCACAGAGCTGCAGCTCTCCCTGCAAGAGCGTGAGTCCCAGCTGAGCG
CCCTGCAGGCTGCCCGGGCGGCCCTGGAGAGCCAGCTTCGCCAGGCAAAGACGGAGTTGGAAGAGACGAC
AGCAGAGGCAGAAGAAGAGATCCAGGCGCTCACGGCGCATAGAGATGAAATCCAGCGCAAATTTGATGCC
CTTCGTAACAGCTGTACTGTGATCACAGACCTGGAGGAGCAGCTCAACCAGCTGACCGAGGACAACGCCG

AGCTTAACAACCAAAATTTCTACTTGTCCAAACAACTCGATGAGGCTTCTGGCGCCAATGACGAGATAGT
ACAGCTACGAAGTGAAGTGGACCATCTCCGCCGTGAGATCACGGAGAGAGAGATGCAGCTCACCAGCCAG
AAGCAGACGATGGAGGCGCTGAAGACCACTTGCACAATGCTGGAAGAGCAGGTCATGGACTTGGAGGCCC
TGAACGATGAGCTGCTGGAGAAGGAGCGCCAGTGGGAGGCGTGGAGGAGCGTCCTCGGCGATGAGAAGTC
CCAGTTTGAGTGTCGGGTTCGAGAGTTACAGAGGATGCTGGACACGGAGAAACAGAGCAGGGCAAGAGCT
GATCAGCGGATCACCGAGTCACGCCAGGTGGTAGAGCTGGCAGTGAAGGAACACAAGGCCGAGATTCTTG
CTCTGCAGCAGGCTCTCAAAGAACAGAAGCTAAAAGCTGAGAGCCTCTCTGACAAGCTCAATGACCTGGA
GAAGAAACATGCCATGCTTGAAATGAATGCCCGAAGTTTACAGCAGAAACTGGAGACTGAACGAGAGCTC
AAACAAAGGCTCCTAGAAGAGCAAGCCAAATTACAGCAGCAGATGGACCTGCAGAAGAATCATATTTTCC
GTCTTACTCAAGGGCTGCAAGAAGCTCTAGATCGGGCTGATCTGCTGAAGACAGAAAGGAGTGATCTTGA
ATATCAACTAGAAAACATTCAGGTTCTCTATTCTCATGAGAAGGTGAAAATGGAAGGTACTATCTCTCAA
CAAACCAAACTCATTGATTTTCTGCAAGCGAAAATGGACCAACCTGCTAAAAAGAAAAAGGGTTTATTTA
GTCGACGGAAAGAGGACCCTGCTTTGCCCACACAGGTTCCTCTGCAGTACAATGAGCTGAAGGTGGCTCT
GGAGAAGGAGAAAGCTCGCTGTGCAGAGCTAGAGGAAGCCCTTCAGAAGACCCGCATCGAGCTCCGGTCT
GCCAGGGAGGAAGCTGCCCACCGGAAGGCCACAGACCACCCGCACCCATCTACGCCAGCCACAGCGAGGC
AGCAGATCGCCATGTCCGCCATCGTGCGGTCCCCCGAGCACCAGCCCAGTGCCATGAGCCTGCTGGCCCC
GCCATCCAGCCGCAGAAAGGAGTCTTCAACTCCAGAGGAATTCAGTCGACGTCTTAAGGAGCGCATGCAC
CACAATATTCCTCACCGATTTAACGTAGGACTGAACATGCGAGCCACAAAGTGTGCTGTTTGTCTGGATA
CTGTGCACTTTGGACGCCAGGCATCCAAATGTCTAGAATGTCAGGTGATGTGTCATCCCAAGTGCTCCAC
GTGCTTGCCAGCTACCTGTGGCCTGCCAGCTGAATATGCCACGCACTTCACTGAGGCCTTCTGCCGTGAC
AAAATGAACTCCCCAGGTCTCCAGACCAAGGAGCCCAGCAGTGGCTTGCACCTGGAAGGGTGGATGAAGG
TGCCCAGGAATAACAAACGAGGACAGCAAGGCTGGGACAGGAAGTACATTGTCCTGGAGGGATCGAAAGT
CCTCATTTATGACAGTGAAGCCAGAGAAGCTGGACAGAGGCCGGTGGAAGAATTTGAGCTGTGCCTTCCC
GATGGGGATGTATCTATTCATGGCGCCGTTGGTGCTTCTGAACTTGCAAATACAGCCAAAGCAGATGTCC
CTTACGTACTGAAGATGGAATCTCACCCGCACACCACCTGCTGGCCCGGGAGAACTCTCTACTTGCTGGC
TCCCAGCTTCCCCGACAAACAGCGCTGGGTCACCGCCTTAGAATCAGTTGTCGCAGGTGGGAGAGTTTCT
AGGGAAAAAGCAGAAGCCGATGCTAAATTGCTTGGAAACTCCCTGTTGAAACTGGAAGGTGATGACCGTC
TAGACATGAACTGCACACTGCCCTTCAGCGACCAGGTGGTGTTGGTGGGCACAGAGGAAGGGCTGTACGC
ACTGAATGTCTTGAAAAACTCCCTCACCCATGTCCCAGGAATTGGAGCCGTCTTCCAAATTTATATCATC
AAGGATCTAGAGAAGCTCCTCATGATAGCAGGAGAAGAGCGGGCCCTGTGTCTTATAGACGTGAAGAAAG
TGAAACAGTCTTTAGCACAGTCTCACCTTCCTGCCCAGCCAGACATCTCGCCTAGTGTTTTCGAAGCTGT
CAAGGGCTGCCACTTGTTCGCCGCTGGCAAGATTGAGAATGGGCTGTGCATCTGTGCAGCCATGCCCAGC
AAAGTTGTCGTTCTCCGCTACAATGAAAACCTCAGCAAGTACTGCATTCGGAAGGAGATAGAGACCTCGG
AGCCCTGCAGCTGCATCCACTTCACCAATTACAGTATCCTCATCGGAACCAATAAATTCTACGAAATCGA
CATGAAGCAGTACACGCTGGAGGAATTCCTCGATAAGAACGACCATTCCTTGGCGCCTGCTGTGTTCGCC
TCCTCTTCCAACAGCTTCCCTGTGTCGGTCGTGCAGGTGAACGGTGCAGGGCAGCGGGAGGAGTACCTGC
TCTGCTTCCACGAATTTGGGGTGTTCGTGGATTCTTATGGAAGACGTAGCCGCACAGATGATCTCAAGTG
GAGTCGCTTACCTTTGGCGTTTGCCTACAGAGAACCCTATTTGTTTGTGACCCACTTCAACTCTCTCGAA
GTAATCGAGATCCAGGCACGCTCCTCTCTGGGGACTCCTGCCCGAGCGTATTTGGAAATCCCCAACCCAC
GCTACCTGGGTCCTGCAATTTCCTCAGGAGCGATTTACCTGGCATCCTCATACCAGGATAAATTAAGGGT
CATTTGCTGCAAAGGAAACCTTGTGAAGGAGTCTGGCACTGACCACCACCGGGGCCCCTCCACCTCCCGC
AGCAGCCCCAACAAGCGAGGCCCACCAACGTACAACGAGCACATCACCAAGCGTGTGGCCTCCAGCCCGG
CGCCCCCGGAAGGGCCCAGCCACCCTCGAGAGCCAAGCACACCGCACCGCTACCGCGAGGGGCGGACGGA
GCTGCGCAGGGACAAGTCCCCCGGCCGCCCCCTGGAGCGGGAGAAGTCCCCGGGCCGGATGCTCAGCACG
CGGAGGGAGCGGTCCCCTGGGAGGCTGTTTGAGGACAGCAGCCGGGGCCGGCTGCCTGTAGGAGCTGTGA
GGACCCCCCTGTCCCAGGTCAACAAGGTCTGGGACCAGTCTTCAGTA
>Odobenus rosmarus divergens XM_004396678.1

ATGTTGAAGTTCAAATATGGAGCACGGAATCTGCTGGACGCTGGTGCTGCTGAACCCATTGCCAACCGGG
CCTCCAGGCTGAATCATTTCTTCCAGGGGAAACCACCCTTTCTGACTCAACAGCAGATGTCTCCTCTTTC
CCGAGAAGGGATATTAGATGCCCTCTTCGTTCTCTTCGGAGAATGCAGTCAGCCTGCTCTGATGAAGATC
AAGCACGTGAGCAACTTTGTCCGGAAGTATTCTGACACCATAGCTGAGTTACAGGAGCTCCAGCCTTCAG
CCAAGGACTTTGAAGTCCGAAGTCTTGTGGGTTGTGGTCACTTTGCCGAAGTGCAGGTGGTAAGGGAGCG
AGCGACCGGGGACATCTATGCCATGAAAGTCATGAAGAAGAAGACCTTGTTGGCCCAAGAGCAGGTTTCA
TTTTTTGAGGAAGAACGGAACATATTATCTTGGAGCACAAGCCCTTGGATCCCCCAGTTACAGTATGCCT
TTCAGGACAAAAATAACCTTTATCTGGTTATGGAATATCAGCCTGGAGGGGACTTGCTGTCACTTTTGAA
TAGATATGAGGACCAGTTAGATGAAAATATGATTCAGTTTTACCTAGCCGAACTGATTTTGGCTGTTCAC
AGCATTCATCAGATGGGATATGTGCATCGAGACATCAAGCCTGAGAACATTCTCATTGACCGAACAGGAC
ACATCAAGCTTGTGGATTTTGGATCAGCGGCTAAAATGAACTCAAATAAGATGGTGAATGCCAAACTCCC
GATTGGGACTCCAGATTACATGGCCCCTGAAGTGTTGACCATGATGAATGGGGATGGGAAAGGCACCTAC
AGTCTCGACTGTGACTGGTGGTCGGTGGGAGTGGTCGCCTATGAAATGGTTTATGGAAGGTCCCCGTTCA
CGGAGGGAACCTCAGCCAGAACCTTCAGTAACATCATGAATTTCCAGCGGTTTTTGAAGTTTCCTGATGA
CCCCAAAGTTAGCGGTGAATTACTTGATCTGATTCAGAGTTTGCTGTGTGGCCAGAAAGAGAGACTGAAG
TTTGAAGGCCTTTGCTGCCACCCTTTCTTCTCTAAAATTGACTGGAATAACATCCGTAACTCTCCTCCCC
CCTTCGTTCCCACCCTCAAGTCTGATGATGACACCTCCAATTTTGATGAACCAGAGAAGAATTCGGGGGT
TTCATCCTCTCCGTGCCAGCTGAACCTCTCAGGTTTCTCGGGCGAAGAACTGCCGTTTGTGGGGTTTTCG
TATAGCAAGGCACTGGGGATTCTTGGTAGATCTGAGTCTGTCGTGTCAAGTCTGGACTCCCCTGCCAAGA
CTAGCTCCATGGAAAAGAAACTTCTCATCAAAAGCAAAGAGCTGCAAGACTCCCAGGACAAGTGTCACAA
GATGGAGCAGGAAATGACCCGGTTACATCGGAGAGTGTCAGAGGTGGAGGCTGTGCTTAGTCAGAAGGAG
GTGGAGCTGAAGGCCTCTGAGACTCAGAGATCCCTCCTGGAGCAGGACCTTGCTACCTACATCACAGAAT
GCAGTAGCTTAAAGCGAAGTTTGGAGCAAGCACGGATGGAGGTGTCCCAGGAGGATGACAAAGCACTGCA
GCTTCTCCATGATATCAGAGAGCAGAGCCGGAAGCTCCAGGAAATCAAAGAGCAGGAGTACCAGGCTCAA
GTGGAAGAAATGAGGTTGATGATGAATCAGTTAGAAGAGGACCTTGTTTCGGCAAGGAGACGGAGTGATC
TCTACGAATCGGAGTTGAGAGAGTCTCGGCTCGCTGCTGAAGAGTTCAAGCGGAAAGCGACAGAATGTCA
GCATAAACTAATGAAGGCTAAGGATCAAGGGAAACCTGAAGTGGGAGAATATTCCAAACTGGAGAAGATC
AATGCTGAGCAACAGCTCAAAATTCAGGAGCTCCAAGAGAAGCTGGAAAAGGCTGTAAAAGCCAGCACAG
AGGCCACTGAACTGCTGCAGAATATCCGCCAGGCAAAGGAGCGAGCCGAACGTGAGCTGGAGAAGCTGCA
GAACCGGGAGGACTCCTCTGAAGGCATAAAAAAGAAGCTGGTGGAGGCCGAGGAACGCCGCCATTCTCTG
GAGAACAAGGTAAAGAGGCTAGAGACCATGGAGCGTAGAGAAAACAGACTGAAGGATGACATCCAGACAA
AATCCCAACAGATCCAGCAGATGGCTGATAAAATTCTGGAGCTGGAGGAGAAGCACCGTGAGGCCCAGGT
CTCAGCCCAGCACCTAGAGGTGCACCTGAAACAGAAAGAACAGCACTACGAGGAAAAAATTAAAGTGTTG
GACAACCAGATAAAGAAAGACCTGGCCGATAAAGAGACTCTGGAGAATCTGATGCAGAGACATGAAGAGG
AGGCCCACGAGAAGGGCAAAATTCTCAGCGAGCAGAAGGCGATGATCAATGCTATGGATTCCAAGATCAG
ATCCCTGGAACAGAGGATTGTGGAACTCTCAGAAGCCAACAAACTTGCGGCAAACAGCAGTCTTTTTACC
CAGAGGAACATGAAGGCCCAGGAAGAGATGATTTCTGAGCTCAGGCAACAGAAGTTCTACCTGGAGACAC
AGGCGGGGAAGCTGGAAGCCCAGAACCGGAAGCTGGAGGAACAGCTGGAGAAAATCAGCCACCAAGACCA
CAGTGACAAGAACCGGCTGCTGGAGCTGGAGACAAGGTTGAGGGAGGTCAGCCTAGAGCACGAGGAGCAG
AAACTGGAGCTAAAGCGCCAGCTCACGGAGCTGCAGCTCTCGCTGCAGGAGCGCGAGTCCCAGCTGACGG
CCCTGCAGGCTGCCCGGGCAGCCCTGGAGAGCCAGCTGCGCCAGGCCAAGACCGAACTGGAAGAGACGAC
GGCGGAGGCGGAAGAGGAGATCCAGGCACTCACGGCCCATAGAGATGAAATCCAGCGCAAATTTGATGCC
CTTCGTAACAGCTGTACTGTAATCACAGACCTGGAGGAGCAGCTAAACCAGCTGACCGAGGACAACGCTG
AGCTCAACAACCAAAATTTCTACTTGTCCAAACAACTCGACGAGGCTTCTGGCGCCAACGATGAGATAGT
ACAATTACGAAGTGAGGTAGACCATCTCCGCCGCGAGATTACGGAGAGGGAGATGCAGCTCACGAGCCAG
AAGCAAACGATGGAGGCTCTGAAGACCACTTGCACGATGCTGGAAGAACAGGTCATGGACCTGGAGGCCC
TGAATGATGAGCTGCTGGAAAAAGAGCGGCAGTGGGAGGCGTGGAGGAGCGTCCTTGGCGACGAGAAGTC

CCAGTTTGAGTGTCGGGTCCGAGAGTTACAGAGGATGCTGGACACCGAGAAGCAGAGCAGGGCGCGGGCC
GACCAGCGGATCACCGAGTCCCGCCAGGTGGTGGAGCTGGCGGTGAAGGAACACAAGGCTGAGATCCTGG
CCCTGCAGCAGGCCCTCAAAGAGCAGAAGCTGAAAGCTGAGAGCCTCTCTGACAAGCTTAATGACCTGGA
GAAGAAACATGCCATGCTTGAAATGAACGCCCGCAGTTTACAGCAGAAACTGGAAACTGAACGGGAGCTC
AAACAGAGGCTTCTGGAAGAGCAAGCCAAATTACAGCAGCAGATGGACCTGCAGAAGAATCACATCTTCC
GTTTGACTCAAGGGCTGCAAGAAGCTCTGGACCGGGCTGATCTGCTGAAGACCGAAAGGAGCGATCTGGA
ATACCAGCTAGAAAACATTCAGGTTCTCTATTCTCACGAAAAGGTGAAAATGGAAGGTACTATTTCTCAA
CAAACCAAACTCATTGACTTTCTGCAAGCCAAAATGGACCAGCCTGCTAAGAAGAAAAAGGGTTTATTTA
GTCGACGGAAAGAGGACCCTGCTTTGCCCACACAGGTTCCTCTGCAGTACAATGAGCTGAAGGTGGCCCT
GGAGAAGGAGAAAGCTCGCTGTGCAGAGCTAGAGGAAGCCCTTCAGAAGACCCGCATCGAGCTCCGGTCT
GCCCGGGAGGAAGCTGCCCACCGGAAGGCCACAGACCATCCGCACCCGTCCACGCCAGCCGCCGCGAGGC
AGCAGATCGCCATGTCCGCTATCGTGCGGTCACCCGAGCACCAGCCCAGTGCCATGAGCCTGCTCGCCCC
GCCGTCCAGCCGCAGAAAGGAGTCGTCAACTCCAGAGGAATTCAGTCGGCGTCTTAAGGAGCGCATGCAC
CACAATATTCCTCACCGATTTAACGTAGGATTGAACATGCGAGCCACAAAGTGTGCTGTGTGTCTGGATA
CTGTGCACTTTGGGCGCCAGGCATCCAAATGTCTTGAATGTCAGGTGATGTGTCATCCCAAGTGCTCCAC
GTGCTTGCCAGCCACCTGTGGCCTGCCAGCCGAGTATGCCACACACTTCACCGAGGCCTTCTGCCGTGAC
AAAATGAACTCTCCAGGTCTCCAGACCAAGGAGCCCAGCAGCGGCTTGCACCTGGAAGGGTGGATGAAGG
TGCCCAGGAATAACAAACGAGGACAGCAAGGCTGGGACCGGAAGTACATTGTCCTGGAGGGATCCAAAGT
CCTCATTTATGACAATGAAGCCAGAGAAGCTGGACAGAGGCCGGTGGAAGAATTTGAGCTGTGCCTTCCC
GACGGGGATGTATCTATTCATGGCGCCGTTGGTGCTTCTGAGCTTGCAAATACAGCCAAAGCAGATGTCC
CCTACATACTGAAGATGGAGTCTCACCCGCACACCACCTGCTGGCCCGGGAGAACGCTCTACTTGCTGGC
TCCCAGCTTCCCCGACAAACAGCGCTGGGTCACTGCCTTAGAGTCTGTTGTCGCAGGTGGGAGAGTTTCT
AGGGAAAAAGCAGAAGCCGATGCTAAATTGCTTGGAAACTCCCTGCTGAAACTGGAAGGTGATGACCGTC
TGGACATGAACTGCACGCTGCCCTTCAGCGACCAGGTGGTGTTGGTGGGCACTGAGGAAGGGCTCTACGC
ACTGAATGTCTTGAAAAACTCCCTCACCCATGTCCCCGGAATTGGAGCGGTCTTCCAAATTTATATCATC
AAGGACCTGGAGAAGCTCCTCATGATAGCAGGAGAAGAGCGGGCTCTGTGCCTTGTTGACGTGAAGAAAG
TGAAGCAGTCCCTCGCGCAAGCTCACCTTCCCGCCCAGCCCGACATCTCCCCCAACGTTTTCGAAGCCGT
GAAGGGCTGCCACTTGTTTGCCGCCGGCAAGATTGAGAACGGGCTCTGCATCTGTGCAGCCATGCCCAGC
AAAGTTGTCATTCTCCGCTACAACGAAAACCTCAGCAAGTACTGCATTCGGAAAGAGATAGAGACCTCAG
AGCCCTGCAGCTGCATCCACTTCACCAATTACAGTATCCTCATCGGAACCAATAAATTCTACGAAATTGA
CATGAAGCAGTACACGCTTGAGGAATTCCTGGATAAGAATGACCATTCCTTGGCGCCTGCTGTGTTTGCC
TCCTCTTCCAACAGTTTCCCTGTCTCGATCGTGCAGGTGAACGGTGCAGGGCAGCGGGAGGAATACCTGC
TCTGCTTCCACGAATTTGGGGTGTTCGTGGATTCTTACGGCAGACGTAGCCGCACAGACGATCTCAAGTG
GAGTCGCTTACCTTTGGCCTTTGCCTACAGAGAACCCTATCTGTTTGTGACCCACTTCAACTCACTCGAA
GTCATTGAGATCCAGGCACGCTCCTCTCTGGGGACTCCTGCCCGAGCGTATTTGGAAATCCCGAACCCAC
GCTACTTGGGCCCTGCAATTTCCTCAGGAGCGATTTACCTGGCGTCCTCATACCAGGATAAATTACGGGT
CATTTGCTGCAAAGGAAACCTCGTGAAGGAGTCTGGCACTGACCACCACCGGGGCCCCCCCACCTCCCGC
AGTAGCCCCAATAAGCGAGGCCCGCCAACTTACAACGAGCACATCACCAAGCGTGTGGCCTCTAGCCCGG
CGCCACCGGAAGGCGCCAGCCACCCGCGAGAGCCAAGCACACCGCACCGCTACCGGGAGGGGCGGACGGA
GCTGCGCAGGGACAAGTCTCCTGGCCGCCCCCTGGAGCGGGAGAAGTCCCCAGGCCGGATGCTCAGCACG
CGGAGGGAACGCTCCCCTGGGAGGCTGTTTGAAGACAGCAGCCGGGGCCGGCTGCCTGTGGGAGCCGTGA
GGACCCCACTGTCCCAGGTCAACAAGGTCTGGGACCAGTCTTCAGTG
>Loxodonta africana XM_010598933.1
ATGTTGAAGTTCAAGTATGGAACGCGGAGTGCGCTGGATGCTGGTGCTGCCGAACCCATTGCCACCCGGG
CCTCCAAGCTGAATCTTTTCTTCCAGGGGAAACCTGCCTTTATGACTCAACAGCAGATGTCCCCTCTTTC
CCGAGAAGGGATATTAGATGCCCTCTTTGTTCTCGTTGAAGAATGTAGTCAGCCTGCTCTGATGAAGATG
AAGCACGTGAGCAACTTTGTCCGCAAGTATTCTGACACTATAGCCGAGTTACAGGAACTCCAGCCTTCCA

CCAAGGACTTTGAAGTCAAAAGCCTGGTAGGTTGTGGTCACTTTGCCTACGTGCAGGTGGTCAGAGAGAA
AGCCACGGGGGACATCTATGCCATGAAAGTGATGAAGAAGAAGGCCTTGTTGGCCCAGGAGCAGGTCTCA
TTTTTTGAGGAAGAGCGGAACATATTATCTCGGAGCACAAGTCCTTGGATCCCGCAATTGCAGTATGCCT
TTCAGGACAAAAATAGCCTTTATCTGGTCATGGAATATCAGCCCGGAGGTGACTTGCTGTCACTCTTGAA
TAGATATGAGGACCAATTAGATGAAAACATGATTCAGTTTTACCTAGCCGAACTGATTTTGGCTGTTCAC
AGCGTTCACCAGATGGGATATGTACATCGAGACGTCAAGCCTGAGAATATTCTTATTGACCGAACAGGGC
ATATTAAGCTGGTGGATTTTGGATCAGCGGCTAAAATGAACTCAAATAAGATGGTGAATGCAAAACTCCC
GATTGGGACCCCGGATTACATGGCCCCTGAAGTGTTGACTGTGATGAATGGGGATGGAAAAGGTGTCTAC
GGCCTGGAGTGCGATTGGTGGTCTGTGGGAGTGATCGCTTATGAGATGATTTATGGAGATCCCCGTTCA
CTGAGGGAACCTCAGCCAGAACGTTCAGTAACATCATGAATTTCCAGCGGTTTTTGAAGTTTCCAGGTGA
CCCCAAAGTTAGCAATGAATGTCTTGATCTGATTCAGAGTTTGTTGTGTGGGCAGAAAGAGAGATTGAAT
TTTGAAGGCCTTTGCTGCCATCCTTTCTTCGCTAAAGTTGACTGGAAGGACATTCGTAACTCTCCTCCTC
CCTTCGTTCCCACCCTCAAGTCTGATGATGACACCTCCAATTTTGATGAACCAGAGAAGAATTCGTGGGT
TTCATCCTCTCCGTGCCAGTTGAACCCCTCAGGTTTCTCTGGTGAAGAGCTGCCGTTTGTGGGATTTTCA
TACAGCAAGGCACTGGGGATTCTTGGTAGATCTGAGTCTGTTGCGTCGGGTCTGGACTCCCCTGCCAAGA
CTAGCTCCATGGAAAAGAAACTTCTCATCAAAAGCAAAGAGCTGCAAGACTCTCAGGACAAGTGTCACAA
GATGGAGCAGGAAATGACCCGGTTACATCGGAGAGTGTCTGAGGTGGAGGCTGTGCTTAGTCAGAAGGAG
GTGGAGCTGAAGGCCTCTGAGACTCAGAGATCCCTCCTGGAGCAGGACCTTGCTACCTACATCACAGAAT
GCAGTAGCTTAAAGCGAAGTTTGGAGCAAGCACGGATGGAAGTGTCTCAGGAGGATGACAAAGCACTGCA
GCTCCTCCACGATATCAGAGAGCAGAGCCGGAAGCTCCAAGAAATCAAAGAGCAGGAGTACCAGGCTCAA
GTGGAAGAAATGAGATTAATGATGAATCAGCTGGAAGAAGACCTTGTCTCGGCAAGAAGACGGAGTGATC
TCTACGAATCAGAGCTGAGAGAGTCTCGGCTCGCTGCTGAGGAATTCAAGCGGAAAGCAACAGAAAGTCA
GCATAAACTTATGAAGGCTAAGGATCAAGGGAAGCCTGAAGTGGGAGAATATGCCAAACTGGAGAAGATC
AATGCTGAGCAGCAGCTCAAAATTCAGGAGCTCCAAGAGAAACTGGAAAAGGCTGTAAAAGCCAGCACAG
AGGCCACCGAGCTACTTCAGAATATCCGCCACGCAAAGGAGCGAGCGGAGCGGGAGCTGGAGAAGCTTCA
GAACCGAGAGGATTCTTCTGAAGGCATAAAAAAGAAGCTGGTGGAGGCTGAGGAACGCCGACATTCTCTG
GAGAACAAGGTAAAGAGGCTAGAGACCATGGAACGTAGAGAAAACAGACTGAAGGATGACATCCAGACAA
AATCCCAACAGATCCAGCAGATGGCTGATAAAATTCTGGAGCTGGAGGAGAAGCACCGGGAGGCCCAGGT
TTCAGCCCAGCACCTAGAGGTGCACCTGAAACAGAAAGAGCAGCACTACGAGGAAAAAATTAAAGTATTA
GATAATCAGATAAAGAAAGACCTGGCTGATAAGGAGACTCTGGAGAACCTGATGCAGAGACACGAAGAGG
AAGCCCATGAGAAGGGCAAAATTCTCAGCGAGCAGAAGGCGATGATCAATGCTATGGATTCCAAGATCAG
ATCCCTGGAACAGAGGATTGTGGAACTGTCTGAAGCCAATAAGCTTGCCGCAAACAGCAGTCTTTTTACC
CAGAGGAATATGAAGGCTCAGGAGGAGATGATTTCAGAGCTCAGGCAACAGAAGTTTTATCTGGAGACGC
AGGCTGGGAAGTTGGAGGCCCAGAACCGAAAGCTGGAGGAGCAACTGGAAAAAATCAGCCACCAGGACCA
CAGCGACAAGAGTCGACTGCTTGAGCTGGAGACACGGCTGAGGGAGGTCAGTTTAGAGCATGAGGAGCAG
AAACTGGAGCTAAAGCGCCAGCTCACAGAGTTGCAGCTCTCCCTTCAAGAGCGTGAGTCCCAGCTCACGG
CCCTCCAGGCCGCCCGGGCAGCCCTGGAGAGCCAGCTTCGCCAGGCGAAGACAGAGCTGGAGGAGACGAC
AGCAGAAGCAGAAGAAGAAATCCAGGCGCTCACGGCACATAGAGATGAAATCCAGCGCAAATTTGATGCT
CTCCGTAACAGCTGTACTGTAATCACAGACCTGGAGGAGCAGCTGACTCAGCTGACAGAGGACAACGCTG
AGCTCAACAACCAAAACTTCTACCTGTCCAAACAACTTGACGAGGCTTCTGGCGCCAACGATGAGATAGC
GCAGCTCCGAAGTGAAGTGGACCATCTCCGCCGTGAAATCACCGAGCGGGAGATGCAGCTTACCAGCCAG
AAGCAAACGATGGAGGCCCTGAAAACCACGTGCACGATGCTGGAGGAACAGGTCATGGATCTGGAAGCCC
TGAACGATGAGTTGCTGGAAAAGGAGCGGCAGTGGGAGGCGTGGAGGAGCGTCCTTGGTGATGAGAAATC
CCAGTTTGAGTGTCGGGTTCGAGAGTTACAGAGGATGTTGGACACCGAAAAGCAGAGCAGGGTGAGAGCT
GACCAGCGGATCACCGAGTCACGCCAAGTGGTGGAGCTGGCGGTGAAGGAGCACAAGGCTGAGATCCTTG
CTCTGCAGCAGGCTCTCAAAGAGCAGAAGCTGAAAGCTGAGAGCCTCTCTGACAAGCTGAATGACCTGGA
AAAGAAGCACGCCATGCTTGAGATGAATGCGCGAAGCTTACAGCAGAAGCTAGAGACCGAGCGAGAACTC

AAACAAAGGCTCCTGGAAGAGCAAGCTAAGTTACAGCAGCAGATGGACCTGCAGAAGAACCACATTTTCC
GCCTGACTCAAGGATTGCAGGAAGCTCTAGACCGGGCTGATTTGCTGAAGACAGAGAGGAGTGATTTGGA
GTATCAGCTGGAAAACATTCAGGTTCTCTATTCTCACGAAAAGGTGAAAATGGAAGGCACTATTTCTCAA
CAAACCAAGCTCATCGATTTTCTGCAAGCCAAAATGGACCAACCTGCCAAAAAGAAAAAGGGTTTATTTA
GTCGACGGAAAGAGGACCCTGCTTTGCCCACACAGGTTCCTCTGCAGTACAATGAGCTGAAGGTGGCCCT
GGAGAAGGAGAAAGCTCGCTGTGCAGAGTTAGAGGAAGCCCTTCAGAAGACCCGCATTGAGCTCCGGTCT
GCCCGAGAGGAAGCTGCCCACCGAAAAGCCACAGACCACCCCCACCCATCTACGCCAGCTACCGCGAGGC
AGCAGATCGCAATGTCTGCCATCGTGCGGTCACCCGAGCACCAGCCTAGTGCCATGAGCCTGCTCGCCCC
ACCTTCCAGCCGCAGAAAAGAGTCTTCGACTCCAGAAGAATTCAGTCGGCGTCTTAAGGAGCGGATGCAC
CACAATATTCCTCACCGATTCAACGTGGGACTGAACATGCGAGCCACAAAGTGCGCTGTGTGTCTGGATA
CTGTGCACTTTGGACGCCAGGCATCCAAATGTCTCGAATGCCAGGTGATGTGTCATCCCAAGTGCTCCAC
GTGCTTGCCAGCTACCTGCGGCCTGCCCGCCGAATACGCCACGCACTTCACTGAGGCCTTCTGCCGTGAC
AAAATGAACTCCCCAGGTCTCCAGACCAAGGAGCCCAGCAGCAGCTTGCACCTAGAGGGGTGGATGAAGG
TGCCCAGGAATAATAAGCGAGGACAGCAAGGCTGGGACAGGAAGTACCTTGTCCTGGAAGGATCTAAAGT
CCTCATTTATGACAATGAAGCCAGAGAAGCTGGACAGAGGCCGGTGGAAGAATTTGAGCTGTGCCTTCCC
GACGGGGACGTATCTATTCATGGCGCCGTTGGTGCTTCCGAACTCGCAAATACAGCCAAAGCAGATGTCC
CATACATACTGAAGATGGAATCTCACCCGCATACCACCTGCTGGCCCGGGAGAACACTCTACTTGCTAGC
TCCCAGCTTCCCCGACAAACAGCGCTGGGTCACTGCCTTAGAGTCAGTGGTCGCAGGTGGGAGAGTGTCT
AGGGAAAAAGCAGAAGCCGACGCTAAATTGCTTGGAAACTCTCTGCTGAAACTGGAGGGTGATGAGCGTC
TCGACATGAACTGCACGCTGCCCTTCAGTGATCAGGTGGTGTTGGTGGGCACTGAGGAAGGGCTGTATGC
ACTGAATGTCTTGAAAAACTCCCTAACCCACGTCCCAGGCATTGGAGCCGTCTTCCAGATTTATATCATC
AAGGACCTGGAGAAGCTACTCATGATAGCAGGGGAAGAACGGGCCCTGTGTCTCGTGGAGGTGAAGAAGG
TGAAGCAGTCCCTGGCACAGTCTCACCTTCCTGCCCAGCCAGACATCTCACCCAACGTTTTCGAAGCCGT
CAAGGGCTGCCACTTGTTTGCTGCTGGCAAGATTGATAACGGGCTCTGCATTTGTGCAGCCATGCCCAGC
AAAGTTGTGATTCTCCGCTACAACGAGAACCTCAGCAAGTACTGTATCCGGAAGGAGATAGAGACCTCAG
AGCCCTGTAGCTGTATCCACTTCACCAATTATAGCATCCTCATTGGCACCAATAAATTCTACGAAATTGA
CATGAAGCAGTACACGCTCGAGGAATTCCTGGATAAGAATGACCATTCCTTGGCGCCTGCTGTGTTTGCC
TCCTCTTCCAACAGCTTCCCGGTCTCCATCGTGCAGGTGAACAGCGCTGGGCAGCGGGAGGAGTACCTGC
TCTGTTTCCACGAATTTGGGGTGTTCGTGGATTCTTACGGAAGACGTAGCCGCACAGATGATCTCAAGTG
GAGTCGCTTGCCTTTGGCCTTTGCTTACAGAGAACCTTATCTGTTTGTGACCCACTTCAACTCGCTCGAA
GTAATTGAGATCCAGGCACGTTCCTCTCTGGGGACCCCTGCCCGAGCGTATTTGGAAATCCCGAATCCAC
GCTACCTGGGCCCTGCAATTTCATCGGGAGCAATTTACCTGGCTTCCTCATACCAGGATAAATTAAGGGT
CATTTGCTGCAAAGGAAACCTTGTGAAGGAGTCCGGCACTGACCACCACCGGGTCCCCTCCACCTCCCGC
AGCCCCAACAAGCGAGGCCCACCGACATACAACGAGCACATCACCAAGCGCGTGGCCTCTAGCCCGGCGC
CCCCCGAAGGCCCCAGCCACCCCCGAGAGCCGAGCACACCCCACCGCTACCGGGAGGGGCGGACAGAGCT
GCGCAGGGACAAGTCCCCGGGCCGACCCCTGGAACGAGAGAAATCTCCGGGCCGGGTGCTCAGCACACGG
AGGGAGCGGTCCCCCGGGAGGCTATTTGAAGACAGCAGCAGGGGCCGGCTGCCCGTGGGGGCTGTGAGAA
CCCCGCTGTCCCAGGTCAATAAGGTCTGGGACCAGTCTTCAGTA

KIF14

KIF14

>human ENST00000367350.4
ATGTCATTACACAGTACTCATAATAGAAATAACAGCGGTGATATTCTTGATATTCCTTCT
TCCCAAAATAGTTCATCACTGAATGCCCTCACCCACAGTAGCCGACTTAAGCTGCATTTG
AAGTCGGATATGTCAGAATGTGAAAATGATGATCCATTATTGAGATCTGCAGGTAAAGTC
AGAGACATAAATAGAACTTATGTTATTTCTGCCAGTAGAAAAACAGCAGACATGCCCCTT
ACCCCTAATCCTGTAGGTAGATTGGCACTTCAGAGGAGAACTACAAGGAACAAAGAATCA

TCTTTGCTTGTTAGTGAGTTGGAAGACACAACTGAAAAAACAGCAGAAACACGTCTTACA
TTACAACGTCGTGCTAAAACAGATTCTGCAGAAAAGTGGAAAACAGCTGAAATAGATTCT
GTCAAAATGACACTGAATGTGGGAGGTGAAACAGAAAATAATGGTGTTTCTAAGGAAAGT
AGAACAAATGTAAGGATTGTAAATAATGCTAAAAACTCTTTTGTTGCCTCTTCTGTACCT
TTAGATGAAGATCCACAGGTCATTGAAATGATGGCTGATAAGAAATACAAAGAAACATTT
TCTGCCCCCAGTAGAGCAAATGAAAATGTTGCACTTAAGTACTCAAGTAATAGACCACCC
ATTGCTTCCCTGAGTCAGACTGAAGTTGTTAGATCAGGACACTTGACAACGAAACCTACT
CAGAGCAAGTTGGATATCAAAGTGTTGGGAACAGGAAACTTGTATCATAGAAGTATTGGG
AAGGAAATTGCAAAAACTTCAAATAAATTTGGGAGCTTAGAAAAAAGAACACCTACAAAA
TGTACAACAGAACACAAACTGACAACAAAGTGCAGCCTGCCTCAGCTTAAGAGCCCAGCT
CCATCAATACTGAAGAATAGAATGTCTAACCTTCAAGTTAAACAAAGACCAAAAAGTTCC
TTTCTTGCAAATAAACAGGAAAGATCCGCAGAAAATACAATTCTTCCCGAAGAAGAAACT
GTAGTTCAGAACACCTCTGCAGGAAAAGACCCCTTAAAAGTAGAGAATAGTCAAGTGACA
GTGGCAGTACGCGTAAGACCTTTCACCAAGAGAGAGAAGATTGAAAAAGCATCCCAGGTA
GTCTTCATGAGTGGGAAAGAAATAACTGTGGAACACCCTGACACGAAACAAGTTTATAAT
TTTATTTATGATGTTTCATTCTGGTCTTTTGATGAATGTCATCCTCACTACGCTAGCCAG
ACAACTGTCTATGAGAAGCTAGCAGCACCACTCCTAGAAAGAGCCTTCGAAGGCTTCAAT
ACCTGTCTTTTTGCTTATGGTCAGACTGGCTCTGGAAAATCATATACGATGATGGGATTT
AGTGAAGAACCAGGAATAATTCCAAGATTTTGTGAAGATCTTTTTTCTCAAGTAGCCAGA
AAACAAACCCAAGAGGTCAGCTATCACATTGAAATGAGCTTCTTTGAAGTATATAATGAA
AAAATTCACGACCTTCTGGTTTGTAAAGATGAAAATGGGCAGAGAAAGCAACCACTGAGA
GTGAGGGAACATCCTGTTTATGGACCATATGTTGAAGCACTGTCAATGAACATTGTCAGT
TCTTACGCTGATATCCAGAGTTGGCTAGAATTGGGAAATAAACAAAGAGCTACTGCTGCT
ACTGGTATGAATGATAAAAGTTCCCGATCTCATTCAGTTTTCACCCTGGTGATGACCCAG
ACCAAGACAGAATTTGTGGAAGGGGAAGAACACGATCACAGAATAACAAGTCGAATTAAC
CTAATAGATCTGGCAGGCAGTGAGCGCTGCTCTACGGCTCACACTAATGGAGATCGACTA
AAGGAAGGTGTGAGTATTAATAAGTCCTTGCTAACTTTGGGAAAAGTTATATCTGCACTT
TCGGAACAAGCAAACCAAAGGAGTGTTTTTATTCCTTATCGTGAATCTGTTCTTACATGG
CTGTTAAAAGAAAGTCTGGGTGGAAATTCAAAAACTGCAATGATTGCTACGATTAGTCCC
GCTGCCAGCAACATAGAAGAAACATTAAGCACACTTAGATATGCTAACCAAGCCCGTTTA
ATAGTCAACATTGCTAAAGTAAATGAAGATATGAACGCTAAGTTAATTAGAGAATTGAAG
GCAGAAATTGCAAAGCTAAAAGCTGCTCAGAGAAACAGTCGGAATATTGACCCTGAACGA
TACAGGCTCTGTCGGCAAGAAATAACATCCTTAAGAATGAAACTGCATCAACAGGAGAGA
GACATGGCAGAAATGCAAAGAGTGTGGAAAGAAAAGTTTGAACAAGCTGAAAAAAGAAAA
CTTCAAGAAACAAAAGAGTTACAGAAAGCAGGAATTATGTTTCAAATGGACAATCATTTA
CCAAACCTTGTTAATCTGAATGAAGATCCACAACTATCTGAGATGCTGCTATATATGATA
AAAGAAGGAACAACTACAGTTGGAAAGTATAAACCAAACTCAAGCCATGATATTCAGTTA
TCTGGGGTGCTGATTGCTGATGATCATTGTACTATCAAAAATTTTGGTGGGACAGTGAGT
ATTATCCCAGTTGGGGAAGCAAAGACATATGTAAATGGAAAACATATTTTGGAAATCACA
GTATTACGTCATGGTGATCGAGTGATTCTTGGTGGAGATCATTATTTTAGATTTAATCAT
CCAGTAGAAGTCCAGAAAGGAAAAAGGCCATCTGGAAGAGATACTCCTATAAGTGAGGGT
CCAAAAGACTTTGAATTTGCAAAAAATGAGTTGCTCATGGCACAGAGATCACAACTTGAA
GCAGAAATAAAAGAGGCTCAGTTGAAGGCAAAGGAAGAAATGATGCAAGGAATCCAGATT
GCAAAAGAAATGGCTCAGCAAGAGCTTTCTTCTCAAAAAGCTGCATATGAAAGCAAAATA
AAAGCACTGGAAGCAGAACTGAGAGAAGAGTCTCAAAGGAAAAAAATGCAGGAAATAAAT
AACCAGAAGGCTAATCACAAAATTGAGGAATTAGAAAAGGCAAAGCAGCATCTTGAACAG
GAAATATATGTCAACAAAAAGCGATTAGAAATGGAAACATTGGCTACAAAACAGGCTTTA

GAAGACCATAGCATCCGCCATGCAAGAATTCTGGAAGCTTTAGAAACTGAAAAGCAAAAA
ATTGCTAAAGAAGTACAAATTCTACAGCAGAATCGGAATAATAGGGATAAAACTTTTACA
GTGCAGACAACTTGGAGCTCTATGAAACTCTCAATGATGATTCAGGAAGCCAATGCTATC
AGCAGCAAATTGAAAACATACTATGTTTTTGGCAGACATGATATATCAGATAAAAGTAGT
TCTGACACTTCTATTCGGGTTCGTAACCTGAAACTAGGAATCTCAACATTCTGGAGTCTG
GAAAAGTTTGAATCTAAACTTGCAGCAATGAAAGAACTTTATGAGAGTAATGGTAGTAAC
AGGGGTGAAGATGCCTTTTGTGATCCTGAAGATGAATGGGAACCCGACATTACAGATGCA
CCAGTTTCTTCACTTTCTAGAAGGAGGAGTAGGAGTTTGATGAAGAACAGAAGAATTTCT
GGTTGTTTACATGACATACAAGTCCATCCAATTAAGAATTTGCATTCTTCACATTCATCA
GGTTTAATGGACAAATCAAGCACTATTTACTCAAATTCAGCAGAGTCCTTTCTTCCTGGA
ATTTGCAAAGAATTGATTGGTTCTTCGTTAGATTTTTTTGGACAGAGTTATGATGAAGAA
AGAACTATAGCAGACAGCCTAATTAATAGTTTTCTTAAAATTTATAATGGGCTATTTGCC
ATTTCCAAGGCTCATGAAGAACAAGATGAAGAAAGTCAAGATAACTTGTTTTCTTCTGAT
CGAGCAATCCAGTCACTTACTATTCAGACTGCATGTGCTTTTGAGCAGCTAGTAGTGCTA
ATGAAACACTGGCTGAGTGATTTACTGCCTTGTACCAACATAGCAAGACTTGAGGATGAG
TTGAGACAAGAAGTTAAAAAACTGGGAGGCTACTTACAGTTATTTTTGCAGGGATGCTGT
TTGGATATTTCATCAATGATAAAAGAGGCTCAAAAGAATGCAATCCAAATTGTACAACAA
GCTGTAAAGTATGTGGGGCAGTTAGCAGTTCTGAAAGGGAGCAAGCTACATTTTCTAGAA
AACGGTAACAATAAAGCTGCCAGTGTCCAGGAGGAATTCATGGATGCTGTTTGTGATGGT
GTAGGCTTAGGAATGAAGATTTTATTAGATTCTGGACTGGAAAAAGCAAAAGAACTTCAG
CATGAACTCTTTAGGCAGTGTACAAAAAATGAGGTTACCAAAGAAATGAAAACTAATGCC
ATGGGATTGATTAGATCTCTTGAAAACATCTTTGCTGAATCGAAAATTAAAAGTTTCAGA
AGGCAAGTACAAGAAGAAAACTTTGAATACCAAGATTTCAAGAGGATGGTTAATCGTGCT
CCAGAATTCTTAAAGTTAAAACATTGCTTAGAGAAAGCTATTGAAATTATTATTTCTGCA
CTGAAAGGATGCCATAGTGATATAAATCTTCTCCAGACTTGTGTTGAAAGTATTCGCAAC
TTGGCCAGTGATTTTTACAGTGACTTCAGTGTGCCTTCTACTTCTGTTGGCAGCTATGAG
AGTAGAGTAACTCACATTGTCCACCAGGAACTAGAATCTCTAGCTAAGTCTCTCCTCTTT
TGTTTTGAATCTGAAGAAAGCCCTGATTTGTTGAAACCCTGGGAAACTTATAATCAAAAT
ACCAAAGAAGAACACCAACAATCTAAATCAAGCGGGATTGACGGCAGTAAGAATAAAGGT
GTACCAAAGCGTGTCTATGAGCTCCATGGCTCATCCCCAGCAGTGAGCTCAGAGGAATGC
ACACCCAGTAGGATTCAGTGGGTG
>chimpanzee ENSPTRT00000003304.5
ATGTCATTACACAGTACTCATAATAGAAATAACAGCGGTGATATTCTTGATATTCCTTCT
TCCCAGAATAGTTCATCACTGAATGCCCTCACCCACAGTAGCCGACTTAAGCTGCATTTG
AAGTCGGATATGTCAGAATGTGAAAATGATGATCCATTATTGAGATCTGCAGGTAAAGTC
AGAGACATAAATAGAACTTATGTTATTTCTGCCAGTAAAAAAACAGCAGACATGCCCCTT
ACCCCTAATCCTGTCGGTAGATTGGCACTTCAGAGGAGAACTACAAGGAACAAAGAATCA
TCTTTGCTTGTTAGTGAGTTGGAAGACACAACTGAAAAAACAGCAGAAACACGTCTTACA
TTACAACGTCGTGCTAAAACAGATTCTGCAGAAAAGTGGAAAACAGCTGAAATAGATTCT
GTCAAAATGACACTGAATGTGGGAGGTGAAACAGAAAATAATGGTGTTTCTAAGGAAAGT
AGAACAAATGTAAGGATTGTAAATAATGCTAAAAACTCTTTTGTTGCCTCTTCTGTACCT
TTAGATGAAGATCCACAGGTCATTGAAATGATGGCTGATAAGAAATACAAAGAAACATTT
TCTGCCCCCAATAGAGCAAATCAAAATGTTGCACTTAAGTACTCAAGTAATAGACCACCC
ATTGCTTCCCTGAGTCAGACTGAAGTTGTTAGATCAGGACACTTGACAACGAAACCTACT
CAGAGCAAGTTGGATATCAAAGTGTTGGGAACAGGAAACTTGTATCATAGAAGTATTGGG
AAGGAAATTGCAAAAACTTCAAATAAATTTGGGAGCTTAGAAAAAAGAACACCTACAAAA
TGTACAACAGAACACAAATTGACACCAAAGTGCAGCCTGCCTCAGCTTAAGAGCCCAGCT

CCATCAATACTGAAGAATAGAATGTCTAACCTTCAAGTTAAACAAAGACCAAAAAGTTCC
CTTCTTGCAAATAAACAGGAAAGGTCCGCAGAAAATACAATTCTTCCCGAAGAACAAACT
GTAGTTCAGAACACCTCTGCAGGAAAAGACCCCTTAAAAGTAGAGAATAGTCAAGTGACA
GTGGCAGTACGCGTAAGACCTTTCACCAAGAGAGAGAAGATCGAAAAAGCATCCCAGGTA
GTCTTCATGAGTGGGAAAGAAATAACTGTGGAACACCCTGACATGAAACAAGTTTATAAT
TTTATTTATGATGTTTCATTCTGGTCTTTTGATGAATGTCATCCTCACTACGCTAGCCAG
ACAACTGTCTATGAGAAGCTAGCAGCACCACTCCTAGAAAGAGCCTTCGAAGGCTTCAAT
ACCTGTCTTTTTGCTTATGGTCAGACTGGCTCTGGAAAATCATATACGATGATGGGATTT
AGTGAAGAACCAGGAATAATTCCAAGATTTTGTGAAGATCTTTTTTCTCAAGTAGCCAGA
AAACAAACCCAAGAGGTCAGCTATCACATTGAAATGAGCTTCTTTGAAGTATATAATGAA
AAAATTCACGACCTTCTGGTTTGTAAAGATGAAAATGGGCAGAGAAAGCAACCACTGAGA
GTGAGGGAACATCCTGTTTATGGACCATATGTTGAAGCACTGTCAATGAACATTGTCAGT
TCTTACGCTGATATCCAGAGTTGGCTAGAATTGGGAAATAAACAAAGAGCTACTGCTGCT
ACTGGTATGAATGACAAAAGTTCCCGATCTCATTCAGTTTTCACCCTGGTGATGACCCAG
ACCAAGACAGAATTTGTGGAAGGGGAAGAACACGATCACAGAATAACAAGTCGAATTAAC
CTAATAGATCTGGCAGGCAGTGAGCGCTGCTCTACGGCTCACACTAATGGAGATCGACTA
AAGGAAGGTGTGAGTATTAATAAGTCCTTGCTAACTTTGGGAAAAGTTGTATCTGCACTT
TCGGAACAAGCAAACCAAAGGAGTGTTTTTATTCCTTATCGTGAATCTGTTCTTACATGG
CTGTTAAAAGAAAGTCTGGGTGGAAATTCAAAAACTGCAATGATTGCTACGATTAGTCCC
GCTGCCAGCAACATAGAAGAAACATTAAGCACACTTAGATATGCTAACCAAGCCCGTTTA
ATAGTCAACATTGCTAAAGTAAATGAAGATATGAACGCTAAGTTAATTAGAGAATTGAAG
GCAGAAATTGCAAAGCTAAAAGCTGCTCAGAGAAACAATCGGAATATTGACCCTGAACGA
TACAGGCTCTGTCGGCAAGAAATAACATCCTTAAGAATGAAACTGCATCAACAGGAGAGA
GACATGGCAGAAATGCAAAGAGTGTGGAAAGAAAAGTTTGAACAAGCTGAAAAAAGAAAA
CTTCAAGAAACAAAAGAGTTACAGAAAGCAGGAATTATGTTTCAAATGGACAATCATTTA
CCAAACCTTGTTAATCTGAATGAAGATCCACAACTATCTGAGATGCTGCTATATATGATA
AAAGAAGGAACAACTACAGTTGGAAAGTATAAACCAAACTCGAGCCATGATATTCAGTTA
TCTGGGGTGCTGATTGCTGATGATCATTGTACTATCAAAAATTTTGGTGGGACAGTGAGT
ATTATCCCAGTTGGGGAAGCAAAGACATATGTAAATGGAAAACATATTTTGGAAATCACA
GTATTACGTCATGGTGATCGAGTGATTCTTGGTGGAGATCATTATTTTAGATTTAATCAT
CCAGTAGAAGTCCAGAAAGGAAAAAGGCCATCTGGAAGAGATACTCCTATAAGTGAGGGT
CCAAAAGACTTTGAATTTGCAAAAAATGAGTTGCTCATGGCACAGAGATCACAACTTGAA
GCAGAAATAAAAGAGGCTCAGTTGAAGGCAAAGGAAGAAATGATGCAAGGAATCCAGATT
GCAAAAGAAATGGCTCAGCAAGAGCTTTCTTCTCAAAAAGCTGCATATGAAAGCAAAATA
AAAGCACTGGAAGCAGAACTGAGAGAAGAGTCTCAAAGGAAAAAAATGCAGGAAATAAAT
AACCAGAAGGCTAATCACAAAATTGAGGAATTAGAAAAGGCAAAGCAGCATCTTGAACAG
GAAATATATGTCAACAAAAAACGATTAGAAATGGAGACTTTGGCTACAAAACAGGCTTTA
GAAGACCATAGCATCCGCCATGCAAGAATTCTGGAAGCTTTAGAAACTGAAAAGCAAAAA
ATTGCTAAAGAAGTACAAATTCTACAGCAGAATCGGAATAATAGGGATAAAACTTTTACA
ATGCAGACAACTTGGAGCTCTATGAAACTCTCAATGATGATTCAGGAAGCCAATGCTATC
AGCAGCAAATTGAAAACATACTATGTTTTTGGCAGACATGATATATCAGATAAAAGTAGT
TCTGACACTTCTATTCGGGTTCGTAACCTGAAACTAGGAATCTCAACATTCTGGAGTCTG
GAAAAGTTTGAATCTAAACTTGCAGCAATGAAAGAACTTTATGAGAGTAATGGTAGTAAC
AGGGGTGAAGATGTCTTTTGTGATCCTGAAGATGAATGGGAACCCGACATTACAGATGCA
CCAGTTTCTTCACTTTCTAGAAGGAGGAGTAGGAGTTTGATGAAGAACAGAAGAATTTCT
GGTTGTTTACATGACATACAAGTCCATCCAATTAAGAATTTGCATTCTTCACATTCATCA
GGTTTAATGGACAAATCAAGCACTATTTACTCAAATTCAGCAGAGTCATTTCTTCCTGGA

ATTTGCAAAGAATTGATTGGTTCTTCATTAGATTTTTTGGACAGAGTTATGATGAAGAA
AGAACTATAGCAGACAGCCTAATTAATAGTTTTCTTAAAATTTATAATGGGCTATTTGCC
ATTTCCAAGGCTCATGAAGAACAAGATGAAGAAAGTCAAGATAACTTGTTTTCTTCTGAT
CGAGCAATCCAGTCACTTACTATTCAGACTGCATGTGCTTTTGAGCAGCTAGTAGTGCTA
ATGAAACACTGGCTGAGTGATTTACTGCCTTGTACCAACATAGCAAGACTTGAGGATGAA
TTGAGACAAGAAGTTAAAAAACTAGGAGGCTACTTACAGTTATTTTTGCAGGGATGCTGT
TCGGATACTTCATCAATGATAAAAGAGGCTCAGAAGAATGCAATCCAAATTGTACAACAA
GCTGTAAAGTATGTGGGGCAGTTAGCAGTTCAGAAAGGGAGCAAGCTACATTTTCTAGAA
AACGGTAACAATAAAGCTGCCGGTGTCCAGGAGGAATTCATGGATGCTGTTTGTGATGGT
GTAGGCTTAGGAATGAAGATTTTATTAGATTCTGGACTGGAAAAAGCAAAAGAACTTCAG
CATGAACTCTTTAGGCAGTGTACAAAAAATGAGGTTACCAAAGAAATGAAAACTAATGCC
ATGGGATTGATTAGATCTCTTGAAAACATCTTTGCTGAATCGAAAATTAAAAGTTTCAGA
AGGCAAGTACAAGAAGAAAACTTTGAATACCAAGATTTCAAGAGGATGGTTAATCGTGCT
CCAGAATTCTTAAAGTTAAAACATTGCTTAGAGAAAGCTATTGAAATTATTATTTCTGCA
CTGAAAGGATGCCATAGTGATATAAATCTTCTCCAGACTTGTGTTGAAAGTATTTGCAAC
TTGGCCAATGATTTTTACAGTGACTTCAGTGTGCCTTCTACTTCTGTTGGCAGCTATGAG
AGTAGAGTAACTCACATTGTCCACCAGGAACTAGAATCTCTAGCTAAGTCTCTCCTCTTT
TGTTTTGAATCTGAAGAAAGCCCTGATTTGTTGAAACCCTGGGAAACTTATAATCAAAAT
ACCAAAGAAGAACACCAACAATCTAAATCAAGCGGGATTGACGGCAGTAAGAATAAAGGT
GTACCAAAGCGTGTCTATGAGCTCCATGGCTCATCCCCAGCAGTGAGCTCAGAGGAATGC
ACACCCAGTAGGATTCAGTGGGTG
>Pongo abelii XM_024237794.1
ATGTCATTACACAGTACTCATAATAGAAATAACAGCGGTGATATTCTTGATATTCCTTCTTCCCAAAATA
GTTCATCACTGAATGCCCTCACCCACAGTAGCCGACTTAAGCTGCATTTGAAGTTGGATATGTCAGAATG
TGAAAATGATGATCCATTATTGAGATCTGCAGGTAAAGTCAGAGACATAAATAGAACTTACGTTATTTCT
GCCAGTAAAAAAACAGCAGACATGCCCCTTACCCCTAATCCTGTAGGTAGATTGGCACTTCAGAGAAGAA
CTACAAGGAACAAAGAATCATCTTTGCTTGGTAGTGAGTTGGAAGACACAACTGAAAAAACAGCAGAAAC
ACGTCTTACATTACAACGTCGTGCTAAAACAGATTCTGCAGAAAAGTGGAAAACAGCTGAAATAGATTCT
GTCAAAATGACACTGAATGTGGGAGGTGAAACAGAAAATAATGGTGTTTCTAAGGAAAGTAGAACAAATG
TAAGGATTGTAAATAATGCTAAAAACTCTTTTGTTGCCTCTTCTGTACCTTTAGATGAAGATCCACAGGT
CATTGAAATGATGGCTGATAAGAAATACAAAGAAACATTTTCTGCCCCTGGTAGAGCAAATGAAAATATT
GCACTTAAGTACTCAAGTAATAGACCACCCATTGCTTCCCTGAGTCAGACTGAAGTTGTTAGATCAGGAC
ACTTGACAACGAAAGCTACTCAGAGCAAGTTGGATATCAAAGTGTTGGGAACAGGAAACTTGTATCATAG
AAGTATTGGGAAGGAAATTGCAAAAACGCCAAATAAATTTGGGAGCTTAGAAAAAAGAACACCTACAAAA
TGTACAACAGAACACAAATTGACACCAAAGTGCGGCCTGCCTCAGCTTAAGAGCCCAGCTCCATCAATAC
TGAAGAATAGAATGTCTAACCTTCAAGTTAAACAAAGACCAAAAAGTTCCTTTCTTGCAAATAAACAGGA
AAGGTCCGCAGAAAATACAATTCTTCCCGAAGAAGAAACTGTAGTTCAGAACACCTCTGCAGCAAAAGAC
CCCTTAAAAGTAGAGAATAGTCAAGTGACAGTGGCAGTACGCGTAAGACCTTTCACCAAGAGAGAGAAGA
TTGAAAAAGCATCCCAGGTAGTCTTCATGAGTGGGAAAGAAATAACTGTGGAACATCCTGACATGAAACA
AGTTTATAATTTTATTTATGATATTTCATTCTGGTCTTTTGATGAATGTCATCCTCACTACGCTAGCCAG
ACAATTGTCTATGAGAAGCTAGCAGCACCACTCCTAGAAAGAGCCTTTGAAGGCTTCAATACCTGTCTTT
TTGCTTATGGTCAGACTGGCTCTGGAAAATCATATACGATGATGGGATTTAGTGAAGAACCAGGAATAAT
TCCAAGATTTTGTGAAGATCTTTTTTCTCAAGTAGCCAGAAAACAAACCCAAGAGGTCAGCTATCACGTT
GAAATGAGCTTCTTTGAAGTATATAATGAAAAAATTCATGACCTTCTGGTTTGTAAAGATGAAAATGGGC
AGAGAAAGCAACCACTGAGAGTGAGGGAACATCCTGTTTATGGACCATATGTTGAAGCACTGTCAATGAA
TGTTGTCAGTTCTTACACTGATATCCAGAGTTGGCTAGAATTGGGAAATAAACAAAGAGCTACTGCTGCT
ACTGGTATGAATGATAAAAGCTCCCGATCTCATTCAGTTTTCACCCTGGTGATGACCCAGACCAAGACAG

AATTTGTGGAAGGGGAAGAACACGATCACAGAATAACAAGTCGAATTAACCTAATAGATCTGGCAGGCAG
TGAGCGCTGCTCTACGGCTCACACTAATGGAGATCGACTAAAGGAAGGTGTGAGTATTAATAAGTCCTTG
CTAACTTTGGGAAAAGTTATATCTGCCCTCTCTGAACAAGCAAACCAAAGGAGAGTTTTTATTCCTTATC
GTGAATCTGTTCTTACATGGCTGTTAAAAGAAAGTCTGGGTGGAAATTCAAAAACTGCAATGATTGCTAC
GATTAGTCCTGCTGCCAGCAACATAGAAGAAACATTAAGCACACTTAGATATGCTAACCAAGCCCGTTTA
ATAGTCAATGTTGCCAAAATAAATGAAGATATGAACGCTAAGTTAATTAGAGAATTGAAGGCAGAAATTG
CAAAGCTAAAAGCTGCTCAGAGAAACAATCGGAATATTGACCCTGAGCAATACAGGCTCTGTCGGCAAGA
AATAACATCCTTAAGAATGAAACTGCATCAACAGGAGAGAGACATGGCAGAAATGCAAAGAGTGTGGAAA
GAAAAGTTTGAACAAGCTGAAAAAAGAAAACTTCAAGAAACTAAAGAGTTACAGAAAGCAGGAATTACAT
TTCAAATGGACAATCATTTACCAAACCTTGTTAATCTGAATGAAGATCCACAACTATCTGAGATGCTGCT
ATATATGATAAAAGAAGGAACAACTACAGTTGGAAAGTATAAACCAAACTCAAGCCATGATATTCAGTTA
TCTGGGGTGCTGATTGCTGATGATCATTGTACTATCAAAAATTTTGGTGGGACAGTGAGTATTATCCCAG
CTGGGGAAGCAAAGACATATGTAAATGGAAAACATATTTTGGAAATCACAGTATTACGTCATGGTGATCG
AGTGATTCTTGGTGGAGATCATTATTTTAGATTTAATCATCCAGTAGAAGTCCAGAAAGGAAAAAGGCCA
TCTGGAAGAGATGCTCCTATAAGTGAGGGTCCAAAAGACTTTGAATTTGCAAAAAATGAGTTGCTCATGG
CACAGAGATCACAACTTGAAGCAGAAATAAAAGAGGCTCAGTTGAAGGCAAAGGAAGAAATGATGCAAGG
AATCCAGATTGCAAAAGAAATGGCTCAGCAAGAGCTTTCTTCTCAAAAAGCTGCATATGAAAGCAAAATA
AAAGCGCTGGAAGCAGAACTGAGAGAAGAGTCTCAAAGGAAAAAAATGCAGGAAATAAATAACCAGAAGG
CTAATCACAAAATTGAGGAATTAGAAAAGGCAAAGCAGCATCTTGAACAGGAAATATATGTCAACAAAAA
GCGATTAGAAATGGAAACTTTGGCTACAAAACAGGCTTTAGAAGACCATAGCATCCGCCATGCAAGAATT
CTGGAAGCTTTAGAAACTGAAAAGCAAAAAATTGCTAAAGAAGTACAAATTCTACAGCAGAATCGGTATA
ATAGGGATAAAACTTTTACAATGCAGACAACTTGGAGCTCTATGAAACTCTCAATGATGATTCAGGAAGC
CAATGCTATCAGCAGCAAATTGAAAACATACTATGTTTTTGGCAGACATGATATATCAGATAAAAGTAGT
TCTGACACTTCTATTCGGGTTCGTAACCTGAAACTAGGAGTCTCAACATTCTGGAGTCTGGAAAAGTTTG
AATCTAAACTTGCAGTGATGAAAGAACTTTATGAGAGTAATGGTAGTAACAGGGGTGAAGATGTCTTTTG
TGATCCTGAAGATGAATGGGAACCCGACATTACGGATGCACCAGTTTCTTCACTTTCTAGAAGGAGGAGT
AGGAGTTTGATGAAGAACAGAAGAATTTCTGGTTGTTTACATGACATACAAGTCCATCCAATTAAGAATT
TGCATTCTTCGCATTCATCAGGTTTAATGGACAAATCAAGTACTATTTACTCAAATTCAGCAGAGTCATT
TCTTCCTGGAATTTGCAAAGAATTGATTGGTTCTTCATTAGATTTTCTTGGACAGAGTTATGATGAAGAA
AGAACTATAGCAGACAGCCTAATTAATAGTTTTCTTAAAATTTATAATGGGCTATTTGCCATTTCCAAGG
CTCATGAAGAACAAGATGAAGAAAGTCAAGATAACTTGTTTTCTTCTGATCGAGCAATCCAGTCACTTAC
TATTCAGACTGCATGTGCTTTTGAGCAGCTAGTACTGCTAATGAAACACTGGCTGAGTGATTTACTGCCT
TGTACCGACATAGCAAGACTTGAGGATGAATTGAGACAAGAAGTTAAAAAACTGGGAGGCTACTTACAGT
TATTTTTGCAGGGATGCTGTTCAGATATTTCATCAATGATAAAAGAGGCTCAAAAGAATGGAATCCAAAT
TGTACAACAAGCTGTAAAGTATGTGGGGCAGTTAGCAGTTCTGAAAGGGAGCAAGCTACATTTTCTAGAA
AATGGTAACAATAAAGCTGCCAGTGTCCAGGAGGAATTCATGGATGCTGTCTGTGATGGTGTAGGCTTAG
GAATGAAGATTTTATTAGATTCTGGACTGGAAAAAGCAAAAGAACTTCAGCATGAACTCTTAAGGCAGTG
TACAAAAAATGAGGTTACCAAAGAAATGAAAACTAATGCCATGGGATTGATTAGATCTCTTGAAAACATC
TTTGCTGAATCGAAAATTAAAAGTTTCAGAAGGCAAGTACAAGAAGAAAACTTTGAATACCAAGCTTTCA
AGAAGATGGTTAATCGTGCTCCAGAATTCTTAAAGTTAAAACATTGCTTAGAGAAAGCTATTGAAATTAT
TATTTCTGCACTGAAAGGATGCAGTAGTGATGTAAATCTTCTCCAGACTTGTGTTGAAAGTATTCGTATC
TTGGCCAGTGATTTTTACAGTGACTTCAGTGTGCCTTCTACTTCTGTTGACAGCTATGAGAGTAGAGTAA
CTCACGTTGTCCACCAGGAACTAGAATCTCTAGCTAAGTCTCTCCTCTTTTATTTTGAATCTGAAGAAAG
TCCTGATTTGTTGAAACCCTGGGAAACTTATAATCAAAATACCAAAGAAGAACACCAACAATCTAAATCA
AGCGGGATTGACGGCAGTAAGAATAAAGGTGTACCAAAGCGTGTCTATGAGCTCCGTGGCTCATCCCCAG
CAGTGAGCTCAGAGGAATGCATACCCAGTAGGATTCAGTGGGTG
>gorilla ENSGGOT00000013873.2

ATGTCATTACACAGTACTCATAATAGAAATAACAGCGGTGATATTCTTGATATTCCTTCT
TCCCAAAATAGTTCATCACTGAATGCCCTCACCCACAGTAGCCGACTTAAGCTGCATTTG
AAGTCGGATATGTCAGAATGTGAAAATGATGATCCATTATTGAGATCTGCAGGTAAAGTC
AGAGACATAAATAGAACTTATGTTATTTCTGCCAGTAAAAAAACAGCAGACATGCCCCTT
ACCCCTAATCCTGTAGGTAGATTGGCACTTCAGAGGAGAACTACAAGGAACAAAGAATCA
TCTTTGCTTGTTAGTGAGTTGGAAGACACAACTGAAAAAACAGCAGAAACACGTCTTACA
TTACAACGTCGTGCTAAAACAGAGTCTGCAGAAAAGTGGAAAACAGCTGAAATAGATTCT
GTCAAAATGACACTGAATGTGGGAGGTGAAACAGAAAATAATGGTGTTTCTAAGGAAAGT
AGAACAAATGTAAGGATTGTAAATAATGCTAAAAACTCTTTTGTTGCCTCTTCTGTACCT
TTAGATGAAGATCCACAGGTCATTGAAATGATGGCTGATAAGAAATACAAAGAAACATTT
TCTGCCCCCGGTAGAGCAAATGAAAATGTTGCACTTAAGTATTCAAGTAATAGACCACCC
ATTGCTTCCCTGAGTCAGACTGAAGTTGTTAGATCAGGACACTTGACAACCAAACCTACT
CAGAGCAAGTTGGATATCAAAGTGTTGGGAACAGGAAACTTGTATCATAGAAGTATTGGG
AAGGAAATTGCAAAAACTTCAAATAAATTTGGGAGCTTAGAAAAAAGAACACCTACAAAA
TGTACAACAGAACACAAATTGACACCAAAGTGCGGCCTGCCTCAGCTTAAGAGCCCAGCT
CCATCAGTACTGAAGAATAGAATGTCTAACCTTCAAGTTAAACAAAGACCAAAAAGTTCC
TTTCTTGCAAATAAACAGGAAAGGTCCGCAGAAAATACAATTCTTCCCGAAGAAGAAACT
GTAGTTCAGAACACCTCTGCAGGAAAAGACCCCTTAAAAGTAGAGAATAGTCAAGTGACA
GTGGCAGTACGCGTAAGACCTTTCACCAAGAGAGAGAAGATTGAAAAAGCATCCCAGGTA
GTCTTCATGAGTGGGAAAGAAATAACTGTGGAACACCCTGACATGAAACAAGTTTATAAT
TTTATTTATGATGTTTCATTCTGGTCTTTTGATGAATGTCATCCTCACTACGCTAGCCAG
ACAACTGTCTATGAGAAGCTAGCAGCACCACTCCTAGAAAGAGCCTTCGAAGGCTTCAAT
ACCTGTCTTTTTGCTTATGGTCAGACTGGCTCTGGAAAATCATATACGATGATGGGATTT
AGTGAAGAACCAGGAATAATTCCAAGATTTTGTGAAGATCTTTTTTCTCAAGTAGCCAGA
AAACAAACCCAAGAGGTCAGCTATCACATTGAAATGAGCTTCTTTGAAGTATATAATGAA
AAAATTCATGACCTTCTGGTTTGTAAAGATGAAAATGGGCAGAGAAAGCAACCACTGAGA
GTGAGGGAACATCCTGTTTATGGACCATATGTTGAAGCACTGTCAATGAACATTGTCAGT
TCTTACGCTGATATCCAGAGTTGGCTAGAATTGGGAAATAAACAAAGAGCTACTGCTGCT
ACTGGTATGAATGATAAAAGTTCCCGATCTCATTCAGTTTTCACCCTGGTGATCACCCAG
ACCAAGACAGAATTTGTGGAAGGGGAAGAACATGATCACAGAATAACAAGTCGAATTAAC
CTAATAGATCTGGCAGGCAGTGAGCGCTGCTCTACGGCTCACACTAATGGAGATCGACTA
AAGGAAGGTGTGAGTATTAATAAGTCCTTGCTAACTTTGGGAAAAGTTATATCTGCACTT
TCGGAACAAGCAAACCAAAGGAGTGTTTTTATTCCTTATCGTGAATCTGTTCTTACATGG
CTGTTAAAAGAAAGTCTGGGTGGAAATTCAAAAACTGCAATGATTGCTACGATTAGTCCC
GCTGCCAGCAACATAGAAGAAACATTAAGCACACTTAGATATGCTAACCAAGCCCGTTTA
ATAGTCAACATTGCTAAAGTAAATGAAGATATGAACGCTAAGTTAGTTAGAGAATTGAAG
GCAGAAATTGCAAAGCTAAAAGCTGCTCAGAGAAACAATCGGAATATTGACCCTGAACGA
TACAGGCTCTGTCGGCAAGAAATAACATCCTTAAGAATGAAACTGCATCAACAGGAGAGA
GACATGGCAGAAATGCAAAGAGTGTGGAAAGAAAAGTTTGAACAAGCTGAAAAAAGAAAA
CTTCAAGAAACAAAAGAGTTACAGAAAGCAGGAATTATGTTTCAAATGGACAATCATTTA
CCAAACCTTGTTAATCTGAATGAAGATCCACAACTATCTGAGATGCTGCTATATATGATA
AAAGAAGGAACAACTACAGTTGGAAAGTATAAACCAAACTCAAGCCATGATATTCAGTTA
TCTGGGGTGCTGATTGCTGATGATCATTGTACTATCAAAAATTTTGGTGGGACAGTGAGT
ATTATCCCAGTTGGGGAAGCAAAGACATATGTAAATGGAAAACATATTTTGGAAATCACA
GTATTACATCATGGTGATCGAGTGATTCTTGGTGGAGATCATTATTTTAGATTTAATCAT
CCAGTAGAAGTCCAGAAAGGAAAAAGGCCATCTGGAAGAGATACTCCTATAAGTGAGGGT
CCAAAAGACTTTGAATTTGCAAAAAATGAGTTGCTCATGGCACAGAGATCACAACTTGAA

GCAGAAATAAAAGAGGCTCAGTTGAAGGCAAAGGAAGAAATGATGCAAGGAATCCAGATT
GCAAAAGAAATGGCTCAGCAAGAGCTTTCTTCTCAAAAAGCTGCATATGAAAGCAAAATA
AAAGCACTGGAAGCAGAACTGAGAGAAGAGTCTCAAAGGAAAAAAATGCAGGAAATAAAT
AACCAGAAGGCTAATCACAAAATTGAGGAATTAGAAAAGGCAAAGCAGCATCTTGAACAG
GAAATATATGTCAACAAAAAGCGATTAGAAATGGAGACTTTGGCTACAAAACAGGCTTTA
GAAGACCATAGCATCCGCCATGCAAGAATTCTGGAAGCTTTAGAAACTGAAAAGCAAAAA
ATTGCTAAAGAAGTACAAATTCTACAGCAGAATCGGAATAATAGGGATAAAACTTTTACA
ATGCAGACAACTTGGAGCTCTATGAAACTCTCAATGATGATTCAGGAAGCCAATGCTATC
AGCAGCAAATTGAAAACATACTATGTTTTTGGCAGACATGATATATCCGATAAAAGTAGT
TCTGACACTTCTATTCGAGTTCGTAACCTGAAACTAGGAATCTCAACATTCTGGAGTCTG
GAAAAGTTTGAATCTAAACTTGCAGCAATGAAAGAACTTTATGAGAGTAATGGTAGTAAC
AGGGGTGAAGATGTCTTTTGTGATCCTGAAGATGAATGGGAACCCGACATTACAGATGCA
CCAGTTTCTTCACTTTCTAGAAGGAGGAGTAGGAGTTTGATGAAGAACAGAAGAATTTCT
GGTTGTTTACATGACATACAAGTCCATCCAATTAAGAATTTGCATTCTTCACATTCATCA
GGTTTAATGGACAAATCAAGCACTATTTACTCAAATTCAGCAGAGTCATTTCTTCCTGGA
ATTTGCAAAGAATTGATTGGTTCTTCATTAGATTTTTTGGACAGAGTTATGATGAAGAA
AGAACTATAGCAGACAGCCTAATTAATAGTTTTCTTAAAATTTATAATGGGCTATTTGCT
ATTTCCAAGGCTCATGAAGAACAAGATGAAGAAAGTCAAGATAACTTGTTTTCTTCTGAT
CGAGCAATCCAGTCACTTACTATTCAGACTGCATGTGCTTTTGAGCAGCTAGTAGTGCTA
ATGAAACACTGGCTGAGTGATTTACTGCCTTGTACCAACATAGCAAGACTTGAGGATGAA
TTGAGACAAGAAGTTAAAAAACTGGGAGGCTACTTACAGTTATTTTTGCAGGGATGCTGT
TCGGATATTTCATCAATGATAAAAGAGGCTCAAAAGAATGCAATCCAAATTGTACAACAA
GCTGTAAAGTATGTGGGGCAGTTAGCAGTTCTGAAAGGGAGCAAGCTGCATTTTCTAGAA
AACGGTAACAATAAAGCTGCCAGTGTCCAGGAGGAATTCATGGATGCTGTTTGTGATGGT
GTAGGCTTAGGAATGAAGATTTTATTAGATTCTGGACTGGAAAAAGCAAAAGAACTTCAG
CATGAACTCTTTAGGCAGTGTACAAAAAAATGAGGTTACCAAAGAAATGAAAACTAATGCC
ATGGGATTGATTAGATCTCTTGAAAACATCTTTGCTGAATCGAAAATTAAAAGTTTCAGA
AGGCAAGTACAAGAAGAAAACTTTGAATACCAAGATTTCAAGAGGATGGTTAATCGTGCT
CCAGAATTCTTAAAGTTAAAACATTGCTTAGAGAAAGCTATTGAAATTATTATTTCTGCA
CTGAAAGGATGCCATAGTGATATAAATCTTCTCCAGACTTGTGTTGAAAGTATTTGCAAC
TTGGGCAGTGATTTTTACAGTGACTTCAGTGTGCCTTCTACTTCTGTTGGCAGCTATGAG
AGTAGAGTAACTCACATTGTCCACCAGGAACTAGAATCTCTAGCTAAGTCTCTCCTCTTT
TGTTTTGAATCTGAAGAAAGCCCTGATCTGTTGAAACCCTGGGAAACTTATAATCAAAAT
ACCAAAGAAGAACACCAACAATCTAAATCAAGCGGGATTGACGGCAGTAAGAATAAAGGT
GTACCAAAGCGTGTCTATGAGCTCCATGGCTCATCCCCAGCAGTGAGCTCAGAGGAATGC
ACACCCAGTAGGATTCAGTGGGTG
>maccaca mulatta ENSMMUT00000006062.3
ATGTCATTACACAGTACTCATAATAGAAATAACCGCGGCGATATTCTTGATATTCCTTCT
TCCCAAAATAGTTCATCACTGAATGCCCTCACCCACAGTAGCCGACTTAAGCTGCATTTG
AAGTCAGATATGTCAGAATGTGAAAATTATGATCCATTATTGAGATCTGCAGGTAAAGTC
AGAGACATAAATAGCACTTACGTTATTTCTGCCAGTGAAAAAACAGCAGACATGCCCCTT
ACCCCTAATCCTGTAGGTAAATTGACACTTCAGAGAAGAACTACAAGGAACAAAGAATCA
TCTTTGCTTGGTAGGGATTTGGAAGATACAACTGAAAAAACAGCAGAAACACATCTTACA
TTACAACGTCGTGCTAAAACAGATTCTGAAGAAAAGTGGAAAACAGCTGAAACAGATTCT
GTCAAAATGACACTGAATGTGGGAGGTGAAACAGAAAATAATGGTGTTTCTAAGGAAAGT
AGAACAAATGTAAAGATTGTAAATAATGCTAAAAACTCTTTTGTTGCCTCTTGTGTACCT
TTAGATAAAGATCCACAAGTCATTGAAATGATGGCTGATAAGAAATACAAAGAAATATTT

TCTGCCCCCAGTAGAGCAAATGAAAATATTGCACTTAAGTACTCAAGTAATAGAGCACCC
ATTGCTTCCCTGAGTCAGACTGAAGTTGTTAGATCAGGACACTTGACAACAAAACCTACT
CAGAGCAAGTTGGATATCAAAGTGTCAGGAACAGGAAACTTGCATCATAGAACTATTGGG
AAGGAAATTGCAAAAACTTCAAATAAATTTGGGAGCTTAGAAAAAAGAACACCTACAAAA
TGTACAACAGAACACAAATTGACACCAAAGTGTGGTCTGCCTCAGCTTAAGAGCCCAGCT
CCATCAATACTGAAGAATAGAATGTCTAACTTTCAAGTTAAACAAAGACCAAAAAGTTCC
TTTCTTGCGAATAAACAGGAAAGGTCAGCAGAAAATACAATCCTTCCTGAAGAAGAAACT
GTAGTTCAGAACACCTCTGCAGGAAAAGACCCCTTAAAAGTAGAGAATAGTCAAGTGACA
GTGGCAGTACGCATAAGGCCTTTCACTAAGAGAGAGAAGATTGAAAAAGCATCCCAGGTG
GTCTTCATGAGTGGGAAAGAAATAACTGTGGAACATCCTGACATGAAACAAGTTTATAGT
TTTATTTATGATGTTTCATTCTGGTCTTTTGATGAATGTCATCCTCACTATGCTAGCCAG
ACAACTGTCTATGAGAAGCTAGCAGCACCACTCCTAGAAAGAGCCTTTGAAGGCTTCAAT
ACCTGTCTTTTTGCTTATGGTCAGACTGGCTCTGGAAAATCATATACGATGATGGGATTT
AGTGAAGAACCAGGAATAATTCCAAGATTTTGTGAAGATCTTTTTTCTCAAGTAGCCAGA
AAACAAACCCAAGAGGTCAGCTATCACATTGAAATGAGCTTCTTTGAAGTCTATAATGAA
AAAATTCATGATCTTCTGGTTTGTAAAGATGAAAATGGGCAGAGAAAGCAACCACTGAGA
GTGAGGGAACATCCTGTTTATGGACCATATGTTGAAGCACTGTCAATGAATGTTGTCAGT
TCTTACACTGATATCCAGAGTTGGCTAGAATTGGGAAATAAACAAAGAGCTACCGCTGCT
ACTGGGATGAATGATAAAAGCTCCCGATCTCATTCAGTTTTCACTCTGGTGATGACCCAG
ACCAAGACAGAATTTGTGGAAGGGGAAGAACATGATCACAGAATAACAAGTCGAATTAAC
TTAATAGATCTGGCAGGCAGTGAGCGCTGCTCTACGGCTCACACTAGTGGAGATCGACTA
AAGGAAGGTGTGAGTATTAATAAGTCCTTGCTAACTTTGGGAAAAGTTATATCTGCACTC
TCTGAACAAGCAAACCAAAAGAGAGTTTTTATTCCTTATCGTGAATCTGTTCTTACATGG
CTGTTAAAAGAAAGTCTGGGTGGAAATTCAAAAACTGCAATGATTGCTACGATTAGTCCT
GCTGCCAGCAACATAGAAGAAACATTAAGCACACTTAGATATGCTAACCAAGCCCGTTTA
ATAGTCAACATTGCCAAAGTAAATGAAGATATGAATGCCAAGTTAATTAGAGAATTGAAG
GCAGAAATTGCAAAGCTAAAAGCTGCTCAGCGAAACAATCGGAAGATTGACCCTGAACAA
TACAGGCTCTGTCGGCAAGAAATAACATCCTTAAGAATGAAACTGCATCAACAGGAGAGA
GACATGGCAGAAATGCAAAGAGTGTGGAAAGAAAAGTTTGAACAAGCTGAAAAAAGAAAA
CTTCAAGAAACAAAAGAGTTACAGAAAGCAGGAATTACATTTCAAATGGACAATCATTTA
CCAAACCTTGTTAATCTGAATGAAGATCCACAACTATCAGAGATGCTGCTATATATGATA
AAAGAAGGAACAACTACAGTTGGAAAGTATAAACCAAACTCAAGCCATGATATTCAGTTA
TCTGGTGTGCTGATTGCTGATGATCATTGTACTATCACAAATTTAGGTGGGACAGTGAGT
ATTATCCCACTTGGGGAAGCAAAGACATACGTAAATGGAAAACGTATTTTGGAACTCACA
GTATTACATCATGGTGATCGAGTGATTCTTGGTGGAGATCATTATTTTAGATTTAATCAT
CCAGTAGAAGTCCAGAAAGGAAAAAGGCCATCTGGAAGAGATACTCCTATAAGTGAGGGT
CCAAAAGACTTTGAATTTGCAAAAAATGAGTTGCTCATGGCACAGAGATCACAACTCGAA
GCAGAAATAAAAGAGGCGCAGTTGAAGGCAAAGGAAGAAATGATGCAAGGAATCCAGATT
GCAAAAGAAATGGCTCAGCAAGAGCTTTCTTCTCAAAAAGCTGCGTATGAAAGCAAAATA
AAAGCACTGGAAGCAGAACTGAGAGAAGAGTCTCAAAGGAAAAAAATGCAGGAAATAAAT
AACCAGAAGGCTAATCACATAATTGAGGAATTAGAAAAGGCAAAGCAGCATCTTGAACAA
GAAATATATGTCAACAAAAAGCGATTAGAAATGGAGACTTTGGCTACAAAACAGGCTTTA
GAAGACCATAGCATCCGCCATGCAAGAATTCTGGAAGCTTTAGAAACTGAAAAGCAAAAA
ATTGCTAAAGAAGTAAAAATTCTACAGCAGAATCGGAGTAATAGGGATAAAACTTTTACA
ATGCAGACAACTTGGAGCTCTATGAAACTCTCAATGATGATTCAGGAAGCCAATGCGATC
AGCAGCAAATTAAAAACATACTATGTTTTTGGCAGACATGATGTATCAGATAAAAGTAGT
TCTGACACTTCTATTCGGGTTCGTAACCTGAAACTAGGAATCTCAACATTCTGGAGTCTG

GAAAAGTTTGAATCTAAACTTGCAGCAATGAAAGAACTTTATGAGAGTAATGGTAGTAAC
AGAGATGAAGATGTCTTTTGTGATCCTGAAGATGAATGGGAACCTGACATTACAGATGCA
CCAGTTTCTTCACTTTCTAGAAGGAGGAGCAGGAGTTTGATGAAGAACAGAAGAATTTCT
GGTTGTTTACATGACATACAAGTTCACCCAATTAAGAATTTGCATTCTTCACATTCATCA
GGTTTAATGGACAAATCAAGCACCATTTACCCAAATTCAGCAGAGTCATTTCTTCCTAGA
ATTTGCAAAGAATTGATTGGTTCTTCATTAGATTTTCTTGGACAGAGTTATGATGAAGAA
AGAACTATAGCAGACAGCCTAATGAATAGTTTTCTTAAAATTTATAATGGGCTATTTGCC
ATTTCCAAGGCTCATGAAGAACAAGATGAAGAAAGTCAAGATAACTTGTTTTCTTCTGAT
CGAGCAATCCAGTCACTTACCATTCAGACTGCATGTGCTTTTGAGCAGCTAGTAGTGCTA
ATGAAACACTGGCTGAGTGATATACTGCCTTGTACCACCAACATAGCAAGACTTGAGGAT
GAATTGAGACAAGAAGTTAAAAAACTGGGAGGCTACTTACAGTTATTTTTGCAGGGATGC
TGTTCGGATATTTCATCAATGATAAAAGAGGCTCAAAAGAATGCAATCCAAATTGTACAA
CAAGCTGTAAAGTATGTGGGGCAGTTAGCAGTTCTGAAAGGGAGCAAGCTACATTTTCTA
GAAAATGGTAACAATAAAGCTGCCAGCGTTCAGGAGGAATTCATGGATGCTATTTGTGAT
GGTGTAGGCTTAGGAATGAAGATTTTATTAGATTCTGGACTTGAAAAAGCAAAAGAACTT
CAGCATGAACTCTTAAGGCAGTGTACAAAAAATGAGGTTACCAAAGAAATGAAAACTAAT
GCCATGGGATTGATTAGATCTCTTGAAAACATCTTTGCCGAATCGAAAATTAAAAGTTTC
CGAAGACAAGTACAAGAAGAAAACTTTGAATACCAAGATTTCAAGAAGATGGCTAATCAT
GCTCCAGAATTCTTAAAGTTGAAACATTGCTTAGAGAAAACTATTGAAATTATTATTTCT
GCACTGAAAGGATGCAGTAGTGATGTAAATCTTCTCCAGAATTGTGTTGAAAGTATTCGC
AACTTGGCCAGTGATTTTTACAGTGACTTCGGTGTGCCTTCTACTTCTGTTGACAGCTAT
GAGAGTAGAGTAACTAATGTTGTCCACAAGGAACTAGAATCTCTAGCTAAGTCTCTCCTC
TTTTGTTTTGAATCTGAAGAAAGCCCTGATTTGTTGAAACCTTGGGAAACTTATAATCAA
AATACCAAAGAAGAACACCAACAATCTAAATCAAGCAGGATTGATGGCAGCAAGAATAAA
GGTGTACCAAAGCGTGTCTATGAGCTCCATGGCTCATCCCCAGCAGTGAGCCCAGAGGAA
TGCACACCCAGTAGGATTCAGTGGGTG
>Carlito syrichta XM_008067107.1
ATGTCAGTACACACTGCTCATAATAGAAAGAACAGCAACATCCTTGGTAGTCCTTCTTCTGAAGAGAGTT
CATCACTGAGTGCTCTCACTCACAGTAGCAGACTTAAGCTGCATTTGAAGTCGGATATGTCAGAATGTGA
AAATGATGATCCATTACTGAGATCTGCATGTAAAATCAGAGACATAAATAGCACCTATGTTATTTCTGCC
GGTAAAAAAACAGGAGATACTCCCCTTAAATCTAACCCTGTGAGTAGCTTGACACTTCAGAGAAGAGCTA
CAAGGAACAAAGAATCATCTTTGCTTGGTGGTGAGCGGGGAGACACAATTGAAAAAACAGCAGAAACATG
TCTTACGTTACAACGTCGTGCTAAAACAGATTCTGCAGAAAAGTGGAAAACAACTCAAATGGATTCTTTC
AAAAAGTTGAAAATGACACAGAATGTGGGTGGTGAAACTGAAACTAATTGTGCCTCTAAGGAAACCAGTA
AAAATGTAAAGATTGTAAGTAATGATAAAAACTCTTTTGTGGCATCTTCTCTATCTTCAGCTAAAGACCC
AAAAGACTTTGAAATGATGGCTGATAAGAAATATAAAGAAACATTTTCTGCCCTCAAAGGGGCAAATGAA
AATGATTCACTTAGGTACTCAAGTAGTAGAGCACCCATTGGTTCCCAGAGTCAGATTGAAGTTGGTAGAT
CAGAACACTTGGCAACAAAACCTATTCAGAGTCAATTGGATATCAAAGTGTCAGGAACAGGAAACTTATA
TCATAGAAGTATTGGGAAGGACATTACAAAAAATTCAAGTAAATTTGGAAGCTTAGAGAAAAGAACGCCT
ACGAAATGTATAACAGAACACAAATTGACACTGAAGTGTGGCATGCCTCAGTTTAAGAGCCCAACTGCAT
CAATACTGAAAAATAGAATGTCTAACCTTCAGGTTAAACAAAGGCCAAAAAGTTCTCTTCTTGCAAATAA
AAGGGAAAGGTCACAAGAAAATACATTACCTCCTGAAGAAGATGCTGCAATTCAGAACACTTCTATAGTA
ACAGACCCATTAAAAGTAGAAAATAGTCAAGTGACAGTGGCAGTACGTGTAAGACCTTTCACCAGGAGAG
AGAAGATTGAAAAGGCATCCCAGGTGGTTTTCATGAATGGAAAAGAAGTAACCGTGGAACATCCTGATAT
GAAACAAGTTTATAATTTTATTTATGACATTTCATTCTGGTCTTTTGATGAATGTCACCCTCACTATGCT
AGCCAGACAACTGTCTATGAGATGCTAGCAGCACCACTTCTAGAAAGAGCCTTTGAAGGCTACAATACCT
GTCTTTTTGCTTATGGTCAGACGGGCTCTGGAAAATCATATACGATGATGGGATTCAGTGAAGAACGAGG

AATAATTCCAAGGTTTTGTGAAGATCTTTTTGCTCAAGTAGCCAAAAAACAAACCCAGGAGGTCAGCTAC
CACCTAGAGATGAGCTTCTTTGAAGTATATAATGAAAAAATTCATGACCTTCTGGTTTGTAAAAGTGAAA
ATGGGCAGAGAAAGCAACCACTGAGAGTACGGAACATCCTATTTCTGGACCATATGTTGAAGCGCTGTC
AATGAATGTTGTCAGTTCTTACTCTGATATCCAGAGCTGGCTAGAATTGGGAAATAAACAAAGAGCAACT
GCTGCTACTGGGATGAATGATAAAAGCTCCCGATCTCATTCAGTTTTCACCCTGGTGATGACCCAGACCA
AGACAGAATTTGTGGAGGGGGAAGAACATGATCACAGAATAACGAGTCGCATAAACTTAGTAGATCTGGC
AGGCAGTGAGCGCTGTTCAACGACGCACACAAGTGGAGATCGGCTGAAGGAAGGTGTGAGTATTAACAAG
TCTTTGCTATCTTTGGGAAAAGTTATATCTGCACTCTCTGAACAAGGAAACCAAAGGAGGGTTTTTATTC
CTTATCGTGAATCTGTTCTTACATGGCTGTTAAAAGAAAGTCTGGGTGGAAATTCAAAAACTGCAATGAT
TGCTACAATTAGTCCCGCTGCCAGCAACATAGAAGAAACCTTAAGCACACTTAGATATGCTAACCAAGCC
CGCTTAATAGTCAACATTGCCAAAGTAAATGAAGATATGAATGCTAAGTTAATTAGAGAACTGAAGGCAG
AAATTGAAAAGCTAAAAACTGCTCAGAAAAACAATCGGAATATTGACCCTGAACGATACAGGCTCTATCA
GCAAGAAATAGCATCCTTAAGAATGAAACTGCATCAGCGGGAGAGAGACATGGCTGAAATGCAAAGAATG
TGGAAAGAAAAGTTTGAACAAGCTGAAAAAAGAAAACTTCAGGAAACAAAGGAGTTACAGAAAGCAGGAA
TTACATTTCAAATGGACAACCATTTGCCAAACCTTGTTAATCTCAGTGAAGATCCACAACTATCAGAGAT
GCTACTATATATGATAAAGGAAGGAACAACTACACTTGGAAAGTATAAACCAAACTCAAGACATGATATT
CAGTTATCTGGGGTGCTGATTGCCGATGATCATTGCACTATCAAAAATTTTGGTGGGACAGTGAGTATTA
TCCCAGTTGGAGATGCAAAGACATATATAAATGGAAAACATATTCTGGAACCCACGGTATTACACCATGG
TGATCGGATAATTCTTGGTGGAGATCATTATTTTAGATTTAATCATCCAGTAGAAGTCCAGAAAGGGAAA
GGACCATCCAGTAAAGATACTCTTATAATTGAGGGTCCAAAAGACTTTGAATTTGCAAAGAATGAGTTGC
TCTTGGCACAGAAATCACAACTTGAAGCAGAAATAAAAGAGGCGCAGTTGAGAGCAAAGGAAGAAATGAT
GCAAGGAATCCAGATTGCAAAAGAGATGGCTCAGCAAGAGCTTTCTTCTCAAAAAGCTGCATATGAAAGC
AAAATAAGAGCACTGGAAGTAGAGCTGAAAGAAGAGTCTCAGAGGAAGAAAATGCAAGAAATAAATAACC
AAAAGGCTAATCACAAAATTGAGGAATTAGAAAAGGCAAAGCACCATCTTGAACAGGAAATATATATCAA
CAAAAAGCGATTAGAAATGGAGACTTTGACTACAAAGCAGGCCTTAGAAGACCACAGCATCCATCATGCA
AGAATTCTGCAAGCTTTAGAAACTGAAAAGCAAAAAATTGCTAGAGAAGTGCAAATTCTACAGCAGAATT
GGAGTAATAGGGATAAAACTTTCACAATTCAGACAAATTGGAACTCCATGAAACTCTCAATGATGATCCA
GGAAGCTAATGCTATCAGCAGCAAATTAAAAATATATTATGTTTTTGGCAGGCATGATGTATCAGGTAGC
TCTGACACTTCTATTCGGGTTCGTAACCTGAAACTAGGGATCTCAACTTTCTGGAGTCTGGAAAAGTTTG
AATCTAAACTTGCAGCAATGAAAGAACTTTATGAGAGCAATGGTAGTAACAGGGGCGAAGATGTCTTTTG
CGATCCTGAGGATGAATGGGAGCCTGACATTACAAATGCACCAATTTCTTCATTTTCTAGAAGGAGGAGC
AGGAGTTTGATGAAGAATAGAAGGATTTCTGATTGTTTACATGACATACAAGTCCACCCAATTCAGAATT
TGCATTCTTCACATTCATCAGGTGTAATGGAGAAACCAAGCACCATTTACTCAAATTCAGGAGAATCATT
TCTTCCTGGAATTTGCAAAGAATTGATTGGTTCATCATTAGCTTTTCTTATACAGAGTTATGATGAAGAA
AACACTATAGCAGATAGCCTGATGAATAATTTTTCAAAAATTTATAAAGGGCTAGTTGCCATTTCCAAAG
CTCATGAGGAACAAGATGAAGAGAGTCAAGATAACTTGTTTTCTTCTGATCGTGCAATCCAGTCACTTAC
TATCCAAATAGCATGTGCTTTTGAGCAGCTTGTGGTGCTAATCAAACACTGGCTGAATGGTTTACTACCT
TATACCAGCACAGCAAGACTTGAGGATGAATTGAAACAAGAAGTTAAAAAACTGGGAGGCTACTTACAGT
TATTTTTGCAGGGATGCTGTTCAGATATTTCATCTATGGTAAAAGAGGCTCACAAGAAAGTGATCCAGAG
TGTACAACAAGCTGTAAAGTGTGTGGGACAGCTAGCAGTTTTGAAAGGGAGCAAGCTACATTTTCTGGAA
AACAGTGACGATGAAGCTGCCAGCATCCAGGAAGAACTTATGGATGCTATTTGTGATGGAGTATGCTTAG
GAATGAATGTGCTATTAGATTCTGGACTAGAAAAAGCAAAAGAACTTCAGCATGAACTCTTAAAGCAGAG
TACACAAAATGAGGTTACCAAACAGATTAAAGCAAATGCCATGGGATTGATTAGATCTCTTGAAAATATC
TTTGCTGAATGGAAAATAAAAGGTTTCAGAACTCGAGTGCAAGAAGATAATTCTGGATACCGATATTCCA
TGAAGATAGTTAATCTTGCACCAGAATTCTTGAAGTTAAAACATTGCTTAGAGAAAACTATTCAAATTAT
TATTTCTTCACTGAGAGGATGCAACAGTGATGAAAATCTCAGTAATTATGTTGAAAGTATTTGCAACTTG
GCCAGTGATTTTCACAATGGCTTCAGTGTGTCCCCCGCTTCTGTTGACAACTCTGAGAATGGAGTACCTC

AAAGTGGCCACAAGGAACTGGAATCTGTGGCTAAGTCACTCCTCTTATGTTTTGAATCTGAAGAAAGACC
TGATTTGGTGAAACCTTGGGAAACTTGTAATCAAAATGCTAGAGAAGAAGAACAACAATCTAAATCCAGC
AAGACAGACTGCAGTAAGAATAAAGGTGTGCCAAAGCGTGTCTATGAACTCCATGGTTCATCCCCAGCGG
CGAGCTCAGAAGAATGCACACCCAGCAGGATTCAGTGGGTG
>Chlorocebus sabaeus XM_007989026.1
ATGTCATTACACAGTACTCATAATAGAAATAACCGCGGCGATATTCTTGATATTCCTTCTTCCCAAAATA
GTTCATCACTGAATGCCCTCACCCACAGTAGCCGACTTAAGCTGCATTTGAAGTCAGATATGTCAGAATG
TGAAAATTATGATCCATTATTGAGATCTGCAGGTAAAGTCAGAGACATAAATAGCACTTACGTTATTTCT
GCCAGTGAAAAAACAGCAGACATGCCCCTTACCCCTAATCCTGTAGGTAAATTGACACTTCAGAGAAGAA
CTACAAGGAACAAAGAATCATCTTTGCTTGGTAGGGATTTGGAAGACACAACTGAAAAAACAGCAGAAAC
ACATCTTACATTACAACGTCGTGCTAAAACAGATTCTGAAGAAAAGTGGAAAACAGCTGAAACAGATTCT
GTCAAAATGACACTGAATGTGGGAGGTGAAACAGAAAATAATGGTGTTTCTAAGGAAAGTAGAACAAATG
TAAAGATTGTAAATAATGCTAAAAACTCTTTTGTTGCCTCTTGTGTACCTTTAGATAAAGATCCACAAGT
CATTGAAATGATGGCTGATAAGAAATACAAAGAAATATTTTCTCCCCCCAGTAGAGCAAATGAAAATATT
GCACTTAAGTACTCAAGTAATAGAGCACCCATTGCTTCCCTGAGTCAGACTGAAGTTGTTAGATCAGGAC
ACTTGACAACAAAACCTACTCAGAGCAAGTTGGATATCAAAGTGTCGGGAACAGGAAACTTGCATCATAG
AACTATTGGGAAGGAAATTGCAAAAACTTCAAATAAATTTGGGAGCTTAGAAAAAAGAACACCTACGAAA
TGTACAACAGAACACAAATTGACACCAAAGTGCGGTCTGCCTCAGCTTAAGAGCCCAGCTCCATCAATAC
TGAAGAATAGAATGTCTAACCTTCAAGTTAAACAAAGACCAAAAAGTTCCTTTCTTGCGAATAAACAGGA
AAGGTCAGCAGAAAATACAATCCTTCCTGAAGAAGAAACTGTAGTTCAGAACACCTCTGCAGGAAAAGAC
CCCTTAAAAGTAGAGAATAGTCAAGTGACAGTGGCAGTACGCGTAAGGCCTTTCACTAAGAGAGAGAAGA
TTGAAAAAGCATCCCAGGTGGTCTTCATGAGTGGGAAAGAAATAACTGTGGAACATCCTGACATGAAACA
AGTTTATAGTTTTATTTATGATGTTTCATTCTGGTCTTTTGATGAATGTCATCCTCACTATGCTAGCCAG
ACAACTGTCTATGAGAAGCTAGCAGCACCACTCCTAGAAAGAGCCTTTGAAGGCTTCAATACCTGTCTTT
TTGCTTATGGTCAGACTGGCTCTGGAAAATCATATACGATGATGGGATTTAGTGAAGAACCAGGAATAAT
TCCAAGATTTTGTGAAGATCTTTTTTCTCAAGTAGCCAGAAAACAAACCCAAGAGGTCAGCTATCACATT
GAAATGAGCTTCTTTGAAGTATATAATGAAAAAATTCATGATCTTCTGGTTTGTAAAGATGAAAATGGGC
AGAGAAAGCAACCACTGAGAGTGAGGGAACATCCTGTTTATGGACCATATGTTGAAGCACTGTCAATGAA
TGTTGTCAGTTCTTACACTGATATCCAGAGTTGGCTAGAATTGGGAAATAAACAAAGAGCTACTGCTGCT
ACTGGGATGAATGATAAAAGCTCCCGATCTCATTCAGTTTTCACCCTGGTGATGACCCAGACCAAGACAG
AATTTGTGGAAGGGGAAGAACATGATCACAGAATAACAAGTCGAATTAACTTAATAGATCTGGCAGGCAG
TGAGCGCTGCTCTACGGCTCACACTAGTGGAGATCGACTAAAGGAAGGTGTGAGTATTAATAAGTCCTTG
CTAACTTTGGGAAAAGTTATATCTGCACTCTCTGAACAAGCAAACCAAAAGAGAGTTTTTATTCCTTATC
GTGAATCTGTTCTTACATGGCTGTTAAAAGAAAGTCTGGGTGGAAATTCAAAAACTGCAATGATTGCTAC
GATTAGTCCTGCTGCCAGCAACGTAGAAGAAACATTAAGCACACTTAGATATGCTAACCAAGCCCGTTTA
ATAGTCAACATTGCCAAAGTAAATGAAGATATGAATGCCAAGTTAATTAGAGAATTGAAGGCAGAAATTG
CAAAGCTAAAAGCTGCTCAGCGAAACAATCGGAATATTGACCCTGAACGATACAGGCTCTGTCGGCAAGA
AATAACATCCTTAAGAATGAAACTGCATCAACAGGAGAGAGACATGGCAGAAATGCAAAGAGTGTGGAAA
GAAAAGTTTGAACAAGCTGAAAAAAGAAAACTTCAAGAAACGAAAGAGTTACAGAAAGCAGGAATTACAT
TTCAAATGGACAATCATTTACCAAACCTTGTTAATCTGAATGAAGATCCACAACTATCAGAGATGCTGCT
ATATATGATAAAAGAAGGAACAACTACAGTTGGAAAGTATAAACCAAACTCAAGCCATGATATTCAGTTA
TCTGGTGTGCTGATTGCTGATGATCATTGTACTATCACAAATTTAGGTGGGACAGTGAGTATTATCCCAC
TTGGGGAAGCAAAGACATACGTAAATGGAAAACTTATTTTGGAACTCACAGTATTACATCATGGTGATCG
AGTGATTCTTGGTGGAGATCATTATTTTAGATTTAATCATCCAGTAGAAGTCCAGAAAGGAAAAAGGCCA
TCTGGAAGAGATACTCCTATAAGTGAGGGTCCAAAAGACTTTGAATTTGCAAAAAATGAGTTGCTCATGG
CACAGAGATCACAACTTGAAGCAGAAATAAAAGAGGCGCAGTTGAAGGCAAAGGAAGAAATGATGCAAGG
AATCCAGATTGCAAAAGAAATGGCTCAGCAAGAGCTTTCTTCTCAAAAAGTTGCATATGAAAGCAAAATA

AAAGCACTGGAAGCAGAACTGAGAGAAGAGTCTCAAAGGAAAAAAATGCAGGAAATAAATAACCAGAAGG
CTAATCACATAATTGAGGAATTAGAAAAGGCAAAGCAGCATCTTGAACAAGAAATATATGTCAACAAAAA
GCGATTAGAAATGGAGACTTTGGCTACAAAACAGGCTTTAGAAGACCATAGCATCCGCCATGCAAGAATT
CTGGAAGCTTTAGAAACTGAAAAGCAAAAAATTGCTAAAGAAGTACAAATTCTACAGCAGAATCGGAGTA
ATAGGGATAAAACTTTTACAATGCAGACAACTTGGAGCTCTATGAAACTCTCAATGATGATTCAGGAAGC
CAATGCGATCAGCAGCAAACTGAAAACATACTATGTTTTTGGCAGACATGATGTATCAGATAAAAGTAGT
TCTGACACTTCTATTCGGGTTCGTAACCTGAAACTAGGAATCTCAACATTCTGGAGTCTGGAAAAGTTTG
AATCTAAACTTGCAGCAATGAAAGAACTTTATGAGAGTAATGGTAGTAACAGAGATGAAGATGTCTTTTG
TGATCCTGAAGATGAATGGGAACCTGACATTACAGATGCACCAGTTTCTTCACTTTCTAGAAGGAGGAGC
AGGAGTTTGATGAAGAACAGAAGAATTTCTGGTTGTTTACATGACATACAAGTTCACCCAATTAAGAAGT
TGCATTCTTCACATTCATCAGGTTTAATGGACAAATCAAGCACCATTTACTCAAATTCAGCAGAGTCATT
TCTTCCTGGAATTTGCAAAGAATTGATTGGTTCTTCATTAGATTTTCTTGGACAGAGTTATGATGAAGAA
AGAACTATAGCAGACAGCCTAATGAATAGTTTTCTTAAAATTTATAATGGGCTATTTGCCATTTCCAAGG
CTCATGAAGAACAAGATGAAGAAAGTCAAGATAACTTGTTTTCTTCTGATCGAGCAATCCAGTCACTTAC
CATTCAGACTGCATGTGCTTTTGAGCAGCTAGTAGTGCTAATGAAACACTGGCTGAGTGATATAGTGCCT
TGTACCACCAACATAGCAAGACTTGAGGATGAATTGAGACAAGAAGTTAAAAAACTGGGAGGCTACTTAC
AGTTATTTTTGCAGGGATGCTGTTCGGATATTTCATCAATGATAAAAGAGGCTCAAAAGAATGCAATCCA
AATTGTACAACAAGCTGTAAAGTATGTGGGGCAGTTAGCAGTTCTGAAAGGGAGCAAGCTACATTTTCTA
GAAAATGGTAACAATAAAGCTGCCCGTGTCCAGGAGGAATTCATGGATGCTATTTGTGATGGTGTAGGCT
TAGGAATGAAGATTTTATTAGATTCTGGACTTGAAAAAGCAAAAGAACTTCAACATGAACTCTTAAGACA
GTGTACAAAAAATGAGGTTACCAAAGAAATGAAAACTAATGCCATGGGATTGATTAGATCTCTTGAAAAC
ATCTTCGCCGAATCGAAAATTAAAAGTTTCCGAAGACAAGTACAAGAAGAAAACTTTGAATACCAAGATT
TCAAGAAGATGGCTAATTGTGCTCCAGAATTCTTAAAGTTAAAACATTGCTTAGAGAAAGCTATTGAAAT
TATTATTTCTGCACTGAAAGGATGCAGTAGTGATGTAAATCTTCTCCAGAATTGTGTTGAAAGTATTCGC
AACTTGGCCAGTGATTTTTACAGTGACTTCGGTGTGCCTTCTACTTCTGTTGACAGCTATGAGAGTAGAG
TAACTAATGTTGTCCACAAGGACCTAGAATCTCTAGCTAAGTCTCTCCTCTTTTGTTTTGAATCTGAAGA
AAGCCCTGATTTGTTGAAACCTTGGGAAACTTATAATCAAAATACCAAAGAAGAATACCAACAATCTAAA
TCAAGTGGGATTGATGGCAGCAAGAATAAAGGTGTACCAAAGCGTGTCTATGAGCTTCATGGCTCATCCC
CAGCAGTGAGCCCAGAGGAATGCACACCCAGTAGGATTCAGTGGGTG

>Nomascus leucogenys XM_003264524.3
ATGTCATTACACAGTACTCATAATAGAAATAACAGCAGTGATATTCTTGATATTCCATCTTCCCAAAATA
GTTCATCACTGAATGCCCTCACCCACAGTAGCCGACTTAAGCTGCATTTGAAGTCGGATATGTCAGAATG
TGAAAATGATGATCCATTATCGAGATCTGCAGGTAAAGTCAGAGACATAAATAGAACTTATGTTATTTCT
GTCAGTAAAAAAACAGCAGACATGCCCCTTACCCCTAATCCTGTAGGTAGATTGGCACTTCAGAGAAGAA
CTACAAGGAACAAAGAATCATCTTTGCTTGGTAGTGAGTTGGAAGACACAGCTGAAAAAATAGCAGAAAC
ACGCCTTACATTACAACGTCGTGCTAAAACAGATTATGCAGAAAAGTGGAAAACAGCTGAAACAGATTCT
GTCAAAATGACACTGAATGTGGGAGGTGAAACAGAAAATAATGGTGTTTCTAAGGAAAGTAGAACAAATG
TAAGGATTGTAAATAATGCTAAAAACTCTTTTGATGCTTCTTCTGTACCTTTAGATGAAGATCCACAAGT
CATTGAAATGATGGCTGATAAGAGATACAAAGAAACATTTTCTGCCCCCAGTAGAGCAAATGAAAATGTT
GCACTTAAGTACTCAAGTAATAGAGCACCCGCTGCTTCCCTGAGTCAGACTGAAGTTGTTAGATCAGGAC
ACTTGACAACGAAACCTACTCAGAGCAAGTTGGATATCAAAGTGTTGGGAACAGGAGACTTGTATCATAG
AAGTATTGGGAAGGAAATTGCAAAAACTTCAAATAAATTTGGGAGCTTAGAAAAAAGAACACCTACAAAA
TGTACACTAGAACACAAATTGACACCAAAGTGCGGCCTGCCTCAGGTTAAGAGCCCAGCTCCATCAATAC
TGAAGAAAAGAATGTCTAACCTTCAAGTTAAACAAAGACCAAAAAGTTCTTGTCTTGCAAATAAACAGGA
AAGGTCCGCAGAAAATACAATCCTTCCCGAAGAAGAAACTGTAGTTCAGAACATCTCTGCAGGAAAAGAC
CCCTTAAAAGTAGAGAATAGTCAAGTGACAGTGGCAGTACGCGTAAGACCTTTCACCAAGAGAGAGAAGA

TTGAAAAAGCATCCCAGGTGGTCTTCATGAGTGGGAAAGAAATAACTGTGGAACATCCTGACATGAAACA
AATTTATAATTTTATTTATGATATTTCATTCTGGTCTTTTGATGAATGTCATCCTCACTACGCTAGCCAG
ACGACTGTCTATGAGAAGCTAGCAGCACCACTCCTAGAAAGAGCCTTCGAAGGCTTCAATACCTGTCTTT
TTGCTTATGGTCAGACTGGCTCTGGAAAATCATATACGATGATGGGATTTAGTGAAGAACCAGGAATAAT
TCCAAGATTTTGTGAAGATCTTTTTTCTCAAGTAGCCGGAAAACAAACCCAAGAGGTCAGCTATCACATT
GAAATGAGCTTCTTTGAAGTATATAATGAAAAAATTCACGACCTTCTGGTTTGTAAAGATGAAAATGGGC
AGAGAAAGCAACCACTGAGAGTGAGGGAACATCCTGTTTATGGACCATATGTTGAAGCACTGTCAATGAA
CATTGTCAGTTCTTACGCTGATATCCAGAGTTGGCTAGAATTGGGAAATAAACAAAGAGCTACTGCTGCT
ACTGGTATGAATGATAAAAGCTCCCGATCTCATTCGGTTTTCACCCTGGTGATGACCCAGACCAAGACAG
AATTTGTGGAAGGGGAAGAACACGATCACAGAATAACAAGTCGAATTAATCTAATAGATCTGGCAGGCAG
TGAGCGCTGCTCTACAGCTCACACTAGTGGAGATCGACTAAAGGAAGGTGTGAGTATTAATAAGTCCTTG
CTAACTTTGGGAAAAGTTATATCTGCACTCTCGGAACAAGCAAACCAAAGGAGAGTTTTTATTCCTTATC
GTGAATCTGTTCTTACATGGCTGTTAAAAGAAAGTCTGGGTGGAAATTCAAAAACTGCAATGATTGCTAC
GATTAGTCCCGCTGCCAGCAACATAGAAGAAACATTAAGCACACTTAGATATGCTAACCAAGCCCGTTTA
ATAGTCAACATTGCCAAAGTAAATGAAGATATGAACGCTAAGTTAATTAGAGAATTGAAGGCAGAAATTG
CAAAGCTAAAAGCTGCTCAGAGAAACAATCGGAATATTGACCCTGAACGATACAGGCTGTGTCGGCAAGA
AATAACATCCTTAAGAATGAAACTGCATCAACAGGAGAGAGACATGGCAGAAATGCAAAGAGCATGGAAA
GAAAAGTTTGAACAAGCTGAAAAAAGAAAACTTCAAGAAACAAAAGAGTTACAGAAAGCAGGAATTACAT
TTCAAATGGACAATCATTTACCAAACCTTGTTAATCTGAATGAAGATCCACAACTATCTGAGATGCTGCT
ATATATGATAAAAGAAGGAACAACTACAGTTGGAAAGTATAAACCAAACTCAAGTCATGATATTCAGTTA
TCTGGGGTGCTGATTGCTGATGATCATTGTACCATCAAAAATTTTGGTGGGACAGTGAGTATTATCCCAG
TTGGGGAAGCAAAGACATATGTAAATGGAAAACATATTTTGGAAATCACAGTATTACATCATGGTGATCG
AGTGATTCTTGGTGGAGATCATTATTTTAGATTTAATCATCCAGTAGAAGTCCAGAAAGGGAAAAGGCCA
TCTGGAAGAGATACTCCTACAAGTGAGGGTCCAAAAGACTTTGAATTTGCAAAAAATGAGTTGCTCATGG
CACAGAGATCACAACTCGAAGCAGAAATAAAAGAGGCTCAGTTGAAGGCAAAGGAAGAAATGATGCAAGG
AATCCAGATTGCAAAAGAAATGGCTCAGCAAGAGCTTTCTTCTCAAAAAGCTGCATATGAAAGCAAAATA
AAAGCACTGGAAGCAGAACTGAGAGAAGAGTCTCAAAGGAAAAAAATGCAGGAAATAAATAACCAGAAGG
CTAATCACAAAATTGAGGAATTAGAAAAGGCAAAGCAGCATCTTGAACAGGAAATATATGTCAACAAAAA
GCGATTAGAAATGGAGACTTTGGCTACAAAACAGGCTTTAGAAGACCATAGCATCCGTCATGCAAGAATT
CTGGAAGCTTTAGAAACTGAAAAGCAAAAAATTGCTAAAGAAGTACAAATTCTACAGCAGAATCGGAGTA
ATAGGGATAAAACTTTTACAATGCAGACAACTTGGAGCTCTATGAAACTCTCAATGATGATTCAGGAAGC
CAATGCTATCAGCAGCAAATTGAAAACATACTATGTTTTTGGCAGACATGATATATCAGATAAAAGTAGT
TCTGACACTTCTATTCGGGTTCGTAATCTGAAACTAGGAATCTCAACATTCTGGAATCTGGAAAAGTTTG
AATCTAAACTTGCAGCAATGAAAGAACTTTATGAGAGTAATGGTAGTAACGGGGGTGAAGATGTCTTTTG
TGATCCTGAAGATGAATGGGAACCCGACATTACAGATGCACCAGTTTCTTCACTTTCTAGAAGGAGGAGT
AGGAGTTTGATGAAGAACAGAAGAATTTCTGGTTGTTTACATGACATACAAGTCCATCCAATTAAGAATT
TGCATTCTTCACATTCATCAGGTTTAATGGACAAATCAAGCACTATTTACTCAAATTCAGCAGAGTCATT
TCTTCCTGGAATTTGCAAAGAATTGATTGGTTCTTCATTAGATTTTCTTGGACAGAGTTATGATGAAGAA
AGAACTATAGCAGACAGCCTAATTAATAGTTTTCTTAAAATTTATAATGGGCTATTTGCCATTTCCAAGG
CTCATGAAGAACAAGATGAAGAAAGTCAAGATAACTTGTTTTCTTCTGATCGAGCAATCCAGTCACTTAC
TATTCAGACTGCATGTGCTTTTGAGCAGCTAGTAGTGCTAATGAAACACTGGCTGAGTGATTTACTGCCT
TGTACCAACATAGCAAGACTTGAGGATGAATTGAGACAAGAAGTTAAAAAACTGGGAGGCTACTTACAGT
TATTTTTGCAGGGATGCTGTTCGGATATTTCATCAATGATAAAAGAGGCTCAAAAGAATGCAATCCAAGT
TGTACAACAAGCTGTAAAGTATGTGGGGCAGTTAGCAGTTCTGAAAGGGAGCAAGCTACATTTTCTAGAA
AACGGTAACAATAAAGCTGCCAGTGTTCAGGAGGAATTCATGGATGCTGTTTGTGATGGTGTAGGCTTAG
GAATGAAGATTTTATTAGATTCCGGACTGGAAAAAGCAAAAGAACTTCAGCATGAACTCTTCAGGCAGTG
TACAAAAAATGAGGTTACCAAAGAAATGAAAACTAATGCCATGGGATTGATTAGATCTCTTGAAAACATC

TTTGCTGAATTGAAAATTAAAAGTTTCAGAAGGCAAGTACAAGAAGAAAACTTTGAACACCAAGATTTCA
AGAAGATGGTTAATCATGCTCCAGAATTCTTAAAGTTAAAACATTGCTTAGAGAAAGCTATTGAAATTAT
TATTTCTGCACTGAAAGGATACAGTAGTGATGAAAATCTTCTCCAGACTTGTGTTGAAAGTATTCGCAAC
TTGGCCAGTGATTTTTACAGTGACTTCAGTGTGCCTTCTACTTCTGTTGACGGCTATGAGAGTAAAGTAA
CTCACGTTGTACACCAGGAACTAGAATCTCTAGCTAAGTCTCTCCTCTTTTGTTTTGAATCTGAAGAAAG
CCCTGATTTGTTGAAACCCTGGGAAACTTATAATCAAAATACCAAAGGAGAACACCAACAATCTAAATCA
AGCGGGATTGATGGCAGTAAGAATAAAGGGGTACCAAAGCGTGTCTATGAGCTCCATGGCTCATCCCCAG
CAGTGAGCTCAGAGGAATGCACACCCAGTAGGATTCAGTGGGTG
>Callithrix jacchus ENSCJAT00000009971.2
ATGTCGTTACACACTACTTATAATAGAAATAACAGCGGCGATATTCTTGATATTCCTTCT
TCCCAAAATAGTTCGTCACTGAATACCCTCACCCACAATAGCCGACTTAAGCTGCATTTG
AAGTCGGATATGTCAGAATGTGAAAATGATGATCCATTATTGAGATCTGCAGGTAGAGTC
AGAGACATAAATAGCACTTATGTTATTTCTGCCAGTAAAAAAACAGGAGACATGCCCCTT
ACCCCTAACCCTGTAGGCAGATTGTCACTTCGGAGAAGAACTACAAGGAACAAAGAATCA
TCTTTGCTTGCTAGTGAGTTGGAAGACACAACTGAAAAAACAGCAGAAACATGTCTTACA
TTACAACGTCGTGCTAAAACAGATTCTGCAGAAAAGTGGAAAACAGCTGAAACAGATTCT
GTCAATAAGTGGCAAATGACACCGAATGTGGGAGGCAAAGCAGAAAATAATTGTGTTTCT
AAGGAAAGTAGAACAAATGTAAAGATTGTAAATAATGCTAAAAACGTTTTTGTGGCTTCT
TCTGTACCTTTAGATGAAGACTCAAAAGTCATTGAAATAATGGCTAATAAGAAATACAAA
GAAACATTTTCTGCCCCCAGTAGGGCAAAAGAAAATGTTGCGCTTAAGTATTCGAGTAAT
AGAGCACCCATTGCTTCCTTGAGCCAGACTGAAGTTGTTAGATCAGGACACTTGGCAACG
AAACCTACTCAGAGCAAGTTGGATATCAAAGGAACAGGAAACTTGTATCAGAGAAGTATT
GGGAAGGAAATTGCAAAAACTTCAAATAAATTTGGGAGCTTAGAAAAGAGAACACCTACC
AAATGTATAACAGAACACAAATTGACACCAAAGTGCAACACACCTCAGCTTAAGAGCCCA
GCTCCATCAATGCTGAAGAATAGAATATCTAACCTTCAAGTTAACCAGAGACCAAAAAGT
TCTCTTCTTGCAAATAAACAGGAAAGGTCAGCAGAAAATACAGTCCTTCCTGAAGAAGAA
ACTGCAGATCAGAACACCTCTGCAGACAAAGACCCCTTAAAAGTAGAGAATAGTCAAGTG
ACAGTGGCAGTACGTGTAAGGCCTTTCACCAACAGAGAGAAGATTGAAAAAGCATCCCAG
GTAGTCTTCATGAATGGGAAAGAGCTAACTGTGGAACATCCTGACATGAAACAAGTTTAT
AATTTTATTTATGATGTTTCATTTTGGTCTTTTGATGAATGTCATCCTGACTATGCTAGC
CAGACAACTGTCTACGAGAAGCTAGCAGCACCACTCCTAGAAAGAGCCTTTGAAGGCTTC
AATACCTGTCTTTTTGCTTATGGTCAGACTGGCTCTGGAAAATCCTATACGATGATGGGA
TTTAGTGAAGAACCAGGAATAATTCCAAGATTTTGTGAAGATCTTTTTTCTCAAGTAGCC
AGAAAACAAACCCAAGAGGTGACCTATCACATTGAAATGAGCTTCTTTGAAGTATATAAT
GAAAAAATTCATGACCTTCTGGTTTGCAAAGGTGAAAGTGGACAGAAAAAGCAACCACTG
AGAGTGAGGGAGCATCCCGTTTATGGACCGTATGTTGAAGCACTGTCAATGAATGTTGTC
AGTTCTTATGCTGATATCCAGAGTTGGCTAGAACTGGGAAATAAACAGAGAGCTACTGCT
GCTACTGGTATGAATGATAAAAGCTCCCGATCTCATTCAGTTTTCACCCTGGTGATGACC
CAGACCAAGACAGAGTTTGTGGAAGGGGAAGAACACGATCACAGAATAACGAGTCGAATT
AACCTAATAGATCTGGCAGGCAGTGAGCGCTGCTCTGTGGCTCACACTAGTGGAGATCGA
CTAAAGGAAGGTGTGAGTATTAATAAGTCCTTGCTAACTTTGGGAAAAGTTATATCTGCA
CTCTCTGAACAAGCAAACCAAAAGAGAGTTTTTATTCCTTATCGTGAATCTGTTCTTACA
TGGCTGTTAAAAGAAAGTCTGGGTGGAAATTCAAAAACTGCAATGATTGCTACGATTAGT
CCTGCTGCCAGCAACATAGAAGAAACATTAAGCACACTTAGATATGCTAACCAAGCCCGT
TTAATAGTTAACATTGCCAAAGTAAATGAAGATATGAATGCTAAGTTAATTAGAGAATTG
AAGGCAGAAATTGAAAAGCTAAAAGCTGCTCAGAGAAACAATCGGAATGTTGACCCTGAA
CGATACAGGCTCTGTCGGCAAGAAATAACATCCTTAAGAATGAAACTGCATCAACAGGAG

AGAGACATGGCAGAAATGCAAAGAGTATGGAAAGAAAAGTTTGAACAAGCTGAAAAAAGA
AAGCTTCAAGAAACCAAAGAGTTACAGAAAGCAGGAATTACATTTCAAATGGACAATCAT
TTACCAAACCTTGTTAATCTTAATGAAGATCCACAACTATCAGAAATGCTGCTATATATG
ATAAAAGAAGGAACAACTACAGTCGGAAAGTATAAACCAAACTCAAGCCATGATATTCAG
TTATCTGGGGTGCTGATTGCCGATGATCATTGTACTATCAAAAATTTTGGTGGAACAGTG
AGTATTATCCCAGTTGGGGAAGCAAAGACATACATAAATGGAAAATGTATTTTGGAACTC
ACAGTATTACATCATGGTGATCGAGTGATTCTTGGTGGAGATCATTATTTTAGATTTAAT
CATCCAGTAGAAGTCCAGAAAGGAAGAAGTCCATCTGGAAGAGACACTCTTATAAGTGAG
GGTCCAAAAGACTTCGAATTTGCAAAAAATGAGTTGCTCATTGCACAAAGATCACAACTC
GAAGCAGAAATAAAAGAGGCGCAGTTGAAGGCCAAGGAAGAAATGATGCAAGGAATCCAG
ATTGCAAAAGAAATGGCTCAGCAAGAGCTTTCTTCTCAAAAAGCTGCATATGAGAACAAA
ATAAAAGCACTGGAAGCAGAACTGAGAGAAGAGTCTCAAAGGAAAAAAATGCAGGAAATA
AATAACCAGAAGGCTAATCACAAAATTGAGGAATTAGAAAAGGCAAAGCAGCATCTTGAA
CAGGAAATATATGTCAACAAAAAGCGATTAGAAATGGAGACTTTGGCTACAAAACAGGCT
TTAGAAGACCATAGCATCCGCCATGCAAGAATTCTGGAAGCTTTAGAAACTGAAAAGCAA
AAAATTGCTAAAGAAGTACAAATTCTACAGCAGAATCGGAGTAATAGGGATAAAACTTTT
ACAACGCAGACAATTTGGAGCTCTATGAAACTCTCAATGATGATTCAGGAAGCCAATGCT
ATTAGCAGCAAATTGAAAGCATACTATGTTTTTGGCAGACATGATGTATCAGATAAAAGT
AGTTCTGGCACTTCTATTAGGGTTCGTAACCTAAAACTAGGAATCTCAACATTCTGGAGT
CTGGAAAAGTTTGAATACAAACTTGCAGCAATGAAAGAACTTTATGAGAGTAATGGTAGT
AACAGGGGTGAAGATGTCTTTTGTGATCCTGAGGACGAATGGGAACCCGACATTACAGAT
GCAGCAGTTTCTTCACTTTCTAGAAGGAGGAGCAGGAGTTTGATGAAGAATGGAAGAATT
TCTGGTTGTTTACATGATATACAAGTCCACCCAATTAAGAATTTGCATTCTTCACATTCA
TCAGGTTTAATGGACAAATCAAGCAGCATTTACACAAATTCGGCAGAGTCATTTCTTCCT
GGAATTTGCAAAGAATTGATTGGTTCTTCATTAGATTTTCTTGGACAGAGTTATGATGAA
GAAAGAACTATAGCAGATAGCCTAATGAATAGTTTTCTTAAAATTTCTAATGGGCTACTT
GCCATTTCCAAGGCTCATGAAGAAGAAGATGAAGAAAGTCAAGATAACTTGTTTTCTTCT
GATCGAGCAACCCAGGCACTTACTATTCAGACTGCATGTGCTTTTGAGCAGCTGGTAGTG
CTAATGAGACACTGGCTGAATGATTTACTGCCTTGTACCAACATAGCAAGACTTGAGGAT
GAATTGAGACAAGAAGTTAAAAAACTGGGAGGCTACTTACAGTTATTTTTACAGGGATGC
TGTTTGGATATTTCCTCAATGATAAAAGAGGCCCAAAAGAATGCAATCCACACTGTACAA
CAAGCTGTAAAGTATGTGGGGCAGTTAGCAGTTCTGAAAGGGAGCAAGCTACATTTTCTG
GAAAACAGTAACAATAAAGCTGGCAGCGTCCAGAGGAGGAATCAGGATGCTATTTGTGAT
GGTGTAGGCTTGGGAATGAAGATTTTATTAGATTCTGGACTAGAAAAAGCAAAAGAACTT
CAGCATGAACTCTTAAGGCAATGTACAAAAAATGAGGTTACCAAAGAAATGAAAACAAGT
GCCATGGGACTGATTAGATCTCTTGAAAACATCTTTGCTGAATTGAAAATTAAAAGTTTC
AGAAGGCAAGTACAAGAAGAAAACTTTGGATACCAAGATTTCAAGAAGATGGCTAATTGT
GCTCCAGAATTCTTAAAGTTAAAACATTGCTTAGAGAAAACTATTGAAGTTGTTATTTCT
GCACTAAAAGGATGCAGTGGTGATGTAAACCTTCTCCAGGATTGTGTTGAAAGTATTTGC
AACTTGGCCAGTGATTTTTACAGAGACCTCAGTGTGCCCTCCACTTCTGTTGAGAGCTAT
GAGGGTAGAGTAACTCACGTTGTCCACAAGGAACTAGAATCTTTAGCTAAGTCTCTTCTC
TTTTGTTTTGAATCTGAAGATAGACCTGATTTGTTGAAACCCTGGGAAACTTATAATCAA
AATACCGAAGAAGAACACCAACAATCTAAATCAAGTGGGATTGACGGCAGTAAGAATAAA
GGTGTACCAAAGCGTGTCTATGAACTCCATGGCTCAGCCCCAGCAGTGGGCTCAGAGGAA
TGCACACCCAGTAGGATTCAGTGGGTG
>Pan paniscus XM_003823058.3
ATGTCATTACACAGTACTCATAATAGAAATAACAGCGGTGATATTCTTGATATTCCTTCTTCCCAAAATA

GTTCATCACTGAATGCCCTCACCCACAGTAGCCGACTTAAGCTGCATTTGAAGTCGGATATGTCAGAATG
TGAAAATGATGATCCATTATTGAGATCTGCAGGTAAAGTCAGAGACATAAATAGAACTTATGTTATTTCT
GCCAGTAAAAAAACAGCAGACATGCCCCTTACCCCTAATCCTGTCGGTAGATTGGCACTTCAGAGGAGAA
CTACAAGGAACAAAGAATCATCTTTGCTTGTTAGTGAGTTGGAAGACACAACTGAAAAAACAGCAGAAAC
ACGTCTTACATTACAACGTCGTGCTAAAACAGATTCTGCAGAAAAGTGGAAAACAGCTGAAATAGATTCT
GTCAAAATGACACTGAATGTGGGAGGTGAAACAGAAAATAATGGTGTTTCTAAGGAAAGTAGAACAAATG
TAAGGATTGTAAATAATGCTAAAAACTCTTTTGTTGCCTCTTCTGTACCTTTAGATGAAGATCCACAGGT
CATTGAAATGATGGCTGATAAGAAATACAAAGAAACATTTTCTGCCCCCAATAGAGCAAATCAAAATGTT
GCACTTAAGTACTCAAGTAATAGACCACCCATTGCTTCCCTGAGTCAGACTGAAGTTGTTAGATCAGGAC
ACTTGACAACGAAACCTACTCAGAGCAAGTTGGATATCAAAGTGTTGGGAACAGGAAACTTGTATCATAG
AAGTATTGGGAAGGAAATTGCAAAAACTTCAAATAAATTTGGGAGCTTAGAAAAAAGAACACCTACAAAA
TGTACAACAGAACACAAATTGACACCAAAGTGCAGCCTGCCTCAGCTTAAGAGCCCAGCTCCATCAATAC
TGAAGAATAGAATGTCTAACCTTCAAGTTAAACAAAGACCAAAAAGTTCCCTTCTTGCAAATAAACAGGA
AAGGTCCGCAGAAAATACAATTCTTCCCGAAGAACAAACTGTAGTTCAGAACACCTCTGCAGGAAAAGAC
CCCTTAAAAGTAGAGAATAGTCAAGTGACAGTGGCAGTACGCGTAAGACCTTTCACCAAGAGAGAGAAGA
TTGAAAAAGCATCCCAGGTAGTCTTCATGAGTGGGAAAGAAATAACTGTGGAACACCCTGACATGAAACA
AGTTTATAATTTTATTTATGATGTTTCATTCTGGTCTTTTGATGAATGTCATCCTCACTACGCTAGCCAG
ACAACTGTCTATGAGAAGCTAGCAGCACCACTCCTAGAAAGAGCCTTCGAAGGCTTCAATACCTGTCTTT
TTGCTTATGGTCAGACTGGCTCTGGAAAATCATATACGATGATGGGATTTAGTGAAGAACCAGGAATAAT
TCCAAGATATTGTGAAGATCTTTTTTCTCAAGTAGCCAGAAAACAAACCCAAGAGGTCAGCTATCACATT
GAAATGAGCTTCTTTGAAGTATATAATGAAAAAATTCACGACCTTCTGGTTTGTAAAGATGAAAATGGGC
AGAGAAAGCAACCACTGAGAGTGAGGGAACATCCTGTTTATGGACCATATGTTGAAGCACTGTCAATGAA
CATTGTCAGTTCTTACGCTGATATCCAGAGTTGGCTAGAATTGGGAAATAAACAAAGAGCTACTGCTGCT
ACTGGTATGAATGACAAAAGTTCCCGATCTCATTCAGTTTTCACCCTGGTGATGACCCAGACCAAGACAG
AATTTGTGGAAGGGGAAGAACACGATCACAGAATAACAAGTCGAATTAACCTAATAGATCTGGCAGGCAG
TGAGCGCTGCTCTACGGCTCACACTAATGGAGATCGACTAAAGGAAGGTGTGAGTATTAATAAGTCCTTG
CTAACTTTGGGAAAAGTTATATCTGCACTTTCGGAACAAGCAAACCAAAGGAGTGTTTTTATTCCTTATC
GTGAATCTGTTCTTACATGGCTGTTAAAAGAAAGTCTGGGTGGAAATTCAAAAACTGCAATGATTGCTAC
GATTAGTCCCGCTGCCAGCAACATAGAAGAAACATTAAGCACACTTAGATATGCTAACCAAGCCCGTTTA
ATAGTCAACATTGCTAAAGTAAATGAAGATATGAACGCTAAGTTAATTAGAGAATTGAAGGCAGAAATTG
CAAAGCTAAAAGCTGCTCAGAGAAACAATCGGAATATTGACCCTGAACGATACAGGCTCTGTCGGCAAGA
AATAACATCCTTAAGAATGAAACTGCATCAACAGGAGAGAGACATGGCAGAAATGCAAAGAGTGTGGAAA
GAAAAGTTTGAACAAGCTGAAAAAAGAAAACTTCAAGAAACAAAAGAGTTACAGAAAGCAGGAATTATGT
TTCAAATGGACAATCATTTACCAAACCTTGTTAATCTGAATGAAGATCCACAACTATCTGAGATGCTGCT
ATATATGATAAAAGAAGGAACAACTACAGTTGGAAAGTATAAACCAAACTCGAGCCATGATATTCAGTTA
TCTGGGGTGCTGATTGCTGATGATCATTGTACTATCAAAAATTTTGGTGGGACAGTGAGTATTATCCCAG
TTGGGGAAGCAAAGACATATGTAAATGGAAAACATATTTTGGAAATCACAGTATTACGTCATGGTGATCG
AGTGATTCTTGGTGGAGATCATTATTTTAGATTTAATCATCCAGTAGAAGTCCAGAAAGGAAAAAGGCCA
TCTGGAAGAGATACTCCTATAAGTGAGGGTCCAAAAGACTTTGAATTTGCAAAAAATGAGTTGCTCATGG
CACAGAGATCACAACTTGAAGCAGAAATAAAAGAGGCTCAGTTGAAGGCAAAGGAAGAAATGATGCAAGG
AATCCAGATTGCAAAAGAAATGGCTCAGCAAGAGCTTTCTTCTCAAAAAGCTGCATATGAAAGCAAAATA
AAAGCACTGGAAGCAGAACTGAGAGAAGAGTCTCAAAGGAAAAAAATGCAGGAAATAAATAACCAGAAGG
CTAATCACAAAATTGAGGAATTAGAAAAGGCAAAGCAGCATCTTGAACAGGAAATATATGTCAACAAAAA
ACGATTAAAAATGGAGACTTTGGCTACAAAACAGGCTTTAGAAGACCATAGCATCCGCCATGCAAGAATT
CTGGAAGCTTTAGAAACTGAAAAGCAAAAAATTGCTAAAGAAGTACAAATTCTACAGCAGAATCGGAATA
ATAGGGATAAAACTTTTACAATGCAGACAACTTGGAGCTCTATGAAACTCTCAATGATGATTCAGGAAGC
CAATGCTATCAGCAGCAAATTGAAAACATACTATGTTTTTGGCAGACATGATATATCAGATAAAAGTAGT

TCTGACACTTCTATTCGGGTTCGTAACCTGAAACTAGGAATCTCAACATTCTGGAGTCTGGAAAAGTTTG
AATCTAAACTTGCAGCAATGAAAGAACTTTATGAGAGTAATGGTAGTAACAGGGGTGAAGATGTCTTTTG
TGATCCTGAAGATGAATGGGAACCCGACATTACAGATGCACCAGTTTCTTCACTTTCTAGAAGGAGGAGT
AGGAGTTTGATGAAGAACAGAAGAATTTCTGGTTGTTTACATGACATACAAGTCCATCCAATTAAGAATT
TGCATTCTTCACATTCATCAGGTTTAATGGACAAATCAAGCACTATTTACTCAAATTCAGCAGAGTCATT
TCTTCCTGGAATTTGCAAAGAATTGATTGGTTCTTCATTAGATTTTTTTGGACAGAGTTATGATGAAGAA
AGAACTATAGCAGACAGCCTAATTAATAGTTTTCTTAAAATTTATAATGGGCTATTTGCCATTTCCAAGG
CTCATGAAGAACAAGATGAAGAAAGTCAAGATAACTTGTTTTCTTCTGATCGAGCAATCCAGTCACTTAC
TATTCAGACTGCATGTGCTTTTGAGCAGCTAGTAGTGCTAATGAAACACTGGCTGAGTGATTTACTGCCT
TGTACCAACATAGCAAGACTTGAGGATGAATTGAGACAAGAAGTTAAAAAACTAGGAGGCTACTTACAGT
TATTTTTGCAGGGATGCTGTTCGGATATTTCATCAATGATAAAAGAGGCTCAGAAGAATGCAATCCAAAT
TGTACAACAAGCTGTAAAGTATGTGGGGCAGTTAGCAGTTCTGAAAGGGAGCAAGCTACATTTTCTAGAA
AACGGTAACAATAAAGCTGCCGGTGTCCAGGAGGAATTCATGGATGCTGTTTGTGATGGTGTAGGCTTAG
GAATGAAGATTTTATTAGATTCTGGACTGGAAAAAGCAAAAGAACTTCAGCATGAACTCTTTAGGCAGTG
TACAAAAAATGAGGTTACCAAAGAAATGAAAACTAATGCCATGGGATTGATTAGATCTCTTGAAAACATC
TTTGCTGAATCGAAAATTAAAAGTTTCAGAAGGCAAGTACAAGAAGAAAACTTTGAATACCAAGATTTCA
AGAGGATGGTTAATCGTGCTCCAGAATTCTTAAAGTTAAAACATTGCTTAGAGAAAGCTATTGAAATTAT
TATTTCTGCACTGAAAGGATGCCATAGTGATATAAATCTTCTCCAGACTTGTGTTGAAAGTATTTGCAAC
TTGGCCAATGATTTTTACAGTGACTTCAATGTGCCTTCTACTTCTGTTGGCAGCTATGAGAGTAGAGTAA
CTCACATTGTCCACCAGGAACTAGAATCTCTAGCTAAGTCTCTCCTCTTTTGTTTTGAATCTGAAGAAAG
CCCTGATTTGTTGAAACCCTGGGAAACTTATAATCAAAATACCAAAGAAGAACACCAACAATCTAAATCA
AGCGGGATTGACGGCAGTAAGAATAAAGGTGTACCAAAGCGTGTCTATGAGCTCCATGGCTCATCCCCAG
CAGTGAGCTCAGAGGAATGCACACCCAGTAGGAGTCAGTGGGTG
>Rhinopithecus roxellana XM_010362665.1
ATGTCGTTACACAGTACTCATAATAGAAATAACCGCGGCGATATTCTTGATATTCCTTCTTCCCAAAATA
GTTCATCACTGAATGCCCTCACCCACAGTAGCCGACTTAAGCTGCATTTGAAGTCAGGTATGTCAGAATG
TGAAAATGATGATCCATTATTGAGATCTGCAGGTAAAGTCAGAGACATAAATAGCACTTACGTTATTTCT
GCCAGTGAAAAAACAGCAAACATGCCCCTTACCCCTAATCCTGTAGGTAGATTGACACTTCAGAGAAGAA
CTACAAGGAACAAAGAATCTTCTTTACTTGGTAGGGATTTGGAAGACACAACTGAAAAAACAGCAGAAAC
ACATCTTGCATTACAACGTCGTGCTAAAACAGATTCTGAAGAAAGTGGAAGACAGCTGAAACAGATTCT
GTCAAAATGACACTGAATGTGGGAGGTGAAACAGAAAATAATGGTGTTTCTAAGGAAAGGAGAACAAATG
TAAGGATTGTAAATAATGCTAAAAACTCTTTTGTTGCCTCTTGTGTACCTTTAGATGAAGATCCACAAGT
CATTGAAATGATGACTGATAAGAAATACAAAGAAACATTTTCTGCCCCCAGTAGAGCAAATGAAAATATT
GCACTTAAGTACTCAAGTAATAGAGCACCCATTGCTTCCCTGAGTCAGACTGAAGTTGTTAGATCAGGAC
ACTTGACAATGAAACCTACTCAGAGCAAGTTGGATATCAAAGTGTCGGGAACAGGAAACTTGCATCATAG
AACTATTGGAAAGGAAATTGCAAAAACTTCAAATAAATTTGGGAGCTTAGAAAAAAGAACACCTACAAAA
TGTACAACAGAACACAAATTGACACCAAAGTGCGGTCTGCCTCAGCTTAAGAGCCCAGCTCCATCAATAC
TGAAGAATAGAATGTCTAACCTTCAAGTTAAACAAAGACCAAAAAGTTCCTTTCTTGCGAATAAACAGGA
AAGGTCAGCAGAAAATACAATCCTTCCTGAAGAAGAAACTGTAGTTCAGAACACCTCTGCAGGAAAAGAC
CCCTTAAAAGTAGAGAATAGTCAAGTGACAGTGGCAGTACGCGTAAGACCTTTCACCAAGAGAGAGAAGA
TTGAAAAAGCATCCCAGGTGGTCTTCATGAGTGGGAAAGAAATAACTGTGGAACATCCTGACATGAAACA
AGTTTATAGTTTTATTTATGATGTTTCATTCTGGTCTTTTGATGAATGTCATCCTCACTACGCTAGCCAG
ACAACTGTCTATGAGAAGCTAGCAGCACCACTCCTAGAAAGAGCCTTTGAAGGCTTCAATACCTGTCTTT
TTGCTTATGGTCAGACTGGCTCTGGAAAATCATATACGATGATGGGATTTAGTGAAGAACCAGGAATAAT
TCCAAGATTTTGTGAAGATCTTTTTTCTCAAGTAGCCGGAAAACAAACCCAAGAGGTCAGCTATCACATT
GAAATGAGCTTCTTTGAAGTATATAATGAAAAAATTCATGATCTTCTGGTTTGTAAAGATGAAAATGGGC
AGAGAAAGCAACCACTGAGAGTGAGGGAACATCCTGTTTATGGACCATATGTTGAAGCACTGTCAATGAA

TGTTGTCAGTTCTTACACTGATATCCAGAGTTGGCTAGAATTGGGAAATAAACAAAGAGCTACTGCTGCT
ACTGGGATGAATGATAAAAGCTCCCGATCTCATTCAGTTTTCACCCTGGTGATGACCCAGACCAAGACAG
AATTTGTGGAAGGGGAAGAACACGATCACAGAATAACAAGTCGAATTAACCTAATAGATCTGGCAGGCAG
TGAGCGCTGCTCTACAGCTCATACTAGTGGAGATCGACTAAAGGAAGGTGTGAGTATTAATAAGTCCTTG
CTAACTTTGGGAAAAGTTATATCTGCACTCTCTGAACAAGCAAACCAAAAGAGAGTTTTTATTCCTTATC
GTGAATCTGTTCTTACATGGCTGTTAAAAGAAAGTCTGGGTGGAAATTCAAAAACTGCAATGATTGCTAC
GATTAGTCCTGCTGCCAGCAACATAGAAGAAACATTAAGCACACTTAGATATGCTAACCAAGCCCGTTTA
ATAGTCAACATTGCCAAAGTAAATGAAGATATGAATGCTAAGTTAATTAGAGAATTGAAGGCAGAAATTG
CAAAGCTAAAAGCTGCTCAGCGAAACAATCGGAATATTGACCCTGAACGATACAGGCTCTGTCGGCAAGA
AATAACATCCTTAAGAATGAAACTGCATCAACAGGAGAGAGACATGGCAGAAATGCAAAGAGTGTGGAAA
GAAAAGTTTGAACAAGCTGAAAAAAGAAAACTTCAAGAAACAAAAGAGTTACAGAAAGCAGGAATTACAT
TTCAAATGGACAATCATTTACCAAATCTTGTTAATCTGAATGAAGATCCACAACTATCAGAGATGCTGCT
ATATATGATAAAAGAAGGAACAACTACAGTTGGAAAGTATAAACCAAACTCAAGCCATGATATTCAGTTA
TCTGGGGTGCTGATTGCTGATGATCATTGTACTATCACAAATTTAGGTGGGACAGTGAGTATTATCCCAC
TTGGGGAAGCAAAGACATACGTAAATGGAAAACATATTTTGGAACTCACAGTGTTACATCATGGTGATCG
AGTGATTCTTGGTGGAGATCATTATTTTAGATTTAATCATCCAGTAGAAGTCCAGAAAGGAAAAAGGCCA
TCCGGAAGAGATACTCCTATAAGTGAGGGTCCAAAAGACTTTGAATTTGCAAAAAATGAGTTGCTCATGG
CACAGAGATCACAACTCGAAGCAGAAATAAAAGAGGCGCAGTTGAAGGCAAAGGAAGAAATGATGCAGGG
AATCCAGATTGCAAAAGAAATGGCTCAGCAAGAGCTTTCTTCTCAAAAAGCTGCGTATGAAAGCAAAATA
AAAGCACTGGAAGCAGAACTGAGAGAAGAGTCTCAAAGGAAAAAAATGCAGGAAATAAATAACCAGAAGG
CTAATCACAAAATTGAGGAATTACAAAAGGCAAAGCAGCATCTTGAACAGGAAATATATGTCAACAAAAA
GCGATTAGAAATGGAGGCTTTGGCTACAAAACAGGCTTTAGAAGACCACAGCATTCGCCATGCAAGAATT
CTGGAAGCTTTAGAAACTGAAAAGCAAAAAATTGCTAAAGAAGTACAAATTCTACAGCAGAATCGTAGTA
ATAGGGATAAAACTTTTACAATGCAGACGACTTGGAGCTCTATGAAACTCTCAATGATGATTCAGGAAGC
CAATGCTATCAGCAGCAAATTGAAAACATACTATGTTTTTGGCAGACATGATATATCAGATAAAAGTAGT
TCTGACACTTCTATTCGGGTTCGTAACCTGAAACTAGGAATCTCAACATTCTGGAGTCTGGAAAAGTTTG
AATCTAAACTTGCAGCAATGAAAGAACTTTATGAGAGTAATGGTAGTAACAGAGATGAAGATGTCTTTTG
TGATCCTGAAGATGAATGGGAACCCGACATTACAGATGCACCAGTTTCTTCACTTTCTAGAAGGAGGAGC
AGGAGTTTGATGAAGAATAGAAGAATTTCTGGTTGTTTACATGACATACAAGTTCACCCAATTAAGAATT
TGCATTCTTCACATTCATCAGGTTTAATGGACAAATCAAGCACCATTTACTCAAATTCAGCAGAGTCATT
TCTTCCTGGAATTTGCAAAGAATTGATTGGTTCTTCATTAGATTTTCTTGGACAGAGTTATGATGAAGAA
AGAACTATAGCAGACAGCCTAATGAATAGTTTTCTTAAAATTTATAATGGGCTATTTGCCATTTCCAAGG
CTCATGAAGAACAAGATGAAGAAAGTCAAGATAACTTGTTTTCTTCTGATCGAGCAATCCAGTCACTTAC
CATTCAGACTGCATGTGCTTTTGAGCAGCTAGTAGTGCTAATGAAACACTGGCTGAGTGATATACTGCCT
TGTACCACCAACATAGCAAGACTTGAGGATGAATTGAGACAAGAAGTTAAAAAACTGGGAGGCTACTTAC
AGTTATTTTTGCAGGGATGCTGTTCGGATATTTCTTCAATGATAAAAGAGGCTCAAAAGAATGCAATCCA
AATTGTACAACAAGCTGTAAAGTATGTGGGGCAGTTAGCAGTTCTGAAAGGGAGCAAGCTGCATTTTCTA
GAAAACGGTAACAATAAAGCTGCCAGTGTCCAGGAGGAATTCATGGATGCTATTTGTGATGGTGTAGGCT
TAGGAATGAAGATTTTATTAGATTCTGGACTTGAAAAAGCAAAAGAACTTCAGCATGAACTCTTAAGGCA
GTGTACAAAAAATGAGGTTACCAAAGAAATGAAAACTAATGCCATGGGATTGATTAGATCTCTTGAAAAC
ATCTTTGCCGAATCGAAAATTAAAAGTTTCCGAAGACAAGTAAAAGAAGAAAACTTTGAATACCAAGATT
TCAAGAAGATGGCTAATCGTGCTCCAGAATTCTTAAAGTTAAAACATTGCTTAGAGAAAGCTATTGAAAT
TATTATTTCTGCACTGAAAGGATGCAGTAGTGATGTAAATCTTCTCCAGAATTGTGTTGAAAGTATTCGC
AACTTGGCCAGTGATTTTTACAGTGACTTCGGTGTGCCTTCTACTTCTGTTGACAGCTATGAGAGTAGAG
TAACTAATGTTGTCCACAAGGAACTAGAATCTCTAGCTAAGTCTCTCCTCTTTTGTTTTGAATCTGAAGA
AAGCCCTGATTTGTTGAAACCCTGGGAAACTTATAATCAAAATACCGAAGAAGAACACCAACAATCTAAA
TCAAGTGGGATTGATGGCAGCAAGAATAAAGGTGTACCAAAGCGTGTCTATGAGCTCCATGGCTCATTCC

CAGCAGTGAGCCCAGAGGAATGCACACCCAGTAGGATTCAGTGGGTG
>Mandrillus leucophaeus XM_011990588.1
ATGTCATTACACAGTACTCATAATAGAAATAACCGCGGCGATATTCTTGATATTCCTTCTTCCCAAAATA
GTTCATCACTGAATGCCCTCACCCACAGTAGCCGACTTAAGCTGCATTTGAAGTCTGATATGTCAGAGTG
TGAAAATTATGATCCATTATTGAGATCTGCAGGTAAAGTCAGAGACATAAATAGCACTTACGTTATTTCT
GCCAGTGAAAAAACAGCAGACATGCCCCTTACCCCTAATCCTGTAGGTAAATTGACACTTCAGAGAAGAA
CTACAAGGAACAAAGAATCATCTTTGCTTGGTAGGGATTTGGAAGACACAACTGAAAAAACAGCAGAAAC
ACATCTTACATTGCAACGTCGTGCTAAAACAGATTCTGAAGAAAAGTGGAAAACAGCTGAAACAGATTCT
GTCAAAATGACACTGAATGTGGGAGGTGAAACAGAAAATAATGGTGTTTCTAAGGAAAGTAGAACAAATG
TAAAGATTGTAAATAATGCTAAAAACTCTTTTGTTGCCTCTTGTGTACCTTTAGATAAAGATCCACAAGT
CATTGAAATGATGGCTGATAAGAAATACAAAGAAATATTTTCTGCTCCCAGTAGAGCAAATGAAAATATT
GCACTTAAGTACTCAAGTAATAGAGCACCCATTGCTTCCCTGAGTCAGACTGAAGTTGTTAGATCAGGAC
ACTTGACAACAAAACCTACTCAGAGCAAGTTGGATATCAAAGTGTCAGGAACAGGAAACTTGCATCATAG
AACTATTGGGAAGGAAATTGCAAAAACTTCAAATAAATTTGGGAGCTTAGAAAAAAGAACACCTACAAAA
TGTACAACAGAACACAAATTGACACCAAAGTGTGGTCTGCCTCAGCTTAAGAGCCCAGCTCCATCAATAC
TGAAGAATAGAATGTCTAACTTTCAAGTTAAACAAAGACCAAAAAGTTCCTTTCTTGCGAATAAACAGGA
AAGGTCAGCAGAAAATACAATCCTTCCTGAAGAAGAAACTGTAGTTCAGAACACCTCTGCAGGAAAAGAC
CCCTTAAAAGTAGAGAATAGTCAAGTGACAGTGGCAGTACGCGTAAGGCCTTTCACTAAGAGAGAGAAGA
TTGAAAAAGCATCCCAGGTGGTCTTCATGAGTGGGAAAGAAATAACTGTGGAACATCCTGACATGAAACA
AGTTTATAGTTTTATTTATGATGTTTCATTGTGGTCTTTTGATGAATGTCATCCTCACTATGCTAGCCAG
ACAACTGTCTATGAGAAGCTAGCAGCACCACTCCTAGAAAGAGCCTTTGAAGGCTTCAATACCTGTCTTT
TTGCTTATGGTCAGACTGGCTCTGGAAAATCATATACGATGATGGGATTTAGTGAAGAACCAGGAATAAT
TCCAAGATTTTGTGAAGATCTTTTTTCTCAAGTAGCCAGAAAACAAACCCAAGAGGTCAGCTATCACATT
GAAATGAGCTTCTTTGAAGTCTATAATGAAAAAATTCATGATCTTCTGGTTTGTAAAGATGAAAATGGGC
AGAGAAAGCAACCACTGAGAGTGAGGGAACATCCTGTTTATGGACCATATGTTGAAGCACTGTCAATGAA
TGTTGTCAGTTCTTACACTGATATCCAGAGTTGGCTAGAATTGGGAAATAAACAAAGAGCTACTGCTGCT
ACTGGGATGAATGATAAAAGCTCCCGATCTCATTCAGTTTTCACCCTGGTGATGACCCAGACCAAGACAG
AATTTGTGGAAGGGGAAGAACATGATCACAGAATAACAAGTCGAATTAACTTAGTAGATCTGGCAGGCAG
TGAGCGCTGCTCTACGGCTCACACTAGTGGAGATCGACTAAAGGAAGGTGTGAGTATTAATAAGTCCTTG
CTAACTTTGGGAAAAGTTATATCTGCACTCTCTGAACAAGCAAACCAAAAAAGAGTTTTTATTCCTTATC
GTGAATCTGTTCTTACATGGCTGTTAAAAGAAAGTCTGGGTGGAAATTCAAAAACTGCAATGATTGCTAC
GATTAGTCCTGCTGCCAGCAACATAGAAGAAACATTAAGCACACTTAGATATGCTAACCAAGCCCGTTTA
ATAGTCAACATTGCCAAAGTAAATGAAGATATGAATGCCAAGTTAATTAGAGAATTGAAGGCAGAAATTG
CAAAGCTAAAAGCTGCTCAGCGAAACAATCGGAATATTGACCCTGAACGATACAGGCTCTGTCGGCAAGA
AATAACATCCTTAAGAATGAAACTGCATCAACAGGAGAGAGACATGGCAGAAATGCAAAGAGTGTGGAAA
GAAAAGTTTGAACAAGCTGAAAAAAGAAAACTTCAAGAAACAAAAGAGTTACAGAAAGCAGGAATTACAT
TTCAAATGGACAATCATTTACCAAACCTTGTTAATCTGAATGAAGATCCACAACTATCAGAGATGCTGCT
ATATATGATAAAAGAAGGAACAACTACAGTTGGAAAGTATAAACCAAACTCAAGCCATGATATTCAGTTA
TCTGGTGTGCTGATTGCTGATGATCATTGTACTATCACAAATTTAGGTGGGACAGTGAGTATTATCCCAC
TTGGGGAAGCAAAGACATACGTAAATGGAAAACGTATTTTGGAACTCACAGTATTACATCATGGTGATCG
AGTGATTCTTGGTGGAGATCATTATTTTAGATTTAATCATCCAGTAGAAGTCCAGAAAGGAAAAAGGCCA
TCTGGAAGAGATACTCCTATAAGTGAGGGTCCAAAAGACTTTGAATTTGCAAAAAATGAGTTGCTCATGG
CACAGAGATCACAACTCGAAGCAGAAATAAAAGAGGCGCAGTTGAAGGCAAAGGAAGAAATGATGCAAGG
AATCCAGATTGCAAAAGAAATGGCTCAGCAAGAGCTTTCTTCTCAAAAAGCTGCGTATGAAAGCAAAATA
AAAGCACTGGAAGCAGAACTGAGAGAAGAGTCTCAAAGGAAAAAAATGCAGGAAATAAATAACCAGAAGG
CTAATCACATAATTGAGGAATTAGAAAAGGCAAAGCAGCATCTTGAACAAGAAATATATGTCAACAAAAA
GCGATTAGAAATGGAGACTTTGGCTACAAAACAGGCTTTAGAAGACCATAGCATCCGCCATGCAAGAATT

CTGGAAGCTTTAGAAACTGAAAAGCAAAAAATTGCTAAAGAAGTACAAATTCTACAGCAGAATCGGAGTA
ATAGGGATAAAACTTTTACAATGCAGACAACTTGGAGCTCTATGAAACTCTCAATGATGATTCAGGAAGC
CAATGCGATCAGCAGCAAACTGAAAACATACTATGTTTTTGGCAGACATGATGTATCAGATAAAAGTAGT
TCTGACACTTCTATTCGGGTTCGTAACCTGAAACTAGGAATCTCAACATTCTGGAGTCTGGAAAAGTTTG
AATCTAAACTTGCAGCAATGAAAGAACTTTATGAGAGTAATGGTAGTAACAGAGATGAAGATGTCTTTTG
TGATCCTGAAGATGAATGGGAACCTGACATTACAGATACACCAGTTTCTTCACTTTCTAGAAGGAGGAGC
AGGAGTTTGATGAAGAACAGAAGAATTTCTGGTTGTTTACATGACATACAAGTTCACCCAATTAAGAATT
TGCATTCTTCACATTCATCAGGTTTAATGGACAAATCAAGCACCATTTACTCAAATTCAGCAGAGTCATT
TCTTCCTGGAATTTGCAAAGAATTGATTGGTTCTTCATTAGATTTTCTTGGACAGAGTTATGATGAAGAA
AGAACTATAGCAGACAGCCTAATGAATAGTTTTCTTAAAATTTATAATGGGCTATTTGCCATTTCCAAGG
CTCATGAAGAACAAGATGAAGAAAGTCAAGATAACTTGTTTTCTTCTGATCGAGCAATCCAGTCACTTAC
CATTCAGACTGCATGTGCTTTTGAGCAGCTAGTAGTGCTAATGAAACACTGGCTGAGTGATATACTGCCT
TGTACCACCAACATAGCAAGACTTGAGGATGAATTGAGACAAGAAGTTAAAAAACTGGGAGGCTACTTAC
AGTTATTTTTGCAGGGATGCTGTTCGGATATTTCATCAATGATAAAAGAGGCTCAAAAGAATGCAATCCA
AATTGTACAACAAGCTGTAAAGTATGTGGGGCAGTTAGCAGTTCTGAAAGGGAGCAAGCTACATTTTCTA
GAAAATGGTAACAATAAAGCTGCCAGTGTCCAGGAGGAATTCATGGATGCTATTTGTGATGGTGTAGGCT
TAGGAATGAAGATTTTATTAGATTCTGGACTTGAAAAAGCAAAAGAACTTCAGCATGAACTCTTAAGGCA
GTGTACAAAAAATGAGGTTACCAAAGAAATGAAAACTAACGCCATGGGATTGATTAGATCTCTTGAAAAC
ATCTTTGCCAAATCGAAAATTAAAAGTTTCCGAAGACAAGTACAAGAAGAAAACTTTGAATATCAAGATT
TCAAGAAGATGGCTAATCATGCTCCAGAATTCTTAAAGTTAAAACATTGCTTAGAGAAAACTATTGAAAT
TATTATTTCTGCACTGAAAGGATGCAGTAGTGATGTAAATCTTCTCCGGAATTGTGTTGAAAGTATTCGC
AACTTGGCCAGTGATTTTTACAGTGACTTCGGTGTGCCTTCTACTTCTGTTGACAGCTATGAGAGTAGAG
TAACTAATGTTGTCCACAAGGAACTAGAATCTCTAGCTAAGTCTCTCCTCTTTTGTTTTGAATCTGAAGA
AAGCCCTGATTTGTTGAAACCTTGGGAAACTTATAATCAAAATACCAAAGAAGAACACCAACAATCTAAA
TCAAGCGGGATTGATGGCAGCAAGAATAAAGGTGTACCCAAGCGTGTCTATGAGCTCCATGGCTCATCCC
CAGCAGTGAGCCCAGAGGAATGCACACCCAGTAGGATTCAGTGGGTG
>Cercocebus atys XM_012036696.1
ATGTCATTACACAGTACTCATAATAGAAATAACCGCGGCGATATTCTTGATATTCCTTCTTCCCAAAATA
GTTCATCACTGAATGCCCTCACCCACAGTAGCCGACTTAAGCTGCATTTGAAGTCAGATATGTCAGAATG
TGAAAATTATGATCCATTATTGAGATCTGCAGGTAAAGTCAGAGACATAAATAGCACTTACGTTATTTCT
GCCAGTGAAAAAACAGCAGACATGCCCCTTACCCCTAATCCTGTAGGTAAATTGACACTTCAGAGAAGAA
CTACAAGGAACAAAGAATCATCTTTGCTTGGTAGGGATTTGGAAGACACAACTGAAAAAACAGCAGAAAC
ACATCTTACATTACAACGTCGTGCTAAAACAGATTCTGAAGAAAAGTGGAAAACAGCTGAAACAGATTCT
GTCAAAATGACACTGAATGTGGGAGGTGAAACAGAAAATAATGGTGTTTCTAAGGAAAGTAGAACAAATG
TAAAGATTGTAAATAATGCTAAAAACTCTTTTGTTGCCTCTTGTGTACCTTTAGATAAAGATCCACAAGT
CATTGAAATGATGGCTGATAAGAAATACAAAGAAATATTTTCTGCCCCCAGTAGAGCAAATGAAAATATT
TCACTTAAGTACTCAAGTAATAGAGCACCCATTGCTTCCCTGAGTCAGACTGAAGTTGTTAGATCAGGAC
ACTTGACAACAAAACCTACTCAGAGCAAGTTGGATATCAAAGTGTCAGGAACAGGAAACTTGCATCATAG
AACTATTGGGAAGGAAATTGCAAAAACTTCAAATAAATTTGGGAGCTTAGAAAAAAGAACACCTACAAAA
TGTACAACAGAACACAAATTGACACCAAAGTGTGGTCTGCCTCAGCTTAAGAGCCCAGCTCCATCAATAC
TGAAGAATAGAATGTCTAACTTTCAAGTTAAACAAAGACCAAAAAGTTCCTTTCTTGCGAATAAACAGGA
AAGGTCAGCAGAAAATACAATCCTTCCTGAAGAAGAAACTGTAGTTCAGAACACCTCTGCAGGAAAAGAC
CCCTTAAAAGTAGAGAACAGTCAAGTGACAGTGGCAGTACGCGTAAGGCCTTTCACTAAGAGAGAGAAGA
TTGAAAAAGCATCCCAGGTGGTCTTCATGAGTGGGAAAGAAATAACTGTAGAACATCCTGACATGAAACA
AGTTTATAGTTTTATTTATGATGTTTCATTCTGGTCTTTTGATGAATGTCATCCTCACTATGCTAGCCAG
ACAACTGTCTATGAGAAGCTAGCAGCACCACTCCTAGAAAGAGCCTTTGAAGGCTTCAATACCTGTCTTT
TTGCTTATGGTCAGACTGGCTCTGGAAAATCATATACGATGATGGGATTTAGTGAAGAACCAGGAATAAT

TCCAAGATTTTGTGAAGATCTTTTTTCTCAAGTAGCCAGAAAACAAACCCAAGAGGTCAGCTATCACATT
GAAATGAGCTTCTTTGAAGTCTATAATGAAAAAATTCATGATCTTCTGGTTTGTAAAGATGAAAATGGGC
AGAGAAAGCAACCACTGAGAGTGAGGGAACATCCTGTTTATGGACCATATGTTGAAGCACTGTCAATGAA
TGTTGTCAGTTCTTACACTGATATCCAGAGTTGGCTAGAATTGGGAAATAAACAAAGAGCTACTGCTGCT
ACTGGGATGAATGATAAAAGCTCCCGATCTCATTCAGTTTTCACCCTGGTGATGACCCAGACCAAGACAG
AATTTGTGGAAGGGGAAGAACATGATCACAGAATAACAAGTCGAATTAACTTAATAGATCTGGCAGGCAG
TGAGCGCTGCTCTACGGCTCACACTAGTGGAGATCGACTAAAGGAAGGTGTGAGTATTAATAAGTCCTTG
CTAACTTTGGGAAAAGTTATATCTGCACTCTCTGAACAAGCAAACCAAAAGAGAGTTTTTATTCCTTATC
GTGAATCTGTTCTTACATGGCTGTTAAAAGAAAGTCTGGGTGGAAATTCAAAAACTGCAATGATTGCTAC
GATTAGTCCTGCTGCCAGCAACATAGAAGAAACATTAAGCACACTTAGATATGCTAACCAAGCCCGTTTA
ATAGTCAACATTGCCAAAGTAAATGAAGATATGAATGCCAAGTTAATTAGAGAATTGAAGGCAGAAATTG
CAAAGCTAAAAGCTGCTCAGCGAAACAATCGGAATATTGACCCTGAACGATACAGGCTCTGTCGGCAAGA
AATAACATCCTTAAGAATGAAACTGCATCAACAGGAGAGAGACATGGCAGAAATGCAAAGAGTGTGGAAA
GAAAAGTTTGAACAAGCTGAAAAAAGAAAACTTCAAGAAACAAAAGAGTTACAGAAAGCAGGAATTACAT
TTCAAATGGACAATCATTTACCAAACCTTGTTAATCTGAATGAAGATCCACAACTATCAGAGATGCTGCT
ATATATGATAAAAGAAGGAACAACTACAGTTGGAAAGTATAAACCAAACTCAAGCCATGATATTCAGTTA
TCTAGTGTGCTGATTGCTGATGATCATTGTACTATCACAAATTTAGGTGGGACAGTGAGTATTATCCCAC
TTGGGGAAGCAAAGACATACGTAAATGGAAAACGTATTTTGGAACTCACAGTATTACATCATGGTGATCG
AGTGATTCTTGGTGGAGATCATTATTTTAGATTTAATCATCCAGTAGAAGTCCAGAAAGGAAAAAGGCCA
TCTGGAAGAGATACTCCTATAAGTGAGGGTCCAAAAGACTTTGAATTTGCAAAAAATGAGTTGCTCATGG
CACAGAGATCACAACTCGAAGCAGAAATAAAAGAGGCGCAGTTGAAGACAAAGGAAGAAATGATGCAAGG
AATCCAGATTGCAAAAGAAATGGCTCAGCAAGAGCTTTCTTCTCAAAAAGCTGCGTATGAAAGCAAAATA
AAAGCACTGGAAGCAGAACTGAGAGAAGAGTCTCAAAGGAAAAAAATGCAGGAAATAAATAACCAGAAGG
CTAATCACATAATTGAGGAATTAGAAAAGGCAAAGCAGCATCTTGAACAAGAAATATATGTCAACAAAAA
GCGATTAGAAATGGAGACTTTGGCTACAAAACAGGCTTTAGAAGACCATAGCATCCGCCATGCAAGAATT
CTGGAAGCTTTAGAAACTGAAAAGCAAAAAATTGCTAAAGAAGTACAAATTCTACAGCAGAATCGGAGTA
ATAGGGATAAAACTTTTACAATGCAGACAACTTGGAGCTCTATGAAACTCTCAATGATGATTCAGGAAGC
CAATGCGATCAGCAGCAAACTGAAAACATACTATGTTTTTGGCAGACATGATGTATCAGATAAAAGTAGT
TCTGACACTTCTATTCGGGTTCGTAACCTGAAACTAGGAATCTCAACATTCTGGAGTCTGGAAAAGTTTG
AATCTAAACTTGCAGCAATGAAAGAACTTTATGAGAGTAATGGTAGTAACAGAGATGAAGATGTCTTTTG
TGATCCTGAAGATGAATGGGAACCTGACATTACAGATGCACCAGTTTCTTCACTTTCTAGAAGGAGGAGC
AGGAGTTTGATGAAGAACAGAAGAATTTCTGGTTGTTTACATGACATACAAGTTCACCCAATTAAGAATT
TGCATTCTTCACATTCATCAGGTTTAATGGACAAATCAAGCACCATTTACTCAAATTCAGCAGAGTCATT
TCTTCCTGGAATTTGCAAAGAATTGATTGGTTCTTCATTAGATTTTCTTGGACAGAGTTATGGTGAAGAA
AGAACTATAGCAGACAGCCTAATGAATAGTTTTCTTAAAATTTATAATGGGCTATTTGCCATTTCCAAGG
CTCATGAAGAACAAGATGAAGAAAGTCAAGATAACTTGTTTTCTTCTGATCGAGCAATCCAGTCACTTAC
CATTCAGACTGCATGTGCTTTTGAGCAGCTAGTAGTGCTAATGAAACACTGGCTGAGTGATATACTGCCT
TGTACCACCAACATAGCAAGACTTGAGGATGAATTGAGACAAGAAGTTAAAAAACTGGGAGGCTACTTAC
AGTTATTTTTGCAGGGATGCTGTTCGGATATTTCATCAATGATAAAAGAGGCTCAAAAGAATGCAATCCA
AATTGTACAACAAGCTGTAAAGTATGTGGGGCAGTTAGCAGTTCTGAAAGGGAGCAAGCTACATTTTCTA
GAAAATGGTAACAATAAAGCTGCCAGCGTCCAGGAGGAATTCATGGATGCTATTTGTGATGGTGTAGGCT
TAGGAATGAAGATTTTATTAGATTCTGGACTTGAAAAAGCAAAAGAACTTCAGCATGAACTCTTAAGGCA
ATGTACAAAAAAATGAGGTTACCAAAGAAATGAAAACTAATGCCATGGGATTGATTAGATCTCTTGAAAAC
ATCTTTGCCAAATCGAAAATTAAAAGTTTCCGAAGACAAGTACAAGAAGAAAACTTTGAATATCAAGATT
TCAAGAAGATGGCTAATCATGCTCCAGAATTCTTAAAGTTAAAACATTGCTTAGAGAAAACTATTGAAAT
TATTATTTCTGCACTGAAAGGATGCAGTAGTGATGTAAATCTTCTCCGGAATTGTGTTGAAAGTATTCGC
AACTTGGCCAGTGATTTTTACAGTGACTTCGGTGTGCCTTCTACTTCTGTTGACAGCTATGAGAGTAGAG

TAACTAATGTTGTCCACAAGGAACTAGAATCTCTAGCTAAGTCTCTCCTCTTTTGTTTTGAATCTGAAGA
AAGCCCTGATTTGTTGAAACCTTGGGAAACTTATAATCAAAATACCAAAGAAGAACACCAACAATCTAAA
TCAAGCGGGATTGATGGCAGCAAGAATAAAGGTGTACCAAAGTGTGTCTATGAGCTCCATGGCTCATCCC
CAGCAGTGAGCCCAGAGGAATGCACACCCAGTAGGATTCAGTGGGTG
>Cebus capucinus imitator XM_017499389.1
ATGTCATTACACACTACTTATAAGAGAAATGACAGCGGCGATATTCTTGATATTCCTTCTTCCCAGAATA
GTTCATCACTCAATAACCTCACCCACAGTAGCCGACTTAAGCTGCATTTGAAGTCGGATATGTCAGAATG
TGAAAATGATGATCCATTATTGAGATCTGCAGGTAAAGTCAGAGACATAAATAGCACTTACATTATTTCT
GCCAGTAAAATAACAGGAGACATGCCCCTTACCCTTAACCCTGTAGGTAGATTGTCACTTCGGAGAAGAA
CTACAAGGAACAAAGAATCATCTTTGCTTGCTAGTGAGTTGGAAGACACAACTGAAAAAACAGCAGAAAC
ATGTCTTACATTACAACGTCGTGCTAAAACAGATTCTGCAGAAAAGTGGAAAACAGCTGAAACAAATTCT
GTCAAAAAGTGGCAAATGACACCGAATGTGGGAAGCAAAATAGAAAGTAATTGTGTTTCTAAGGAAAGTA
GAACAAATGTAAAGATTGTAAATAATGCTAAAAACTCTTTTGTGGCTTCTTCTGTACCTTTAGATGAAGA
TTCAAAAGTCATTGAAATAATGGCTAATAAGAAATACAAAGGAACATTTTCTGCCCCCAGTAGGGCAAAA
GAAAATGTTGTGCTTAAGTACTCGAGTAATAGAACACCCATTGCTTCCTTGAGTCAGACTGAAGTTGTTA
GATCAGGACACTTGGCAACGAAACCTACTCAGAGCAAGCTGGATATCAAAGTGTCGGAAACAGGAAACTT
GTATCATAGAAGTATTGGGAAGGAAATTGCAAAAACTTCAACTAAATTTGGGAGCTTAGAAAAGAGAACA
CCTACCAAATGTATAACAGAACACAAATTGACACCAAAGTGCAGCACACCTCAGCTTAAGAGCCCAGCTT
CATCAATACTGAAGAATAGAATGTCTAACCTTCAAGTTAAACAACGACCAAAAAGTTCCCTTCTTGCAAA
TAAACAGGAAAGGTCAGCAGAAAATACAATCCTTCCTGAAGAAGAAAATGCATATCAGAACACCTCTGCA
GACAAAGACCCCTTAAAAGTAGAGAATAGTCAAGTGACAGTGGCAGTACGCGTAAGGCCTTTCACCAAGA
GAGAGAAGATTGAAAAAGCATCCCAGGTAGTCTTCATGAATGGGAAAGAGCTAACTGTGGAACATCCTGA
CATGAAACAAGTCTACAATTTTATTTATGATGTTTCATTTTGGTCTTTTGATGAATGTCACCCTCACTAC
GCTAGCCAGACAACTGTCTATGAGAAGCTAGCAGCACCACTCCTAGAAAGAGCCTTTGAAGGCTTCAATA
CCTGTCTTTTTGCTTATGGTCAGACTGGCTCTGGAAAATCCTATACGATGATGGGATTTAGCGAAGAACC
AGGAATAATTCCAAGATTTTGTGAAGATCTTTTTTCTCAAGTAGCCAGAAAACAAACCCAAGAGGTCAGC
TATCACATTGAAATGAGCTTCTTTGAAGTATATAATGAAAAAATTCATGACCTTCTGGTTTGTAAAGGTG
AAAATGGGCAGAGAAAGCAACCACTGAGAGTAAGGGAGCATCCTGTTTATGGACCATATGTTGAAGCACT
GTCAATGAATGTTGTCAGTTCTTATGCTGATATCCAGAGTTGGCTAGAATTGGGAAATAAACAGAGAGCT
ACTGCTGCTACTGGTATGAATGATAAAAGTTCCCGATCTCATTCCGTTTTCACCCTGGTGATGACCCAGA
CCAAGACAGAATTTGTGGAAGGGGAAGAACACGATCACAGAATAACGAGTCGAATTAACCTAATAGATCT
GGCAGGCAGCGAGCGCTGCTCTGCGGCTCACACTGGTGGGGATCGACTGAAGGAAGGTGTGAGTATTAAT
AAGTCCTTGCTAACTTTGGGAAAAGTTATATCTGCACTCTCTGAACAAGCAAACCAAAAGAGAGTTTTTA
TTCCTTATCGTGAATCTGTTCTTACATGGCTGTTAAAAGAAAGTCTGGGTGGAAATTCAAAAACTGCAAT
GATTGCTACGATTAGTCCCGCTGCCAGCAACATAGAAGAAACATTAAGCACACTTAGATATGCTAACCAA
GCCCGTTTAATAGTCAACATTGCCAAAGTAAATGAAGATATGAATGCTAAGTTAATTAGAGAATTGAAGG
CAGAAATTGAAAAGCTAAAAGCTGCTCAGAGAAACAATCGGAATATTGACCCTGAACGATACAGGCTCTG
TCGGCAAGAAATAACATCCTTAAGAATGAAACTGCATCAGCAGGAGAGAGACATGGCAGAAATGCAAAGA
CTATGGAAAGAAAAGTTTGAACAAGCTGAAAAAAGAAAACTTCAAGAAACAAAAGAGTTACAGAAAGCAG
GAATTACATTTCAAATGGACAATCATTTGCCAAACCTTGTTAATCTGAATGAAGATCCACAGCTATCAGA
GATGCTGCTATATATGATAAAAGAAGGAACAACTACAGTTGGAAAGTATAAGCCAAACTCAAGCCATGAT
ATTCAGTTATCTGGGGTGCTGATTGCTGATGATCATTGTATTATCAAAAATTTTGGTGGAACAGTGAGTA
TTGTCCCAGTTGGGGAAGCAAAGACATACATAAATGGAAAACATATTTTGGAACTCACAGTATTACATCA
TGGTGATCGAGTGATTCTTGGTGGAGATCATTATTTTAGATTTAACCATCCAGTAGAAGTCCAGAAAGGA
GGAAGGCCATCTGGAAGAGATCCTCTTATAAGTGAGGGTCCAAAAGACTTCGAATTTGCAAAAAATGAGT
TGCTCATGGCACAGAGATCACAACTCGAAGCAGAAATAAAAGAGGCACAGTTGAAGGCCAAGGAAGAAAT
GATGCAAGGAATCCAGATTGCAAAAGAAATGGCTCAGCAAGAGCTTTCTTCTCAAAAAGCTGCATATGAG

AGCAAAATAAAAGCATTGGAAGCAGAATTGAGAGAAGAGTCTCAAAGGAAAAAAATGCAGGAAATAAATA
ACCAGAAGGCTAATCACAAAATTGAGGAATTAGAAAAGGCAAAGCAGCATCTTGAACAGGAAATATATGT
CAACAAAAAGCGATTAGAAATGGAGACTTTGGCTACGAAACAGGCTTTAGAAGACCATAGCATCCGCCAT
GCAAGAATTCTGGAAGCTTTAGAAACTGAAAAGCAAAAAATTGCTAAAGAAGTACAAATTCTACAGCAGA
ATCGGAGCAATAGGGATAAAACTTTTACAATGCAGACAACTTGGAGCTCCATGAAACTCTCAATGATGAT
TCAGGAAGCCAATGCTATTAGCAGCAAATTGAAAACATACTATGTTTTTGGCAGACATGATGTATCAGAT
GGAAGTAGTTCTGACACTTCTATTCGGGTTCGTAACCTAAAACTAGGAATCTCAACATTCTGGAGTCTGG
AAAAGTTTGAATCTAAACTTGCAGCAATGAAAGAACTTTATGAGAGTAATGGTAGTAACAGGGGTGAAGA
TGTCTTTTGTGATCCTGAAGACGAATGGGAACCCGACATTACAGATGCAGCAGTTTCTTCACTTTCTAGA
AGGAGGAGCAGGAGTTTGATGAAGAATAGAAGAATTTCTGGTTGTTTACATGATATACAAGTCCACCCAA
TTAAGAATTTGCATTCTTCACATTCATCAGGTTTAATGGACAAATCAAGCACCATTTACTCAAATTCGGC
AGAGTCATTTCTTCCTGGAATTTGCAAAGAATTGATTGGTTCTTCATTAGATTTTCTTGGACAGAGCTAT
GATGAAGAAAGAACTATAGCAGATAGCCTAATGAATAGTTTTCTTAAAATTTATAATGGGCTACTTGCCA
TTTCCAAGGCTCATGAAGAACAAGATGAAGAAAGTCAAGATAACTTGTTTTCTTCTGATCGAGCAACCCA
GGCACTTACTATTCAGACTGCATGTGCTTTTGAGCAGCTAGTGGTGCTGATGAAACACTGGCTGAATGAT
TTACTGCCTTGTGCCAACATAGCAAGACTTGAAGATGAATTGAGACAAGAAGTTAAAAAACTGGGAGGCT
ACTTACAGTTATTTTTGCAGGGATGCTGTTCGGATATTTCATCAATGATAAAAGAGGCCCAAAAGAATGC
AATCCAAATTGTACAACAAGCTGTAAAGTATGTGGGGCAGTTAGCAGTTCTGAATGGGAGCAAGTTACAT
TTTCTGGAAAACAGTAACAGTAAAGCTGCCAGCGTCCAGGGGGAATTAATGGATGCTATTTGTGATGGTG
TAGGCTTGGGAATGAAGATTTTATTAGATTCTGGACTAGAAAAAGCAAAAGAACTTCAGCATGAACTCTT
AAGGCAATGTACAGGAAATGAGGTTACCAAAGAAATGAAAACAAGTGCCATGGGATTGATCAGATCTCTT
GAAAACATCTTTGCTGAATCGAAAATTAGAAGTTTCAGAAGGCAAGTACAAGAAGAGCATTTTGGATACC
AAGATTTCAAGAAGATGGTCAATTGTGCTCCAGAATTCTTAAAGTTAAAACATTGCTTAGAGAAAACTAT
TGAAGTTGTTATTTCTGCACTGAAAGGATGCAGTGGTGATGTAAATCTTCTCCAGAATTGTGTTGAAAGT
ATTTGCAACTTGGCCAGTGATTTTTACAGAGACCTCAGTGTGCCCTCTACTTCTGTTGACAGCTATGAGG
GTAGAGTAACTCACGTTGTCCACAAGGAACTAGAATCTCTAGCTAAGTCTCTTCTCTTTTGTTTTCAATC
TGAAGATGGACCTGATTTGTTGAAACCCTGGGAAACTTATAATCAAAATACCGAAGAAGAACACCAACAA
TCTAAATCAAGTGTTATTGACGGCAGTAAGAATAAAGGTGTACCAAAGCATGTCTATGAACTCCATGGCT
CATCCCCAGCAGTGAGCTCAGAGGAATGCACACCCAGTAGGATTCAGTGGGTG
>Saimiri boliviensis boliviensis XM_003938246.2
ATGTCATTACACACTACTTATAATAGAAATAACAGCGGCGATATTCTTGATATTCCTCCTTCCCAAAATA
GTTCATCACTCAATACCCACACCCACAGTAGCCGACTTAAGCTGCATTTGAAGTCGGATATGTCAGAATG
TGAAAATGATGATCTATTATTGAGATCTGTAGGTAAAGTCAGAGACATAAATAGCACTTACATTATTTCT
GCCAGTAAAAAAACAGGAGATGTGACCCTTACCCCTAACCCTGTAGGTAGATTGTCACTTCGGAGAAGAA
CTACAAGGAACAAAGAATCGTCTTTGCTTGCTAGTGAGTTGGAAGACACAACTGAAAAAACATCAGAAAC
ATGTCTCGCATTACAACGTGCTCAAACAGATTCTGCAGAACAAAAACCTACTCAGAGCAAGTGGAAAACA
GCTGAAACAGATTCTGTCAAAAAATGGCAAATGACAGCGAATGTGGGAGGCAAAATAGGAAGTAATTGTG
TTTCTAAGGAAAGTAGAACAAATGTAAAGATTGTAAATAATGCTAAAAACTCCTTCGTGGCTTCTTCTGT
ACCTTTAGATGAAGACTCAGACTCAAAAGTCATTGAAATAATGGCTAATAAGAAATACAAAGAAACATTT
TCTGCCCCCAGTAGGGCAAAAGAAAATGTTGTGCTTAAGTACTCGCGTAATAGAACACCCATTGCTTCCT
TGAGTCAGACTGAAGTTGTTAGATCAGGACACTTGGCAATGAAACCTACTCAGAGCAAGTTGGATATCAA
AGTGTCGGGAACAGGAAACTTGTATCATAGAAGTATTGGGAAGGAAACTGCAAAAACTTCAAATAAATTT
GGGAGCTTAGAAAAGAGGACACCTACCAAATGTATAACAGAACACAAATTGACACCAAAGTGCAGCACAC
CTCAGCTTAAGAGCCCAGCTCCATCAATACTGAAGAATAGAATGTCTAACCTTCAAGTTAAACAAAGACC
AAAAAGTTCCCTTCTTGCCAATAAACAGGAAAGGTCAGCAGAAAATACAATCCTTCCCGAAGAAGAAACT
GCAGGTCAGAACACCTCTGCAGACAAAGACCCCTTAAAAGTAGAGAATAGTCAAGTGACAGTGGCAGTAC
GCGTAAGGCCTTTCACCATGAGAGAGAAGATTGAAAAAGCATCCCAGGTAGTCTTCATGAATGGGAAAGA

GCTAACTGTGGAACATCCTGACATGAAACAAGTTTACAATTTTACTTATGATGTTTCATTTTGGTCTTTT
GATGAATGTCACCCTCACTATGCTTGCCAGACAACTGTCTATGAGAAGCTAGCAGCACCACTCCTGGGAA
GAGCCTTTGAAGGCTTCAATACCTGTCTTTTTGCTTATGGTCAGACTGGCTCTGGAAAATCTTATACGAT
GATGGGATTTAGTGAAGAACCAGGAATAATTCCAAGATTTTGTGAAGATCTTTTTTCTCAAGTAGCCAGA
AAACAAACCCAAGAGGTCAGCTATCACATTGAAATGAGCTTCTTTGAAGTATATAATGAAAAAATTCATG
ACCTTCTGGTTTGTAAAGGTGAAAATGGGCAGAGAAAGCAACCACTGAGGGTGAGGGAGCATCCTGTTTA
TGGACCATATGTTGAAGCACTGTCAATGAATGTTGTCAGTTCTTATGCTGATATCCAGAGTTGGCTAGAA
TTGGGAAATAAACAGAGAGCTACTGCTGCTACTGGTATGAATGATAAAAGTTCCCGATCTCATTCAGTTT
TCACCCTGGTGATGACCCAGACCAAGACAGAATTTGTGGAAGGGGAAGAACACGATCACAGAATAACGAG
TCGAATTAACCTAATAGATCTGGCAGGCAGTGAGCGCTGCTCTGCGGCTCACACTAGTGGAGATCGACTG
AAGGAAGGTGTGAGTATTAATAAGTCCTTGCTAACTTTGGGAAAAGTTATATCTGCACTCTCTGAACAAG
CAAACCAAAAGAGAGTTTTTATTCCTTATCGTGAATCTGTTCTTACATGGCTGTTAAAAGAAAGTCTGGG
TGGAAATTCAAAAACTGCAATGATTGCTACAATTAGTCCCGCTGCCAGCAACGTAGAAGAAACATTAAGC
ACACTTAGATATGCTAACCAAGCCCGTTTAATAGTGAACATTGCCAAAGTAAATGAAGATATGAATGCTA
AGTTAATTAGAGAATTGAAGGCAGAAATTGAAAAGCTAAAAGCTGCTCAGAGAAACAATCGGAATATTGA
CCCGGAACGATACAGACTCTGTCGGCAAGAAATAACATCCTTAAGAATGAAACTGCATCAACAGGAGAGA
GACATGGCAGAAATGCAAAGAGTATGGAAAGAAAAGTTTGACCAAGCTGAAAAAAGAAAACTTCAAGAAA
CAAAAGAGTTACAGAAAGCAGGAATTACATTTCAAATGGACAATCATTTGCCAAACCTTGTTAATCTGAA
TGAAGATCCACAGCTATCAGAGATGCTGCTATATATGATAAAAGAAGGAACAACTACAGTCGGAAAGTAC
AAACCAAACTCAAGCCATGATATTCAGTTATCTGGGGTGCTGATTGCTGATGATCATTGTACTATCAAAA
ATTTTGGTGGAACAGTGAGTATTATCCCAGTTGGGGAAGCAAAGACATACATAAATGGAAAACGTATTTT
GGAACTCACAGTATTACATCATGGTGATCGAGTGATTCTTGGTGGAGATCATTATTTTAGATTTAACCAT
CCAGTAGAAGTCCAGAAAGGAAAAAGGCCATCTGGAAGAGATCCTGTTATAAGCGAGGGTCCAAAAGACT
TCGAATTTGCAAAAAATGAGTTGCTCATGGCACAGAGATCAGAACTCGAAGCAGAAATGAAAGAGGCACA
GTTGAAGGCCAAGGAAGAAATGATGCAAGGGATCCAGATTGCAAAAGAAATGGCTCGGCAGGAGCTTGCC
TCCCAAAGAGCTGTGTATGAGACCAAGATAAACGCGCTGGAGGCAGAACTGAGAGAAGAGTCTCAAAGGA
AAAAAATGCAGGAAATAAATAACCAGAAGGCTAATCACAAAATTGAGGAATTAGAAAAGGCAAAACAGCA
TCTTGAACAGGAAATATATGTCAACAAAAAGCGATTAGAAATGGAGACTTTGGCTACAAAACAGGCTTTA
GAAGATCATAGCATCCGCCATGCAAGAATTCTGGAAGCTTTAGAAACTGAAAAGCAAAAAATTGCTAAAG
AAGTACAAATTCTACAGCAGAATCGGAGTAATAGGGATAAAACTTTTACAATGCAGACAACTTGGAGCTC
CCTGAAACTCTCAGTGATGATTCAGGAAGCCAATGCTATTAGCAGCAAATTGAAAACATACTATGTTTTT
GGCAGACATGATGTATCAGATAAAAGTAGTTCTGACACTTTTATTCGGGTTCGTAACCTAAAACTAGGAA
TCTCAACATTCTGGAGTCTGGAAAAGTTTGAATCTAAACTTGCAGCAATGAAAGAACTTTATGAGAGTAA
CGGTAGTAACAGGGGTGAAGATGTCTTTTGTGATCCTGAAGACGAATGGGAACCCGACATTACAGATGCA
GCAGTTTCTTCACTTTCTAAAAGGAGGAGCAGGAGTTTGATGAAGAATAGAAGAATTTCTGGTTGTTTAC
ATGATATACAAGTCCACCCAATTAAGAATTTGCATTCTTCACATTCATCAGGTTTAATGGACAAATCAAA
TACCATTTACTCAAATTTGGCAGAGTCATTTCTTCCTGGAATTTGCAAAGAATTGATTGGTTCTTCATTA
GATTTTCTTGGACAGAGTTATGATGAAGAAAGAACTATAGCAGATAGCCTAATGAATAGTTTTCTTAAAA
TTTATAATGGGCTACTTGCCATTGCCAAGGCTCACGAAGAACAAGATGAAGAAAGTCAAGATAACTTGTT
TTCTTCTGATCGAGCAACCCAGGCACTTACTATTCAGACTGCATGTGCTTTTGAGCAGCTAGTGGTGCTG
ATGAAACACTGGCTGAGTGATTTACTGCCTTGTACCAACATAGCAAGACTTGAGGATGAACTGAGACAAG
AAGTTAAAAAACTGGGAGGCTACTTACAGTTATTTTTGCAGGGATGCTGTTCGGATATTTCATCAATGAT
AAAAGAGGCCCAAAAGAATGCAATCCAAATTGTACATCAGGCTGTGAAGTATGTGGGGCAGTTAGCAGTT
CTGAAAGGGAGCAAGCTACATTTTCTGGAAAACAGTAACAATAAAACTGCCAGCGTCCAGGAGGAATTCA
TGGATGCTATTTGTGATGGTGTAGGCTTGGGAGTGGAAATTTTATTAGATTCTGGACTAGAAAAAGCAAA
AGAACTTCAGCATGAACTCTTAAGGCAATGTACAAAAAAATGAGGTTACCAAAGAAATGAAAACAAGTGCC
ATGGGATTGATTAGATCTCTTGAAAACATCTTTGCTGAATCGAAAATTAAAAGTTTCAGAAGGCAGGTAC

AAGAAGAACACTTTGGATACCAAGATTTCAAGAAGATGGTTAACTGTGCTCCAGAATTCTTAAAGTTACA
ACATTGCTTAGAGAAAACTATTGAAGTTGTTATTTCTGCACTGAAAGGATGCAGTGGTGACGTAAATCTT
CTTCAGAATTGTGTTGAAAGTATTTGCAACTTGGCCAGTGATTTTTACAGAGACCTCAGTGTGCCCTCTA
CTTCTGTTGACAGCCATGAGGGTAGAGTAACTCACGTTGTCCACAAGGAACTAGAATCTCTAGCTAAGTC
TCTTCTCTTTTGTTTTGAATCTGAAGATAGACCTGATTTGTTGAAACCCTGGGAAACTTATCATCAAAAT
ACCGAAGAAGAAAACCAACAATCTAAATCAAGTGGGATTGACGGCAGTAAGAATAAAGGTGTACCAAAGC
ATGTCTATGAACTCCATGGCTCATCCCCAGCAGTGAGCTCAGAGGAATGCACACCCAGTAGGATTCAGTG
GGTG
>Aotus nancymaae XM_012459565.1
ATGTCATTAAACACTACTTATAATAGAAATAACAGCGGCGATATTCTTGCTATTCCTTCTTCCCAAAATA
GTTCATCACTCAATACCCTCACCCACGGTAGCCGACTTAAGCTGCATTTGAAGTCGGATGTGTCAGAATG
TGAAAATGATGATCTATTATTGAGATCTGCAGGTAAAGTCAGAGACATAAATAGCACTTACGTTATTTCT
GCCAGTAAAAAAGCAGGAGACATGCCCCTTACCCCTAACCCTGTAGGTAGATTGTCACTTCGGAGAAGAA
CTACAAGGAACAAAGAATCATCTTTGCTTGCTAGTGAGTTCAAAGACACAACTGAAAAAACAGCAGAAAC
ATGTCTTATATTACAACGTTGTGCTAACACAGCTTCTGCAGAAAAGTGGAAAACAGCTGAAACAGATTCT
GTCAAAAAGTGGCAAGTGACACCGAATGTGGGAGGCAAAATAGAAAGTAATTGTGTTTCTAAGGAAAGTA
GAACAAATGTAAAGACTGTAAATAATGCTAAAAACTCTTTTGTGACTTCTTCTGTACCTTTAGATGAAGA
CTCAAAAGTCATTGAAATAATGGCTAATAAGAAATACAAAGAAACATTTTCTGCCCCTAGTAGGGCAAAA
GAAAATGTTGTGCTTAAGTACTCGAGTAATAGAGCACCCACTGCTTCCTTGAATCAGACTGAAGTTGTTA
GATCAGGACACTTGGCAACGAAACCTACTCAGAGCAAGTTGGATATCAAAGTGTCGGGAACAGGAAACTT
GTATCATAGAAGTATTGGGAAGGAAATTGCAAAAGCTTCAAATAAATTTGGGAGCTTAGAAAAGAGAACA
CCTACCAAATGTATAACAGAACACAAATTGACACCAAAGTGCAGCACACCTCAGCTTAAGAGCCCAGCTC
CATCAATACTGAAGAATAGAATGTCTAACCTTCAAGTTAAACAAAGACCAAAAAGTTCCCTTCTTGCAAA
TAAACAGGAAAGGTCAGCAGAAAATACAATCCTTCCTGAAGAAGAAACTGCAGGTCAGAACACCTCTGCA
GACAAAGACCCCTTAAAAGTAGAGAATAGTCAAGTGACAGTGGCAGTACGCATAAGGCCCTTCACCAAGA
GAGAGAAGATTGAAAAAGCATCCCAGGTAGTCTTCGTGAATGGGAAAGAGCTAACTGTGGAACATCCTGA
CATGAAACAAGTTTACAATTTTATTTATGATGTTTCATTTTGGTCTTTTGATGAATGTCACCCTCACTAC
GCTAGCCAGACAACTGTCTATGAGAAGCTAGCAGCACCACTCCTAGAAAGAGCCTTTGAAGGCTTTAATA
CCTGTCTTTTTGCTTATGGTCAGACTGGCTCTGGAAAATCCTATACGATGATGGGATTTAGTGAAGAACC
AGGAATAATTCCAAGATTTTGTGAAGATCTTTTTTCTCAAGTAGCCAGAAAACAAACCCAAGAGGTCAGC
TATCACATTGAAATGAGCTTCTTTGAAGTATATAATGAAAAAATTCATGACCTTCTGGTTTGTAAAGGTG
AAAATGGGCAGAGAAAGCAACCACTGAGAGTGAGGGAACATCCTGTTTATGGACCATATGTTGAAGCACT
GTCAATGAATGTTGTCAGTTCTTATGCTGATATCCAGAGTTGGCTAGAACTGGGAAATAAACAGAGAGCT
ACTGCTGCTACTGGTATGAATGATAAAAGTTCCCGATCTCACTCAGTTTTCACCCTGGTGATGACCCAGA
CCAAGACAGAGTTTGTGGAAGGGGAAGAACACGATCACAGAATAATGAGTCGAATTAACCTAATAGATCT
GGCAGGCAGTGAGCGCTGCTCTGCGGCTCACACTAATGGAGATCGACTAAAGGAAGGTGTGAGTATTAAT
AAGTCCTTGCTAACTTTGGGAAAAGTTATATCTGCACTCTCTGAACAAGCAAACCAAAAGAGAGTTTTTA
TTCCTTATCGTGAATCTGTTCTTACATGGCTGTTAAAAGAAAGTCTGGGTGGAAATTCAAAAACTGCAAT
GATTGCTACGATTAGTCCCGCTGCCAGCAACATAGAAGAAACATTAAGCACACTTAGATATGCTAACCAA
GCCCGTTTAATAGTCAACATTGCCAAAGTAAATGAAGATACGAATGCTAAGTTAATTAGAGAATTGAAGG
CAGAAATTGAAAAGCTAAAAGCCGCTCAGAGAAACAATCGGAATATTGACCCCGAACGATACAGGCTCTG
TCGGCAGGAAATAACATCCTTAAGAATGAAACTGCATCAGCAGGAGAGAGACATGGCAGAAATGCAGAGA
GTATGGAAAGAAAAGTTTGAACAAGCTGAGAAAAGAAAACTTCAAGAAACAAAAGAGTTACAGAAAGCAG
GAATTACATTTCAAATGGACAATAATTTACCAAACCTTGTTAATCTGAATGAAGATCCACAACTATCAGA
GATGCTGCTATATATGATAAAAGAAGGAACAACTACAGTCGGAAAGTATAAACCAGACTCAAGCCATGAT
ATTCAGTTATCTGGGGTGCTGATTGCTGATGATCATTGTACTATCAAAAATTTTGGTGGAACAGTGAGTA
TTATCCCAGTTGGGGAAGCAAAGACATACATAAATGGAAAACATATTTTGGAACTCACAGTATTACATCA

TGGTGATCGAGTGATTCTTGGTGGAGATCATTATTTTAGATTTAATCATCCAGTAGAAGTCCAGAAAGGA
AGAAGGCCATCCGGAAGAGACACTTTTATAAGTGAGGGTTCAAAAGACTTCGAATTTGCAAAAAATGAGT
TGCTTATGGCACAGAGATCACAACTCGAAGCAGAAATAAAAGAGGCGCAGTTGAAGGCCAAGGAAGAAAT
GATGCAAGGAATCCAGATTGCAAAAGAAATGGCTCAGCAAGAGCTTTCTTCTCAAAAAGCTGCATATGAG
AGCAAAATAAAAGCATTGGAAGCAGAACTGAGAGAAGAGTCTCAAAGGAAAAAAATACAGGAAATAAATA
ACCAGAAGGCTAATCACAAAATTGAGGAATTAGAAAAGGCAAAGCAGCATCTTGAACAGGAAATATATTT
CAACAAAAAGCGATTAGAAATGGAGACTTTGGCTACAAAACAGGCTTTAGAAGACCATAGCATCCGCCAT
GCAAGAATTCTGGAAGCTTTAGAAACTGAAAAGCAAAAAATTGCTAAAGAAGTACAAATTCTACAGCAGA
ATCGGAGTAATAGGGATAAAACTTTTACAATGCAGACAACTTGGAGCTCTATGAAACTCTCAATGATGAT
TCAGGAAGCCAATGCTATTAGCAGCAAATTGAAAACATATTATGTTTTTGGCAGACATGATGCGTCAGAT
AAAAGTAGTTCTGACACTTCTATTCGGGTTCGTAACCTAAAACTAGGAATCTCAACATTCTGGAGTCTGG
AAAAGTTTGAATCTAAACTTGCAGCAATGAAAGAACTTTATGAGAGTAATGGTAGTAACAGGGGTGAATA
TGTCTTTTGTGATCCTGAAGACGAATGGGAACCCGACATTACAGATGCAGCAGTTTCTTCACTTTCTAGA
AGGAGGAGCAGGAGTTTGATGAAGAATAGAAGAATTTCTGGTTGTTTACATGATATACAAGTCCACCCAA
TTAAGAATTTGCATTCTTCACATTCATCAGGTTTAATGGACAAATCAAGCACCATTTACTCAAATTCGGC
AGAGTCATTTCTTCCTGGAATTTGCAAAGAATTGATTGGTTCTTCATTAGATTTTCTTGGACAGAGTTAT
GACGAAGAAAGAACTATAGCAGATAGCCTAATGAATAGTTTTCTTAAAATTTATAATGGGCTACTTGCCA
TTTCCAAGGCTCATGAAGAACAAGATGAAGAAAGTCAAGATAACTTGTTTTCTTCTGATCGAGCAACCCA
GGCACTTACTATTCAGACTGCATGTGCTTTTGAGCGGCTGGTAGTGCTAATGAAACACTGGCTGAGTGAT
TTACTGCCTTGTACCAACATAGCAGGACTTGAGGATGAACTGAGACAAGAAGTTAAAAAACTGGGAGGCT
ACTTACAGTTATTTTTGCAGGGATGCTGTTCGGATATTTCATCAATGATAAAAGAGGCCCAAAAGAATGC
AATCCAAATTGTACAACAAGCTGTGAAGTATGTGGGGCAGTTAGCAGTTCTGAAAGGGAGCAAGCTACAT
TTTCTGGAAAACAGTAACAATAAAGCTGCCAGCGTCCAGGAGGAATTCATGGATGCTATTTGTGATGGTG
TAGGCTTGGGAATGAAGATTTTATTAGATTCTGGACTAGAAAAAGCAAAAGAACTTCAGCATGAACTCTT
AAGGCAATGTACAAAAAACGAGGTTACCAAAGAAATGAAAACAAGTGCCATGGGATTGATTAGATCTCTT
GAAAACATCTTTGCTGAATTGAAAATTAAAAGTTTCAGAAGGCAAGTACAAGAAGAACACTTTGGACACC
AAGATTTCAAGAAGATGGTTAATCGTGCTCCAGAATTCTTAAAGTTAAAACATTGCTTGGAGAAAACTAT
TGAAGTTGTTATTTCTGCACTGAAAGGATGCAGTGGTGATGTAAATCTTCTCCAGAATTGTGTTGAAAGT
ATTTGCAACTTGGCCAGTGATTTTTACAGAGACCTCAGTGTGCCCTCTACTTCTGTTGACAGCTATGAGG
GTAGAGTAACTCACGTTGTCCACAAGGAACTAGAATCTCTAGCTAAGTCTCTTCTCTTTTGTTTTGAATC
TGAAGATAGACCTGATTTGTTGAAACCCTGGGAAACTTATAATCAAAATACCGGAGAAGAACACCAGCAA
TCTAAATCAAGTGGGATTGACGGCAGTAAGAATAAAGGTGTACCAAAGCATGTCTATGAACTCCATGGCT
CATCCCCAGCATTGAGCTCAGAGGAATGCACACCCCGTAGGATTGAGTGGGTG
>micrcebus murinus XM_020284316.1
ATGTCAGTACACACTGCTCATAATAGAAATAACAGCATTACTTCTACCCCAAAGAGTTCTTCACTAAGCG
CCCTCACCCACAGTAGCAGACTAAAGCTGCATTTGAAGTCGGATATGTCAGAATGTGAAAATGATGATCC
GTTATTGAGATCTGCAAGTAAAACCAGAGACATAAATAGCACTTACGTTATTTCTGCCAGTACGAAAACA
GAAGGTATATCCCTTACCCCTAACCCTGTAGGTAAATTGGCACTTCAGAGAAGAACTACAAGGAACAAAG
AATCATCTTTGCTTGGTAGTGAGTCAGGAGAAACAACTGAAAAAACAGCAGAAAAATGCCTTACATTACA
ACGTCGTGCTAAAACAGATTCTTCGGAAAAGTGGAAAACAGCTAAAACAGATTACATCGAGAAGTGGAAA
ACAATGCAGGATGTGGGAGGCGAAAGAGAAAATAATTGTGCTTCAAAGGAAACTACTACAAATGTAAATG
TTGTAAATAATGTTAAAAACTCTTTTGTTGCATCTTCTATACCTTTAGCTAAAGATCCAAAAGACATTGA
AATGGTGGCTGATGAGAAATACAAAGAAACATTTTCTGCCAGCAGTGGGGCAAATGAAAATGTTGCACTT
AAATACTCAAGTAACCGAGCACCCATTGGTTCCCAGGATCAAACTGAAGTTGTTAAATCAGGACACTTGG
CAGCGAAACCTATTCAAAGCAAGTTGGATGTGAAAGTGTTAGGAATAGGAAACTTACATCATAGAAGTAT
CGGGAAGGACCTTGCAAAAAATTCAAATAAATTTGGAAGCTTAGAAAAAACACCTATGAAATGTATAACA
GACCACAAATTGACACCAAAGAGTGGCATGCCTCAGCTTAAGAGCCCAGCAGCCTCTGTACTCAAGAATA

GGATGCCTAACCTTCAAGTTAAACAAAGGCCAAAAAGTTCTCTTCTTGCAAATAAAAGGGAACGTTCACA
AGAAAATACACTCCCTGCTATGGAAGAAACTGCAGTTCAGAACACCTCTAAAGAAACAGACCCCTTAAAA
GTAGAGAACAGTCAAGTGACAGTGGCAGTACGCATAAGGCCTTTCACCAAGAGAGAGAAGATTGAAAAAG
CTTCCCAGGTAGTCTTCATGAATGGGGAAGAAATAACTGTGGAACATCCTGACATGAAACAAGTTTATAA
TTTTATTTATGATGTTTCATTCTGGTCTTTTGATGAATGTCATCCTCACTATGCTAGCCAGACAACTGTC
TATGAGAGGCTAGCAGCACCACTCCTAGAGAGAGCCTTTGAAGGCTATAATACCTGTCTTTTTGCCTATG
GTCAGACTGGCTCTGGAAAATCATATACGATGATGGGATTTAGTGAAGAACCAGGAATAATTCCAAGATT
TTGTGAAGATCTTTTTGCTCAAGTAGCCAAAAAACAATCCCAAGAGGTCAGCTATCACCTTGAAATGAGC
TTCTTTGAAGTATATAACGAAAAAATTCATGACCTTCTGGTTTGTAAAGGTGAAAATGGGCAGAGAAAGC
AACCCGTAAGATTAAAACAATCTATTATTTGTTTTTATTTTCTATTCTTGAATGTTGTCAGTTCTTACTC
TGATATCAAGAGTTGGCTAGAATTGGGAAATAAACAAAGAGCAACAGCTGCTACTGGTATGAATGATAAA
AGCTCCCGATCTCATTCAGTTTTTACCCTCGTGATGACCCAGACCAAGACGGAATTTGTGGAAGGGGAAG
AGCACGATCACAGAATAACAAGTCGCATAAACCTGATAGATCTGGCAGGCAGCGAACGCTGCTCTACAGC
TCGCACCAGTGGAGATCGGCTGAAGGAAGGTGTGAGTATTAACAAGTCCTTGCTAACTTTGGGAAAAGTT
ATATCTGCACTCTCTGAACAAGCAAACCAAAAGAGGGTTTTTATTCCTTATCGTGAATCTGTTCTTACAT
GGCTGTTAAAAGAAAGTCTGGGTGGAAATTCAAAAACTGCAATGATTGCTACAATTAGTCCTGCTGCCAG
CAACATAGAAGAAACATTAAGCACGCTTAGATATGCTAGCCAAGCCCGTTTGATAGTCAATATCGCCAAA
GTAAATGAAGATATGAATGCTAAGCTAATTAGAGAATTGAAAGCAGAAATTGAAAAGCTAAAAGCTGCTC
AGAGAAACAGTCGGAATATTGACCCTGAACGATATAGGCTCTGTCGGCAAGAAATAACATCCTTAAGAAT
GAAACTGCATCAACAGGAGAGAGACATGGCAGAGATGCAAAGAGTGTGGAAAGAAAAGTTTGAACAAGCT
GAAAGAAGAAAACTTCAAGAAACAAAGGAATTGCAGAAAGCAGGAATTACATTTCAAATGGACAACCACT
TGCCAAACCTCGTTAATCTCAATGAAGATCCACAACTATCGGAGATGCTGCTATACATGATAAAAGAAGG
AACAACTACAGTTGGAAAGTATAAACCAAACTCAAGCCATGACATTCAGTTATCTGGAGTGCTGATTGCT
GATGATCATTGTTCTATCAAAAATTTTGGTGGGACAGTGAGTATCATCCCAGTTGGAGAAGCAAAGACAT
ATATAAATGGAAAATGTATTTTGGAACCCACAGTATTACATCATGGTGATCGGGTGATTCTTGGCGGAGA
TCATTATTTTAGATTTAATCATCCAGTGGAAGTCCAGAAAGGAAAACGACCGTCTGGTAGAGATACTCTT
ATAAGCGAGGGTCCAAAAGACTTTGAATTTGCAAAAAATGAGTTGCTCATGGCACAAAGATCACAACTTG
AAGCGGAAATAAATCAGGCACAGTTGAAAGCAAAGGAAGAAATGATGCAAGGAATCCAAATTGCAAAAGA
AATGGCTCAGCAAGAGCTTTCTTCTCAAAAAGCTGCATATGAAAGCAAAATAAAAGCACTGGAAGCTGAA
CTGAAAGAAGAGTCTCAGAGGAAGAAAATGCAGGAAATAAATAACCAAAAGGCTAATCACAAAATTGAGG
AATTAGAAAAGGCAAAGAAGCATCTTGAACAGGAAATATATGTCAACAAAAAGCGTTTAGAAATGGAGAC
TTTGGCGACAAAGCAGGCTTTAGAAGACCATAGCATCCGCCATGCAAGAATTCTGGAAGCGTTAGAAACT
GAAAAACAAAAAATTGCTAAAGAAGTAGAAATTCTACAGCAGAATCGGAGTAATAGGGATAAAACTTTTA
CAATTCAAACAAATTGGAGCTCCATGAAACTCTCAATGATGATTCAGGAAGCCAATGCTATCAGCAGCAA
ATTAAAAACATACTATGTTTTTGGCAGACATGATGTATCAGATAAAGGTAGTTCTGACACTTCTATTCGG
ATTCGGAACCTGAAACTAGGGATCTCAACTTTCTGGAGTCTGGAAAAGTTTGAATCTAAACTTGCAGCAA
TGAAAGAACTTTATGAGAGTAATGGTAGCAACAGGAGTGAAGATGTCTTTTGTGATCCTGAAGATGAATG
GGAACCTGACATTACAAATGCTCCCATTTCTTCATATTCTAGAAGGAGGAGCAGGAGTCTGATGAAGAAC
AGAAGAATTTCTGGTTGTTTACATGACATACAAGTCTACCCAATTCAGAATTTACATTCTTCACGTTCAT
CAGGTGTAATGGAGAAATCAAGCACCATTTACTCAAATTCAGCAGAATCATTTCTTCCTGGAATTTGCAA
AGAACTGATTGGTTCATCATTAGATTTTCTTGGACAGAGTTATGATGAAGAAAAAACTATAGCAGATAGC
CTGATGAAGAATTTTCTCAAAATTTATGATGCGCTATTTGCCATTTCCAAAGCTCATGAAGAACAAGATG
AAGAAAGTCAAGACAACTTGTTTTCTTCTGATCGAGCAACCCAGTCACTTACTATCCAGATGGCATGTGC
TTTCGAGCAGCTTGTGGTGCTAATCAGACATTGGCTGAATGATCTTCTACCTTGTACTAGCATAGCAAAA
CTTGAGGATGAATTGAGAGAAGAAGTTAAAAAACTGGGAGGCTACTTACAGTTATTTTTGCAGGGATGCT
GTTCAGATATTTCATCTATGGTAAAAGAGGCTCACAAGAAAGTGATCCAAAGTGTACAACAAGCTGTCAA
GTGTGTGGGGCAGTTAGCAGTTCTGAAAGGGAGCAAGCTACATTTTCTGGAAAACAGTGACAATAAAGCT

GCCAGTGTCCAGGAGGAATTCATGGATGCTGTTTGTGATGGTGTAGGCTTAGGAATGAAGATCCTATTAG
ATTCTGGACTGGAAAAAGCAAAAGAACTTCAGCATGAACTCTTAAGGCAATGTACACAAAGTGAGGTTAC
CAAACAGATGAAAGCTAATGCCATGGGATTGATTAGATCTCTTGAAAACATCTTTGCTGAATGGAAAATA
AAAAGTTTCAGAACTCAAGTATCAGAAGAAACTCCTGGATACCAAGATTTCAAGAAGATGATTAATCTTG
CTCCAGAATTCTTGAAGTTAAAACATTGCTTAGAGCAAACTATTCAAATTATTATTTCTTCACTGAGAGG
ATGCAGCAATGATGTAAATCTTCTTAAGAATTGTGTGGAAAGTATTTGCAGCTTGGCCGGCGATTTTAGC
AGTGACTGCAGTGTGCCCTCCACCTCTGCTGACGGCTGTGAGGACAGAGGGCCTCACGTTGGCCACAGGG
AAGTAGAGTCTCTAGCTAAGTCACTCCTCTTCTATTTTGAATCTGAAGAAAGCCCCGATTTGTTGAAACC
CTGGGAAACTTGTAACCAAAACCCCATAGAAGAAGAACAACAGCAGTTAAAATCAAGCAGGGCTGACTGC
AGTAAGGAAAAAGGAGTTCCAAAGCGTGTGTACGAACTCCACGGCTCATCGCCGGCAGTGAGCTCAGAGG
GAGGCACACCCAGCAGGATTCAGTGGGTG
>Galeopterus variegatus XM_008569580.1
ATGTCAGTATACACTGCTCATAATAGAAATAACAGCCGTATCCTTGGTACTCCTTCTTCTCAAAAGAGTT
CATCACTGAGTGTCCTCACCCACAGTAACAGACTTAAGCTGCATTTGAACTCAAATATGTCAGAACGTGA
AAATGATAATCCGTTACTGAGATCTGCCAGTAAAATCAGAGACATAAATAGCACTTATATTATTTCTGCC
TGTAGAAAACCAGAAGATACACCCGTTACCCCTAACCCTGTAGGTAGATTGGCACTTCAGAGAAGAGCTA
CAAGGAACACAGAATCATCTTTGCTTGGTAGTGAATTGGGAGACTCAACTGAAAAAACAGCAGAAAAACG
TCTTATATTACAGCGTCGTGTTAAAACAGATTCTGTGGAAAAGTGGAAAACAGCTAAGACAGATTCTGCC
AAAAAGTGGAAAATAACGCAGAATGTGGGCAGTGAAATAGGAAATAATTGTGCTTCAGAGGAAACTAGTA
CAAATGTAAAGACTGTAAATAATGATAAAAACTCTTTTGTTGCACCTTCTGTACCTTTAGCTGAAGATCC
AAAAGACATTGAAATGATGGCTGATGAGAGATATAAAGAAACATTTTCTGCCATCAGTGGGGCAAATGGA
AATGTCGCACTGAAGTGCTCAAGTAATACAGCACTGGTTGGTTCCCAGAGTCAGGCCGAAGTTGTTAGAT
CAGGACACTTGGCAACAAAACCTGTTCAGAATAAGTTGGATATCAAAGTGTCGGGAACAGGAAACTTGCA
TCATAGAAGTATTCGGAAAAATCCAAATAAATTTGGAAGCTTAGAAAAAAGAACACCTACAAAATGTATA
ACAGAACACTCATTGATGCCGAAGTGCAGCACGCCTCAGTTTGAGAGCCCAGCTGCCTCAGTACTGAAGA
ATAGGATGCCTAACCTTCAAGTTAGACAAAGGCCAAAAGACTCTTTTCTTGCAAATAAAAAGGAAAAGTC
ACAAGAAAATACACTTCCTCTTGAAGAAGAAAGTGCGGTTCAGAACACCTCTACAGAAAAAGACCTCTTA
AAAGTAGAGAATAGTCAAGTGACAGTGGCCGTACGTGTAAGGCCTTTCACCAAGAGAGAGAAGATTGAAA
AAGCATCCCAGGTGGTCTTCATGAATGCGGAAGAAATAACTGTGGAACACCCTGATATGAAACAAATTTA
TAATTTTATGTATGATGTTTCGTTGTGGTCTTTTGATGAATGTCATCCTCACTATGCTAGCCAAACAACC
GTCTACAAGACTCTAGCAGCACCACTCCTAGAAAGTGCCTTTGAAGGTTACAATACCTGTCTCTTTGCTT
ATGGCCAGACTGGCTCTGGAAAATCATATACGATGATGGGATTTAGTGAAGAACCAGGAATAATTCCAAG
ATTTTGTGAAGATCTTTTTGCTCAAGTAGCCCAAAAACGGTCCCAAGAGGTCAACTATCACCTTGAAATG
AGCTTCTTTGAAGTATATAATGAAAAAATTCATGACCTTCTGGTTTGTAAAGGTGAAAATGGGCAGAGAA
AGCAACCGCTGAGAGTAAGGGAGCATCCCATTTCCGGTCCGTATGTTGAAGCGCTGTCAATGAATGTTGT
CAGTTCTTACTCTGATATCCAGAGTTGGCTAGAATTGGGAAATAAACAAAGAGCAACTGCTGCTACTGGT
ATGAATGATAAAAGCTCACGGTCTCATTCAGTGTTTACCCTGGTGATGACCCAGACCAAGACAGAATTTG
TGGAAGGGGAAGAACACGATCACAGAATAACAAGCCGCATAAACCTAATTGATCTGGCAGGCAGCGAGCG
CTGCTCCAGGGCTCACACTAGTGGAGATCGACTGAAGGAAGGTGTGAGTATTAACAAGTCCTTGCTAACT
CTGGGAAAGGTTATATCTGCACTCTCTGAACAAGCAAACGGAAAGAGAGTTTTTATTCCTTATCGTGAAT
CTGTTCTAACATGGCTATTAAAGGAAAGTCTGGGTGGAAATTCAAAAACTACAATGATTGCTACGATCAG
TCCTGCTGCCAGCAACATAGAAGAAACATTAAGCACACTTAGATATGCTAACCAAGCCCGTTTGATAGTC
AACATCGCCAAAGTAAATGAAGATATGAATGCTAAGTTAATTAGAGAACTGAAAGCAGAAATTGAAAAGC
TAAAAGCTGCTCAGAGAAACAGTCGGAATATTGACCCAGAACGATACAGGCTCTATCGGCAAGAAATAAC
ATCCTTAAGAATGAAGCTTCATCAACAGGAGAAGGACATGGCAGAAATGCAAAGAGTGTGGAAAGAAAAG
TTTGAACAAGCTGAAAAGAGAAAACTTCAAGAAACAAAGGAGTTGCAGAAAGCAGGAATTACATTTCAAA
TGGACAACCATTTGCCAAACCTCGTTAATCTCAATGAAGATCCACAACTATCGGAAATGCTGCTATATAT

GATAAAAGAAGGGACAACTACAGTTGGAAAGTATAAACCAAACTCAGGCCATGATATTCAGTTATCTGGG
GTGCTGATTGCTGATGATCATTGTACCATCAAAAATTTTGGTGGGACAGTGAGTATTGTCCCAGTTGGTG
AAGCAAAGACATATGTAAATGGAAAACATATTTTGGAATCCACAGTATTACATCATGGTGATCGGGTGAT
TCTTGGTGGAGATCATTATTTTAGATTTAATCATCCAGTGGAAGTCCAGAAAGGAAAAAGACCATTGGGC
AGAGATACTCTTACAAGCGAGGGTCCAAAAGACTTTGAATTTGCAAAAAATGAGTTGCTCATGGCACAGC
GATCACAACTTGAAGCAGAAATAAAAGAGGCGAGGTTGAAAGCAAAGGAAGAAATGATGCAAGGAATCCA
AATTGCAAAAGAAATGGCTCAGCAAGAGCTTTCTTCTCAAAAAGCTGCATATGAAAGCAAAATAAAAGCA
CTGGAAGCAGAACTGAAAGAAGAGTCTCAAAGGAAGAAAATGCAGGAAATAAATAACCAAAAGGCTAATC
ACAAAATTGAGGAATTAGAAAAGGCAAAACAACACCTTGAACAGGAAATATATGTCAACAAAAAGCGATT
AGAAATGGAGACTTTGGCTACAAAGCAGGCCTTAGAAGACCACAGCATTCGTCATGCAAGAATTCTCGAA
GCTTTAGAAACTGAAAAGCAAAAAATTGCTAAAGAAGTACAAATTCTACAGCAGAATCAGAGTAATAGGG
ATAAAACTTTTACAATTCAGCCAAATTGGAGCTCCATGAAACTCTCAATGATGATTCAGGAAGCCAATGC
CATCAGCAACAAATTTAAAAAATGTTATGTTTTTGGCAGACACGATGTTTCCGATAAAGGTGGTTCTGAC
ACTTCCATTCGGGTTCGTAATCTGCAACTAGGGATATCAACTTTCTGGAGTCTGGAAAAGTTTGAATCTA
AACTTGCAGCAATGAAAGAACTTTACGAGAGTAATGGTAGTAACAGGGGTGAAGATGTCTTTTGTGATCC
TGAAGATGAATGGGAGCCTGACATTACAAATGCACCAGTTTCTTCATTGTCTAGAAGGAGGAGCAGAAGT
TTGATGAAGAATCGAAGAATTTCTGGTTGTTTACATGACATACCAGTCCACCCAGTTCAGAATTTGCATT
CTTCACATTCATCAGGCTTAATGGGGAAATCAAGCACCATTTACTCAAATTCAGCAGAATCATTTCTTCC
TGGAATTTGCAAAGAATTGATTGGTTTATCATTAGATTTTCTTGGACAGAGTTATGATGAAGAAGAAAGT
ATAGCAGATAGCCTGATGAATAATTTTCTTAAAATTTATAATGGTCTATTAGCCATTTCCAAAGCTCATG
AAGAACAAGATGAAGAAAGTCAAGATAACCTGTTCTCTTCTGATCGAGCAACCCAGTCACTTACTATCCA
GATTGCATGTGCTTTTGAGCAGCTTGTGGTGCTAATCAAACACTGGCTGAGTGATTTTTTACCTTGTTCC
AACGTAGCAAGACTTGAAGATGAATTGAGACAAGAAGTTAAAAAACTGGGAGGCTACTTACAGTTATTTT
TGCAGGGATGCTGTTCAGATATTTTGTCAATGGTAAAAGAAGCTCAAAAGAAAGTGATCCAGATTGTACA
ACAAGCTGTAAAGTATGTGGGACAATTAGCAGTTCTGAAAGGGAGCAAGCTACATTTTCTGGAAAACAGT
AACAATAAGGCTGTCAGTGTCCAGGAGGAATTCATGAATGCTGTTTGTGATGGTGTAGGCTTAGGAATGA
AGGTTCTATTAGATTCTGGACTGGAAAAAGTAGAAGAACTTCAGCATGAACTCTTAAGGCAGTGTATACA
AAATGAGGTTACTAAACAGATGAAAGCTAACGCCATGGGATTGATCAGATCTCTTGAAAACATCTTTACT
GAATGGAAAACAAAAAGTTTCAGAACTCAAGTACAAGAAGAAAATTCTGGATATCAAGATTCCAAGAAGA
TGATTAACCTTGCCCCAGAATTCTTGAAGTTAAAACATTGCTTCGAGAAAACTATTCAAATTATTATTTC
TGCACTGAGAGGATGCCACAGTGATATAAGTCTTCTCAAGAACTGCGTTGAAAGTATTTGCAACTTGGCC
AGTGATTTTCAGAATGAATGCGTGACCTCTACGTCCGTTGACAGCTGTGAGAATGGCATACCCCAAGTTC
GCCACAGGGAACTGGAATCTCTAGCTAAGTCACTCCTCTTATGTTTTGCACCTGAAGAAAGACCCAGTTT
GTTGAAACCGTGGGAAACTTGTAATCAAGATACCAGAGAAGAACAACCACCAGAAGCTAAATCAGGCAGA
ACTGACTGCAGTCGGAATGAAGGTGTACCAGAGCGTGTCTGTGCACACAATGGCTCGTCCCCAGCAGTGA
GCTCGGAGGGATGCAAACCCAGTAGGATTCAGTGGGTG
>Mus musculus ENSMUST00000189413.6
ATGTCAGTACACACTTCGCATAGCAGACACAACATCGGAAGCCTGGAGGTTTCTTCTTCA
CAGAAGATTTCAGCATCCAGTGGCCTCGTCCACAGCAGCCGGCTGGAACTGCACTTGAAG
GCAGATATGTCAGAGTGTGAGAATCATGATCCATTCGTGAATGCTGGAAGTAAAACCATC
GACATAAATAGCACTTATGTTATCTCTGCCTGTAAAAAAACAAGAGAGACTCCTGTTACC
TCTGACCCCCGGAGACTGAGCCTCCAGAGAAGGGCTACTTGTGGGGACAGAGAGTCGTCT
TTACTTGGAAGTGAGTTGGGAAACAGAAGAACAGCAGACACAAGTCTTAGGTTACAACGG
CGGCACGGTAGAGCGGATTATGTGGGAAAGTGGGAGACATTGAATCCTGTGGGAGGTAAC
CCAGGAAGTGACTCTGCCTCCCAGGCATCGAGGACAGAAGCAAAGGGTGTAAATAATGAT
ACCCGTGTCCTGTCGTCTGTCGTCTCCGTGAAAGACTCCAACGACACCGGGTTGACGAGA
TGCAAAGACCCAGGTCCTCCCGTTGGTGCCTCTAATGAAAAGGTGACAGTTAAGGACACA

AACAGTAGAGCGCCTGTGGGTTCCCAGCGTCAGACTGAAGCTATGAGATCAGGACACTTA
GTGGTGCAACTGACCGAGAGCAAGTCTGATACCCCAGTGTCAGGAGGAAGAAACTCACAC
CGTGGGAATGCTGGCAAGGACACTGCTAAACAAGTCGGTACATTTGGAAGCTCAGATACA
AGAACCCCAGTGAAATGTGTTTTAGAACACAGATGGACACCAAGGCATGACCCGCCTCCA
CCGAAAAGCCCAGCTTTATCCACACCGAAAAACAATGGGAAGGACATCCCGAAACATGGC
AGTACATTCAGAAGCGCGAGCTCAGAATCGAGGACCCCGGTAAAATGTGTCCCAGAACAC
AGATGGACACCAAGGCATGACCTGCCTCCACCGAAAAGCCCAGCTTTATCCACACTGAAA
AACAGGATTGCAAGCCCTCGAGTTAAACCGAGGCCCAAAAGTTCTCTTTTTGCAAATAAA
AGGGAAAGCTCACGAGAAAGCACACTCCCTCCAGAAGAAAACAGTCTAGTTCAGAAGACC
TTTACAGAGCCAGACTCCTTAAAAGTAGAGAACAGTCAGGTGACCGTGGCGGTGCGCGTG
AGGCCTTTCAGCAAAAGAGAGAAGACTGAGAAAGCATCCCAGGTTGTCTTCACCAATGGG
GAAGAGATAACTGTGGAGCATCCCGATATGAAACAAGTTTACTCTTTTATTTACGATGTT
TCCTTTTGGTCTTTTGATGAATGTCACCCTGGCTATGCCAGCCAGACAACTGTATATGAG
ACACTAGCTGCACCACTCCTGGACAGAGCTTTTGAAGGCTATAACACCTGTCTCTTTGCT
TATGGCCAGACTGGCTCTGGAAAGTCCTATACGATGATGGGGCTTAATGAAGAACCAGGA
ATAATTCCTAGATTCTGTGAAGATCTCTTTGCTCAAATAGCCAAAAAACAAACCTCAGAG
GTCAGCTACCATCTTGAAATGAGCTTCTTTGAAGTTTATAATGAAAAAATTCATGATCTT
CTGGTTTGTAAAGGTGAAAATGGGCAGAGGAAACAACCACTGCGCGCGAGGGAGCACCCT
GTTTCTGGACCGTATGTTGAAGGCCTGTCAATGAATGTTGTGAGTTCTTACTCTGATATT
CAGAGCTGGCTGGAACTGGGAAATAAACAGAGAGCCACGGCGGCCACCGGCATGAACGAT
AAAAGCTCCCGGTCTCATTCTGTGTTCACCCTGGTGATGACACAGACCAAGACAGAGGTG
GTGGAGGGAGAGGAACATGACCACAGGATCACGAGCCGCATCAACCTTGTGGACCTGGCC
GGCAGTGAGCGCTGCTCCACCGCCCACTCGAGTGGGCAGCGACTGAAGGAAGGTGTGAGC
ATTAACAAGTCCTTGCTGACTTTGGGGAAGGTCATATCTGCACTCTCCGAGCAAGCAAAC
GGAAAGAGGGTTTTTATTCCATATCGGGAATCCACTCTTACATGGCTATTAAAAGAAAGC
CTGGGTGGAAATTCAAAAACAGCCATGATTGCTACCGTCAGTCCCGCTGCCAGCAACATA
GAGGAAACGCTGAGCACACTCAGATACGCCACCCAAGCCCGCCTGATAGTCAATATCGCC
AAAGTCAACGAGGACATGAATGCAAAGTTGATCAGAGAGTTGAAAGCAGAAATTGAAAAG
TTAAAAGCTGCCCAGAGAAGCAATCGGAACATTGACCCTGAACGGTACCGACTCTGTCGG
CAAGAGATAACGTCCTTAAGGATGAAGCTGCATCAGCAGGAGAGAGACATGGCAGAGATT
CAGAGAGTATGGAAGGAAAAGTTTGAACAAGCTGAAAAAAGAAAACTTCAAGAGACAAAG
GAGTTACAGAAAGCAGGAGTTACATTTCAAATGGACAACCACTTGCCAAACCTTGTCAAT
CTCAATGAAGACCCACAGCTGTCGGAGATGCTGCTCTACATGGTAAAGGAAGGAGTGACC
ACAGTTGGAAAGCACACACCAAGCTCAAGCCATGACATCCAGTTGTCTGGAGTGCTGATT
GCCGATGATCACTGTACTATCAGAAATTTTGGAGGAACAGTGAGTATTGTCCCAGCTGGA
GAAGCAAAGACATATGTAAACGGGACGCACATCTCGGAGCCCACAGTGTTACATCATGGT
GATCGGGTGGTTCTTGGTGGAGATCATTATTTTAGATTTAATCATCCAGTTGAAGTCCAG
AAAGGAAAAAAACTGTCAAGTAGAAATAATCTCACAACAAGCGAAGGCCCAAAAGATTTT
GAGTTTGCCAAAAATGAGTTACTTACAGCACAGAGGTCTCGGCTTGAGGCAGAAATTAAA
GATGCGCAGCTGAAGGCGAAGGAAGAAATGATGCAAGGAATTCAGATCGCAAAGGAGATG
GCCCAGCAAGAACTTTCTTCCCAAAAAGCTGTGTATGAGCGCAAAATCCAGGCCCTAGAG
GCGGAGCTGAGGGAAGAGTCTCAAAGGAAGAGATTGGAGGAATTAAATAACCAAAAAGCT
AGTCACAAGATTGAGGAGTTGGAAAGGGCGAAGCAGCATCTTGAACAGGAAGTGTACGTG
AACAAAAGGCGACTGGAGATGGAGACCCTGGCCACAAAGCAGGCCTTAGAAGACCATAGA
ATCCGACATGCAAGGATCTTAGAAGCTTTAGAAATTGAAAAGCAAAAGATCGCTGAAGAA
GTACAAATGCTTCAGGAGAATCGGGGAAACAGGGATAAAACTTTCACCATTCAGCCAAAC
TGGAACTCCATGAAACTCTCCACGATGATTCAGGAAGCCAATGCCATCAGTGACAAATTT

AAAAAGTGCTATATTTTTGGCAGACATGATGCATCAGACAAAGGCCGTTCTGATACCTCT
GTTCGAGTTCGTAACCTCCAGCTTGGGATCTCAACTTTCTGGAGCCTGGAAAAGTTTGAA
TCTAAACTTGCAGCAATGAAAGAGCTTTATGAGAGTAACGGTGGTGACAGGGATGAGGAT
GTCTTCTGTGACCCTGCAGATGAGTGGGAGCCCGACATTACAAGCACACCAGTTTCTTCC
CTTTCTAGAAGGAGGAGCAGAAGTTTGATGAAGAACAGGAGAGTCTCTGGCTGTCTGCAT
GACATCCATCCGATTCAGAGCATGCAGTCTTCACACTCATCTGGATTAATGGAAAAACCG
AGCACCATTTACTCAAATTCATCAGAGTCCTTTCTTCCCGGGATTTGCAAAGAATTGATT
GGCTCATCAATAGATTTCCTCGGACAGAGTTTCGATGAAGAGAAAACTATAGCAGATAGC
CTGATAAATAATCTCCTTAGGCTTCATAATGGGGTGATTGCCATCTCCAAAGCCCATGAA
GAGCAGGATGAAGAAAGTCAAGATAACCTGTTCTCTGACCGGGCGGCACAGGCTCTTACC
ATCCAGGTTGCTTGTGCCTTTGAGCAGCTTGTGGTTTTGTTCAAACACTGGCTGGGTGAT
TTTCTACCTTGTACTGGCTCAGCGAGGCTTGAAGACGAATTGAGACAAGATATTAAAAAA
CTCGGAGGCTATTTGCAATTGTTTTTGCAGGGATGCTGTTCGGATATTTCATCGATGGTT
AAAGAGGCTCAAAACAAAGTCATGAAGATTATACAGCAGGCTGTTCAGTGTGTGGGTCAG
CTAGCTGTTCTGAAAGGGAGCAAGCTCTGTGTTCTGGAAAACAGCAGCAAAGTCTCCAGT
ACCCAGGAGTTCATGGCCGCTCTCCAGGATGGTGTAACCTCTGGGATGAAGAGTCTCTTA
GACTCTGGGCTGGAGACCGCGCAAGACCTCAGGCAGGACCTCTCCAGGCAGAGTGCACGA
GAGGAGGTCACTAAGCAGATGAAAGCCAGTACTGTGGAGTGGGTCGGGTCTCTGGAAAAT
GCTGTCGCTGAATGGAGGACGAAAAGCTTCAGAACTCAAGCACAAGAAGGTTCTAGACAA
CAAGTTTCCAAGCTGTTAAGCCTTGCGTCAGAATTCTTGAAGCTGAAGTCGTGCTTGCAA
CAAACCGTTGAGATGATTGTATCTGCGCTGAGAGGGTGCCCCAGTGACTTGCACTGTCTC
AGAAGCTGCACCGAGACTATCTGCAGCCTGGCTCGCAAGCTTCACAGTGACTTCAGCGCA
CACTCTGCCTCTGCTGGCAGCTGCGGAAATGAACTGCCTCGTGCTGACTGTGAGGAACTG
GAGTCTCTAGCGAAGTCACTCCTCCTATGTTTTGAATGTGGAGAAAGCCCTGGTTTGTCG
AAACCCTGGGAGTCTTGTTCTTCGAATAGCAAGGAAGAGCAGTGCAAGTCAGACAGGGCT
GACTGCGGGAAAAGCGGGCCAAGACGCGCCTGTGAACCACACGGAGACGCAACCCCAGCA
GTGTCCTCTGGGGACTGCACCCCGAATAGGATTCAGTGGGTA
>Heterocephalus glaber ENSHGLT00100024257.1
ATGTCAGTACATACTATTCATAATAGAAATAACAGTGATTGCCTGAAGGTTCCTTCTTTG
CAAAAGAGTTCATCACTGAGTGGCCTCACTGATAGTAGCCAATTTAAGTTGCCTTTGAAG
TCAGATATGTCAGAATATGAAAAGGATGATCCATTATTGAGATCTGCATGTAAAATCAGA
GACTTAAATAGCACTTATGTTATTTCTGCCTGTAAAACAACAGGAGATACGCCCCTTACC
ACTAACCCCATGGGTAGATTGACACTTCAGAGAAGAGCTACAAGAAACAAAGAGTCCTCT
TTGCTTAGTAGTAAGACTACAGATACAACTGAAAAAACAGCTGAAACAAGTCTTACCTTA
CAGCGGCGTGCTAAACCGGTTTCTGTGGAGAAGTGGAAAACAGCTAAAGCAGATTCTGTT
GAAAAGTGGAAAACCAGGCAGAATGTGGGAAGTGAAACAGAAAGTAATTGTGCTTCACAG
AGAACTAGGACAAATGTAAAGATTGTAAATAACAATAAAAATTCTTTTGTTGCATCTTCT
ATACAGATAGCTGAGAACTCAAAAGACATTGCATTCATGGTTGATCAGAAATATAAAGAA
ACATTTTCTCCTCTCCGTGGGGGAAATGAAAATGTTGCGCTTAAGCACTCAAGCAGTAGT
ACACCCATGGGTTCCCAGGATCGGAGTGAGGCTGTTGGACCAGGACCACTGGCAATGAAG
CCTGTTCAGAGTAACTTGAATATCAAAGTGTCGGTAACAGGAAACTTACATCCTAGTATT
GGGAAGGACATTGCAAAAAATTCAAGTAAATTTGGCAACTTAGAAAAAAGAACCCCCATG
AAATGTATGACAGAACACATATCAACACCAAAGCATGACCTGCCTCACCTTAAGAGCCCA
GGTACATCAATACTGAAAAATAGGACGCCTAGCCTTCAAGTTAAACAAAGACCAAAAAGT
TCTCTTCTTGCAAGTAAAAGGGAAATTTCTCAAAAAAATGAACTCCTTATTGAAGAAGAA
ACTGCAGTTCAGAACACCTCTACAGAAACAGACCCCTTAAAAGTAGAGAATAGTCATGTG
ACTGTGGCAGTACGTGTACGGCCTTTCAGCAAAAGAGAGAAGATTGAAAAAGCATCTCAG

GTGGTCTTCATGAATGGGGAGGAAATAACTGTGGAACATCCTGACATGAAACAAGTTTAT
ACATTTATTTATAATGTTTCATTCTGGTCTTTTGATGAATGTCATCCTCACTATGCCAGC
CAGATGACTGTCTATGAGACACTGGCGGCACCACTTCTAGAAAGAGCCTTTGAAGGCTAC
AACACCTGTCTCTTTGCTTATGGTCAGACTGGCTCTGGAAAGTCATACACAATGATGGGA
TTTAGTGAAGAACCAGGAATAATTCCAAGATTTTGTGAAGATCTTTTTGCTCAAGTAGCC
AAAAAACAAACTGAAGAGGTCAGCTATCATTTTGAAATGAGCTTCTTTGAAGTATATAAT
GAAAAAATTCATGACCTTCTGGTTTGTAAAGGTGAAAATGGGCAGAGAAAGCAACCGCTG
AGAGTAAGGGAGCATCCTGTTTCTGGACCATTTGTTGAAGCTCTGTCAATGAATGTTATT
AGTTCTTATTCCGATATTCAGAGTTGGCTGCAATTGGGAAATAAACAGAGAGCAACTGCT
GCTACTGGTATGAATGATAAAAGTTCCCGTTCTCATTCAGTTTTTACCCTGGTGATGACC
CAGACCAAGACAGAAATTGTGGAAGGGGAAGAACATGATCACAGAATTACAAGCCGCATA
AATCTGGTAGATCTGGCAGGCAGTGAGCGCTGTTCTGCATCCCACACTAGTGGAGATCGA
CTAAAGGAAGGTGTGAGCATTAACAAGTCCTTGCTAACTTTGGGAAAGGTTATATCTGCA
CTTTCTGAACAAGCAAACCGAAAGAGGGTTTTTATTCCTTATCGTGAATCTGTTCTTACA
TGGTTATTAAAAGAGAGTCTGGGTGGAAATTCAAAAACTGCAATGATTGCTACAATCAGT
CCTGCTGCCAGCAACATAGAGGAAACATTAAGCACACTTAGATATGCTAACCAAGCTCGT
TTGATAGTCAACATCGCTAGAGTAAATGAAGACATGAATGCCAAGTTAATTAGAGAATTG
AAAGCAGAAATTGAAAAGCTAAAAGCTGCTCAGAGAAACAATCTGAATATTGACCCTGAA
CGATACAGGCTTTGTCGGCAAGAAATCACATCTTTACGCATGAAACTCCATCAGCAGGAG
AGAGACATGGCAGAAATGCAGAGAGTATGGAAAGAAAAGTTTGAACAAGCTGAAAAAAGA
AAACTTCAAGAAACAAAGGAGTTACAGAAAGCAGGAATTACATTTCAAATGGACAACCAT
TTGCCAAACCTTGTTAATCTCAATGAGGATCCACAACTATCTGAGATGCTGCTATATATG
ATAAAGGAAGGAACAACAACAGTTGGAAAGTATAAACCAAACTCAAGCCATGATATTCAG
TTATCGGGGGTGCTGATTGCTGATGATCATTGTACTGTCAAAAATTTTGGAGGGACAGTA
AGCATTATCCCAGTTGGTGAAGCAAAGACATTTGTGAATGGAAAACATATTTTGGAACCC
ACAGTGTTACATCATGGTGATCGGGTGATTCTTGGTGGAGATCATTATTTTAGATTTAAT
CACCCAGCTGAAGTCCAGAAAGGGAAAAGGCCATCTAGTAGAGATACTCTTATAAACGAG
GGTCCTAAAGACTTTGAATTTGCCAAAAATGAGTTACTCATGGCACAGAGATCACAACTT
GAGGCAGAAATAAAAGAAGCACAGTTGAAGGCAAAGGAAGAAATGATGCAAGGAATCCAA
ATTGCAAAAGAAATGGCTCAGCAAGAACTTTCTTCTCAAAAATCTGCATATGAAAGCAAA
ATACGAGCACTAGAAGCAGAACTGAGAGAAGAGTCTCAAAGGAAGAAAATGCAGGAAATA
AATAACCAAAAGGCTAATGACAAAATTGAAGAATTAGAAAAGACAAAGCTGCATCTTGAA
CAGGAAGTATGTGTCAATAAAAAACGATTAGAAATGGAAACTTTGGCTACGAAGCAGGCT
TTAGAAGACCACAGGATCCGTCATGCAAAAATTCTGGAAGCTTTAGAAGCTGAAAAGCAA
AAAATTGCTAAGGAAGTACAGATTCTACAGCAGAATCGGAGTAACAGGGATAAAACTTTT
ACAATTCAGCCAAATTGGAGCTCCATGAAAGTCTCAATGATGATTCAGGAAGCGAATGCC
ATCAGCGGCAAGTTAAAAAAACACTATGTTTTTGGCAGACATGATGCATCAGATAAAGGT
AGTTCTGAGACTTCTATTCGAGTTCGTAACCTGCAGCTTGGGGTGTCAACTTTCTGGAGT
CTCGAAAAGTTTGAATATAAACTTGCAGCAATGAAAGAACTTTATGAGAATAATGGTAGT
AACAAAGGTGATGATATTTTTTGTGATCCTGAAGATGAATGGGAACCTGATATTACAAAT
GCACCAGTTTCTTCATTTTCTAGAAGGAGGAGCAGGAGTTTGATGAAGAATAGAAGAATT
TCTGATTGTTTACATGATATACAAGTCCACCAAATTCATAATCTGTCTTCTTCACATTCA
TCAGGTTTAATGGAGAAATCAAGCACCATTTACTCAACTTCAGCAGAATCATTTCTTCCT
GGAATATGCAAAGAATTGATTGGTTCATCAGTGGATTTTCTTAGATACAGTTTTGATGAA
GAAAAAACTATAGCAGATAGCCTGATGAGTAAACTTCTAAAAATTTATAATGGGCTGCTT
GTCATCTCCAAAGCTCATGAAGAACAAGATGAAGAAAGTCAAGATAACTTGTTCTCTTCT
GATCGAGCAGTCCAGTCACTTACCATCCAGATTGCTTGTGCTTTTGAACAACTTGTGGTT

TTAATCAAACACTGGGTGAACGATTTTCTACCTTCTACCAGCATAGCAAAACTGGAAGAT
GAATTGAGACAAGAAGTTAAAGAACTGGGAAGCTACTTACAGTTATTTTTGCAGGGATGC
TGTTCGGATATTCCATCCATGGTAAAAGAGGCTCAAAAGAAAGTGATCCAAAGTATACAA
CAGGCTGTTAAGTATGTGGGAAAATTAGCAGTTCTAAAAGGGAACAAGCTGTATTTTCTG
GAAAACAGTAACAACAAGGCTGCCAGTGGCCAGGAGGAATTCATGGATGTTATTTGTGAT
AGTGTAGGCTCAGGAATGAAGATTCTGTTAGATTCTGGACTGGAAAAAACAAAAGAACTT
CAGCAAGACCTCTTAAGGCATTGTGCACAAAATGAGGTTACCAAACAGATGAAAGCAAAT
GCCATGGGATTAATCAAATCTCTTGAAAATATCTTTTCTGAATGGAAAACAAAAAGCTTC
AGAACTCAAGTACAAGAAGACGATTCTGGATACCAAGATTCCAAGAAGATAACTGATCTT
GCCCTAAAATTCCTGAACTTAAACCACTGCTTAGAGAAAATCATGCAAATTATTATTTCT
GCACTGACAGGATGCTACAGCGATGTGCATCTCCTCAAGAATTGTGTTGAAACGATTTGC
AGCTTGGTCAGTGATCTTTGCAGTGACTTCAGTGAGCCCTCCACTTCTTTTGACTGCTGT
GAGAAGAAAATTTCTCAAGTTGGCCACAGGGAACTAGAATCTCTAGCTAAGTCACTCCTC
CTATGTTTTGAATCTGAAGAAAGACTTGATTTGTTCAAATCCTGGGAATCTTATAATCAA
GATACCAGAGGAGAAGAAAAAGAAGAATCTAATTCAAGCTGGCCTGGCTGCAGTAGGAAT
AAAGGTGTACCGAAGCGTGTCTATGAACTGCGTGAGGCGTCCCCAGCAGTGAGCTCTGGG
GTATGCACACCCAGTAGGATTCAGTGGGTG
>Rattus norvegicus ENSRNOT00000012038.7
ATGTCGGTACACACTTCTCATAGCAGGCACAACATCGGGAGCCTGGAAGTGTCTTCTTCA
CAGAAGATTTCGGCATCGAGTGGCCTCGTCCACAGCAGCCGACTGGAACTGCGCTTGAAG
TCAGATATGTCAGAGTGTGAAAATCACGATCCATTAGCGAATGCTGGAACCAAAACCATA
GACATAAACAGCACTTATGTTATCTCTGCCTGTAAAAAAGCAAGAGAGACGCCCGCTACT
CCTGATCCCGGGAGACTGACCCTTCAGAGAAGGACTACTTGGAACAAAGAATCCTCTTTA
CTTGGTAGTGAGTCGGGGCATCCTACTGGAAGAACAGCAGACACAAGTCTTAGGTTACAA
CGTCGTCCGCTCGCTAGAGCAGATTATGGGGAAAAGTGGGAGACATCGAATCCTGTGGGA
GGTAATCCAGGAAGTAAGTCTGCTTCACAGAAAGGGAGGACAGAGGCAAAACTTGTAAAT
AATGACTCCTGTGCCCTGTCTTCCGTAGTTTCTGAGAAAGACTCAAATGACACCGGGTTG
ACAAAACGCAAAGACCCATGTCCTGCCCTCAGCACCTCTAATGAGAAGGTGACAGTTAAG
AACCCAAACAGTAGAGCACCTATTGGTTCCCAGCGTCAGACTGAAGCTATGAGATCGGGA
CACTTAGTGGTACAACCGGTTGAGAACAAGGCAGATATCCAAGTGTCAGGAGGGGGGAAC
TCACACCATGGAAATGCTGGGAAGGGCATTGCAAAACACACCAGTACATTCGGAAGCTCA
GAAACACGGACCCCGGCGAAATGTGTTTCAGAACATAGATCGGCACCACGGCATGACCTG
CCGCCACCTAAAAGCCCAGCTTTATCCACACAGAAAAACAATGGCAAGGACATTGCAAAA
CATGTCAGTACATTCAGAAACTCAAGCTCAGAAACACAGACCCCGGCAAAATGTGTTTTA
GAACACAGATGGGCACCAAGGCATGGCCTGCCACCCCCTAAAAGCCCGGCTTTATCAACA
CTGAAGAACAGGATTGCGAGCCCTCGAGTTAAACCAAGGCCAAAAAGTTCTCTCCTTGCA
AATAAAAGCTCACAAGAAAGCACGCTCCCTCCTGAAGAAAAAAGTACAGTTCAGAATACC
TTTACAGAACCAGACTCCTTAAAAGTAGAAAACAGTCAGGTGACAGTGGCTGTGCGTGTG
AGGCCTTTCAGCAAAAGAGAGAAGACTGAGAAAGCATCCCAGGTTGTCTTCAGGAATGGG
GAAGAAATAACCGTGGAACATCCCGATATGAAACAAATTTACTCTTTTATTTACGATGTT
GCGTTTTGGTCTTTTGATGAATGTCACCCTGGCTACGCCAACCAGACAACTGTGTATGAG
ACACTGGCAGCGCCACTCCTGGACAGAGCTTTCGAAGGCTATAATACCTGCCTCTTTGCT
TATGGCCAGACTGGCTCTGGGAAGTCCTATACGATGATGGGGCTTAATGAAGAGCCAGGG
ATAATTCCAAGATTTTGCGAAGACCTTTTTGCTCAAATCGCCAAAAAACAAGCCTCAGAG
GTCAGCTACCATCTTGAAATGAGCTTCTTTGAAGTTTATAATGAAAAAATTCATGATCTT
CTGGTTTGTAAAGGAGAAAATGGGCAGAGAAAACAACCACTGCGAAGTGAGGGGGAGCAT
CCTGTTTCTGGAGCATATGTTGAAGGTCGTGTCAATGTAAGTGCTCCACTCAGCCAGCAA

GAGAGCTGGCTGGAACTGGGAAATAAACAAAGAGCCACGGCGGCCACTGGCATGAACGAT
AAAAGCTCCCGCTCTCATTCCGTTTTCACCCTGGTGATGACGCAGACCAAGACAGAGTTT
GTGGAGGGGGAAGAGCATGACCACAGGATCACGAGCCGCATAAACCTGATAGATCTGGCT
GGCAGTGAGCGCTGCTCTACAGCCCACTCCAGTGGGGAGCGACTGAAGGAAGGTGTGAGC
ATTAACAAGTCCTTGCTAACTTTGGGGAAGGTCATATCTGCACTCTCTGAGCAAGCAAAC
GGAAAGAGGGTCTTCATTCCATATCGCGAGTCCACTCTTACATGGCTATTAAAAGAAAGC
CTAGGTGGAAATTCAAAGACAGCAATGATTGCTACCATCAGTCCCGCTGCTAGCAACATA
GAAGAAACGCTGAGCACACTCAGATATGCCACTCAAGCCCGCCTGATAGTCAATGTTGCC
AAAGTCAACGAGGACATGAATGCAAAGTTAATCAGAGTACTCCGAACCAGGGCCTCTCTC
GGTAAGCCCTCATGTTCCTTCACACCTAATTTCCTTCCCAAAGAGTATACTCTTGTCGGC
AAAGAGATATCGTACCCTAGAGGAGAACTGCCTCAGCAGGAGAGAACATGGCAGAAGATA
CAAAGAGTGTGGAAGGAAAAGTTTGAACAAGCTGAAAAAAGAAAACTTCAAGAGACAAAG
GAATTACAGAAAGCAGGAGTTACCTTTCAAATGGACAACCACTTGCCAAACCTTGTTAAT
CTCAATGAAGACCCACAGCTGTCAGAGATGCTGCTCTACATGGTAAAGGAGGGAATAACT
ACAGTTGGAAAGCACACGCCAAACTCAAGCCACGACATCCAGTTGTCTGGAGTGCTGATT
GCCGATGATCACTGTACTATCAGAAGTTATGGAGGAACAGTGAGCATCGTCCCGGCTGGA
GAAGCGAAGACATACGTAAATGGGATGCACATCTCGGAGCCCACAGTATTACACCATGGT
GATCGGGTGGTTCTTGGTGGAGATCATTATTTTAGATTTAATCATCCAGTTGAAGTCCAG
AAAGGAAAAAAAACGAGTAGAAATAATCTCACAAGTGAAGGCCCAAAAGACTTTGAGTTT
GCCAAAAACGAGTTACTTGCGGCCCAGAGATCTCGGCTTGAAGCAGAAATAAAAGACGCA
CAGTTGAAGGCGAAGGAAGAAATGATGCAAGGGATTCAGATTGCAAAGGAGATGGCCCAG
CAGGAGCTTTCTTCCCAGAGGGCTGCCTATGAGAACAAAATCCGGGCCCTGGAGGCAGAG
CTGAGGGAAGAGTCTCAAAGGAAGAGATTGGAGGAAATAAACAACCAAAAAGCTAGTCAC
AAGATTGAGGAGCTGGAAAAGGCGAAGCAGCATCTTGAACAGGAAGTGTACGTGAACAAA
AGGCGACTGGAGATGGAGACCCTGGCCACAAAGCAGGCCTTAGAAGACCATAGAATCCGA
CACGCAAGGATCCTAGAAGCTTTAGAACGTGAAAAGCAAAAGATCGCTGAAGAAGTACAA
ATTCTTCAGGAGAATCGAGGAAACAGGAATAAAACTTTTACCATTCAGCCAAACTGGAGC
TCCATGAAGCTCTCCACGATGATCCAGGAAGCCAATGCCATCAGTGACAAATTTAAAAAG
CACTATGTGTTTGGCAGACATGATGCATCAGACAAAGGTCGTTCTGATCCTTCCTGTGAA
CTTCAGGGCAGCCAGGTGGGAGTTAACACAGCATGGTCTATAGAGCAATTCCGTTTATTT
CTGGCTGTTATGATTCAACTTTATGAGAGTAACAGTGGTACCAGGGGTGAGGATGTCTTT
TGTGACCCTGAAGATGAGTGGGAACCGGACATTACAAGCACACCAGTTTCTTCCCTCTCT
AGAAGGAGGAGCAGAAGCTTGATGAAGAACAGGAGAGTCTCCGGTTGTCTGCATGACATT
CATCCGATTCAGAGTATGCATCCTTCACACACATCAGGATTAATGGAAAAACCAAGCACC
ATCTATTCAAATTCATCAGAATCATTTCTTCCTGGAATTTGCAAAGAACTGATTGGCTCA
TCGATAGATTTCCTTGGACAGAGTTTCGATGAAGAGAAAACTATAGCCGATAGCCTGATA
AATAACCTTCTTAAACTTTATAATGGAGTGATTGCCATCTCCAAAGCCCATGAAGAACAG
GATGAAGAAAGTCAAGATAATGTGTTCTCTGACCGGGCAGCACAGGCTCTTACCATCCAG
GTTGCGTGTGCCTTTGAGCAGCTTGTGGTCTTGTTCAAACACTGGCTGGATGATTTTCCG
CCTTGTAGCGGCTCAGCGCGACTTGAAGAGGAATTGCGACAAGATATTAAAAAACTGGGG
GGCTATTTGCAATTGTTTTTGCAGGGATGCTGCTCAGATATTTCGTCGATGGTAAAAGAG
GCTCAAAACAAAGTCACCGAGATTATACAGCAGGCTGTTGGGCATGTGGCTCAGCTAGCT
GTTCTGAAAGGCAGCAAGCTCTGTGTTCTGGAAGACAGCAACAAGGTTTCCAGTGCCCAG
GAGCTCATGGCTGCTCTCCAGGATGGCACAGCCTCGGGGATGAGGAGTCTCTTAGACTCC
GGGCTGGAGACTGCACAGGAACTCAGGCAGGACGTCTCCAGGCAGAGTGCACCAGAGGAG
GTCACCAGGCAGATGAAAGCTAATGCTGTGGAACTGGTCGGGTCTCTTGAAAATATCTTT
GCGGAATGGAGGACGAAAAGCTTCAGAACTCAAGTAGAAGAAGGCTCTAGACAACAAGGC

TCCAAGGTGTTAAACCTCGCATCAGAATTCTTGAAGCTGAAGTCTTGCTTACAGCAAACC
ATTGAGATGGTTGTGTCTGCGTTGAGAGGGTGCCCCAGTGACTTGCGCTGTCTCAGAAGC
TGTACAGAAACTATCTGCGGCCTGGCTCACAAGCTTCATGGTGGCTTCAGTGCCCTCTCT
GACTCTGCAGGCAGCTGTGGAAATGAGCAACCTCGTGCTGTCTGGGAGGAACTGGAATCT
CTAGCTAAATCACTCCTCCTGTGTTTTGAACATGGAGAAAGACCTAGTTTCCTGAAACCC
TGGGAATCTTGTACTTCGAATAGCACGGAAGAGCAGCGAGAGTCAGACAGGGCTGACTGC
AGTAGAAGCGTGCCAGGACGTGCTTGTGAACCACACCGAGACCCGACCCCAGCAGAGTCC
TCTGGGGACGGCACCCCCAGTAGGATTCAGTGGGTA
>Chinchilla lanigera ENSCLAT00000014514.1
ATGTCAGCACACACTATTCATAATAGAAGTAACCGTGATTTCCTGGAAGTTCCTTCTTCC
CAAAAGAGTTCATCACTGAGTACCCTCACCCACAGTAGCCGATTCAAGTTTCCTTTGAAG
TCAGAGATGTCAGAATATGAAAAGGGTGATTCATTATTGAGATCTGCACGTACAATCAGA
GACTTAAATAGCACTTATGTTATTTCTGCCTGTAAAACAACAGGAGATATGCCCCTTACC
ATTAACCCTACAGAGAGATTGACACTTCAGAGAAGAGCTACAAGAAACAAAGAATCCTCT
TTACTTAGTAGTGAGTCTAGAGACACAACTGAAAAGACAGCTGAAGCAAGTCTTACTCTA
CAGCGTCGTGCTAAAACAGTTTCTGTAGAAAAGTGGAAAACAGCTAAAGCAGATTCTGTT
GCGAAGTGGAAAACACGGCAGGATGTGGGAAGTGAAACAGAAAGTAATTGTGCTTCACAG
AGAACTAGGACAAATGTAAAGTTTGTAAATAACAATAAAAATTCTTTTGTTACATCTTCT
ACACAGATAGCTGAAGACTCAAAAGACGCTATGTTAATGGTTGATCAGAAATATAAAACA
TTTTCTGCCCCCAGTGGGGCAAATGAAAATACGCTTAAGCACTCAAGCAGTAGCACACTT
GTGGGTTCCCAGGATCAGAGGGAGGCTGGTGGACCAGGACCACTGGCAGCAAAACCTATC
CAGAGTAACTTGAATATCAAAGTGTCTGGAACAGGAAACTTACATCCTAGTACTGGGAAG
GACATTGCAAAAAATTCAAGTAAATTTGGAAGTTTAGACAAAAGAAACCCTGTGAAATGT
ATGACAGAATACAAATCGACACCGAAGCATGACCTGCCTCAGCTCAAGAGCTCAGGTACA
TCGATACTGAAAAATAGGACGCCTAGCCTTCAAGTTAAACAAAGGCCAAAAAGTTCTCTT
CTTTCAAGTAAAAGGGAAATTTCTCAAAAAAATACACTCCCTATTCAAGAAACTGAAGTT
CAGAACACCTCTATAGAAACAGACTTCCTAAAAATAGAGAATAGTCAAGTAACTGTGGCA
GTACGTGTAAGGCCTTTCAGCAAAAGAGAGAAGATTGAAAAGGCATCTCAGGTGGTCTTC
ATGAACGGGGAAGAAATAATCGTGGAACATCCTGACATGAAGCAAGTTTATAGTTTCATT
TATGATGTTTCATTCTGGTCTTTTGATCAGTGTCATCCTCACTACGCTAGCCAGGTGACT
GTCTATGAGACATTGGCAGCACCACTTCTAGAAAGAGCCTTTGAAGGCTACAACACCTGT
CTCTTTGCTTATGGTCAGACTGGCTCTGGAAAGTCATACACGATGATGGGATTTAGTGAA
GAACCAGGAATAATTCCAAGATTTTGTGAAGATCTTTTTGCTCAAGTAGCCAAAAAACAA
ACCCAAGAGGTCAGCTATCATTTTGAAATGAGCTTCTTTGAAGTATATAATGAAAAAATT
CATGACCTTCTGGTTTGTAAAGGTGAAAATGGTCAGAGAAAGCAACCACTGAGAGTGAGA
GAGCATCCTGTTTCTGGACCATTTGTTGAAGCTCTGTCAATGAATGTTATTAGTTCTTAC
TCCGATATCCAGAGCTGGCTAGAACTGGGAAATAAACAAAGGGCAACTGCTGCGACTGGT
ATGAATGATAAAAGCTCCCGTTCTCATTCAGTTTTTACCCTGGTGATGACCCAGACCAAG
ACAGAAACCGTGGAAGGGGAAGAACATGATCACAGAATAATAAGCCGCATCAATCTGGTA
GACCTGGCAGGCAGTGAGCGCTGTTCTGCAGCCCACACTAGTGAAGATCGACTAAAGGAA
GGTGTGAGCATTAACAAGTCCTTACTAACTTTGGGAAAAGTTATATCTGCACTTTCTGAA
CAAGCAAACCGAAAGAGAGTTTTTATTCCTTACCGTGAATCTGTTCTTACGTGGTTATTA
AAAGAAAGTCTAGGTGGAAATTCAAAAACTGCAATGATTGCTACAATCAGCCCTGCTGCC
AGCAACATAGAAGAAACATTAAGCACACTTAGATATGCTAACCAAGCTCGTCTGATAGTC
AACATCGCCAGAGTAAATGAGGATATAAATGCGAAGTTAATTAGAGAACTGAAAGCAGAA
ATTGAAAAGCTAAAAGCTGCTCAGAGAAACAATCTGAATATTGACCCTGAACGATATAGG
CTTTGTCGGCAAGAAATCACATCCTTACGGATGAAACTCCATCAGCAGGAGAGAGACATG

GCAGAAATGCAAAGAGTATGGAAAGAAAAGTTTGAACAAGCTGAAAAAAGGAAACGTCAA
GAAACAAAGGAGTTACAGAAAGCAGGAATTACATTTCAGATGGACAACCGTTTGCCAAAC
CTTGTTAATCTGAATGAGGATCCACAACTATCTGAGATGCTACTATATATGATAAAAGAA
GGAACAACTACAGTTGGAAAGAATAAACCAAATTCAAGCCATGACATTCAGTTATCTGGG
GTACTAATCGCTGATGATCACTGTACTGTCAGAAATTTTGGAGGGACAGTGAGTATTATC
CCAGTTGCTGAAGCAAAGACATATGTGAATGGGAAACATATTTTGGAACCCACAGTGTTG
CATCATGGTGATCGGGTGATTCTTGGTGGAGATCATTATTTTAGATTTAATCACCCAGTT
GAAGTCCAGAAAGGGAAAAGGCCATCTAGTAGAGATACTTTTATAAGCGAGGGTCCTAAA
GACTTTGAATTTGCCAAAAATGAGTTACTCATGGCACAGAGATCGCAACTTGAGGCAGAA
ATAAAAGAGGCACAGTTGAAGGCAAAGGAAGAAATGATGCAAGGAATCCAAATTGCAAAA
GAAATGGCTCAGCAAGAACTTTCTTCTCAAAAAGCTGCATATGAAAGCAAAATACAAGCA
CTAGAAGCAGAACTGAGAGAAGAATCTCAAAGGAAGAAAATGCAGGAAATAAGTAACCAA
AAGGCTAGTCACAAAATTGAGGAATTAGAAAAGACAAAGCTGCATCTTGAACAGGAAGTG
TATGTCAATAAAAAGCGATTAGAAATGGAAACTTTGGCTACAAAGCAGGCTTTAGAAGAC
CACAGCATCCGTCATGCAAAAATTCTGGAAGCTTTAGAAACTGAAAAGCAAAAAATTGCT
AAAGAAGTACAACTTTTACAGCAGAGTCGGAATAATAGGGGTAAAACTTTTACAATTCAG
CCAAGTTGGAGCTCCATGAAAGTCTCAATGATGATTCAGGAAGCCAATACCATCAGCAGC
AAGTTAAAAAAATACTATGTTTTTGGCAGGCATGATGCATCAGTTAAAGGTAGTTCTGAG
ACATCTATTCGAGTTCGTAACCTGCAGCTTGGGGTCTCAACTTTCTGGAGTCTTGAAAAG
TTTGAATCTAAACTGGCAGCAATGAAAGAACTTTATGAGAGTAATGGTACTAACAGAGGT
GAAGATGTTTTCTGTGACCCTGAAGATGAATGGGAACCTGACATTACAAATGCACCAGTT
TCTTCATTTTCCAGAAGGAGGAGCAGGAGTTTGATGAAAAATAGAAGAATTTCTGATTGT
TTACATGATATACAAGTCCACCCAATGCAGAATCTGTGTTCTTCACGTTCATCAGGTTTA
ATGGAGAAACCAAGCACCATTTACTCAACTTCAGCAGAATCATTTCTTCCTGGAATATGC
AAAGAATTGATTGGTTCATCAATGAATTTTCTTGGACATAGTTTTGATGAAGAAAAAACT
ATAGCAGATAGTCTGATAAATAATCTTTTAAAAATTTATAATGGGCTACTTGTCATCTCC
AAAGCTCATGAAGAACAAGACGAAGAAAGTCAAGACAACTTGTTCTCTTCAGATCGAGCA
GTGCAGTCATGTACTGTCCAGATTGCTTGCTCTTTTGAGCAGCTTGTGGTTTTAATCAGA
CACTGGCTGAACGATTTCCTACCTTGTACCTGCATAGCAAAACTGGAAGATGAATTGAGA
CAGGAAATGAAAAAACTGGGAGGCTACTTACAGTTATTTTTGCAGGGATGCTGTTCAGAT
ATTTCATCAATGGTAAAAGAAGCTGAAAAGAAAGTGATCCAGATCATACAACGAGCTGTT
AAGTATGTGGGAAAGTTAGCAGTTCTAAAAGGGAACAAGCTGTATTTTCTGGAAGGCAGT
CACAATAAGGCTGCCAATGTCCAGGAGGAATTCATCGATGCTATTTGTGATGGTGTAGGC
TCAGGAATGAAGATTCTGTTAGATTCTAGTCTGGAAAAAGCAAAAGAACTTGAATATGAC
CTCTTACGGGAATGTGCACAAAATGAGGTTACCAAACAGATGAAAGCAAATGCCATAGAA
CTGGTCAGATCTCTTGAAAATATCTTTTCTGAATGGAAAACAAAAAGCTTCAGAACTCAA
GTACAAGAAGATGATTCTGGATACCAGGATTCCAAGAAGATAACTGACCTTGCCCTGAAA
TTGTTGAAGTTAAAGCACTGCTTAGAGAAAGCCATTCAAATTAGTATTTCTACACTGAGA
GGATGCTGCAGCGATGTGCATCTCCTCAAGAATTGTGTCGAGACAATTTGCAGCTTGGTC
AGCGATATTTGCAATGACTTCAGTGAGCTCTCTACTTCTGTTGACCACTGTGAAAATAAA
ATATCTCAAGTTGACCACAGGGAACTAGAATCTCTAGCTAAGTCACTCCTCTTATGTTTT
GAATCTGAAGAAAGACCTGATTTATTGAAACCCTGGGAATCTTGTAATCAAGATACTGGA
GGAGGAGAAGAAAAAGAAGAATCTAACTCAGGCCAGACTGGGTGCAGTAGGAATAAAAGT
GTACCGAAGCGTGTCTATGAACTGCATGAGGCGTGCCCAGCAGCGAGCTCGGAGGTATGC
ACACCCAGTAGGGTTCAGTGGGTG
>Canis lupus familiaris ENSCAFT00000017720.4
ATGTCCATATACACCACGTATAATAAAAATAACAGCAATATCCTTGGTATACCTTCTTCC

CAAAGGAGTTCACCATTGAATGTCCTCACCCATAGTAGCAGACGTAAGCTGCATTTGAAT
TCAGATATGTCAAAATGTGAAAATGATGATCCATTATTGAAATCTGCAAGTAAAATCAGA
GACATAAATAGTACTTATGTTATTTCTGCCTGTAAGGAAACAGGAGATGTACCTCTTACC
CCTAACCCCACAGGTAGATTGGCACTTCAGAGAAGAGTTACAAGGAACAAAGAATCATCC
TTGTGTGGCAGAGAGTTGGGAGACTCGACTGAAAAAACTGCAGAAACACGTCTTACATTA
CAACGTCGTGCTAAAACAGAGTCTGTGGCAAAGTGGAAAACAGCTCGAACAGAATCTGTG
AAAACAACACAGAATGTGGGAAGTGCAACAGAAAAGGATTGTGCTTCCCTGGAAGCTAGT
ACGAACGTAACAATTGTAAATAAAGATAAAAACTCTTTTGTTGCATCTTCTGTACCTTTA
GCCAAAGACCCAAAAGACATTGAAATGATGGCTGATGAAAAACACAAAGAAACCTTTTCT
AAAGCTCTCCCTGGGGCAAGTGAAAATGTTGCACTTAAATACTTAAGAAATAGTGCACCC
ACTGATTCCCAGAGTCAGACTAAAGTTGTCCCATCCGGCCACTTGGCAACAAAACCTCTT
CAGAGCAAGTTGAACACCAAAGTGCCAGGAACAGGAGCTTTACATCATGGAAGTATTGGG
AAGGATATGGCGAAACATTCAAATAAGTTTGAAAGCTTAGAAAAAACACCTACAAAATAC
ATTGTAGGGCACAAACTGACACCAAGGTGTGGCACGCCACAGCTTACGAGCCCAGCTGCA
TCAATACTGAAAAATAGGATGCCTAGCCTCCAAGTTAAAAAGCCAAGAAGCTCTGTTCTA
GCAAATGAAAGGGAAAGGTCACAAGAAAATATACTCCCTCTTGAAGAAGAAATTACAGGC
CAGTACACCTCTATAGAGACAGACCCCTTAAAAGTAGAGAATAGTCAAGTGACAGTGGCA
GTACGTGTGAGGCCTTTCAGCAAGAGAGAGATGGTTGAAAAAGCATGCCAGGTGGTCTTC
ATGAATGGGGAAGAAATAACTGTGGAACATCCTGACATGAAACAAGTTTATAATTTTATT
TATAATATTGGATTCTGGTCTTTTGATGAATGCCATCCAAATTATGCCAGTCAGACAACA
GTTTATGAGACTCTAGCAGTACCACTCCTAGAACGTGCCTTTGAAGGCTACAACACCTGT
CTCTTCGCTTATGGTCAGACTGGCTCTGGAAAATCATATACGATGATGGGACTTAATGAA
GAACCAGGAATAATTCCAAGGTTTTGTGAAGATATTTTTGCTCAAGTAGCCAAAAAACAA
ACCCAAGAGGTCAGCTTCCATCTTGAAATGAGCTTTTTTGAAGTATATAATGAAAAAATT
CATGACCTTCTGGTTTGTAAAGGCGAAAATGGGCAGAGAAAGCAGCCACTGAGAGTAAGA
GAGCATCCTGTCTCTGGACCATATGTTGAAGCTCTGTCAATGAATGTTGTCAATTCTTAC
TCTGATATCCAGAGCTGGCTAGAATTGGGAAATAAACAAAGAGCGACTGCTGCTACTGGT
ATGAATGATAAAAGCTCCCGATCGCATTCAGTTTTCACCCTGGTGATGACCCAGACCAAG
ACAGAATCTGTTGATGGGGAAGAACATGATCACAGAATAACGAGCCGAATAAACCTGATA
GATCTGGCGGGTAGTGAGCGCTGCTCCACGGCTCAAACTAGTGGAGATCGACTGAAGGAA
GGTGTGAGTATTAACAAGTCCTTGCTCACATTGGGAAAGGTTATATCTGCACTTTCTGAA
CAAGCAAACGGAAAGAGAGTTTTTATTCCTTACCGTGAATCTGTTCTTACATGGCTGTTA
AAAGAAAGTCTGGGTGGAAATTCAAAAACTGCAATGATCGCTACAATCAGTCCCGCTGCC
AGCAACATTGAAGAAACATTAAACACACTTAGATATGCCAGCCAAGCTCGTATGATAGTC
AATATCGCCAAAGTAAATGAAGATATGAATGCTAAGTTAATTAGAGAATTGAAAGCAGAA
ATTGAAAAGCTAAAAGCTGCTCAAAGAAATAATCGAAATATTGACCCCGAACGATATAGG
CTGTGCCGGCAAGAAATAACATCCTTAAGAATGAAACTGCATCAGCAAGAGAGAGACATG
ACAGAAATGCAAAGAGCATGGAAAGAAAAACTTGAACAAGCTGAAAGAAGAAAACTTCAA
GAAACAAAGGAGTTACAGAAAGCAGGAATTACATTTCAAATGGACAATCATTTGCCAAAC
CTTGTCAATCTCAATGAAGATCCTCAGCTGTCAGAGATGCTATTATATATGATAAAAGAA
GGAATGACAACAGTCGGAAAGTATAAACCAAACTCAAGCCATGATATTCAGTTATCTGGA
GTGCTGATTGCTGATGATCATTGTACCATAAAAAATTTTGATGGGATAGTGAGTATCATC
CCAATTGGAGAAGCAAAGACATATATAAATGGAAAACATATTTTGGAATCCACAGTATTA
CATCATGGTGATCGGGTGATTCTTGGTGGAGATCATTATTTTAGATTTAATCATCCTGTA
GAAGTCCAGAAAGGAAAAAGACCATCTGGTGGAGATACTCTTACAAACGAGGGTCCAAAA
GACTTTGAATTTGCAAAAAATGAGTTGCTTATGGCACAGAGATCGCAACTTGAAGCAGAA
ATAAAAGAGGCACATTTGAAAGCAAAGGAAGAAATGATGCAAGGAATCCAAATTGCAAAA

GAAATGGCTCAGCAAGAGCTTTCTTCTCAAAAAGCTGCATATGAAAGCAAAATAAAAGCC
TTGGAAGCAGAACTGATAGAAGAATCTCAAAGGAAGAAAATGCAGGAAATAAATAATCAA
AAGGCTAATGACAAAATTGAGGAATTAGAAAAGACAAAACAGCGACTTGAACAGGAAATC
TATGTCAACAAAAAGCGGTTAGAAATGGAGACTTTGGCTGCGAAACAGGCTTTAGAAGAC
CACAGTATCCGCCATGCAAGAATTCTGGAAGCTTTAGAAACTGAGAAGCAAAAAATTGCT
AAAGAAGTACAAATATTACTGCAAAATCAAAATAATAGAGAGAAAACTTTTTAATTCAG
ACAAATTGGAACTCCATGAAACTTTCAATGATGATTCAGGAAGCCAATGCCATCAGCAAT
AAATTTAAAAAATGTTATGTTTTTGGCAGACATGATGTATCAGATAAAGGAAGTAGTTCT
GACATTTGCGTTCGGGTTCGTAACCTACAACTAGGGATCTCAACTTTCTGGAGTTTGGAA
AAGTTTGAATCTAAGCTTGCAGCAATGAAAGAACTTTATGAGAGTAATAATAGTAACAAG
AGTGAAGATGTCTTTTGTGATCCTGAAGATGAATGGGAACCTGACATTACAAATGTGCCA
GTTTCTTCCTTTTCTAGAAGGAGGAGCCGGAGCTTGATGAAGAATAGAAGAATTTCTGGT
TGTTTATATGACATACAAGCCCACCCAATTCAGAGTTTGCATTCTTCCCATTCATCAGGC
TTAATGGAGAAGTCGAGCACTATTTACTCAAGTTCAGCAGAATCTTTTCTTCCTGGAATT
TGCAAAGAATTGATTGGGTCATCACTAGATTTTCTTGGACAGAGTTATGATGAAGAAAAA
ACCATGGCGGACAGCCTGATTAATAATTTTCTTAAAATTTATCATGGGCTATTTGCCATT
TCCAAAGCCCATGAAGAACAAGATGAAGAAAGTCCAGATAACTTGTTCTCTTCCGATCGA
GCAGCTCAGGCACTTACTATCCAGGTCACATGTGCTTTTGAGCAGCTTGTGGTACTAATC
AAACATTGGTTGGATGATACTCTGCCTTGTACCAGCACAGCAAGACTTGAAGAGGAATTG
AGACAAGAGGTTAAAAAACTAGGAGGCTACTTACAGTTATTTTTGCAGGGATGTTGTTCA
GATATCTCGTCAATGGTAAAAGAGGCTCAAAAGAAAATAATCCAGACTATACAACAAGCT
GTAAAGTATGTGGGACAATTAGCAGTTCTTAAAGGCAGCAAGTTACATTTCCTGAGAAAC
AGCAGCAATAAGGTTGCTAGTGTTCAGGAGGATTTCATGGATGCTCTTTGTGATGGTGTA
AGTTTAGGAATGAAGATTCTATTAGATTCTGGACTAGAAAAAGCAAAAGAACTTCAGCAT
GAACTGTTAAGGCAGTGTACCCAAAATGAGGTTACCAAACAGATGAAAGCTAAAGCCACG
GAATTGATTGGATGTCTTGAAAATATGTTTGCTGAGTGGAAAACAAAAAGTTTCAGAACT
CAACTACAACAGAATTCTGGATACCAAGATGTGAAGAAGATGGTTAATTTTGCACCAGAA
TTCCTGAAGTTAAAACATTGCTTGGAGCAAACTATTCAGATTATTATTTCTGCATTGAGA
GGATGCCATAGTGACAAAAATCTTCTCCAGAATTGTGTTGAAAGTCTTTGCAGCTTGGCC
AAGGATTTTCATGATGACTTGAGCATGTGCTCTACTTCTCTTGACAACTGTGAGAAGAGA
ATACCCCAAGTTGGCCACGGGGGACTAGAATCTGTAGTCAAGTCACTCCTCTTATGTTTG
GAATCTGAAGAAAGACCTGATTTGTTGAAATCCTGGGAAACTTGTAATCAAAATACCAGG
GAAGAAGGACAACAATCTAAATCAAGCAGGACTGATTCCGTTAGGAATAAAGGTGTACCA
AAGCGTGTCTATGAACTCCATGGCTCATCCCCAGCAGGGACCTCAGAGGAAAGCACACCC
AGGAGGATTCAGTGGGTG
>Loxodonta africana ENSLAFT00000016779.4
ATGTCAGTATACGCTGCTCGTAATCGAAATGACAGCAATATCCTTGGTATTCCTTCTTCC
CAAAAGAATTCCTCACTGAATGTCCTCTCCCAAGGTAGTGGACTTAAGCTGCATTCAAAG
TCAAATATGTCAGAATGCGAAAATGATGATCCATTATTTGGATCTGCAAGTAAAATCAAA
GACGTAAATAGCACTTACGTTATTTCTGCCTGTAAAAAAACGGGAGATCTGCCCCTGACC
CCTAAACCTGTAGGTGGATTGACACTTCAGAGAAGAGTTACAAGGGACAAAGAATCGTCT
TTGCTTGGTAGTGAGCTGGGAGAGACTGCTGAAAAACGTCTTACGTTACAACGTCGTGCT
AAAACAGATTCTATGGAAAAGTGGGAAACAACGCAGAAAATGGGAGGTGAGACAGAAAAT
ACTTGTGCTTCACAGGAAATTAGTAGAAATGTAAAGACTGTAAATACTGATAAAAACTCT
TGTGTTGCATCTTCTGTACCTTTAGCTGAAGACTCAAAAAATGTTGAAATGATGACTGAT
GAAAAATACAAACAGACATTTTCAGCCCCCAGAGATGGAAATGAAAATGGAAATGTTGTG
CTTAAGTACTTAAGCCATAGAACACCCACTGGTTCTCGGAGTCATATTGAAGCTGTTAGA

TCAGGACGCTTGGTAACAAAACCCTCTCAGAACAAGTTGGATATCAGAGTGTCTGGAACA
GAAAACTTATATCATAGAAGTATTGGGAAGGACATTGTGAAAAATTCAGCCATTAAATTG
GGAGCTTTAGAAAAAGGAAGAACACCTGTGAAATGCGTAACAGAACACAGATTAACGCCA
AAGCGCAGTGTGCCTCAGTTGAAGAGCCCAGCTGCATCAGTACTGAAAAATCGAATGCCC
AGCCTTCAAGTTAAACAAAGGCCAAAAAGTTCTCTTCTTGGAAATAAAACTGAAAGGTCA
CAAGAAAATACGCTCCCTCCTGAAGAAGAAGCTGCAGCTCAGAACACCGATATAAAAACA
GACCCCTTAAAAGCAGAGAATAGTCAAGTGACCGTGGCAGTACGCATAAGGCCTTTCAGC
AAGAGAGAAAAGATGGAAAAAGCATCCCAGGTGGTCTTCATGAATGGAGAGGAAATAACT
GTGCAACATCCTGACATGAAACAAGCTTACAATTTTATTTATGATGTTTCATTCTGGTCT
TTTGATGAATGTCATCCTAACTATGCTAGCCAGACAACTGTTTATGAGACGTTAGCAGTA
CCTCTCCTAGAAAGAGCCTTTGAGGGCTACAATACCTGTCTCTTTGCTTATGGTCAGACT
GGCTCTGGAAAATCATATACGATGATGGGATTTAGCGAAGAACCAGGAATAATTCCAAGA
TTTTGTCAAGATCTTTTTGCGCAAGTAGCCAAAAAACGAACGCAAGAGGTCAGCTATCAC
CTTGAAATGAGCTTCTTTGAAGTATATAATGAGAAAATTCACGATCTCCTGGTTTGTAAA
GGTGAAAATGGGCAGAGGAAGCAACCTCTGAGACTGAGGGAGCATCCTATTTCTGGACCC
TATGTGGAAGCGCTGTCAATGAATGTTGTCAGTTCTTACTCTGATATCCAGAGTTGGCTA
GAATTGGGAAATAAACAAAGAGCAACTGCCGCTACTGGCATGAATGATAAAAGCTCCCGA
TCTCATTCCGTTTTCACCCTGGTGATGACCCAGACCAAGACGGAATTGGTGGAGGGGGAA
GAGCATGATCACAGAATCACGAGCCGCATGAATCTGATAGACCTGGCGGGCAGTGAGCGC
TGCTCTGCAGCGCACACTAGTGGAGATCGGCTGAAGGAAGGTGTGAGTATTAACAAGTCC
TTGCTGACTTTGGGAAAGGTTATATCCGCACTCTCTGAACAAGCGAACCAGAGGAGAGTT
TTTATTCCTTATCGTGAATCAGTTCTTACATGGCTGTTAAAAGAAAGTCTGGGTGGAAAT
TCAAAAACTGCAATGATTGCGACAGTCAGTCCTGCCGGCAGCAGTATAGATGAAACCCTA
AGCACGCTTAGATATGCTAACCAAGCCCGTTTGATAGTCAACATTGCCAAAGTAAATGAA
GATACGAATGCTAAGTTAATCAGAGAGTTGAAAGCAGAAATTGAAAAGCTAAAAGCTGCT
CAAAGAAACAATCGACATATTGACCCTGAACGATATAGGCTCTGTCGGCAAGAAATAACA
TCCCTAAGAATGAAACTGCATCAGCAGGAGAAAGACATGGCAGAAATGCAGAGAGTATGG
AAAGAAAAGTTTGAACAAGCTGAAAAAAGAAAACTTCAAGAAACAAAAGAGTTACAGAAA
GCAGGAATTACATTCCAAATGGACAACCACTTGCCAAACCTTGTCAATCTCAATGAAGAT
CCTCAGCTATCGGAGATGTTGCTATATATGATAAAAGAAGGAAAAACCACAGTTGGAAAG
TATAAACCAAACTCAAGCCATGATATTCAGTTATCTGGGGCGCTGATTGCTGATGATCAT
TGTACCATCGAAAATTTTGACGGGACAGTGAGTATTATCCCAGTTGGGGAAGCAAAGACT
TATGTTAATGGAAGACATATTTTGGAAGCCACAGTATTACATCATGGTGATCGGGTAATT
CTTGGAGGTGATCATTATTTTAGATTTAATCATCCAGTAGAAGTCCAGAAAGGGCAAAGA
CCATCCTGTAGAGATGCTGATACTCTTATAAGCGAGGGTACAAAAGACTTTGAATTTGCA
AAAAATGAGTTGCTCCTAGCCCAGAGATCACAACTTGAAGCAGAAATAGAAGAGGCGCGG
TTGAAAGCAAAGGAAGAAATGATGCAAGGAATCCAAATTGCAAAAGAAATGGCTCAGCAA
GAGCTTTCTTCTCAAAAAGCTGCATATGAAAGCAAAATAAAAGCGTTGGAAGCAGAACTG
GAAGAAGAGTCTCAAAGGAAGAAAATGCAGGAAATAAATAACCAAAAGGCTAATCACAAA
ATTGAGGAATTAGAAAAGGCAAAGCAGCATCTTGAACAGGAAATATATGTAAACAAAAAG
CGGTTAGAAATGGAGACTTTGGCTACAAAACAGGCTTTAGAAGACCACAGCATCCGCCAT
GCAAAAATTCTGGAAGCTTTAGAAACTGAGAAGCAAAAAATTGCTAAAGAAGTACAAAGT
CTACAGCAGAATCAGAGTAATAGAGATAAAACTTTTACAATTCAGCCGAATTGGAGCTCC
ATGAAGCTCTCAATGAGGATTCAGGAAGCCAATGCCATCAGCAACAAATTAAGAAAATAT
TATGTTTTTGGCAGACATGATGTATCAGATAGAGGAAGTTCTGAAACTTCTGTTCAGGTT
CGGGTTCGTAACCTGCAACTAGGGATCTCAACCTTCTGGAGTCTGGAAAAGTTTGAATCT
AAGCTTGCAGCAATGAAAGAGCTTTATGAGAGTAATGGTAGTAACAGGGGTGAAGATGTC

TTTTGTGATCCTGAAGATGAGTGGGAACCTGACATTACAAATGCACCAGTTTCTTCATTT
TCTAGAAGGAGGAGCAGGAGTTTGATGAAGAATAGAAGAATTTCTGGTTGTTTACGTGAC
ATACAAGTCCACCCAACTCAGAATTTGCATTCTTCACGTTCGTCAGGCTTAATGGAGAAA
TCAAGTACCATTTACTCCAATTCAGCAGAATCATTTCTTCCTGGAATTTGCAAAGAATTG
ATTGGTTCATCATTAAATTTTCTTGGACAGAGTTATGATGAAGAAAAAACCATGGCAGAC
AGTCTAATTAATAACTTTCTTACAATTTATAATGGCATATTTGCCATTTCCAAAGCCCAT
GAAGAACAAGACGAAGAAAGTCATGATAACTTGTTCTCTTCCGATCGAGCAATCCAGTCA
CTTACTATCCAGATAGCATGTGCTTTTGAGCAGCTCGTGGTGCTGATCAAACACTGGCTG
AATGATGTCCTAGCTTGCAAAAGCACAGCAAGACTGGATGATGAGTTGAGACAAGAAGTT
AAGAAACTGGGAGGCTATTTGCAGTTATTTTTGCAGGGATGTTGTTCGGATATCTCATCA
ATGGTAAAAGAGGCTCAAAAGAAAGTGATTCAAATTGTACAGCAGGCTGTAAAGTACGTG
GGACAATTAGCAGTTCTGAAAGGGAGCAAGCTACGTTTTCTGGAAAACAGTAACAACAAG
GCTGTCAGCCTCCAGGAGGATTTTATGGATGCTCTTTGTGATGGTGTAGGCTTAGGAATG
AAGATTCTATTAGATTCTGGACTAGAAAAAACAAAAGAACTTCAGCCTGAACTCTTAAGG
CTGTGTGCACAAAACGAGGTTACCATACAGATGAAAGCTAATGCCATGGGACTGATTAAA
TCTCTTGAAAATGTCTTTACTGAATGGAAAACAAAAAGTTTCAGAACTCAAGTACGAGAA
GAAAACTGTGGGTACCAAGATCTGAAGAAGATGGTTAATCTTGCCCCAGAGTTCTTGAAG
TTAAAACATTGCTTAGACCAAACTGTTCAAATTATTATTTCTACATTGAGAGGATGCCAC
AGTGATGTAAGTCTTCTCAAGAGGTGTGTTGAAAGTATTTGCAACTTCGCCAGTAATTTT
CATGATTACTTCAGTCACCCCTGTAATTCTGTCAACAGCTGTGAGAATAGAGCACCTCCA
GTTGGCCACAGGGAGCTAGAATCTCTAGCTAAGTCACTCCTCTTATGTTTTGAATCAGAA
GAAAGACCTGATTTATTGAAACCCCGGGAAACTTGTAATCAAAGTACCAGAGAACAACAA
TCTAAATCAAGTAGGACTGAATCTGTTAGGAAAAAAGGTATACCAAAGCGGGTCTATGAA
CTCCATGGCTTGTCTTCAGGAGTGACCTCGGAGGAAAACACATCCAGTAGGATTCAGTGG
GTG
>Mustela putorius furo ENSMPUT00000010565.1
ATGTCAGTATACACCACACATAGTAGAAATAACAGGGATATCCTTGGTATACCTTCTTCC
CAAAAGAGTTCATCACTGAATGTCCTCACCCATAGTAGCAGACGTAAACTGCATTTGAAT
TCGGATATGTCAGAATGTGAAAATGATGATCCGTTTTTGAGATCTGCAAGTAAAATCAGA
GACATAAATTGTACTTACGTGATTTCAGCCTGTAAAAAAACAGGAGAGGTTCCTCTTACC
CCTGACCCTGTAGGTAGATTGACACTTCAGAGAAGAGTTACAAGGAACAAAGAATCATCT
TCACATGGCAGTGAGTTGGGAGACTCAACCGAAAAAACTGCAGAGACACGTCTTACATTA
CAGCGTCGTGCTAAAACAGGATCTGTGGGAAAGTGGAGAACAACTCAAACGGATTCTGTG
GAAAAGGGGAAAACAGCTCAAACAGATTGTGTAAAAAAGTGGAAAACAACACAGAATGTG
GGAAGCCCAACAGAAAACGATTGTGCTTCCCTGGAAGTGAGGAGAAAGGTAACAGTTGTA
AATAATGATAAAGACTCTTCTGTTGCACCTTTTGTACCTTTAGCAGAAGGCCCCAAAGAC
GTTGAAATGATGAATGATGAAAAACACAAAGAAACGTCTTCTGCCCTTCTTGGGGCAAAT
GAACATGTTGCACTTCAATACTTAAGTAATAGAGCACCCACTGATACCCAGAGTCAGACT
AAAGTTGTTCCATCCAGACACTTGGCAACAAAGCCTCTTCAGAGCAAGTTGAGTATCAAA
GTGTCAGGAACAGGAAACTTACATCAGCGAAGTACTGGGAAGGATATGGCAAAAAATTCA
AATAAGTTTGAAAGCTTAGAAAAGAGAACACCTACGAAATACATCATAGGACACAAATGG
ACACCAAAGTGTGGCACGCCACAGCTTATGAGCCCAGCTGCATCGACACCGAAAAATAGG
GTGTCTGGCCTCCAAGTTCAACAAAAGCCAAGAGGTTCTCTTCTAGCAAATGAAAGGGAA
AGGTCACAAGAAAATACACTCCCTCTTGAAGAGGAAACGGCAGGTCGGAACACTTCTATA
GAAGCAGACCCCCTAAAAGTAGAGAACAGTCAAGTGACAGTGGCAATACGTGTGAGGCCT
TTCAGCAAAAGAGAGAAGGTTGAAAAGGCATCCCAGGTGGTCTTCATGAATGGGGAAGAA
ATAGTTGTGGAACATCCTGACATGAAACAAGTTTATAATTTTATTTATGATCTTACATTC

TGGTCTTTTGATGAATGTCATCCCAACTATGCTAGCCAGACAACTGTTTATGAGATGCTA
GCTGTACCCCTCCTAGAACGCGCCTTTGAAGGCTATAATACCTGTCTGTTCGCTTATGGT
CAGACTGGCTCAGGAAAATCATATACGATGATGGGACTTAGTGAAGAACCAGGAATAATT
CCAAGGTTTTGTGAAGACCTTTTTGCTCAAGTAGCCAAAAAACAAACTCAGGAGGTCAGC
TACCACCTTGAAATGAGCTTTTTTGAAGTGTATAATGAAAAAATTCATGACCTTCTGGTT
TGTAAAGGAGAAAACGGGCAGAGAAAGCAACCACTGAGAGTAAGGGAGCATCCTGTCTCT
GGGCCGTATGTCGAAGCTCTGTCAATGAATGTTGTCAGTTCTTACTCTGACATCCAGAGT
TGGCTAGAATTGGGAAATAAACAAAGGGCAACTGCTGCTACTGGTATGAATGATAAAAGC
TCCCGATCGCATTCGGTTTTCACCCTGGTAATGACCCAGACCAAGACAGAATCTGTGGAA
GGGGAAGAACATGATCACAGAATAATGAGTCGGATAAACCTGATAGATCTGGCGGGCAGT
GAGCGCTGCTCCACAGCTCAAACTAGCGGAGATCGACTGAAGGAAGGTGGGAGTATTAAT
AAGTCTTTGCTCGTGCTGGGAAAGGTCATATCGGCTCTTTCTGAACAAGCAAACCGAAAG
AGAGTATTTATTCCTTACCGTGAATCTGTTCTTACATGGCTGTTAAAAGAAAGTTTGGGT
GGAAATTCAAAAACTGCAATGATCGCTACTGTCAGTCCCGCTGCCAACAACATTGAAGAA
ACTTTAAGCACACTGAGATATGCCAGCCAAGCCCGTATGATAGTCAATATTGCCAAAGTA
AATGAAGATATGAATGCTAAGTTAATTAGAGAATTGAAAGCAGAAATTGAAAAGCTAAAA
GCTGCTCAAAGGAACAGTCGGAATATTGACCCTGAACGATATAGGCTATGCCGGCAAGAA
ATAACATCCTTAAGAATGAAACTGCACCAACAGGAGAGAGACATAGCAGAAATGCAAAGA
GCATGGAAAGAAAAATTTGAACAAGCTGAAAGAAGAAAACTTCAAGAAACAAAGGCATTA
CAGAAAGCAGGAATTACATTTCAAATGGACAACCATTTGCCAAACCTCGTCAATCTCAAT
GAAGACCCTCAATTATCAGAGATGCTGTTATATATGATAAAAGAAGGAACGACAACAGTG
GGAAAGTATAAACCAAACTCAAGCCATGACATTCAATTATCTGGGGTACTGATTGCTGAT
GATCATTGTACTATAAAAAATTTTGATGGAATAGTGAGTATCATCCCAGTTGGAGAAGCA
AAGACATATATAAATGGAAAACACATTTTGGAATCCACAGTATTACATCATGGTGATCGG
GTGATTCTTGGTGGAGATCATTATTTTAGATTTAATCATCCAGTAGAAGTCCAGAAAGGA
AAAAGACCATCCGGTAGAGATACTCTTATAAGCGAGGGTCCAAAAGACTTTGAATTTGCA
AAAAATGAGTTGCTCATTGCACAGAGATCACAACTTGAAGCAGAAATAAAAGAGGCACAA
TTGAGAGCAAAGGAAGAAATGATGCAAGGAATCCAAATTGCAAAAAAAATGGCTCAACAA
GAGCTTTCTTCTCAAAAAGCTGCATATGAAAGCAAAATAAAAACATTGGAAGCAGAACTG
AAGGAAGAGGCTCAAAGGAAGAAAATGCAGGAAATAAATAACCAGAAGGCTAATGACAAA
ATTGAGGAATTAGAAAAGGCAAAACAGCAACTTGAGCAGGAAATCTATGTCAACAAAAAG
CGATTAGAAATGGAGACTTTGGCTGCAAAACAGGCTTTAGAAGATCATAGCATCCGCCAT
GCAAGAATTCTGGAAGCTTTAGAAACTGAGAAGCAAAAAATTGCTAAAGAAGTACAAATA
CTGCAGCAAAATCAAAGTAATAGAGATAAAACTTTTTTAATTCAGACAAATTGGAGCTCC
ATGAAACTCTCAATGATGATTCAGGAAGCCAATGCCATCAGCCACAAATTTAAAAAATGT
TATGTTTTTAGCAGACATGATGTATCAGATAAAGGAAGTAATTCTGACACTTATGTTCGG
GTTCGTAACCTACAACTAGGGATCTCAACTTTCTGGAGTCTGGAAAAGTTTGAATCTAAG
CTTGCAGCAATGAAAGAACTTTATGAGAGTAATAATAATAACAAGAGTGATGATGTCTTT
TGTGATCCTGAAGATGAATGGGAACCTGACATTACAAATGCACCGGTTTCTTCCTTTTCT
AGAAGGAGGAGCAGGAGCTTGATGAAGAATAGAAGAATTTCTGGTTGTTTACATGACATA
CAAGCCCACCCAATTCAGGATTTGCATTCTTCCCATTCATCAGGATTACTGGAGAAATCG
AGCCCTATTTACTCAAGTTTAGCAGAATCTTTTCTTCCTGGAATTTGCAAAGAATTGATT
GGGTCATCCTTAAGTTTTCTTGGACAGAGTTATGATGAAGAAAAAACCATGGCTGACAGT
CTGATGGATAATTTTCTTAAAATTTATCACGGACTATTTGCCATTTCCAAAGCTCATGAA
GAACAAGATGAAGAAAGTCAAGAAAACTTGTTCTCTTCTGATCGAACAACCCAGTCACTT
ACAATCCAGGTCACCTGTGCTTTTGAGCAGCTCGTGGTGCTAATCAAACACTGGCTGAAT
GAAATTCTGCCTTGTATCAGCACAGCAAGGCTTGAAGATGAATTGAGACAAGAGATCAAA

AAACTAGGAGGCTACATACAGTTATTTTTACAGGGATGTTGTTCAGATATCTCATCAATG
GTAAAAGAGGCTCAAACTAAAATAATCCGAATTGTACAACAAGCTGTAAAGTATGTGGGA
CAGTTAGCAGTTCTTAAAGGGAGCAAGCTGCATTTTCTGGAAAATAGTAGCAATAAGGCT
GCCGGTATTCAGGAGGACTTCATGGATGCTCTTTGTGATGGTGTAGGCTTAGGAATGAAG
ATTCTATTAGATTCTGGACTAGAAAAAGCAAAAGAACTTCAGCATGAACTCTTAAGTCAG
TGTACCCAAAATGAGGTTACCAAACAGATGAAAGGTAAAGCCATGGGATTGATTGGATGT
CTTGAAAACATGTTTGCTGAGTGGAAAACAAAAAGTTTTAGAACTGAACTACAACAAGAA
GGTCCTGGATACCAAGATTTGAAGAAGACGATTAATCTTGCACAGGAATTCCTGAAGTTA
AAACATTGCTTGGAGCAAACTATTCAAATTATTATTTCTGCACTGAGAGGATGCCACATG
GACAAAAATATTCTCAAGAATTGTGTTGAAAGTCTTTGCAACTTGGCCAAGGATTTTCAT
GATGATTTGAGCATGTGCTCTACTTCTCTTGACAGCTGTGAGAAGAGACTACCTCGAGTT
GGCCATGGGGGACTAGAATCTCTAGCCAAGTCACTCCTCTTATGTTTTGAATCAGAGGAA
AGAGCTGATTTGTTGAATCCCTGGGAAACTTGTAATCAAAATACCAGAGAAGAAGGACGG
CAATCTAAATCAAGCAGGACCGACTCCATTAGGAATAAAGGTGTACCAAAGCGTGTCTTT
GAACTCCATGGCTCGTCCCCAGCAGTGACCTCAGAGGACAGCATGCCCAGTAGGATTCAG
TGGGTG
>Ovis aries ENSOART00000017553.1
ATGTCAGCCTCCACCGCACACAGCAGAAATGACAGAGATCTCCTTGGTGTTCCTTCCACA
AAGACGTCATCCCTGAGTGTCCTCCCCCAGGGCAGCAAGCTGAAGCTGCATCCGAGGTCA
GACTCGTCAGAGTGTGAAAATGATGATCCGTTACTGAGATCTGCAAGTAAAGTCAGAGAC
ATCAACAGCACTTACGTTATTTCTGCCTGTAAGAAAACAGAAGACACACGCCTCCTTCCT
AACCCTGTGGGGAGGTTGACACTTCAGAGGAGAGCGACCAGGAGCAAGGAACCCTCTTTG
CTTGGTCGTGAGTCGGGAGACGCCGGTGCGAAAACGGCAGAAACACGCCTTGCATTACAG
CGTCGGACTAAGACAGATTACATGGGAAAGTGGAAAGGAATGCAGGACTTGGGCGGCAGG
ACAGAAAATAGTCGGCCTTCACCAGAAACTGGTATAAGTGTAAAAGCTGTAAGCAGTGAT
AAAGACTTGCCTGTTGCACCTTCTGTGCCTTTAACTCCAGACCCCAAAGACCCTGAAATG
AAGGCTGATGGAAAATGCGCAGCGACGCGTTCTGCCCTCAGTGGGGTGGGTGAGAACGTT
GCCCTTAAGTCCCTACGTGAGCGAGCACCCGCCGGTTCCCAGAGTCAGACTGAAGCTGTT
CGCACACCGTGCGTGGCAGTTAGGCCCACTGAGAGCAGGTTGGAGACCAGAGAGTCAGGA
GCAGGGGCCTCGCATCACAGGGGTGCTGAGAAGGAAGCCACGAAACTTCCACGTAGATTT
GAAAGCTTAGAGAAAAGGACACCCTTCAAGTGTATTTCAGAGCTCAGGTCGACACCTAGG
CGTGGCGTCCTGCAGCCTCCGAGCCCCGCTGCCTTGGTTCTGAGAAGCAAGAGGCCCACC
CTGCAAGTTAAACAGACGCCCCAGAGCTCTCTTCTTGCAAGTCAGAGGGAAAGGTCACGG
GAAAATACATTCCTTCTTGAGGAAGAAACCGCACTTCAGAAAACCTCTGCAGAAACAGGG
CCCCTGAAGGTGGAGAGCAGCCAAGTGACAGTGGCGGTCCTTCCAAAACCCTTTCCAGAA
AGGGAGAAGAGAGAAGGCGCGCCTCAGGCGGTCTTCCTGGATGGGGAGGAAATCGCTGTG
GAGCACCCTGGCACGCGACAGGTGTACAGCTTTGCCTACGATCTTTGCTTTTGGTCCGTG
GACGAGCGCCACCCGCGCTTCGCGAGCCAGATGGCCGTGTACCAGGCGCTGGCGGCCCCG
CTGCTAGGACAGGCCTTCCAAGGCTTCAACACCTGTCTGTTCGCGTATGGCCAGACTGGC
TCTGGAAAGTCATACACGATGATGGGCTTTAGTGAAGAACCAGGAATAATTCCAAGATTT
TGTGAAGATCTTTTTGCTGAAATAGCCAAAAAACAAACGGAAGAGGCCAGCTACCACCTT
GAGATGAGCTTCTTCGAAGTCTACAATGAAAGAATTCATGACCTTCTTGTTTGTAAAGGT
GAAAATGGGCAGAGAAAGCAAACGCTGCGAGTGCGGGAGCACCCTGCCTCGGGCCCGTAC
ATCGAGGGCCTGTCCACGAATGTTGTCAGTTCTTATTCTGATATCCAGGTCTGGCTGGAG
CTGGGGAACAAGCAGAAGGCCACAGCCGCCACCAGCATGAACGACAAGAGCTCGCGCTCT
CACTCCGTGCTCACGCTGGTGATGACCCAGGCCAAGACAGAATTTGTGGAGGGGCAGGAG
CTGGACCACAGAATTCGGAGCCGCATAAACCTCGTGGACCTGGCTGGCAGTGAGCGGTGT

TCAGCGACCGGGACCAGCGGGGAGCGGCTGAAGGCTGGGATAGTGCGGGTGAATGATGGA
TCAGTGTGTCTTTATTATGTTTTAATTGCCACCTCAAAGCTTCTCAGAGAGCACCTCAAG
CTTATTTTTCCCTCTCAAATAAGGCTTTTGCATATGTTGGTAACAGAGAACAAAGCTTGT
GTTTATGATGTTCATCCATCATTTTCTGCACTTGCAAGCAGGGGCTTTGAACCATCGTGG
TGGCTCCAGAATTTCACTAAAGATAGGATAGTTACCCACGTTTTAAAATCCCCGCACAGA
CTGTTGTTTCACAAATGTTTAGAATTGAAAGCAGAGATTGAAAAGTTAAAAGCTGCCCAA
AGAAGCAGTCAGAACATTGACCCTGAGCGATACAGGCTCTGCCGGCAAGAAATAACCTCC
TTGAGAATGAAGCTGCATCAGCAGGAGAGACACATGGCAGACATGCAGAGCGAAGAAAAG
CTTGAACAAGCCGAAAAAAGAAGACTTCAAGTAACAGAGGAGCTGCAGAAAGTAGGAATT
GCCTTTCGAATGGATGACCATTTGCCAAACCTGGTCAATCTCAGCGAAGACCCTCAGCTG
TCAGAGATCCTGTCATACATGATTAAAGAGGGGACAACCACAGTCGGAAAGTGCAGACCC
GGCTCCAGCCACGATATCCAGCTGTCGGGGGTGCTGATCGCCGACGACCACTGCACCATC
ACCAATTCCGATGGGACAGTGAGTATCATCCCGGCGGGGGAAGCGAAGACATACGTAAAT
GGAAAGCTGATTTCGGAGCCGACGGTGTTGCACCATGGTGATCGGGTGATTCTTGGTGGC
GATCATTATTTTAGATTTAATCATCCAGTGGAAGTCCAGAAAAGGGAGCAGCCACCCAGC
AGAGAGAACCCTGTGAGCAAGGGCCCAGAAGACTTCGAGTCAGCCAGAAACGATTTGCTG
ATGGCCCAGAGGTTACAGCTTGAGGCAGAAATAAAGGAGGCTGAGGTGAGAGCCAAGGAG
GAGATGATGCAAGGGATCCAGATCGTGAAAGAAATGGCCCAGCAAGAGCTCTCCTCTCAG
AAAGCTGCGTATGAGAGCAAGATCAAAGTGCTGGAGGCAGAGCTGAAAGAAGAATCTCAA
AGGAAGAAGATCCAGGAAATAAATAACGAAAAGGCGAATCACAAGATTGAGGAACTGGAA
AAGGCGAAGCAGCGCTTTGAGCAGGAAATTCACGTCAACAGGAAGCGGCTGGAAATGGAG
GCCCTGGCGGCGAAGCAGGCTTTAGAAGACCACAGGATTCGGCACGCAAAAATTCTGGAA
GCTCTAGAAACAGAGAAGCAAAGGATTGCTCAGGAAGTGCAGATTCTGCAGCAGAATCAG
AGCCACAGGGATAAAGCCTTCGCAGCGCAGCCCAGTTGGAGCTCCATGAAGCTGTCCGTC
ATGATTCAGGAAGCCAACGCCATCAGCAGCAGGCTAAACAGGAATTACGTTTTTGGCAGA
CATGTGGTGTCAGATAAAGGAAGCAGCTCTGACGCTTGTATTCGGGTCCGTAACCTGCAG
CTCGGGGTCTCAACCGTCTGGAGTCTGGAAAAGTTTGAGTCTAAGCTTGCGGCAATGAAG
GAACTTTATGAGAGCCGTGGCAGTAGCCGGGGTGAAGATGTCTTTTGTGGCCTGAAGAT
GAGTGGGAACCCGACATCACCCATGTTCCTGTTTCTTCCTTTTCCAGGAGGAGGAGCAGA
AGTCTGCTGAACAACAGGAGGGTTTCCGGCTGTCTGCACGGCGTCGAGGCCCACGCGGGC
CGGGACCTGCGTTCTTCAGGCTGTGCAGACACAGGCTTAGTGGGGAAATCCAGCCCTGTT
TACCTGGACTCAGCGGAACCCTTCCTTCCTGGCATTTGCAAAGAGTTGATCGGCTCGTCG
TTAGAGCTTCTTGGACAGAGTGATGATGAAGAAGGCACTGTGGCAGACAGCCTGATGAGC
AGTTTCCTCAGAATCCACGACGGGGTACTGGCCGTGTCCAGAGCCCACGAAGAGCAGGAC
GAGGACAGTCAGTGTAGCTTGTTCACTGACCGAGCAAACCAGTCGCTCGCAGTCCAGGTG
ACAAGCGCGTTCGAGCAGCTTGCGGTGCTGACGGGACCCTGGGTGAGCGGTGTCCTCCTG
GAGGCTGGCCGGGCGGCCCTGCTGGCCGAGCTGCGGCAGGAAGTGAGGACGCTAGGGAGC
CGCTTGCAGCTGTTTCTGCAGGGATGCTCTTCGGATATTTCACCGATGGTCAAAGAGGCT
CAAAAGCAAGTGATCCAAACCGTGTGCCGAGCCGTCGCGTGTGTGGGGCGGTCAGTGGCC
CTCACTGGGGGCCGGCTGCATCTGCTGGAGAGTGGCGCTCACGGGGCCCCGGGCCTGCAG
GATGATTTTGTGGGTGCTGTTTGTGATGGTGTAGACATGGGAGTCAGGAGTCTCCTCGAT
GCTGGGATAGAAAAAGCAGAAGAGCTTGAGCGTGAACTTGGAAGACAGAGTCCCCAAGAT
GAGGTTACCAAACAGATGAAAGCTAATGCCCTGGGACTGATCGAATCCCTCGGAAACCTC
TTCGCTGAATGGAAAACAAAAAGCTTCAGAACTCAAGTACAAGAAGAAAATTCTAAATAT
AAAGATTTGAAGATGATGATCAATCTTGCCCAAGAATTCTTCAAGTTAAAATGTGGCTTA
GAGCAAACTATTCAAATTATTATTTCTGCGCTGAGAGGGCACCGCGGGGACGTGGCCCTC
CTCCAGGACTGTGTGCAGCGTCTCTGCAGCTCTGCCCGGGGTCTCCATGGCGACCGGGCT

GGGTGCCCTGAGGACAGCAGAGCCCAAGCCAGCCGCCGGGAGCTGGAGTCCCGCGCCACC
TCACTGCTCTTGGGCTTTGAATTTGAAGAAGGACCTGTTTTGTGGGGGCCCTGGGAAGCT
TGCGATCAGGGTCCCAGAGCAGCAGAACAAGAGCAGCCCAGGTCGGACAGGCCCGGCCCC
CAGAGGACCCGAGGAGTGCCCAAGCGGGTGTACGAGCTCCCGGGCCCGGCCCTGGGGGGC
TCAGAGCAGGGTGTGCTGCCCACCCGGGAGAGCGCCGGGGAGGGATCTCCTCCCGCCCGA
GAGCAG
>Ailuropoda melanoleuca ENSAMET00000013855.1
ATGTCAGTATACAATGCACATAATAGAAATAACAGGGATGTCCTTGGTATACCTTCTTCG
CCAAAGAGTTCATCACTGAATGTCCTCACCCATAGTAGCAGACGTAAGCTGCATTTGAAT
TCGGATATGTCAGAATGTGAAAATGATGATCCATTATTGAGATCTGCAAGTAAAATGAGA
GACATAAATAGTACTTATGTGATTTCTGCCTGTAAAAAAACAGGAGAGGTACCTCTTACC
CCTGACCCCGTAGGTAGATTGGCACTTCAGAGAAGAGTTACAAGGAACAAAGAATCATCC
TTGTGTGGCAGTGAGCTGGGAGCCTCAACTGAAAAAACTGCAGAGACACGTCTTACATTA
CAACGTCGTGCTAAAACAGAATCTGTGGAAAAGTGGAAAACAGCTCAAACAGATTCTGTG
AAAATAACACAGAATGTGGGAAGCCCCACAGAAAATGATTGTGCTTCCCTGGAAGCGAGG
AGAAAGGTAACAATCATAAATAATGATAAAGACTCTTTCGCTGCACCTTTTGTACCTTTA
GCCGAAGACCCAAAAGACATTGAAATGATGGCTGATGAAAAACACAAGGAAACATTTTCT
GCCCTCCCTGGGGCAAATGAACACGTTGCACTTAAATACTTAAGTAATAGAGCACCCACT
GATCCCCAGAGTCAGACTAAAGTTGTTTCCTCTGGACACTTGACAACAAAGCCTCTTCAG
AGCAAGTTGAACATCAAAGTGCCAGGAACAGCAAACTTACATCACAGAAGTATCGGGAAG
GATATGGCAAAAAATTCAAATAAATTTGAGAGCTTAGAGAAAAGAACACCTGTGAAGTAC
ATTGTAGGACACAAATGGACACCGAAGTGTGGCACGCCACAGCTTACGGGCCCAGCTGCG
TCGACACTGAAAAATAGGGTGCCAGGCCTCCAAGTTAAACAAAAGCCAAGAAGTTCTCTT
CTAACAAATGAAAGGGAAAGGTCACAAGAAAATACACTCCCTCTTGAAGAGGAAATGGCA
GGTCGGAACACCTCTATAGAAACAGACCCCTTAAAAGTAGAGAACAGTCAAGTGACAGTG
GCAGTACGTGTGAGGCCTTTCAGCAAGAGAGAGAAGGTTGAAAAAGCATCCCAGGTGGTT
TTCATGAATGGGGAAGAAATAGCTGTGGAACATCCTGACATGAAGCAAGTTTATAATTTT
CTTTATGATGTTGCGTTCTGGTCTTTTGATGAATGCCATCCCAACTATGCTAGCCAGACA
ACTGTTTATCAGACGCTAGCAGTACCGCTCCTAGAACGCGCTTTCGAAGGCTACAATACC
TGTCTCTTCGCTTATGGTCAGACCGGCTCTGGAAAATCATATACGATGATGGGACTTAGT
GAAGAACCAGGAATAATTCCAAGGTTTGTGAAGATCTTTTTGCTCAAGTAGCCAAAAAA
CAAACCCAAGAGGTCAGCTACCACCTTGAAATGAGCTTTTTTGAAGTATATAATGAAAAA
ATTCATGATCTTCTGGTTTGTAAAGGCGAAAACGGGCAGAGAAAGCAACCACTGAGAGTA
AGGGAGCACCCTGTCTCTGGGCCGTATGTTGAAGCTCTGTCAATGAATGTTGTCAGTTCT
TACTCTGATATCCAGAGTTGGCTAGAATTGGGAAATAAACAAAGAGCAACTGCTGCTACT
GGTATGAACGATAAAAGCTCTCGCTCGCATTCGGTTTTCTCCTTGGTGATGACCCAGACC
AAGACAGAATCTGTGGAAGGGGAAGAACACGATCACAGAATAACGAGTCGGATAAACCTG
ATAGATCTGGCGGGCAGCGAGCGCTGTTCCACAGCTCAAGCCAGCGGAGATCGACTGAAG
GAAGGTGTGAGTATTAACAAGTCCTTGCTCACATTGGGAAAGGTTATATCTGCGCTTTCT
GAACAAGCAAACCGAAAGAGAGTTTTTATTCCTTACCGTGAATCTGTTCTTACATGGCTG
CTAAAAGAAAGTCTGGGTGGAAATTCAAAAACTGCAATGATTGCTACCATCAGTCCCGCT
GCCAACAACATTGAAGAAACATTAAGCACACTGAGATATGCCAGCCAAGCCCGAATGATA
GTCAATATAGCCAAAGTAAACGAAGATGTGAATGCTAAGTTGATTAGAGAATTGAAAGCA
GAAATTGAAAAGCTAAAAGCTGCTCAAAGAAACAGTCGAAATATTGACCCTGAACGATAT
AGGCTGTGCCGGCAAGAAATAACATCCTTAAGAATGAAACTGCATCAACAGGAGAGAGAC
ATGGCAGAAATGCAAAGAGTGTGGAAAGAAAAAATTTGAACAAGCTGAAAGAAGAAAACTT
CAAGAAACAAAGGAGTTACAGAAAGCAGGAATTACATTTCAAATGGACAACCATTTGCCA

AACCTCGTCAATCTCAATGAAGACCCTCAGCTATCAGAGATGCTGTTATATATGATAAAA
GAGGGAACGACGACAGTAGGAAAGTATAAACCAAACTCCAGCCACGATATTCAGTTATCT
GGGGTGCTGATTGCTGATGATCATTGTACGATAAAAAATTTTGATGGGATAGTGAGTATC
ATCCCAGTTGGAGAAGCAAAAACATATATAAATGGAAAACACATTTTGGAATCCACAGTA
CTACATCATGGTGATCGGGTGATTCTAGGTGGAGATCATTATTTTAGATTTAATCATCCG
GTAGAAGTCCAGAAAGGAAAAAGACCATCTGGTAGAGATACCCTTACAAGTGAGGGTCCA
AAAGACTTTGAATTTGCAAAAAATGAGTTGCTCATGGCACAGAGGTCACAACTTGAAGCA
GAAATAAAAGAGGCACAATTGAAAGCAAAGGAAGAAATGATGCAAGGAATCCAAATTGCA
AAAGAAATGGCTCAGCAAGAGCTTTCTTCTCAAAAAGCTGCGTATGAAAGCAAAATAAAA
GCATTAGAAGCAGAACTGAAAGAAGAAGCTCAAAGGAAGAAAATGCAGGAAATAAATAAC
CAAAAGGCTAATGACAAAATTGAGGAATTAGAAAAGGCAAAACAGCGACTTGAACAGGAA
ATCTATGTCAACAAAAAGCGATTAGAAATGGAGACTTTGGCTACAAAACAGGCTTTAGAA
GACCACAGCATTCGCCATGCAAGAATTCTGGAAGCTTTAGAAACTGAGAAGCAGAAAATA
GCTAAAGAAGTACAAATACTGCAGCAAAATCAAAGTAATAGAGATAAAACTTTTTTAATT
CAGACCAATTGGAGCTCCATGAAGCTCTCAATGATGATTCAAGAAGCCAATGCCATCAGC
AGCAAATTTAAAAAATGTTATGTTTTTGGCAGACATGATGTATCAGATAAAGGAAGTAGT
TCTGACACTTCTGTTCGGGTTCGTAACCTACAACTAGGGATCTCAACTTTCTGGAGTCTG
GAAAAGTTTGAATCTAAGCTTGCAGCAATGAAAGAACTTTACGAGAGCAATAACAGTAAC
AAGAGTGAAGATGTCTTTTGTGATCCTGAAGATGAATGGGAACCTGACATTACAAATGCA
CCAGTTTCTTCCTTTTCTAGAAGGAGGAGCAGGAGCTTGATGAAGAATAGAAGAATTTCT
GGTTGTTTACATGATATACAAGCCCACCCACTGCAGAATTTGCATTCTTCCCATTCATCA
GGCATACTGGAGAAATCGAGCGCTATTTACTCAAGTTCAGCAGAATCTTTTCTTCCTGGA
ATTTGCAAAGAATTGATTGGGTCATCCTTAGATTTTCTTGGACAGAGTTATGATGAAGAA
AAAACCATGGCAGACAGCCTGATTAATAATTTCCTTAAAATTTATCACGGGCTATTTGCC
ATTTCCAAAGCTCATGAGGAACAAGATGAAGAAAGTCAAGATAACTTGTTCTCTTCTGAT
CGAGCAACGCAGTCACTGACTATCCAGGTCACATGTGCTTTTGAGCAGCTCGTGGTGCTA
GTCAAACACTGGCTGAATGATATTGTGCCTTGTGCCAGCACAGCAAGACTTGAAGATGAA
TTGAGACAAGAGGTTAAAAAACTAGGAGGCTACTTACAGTTATTTTTACAGGGATGTTGT
TCAGATATCTCATCAATGGTAAAAGAGGCTCAAAAGAAAATAATCCAAATTGTACAACAA
GCTGTAAAGTATGTGGGACAGTTAGCAATTCTTAAAGGGAGCAAGCTGCATTTTCTGGAA
AATAGTAGCAACAAGGCTGCCAGTGTTCAGGAGGATTTCGTGGATGCTCTTTGTGATGGT
GTAGGCTTAGGAATGAAGATTCTATTAGATGCTGGACTGGAAAAAGCAAAAGAACTTCGG
CATGAACTCTTAAGGCAGTGTACCCAAAATGAGGTTACTAAACAGATGAAAGCTAAAGCC
ATGGGACTGATTGGATGTCTTGAAAACATGTTTGCTGAGTGGAAAACAAAAAGTTTCAGA
ACTGAACTACAACAAGAAAATTCTGGATACCAGGATTTGAAGAAGATGGTTAATCTTGCA
CCAGAATTACTGAAGTTAAAACATTGCTTGGAGCAAACTATTCAAATTATTATTTCTGCA
CTGAGAGGATGCCACAGTGACAAAGATCTTCTGAAGAATTGTGTTGAAAGTCTTTGCAAC
TTGGCCAAGGATTTTCATGATGACTTGAGCATGCGCTCTACTTCTCTTGACAGCTGTGAG
AAGAGAATACCTCAAGTTGGCCACGGGGAACTAGAATCTCTAGTCAAGTCACTCCTCTTA
TGTTTTGAATCTGAAGAAAGACCTGATTTGTTGAATCCCTGGGAACCTTGTAACCAAAAT
ACCAGAGAAGAAGGACAGCAATCTAAATCAAGCAGGACCGACTCCGTTAGGAGTAAAGGT
GTACCAAAGCGTGTCTATGAACTCCATGGCTTGTTGCCAGCAGTGACCTCAGAGGACAGC
ACACCCAGTAGGATTCAGTGGGTG
>Felis catus ENSFCAT00000029134.1
ATGTCCGTGTTCACCACACATAATAGAAACAACAGCGATATCCTTGGTATACCTCCTTCC
CCAGGGAGTTCCTCGCTGAATGTCCTCACCCATGGTAGCAGACCTGAGCTGCATGTGATG
TCGGATATGACAGAATGTGAACACGATGATCCATTATTGAGATCGGCAAGGAAAATCAGA

GACATAAACAGTACTTATGTGATTTCTGCCTGTAAAAAAACAGGAGATGTACCTCTGACC
CCTAACCCTGTAGGTAGATTGGCACTTCAGAGAAGAGTCACAAGGAACAAAGAATCCTCT
TTGCTTGGCAGTGAGTTGGGAGACTCCGCCGAAAACACTGCAGAAACACGTCTTACGTTG
CGGCGGCGTGCTAAAACAGAATCAGTGGAGAAGCGGAAAACAGCTCAAACAGATTGTGGG
GAAAAGTGGAAAACAACACAGAATGTGGGCAGTGCAGCCGAAAATGATGGTGCCTCACTG
GAAGCTAGTACAAATGCAAGGACTGTACATAAAGATAAAAACCCTTGTATTGCACCGGTG
GTACCTTTATCGGACCCAAAACGCATTGAAATGACGGCCGATGAAAAACACAAAGAGACA
TTTGTTGCCCTCCCTGGGGCAAATGAAAAGGTCGCACTTAAGTACTTAAGTAACAGAGCA
CCCACTGGTTCCCAGGGTCAGACTGAAGCTGTTCCATCGGGACGCTTGGCAACCAAACCT
CTTCCGAGCAAGGTGAACATCAAAGTGTCCGGGAACCTACCTCACAGAAGTATTGGGAAG
GATACTGCAAAATCTGCAAATAAATTTGAAAGCTTAGAAAAAAGAAGAACACCTACAAAA
TACATCGCAGGACACCGATTGACACCGAAGTGTGACACGTCACAGCTGACGAGCCCAGCT
GCGTCGTCACTGCTGAAAAAGAGGGTGCCTAGCCTGCAAGCTACACAAAAGCGAACAAGT
TCTCTGCCTGCAAATGAAAGGGAAAGGTCACGAGAAAATACACTCCCTCTTGAAGAGGAA
ACCGCAGGTCAGAACCCCCCTGCAGAGACAGACCCCTTAAAGGTGGAGAACAGTCAAGTG
ACAGTGGCAGTACGGGTGAGGCCTTTCAGCAAGAGAGAGAAGGTTGAAAAAGCATCCCAG
GTGGTCTTCATGAACGGAGAGGAAATAGCTGTGGAACATCCCGACATGAAACAAGTTTAT
AATTTTATTTATGATGTTGCATTCTGGTCTTTTGATGAATCCCATCCAAACTATGCCAGC
CAGACAACTGTTTATGAGACCCTAGCTATTCCGCTCCTGGAACGAGCCTTTGAAGGCTAC
AATGCCTGTCTCTTTGCTTATGGTCAGACCGGCTCTGGAAAATCATATACGATGATGGGA
CTTAGTGAGGAACCAGGAATAATTCCAAGGTTTTGTGAAGATCTTTTTGCTCAAGTAGCC
AAAAAACAAACCCAAGAGGTCAGCTACCACCTTGAAATGAGCTTTTTTGAAGTATATAAT
GAAAAAATTCATGACCTTCTGGTTTGTAAAGGTGAAAATGGGCAGAGAAAGCAACCACTG
AGAGTAAGGGAGCATCCTGTTTCTGGACCATATGTTGAAGCTCTGTCAATGAATGTTGTC
AGTTCTTACTCTGATATCCAGAGTTGGCTAGAATTGGGAAATAAACAAAGAGCTACGGCT
GCTACTGGTATGAATGATAAAAGCTCCCGATCGCATTCAGTTTTTACCCTGGTGATGACC
CAGACCAAGATAGAATCTGTGGAAGGTGAAGAACATGATCACAGAATAACAAGTCGGATA
AACTTGATAGATCTGGCGGGCAGTGAACGCTGCTCCACAGCTCAGACTAGTGGAGAGCGC
CTGAAGGAAGGTGTGAGTATTAACAAGTCCCTGCTCACATTGGGAAAGGTTATATCTGCG
CTTTCTGAACAAGCAAACCGGAAGAGAGGTTTTGTTCCTTACCGTGAATCTGTTCTTACA
TGGCTGTTAAAAGAAAGTCTGGGTGGAAACTCAAAAACTGCAATGATTGCTACAGTCAGT
CCTGCTGCCAGCAACATTGAGGAAACATTAAGCACCCTTAGATATGCCAGCCAAGCCCGC
ATGATAATCAATATCGCCAAAGTAAATGAAGATATGAATGCTAAGTTAATTAGAGAATTG
AAAGCGGAAATTGAAAAGCTAAAAGCTGCTCAAAGAAACAGCCGAAATATTGACCCCGAA
CGATATAGGCTGTGCCGGCAAGAAATTACATCCTTAAGAATGAAACTGCATCAACAGGAA
AGAGACATGGCAGAGATGCACAGAGCCTGGAAAGAAAAATTTGAACAAGCCGAAAAAAGA
AAACTTCAAGAAACCAAGGAGTTACAGAAAGCAGGAATTACATTTCAAATGGACAACCAT
TTGCCGAACCTTGTCAATCTCAATGAAGATCCTCAGCTATCAGAGATGCTATTATATATG
ATAAAAGAAGGAACAACGACAGTTGGAAAGTATGGACCAAACTCAAGCCATGATATTCAG
TTATCTGGGGTGCTGATTGCTGATGATCACTGTATTATAAACAATTCTGATGGGATAGTG
AGTATCATCCCAGTTGGAGAGGCAAAGACATATATAAATGGAAAACATATTTTGGAATCC
ACAGTATTACATCATGGTGATCGGGTGATTCTTGGTGGAGATCATTATTTTAGATTTAAT
CATCCCGTAGAAGTCCAGAAAGGAAAAAGGCCATCTGGTAGAGATACTCTTATAAGTGAG
GGTCCAAAAGACTTTGAATTTGCAAAAAATGAGTTGCTCATGGCACAGAGATCACAACTT
GAAGCAGAAATAAAAGAGGCACAATTGAGAGCGAAGGAAGAAATGATACAAGGAATCCAA
ATTGCAAAAGAAATGGCTCGGCAAGAGCTTTCCTCTCAAAAAGCTGCATATGAAAACAAA
ATAAAAGCATTGGAAGCAGAACTGAAAGAAGAGTCTCAAAGGAAGAAAATGCAGGAAATA

AATAACCAAAAGGCTAATGACAAAATCGAGGAATTAGAAAAGGCAAAACAGCGTCTTGAA
CAAGAAATATATGTCAACAAAAAGCGATTAGAAATGGAGACTTTGGCTGCAAAACAGGCT
TTAGAAGACCACAGCATCCGCCATGCAAGAATTCTGGAAGCTTTAGAAACTGAGAAGCAA
AAAATTGCTAAAGAAGTACAAATCCTACAGCAAAATCAGAGTAATAGGGATAAAACTTTT
TTAATTCAGCCAAATTGGAGCTCTATGAAACTCTCAATGATGATTCAGGAAGCGAATGCC
ATCAGCAACAAATTTAAAAAATATTATGTTTTTGGCAGACATGATGTATCAGATAAAGGA
AGTAGTTTGGACACTTCTGTGCGGGTTCGTAACTTACAACTAGGGATCTCAACTTTCTGG
AGTCTGGAAAAATTTGAATCTAAGCTGGCAGCAATGAAAGAACTTTATGAGAGTAATAGT
AATAACAAAAGTGAAGATATCTTTTGTGATCCTGAAGATGAATGGGAACCTGACATTACA
AATGCACCAGTTTCTTCCCTTTCTAGAAGGAGGAGCAAAAGTTTGATGAAGAACAGGAGA
ATTTCTGGTTGTTTACATGATATACAAGCCTACCCAATTCAGAATTTGCATTCTTCCCAT
TCATCAGATATAGGCTTACTGGAGAAATCGAGCACCATTTCCCCAAGTTCAGCAGAATCT
TTTCTTCCTGGAATTTGCAAAGAATTGATTGGGTCATCGTTAGATTTTCTTGGACAGAGT
TATGATGAAGAAAAAAGCATGGCAGACAGCCTGATTAATAATTTTCTTAAAATTTATCAT
GGACTATTTGCCATTTCCAAAGCTCATGAAGAACAAGATGAAGAAAGTCAAGATAACTTG
TTCTCTTCTGATCGAGCAACCCAGTCACTTACTATCCAGGTCACATGTGCTTTTGAGCAG
CTTGTGGTTCTAATCAAACACTGGCTGGATGATATTCTACCTTGTACCAGCACAGCAAGA
CTTGAAGATGAACTGAGACAAGAGGTTAAAAAACTAGGAGGCTACTTACAGTTATTTTTG
CAGGGATGCTGTTCAGATATCTCATCAATGGTAAAAGAAGCTCAAAACAAAATAATCCAA
ATTGTACAACAAGCTGTGAAGTATGTGGGACAGTTAGCAGTTCTTAAAGGGAGCGAGCTA
CATTTTCTGGAAAACAGTAGCAATAAGGCTACCGGTGTTCAGGAGGATTTCATGGATGCT
CTTTGTGATGGTGTAGGCTTAGGAGTGAAGATTCTATTAGATTCTGGACTAGAAAAAGCA
AAAGAACTTCAGCATGGACTCTTAAGGCAGTGTACCCAAAATGAGGTTACCAAACAGATG
AAAGCTAATGCCATGGGATTGATTGGATCTCTTGAAAACATCTTTGCTGAGTGGAAAACA
AAAAGTTTCAGAAGCAAACTACAACAAGAAAATTCTGGATACCAAGATTTGAAGAAGATG
GTTAATCTTGCTCCAAAATTCTTGAAGTTAAAACATTACTTAGAGCAAACTATTCAAATT
ATTATTTCTGCACTGAGAGGATGCCACAGTGACAAAAATCTTCTCAAGAATTGTGTTGAA
AGTCTGTGCAACTTGACCAAGGATTTTCATGATGACTTCAGCATTCACTCTGCTTCTCTT
GACAGCTGTGAGAAGAGAATGCCTCAGGGCAGCCACAGGGGGCTAGAGGCTCTAGTCAAG
TCACTCCTCTTCTGTTTTGAATCTCAAGAAAGTCCTAATTTGTTGAAACCCTGGGAAACT
TGTAATCAGAATACCGGAGAAGAAGGACAACAACCTAAATCAAGCAGGACTGACTCCGTG
AGGAATAAAGGTGTACCAAAGCGTGTCTATGAACTCCATGGCTCATACCCAGCAGTGGCG
TCAGAGGAAAGCACACCCAGTAGGATTCAGTGGGTA
>Odobenus rosmarus divergens XP_004392783.1
ATGTCAGTATACACCGCACATAATAGAAATAACAGGGATATCCTTGGTATACCTTCTTCCCAAAAGAGTT
CATCACTGAATAGCCTCACCCATAGTAGCAGACGTAAGCTGCATTTGAATTCGGGTATGTCAGAATGTGA
AAATGATGATCCATTATTGAGATCTGCAAGTAAAATCAGAGACATAAATAGTACTTATGTGATTTCTGCC
TGTAAAAAAACAGGAGAGGTACCTCTTACCCCTGACCCTGCAGGTAGATTGGCACTTCAGAGAAGAGTTA
CAAGGAACAAAGAATCATCTTTGCGTGGCAGTGAGTTGGGAGACTCAACTGAAAAAACTGCAGAAACACG
TCTTACATTACAACGTCGTGCTAAAACAGAATCTGTGGAAAAGTGGAAAACAGCTCAAACAGACTCTTGG
AAAACAACACAGAATGTGGGAAACCCAACAGAAAATGATTGTGCTTCCCTGGAAACGAGGAGAAAGGTAA
CAATTGTAAATAATGGTAAAGACCCTTCTGTTGCATCTTTTGTACCTTTAGCCAAAGACGGAAAAGACAT
TGAAATGATGGCTGATGAAAAACACAAAGAAACATTTTCTGCCCTCCTTGGGGCAAATGAACATGTCGCA
CTTCAATACTTAAGTAATAGAGCACCCACTGATTCCCAGAGTCAGACTAAAGTTGTTCCATCCGGACACT
TGGCAACAAAGCCTCTTCAGAGCAAGTTGAACATCAAAGTGCCAGGAACAGGAAACTTGTATCATAGAAG
TATTGGGAAGGATATGGCAAAAAATTCAAATAAATTTGAAAGCTTAGAAAAAAGAACACCTACAAAGTAC
ATTGTAGGACACAAATCGACACCGAAGTGTGGCACGCCACAGCTTACAAGCCCAGCTGCATCGATACTGA

AAAATAGGGTGCCTAGCCTCCAAGTTAAACAAAAGCCAAGAAGTTCTCTTCTAGCAAATGAAAAGGAAAG
GTCACAAGAAAATACACTCCTTCTTGAAGAGGAAACAACAGGTCGGAACACCTCTATAGAAACAGACCCC
TTAAAAGTAGAGAACAGTCAAGTGACAGTGGCAGTACGTGTGAGGCCTTTCAGCAAGAGAGAGAAGGTTG
AAAAAGCATCCCAGGTGGTCTTCATGAATGGGGAAGAAATAGCTGTGGAACATCCGGACATGAAACAAGT
TTATAATTTTATTTATGATGTTGCATTCTGGTCTTTTGATGAATGCCATCCCAACTATGCTAGCCAGACG
ACTGTTTATGAGACACTAGCAGTACCCCTCCTCGAGCGTGCCTTTGAAGGCTACAATACCTGTCTCTTTG
CTTATGGTCAGACCGGCTCTGGAAAATCATATACGATGATGGGACTTAGTGAAGAACCAGGAATAATTCC
AAGGTTTTGTGAAGATCTTTTTGCTCAAGTAGCCAAAAAACAAACTCAGGAGGTCAGCTATCACCTTGAA
ATGAGCTTTTTTGAAGTATATAATGAAAAAATTCATGACCTTCTGGTTTGTAAAGGCGAAAACGGACAGA
GAAAGCAACCACTGAGAGTAAGGGAGCATCCTGTCTCTGGACCATATGTTGAAGCTTTGTCAATGAATGT
TGTCAGTTCTTACTCTGATATCCAGAGTTGGCTAGAATTGGGAAATAAACAAAGAGCGACTGCTGCTACT
GGTATGAATGATAAAAGCTCCCGATCGCATTCGGTTTTCACCCTGGTGATGACCCAGACCAAGACGGAAT
CTGTGGAAGGGGAGGAACATGATCACAGAATAACGAGTCGGATAAACCTGATAGATCTGGCAGGCAGTGA
GCGCTGCTCCACAGCTCAAACTAATGGAGATCGACTGAAGGAAGGTGTGAGTATTAACAAGTCCTTGCTC
ACATTGGGAAAGGTTATATCTGCACTTTCTGAACAAGCAAACCGAAAGAGAGTTTTTATTCCTTACCGTG
AATCTGTTCTTACATGGCTGTTAAAAGAAAGTCTGGGTGGAAATTCCAAAACTGCAATGATCGCCACCAT
CAGTCCTGCTGCCAACAACGTTGAAGAAACATTAAGCACGCTGAGATATGCTAGCCAAGCCCGTATGATA
GTCAATATCGCCAAAGTAAATGAAGATATGAATGCTAAGTTAATTAGAGAATTGAAAGCAGAAATCGAAA
AGCTAAAAGCTGCTCAAAGAAACAGTCGAAATATTGACCCTGACAGATATAGGCTGTGCCGGCAAGAAAT
AACATCCTTAAGAATGAAACTGCATCAACAGGAGAGAGACATGGCAGAAATGCAAAGAGCATGGAAAGAA
AAATTTGAACAAGCTGAAAGAAGAAAACTTCAAGAAACAAAGGAGTTACAGAAAGCAGGAATTACATTTC
AAATCGACAACCATTTGCCAAACCTTGTCAATCTCAATGAAGATCCTCAGCTATCAGAGATGCTATTATA
TATGATAAAAGAAGGAACGACGACAGTTGGAAAGTATAAACCAAACTCAAGCCATGATATTCAGTTATCT
GGGGTGCTGATTGCTGATGATCATTGTACTATAAAAAATTTTGATGGAATAGTGAGTATCATCCCAGTTG
GAGAAGCAAAGACATATATAAATGGAAAACATATTTTGGAATCCACAGTATTACATCATGGTGATCGGGT
GATTCTTGGTGGAGATCATTATTTTAGATTTAATCATCCAGTAGAAGTCCAGAAAGGAAAAAGACCATCC
GGTAGAGATACTCTTACAAGCGAGGGTCCAAAAGACTTTGAATTTGCAAAAAATGAGTTGCTCATGGCAC
AAAGATCACAACTTGAAGCAGAAATAAAAGAGGCACAATTGAGAGCAAAGGAAGAAATGATGCAAGGAAT
CCAAATTGCAAAAGAAATGGCTCAGCAAGAGCTTTCTTCTCAAAAAGCTGCCTATGAAAGCAAAATAAAA
GCATTGGAAGCAGAACTGAAGGAAGAGGCTCAAAGGAAGAAAATCCAGGAAATAAATAACCAAAAGGCTA
ATGACAAAATTGAGGAATTAGAAAAGGCAAAACAGCGACTTGAACAGGAAATCTATGTCAACAAAAAGCG
ATTAGAAATGGAGACTTTGGCTGCAAAACAGGCTTTAGAAGACCACAGCATCCGCCATGCAAGAATTCTG
GAAGCTTTAGAAACTGAGAAGCAAAAAATTGCTAAAGAAGTACAAATACTACAGCAAAATCAAAGTAATA
GAGATAAAACTTTTTTAATTCAGACAAATTGGAGCTCCATGAAACTCTCAATGATGATTCAGGAAGCCAA
TGCCATCAGCAGCAAATTTAAAAAATGTTATGTTTTTGGCAGACATGATATATCAGATAAAGGAAGTAGT
TCTGATACTTCTGTTCGGGTTCGTAACCTACAACTAGGGATCTCAACTTTCTGGAGCCTGGAAAAGTTTG
AATCTAAGCTTGCAGCAATGAAAGAACTTTATGAGAGTAATAATAGTAACAAGAGTGAAGATGTCTTTTG
TGATCCTGAAGATGAATGGGAACCTGACATTACAAATGCACCAGTGTCTTCTTTTTCTAGAAGGAGGAGC
AGGAGCTTGATGAAGAATAGAAGAATTTCTGGTTGTTTACATGATATACAAGCCCACCCAATTCAGAATT
TGAATTCTTCCCATTCATCAGGCTTACTGGAGAAATCGAGCACTATTTACTCAAGTTCAGCAGAATCTTT
TCTTCCTGGAATTTGCAAAGAATTGATTGGGTCATCCTTAGATTTTCTTGGACAGAGTTATGATGAAGAA
AAAACCATGGCAGACAGCCTGATTAATAATTTTCTTAAAATTTATCACGGGCTATTTGCCATTTCCAAAG
CTCATGAAGAACAAGATGAAGAAAGTCAAGATAACTTGTTCTCTTCTGATCGAGCAATCCAGGCACTTAC
TATCCAGGTCACATGTGCTTTTGAGCAGCTTGTGGTGCTGATCAAACATTGGCTAAATGATACTCTGCCT
TGTACCAGCACAGCAAGACTTGAAGATGAATTGAGACAAGAGGTTAAAAAACTAGGAGGCTACTTACAAT
TATTTTTGCAGGGATGTTGTTCAGATATCTCATCAATGGTAAAGGAGGCTCAAAAGAAAATAATCCAAAT
TGTACAACAAGCTGTAAAGTATGTGGGACAGTTAGCAGTTCTTAAAGGGAGCAAGCTGCATTTTCTGGAA

AATAGTAGCAACAAGGCTGCCAGTGTTCAGGAGGATTTCGTGGATGCTCTTTGTGATGGTATAGGCTTAG
GAATGAAGATCCTATTAGATTCTGGGATAGAAAAAGCAAAAGAACTTCAACATGAACTCTTAAGGCAATG
TACCCAAAACGAGGTTGCCAAACAGATGAAAGCTAAAGCCATGGGATTGATTGGATGTCTTGAAAACATG
TTTGCTGAGTGGAAAACAAAAAGTTTCAGAACTGAACTACAACAAGAAAATTCTGGATACCAAGATTTGA
AGAAGATGGTTAATCTTGCACCAGAATTCCTGAAGTTAAAACATTGCTTGGAGCAAACTATTCAAATTAT
TATTTCTGCACTGAGAGGATTCCACAGTGACAAAAATCTTCTCAAGAATTGTGTTGAAAGTCTTTGCAGC
TTGGCCAAGGATCTTCATGATGACTTGAGCATGCGCTCTACTTCTCTTGACAGCTGTGAGAAGAAAATAC
CTCAAGTTGGCCATGGGGGACTAGAATCTCTAGTCAAGTCACTCCTCTTATGTTTTGAATCTGAAGAAAG
ACCTGATTTGTTGAATCCCTGGGAAACGTGTAACCAAAATACCAGAGAAGAAGGACAACAATCTAAATCA
AGCAGGACCGACTCTGTTAGGAACAAAGGTGTACCAAAGCAAGTGTATGAACTCCATGGCTTGTCCCCAG
CAGTGACTTCAGAGGACAGCGCACCCATTAGGATTCAGTGGGTT

Neandertals Protein sequence

> CEP135

MTTAVERKYINIRKRLDQLGYRQTLTVECLPLVEKLFSDLVHTTESLRQSKLSAVKAEKE
SANFDFVLEPYKLENARLSRENNELYLELMKLREHSDQHVKELKTSLKKCARETADLKFL
NNQYAHKLKLLEKESKAKNERIQQLQEKNLHAVVQTPGGKKRSIAFRRQRMQIDEPVPPS
EVSSYPVPQPDDPYIADLLQVADNRIQELQQEVHQLQEKLAMMESGVRDYSKQIELRERE
IERLSVALDGGRSPDVLSLESRNKTNEKLIAHLNIQVDFLQQANKDLEKRIRELMETKET
VTSEVVNLSNKNEKLCQELTEIDQLAQQLERHKEEVLETADKELGEAKKEIKRKLSEMQD
LEETMAKLQLELNLCQKEKERLSDELLVKSDLETVVHQLEQEKQRLSKKVESFAVTERQL
TLEVERMRLEHGIKRRDRSPSRLDTFLKGIEEERDYYKKELERLQHIIQRRSCSTSYSAR
EKSSIFRTPEKGDYNSEIHQITRERDELQRMLERFEKYMEDIQSNVKLLTAERDKLSVLY
NEAQEELSALRKESTQTTAPHNIVSLMEKEKELALSDLRRIMAEKEALREKLEHIEEVSL
FGKSELEKTIEHLTCVNHQLESEKYELKSKVLIMKETIESLENKLKVQAQKFSHVAGDSS
HQKTEVNSLRIVNEQLQRSVDDYQHRLSIKRGELESAQAQIKILEEKIDELNLKMTSQDE
EAHVMKKTIGVIDKEKDFLQETVDEKTEKIANLQENLANKEKAVAQMKIMISECESSVNQ
LKETLVNRDREINSLRRQLDAAHKELDEVGRSREIAFKENRRLQDDLATMARENQEISLE
LEAAVQEKEEMKSRVHKYITEVSRWESLMAAKEKENQDLLDRFQMLHNRAEDWEVKAHQA
EGESSSVRLELLSIDTERRHLRERVELLEKEIQEHINAHHAYESQISSMAKAMSRLEEEL
RHQEDEKATVLNDLSSLRELCIKLDSGKDIMTQQLNSKNLEFERVVVELENVKSESDLLK
KQLSNERHTVKNLESLLATNRDKEFHSHLTSHEKDTEIQLLKEKLTLSESKLTSQSRENT
MLRAKVAQLQTDYDALKRQISTERYERERAIQEMRRHGLATPPLSSTLRSPSHSPEHRNV

>ZNF335

MEENEVESSSDAAPGPGRPEEPSESGLGVGTSEAVSADSSDAAAAPGQAEADDSGVGQSS
DRGSRSQEEVSESSSSADPLPNSYLPDSSSVSHGPVAGVTGGPPALVHSSALPDPNMLVS
DCTASSSDLGSAIDKIIESTIGPDLIQNCITVTSAEDGGAETTRYLILQGPDDGAPMTSP
MSSSTLAHSLAAIEALADGPTSTSTCLEAQGGPSSPVQLPPASGAEEPDLQSLEAMMEVV
VVQQFKCKMCQYRSSTKATLLRHMRERHFRPVAAAAAAAGKKGRLRKWSTSTKTQEEEGP
EEEDDDDIVDAGAIDDLEEDSDYNPAEDEPRGRQLRLQRPTPSTPRPRRRPGRPRKLPRL
EISDLPDGVEGEPLVSSQSGQSPPEPQDPEAPSSSGPGHLVAMGKVSRTPVEAGVSQSDA
ENAAPSCPDEHDTLPRRRGRPSRRFLGKKYRKYYYKSPKPLLRPFLCRICGSRFLSHEDL

RFHVNSHEAGDPQLFKCLQCSYRSRRWSSLKEHMFNHVGSKPYKCDECSYTSVYRKDVIR
HAAVHSRDRKKRPDPTPKLSSFPCPVCGRVYPMQKRLTQHMKTHSTEKPHMCDKCGKSFK
KRYTFKMHLLTHIQAVANRRFKCEFCEFVCEDKKALLNHQLSHVSDKPFKCSFCPYRTFR
EDFLLSHVAVKHTGAKPFACEYCHFSTRHKKNLRLHVRCRHASSFEEWGRRHPEEPPSRR
RPFFSLQQIEELKQQHSAAPGPPPSSPGPPEIPPEATTFQSSEAPSLLCSDTLGGATIIY
QQGAEESTAMATQTALDLLLNMSAQRELGGTALQVAVVKSEDVEAGLASPGGQPSPEGAT
PQVVTLHVAEPGGGAAAESQLGPPDLPQITLAPGPFGGTGYSVITAPPMEEGTSAPGTPY
SEEPAGEAAQAVVVSDTLKEAGTHYIMATDGTQLHHIELTADGSISFPSPDALASGAKWP
LLQCGGLPRDGPEPPSPAKTHCVGDSQSSASSPPATSKALGLAVPPSPPSAATAASKKFS
CKICAEAFPGRAEMESHKRAHAGPGAFKCPDCPFSARQWPEVRAHMAQHSSLRPHQCSQC
SFASKNKKDLRRHMLTHTKEKPFACHLCGQRFNRNGHLKFHIQRLHSPDGRKSGTPTARA
PTQTPTQTIILNSDDETLATLHTALQSSHGVLGPERLQQALSQEHIIVAQEQTVTNQEEA
AYIQEITTADGQTVQHLVTSDNQVQYIISQDGVQHLLPQEYVVVPEGHHIQVQEGQITHI
QYEQGAPFLQESQIQYVPVSPGQQLVTQAQLEAAAHSAVTAVADAAMAQAQGLFGTDETV
PEHIQQLQHQGIEYDVITLADD

>PHC1

METESEQNSNSTNGSSSSGGSSRPQIAQMSLYERQAVQALQALQRQPNAAQYFHQFMLQQ
QLSNAQLHSLAAVQQATIAASRQASSPNTSTTQQQTTTTQASINLATTSAAQLISRSQSV
SSPSATTLTQSVLLGNTTSPPLNQSQAQMYLRPQLGNLLQVNRTLGRNVPLASQLILMPN
GAVAAVQQEVPSAQSPGVHADADQVQNLAVRNQQASAQGPQMQGSTQKAIPPGASPVSSL
SQASSQALAVAQASSGATNQSLNLSQAGGGSGNSIPGSMGPGGGGQAHGGLGQLPSSGMG
GGSCXXXXTGVVQPLPAAQTVTVSQGSQTEAESAAAKKAEADGSGQQNVGMNLTRTATPA
PSQTLISSATYTQIQPHSLIQQQQQIHLQQKQVVIQQQIAIHHQQQFQHRQSQLLHTATH
LQLAQQQQQQQQQQQQQQPQATTLTAPQPPQVPPTQQVPPSQSQQQAQTLVVQPMLQSS
PLSLPPDAAPKPPIPIQSKPPVAPIKPPQLGAAKMSAAQQPPPHIPVQVVGTRQPGTAQA
QALGLAQLAAAVPTSRGMPGTVQSGQAHLASSPPSSQAPGALQECPPTLAPGMTLAPVQG
TAHVVKGGATTSSPVVAQVPAAFYMQSVHLPGKPQTLAVKRKADSEEERDDVSTLGSMLP
AKASPVAESPKVMDEKSSLGEKAESVANVNANTPSSELVALTPAPSVPPPTLAMVSRQMG
DSKPPQAIVKPQILTHIIEGFVIQEGAEPFPVGCSQLLKESEKPLQTGLPTGLTENQSGG
PLGVDSPSAELDKKANLLKCEYCGKYAPAEQFRGSKRFCSMTCAKRYNVSCSHQFRLKRK
KMKEFQEANYARVRRRGPRRSSSDIARAKIQGKCHRGQEDSSRGSDNSSYDEALSPTSPG
PLSVRAGHGERDLGNPNTAPPTPELHGINPVFLSSNPSRWSVEEVYEFIASLQGCQEIAE
EFRSQEIDGQALLLLKEEHLMSAMNIKLGPALKICAKINVLKET

>CDK6

MEKDGLCRADQQYECVAEIGEGAYGKVFKARDLKNGGRFVALKRVRVQTGEEGMPLSTIR
EVAVLRHLETFEHPNVVRLFDVCTVSRTDRETKLTLVFEHVDQDLTTYLDKVPEPGVPTE
TIKDMMFQLLRGLDFLHSHRVVHRDLKPQNILVTSSGQIKLADFGLARIYSFQMALTSVV
VTLWYRAPEVLLQSSYATPVDLWSVGCIFAEMFRRKPLFRGSSDVDQLGKILDVIGLPGE
EDWPRDVALPRQAFHSKSAQPIEKFVTDIDELGKDLLLKCLTFNPAKRISAYSALSHPYFQ
DLERCKENLDSHLPPSQNTSELNTA

>SASS6

MSQVLFHQLVPLQVKCKDCEERRVSIRMSIELQSVSNPVHRKDLVIRLTDDTDPFFLYNL
VISEEDFQSLKFQQGLLVDFLAFPQKFIDLLQQCTQEHAKEIPRFLLQLVSPAAILDNSP
AFLNVVETNPFKHLTHLSLKLLPGNDVEIKKFLAGCLKCSKEEKLSLMQSLDDATKQLDF
TRKTLAEKKQELDKLRNEWASHTAALTNKHSQELTNEKEKALQAQVQYQQQHEQQKKDLE
ILHQQNIHQLQNRLSELEAANKDLTERKYKGDSTIRELKAKLSGVEEELQRTKQEVLSLR
RENSTLDVECHEKEKHVNQLQTKVAVLEQEIKDKDQLVLRTKEAFDTIQEQKVVLEENGE
KNQVQLGKLEATIKSLSAELLKANEIIKKLQGDLKTLMGKLKLKNTVTIQQEKLLAEKEE
KLQKEQKELQDVGQSLRIKEQEVCKLQEQLEATVKKLEESKQLLKNNEKLITWLNKELNE
NQLVRKQDVLGPSTTPPAHSSSNTIRSGISPNLNVVDGRLTYPTCGIGYPVSSAFAFQNT
FPHSISAKNTSHPGSGTKVQFNLQFTKPNASLGDVQSGATISMPCSTDKENGENVGLESK
YLKKREDSIPLRGLSQNLFSNSDHQRDGTLGALHTSSKPTALPSASSAYFPGQLPNS

>MFSD2A

MAKGEGAESGSAAGLLPTSILQSTERPAQVKKEPKKKKQQLSVCNKLCYALGGAPYQVTG
CALGFFLQIYLLDVAQVGPFSASIILFVGRAWDAITDPLVGLCISKSPWTCLGRLMPWII
FSTPLAVIAYFLIWFVPDFPHGQTYWYLLFYCLFETMVTCFHVPYSALTMFISTEQTERD
SATAYRMTVEVLGTVLGTAIQGQIVGQADTPCFQDLNSSTVASQSANHTHGTTSHRETQK
AYLLAAGVIVCIYIICAVILILGVREQREPYEAQQSEPIAYFRGLRLVMSHGPYIKLITG
FLFTSLAFMLVEGNFVLFCTYTLGFRNEFQNLLLAIMLSATLTIPIWQWFLTRFGKKTAV
YVGISSAVPFLILVALMESNLIITYAVAVAAGISVAAAFLLPWSMLPDVIDDFHLKQPHF
HGTEPIFFSFYVFFTKFASGVSLGISTLSLDFAGYQTRGCSQPERVKFTLNMLVTMAPIV
LILLGLLLFKMYPIDEERRRQNKKALQALRDEASSSGCSETDSTELASIL

>CIT

MLKFKYGARNPLDAGAAEPIASRASRLNLFFQGKPPFMTQQQMSPLSREGILDALFVLFE
ECSQPALMKIKHVSNFVWKYSDTIAELQELQPSAKDFEVRSLVGCGHFAEVQVVREKATG
DIYAMKVMKKKALLAQEQVSFFEEERNILSRSTSPWIPQLQYAFQDKNHLYLVMEYQPGG
DLLSLLNRYEDQLDENLIQFYLAELILAVHSVHLMGYVHRDIKPENILVDRTGHIKLVDF
GSAAKMNSNKMVNAKLPIGTPDYMAPEVLTVMNGDGKGTYGLDCDWWSVGVIAYEMIYGR
SPFAEGTSARTFNNIMNFQRFLKFPDDPKVSSDFLDLIQSLLCGQKERLKFEGLCCHPFF
SKIDWNNIRNSPPPFVPTLKSDDDTSNFDEPEKNSWVSSSPCQLSPSGFSGEELPFVGFS
YSKALGILGRSESVVSGLDSPAKTSSMEKKLLIKSKELQDSQDKCHKMEQEMTRLHRRVS
EVEAVLSQKEVELKASETQRSLLEQDLATYITECSSLKRSLEQARMEVSQEDDKALQLLH
DIREQSRKLQEIKEQEYQAQVEEMRLMMNQLEEDLVSARRRSDLYESELRESRLAAEEFK
RKATECQHKLLKAKDQGKPEVGEYAKLEKINAEQQLKIQELQEKLEKAVKASTEATELLQ
NIRQAKERAERELEKLQNREDSSEGIRKKLVEAEERRHSLENKVKRLETMERRENRLKDD
IQTKSQQIQQMADKILELEEKHREAQVSAQHLEVHLKQKEQHYEEKIKVLDNQIKKDLAD
KETLENMMQRHEEEAHEKGKILSEQKAMINAMDSKIRSLEQRIVELSEANKLAANSSLFT
QRNMKAQEEMISELRQQKFYLETQAGKLEAQNRKLEEQLEKISHQDHSDKNRLLELETRL
REVSLEHEEQKLELKRQLTELQLSLQERESQLTALQAARAALESQLRQAKTELEETTAEA
EEEIQALTAHRDEIQRKFDALRNSCTVITDLEEQLNQLTEDNAELNNQNFYLSKQLDEAS
GANDEIVQLRSEVDHLRREITEREMQLTSQKQTMEALKTTCTMLEEQVMDLEALNDELLE
KERQWEAWRSVLGDEKSQFECRVRELQRMLDTEKQSRARADQRITESRQVVELAVKEHKA

EILALQQALKEQKLKAESLSDKLNDLEKKHAMLEMNARSLQQKLETERELKQRLLEEQAK
LQQQMDLQKNHIFRLTQGLQEALDRADLLKTERSDLEYQLENIQVLYSHEKVKMEGTISQ
QTKLIDFLQAKMDQPAKKKKGLFSRRKEDPALPTQVPLQYNELKLALEKEKARCAELEEA
LQKTRIELRSAREEAAHRKATDHPHPSTPATARQQIAMSAIVRSPEHQPSAMSLLAPPSS
RRKESSTPEEFSRRLKERMHHNIPHRFNVGLNMRATKCAVCLDTVHFGRQASKCLECQVM
CHPKCSTCLPATCGLPAEYATHFTEAFCRDKMNSPGLQTKEPSSSLHLEGWMKVPRNNKR
GQQGWDRKYIVLEGSKVLIYDNEAREAGQRPVEEFELCLPDGDVSIHGAVGASELANTAK
ADVPYILKMESHPHTTCWPGRTLYLLAPSFPDKQRWVTALESVVAGGRVSREKAEADAKL
LGNSLLKLEGDDRLDMNCTLPFSDQVVLVGTEEGLYALNVLKNSLTHVPGIGAVFQIYII
KDLEKLLMIAGEERALCLVDVKKVKQSLAQSHLPAQPDISPNIFEAVKGCHLFGAGKIEN
GLCICAAMPSKVVILRYNENLSKYCIRKEIETSEPCSCIHFTNYSILIGTNKFYEIDMKQ
YTLEEFLDKNDHSLAPAVFAASSNSFPVSIVQVNSAGQREEYLLCFHEFGVFVDSYGRRS
RTDDLKWSRLPLAFAYREPYLFVTHFNSLEVIEIQARSSAGTPARAYLDIPNPRYLGPAI
SSGAIYLASSYQDKLRVICCKGNLVKESGTEHHRGPSTSRSSPNKRGPPTYNEHITKRVA
SSPAPPEGPSHPREPSTPHRYREGRTELRRDKSPGRPLEREKSPGRMLSTRRERSPGRLF
EDSSRGRLPAGAVRTPLSQVNKVWDQSSV

>KIF14

MSLHSTHNRNNSGDILDIPSSQNSSSLNALTHSSRLKLHLKSDMSECENDDPLLRSAGKV
RDINRTYVISASRKTADMPLTPNPVGRLALQRRTTRNKESSLLVSELEDTTEKTAETRLT
LQRRAKTDSAEKWKTAEIDSVKMTLNVGGETENNGVSKESRTNVRIVNNAKNSFVASSVP
LDEDPQVIEMMADKKYKETFSAPSRANENVALKYSSNRPPIASLSQTEVVRSGHLTTKPT
QSKLDIKVLGTGNLYHRSIGKEIAKTSNKFGSLEKRTPTKCTTEHKLTTKCSLPQLKSPA
PSILKNRMSNLQVKQRPKSSFLANKQERSAENTILPEEETVVQNTSAGKDPLKVENSQVT
VAVRVRPFTKREKIEKASQVVFMSGKEITVEHPDTKQVYNFIYDVSFWSFDECHPHYASQ
TTVYEKLAAPLLERAFEGFNTCLFAYGQTGSGKSYTMMGFSEEPGIIPRFCEDLFSQVAR
KQTQEVSYHIEMSFFEVYNEKIHDLLVCKDENGQRKQPLRVREHPVYGPYVEALSMNIVS
SYADIQSWLELGNKQRATAATGMNDKSSRSHSVFTLVMTQTKTEFVEGEEHDHRITSRIN
LIDLAGSERCSTAHTNGDRLKEGVSINKSLLTLGKVISALSEQANQRSVFIPYRESVLTW
LLKESLGGNSKTAMIATISPAASNIEETLSTLRYANQARLIVNIAKVNEDMNAKLIRELK
AEIAKLKAAQRNSRNIDPERYRLCRQEITSLRMKLHQQERDMAEMQRVWKEKFEQAEKRK
LQETKELQKAGIMFQMDNHLPNLVNLNEDPQLSEMLLYMIKEGTTTVGKYKPNSSHDIQL
SGVLIADDHCTIKNFGGTVSIIPVGEAKTYVNGKHILEITVLRHGDRVILGGDHYFRFNH
PVEVQKGKRPSGRDTPISEGPKDFEFAKNELLMAQRSQLEAEIKEAQLKAKEEMMQGIQI
AKEMAQQELSSQKAAYESKIKALEAELREESQRKKMQEINNQKANHKIEELEKAKQHLEQ
EIYVNKKRLEMETLATKQALEDHSIRHARILEALETEKQKIAKEVQILQQNRNNRDKTFT
VQTTWSSMKLSMMIQEANAISSKLKTYYVFGRHDISDKSSSDTSIRVRNLKLGISTFWSL
EKFESKLAAMKELYESNGSNRGEDAFCDPEDEWEPDITDAPVSSLSRRRSRSLMKNRRIS
GCLHDIQVHPIKNLHSSHSSGLMDKSSTIYSNSAESFLPGICKELIGSSLDFFGQSYDEE
RTIADSLINSFLKIYNGLFAISKAHEEQDEESQDNLFSSDRAIQSLTIQTACAFGQLVVL
MKHWLSDLLPCTNIARLEDELRQEVKKLGGYLQLFLQGCCLDISSMIKEAQKNAIQIVQQ
AVKYVGQLAVLKGSKLHFLENGHNKAASVQEEFMDAVCDGVGLGMKILLDSGLEKAKELQ

HELFRQCTKNEVTKEMKTNAMGLIRSLENIFAESKIKSFRRQVQEENFEYQDFKRMVNRA
PEFLKLKHCLEKAIEIIISALKGCHSDINLLQTCVESIRNLASDFYSDFSVPSTSVGSYE
SRVTHIVHQELESLAKSLLFCFESEESPDLLKPWETYNQNTKEEHQQSKSSGIDGSKNKG
VPKHVYELHGSSPAVSSEECTPSRIQWV

Denisovans Protein sequence

>CEP135

MTTAVERKYINIRKRLDQLGYRQTLTVECLPLVEKLFSDLVHTTESLRQSKLSAVKAEKE
SANFDFVLEPYKLENARLSRENNELYLELMKLREHSDQHVKELKTSLKKCARETADLKFL
NNQYAHKLKLLEKESKAKNERIQQLQEKNLHAVVQTPGGKKRSIAFRRQRMQIDEPVPPS
EVSSYPVPQPDDPYIADLLQVADNRIQELQQEVHQLQEKLAMMESGVRDYSKQIELRERE
IERLSVALDGGRSPDVLSLESRNKTNEKLIAHLNIQVDFLQQANKDLEKRIRELMETKET
VTSEVVNLSNKNEKLCQELTEIDQLAQQLERHKEEVLETADKELGEAKKEIKRKLSEMQD
LEETMAKLQLELNLCQKEKERLSDELLVKSDLETVVHQLEQEKQRLSKKVESFAVTERQL
TLEVERMRLEHGIKRRDRSPSRLDTFLKGIEEERDYYKKELERLQHIIQRRSCSTSYSAR
EKSSIFRTPEKGDYNSEIHQITRERDELQRMLERFEKYMEDIQSNVKLLTAERDKLSVLY
NEAQEELSALRKESTQTTAPHNIVSLMEKEKELALSDLRRIMAEKEALREKLEHIEEVSL
FGKSELEKTIEHLTCVNHQLESEKYELKSKVLIMKETIESLENKLKVQAQKFSHVAGDSS
HQKTEVNSLRIVNEQLQRSVDDYQHRLSIKRGELESAQAQIKILEEKIDELNLKMTSQDE
EAHVMKKTIGVIDKEKDFLQETVDEKTEKIANLQENLANKEKAVAQMKIMISECESSVNQ
LKETLVNRDREINSLRRQLDAAHKELDEVGRSREIAFKENRRLQDDLATMARENQEISLE
LEAAVQEKEEMKSRVHKYITEVSRWESLMAAKEKENQDLLDRFQMLHNRAEDWEVKAHQA
EGESSSVRLELLSIDTERRHLRERVELLEKEIQEHINAHHAYESQISSMAKAMSRLEEEL
RHQEDEKATVLNDLSSLRELCIKLDSGKDIMTQQLNSKNLEFERVVVELENVKSESDLLK
KQLSNERHTVKNLESLLATNRDKEFHSHLTSHEKDTEIQLLKEKLTLSESKLTSQSRENT
MLRAKVAQLQTDYDALKRQISTERYERERAIQEMRRHGLATPPLSSTLRSPSHSPEHRNV

>ZNF335

MEENEVESSSDAAPGPGRPEEPSESGLGVGTSEAVSADSSDAAAAPGQAEADDSGVGQSS
DRGSRSQEEVSESSSSADPLPNSYLPDSSSVSHGPVAGVTGGPPALVHSSALPDPNMLVS
DCTASSSDLGSAIDKIIESTIGPDLIQNCITVTSAEDGGAETTRYLILQGPDDGAPMTSP
MSSSTLAHSLAAIEALADGPTSTSTCLEAQGGPSSPVQLPPASGAEEPDLQSLEAMMEVV
VVQQFKCKMCQYRSSTKATLLRHMRERHFRPVAAAAAAAGKKGRLRKWSTSTKTQEEEGP
EEEDDDDIVDAGAIDDLEEDSDYNPAEDEPRGRQLRLQRPTPSTPRPRRRPGRPRKLPPL
EISDLPDGVEGEPLVSSQSGQSPREPQDPEAPSSSGPGHLVAMGKVSRTPVEAGVSQSDA
ENAAPSCPDEHDTLPRRRGRPSRRFLGKKYRKYYYKSPKPLLRPFLCRICGSRFLSHEDL
RFHVNSHEAGDPQLFKCLQCSYRSRRWSSLKEHMFNHVGSKPYKCDECSYTSVYRKDVIR
HAAVHSRDRKKRPDPTPKLSSFPCPVCGRVYPMQKRLTQHMKTHSTEKPHMCDKCGKSFK
KRYTFKMHLLTHIQAVANRRFKCEFCEFVCEDKKALLNHQLSHVSDKPFKCSFCPYRTFR
EDFLLSHVAVKHTGAKPFACEYCHFSTRHKKNLRLHVRCRHASSFEEWGRRHPEEPPSRR
RPFFSLQQIEELKQQHSAAPGPPPSSPGPPEIPPEATTFQSSEAPSLLCSDTLGGATIIY
QQGAEESTAMATQTALDLLLNMSAQRELGGTALQVAVVKSEDVEAGLASPGGQPSPEGAT
PQVVTLHVAEPGGGAAAESQLGPPDLPQITLAPGPFGGTGYSVITAPPMEEGTSAPGTPY

SEEPAGEAAQAVVVSDTLKEAGTHYIMATDGTQLHHIELTADGSISFPSPDALASGAKWP
LLQCGGLPRDGPEPPSPAKTHCVGDSQSSASSPPATSKALGLAVPPSPPSAATAASKKFS
CKICAEAFPGRAEMESHKRAHAGPGAFKCPDCPFSARQWPEVRAHMAQHSSLRPHQCSQC
SFASKNKKDLRRHMLTHTKEKPFACHLCGQRFNRNGHLKFHIQRLHSPDGRKSGTPTARA
PTQTPTQTIILNSDDETLATLHTALQSSHGVLGPERLQQALSQEHIIVAQEQTVTNQEEA
AYIQEITTADGQTVQHLVTSDNQVQYIISQDGVQHLLPQEYVVVPEGHHIQVQEGQITHI
QYEQGAPFLQESQIQYVPVSPGQQLVTQAQLEAAAHSAVTAVADAAMAQAQGLFGTDETV
PEHIQQLQHQGIEYDVITLADD

>PHC1

METESEQNSNSTNGSSSSGGSSRPQIAQMSLYERQAVQALQALQRQPNAAQYFHQFMLQQ
QLSNAQLHSLAAVQQATIAASRQASSPNTSTTQQQTTTTQASINLATTSAAQLISRSQSV
SSPSATTLTQSVLLGNTTSPPLNQSQAQMYLRPQLGNLLQVNRTLGRNVPLASQLILMPN
GAVAAVQQEVPSAQSPGVHADADQVQNLAVRNQQASAQGPQMQGSTQKAIPPGASPVSSL
SQASSQALAVAQASSGATNQSLNLSQAGGGSGNSIPGSMGPGGGGQAHGGLGQLPSSGMG
GGXXXXXGTGVVQPLPAAQTVTVSQGSQTEAESAAAKKAEADGSGQQNVGMNLTRTATPA
PSQTLISSATYTQIQPHSLIQQQQQIHLQQKQVVIQQQIAIHHQQQFQHRQSQLLHTATH
LQLAQQQQQQQQQQQQQQQPQATTLTAPQPPQVPPTQQVPPSQSQQQAQTLVIQPMLQSS
PLSLPPDAAPKPPIPIQSKPPVAPIKPPQLGAAKMSAAQQPPPHIPVQVVGTRQPGTAQA
QALGLAQLAAAVPTSRGMPGTVQSGQAHLASSPPSSQAPGALQECPPTLAPGMTLAPVQG
TAHVVKGGATTSSPVVAQVPAAFYMQSVHLPGKPQTLAVKRKADSEEERDDVSTLGSMLP
AKASPVAESPKVMDEKSSLGEKAESVANVNANAPSSELVALTPAPSVPPPTLAMVSRQMG
DSKPPQAIVKPQILTHIIEGFVIQEGAEPFPVGCSQLLKESEKPLQTGLPTGLTENQSGG
PLGVDSPSAELDKKANLLKCEYCGKYAPAEQFRGSKRFCSMTCAKRYNVSCSHQFRLKRK
KMKEFQEANYARVRRRGPRRSSSDIARAKIQGKCHRGQEDSSRGSDNSSYDEALSPTSPG
PLSVRAGHGERDLGNPNTAPPTPELHGINPVFLSSNPSRWSVEEVYEFIASLQGCQEIAE
EFRSQEIDGQALLLLKEEHLMSAMNIKLGPALKICAKINVLKET

>CDK6

MEKDGLCRADQQYECVAEIGEGAYGKVFKARDLKNGGRFVALKRVRVQTGEEGMPLSTIR
EVAVLRHLETFEHPNVVRLFDVCTVSRTDRETKLTLVFEHVDQDLTTYLDKVPEPGVPTE
TIKDMMFQLLRGLDFLHSHRVVHRDLKPQNILVTSSGQIKLADFGLARIYSFQMALTSVV
VTLWYRAPEVLLQSSYATPVDLWSVGCIFAEMFRRKPLFRGSSDVDQLGKILDVIGLPGE
EDWPRDVALPRQAFHSKSAQPIEKFVTDIDELGKDLLLKCLTFNPAKRISAYSALSHPYFQ
DLERCKENLDSHLPPSQNTSELNTA

>SASS6

MSQVLFHQLVPLQVKCKDCEERRVSIRMSIELQSVSNPVHRKDLVIRLTDDTDPFFLYNL
VISEEDFQSLKFQQGLLVDFLAFPQKFIDLLQQCTQEHAKEIPRFLLQLVSPAAILDNSP
AFLNVVETNPFKHLTHLSLKLLPGNDVEIKKFLAGCLKCSKEEKLSLMQSLDDATKQLDF
TRKTLAEKKQELDKLRNEWASHTAALTNKHSQELTNEKEKALQAQVQYQQQHEQQKKDLE
ILHQQNIHQLQNRLSELEAANKDLTERKYKGDSTIRELKAKLSGVEEELQRTKQEVLSLR
RENSTLDVECHEKEKHVNQLQTKVAVLEQEIKDKDQLVLRTKEAFDTIQEQKVVLEENGE
KNQVQLGKLEATIKSLSAELLKANEIIKKLQGDLKTLMGKLKLKNTVTIQQEKLLAEKEE

KLQKEQKELQDVGQSLRIKEQEVCKLQEQLEATVKKLEESKQLLKNNEKLITWLNKELNE
NQLVRKQDVLGPSTTPPAHSSSNTIRSGISPNLNVVDGRLTYPTCGIGYPVSSAFAFQNT
FPHSISAKNTSHPGSGTKVQFNLQFTKPNASLGDVQSGATISMPCSTDKENGENVGLESK
YLKKREDSIPLRGLSQNLFSNSDHQRDGTLGALHTSSKPTALPSASSAYFPGQLPNS

>MFSD2A

MAKGEGAESGSAAGLLPTSILQSTERPAQVKKEPKKKKQQLSVCNKLCYALGGAPYQVTG
CALGFFLQIYLLDVAQVGPFSASIILFVGRAWDAITDPLVGLCISKSPWTCLGRLMPWII
FSTPLAVIAYFLIWFVPDFPHGQTYWYLLFYCLFETMVTCFHVPYSALTMFISTEQTERD
SATAYRMTVEVLGTVLGTAIQGQIVGQADTPCFQDLNSSTVASQSANHTHGTTSHRETQK
AYLLAAGVIVCIYIICAVILILGVREQREPYEAQQSEPIAYFRGLRLVMSHGPYIKLITG
FLFTSLAFMLVEGNFVLFCTYTLGFRNEFQNLLLAIMLSATLTIPIWQWFLTRFGKKTAV
YVGISSAVPFLILVALMESNLIITYAVAVAAGISVAAAFLLPWSMLPDVIDDFHLKQPHF
HGTEPIFFSFYVFFTKFASGVSLGISTLSLDFAGYQTRGCSQPERVKFTLNMLVTMAPIV
LILLGLLLFKMYPIDEERRRQNKKALQALRDEASSSGCSETDSTELASIL

>CIT

MLKFKYGARNPLEAGAAEPIASRASRLNLFFQGKPPFMTQQQMSPLSREGILDALFVLFE
ECSQPALMKIKHVSNFVWKYSDTIAELQELQPSAKDFEVRSLVGCGHFAEVQVVREKATG
DIYAMKVMKKKALLAQEQVSFFEEERNILSRSTSPWIPQLQYAFQDKNHLYLVMEYQPGG
DLLSLLNRYEDQLDENLIQFYLAELILAVHSVHLMGYVHRDIKPENILVDRTGHIKLVDF
GSAAKMNSNKMVNAKLPIGTPDYMAPEVLTVMNGDGKGTYGLDCDWWSVGVIAYEMIYGR
SPFAEGTSARTFNNIMNFQRFLKFPDDPKVSSDFLDLIQSLLCGQKERLKFEGLCCHPFF
SKIDWNNIRNSPPPFVPTLKSDDDTSNFDEPEKNSWVSSSPCQLSPSGFSGEELPFVGFS
YSKALGILGRSESVVSGLDSPAKTSSMEKKLLIKSKELQDSQDKCHKMEQEMTRLHRRVS
EVEAVLSQKEVELKASETQRSLLEQDLATYITECSSLKRSLEQARMEVSQEDDKALQLLH
DIREQSRKLQEIKEQEYQAQVEEMRLMMNQLEEDLVSARRRSDLYESELRESRLAAEEFK
RKATECQHKLLKAKDQGKPEVGEYAKLEKINAEQQLKIQELQEKLEKAVKASTEATELLQ
NIRQAKERAERELEKLQNREDSSEGIRKKLVEAEERRHSLENKVKRLETMERRENRLKDD
IQTKSQQIQQMADKILELEEKHREAQVSAQHLEVHLKQKEQHYEEKIKVLDNQIKKDLAD
KETLENMMQRHEEEAHEKGKILSEQKAMINAMDSKIRSLEQRIVELSEANKLAANSSLFT
QRNMKAQEEMISELRQQKFYLETQAGKLEAQNRKLEEQLEKISHQDHSDKNRLLELETRL
REVSLEHEEQKLELKRQLTELQLSLQERESQLTALQAARAALESQLRQAKTELEETTAEA
EEEIQALTAHRDEIQRKFDALRNSCTVITDLEEQLNQLTEDNAELNNQNFYLSKQLDEAS
GANDEIVQLRSEVDHLRREITEREMQLTSQKQTMEALKTTCTMLEEQVMDLEALNDELLE
KERQWEAWRSVLGDEKSQFECRVRELQRMLDTEKQSRARADQRITESRQVVELAVKEHKA
EILALQQALKEQKLKAESLSDKLNDLEKKHAMLEMNARSLQQKLETERELKQRLLEEQAK
LQQQMDLQKNHIFRLTQGLQEALDRADLLKTERSDLEYQLENIQVLYSHEKVKMEGTISQ
QTKLIDFLQAKMDQPAKKKKGLFSRRKEDPALPTQVPLQYNELKLALEKEKARCAELEEA
LQKTRIELRSAREEAAHRKATDHPHPSTPATARQQIAMSAIVRSPEHQPSAMSLLAPPSS
RRKESSTPEEFSRRLKERMHHNIPHRFNVGLNMRATKCAVCLDTVHFGRQASKCLECQVM
CHPKCSTCLPATCGLPAEYATHFTEAFCRDKMNSPGLQTKEPSSSLHLEGWMKVPRNNKR
GQQGWDRKYIVLEGSKVLIYDNEAREAGQRPVEEFELCLPDGDVSIHGAVGASELANTAK

ADVPYILKMESHPHTTCWPGRTLYLLAPSFPDKQRWVTALESVVAGGRVSREKAEADAKL
LGNSLLKLEGDDRLDMNCTLPFSDQVVLVGTEEGLYALNVLKNSLTHVPGIGAVFQIYII
KDLEKLLMIAGEERALCLVDVKKVKQSLAQSHLPAQPDISPNIFEAVKGCHLFGAGKIEN
GLCICAAMPSKVVILRYNENLSKYCIRKEIETSEPCSCIHFTNYSILIGTNKFYEIDMKQ
YTLEEFLDKNDHSLAPAVFAASSNSFPVSIVQVNSAGQREEYLLCFHEFGVFVDSYGRRS
RTDDLKWSRLPLAFAYREPYLFVTHFNSLEVIEIQARSSAGTPARAYLDIPNPRYLGPAI
SSGAIYLASSYQDKLRVICCKGNLVKESGTEHHRGPSTSRSSPNKRGPPTYNEHITKRVA
SSPAPPEGPSHPREPSTPHRYREGRTELRRDKSPGRPLEREKSPGRMLSTRRERSPGRLF
EDSSRGRLPAGAVRTPLSQVNKVWDQSSV
>KIF14
MSLHSTHNRNNSGDILDIPSSQNSSSLNALTHSSRLKLHLKSDMSECENDDPLLRSAGKV
RDINRTYVISASRKTADMPLTPNPVGRLALQRRTTRNKESSLLVSELEDTTEKTAETRLT
LQRRAKTDSAEKWKTAEIDSVKMTLNVGGETENNGVSKESRTNVRIVNNAKNSFVASSVP
LDEDPQVIEMMADKKYKETFSAPSRANENVALKYSSNRPPIASLSQTEVVRSGHLTTKPT
QSKLDIKVLGTGNLYHRSIGKEIAKTSNKFGSLEKRTPTKCTTEHKLTTKCSLPQLKSPA
PSILKNRMSNLQVKQRPKSSFLANKQERSTENTILPEEETVVQNTSAGKDPLKVENSQVT
VAVRVRPFTKREKIEKASQVVFMSGKEITVEHPDTKQVYNFIYDVSFWSFDECHPHYASQ
TTVYEKLAAPLLERAFEGFNTCLFAYGQTGSGKSYTMMGFSEEPGIIPRFCEDLFSQVAR
KQTQEVSYHIEMSFFEVYNEKIHDLLVCKDENGQRKQPLRVREHPVYGPYVEALSMNIVS
SYADIQSWLELGNKQRATAATGMNDKSSRSHSVFTLVMTQTKTEFVEGEEHDHRITSRIN
LIDLSGSERCSTAHTNGDRLKEGVSINKSLLTLGKVISALSEQANQRSVFIPYRESVLTW
LLKESLGGNSKTAMIATISPAASNIEETLSTLRYANQARLIVNIAKVNEDMNAKLIRELK
AEIAKLKAAQRNSRNIDPERYRLCRQEITSLRMKLHQQERDMAEMQRVWKEKFEQAEKRK
LQETKELQKAGIMFQMDNHLPNLVNLNEDPQLSEMLLYMIKEGTTTVGKYKPNSSHDIQL
SGVLIADDHCTIKNFGGTVSIIPVGEAKTYVNGKHILEITVLRHGDRVILGGDHYFRFNH
PVEVQKGKRPSGRDTPISEGPKDFEFAKNELLMAQRSQLEAEIKEAQLKAKEEMMQGIQI
AKEMAQQELSSQKAAYESKIKALEAELREESQRKKMQEINNQKANHKIEELEKAKQHLEQ
EIYVNKKRLEMETLATKQALEDHSIRHARILEALETEKQKIAKEVQILQQNRNNRDKTFT
VQTTWSSMKLSMMIQEANAISSKLKTYYVFGRHDISDKSSSDTSIRVRNLKLGISTFWSL
EKFESKLAAMKELYESNGSNRGEDAFCDPEDEWEPDITDAPVSSLSRRRSRSLMKNRRIS
GCLHDIQVHPIKNLHSSHSSGLMDKSSTIYSNSAESFLPGICKELIGSSLDFFGQSYDEE
RTIADSLINSFLKIYNGLFAISKAHEEQDEESQDNLFSSDRAIQSLTIQTACAFEQLVVL
MKHWLSDLLPCTNIARLEDELRQEVKKLGGYLQLFLQGCCLDISSMIKEAQKNAIQIVQQ
AVKYVGQLAVLKGSKLHFLENGNNKAASVQEEFMDAVCDGVGLGMKILLDSGLEKAKELQ
HELFRQCTKNEVTKEMKTNAMGLIRSLENIFAESKIKSFRRQVQEENFEYQDFKRMVNRA
PEFLKLKHCLEKAIEIIISALKGCHSDINLLQTCVESIRNLASDFYSDFSVPSTSVGSYE
SRVTHIVHQELESLAKSLLFCFESEESPDLLKPWETYNQNTKEEHQQSKSSGIDGSKNKG
VPKRVYELHGSSPAVSSEECTPSRIQWV
